# Genome-Wide Association Study on Immunoglobulin G Glycosylation Patterns

**DOI:** 10.3389/fimmu.2018.00277

**Published:** 2018-02-26

**Authors:** Annika Wahl, Erik van den Akker, Lucija Klaric, Jerko Štambuk, Elisa Benedetti, Rosina Plomp, Genadij Razdorov, Irena Trbojević-Akmačić, Joris Deelen, Diana van Heemst, P. Eline Slagboom, Frano Vučković, Harald Grallert, Jan Krumsiek, Konstantin Strauch, Annette Peters, Thomas Meitinger, Caroline Hayward, Manfred Wuhrer, Marian Beekman, Gordan Lauc, Christian Gieger

**Affiliations:** ^1^Research Unit Molecular Epidemiology, Helmholtz Zentrum München – German Research Center for Environmental Health, Neuherberg, Germany; ^2^Institute of Epidemiology 2, Helmholtz Zentrum München – German Research Center for Environmental Health, Neuherberg, Germany; ^3^Molecular Epidemiology, Department of Biomedical Data Sciences, Leiden University Medical Center (LUMC), Leiden, Netherlands; ^4^Pattern Recognition and Bioinformatics, Delft University of Technology, Delft, Netherlands; ^5^Genos Glycoscience Research Laboratory, Zagreb, Croatia; ^6^MRC Human Genetics Unit, Institute of Genetics and Molecular Medicine, University of Edinburgh, Edinburgh, United Kingdom; ^7^Centre for Global Health Research Population Health Sciences, School of Molecular, Genetic and Population Health Sciences, Usher Institute of Population Health Sciences and Informatics, University of Edinburgh, Edinburgh, United Kingdom; ^8^Institute of Computational Biology, Helmholtz Zentrum München – German Research Center for Environmental Health, Neuherberg, Germany; ^9^Center for Proteomics and Metabolomics, Leiden University Medical Center (LUMC), Leiden, Netherlands; ^10^Max Planck Institute for Biology of Ageing, Köln, Germany; ^11^Department of Internal Medicine, Section Gerontology and Geriatrics, Leiden University Medical Center (LUMC), Leiden, Netherlands; ^12^German Center for Diabetes Research (DZD), Neuherberg, Germany; ^13^Institute of Genetic Epidemiology, Helmholtz Zentrum München – German Research Center for Environmental Health, Neuherberg, Germany; ^14^IBE, Faculty of Medicine, LMU Munich, Munich, Germany; ^15^Institute of Human Genetics, Helmholtz Zentrum München – German Research Center for Environmental Health, Neuherberg, Germany; ^16^Faculty of Pharmacy and Biochemistry, University of Zagreb, Zagreb, Croatia

**Keywords:** genome-wide association study, immunoglobulin G, glycosylation, *RUNX3*, LC–ESI-MS

## Abstract

Immunoglobulin G (IgG), a glycoprotein secreted by plasma B-cells, plays a major role in the human adaptive immune response and are associated with a wide range of diseases. Glycosylation of the Fc binding region of IgGs, responsible for the antibody’s effector function, is essential for prompting a proper immune response. This study focuses on the general genetic impact on IgG glycosylation as well as corresponding subclass specificities. To identify genetic loci involved in IgG glycosylation, we performed a genome-wide association study (GWAS) on liquid chromatography electrospray mass spectrometry (LC–ESI-MS)—measured IgG glycopeptides of 1,823 individuals in the Cooperative Health Research in the Augsburg Region (KORA F4) study cohort. In addition, we performed GWAS on subclass-specific ratios of IgG glycans to gain power in identifying genetic factors underlying single enzymatic steps in the glycosylation pathways. We replicated our findings in 1,836 individuals from the Leiden Longevity Study (LLS). We were able to show subclass-specific genetic influences on single IgG glycan structures. The replicated results indicate that, in addition to genes encoding for glycosyltransferases (i.e., *ST6GAL1, B4GALT1, FUT8*, and *MGAT3*), other genetic loci have strong influences on the IgG glycosylation patterns. A novel locus on chromosome 1, harboring *RUNX3*, which encodes for a transcription factor of the runt domain-containing family, is associated with decreased galactosylation. Interestingly, members of the *RUNX* family are cross-regulated, and *RUNX3* is involved in both IgA class switching and B-cell maturation as well as T-cell differentiation and apoptosis. Besides the involvement of glycosyltransferases in IgG glycosylation, we suggest that, due to the impact of variants within *RUNX3*, potentially mechanisms involved in B-cell activation and T-cell differentiation during the immune response as well as cell migration and invasion involve IgG glycosylation.

## Introduction

Glycosylation is among the most abundant post-translational protein modifications (1) and defects therein can lead to severe diseases (2–4), and aberrant glycosylation patterns are likewise associated with different types of cancer (5–12). A complex dynamic network, including genetic and epigenetic factors, regulates the glycosylation pathways, involving various enzymes taking part in these processes (13–15). Whereas most of the enzyme activities, as well as substrate specificity, are supported by *in vitro* experiments, *in vivo* experimental validation, taking into account the complex intracellular processes, is still unfeasible (16). To deepen our understanding of glycan biosynthesis and its role in the pathophysiology of many diseases, it is imperative, however, that we identify all factors involved in glycosylation pathways.

The best described glycoprotein so far is immunoglobulin G (IgG) (17). Its glycosylation is thought to have important regulatory functions in the immune response (18) and has been associated with various diseases, such as rheumatoid arthritis (19) and different types of cancers (10, 11). Also within the healthy population, a high interindividual variability in IgG glycosylation patterns is observed, that is, partly attributable to a heritable component (14, 20). With the development of high-throughput glycosylation techniques, it has now become feasible to analyze glycosylation profiles and their relation with genetics at a population level. A first genome-wide association study (GWAS) by Lauc et al. (21)., including 2,247 individuals from four European cohorts (CROATIA-Vis, CROATIA-Korcula, Orkney Complex Disease Study and Northern Swedish Population Health Study), identified four loci encoding glycosyltransferases associated with IgG *N*-glycans. The authors likewise propose that five additional loci are involved in IgG glycosylation showing that a GWAS can be used to identify genetic loci controlling glycosylation of a single plasma protein (21). They replicated the association of two of their loci, *MGAT3* and *B4GALT1*, in a cohort of MALDI–TOF MS (matrix-assisted laser desorption/ionization time-of-flight mass spectrometry) measured glycan data from the Leiden Longevity Study (LLS) (22). A recent study by Shen et al. (23) used a multiphenotype approach to analyze the genetic background of IgG glycosylation. Here, the authors examine IgG glycan structures measured by ultra-performance liquid chromatography [(UPLC) (24)] in a multivariate way and thereby detect five novel genetic loci that are associated with combinations of IgG glycan traits.

In contrast to UPLC, used by Lauc et al. and Shen et al., the liquid chromatography electrospray mass spectrometry (LC–ESI-MS) method allows for subclass-specific quantification of *N*-linked glycans. It has been shown that the IgG subclasses, IgG1–IgG4, not only differ in their structure, especially within the hinge region of the glycoprotein, but also in their effector functions (17, 25). Besides differences in the number of disulfide bonds and the length and flexibility of the hinge region, glycosylation profiles also differ between the four IgG subclasses (26). While IgG2 is characterized by a higher degree of core-fucosylation and a low level of galactosylation, IgG1 shows a particularly high level of galactosylation for both neutral and sialylated structures (26). IgG4, on the other hand, shows a high level of core-fucosylated complexes with bisecting *N*-acetylglucosamine (GlcNAc) (26). How these subclass-specific glycosylation profiles are realized and what their specific contributions are in the pathophysiology of diseases remains largely illusive.

Previous GWAS on serum metabolite levels have indicated that analyzing enzyme substrate-product ratios benefits in gain by power for detecting associated genetic loci over analyzing single metabolites (27). Due to the LC–ESI-MS method, we are able to derive different types of IgG glycan traits to address the genetic background of the IgG glycan synthesis, including within-subclass ratios representing the addition of one monosaccharide at a time, i.e., a single pathway step within IgG glycan synthesis. The same approach was utilized to validate pathway steps inferred by a network-based approach in Benedetti et al. (28). Here, the authors included GWAS data on ratios of IgG glycan structures representing specific, established and newly predicted enzymatic pathway steps. The GWAS data as well as additional laboratory experiments verified the hypotheses drawn from the network analysis. In contrast to Benedetti et al. we extend the list of ratios to all possible one-step pathway steps independent of any prior selection. Furthermore, to challenge the assumption of similar genetic control of glycan biosynthesis for all IgG subclasses (16), we additionally compute subclass-specific IgG glycan traits containing between-subclass ratios and subclass-specific IgG glycan proportions. Furthermore, we include summarizing traits for IgG glycan structures to capture general trends associated with variations in genetic loci as well as additional biologically meaningful glycosylation traits.

By means of a GWAS including these newly derived traits of the LC–ESI-MS measured IgG glycopeptides in our discovery cohort from the KORA F4 study (*n* = 1,823), and a replication of the results for the same glycan panel in an additional 1,800 samples from LLS, we want to further investigate the underlying genetic control of IgG glycosylation.

## Results

**Figure 1:**
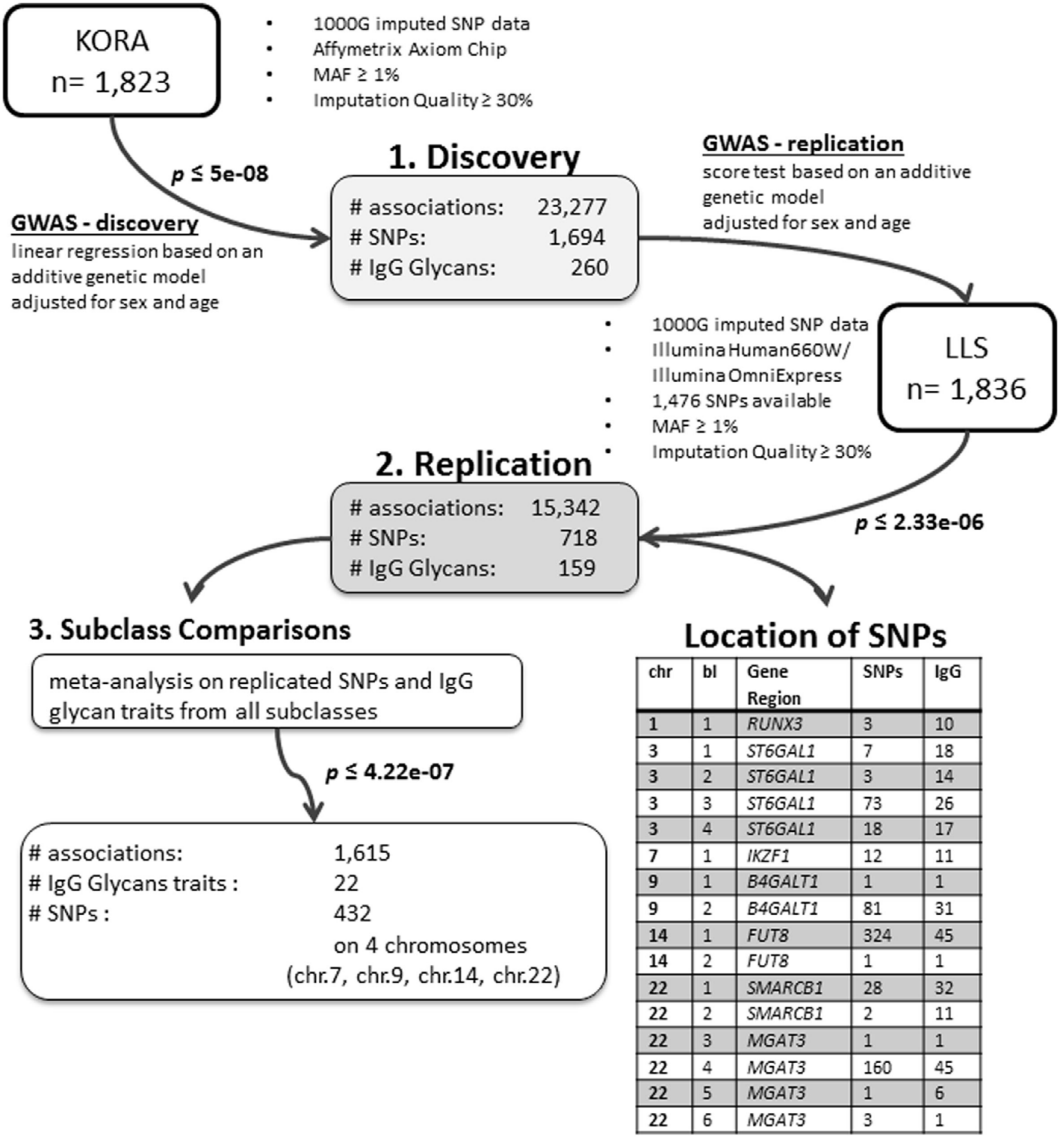
Overview of the study and analyses.

We conducted an age- and sex-adjusted genome-wide association scan on 376 glycan traits, including 50 initial measured IgG glycopeptides, 155 summarizing derived traits, 95 within-subclass ratios, 40 between-subclass ratios, and 36 glycan proportions (see Figure 1 for an overview; Table S1 in Supplementary Material; and Section “Materials and Methods” for further details). In our discovery cohort, KORA F4 (*n* = 1,823, study characteristics in Table S2 in Supplementary Material), 23,277 associations between 1,694 SNPs and 260 traits reached the suggestive significance threshold (*p* < 5 × 10^−8^, Bonferroni corrected), out of which 14,425 associations (848 SNPs and 164 traits) reached genome-wide significance (*p* < 1 × 10^−9^, Bonferroni corrected). Explained variances in the discovery cohort ranged from 1.4 to 14.1% (Table S3 in Supplementary Material).

**Figure 2:**
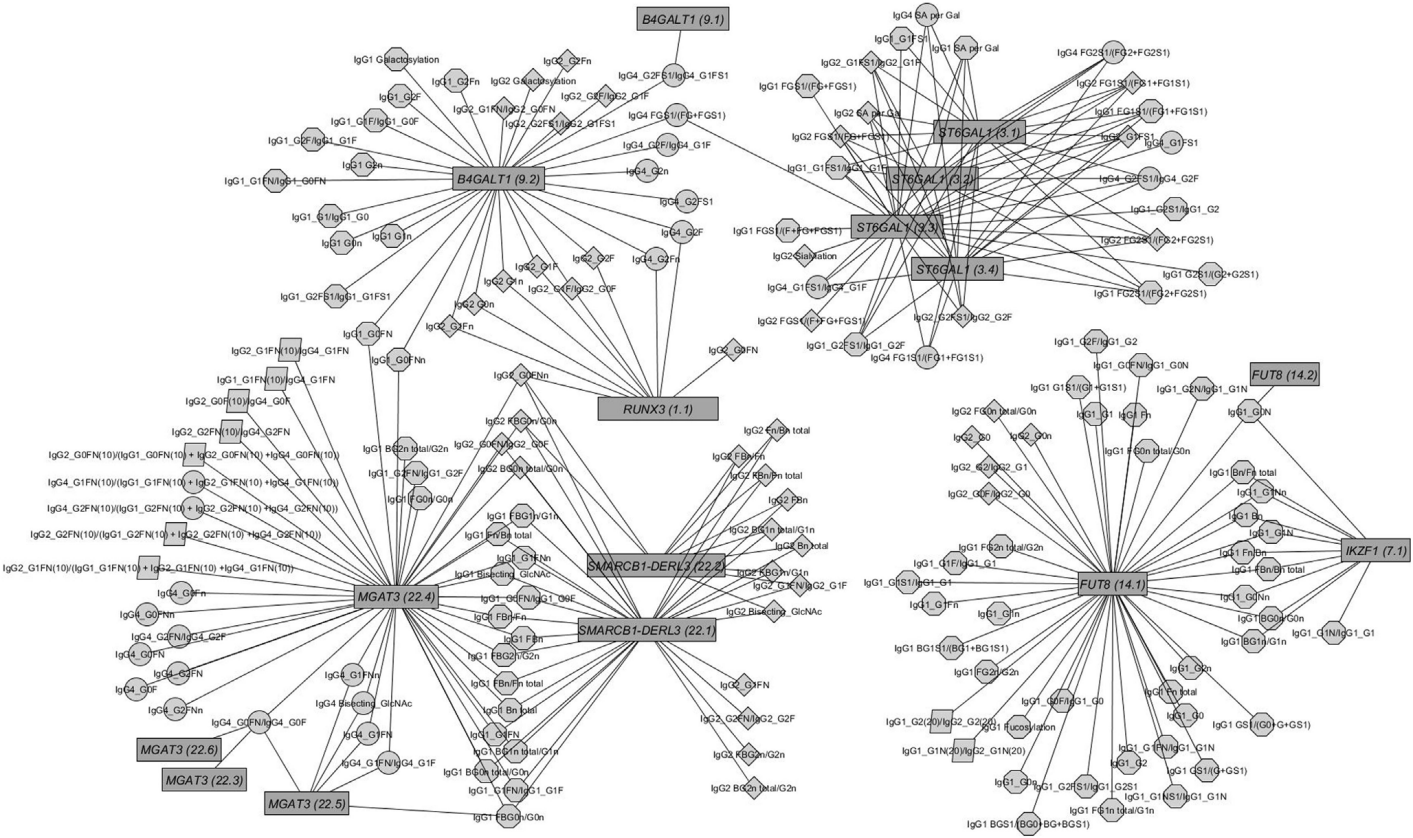
Network of replicated associations immunoglobulin G (IgG) glycan traits (circles) for different subclasses (octagon: IgG1; diamonds: IgG2; circles: IgG4; parallelogram: subclass comparisons) and their associations to the seven replicated loci (edges only for replicated results).

Out of the suggestive 1,694 SNPs in the discovery, 1,476 SNPs were available for replication in the LLS cohort. The list of 1,801 SNP-trait associations excluded from the replication can be seen in Table S4 in Supplementary Material. For the replication, we used in total 21,476 associations between 1,476 SNPs and 253 phenotypic traits and set our Bonferroni-corrected replication significance threshold to 2.33 × 10^–6^. From the 21,476 associations available for replication, we replicated 15,342 associations between 159 traits and 718 SNPs, which are displayed in Figure 2 (network representation) and Figure S1 (Manhattan plot) and Table S5 (all replicated results) in Supplementary Material. Table 1 summarizes the mentioned results. This table presents the associated genomic loci with *p*-values and effect sizes from both cohorts and associated IgG glycan traits and their directions of association.

**Table 1:** Summarized table of replicated associations.

Chromosome	LD-block per chromosome	Minimal position	Maximal position	Gene locus	Number of associated replicated SNPs	Number of associated replicated traits	Number of traits with positive effect estimates^a^	Number of traits with negative effect estimates^a^	Replicated SNP with lowest *p*-value*	Replicated trait with lowest *p*-value*	*p*-Value in discovery (KORA) for the lead SNP–glycan combination	Effect size in discovery (KORA) for the lead SNP–glycan combination	*p*-Value in replication [Leiden Longevity Study (LLS)] for the lead SNP–glycan combination	Effect size in replication (LLS) for the lead SNP–glycan combination	Effect allele for lead SNP	Other allele for lead SNP	Effect allele frequency in the discovery (KORA)	Other associated traits
1	1	25296560	25298841	*RUNX3*	3	10	3	7	rs16830188	LC_IGP90	1.71E−14	1.00868	1.27E−06	0.7404	T	C	0.0179878	LC_IGP_R62#, LC_IGP133#, LC_IGP135, LC_IGP144, LC_IGP145#, LC_IGP175#, LC_IGP199#, LC_IGP88#, LC_GP89#

3	1	186705790	186708013	*ST6GAL1*^a^	7	18	0	18	rsl30S2825	LC_IGPI22	6.25E−21	−0.347613	1.60E−23	−0.4665	C	G	0.54829256	LC_IGP_R34, LC_IGP_R35, LC_IGP_R74, LC_IGP_R75, LC_IGP_R92, LC_IGP_R93, LC_IGP111, LC_IGP120, LC_IGP123, LC_IGP186, LC_IGP189, LC_IGP190, LC_IGP25, LC_IGP36, LC_IGP37, LC_IGP7, LC_IGP93

3	2	186708571	186711453	*ST6GAL1*^a^	3	14	0	14	rs4012171	LC_IGPI22	1.22E−17	−0.354924	2.79E−19	−0.4471	C	A	0.75708572	LC_IGP_R34, LC_IGP_R35, LC_IGP_R74, LC_IGP_R75, LC_IGP_R93, LC_IGP111, LC_IGP120, LC_IGP123, LCJGP190, LC_IGP25, LC_IGP36, LC_IGP37, LC_IGP93

3	3	186712711	186744563	*ST6GAL1*^a^	73	26	0	26	rs11710456	LC_IGP R74	1.17E−56	−0.608772	6.30E−73	−0.7558	A	G	0.26023337	LC_IGP_R33, LC_IGP_R34, LC_IGP_R35, LC_IGP_R75, LC_IGP_R92, LC_IGP_R93, LC_IGP110, LC_IGP111, LC_IGP120, LC_IGP121, LC_IGP122, LC_IGP123, LC_IGP179, LC_IGP186, LC_IGP187, LC_IGP189, LC_IGP190, LC_IGP25, LC_IGP29, LC_IGP34, LC_IGP35, LC_IGP36, LC_IGP37, LC_IGP7, LC_IGP93

3	4	186754722	186782999	*ST6GAL1*^a^	18	17	16	17	rs57679165	LC_IGP_R74	3.72E−20	−0.41011	4.60E−23	−0.4698	G	C	0.18513165	LC_IGP_R34, LC_IGP_R35, LC_IGP_R75, LC_IGP_R92, LC_IGP_R93, LC_IGP111, LC_IGP120, LC_IGP122, LC_IGP123, LC_IGP186, LC_IGP189, LC_IGP190, LC_IGP25, LC_IGP36, LC_IGP37, LC_IGP93

7	1	50336551	50355207	*IKZF1*^a^	12	11	2	9	rs7782210	LC_IGP56	2.76E−09	−0.220405	2.38E−13	−0.2853	G	A	0.35729058	LC_IGP_R16, LC_IGP14, LC_IGP15, LC_IGP55, LC_IGP77, LC_IGP78, LC_IGP79, LC_IGP81#, LC_IGP84#, LC_IGP86

9	1	33113322	33113322	*B4GALT1*^a^	1	1	0	1	rs7019909	LC_IGP_R89	2.90E−08	−0.30427	1.52E−07	−0.3075	T	C	0.12458268	

9	2	33119241	33180813	*B4GALT1*^a^	81	31	17	15	rs12342831	LC_IGP_R89	1.79E−17	−0.3271−43	2.96E−20	−0.3813	C	T	0.2611263	LC_IGP_R20, LC_IGP_R22, LC_IGP_R23, LC_IGP_R28, LC_IGP_R29, LC_IGP_R62, LC_IGP_R63, LC_IGP_R68, LC_IGP_R69, LC_IGP_R88, LC_IGP109, LC_IGP133, LC_IGP134, LC_IGP144#, LC_IGP145, LC_IGP175, LC_IGP180, LC_IGP187, LC_IGP199, LC_IGP205, LC_IGP23, LC_IGP3, LC_IGP4#, LC_IGP48, LC_IGP49#, LC_IGP58#, LC_IGP59, LC_IGP60, LC_IGP88, LC_IGP89

14	1	65734600	66262963	*FUT8*^a^	324	45	44	42	rsll158592	LC_IGP11	1.32E−24	−0.348762	2.39E−19	−0.3429	T	G	0.49714172	LC_IGP_Rl#, LC_IGP_R2#, LC_IGP_R26#, LC_IGP_R3#, LC_IGP_R32#, LC_IGP_R36#, LC_IGP_R4#, LC_IGP_R41#, LC_IGP_R5#, LC_IGP_R64#, LC_IGP_R8#, LC_IGP_SC13, LC_IGP_SC15, LC_IGP12, LC_IGP13, LC_IGP138, LC_IGP14, LC_IGP148#, LC_IGP15, LC_IGP21#, LC_IGP26#, LC_IGP27#, LC_IGP28#, LC_IGP31#, LC_IGP32#, LC_IGP47#, LC_IGP52, LC_IGP53, LC_IGP54, LC_IGP55, LC_IGP56, LC_IGP61#, LC_IGP62#, LC_IGP63#, LC_IGP64#, LC_IGP65#, LC_IGP68#, LC_IGP77, LC_IGP78, LC_IGP79, LC_IGP81#, LC_IGP84#, LC_IGP86, LC_IGP97

14	2	66275755	66275755	*FUT8*^a^	1	1	1	0	rs4899183	LC_IGP14	1.17E−08	0.210149	1.96E−06	0.191	G	A	0.65685741	

22	1	24100654	24179922	*SMARCB1-DERL3*^a^	28	32	7	30	rs2186369	LC_IGP108	1.55E−09	−0.286669	2.50E−13	−0.3778	G	T	0.17226762	LC_IGP_R11, LC_IGP_R12, LC_IGP_R51, LC_IGP_R52, LC_IGP_R53, LC_IGP135, LC_IGP155, LC_IGP156, LC_IGP157, LC_IGP158, LC_IGP159, LC_IGP160, LC_IGP161, LC_IGP162, LC_IGP168, LC_IGP169, LC_IGP171#, LC_IGP22, LC_IGP5, LC_IGP50, LC_IGP69, LC_IGP70, LC_IGP71, LC_IGP72, LC_IGP73, LC_IGP74, LC_IGP75, LC_IGP82, LC_IGP83, LC_IGP85#, LC_IGP91

22	2	24182500	24189032	*SMARC81-DERL3*^a^	2	11	1	10	rs6519476	LC_IGP _55	1.19E−09	−0.259124	9.57E−09	−0.2448	A	G	0.25942042	LC_IGP_R52, LC_IGP108, LC_IGP135, LC_IGP156, LC_IGP157, LC_IGP159, LC_IGP161, LC_IGP168, LC_IGP169, LC_IGP171#

22	3	39737929	39737929	*MGAT3*^a^	1	1	0	1	rsl37680	LC_IGP_R81	7.82E−11	−0.271863	3.35E−07	−0.2543	T	C	0.59436303	

22	4	39738425	39860868	*MGAT3*^a^	160	45	36	42	rs73167342	LC_IGP_R81	7.71E−35	−0.455612	2.53E−38	−0.5559	G	C	0.662518	LC_IGP_R11, LC_IGP_R12, LC_IGP_R13, LC_IGP_R51, LC_IGP_R82, LC_IGP_R83, LC_IGP_SC25#, LC_IGP_SC31, LC_IGP_SC35#, LC_IGP_SC36#, LC_IGP135, LC_IGP156, LC_IGP160, LC_IGP173#, LC_IGP176, LC_IGP177, LC_IGP178, LC_IGP183, LC_IGP197#, LC_IGP200, LC_IGP201, LC_IGP202, LC_IGP22, LC_IGP4, LC_IGP49, LC_IGP5, LC_IGP50, LC_IGP66#, LC_IGP69, LC_IGP70, LC_IGP71, LC_IGP72, LC_IGP73, LC_IGP74, LC_IGP75, LC_IGP76, LC_IGP82, LC_IGP83, LC_IGP85#, LC_IGPRG14#, LC_IGPRG15#, LC_IGPRG16#, LC_IGPRG25, LC_IGPRG26

22	5	39873937	39873937	*MGAT3*^a^	1	6	6	0	rsl2484278	LC_IGP_R81	3.66E−12	0.320619	2.56E−10	0.287	A	G	0.24144208	LC_IGP_R82, LC_IGP177, LC_IGP177, LC_IGP183, LC_IGP201, LC_IGP70

22	6	39889080	39893932	*MGAT3*^a^	3	1	1	0	rs34692520	LC_IGP_R81	1.39E−12	0.308965	I.09E−06	0.229	G	C	0.24220301	

*Lowest p-value to any glycan trait from either the discovery or replication cohort.

*^a^Effect estimates for any SNP within the linkage disequilibrium-Block*.

*#SNP effect in opposite direction to most significant trait*.

The *replicated traits* cover all types of glycan traits and all IgG subclasses: 22 (out of 50) initial IgG glycopeptides, 87 (out of 155) summarizing derived traits, 39 (out of 95) within-subclass ratios, 6 (out of 40) between-subclass ratios, and 5 (out of 36) glycan proportions. Effects for all replicated associations are in the same direction and of similar magnitude as in the discovery cohort (part 2 in Figure 1).

The *replicated SNPs* are spread over *seven independent loci* on six chromosomes [chromosome 1: 25,296,560–25,298,841 (6,809 bp upstream of *RUNX3*), chromosome 3: 186,705,790–186,782,999 (*ST6GAL1*), chromosome 7: 50,336,551–50,355,207 (*IKZF1*), chromosome 9: 33,113,322–33,180,813 (*B4GALT1*), chromosome 14: 65,734,600–66,275,755 (*FUT8*), chromosome 22: 24,100,654–24,189,032 (*SMARCB1/DERL3*), and chromosome 22: 39,737,929–39,893,932 (*MGAT3*)]. An overview of the associated traits per locus can be found in Table S8 in Supplementary Material and is shortly given in Table 1. With our study, we can confirm six of the loci associated with UPLC-measured IgG glycan traits (21, 23) being associated with LC–ESI-MS-measured IgG glycan structures in a comparable way (see the Supplementary note and Table S13 in Supplementary Material for additional details). In addition, we detect a novel locus at *RUNX3* (chromosome 1p36.11).

On chromosome 1, three SNPs (rs16830188, rs10903120, and rs11270291) have significant impact on glycan traits. A multivariate analysis in KORA F4 reveals that the three SNPs describe one locus, with rs16830188 being the most influential SNP (see Table S10 in Supplementary Material). These SNPs are in high linkage disequilibrium (LD) (*r*^2^ ≥ 0.5) and are flanking the gene *RUNX3* (see Figure S6A in Supplementary Material). The T-allele of the most significant marker for all associated glycan traits, rs16830188, is associated with an increase in agalactosylated structures and a decrease in mono- and digalactosylated structures. In addition, this SNP has the largest effect sizes for all associated glycan traits and explains 1.4 to 3.5% of the variance of the associated traits (see Table S3 in Supplementary Material). The genetic variants within *RUNX3* especially affect IgG glycan traits from IgG2 and IgG4, illustrating the merit of the subclass-specific analysis.

**Figure 3:**
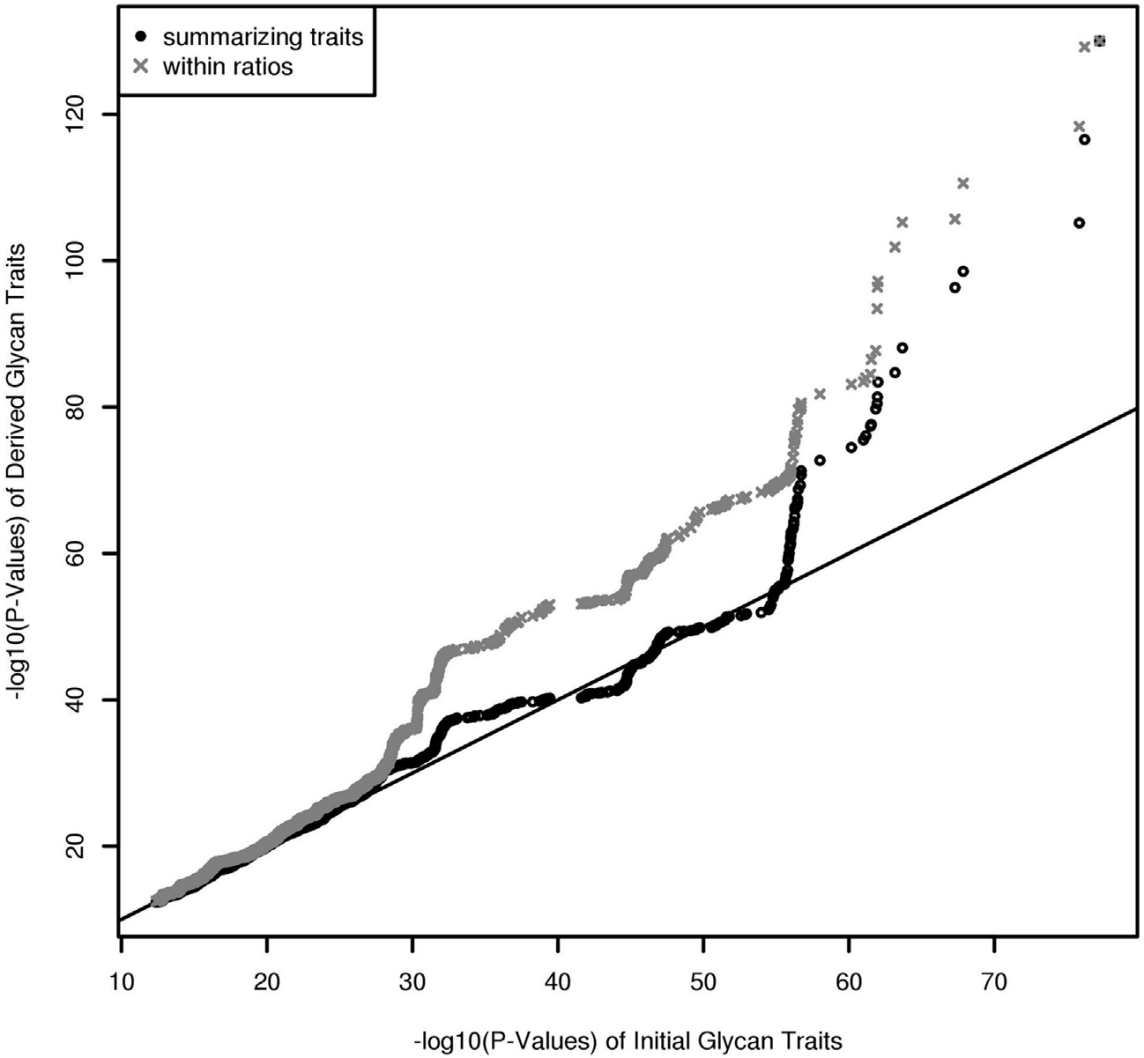
QQ-Plot (−log10(*p*-values)) for comparison of the results from initial glycopeptide traits and from within-subclass ratios and summarizing derived traits (results are obtained from a Meta-Analysis of the Replicated Associations).

In contrast to UPLC, LC–ESI-MS is suited for quantifying subclass-specific IgG glycan structures and thus for analyzing within-subclass *ratios* that represent single pathway steps in IgG glycan synthesis, as well as between-subclass ratios and glycan proportions. Using QQ-plots to compare the associations obtained with initial IgG glycan traits versus within-subclass ratios, we clearly demonstrate a *gain in power* for the latter analytical approach (Figure 3). These ratios even outperform the summarizing derived traits. Subclass specificity assessed by glycan proportions and between-subclass ratios perform almost as good as initial measured IgG glycopeptides, except for associations with very low *p-*values (1 × 10^–18^) (Figure S3A in Supplementary Material). In addition, we performed meta-analyses on replicated SNP–glycan associations of the two cohorts and statistically compared the strength of the associations of the same glycan trait for different subclasses.

Except for four IgG glycan ratios (IgG2_G2/IgG2_G1, IgG1_G2N/IgG1_G1N, IgG1_G1NS1/IgG1_G1N, and IgG1_G1S1/IgG1_G1), the associations of *within-subclass ratios* support the known functions of the glycosyltransferases within the IgG glycan synthesis across the subclasses (see Figures S4A–F in Supplementary Material). Ratios of monogalactosylated over agalactosylated structures are associated with SNPs within the galactosyltransferase *B4GALT1* locus; ratios of structures with bisecting GlcNAc over structures without bisecting GlcNAc associate with SNPs within the *N*-acetylglucosaminyltransferase *MGAT3* locus; ratios of sialylated over non-sialylated glycan traits associate to variants within the sialyltransferase *ST6GAL1* locus and ratios of fucosylated over non-fucosylated structures associate with fucosyltransferase *FUT8*.

Besides known pathway steps in IgG glycan synthesis and inferred reactions from network analyses (28), the results hint at hitherto unknown enzymatic reaction steps catalyzed by the known glycosyltransferases, e.g., associations between variants of *FUT8* and IgG1_G0FN/IgG1_G0N.

*Subclass comparisons* of meta-analyzed data revealed that 22 glycan traits are significantly different associated with 432 SNPs on 4 chromosomes (Table S7 in Supplementary Material contains all results from the statistical tests, an overview is given in Table S8 in Supplementary Material and, graphically, in Figures S2 and S4F in Supplementary Material). In addition, as stated before, associations to five between-subclass ratios and six glycan proportions were replicated. Taken together, the major difference of IgG glycan traits on different subclasses lies in bisecting and fucosylation. We found 13 IgG glycans to be significantly different associated with SNPs at *FUT8* between IgG1 and IgG2. In addition, the neutral glycan traits G0n, G1n, and G2n showed significantly different behaviors between IgG1 and IgG2 as well as between IgG2 and IgG4 as the T-allele of the strongest SNP in this locus (*FUT8*), rs11158592, was significantly negative associated with these traits in IgG1 and IgG2 but not in IgG4.

Furthermore, for the association between SNPs within *MGAT3* and IgG glycan traits, we detected two traits being significantly different between IgG1 and IgG2, 10 glycan traits differing between IgG2 and IgG4, and only one glycan trait being different for IgG1 and IgG4 (G1FN). Almost all of the significantly differing glycan structures contain a bisecting GlcNAc. In addition, within-subclass ratios representing the addition of a GlcNAc (G1FN/G1F, G2FN/G2F, and G0FN/G0F) are significantly differently associated with the *MGAT3* locus for the IgG subclasses.

## Discussion

Our study attempts to deepen the knowledge of genetic influence on IgG glycosylation and to disclose possible subclass specificity in the synthesis pathways. We used the LC–ESI-MS-measured glycopeptides in both the discovery and replication cohort. In contrast to the UPLC and MALDI–TOF MS data used in Ref. (21, 23), we quantify subclass-specific attached *N*-glycans, and the traits are comparable between the discovery and replication study.

With the new analytical method, LC–ESI-MS, we confirmed the association of IgG glycosylation to six of the loci previously identified with UPLC (21, 23) and, moreover, detect a novel locus, *RUNX3*, on chromosome 1p36.11. Unfortunately, we could not verify any of the additional proposed loci proposed by Shen et al. (23), probably due to power reasons and difference in statistical methodology (multivariate vs univariate approach). In addition, it has to be highlighted, however, that IgG glycan traits originating from the two analytical methods cannot be combined straightforwardly (see the Supplementary note and Table S10 in Supplementary Material).

*RUNX3* encodes for a transcription factor of the runt domain-containing family. It is located on chromosome 1 and three variants within this locus are associated with 10 phenotypic traits. All three SNPs are in high LD to each other (*r*^2^ ≥ 0.5). *RUNX3* and other transcription factors of the runt-family have a large impact on hematopoiesis (29). Methylation of *RUNX3* promoters has an impact on several diseases (30–32), as well as on inflammation and immune response (33–35). In particular, *RUNX3* could be linked to B-cell maturation (36).

In addition, the transcription factor has been shown to contribute greatly to the regulation of apoptosis in cancer metastasis in general (37) and in the differentiation of T-cells to CD4+ and CD8+ T-cells in particular (38–41). While IgG is secreted by differentiated B-cells, it nonetheless has been shown that IgG1 glycosylation is dependent on B cell stimuli during their differentiation. These stimuli include T-cell derived cytokines and metabolites (42). By influencing T-cell differentiation, *RUNX3* could likely indirectly influence the glycosylation of antibodies produced by B-cells. Thus, T-cell differentiation may stimulate B-cell activation and influence the glycosylation of their secreted antibodies.

The opposing effect directions for structures with and without attached galactose lead to the hypothesis that the *RUNX3* locus plays an important role in galactosylation. There is a striking overlap between glycan traits associated with the *RUNX3* locus and the *B4GALT1* locus, supporting this hypothesis. Interestingly, a similar feature as for *RUNX3*, namely altering the differentiation process of T-cells, is attributed to the enzymes of the Ikaros family including *IKZF1* (43, 44). However, in our study no glycan traits overlapped for the two loci. Potentially, the two transcription factors regulate different glycosyltransferases.

While the other six loci have been described before in Ref. (21, 23) to be associated with *N*-glycan biosynthesis, variants in the *RUNX3* locus are novel candidates from our study. Since only glycan traits from less abundant IgG2 (IgG2/3 in KORA) and IgG4 were associated (17), it is reasonable to assume that the reason why the locus on chromosome 1 could not be detected before by the UPLC is because this technique does not provide information about *N*-glycosylation that is subclass specific, but instead results in total IgG *N*-glycans quantification and thus, a larger sample size may have been needed for UPLC data. Indeed, subclass-specific analyses reveal this association presumably due to higher power for the subclass-specific associations.

The IgG glycan traits based upon the two analytical methods, UPLC and LC–ESI-MS are not entirely comparable. A benefit of the LC–ESI-MS method is the subclass-specific IgG glycosylation measurements, with the drawback of non-separable IgG2 and IgG3 in the discovery cohort, which is due to the identical peptide moieties (E293EQFNSTFR301) of their tryptic Fc glycopeptides in Caucasians (45). Nevertheless, we were able to compare SNP associations for similar glycan traits between the subclasses and examine the IgG glycan synthesis separately for each subclass.

The within-subclass ratios representing enzymatic pathway steps are mainly associated with the assumed genetic loci coding for known glycosyltransferases (16) (see Figures S4C–E in Supplementary Material). These traits contain not only well-known enzymatic reactions within IgG glycan synthesis (Figure S4A in Supplementary Material), inferred reactions based on network analysis as in Ref. (28) but also all possible ratios representing the addition of one monosaccharide at a time (Figure S4B in Supplementary Material). Comparing the results from the known enzymatic steps and other possible one-step pathway relations suggests the existence of several of the latter (see Figure S3B in Supplementary Material), even in addition to the pathway steps supposed by Benedetti et al. (28). However, few of the within-subclass ratios are associated with variants from different genetic loci (see Figure 2).

The comparison of IgG glycan traits across subclasses leads to the hypothesis that fucosylation catalyzed by Fut8 and the addition of bisecting GlcNAc supported by Mgat3 is realized to different extent between the IgG subclasses. Fucosylation seems to be especially different between IgG1 and IgG2 while bisection is mostly differing between IgG2 and IgG4. For more details, see the supplementary note in Data Sheet S1 Supplementary Material. Still, functional studies are needed to elucidate the underlying mechanisms, especially with regard to subclass specificity. Indeed, it has been shown that specific subclasses and their attached glycan structure are highly relevant as biomarkers for diseases and even more when used in antibody therapy (46–50).

The obtained results help to broaden our knowledge on the pathway steps of IgG glycan synthesis in general, and, specifically the differences for each IgG subclass. While the initial glycan traits outperform the between-subclass ratios and glycan proportions, the findings from comparing SNP–glycan associations across subtypes (Table S5 and Figures S2 and S4F in Supplementary Material) hint at altered glycan synthesis for the different IgG subclasses.

## Conclusion

Summarizing, our analysis yields 159 phenotypic traits based on LC–ESI-MS measured IgG glycopeptide structures being significantly associated with 718 genetic variants on seven distinct loci. For UPLC-measured IgG glycans, six out of the seven loci have been shown to influence IgG glycosylation (21, 23). The new gene found to be associated with LC–ESI-MS measured IgG glycopeptide traits is *RUNX3* on chromosome 1. Ratios of IgG glycans representing enzymatic pathway steps within the *N*-glycan biosynthesis are predominantly associated with genetic variants within regions of *a priori* suggested genes encoding for known glycosyltransferases. Subclass comparisons point to specific behavior of variants covering the *MGAT3* locus on chromosome 22 and the *FUT8* locus on chromosome 14.

## Materials and Methods

### Discovery Cohort—KORA F4

The KORA F4 study, conducted in 2006–2008, is an independent population-based health survey (51) and was performed as a follow-up of the KORA S4 study (1999–2001) (52). The study followed the recommendations of the Declaration of Helsinki and was approved by the local ethical committees. In the F4 follow-up, a total of 3,080 persons participated of whom 1,823 individuals were available for the genome-wide association scan of IgG glycopeptides traits. Genotyping was realized with the Affymetrix Axiom Chip (53, 54). Prephasing was done by SHAPEIT v2 and imputation was carried out by IMPUTE v2.3.0 using 1000 Genome (phase 1 integrated haplotypes CEU) as a reference panel. SNPs were non-monomorphic and filtered based on their call rate (98%), their minor allele frequency (>1%) and were excluded if they significantly aberrated from the Hardy–Weinberg Equilibrium (*p* < 5 × 10^−6^). All individuals were of European ancestry and samples with mismatching phenotypic and genetic gender were excluded. These criteria led to a total of 18,185,628 SNPs. After the analysis, we additionally excluded SNPs with imputation quality defined by IMPUTE lower than 30%.

A total of 1,823 individuals from the KORA F4 cohort were used for discovery. The samples include 935 women and 888 men ranging from 32 to 81 years, with mean age of 62.56 years (SD = 9.89) (see Table S2 in Supplementary Material for more details).

### Replication Cohort—LLS

The LLS followed the recommendations of the Declaration of Helsinki, the study protocol was approved by the local medical ethical committee and good clinical practice guidelines were maintained.

The LLS examined long-lived siblings of European descent together with their offspring and the partners of the offspring. Families with at least two long-lived siblings (age ≥89 for man, age ≥91 for women) were recruited. This age category represented <0.5% of the Dutch population in 2001 (22). In total, 944 long-lived individuals (age range 89–104), 1,671 of their offspring (age range 39–81), and 744 partners thereof (60 years, 36–79) were included (55). DNA genotyping for LLS was performed at baseline as described in detail in Ref. (56) with the Illumina Human660W and Illumina OmniExpress arrays. Genotype imputation was performed using IMPUTE v2.2 (beta) with the 1000 Genome (phase 1 integreated haplotypes CEU) as reference panel. Quality control included SNP-wise call rate (95%), their minor allele frequency (>1%) and derivation from the Hardy–Weinberg equilibrium (*p* < 1 × 10^−4^). As for KORA F4, we excluded SNPs with imputation quality lower than 30% as provided by IMPUTE. For the current genome-wide association analysis with IgG glycopeptide measurements, 1,836 samples of offspring and their partners were available.

### Measurement of IgG Glycosylation

#### IgG Isolation

As described in Ref. (20), IgG was isolated from plasma by affinity chromatography using 96-well protein G monolithic plates (BIA Separations, Ljubljana, Slovenia) for KORA F4 samples and Protein A Sepharose Fast Flow beads (GE Healthcare, Uppsala, Sweden) for the LLS samples. For the KORA F4 sample analysis, 100 µL of plasma was first diluted 10× with 1× PBS and then filtered through 0.45 µm GHP filter plate (Pall Corporation, Ann Arbor, MI, USA). Following, it was applied to the protein plate and instantly washed. With 1 mL of 0.1 M formic acid (Merck, Darmstadt, Germany), the IgGs were eluted from the protein plate and neutralized with 1 M ammonium bicarbonate (Acros Organics, NJ, USA). For the LLS sample analysis, 2 µL of plasma was incubated together with 15 µL of Protein A beads in a total volume of approximately 180 µL PBS in 96-well filter plates. The samples were then washed thrice with PBS and thrice with MilliQ-purified water, before elution with 0.1 M formic acid (Fluka, Steinheim, Germany). The samples were subsequently dried in a vacuum concentrator for 2 h at 60°C.

Due to the different IgG isolation procedures for KORA F4 and LLS, we obtained subclass-specific measurements for IgG1, IgG2/IgG3, and IgG4 in KORA F4 and IgG1, IgG2, and IgG4 in LLS. IgG3 is less abundant compared with IgG2 and we thus denote the IgG2/IgG3 mixture in KORA as IgG2 only.

#### IgG Tryptic Digestion and Purification

Isolated IgG (approximately 25 µg) was resuspended in 40 µL of ammonium bicarbonate containing 200 ng of trypsin (Worthington, USA for KORA F4 samples; sequencing grade modified trypsin, Promega, Madison, WI, USA for LLS samples) and digested at 37°C over night. The KORA F4 samples underwent an additional purification step: resulting tryptic glycopeptides were purified by reverse phase solid phase extraction using Chromabond C18 ec beads (Macherey-Nagel, Germany). C18 beads were activated by 80% acetonitrile (ACN), 0.1% trifluoroacetic acid (TFA) (Sigma-Aldrich, USA) and conditioned with 0.1% TFA. Tryptic digest was diluted 10× with 0.1% TFA and loaded onto C18 beads. Beads were washed with 0.1% TFA and glycopeptides eluted with 20% ACN and 0.1% TFA. Tryptic glycopeptides were dried by vacuum centrifugation and dissolved in 20 µL of ultrapure water.

#### LC–ESI-MS/MS Analysis of IgG Tryptic Glycopeptides

For the KORA F4 study, tryptic glycopeptides were analyzed on nanoACQUITY UPLC system (Waters, USA) coupled to Compact mass spectrometer (Bruker Daltonics, Germany) *via* a capillary electrophoresis electrospray (ESI) interface (Agilent Technologies, Santa Clara, CA, USA). A sheath liquid (50% isopropanol, 20% proprionic acid) was pumped at a flow rate of 2 µL/min. Nine µL of IgG tryptic glycopeptides was loaded on Acclaim PepMap100 C8 (5 mm × 300 μm i.d.) trap column. The glycopeptides were washed 1 min with 0.1% TFA (solvent A) at a flow rate of 40 µL/min and separated on an HALO C18 nanoLC column (50 mm × 75 μm i.d., 2.7 µm HALO fused core particles) (Advanced Materials Technology, USA) at 30°C, using a 3.5 min gradient from 19 to 25% solvent B (80% ACN) at 1 µL/min flow rate. Mass spectra were acquired from 500 to 2,000 *m*/*z* units with two averages at a frequency of 0.5 Hz. The quadrupole ion energy and collision energy were set to 4 eV. NanoACQUITY UPLC system was operated under MassLynx software version 4.1 and the Bruker micrOTOF-Q was operated under HyStar software, version 3.2. Data extraction was performed using an in-house Python script. In short, data were *m*/*z* recalibrated based on a subset of hand-picked analytes having a high signal-to-noise ratio and the expected isotopic distribution. Intensities for the top four isotopologues were extracted using a 10 ppm *m*/*z* window. Retention times were aligned toward the cohort median and retention time bins were determined for the analytes. All of the signals belonging to a single analyte for every sample were summed up.

For the LLS study, the IgG glycopeptide samples were analysed using an Ultimate 3000 RSLCnano liquid chromatography system (Dionex, Sunnyvale, CA, USA) coupled to a Maxis Impact quadrupole time-of-flight-MS (micOTOF-Q, Bruker Daltonics), as described previously (57). Samples were run over a trap column (Acclaim PepMap100 C18, 5 mm × 300 µm i.d., Dionex, Sunnyvale, CA, USA) and a separation column (Ascentis Express C18 nanoLC, 50 mm× 75 µm i.d., 2.7 µm HALO fused core particles; Supelco, Bellefonte, PA, USA). A linear gradient was used with a flow rate of 0.9 µL/min, with solvent A consisting of 0.1% TFA and B of 95% ACN: *t* = 0, 3% solvent B; *t* = 2, 6%; *t* = 4.5, 18%; *t* = 5, 30%; *t* = 7, 30%; *t* = 8, 0%; *t* = 11, 0%. The LC was coupled to the MS *via* a sheath-flow electrospray (ESI) interface (Agilent Technologies, Santa Clara, CA, USA). A sheath flow, consisting of 50% isopropanol, 20% proprionic acid, and 30% MilliQ-purified water, was applied with a flow rate of 2 µL/min, along with nitrogen gas at 4 L/min. Mass spectra were acquired within an *m*/*z* range of 600–2,000 at a frequency of 0.5 Hz. LC–MS data were examined and calibrated using Compass Data Analysis 4.2 (Bruker Daltonics), and retention time alignment was done using Msalign. In-house developed software Xtractor 2D (see http://ms-utils.org/Xtractor/) was used to extract signal intensity data. For each type of glycopeptide, the background-subtracted signal intensity of the first three isotopic peaks in both 2+ and 3+ charge state were summed.

For the following analyses we used the most prominent measureable 20 glycopeptides in subclasses IgG1 and IgG2 (a mixture of IgG2 and IgG3 in KORA F4, IgG2 only in LLS) and the most prominent and identifiable 10 fucosylated glycopeptides in IgG4, since peaks belonging to afucosylated IgG4 glycans overlapped with those of earlier eluting and much higher abundant IgG1 glycans.

### Preprocessing of IgG Glycopeptides

Glycosylation is highly differing between individuals. Absolute values of peaks obtained by the LC–ESI-MS method are not comparable. We normalize glycopeptides per subclass by total area normalization as defined in the R-package “glycanr” (R-package version 0.3.0) (58), taking their relative abundance within subclasses as phenotypes and input variables for ratios. Batch correction per subclass was performed with the ComBat (59) algorithm of the R-package “sva” (R-package version 3.14.0) (60). To meet the assumptions for ComBat batch correction, samples were log-transformed before applying the algorithm and exponentiated afterward to regain the original scale. Derived traits have been computed from batch corrected glycan measurements.

Summarizing derived traits per subclass were computed as described in S1 using the ildt function from glycanr package (58). Ratios within subclasses were defined as product over substrate for all possible one-step reactions in the pathways, based on the assumption that single sugar molecules can only be added and not removed (61). Ratios between subclasses were calculated as described in Data Sheet S1 in Supplementary Material. Here, we do not assume an actual product-substrate relationship. For ratios including glycopeptides traits from IgG4, we renormalized the glycopeptide traits on all corresponding fucosylated traits only. All ratios were log-transformed.

For the glycan proportions, i.e., normalization per specific glycopeptide trait in total Fc IgG glycopeptides, we also used the total area normalized traits as input. We only calculated per glycan normalization for core-fucosylated glycopeptides as others are not available for IgG4. In addition, we computed the sums per IgG subclass as ratios of these sums. Again, the ratios were log-transformed before any further analyses. For the discovery cohort, characteristics of all IgG glycan traits can be found in Table S2 in Supplementary Material.

### IgG Glycan Traits

With LC–ESI-MS, 50 initial glycopeptides from different IgG subclasses were measured and quantified. For IgG1 and IgG2 (IgG2/3 in KORA), 20 initial glycopeptides are available, for IgG4, only glycopeptides with core-fucosylation (10 glycopeptides) were measured.

From these 50 initial glycopeptides, summarizing traits per subclass were derived [as seen in Table S1 in Supplementary Material and in Ref. (24)], including, e.g., “Percentage of IgG1 Fucosylation” (sum of all fucosylated glycan traits in IgG) or the “ratio of afucosylated monosialylated structures with and without bisecting GlcNAc in total IgG1 glycans” (the ratio of sum of afucosylated monosialylated structures with bisecting GlcNAcs over the sum of afucosylated monosialylated structures without bisecting GlcNAcs in total IgG1 glycan traits).

In addition, we included all one-step pathway ratios of product over substrate possible within each subclass, e.g., IgG1_G0F/IgG1_G0 (see Figure S4B in Supplementary Material). The ratios describe reactions that are already known to be part of the IgG glycosylation biosynthesis as well as reactions that can be derived on the assumption of the addition of one monosaccharide at a time, but which are hitherto unknown.

To analyze differing glycosylation pathways for the subclasses, we included ratios of glycopeptides across subclasses in our analysis, e.g., IgG1_G0/IgG2_G0. Glycopeptide traits being used for ratios with IgG4 were additionally normalized on their respective fucosylated glycopeptides only.

For detecting genetic influence on the abundances of the IgG subclasses, we additionally normalized the traits “per glycan” [e.g., IgG1_G0F/(IgG1_G0F + IgG2_G0F + IgG4_G0F)] and included the newly normalized glycopeptides, the subclass-specific sums, and ratios thereof in the analyses. These traits describe the relative abundance of the attached glycopeptide structure for the specific subclass.

All traits declared as “ratios” were log-transformed before any statistical analysis.

For a complete list of all 376 phenotypic traits, please see Table S1 in Supplementary Material.

### Genome-Wide Association Analysis and Meta-analysis

We performed a genome-wide association analysis in the discovery cohort, KORA F4. First, each phenotype (see “IgG glycan traits” for explanation) was adjusted for sex and age and regression residuals were inverse normal rank transformed to assure normal distribution. The transformed residuals were used for association analysis in a linear model performed with snptest v2.5.1 software (62) using an additive genetic model.

The threshold determining the suggestive SNPs was set to 5 × 10^−8^. Given that we performed 376 different GWASs, the genome-wide significant threshold for the discovery was additionally adjusted for the number of initially measured glycopeptide traits and thus set to 1 × 10^−9^ (5 × 10^−8^/50 initially measured glycopeptides). All derived traits and different ratios are a function of initially measured glycan traits and are therefore dependent on these traits. We acknowledge that there might be less independent traits within initially measured glycans, but we decided to be more conservative and correct for 50 tests.

For the LLS cohort, we computed linear regression models for the suggestive associations from the discovery only. Similar to the discovery cohort, each trait was adjusted for sex and age first, and the obtained residuals were normally rank transformed. For LLS, a score test including family information (63) was conducted with the C++ program QTassoc [see http://www.lumc.nl/uh, under GWAS Software (63)]. The Bonferroni-corrected replication threshold was set to 2.33 × 10^−6^ (0.05/number of replicated associations).

For all replicated trait-SNP associations (*p* < 2.33 × 10^−6^), we additionally performed an inverse variance-weighted fixed effects meta-analysis on the summary statistics of the two cohorts. The meta-analysis was performed using the software METAL (64). We included similar traits for all three IgG subclasses. Based on the results from the meta-analysis, we performed *t*-tests to determine statistical significant differences between the same traits for the different IgG subclasses. In the subclass comparisons, we included all glycopeptide traits being available in at least two of the subclasses. We then analyzed parallels between the significantly different glycopeptide traits for the IgG subclasses and the replicated associations for ratios of IgG glycopeptides from different subclasses.

### Relation of Replicated SNPs

For all replicated SNPs, we obtain the information on their LD from SNiPA (65) (last update version from November 2015, setting: GRCh 37, 1000 Genomes Phase 3 v5, European, Ensembl82). As for some variants, LD information is missing, we next generated LD-blocks of replicated markers. All variants in LD with *r*^2^ ≥ 0.5 were assigned to the same LD-block. Hereby, SNPs could be assigned to more than one LD-block. We then merged LD-blocks that were overlapping position-wise (see Figures S5A–F in Supplementary Material). This step takes care of SNPs for which no LD information was available but which are still situated in a highly heritable genetic region. The remaining (now larger) LD blocks can thus be clearly separated by positions on the chromosome and can be more easily observed by specific regional plots (see Figure S6 in Supplementary Material). This approach helps to summarize variants within loci. However, it does not account for functional similarity between markers or their relevance on IgG glycopeptide traits.

Information on the position of SNPs and their genetic features are obtained from the UCSC Genome Browser on Human [February 2009 (GRCh37/hg19)] (66).

For replicated SNPs on chromosome 1, we additionally performed multivariate linear models with the data from the discovery cohort, KORA F4, and settings as in the original model. For associated glycopeptide traits, models included one up to all three of the replicated SNPs on chromosome 1. We compared the significance of the single SNPs in the joint models as well as the added explained variance of the glycan traits.

## Ethics Statement

The KORA study was carried out in accordance with the recommendations of good clinical practice guidelines and the guidelines of the KORA study group, with written informed consent from all subjects. All subjects gave written informed consent in accordance with the Declaration of Helsinki. The protocol was approved by the KORA study group. The LLS study was carried out in accordance with the recommendations of good clinical practice guidelines and the guidelines of the medical ethics committee of the Leiden University Medical Center, with written informed consent from all subjects. All subjects gave written informed consent in accordance with the Declaration of Helsinki. The protocol was approved by the medical ethics committee of the Leiden University Medical Center.

## Author Contributions

Performed wet lab experiments and data extraction: JŠ, IT-A, and GR, and RP. Performed data cleaning: LK, FV, EA, AW, JD, and MB. Performed GWAS: AW and EA. Analyzed the data: AW, EA, LK, JŠ, IT-A, GR, and EB. Contributed data/reagents/material/analysis tools: MB, JD, DH, RP, HG, JK, KS, AP, TM, MW, PS, GL, and CG. Supervised the research: M, CH, PS, GL, and CG. Wrote the manuscript: AW, EA, and LK. All the authors discussed the results and reviewed the final manuscript.

## Conflict of Interest Statement

GL is the founder and owner of Genos Ltd., a private research organization that specializes in high-throughput glycomic analysis and has several patents in this field. LK, JŠ, IT-A, GR, and FV are employees of Genos Ltd.

## Supplementary Material

Figure S1 Mirrored Manhattan plot of all suggestive (*p* < 5 × 10^−8^) and replicated (*p* < 2.33 × 10^−6^) SNPs with their minimal *p*-values for any association.

Figure S2 Network of significantly different associated IgG glycopeptide traits and SNPs summarized by linkage disequilibrium-blocks IgG glycan traits (circles) for different subclass comparisons (red: IgG1 vs. IgG4; light green: IgG1 vs. IgG2; blue: IgG2 vs. IgG4; purple: glycan proportions for IgG4; and green: glycan proportions for IgG2) and their associations to the replicated loci (edges only for replicated results).

Figure S3 **(A)** QQ-Plot (−log10(*p*-values)) for comparison of results from initial glycopeptide traits and from between-subclass ratios and glycan proportions (results are obtained from the discovery cohort only). **(B)** QQ-Plot (−log10(*p*-values)) for comparison of results from all known pathway steps and additionally suggested pathway steps (known relations include associations as displayed in Figures S4; predicted relation includes associations represented with dashed lines in Figures S4; results are obtained from the meta-analysis).

Figure S4 Enzymatic pathway steps in IgG glycosylation. (Colors indicate the enzymes responsible for the attachment of monosaccharides.) **(A)** Known enzymatic pathway steps in IgG glycosylation (the glycosylation pathways are assumed to be the same for all IgG subclasses). **(B)** Known and suggested (dashed) one-step enzymatic pathway steps in IgG glycosylation (the glycosylation pathways are assumed to be the same for all IgG subclasses). **(C)** Replicated associations for IgG1 within the IgG glycan synthesis. **(D)** Replicated associations for IgG2 (IgG2/3) within the IgG glycan synthesis. **(E)** Replicated associations for IgG4 within the IgG glycan synthesis.**(F)** Replicated associations for between-subclass ratios and glycan proportions as well as significant subclass comparisons based on meta-analyzed data.

Figure S5 **(A–G)** Representation of the linkage disequilibrium-blocks summarizing the replicated SNPs for each gene region. **(A)** *RUNX3* (chr.1), **(B)** *ST6GAL1* (chr.3), **(C)** *IKZF1* (chr.7), **(D)** *B4GALT1* (chr.9), **(E)** *FUT8* (chr.14), **(F)** *SMARCB1-DERL3* (chr.22), and **(G)** *MGAT3* (chr.22).

Figure S6 **(A–G)** Regional association plots for SNPs and their lowest *p*-values for any association for each linkage disequilibrium (LD)-block (only if LD information was available). **(A)** *RUNX3* (chr.1), LD-block 1, [**(B)**, i–iv] *ST6GAL1* (chr.3), LD-block 1–LD-block 4, **(C)** *IKZF1* (chr.7), LD-block 1, [**(D)** i, ii] *B4GALT1* (chr.9), LD-block 1, and LD-block 2, [**(E)**, i, ii] *FUT8* (chr.14), LD-block 1, and LD-block 2, [**(F)**, i, ii] *SMARCB1-DERL3* (chr.22), LD-block 1, and LD-block 2, and [**(G)**, i–iii] *MGAT3* (chr.22), LD-block 2–LD-block 4.

Table S1 Description of immunoglobulin G (IgG) glycopeptide traits.

Table S2 Study characteristics of the discovery cohort KORA.

Table S3 Explained variance of the immunoglobulin G (IgG) glycopeptide traits by the single SNPs in KORA.

Table S4 List of association in KORA F4 to SNPs excluded for the replication due to unavailability in Leiden Longevity Study.

Table S5 List of all replicated associations.

Table S6 List of replicated phenotypic traits for each gene region.

Table S7 Complete list of results for subclass comparisons of immunoglobulin G (IgG) glycopeptide traits.

Table S8 Overview of results from subclass comparisons of immunoglobulin G (IgG) glycopeptide traits.

Table S9 Results from joint linear models for replicated SNPs on chromosome 1.

Table S10 Comparison of ultra-performance liquid chromatography (UPLC)-measured and LC/MS-measured immunoglobulin G (IgG) glycan traits [adapted from Huffman et al. (24)].

Table S11 Replicated association and comparison to the study by Lauc et al. (21).

Table S12 Summary of replicated association and comparison to study by Lauc et al. (21).

Table S13 Confirmation of loci reported in Lauc et al. (21).
