# Supplementary figures and images for: Genome-Wide Association Study on Immunoglobulin G Glycosylation Patterns
Source: Front Immunol. 2018 Feb 26;9:277. doi: 10.3389/fimmu.2018.00277 (PMC5834439; doi:10.3389/fimmu.2018.00277)

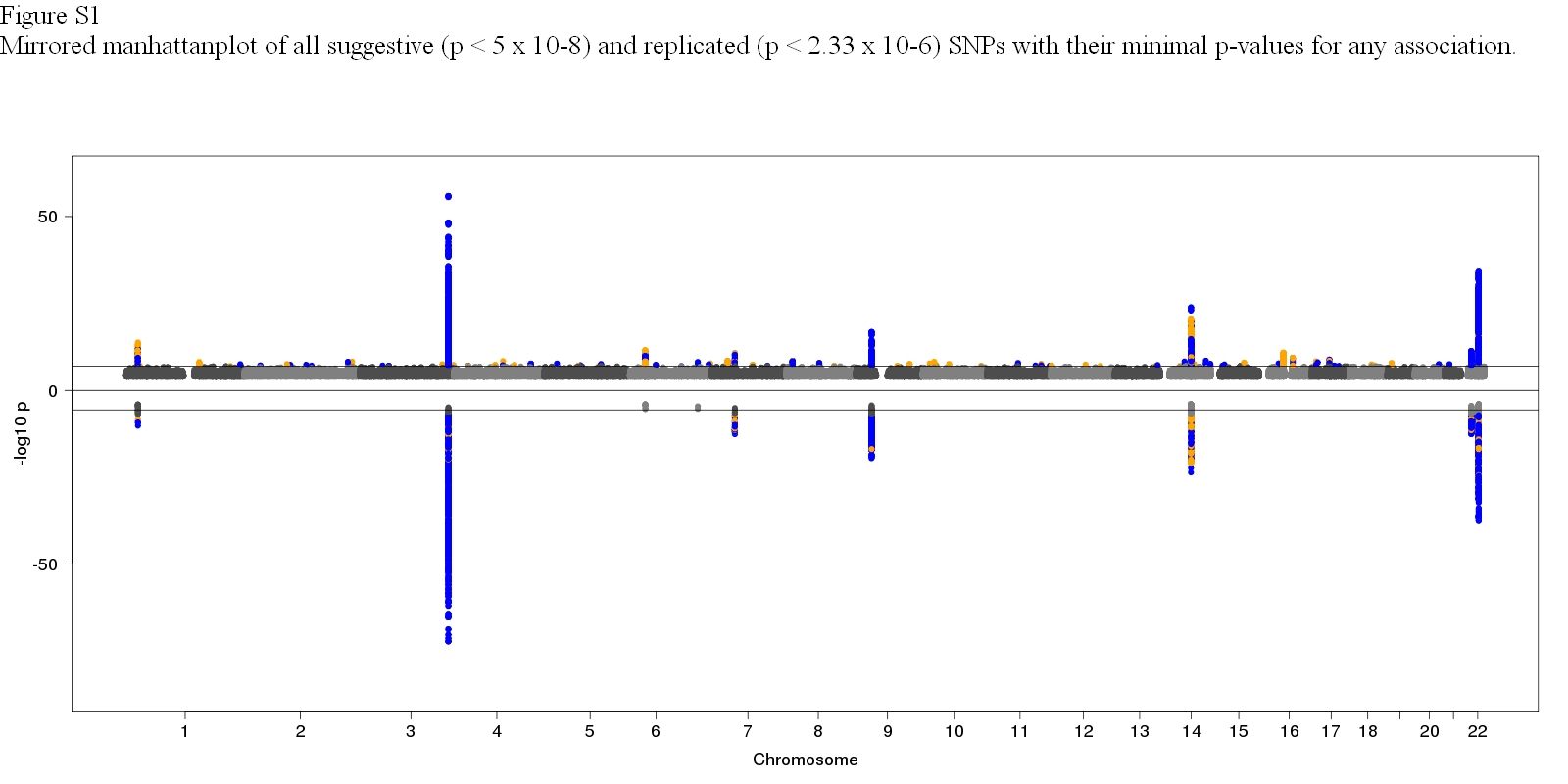

Supplement: Figure S1 — Mirrored Manhattan plot of all suggestive (p < 5 × 10^−8^) and replicated (p < 2.33 × 10^−6^) SNPs with their minimal p-values for any association. [file Image_1.jpeg]

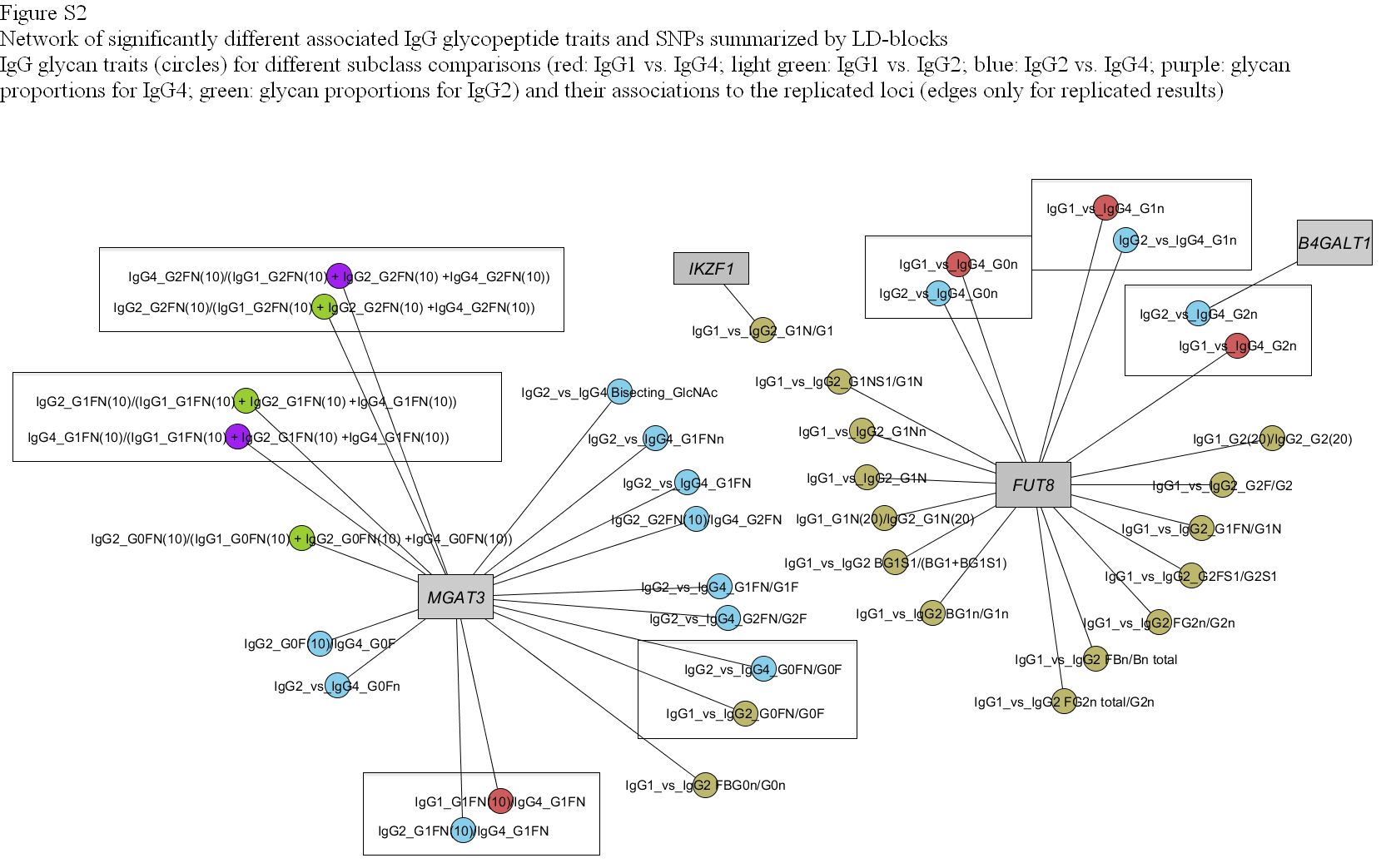

Supplement: Figure S2 — Network of significantly different associated IgG glycopeptide traits and SNPs summarized by linkage disequilibrium-blocks IgG glycan traits (circles) for different subclass comparisons (red: IgG1 vs. IgG4; light green: IgG1 vs. IgG2; blue: IgG2 vs. IgG4; purple: glycan proportions for IgG4; and green: glycan proportions for IgG2) and their associations to the replicated loci (edges only for replicated results). [file Image_2.jpeg]

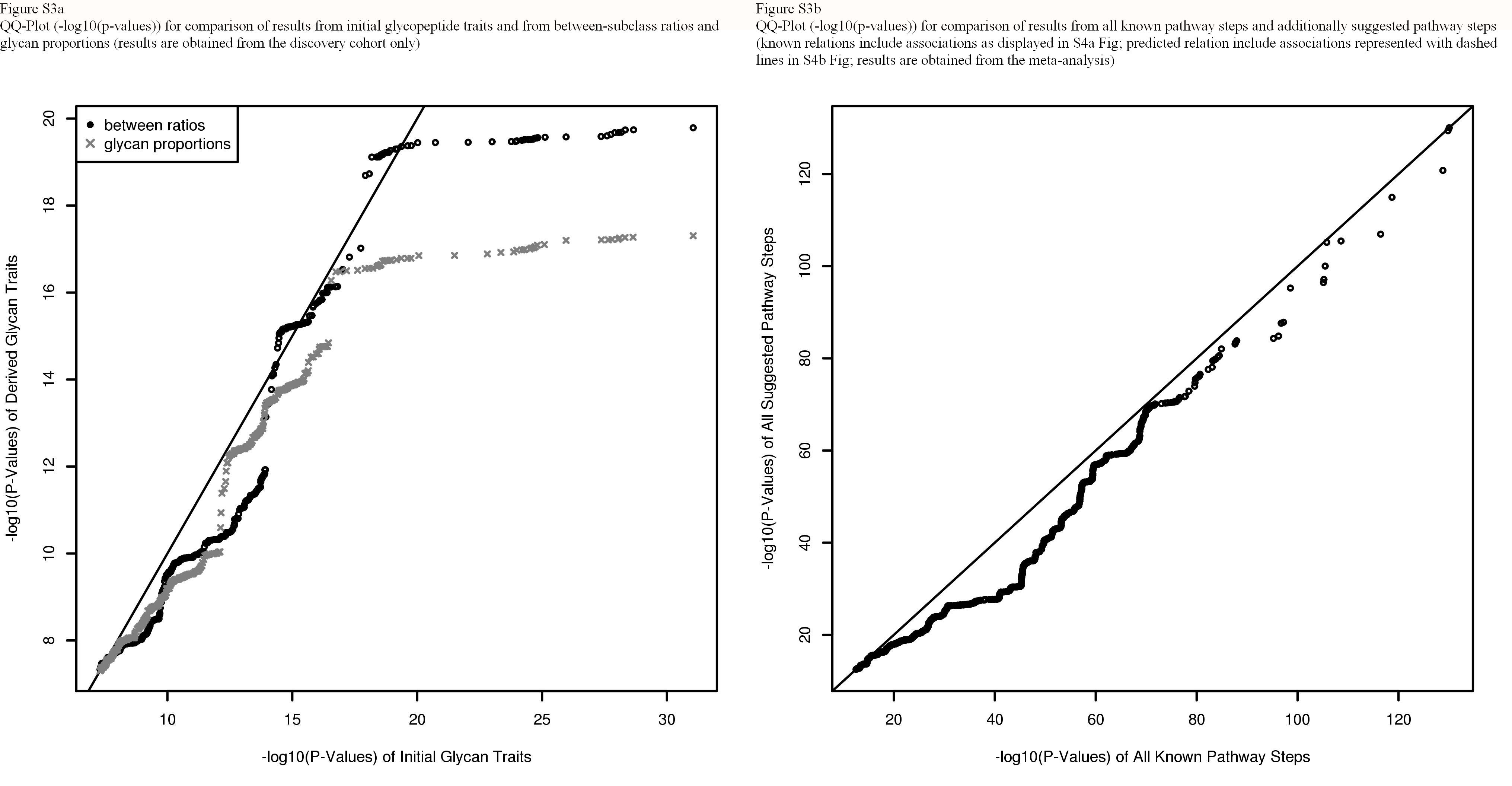

Supplement: Figure S3 — (A) QQ-Plot (−log10(p-values)) for comparison of results from initial glycopeptide traits and from between-subclass ratios and glycan proportions (results are obtained from the discovery cohort only). (B) QQ-Plot (−log10(p-values)) for comparison of results from all known pathway steps and additionally suggested pathway steps (known relations include associations as displayed in Figures S4; predicted relation includes associations represented with dashed lines in Figures S4; results are obtained from the meta-analysis). [file Image_3.jpeg]

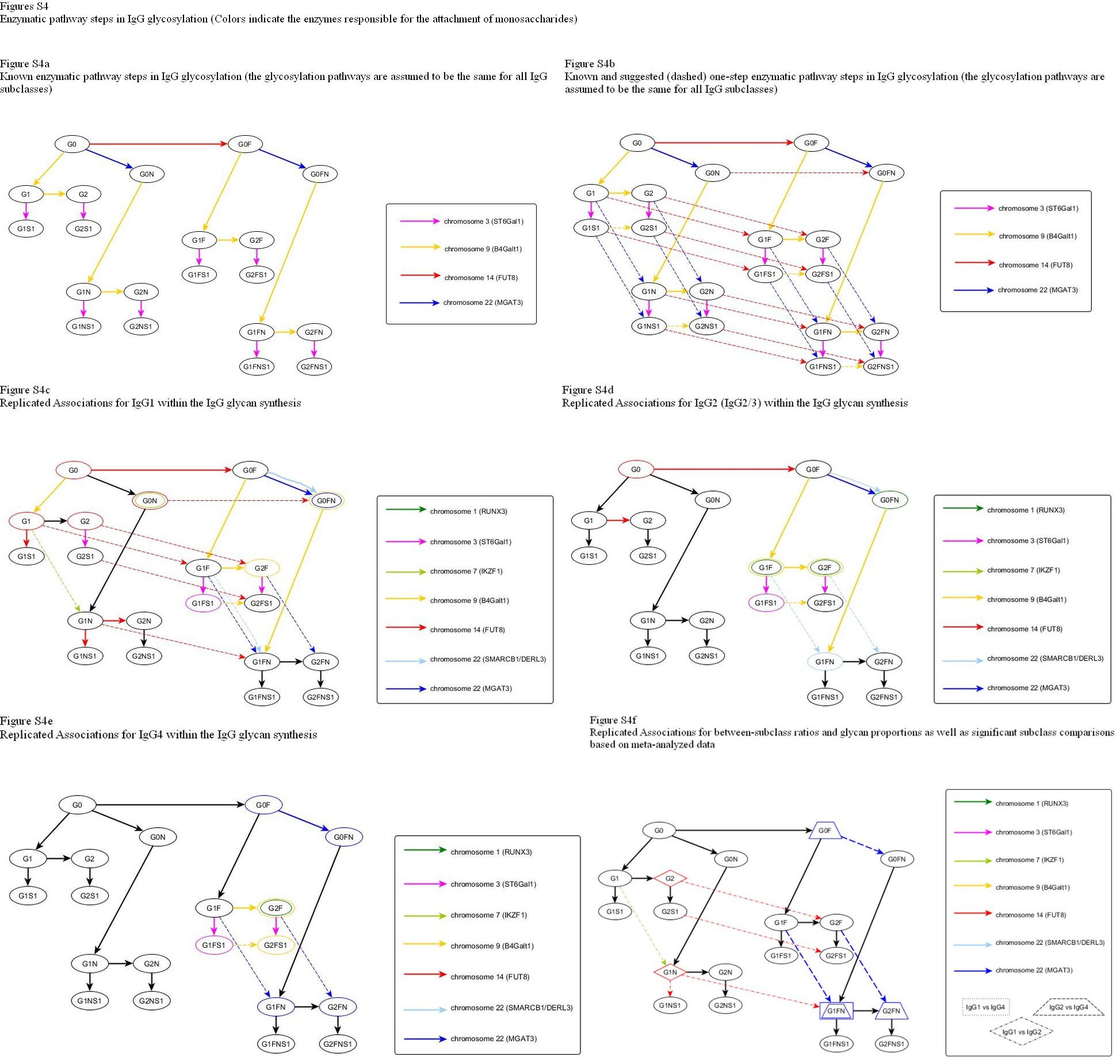

Supplement: Figure S4 — Enzymatic pathway steps in IgG glycosylation. (Colors indicate the enzymes responsible for the attachment of monosaccharides.) (A) Known enzymatic pathway steps in IgG glycosylation (the glycosylation pathways are assumed to be the same for all IgG subclasses). (B) Known and suggested (dashed) one-step enzymatic pathway steps in IgG glycosylation (the glycosylation pathways are assumed to be the same for all IgG subclasses). (C) Replicated associations for IgG1 within the IgG glycan synthesis. (D) Replicated associations for IgG2 (IgG2/3) within the IgG glycan synthesis. (E) Replicated associations for IgG4 within the IgG glycan synthesis.(F) Replicated associations for between-subclass ratios and glycan proportions as well as significant subclass comparisons based on meta-analyzed data. [file Image_4.jpeg]

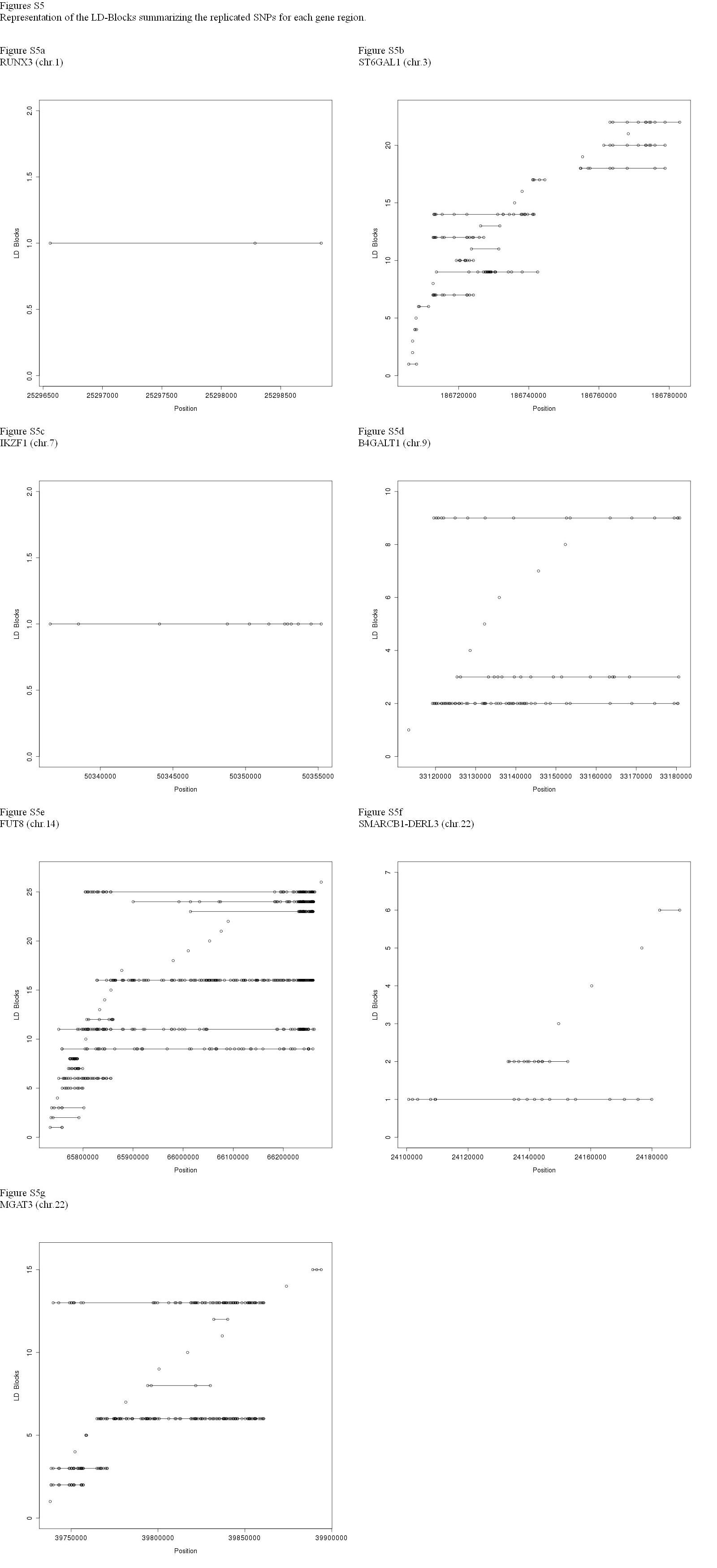

Supplement: Figure S5 — (A–G) Representation of the linkage disequilibrium-blocks summarizing the replicated SNPs for each gene region. (A) RUNX3 (chr.1), (B) ST6GAL1 (chr.3), (C) IKZF1 (chr.7), (D) B4GALT1 (chr.9), (E) FUT8 (chr.14), (F) SMARCB1-DERL3 (chr.22), and (G) MGAT3 (chr.22). [file Image_5.jpeg]

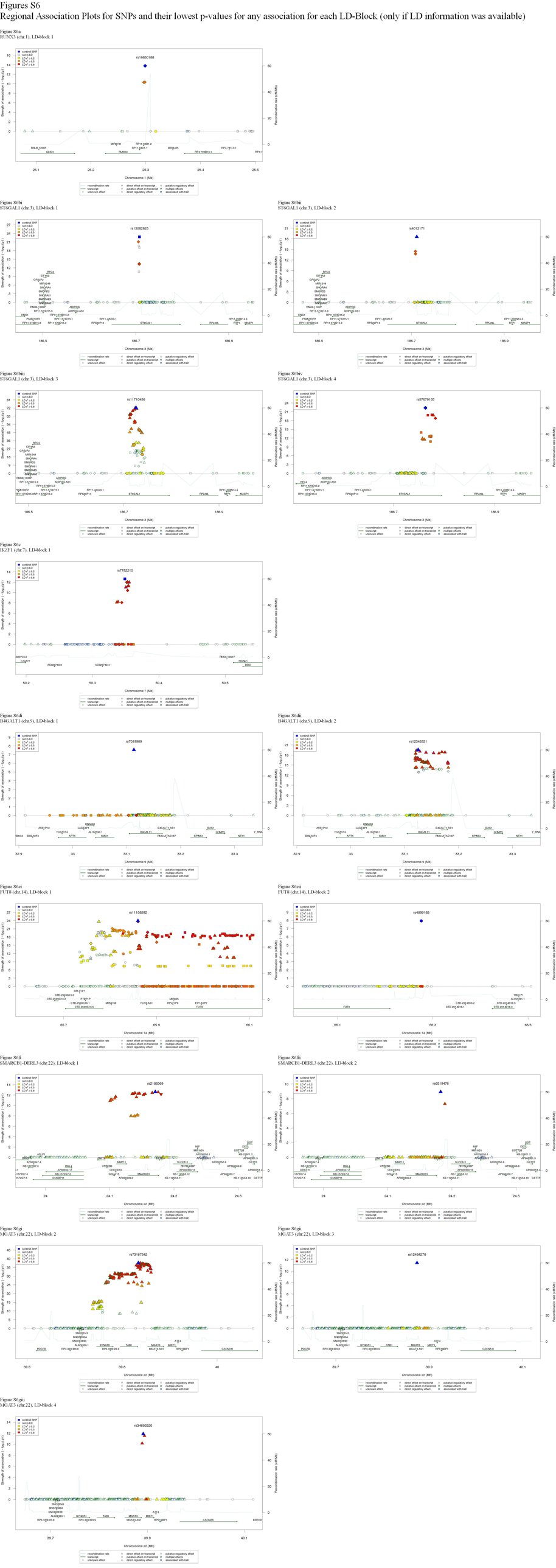

Supplement: Figure S6 — (A–G) Regional association plots for SNPs and their lowest p-values for any association for each linkage disequilibrium (LD)-block (only if LD information was available). (A) RUNX3 (chr.1), LD-block 1, [(B), i–iv] ST6GAL1 (chr.3), LD-block 1–LD-block 4, (C) IKZF1 (chr.7), LD-block 1, [(D) i, ii] B4GALT1 (chr.9), LD-block 1, and LD-block 2, [(E), i, ii] FUT8 (chr.14), LD-block 1, and LD-block 2, [(F), i, ii] SMARCB1-DERL3 (chr.22), LD-block 1, and LD-block 2, and [(G), i–iii] MGAT3 (chr.22), LD-block 2–LD-block 4. [file Image_6.jpeg]
