# Supplementary material for: Genome-Wide Association Study on Immunoglobulin G Glycosylation Patterns
Source: Front Immunol. 2018 Feb 26;9:277. doi: 10.3389/fimmu.2018.00277 (PMC5834439; doi:10.3389/fimmu.2018.00277)
Supplement: Table S1 — Description of immunoglobulin G (IgG) glycopeptide traits. [file Table_1.PDF]

IgG Glycan Traits

Group	GWAS Code	Trait	Description	Formula
**Initial IgG1 Glycan Traits**				
Initial IgG1 glycans (total)	**LC_IGP1**	IgG1_G0F	*The percentage of G0F glycan in total IgG1 glycans*	
	**LC_IGP2**	IgG1_G1F	*The percentage of G1F glycan in total IgG1 glycans*	
	**LC_IGP3**	IgG1_G2F	*The percentage of G2F glycan in total IgG1 glycans*	
	**LC_IGP4**	IgG1_G0FN	*The percentage of G0FN glycan in total IgG1 glycans*	
	**LC_IGP5**	IgG1_G1FN	*The percentage of G1FN glycan in total IgG1 glycans*	
	**LC_IGP6**	IgG1_G2FN	*The percentage of G2FN glycan in total IgG1 glycans*	
	**LC_IGP7**	IgG1_G1FS1	*The percentage of G1FS1 glycan in total IgG1 glycans*	
	**LC_IGP8**	IgG1_G2FS1	*The percentage of G2FS1 glycan in total IgG1 glycans*	
	**LC_IGP9**	IgG1_G1FNS1	*The percentage of G1FNS1 glycan in total IgG1 glycans*	
	**LC_IGP10**	IgG1_G2FNS1	*The percentage of G2FNS1 glycan in total IgG1 glycans*	
	**LC_IGP11**	IgG1_G0	*The percentage of G0 glycan in total IgG1 glycans*	
	**LC_IGP12**	IgG1_G1	*The percentage of G1 glycan in total IgG1 glycans*	
	**LC_IGP13**	IgG1_G2	*The percentage of G2 glycan in total IgG1 glycans*	
	**LC_IGP14**	IgG1_G0N	*The percentage of G0N glycan in total IgG1 glycans*	
	**LC_IGP15**	IgG1_G1N	*The percentage of G1N glycan in total IgG1 glycans*	
	**LC_IGP16**	IgG1_G2N	*The percentage of G2N glycan in total IgG1 glycans*	
	**LC_IGP17**	IgG1_G1S1	*The percentage of G1S1 glycan in total IgG1 glycans*	
	**LC_IGP18**	IgG1_G2S1	*The percentage of G2S1 glycan in total IgG1 glycans*	
	**LC_IGP19**	IgG1_G1NS1	*The percentage of G1NS1 glycan in total IgG1 glycans*	
	**LC_IGP20**	IgG1_G2NS1	*The percentage of G2NS1 glycan in total IgG1 glycans*	
**Summarizing IgG1 Traits**				
Summarizing IgG1 glycans (total)	**LC_IGP21**	IgG1 Fucosylation	*The percentage of IgG1 core fucosylation*	*SUM(G0F+G1F+G2F+G0FN+G1FN+G2FN+G1FS1+G2FS1+G1FNS1+G2FNS1)*
	**LC_IGP22**	IgG1 Bisecting_GlcNAc	*The incidence of bisecting GlcNAc of IgG1*	*SUM(G0FN+G1FN+G2FN+G1FNS1+G2FNS1+G0N+G1N+G2N+G1NS1+G2NS1)*
	**LC_IGP23**	IgG1 Galactosylation	*The percentage of IgG1 galactosylation*	*SUM(G1F+G1FN+G1FS1+G1FNS1+G1+G1N+G1S1+G1NS1)*0.5+SUM(G2F+G2FN+G2FS1+G2FNS1+G2+G2N+G2S1+G2NS1)*
	**LC_IGP24**	IgG1 Sialylation	*The percentage of IgG1 sialylation*	*SUM(G1FS1+G2FS1+G1FNS1+G2FNS1+G1S1+G2S1+G1NS1+G2NS1)*
	**LC_IGP25**	IgG1 SA per Gal	*The number of sialic acid moieties on galactose moieties in total IgG1 glycans*	*IgG1 Sialylation/IgG1 Galactosylation*
	**LC_IGP26**	IgG1 GS1/(G+GS1)	*The percentage of monosialylation of afucosylated galactosylated structures without bisecting GlcNAc in total IgG1 glycans*	*SUM(G1S1+G2S1)/SUM(G1+G1S1+G2+G2S1)*100*
	**LC_IGP27**	IgG1 GS1/(G0+G+GS1)	*The percentage of monosialylation of all afucosylated structures without bisecting GlcNAc in total IgG1 glycans*	*SUM(G1S1+G2S1)/SUM(G0+G1+G1S1+G2+G2S1)*100*
	**LC_IGP28**	IgG1 G1S1/(G1+G1S1)	*The percentage of monosialylation of afucosylated monogalactosylated (without bisecting GlcNAc) structures in total IgG1 glycans*	*G1S1/SUM(G1+G1S1)*100*
	**LC_IGP29**	IgG1 G2S1/(G2+G2S1)	*The percentage of monosialylation of afucosylated digalactosylated (without bisecting GlcNAc) structures in total IgG1 glycans*	*G2S1/SUM(G2+G2S1)*100*
	**LC_IGP30**	IgG1 BGS1/(BG+BGS1)	*The percentage of monosialylation of afucosylated galactosylated structures with bisecting GlcNAc in total IgG1 glycans*	*SUM(G1NS1+G2NS1)/SUM(G1N+G1NS1+G2N+G2NS1)*100*
	**LC_IGP31**	IgG1 BGS1/(BG0+BG+BGS1)	*The percentage of monosialylation of all afucosylated structures with bisecting GlcNAc in total IgG1 glycans*	*SUM(G1NS1+G2NS1)/SUM(G0N+G1N+G1NS1+G2N+G2NS1)*100*
	**LC_IGP32**	IgG1 BG1S1/(BG1+BG1S1)	*The percentage of monosialylation of afucosylated monogalactosylated (with bisecting GlcNAc) structures in total IgG1 glycans*	*G1NS1/SUM(G1N+G1NS1)*100*
	**LC_IGP33**	IgG1 BG2S1/(BG2+BG2S1)	*The percentage of monosialylation of afucosylated digalactosylated (with bisecting GlcNAc) structures in total IgG1 glycans*	*G2NS1/SUM(G2N+G2NS1)*100*
	**LC_IGP34**	IgG1 FGS1/(FG+FGS1)	*The percentage of monosialylation of fucosylated galactosylated structures without bisecting GlcNAc in total IgG1 glycans*	*SUM(G1FS1+G2FS1)/SUM(G1F+G1FS1+G2F+G2FS1)*100*
	**LC_IGP35**	IgG1 FGS1/(F+FG+FGS1)	*The percentage of monosialylation of all fucosylated structures without bisecting GlcNAc in total IgG1 glycans*	*SUM(G1FS1+G2FS1)/SUM(G0F+G1F+G1FS1+G2F+G2FS1)*100*
	**LC_IGP36**	IgG1 FG1S1/(FG1+FG1S1)	*The percentage of monosialylation of fucosylated monogalactosylated (without bisecting GlcNAc) structures in total IgG1 glycans*	*G1FS1/SUM(G1F+G1FS1)*100*
	**LC_IGP37**	IgG1 FG2S1/(FG2+FG2S1)	*The percentage of monosialylation of fucosylated digalactosylated (without bisecting GlcNAc) structures in total IgG1 glycans*	*G2FS1/SUM(G2F+G2FS1)*100*
	**LC_IGP38**	IgG1 FBGS1/(FBG+FBGS1)	*The percentage of monosialylation of fucosylated galactosylated structures with bisecting GlcNAc in total IgG1 glycans*	*SUM(G1FNS1+G2FNS1)/SUM(G1FN+G1FNS1+G2FN+G2FNS1)*100*
	**LC_IGP39**	IgG1 FBGS1/(FB+FBG+FBGS1)	*The percentage of monosialylation of all fucosylated structures with bisecting GlcNAc in total IgG1 glycans*	*SUM(G1FNS1+G2FNS1)/SUM(G0FN+G1FN+G1FNS1+G2FN+G2FNS1)*100*
	**LC_IGP40**	IgG1 FBG1S1/(FBG1+FBG1S1)	*The percentage of monosialylation of fucosylated monogalactosylated (with bisecting GlcNAc) structures in total IgG1 glycans*	*G1FNS1/SUM(G1FN+G1FNS1)*100*
	**LC_IGP41**	IgG1 FBG2S1/(FBG2+FBG2S1)	*The percentage of monosialylation of fucosylated digalactosylated (with bisecting GlcNAc) structures in total IgG1 glycans*	*G2FNS1/SUM(G2FN+G2FNS1)*100*
	**LC_IGP42**	IgG1 BS1/S1	*Ratio of afucosylated monosialylated structures with and without bisecting GlcNAc in total IgG1 glycans*	*SUM(G1NS1+G2NS1)/SUM(G1S1+G2S1)*
	**LC_IGP43**	IgG1 FBS1/FS1	*Ratio of fucosylated monosialylated structures with and without bisecting GlcNAc in total IgG1 glycans*	*SUM(G1FNS1+G2FNS1)/SUM(G1FS1+G2FS1)*
	**LC_IGP44**	IgG1 BS1/(S1+BS1)	*The incidence of bisecting GlcNAc in all afucosylated monosialylated structures in total IgG1 glycans*	*SUM(G1NS1+G2NS1)/SUM(G1S1+G1NS1+G2S1+G2NS1)*
	**LC_IGP45**	IgG1 FBS1/(FS1+FBS1)	*The incidence of bisecting GlcNAc in all fucosylated monosialylated structures in total IgG1 glycans*	*SUM(G1FNS1+G2FNS1)/SUM(G1FS1+G1FNS1+G2FS1+G2FNS1)*
IgG1 glycans (neutral)	**LC_IGP46**	IgG1_G0Fn	*The percentage of G0F glycan in neutral IgG1 glycans*	
	**LC_IGP47**	IgG1_G1Fn	*The percentage of G1F glycan in neutral IgG1 glycans*	
	**LC_IGP48**	IgG1_G2Fn	*The percentage of G2F glycan in neutral IgG1 glycans*	
	**LC_IGP49**	IgG1_G0FNn	*The percentage of G0FN glycan in neutral IgG1 glycans*	
	**LC_IGP50**	IgG1_G1FNn	*The percentage of G1FN glycan in neutral IgG1 glycans*	
	**LC_IGP51**	IgG1_G2FNn	*The percentage of G2FN glycan in neutral IgG1 glycans*	
	**LC_IGP52**	IgG1_G0n	*The percentage of G0 glycan in neutral IgG1 glycans*	
	**LC_IGP53**	IgG1_G1n	*The percentage of G1 glycan in neutral IgG1 glycans*	
	**LC_IGP54**	IgG1_G2n	*The percentage of G2 glycan in neutral IgG1 glycans*	
	**LC_IGP55**	IgG1_G0Nn	*The percentage of G0N glycan in neutral IgG1 glycans*	
	**LC_IGP56**	IgG1_G1Nn	*The percentage of G1N glycan in neutral IgG1 glycans*	
	**LC_IGP57**	IgG1_G2Nn	*The percentage of G2N glycan in neutral IgG1 glycans*	
Summarizing IgG1 glycans (neutral)	**LC_IGP58**	IgG1 G0n	*The percentage of agalactosylated structures in neutral IgG1 glycan fraction*	*SUM(G0n+G0Fn+G0FNn+G0Nn)*
	**LC_IGP59**	IgG1 G1n	*The percentage of monogalactosylated structures in neutral IgG1 glycan fraction*	*SUM(G1n+G1Fn+G1FNn+G1Nn)*
	**LC_IGP60**	IgG1 G2n	*The percentage of digalactosylated structures in neutral IgG1 glycan fraction*	*SUM(G2n+G2Fn+G2FNn+G2Nn)*
	**LC_IGP61**	IgG1 Fn total	*The percentage of all fucosylated (+/- bisecting GlcNAc) structures in neutral IgG1 glycan fraction*	*SUM(G0Fn+G0FNn+G1Fn+G1FNn+G2Fn+G2FNn)*
	**LC_IGP62**	IgG1 FG0n total/G0n	*The percentage of fucosylation of agalactosylated structures in neutral IgG1 glycan fraction*	*SUM(G0Fn+G0FNn)/G0n*100*
	**LC_IGP63**	IgG1 FG1n total/G1n	*The percentage of fucosylation of monogalactosylated structures in neutral IgG1 glycan fraction*	*SUM(G1Fn+G1FNn)/G1n*100*
	**LC_IGP64**	IgG1 FG2n total/G2n	*The percentage of fucosylation of digalactosylated structures in neutral IgG1 glycan fraction*	*SUM(G2Fn+G2FNn)/G2n*100*
	**LC_IGP65**	IgG1 Fn	*The percentage of fucosylated (without bisecting GlcNAc) structures i in neutral IgG1 glycan fraction*	*SUM(G0Fn+G1Fn+G2Fn)*
	**LC_IGP66**	IgG1 FG0n/G0n	*The percentage of fucosylation (without bisecting GlcNAc) of agalactosylated structures in neutral IgG1 glycan fraction*	*G0Fn/G0n*100*
	**LC_IGP67**	IgG1 FG1n/G1n	*The percentage of fucosylation (without bisecting GlcNAc) of monogalactosylated structures in neutral IgG1 glycan fraction*	*G1Fn/G1n*100*
	**LC_IGP68**	IgG1 FG2n/G2n	*The percentage of fucosylation (without bisecting GlcNAc) of digalactosylated structures in neutral IgG1 glycan fraction*	*G2Fn/G2n*100*
	**LC_IGP69**	IgG1 FBn	*The percentage of fucosylated (with bisecting GlcNAc) structures in neutral IgG1 glycan fraction*	*SUM(G0FNn+G1FNn+G2FNn)*
	**LC_IGP70**	IgG1 FBG0n/G0n	*The percentage of fucosylation (with bisecting GlcNAc) of agalactosylated structures in neutral IgG1 glycan fraction*	*G0FNn/G0n*100*
	**LC_IGP71**	IgG1 FBG1n/G1n	*The percentage of fucosylation (with bisecting GlcNAc) of monogalactosylated structures in neutral IgG1 glycan fraction*	*G1FNn/G1n*100*
	**LC_IGP72**	IgG1 FBG2n/G2n	*The percentage of fucosylation (with bisecting GlcNAc) of digalactosylated structures in neutral IgG1 glycan fraction*	*G2FNn/G2n*100*
	**LC_IGP73**	IgG1 Bn total	*The incidence of bisecting GlcNAc (+/- core Fuc) in neutral IgG1 glycan fraction*	*SUM(G0Nn+G1Nn+G2Nn+G0FNn+G1FNn+G2FNn)*
	**LC_IGP74**	IgG1 BG0n total/G0n	*The incidence of bisecting GlcNAc (+/- core Fuc) in agalactosylated structures in neutral IgG1 glycan fraction*	*SUM(G0Nn+G0FNn)/G0n*100*
	**LC_IGP75**	IgG1 BG1n total/G1n	*The incidence of bisecting GlcNAc (+/- core Fuc) in monogalactosylated structures in neutral IgG1 glycan fraction*	*SUM(G1Nn+G1FNn)/G1n*100*
	**LC_IGP76**	IgG1 BG2n total/G2n	*The incidence of bisecting GlcNAc (+/- core Fuc) in digalactosylated structures in neutral IgG1 glycan fraction*	*SUM(G2Nn+G2FNn)/G2n*100*
	**LC_IGP77**	IgG1 Bn	*The incidence of bisecting GlcNAc (without core Fuc) in neutral IgG1 glycan fraction*	*SUM(G0Nn+G1Nn+G2Nn)*
	**LC_IGP78**	IgG1 BG0n/G0n	*The incidence of bisecting GlcNAc (without core Fuc) in agalactosylated structures in neutral IgG1 glycan fraction*	*G0Nn/G0n*100*
	**LC_IGP79**	IgG1 BG1n/G1n	*The incidence of bisecting GlcNAc (without core Fuc) in monogalactosylated structures in neutral IgG1 glycan fraction*	*G1Nn/G1n*100*
	**LC_IGP80**	IgG1 BG2n/G2n	*The incidence of bisecting GlcNAc (without core Fuc) in digalactosylated structures in neutral IgG1 glycan fraction*	*G2Nn/G2n*100*
	**LC_IGP81**	IgG1 Fn/Bn	*Ratio of fucosylated structures without bisecting GlcNAc and afucosylated structures with bisecting GlcNAc in neutral IgG1 glycan fraction*	*Fn/Bn*
	**LC_IGP82**	IgG1 FBn/Fn	*Ratio of fucosylated structures with and without bisecting GlcNAc in neutral IgG1 glycan fraction*	*FBn/Fn*
	**LC_IGP83**	IgG1 FBn/Fn total	*The incidence of bisecting GlcNAc in all fucosylated structures in neutral IgG1 glycan fraction*	*FBn/Fn total*100*
	**LC_IGP84**	IgG1 FBn/Bn total	*The percentage of fucosylation in all structures with bisecting GlcNAc in neutral IgG1 glycan fraction*	*FBn/Bn total*100*

	**LC_IGP85**	IgG1 Fn/Bn total	*Ratio of fucosylated non-bisecting GlcNAc structures and all structures with bisecting GlcNAc in neutral IgG1 glycans fraction*	*Fn/Bn total*
	**LC_IGP86**	IgG1 Bn/Fn total ‰	*Ratio of structures with bisecting GlcNAc and all fucosylated structures (+/- bisecting GlcNAc) in neutral IgG1 glycan fraction*	*Bn/Fn total*1000*
**Initial IgG2 Traits**				
Initial IgG2 glycans (total)	**LC_IGP87**	IgG2_G0F	*The percentage of G0F glycan in total IgG2 glycans*	
	**LC_IGP88**	IgG2_G1F	*The percentage of G1F glycan in total IgG2 glycans*	
	**LC_IGP89**	IgG2_G2F	*The percentage of G2F glycan in total IgG2 glycans*	
	**LC_IGP90**	IgG2_G0FN	*The percentage of G0FN glycan in total IgG2 glycans*	
	**LC_IGP91**	IgG2_G1FN	*The percentage of G1FN glycan in total IgG2 glycans*	
	**LC_IGP92**	IgG2_G2FN	*The percentage of G2FN glycan in total IgG2 glycans*	
	**LC_IGP93**	IgG2_G1FS1	*The percentage of G1FS1 glycan in total IgG2 glycans*	
	**LC_IGP94**	IgG2_G2FS1	*The percentage of G2FS1 glycan in total IgG2 glycans*	
	**LC_IGP95**	IgG2_G1FNS1	*The percentage of G1FNS1 glycan in total IgG2 glycans*	
	**LC_IGP96**	IgG2_G2FNS1	*The percentage of G2FNS1 glycan in total IgG2 glycans*	
	**LC_IGP97**	IgG2_G0	*The percentage of G0 glycan in total IgG2 glycans*	
	**LC_IGP98**	IgG2_G1	*The percentage of G1 glycan in total IgG2 glycans*	
	**LC_IGP99**	IgG2_G2	*The percentage of G2 glycan in total IgG2 glycans*	
	**LC_IGP100**	IgG2_G0N	*The percentage of G0N glycan in total IgG2 glycans*	
	**LC_IGP101**	IgG2_G1N	*The percentage of G1N glycan in total IgG2 glycans*	
	**LC_IGP102**	IgG2_G2N	*The percentage of G2N glycan in total IgG2 glycans*	
	**LC_IGP103**	IgG2_G1S1	*The percentage of G1S1 glycan in total IgG2 glycans*	
	**LC_IGP104**	IgG2_G2S1	*The percentage of G2S1 glycan in total IgG2 glycans*	
	**LC_IGP105**	IgG2_G1NS1	*The percentage of G1NS1 glycan in total IgG2 glycans*	
	**LC_IGP106**	IgG2_G2NS1	*The percentage of G2NS1 glycan in total IgG2 glycans*	
**Summarizing IgG2 Traits**				
Summarizing IgG2 glycans (total)	**LC_IGP107**	IgG2 Fucosylation	*The percentage of IgG2 core fucosylation*	*SUM(G0F+G1F+G2F+G0FN+G1FN+G2FN+G1FS1+G2FS1+G1FNS1+G2FNS1)*
	**LC_IGP108**	IgG2 Bisecting GlcNAc	*The incidence of bisecting GlcNAc of IgG2*	*SUM(G0FN+G1FN+G2FN+G1FNS1+G2FNS1+G0N+G1N+G2N+G1NS1+G2NS1)*
	**LC_IGP109**	IgG2 Galactosylation	*The percentage of IgG2 galactosylation*	*SUM(G1F+G1FN+G1FS1+G1FNS1+G1+G1N+G1S1+G1NS1)*0.5+SUM(G2F+G2FN+G2FS1+G2FNS1+G2+G2N+G2S1+G2NS1)*
	**LC_IGP110**	IgG2 Sialylation	*The percentage of IgG2 sialylation*	*SUM(G1FS1+G2FS1+G1FNS1+G2FNS1+G1S1+G2S1+G1NS1+G2NS1)*
	**LC_IGP111**	IgG2 SA per Gal	*The number of sialic acid moieties on galactose moieties in total IgG2 glycans*	*IgG2 Sialylation/IgG2 Galactosylation*
	**LC_IGP112**	IgG2 GS1/(G+GS1)	*The percentage of monosialylation of afucosylated galactosylated structures without bisecting GlcNAc in total IgG2 glycans*	*SUM(G1S1+G2S1)/SUM(G1+G1S1+G2+G2S1)*100*
	**LC_IGP113**	IgG2 GS1/(G0+G+GS1)	*The percentage of monosialylation of all afucosylated structures without bisecting GlcNAc in total IgG2 glycans*	*SUM(G1S1+G2S1)/SUM(G0+G1+G1S1+G2+G2S1)*100*
	**LC_IGP114**	IgG2 G1S1/(G1+G1S1)	*The percentage of monosialylation of afucosylated monogalactosylated (without bisecting GlcNAc) structures in total IgG2 glycans*	*G1S1/SUM(G1+G1S1)*100*
	**LC_IGP115**	IgG2 G2S1/(G2+G2S1)	*The percentage of monosialylation of afucosylated digalactosylated (without bisecting GlcNAc) structures in total IgG2 glycans*	*G2S1/SUM(G2+G2S1)*100*
	**LC_IGP116**	IgG2 BGS1/(BG+BGS1)	*The percentage of monosialylation of afucosylated galactosylated structures with bisecting GlcNAc in total IgG2 glycans*	*SUM(G1NS1+G2NS1)/SUM(G1N+G1NS1+G2N+G2NS1)*100*
	**LC_IGP117**	IgG2 BGS1/(BG0+BG+BGS1)	*The percentage of monosialylation of all afucosylated structures with bisecting GlcNAc in total IgG2 glycans*	*SUM(G1NS1+G2NS1)/SUM(G0N+G1N+G1NS1+G2N+G2NS1)*100*
	**LC_IGP118**	IgG2 BG1S1/(BG1+BG1S1)	*The percentage of monosialylation of afucosylated monogalactosylated (with bisecting GlcNAc) structures in total IgG2 glycans*	*G1NS1/SUM(G1N+G1NS1)*100*
	**LC_IGP119**	IgG2 BG2S1/(BG2+BG2S1)	*The percentage of monosialylation of afucosylated digalactosylated (with bisecting GlcNAc) structures in total IgG2 glycans*	*G2NS1/SUM(G2N+G2NS1)*100*
	**LC_IGP120**	IgG2 FGS1/(FG+FGS1)	*The percentage of monosialylation of fucosylated galactosylated structures without bisecting GlcNAc in total IgG2 glycans*	*SUM(G1FS1+G2FS1)/SUM(G1F+G1FS1+G2F+G2FS1)*100*
	**LC_IGP121**	IgG2 FGS1/(F+FG+FGS1)	*The percentage of monosialylation of all fucosylated structures without bisecting GlcNAc in total IgG2 glycans*	*SUM(G1FS1+G2FS1)/SUM(G0F+G1F+G1FS1+G2F+G2FS1)*100*
	**LC_IGP122**	IgG2 FG1S1/(FG1+FG1S1)	*The percentage of monosialylation of fucosylated monogalactosylated (without bisecting GlcNAc) structures in total IgG2 glycans*	*G1FS1/SUM(G1F+G1FS1)*100*
	**LC_IGP123**	IgG2 FG2S1/(FG2+FG2S1)	*The percentage of monosialylation of fucosylated digalactosylated (without bisecting GlcNAc) structures in total IgG2 glycans*	*G2FS1/SUM(G2F+G2FS1)*100*
	**LC_IGP124**	IgG2 FBGS1/(FBG+FBGS1)	*The percentage of monosialylation of fucosylated galactosylated structures with bisecting GlcNAc in total IgG2 glycans*	*SUM(G1FNS1+G2FNS1)/SUM(G1FN+G1FNS1+G2FN+G2FNS1)*100*
	**LC_IGP125**	IgG2 FBGS1/(FB+FBG+FBGS1)	*The percentage of monosialylation of all fucosylated structures with bisecting GlcNAc in total IgG2 glycans*	*SUM(G1FNS1+G2FNS1)/SUM(G0FN+G1FN+G1FNS1+G2FN+G2FNS1)*100*
	**LC_IGP126**	IgG2 FBG1S1/(FBG1+FBG1S1)	*The percentage of monosialylation of fucosylated monogalactosylated (with bisecting GlcNAc) structures in total IgG2 glycans*	*G1FNS1/SUM(G1FN+G1FNS1)*100*
	**LC_IGP127**	IgG2 FBG2S1/(FBG2+FBG2S1)	*The percentage of monosialylation of fucosylated digalactosylated (with bisecting GlcNAc) structures in total IgG2 glycans*	*G2FNS1/SUM(G2FN+G2FNS1)*100*
	**LC_IGP128**	IgG2 BS1/S1	*Ratio of afucosylated monosialylated structures with and without bisecting GlcNAc in total IgG2 glycans*	*SUM(G1NS1+G2NS1)/SUM(G1S1+G2S1)*
	**LC_IGP129**	IgG2 FBS1/FS1	*Ratio of fucosylated monosialylated structures with and without bisecting GlcNAc in total IgG2 glycans*	*SUM(G1FNS1+G2FNS1)/SUM(G1FS1+G2FS1)*
	**LC_IGP130**	IgG2 BS1/(S1+BS1)	*The incidence of bisecting GlcNAc in all afucosylated monosialylated structures in total IgG2 glycans*	*SUM(G1NS1+G2NS1)/SUM(G1S1+G1NS1+G2S1+G2NS1)*
	**LC_IGP131**	IgG2 FBS1/(FS1+FBS1)	*The incidence of bisecting GlcNAc in all fucosylated monosialylated structures in total IgG2 glycans*	*SUM(G1FNS1+G2FNS1)/SUM(G1FS1+G1FNS1+G2FS1+G2FNS1)*
IgG2 glycans (neutral)	**LC_IGP132**	IgG2_G0Fn	*The percentage of G0F glycan in neutral IgG2 glycans*	
	**LC_IGP133**	IgG2_G1Fn	*The percentage of G1F glycan in neutral IgG2 glycans*	
	**LC_IGP134**	IgG2_G2Fn	*The percentage of G2F glycan in neutral IgG2 glycans*	
	**LC_IGP135**	IgG2_G0FNn	*The percentage of G0FN glycan in neutral IgG2 glycans*	
	**LC_IGP136**	IgG2_G1FNn	*The percentage of G1FN glycan in neutral IgG2 glycans*	
	**LC_IGP137**	IgG2_G2FNn	*The percentage of G2FN glycan in neutral IgG2 glycans*	
	**LC_IGP138**	IgG2_G0n	*The percentage of G0 glycan in neutral IgG2 glycans*	
	**LC_IGP139**	IgG2_G1n	*The percentage of G1 glycan in neutral IgG2 glycans*	
	**LC_IGP140**	IgG2_G2n	*The percentage of G2 glycan in neutral IgG2 glycans*	
	**LC_IGP141**	IgG2_G0Nn	*The percentage of G0N glycan in neutral IgG2 glycans*	
	**LC_IGP142**	IgG2_G1Nn	*The percentage of G1N glycan in neutral IgG2 glycans*	
	**LC_IGP143**	IgG2_G2Nn	*The percentage of G2N glycan in neutral IgG2 glycans*	
Summarizing IgG2 glycans (neutral)	**LC_IGP144**	IgG2 G0n	*The percentage of agalactosylated structures in neutral IgG2 glycan fraction*	*SUM(G0n+G0Fn+G0FNn+G0Nn)*
	**LC_IGP145**	IgG2 G1n	*The percentage of monogalactosylated structures in neutral IgG2 glycan fraction*	*SUM(G1n+G1Fn+G1FNn+G1Nn)*
	**LC_IGP146**	IgG2 G2n	*The percentage of digalactosylated structures in neutral IgG2 glycan fraction*	*SUM(G2n+G2Fn+G2FNn+G2Nn)*
	**LC_IGP147**	IgG2 Fn total	*The percentage of all fucosylated (+/- bisecting GlcNAc) structures in neutral IgG2 glycan fraction*	*SUM(G0Fn+G0FNn+G1Fn+G1FNn+G2Fn+G2FNn)*
	**LC_IGP148**	IgG2 FG0n total/G0n	*The percentage of fucosylation of agalactosylated structures in neutral IgG2 glycan fraction*	*SUM(G0Fn+G0FNn)/G0n*100*
	**LC_IGP149**	IgG2 FG1n total/G1n	*The percentage of fucosylation of monogalactosylated structures in neutral IgG2 glycan fraction*	*SUM(G1Fn+G1FNn)/G1n*100*
	**LC_IGP150**	IgG2 FG2n total/G2n	*The percentage of fucosylation of digalactosylated structures in neutral IgG2 glycan fraction*	*SUM(G2Fn+G2FNn)/G2n*100*
	**LC_IGP151**	IgG2 Fn	*The percentage of fucosylated (without bisecting GlcNAc) structures in neutral IgG2 glycan fraction*	*SUM(G0Fn+G1Fn+G2Fn)*
	**LC_IGP152**	IgG2 FG0n/G0n	*The percentage of fucosylation (without bisecting GlcNAc) of agalactosylated structures in neutral IgG2 glycan fraction*	*G0Fn/G0n*100*
	**LC_IGP153**	IgG2 FG1n/G1n	*The percentage of fucosylation (without bisecting GlcNAc) of monogalactosylated structures in neutral IgG2 glycan fraction*	*G1Fn/G1n*100*
	**LC_IGP154**	IgG2 FG2n/G2n	*The percentage of fucosylation (without bisecting GlcNAc) of digalactosylated structures in neutral IgG2 glycan fraction*	*G2Fn/G2n*100*
	**LC_IGP155**	IgG2 FBn	*The percentage of fucosylated (with bisecting GlcNAc) structures in neutral IgG2 glycan fraction*	*SUM(G0FNn+G1FNn+G2FNn)*
	**LC_IGP156**	IgG2 FBG0n/G0n	*The percentage of fucosylation (with bisecting GlcNAc) of agalactosylated structures in neutral IgG2 glycan fraction*	*G0FNn/G0n*100*
	**LC_IGP157**	IgG2 FBG1n/G1n	*The percentage of fucosylation (with bisecting GlcNAc) of monogalactosylated structures in neutral IgG2 glycan fraction*	*G1FNn/G1n*100*
	**LC_IGP158**	IgG2 FBG2n/G2n	*The percentage of fucosylation (with bisecting GlcNAc) of digalactosylated structures in neutral IgG2 glycan fraction*	*G2FNn/G2n*100*
	**LC_IGP159**	IgG2 Bn total	*The incidence of bisecting GlcNAc (+/- core Fuc) in neutral IgG2 glycan fraction*	*SUM(G0Nn+G1Nn+G2Nn+G0FNn+G1FNn+G2FNn)*
	**LC_IGP160**	IgG2 BG0n total/G0n	*The incidence of bisecting GlcNAc (+/- core Fuc) in agalactosylated structures in neutral IgG2 glycan fraction*	*SUM(G0Nn+G0FNn)/G0n*100*
	**LC_IGP161**	IgG2 BG1n total/G1n	*The incidence of bisecting GlcNAc (+/- core Fuc) in monogalactosylated structures in neutral IgG2 glycan fraction*	*SUM(G1Nn+G1FNn)/G1n*100*
	**LC_IGP162**	IgG2 BG2n total/G2n	*The incidence of bisecting GlcNAc (+/- core Fuc) in digalactosylated structures in neutral IgG2 glycan fraction*	*SUM(G2Nn+G2FNn)/G2n*100*
	**LC_IGP163**	IgG2 Bn	*The incidence of bisecting GlcNAc (without core Fuc) in neutral IgG2 glycan fraction*	*SUM(G0Nn+G1Nn+G2Nn)*
	**LC_IGP164**	IgG2 BG0n/G0n	*The incidence of bisecting GlcNAc (without core Fuc) in agalactosylated structures in neutral IgG2 glycan fraction*	*G0Nn/G0n*100*
	**LC_IGP165**	IgG2 BG1n/G1n	*The incidence of bisecting GlcNAc (without core Fuc) in monogalactosylated structures in neutral IgG2 glycan fraction*	*G1Nn/G1n*100*
	**LC_IGP166**	IgG2 BG2n/G2n	*The incidence of bisecting GlcNAc (without core Fuc) in digalactosylated structures in neutral IgG2 glycan fraction*	*G2Nn/G2n*100*
	**LC_IGP167**	IgG2 Fn/Bn	*Ratio of fucosylated structures without bisecting GlcNAc and afucosylated structures with bisecting GlcNAc in neutral IgG2 glycan fraction*	*Fn/Bn*
	**LC_IGP168**	IgG2 FBn/Fn	*Ratio of fucosylated structures with and without bisecting GlcNAc in neutral IgG2 glycan fraction*	*FBn/Fn*
	**LC_IGP169**	IgG2 FBn/Fn total	*The incidence of bisecting GlcNAc in all fucosylated structures in neutral IgG2 glycan fraction*	*FBn/Fn total*100*
	**LC_IGP170**	IgG2 FBn/Bn total	*The percentage of fucosylation in all structures with bisecting GlcNAc in neutral IgG2 glycan fraction*	*FBn/Bn total*100*

	**LC_IGP171**	IgG2 Fn/Bn total	*Ratio of fucosylated non-bisecting GlcNAc structures and all structures with bisecting GlcNAc in neutral IgG2 glycans fraction*	*Fn/Bn total*
	**LC_IGP172**	IgG2 Bn/Fn total ‰	*Ratio of structures with bisecting GlcNAc and all fucosylated structures (+/- bisecting GlcNAc) in neutral IgG2 glycan fraction*	*Bn/Fn total*1000*
**Initial IgG4 Traits**				
Initial IgG4 glycans (total)	**LC_IGP173**	IgG4_G0F	*The percentage of G0F glycan in total IgG4 glycans*	
	**LC_IGP174**	IgG4_G1F	*The percentage of G1F glycan in total IgG4 glycans*	
	**LC_IGP175**	IgG4_G2F	*The percentage of G2F glycan in total IgG4 glycans*	
	**LC_IGP176**	IgG4_G0FN	*The percentage of G0FN glycan in total IgG4 glycans*	
	**LC_IGP177**	IgG4_G1FN	*The percentage of G1FN glycan in total IgG4 glycans*	
	**LC_IGP178**	IgG4_G2FN	*The percentage of G2FN glycan in total IgG4 glycans*	
	**LC_IGP179**	IgG4_G1FS1	*The percentage of G1FS1 glycan in total IgG4 glycans*	
	**LC_IGP180**	IgG4_G2FS1	*The percentage of G2FS1 glycan in total IgG4 glycans*	
	**LC_IGP181**	IgG4_G1FNS1	*The percentage of G1FNS1 glycan in total IgG4 glycans*	
	**LC_IGP182**	IgG4_G2FNS1	*The percentage of G2FNS1 glycan in total IgG4 glycans*	
**Summarizing IgG4 Traits**				
Summarizing IgG4 glycans (total)	**LC_IGP183**	IgG4 Bisecting GlcNAc	*The incidence of bisecting GlcNAc of IgG4*	*SUM(G0FN+G1FN+G2FN)*
	**LC_IGP184**	IgG4 Galactosylation	*The percentage of IgG4 galactosylation*	*SUM(G1F+G1FN+G1FS1+G1FNS1)*0.5+SUM(G2F+G2FN+G2FS1+G2FNS1)*
	**LC_IGP185**	IgG4 Sialylation	*The percentage of IgG4 sialylation*	*SUM(G1FS1+G2FS1+G1FNS1+G2FNS1)*
	**LC_IGP186**	IgG4 SA per Gal	*The number of sialic acid moieties on galactose moieties in total IgG4 glycans*	*IgG4 Sialylation/IgG4 Galactosylation*
	**LC_IGP187**	IgG4 FGS1/(FG+FGS1)	*The percentage of monosialylation of fucosylated galactosylated structures without bisecting GlcNAc in total IgG4 glycans*	*SUM(G1FS1+G2FS1)/SUM(G1F+G1FS1+G2F+G2FS1)*100*
	**LC_IGP188**	IgG4 FGS1/(F+FG+FGS1)	*The percentage of monosialylation of all fucosylated structures without bisecting GlcNAc in total IgG4 glycans*	*SUM(G1FS1+G2FS1)/SUM(G0F+G1F+G1FS1+G2F+G2FS1)*100*
	**LC_IGP189**	IgG4 FG1S1/(FG1+FG1S1)	*The percentage of monosialylation of fucosylated monogalactosylated (without bisecting GlcNAc) structures in total IgG4 glycans*	*G1FS1/SUM(G1F+G1FS1)*100*
	**LC_IGP190**	IgG4 FG2S1/(FG2+FG2S1)	*The percentage of monosialylation of fucosylated digalactosylated (without bisecting GlcNAc) structures in total IgG4 glycans*	*G2FS1/SUM(G2F+G2FS1)*100*
	**LC_IGP191**	IgG4 FBGS1/(FBG+FBGS1)	*The percentage of monosialylation of fucosylated galactosylated structures with bisecting GlcNAc in total IgG4 glycans*	*SUM(G1FNS1+G2FNS1)/SUM(G1FN+G1FNS1+G2FN+G2FNS1)*100*
	**LC_IGP192**	IgG4 FBGS1/(FB+FBG+FBGS1)	*The percentage of monosialylation of all fucosylated structures with bisecting GlcNAc in total IgG4 glycans*	*SUM(G1FNS1+G2FNS1)/SUM(G0FN+G1FN+G1FNS1+G2FN+G2FNS1)*100*
	**LC_IGP193**	IgG4 FBG1S1/(FBG1+FBG1S1)	*The percentage of monosialylation of fucosylated monogalactosylated (with bisecting GlcNAc) structures in total IgG4 glycans*	*G1FNS1/SUM(G1FN+G1FNS1)*100*
	**LC_IGP194**	IgG4 FBG2S1/(FBG2+FBG2S1)	*The percentage of monosialylation of fucosylated digalactosylated (with bisecting GlcNAc) structures in total IgG4 glycans*	*G2FNS1/SUM(G2FN+G2FNS1)*100*
	**LC_IGP195**	IgG4 FBS1/FS1	*Ratio of fucosylated monosialylated structures with and without bisecting GlcNAc in total IgG4 glycans*	*SUM(G1FNS1+G2FNS1)/SUM(G1FS1+G2FS1)*
	**LC_IGP196**	IgG4 FBS1/(FS1+FBS1)	*The incidence of bisecting GlcNAc in all fucosylated monosialylated structures in total IgG4 glycans*	*SUM(G1FNS1+G2FNS1)/SUM(G1FS1+G1FNS1+G2FS1+G2FNS1)*
IgG4 glycans (neutral)	**LC_IGP197**	IgG4_G0Fn	*The percentage of G0F glycan in neutral IgG4 glycans*	
	**LC_IGP198**	IgG4_G1Fn	*The percentage of G1F glycan in neutral IgG4 glycans*	
	**LC_IGP199**	IgG4_G2Fn	*The percentage of G2F glycan in neutral IgG4 glycans*	
	**LC_IGP200**	IgG4_G0FNn	*The percentage of G0FN glycan in neutral IgG4 glycans*	
	**LC_IGP201**	IgG4_G1FNn	*The percentage of G1FN glycan in neutral IgG4 glycans*	
	**LC_IGP202**	IgG4_G2FNn	*The percentage of G2FN glycan in neutral IgG4 glycans*	
Summarizing IgG4 glycans (neutral)	**LC_IGP203**	IgG4 G0n	*The percentage of agalactosylated structures in neutral IgG4 glycan fraction*	*SUM(G0F+G0FN)*
	**LC_IGP204**	IgG4 G1n	*The percentage of monogalactosylated structures in neutral IgG4 glycan fraction*	*SUM(G1F+G1FN)*
	**LC_IGP205**	IgG4 G2n	*The percentage of digalactosylated structures in neutral IgG4 glycan fraction*	*SUM(G2F+G2FN)*

**Within Subclass Ratios**				
Pathway ratios for IgG1	**LC_IGP_R1**	IgG1_G0F/IgG1_G0	*The relation of G0F to G0 in IgG1 describing the pathway step from G0 to G0F in IgG1*	*IgG1_G0F/IgG1_G0*
	**LC_IGP_R2**	IgG1_G1F/IgG1_G1	*The relation of G1F to G1 in IgG1 describing the pathway step from G1 to G1F in IgG1*	*IgG1_G1F/IgG1_G1*
	**LC_IGP_R3**	IgG1_G2F/IgG1_G2	*The relation of G2F to G2 in IgG1 describing the pathway step from G2 to G2F in IgG1*	*IgG1_G2F/IgG1_G2*
	**LC_IGP_R4**	IgG1_G0FN/IgG1_G0N	*The relation of G0FN to G0N in IgG1 describing the pathway step from G0N to G0FN in IgG1*	*IgG1_G0FN/IgG1_G0N*
	**LC_IGP_R5**	IgG1_G1FN/IgG1_G1N	*The relation of G1FN to G1N in IgG1 describing the pathway step from G1N to G1FN in IgG1*	*IgG1_G1FN/IgG1_G1N*
	**LC_IGP_R6**	IgG1_G2FN/IgG1_G2N	*The relation of G2FN to G2N in IgG1 describing the pathway step from G2N to G2FN in IgG1*	*IgG1_G2FN/IgG1_G2N*
	**LC_IGP_R7**	IgG1_G1FS/IgG1_G1S	*The relation of G1FS to G1S in IgG1 describing the pathway step from G1S to G1FS in IgG1.*	*IgG1_G1FS/IgG1_G1S*
	**LC_IGP_R8**	IgG1_G2FS/IgG1_G2S	*The relation of G2FS to G2S in IgG1 describing the pathway step from G2S to G2FS in IgG1.*	*IgG1_G2FS/IgG1_G2S*
	**LC_IGP_R9**	IgG1_G1FNS/IgG1_G1NS	*The relation of G1FNS to G1NS in IgG1 describing the pathway step from G1NS to G1FNS in IgG1.*	*IgG1_G1FNS/IgG1_G1NS*
	**LC_IGP_R10**	IgG1_G2FNS/IgG1_G2NS	*The relation of G2FNS to G2NS in IgG1 describing the pathway step from G2NS to G2FNS in IgG1.*	*IgG1_G2FNS/IgG1_G2NS*
	**LC_IGP_R11**	IgG1_G0FN/IgG1_G0F	*The relation of G0FN to G0F in IgG1 describing the pathway step from G0F to G0FN in IgG1.*	*IgG1_G0FN/IgG1_G0F*
	**LC_IGP_R12**	IgG1_G1FN/IgG1_G1F	*The relation of G1FN to G1F in IgG1 describing the pathway step from G1F to G1FN in IgG1.*	*IgG1_G1FN/IgG1_G1F*
	**LC_IGP_R13**	IgG1_G2FN/IgG1_G2F	*The relation of G2FN to G2F in IgG1 describing the pathway step from G2F to G2FN in IgG1.*	*IgG1_G2FN/IgG1_G2F*
	**LC_IGP_R14**	IgG1_G1FNS/IgG1_G1FS	*The relation of G1FNS to G1FS in IgG1 describing the pathway step from G1FS to G1FNS in IgG1.*	*IgG1_G1FNS/IgG1_G1FS*
	**LC_IGP_R15**	IgG1_G2FNS/IgG1_G2FS	*The relation of G2FNS to G2FS in IgG1 describing the pathway step from G2FS to G2FNS in IgG1.*	*IgG1_G2FNS/IgG1_G2FS*
	**LC_IGP_R16**	IgG1_G1N/IgG1_G1	*The relation of G1N to G1 in IgG1 describing the pathway step from G1 to G1N in IgG1.*	*IgG1_G1N/IgG1_G1*
	**LC_IGP_R17**	IgG1_G2N/IgG1_G2	*The relation of G2N to G2 in IgG1 describing the pathway step from G2 to G2N in IgG1.*	*IgG1_G2N/IgG1_G2*
	**LC_IGP_R18**	IgG1_G1NS/IgG1_G1S	*The relation of G1NS to G1S in IgG1 describing the pathway step from G1S to G1NS in IgG1.*	*IgG1_G1NS/IgG1_G1S*
	**LC_IGP_R19**	IgG1_G2NS/IgG1_G2S	*The relation of G2NS to G2S in IgG1 describing the pathway step from G2S to G2NS in IgG1.*	*IgG1_G2NS/IgG1_G2S*
	**LC_IGP_R20**	IgG1_G1/IgG1_G0	*The relation of G1 to G0 in IgG1 describing the pathway step from G0 to G1 in IgG1.*	*IgG1_G1/IgG1_G0*
	**LC_IGP_R21**	IgG1_G1N/IgG1_G0N	*The relation of G1N to G0N in IgG1 describing the pathway step from G0N to G1N in IgG1.*	*IgG1_G1N/IgG1_G0N*
	**LC_IGP_R22**	IgG1_G1F/IgG1_G0F	*The relation of G1F to G0F in IgG1 describing the pathway step from G0F to G1F in IgG1.*	*IgG1_G1F/IgG1_G0F*
	**LC_IGP_R23**	IgG1_G1FN/IgG1_G0FN	*The relation of G1FN to G0FN in IgG1 describing the pathway step from G0FN to G1FN in IgG1.*	*IgG1_G1FN/IgG1_G0FN*
	**LC_IGP_R24**	IgG1_G2/IgG1_G1	*The relation of G2 to G1 in IgG1 describing the pathway step from G1 to G2 in IgG1.*	*IgG1_G2/IgG1_G1*
	**LC_IGP_R25**	IgG1_G2S/IgG1_G1S	*The relation of G2S to G1S in IgG1 describing the pathway step from G1S to G2S in IgG1.*	*IgG1_G2S/IgG1_G1S*
	**LC_IGP_R26**	IgG1_G2N/IgG1_G1N	*The relation of G2N to G1N in IgG1 describing the pathway step from G1N to G2N in IgG1.*	*IgG1_G2N/IgG1_G1N*
	**LC_IGP_R27**	IgG1_G2NS/IgG1_G1NS	*The relation of G2NS to G1NS in IgG1 describing the pathway step from G1NS to G2NS in IgG1.*	*IgG1_G2NS/IgG1_G1NS*
	**LC_IGP_R28**	IgG1_G2F/IgG1_G1F	*The relation of G2F to G1F in IgG1 describing the pathway step from G1F to G2F in IgG1.*	*IgG1_G2F/IgG1_G1F*
	**LC_IGP_R29**	IgG1_G2FS/IgG1_G1FS	*The relation of G2FS to G1FS in IgG1 describing the pathway step from G1FS to G2FS in IgG1.*	*IgG1_G2FS/IgG1_G1FS*
	**LC_IGP_R30**	IgG1_G2FNS/IgG1_G1FNS	*The relation of G2FNS to G1FNS in IgG1 describing the pathway step from G1FNS to G2FNS in IgG1.*	*IgG1_G2FNS/IgG1_G1FNS*
	**LC_IGP_R31**	IgG1_G2FN/IgG1_G1FN	*The relation of G2FN to G1FN in IgG1 describing the pathway step from G1FN to G2FN in IgG1.*	*IgG1_G2FN/IgG1_G1FN*
	**LC_IGP_R32**	IgG1_G1S/IgG1_G1	*The relation of G1S to G1 in IgG1 describing the pathway step from G1 to G1S in IgG1.*	*IgG1_G1S/IgG1_G1*
	**LC_IGP_R33**	IgG1_G2S/IgG1_G2	*The relation of G2S to G2 in IgG1 describing the pathway step from G2 to G2S in IgG1.*	*IgG1_G2S/IgG1_G2*
	**LC_IGP_R34**	IgG1_G1FS/IgG1_G1F	*The relation of G1FS to G1F in IgG1 describing the pathway step from G1F to G1FS in IgG1.*	*IgG1_G1FS/IgG1_G1F*
	**LC_IGP_R35**	IgG1_G2FS/IgG1_G2F	*The relation of G2FS to G2F in IgG1 describing the pathway step from G2F to G2FS in IgG1.*	*IgG1_G2FS/IgG1_G2F*
	**LC_IGP_R36**	IgG1_G1NS/IgG1_G1N	*The relation of G1NS to G1N in IgG1 describing the pathway step from G1N to G1NS in IgG1.*	*IgG1_G1NS/IgG1_G1N*
	**LC_IGP_R37**	IgG1_G2NS/IgG1_G2N	*The relation of G2NS to G2N in IgG1 describing the pathway step from G2N to G2NS in IgG1.*	*IgG1_G2NS/IgG1_G2N*
	**LC_IGP_R38**	IgG1_G1FNS/IgG1_G1FN	*The relation of G1FNS to G1FN in IgG1 describing the pathway step from G1FN to G1FNS in IgG1.*	*IgG1_G1FNS/IgG1_G1FN*
	**LC_IGP_R39**	IgG1_G0N/IgG1_G0	*The relation of G0N to G0 in IgG1 describing the pathway step from G0 to G0N in IgG1.*	*IgG1_G0N/IgG1_G0*
	**LC_IGP_R40**	IgG1_G2FNS/IgG1_G2FN	*The relation of G2FNS to G2FN in IgG1 describing the pathway step from G2FN to G2FNS in IgG1.*	*IgG1_G2FNS/IgG1_G2FN*
	**LC_IGP_R41**	IgG2_G0F/IgG2_G0	*The relation of G0F to G0 in IgG2 describing the pathway step from G0 to G0F in IgG2*	*IgG2_G0F/IgG2_G0*
	**LC_IGP_R42**	IgG2_G1F/IgG2_G1	*The relation of G1F to G1 in IgG2 describing the pathway step from G1 to G1F in IgG2*	*IgG2_G1F/IgG2_G1*
	**LC_IGP_R43**	IgG2_G2F/IgG2_G2	*The relation of G2F to G2 in IgG2 describing the pathway step from G2 to G2F in IgG2*	*IgG2_G2F/IgG2_G2*

	LC_IGP_R44	IgG2_G0FN/IgG2_G0N	*The relation of G0FN to G0N in IgG2 describing the pathway step from G0N to G0FN in IgG2*	*IgG2_G0FN/IgG2_G0N*
	LC_IGP_R45	IgG2_G1FN/IgG2_G1N	*The relation of G1FN to G1N in IgG2 describing the pathway step from G1N to G1FN in IgG2*	*IgG2_G1FN/IgG2_G1N*
	LC_IGP_R46	IgG2_G2FN/IgG2_G2N	*The relation of G2FN to G2N in IgG2 describing the pathway step from G2N to G2FN in IgG2*	*IgG2_G2FN/IgG2_G2N*
	LC_IGP_R47	IgG2_G1FS/IgG2_G1S	*The relation of G1FS to G1S in IgG2 describing the pathway step from G1S to G1FS in IgG2*	*IgG2_G1FS/IgG2_G1S*
	LC_IGP_R48	IgG2_G2FS/IgG2_G2S	*The relation of G2FS to G2S in IgG2 describing the pathway step from G2S to G2FS in IgG2*	*IgG2_G2FS/IgG2_G2S*
	LC_IGP_R49	IgG2_G1FNS/IgG2_G1NS	*The relation of G1FNS to G1NS in IgG2 describing the pathway step from G1NS to G1FNS in IgG2*	*IgG2_G1FNS/IgG2_G1NS*
	LC_IGP_R50	IgG2_G2FNS/IgG2_G2NS	*The relation of G2FNS to G2NS in IgG2 describing the pathway step from G2NS to G2FNS in IgG2*	*IgG2_G2FNS/IgG2_G2NS*
	LC_IGP_R51	IgG2_G0FN/IgG2_G0F	*The relation of G0FN to G0F in IgG2 describing the pathway step from G0F to G0FN in IgG2*	*IgG2_G0FN/IgG2_G0F*
	LC_IGP_R52	IgG2_G1FN/IgG2_G0F	*The relation of G1FN to G1F in IgG2 describing the pathway step from G1F to G1FN in IgG2*	*IgG2_G1FN/IgG2_G0F*
	LC_IGP_R53	IgG2_G2FN/IgG2_G2F	*The relation of G2FN to G2F in IgG2 describing the pathway step from G2F to G2FN in IgG2*	*IgG2_G2FN/IgG2_G2F*
	LC_IGP_R54	IgG2_G1FNS/IgG2_G1FS	*The relation of G1FNS to G1FS in IgG2 describing the pathway step from G1FS to G1FNS in IgG2*	*IgG2_G1FNS/IgG2_G1FS*
	LC_IGP_R55	IgG2_G2FNS/IgG2_G2FS	*The relation of G2FNS to G2FS in IgG2 describing the pathway step from G2FS to G2FNS in IgG2*	*IgG2_G2FNS/IgG2_G2FS*
	LC_IGP_R56	IgG2_G1N/IgG2_G1	*The relation of G1N to G1 in IgG2 describing the pathway step from G1 to G1N in IgG2*	*IgG2_G1N/IgG2_G1*
	LC_IGP_R57	IgG2_G2N/IgG2_G2	*The relation of G2N to G2 in IgG2 describing the pathway step from G2 to G2N in IgG2*	*IgG2_G2N/IgG2_G2*
	LC_IGP_R58	IgG2_G1NS/IgG2_G1S	*The relation of G1NS to G1S in IgG2 describing the pathway step from G1S to G1NS in IgG2*	*IgG2_G1NS/IgG2_G1S*
	LC_IGP_R59	IgG2_G2NS/IgG2_G2S	*The relation of G2NS to G2S in IgG2 describing the pathway step from G2S to G2NS in IgG2*	*IgG2_G2NS/IgG2_G2S*
Pathway Ratios for IgG2	LC_IGP_R60	IgG2_G1/IgG2_G0	*The relation of G1 to G0 in IgG2 describing the pathway step from G0 to G1 in IgG2*	*IgG2_G1/IgG2_G0*
	LC_IGP_R61	IgG2_G1N/IgG2_G0N	*The relation of G1N to G0N in IgG2 describing the pathway step from G0N to G1N in IgG2*	*IgG2_G1N/IgG2_G0N*
	LC_IGP_R62	IgG2_G1F/IgG2_G0F	*The relation of G1F to G0F in IgG2 describing the pathway step from G0F to G1F in IgG2*	*IgG2_G1F/IgG2_G0F*
	LC_IGP_R63	IgG2_G1FN/IgG2_G0FN	*The relation of G1FN to G0FN in IgG2 describing the pathway step from G0FN to G1FN in IgG2*	*IgG2_G1FN/IgG2_G0FN*
	LC_IGP_R64	IgG2_G2/IgG2_G1	*The relation of G2 to G1 in IgG2 describing the pathway step from G1 to G2 in IgG2*	*IgG2_G2/IgG2_G1*
	LC_IGP_R65	IgG2_G2S/IgG2_G1S	*The relation of G2S to G1S in IgG2 describing the pathway step from G1S to G2S in IgG2*	*IgG2_G2S/IgG2_G1S*
	LC_IGP_R66	IgG2_G2N/IgG2_G1N	*The relation of G2N to G1N in IgG2 describing the pathway step from G1N to G2N in IgG2*	*IgG2_G2N/IgG2_G1N*
	LC_IGP_R67	IgG2_G2NS/IgG2_G1NS	*The relation of G2NS to G1NS in IgG2 describing the pathway step from G1NS to G2NS in IgG2*	*IgG2_G2NS/IgG2_G1NS*
	LC_IGP_R68	IgG2_G2F/IgG2_G1F	*The relation of G2F to G1F in IgG2 describing the pathway step from G1F to G2F in IgG2*	*IgG2_G2F/IgG2_G1F*
	LC_IGP_R69	IgG2_G2FS/IgG2_G1FS	*The relation of G2FS to G1FS in IgG2 describing the pathway step from G1FS to G2FS in IgG2*	*IgG2_G2FS/IgG2_G1FS*
	LC_IGP_R70	IgG2_G2FNS/IgG2_G1FNS	*The relation of G2FNS to G1FNS in IgG2 describing the pathway step from G1FNS to G2FNS in IgG2*	*IgG2_G2FNS/IgG2_G1FNS*
	LC_IGP_R71	IgG2_G2FN/IgG2_G1FN	*The relation of G2FN to G1FN in IgG2 describing the pathway step from G1FN to G2FN in IgG2*	*IgG2_G2FN/IgG2_G1FN*
	LC_IGP_R72	IgG2_G1S/IgG2_G1	*The relation of G1S to G1 in IgG2 describing the pathway step from G1 to G1S in IgG2*	*IgG2_G1S/IgG2_G1*
	LC_IGP_R73	IgG2_G2S/IgG2_G2	*The relation of G2S to G2 in IgG2 describing the pathway step from G2 to G2S in IgG2*	*IgG2_G2S/IgG2_G2*
	LC_IGP_R74	IgG2_G1FS/IgG2_G1F	*The relation of G1FS to G1F in IgG2 describing the pathway step from G1F to G1FS in IgG2*	*IgG2_G1FS/IgG2_G1F*
	LC_IGP_R75	IgG2_G2FS/IgG2_G2F	*The relation of G2FS to G2F in IgG2 describing the pathway step from G2F to G2FS in IgG2*	*IgG2_G2FS/IgG2_G2F*
	LC_IGP_R76	IgG2_G1NS/IgG2_G1N	*The relation of G1NS to G1N in IgG2 describing the pathway step from G1N to G1NS in IgG2*	*IgG2_G1NS/IgG2_G1N*
	LC_IGP_R77	IgG2_G2NS/IgG2_G2N	*The relation of G2NS to G2N in IgG2 describing the pathway step from G2N to G2NS in IgG2*	*IgG2_G2NS/IgG2_G2N*
	LC_IGP_R78	IgG2_G1FNS/IgG2_G1FN	*The relation of G1FNS to G1FN in IgG2 describing the pathway step from G1FN to G1FNS in IgG2*	*IgG2_G1FNS/IgG2_G1FN*
	LC_IGP_R79	IgG2_G0N/IgG2_G0	*The relation of G0N to G0 in IgG2 describing the pathway step from G0 to G0N in IgG2*	*IgG2_G0N/IgG2_G0*
	LC_IGP_R80	IgG2_G2FNS/IgG2_G2FN	*The relation of G2FNS to G2FN in IgG2 describing the pathway step from G2FN to G2FNS in IgG2*	*IgG2_G2FNS/IgG2_G2FN*
	LC_IGP_R81	IgG4_G0FN/IgG4_G0F	*The relation of G0FN to G0F in IgG4 describing the pathway step from G0F to G0FN in IgG4*	*IgG4_G0FN/IgG4_G0F*
	LC_IGP_R82	IgG4_G1FN/IgG4_G0F	*The relation of G1FN to G1F in IgG4 describing the pathway step from G1F to G1FN in IgG4*	*IgG4_G1FN/IgG4_G0F*
	LC_IGP_R83	IgG4_G2FN/IgG4_G2F	*The relation of G2FN to G2F in IgG4 describing the pathway step from G2F to G2FN in IgG4*	*IgG4_G2FN/IgG4_G2F*
	LC_IGP_R84	IgG4_G1FNS/IgG4_G1FS	*The relation of G1FNS to G1FS in IgG4 describing the pathway step from G1FS to G1FNS in IgG4*	*IgG4_G1FNS/IgG4_G1FS*
	LC_IGP_R85	IgG4_G2FNS/IgG4_G2FS	*The relation of G2FNS to G2FS in IgG4 describing the pathway step from G2FS to G2FNS in IgG4*	*IgG4_G2FNS/IgG4_G2FS*
	LC_IGP_R86	IgG4_G1F/IgG4_G0F	*The relation of G1F to G0F in IgG4 describing the pathway step from G0F to G1F in IgG4*	*IgG4_G1F/IgG4_G0F*
	LC_IGP_R87	IgG4_G1FN/IgG4_G0FN	*The relation of G1FN to G0FN in IgG4 describing the pathway step from G0FN to G1FN in IgG4*	*IgG4_G1FN/IgG4_G0FN*
Pathway Ratios for IgG4	LC_IGP_R88	IgG4_G2F/IgG4_G1F	*The relation of G2F to G1F in IgG4 describing the pathway step from G1F to G2F in IgG4*	*IgG4_G2F/IgG4_G1F*
	LC_IGP_R89	IgG4_G2FS/IgG4_G1FS	*The relation of G2FS to G1FS in IgG4 describing the pathway step from G1FS to G2FS in IgG4*	*IgG4_G2FS/IgG4_G1FS*
	LC_IGP_R90	IgG4_G2FNS/IgG4_G1FNS	*The relation of G2FNS to G1FNS in IgG4 describing the pathway step from G1FNS to G2FNS in IgG4*	*IgG4_G2FNS/IgG4_G1FNS*
	LC_IGP_R91	IgG4_G2FN/IgG4_G1FN	*The relation of G2FN to G1FN in IgG4 describing the pathway step from G1FN to G2FN in IgG4*	*IgG4_G2FN/IgG4_G1FN*
	LC_IGP_R92	IgG4_G1FS/IgG4_G1F	*The relation of G1FS to G1F in IgG4 describing the pathway step from G1F to G1FS in IgG4*	*IgG4_G1FS/IgG4_G1F*
	LC_IGP_R93	IgG4_G2FS/IgG4_G2F	*The relation of G2FS to G2F in IgG4 describing the pathway step from G2F to G2FS in IgG4*	*IgG4_G2FS/IgG4_G2F*
	LC_IGP_R94	IgG4_G1FNS/IgG4_G1FN	*The relation of G1FNS to G1FN in IgG4 describing the pathway step from G1FN to G1FNS in IgG4*	*IgG4_G1FNS/IgG4_G1FN*
	LC_IGP_R95	IgG4_G2FNS/IgG4_G2FN	*The relation of G2FNS to G2FN in IgG4 describing the pathway step from G2FN to G2FNS in IgG4*	*IgG4_G2FNS/IgG4_G2FN*
**Between Subclass Ratios**				
	LC_IGP_SC1	IgG1_G0F/IgG2_G0F	*The relation of percentage of IgG1_G0F in total IgG1 glycans and percentage of IgG2_G0F in total IgG2 glycans*	*IgG1_G0F/IgG2_G0F*
	LC_IGP_SC2	IgG1_G1F/IgG2_G1F	*The relation of percentage of IgG1_G1F in total IgG1 glycans and percentage of IgG2_G1F in total IgG2 glycans*	*IgG1_G1F/IgG2_G1F*
	LC_IGP_SC3	IgG1_G2F/IgG2_G2F	*The relation of percentage of IgG1_G2F in total IgG1 glycans and percentage of IgG2_G2F in total IgG2 glycans*	*IgG1_G2F/IgG2_G2F*
	LC_IGP_SC4	IgG1_G0FN/IgG2_G0FN	*The relation of percentage of IgG1_G0FN in total IgG1 glycans and percentage of IgG2_G0FN in total IgG2 glycans*	*IgG1_G0FN/IgG2_G0FN*
	LC_IGP_SC5	IgG1_G1FN/IgG2_G1FN	*The relation of percentage of IgG1_G1FN in total IgG1 glycans and percentage of IgG2_G1FN in total IgG2 glycans*	*IgG1_G1FN/IgG2_G1FN*
	LC_IGP_SC6	IgG1_G2FN/IgG2_G2FN	*The relation of percentage of IgG1_G2FN in total IgG1 glycans and percentage of IgG2_G2FN in total IgG2 glycans*	*IgG1_G2FN/IgG2_G2FN*
	LC_IGP_SC7	IgG1_G1FS1/IgG2_G1FS1	*The relation of percentage of IgG1_G1FS1 in total IgG1 glycans and percentage of IgG2_G1FS1 in total IgG2 glycans*	*IgG1_G1FS1/IgG2_G1FS1*
	LC_IGP_SC8	IgG1_G2FS1/IgG2_G2FS1	*The relation of percentage of IgG1_G2FS1 in total IgG1 glycans and percentage of IgG2_G2FS1 in total IgG2 glycans*	*IgG1_G2FS1/IgG2_G2FS1*
	LC_IGP_SC9	IgG1_G1FNS1/IgG2_G1FNS1	*The relation of percentage of IgG1_G1FNS1 in total IgG1 glycans and percentage of IgG2_G1FNS1 in total IgG2 glycans*	*IgG1_G1FNS1/IgG2_G1FNS1*
Subclass Ratios for IgG1 and IgG2	LC_IGP_SC10	IgG1_G2FNS1/IgG2_G2FNS1	*The relation of percentage of IgG1_G2FNS1 in total IgG1 glycans and percentage of IgG2_G2FNS1 in total IgG2 glycans*	*IgG1_G2FNS1/IgG2_G2FNS1*
	LC_IGP_SC11	IgG1_G0/IgG2_G0	*The relation of percentage of IgG1_G0 in total IgG1 glycans and percentage of IgG2_G0 in total IgG2 glycans*	*IgG1_G0/IgG2_G0*
	LC_IGP_SC12	IgG1_G1/IgG2_G1	*The relation of percentage of IgG1_G1 in total IgG1 glycans and percentage of IgG2_G1 in total IgG2 glycans*	*IgG1_G1/IgG2_G1*
	LC_IGP_SC13	IgG1_G2/IgG2_G2	*The relation of percentage of IgG1_G2 in total IgG1 glycans and percentage of IgG2_G2 in total IgG2 glycans*	*IgG1_G2/IgG2_G2*
	LC_IGP_SC14	IgG1_G0N/IgG2_G0N	*The relation of percentage of IgG1_G0N in total IgG1 glycans and percentage of IgG2_G0N in total IgG2 glycans*	*IgG1_G0N/IgG2_G0N*
	LC_IGP_SC15	IgG1_G1N/IgG2_G1N	*The relation of percentage of IgG1_G1N in total IgG1 glycans and percentage of IgG2_G1N in total IgG2 glycans*	*IgG1_G1N/IgG2_G1N*
	LC_IGP_SC16	IgG1_G2N/IgG2_G2N	*The relation of percentage of IgG1_G2N in total IgG1 glycans and percentage of IgG2_G2N in total IgG2 glycans*	*IgG1_G2N/IgG2_G2N*
	LC_IGP_SC17	IgG1_G1S1/IgG2_G1S1	*The relation of percentage of IgG1_G1S1 in total IgG1 glycans and percentage of IgG2_G1S1 in total IgG2 glycans*	*IgG1_G1S1/IgG2_G1S1*
	LC_IGP_SC18	IgG1_G2S1/IgG2_G2S1	*The relation of percentage of IgG1_G2S1 in total IgG1 glycans and percentage of IgG2_G2S1 in total IgG2 glycans*	*IgG1_G2S1/IgG2_G2S1*
	LC_IGP_SC19	IgG1_G1NS1/IgG2_G1NS1	*The relation of percentage of IgG1_G1NS1 in total IgG1 glycans and percentage of IgG2_G1NS1 in total IgG2 glycans*	*IgG1_G1NS1/IgG2_G1NS1*
	LC_IGP_SC20	IgG1_G2NS1/IgG2_G2NS1	*The relation of percentage of IgG1_G2NS1 in total IgG1 glycans and percentage of IgG2_G2NS1 in total IgG2 glycans*	*IgG1_G2NS1/IgG2_G2NS1*
**Total Area Normalization on Fucosylated Glycan Traits Only (Not Used in Analysis)**				
	LC_IGPSC_tmp21	IgG1_G0F(10)	*The percentage of IgG1_G0F in fucosylated IgG1 glycans*	*IgG1_G0F/SUM(G0F+G1F+G2F+G0FN+G1FN+G2FN+G1FS1+G2FS1+G1FNS1+G2FNS1)*
	LC_IGPSC_tmp22	IgG1_G1F(10)	*The percentage of IgG1_G1F in fucosylated IgG1 glycans*	*IgG1_G1F/SUM(G0F+G1F+G2F+G0FN+G1FN+G2FN+G1FS1+G2FS1+G1FNS1+G2FNS1)*
	LC_IGPSC_tmp23	IgG1_G2F(10)	*The percentage of IgG1_G2F in fucosylated IgG1 glycans*	*IgG1_G2F/SUM(G0F+G1F+G2F+G0FN+G1FN+G2FN+G1FS1+G2FS1+G1FNS1+G2FNS1)*
	LC_IGPSC_tmp24	IgG1_G0FN(10)	*The percentage of IgG1_G0FN in fucosylated IgG1 glycans*	*IgG1_G0FN/SUM(G0F+G1F+G2F+G0FN+G1FN+G2FN+G1FS1+G2FS1+G1FNS1+G2FNS1)*
IgG1 normalized on fucosylated IgG1 Glycans	LC_IGPSC_tmp25	IgG1_G1FN(10)	*The percentage of IgG1_G1FN in fucosylated IgG1 glycans*	*IgG1_G1FN/SUM(G0F+G1F+G2F+G0FN+G1FN+G2FN+G1FS1+G2FS1+G1FNS1+G2FNS1)*
	LC_IGPSC_tmp26	IgG1_G2FN(10)	*The percentage of IgG1_G2FN in fucosylated IgG1 glycans*	*IgG1_G2FN/SUM(G0F+G1F+G2F+G0FN+G1FN+G2FN+G1FS1+G2FS1+G1FNS1+G2FNS1)*

	**LC_IGPSC_tmp27**	IgG1_G1FS1(10)	*The percentage of IgG1_G1FS1 in fucosylated IgG1 glycans*	*IgG1_G1FS1/SUM(G0F+G1F+G2F+G0FN+G1FN+G2FN+G1FS1+G2FS1+G1FNS1+G2FNS1)*
	**LC_IGPSC_tmp28**	IgG1_G2FS1(10)	*The percentage of IgG1_G2FS1 in fucosylated IgG1 glycans*	*IgG1_G2FS1/SUM(G0F+G1F+G2F+G0FN+G1FN+G2FN+G1FS1+G2FS1+G1FNS1+G2FNS1)*
	**LC_IGPSC_tmp29**	IgG1_G1FNS1(10)	*The percentage of IgG1_G1FNS1 in fucosylated IgG1 glycans*	*IgG1_G1FNS1/SUM(G0F+G1F+G2F+G0FN+G1FN+G2FN+G1FS1+G2FS1+G1FNS1+G2FNS1)*
	**LC_IGPSC_tmp30**	IgG1_G2FNS1(10)	*The percentage of IgG1_G2FNS1 in fucosylated IgG1 glycans*	*IgG1_G2FNS1/SUM(G0F+G1F+G2F+G0FN+G1FN+G2FN+G1FS1+G2FS1+G1FNS1+G2FNS1)*
IgG2 normalized on fucosylated IgG2 Glycans	**LC_IGPSC_tmp31**	IgG2_G0F(10)	*The percentage of IgG2_G0F in fucosylated IgG2 glycans*	*IgG2_G0F/SUM(G0F+G1F+G2F+G0FN+G1FN+G2FN+G1FS1+G2FS1+G1FNS1+G2FNS1)*
	**LC_IGPSC_tmp32**	IgG2_G1F(10)	*The percentage of IgG2_G1F in fucosylated IgG2 glycans*	*IgG2_G1F/SUM(G0F+G1F+G2F+G0FN+G1FN+G2FN+G1FS1+G2FS1+G1FNS1+G2FNS1)*
	**LC_IGPSC_tmp33**	IgG2_G2F(10)	*The percentage of IgG2_G2F in fucosylated IgG2 glycans*	*IgG2_G2F/SUM(G0F+G1F+G2F+G0FN+G1FN+G2FN+G1FS1+G2FS1+G1FNS1+G2FNS1)*
	**LC_IGPSC_tmp34**	IgG2_G0FN(10)	*The percentage of IgG2_G0FN in fucosylated IgG2 glycans*	*IgG2_G0FN/SUM(G0F+G1F+G2F+G0FN+G1FN+G2FN+G1FS1+G2FS1+G1FNS1+G2FNS1)*
	**LC_IGPSC_tmp35**	IgG2_G1FN(10)	*The percentage of IgG2_G1FN in fucosylated IgG2 glycans*	*IgG2_G1FN/SUM(G0F+G1F+G2F+G0FN+G1FN+G2FN+G1FS1+G2FS1+G1FNS1+G2FNS1)*
	**LC_IGPSC_tmp36**	IgG2_G2FN(10)	*The percentage of IgG2_G2FN in fucosylated IgG2 glycans*	*IgG2_G2FN/SUM(G0F+G1F+G2F+G0FN+G1FN+G2FN+G1FS1+G2FS1+G1FNS1+G2FNS1)*
	**LC_IGPSC_tmp37**	IgG2_G1FS1(10)	*The percentage of IgG2_G1FS1 in fucosylated IgG2 glycans*	*IgG2_G1FS1/SUM(G0F+G1F+G2F+G0FN+G1FN+G2FN+G1FS1+G2FS1+G1FNS1+G2FNS1)*
	**LC_IGPSC_tmp38**	IgG2_G2FS1(10)	*The percentage of IgG2_G2FS1 in fucosylated IgG2 glycans*	*IgG2_G2FS1/SUM(G0F+G1F+G2F+G0FN+G1FN+G2FN+G1FS1+G2FS1+G1FNS1+G2FNS1)*
	**LC_IGPSC_tmp39**	IgG2_G1FNS1(10)	*The percentage of IgG2_G1FNS1 in fucosylated IgG2 glycans*	*IgG2_G1FNS1/SUM(G0F+G1F+G2F+G0FN+G1FN+G2FN+G1FS1+G2FS1+G1FNS1+G2FNS1)*
	**LC_IGPSC_tmp40**	IgG2_G2FNS1(10)	*The percentage of IgG2_G2FNS1 in fucosylated IgG2 glycans*	*IgG2_G2FNS1/SUM(G0F+G1F+G2F+G0FN+G1FN+G2FN+G1FS1+G2FS1+G1FNS1+G2FNS1)*
IgG4 normalized on fucosylated IgG4 Glycans	**LC_IGPSC_tmp41**	IgG4_G0F(10)	*The percentage of IgG4_G0F in fucosylated IgG4 glycans*	*IgG4_G0F/SUM(G0F+G1F+G2F+G0FN+G1FN+G2FN+G1FS1+G2FS1+G1FNS1+G2FNS1)*
	**LC_IGPSC_tmp42**	IgG4_G1F(10)	*The percentage of IgG4_G1F in fucosylated IgG4 glycans*	*IgG4_G1F/SUM(G0F+G1F+G2F+G0FN+G1FN+G2FN+G1FS1+G2FS1+G1FNS1+G2FNS1)*
	**LC_IGPSC_tmp43**	IgG4_G2F(10)	*The percentage of IgG4_G2F in fucosylated IgG4 glycans*	*IgG4_G2F/SUM(G0F+G1F+G2F+G0FN+G1FN+G2FN+G1FS1+G2FS1+G1FNS1+G2FNS1)*
	**LC_IGPSC_tmp44**	IgG4_G0FN(10)	*The percentage of IgG4_G0FN in fucosylated IgG4 glycans*	*IgG4_G0FN/SUM(G0F+G1F+G2F+G0FN+G1FN+G2FN+G1FS1+G2FS1+G1FNS1+G2FNS1)*
	**LC_IGPSC_tmp45**	IgG4_G1FN(10)	*The percentage of IgG4_G1FN in fucosylated IgG4 glycans*	*IgG4_G1FN/SUM(G0F+G1F+G2F+G0FN+G1FN+G2FN+G1FS1+G2FS1+G1FNS1+G2FNS1)*
	**LC_IGPSC_tmp46**	IgG4_G2FN(10)	*The percentage of IgG4_G2FN in fucosylated IgG4 glycans*	*IgG4_G2FN/SUM(G0F+G1F+G2F+G0FN+G1FN+G2FN+G1FS1+G2FS1+G1FNS1+G2FNS1)*
	**LC_IGPSC_tmp47**	IgG4_G1FS1(10)	*The percentage of IgG4_G1FS1 in fucosylated IgG4 glycans*	*IgG4_G1FS1/SUM(G0F+G1F+G2F+G0FN+G1FN+G2FN+G1FS1+G2FS1+G1FNS1+G2FNS1)*
	**LC_IGPSC_tmp48**	IgG4_G2FS1(10)	*The percentage of IgG4_G2FS1 in fucosylated IgG4 glycans*	*IgG4_G2FS1/SUM(G0F+G1F+G2F+G0FN+G1FN+G2FN+G1FS1+G2FS1+G1FNS1+G2FNS1)*
	**LC_IGPSC_tmp49**	IgG4_G1FNS1(10)	*The percentage of IgG4_G1FNS1 in fucosylated IgG4 glycans*	*IgG4_G1FNS1/SUM(G0F+G1F+G2F+G0FN+G1FN+G2FN+G1FS1+G2FS1+G1FNS1+G2FNS1)*
	**LC_IGPSC_tmp50**	IgG4_G2FNS1(10)	*The percentage of IgG4_G2FNS1 in fucosylated IgG4 glycans*	*IgG4_G2FNS1/SUM(G0F+G1F+G2F+G0FN+G1FN+G2FN+G1FS1+G2FS1+G1FNS1+G2FNS1)*
Subclass Ratios for IgG1 and IgG4 on Total Area Norm	**LC_IGP_SC21**	IgG1_G0F(10)/IgG4_G0F	*The relation of percentage of IgG1_G0F in fucosylated IgG1 glycans and percentage of IgG4_G0F in total IgG4 glycans (only fucosylated available)*	*IgG1_G0F(10)/IgG4_G0F(10)*
	**LC_IGP_SC22**	IgG1_G1F(10)/IgG4_G1F	*The relation of percentage of IgG1_G1F in fucosylated IgG1 glycans and percentage of IgG4_G1F in total IgG4 glycans (only fucosylated available)*	*IgG1_G1F(10)/IgG4_G1F(10)*
	**LC_IGP_SC23**	IgG1_G2F(10)/IgG4_G2F	*The relation of percentage of IgG1_G2F in fucosylated IgG1 glycans and percentage of IgG4_G2F in total IgG4 glycans (only fucosylated available)*	*IgG1_G2F(10)/IgG4_G2F(10)*
	**LC_IGP_SC24**	IgG1_G0FN(10)/IgG4_G0FN	*The relation of percentage of IgG1_G0FN in fucosylated IgG1 glycans and percentage of IgG4_G0FN in total IgG4 glycans (only fucosylated available)*	*IgG1_G0FN(10)/IgG4_G0FN(10)*
	**LC_IGP_SC25**	IgG1_G1FN(10)/IgG4_G1FN	*The relation of percentage of IgG1_G1FN in fucosylated IgG1 glycans and percentage of IgG4_G1FN in total IgG4 glycans (only fucosylated available)*	*IgG1_G1FN(10)/IgG4_G1FN(10)*
	**LC_IGP_SC26**	IgG1_G2FN(10)/IgG4_G2FN	*The relation of percentage of IgG1_G2FN in fucosylated IgG1 glycans and percentage of IgG4_G2FN in total IgG4 glycans (only fucosylated available)*	*IgG1_G2FN(10)/IgG4_G2FN(10)*
	**LC_IGP_SC27**	IgG1_G1FS1(10)/IgG4_G1FS1	*The relation of percentage of IgG1_G1FS1 in fucosylated IgG1 glycans and percentage of IgG4_G1FS1 in total IgG4 glycans (only fucosylated available)*	*IgG1_G1FS1(10)/IgG4_G1FS1(10)*
	**LC_IGP_SC28**	IgG1_G2FS1(10)/IgG4_G2FS1	*The relation of percentage of IgG1_G2FS1 in fucosylated IgG1 glycans and percentage of IgG4_G2FS1 in total IgG4 glycans (only fucosylated available)*	*IgG1_G2FS1(10)/IgG4_G2FS1(10)*
	**LC_IGP_SC29**	IgG1_G1FNS1(10)/IgG4_G1FNS1	*The relation of percentage of IgG1_G1FNS1 in fucosylated IgG1 glycans and percentage of IgG4_G1FNS1 in total IgG4 glycans (only fucosylated available)*	*IgG1_G1FNS1(10)/IgG4_G1FNS1(10)*
	**LC_IGP_SC30**	IgG1_G2FNS1(10)/IgG4_G2FNS1	*The relation of percentage of IgG1_G2FNS1 in fucosylated IgG1 glycans and percentage of IgG4_G2FNS1 in total IgG4 glycans (only fucosylated available)*	*IgG1_G2FNS1(10)/IgG4_G2FNS1(10)*
Subclass Ratios for IgG2 and IgG4 on Total Area Norm	**LC_IGP_SC31**	IgG2_G0F(10)/IgG4_G0F	*The relation of percentage of IgG2_G0F in fucosylated IgG2 glycans and percentage of IgG4_G0F in total IgG4 glycans (only fucosylated available)*	*IgG2_G0F(10)/IgG4_G0F(10)*
	**LC_IGP_SC32**	IgG2_G1F(10)/IgG4_G1F	*The relation of percentage of IgG2_G1F in fucosylated IgG2 glycans and percentage of IgG4_G1F in total IgG4 glycans (only fucosylated available)*	*IgG2_G1F(10)/IgG4_G1F(10)*
	**LC_IGP_SC33**	IgG2_G2F(10)/IgG4_G2F	*The relation of percentage of IgG2_G2F in fucosylated IgG2 glycans and percentage of IgG4_G2F in total IgG4 glycans (only fucosylated available)*	*IgG2_G2F(10)/IgG4_G2F(10)*
	**LC_IGP_SC34**	IgG2_G0FN(10)/IgG4_G0FN	*The relation of percentage of IgG2_G0FN in fucosylated IgG2 glycans and percentage of IgG4_G0FN in total IgG4 glycans (only fucosylated available)*	*IgG2_G0FN(10)/IgG4_G0FN(10)*
	**LC_IGP_SC35**	IgG2_G1FN(10)/IgG4_G1FN	*The relation of percentage of IgG2_G1FN in fucosylated IgG2 glycans and percentage of IgG4_G1FN in total IgG4 glycans (only fucosylated available)*	*IgG2_G1FN(10)/IgG4_G1FN(10)*
	**LC_IGP_SC36**	IgG2_G2FN(10)/IgG4_G2FN	*The relation of percentage of IgG2_G2FN in fucosylated IgG2 glycans and percentage of IgG4_G2FN in total IgG4 glycans (only fucosylated available)*	*IgG2_G2FN(10)/IgG4_G2FN(10)*
	**LC_IGP_SC37**	IgG2_G1FS1(10)/IgG4_G1FS1	*The relation of percentage of IgG2_G1FS1 in fucosylated IgG2 glycans and percentage of IgG4_G1FS1 in total IgG4 glycans (only fucosylated available)*	*IgG2_G1FS1(10)/IgG4_G1FS1(10)*
	**LC_IGP_SC38**	IgG2_G2FS1(10)/IgG4_G2FS1	*The relation of percentage of IgG2_G2FS1 in fucosylated IgG2 glycans and percentage of IgG4_G2FS1 in total IgG4 glycans (only fucosylated available)*	*IgG2_G2FS1(10)/IgG4_G2FS1(10)*
	**LC_IGP_SC39**	IgG2_G1FNS1(10)/IgG4_G1FNS1	*The relation of percentage of IgG2_G1FNS1 in fucosylated IgG2 glycans and percentage of IgG4_G1FNS1 in total IgG4 glycans (only fucosylated available)*	*IgG2_G1FNS1(10)/IgG4_G1FNS1(10)*
	**LC_IGP_SC40**	IgG2_G2FNS1(10)/IgG4_G2FNS1	*The relation of percentage of IgG2_G2FNS1 in fucosylated IgG2 glycans and percentage of IgG4_G2FNS1 in total IgG4 glycans (only fucosylated available)*	*IgG2_G2FNS1(10)/IgG4_G2FNS1(10)*
**Glycan Proportions**				
IgG1 Glycans Percentage per Glycan on Total Area Norm	**LC_IGPRG1**	IgG1_G0F_pG	*The percentage of IgG1_G0F in G0F glycans for IgG1, IgG2 and IgG4*	*IgG1_G0F(10)/ SUM(IgG1_G0F(10)+IgG2_G0F(10)+IgG4_G0F(10))*
	**LC_IGPRG2**	IgG1_G1F_pG	*The percentage of IgG1_G1F in G1F glycans for IgG1, IgG2 and IgG4*	*IgG1_G1F(10)/ SUM(IgG1_G1F(10)+IgG2_G1F(10)+IgG4_G1F(10))*
	**LC_IGPRG3**	IgG1_G2F_pG	*The percentage of IgG1_G2F in G2F glycans for IgG1, IgG2 and IgG4*	*IgG1_G2F(10)/ SUM(IgG1_G2F(10)+IgG2_G2F(10)+IgG4_G2F(10))*
	**LC_IGPRG4**	IgG1_G0FN_pG	*The percentage of IgG1_G0FN in G0FN glycans for IgG1, IgG2 and IgG4*	*IgG1_G0FN(10)/ SUM(IgG1_G0FN(10)+IgG2_G0FN(10)+IgG4_G0FN(10))*
	**LC_IGPRG5**	IgG1_G1FN_pG	*The percentage of IgG1_G1FN in G1FN glycans for IgG1, IgG2 and IgG4*	*IgG1_G1FN(10)/ SUM(IgG1_G1FN(10)+IgG2_G1FN(10)+IgG4_G1FN(10))*
	**LC_IGPRG6**	IgG1_G2FN_pG	*The percentage of IgG1_G2FN in G2FN glycans for IgG1, IgG2 and IgG4*	*IgG1_G2FN(10)/ SUM(IgG1_G2FN(10)+IgG2_G2FN+IgG4_G2FN(10))*
	**LC_IGPRG7**	IgG1_G1FS1_pG	*The percentage of IgG1_G1FS1 in G1FS1 glycans for IgG1, IgG2 and IgG4*	*IgG1_G1FS1(10)/ SUM(IgG1_G1FS1(10)+IgG2_G1FS1(10)+IgG4_G1FS1(10))*
	**LC_IGPRG8**	IgG1_G2FS1_pG	*The percentage of IgG1_G2FS1 in G2FS1 glycans for IgG1, IgG2 and IgG4*	*IgG1_G2FS1(10)/ SUM(IgG1_G2FS1(10)+IgG2_G2FS1(10)+IgG4_G2FS1(10))*
	**LC_IGPRG9**	IgG1_G1FNS1_pG	*The percentage of IgG1_G1FNS1 in G1FNS1 glycans for IgG1, IgG2 and IgG4*	*IgG1_G1FNS1(10)/ SUM(IgG1_G1FNS1(10)+IgG2_G1FNS1(10)+IgG4_G1FNS1(10))*
	**LC_IGPRG10**	IgG1_G2FNS1_pG	*The percentage of IgG1_G2FNS1 in G2FNS1 glycans for IgG1, IgG2 and IgG4*	*IgG1_G2FNS1(10)/ SUM(IgG1_G2FNS1(10)+IgG2_G2FNS1(10)+IgG4_G2FNS1(10))*
IgG2 Glycans Percentage per Glycan on Total Area Norm	**LC_IGPRG11**	IgG2_G0F_pG	*The percentage of IgG2_G0F in G0F glycans for IgG1, IgG2 and IgG4*	*IgG2_G0F(10)/ SUM(IgG1_G0F(10)+IgG2_G0F(10)+IgG4_G0F(10))*
	**LC_IGPRG12**	IgG2_G1F_pG	*The percentage of IgG2_G1F in G1F glycans for IgG1, IgG2 and IgG4*	*IgG2_G1F(10)/ SUM(IgG1_G1F(10)+IgG2_G1F(10)+IgG4_G1F(10))*
	**LC_IGPRG13**	IgG2_G2F_pG	*The percentage of IgG2_G2F in G2F glycans for IgG1, IgG2 and IgG4*	*IgG2_G2F(10)/ SUM(IgG1_G2F(10)+IgG2_G2F(10)+IgG4_G2F(10))*
	**LC_IGPRG14**	IgG2_G0FN_pG	*The percentage of IgG2_G0FN in G0FN glycans for IgG1, IgG2 and IgG4*	*IgG2_G0FN(10)/ SUM(IgG1_G0FN(10)+IgG2_G0FN(10)+IgG4_G0FN(10))*
	**LC_IGPRG15**	IgG2_G1FN_pG	*The percentage of IgG2_G1FN in G1FN glycans for IgG1, IgG2 and IgG4*	*IgG2_G1FN(10)/ SUM(IgG1_G1FN(10)+IgG2_G1FN(10)+IgG4_G1FN(10))*
	**LC_IGPRG16**	IgG2_G2FN_pG	*The percentage of IgG2_G2FN in G2FN glycans for IgG1, IgG2 and IgG4*	*IgG2_G2FN/(10) SUM(IgG1_G2FN(10)+IgG2_G2FN(10)+IgG4_G2FN(10))*
	**LC_IGPRG17**	IgG2_G1FS1_pG	*The percentage of IgG2_G1FS1 in G1FS1 glycans for IgG1, IgG2 and IgG4*	*IgG2_G1FS1(10)/ SUM(IgG1_G1FS1(10)+IgG2_G1FS1(10)+IgG4_G1FS1(10))*
	**LC_IGPRG18**	IgG2_G2FS1_pG	*The percentage of IgG2_G2FS1 in G2FS1 glycans for IgG1, IgG2 and IgG4*	*IgG2_G2FS1(10)/ SUM(IgG1_G2FS1(10)+IgG2_G2FS1(10)+IgG4_G2FS1(10))*
	**LC_IGPRG19**	IgG2_G1FNS1_pG	*The percentage of IgG2_G1FNS1 in G1FNS1 glycans for IgG1, IgG2 and IgG4*	*IgG2_G1FNS1(10)/ SUM(IgG1_G1FNS1(10)+IgG2_G1FNS1(10)+IgG4_G1FNS1(10))*
	**LC_IGPRG20**	IgG2_G2FNS1_pG	*The percentage of IgG2_G2FNS1 in G2FNS1 glycans for IgG1, IgG2 and IgG4*	*IgG2_G2FNS1(10)/ SUM(IgG1_G2FNS1(10)+IgG2_G2FNS1(10)+IgG4_G2FNS1(10))*
IgG4 Glycans Percentage per Glycan on Total Area Norm	**LC_IGPRG21**	IgG4_G0F_pG	*The percentage of IgG4_G0F in G0F glycans for IgG1, IgG2 and IgG4*	*IgG4_G0F/ SUM(IgG1_G0F(10)+IgG2_G0F(10)+IgG4_G0F(10))*
	**LC_IGPRG22**	IgG4_G1F_pG	*The percentage of IgG4_G1F in G1F glycans for IgG1, IgG2 and IgG4*	*IgG4_G1F/ SUM(IgG1_G1F(10)+IgG2_G1F(10)+IgG4_G1F(10))*
	**LC_IGPRG23**	IgG4_G2F_pG	*The percentage of IgG4_G2F in G2F glycans for IgG1, IgG2 and IgG4*	*IgG4_G2F/ SUM(IgG1_G2F(10)+IgG2_G2F(10)+IgG4_G2F(10))*
	**LC_IGPRG24**	IgG4_G0FN_pG	*The percentage of IgG4_G0FN in G0FN glycans for IgG1, IgG2 and IgG4*	*IgG4_G0FN/ SUM(IgG1_G0FN(10)+IgG2_G0FN(10)+IgG4_G0FN(10))*
	**LC_IGPRG25**	IgG4_G1FN_pG	*The percentage of IgG4_G1FN in G1FN glycans for IgG1, IgG2 and IgG4*	*IgG4_G1FN/ SUM(IgG1_G1FN(10)+IgG2_G1FN(10)+IgG4_G1FN(10))*
	**LC_IGPRG26**	IgG4_G2FN_pG	*The percentage of IgG4_G2FN in G2FN glycans for IgG1, IgG2 and IgG4*	*IgG4_G2FN/ SUM(IgG1_G2FN(10)+IgG2_G2FN+(10)IgG4_G2FN(10))*
	**LC_IGPRG27**	IgG4_G1FS1_pG	*The percentage of IgG4_G1FS1 in G1FS1 glycans for IgG1, IgG2 and IgG4*	*IgG4_G1FS1/ SUM(IgG1_G1FS1(10)+IgG2_G1FS1(10)+IgG4_G1FS1(10))*
	**LC_IGPRG28**	IgG4_G2FS1_pG	*The percentage of IgG4_G2FS1 in G2FS1 glycans for IgG1, IgG2 and IgG4*	*IgG4_G2FS1/ SUM(IgG1_G2FS1(10)+IgG2_G2FS1(10)+IgG4_G2FS1(10))*
	**LC_IGPRG29**	IgG4_G1FNS1_pG	*The percentage of IgG4_G1FNS1 in G1FNS1 glycans for IgG1, IgG2 and IgG4*	*IgG4_G1FNS1/ SUM(IgG1_G1FNS1(10)+IgG2_G1FNS1(10)+IgG4_G1FNS1(10))*
	**LC_IGPRG30**	IgG4_G2FNS1_pG	*The percentage of IgG4_G2FNS1 in G2FNS1 glycans for IgG1, IgG2 and IgG4*	*IgG4_G2FNS1/ SUM(IgG1_G2FNS1(10)+IgG2_G2FNS1(10)+IgG4_G2FNS1(10))*
Sums of Glycans per Subclasses on Total Area Norm	**LC_IGPRG31**	sumIgG1	*The sum of the percentages of glycans of IgG1*	*SUM(IgG1_G0F_pG+IgG1_G1F_pG+IgG1_G2F_pG+IgG1_G0FN_pG+IgG1_G1FN_pG+IgG1_G2FN_pG+IgG1_G1FS1_pG+IgG1_G2FS1_pG+IgG1_G1FNS1_pG+IgG1_G2FNS1_pG)*
	**LC_IGPRG32**	sumIgG2	*The sum of the percentages of glycans of IgG2*	*SUM(IgG2_G0F_pG+IgG2_G1F_pG+IgG2_G2F_pG+IgG2_G0FN_pG+IgG2_G1FN_pG+IgG2_G2FN_pG+IgG2_G1FS1_pG+IgG2_G2FS1_pG+IgG2_G1FNS1_pG+IgG2_G2FNS1_pG)*
	**LC_IGPRG33**	sumIgG4	*The sum of the percentages of glycans of IgG4*	*SUM(IgG4_G0F_pG+IgG4_G1F_pG+IgG4_G2F_pG+IgG4_G0FN_pG+IgG4_G1FN_pG+IgG4_G2FN_pG+IgG4_G1FS1_pG+IgG4_G2FS1_pG+IgG4_G1FNS1_pG+IgG4_G2FNS1_pG)*
	**LC_IGP_RG34**	sumIgG1/sumIgG4	*The relation of the percentage of the sum of the percentages of glycans in IgG1*	*sumIgG1/sumIgG4*
	**LC_IGP_RG35**	sumIgG2/sumIgG4	*The relation of the percentage of the sum of the percentages of glycans in IgG2*	*sumIgG2/sumIgG4*

**LC_IGP_RG36**	sumIgG1/sumIgG2	*The relation of the percentage of the sum of the percentages of glycans in IgG4*	*sumIgG1/sumIgG2*
