# Supplementary material for: Genome-Wide Association Study on Immunoglobulin G Glycosylation Patterns
Source: Front Immunol. 2018 Feb 26;9:277. doi: 10.3389/fimmu.2018.00277 (PMC5834439; doi:10.3389/fimmu.2018.00277)
Supplement: Table S2 — Study characteristics of the discovery cohort KORA. [file Table_2.PDF]

Study Characteristics for KORA

GWAS Code	n	Mean	Median	Minimum	Maximum	Standard Deviation	Interquartile Range
LC_IGP_R1	1819	2.557658	2.564603	0.5345351	5.04359	0.552436	0.7414257
LC_IGP_R10	1800	0.1764059	0.1982109	-2.350257	2.185829	0.5525667	0.6915792
LC_IGP_R11	1821	-1.38716	-1.38342	-2.629209	1.445383	0.2473286	0.305859
LC_IGP_R12	1820	-1.26483	-1.259033	-2.815412	-0.4340471	0.2354254	0.3135442
LC_IGP_R13	1818	-2.211736	-2.21591	-3.380404	-0.4009082	0.256279	0.3302451
LC_IGP_R14	1803	-1.126819	-1.126643	-2.629632	0.2685622	0.3075631	0.3859842
LC_IGP_R15	1818	-2.508891	-2.507339	-4.285896	0.8122876	0.4108253	0.5037959
LC_IGP_R16	1804	-1.225821	-1.212876	-3.133044	0.6745939	0.3505077	0.3862617
LC_IGP_R17	1800	-1.584757	-1.599721	-3.39324	0.431661	0.3933104	0.5130587
LC_IGP_R18	1814	-1.252067	-1.227637	-4.046586	0.6984028	0.5192009	0.652381
LC_IGP_R19	1799	-0.4330168	-0.4369048	-2.350079	1.827594	0.606384	0.7839063
LC_IGP_R2	1819	2.311744	2.330755	0.191448	3.638419	0.4587795	0.6354098
LC_IGP_R20	1818	0.3395476	0.3222372	-0.7906197	3.154059	0.3406445	0.4310292
LC_IGP_R21	1803	0.212931	0.2069086	-1.100092	1.582306	0.389579	0.4829959
LC_IGP_R22	1821	0.09451894	0.1116488	-1.154662	1.077431	0.3029634	0.4036395
LC_IGP_R23	1819	0.2192059	0.2170581	-1.170053	1.501089	0.3080492	0.4050958
LC_IGP_R24	1818	-0.566715	-0.574609	-1.771767	0.3962367	0.2654406	0.3492721
LC_IGP_R25	1817	0.3117286	0.3083443	-1.987902	2.415697	0.448225	0.5615357
LC_IGP_R26	1800	-0.9228295	-0.9531669	-2.643994	0.8304444	0.4131258	0.5052993
LC_IGP_R27	1796	1.131069	1.080168	-0.9767197	3.813221	0.6127158	0.7802045
LC_IGP_R28	1820	-0.9771426	-0.986816	-1.671104	-0.2029261	0.2463902	0.3216625
LC_IGP_R29	1817	1.065374	1.052348	-0.7922975	2.173805	0.3261536	0.4100399
LC_IGP_R3	1818	1.902197	1.902736	0.737542	3.309211	0.3950559	0.5453329
LC_IGP_R30	1805	-0.3176653	-0.2857359	-2.134028	1.146021	0.3713657	0.4480281
LC_IGP_R31	1818	-1.924627	-1.938499	-2.678853	0.01027563	0.2423779	0.3057486
LC_IGP_R32	1816	-1.954209	-1.973804	-3.205871	-0.07401565	0.4412025	0.5918864
LC_IGP_R33	1818	-1.077039	-1.059785	-2.541301	0.1800043	0.2681504	0.3016974
LC_IGP_R34	1817	-2.768481	-2.766594	-4.505908	-2.028919	0.1708294	0.2017934
LC_IGP_R35	1820	-0.7254182	-0.7240236	-3.223991	0.07864253	0.1808086	0.192486
LC_IGP_R36	1802	-1.983895	-2.011425	-4.286993	0.795154	0.6604832	0.8635032

Age

Samples	1823
Mean	62.56665
SD	9.889168
min	32
max	81

Sex

women	935
men	888
%women	0.512891

LC_IGP_R37	1788	0.07354986	0.05076495	-1.955487	2.238489	0.4974415	0.6289327
LC_IGP_R38	1805	-2.632252	-2.637246	-4.063109	-1.046029	0.3137483	0.3987614
LC_IGP_R39	1812	-1.095459	-1.098474	-2.84387	1.138524	0.3689306	0.4266073
LC_IGP_R4	1814	2.264532	2.242137	0.4786843	4.913141	0.5660834	0.7401642
LC_IGP_R40	1816	-1.021912	-1.014084	-2.366259	0.5615654	0.3392183	0.4393171
LC_IGP_R41	1817	3.379709	3.388501	1.35505	4.973315	0.4792449	0.6688372
LC_IGP_R42	1817	2.555401	2.561177	-0.3597875	4.193181	0.4639631	0.5986903
LC_IGP_R43	1819	1.655556	1.666528	-0.3533712	3.067189	0.4801752	0.6179551
LC_IGP_R44	1812	3.153077	3.17573	0.7248161	4.633051	0.5264667	0.6889192
LC_IGP_R45	1818	1.66948	1.684766	-0.1309449	3.158903	0.534572	0.7035602
LC_IGP_R46	1817	1.035237	1.040334	-0.8332803	2.974839	0.5339136	0.6644491
LC_IGP_R47	1816	2.260743	2.273681	-1.156507	3.559498	0.4157707	0.5224483
LC_IGP_R48	1819	2.281555	2.289655	0.05016709	4.064402	0.4267356	0.5693205
LC_IGP_R49	1810	0.2838378	0.2811785	-1.48246	3.082172	0.5002375	0.6521024
LC_IGP_R5	1806	2.276131	2.258103	0.5860739	4.746194	0.5365494	0.7360684
LC_IGP_R50	1806	0.1933049	0.2001579	-1.621448	2.173263	0.4927982	0.6121407
LC_IGP_R51	1821	-1.527547	-1.531513	-2.232441	-0.2730191	0.2309252	0.297175
LC_IGP_R52	1819	-1.83455	-1.839567	-2.649482	-0.8693269	0.2372789	0.3052066
LC_IGP_R53	1821	-1.903315	-1.915159	-3.028128	-0.8334327	0.2460319	0.3264604
LC_IGP_R54	1817	-2.247763	-2.256694	-3.392689	-0.2088209	0.3847774	0.496583
LC_IGP_R55	1818	-2.562935	-2.557417	-4.012804	-1.088754	0.3790142	0.4946793
LC_IGP_R56	1814	-0.9479266	-0.9534293	-2.157412	0.9669001	0.3483922	0.4090734
LC_IGP_R57	1816	-1.282469	-1.283385	-2.663869	0.4939329	0.3515844	0.4454075
LC_IGP_R58	1809	-0.2679584	-0.2669867	-2.676964	1.611146	0.5375163	0.6768967
LC_IGP_R59	1806	-0.4723689	-0.4469922	-2.674433	0.9747786	0.4666995	0.5961225
LC_IGP_R6	1799	1.274834	1.273025	-0.2187524	3.386814	0.4159017	0.5207601
LC_IGP_R60	1813	0.5026233	0.4617503	-0.6501885	3.835353	0.4252403	0.5117329
LC_IGP_R61	1811	0.8542112	0.8355103	-0.9035979	2.669748	0.4937062	0.6044773
LC_IGP_R62	1821	-0.3204535	-0.3179997	-1.770629	0.7047194	0.3097567	0.3817912
LC_IGP_R63	1820	-0.6266687	-0.6269267	-1.755909	0.809863	0.2987171	0.3742485
LC_IGP_R64	1814	-0.499014	-0.5051339	-1.404703	1.27281	0.2618567	0.3290892
LC_IGP_R65	1816	0.1592979	0.1570148	-1.595714	2.159495	0.3797995	0.4601072
LC_IGP_R66	1816	-0.8323848	-0.824392	-2.104862	0.5864282	0.3552693	0.4490589

LC_IGP_R67	1800	-0.04480253	-0.03779206	-2.083832	1.647284	0.446828	0.5478618
LC_IGP_R68	1821	-1.396229	-1.401504	-2.0966	-0.01923613	0.2285224	0.3120953
LC_IGP_R69	1820	0.1799198	0.1705164	-1.130087	1.741866	0.2036591	0.2568989
LC_IGP_R7	1816	1.499337	1.490814	-0.8095361	3.03767	0.3821577	0.4829559
LC_IGP_R70	1817	-0.134798	-0.1187724	-1.56646	1.314891	0.3455607	0.4308716
LC_IGP_R71	1820	-1.466315	-1.471207	-2.354838	-0.3980567	0.2056261	0.2582325
LC_IGP_R72	1812	-1.685501	-1.666858	-3.583422	0.7775225	0.4881355	0.6117952
LC_IGP_R73	1817	-1.028792	-1.008746	-3.230062	0.3223649	0.3656265	0.4734063
LC_IGP_R74	1821	-1.98039	-1.980608	-3.178248	-0.1236571	0.1732933	0.2085757
LC_IGP_R75	1821	-0.4043703	-0.4042475	-2.066453	1.456152	0.1911633	0.2308832
LC_IGP_R76	1810	-1.006377	-0.996661	-3.368433	0.8683913	0.4787179	0.6097235
LC_IGP_R77	1804	-0.2173411	-0.1982562	-2.051704	1.452791	0.4465435	0.5552269
LC_IGP_R78	1817	-2.394275	-2.409329	-3.669029	-0.576792	0.3901464	0.5063085
LC_IGP_R79	1809	-1.30112	-1.320864	-2.740291	1.97205	0.4970233	0.599384
LC_IGP_R8	1819	2.252768	2.253862	-0.8742467	4.135896	0.4369713	0.5544157
LC_IGP_R80	1818	-1.061606	-1.061963	-2.445143	0.5691945	0.3444155	0.4345056
LC_IGP_R81	1821	-1.131456	-1.137683	-2.209425	0.4990468	0.3024944	0.3884682
LC_IGP_R82	1821	-1.195518	-1.198465	-2.213852	0.1001384	0.2893309	0.380084
LC_IGP_R83	1821	-1.81295	-1.804611	-3.927081	0.2499478	0.3941058	0.4669865
LC_IGP_R84	1820	-1.356327	-1.404595	-2.670104	1.323259	0.5570663	0.7122751
LC_IGP_R85	1818	-2.630569	-2.666196	-4.148516	0.2645583	0.5564134	0.7045846
LC_IGP_R86	1823	-0.2780587	-0.2485236	-1.419771	0.6190547	0.3163714	0.4380609
LC_IGP_R87	1820	-0.3415703	-0.3288847	-1.532471	1.09212	0.3286793	0.4299075
LC_IGP_R88	1823	-1.154077	-1.154965	-2.095024	0.8901596	0.2576795	0.3405915
LC_IGP_R89	1821	0.4691303	0.4744788	-0.6628051	1.604974	0.2972763	0.3792515
LC_IGP_R9	1801	1.626056	1.57739	-0.41537	4.218735	0.5305599	0.670162
LC_IGP_R90	1817	-0.8044815	-0.7972853	-2.496367	0.9768458	0.3863435	0.4587273
LC_IGP_R91	1820	-1.772756	-1.751732	-4.181546	-0.1637475	0.3456517	0.3920477
LC_IGP_R92	1822	-1.660523	-1.670717	-3.155487	-0.09119216	0.2094952	0.2340547
LC_IGP_R93	1821	-0.0369395	-0.03610021	-1.718418	1.698843	0.21174	0.2463252
LC_IGP_R94	1820	-1.821834	-1.877839	-3.368374	0.5328049	0.5133788	0.6609253
LC_IGP_R95	1817	-0.8523308	-0.8918655	-2.270811	1.335968	0.4943929	0.581539
LC_IGP_RG34	1796	-0.1286193	-0.1187796	-0.8791783	0.3645716	0.1471468	0.1884241

LC_IGP_RG35	1796	-0.2494821	-0.2420522	-1.009327	0.338794	0.1503178	0.1796791
LC_IGP_RG36	1796	0.1208628	0.1197834	-0.3626277	0.6246748	0.1060264	0.1307594
LC_IGP_SC1	1821	-0.3437222	-0.3412626	-1.41251	0.4667958	0.1553341	0.1952502
LC_IGP_SC10	1816	0.2249771	0.2520192	-1.898584	1.799056	0.4233997	0.5178746
LC_IGP_SC11	1814	0.4781492	0.4789423	-2.753927	1.900377	0.4102915	0.5034856
LC_IGP_SC12	1814	0.318137	0.3394565	-1.476229	1.882909	0.4887039	0.6183064
LC_IGP_SC13	1816	0.2480217	0.2778202	-1.834645	1.613232	0.5301801	0.7014704
LC_IGP_SC14	1807	0.684806	0.7169597	-1.843081	2.539235	0.5750874	0.7115603
LC_IGP_SC15	1804	0.04284694	0.09153511	-2.517318	2.029845	0.6415569	0.8066412
LC_IGP_SC16	1798	-0.05007309	-0.009549963	-2.564938	1.803248	0.5848536	0.7753365
LC_IGP_SC17	1814	0.05014085	0.05979552	-2.102679	1.842693	0.4706856	0.5656267
LC_IGP_SC18	1817	0.1993726	0.2283279	-2.575624	2.061674	0.514782	0.665596
LC_IGP_SC19	1807	-0.9335963	-0.9134205	-3.470971	1.333757	0.6627811	0.8395784
LC_IGP_SC2	1821	0.07208008	0.07003351	-0.3039303	0.6710201	0.1111992	0.133795
LC_IGP_SC20	1787	0.2403644	0.2333872	-2.877558	2.813891	0.6116159	0.7793078
LC_IGP_SC21	1797	-0.1221105	-0.1302628	-0.7202616	1.15143	0.1735048	0.2021516
LC_IGP_SC22	1797	0.2511184	0.2382205	-0.159332	0.9347243	0.1146166	0.1374242
LC_IGP_SC23	1797	0.4295488	0.4220119	-0.6842059	1.529034	0.2129768	0.2665035
LC_IGP_SC24	1797	-0.3771833	-0.3615101	-1.442468	0.697411	0.2463362	0.3074198
LC_IGP_SC25	1797	0.1845061	0.1861119	-0.7638944	0.8638415	0.1662139	0.2028595
LC_IGP_SC26	1797	0.03423414	0.02678917	-1.929352	2.558872	0.3610554	0.4121049
LC_IGP_SC27	1797	-0.855292	-0.8528442	-2.220135	0.2376755	0.1814567	0.190787
LC_IGP_SC28	1797	-0.2580018	-0.2505541	-1.892092	0.6413918	0.2498785	0.3096982
LC_IGP_SC29	1797	-0.6207758	-0.5430021	-3.395735	1.070487	0.600999	0.7787886
LC_IGP_SC3	1820	0.4921201	0.4913332	-0.3275011	1.265876	0.1777604	0.228202
LC_IGP_SC30	1797	-0.136896	-0.07978609	-2.898278	1.428092	0.5636427	0.6830244
LC_IGP_SC31	1812	0.1947715	0.1849667	-0.3773685	1.362111	0.1811214	0.2152641
LC_IGP_SC32	1812	0.1528903	0.1470746	-0.7242737	0.860408	0.1404349	0.1681224
LC_IGP_SC33	1812	-0.08961449	-0.08921954	-1.373995	0.7936304	0.2479359	0.3123174
LC_IGP_SC34	1812	-0.2006705	-0.1992537	-1.232427	0.9028454	0.2339026	0.2959809
LC_IGP_SC35	1812	-0.4858399	-0.4810096	-1.506292	0.5668112	0.2016422	0.2520383
LC_IGP_SC36	1812	-0.1782024	-0.2014887	-2.436626	2.019729	0.3849848	0.4531456
LC_IGP_SC37	1812	-0.1679325	-0.1558692	-1.308215	0.4887063	0.2018325	0.2380486

LC_IGP_SC38	1812	-0.457842	-0.4684943	-1.54854	1.159963	0.2924308	0.3699842
LC_IGP_SC39	1812	-1.058183	-1.015688	-4.062527	1.013889	0.6074074	0.7345985
LC_IGP_SC4	1821	-0.2015671	-0.1986082	-1.427227	1.699627	0.1984701	0.2323881
LC_IGP_SC40	1812	-0.3873858	-0.3602531	-3.102433	1.348495	0.5688199	0.6954829
LC_IGP_SC5	1818	0.6427055	0.6394194	-0.4205361	1.334672	0.1674322	0.2111163
LC_IGP_SC6	1818	0.184789	0.180714	-0.9700454	1.13765	0.2489363	0.3302072
LC_IGP_SC7	1816	-0.7136298	-0.7146269	-1.625814	0.2086197	0.1891462	0.228029
LC_IGP_SC8	1820	0.1749854	0.1777577	-1.670855	2.129609	0.2449437	0.292779
LC_IGP_SC9	1802	0.4096432	0.4275591	-1.376775	1.719483	0.3656002	0.4144062
LC_IGP1	1822	26.1285	25.84306	5.4094	55.53319	6.455143	8.42037
LC_IGP10	1818	0.4468332	0.4292697	0.06799873	2.193963	0.1772164	0.2257727
LC_IGP100	1812	0.3778971	0.3256751	0.08137774	2.468975	0.223596	0.2267869
LC_IGP101	1819	0.8825679	0.7758665	0.17461	9.928279	0.50611	0.5238917
LC_IGP102	1817	0.3865693	0.3377497	0.0723265	1.711353	0.2140819	0.232557
LC_IGP103	1816	0.406304	0.3691893	0.1032629	3.453145	0.1952062	0.1995525
LC_IGP104	1820	0.4845514	0.4401369	0.05254244	5.301132	0.2555031	0.2617841
LC_IGP105	1811	0.3150654	0.2807355	0.04162053	1.424883	0.1557474	0.165512
LC_IGP106	1807	0.3045653	0.27337	0.06458656	2.98993	0.1624979	0.1694596
LC_IGP107	1817	91.42547	91.87658	77.90541	100.7219	3.163292	3.842503
LC_IGP108	1790	16.1924	15.98892	9.172099	31.27126	2.751323	3.597034
LC_IGP109	1791	34.15078	33.80599	15.94123	54.42305	5.79619	7.279071
LC_IGP11	1819	2.231121	1.953372	0.1004328	9.78285	1.170664	1.400529
LC_IGP110	1797	10.38367	10.10964	4.511919	23.38103	2.399871	2.973065
LC_IGP111	1791	15.11511	15.0733	7.345922	25.13883	1.683726	2.116702
LC_IGP112	1810	20.79762	20.45852	6.300599	45.53341	5.819099	7.72049
LC_IGP113	1807	15.76989	15.47142	5.303643	36.23489	4.345135	5.439913
LC_IGP114	1812	16.65348	15.88436	2.702959	68.51459	6.519976	8.192627
LC_IGP115	1817	26.92337	26.72253	3.804998	57.99005	6.855871	9.237644
LC_IGP116	1798	34.03811	33.92591	9.858317	61.33416	7.910672	10.53678
LC_IGP117	1791	28.24092	28.20493	7.225609	54.29596	6.719455	9.106065
LC_IGP118	1810	27.74172	26.95984	3.329672	70.44108	9.089833	11.98131
LC_IGP119	1804	44.86079	45.05977	11.38803	81.04276	10.42219	13.63761
LC_IGP12	1819	3.01398	2.748532	0.7251963	22.43758	1.377745	1.636528

LC_IGP120	1820	19.73574	19.50466	8.916347	46.44636	2.939209	3.598674
LC_IGP121	1819	10.56469	10.23396	4.005025	31.1502	2.830864	3.390101
LC_IGP122	1821	12.2485	12.12541	3.999255	46.91251	1.970808	2.221317
LC_IGP123	1821	40.10857	40.02923	11.24004	81.09434	4.482822	5.538455
LC_IGP124	1817	12.76667	12.20331	4.079472	46.26476	3.882122	4.625509
LC_IGP125	1817	5.493035	5.255554	1.452805	26.82564	1.876388	2.178996
LC_IGP126	1817	8.858093	8.246406	2.486707	35.96711	3.385656	3.871859
LC_IGP127	1818	26.22175	25.69345	7.979444	63.85773	6.615406	8.240155
LC_IGP128	1798	0.747453	0.7005901	0.1104962	2.190433	0.2927	0.3624232
LC_IGP129	1817	0.09660512	0.08989161	0.02956932	0.3790293	0.03580568	0.04111923
LC_IGP13	1819	1.735553	1.564489	0.2971756	6.060255	0.8182	1.03515
LC_IGP130	1798	0.4128233	0.4119688	0.09950163	0.6865629	0.09143683	0.1229781
LC_IGP131	1817	0.08716511	0.08247757	0.02872009	0.2748523	0.02854177	0.0344578
LC_IGP132	1802	40.77087	40.85079	19.05612	69.91225	6.963078	9.389124
LC_IGP133	1802	29.4815	29.77133	11.90088	42.62945	4.321206	5.696366
LC_IGP134	1802	7.625319	7.211691	2.203586	19.05194	2.620733	3.162704
LC_IGP135	1802	8.914858	8.671616	4.634005	23.92999	1.969101	2.42418
LC_IGP136	1802	4.806927	4.70234	1.678507	10.19936	1.204547	1.585143
LC_IGP137	1802	1.14191	1.060505	0.2625327	3.210702	0.4095892	0.5143967
LC_IGP138	1802	1.492136	1.353771	0.2875157	5.835637	0.6689934	0.7985949
LC_IGP139	1802	2.432542	2.235951	0.4971908	8.058147	1.013952	1.194245
LC_IGP14	1814	0.7572599	0.6584011	0.04589963	7.855713	0.4435794	0.4837262
LC_IGP140	1802	1.50484	1.322908	0.3764764	5.446688	0.7140704	0.77791
LC_IGP141	1802	0.4223004	0.3653926	0.08973971	2.482977	0.2418123	0.2551792
LC_IGP142	1802	0.9749499	0.8659713	0.1873556	3.900179	0.4961157	0.5845131
LC_IGP143	1802	0.4318378	0.3770033	0.07649104	1.90776	0.2384464	0.2621927
LC_IGP144	1802	51.60017	51.75225	27.05653	80.26068	7.726845	9.924018
LC_IGP145	1802	37.69592	37.95227	15.99725	53.07396	4.795975	6.160936
LC_IGP146	1802	10.70391	10.25287	3.534935	24.25815	3.246448	4.151654
LC_IGP147	1802	92.74139	93.23192	77.86637	97.57606	2.66977	3.293326
LC_IGP148	1802	96.19986	96.58508	80.72743	98.94659	1.740845	2.061487
LC_IGP149	1802	90.84014	91.55222	73.15133	97.41617	3.848189	4.62933
LC_IGP15	1806	0.9314829	0.8442882	0.07655251	3.412224	0.4918052	0.6529589

LC_IGP150	1802	81.52553	82.63029	50.58012	95.38326	7.044042	8.746899
LC_IGP151	1802	77.8777	78.10981	58.13974	87.35584	3.874542	5.202096
LC_IGP152	1802	78.83719	79.07654	54.97019	88.73389	3.946735	5.136627
LC_IGP153	1802	78.11918	78.51407	57.09651	87.65547	4.433259	5.75506
LC_IGP154	1802	70.79372	71.56196	43.52134	85.53326	6.944947	9.102306
LC_IGP155	1802	14.8637	14.61982	8.157254	28.93589	2.688678	3.331153
LC_IGP156	1802	17.36267	17.08704	9.486313	33.62837	3.178242	4.117785
LC_IGP157	1802	12.72096	12.4564	5.887799	27.0873	2.607545	3.371882
LC_IGP158	1802	10.73181	10.49472	3.616502	22.72902	2.286435	2.960477
LC_IGP159	1802	16.69278	16.42625	9.082308	31.45561	2.977229	3.808239
LC_IGP16	1801	0.352628	0.3222465	0.04687525	1.185569	0.1605356	0.1926366
LC_IGP160	1802	18.20841	17.91657	9.744608	35.23297	3.355646	4.332292
LC_IGP161	1802	15.34947	15.09906	8.039538	30.22097	3.006157	3.980158
LC_IGP162	1802	14.8763	14.55665	7.887493	34.04517	3.131356	4.062968
LC_IGP163	1802	1.829088	1.640786	0.5027156	7.479133	0.83686	1.006609
LC_IGP164	1802	0.8457389	0.710763	0.1449509	5.207134	0.5251388	0.5334997
LC_IGP165	1802	2.628509	2.322644	0.5285596	10.76378	1.367895	1.630344
LC_IGP166	1802	4.144489	3.787352	0.5444947	23.47642	2.052098	2.423231
LC_IGP167	1802	51.88145	47.09816	8.604583	166.0482	24.11023	31.14771
LC_IGP168	1802	0.1926336	0.1869609	0.09610792	0.4591697	0.04335743	0.0523096
LC_IGP169	1802	16.04459	15.75122	8.768107	31.46787	2.960633	3.697001
LC_IGP17	1819	0.4196517	0.3939067	0.07890266	4.306574	0.185415	0.1901489
LC_IGP170	1802	89.11646	89.9797	67.29592	96.94686	4.235503	5.330658
LC_IGP171	1802	4.84836	4.748864	1.92499	9.590959	1.069709	1.387386
LC_IGP172	1802	19.97142	17.55943	5.175953	91.84557	9.860506	11.40192
LC_IGP173	1823	32.74768	32.37846	10.43417	65.99876	7.281043	9.663401
LC_IGP174	1823	24.45186	24.57293	11.09621	34.57662	3.592904	4.920869
LC_IGP175	1823	8.009018	7.865952	2.484949	27.02503	2.475072	3.28982
LC_IGP176	1821	10.73821	10.51284	4.104545	24.18649	3.15889	4.024952
LC_IGP177	1821	7.515645	7.313734	2.712131	17.94105	1.75222	2.231001
LC_IGP178	1821	1.38422	1.266818	0.08152761	9.607612	0.6931039	0.7155199
LC_IGP179	1822	4.680254	4.62953	1.431467	11.79832	0.910799	1.06508
LC_IGP18	1819	0.6124824	0.5376028	0.09833472	6.82786	0.3640072	0.3854131

LC_IGP180	1821	7.807839	7.527445	1.610141	29.08257	2.758056	3.415011
LC_IGP181	1820	1.447891	1.101077	0.2521604	14.81673	1.218872	0.8725862
LC_IGP182	1818	0.6274652	0.5158017	0.0912688	5.429235	0.4508829	0.3715013
LC_IGP183	1816	21.68875	21.30927	9.651767	48.6057	4.83984	6.205965
LC_IGP184	1816	36.85422	37.00702	17.26415	64.48206	7.033344	9.354336
LC_IGP185	1817	14.55065	14.14773	5.525432	39.45251	3.953405	4.552135
LC_IGP186	1816	19.59638	19.3804	9.141347	36.71485	2.698643	3.085286
LC_IGP187	1821	27.57163	27.36658	6.830465	55.55129	4.153676	5.074876
LC_IGP188	1821	16.11136	15.74526	4.439301	41.395	4.260839	5.191807
LC_IGP189	1822	16.167	15.83286	4.087561	47.72177	2.967927	3.132441
LC_IGP19	1815	0.1282273	0.1160663	0.01317227	0.6555213	0.0685027	0.08313218
LC_IGP190	1821	49.0857	49.09759	15.20751	84.53836	5.118481	6.148244
LC_IGP191	1816	17.71904	16.06528	5.180759	56.24942	7.134828	8.3371
LC_IGP192	1816	9.144912	7.899229	1.400523	43.91892	4.887219	4.822811
LC_IGP193	1820	15.043	13.26373	3.329862	63.01371	7.238936	7.814839
LC_IGP194	1817	30.72781	29.0725	9.356945	79.18261	10.51018	11.97243
LC_IGP195	1817	0.1709155	0.1381969	0.03981943	1.883369	0.1325282	0.09767087
LC_IGP196	1817	0.138052	0.1214174	0.03829457	0.6531834	0.07256945	0.07423472
LC_IGP197	1819	38.35745	38.04234	15.03399	62.25282	7.091648	9.378611
LC_IGP198	1819	28.92381	29.17101	13.88253	40.49717	4.556044	6.504231
LC_IGP199	1819	9.537875	9.285431	2.700304	22.01871	3.200605	4.211098
LC_IGP2	1821	28.01488	28.13439	16.81636	46.09392	3.03607	3.989746
LC_IGP20	1800	0.388559	0.3557898	0.04734061	2.402219	0.2038894	0.2198002
LC_IGP200	1819	12.60947	12.34734	5.295393	26.95745	3.488882	4.508295
LC_IGP201	1819	8.916615	8.665802	3.124454	24.17501	2.305916	2.851871
LC_IGP202	1819	1.654776	1.48828	0.08910909	13.84305	0.90806	0.8964956
LC_IGP203	1819	50.96692	50.56954	27.13645	77.34025	8.473546	11.62573
LC_IGP204	1819	37.84043	38.45479	19.00696	51.23479	5.222075	7.433248
LC_IGP205	1819	11.19265	10.9522	3.101198	31.49993	3.775412	4.875722
LC_IGP21	1801	89.09374	89.7892	69.86524	98.09314	3.996363	5.234975
LC_IGP22	1778	19.37849	19.18438	7.446865	33.6609	3.230237	4.141048
LC_IGP23	1773	42.40843	42.27424	18.29496	66.92002	6.929697	8.897463
LC_IGP24	1785	9.717588	9.483912	3.225323	21.76406	2.367805	3.003742

LC_IGP25	1773	11.34763	11.26566	5.511239	17.55733	1.317925	1.647691
LC_IGP26	1816	18.23678	17.72652	6.311742	46.32184	4.403905	5.550083
LC_IGP27	1816	13.35349	12.81568	3.771755	37.12295	4.163739	5.350675
LC_IGP28	1816	13.20112	12.19809	3.894537	48.15045	5.233219	6.405665
LC_IGP29	1818	25.7336	25.73506	7.301308	54.488	4.914252	5.737441
LC_IGP3	1821	10.91941	10.69171	3.333924	22.98971	3.085313	4.029335
LC_IGP30	1783	30.08451	28.40305	7.394658	76.1044	10.36495	13.88649
LC_IGP31	1782	21.63312	20.29397	5.144579	65.78325	8.512886	11.14955
LC_IGP32	1802	13.7767	11.80087	1.35598	68.89369	8.088954	9.283405
LC_IGP33	1788	51.70545	51.26885	12.39563	90.3653	11.59023	15.57552
LC_IGP34	1817	15.3349	15.14884	1.783014	27.88957	2.526315	2.968012
LC_IGP35	1817	9.910784	9.620188	1.328652	23.98698	2.664227	3.265407
LC_IGP36	1817	5.974926	5.915627	1.092293	11.61999	0.9409355	1.125637
LC_IGP37	1820	32.74865	32.65076	3.82728	51.96505	3.548737	4.236988
LC_IGP38	1803	10.1889	9.884551	3.684672	28.21628	2.697555	3.191349
LC_IGP39	1803	6.244363	6.046885	1.746085	18.06487	1.851358	2.31169
LC_IGP4	1822	6.627905	6.377749	1.762951	27.88939	2.091212	2.397652
LC_IGP40	1805	6.982774	6.677944	1.69048	25.99884	2.175879	2.486051
LC_IGP41	1816	26.97072	26.61814	8.578205	63.68147	6.520935	8.557238
LC_IGP42	1794	0.5549514	0.4951602	0.08337665	3.203928	0.2894666	0.2684371
LC_IGP43	1803	0.1536795	0.1417889	0.03958274	1.540659	0.06705188	0.06159712
LC_IGP44	1794	0.3395829	0.3311754	0.07695999	0.7621272	0.09792681	0.1184118
LC_IGP45	1803	0.1308805	0.1241814	0.03807561	0.6064014	0.04156379	0.04688424
LC_IGP46	1794	29.02399	28.76689	10.46012	59.72213	6.561429	8.615498
LC_IGP47	1794	31.19298	31.38622	17.73656	41.99733	3.578829	4.935857
LC_IGP48	1794	12.20006	11.88135	3.516359	27.41726	3.736723	4.821761
LC_IGP49	1794	7.323871	7.104368	2.261723	18.59196	2.066686	2.532024
LC_IGP5	1820	8.00637	7.921323	2.480622	13.72795	1.506194	2.020167
LC_IGP50	1794	8.928519	8.837491	3.1177	15.75691	1.722156	2.342879
LC_IGP51	1794	1.34911	1.258279	0.3415862	3.887475	0.4713155	0.5911068
LC_IGP52	1794	2.46551	2.176815	0.1177397	10.35644	1.264026	1.53205
LC_IGP53	1794	3.333201	3.046677	0.8063794	9.974104	1.425569	1.829331
LC_IGP54	1794	1.934773	1.727408	0.3134373	6.964589	0.9394116	1.170271

LC_IGP55	1794	0.8246583	0.7314904	0.04998579	3.048872	0.4303114	0.5236261
LC_IGP56	1794	1.030485	0.9445127	0.08895682	3.582566	0.5415972	0.7239539
LC_IGP57	1794	0.3928493	0.3545755	0.04997208	1.376642	0.1825713	0.2179701
LC_IGP58	1794	39.63803	39.32778	16.10415	72.13204	8.075991	10.83962
LC_IGP59	1794	44.48518	45.10208	23.53208	54.94067	3.951721	5.03579
LC_IGP6	1818	1.211301	1.139068	0.3176272	8.3887	0.4320827	0.4990316
LC_IGP60	1794	15.87679	15.4047	4.335875	33.94613	4.732587	5.931041
LC_IGP61	1794	90.01852	90.70247	68.08925	97.32304	4.034825	5.303393
LC_IGP62	1794	91.52546	92.32704	63.97661	99.12492	4.08656	5.155533
LC_IGP63	1794	90.21638	90.95039	71.52374	97.53442	4.050458	5.297565
LC_IGP64	1794	85.38992	85.97667	67.82488	95.55306	4.613512	6.299324
LC_IGP65	1794	72.41702	72.76015	51.96477	86.14878	4.867288	6.646561
LC_IGP66	1794	73.06649	73.60071	48.79758	87.84586	5.124296	6.944317
LC_IGP67	1794	70.13429	70.34896	49.98645	84.03167	5.222591	7.215481
LC_IGP68	1794	76.79593	77.32613	58.95019	90.20581	4.965745	6.715875
LC_IGP69	1794	17.6015	17.3501	5.838141	32.36594	3.099683	4.078623
LC_IGP7	1817	1.77285	1.754707	0.5090409	3.907978	0.3000904	0.3702356
LC_IGP70	1794	18.45897	18.26598	5.870323	33.58663	3.364489	4.342122
LC_IGP71	1794	20.08209	19.83037	6.374685	36.08761	3.505328	4.774933
LC_IGP72	1794	8.593991	8.374252	3.650932	18.35911	1.927915	2.461257
LC_IGP73	1794	19.84949	19.66258	7.038298	35.06771	3.39062	4.405761
LC_IGP74	1794	20.59741	20.43136	7.140385	36.7301	3.640868	4.795538
LC_IGP75	1794	22.39065	22.25817	7.311009	39.31841	3.876297	5.19329
LC_IGP76	1794	11.13701	10.85653	5.407402	21.1584	2.431526	3.130005
LC_IGP77	1794	2.247993	2.06213	0.3171711	7.249977	1.029538	1.354979
LC_IGP78	1794	2.138439	1.933728	0.1003054	8.236043	1.109633	1.413637
LC_IGP79	1794	2.308557	2.096591	0.176165	8.325272	1.177201	1.591397
LC_IGP8	1822	5.395025	5.142217	0.4424564	25.5117	1.916849	2.142732
LC_IGP80	1794	2.543022	2.365393	0.3526343	8.150201	1.071975	1.28443
LC_IGP81	1794	40.77456	35.14051	7.167577	271.6161	23.39655	26.37524
LC_IGP82	1794	0.2458405	0.2402034	0.07061816	0.5469337	0.05510289	0.07053886
LC_IGP83	1794	19.57982	19.36807	6.596018	35.35599	3.471033	4.568478
LC_IGP84	1794	88.73024	89.39383	69.05462	97.59712	4.62418	5.958937

LC_IGP85	1794	3.791516	3.687153	1.683056	11.74601	0.8890938	1.103779
LC_IGP86	1794	25.49213	22.75702	3.269077	95.91182	12.98938	16.13143
LC_IGP87	1822	36.42736	36.3212	6.696858	70.75938	7.275505	9.566869
LC_IGP88	1822	26.14259	26.4369	11.10415	51.81905	3.482087	4.517872
LC_IGP89	1822	6.736365	6.441443	2.137771	23.95032	2.167443	2.642822
LC_IGP9	1805	0.5911861	0.5674166	0.1649091	1.836016	0.1796326	0.2205152
LC_IGP90	1822	7.948146	7.733141	3.782501	22.99041	1.870048	2.33019
LC_IGP91	1820	4.257154	4.191706	1.698846	11.50247	1.01912	1.34321
LC_IGP92	1821	1.010588	0.949894	0.2395422	4.659095	0.3516576	0.4278575
LC_IGP93	1821	3.64534	3.620199	1.086301	9.812544	0.7326636	0.9001733
LC_IGP94	1821	4.506527	4.252315	1.16083	12.83186	1.505078	1.778613
LC_IGP95	1817	0.4014299	0.3786477	0.1067128	1.824388	0.1513255	0.1756672
LC_IGP96	1818	0.3559607	0.3364332	0.07100582	2.091303	0.145546	0.1751512
LC_IGP97	1817	1.332224	1.199485	0.2318754	5.931395	0.6127472	0.7039243
LC_IGP98	1817	2.191397	1.996346	0.4473482	17.68469	1.04282	1.062615
LC_IGP99	1819	1.351174	1.18669	0.3492373	9.248627	0.6890741	0.6874547
LC_IGPRG1	1796	0.2853332	0.2850905	0.1937887	0.409909	0.02494064	0.03187333
LC_IGPRG10	1796	0.3411757	0.3426433	0.04940547	0.6435223	0.09131882	0.122369
LC_IGPRG11	1796	0.3914428	0.3900273	0.2701847	0.5652335	0.03184312	0.04010271
LC_IGPRG12	1796	0.3377863	0.3373934	0.1879704	0.418417	0.02275582	0.02705259
LC_IGPRG13	1796	0.265384	0.2649168	0.1074397	0.4211442	0.03483979	0.04492243
LC_IGPRG14	1796	0.3263745	0.3248529	0.1837796	0.4996946	0.03824048	0.04902116
LC_IGPRG15	1796	0.2189938	0.2184364	0.1072855	0.3613589	0.02730523	0.03503022
LC_IGPRG16	1796	0.2915936	0.2874908	0.07094635	0.5175571	0.05460424	0.06774895
LC_IGPRG17	1796	0.3726596	0.3733315	0.1609521	0.5379878	0.04133225	0.05215946
LC_IGPRG18	1796	0.2640263	0.2600308	0.1176308	0.6247643	0.04719293	0.05840151
LC_IGPRG19	1796	0.1881015	0.1840956	0.01616798	0.5995806	0.0659483	0.08331571
LC_IGPRG2	1796	0.3721104	0.3708933	0.2872251	0.4651527	0.01846392	0.02174552
LC_IGPRG20	1796	0.2674419	0.2621161	0.03857159	0.6806442	0.08104911	0.102245
LC_IGPRG21	1796	0.321262	0.3227376	0.1352901	0.4337535	0.03498673	0.0417707
LC_IGPRG22	1796	0.2883482	0.290081	0.1656884	0.401972	0.02383352	0.02889874
LC_IGPRG23	1796	0.2891219	0.2881361	0.1275872	0.5193009	0.04258216	0.05501249
LC_IGPRG24	1796	0.3970888	0.3928089	0.1711941	0.6417874	0.05256229	0.06514746

LC_IGPRG25	1796	0.3528449	0.3514619	0.1961934	0.5698103	0.03655903	0.04342194
LC_IGPRG26	1796	0.3480969	0.3462664	0.0472109	0.8522476	0.07523792	0.09193018
LC_IGPRG27	1796	0.4370728	0.4347396	0.2733693	0.6864176	0.04253167	0.04735573
LC_IGPRG28	1796	0.4126548	0.4114747	0.1993613	0.7027547	0.05762378	0.07265042
LC_IGPRG29	1796	0.5194636	0.5087059	0.1911392	0.9894632	0.1282855	0.1731765
LC_IGPRG3	1796	0.4437619	0.4424877	0.2557467	0.6020345	0.03765192	0.04934799
LC_IGPRG30	1796	0.3888944	0.3727942	0.1222035	0.9244965	0.1167794	0.1479189
LC_IGPRG31	1796	3.298173	3.30145	2.250607	4.174687	0.2377866	0.3138324
LC_IGPRG32	1796	2.923804	2.926977	1.904719	4.025655	0.2257473	0.2846566
LC_IGPRG33	1796	3.754848	3.724465	2.67832	5.871909	0.3267545	0.3976409
LC_IGPRG4	1796	0.2741027	0.2763483	0.1116259	0.4238523	0.03604061	0.04566555
LC_IGPRG5	1796	0.4259669	0.4254499	0.2261537	0.5701311	0.03230675	0.03946014
LC_IGPRG6	1796	0.3581742	0.3562162	0.1165781	0.6021773	0.05661348	0.07079552
LC_IGPRG7	1796	0.187602	0.1869661	0.066821	0.355081	0.02360975	0.02673199
LC_IGPRG8	1796	0.3208403	0.3199083	0.07562953	0.4865309	0.04306575	0.05411881
LC_IGPRG9	1796	0.2891052	0.293806	0.03050367	0.6094451	0.08993987	0.1213769
LC_IGPSC_tmp2[illegible]	1801	29.28504	29.01882	10.29296	58.79804	6.77837	8.875527
LC_IGPSC_tmp2[illegible]	1801	31.4364	31.65563	17.58363	44.43716	3.059456	4.01634
LC_IGPSC_tmp2[illegible]	1801	12.28509	11.99423	3.48604	24.74428	3.538352	4.646583
LC_IGPSC_tmp2[illegible]	1801	7.405476	7.167134	1.891073	19.4757	2.152889	2.732592
LC_IGPSC_tmp2[illegible]	1801	9.018415	8.95038	2.6609	15.02484	1.733842	2.319186
LC_IGPSC_tmp2[illegible]	1801	1.361796	1.280558	0.3363008	3.816545	0.4643021	0.5801505
LC_IGPSC_tmp2[illegible]	1801	1.989721	1.964032	0.8467838	4.234709	0.3229826	0.3970714
LC_IGPSC_tmp2[illegible]	1801	6.051383	5.770135	0.8046638	16.89659	2.08291	2.482986
LC_IGPSC_tmp2[illegible]	1801	0.6650677	0.6368977	0.1784313	2.087308	0.2054101	0.2588195
LC_IGPSC_tmp3[illegible]	1801	0.5016151	0.4765459	0.07562745	2.638114	0.2015169	0.2601743
LC_IGPSC_tmp3[illegible]	1817	39.80377	39.9149	17.60064	70.25225	7.392825	9.551527
LC_IGPSC_tmp3[illegible]	1817	28.59816	28.9129	11.95876	37.81399	3.662019	4.830779
LC_IGPSC_tmp3[illegible]	1817	7.366302	7.046532	2.216281	16.93865	2.349746	2.918507
LC_IGPSC_tmp3[illegible]	1817	8.699304	8.468536	4.163845	23.79001	2.032391	2.507765
LC_IGPSC_tmp3[illegible]	1817	4.660194	4.567723	1.68667	9.675016	1.124183	1.457764
LC_IGPSC_tmp3[illegible]	1817	1.105403	1.039281	0.259844	2.879618	0.3809043	0.4855947
LC_IGPSC_tmp3[illegible]	1817	3.988553	3.978556	1.543168	7.839812	0.7956425	0.9627653

LC_IGPSC_tmp3[illegible]	1817	4.94582	4.665881	1.342131	15.54907	1.690334	1.999971
LC_IGPSC_tmp3[illegible]	1817	0.4413738	0.4129037	0.1095023	2.210712	0.1756001	0.1950571
LC_IGPSC_tmp4[illegible]	1817	0.3911139	0.3674612	0.07419844	2.534148	0.1667598	0.1943274
LC_IGPSC_tmp4[illegible]	1816	32.95745	32.61667	10.53406	58.14753	7.263467	9.740726
LC_IGPSC_tmp4[illegible]	1816	24.6125	24.75352	12.8774	34.86603	3.581812	4.958181
LC_IGPSC_tmp4[illegible]	1816	8.048882	7.908036	2.504795	16.40191	2.431997	3.276707
LC_IGPSC_tmp4[illegible]	1816	10.80492	10.57862	4.262385	23.82275	3.167647	4.108467
LC_IGPSC_tmp4[illegible]	1816	7.554195	7.356497	2.834014	16.91045	1.746686	2.232321
LC_IGPSC_tmp4[illegible]	1816	1.383512	1.273242	0.08047908	9.699589	0.6722884	0.7202446
LC_IGPSC_tmp4[illegible]	1816	4.704854	4.655306	1.462487	11.91127	0.9048199	1.046909
LC_IGPSC_tmp4[illegible]	1816	7.848008	7.591268	1.595213	28.41894	2.741573	3.396468
LC_IGPSC_tmp4[illegible]	1816	1.455393	1.111679	0.2468139	14.60713	1.234949	0.8829475
LC_IGPSC_tmp5[illegible]	1816	0.6302883	0.5197176	0.09249681	5.111282	0.4495626	0.3720235
