# Supplementary material for: Genome-Wide Association Study on Immunoglobulin G Glycosylation Patterns
Source: Front Immunol. 2018 Feb 26;9:277. doi: 10.3389/fimmu.2018.00277 (PMC5834439; doi:10.3389/fimmu.2018.00277)
Supplement: Table S3 — Explained variance of the immunoglobulin G (IgG) glycopeptide traits by the single SNPs in KORA. [file Table_3.PDF]

Explained Variances in Disocvery Cohort KORA

GWAS Code	Trait	Chromosome	LD-Block per Chromosome	n(KORA)	adjusted R^2 (KORA)	associated SNP (KORA)
LC_IGP145	IgG2 G1n	1	1	1624	0.018	rs16830188
LC_IGP144	IgG2 G0n	1	1	1624	0.020	rs16830188
LC_IGP_R62	IgG2_G1F/IgG2_G0F	1	1	1639	0.020	rs16830188
LC_IGP175	IgG4_G2F	1	1	1641	0.023	rs16830188
LC_IGP199	IgG4_G2Fn	1	1	1637	0.023	rs16830188
LC_IGP89	IgG2_G2F	1	1	1640	0.023	rs16830188
LC_IGP88	IgG2_G1F	1	1	1640	0.026	rs16830188
LC_IGP133	IgG2_G1Fn	1	1	1624	0.029	rs16830188
LC_IGP135	IgG2_G0FNn	1	1	1624	0.032	rs16830188
LC_IGP90	IgG2_G0FN	1	1	1640	0.035	rs16830188
LC_IGP_R92	IgG4_G1FS1/IgG4_G1F	3	1	1640	0.019	rs13082825
LC_IGP189	IgG4 FG1S1/(FG1+FG1S1)	3	1	1640	0.019	rs13082825
LC_IGP186	IgG4 SA per Gal	3	1	1634	0.020	rs13082825
LC_IGP7	IgG1_G1FS1	3	1	1635	0.022	rs35832957
LC_IGP25	IgG1 SA per Gal	3	1	1596	0.027	rs11923417
LC_IGP_R93	IgG4_G2FS1/IgG4_G2F	3	1	1639	0.028	rs13082825
LC_IGP190	IgG4 FG2S1/(FG2+FG2S1)	3	1	1639	0.028	rs13082825
LC_IGP_R34	IgG1_G1FS1/IgG1_G1F	3	1	1635	0.029	rs35832957
LC_IGP36	IgG1 FG1S1/(FG1+FG1S1)	3	1	1635	0.030	rs35832957
LC_IGP_R75	IgG2_G2FS1/IgG2_G2F	3	1	1639	0.031	rs13082825
LC_IGP123	IgG2 FG2S1/(FG2+FG2S1)	3	1	1639	0.031	rs13082825
LC_IGP93	IgG2_G1FS1	3	1	1639	0.036	rs13082825
LC_IGP111	IgG2 SA per Gal	3	1	1615	0.038	rs11923417
LC_IGP120	IgG2 FGS1/(FG+FGS1)	3	1	1638	0.038	rs11923417
LC_IGP_R35	IgG1_G2FS1/IgG1_G2F	3	1	1638	0.048	rs11923417
LC_IGP37	IgG1 FG2S1/(FG2+FG2S1)	3	1	1638	0.048	rs11923417
LC_IGP122	IgG2 FG1S1/(FG1+FG1S1)	3	1	1639	0.053	rs13082825
LC_IGP_R74	IgG2_G1FS1/IgG2_G1F	3	1	1639	0.053	rs13082825
LC_IGP_R93	IgG4_G2FS1/IgG4_G2F	3	2	1639	0.019	rs4012171
LC_IGP190	IgG4 FG2S1/(FG2+FG2S1)	3	2	1639	0.019	rs4012171
LC_IGP93	IgG2_G1FS1	3	2	1639	0.024	rs4012171
LC_IGP25	IgG1 SA per Gal	3	2	1596	0.025	rs4012171
LC_IGP36	IgG1 FG1S1/(FG1+FG1S1)	3	2	1635	0.025	rs4012171
LC_IGP_R34	IgG1_G1FS1/IgG1_G1F	3	2	1635	0.025	rs4012171
LC_IGP120	IgG2 FGS1/(FG+FGS1)	3	2	1638	0.027	rs4012171
LC_IGP_R75	IgG2_G2FS1/IgG2_G2F	3	2	1639	0.027	rs4012171
LC_IGP123	IgG2 FG2S1/(FG2+FG2S1)	3	2	1639	0.027	rs4012171
LC_IGP111	IgG2 SA per Gal	3	2	1615	0.036	rs4012171
LC_IGP_R35	IgG1_G2FS1/IgG1_G2F	3	2	1638	0.037	rs4012171
LC_IGP37	IgG1 FG2S1/(FG2+FG2S1)	3	2	1638	0.037	rs4012171
LC_IGP122	IgG2 FG1S1/(FG1+FG1S1)	3	2	1639	0.044	rs4012171
LC_IGP_R74	IgG2_G1FS1/IgG2_G1F	3	2	1639	0.044	rs4012171
LC_IGP35	IgG1 FGS1/(F+FG+FGS1)	3	3	1635	0.018	rs6764279
LC_IGP_R33	IgG1_G2S1/IgG1_G2	3	3	1636	0.022	rs62292620
LC_IGP29	IgG1 G2S1/(G2+G2S1)	3	3	1636	0.022	rs62292620
LC_IGP110	IgG2 Sialylation	3	3	1619	0.030	rs6788832
LC_IGP121	IgG2 FGS1/(F+FG+FGS1)	3	3	1637	0.030	rs6764279
LC_IGP179	IgG4_G1FS1	3	3	1640	0.048	rs6764279
LC_IGP34	IgG1 FGS1/(FG+FGS1)	3	3	1635	0.051	rs11710456
LC_IGP187	IgG4 FGS1/(FG+FGS1)	3	3	1639	0.057	rs7621161
LC_IGP7	IgG1_G1FS1	3	3	1635	0.059	rs11710456
LC_IGP25	IgG1 SA per Gal	3	3	1596	0.069	rs7621161
LC_IGP_R75	IgG2_G2FS1/IgG2_G2F	3	3	1639	0.077	rs7621161

>= 10 %
>= 5 %

LC_IGP123	IgG2 FG2S1/(FG2+FG2S1)	3	3	1639	0.077	rs7621161
LC_IGP186	IgG4 SA per Gal	3	3	1634	0.078	rs11710456
LC_IGP_R34	IgG1_G1FS1/IgG1_G1F	3	3	1635	0.080	rs7621161
LC_IGP36	IgG1 FG1S1/(FG1+FG1S1)	3	3	1635	0.080	rs7621161
LC_IGP93	IgG2_G1FS1	3	3	1639	0.080	rs6764279
LC_IGP_R92	IgG4_G1FS1/IgG4_G1F	3	3	1640	0.081	rs7621161
LC_IGP189	IgG4 FG1S1/(FG1+FG1S1)	3	3	1640	0.081	rs7621161
LC_IGP_R93	IgG4_G2FS1/IgG4_G2F	3	3	1639	0.083	rs7621161
LC_IGP190	IgG4 FG2S1/(FG2+FG2S1)	3	3	1639	0.083	rs7621161
LC_IGP120	IgG2 FGS1/(FG+FGS1)	3	3	1638	0.091	rs11710456
LC_IGP111	IgG2 SA per Gal	3	3	1615	0.106	rs11710456
LC_IGP_R35	IgG1_G2FS1/IgG1_G2F	3	3	1638	0.113	rs7621161
LC_IGP37	IgG1 FG2S1/(FG2+FG2S1)	3	3	1638	0.113	rs7621161
LC_IGP122	IgG2 FG1S1/(FG1+FG1S1)	3	3	1639	0.141	rs11710456
LC_IGP_R74	IgG2_G1FS1/IgG2_G1F	3	3	1639	0.141	rs11710456
LC_IGP25	IgG1 SA per Gal	3	4	1596	0.019	rs57679165
LC_IGP_R92	IgG4_G1FS1/IgG4_G1F	3	4	1640	0.021	rs57679165
LC_IGP189	IgG4 FG1S1/(FG1+FG1S1)	3	4	1640	0.021	rs57679165
LC_IGP_R34	IgG1_G1FS1/IgG1_G1F	3	4	1635	0.023	rs57679165
LC_IGP36	IgG1 FG1S1/(FG1+FG1S1)	3	4	1635	0.023	rs57679165
LC_IGP186	IgG4 SA per Gal	3	4	1634	0.023	rs57679165
LC_IGP93	IgG2_G1FS1	3	4	1639	0.023	rs57679165
LC_IGP_R75	IgG2_G2FS1/IgG2_G2F	3	4	1639	0.027	rs57679165
LC_IGP123	IgG2 FG2S1/(FG2+FG2S1)	3	4	1639	0.027	rs57679165
LC_IGP_R93	IgG4_G2FS1/IgG4_G2F	3	4	1639	0.029	rs57679165
LC_IGP190	IgG4 FG2S1/(FG2+FG2S1)	3	4	1639	0.029	rs57679165
LC_IGP120	IgG2 FGS1/(FG+FGS1)	3	4	1638	0.030	rs57679165
LC_IGP111	IgG2 SA per Gal	3	4	1615	0.034	rs57679165
LC_IGP_R35	IgG1_G2FS1/IgG1_G2F	3	4	1638	0.034	rs57679165
LC_IGP37	IgG1 FG2S1/(FG2+FG2S1)	3	4	1638	0.034	rs57679165
LC_IGP122	IgG2 FG1S1/(FG1+FG1S1)	3	4	1639	0.049	rs57679165
LC_IGP_R74	IgG2_G1FS1/IgG2_G1F	3	4	1639	0.049	rs57679165
LC_IGP84	IgG1 FBn/Bn total	7	1	1614	0.018	rs7804185
LC_IGP14	IgG1_G0N	7	1	1633	0.019	rs7804185
LC_IGP_R16	IgG1_G1N/IgG1_G1	7	1	1624	0.020	rs7804185
LC_IGP15	IgG1_G1N	7	1	1626	0.021	rs7804185
LC_IGP56	IgG1_G1Nn	7	1	1614	0.021	rs7804185
LC_IGP79	IgG1 BG1n/G1n	7	1	1614	0.021	rs7804185
LC_IGP55	IgG1_G0Nn	7	1	1614	0.023	rs7804185
LC_IGP78	IgG1 BG0n/G0n	7	1	1614	0.023	rs7804185
LC_IGP86	IgG1 Bn/Fn total	7	1	1614	0.026	rs7804185
LC_IGP77	IgG1 Bn	7	1	1614	0.027	rs7804185
LC_IGP81	IgG1 Fn/Bn	7	1	1614	0.027	rs7804185
LC_IGP_R89	IgG4_G2FS1/IgG4_G1FS1	9	1	1639	0.017	rs7019909
LC_IGP175	IgG4_G2F	9	2	1641	0.015	rs1969977
LC_IGP109	IgG2 Galactosylation	9	2	1615	0.017	rs12337458
LC_IGP88	IgG2_G1F	9	2	1640	0.017	rs113197944
LC_IGP_R68	IgG2_G2F/IgG2_G1F	9	2	1639	0.017	chr9:33152337:D
LC_IGP134	IgG2_G2Fn	9	2	1624	0.018	rs12337458
LC_IGP89	IgG2_G2F	9	2	1640	0.018	rs12337458
LC_IGP144	IgG2 G0n	9	2	1624	0.018	rs3824459
LC_IGP_R28	IgG1_G2F/IgG1_G1F	9	2	1638	0.018	rs10758195
LC_IGP_R62	IgG2_G1F/IgG2_G0F	9	2	1639	0.018	rs3824459
LC_IGP_R20	IgG1_G1/IgG1_G0	9	2	1636	0.019	rs3758273
LC_IGP_R29	IgG1_G2FS1/IgG1_G1FS1	9	2	1635	0.019	chr9:33152337:D
LC_IGP199	IgG4_G2Fn	9	2	1637	0.019	rs10971434

LC_IGP133	IgG2_G1Fn	9	2	1624	0.019	rs113197944
LC_IGP_R69	IgG2_G2FS1/IgG2_G1FS1	9	2	1638	0.020	rs12342831
LC_IGP145	IgG2 G1n	9	2	1624	0.020	rs113197944
LC_IGP205	IgG4_G2n	9	2	1637	0.020	rs10971434
LC_IGP_R63	IgG2_G1FN/IgG2_G0FN	9	2	1638	0.020	rs3824459
LC_IGP59	IgG1 G1n	9	2	1614	0.020	rs10121006
LC_IGP187	IgG4 FGS1/(FG+FGS1)	9	2	1639	0.022	rs10738907
LC_IGP60	IgG1 G2n	9	2	1614	0.022	rs3780481
LC_IGP48	IgG1_G2Fn	9	2	1614	0.023	rs12337458
LC_IGP_R22	IgG1_G1F/IgG1_G0F	9	2	1639	0.023	rs12337458
LC_IGP3	IgG1_G2F	9	2	1639	0.024	rs12337458
LC_IGP23	IgG1 Galactosylation	9	2	1596	0.025	rs3780481
LC_IGP180	IgG4_G2FS1	9	2	1639	0.025	rs1969977
LC_IGP4	IgG1_G0FN	9	2	1640	0.026	rs3780481
LC_IGP58	IgG1 G0n	9	2	1614	0.027	rs12337458
LC_IGP49	IgG1_G0FNn	9	2	1614	0.027	rs3758273
LC_IGP_R23	IgG1_G1FN/IgG1_G0FN	9	2	1637	0.031	rs12337458
LC_IGP_R88	IgG4_G2F/IgG4_G1F	9	2	1641	0.035	chr9:33152337:D
LC_IGP_R89	IgG4_G2FS1/IgG4_G1FS1	9	2	1639	0.043	rs12342831
LC_IGP_SC13	IgG1_G2(20)/IgG2_G2(20)	14	1	1635	0.018	rs10138570
LC_IGP_R41	IgG2_G0F/IgG2_G0	14	1	1636	0.019	rs59853675
LC_IGP31	IgG1 BGS1/(BG0+BG+BGS1)	14	1	1604	0.019	rs883081
LC_IGP47	IgG1_G1Fn	14	1	1614	0.019	rs11158592
LC_IGP32	IgG1 BG1S1/(BG1+BG1S1)	14	1	1622	0.019	rs11628196
LC_IGP_R36	IgG1_G1NS1/IgG1_G1N	14	1	1622	0.019	rs11628196
LC_IGP54	IgG1_G2n	14	1	1614	0.019	rs1760978
LC_IGP26	IgG1 GS1/(G+GS1)	14	1	1635	0.020	rs11158592
LC_IGP148	IgG2 FG0n total/G0n	14	1	1624	0.021	rs12586842
LC_IGP_SC15	IgG1_G1N(20)/IgG2_G1N(20)	14	1	1624	0.021	rs10138570
LC_IGP13	IgG1_G2	14	1	1637	0.022	rs11847263
LC_IGP_R64	IgG2_G2/IgG2_G1	14	1	1634	0.022	rs11621680
LC_IGP68	IgG1 FG2n/G2n	14	1	1614	0.023	rs7152441
LC_IGP65	IgG1 Fn	14	1	1614	0.025	rs11158592
LC_IGP27	IgG1 GS1/(G0+G+GS1)	14	1	1635	0.025	rs11158592
LC_IGP97	IgG2_G0	14	1	1636	0.026	rs11158592
LC_IGP_R8	IgG1_G2FS1/IgG1_G2S1	14	1	1637	0.026	rs11158592
LC_IGP138	IgG2_G0n	14	1	1624	0.027	rs4080329
LC_IGP_R32	IgG1_G1S1/IgG1_G1	14	1	1635	0.028	rs10138570
LC_IGP15	IgG1_G1N	14	1	1626	0.029	rs11158592
LC_IGP_R26	IgG1_G2N/IgG1_G1N	14	1	1620	0.029	rs883081
LC_IGP28	IgG1 G1S1/(G1+G1S1)	14	1	1635	0.029	rs10138570
LC_IGP56	IgG1_G1Nn	14	1	1614	0.030	rs11158592
LC_IGP77	IgG1 Bn	14	1	1614	0.034	rs11158592
LC_IGP79	IgG1 BG1n/G1n	14	1	1614	0.034	rs11158592
LC_IGP81	IgG1 Fn/Bn	14	1	1614	0.035	rs11158592
LC_IGP78	IgG1 BG0n/G0n	14	1	1614	0.036	rs883081
LC_IGP86	IgG1 Bn/Fn total	14	1	1614	0.036	rs11158592
LC_IGP_R4	IgG1_G0FN/IgG1_G0N	14	1	1633	0.036	rs883081
LC_IGP_R5	IgG1_G1FN/IgG1_G1N	14	1	1626	0.037	rs11158592
LC_IGP64	IgG1 FG2n total/G2n	14	1	1614	0.038	rs11158593
LC_IGP84	IgG1 FBn/Bn total	14	1	1614	0.040	rs11158592
LC_IGP21	IgG1 Fucosylation	14	1	1622	0.042	rs11158593
LC_IGP_R3	IgG1_G2F/IgG1_G2	14	1	1636	0.042	rs11158593
LC_IGP14	IgG1_G0N	14	1	1633	0.042	rs883082
LC_IGP53	IgG1_G1n	14	1	1614	0.042	rs11158592
LC_IGP55	IgG1_G0Nn	14	1	1614	0.043	rs883082

LC_IGP12	IgG1_G1	14	1	1637	0.046	rs11158592
LC_IGP61	IgG1 Fn total	14	1	1614	0.048	rs11158592
LC_IGP63	IgG1 FG1n total/G1n	14	1	1614	0.048	rs11158592
LC_IGP_R2	IgG1_G1F/IgG1_G1	14	1	1637	0.050	rs11158592
LC_IGP62	IgG1 FG0n total/G0n	14	1	1614	0.052	rs11158592
LC_IGP_R1	IgG1_G0F/IgG1_G0	14	1	1637	0.052	rs11158592
LC_IGP52	IgG1_G0n	14	1	1614	0.059	rs11158592
LC_IGP11	IgG1_G0	14	1	1637	0.060	rs11158592
LC_IGP14	IgG1_G0N	14	2	1633	0.020	rs4899183
LC_IGP91	IgG2_G1FN	22	1	1638	0.018	rs34556611
LC_IGP_R12	IgG1_G1FN/IgG1_G1F	22	1	1638	0.018	rs12167679
LC_IGP72	IgG1 FBG2n/G2n	22	1	1614	0.019	rs34556611
LC_IGP160	IgG2 BG0n total/G0n	22	1	1624	0.019	rs17548631
LC_IGP_R51	IgG2_G0FN/IgG2_G0F	22	1	1639	0.020	rs17548631
LC_IGP158	IgG2 FBG2n/G2n	22	1	1624	0.020	rs17548631
LC_IGP_R11	IgG1_G0FN/IgG1_G0F	22	1	1639	0.020	rs9620326
LC_IGP75	IgG1 BG1n total/G1n	22	1	1614	0.020	rs12167679
LC_IGP74	IgG1 BG0n total/G0n	22	1	1614	0.020	rs12167679
LC_IGP85	IgG1 Fn/Bn total	22	1	1614	0.020	rs12167679
LC_IGP50	IgG1_G1FNn	22	1	1614	0.020	rs73396549
LC_IGP71	IgG1 FBG1n/G1n	22	1	1614	0.020	rs12167679
LC_IGP5	IgG1_G1FN	22	1	1638	0.020	rs73396549
LC_IGP162	IgG2 BG2n total/G2n	22	1	1624	0.021	rs112775985
LC_IGP171	IgG2 Fn/Bn total	22	1	1624	0.022	rs17548631
LC_IGP161	IgG2 BG1n total/G1n	22	1	1624	0.023	rs17548631
LC_IGP82	IgG1 FBn/Fn	22	1	1614	0.023	rs12167679
LC_IGP83	IgG1 FBn/Fn total	22	1	1614	0.023	rs12167679
LC_IGP70	IgG1 FBG0n/G0n	22	1	1614	0.023	rs9620326
LC_IGP156	IgG2 FBG0n/G0n	22	1	1624	0.023	rs17548631
LC_IGP73	IgG1 Bn total	22	1	1614	0.024	rs12167679
LC_IGP157	IgG2 FBG1n/G1n	22	1	1624	0.024	rs9620326
LC_IGP_R52	IgG2_G1FN/IgG2_G1F	22	1	1637	0.024	rs9620326
LC_IGP69	IgG1 FBn	22	1	1614	0.024	rs12167679
LC_IGP_R53	IgG2_G2FN/IgG2_G2F	22	1	1639	0.025	rs17548631
LC_IGP135	IgG2_G0FNn	22	1	1624	0.025	rs12167679
LC_IGP22	IgG1 Bisecting_GlcNAc	22	1	1600	0.025	rs12167679
LC_IGP159	IgG2 Bn total	22	1	1624	0.025	rs17548631
LC_IGP108	IgG2 Bisecting_GlcNAc	22	1	1613	0.026	rs17548631
LC_IGP168	IgG2 FBn/Fn	22	1	1624	0.026	rs17548631
LC_IGP169	IgG2 FBn/Fn total	22	1	1624	0.026	rs17548631
LC_IGP155	IgG2 FBn	22	1	1624	0.028	rs17548631
LC_IGP135	IgG2_G0FNn	22	2	1624	0.015	rs6519476
LC_IGP171	IgG2 Fn/Bn total	22	2	1624	0.016	rs6519476
LC_IGP156	IgG2 FBG0n/G0n	22	2	1624	0.016	rs6519476
LC_IGP161	IgG2 BG1n total/G1n	22	2	1624	0.018	rs6519476
LC_IGP168	IgG2 FBn/Fn	22	2	1624	0.018	rs6519476
LC_IGP108	IgG2 Bisecting_GlcNAc	22	2	1613	0.018	rs6519476
LC_IGP159	IgG2 Bn total	22	2	1624	0.018	rs6519476
LC_IGP169	IgG2 FBn/Fn total	22	2	1624	0.018	rs6519476
LC_IGP157	IgG2 FBG1n/G1n	22	2	1624	0.019	rs6519476
LC_IGP_R52	IgG2_G1FN/IgG2_G1F	22	2	1637	0.019	rs6519476
LC_IGP155	IgG2 FBn	22	2	1624	0.020	rs6519476
LC_IGP_R81	IgG4_G0FN/IgG4_G0F	22	3	1639	0.014	rs137680
LC_IGPRG14	IgG2_G0FN(10)/(IgG1_G0FN(10) + IgG2_G0FN(10) +IgG4_G0FN(10))	22	4	1617	0.017	rs5750830
LC_IGP202	IgG4_G2FNn	22	4	1637	0.018	rs8136980
LC_IGP_SC31	IgG2_G0F(10)/IgG4_G0F	22	4	1630	0.019	rs5750828

LC_IGP76	IgG1 BG2n total/G2n	22	4	1614	0.019	rs1005522
LC_IGP178	IgG4_G2FN	22	4	1639	0.020	rs8136980
LC_IGP_R13	IgG1_G2FN/IgG1_G2F	22	4	1636	0.020	rs1005522
LC_IGP160	IgG2 BG0n total/G0n	22	4	1624	0.021	rs5750828
LC_IGP135	IgG2_G0FNn	22	4	1624	0.022	rs1005522
LC_IGP_R51	IgG2_G0FN/IgG2_G0F	22	4	1639	0.022	rs73167342
LC_IGP173	IgG4_G0F	22	4	1641	0.022	rs73167342
LC_IGPRG26	IgG4_G2FN(10)/(IgG1_G2FN(10) + IgG2_G2FN(10) +IgG4_G2FN(10))	22	4	1617	0.023	rs909674
LC_IGPRG16	IgG2_G2FN(10)/(IgG1_G2FN(10) + IgG2_G2FN(10) +IgG4_G2FN(10))	22	4	1617	0.025	rs909674
LC_IGP156	IgG2 FBG0n/G0n	22	4	1624	0.025	rs73167342
LC_IGP66	IgG1 FG0n/G0n	22	4	1614	0.025	rs5750828
LC_IGP72	IgG1 FBG2n/G2n	22	4	1614	0.026	rs1005522
LC_IGP_R83	IgG4_G2FN/IgG4_G2F	22	4	1639	0.027	rs8136980
LC_IGPRG15	IgG2_G1FN(10)/(IgG1_G1FN(10) + IgG2_G1FN(10) +IgG4_G1FN(10))	22	4	1617	0.029	rs5750828
LC_IGP_SC36	IgG2_G2FN(10)/IgG4_G2FN	22	4	1630	0.029	rs909674
LC_IGP197	IgG4_G0Fn	22	4	1637	0.030	rs5750828
LC_IGP_SC25	IgG1_G1FN(10)/IgG4_G1FN	22	4	1618	0.031	rs5757683
LC_IGP75	IgG1 BG1n total/G1n	22	4	1614	0.031	rs5750828
LC_IGP_R12	IgG1_G1FN/IgG1_G1F	22	4	1638	0.034	rs5750828
LC_IGP50	IgG1_G1FNn	22	4	1614	0.035	rs5750828
LC_IGP4	IgG1_G0FN	22	4	1640	0.037	rs1005522
LC_IGP176	IgG4_G0FN	22	4	1639	0.038	rs5757673
LC_IGP5	IgG1_G1FN	22	4	1638	0.038	rs5750828
LC_IGP85	IgG1 Fn/Bn total	22	4	1614	0.039	rs5750828
LC_IGP200	IgG4_G0FNn	22	4	1637	0.043	rs1005522
LC_IGP49	IgG1_G0FNn	22	4	1614	0.044	rs1005522
LC_IGP22	IgG1 Bisecting_GlcNAc	22	4	1600	0.045	rs5750828
LC_IGPRG25	IgG4_G1FN(10)/(IgG1_G1FN(10) + IgG2_G1FN(10) +IgG4_G1FN(10))	22	4	1617	0.045	rs5757683
LC_IGP71	IgG1 FBG1n/G1n	22	4	1614	0.046	rs1005522
LC_IGP_SC35	IgG2_G1FN(10)/IgG4_G1FN	22	4	1630	0.048	rs9306335
LC_IGP73	IgG1 Bn total	22	4	1614	0.053	rs1005522
LC_IGP82	IgG1 FBn/Fn	22	4	1614	0.057	rs1005522
LC_IGP83	IgG1 FBn/Fn total	22	4	1614	0.057	rs1005522
LC_IGP_R82	IgG4_G1FN/IgG4_G1F	22	4	1639	0.058	rs5757681
LC_IGP183	IgG4 Bisecting_GlcNAc	22	4	1634	0.059	rs73167342
LC_IGP201	IgG4_G1FNn	22	4	1637	0.063	rs10644269
LC_IGP69	IgG1 FBn	22	4	1614	0.067	rs1005522
LC_IGP177	IgG4_G1FN	22	4	1639	0.072	rs10644269
LC_IGP74	IgG1 BG0n total/G0n	22	4	1614	0.075	rs1005522
LC_IGP_R11	IgG1_G0FN/IgG1_G0F	22	4	1639	0.078	rs5757678
LC_IGP_R81	IgG4_G0FN/IgG4_G0F	22	4	1639	0.083	rs73167342
LC_IGP70	IgG1 FBG0n/G0n	22	4	1614	0.093	rs1005522
LC_IGP201	IgG4_G1FNn	22	5	1637	0.023	rs12484278
LC_IGP_R82	IgG4_G1FN/IgG4_G1F	22	5	1639	0.024	rs12484278
LC_IGP177	IgG4_G1FN	22	5	1639	0.026	rs12484278
LC_IGP70	IgG1 FBG0n/G0n	22	5	1614	0.027	rs12484278
LC_IGP183	IgG4 Bisecting_GlcNAc	22	5	1634	0.029	rs12484278
LC_IGP_R81	IgG4_G0FN/IgG4_G0F	22	5	1639	0.032	rs12484278
LC_IGP_R81	IgG4_G0FN/IgG4_G0F	22	6	1639	0.024	rs34692520
