# Supplementary material for: Genome-Wide Association Study on Immunoglobulin G Glycosylation Patterns
Source: Front Immunol. 2018 Feb 26;9:277. doi: 10.3389/fimmu.2018.00277 (PMC5834439; doi:10.3389/fimmu.2018.00277)
Supplement: Table S4 — List of association in KORA F4 to SNPs excluded for the replication due to unavailability in Leiden Longevity Study. [file Table_4.PDF]

List of Associations That Are Suggestive in the Discovery
but Excluded from Replication Due to Missing Genotypes in the Replication Cohort

SNP	Chromosome	Position	GWAS Code	*p*	beta
rs72416175	14	66116826	LC_IGP_R1	1.63E-08	-0.253984
rs11414976	14	65781578	LC_IGP_R1	7.01E-12	-0.382776
rs34443059	14	66225456	LC_IGP_R1	1.90E-15	0.27213
rs34742130	14	65869387	LC_IGP_R1	2.47E-09	0.216552
rs201067714	14	65772934	LC_IGP_R1	2.33E-09	-0.27608
rs200274176	14	66069056	LC_IGP_R1	8.69E-14	0.261413
rs10572775	14	66055427	LC_IGP_R1	9.95E-16	0.27487
rs5809277	14	65968613	LC_IGP_R1	1.43E-09	0.218291
rs33927018	14	66029423	LC_IGP_R1	2.44E-10	0.229727
rs59312400	14	66249255	LC_IGP_R1	5.80E-14	-0.294636
rs147422602	14	65990143	LC_IGP_R1	5.50E-17	0.286149
rs59850638	14	65772935	LC_IGP_R1	3.60E-09	-0.272643
rs201678871	14	66232956	LC_IGP_R1	2.22E-13	0.250391
rs144508295	14	65811418	LC_IGP_R1	5.56E-12	0.245866
rs71126773	14	66103040	LC_IGP_R1	2.03E-16	0.280192
rs199534978	14	66076028	LC_IGP_R1	7.50E-17	0.28341
rs200183327	14	65980373	LC_IGP_R1	2.10E-10	0.230809
rs34307226	14	65844447	LC_IGP_R1	8.93E-17	-0.316525
rs34263575	14	66160754	LC_IGP_R1	1.29E-16	0.282419
rs200160352	14	66264400	LC_IGP_R1	1.43E-08	-0.214813
rs201712507	14	65856090	LC_IGP_R1	2.42E-09	-0.256732
rs34785787	14	65878211	LC_IGP_R1	1.06E-09	0.219964
rs142437037	14	65986959	LC_IGP_R1	4.31E-10	0.227508
rs145473242	14	65826211	LC_IGP_R1	1.05E-09	-0.260816
rs5809288	14	66237586	LC_IGP_R1	1.44E-13	0.253323
rs34746444	14	66195404	LC_IGP_R1	4.07E-16	0.28332
rs35402762	14	65984685	LC_IGP_R1	8.04E-10	0.22135
rs61602020	14	65774613	LC_IGP_R1	1.88E-10	-0.28827
rs3216656	14	66113327	LC_IGP_R1	8.95E-17	0.28367
rs11284050	14	65884030	LC_IGP_R1	7.23E-11	0.236731
rs35439429	14	66063919	LC_IGP_R1	1.69E-12	0.248442
rs71126788	14	66232958	LC_IGP_R1	2.22E-13	0.25039
rs72090965	14	65829951	LC_IGP_R1	5.09E-14	0.261526
rs112066572	14	65812365	LC_IGP_R1	1.35E-09	-0.258602
rs139022691	14	65778230	LC_IGP_R1	1.29E-10	0.288821
rs201643791	22	39743579	LC_IGP_R11	3.07E-11	-0.247284
rs199739333	22	39777821	LC_IGP_R11	1.49E-25	-0.376981
rs201623089	1	25297520	LC_IGP_R12	2.85E-11	0.778914
rs199739333	22	39777821	LC_IGP_R12	2.26E-12	-0.255164
rs186127900	1	25318225	LC_IGP_R12	2.02E-08	0.982031
rs150110347	1	25308762	LC_IGP_R12	2.26E-09	1.04762
rs150025803	1	25024202	LC_IGP_R18	2.29E-08	0.919606
rs34443059	14	66225456	LC_IGP_R2	2.57E-16	0.281057
rs145551505	4	128146106	LC_IGP_R2	4.95E-08	0.967254
rs182340047	4	128218015	LC_IGP_R2	4.61E-08	0.967352
rs59312400	14	66249255	LC_IGP_R2	5.05E-12	-0.271863

rs10572775	14	66055427	LC_IGP_R2	8.28E-17	0.285667
rs59850638	14	65772935	LC_IGP_R2	4.65E-09	-0.271405
rs147422602	14	65990143	LC_IGP_R2	7.99E-18	0.29441
rs72090965	14	65829951	LC_IGP_R2	2.28E-15	0.275727
rs35402762	14	65984685	LC_IGP_R2	5.68E-09	0.210566
rs201678871	14	66232956	LC_IGP_R2	4.35E-14	0.258287
rs142437037	14	65986959	LC_IGP_R2	2.09E-09	0.219017
rs200274176	14	66069056	LC_IGP_R2	4.45E-13	0.254553
rs139022691	14	65778230	LC_IGP_R2	3.60E-10	0.282557
rs5809277	14	65968613	LC_IGP_R2	8.13E-09	0.208609
rs61602020	14	65774613	LC_IGP_R2	3.32E-10	-0.285082
rs201067714	14	65772934	LC_IGP_R2	4.25E-09	-0.272282
rs34263575	14	66160754	LC_IGP_R2	2.96E-17	0.28897
rs144508295	14	65811418	LC_IGP_R2	2.69E-12	0.250205
rs34742130	14	65869387	LC_IGP_R2	1.01E-08	0.208696
rs11414976	14	65781578	LC_IGP_R2	2.30E-10	-0.354876
rs71126788	14	66232958	LC_IGP_R2	4.35E-14	0.258285
rs34746444	14	66195404	LC_IGP_R2	6.67E-15	0.272335
rs199534978	14	66076028	LC_IGP_R2	1.48E-17	0.290486
rs200183327	14	65980373	LC_IGP_R2	1.17E-09	0.221713
rs3216656	14	66113327	LC_IGP_R2	1.38E-17	0.291735
rs34785787	14	65878211	LC_IGP_R2	7.20E-09	0.209321
rs11284050	14	65884030	LC_IGP_R2	6.60E-10	0.225101
rs34307226	14	65844447	LC_IGP_R2	5.12E-14	-0.287823
rs200160352	14	66264400	LC_IGP_R2	1.72E-09	-0.228564
rs35439429	14	66063919	LC_IGP_R2	1.97E-12	0.248255
rs71126773	14	66103040	LC_IGP_R2	2.91E-17	0.28862
rs33927018	14	66029423	LC_IGP_R2	1.35E-09	0.220653
rs5809288	14	66237586	LC_IGP_R2	2.99E-14	0.26095
rs111399879	9	33166711	LC_IGP_R20	1.53E-08	0.764889
rs200345855	9	33118502	LC_IGP_R20	2.08E-08	0.794504
rs200345855	9	33118502	LC_IGP_R22	2.15E-09	0.851033
rs111399879	9	33166711	LC_IGP_R22	3.78E-10	0.848785
rs111399879	9	33166711	LC_IGP_R23	1.33E-13	0.999866
rs200345855	9	33118502	LC_IGP_R23	1.06E-12	1.00914
rs34263575	14	66160754	LC_IGP_R26	1.12E-11	0.234067
rs10572775	14	66055427	LC_IGP_R26	9.25E-12	0.235595
rs5809288	14	66237586	LC_IGP_R26	1.11E-08	0.197525
rs199534978	14	66076028	LC_IGP_R26	4.37E-12	0.237689
rs35232307	15	29348679	LC_IGP_R26	3.29E-08	-1.48311
rs34307226	14	65844447	LC_IGP_R26	2.87E-08	-0.213212
rs3216656	14	66113327	LC_IGP_R26	2.55E-12	0.241049
rs147422602	14	65990143	LC_IGP_R26	1.24E-12	0.244912
rs71126788	14	66232958	LC_IGP_R26	1.50E-08	0.195012
rs201678871	14	66232956	LC_IGP_R26	1.50E-08	0.195014
rs71126773	14	66103040	LC_IGP_R26	2.58E-12	0.240796
rs72090965	14	65829951	LC_IGP_R26	1.19E-08	0.199706
rs200274176	14	66069056	LC_IGP_R26	4.16E-08	0.194421
rs34746444	14	66195404	LC_IGP_R26	1.57E-08	0.19934
rs34443059	14	66225456	LC_IGP_R26	6.13E-10	0.213923

rs201675008	9	33120203	LC_IGP_R28	4.93E-08	-0.226949
rs3837267	9	33150971	LC_IGP_R28	2.21E-08	-0.21778
rs3837267	9	33150971	LC_IGP_R29	2.88E-08	-0.216176
rs33927018	14	66029423	LC_IGP_R3	1.07E-08	0.208435
rs147422602	14	65990143	LC_IGP_R3	7.08E-16	0.276903
rs10572775	14	66055427	LC_IGP_R3	5.80E-15	0.268474
rs201067714	14	65772934	LC_IGP_R3	6.83E-09	-0.268766
rs200160352	14	66264400	LC_IGP_R3	1.65E-08	-0.21463
rs139022691	14	65778230	LC_IGP_R3	4.06E-09	0.265451
rs199534978	14	66076028	LC_IGP_R3	7.30E-16	0.275297
rs200183327	14	65980373	LC_IGP_R3	1.02E-08	0.20899
rs59312400	14	66249255	LC_IGP_R3	6.47E-12	-0.270646
rs61602020	14	65774613	LC_IGP_R3	3.30E-09	-0.268815
rs59850638	14	65772935	LC_IGP_R3	7.30E-09	-0.268069
rs3216656	14	66113327	LC_IGP_R3	1.12E-15	0.274484
rs71126773	14	66103040	LC_IGP_R3	3.72E-15	0.269269
rs71126788	14	66232958	LC_IGP_R3	7.94E-12	0.234562
rs34443059	14	66225456	LC_IGP_R3	1.37E-14	0.264723
rs72090965	14	65829951	LC_IGP_R3	1.26E-13	0.258342
rs201678871	14	66232956	LC_IGP_R3	7.94E-12	0.234564
rs34307226	14	65844447	LC_IGP_R3	5.12E-13	-0.276393
rs200274176	14	66069056	LC_IGP_R3	2.96E-11	0.234211
rs142437037	14	65986959	LC_IGP_R3	2.65E-08	0.203696
rs144508295	14	65811418	LC_IGP_R3	1.71E-10	0.228777
rs11414976	14	65781578	LC_IGP_R3	3.47E-09	-0.331679
rs11284050	14	65884030	LC_IGP_R3	1.80E-08	0.205526
rs5809288	14	66237586	LC_IGP_R3	5.08E-12	0.237648
rs34746444	14	66195404	LC_IGP_R3	1.55E-13	0.258411
rs35439429	14	66063919	LC_IGP_R3	6.36E-11	0.231078
rs34263575	14	66160754	LC_IGP_R3	2.61E-15	0.271141
rs11348642	6	166262718	LC_IGP_R31	3.19E-08	-0.19604
rs201389301	17	38075009	LC_IGP_R32	1.82E-08	0.194334
rs199534978	14	66076028	LC_IGP_R32	1.68E-10	0.218389
rs5809288	14	66237586	LC_IGP_R32	3.49E-08	0.189954
rs34443059	14	66225456	LC_IGP_R32	1.90E-09	0.206821
rs201678871	14	66232956	LC_IGP_R32	2.97E-08	0.190169
rs200019676	17	38073841	LC_IGP_R32	3.86E-08	0.194995
rs10572775	14	66055427	LC_IGP_R32	3.85E-10	0.21549
rs200306858	17	38025643	LC_IGP_R32	1.50E-08	-0.197469
rs147422602	14	65990143	LC_IGP_R32	1.68E-10	0.219573
rs59458272	17	38020058	LC_IGP_R32	9.25E-09	-0.199071
rs34307226	14	65844447	LC_IGP_R32	1.88E-08	-0.215468
rs34746444	14	66195404	LC_IGP_R32	2.66E-08	0.195076
rs36084703	17	38063980	LC_IGP_R32	9.21E-09	-0.199768
rs113897057	17	37975214	LC_IGP_R32	9.67E-09	-0.199206
rs72538185	17	37916390	LC_IGP_R32	1.31E-08	0.197897
rs71126788	14	66232958	LC_IGP_R32	2.97E-08	0.190167
rs71971950	17	38076198	LC_IGP_R32	2.61E-08	0.193404
rs71126773	14	66103040	LC_IGP_R32	1.15E-09	0.20875
rs3216656	14	66113327	LC_IGP_R32	3.82E-10	0.214829

rs34233420	17	38004929	LC_IGP_R32	1.41E-08	-0.197108
rs72090965	14	65829951	LC_IGP_R32	2.20E-10	0.221298
rs34263575	14	66160754	LC_IGP_R32	9.67E-10	0.210015
rs200484497	17	38076205	LC_IGP_R32	4.28E-08	0.19445
rs146378727	17	38073837	LC_IGP_R32	2.26E-08	0.198076
rs148094956	17	38039561	LC_IGP_R32	7.88E-09	-0.201103
rs200216139	17	38032132	LC_IGP_R32	8.80E-09	-0.199915
rs35456268	3	186721290	LC_IGP_R33	3.30E-08	-0.226112
rs148580052	3	186720957	LC_IGP_R33	3.29E-08	-0.226177
rs59111563	3	186722848	LC_IGP_R33	3.34E-08	-0.225838
rs61210528	3	186742220	LC_IGP_R34	1.37E-10	-0.310169
rs59111563	3	186722848	LC_IGP_R34	1.47E-27	-0.433176
rs200317857	3	186713293	LC_IGP_R34	1.28E-10	-0.259861
rs71634023	3	186712738	LC_IGP_R34	2.18E-21	-0.393423
rs35456268	3	186721290	LC_IGP_R34	2.22E-27	-0.432084
rs201540659	3	186713306	LC_IGP_R34	1.04E-11	-0.331382
rs199533504	3	186713310	LC_IGP_R34	5.23E-13	-0.275808
rs11394645	3	186728353	LC_IGP_R34	7.32E-14	-0.307623
rs148580052	3	186720957	LC_IGP_R34	2.48E-27	-0.431773
rs199533504	3	186713310	LC_IGP_R35	1.30E-19	-0.347084
rs148580052	3	186720957	LC_IGP_R35	1.43E-39	-0.52418
rs200317857	3	186713293	LC_IGP_R35	1.44E-18	-0.356083
rs201540659	3	186713306	LC_IGP_R35	3.70E-15	-0.384998
rs35456268	3	186721290	LC_IGP_R35	1.28E-39	-0.524409
rs202164259	3	186711347	LC_IGP_R35	1.26E-12	-0.36382
rs11394645	3	186728353	LC_IGP_R35	1.04E-17	-0.353953
rs61210528	3	186742220	LC_IGP_R35	5.29E-15	-0.379387
rs59111563	3	186722848	LC_IGP_R35	9.06E-40	-0.524967
rs71634023	3	186712738	LC_IGP_R35	2.24E-31	-0.482918
rs34263575	14	66160754	LC_IGP_R36	2.48E-08	0.191839
rs10572775	14	66055427	LC_IGP_R36	4.90E-08	0.188228
rs71126773	14	66103040	LC_IGP_R36	2.70E-08	0.191071
rs3216656	14	66113327	LC_IGP_R36	1.93E-08	0.193151
rs199534978	14	66076028	LC_IGP_R36	1.52E-08	0.19395
rs147422602	14	65990143	LC_IGP_R36	6.10E-09	0.200249
rs34263575	14	66160754	LC_IGP_R4	7.78E-15	0.266949
rs34307226	14	65844447	LC_IGP_R4	6.22E-13	-0.275972
rs59312400	14	66249255	LC_IGP_R4	4.84E-11	-0.259802
rs142437037	14	65986959	LC_IGP_R4	6.34E-09	0.213274
rs10572775	14	66055427	LC_IGP_R4	6.69E-14	0.258265
rs3216656	14	66113327	LC_IGP_R4	4.28E-15	0.269309
rs61602020	14	65774613	LC_IGP_R4	9.16E-10	-0.278328
rs59850638	14	65772935	LC_IGP_R4	1.26E-08	-0.264115
rs71126773	14	66103040	LC_IGP_R4	1.66E-14	0.263303
rs5809288	14	66237586	LC_IGP_R4	7.82E-12	0.23585
rs11414976	14	65781578	LC_IGP_R4	1.79E-08	-0.316384
rs139022691	14	65778230	LC_IGP_R4	9.06E-10	0.276488
rs71126788	14	66232958	LC_IGP_R4	1.88E-11	0.230634
rs34746444	14	66195404	LC_IGP_R4	1.51E-11	0.237168
rs200274176	14	66069056	LC_IGP_R4	6.81E-11	0.230443

rs35439429	14	66063919	LC_IGP_R4	4.63E-10	0.221026
rs72090965	14	65829951	LC_IGP_R4	9.83E-13	0.249231
rs11284050	14	65884030	LC_IGP_R4	3.58E-08	0.201966
rs144508295	14	65811418	LC_IGP_R4	1.40E-10	0.230531
rs201067714	14	65772934	LC_IGP_R4	7.99E-09	-0.267855
rs34443059	14	66225456	LC_IGP_R4	1.06E-13	0.256156
rs147422602	14	65990143	LC_IGP_R4	3.46E-15	0.270726
rs199534978	14	66076028	LC_IGP_R4	4.38E-14	0.258376
rs201678871	14	66232956	LC_IGP_R4	1.88E-11	0.230636
rs147422602	14	65990143	LC_IGP_R41	4.21E-08	0.18858
rs199534978	14	66076028	LC_IGP_R41	4.98E-08	0.186528
rs34746444	14	66195404	LC_IGP_R41	1.91E-08	0.196672
rs3216656	14	66113327	LC_IGP_R41	2.78E-08	0.190681
rs59312400	14	66249255	LC_IGP_R41	1.33E-08	-0.22375
rs34443059	14	66225456	LC_IGP_R41	4.61E-08	0.188236
rs34307226	14	65844447	LC_IGP_R41	8.12E-09	-0.220668
rs34263575	14	66160754	LC_IGP_R41	3.84E-08	0.188888
rs9271573	6	32590501	LC_IGP_R43	2.86E-08	0.191827
rs28383895	6	32583885	LC_IGP_R43	2.81E-09	0.207154
rs199901558	6	32591315	LC_IGP_R43	4.02E-08	0.189845
rs28383233	6	32584153	LC_IGP_R43	2.34E-09	0.208398
rs35551548	6	32584192	LC_IGP_R43	4.88E-09	0.205451
rs199901558	6	32591315	LC_IGP_R45	6.98E-09	0.201047
rs28383895	6	32583885	LC_IGP_R45	4.59E-09	0.20514
rs35551548	6	32584192	LC_IGP_R45	2.19E-09	0.210821
rs9271573	6	32590501	LC_IGP_R45	3.81E-09	0.204449
rs28383233	6	32584153	LC_IGP_R45	4.23E-09	0.205796
rs78146160	6	32520157	LC_IGP_R45	1.08E-08	0.207188
rs33927018	14	66029423	LC_IGP_R5	4.24E-08	0.201196
rs5809288	14	66237586	LC_IGP_R5	1.34E-11	0.234223
rs35439429	14	66063919	LC_IGP_R5	2.14E-10	0.226095
rs59312400	14	66249255	LC_IGP_R5	1.61E-10	-0.253277
rs11284050	14	65884030	LC_IGP_R5	2.99E-08	0.203677
rs142437037	14	65986959	LC_IGP_R5	1.46E-08	0.208946
rs200274176	14	66069056	LC_IGP_R5	1.27E-10	0.228001
rs10572775	14	66055427	LC_IGP_R5	7.17E-14	0.258971
rs34746444	14	66195404	LC_IGP_R5	1.77E-11	0.237254
rs200183327	14	65980373	LC_IGP_R5	3.45E-08	0.20274
rs71126773	14	66103040	LC_IGP_R5	2.87E-14	0.261997
rs34263575	14	66160754	LC_IGP_R5	3.17E-14	0.26193
rs71126788	14	66232958	LC_IGP_R5	3.13E-11	0.229091
rs34307226	14	65844447	LC_IGP_R5	2.20E-12	-0.269953
rs34443059	14	66225456	LC_IGP_R5	2.81E-13	0.252781
rs199534978	14	66076028	LC_IGP_R5	4.45E-14	0.259428
rs147422602	14	65990143	LC_IGP_R5	5.34E-15	0.269977
rs60977949	14	65737104	LC_IGP_R5	3.41E-08	-0.218081
rs3216656	14	66113327	LC_IGP_R5	1.13E-14	0.266236
rs72090965	14	65829951	LC_IGP_R5	1.31E-13	0.259544
rs144508295	14	65811418	LC_IGP_R5	1.18E-10	0.232334
rs201678871	14	66232956	LC_IGP_R5	3.13E-11	0.229093

rs199739333	22	39777821	LC_IGP_R51	1.87E-09	-0.219628
rs79472072	1	25397198	LC_IGP_R52	3.86E-08	0.917523
rs201623089	1	25297520	LC_IGP_R52	5.19E-14	0.883437
rs186127900	1	25318225	LC_IGP_R52	6.11E-12	1.20623
rs150110347	1	25308762	LC_IGP_R52	1.59E-13	1.29542
rs143916866	1	24967537	LC_IGP_R52	1.75E-08	0.860336
rs150025803	1	25024202	LC_IGP_R52	7.44E-11	1.06884
rs186127900	1	25318225	LC_IGP_R53	3.13E-09	1.04379
rs201623089	1	25297520	LC_IGP_R53	8.39E-14	0.878452
rs150110347	1	25308762	LC_IGP_R53	2.79E-11	1.17359
rs111643673	4	162672192	LC_IGP_R55	2.31E-08	-0.590649
rs144081189	16	21271626	LC_IGP_R56	4.47E-08	-0.273494
rs199993157	14	72512919	LC_IGP_R6	2.48E-08	0.20041
rs111399879	9	33166711	LC_IGP_R62	1.86E-08	0.763867
rs200345855	9	33118502	LC_IGP_R63	1.44E-08	0.806689
rs111399879	9	33166711	LC_IGP_R63	4.90E-09	0.793781
rs3216656	14	66113327	LC_IGP_R64	4.21E-09	0.202875
rs147422602	14	65990143	LC_IGP_R64	4.99E-09	0.202315
rs199534978	14	66076028	LC_IGP_R64	8.84E-10	0.210796
rs10572775	14	66055427	LC_IGP_R64	5.83E-09	0.201701
rs34263575	14	66160754	LC_IGP_R64	9.05E-09	0.198651
rs71126773	14	66103040	LC_IGP_R64	1.52E-08	0.195326
rs199517178	9	33152342	LC_IGP_R69	1.20E-08	-0.236384
rs3837267	9	33150971	LC_IGP_R69	1.05E-08	-0.223059
rs146722048	7	34926893	LC_IGP_R7	6.96E-09	-1.13506
rs202164259	3	186711347	LC_IGP_R74	2.74E-14	-0.38851
rs61210528	3	186742220	LC_IGP_R74	5.56E-15	-0.379118
rs148580052	3	186720957	LC_IGP_R74	8.47E-49	-0.580966
rs59111563	3	186722848	LC_IGP_R74	5.78E-49	-0.581354
rs199533504	3	186713310	LC_IGP_R74	6.41E-20	-0.349763
rs201540659	3	186713306	LC_IGP_R74	3.97E-17	-0.411337
rs35456268	3	186721290	LC_IGP_R74	7.59E-49	-0.581145
rs11394645	3	186728353	LC_IGP_R74	3.53E-18	-0.358938
rs71634023	3	186712738	LC_IGP_R74	3.14E-40	-0.546968
rs200317857	3	186713293	LC_IGP_R74	1.16E-19	-0.366903
rs11394645	3	186728353	LC_IGP_R75	1.06E-12	-0.295355
rs199533504	3	186713310	LC_IGP_R75	1.20E-13	-0.285565
rs148580052	3	186720957	LC_IGP_R75	3.13E-26	-0.426112
rs35456268	3	186721290	LC_IGP_R75	2.79E-26	-0.426463
rs61210528	3	186742220	LC_IGP_R75	5.38E-09	-0.284358
rs201540659	3	186713306	LC_IGP_R75	2.05E-11	-0.329061
rs200317857	3	186713293	LC_IGP_R75	2.03E-12	-0.285896
rs71634023	3	186712738	LC_IGP_R75	7.86E-20	-0.381067
rs59111563	3	186722848	LC_IGP_R75	1.92E-26	-0.427468
rs71126773	14	66103040	LC_IGP_R8	4.18E-10	0.214597
rs10572775	14	66055427	LC_IGP_R8	1.37E-09	0.209049
rs199534978	14	66076028	LC_IGP_R8	6.66E-10	0.211545
rs34746444	14	66195404	LC_IGP_R8	1.52E-08	0.198664
rs71126788	14	66232958	LC_IGP_R8	3.35E-08	0.189785
rs34307226	14	65844447	LC_IGP_R8	2.74E-09	-0.228185

rs34443059	14	66225456	LC_IGP_R8	4.91E-09	0.201865
rs72090965	14	65829951	LC_IGP_R8	7.04E-09	0.202401
rs201678871	14	66232956	LC_IGP_R8	3.35E-08	0.189787
rs5809288	14	66237586	LC_IGP_R8	1.90E-08	0.193897
rs34263575	14	66160754	LC_IGP_R8	4.29E-10	0.214767
rs3216656	14	66113327	LC_IGP_R8	3.88E-10	0.215147
rs147422602	14	65990143	LC_IGP_R8	2.58E-10	0.217729
rs199739333	22	39777821	LC_IGP_R81	1.06E-28	-0.402759
rs201643791	22	39743579	LC_IGP_R81	1.44E-14	-0.287514
rs199739333	22	39777821	LC_IGP_R82	7.20E-21	-0.33979
rs201643791	22	39743579	LC_IGP_R82	1.81E-08	-0.21029
rs199739333	22	39777821	LC_IGP_R83	4.80E-10	-0.227521
rs114210228	6	32350107	LC_IGP_R84	3.60E-08	0.199389
rs114752560	6	32343714	LC_IGP_R84	4.00E-08	0.198706
rs151183997	16	31376341	LC_IGP_R84	2.40E-10	0.246849
rs145068357	16	31377244	LC_IGP_R84	6.02E-10	0.24221
rs66751942	6	32377716	LC_IGP_R84	4.94E-08	0.197582
rs3129954	6	32365580	LC_IGP_R84	4.11E-08	0.198495
rs114845955	6	32346772	LC_IGP_R84	3.64E-08	0.199329
rs10574650	16	31342116	LC_IGP_R84	2.86E-09	0.216692
rs3129951	6	32358286	LC_IGP_R84	2.40E-08	0.201818
rs115825744	6	32348309	LC_IGP_R84	3.62E-08	0.19936
rs3117098	6	32358513	LC_IGP_R84	3.26E-08	0.199857
rs3129955	6	32365840	LC_IGP_R84	3.56E-08	0.19946
rs116454000	6	32342822	LC_IGP_R84	4.02E-08	0.198652
rs115295735	6	32359763	LC_IGP_R84	3.19E-08	0.200213
rs3129948	6	32354644	LC_IGP_R84	3.56E-08	0.19946
rs151183997	16	31376341	LC_IGP_R85	5.47E-11	0.254625
rs145068357	16	31377244	LC_IGP_R85	1.57E-10	0.249457
rs10574650	16	31342116	LC_IGP_R85	2.27E-10	0.230369
rs201675008	9	33120203	LC_IGP_R88	7.88E-14	-0.308742
rs200647949	9	33165303	LC_IGP_R88	9.00E-10	-0.290574
rs201112848	9	33135887	LC_IGP_R88	6.01E-12	-0.254415
rs60722783	9	33147527	LC_IGP_R88	1.70E-09	-0.263978
rs58960269	9	33165305	LC_IGP_R88	3.38E-10	-0.240386
rs59286403	9	33147529	LC_IGP_R88	1.70E-09	-0.26398
rs149980337	9	33122509	LC_IGP_R88	2.05E-09	-0.227687
rs200828024	9	33152359	LC_IGP_R88	2.90E-11	-0.243935
rs150025803	1	25024202	LC_IGP_R88	2.86E-08	-0.908703
rs201949441	9	33136986	LC_IGP_R88	9.38E-12	-0.249106
rs3837267	9	33150971	LC_IGP_R88	2.67E-14	-0.294283
rs199517178	9	33152342	LC_IGP_R88	2.09E-13	-0.302133
rs200559519	9	33136992	LC_IGP_R88	8.88E-11	-0.241014
rs59286403	9	33147529	LC_IGP_R89	1.75E-11	-0.294367
rs60722783	9	33147527	LC_IGP_R89	1.75E-11	-0.294364
rs201675008	9	33120203	LC_IGP_R89	1.96E-16	-0.339318
rs200828024	9	33152359	LC_IGP_R89	5.33E-14	-0.275379
rs150025803	1	25024202	LC_IGP_R89	3.24E-08	-0.905008
rs58960269	9	33165305	LC_IGP_R89	7.79E-12	-0.261646
rs201949441	9	33136986	LC_IGP_R89	9.81E-15	-0.282376

rs201734679	9	33150136	LC_IGP_R89	1.04E-08	0.291463
rs200559519	9	33136992	LC_IGP_R89	8.59E-14	-0.27673
rs199517178	9	33152342	LC_IGP_R89	2.86E-15	-0.324572
rs3837267	9	33150971	LC_IGP_R89	2.46E-17	-0.326746
rs149980337	9	33122509	LC_IGP_R89	1.49E-11	-0.256022
rs200647949	9	33165303	LC_IGP_R89	2.31E-11	-0.316815
rs201112848	9	33135887	LC_IGP_R89	2.67E-14	-0.281091
rs113831691	2	217985392	LC_IGP_R91	3.32E-08	-0.96445
rs59111563	3	186722848	LC_IGP_R92	4.86E-27	-0.431421
rs148580052	3	186720957	LC_IGP_R92	6.84E-27	-0.430623
rs199533504	3	186713310	LC_IGP_R92	1.01E-11	-0.261584
rs71634023	3	186712738	LC_IGP_R92	4.22E-23	-0.411966
rs11394645	3	186728353	LC_IGP_R92	2.87E-10	-0.261344
rs200317857	3	186713293	LC_IGP_R92	5.87E-09	-0.236745
rs35456268	3	186721290	LC_IGP_R92	6.31E-27	-0.43084
rs148580052	3	186720957	LC_IGP_R93	5.47E-29	-0.449572
rs35456268	3	186721290	LC_IGP_R93	5.20E-29	-0.449672
rs71634023	3	186712738	LC_IGP_R93	2.27E-24	-0.42555
rs201540659	3	186713306	LC_IGP_R93	4.90E-09	-0.28836
rs202164259	3	186711347	LC_IGP_R93	5.39E-09	-0.3007
rs59111563	3	186722848	LC_IGP_R93	4.58E-29	-0.449743
rs199533504	3	186713310	LC_IGP_R93	8.07E-14	-0.288015
rs11394645	3	186728353	LC_IGP_R93	1.23E-12	-0.295243
rs200317857	3	186713293	LC_IGP_R93	1.57E-10	-0.261158
rs10574650	16	31342116	LC_IGP_R94	2.53E-09	0.217843
rs151183997	16	31376341	LC_IGP_R94	5.16E-10	0.242741
rs2050188	6	32339897	LC_IGP_R94	2.29E-08	0.199145
rs3129951	6	32358286	LC_IGP_R94	4.95E-08	0.197652
rs145068357	16	31377244	LC_IGP_R94	1.78E-09	0.235982
rs10574650	16	31342116	LC_IGP_R95	3.27E-09	0.214699
rs145068357	16	31377244	LC_IGP_R95	1.01E-08	0.22315
rs151183997	16	31376341	LC_IGP_R95	3.08E-09	0.22996
rs10574650	16	31342116	LC_IGP_RG34	2.10E-08	-0.204677
rs9268978	6	32434978	LC_IGP_RG34	2.02E-08	-0.280043
rs9271366	6	32586854	LC_IGP_RG34	2.39E-08	-0.266673
rs114210228	6	32350107	LC_IGP_RG34	4.98E-08	-0.197878
rs9268925	6	32432969	LC_IGP_RG34	1.81E-08	-0.281138
rs66500466	6	32590362	LC_IGP_RG34	2.89E-08	-0.270723
rs199625001	6	32556155	LC_IGP_RG34	1.98E-08	-0.273511
rs114249283	6	32362416	LC_IGP_RG34	8.40E-09	-0.249194
rs3129951	6	32358286	LC_IGP_RG34	3.71E-08	-0.199638
rs3135352	6	32392906	LC_IGP_RG34	4.18E-08	-0.272285
rs3129948	6	32354644	LC_IGP_RG34	4.86E-08	-0.198034
rs3104373	6	32600375	LC_IGP_RG34	2.61E-08	-0.280212
rs9268927	6	32433085	LC_IGP_RG34	2.02E-08	-0.280043
rs151183997	16	31376341	LC_IGP_RG34	7.59E-09	-0.225717
rs3129889	6	32413545	LC_IGP_RG34	2.95E-08	-0.275466
rs145068357	16	31377244	LC_IGP_RG34	8.39E-09	-0.225892
rs3129955	6	32365840	LC_IGP_RG34	4.86E-08	-0.198034
rs200533339	6	32519905	LC_IGP_RG34	2.06E-08	-0.268897

rs9269520	6	32543617	LC_IGP_RG34	1.94E-08	-0.302576
rs114619532	6	32367017	LC_IGP_RG34	3.50E-08	-0.270367
rs3135350	6	32392981	LC_IGP_RG34	4.18E-08	-0.272285
rs70993876	6	32519338	LC_IGP_RG34	4.08E-08	-0.267197
rs3135388	6	32413051	LC_IGP_RG34	2.87E-08	-0.275559
rs34233420	17	38004929	LC_IGP_SC12	4.00E-09	0.204407
rs34233420	17	38004929	LC_IGP_SC13	1.25E-08	0.198333
rs35551548	6	32584192	LC_IGP_SC15	1.69E-08	0.198996
rs28383233	6	32584153	LC_IGP_SC15	4.76E-08	0.191507
rs61602020	14	65774613	LC_IGP_SC15	3.21E-08	0.252273
rs139022691	14	65778230	LC_IGP_SC15	2.23E-08	-0.253355
rs67786416	17	38062942	LC_IGP_SC15	3.50E-08	0.197066
rs71152617	17	38032200	LC_IGP_SC15	4.95E-08	0.19149
rs72090965	14	65829951	LC_IGP_SC15	5.59E-09	-0.203957
rs34233420	17	38004929	LC_IGP_SC15	5.07E-09	0.203507
rs199739333	22	39777821	LC_IGP_SC25	3.76E-11	0.242446
rs3129868	6	32404377	LC_IGP_SC29	7.77E-09	-0.281126
rs3135350	6	32392981	LC_IGP_SC29	1.00E-08	-0.285636
rs9269081	6	32441100	LC_IGP_SC29	5.98E-09	-0.210446
rs28631719	6	32625409	LC_IGP_SC29	6.90E-09	-0.293772
rs199520858	6	32521867	LC_IGP_SC29	2.47E-08	-0.271955
rs114005501	6	32514302	LC_IGP_SC29	3.93E-08	-0.255808
rs3129955	6	32365840	LC_IGP_SC29	1.66E-11	-0.244749
rs3104373	6	32600375	LC_IGP_SC29	4.53E-09	-0.296316
rs77734842	6	32503641	LC_IGP_SC29	3.85E-08	-0.242329
rs3129954	6	32365580	LC_IGP_SC29	2.08E-11	-0.24352
rs1966001	6	32581782	LC_IGP_SC29	2.31E-08	-0.260181
rs74678949	6	32577197	LC_IGP_SC29	2.89E-08	-0.258455
rs199784558	6	32513542	LC_IGP_SC29	3.85E-08	-0.249765
rs3135388	6	32413051	LC_IGP_SC29	7.40E-09	-0.28818
rs115825744	6	32348309	LC_IGP_SC29	1.70E-11	-0.244653
rs182016754	6	32352737	LC_IGP_SC29	7.92E-09	-0.241162
rs116192447	6	32378940	LC_IGP_SC29	2.11E-11	-0.243669
rs146620734	6	32352220	LC_IGP_SC29	1.47E-08	-0.257234
rs201875530	6	32551762	LC_IGP_SC29	3.06E-08	-0.235449
rs114210228	6	32350107	LC_IGP_SC29	1.69E-11	-0.244681
rs9270656	6	32566011	LC_IGP_SC29	2.31E-08	-0.260181
rs112971792	6	32380398	LC_IGP_SC29	5.29E-11	-0.240171
rs9268927	6	32433085	LC_IGP_SC29	3.29E-09	-0.296408
rs70993876	6	32519338	LC_IGP_SC29	2.92E-09	-0.290024
esv2661745	6	32480579	LC_IGP_SC29	1.23E-08	-0.307797
rs9268925	6	32432969	LC_IGP_SC29	2.16E-09	-0.299996
rs114693266	6	32380717	LC_IGP_SC29	2.54E-11	-0.242803
rs9269448	6	32542011	LC_IGP_SC29	3.87E-08	-0.288535
rs6923504	6	32428186	LC_IGP_SC29	1.34E-08	-0.201716
rs3117098	6	32358513	LC_IGP_SC29	1.35E-11	-0.245619
rs114249283	6	32362416	LC_IGP_SC29	1.13E-08	-0.248081
rs114845955	6	32346772	LC_IGP_SC29	1.71E-11	-0.244622
rs149028172	6	32449523	LC_IGP_SC29	4.79E-09	-0.215587
rs9269298	6	32539536	LC_IGP_SC29	4.28E-08	-0.246613

rs147401848	6	32406843	LC_IGP_SC29	8.62E-09	-0.280428
rs10574650	16	31342116	LC_IGP_SC29	4.93E-09	-0.21449
rs113738020	6	32540158	LC_IGP_SC29	1.69E-08	-0.277403
rs116350876	6	32452470	LC_IGP_SC29	8.41E-09	-0.202986
rs2050188	6	32339897	LC_IGP_SC29	3.15E-10	-0.224901
rs200121045	6	32551756	LC_IGP_SC29	4.24E-08	-0.235541
rs34009248	6	32513543	LC_IGP_SC29	3.83E-08	-0.255441
rs149826815	6	32368593	LC_IGP_SC29	8.11E-10	-0.220502
rs114752560	6	32343714	LC_IGP_SC29	1.92E-11	-0.243976
rs200533339	6	32519905	LC_IGP_SC29	1.00E-09	-0.29395
rs114619532	6	32367017	LC_IGP_SC29	1.07E-08	-0.281526
rs199625001	6	32556155	LC_IGP_SC29	1.17E-09	-0.297416
rs9271160	6	32577646	LC_IGP_SC29	2.31E-08	-0.260181
rs116454000	6	32342822	LC_IGP_SC29	1.92E-11	-0.243924
rs66500466	6	32590362	LC_IGP_SC29	2.60E-09	-0.291589
rs3135352	6	32392906	LC_IGP_SC29	1.00E-08	-0.285636
rs3135391	6	32410987	LC_IGP_SC29	1.34E-08	-0.283067
rs9271203	6	32578885	LC_IGP_SC29	2.31E-08	-0.260181
rs115295735	6	32359763	LC_IGP_SC29	1.35E-11	-0.24591
rs66751942	6	32377716	LC_IGP_SC29	2.09E-11	-0.243798
rs9269203	6	32449775	LC_IGP_SC29	2.89E-08	-0.193659
esv2661368	6	32514888	LC_IGP_SC29	2.56E-08	-0.273073
rs4639377	6	32625283	LC_IGP_SC29	1.49E-08	-0.279693
rs9269675	6	32546515	LC_IGP_SC29	2.43E-08	-0.293833
rs200565579	6	32551764	LC_IGP_SC29	4.53E-08	-0.23497
rs6903608	6	32428285	LC_IGP_SC29	1.49E-08	-0.201244
rs9281913	6	32567633	LC_IGP_SC29	8.68E-09	-0.297301
rs9268978	6	32434978	LC_IGP_SC29	3.29E-09	-0.296408
rs141034368	6	32353166	LC_IGP_SC29	4.53E-08	-0.211661
rs3129889	6	32413545	LC_IGP_SC29	6.30E-09	-0.289671
rs9269190	6	32448500	LC_IGP_SC29	1.74E-08	-0.200022
rs9271191	6	32578449	LC_IGP_SC29	1.78E-08	-0.263478
rs76485153	6	32520259	LC_IGP_SC29	2.74E-08	-0.25848
rs151183997	16	31376341	LC_IGP_SC29	1.38E-09	-0.237545
rs9269520	6	32543617	LC_IGP_SC29	1.42E-09	-0.327184
rs9271366	6	32586854	LC_IGP_SC29	9.80E-10	-0.293007
rs3129948	6	32354644	LC_IGP_SC29	1.66E-11	-0.244749
rs9268880	6	32431358	LC_IGP_SC29	1.34E-08	-0.201716
rs142975901	6	32517357	LC_IGP_SC29	1.63E-08	-0.259215
rs3129951	6	32358286	LC_IGP_SC29	9.14E-12	-0.247708
rs3129882	6	32409530	LC_IGP_SC29	4.05E-08	-0.190816
rs145068357	16	31377244	LC_IGP_SC29	2.36E-09	-0.235052
rs10574650	16	31342116	LC_IGP_SC30	3.73E-09	-0.214749
rs151183997	16	31376341	LC_IGP_SC30	1.25E-09	-0.23663
rs145068357	16	31377244	LC_IGP_SC30	3.27E-09	-0.231438
rs142076303	18	45653209	LC_IGP_SC34	3.19E-08	0.278837
rs199739333	22	39777821	LC_IGP_SC35	9.61E-17	0.303055
rs201643791	22	39743579	LC_IGP_SC35	1.47E-09	0.226487
rs199739333	22	39777821	LC_IGP_SC36	1.41E-08	0.207763
chr1:240956912:I	1	240956912	LC_IGP_SC36	2.73E-08	-0.596808

rs114210228	6	32350107	LC_IGP_SC39	1.10E-09	-0.220791
rs149826815	6	32368593	LC_IGP_SC39	4.63E-08	-0.195575
rs115295735	6	32359763	LC_IGP_SC39	8.92E-10	-0.222066
rs66751942	6	32377716	LC_IGP_SC39	1.24E-09	-0.22032
rs3129951	6	32358286	LC_IGP_SC39	6.00E-10	-0.224134
rs112971792	6	32380398	LC_IGP_SC39	2.13E-09	-0.218441
rs114693266	6	32380717	LC_IGP_SC39	1.50E-09	-0.219257
rs3129948	6	32354644	LC_IGP_SC39	1.13E-09	-0.220658
rs2050188	6	32339897	LC_IGP_SC39	1.67E-09	-0.214609
rs10574650	16	31342116	LC_IGP_SC39	1.09E-08	-0.208856
rs3129955	6	32365840	LC_IGP_SC39	1.13E-09	-0.220658
rs114752560	6	32343714	LC_IGP_SC39	9.96E-10	-0.221354
rs3129954	6	32365580	LC_IGP_SC39	1.39E-09	-0.219401
rs145068357	16	31377244	LC_IGP_SC39	1.12E-08	-0.223997
rs116454000	6	32342822	LC_IGP_SC39	9.61E-10	-0.221526
rs114845955	6	32346772	LC_IGP_SC39	1.09E-09	-0.220889
rs115825744	6	32348309	LC_IGP_SC39	1.10E-09	-0.22084
rs151183997	16	31376341	LC_IGP_SC39	1.06E-08	-0.223542
rs116192447	6	32378940	LC_IGP_SC39	1.24E-09	-0.220266
rs3117098	6	32358513	LC_IGP_SC39	8.37E-10	-0.222191
rs111399879	9	33166711	LC_IGP_SC4	3.43E-08	-0.748391
rs10574650	16	31342116	LC_IGP_SC40	1.21E-10	-0.234431
rs151183997	16	31376341	LC_IGP_SC40	8.12E-10	-0.239318
rs145068357	16	31377244	LC_IGP_SC40	7.73E-10	-0.24051
rs143099595	7	87828627	LC_IGP_SC7	4.80E-08	0.431621
rs9271573	6	32590501	LC_IGP101	1.21E-08	-0.19765
rs199901558	6	32591315	LC_IGP101	2.24E-08	-0.194084
rs150025803	1	25024202	LC_IGP108	5.17E-10	1.0214
rs150110347	1	25308762	LC_IGP108	1.42E-11	1.18752
rs186127900	1	25318225	LC_IGP108	5.53E-10	1.08939
rs201623089	1	25297520	LC_IGP108	1.71E-10	0.751453
rs34263575	14	66160754	LC_IGP11	3.88E-19	-0.304739
rs35402762	14	65984685	LC_IGP11	2.07E-11	-0.241249
rs202063544	14	65803008	LC_IGP11	3.57E-08	0.36226
rs201067714	14	65772934	LC_IGP11	8.99E-09	0.265958
rs200019676	17	38073841	LC_IGP11	1.86E-08	-0.19927
rs10572775	14	66055427	LC_IGP11	2.41E-18	-0.298733
rs34307226	14	65844447	LC_IGP11	1.07E-18	0.33563
rs72090965	14	65829951	LC_IGP11	4.38E-15	-0.272358
rs35439429	14	66063919	LC_IGP11	1.30E-14	-0.270958
rs148094956	17	38039561	LC_IGP11	3.76E-08	0.191559
rs145473242	14	65826211	LC_IGP11	1.52E-10	0.273672
rs201712507	14	65856090	LC_IGP11	3.76E-10	0.26948
rs199534978	14	66076028	LC_IGP11	1.56E-19	-0.306814
rs60977949	14	65737104	LC_IGP11	1.66E-08	0.221097
rs33927018	14	66029423	LC_IGP11	5.26E-12	-0.250142
rs5809288	14	66237586	LC_IGP11	1.70E-14	-0.262821
rs61602020	14	65774613	LC_IGP11	7.76E-10	0.278499
rs200306858	17	38025643	LC_IGP11	3.63E-08	0.191968
rs59850638	14	65772935	LC_IGP11	1.06E-08	0.264487

rs57476621	14	66122725	LC_IGP11	3.56E-09	-0.212381
rs72538185	17	37916390	LC_IGP11	2.37E-08	-0.194198
rs71126773	14	66103040	LC_IGP11	1.10E-18	-0.300462
rs201678871	14	66232956	LC_IGP11	2.54E-14	-0.260059
rs11414976	14	65781578	LC_IGP11	4.03E-12	0.387264
rs146378727	17	38073837	LC_IGP11	1.48E-08	-0.20044
rs36084703	17	38063980	LC_IGP11	2.31E-08	0.194128
rs75635798	14	65809257	LC_IGP11	4.37E-08	0.359112
rs200183327	14	65980373	LC_IGP11	4.41E-12	-0.251326
rs59312400	14	66249255	LC_IGP11	5.48E-15	0.306432
rs200160352	14	66264400	LC_IGP11	6.93E-09	0.219531
rs34443059	14	66225456	LC_IGP11	1.73E-17	-0.291107
rs59318404	14	65803010	LC_IGP11	3.57E-08	0.362262
rs3216656	14	66113327	LC_IGP11	1.87E-19	-0.307205
rs200216139	17	38032132	LC_IGP11	1.94E-08	0.195071
rs113897057	17	37975214	LC_IGP11	5.25E-09	0.202526
rs34746444	14	66195404	LC_IGP11	2.59E-18	-0.303595
rs59458272	17	38020058	LC_IGP11	6.93E-09	0.200536
rs5809277	14	65968613	LC_IGP11	3.19E-11	-0.239229
rs11284050	14	65884030	LC_IGP11	1.38E-12	-0.257246
rs112066572	14	65812365	LC_IGP11	2.43E-10	0.270117
rs147422602	14	65990143	LC_IGP11	8.78E-20	-0.310547
rs72416175	14	66116826	LC_IGP11	4.92E-09	0.263129
rs34785787	14	65878211	LC_IGP11	3.75E-11	-0.238358
rs144508295	14	65811418	LC_IGP11	1.10E-12	-0.253947
rs34233420	17	38004929	LC_IGP11	1.20E-08	0.197848
rs139022691	14	65778230	LC_IGP11	6.11E-10	-0.278264
rs200274176	14	66069056	LC_IGP11	7.01E-16	-0.282536
rs71126788	14	66232958	LC_IGP11	2.54E-14	-0.260056
rs34742130	14	65869387	LC_IGP11	6.12E-11	-0.237359
rs142437037	14	65986959	LC_IGP11	8.50E-12	-0.248745
rs59111563	3	186722848	LC_IGP110	6.14E-09	-0.238432
rs35456268	3	186721290	LC_IGP110	6.31E-09	-0.23842
rs148580052	3	186720957	LC_IGP110	6.41E-09	-0.238352
rs35456268	3	186721290	LC_IGP111	1.96E-34	-0.492243
rs201540659	3	186713306	LC_IGP111	1.83E-12	-0.348067
rs202164259	3	186711347	LC_IGP111	3.18E-10	-0.325106
rs200317857	3	186713293	LC_IGP111	5.68E-15	-0.318551
rs59111563	3	186722848	LC_IGP111	1.24E-34	-0.493303
rs199533504	3	186713310	LC_IGP111	1.89E-14	-0.29653
rs11394645	3	186728353	LC_IGP111	4.36E-13	-0.303126
rs148580052	3	186720957	LC_IGP111	2.26E-34	-0.491874
rs71634023	3	186712738	LC_IGP111	1.88E-26	-0.445084
rs61210528	3	186742220	LC_IGP111	6.33E-11	-0.320632
rs5809288	14	66237586	LC_IGP12	5.88E-13	-0.247482
rs59850638	14	65772935	LC_IGP12	1.06E-08	0.264942
rs201067714	14	65772934	LC_IGP12	7.21E-09	0.268132
rs200274176	14	66069056	LC_IGP12	1.81E-12	-0.247792
rs10572775	14	66055427	LC_IGP12	1.21E-15	-0.274674
rs201678871	14	66232956	LC_IGP12	8.55E-13	-0.244786

rs5809277	14	65968613	LC_IGP12	1.81E-08	-0.203645
rs71126773	14	66103040	LC_IGP12	4.40E-16	-0.277701
rs142437037	14	65986959	LC_IGP12	4.68E-09	-0.214132
rs200183327	14	65980373	LC_IGP12	1.89E-09	-0.218841
rs59312400	14	66249255	LC_IGP12	4.12E-12	0.272872
rs34263575	14	66160754	LC_IGP12	3.98E-16	-0.278506
rs33927018	14	66029423	LC_IGP12	2.05E-09	-0.218118
rs34443059	14	66225456	LC_IGP12	5.92E-15	-0.267945
rs71126788	14	66232958	LC_IGP12	8.55E-13	-0.244784
rs61602020	14	65774613	LC_IGP12	6.11E-10	0.280694
rs145473242	14	65826211	LC_IGP12	3.16E-08	0.237265
rs199534978	14	66076028	LC_IGP12	1.29E-16	-0.281896
rs139022691	14	65778230	LC_IGP12	6.02E-10	-0.278863
rs11284050	14	65884030	LC_IGP12	1.20E-09	-0.221603
rs34746444	14	66195404	LC_IGP12	3.12E-14	-0.265454
rs3216656	14	66113327	LC_IGP12	2.12E-16	-0.280862
rs34307226	14	65844447	LC_IGP12	2.33E-14	0.291529
rs72090965	14	65829951	LC_IGP12	1.65E-14	-0.26709
rs200160352	14	66264400	LC_IGP12	1.70E-08	0.21413
rs11414976	14	65781578	LC_IGP12	4.53E-10	0.348893
rs35402762	14	65984685	LC_IGP12	1.18E-08	-0.206076
rs34785787	14	65878211	LC_IGP12	1.64E-08	-0.204224
rs147422602	14	65990143	LC_IGP12	1.47E-16	-0.282866
rs34742130	14	65869387	LC_IGP12	2.09E-08	-0.204078
rs144508295	14	65811418	LC_IGP12	7.60E-12	-0.244914
rs35439429	14	66063919	LC_IGP12	5.89E-12	-0.242783
rs148580052	3	186720957	LC_IGP120	1.13E-29	-0.454295
rs61210528	3	186742220	LC_IGP120	5.31E-10	-0.302764
rs201540659	3	186713306	LC_IGP120	1.38E-11	-0.332217
rs35456268	3	186721290	LC_IGP120	1.03E-29	-0.454518
rs202164259	3	186711347	LC_IGP120	1.09E-08	-0.293509
rs59111563	3	186722848	LC_IGP120	8.06E-30	-0.45499
rs71634023	3	186712738	LC_IGP120	5.29E-24	-0.421583
rs199533504	3	186713310	LC_IGP120	1.19E-12	-0.274087
rs200317857	3	186713293	LC_IGP120	2.37E-14	-0.310093
rs11394645	3	186728353	LC_IGP120	7.22E-11	-0.270818
rs35456268	3	186721290	LC_IGP121	1.37E-09	-0.24721
rs59111563	3	186722848	LC_IGP121	1.38E-09	-0.246977
rs148580052	3	186720957	LC_IGP121	1.38E-09	-0.247198
rs61210528	3	186742220	LC_IGP122	5.69E-15	-0.378959
rs201540659	3	186713306	LC_IGP122	3.91E-17	-0.41141
rs199533504	3	186713310	LC_IGP122	6.47E-20	-0.349712
rs148580052	3	186720957	LC_IGP122	9.82E-49	-0.58057
rs71634023	3	186712738	LC_IGP122	3.70E-40	-0.546462
rs202164259	3	186711347	LC_IGP122	2.63E-14	-0.388756
rs200317857	3	186713293	LC_IGP122	1.30E-19	-0.366409
rs59111563	3	186722848	LC_IGP122	6.67E-49	-0.580974
rs35456268	3	186721290	LC_IGP122	8.79E-49	-0.580753
rs11394645	3	186728353	LC_IGP122	3.36E-18	-0.359155
rs71634023	3	186712738	LC_IGP123	7.66E-20	-0.381172

rs148580052	3	186720957	LC_IGP123	3.06E-26	-0.426174
rs200317857	3	186713293	LC_IGP123	2.01E-12	-0.285927
rs199533504	3	186713310	LC_IGP123	1.24E-13	-0.285371
rs11394645	3	186728353	LC_IGP123	1.09E-12	-0.295238
rs59111563	3	186722848	LC_IGP123	1.88E-26	-0.427529
rs61210528	3	186742220	LC_IGP123	5.12E-09	-0.284745
rs35456268	3	186721290	LC_IGP123	2.73E-26	-0.426524
rs201540659	3	186713306	LC_IGP123	1.98E-11	-0.329271
rs149548422	4	162694485	LC_IGP129	3.54E-08	-0.600309
rs111643673	4	162672192	LC_IGP129	2.05E-08	-0.595979
rs72090965	14	65829951	LC_IGP13	4.46E-08	-0.191467
rs111643673	4	162672192	LC_IGP131	1.83E-08	-0.598035
rs149548422	4	162694485	LC_IGP131	3.21E-08	-0.602146
rs201623089	1	25297520	LC_IGP133	2.53E-08	-0.658549
rs143916866	1	24967537	LC_IGP133	1.75E-08	-0.86308
rs150025803	1	25024202	LC_IGP133	1.12E-10	-1.06208
rs111399879	9	33166711	LC_IGP133	8.36E-09	0.782102
rs200345855	9	33118502	LC_IGP133	2.82E-08	0.790552
rs201623089	1	25297520	LC_IGP135	1.46E-10	0.752807
rs143916866	1	24967537	LC_IGP135	9.53E-09	0.874566
rs150025803	1	25024202	LC_IGP135	2.32E-12	1.14808
rs199739333	22	39777821	LC_IGP135	1.02E-08	-0.210022
rs150110347	1	25308762	LC_IGP135	1.65E-09	1.05938
rs34307226	14	65844447	LC_IGP138	1.42E-10	0.245907
rs200274176	14	66069056	LC_IGP138	2.53E-08	-0.196704
rs10572775	14	66055427	LC_IGP138	2.45E-10	-0.218642
rs199534978	14	66076028	LC_IGP138	7.11E-11	-0.223512
rs59312400	14	66249255	LC_IGP138	1.43E-10	0.252713
rs34263575	14	66160754	LC_IGP138	7.55E-11	-0.224161
rs34443059	14	66225456	LC_IGP138	1.75E-10	-0.220343
rs5809288	14	66237586	LC_IGP138	3.67E-09	-0.203653
rs71126788	14	66232958	LC_IGP138	2.85E-09	-0.204277
rs201678871	14	66232956	LC_IGP138	2.85E-09	-0.204277
rs3216656	14	66113327	LC_IGP138	5.08E-11	-0.22601
rs71126773	14	66103040	LC_IGP138	1.33E-10	-0.220932
rs72090965	14	65829951	LC_IGP138	3.17E-08	-0.193568
rs147422602	14	65990143	LC_IGP138	6.91E-11	-0.224881
rs34746444	14	66195404	LC_IGP138	3.11E-11	-0.233017
rs11414976	14	65781578	LC_IGP14	5.65E-10	0.347882
rs10572775	14	66055427	LC_IGP14	1.08E-15	-0.275902
rs200183327	14	65980373	LC_IGP14	9.52E-09	-0.210115
rs59312400	14	66249255	LC_IGP14	8.38E-12	0.269707
rs11284050	14	65884030	LC_IGP14	2.22E-09	-0.218966
rs59850638	14	65772935	LC_IGP14	1.44E-08	0.262977
rs33927018	14	66029423	LC_IGP14	1.35E-08	-0.207733
rs34443059	14	66225456	LC_IGP14	1.56E-16	-0.283744
rs34307226	14	65844447	LC_IGP14	1.75E-13	0.282371
rs201067714	14	65772934	LC_IGP14	1.33E-08	0.263843
rs34746444	14	66195404	LC_IGP14	7.74E-14	-0.262265
rs5809288	14	66237586	LC_IGP14	7.48E-15	-0.267428

rs60977949	14	65737104	LC_IGP14		2.73E-08	0.218425
rs142437037	14	65986959	LC_IGP14		5.66E-10	-0.227455
rs61602020	14	65774613	LC_IGP14		6.63E-10	0.280559
rs147422602	14	65990143	LC_IGP14		1.53E-17	-0.292596
rs71126773	14	66103040	LC_IGP14		1.75E-16	-0.282193
rs200274176	14	66069056	LC_IGP14		9.98E-12	-0.240208
rs144508295	14	65811418	LC_IGP14		6.23E-12	-0.246731
rs199534978	14	66076028	LC_IGP14		6.77E-16	-0.275942
rs3216656	14	66113327	LC_IGP14		3.56E-17	-0.288734
rs71126788	14	66232958	LC_IGP14		2.00E-14	-0.262155
rs34263575	14	66160754	LC_IGP14		6.17E-17	-0.286758
rs35439429	14	66063919	LC_IGP14		4.75E-11	-0.233136
rs201678871	14	66232956	LC_IGP14		2.00E-14	-0.262158
rs139022691	14	65778230	LC_IGP14		6.01E-10	-0.279321
rs72090965	14	65829951	LC_IGP14		6.04E-14	-0.262027
rs111399879	9	33166711	LC_IGP144		2.69E-08	-0.755283
rs150025803	1	25024202	LC_IGP144		4.21E-08	0.904513
rs200345855	9	33118502	LC_IGP145		2.43E-08	0.79542
rs111399879	9	33166711	LC_IGP145		7.56E-09	0.785589
rs59249257	2	129965822	LC_IGP148		3.91E-08	-0.315743
rs59312400	14	66249255	LC_IGP148		3.76E-08	-0.217391
rs10572775	14	66055427	LC_IGP148		2.46E-08	0.193039
rs34263575	14	66160754	LC_IGP148		5.26E-09	0.201516
rs3216656	14	66113327	LC_IGP148		2.86E-09	0.20479
rs34443059	14	66225456	LC_IGP148		5.31E-09	0.201922
rs34307226	14	65844447	LC_IGP148		3.92E-08	-0.211233
rs71126773	14	66103040	LC_IGP148		6.66E-09	0.199872
rs34746444	14	66195404	LC_IGP148		1.40E-09	0.212909
rs199534978	14	66076028	LC_IGP148		1.51E-08	0.194587
rs147422602	14	65990143	LC_IGP148		5.30E-09	0.201712
rs3216656	14	66113327	LC_IGP15		1.39E-11	-0.233386
rs34307226	14	65844447	LC_IGP15		5.51E-10	0.238794
rs199534978	14	66076028	LC_IGP15		3.54E-11	-0.227966
rs34263575	14	66160754	LC_IGP15		3.27E-11	-0.22925
rs59312400	14	66249255	LC_IGP15		7.03E-09	0.229489
rs200274176	14	66069056	LC_IGP15		4.11E-08	-0.194748
rs71126788	14	66232958	LC_IGP15		6.90E-10	-0.212891
rs147422602	14	65990143	LC_IGP15		6.13E-12	-0.23783
rs71126773	14	66103040	LC_IGP15		1.86E-11	-0.231743
rs34746444	14	66195404	LC_IGP15		1.30E-09	-0.214261
rs144508295	14	65811418	LC_IGP15		2.21E-09	-0.215808
rs72090965	14	65829951	LC_IGP15		2.41E-11	-0.234336
rs34443059	14	66225456	LC_IGP15		4.94E-11	-0.227744
rs10572775	14	66055427	LC_IGP15		9.98E-11	-0.224141
rs201678871	14	66232956	LC_IGP15		6.90E-10	-0.212892
rs5809288	14	66237586	LC_IGP15		3.20E-10	-0.217861
rs28383895	6	32583885	LC_IGP150		1.56E-08	0.198675
rs35551548	6	32584192	LC_IGP150		2.13E-08	0.198116
rs28383233	6	32584153	LC_IGP150		1.27E-08	0.200084
rs201623089	1	25297520	LC_IGP153		4.84E-09	-0.690301

rs150110347	1	25308762	LC_IGP153	3.93E-08	-0.969381
rs186127900	1	25318225	LC_IGP155	3.24E-09	1.03919
rs201623089	1	25297520	LC_IGP155	5.96E-11	0.769171
rs199739333	22	39777821	LC_IGP155	3.32E-08	-0.202794
rs150025803	1	25024202	LC_IGP155	2.46E-09	0.979211
rs150110347	1	25308762	LC_IGP155	1.31E-10	1.1293
rs199739333	22	39777821	LC_IGP156	2.72E-10	-0.231415
rs150025803	1	25024202	LC_IGP157	1.54E-10	1.0518
rs201623089	1	25297520	LC_IGP157	9.37E-13	0.839371
rs143916866	1	24967537	LC_IGP157	1.97E-08	0.85786
rs186127900	1	25318225	LC_IGP157	2.79E-11	1.16903
rs150110347	1	25308762	LC_IGP157	1.01E-12	1.25297
rs186127900	1	25318225	LC_IGP158	1.45E-09	1.06491
rs201623089	1	25297520	LC_IGP158	8.62E-13	0.841747
rs150110347	1	25308762	LC_IGP158	1.41E-11	1.19003
rs201623089	1	25297520	LC_IGP159	1.43E-09	0.711913
rs150025803	1	25024202	LC_IGP159	1.68E-08	0.92679
rs186127900	1	25318225	LC_IGP159	4.48E-09	1.02979
rs150110347	1	25308762	LC_IGP159	1.72E-10	1.12193
rs199739333	22	39777821	LC_IGP160	6.65E-09	-0.212838
rs201623089	1	25297520	LC_IGP161	9.58E-12	0.800837
rs186127900	1	25318225	LC_IGP161	4.80E-11	1.15415
rs150110347	1	25308762	LC_IGP161	1.14E-12	1.24908
rs150025803	1	25024202	LC_IGP161	1.10E-10	1.05914
rs143916866	1	24967537	LC_IGP161	3.36E-08	0.843131
rs186127900	1	25318225	LC_IGP162	1.52E-08	0.998535
rs201623089	1	25297520	LC_IGP162	7.11E-10	0.728143
rs150110347	1	25308762	LC_IGP162	2.37E-10	1.11845
rs199901558	6	32591315	LC_IGP165	4.11E-08	-0.191154
rs9271573	6	32590501	LC_IGP165	2.31E-08	-0.194577
rs186127900	1	25318225	LC_IGP168	3.17E-09	1.03946
rs201623089	1	25297520	LC_IGP168	4.70E-11	0.772993
rs150025803	1	25024202	LC_IGP168	8.32E-09	0.94595
rs150110347	1	25308762	LC_IGP168	1.16E-10	1.13204
rs186127900	1	25318225	LC_IGP169	3.30E-09	1.03834
rs201623089	1	25297520	LC_IGP169	4.96E-11	0.772148
rs150025803	1	25024202	LC_IGP169	8.57E-09	0.945247
rs150110347	1	25308762	LC_IGP169	1.22E-10	1.13099
rs146722048	7	34926893	LC_IGP17	3.21E-09	1.15903
rs201623089	1	25297520	LC_IGP171	1.73E-09	-0.708363
rs150110347	1	25308762	LC_IGP171	6.38E-10	-1.08679
rs186127900	1	25318225	LC_IGP171	1.35E-08	-0.997504
rs150025803	1	25024202	LC_IGP175	4.10E-10	-1.02166
rs201675008	9	33120203	LC_IGP175	3.96E-08	-0.227748
rs199775279	1	25101702	LC_IGP175	4.29E-08	-0.927173
rs201643791	22	39743579	LC_IGP176	4.70E-08	-0.205155
rs150025803	1	25024202	LC_IGP176	1.89E-08	0.927709
rs199739333	22	39777821	LC_IGP176	2.43E-14	-0.27918
rs201643791	22	39743579	LC_IGP177	2.47E-10	-0.236198
rs199739333	22	39777821	LC_IGP177	3.36E-25	-0.375016

rs113831691	2	217985392	LC_IGP178		6.02E-09	-1.01898
rs201540659	3	186713306	LC_IGP179		2.82E-08	-0.272496
rs59111563	3	186722848	LC_IGP179		1.66E-16	-0.332633
rs200317857	3	186713293	LC_IGP179		3.38E-09	-0.240358
rs35456268	3	186721290	LC_IGP179		1.83E-16	-0.332441
rs71634023	3	186712738	LC_IGP179		9.32E-15	-0.324238
rs199533504	3	186713310	LC_IGP179		5.16E-11	-0.252471
rs148580052	3	186720957	LC_IGP179		1.89E-16	-0.332328
rs11394645	3	186728353	LC_IGP179		2.26E-08	-0.231949
rs201112848	9	33135887	LC_IGP180		1.89E-10	-0.236938
rs60722783	9	33147527	LC_IGP180		4.64E-08	-0.240862
rs150025803	1	25024202	LC_IGP180		3.47E-09	-0.971089
rs201675008	9	33120203	LC_IGP180		6.04E-11	-0.272233
rs200559519	9	33136992	LC_IGP180		3.95E-09	-0.220062
rs3837267	9	33150971	LC_IGP180		1.10E-10	-0.251248
rs199517178	9	33152342	LC_IGP180		5.27E-10	-0.2574
rs200828024	9	33152359	LC_IGP180		5.30E-09	-0.21541
rs59286403	9	33147529	LC_IGP180		4.64E-08	-0.240867
rs143916866	1	24967537	LC_IGP180		3.63E-08	-0.841393
rs201949441	9	33136986	LC_IGP180		7.55E-10	-0.226287
rs116192447	6	32378940	LC_IGP181		7.87E-09	0.208978
rs3117098	6	32358513	LC_IGP181		4.71E-09	0.211742
rs116454000	6	32342822	LC_IGP181		5.57E-09	0.210857
rs9269081	6	32441100	LC_IGP181		3.77E-08	0.198047
rs151183997	16	31376341	LC_IGP181		2.23E-10	0.247359
rs3129955	6	32365840	LC_IGP181		4.86E-09	0.211718
rs114693266	6	32380717	LC_IGP181		1.08E-08	0.207158
rs115825744	6	32348309	LC_IGP181		4.96E-09	0.21161
rs112971792	6	32380398	LC_IGP181		1.96E-08	0.20462
rs3129954	6	32365580	LC_IGP181		5.58E-09	0.210842
rs114845955	6	32346772	LC_IGP181		4.99E-09	0.211576
rs10574650	16	31342116	LC_IGP181		3.34E-09	0.215837
rs145068357	16	31377244	LC_IGP181		5.76E-10	0.242558
rs149028172	6	32449523	LC_IGP181		1.89E-08	0.206098
rs114210228	6	32350107	LC_IGP181		4.93E-09	0.211642
rs2050188	6	32339897	LC_IGP181		5.48E-09	0.207388
rs3129948	6	32354644	LC_IGP181		4.86E-09	0.211718
rs114752560	6	32343714	LC_IGP181		5.57E-09	0.210893
rs115295735	6	32359763	LC_IGP181		4.70E-09	0.211973
rs66751942	6	32377716	LC_IGP181		7.28E-09	0.20951
rs3129951	6	32358286	LC_IGP181		3.65E-09	0.213314
rs145068357	16	31377244	LC_IGP182		2.08E-09	0.233719
rs10574650	16	31342116	LC_IGP182		1.15E-08	0.207542
rs151183997	16	31376341	LC_IGP182		9.47E-10	0.237731
rs199739333	22	39777821	LC_IGP183		4.97E-22	-0.350798
rs201643791	22	39743579	LC_IGP183		2.34E-10	-0.237287
rs150025803	1	25024202	LC_IGP184		4.85E-08	-0.898471
rs200317857	3	186713293	LC_IGP186		4.57E-11	-0.268572
rs35456268	3	186721290	LC_IGP186		1.49E-27	-0.438538
rs148580052	3	186720957	LC_IGP186		1.60E-27	-0.438352

rs59111563	3	186722848	LC_IGP186	1.21E-27	-0.438934
rs199533504	3	186713310	LC_IGP186	6.03E-13	-0.277771
rs201540659	3	186713306	LC_IGP186	5.05E-09	-0.288123
rs71634023	3	186712738	LC_IGP186	1.14E-23	-0.419632
rs11394645	3	186728353	LC_IGP186	1.34E-10	-0.267309
rs478937	11	114324183	LC_IGP186	2.10E-08	0.228464
rs201112848	9	33135887	LC_IGP187	8.34E-11	-0.241309
rs200317857	3	186713293	LC_IGP187	4.87E-08	-0.222664
rs35456268	3	186721290	LC_IGP187	4.60E-19	-0.360709
rs148580052	3	186720957	LC_IGP187	4.76E-19	-0.360623
rs59111563	3	186722848	LC_IGP187	4.30E-19	-0.360713
rs71634023	3	186712738	LC_IGP187	8.86E-17	-0.348724
rs199533504	3	186713310	LC_IGP187	4.35E-09	-0.226697
rs200828024	9	33152359	LC_IGP187	1.31E-08	-0.209652
rs201949441	9	33136986	LC_IGP187	1.19E-09	-0.22349
rs11394645	3	186728353	LC_IGP187	3.02E-08	-0.230581
rs200559519	9	33136992	LC_IGP187	8.06E-09	-0.215484
rs71634023	3	186712738	LC_IGP189	3.87E-23	-0.412355
rs199533504	3	186713310	LC_IGP189	8.68E-12	-0.262446
rs200317857	3	186713293	LC_IGP189	5.89E-09	-0.236746
rs35456268	3	186721290	LC_IGP189	6.45E-27	-0.430811
rs148580052	3	186720957	LC_IGP189	7.00E-27	-0.43059
rs11394645	3	186728353	LC_IGP189	2.75E-10	-0.261648
rs59111563	3	186722848	LC_IGP189	4.94E-27	-0.431412
rs148580052	3	186720957	LC_IGP190	5.39E-29	-0.449639
rs71634023	3	186712738	LC_IGP190	2.21E-24	-0.425667
rs201540659	3	186713306	LC_IGP190	4.69E-09	-0.288723
rs35456268	3	186721290	LC_IGP190	5.12E-29	-0.449738
rs11394645	3	186728353	LC_IGP190	1.18E-12	-0.29548
rs59111563	3	186722848	LC_IGP190	4.51E-29	-0.449809
rs200317857	3	186713293	LC_IGP190	1.49E-10	-0.261465
rs202164259	3	186711347	LC_IGP190	5.14E-09	-0.301112
rs199533504	3	186713310	LC_IGP190	7.63E-14	-0.288302
rs151183997	16	31376341	LC_IGP191	1.44E-11	0.263281
rs10574650	16	31342116	LC_IGP191	6.41E-11	0.238282
rs145068357	16	31377244	LC_IGP191	6.65E-11	0.255533
rs10574650	16	31342116	LC_IGP192	5.78E-09	0.212313
rs2050188	6	32339897	LC_IGP192	2.94E-08	0.196934
rs151183997	16	31376341	LC_IGP192	8.93E-10	0.238779
rs145068357	16	31377244	LC_IGP192	3.35E-09	0.231387
rs2050188	6	32339897	LC_IGP193	1.52E-08	0.201716
rs3129951	6	32358286	LC_IGP193	4.16E-08	0.198824
rs3117098	6	32358513	LC_IGP193	4.89E-08	0.197739
rs145068357	16	31377244	LC_IGP193	2.30E-09	0.234427
rs10574650	16	31342116	LC_IGP193	3.31E-09	0.216303
rs151183997	16	31376341	LC_IGP193	6.14E-10	0.24175
rs151183997	16	31376341	LC_IGP194	2.97E-09	0.230213
rs145068357	16	31377244	LC_IGP194	9.84E-09	0.223367
rs10574650	16	31342116	LC_IGP194	3.25E-09	0.214757
rs145068357	16	31377244	LC_IGP195	1.70E-10	0.250386

rs10574650	16	31342116	LC_IGP195	4.97E-10	0.227283
rs151183997	16	31376341	LC_IGP195	4.83E-11	0.256778
rs151183997	16	31376341	LC_IGP196	2.95E-11	0.259575
rs145068357	16	31377244	LC_IGP196	1.00E-10	0.253473
rs10574650	16	31342116	LC_IGP196	2.46E-10	0.23122
rs199739333	22	39777821	LC_IGP197	4.42E-10	0.228897
rs146492780	6	32591008	LC_IGP198	2.56E-08	0.338917
rs200755517	6	32590033	LC_IGP198	2.56E-08	0.338925
rs143645736	6	32594132	LC_IGP198	2.58E-08	0.338842
rs200675822	6	32619006	LC_IGP198	3.69E-08	0.321684
rs113273341	6	32602495	LC_IGP198	2.56E-08	0.338918
rs200778353	6	32599462	LC_IGP198	2.56E-08	0.338924
esv2667279	6	32600932	LC_IGP198	2.45E-08	0.340396
rs34645399	6	32589169	LC_IGP198	3.88E-08	0.334924
rs139474416	6	32620609	LC_IGP198	3.31E-08	0.322619
rs150830913	6	32607756	LC_IGP198	2.64E-08	0.338973
rs201675008	9	33120203	LC_IGP199	1.08E-08	-0.237359
rs199775279	1	25101702	LC_IGP199	2.08E-08	-0.954561
rs150025803	1	25024202	LC_IGP199	3.02E-10	-1.03025
rs143916866	1	24967537	LC_IGP199	3.81E-08	-0.83654
rs199517178	9	33152342	LC_IGP199	2.71E-08	-0.229788
rs3837267	9	33150971	LC_IGP199	1.95E-08	-0.218186
rs34443059	14	66225456	LC_IGP2	4.43E-08	0.188252
rs199739333	22	39777821	LC_IGP200	8.34E-16	-0.294306
rs201643791	22	39743579	LC_IGP200	6.20E-09	-0.218125
rs199739333	22	39777821	LC_IGP201	1.31E-21	-0.345855
rs201643791	22	39743579	LC_IGP201	8.45E-09	-0.215013
rs113831691	2	217985392	LC_IGP202	7.56E-09	-1.01243
rs150025803	1	25024202	LC_IGP205	1.18E-08	-0.935236
rs201675008	9	33120203	LC_IGP205	7.70E-09	-0.2401
rs199517178	9	33152342	LC_IGP205	1.61E-08	-0.233852
rs3837267	9	33150971	LC_IGP205	1.33E-08	-0.221069
rs35439429	14	66063919	LC_IGP21	2.44E-11	0.237127
rs201067714	14	65772934	LC_IGP21	3.35E-09	-0.27503
rs5809288	14	66237586	LC_IGP21	1.04E-12	0.24644
rs10572775	14	66055427	LC_IGP21	1.59E-14	0.265363
rs116315577	4	104183168	LC_IGP21	3.67E-09	1.14454
rs200183327	14	65980373	LC_IGP21	1.04E-08	0.209832
rs33927018	14	66029423	LC_IGP21	1.13E-08	0.209092
rs34443059	14	66225456	LC_IGP21	3.02E-14	0.262502
rs199534978	14	66076028	LC_IGP21	2.37E-15	0.271661
rs201678871	14	66232956	LC_IGP21	2.85E-12	0.240714
rs11284050	14	65884030	LC_IGP21	1.10E-08	0.209559
rs59312400	14	66249255	LC_IGP21	8.77E-12	-0.270072
rs200274176	14	66069056	LC_IGP21	7.20E-12	0.242371
rs72090965	14	65829951	LC_IGP21	1.10E-13	0.259687
rs3216656	14	66113327	LC_IGP21	2.35E-15	0.272638
rs71126788	14	66232958	LC_IGP21	2.85E-12	0.240713
rs11414976	14	65781578	LC_IGP21	6.75E-10	-0.346959
rs147422602	14	65990143	LC_IGP21	1.20E-15	0.275965

rs34263575	14	66160754	LC_IGP21	3.70E-15	0.270921
rs59850638	14	65772935	LC_IGP21	3.34E-09	-0.274844
rs142437037	14	65986959	LC_IGP21	1.46E-08	0.208382
rs71126773	14	66103040	LC_IGP21	4.10E-15	0.270094
rs34746444	14	66195404	LC_IGP21	7.44E-13	0.252177
rs34307226	14	65844447	LC_IGP21	3.03E-13	-0.280161
rs144508295	14	65811418	LC_IGP21	1.70E-10	0.229836
rs200160352	14	66264400	LC_IGP21	1.96E-09	-0.229682
rs139022691	14	65778230	LC_IGP21	4.19E-10	0.282523
rs61602020	14	65774613	LC_IGP21	4.51E-10	-0.283988
rs201623089	1	25297520	LC_IGP22	2.34E-09	0.709255
rs199739333	22	39777821	LC_IGP22	4.20E-15	-0.288404
rs201028197	22	24142976	LC_IGP22	4.04E-09	0.236704
rs199766327	22	24142978	LC_IGP22	6.37E-09	0.233768
rs200345855	9	33118502	LC_IGP23	5.77E-10	0.880253
rs111399879	9	33166711	LC_IGP23	1.43E-10	0.868352
rs200317857	3	186713293	LC_IGP25	3.00E-12	-0.285407
rs201540659	3	186713306	LC_IGP25	1.35E-09	-0.29923
rs59111563	3	186722848	LC_IGP25	6.20E-23	-0.401466
rs71634023	3	186712738	LC_IGP25	3.31E-19	-0.378766
rs35456268	3	186721290	LC_IGP25	8.32E-23	-0.400585
rs199533504	3	186713310	LC_IGP25	2.68E-11	-0.259083
rs11394645	3	186728353	LC_IGP25	1.40E-09	-0.253367
rs148580052	3	186720957	LC_IGP25	8.91E-23	-0.40037
rs61210528	3	186742220	LC_IGP25	1.52E-09	-0.295555
rs10572775	14	66055427	LC_IGP26	3.08E-08	0.191271
rs147422602	14	65990143	LC_IGP26	2.36E-08	0.192607
rs3216656	14	66113327	LC_IGP26	3.80E-08	0.189395
rs199534978	14	66076028	LC_IGP26	4.46E-08	0.187789
rs3216656	14	66113327	LC_IGP27	1.94E-09	0.206275
rs34746444	14	66195404	LC_IGP27	1.16E-08	0.200241
rs10572775	14	66055427	LC_IGP27	1.79E-09	0.207412
rs34263575	14	66160754	LC_IGP27	4.11E-09	0.202239
rs71126773	14	66103040	LC_IGP27	9.87E-09	0.196964
rs199534978	14	66076028	LC_IGP27	2.24E-09	0.2048
rs34443059	14	66225456	LC_IGP27	1.80E-08	0.194186
rs147422602	14	65990143	LC_IGP27	1.01E-09	0.210274
rs146378727	17	38073837	LC_IGP28	3.28E-08	0.195763
rs201389301	17	38075009	LC_IGP28	2.39E-08	0.192702
rs10572775	14	66055427	LC_IGP28	1.93E-10	0.219095
rs59458272	17	38020058	LC_IGP28	1.14E-08	-0.197838
rs34746444	14	66195404	LC_IGP28	1.40E-08	0.198918
rs147422602	14	65990143	LC_IGP28	9.32E-11	0.222567
rs36084703	17	38063980	LC_IGP28	1.19E-08	-0.198265
rs200306858	17	38025643	LC_IGP28	1.92E-08	-0.195982
rs71126788	14	66232958	LC_IGP28	1.87E-08	0.192858
rs201678871	14	66232956	LC_IGP28	1.87E-08	0.19286
rs5809288	14	66237586	LC_IGP28	2.25E-08	0.192534
rs34233420	17	38004929	LC_IGP28	1.37E-08	-0.197248
rs34307226	14	65844447	LC_IGP28	1.71E-08	-0.216036

rs34263575	14	66160754	LC_IGP28	5.43E-10	0.213086
rs72538185	17	37916390	LC_IGP28	1.52E-08	0.197011
rs3216656	14	66113327	LC_IGP28	2.12E-10	0.217905
rs71971950	17	38076198	LC_IGP28	3.65E-08	0.191339
rs34443059	14	66225456	LC_IGP28	1.11E-09	0.209723
rs113897057	17	37975214	LC_IGP28	1.17E-08	-0.198056
rs71126773	14	66103040	LC_IGP28	6.37E-10	0.211922
rs148094956	17	38039561	LC_IGP28	9.89E-09	-0.199753
rs200216139	17	38032132	LC_IGP28	1.10E-08	-0.198571
rs199534978	14	66076028	LC_IGP28	9.15E-11	0.221471
rs72090965	14	65829951	LC_IGP28	1.80E-10	0.222326
rs35456268	3	186721290	LC_IGP29	3.04E-08	-0.226672
rs59111563	3	186722848	LC_IGP29	3.07E-08	-0.226419
rs148580052	3	186720957	LC_IGP29	3.03E-08	-0.226733
rs200345855	9	33118502	LC_IGP3	3.62E-10	0.888241
rs111399879	9	33166711	LC_IGP3	1.64E-10	0.863408
rs147422602	14	65990143	LC_IGP31	2.72E-08	0.192193
rs34307226	14	65844447	LC_IGP31	2.30E-08	-0.214533
rs34263575	14	66160754	LC_IGP32	2.62E-08	0.191471
rs3216656	14	66113327	LC_IGP32	2.16E-08	0.192457
rs71126773	14	66103040	LC_IGP32	2.99E-08	0.190404
rs199534978	14	66076028	LC_IGP32	1.68E-08	0.193353
rs147422602	14	65990143	LC_IGP32	7.05E-09	0.199374
rs35456268	3	186721290	LC_IGP34	1.13E-17	-0.34579
rs71634023	3	186712738	LC_IGP34	1.76E-14	-0.321298
rs199533504	3	186713310	LC_IGP34	1.09E-08	-0.220499
rs59111563	3	186722848	LC_IGP34	9.61E-18	-0.346239
rs148580052	3	186720957	LC_IGP34	1.17E-17	-0.345673
rs200317857	3	186713293	LC_IGP34	3.40E-10	-0.255561
rs201540659	3	186713306	LC_IGP36	1.13E-11	-0.330876
rs200317857	3	186713293	LC_IGP36	1.36E-10	-0.259572
rs61210528	3	186742220	LC_IGP36	1.56E-10	-0.309349
rs35456268	3	186721290	LC_IGP36	1.72E-27	-0.433109
rs199533504	3	186713310	LC_IGP36	5.29E-13	-0.275831
rs11394645	3	186728353	LC_IGP36	8.13E-14	-0.307149
rs148580052	3	186720957	LC_IGP36	1.92E-27	-0.432799
rs71634023	3	186712738	LC_IGP36	1.50E-21	-0.395107
rs59111563	3	186722848	LC_IGP36	1.14E-27	-0.434199
rs201540659	3	186713306	LC_IGP37	3.52E-15	-0.385292
rs35456268	3	186721290	LC_IGP37	1.23E-39	-0.524535
rs199533504	3	186713310	LC_IGP37	1.40E-19	-0.346782
rs200317857	3	186713293	LC_IGP37	1.56E-18	-0.355726
rs148580052	3	186720957	LC_IGP37	1.37E-39	-0.524305
rs202164259	3	186711347	LC_IGP37	1.32E-12	-0.363488
rs61210528	3	186742220	LC_IGP37	5.19E-15	-0.379504
rs71634023	3	186712738	LC_IGP37	2.06E-31	-0.483204
rs11394645	3	186728353	LC_IGP37	1.10E-17	-0.35367
rs59111563	3	186722848	LC_IGP37	8.65E-40	-0.525102
rs199739333	22	39777821	LC_IGP4	1.01E-13	-0.27162
rs111399879	9	33166711	LC_IGP4	2.40E-11	-0.902729

rs200345855	9	33118502	LC_IGP4	1.74E-10	-0.905039
rs147983069	11	65564858	LC_IGP4	3.64E-08	-0.314822
rs144509153	11	65564142	LC_IGP4	3.48E-08	-0.315197
rs71126773	14	66103040	LC_IGP47	3.17E-08	0.191309
rs34263575	14	66160754	LC_IGP47	4.43E-08	0.189572
rs10572775	14	66055427	LC_IGP47	4.34E-08	0.190185
rs147422602	14	65990143	LC_IGP47	1.22E-08	0.197489
rs3216656	14	66113327	LC_IGP47	2.80E-08	0.1922
rs34443059	14	66225456	LC_IGP47	1.84E-08	0.195184
rs200345855	9	33118502	LC_IGP48	7.94E-10	0.87045
rs111399879	9	33166711	LC_IGP48	3.56E-10	0.847014
rs111399879	9	33166711	LC_IGP49	1.72E-11	-0.908738
rs200345855	9	33118502	LC_IGP49	1.48E-10	-0.907869
rs199739333	22	39777821	LC_IGP49	2.07E-14	-0.281422
rs201623089	1	25297520	LC_IGP5	1.48E-08	0.666933
rs199739333	22	39777821	LC_IGP5	8.43E-14	-0.272237
rs35184810	8	69265871	LC_IGP50	3.25E-08	-0.294703
rs199739333	22	39777821	LC_IGP50	1.31E-12	-0.26109
rs11414976	14	65781578	LC_IGP52	5.04E-12	0.390084
rs139022691	14	65778230	LC_IGP52	1.69E-09	-0.27332
rs3216656	14	66113327	LC_IGP52	1.71E-19	-0.309928
rs33927018	14	66029423	LC_IGP52	6.43E-12	-0.251522
rs59458272	17	38020058	LC_IGP52	1.38E-08	0.197758
rs200183327	14	65980373	LC_IGP52	5.38E-12	-0.25272
rs60977949	14	65737104	LC_IGP52	1.78E-08	0.221872
rs201067714	14	65772934	LC_IGP52	1.92E-08	0.261612
rs34742130	14	65869387	LC_IGP52	7.79E-11	-0.238327
rs59318404	14	65803010	LC_IGP52	1.98E-08	0.372563
rs200216139	17	38032132	LC_IGP52	3.92E-08	0.192048
rs72090965	14	65829951	LC_IGP52	1.36E-15	-0.278771
rs10572775	14	66055427	LC_IGP52	2.20E-18	-0.301416
rs59312400	14	66249255	LC_IGP52	3.78E-15	0.309972
rs200019676	17	38073841	LC_IGP52	3.94E-08	-0.195801
rs36084703	17	38063980	LC_IGP52	3.97E-08	0.191963
rs148094956	17	38039561	LC_IGP52	4.48E-08	0.191641
rs34233420	17	38004929	LC_IGP52	2.47E-08	0.194823
rs75635798	14	65809257	LC_IGP52	2.48E-08	0.369159
rs200160352	14	66264400	LC_IGP52	8.02E-09	0.219755
rs112066572	14	65812365	LC_IGP52	3.86E-10	0.268879
rs59850638	14	65772935	LC_IGP52	2.25E-08	0.26014
rs72416175	14	66116826	LC_IGP52	5.59E-09	0.264021
rs57476621	14	66122725	LC_IGP52	2.54E-09	-0.216333
rs142437037	14	65986959	LC_IGP52	8.05E-12	-0.251596
rs34307226	14	65844447	LC_IGP52	1.26E-18	0.336717
rs61602020	14	65774613	LC_IGP52	2.14E-09	0.273532
rs5809288	14	66237586	LC_IGP52	1.75E-14	-0.264475
rs113897057	17	37975214	LC_IGP52	1.07E-08	0.199669
rs200274176	14	66069056	LC_IGP52	1.95E-15	-0.280655
rs202063544	14	65803008	LC_IGP52	1.98E-08	0.372562
rs34746444	14	66195404	LC_IGP52	2.09E-18	-0.307186

rs71126773	14	66103040	LC_IGP52	9.25E-19	-0.303455
rs199534978	14	66076028	LC_IGP52	1.78E-19	-0.308763
rs201712507	14	65856090	LC_IGP52	6.01E-10	0.268179
rs146378727	17	38073837	LC_IGP52	2.36E-08	-0.198645
rs34785787	14	65878211	LC_IGP52	7.67E-11	-0.236715
rs35402762	14	65984685	LC_IGP52	4.34E-11	-0.239572
rs201678871	14	66232956	LC_IGP52	2.60E-14	-0.261731
rs5809277	14	65968613	LC_IGP52	6.74E-11	-0.237442
rs34263575	14	66160754	LC_IGP52	3.30E-19	-0.307729
rs145473242	14	65826211	LC_IGP52	2.42E-10	0.272496
rs71126788	14	66232958	LC_IGP52	2.60E-14	-0.261728
rs34443059	14	66225456	LC_IGP52	1.85E-17	-0.292988
rs35439429	14	66063919	LC_IGP52	4.22E-14	-0.268086
rs11284050	14	65884030	LC_IGP52	2.91E-12	-0.255893
rs144508295	14	65811418	LC_IGP52	2.82E-13	-0.262391
rs147422602	14	65990143	LC_IGP52	7.79E-20	-0.313395
rs200274176	14	66069056	LC_IGP53	1.62E-11	-0.239344
rs142437037	14	65986959	LC_IGP53	1.36E-08	-0.210018
rs34443059	14	66225456	LC_IGP53	4.95E-14	-0.260946
rs5809288	14	66237586	LC_IGP53	3.47E-12	-0.241055
rs34263575	14	66160754	LC_IGP53	2.78E-15	-0.272928
rs59850638	14	65772935	LC_IGP53	3.34E-08	0.257705
rs139022691	14	65778230	LC_IGP53	4.17E-09	-0.267455
rs71126788	14	66232958	LC_IGP53	5.12E-12	-0.238246
rs201678871	14	66232956	LC_IGP53	5.12E-12	-0.238248
rs35439429	14	66063919	LC_IGP53	2.46E-11	-0.238067
rs33927018	14	66029423	LC_IGP53	8.86E-09	-0.211636
rs201067714	14	65772934	LC_IGP53	2.35E-08	0.260739
rs72090965	14	65829951	LC_IGP53	1.67E-14	-0.268757
rs34307226	14	65844447	LC_IGP53	3.93E-13	0.279295
rs71126773	14	66103040	LC_IGP53	2.93E-15	-0.272302
rs59312400	14	66249255	LC_IGP53	2.77E-11	0.263986
rs11284050	14	65884030	LC_IGP53	1.02E-08	-0.211047
rs200183327	14	65980373	LC_IGP53	8.19E-09	-0.212361
rs10572775	14	66055427	LC_IGP53	8.08E-15	-0.269076
rs3216656	14	66113327	LC_IGP53	1.71E-15	-0.274771
rs144508295	14	65811418	LC_IGP53	2.10E-11	-0.2417
rs200160352	14	66264400	LC_IGP53	4.10E-08	0.209727
rs11414976	14	65781578	LC_IGP53	1.46E-08	0.321819
rs199534978	14	66076028	LC_IGP53	1.62E-15	-0.274091
rs147422602	14	65990143	LC_IGP53	1.17E-15	-0.276895
rs34746444	14	66195404	LC_IGP53	7.41E-13	-0.253415
rs61602020	14	65774613	LC_IGP53	4.47E-09	0.268832
rs5809288	14	66237586	LC_IGP55	1.30E-14	-0.266504
rs144508295	14	65811418	LC_IGP55	1.05E-11	-0.245247
rs11284050	14	65884030	LC_IGP55	1.65E-09	-0.22201
rs72090965	14	65829951	LC_IGP55	7.21E-14	-0.262195
rs200183327	14	65980373	LC_IGP55	4.06E-09	-0.216614
rs11414976	14	65781578	LC_IGP55	3.91E-09	0.334242
rs33927018	14	66029423	LC_IGP55	5.85E-09	-0.214152

rs201678871	14	66232956	LC_IGP55	3.41E-14	-0.261275
rs10572775	14	66055427	LC_IGP55	4.99E-16	-0.280783
rs34443059	14	66225456	LC_IGP55	1.26E-16	-0.286174
rs71126773	14	66103040	LC_IGP55	7.52E-17	-0.287297
rs34746444	14	66195404	LC_IGP55	1.99E-14	-0.27003
rs59312400	14	66249255	LC_IGP55	6.71E-12	0.271982
rs140541167	8	13167855	LC_IGP55	4.13E-08	-0.416083
rs200274176	14	66069056	LC_IGP55	2.76E-12	-0.248157
rs199534978	14	66076028	LC_IGP55	1.89E-16	-0.282867
rs144170874	8	13159890	LC_IGP55	3.76E-08	-0.414525
rs142437037	14	65986959	LC_IGP55	5.03E-10	-0.229725
rs71126788	14	66232958	LC_IGP55	3.41E-14	-0.261272
rs60977949	14	65737104	LC_IGP55	1.03E-08	0.226119
rs3216656	14	66113327	LC_IGP55	1.62E-17	-0.29358
rs35439429	14	66063919	LC_IGP55	2.39E-11	-0.238193
rs139022691	14	65778230	LC_IGP55	5.33E-09	-0.265589
rs34307226	14	65844447	LC_IGP55	1.49E-13	0.284194
rs34263575	14	66160754	LC_IGP55	2.58E-17	-0.29196
rs147422602	14	65990143	LC_IGP55	6.74E-18	-0.297549
rs61602020	14	65774613	LC_IGP55	6.13E-09	0.266408
rs34746444	14	66195404	LC_IGP56	4.08E-10	-0.221642
rs3216656	14	66113327	LC_IGP56	5.89E-12	-0.238507
rs34443059	14	66225456	LC_IGP56	3.16E-11	-0.23077
rs71126773	14	66103040	LC_IGP56	7.17E-12	-0.23737
rs34263575	14	66160754	LC_IGP56	1.28E-11	-0.234866
rs34307226	14	65844447	LC_IGP56	3.87E-10	0.241734
rs5809288	14	66237586	LC_IGP56	2.91E-10	-0.21904
rs71126788	14	66232958	LC_IGP56	6.18E-10	-0.21415
rs59312400	14	66249255	LC_IGP56	8.27E-09	0.229225
rs201678871	14	66232956	LC_IGP56	6.18E-10	-0.214151
rs10572775	14	66055427	LC_IGP56	3.86E-11	-0.229932
rs200274176	14	66069056	LC_IGP56	1.70E-08	-0.201118
rs144508295	14	65811418	LC_IGP56	1.50E-09	-0.218685
rs35439429	14	66063919	LC_IGP56	4.29E-08	-0.196112
rs72090965	14	65829951	LC_IGP56	4.31E-11	-0.231805
rs147422602	14	65990143	LC_IGP56	2.61E-12	-0.242893
rs199534978	14	66076028	LC_IGP56	1.70E-11	-0.232528
rs111399879	9	33166711	LC_IGP58	1.39E-11	-0.914805
rs200345855	9	33118502	LC_IGP58	6.83E-11	-0.926367
rs111399879	9	33166711	LC_IGP59	5.75E-09	0.789512
rs200345855	9	33118502	LC_IGP59	2.09E-08	0.796861
rs111399879	9	33166711	LC_IGP60	9.38E-10	0.827984
rs200345855	9	33118502	LC_IGP60	2.93E-09	0.842328
rs200183327	14	65980373	LC_IGP61	1.06E-09	0.224618
rs5809288	14	66237586	LC_IGP61	1.08E-13	0.257191
rs200274176	14	66069056	LC_IGP61	8.68E-13	0.253812
rs10572775	14	66055427	LC_IGP61	3.56E-16	0.282212
rs142437037	14	65986959	LC_IGP61	1.13E-09	0.225106
rs34443059	14	66225456	LC_IGP61	1.23E-15	0.276864
rs59312400	14	66249255	LC_IGP61	4.79E-13	-0.286364

rs139022691	14	65778230	LC_IGP61		3.10E-10	0.286197
rs201067714	14	65772934	LC_IGP61		2.00E-09	-0.279862
rs35439429	14	66063919	LC_IGP61		3.87E-12	0.247437
rs71126773	14	66103040	LC_IGP61		1.04E-16	0.286048
rs34746444	14	66195404	LC_IGP61		9.85E-15	0.273202
rs145473242	14	65826211	LC_IGP61		1.25E-08	-0.246
rs61602020	14	65774613	LC_IGP61		3.42E-10	-0.287542
rs33927018	14	66029423	LC_IGP61		1.23E-09	0.223495
rs201678871	14	66232956	LC_IGP61		2.11E-13	0.253188
rs72090965	14	65829951	LC_IGP61		1.69E-15	0.278666
rs3216656	14	66113327	LC_IGP61		4.42E-17	0.289684
rs5809277	14	65968613	LC_IGP61		2.05E-08	0.204971
rs34742130	14	65869387	LC_IGP61		1.48E-08	0.208451
rs11414976	14	65781578	LC_IGP61		6.25E-10	-0.350931
rs59850638	14	65772935	LC_IGP61		2.94E-09	-0.276768
rs34263575	14	66160754	LC_IGP61		7.34E-17	0.287864
rs71126788	14	66232958	LC_IGP61		2.11E-13	0.253186
rs147422602	14	65990143	LC_IGP61		2.69E-17	0.292205
rs34785787	14	65878211	LC_IGP61		1.62E-08	0.206444
rs34307226	14	65844447	LC_IGP61		5.37E-15	-0.300469
rs144508295	14	65811418	LC_IGP61		1.72E-12	0.254396
rs199534978	14	66076028	LC_IGP61		6.04E-17	0.28748
rs200160352	14	66264400	LC_IGP61		9.05E-09	-0.219631
rs201712507	14	65856090	LC_IGP61		2.47E-08	-0.242525
rs11284050	14	65884030	LC_IGP61		1.11E-09	0.224387
rs112066572	14	65812365	LC_IGP61		1.87E-08	-0.24257
rs35402762	14	65984685	LC_IGP61		1.43E-08	0.207022
rs142437037	14	65986959	LC_IGP62		2.07E-10	0.234262
rs34443059	14	66225456	LC_IGP62		3.70E-16	0.281207
rs72416175	14	66116826	LC_IGP62		2.45E-08	-0.252892
rs34307226	14	65844447	LC_IGP62		6.88E-17	-0.31974
rs200274176	14	66069056	LC_IGP62		8.05E-14	0.26437
rs10572775	14	66055427	LC_IGP62		1.32E-16	0.285619
rs200183327	14	65980373	LC_IGP62		2.28E-10	0.232853
rs201067714	14	65772934	LC_IGP62		1.67E-09	-0.280585
rs201678871	14	66232956	LC_IGP62		8.07E-14	0.256936
rs5809277	14	65968613	LC_IGP62		4.23E-09	0.214159
rs200160352	14	66264400	LC_IGP62		1.95E-08	-0.214173
rs34742130	14	65869387	LC_IGP62		4.12E-09	0.215832
rs59312400	14	66249255	LC_IGP62		3.64E-14	-0.299014
rs144508295	14	65811418	LC_IGP62		6.26E-13	0.25875
rs59850638	14	65772935	LC_IGP62		2.65E-09	-0.27692
rs199534978	14	66076028	LC_IGP62		1.66E-17	0.291903
rs61602020	14	65774613	LC_IGP62		2.21E-10	-0.289932
rs145473242	14	65826211	LC_IGP62		2.32E-09	-0.257446
rs72090965	14	65829951	LC_IGP62		3.33E-15	0.275163
rs11284050	14	65884030	LC_IGP62		1.52E-10	0.235142
rs147422602	14	65990143	LC_IGP62		5.35E-18	0.297823
rs35439429	14	66063919	LC_IGP62		2.38E-12	0.249275
rs201712507	14	65856090	LC_IGP62		5.29E-09	-0.253255

rs11414976	14	65781578	LC_IGP62	2.86E-11	-0.37639
rs139022691	14	65778230	LC_IGP62	1.57E-10	0.290253
rs33927018	14	66029423	LC_IGP62	2.74E-10	0.231549
rs5809288	14	66237586	LC_IGP62	4.49E-14	0.260544
rs71126773	14	66103040	LC_IGP62	2.47E-17	0.291101
rs35402762	14	65984685	LC_IGP62	2.62E-09	0.216793
rs112066572	14	65812365	LC_IGP62	2.85E-09	-0.255505
rs60977949	14	65737104	LC_IGP62	4.00E-08	-0.216494
rs71126788	14	66232958	LC_IGP62	8.07E-14	0.256934
rs34785787	14	65878211	LC_IGP62	3.25E-09	0.215713
rs34263575	14	66160754	LC_IGP62	1.22E-17	0.294274
rs3216656	14	66113327	LC_IGP62	8.72E-18	0.295376
rs34746444	14	66195404	LC_IGP62	2.91E-16	0.287635
rs201678871	14	66232956	LC_IGP63	7.23E-14	0.258183
rs59850638	14	65772935	LC_IGP63	9.04E-09	-0.268319
rs10572775	14	66055427	LC_IGP63	4.03E-17	0.291203
rs199534978	14	66076028	LC_IGP63	1.97E-17	0.292093
rs200274176	14	66069056	LC_IGP63	8.78E-13	0.253938
rs33927018	14	66029423	LC_IGP63	1.45E-09	0.222673
rs200183327	14	65980373	LC_IGP63	1.24E-09	0.22383
rs34443059	14	66225456	LC_IGP63	2.66E-16	0.283396
rs5809288	14	66237586	LC_IGP63	4.19E-14	0.261615
rs34263575	14	66160754	LC_IGP63	1.42E-17	0.294556
rs59312400	14	66249255	LC_IGP63	6.73E-12	-0.272202
rs35402762	14	65984685	LC_IGP63	1.55E-08	0.206671
rs71126788	14	66232958	LC_IGP63	7.23E-14	0.258182
rs72090965	14	65829951	LC_IGP63	4.29E-16	0.28463
rs144508295	14	65811418	LC_IGP63	1.47E-12	0.255348
rs142437037	14	65986959	LC_IGP63	8.76E-10	0.226725
rs201067714	14	65772934	LC_IGP63	7.89E-09	-0.269585
rs139022691	14	65778230	LC_IGP63	1.43E-09	0.275536
rs60977949	14	65737104	LC_IGP63	4.64E-08	-0.216112
rs3216656	14	66113327	LC_IGP63	6.57E-18	0.29734
rs71126773	14	66103040	LC_IGP63	1.48E-17	0.293976
rs35439429	14	66063919	LC_IGP63	2.04E-12	0.250758
rs11284050	14	65884030	LC_IGP63	1.37E-09	0.223301
rs34742130	14	65869387	LC_IGP63	1.44E-08	0.208796
rs34307226	14	65844447	LC_IGP63	7.05E-14	-0.288179
rs200160352	14	66264400	LC_IGP63	6.31E-09	-0.222082
rs11414976	14	65781578	LC_IGP63	3.87E-09	-0.334646
rs34746444	14	66195404	LC_IGP63	2.42E-14	0.269394
rs34785787	14	65878211	LC_IGP63	1.94E-08	0.205452
rs147422602	14	65990143	LC_IGP63	3.27E-18	0.300591
rs61602020	14	65774613	LC_IGP63	1.43E-09	-0.277504
rs5809277	14	65968613	LC_IGP63	2.23E-08	0.204592
rs10572775	14	66055427	LC_IGP64	1.69E-13	0.256095
rs199534978	14	66076028	LC_IGP64	2.97E-14	0.262142
rs34443059	14	66225456	LC_IGP64	3.04E-13	0.253212
rs34263575	14	66160754	LC_IGP64	7.14E-14	0.259313
rs201067714	14	65772934	LC_IGP64	4.13E-08	-0.256635

rs59850638	14	65772935	LC_IGP64	4.68E-08	-0.255417
rs5809288	14	66237586	LC_IGP64	9.12E-11	0.225179
rs200274176	14	66069056	LC_IGP64	3.51E-10	0.223525
rs35439429	14	66063919	LC_IGP64	6.76E-10	0.220704
rs139022691	14	65778230	LC_IGP64	2.31E-08	0.254849
rs147422602	14	65990143	LC_IGP64	2.12E-14	0.265008
rs144508295	14	65811418	LC_IGP64	5.16E-10	0.22484
rs71126788	14	66232958	LC_IGP64	1.38E-10	0.222152
rs3216656	14	66113327	LC_IGP64	3.50E-14	0.262298
rs34746444	14	66195404	LC_IGP64	1.81E-12	0.249583
rs59312400	14	66249255	LC_IGP64	4.32E-11	-0.261888
rs201678871	14	66232956	LC_IGP64	1.38E-10	0.222153
rs11414976	14	65781578	LC_IGP64	2.19E-08	-0.318462
rs72090965	14	65829951	LC_IGP64	7.08E-13	0.252125
rs61602020	14	65774613	LC_IGP64	1.85E-08	-0.258405
rs71126773	14	66103040	LC_IGP64	1.32E-13	0.25616
rs34307226	14	65844447	LC_IGP64	2.21E-12	-0.270782
rs34443059	14	66225456	LC_IGP65	5.48E-10	0.214899
rs34307226	14	65844447	LC_IGP65	4.47E-08	-0.210656
rs34746444	14	66195404	LC_IGP65	7.54E-09	0.204147
rs34263575	14	66160754	LC_IGP65	1.16E-09	0.210434
rs3216656	14	66113327	LC_IGP65	8.88E-10	0.21175
rs199534978	14	66076028	LC_IGP65	1.15E-09	0.209651
rs71126773	14	66103040	LC_IGP65	5.67E-10	0.21401
rs10572775	14	66055427	LC_IGP65	6.19E-09	0.201578
rs201678871	14	66232956	LC_IGP65	5.87E-10	0.21347
rs72090965	14	65829951	LC_IGP65	1.03E-09	0.213892
rs5809288	14	66237586	LC_IGP65	4.47E-10	0.215767
rs147422602	14	65990143	LC_IGP65	4.00E-10	0.216448
rs71126788	14	66232958	LC_IGP65	5.87E-10	0.213471
rs144508295	14	65811418	LC_IGP65	2.02E-08	0.202244
rs13397293	2	226446404	LC_IGP65	5.00E-09	0.342785
rs28539402	2	226442743	LC_IGP65	7.21E-09	0.340685
rs34263575	14	66160754	LC_IGP66	4.30E-09	0.202858
rs72090965	14	65829951	LC_IGP66	4.08E-09	0.20585
rs71126788	14	66232958	LC_IGP66	1.02E-09	0.210188
rs34746444	14	66195404	LC_IGP66	1.43E-08	0.200094
rs71126773	14	66103040	LC_IGP66	2.86E-09	0.204854
rs13397293	2	226446404	LC_IGP66	3.44E-08	0.323198
rs199739333	22	39777821	LC_IGP66	2.70E-08	0.204833
rs144508295	14	65811418	LC_IGP66	2.82E-08	0.199909
rs199534978	14	66076028	LC_IGP66	7.09E-09	0.199206
rs34443059	14	66225456	LC_IGP66	2.56E-09	0.206149
rs5809288	14	66237586	LC_IGP66	7.94E-10	0.212387
rs10572775	14	66055427	LC_IGP66	3.20E-08	0.191664
rs147422602	14	65990143	LC_IGP66	2.29E-09	0.206659
rs201678871	14	66232956	LC_IGP66	1.02E-09	0.210187
rs3216656	14	66113327	LC_IGP66	4.42E-09	0.202571
rs28539402	2	226442743	LC_IGP66	4.69E-08	0.321389
rs71126773	14	66103040	LC_IGP67	1.62E-08	0.195171

rs3216656	14	66113327	LC_IGP67	2.53E-08	0.192677
rs5809288	14	66237586	LC_IGP67	1.91E-08	0.194639
rs34443059	14	66225456	LC_IGP67	2.17E-08	0.194064
rs34263575	14	66160754	LC_IGP67	4.34E-08	0.189558
rs72090965	14	65829951	LC_IGP67	2.81E-08	0.194713
rs71126788	14	66232958	LC_IGP67	2.51E-08	0.192246
rs201678871	14	66232956	LC_IGP67	2.51E-08	0.192244
rs199534978	14	66076028	LC_IGP67	3.58E-08	0.18996
rs147422602	14	65990143	LC_IGP67	1.27E-08	0.197126
rs5809288	14	66237586	LC_IGP68	6.34E-09	0.201979
rs72090965	14	65829951	LC_IGP68	6.68E-10	0.217226
rs34746444	14	66195404	LC_IGP68	1.58E-09	0.214086
rs34263575	14	66160754	LC_IGP68	2.30E-10	0.220098
rs71126773	14	66103040	LC_IGP68	1.55E-10	0.221857
rs59312400	14	66249255	LC_IGP68	2.75E-08	-0.221089
rs147422602	14	65990143	LC_IGP68	6.73E-11	0.226769
rs34307226	14	65844447	LC_IGP68	2.14E-09	-0.23133
rs10572775	14	66055427	LC_IGP68	6.83E-10	0.214825
rs71126788	14	66232958	LC_IGP68	7.63E-09	0.200124
rs3216656	14	66113327	LC_IGP68	1.23E-10	0.223234
rs201678871	14	66232956	LC_IGP68	7.63E-09	0.200124
rs199534978	14	66076028	LC_IGP68	1.44E-10	0.221655
rs34443059	14	66225456	LC_IGP68	1.97E-10	0.221319
rs144508295	14	65811418	LC_IGP68	2.60E-08	0.201562
rs201623089	1	25297520	LC_IGP69	3.37E-09	0.697662
rs199766327	22	24142978	LC_IGP69	4.12E-08	0.219561
rs199739333	22	39777821	LC_IGP69	4.63E-20	-0.334887
rs201028197	22	24142976	LC_IGP69	2.89E-08	0.221946
rs61210528	3	186742220	LC_IGP7	8.89E-10	-0.296397
rs35456268	3	186721290	LC_IGP7	7.16E-21	-0.375373
rs200317857	3	186713293	LC_IGP7	2.04E-10	-0.257124
rs199533504	3	186713310	LC_IGP7	6.90E-12	-0.262382
rs71634023	3	186712738	LC_IGP7	4.98E-17	-0.348662
rs201540659	3	186713306	LC_IGP7	1.89E-10	-0.310761
rs59111563	3	186722848	LC_IGP7	6.19E-21	-0.375663
rs11394645	3	186728353	LC_IGP7	2.67E-12	-0.287975
rs148580052	3	186720957	LC_IGP7	7.42E-21	-0.375286
rs199739333	22	39777821	LC_IGP70	8.24E-28	-0.39729
rs201643791	22	39743579	LC_IGP70	4.39E-12	-0.259877
rs142347605	22	39964098	LC_IGP70	4.17E-08	0.234463
rs201623089	1	25297520	LC_IGP71	4.45E-10	0.735222
rs199739333	22	39777821	LC_IGP71	7.43E-14	-0.274167
rs186127900	1	25318225	LC_IGP71	4.11E-08	0.974218
rs150110347	1	25308762	LC_IGP71	1.38E-08	1.01686
rs142858044	2	89428859	LC_IGP72	2.59E-08	0.673602
rs199766327	22	24142978	LC_IGP73	2.24E-08	0.223565
rs201623089	1	25297520	LC_IGP73	5.55E-09	0.687255
rs199739333	22	39777821	LC_IGP73	7.86E-16	-0.294692
rs201028197	22	24142976	LC_IGP73	1.48E-08	0.226317
rs201643791	22	39743579	LC_IGP74	4.23E-09	-0.220674

rs199739333	22	39777821	LC_IGP74	9.98E-22	-0.349138
rs201623089	1	25297520	LC_IGP75	3.67E-10	0.738348
rs199739333	22	39777821	LC_IGP75	6.01E-11	-0.240202
rs150110347	1	25308762	LC_IGP75	1.89E-08	1.00672
rs34746444	14	66195404	LC_IGP77	1.75E-11	-0.238246
rs3216656	14	66113327	LC_IGP77	2.55E-13	-0.253306
rs5809288	14	66237586	LC_IGP77	9.75E-12	-0.236397
rs144508295	14	65811418	LC_IGP77	3.72E-10	-0.226604
rs142437037	14	65986959	LC_IGP77	4.12E-08	-0.203267
rs34307226	14	65844447	LC_IGP77	3.34E-11	0.25589
rs34263575	14	66160754	LC_IGP77	4.15E-13	-0.251246
rs72090965	14	65829951	LC_IGP77	6.36E-12	-0.241454
rs71126773	14	66103040	LC_IGP77	4.80E-13	-0.250214
rs199534978	14	66076028	LC_IGP77	1.06E-12	-0.245908
rs10572775	14	66055427	LC_IGP77	2.55E-12	-0.243338
rs35439429	14	66063919	LC_IGP77	4.02E-09	-0.210455
rs34443059	14	66225456	LC_IGP77	7.55E-13	-0.248922
rs59312400	14	66249255	LC_IGP77	4.91E-10	0.247287
rs71126788	14	66232958	LC_IGP77	2.52E-11	-0.230767
rs147422602	14	65990143	LC_IGP77	1.19E-13	-0.257258
rs200274176	14	66069056	LC_IGP77	1.23E-09	-0.216501
rs201678871	14	66232956	LC_IGP77	2.52E-11	-0.230768
rs147422602	14	65990143	LC_IGP78	1.61E-14	-0.265467
rs34746444	14	66195404	LC_IGP78	7.50E-12	-0.241903
rs61602020	14	65774613	LC_IGP78	1.33E-08	0.260264
rs34263575	14	66160754	LC_IGP78	3.10E-14	-0.262278
rs59312400	14	66249255	LC_IGP78	1.57E-10	0.253572
rs71126773	14	66103040	LC_IGP78	6.31E-14	-0.258763
rs201678871	14	66232956	LC_IGP78	6.28E-12	-0.237026
rs34307226	14	65844447	LC_IGP78	7.15E-12	0.263742
rs200274176	14	66069056	LC_IGP78	4.96E-10	-0.221002
rs142437037	14	65986959	LC_IGP78	2.68E-08	-0.205516
rs199534978	14	66076028	LC_IGP78	2.09E-13	-0.252739
rs5809288	14	66237586	LC_IGP78	2.66E-12	-0.242098
rs11414976	14	65781578	LC_IGP78	3.93E-08	0.311825
rs3216656	14	66113327	LC_IGP78	2.56E-14	-0.262939
rs34443059	14	66225456	LC_IGP78	1.25E-13	-0.256561
rs144508295	14	65811418	LC_IGP78	8.55E-11	-0.234058
rs71126788	14	66232958	LC_IGP78	6.28E-12	-0.237025
rs72090965	14	65829951	LC_IGP78	2.58E-12	-0.245247
rs139022691	14	65778230	LC_IGP78	1.07E-08	-0.260054
rs35439429	14	66063919	LC_IGP78	4.57E-09	-0.209181
rs10572775	14	66055427	LC_IGP78	5.37E-13	-0.250076
rs3216656	14	66113327	LC_IGP79	2.36E-13	-0.253624
rs71126788	14	66232958	LC_IGP79	6.28E-11	-0.226114
rs34263575	14	66160754	LC_IGP79	5.76E-13	-0.249677
rs5809288	14	66237586	LC_IGP79	2.83E-11	-0.231052
rs59312400	14	66249255	LC_IGP79	3.22E-09	0.23537
rs72090965	14	65829951	LC_IGP79	5.21E-12	-0.242392
rs71126773	14	66103040	LC_IGP79	3.50E-13	-0.25162

rs34307226	14	65844447	LC_IGP79		1.11E-10	0.248985
rs60977949	14	65737104	LC_IGP79		4.66E-08	0.216201
rs10572775	14	66055427	LC_IGP79		1.64E-12	-0.245403
rs147422602	14	65990143	LC_IGP79		8.90E-14	-0.258518
rs34443059	14	66225456	LC_IGP79		1.63E-12	-0.245252
rs144508295	14	65811418	LC_IGP79		3.79E-10	-0.226464
rs34746444	14	66195404	LC_IGP79		4.08E-11	-0.23385
rs199534978	14	66076028	LC_IGP79		1.02E-12	-0.246017
rs201678871	14	66232956	LC_IGP79		6.28E-11	-0.226115
rs200274176	14	66069056	LC_IGP79		2.43E-09	-0.212561
rs35439429	14	66063919	LC_IGP79		6.74E-09	-0.207371
rs34307226	14	65844447	LC_IGP81		2.31E-11	-0.25782
rs142437037	14	65986959	LC_IGP81		4.30E-08	0.202871
rs10572775	14	66055427	LC_IGP81		1.48E-12	0.245814
rs147422602	14	65990143	LC_IGP81		5.81E-14	0.260334
rs59312400	14	66249255	LC_IGP81		3.22E-10	-0.24973
rs71126773	14	66103040	LC_IGP81		2.18E-13	0.253702
rs34443059	14	66225456	LC_IGP81		3.36E-13	0.252538
rs144508295	14	65811418	LC_IGP81		1.62E-10	0.231065
rs35439429	14	66063919	LC_IGP81		3.40E-09	0.211321
rs34263575	14	66160754	LC_IGP81		2.08E-13	0.254284
rs3216656	14	66113327	LC_IGP81		1.30E-13	0.256233
rs34746444	14	66195404	LC_IGP81		9.51E-12	0.241197
rs199534978	14	66076028	LC_IGP81		4.65E-13	0.249576
rs71126788	14	66232958	LC_IGP81		7.79E-12	0.236444
rs72090965	14	65829951	LC_IGP81		2.55E-12	0.245798
rs5809288	14	66237586	LC_IGP81		3.09E-12	0.241851
rs201678871	14	66232956	LC_IGP81		7.79E-12	0.236445
rs200274176	14	66069056	LC_IGP81		1.00E-09	0.217531
rs201623089	1	25297520	LC_IGP82		1.01E-09	0.720178
rs201028197	22	24142976	LC_IGP82		3.28E-08	0.220998
rs199739333	22	39777821	LC_IGP82		3.78E-17	-0.307934
rs199766327	22	24142978	LC_IGP82		4.88E-08	0.218315
rs201623089	1	25297520	LC_IGP83		1.01E-09	0.720075
rs199766327	22	24142978	LC_IGP83		4.86E-08	0.218308
rs199739333	22	39777821	LC_IGP83		3.29E-17	-0.308477
rs201028197	22	24142976	LC_IGP83		3.26E-08	0.221012
rs201678871	14	66232956	LC_IGP84		1.26E-11	0.234635
rs200183327	14	65980373	LC_IGP84		6.82E-09	0.214202
rs142437037	14	65986959	LC_IGP84		1.98E-09	0.222515
rs72090965	14	65829951	LC_IGP84		3.09E-13	0.256406
rs34263575	14	66160754	LC_IGP84		2.77E-15	0.273865
rs200274176	14	66069056	LC_IGP84		1.12E-11	0.242007
rs59312400	14	66249255	LC_IGP84		9.93E-12	-0.270725
rs5809288	14	66237586	LC_IGP84		4.98E-12	0.240116
rs34746444	14	66195404	LC_IGP84		7.77E-13	0.25404
rs60977949	14	65737104	LC_IGP84		2.10E-08	-0.222133
rs10572775	14	66055427	LC_IGP84		1.14E-14	0.268479
rs34443059	14	66225456	LC_IGP84		4.45E-14	0.262303
rs34307226	14	65844447	LC_IGP84		1.80E-13	-0.284208

rs33927018	14	66029423	LC_IGP84	8.37E-09	0.212704
rs35439429	14	66063919	LC_IGP84	5.73E-11	0.234447
rs3216656	14	66113327	LC_IGP84	1.53E-15	0.276167
rs199534978	14	66076028	LC_IGP84	7.75E-15	0.268382
rs71126773	14	66103040	LC_IGP84	5.54E-15	0.270514
rs147422602	14	65990143	LC_IGP84	9.60E-16	0.278652
rs144508295	14	65811418	LC_IGP84	7.60E-11	0.235705
rs61602020	14	65774613	LC_IGP84	1.29E-08	-0.261632
rs71126788	14	66232958	LC_IGP84	1.26E-11	0.234633
rs11284050	14	65884030	LC_IGP84	5.05E-09	0.216072
rs139022691	14	65778230	LC_IGP84	1.34E-08	0.259458
rs145473242	14	65826211	LC_IGP84	4.93E-08	-0.236556
rs201623089	1	25297520	LC_IGP85	8.85E-09	-0.67852
rs199739333	22	39777821	LC_IGP85	3.50E-13	0.266718
rs71126773	14	66103040	LC_IGP86	6.78E-14	-0.259036
rs11284050	14	65884030	LC_IGP86	2.84E-08	-0.204876
rs142437037	14	65986959	LC_IGP86	1.82E-08	-0.208472
rs72090965	14	65829951	LC_IGP86	9.31E-13	-0.250707
rs34746444	14	66195404	LC_IGP86	3.29E-12	-0.246538
rs61602020	14	65774613	LC_IGP86	3.40E-08	0.253452
rs200183327	14	65980373	LC_IGP86	4.51E-08	-0.201868
rs59312400	14	66249255	LC_IGP86	8.86E-11	0.257556
rs10572775	14	66055427	LC_IGP86	3.92E-13	-0.252111
rs144508295	14	65811418	LC_IGP86	7.98E-11	-0.234968
rs34263575	14	66160754	LC_IGP86	5.84E-14	-0.260082
rs34307226	14	65844447	LC_IGP86	4.70E-12	0.266628
rs71126788	14	66232958	LC_IGP86	5.10E-12	-0.238582
rs35439429	14	66063919	LC_IGP86	1.18E-09	-0.217477
rs201678871	14	66232956	LC_IGP86	5.10E-12	-0.238583
rs3216656	14	66113327	LC_IGP86	3.56E-14	-0.262098
rs34443059	14	66225456	LC_IGP86	1.34E-13	-0.256812
rs5809288	14	66237586	LC_IGP86	1.97E-12	-0.244079
rs199534978	14	66076028	LC_IGP86	1.37E-13	-0.255238
rs139022691	14	65778230	LC_IGP86	3.15E-08	-0.252258
rs147422602	14	65990143	LC_IGP86	1.65E-14	-0.26598
rs200274176	14	66069056	LC_IGP86	2.89E-10	-0.224476
rs150025803	1	25024202	LC_IGP88	8.08E-10	-1.01191
rs111399879	9	33166711	LC_IGP88	4.37E-08	0.743354
rs150110347	1	25308762	LC_IGP90	1.92E-09	1.05558
rs150025803	1	25024202	LC_IGP90	5.11E-13	1.18211
rs143916866	1	24967537	LC_IGP90	3.09E-09	0.903159
rs201623089	1	25297520	LC_IGP90	4.29E-11	0.774329
rs35456268	3	186721290	LC_IGP93	2.11E-26	-0.428674
rs200317857	3	186713293	LC_IGP93	1.88E-14	-0.311934
rs201540659	3	186713306	LC_IGP93	3.43E-10	-0.309456
rs71634023	3	186712738	LC_IGP93	2.40E-22	-0.406946
rs11394645	3	186728353	LC_IGP93	1.37E-09	-0.252558
rs202164259	3	186711347	LC_IGP93	8.59E-09	-0.296019
rs61210528	3	186742220	LC_IGP93	2.08E-09	-0.292833
rs59111563	3	186722848	LC_IGP93	2.17E-26	-0.428232

rs148580052	3	186720957	LC_IGP93	2.16E-26	-0.428666
rs199533504	3	186713310	LC_IGP93	4.77E-12	-0.266963
rs201678871	14	66232956	LC_IGP97	5.77E-09	-0.19959
rs3216656	14	66113327	LC_IGP97	8.55E-11	-0.222508
rs34307226	14	65844447	LC_IGP97	1.59E-10	0.244608
rs10572775	14	66055427	LC_IGP97	3.67E-10	-0.215649
rs34443059	14	66225456	LC_IGP97	3.17E-10	-0.216377
rs71126773	14	66103040	LC_IGP97	2.35E-10	-0.217126
rs147422602	14	65990143	LC_IGP97	1.13E-10	-0.221519
rs71126788	14	66232958	LC_IGP97	5.77E-09	-0.199589
rs5809288	14	66237586	LC_IGP97	6.93E-09	-0.199332
rs200274176	14	66069056	LC_IGP97	4.01E-08	-0.193336
rs34746444	14	66195404	LC_IGP97	1.34E-10	-0.224501
rs34263575	14	66160754	LC_IGP97	1.31E-10	-0.220491
rs199534978	14	66076028	LC_IGP97	7.02E-11	-0.222707
rs72090965	14	65829951	LC_IGP97	4.95E-08	-0.190358
rs59312400	14	66249255	LC_IGP97	2.48E-10	0.249003
rs67876958	6	32653806	LC_IGP99	2.40E-08	-0.221343
rs10574650	16	31342116	LC_IGP99	2.61E-08	-0.204024
rs9275175	6	32654147	LC_IGP99	1.51E-08	-0.224507
rs151183997	16	31376341	LC_IGP99	3.68E-08	-0.215777
rs199739333	22	39777821	LC_IGPRG15	1.21E-10	0.237593
chr1:240956912:I	1	240956912	LC_IGPRG16	4.58E-08	-0.589651
rs117770678	21	24652678	LC_IGPRG16	2.79E-08	-1.12509
rs114564778	6	32177263	LC_IGPRG25	2.40E-08	0.215007
rs114247475	6	32167009	LC_IGPRG25	4.25E-08	0.210892
rs199739333	22	39777821	LC_IGPRG25	2.33E-15	-0.289914
rs201186886	6	32490662	LC_IGPRG25	5.00E-08	0.255053
rs148630977	6	32161034	LC_IGPRG25	2.69E-08	0.215227
rs201643791	22	39743579	LC_IGPRG25	1.61E-08	-0.211824
rs3129954	6	32365580	LC_IGPRG29	5.85E-12	0.250066
rs9269081	6	32441100	LC_IGPRG29	1.22E-09	0.219765
rs3104373	6	32600375	LC_IGPRG29	9.91E-09	0.289684
rs147401848	6	32406843	LC_IGPRG29	2.91E-08	0.270282
rs149826815	6	32368593	LC_IGPRG29	4.37E-10	0.223967
rs10574650	16	31342116	LC_IGPRG29	8.19E-10	0.225008
rs6923504	6	32428186	LC_IGPRG29	2.44E-09	0.211715
rs200565579	6	32551764	LC_IGPRG29	1.19E-08	0.244827
rs146620734	6	32352220	LC_IGPRG29	4.80E-08	0.247914
rs201875530	6	32551762	LC_IGPRG29	8.36E-09	0.244776
rs116350876	6	32452470	LC_IGPRG29	3.01E-09	0.208897
rs182016754	6	32352737	LC_IGPRG29	4.57E-09	0.244943
rs9269190	6	32448500	LC_IGPRG29	3.19E-09	0.210043
rs3135388	6	32413051	LC_IGPRG29	1.59E-08	0.28168
rs66751942	6	32377716	LC_IGPRG29	5.46E-12	0.250738
rs114210228	6	32350107	LC_IGPRG29	4.66E-12	0.251305
rs3129951	6	32358286	LC_IGPRG29	2.38E-12	0.254548
rs66500466	6	32590362	LC_IGPRG29	1.00E-08	0.280627
rs2050188	6	32339897	LC_IGPRG29	1.76E-11	0.24022
rs9271366	6	32586854	LC_IGPRG29	3.67E-09	0.2828

rs3135391	6	32410987	LC_IGPRG29	3.50E-08	0.274782
rs114845955	6	32346772	LC_IGPRG29	4.67E-12	0.251295
rs9268925	6	32432969	LC_IGPRG29	5.63E-09	0.292098
rs3129955	6	32365840	LC_IGPRG29	4.65E-12	0.251309
rs3135350	6	32392981	LC_IGPRG29	2.69E-08	0.277213
rs112971792	6	32380398	LC_IGPRG29	1.21E-11	0.247996
rs6903608	6	32428285	LC_IGPRG29	3.64E-09	0.209615
rs9271191	6	32578449	LC_IGPRG29	4.28E-08	0.256357
rs9268978	6	32434978	LC_IGPRG29	8.65E-09	0.288351
rs114249283	6	32362416	LC_IGPRG29	6.98E-09	0.251539
rs114752560	6	32343714	LC_IGPRG29	4.92E-12	0.250988
rs9269203	6	32449775	LC_IGPRG29	4.50E-09	0.204602
rs9281913	6	32567633	LC_IGPRG29	1.54E-08	0.292258
rs28631719	6	32625409	LC_IGPRG29	1.64E-08	0.286333
rs3135352	6	32392906	LC_IGPRG29	2.69E-08	0.277213
rs200121045	6	32551756	LC_IGPRG29	1.09E-08	0.245475
rs3129882	6	32409530	LC_IGPRG29	9.20E-09	0.199567
rs115825744	6	32348309	LC_IGPRG29	4.66E-12	0.251301
rs200533339	6	32519905	LC_IGPRG29	5.45E-09	0.280751
rs116454000	6	32342822	LC_IGPRG29	4.87E-12	0.251008
rs3129889	6	32413545	LC_IGPRG29	1.46E-08	0.282601
rs151183997	16	31376341	LC_IGPRG29	3.11E-10	0.246618
rs149028172	6	32449523	LC_IGPRG29	1.53E-09	0.222405
rs9268880	6	32431358	LC_IGPRG29	2.44E-09	0.211715
rs114693266	6	32380717	LC_IGPRG29	6.70E-12	0.249722
rs9269520	6	32543617	LC_IGPRG29	4.69E-09	0.316651
rs3129868	6	32404377	LC_IGPRG29	3.01E-08	0.269868
rs199625001	6	32556155	LC_IGPRG29	3.23E-09	0.289402
rs3129948	6	32354644	LC_IGPRG29	4.65E-12	0.251309
rs115295735	6	32359763	LC_IGPRG29	3.70E-12	0.252532
rs70993876	6	32519338	LC_IGPRG29	1.08E-08	0.279429
rs145068357	16	31377244	LC_IGPRG29	3.85E-10	0.246234
rs141034368	6	32353166	LC_IGPRG29	1.10E-08	0.221107
rs3117098	6	32358513	LC_IGPRG29	3.62E-12	0.252369
rs116192447	6	32378940	LC_IGPRG29	5.47E-12	0.250653
rs9268927	6	32433085	LC_IGPRG29	8.65E-09	0.288351
esv2661368	6	32514888	LC_IGPRG29	4.34E-08	0.268533
rs10574650	16	31342116	LC_IGPRG30	3.63E-11	0.241185
rs8014442	14	106807666	LC_IGPRG30	3.82E-08	-0.397107
rs145068357	16	31377244	LC_IGPRG30	7.47E-11	0.254839
rs151183997	16	31376341	LC_IGPRG30	4.08E-11	0.257382
rs117770678	21	24652678	LC_IGPRG32	3.64E-08	-1.11935
rs9268925	6	32432969	LC_IGPRG33	2.67E-08	0.278415
rs9268978	6	32434978	LC_IGPRG33	2.77E-08	0.277942
rs9271366	6	32586854	LC_IGPRG33	4.94E-08	0.261214
rs8014442	14	106807666	LC_IGPRG33	2.06E-08	-0.405921
rs10574650	16	31342116	LC_IGPRG33	4.84E-09	0.214179
rs114249283	6	32362416	LC_IGPRG33	1.58E-08	0.24515
rs3104373	6	32600375	LC_IGPRG33	2.69E-08	0.280617
rs9268927	6	32433085	LC_IGPRG33	2.77E-08	0.277942

rs3135388	6	32413051	LC_IGPRG33	3.37E-08	0.274796
rs145068357	16	31377244	LC_IGPRG33	2.76E-09	0.233591
rs3129889	6	32413545	LC_IGPRG33	3.73E-08	0.274058
rs151183997	16	31376341	LC_IGPRG33	3.45E-09	0.231323
rs111399879	9	33166711	LC_IGPRG4	4.56E-08	-0.746165
rs150025803	1	25024202	LC_IGPRG7	2.10E-09	0.992697
rs114693266	6	32380717	LC_IGPRG9	2.81E-10	-0.230069
rs3129955	6	32365840	LC_IGPRG9	1.71E-10	-0.232577
rs3129954	6	32365580	LC_IGPRG9	2.09E-10	-0.231395
rs199520858	6	32521867	LC_IGPRG9	2.99E-08	-0.270617
rs9269081	6	32441100	LC_IGPRG9	3.86E-08	-0.199225
rs74678949	6	32577197	LC_IGPRG9	4.89E-08	-0.254464
rs3104373	6	32600375	LC_IGPRG9	1.01E-08	-0.289856
rs9268927	6	32433085	LC_IGPRG9	6.44E-09	-0.291177
rs116192447	6	32378940	LC_IGPRG9	2.36E-10	-0.230952
rs3129868	6	32404377	LC_IGPRG9	1.03E-08	-0.279097
rs28631719	6	32625409	LC_IGPRG9	1.27E-08	-0.288904
rs147401848	6	32406843	LC_IGPRG9	1.30E-08	-0.277339
rs9269448	6	32542011	LC_IGPRG9	3.86E-08	-0.288869
rs3135388	6	32413051	LC_IGPRG9	1.43E-08	-0.282946
rs199784558	6	32513542	LC_IGPRG9	3.66E-08	-0.250404
esv2661745	6	32480579	LC_IGPRG9	1.05E-08	-0.309507
rs114210228	6	32350107	LC_IGPRG9	1.76E-10	-0.232431
rs3135350	6	32392981	LC_IGPRG9	1.54E-08	-0.282333
rs116350876	6	32452470	LC_IGPRG9	3.35E-08	-0.194872
rs3135352	6	32392906	LC_IGPRG9	1.54E-08	-0.282333
rs115825744	6	32348309	LC_IGPRG9	1.78E-10	-0.232372
rs3117098	6	32358513	LC_IGPRG9	1.47E-10	-0.233198
rs146620734	6	32352220	LC_IGPRG9	1.67E-08	-0.256553
rs114619532	6	32367017	LC_IGPRG9	7.57E-09	-0.284651
rs149028172	6	32449523	LC_IGPRG9	2.55E-08	-0.205492
rs2050188	6	32339897	LC_IGPRG9	7.11E-09	-0.207393
rs151183997	16	31376341	LC_IGPRG9	3.98E-08	-0.215803
rs200533339	6	32519905	LC_IGPRG9	1.51E-09	-0.291103
rs9270656	6	32566011	LC_IGPRG9	3.86E-08	-0.2563
rs112971792	6	32380398	LC_IGPRG9	6.08E-10	-0.226934
rs113738020	6	32540158	LC_IGPRG9	2.01E-08	-0.276219
rs9271160	6	32577646	LC_IGPRG9	3.86E-08	-0.2563
rs114752560	6	32343714	LC_IGPRG9	2.16E-10	-0.23126
rs66500466	6	32590362	LC_IGPRG9	4.38E-09	-0.287733
rs3135391	6	32410987	LC_IGPRG9	2.39E-08	-0.278421
rs114845955	6	32346772	LC_IGPRG9	1.80E-10	-0.23231
rs1966001	6	32581782	LC_IGPRG9	3.86E-08	-0.2563
rs3129948	6	32354644	LC_IGPRG9	1.71E-10	-0.232577
rs115295735	6	32359763	LC_IGPRG9	1.45E-10	-0.23355
rs70993876	6	32519338	LC_IGPRG9	5.06E-09	-0.28593
rs4639377	6	32625283	LC_IGPRG9	2.13E-08	-0.276987
rs9268978	6	32434978	LC_IGPRG9	6.44E-09	-0.291177
rs66751942	6	32377716	LC_IGPRG9	2.31E-10	-0.23114
rs76485153	6	32520259	LC_IGPRG9	3.63E-08	-0.256475

rs9271203	6	32578885	LC_IGPRG9	3.86E-08	-0.2563
rs142975901	6	32517357	LC_IGPRG9	7.79E-09	-0.265244
rs9268925	6	32432969	LC_IGPRG9	4.32E-09	-0.294649
rs199625001	6	32556155	LC_IGPRG9	2.80E-09	-0.290901
rs114249283	6	32362416	LC_IGPRG9	3.33E-08	-0.240307
rs3129889	6	32413545	LC_IGPRG9	1.18E-08	-0.284705
rs3129951	6	32358286	LC_IGPRG9	1.15E-10	-0.23462
rs9271191	6	32578449	LC_IGPRG9	3.06E-08	-0.259418
rs9269675	6	32546515	LC_IGPRG9	2.34E-08	-0.294495
rs116454000	6	32342822	LC_IGPRG9	2.20E-10	-0.231114
rs9281913	6	32567633	LC_IGPRG9	2.25E-08	-0.289243
rs9269520	6	32543617	LC_IGPRG9	2.95E-09	-0.321142
rs182016754	6	32352737	LC_IGPRG9	3.03E-08	-0.23189
rs149826815	6	32368593	LC_IGPRG9	3.69E-09	-0.212071
rs9271366	6	32586854	LC_IGPRG9	2.00E-09	-0.287868
