# Supplementary material for: Genome-Wide Association Study on Immunoglobulin G Glycosylation Patterns
Source: Front Immunol. 2018 Feb 26;9:277. doi: 10.3389/fimmu.2018.00277 (PMC5834439; doi:10.3389/fimmu.2018.00277)
Supplement: Table S5 — List of all replicated associations. [file Table_5.PDF]

All Replicated Associations

SNP	GWAS Code	Trait	Chromosome	Position	LD-Block per Chromosome	*p* (KORA)	*p* (LLS)	Effect Direction	beta(KORA)	beta(LLS)	Strand (KORA)	Effect Allele (KORA)	Other Allele (KORA)	Effect Allele Frequency (KORA)	Standard Error of beta(KORA)	Strand (LLS)	Effect Allele (LLS)	Other Allele (LLS)	Effect Allele Frequency (LLS)	Standard Error of beta(LLS)
rs16830188	LC_IGP90	IgG2_G0FN	1	25298841	1	1.71E-14	1.27E-06	+	1.00868	0.7404	+	T	C	0.017987805	0.130298	+	T	C	0.0212	0.1528
rs16830188	LC_IGP135	IgG2_G0FNn	1	25298841	1	1.60E-13	1.85E-06	+	0.970599	0.7348	+	T	C	0.018165025	0.130422	+	T	C	0.0212	0.1541
rs16830188	LC_IGP133	IgG2_G1Fn	1	25298841	1	2.04E-12	1.06E-09	-	-0.930239	-0.8772	+	T	C	0.018165025	0.131258	+	T	C	0.0212	0.1438
rs16830188	LC_IGP88	IgG2_G1F	1	25298841	1	2.45E-11	3.97E-10	-	-0.883488	-0.9129	+	T	C	0.017987805	0.131414	+	T	C	0.0212	0.146
rs10903120	LC_IGP90	IgG2_G0FN	1	25298284	1	4.63E-11	2.27E-09	+	0.772519	0.8383	+	A	G	0.028229868	0.116567	+	A	G	0.0293	0.1403
rs112702191	LC_IGP90	IgG2_G0FN	1	25296560	1	5.17E-11	1.36E-07	+	0.762368	0.7314	+	C	A	0.029792907	0.115326	+	C	A	0.031	0.1388
rs10903120	LC_IGP135	IgG2_G0FNn	1	25298284	1	1.59E-10	3.22E-09	+	0.750836	0.8392	+	A	G	0.02846402	0.116627	+	A	G	0.0293	0.1417
rs112702191	LC_IGP135	IgG2_G0FNn	1	25296560	1	2.17E-10	2.43E-07	+	0.737381	0.7236	+	C	A	0.030039199	0.1154	+	C	A	0.0311	0.1402
rs16830188	LC_IGP89	IgG2_G2F	1	25298841	1	2.97E-10	1.85E-06	-	-0.831333	-0.7859	+	T	C	0.017987805	0.131127	+	T	C	0.0212	0.1648
rs16830188	LC_IGP199	IgG4_G2Fn	1	25298841	1	5.07E-10	4.29E-07	-	-0.817962	-0.914	+	T	C	0.01802077	0.130772	+	T	C	0.0212	0.1808
rs16830188	LC_IGP175	IgG4_G2F	1	25298841	1	5.60E-10	1.73E-07	-	-0.815341	-0.9452	+	T	C	0.017976843	0.130686	+	T	C	0.0212	0.1809
rs16830188	LC_IGP_R62	IgG2_G1F/IgG2_G0F	1	25298841	1	3.58E-09	8.44E-07	-	-0.782722	-0.782	+	T	C	0.01799878	0.131885	+	T	C	0.0212	0.1588
rs16830188	LC_IGP144	IgG2 G0n	1	25298841	1	4.68E-09	1.08E-06	+	0.776892	0.777	+	T	C	0.018165025	0.131899	+	T	C	0.0212	0.1593
rs112702191	LC_IGP133	IgG2_G1Fn	1	25296560	1	2.73E-08	5.98E-10	-	-0.649538	-0.8307	+	C	A	0.030039199	0.116299	+	C	A	0.0311	0.1342
rs10903120	LC_IGP133	IgG2_G1Fn	1	25298284	1	2.83E-08	6.53E-11	-	-0.655848	-0.8725	+	A	G	0.02846402	0.117559	+	A	G	0.0293	0.1336
rs16830188	LC_IGP145	IgG2 G1n	1	25298841	1	3.55E-08	2.53E-07	-	-0.732372	-0.7958	+	T	C	0.018165025	0.132235	+	T	C	0.0212	0.1544
rs11923417	LC_IGP_R34	IgG1_G1FS1/IgG1_G1F	3	186705790	1	2.85E-08	3.94E-17	-	-0.226888	-0.4207	+	C	G	0.638478899	0.0406769	+	C	G	0.6235	0.05
rs35832957	LC_IGP_R34	IgG1_G1FS1/IgG1_G1F	3	186707884	1	2.97E-09	6.59E-16	-	-0.319878	-0.4795	+	T	C	0.152404188	0.0536119	+	T	C	0.1933	0.0594
rs11923417	LC_IGP_R35	IgG1_G2FS1/IgG1_G2F	3	186705790	1	1.93E-17	1.16E-12	-	-0.346943	-0.3398	+	C	G	0.637718559	0.0403695	+	C	G	0.6235	0.0478
rs7635748	LC_IGP_R35	IgG1_G2FS1/IgG1_G2F	3	186706893	1	1.02E-08	1.36E-08	-	-0.428664	-0.4814	+	A	C	0.082027546	0.0744565	+	A	C	0.0985	0.0848
rs7622117	LC_IGP_R35	IgG1_G2FS1/IgG1_G2F	3	186707554	1	1.43E-09	8.41E-08	-	-0.22972	-0.2446	+	A	G	0.467042715	0.0377396	+	A	G	0.4521	0.0457
rs7622133	LC_IGP_R35	IgG1_G2FS1/IgG1_G2F	3	186707602	1	1.76E-09	1.24E-07	-	-0.228635	-0.2421	+	T	G	0.467234412	0.0377737	+	T	G	0.4521	0.0458
rs35832957	LC_IGP_R35	IgG1_G2FS1/IgG1_G2F	3	186707884	1	2.07E-10	2.57E-12	-	-0.344969	-0.4008	+	T	C	0.152137905	0.0539289	+	T	C	0.1933	0.0573
rs13082825	LC_IGP_R35	IgG1_G2FS1/IgG1_G2F	3	186708013	1	1.23E-16	1.95E-13	-	-0.307823	-0.33	+	C	G	0.547775641	0.0367812	+	C	G	0.5381	0.0449
rs11923417	LC_IGP_R74	IgG2_G1FS1/IgG2_G1F	3	186705790	1	5.82E-20	8.69E-22	-	-0.372689	-0.4747	+	C	G	0.638042099	0.0402155	+	C	G	0.6235	0.0495
rs7635748	LC_IGP_R74	IgG2_G1FS1/IgG2_G1F	3	186706893	1	1.77E-08	2.17E-11	-	-0.420991	-0.5907	+	A	C	0.082274422	0.0743582	+	A	C	0.0985	0.0882
rs36011306	LC_IGP_R74	IgG2_G1FS1/IgG2_G1F	3	186706897	1	4.81E-09	2.35E-20	-	-0.313931	-0.5386	+	C	T	0.158015199	0.0533429	+	C	T	0.1784	0.0583
rs7622117	LC_IGP_R74	IgG2_G1FS1/IgG2_G1F	3	186707554	1	3.50E-14	7.33E-10	-	-0.286643	-0.2944	+	A	G	0.467837076	0.0374874	+	A	G	0.4521	0.0478
rs7622133	LC_IGP_R74	IgG2_G1FS1/IgG2_G1F	3	186707602	1	5.66E-14	7.42E-10	-	-0.284534	-0.2951	+	T	G	0.468038743	0.0375263	+	T	G	0.4521	0.0479
rs35832957	LC_IGP_R74	IgG2_G1FS1/IgG2_G1F	3	186707884	1	1.08E-13	1.08E-19	-	-0.400895	-0.5394	+	T	C	0.152626027	0.0534919	+	T	C	0.1933	0.0594
rs13082825	LC_IGP_R74	IgG2_G1FS1/IgG2_G1F	3	186708013	1	5.69E-21	1.62E-23	-	-0.347978	-0.4666	+	C	G	0.548292556	0.0365327	+	C	G	0.5381	0.0467
rs11923417	LC_IGP_R75	IgG2_G2FS1/IgG2_G2F	3	186705790	1	1.87E-10	2.55E-18	-	-0.261448	-0.4321	+	C	G	0.63759701	0.0407745	+	C	G	0.6235	0.0495
rs7622117	LC_IGP_R75	IgG2_G2FS1/IgG2_G2F	3	186707554	1	1.51E-08	1.76E-08	-	-0.215133	-0.2678	+	A	G	0.467236405	0.0378128	+	A	G	0.4521	0.0475
rs7622133	LC_IGP_R75	IgG2_G2FS1/IgG2_G2F	3	186707602	1	1.91E-08	1.70E-08	-	-0.213769	-0.2687	+	T	G	0.467437767	0.0378463	+	T	G	0.4521	0.0476
rs35832957	LC_IGP_R75	IgG2_G2FS1/IgG2_G2F	3	186707884	1	1.77E-09	2.22E-15	-	-0.325982	-0.4728	+	T	C	0.152346511	0.0538642	+	T	C	0.1933	0.0596
rs13082825	LC_IGP_R75	IgG2_G2FS1/IgG2_G2F	3	186708013	1	5.58E-11	5.46E-19	-	-0.244539	-0.4157	+	C	G	0.547688835	0.0370574	+	C	G	0.5381	0.0467
rs13082825	LC_IGP_R92	IgG4_G1FS1/IgG4_G1F	3	186708013	1	2.56E-08	6.01E-14	-	-0.207716	-0.3452	+	C	G	0.547678963	0.0371155	+	C	G	0.5381	0.046
rs11923417	LC_IGP_R93	IgG4_G2FS1/IgG4_G2F	3	186705790	1	2.98E-08	2.02E-12	-	-0.228317	-0.3394	+	C	G	0.637574131	0.0409936	+	C	G	0.6235	0.0483
rs13082825	LC_IGP_R93	IgG4_G2FS1/IgG4_G2F	3	186708013	1	6.68E-09	6.80E-14	-	-0.217154	-0.3416	+	C	G	0.547669921	0.03725	+	C	G	0.5381	0.0456
rs11923417	LC_IGP111	IgG2 SA per Gal	3	186705790	1	9.12E-14	7.67E-19	-	-0.306342	-0.4386	+	C	G	0.637641486	0.0407432	+	C	G	0.6231	0.0495
rs7622117	LC_IGP111	IgG2 SA per Gal	3	186707554	1	9.12E-10	4.26E-09	-	-0.233501	-0.2795	+	A	G	0.467117667	0.0379014	+	A	G	0.4519	0.0476
rs7622133	LC_IGP111	IgG2 SA per Gal	3	186707602	1	1.23E-09	3.65E-09	-	-0.231883	-0.2815	+	T	G	0.467311744	0.0379382	+	T	G	0.4519	0.0477
rs35832957	LC_IGP111	IgG2 SA per Gal	3	186707884	1	5.83E-10	4.36E-16	-	-0.337779	-0.485	+	T	C	0.152112006	0.0541927	+	T	C	0.1937	0.0597
rs13082825	LC_IGP111	IgG2 SA per Gal	3	186708013	1	1.25E-13	6.12E-20	-	-0.277661	-0.4263	+	C	G	0.547598172	0.0371375	+	C	G	0.538	0.0466
rs11923417	LC_IGP120	IgG2 FGS1/(FG+FGS1)	3	186705790	1	6.86E-14	1.30E-14	-	-0.307003	-0.3722	+	C	G	0.637821123	0.0406279	+	C	G	0.6235	0.0483
rs13082825	LC_IGP120	IgG2 FGS1/(FG+FGS1)	3	186708013	1	5.65E-14	3.62E-14	-	-0.280245	-0.3456	+	C	G	0.548016789	0.0369597	+	C	G	0.5381	0.0456
rs35832957	LC_IGP120	IgG2 FGS1/(FG+FGS1)	3	186707884	1	2.13E-08	1.07E-12	-	-0.303876	-0.4173	+	T	C	0.152432271	0.0539873	+	T	C	0.1933	0.0586
rs7622133	LC_IGP120	IgG2 FGS1/(FG+FGS1)	3	186707602	1	6.96E-10	9.27E-07	-	-0.234636	-0.2291	+	T	G	0.46771398	0.0378202	+	T	G	0.4521	0.0467
rs7622117	LC_IGP120	IgG2 FGS1/(FG+FGS1)	3	186707554	1	4.46E-10	1.39E-06	-	-0.237086	-0.2248	+	A	G	0.46751219	0.0377819	+	A	G	0.4521	0.0466
rs11923417	LC_IGP122	IgG2 FG1S1/(FG1+FG1S1)	3	186705790	1	5.88E-20	8.99E-22	-	-0.372627	-0.4744	+	C	G	0.638042099	0.0402141	+	C	G	0.6235	0.0495
rs7635748	LC_IGP122	IgG2 FG1S1/(FG1+FG1S1)	3	186706893	1	1.89E-08	2.18E-11	-	-0.420127	-0.5903	+	A	C	0.082274422	0.0743581	+	A	C	0.0985	0.0882
rs36011306	LC_IGP122	IgG2 FG1S1/(FG1+FG1S1)	3	186706897	1	5.10E-09	2.48E-20	-	-0.313412	-0.5383	+	C	T	0.158015199	0.0533426	+	C	T	0.1784	0.0583
rs7622117	LC_IGP122	IgG2 FG1S1/(FG1+FG1S1)	3	186707554	1	3.53E-14	7.31E-10	-	-0.28659	-0.2944	+	A	G	0.467837076	0.0374861	+	A	G	0.4521	0.0478
rs7622133	LC_IGP122	IgG2 FG1S1/(FG1+FG1S1)	3	186707602	1	5.72E-14	7.39E-10	-	-0.284467	-0.2951	+	T	G	0.468038743	0.0375251	+	T	G	0.4521	0.0479
rs35832957	LC_IGP122	IgG2 FG1S1/(FG1+FG1S1)	3	186707884	1	1.13E-13	1.12E-19	-	-0.400585	-0.5392	+	T	C	0.152626027	0.0534911	+	T	C	0.1933	0.0594
rs13082825	LC_IGP122	IgG2 FG1S1/(FG1+FG1S1)	3	186708013	1	6.25E-21	1.60E-23	-	-0.347613	-0.4665	+	C	G	0.548292556	0.0365333	+	C	G	0.5381	0.0467
rs6764279	LC_IGP123	IgG2 FG2S1/(FG2+FG2S1)	3	186723975	3	4.72E-30	3.51E-59	-	-0.447859	-0.6716	+	A	C	0.260153447	0.0385396	+	A	C	0.293	0.0414
rs11710456	LC_IGP_R34	IgG1_G1FS1/IgG1_G1F	3	186725887	3	2.36E-31	1.23E-61	-	-0.45159	-0.6992	+	A	G	0.260867584	0.0379623	+	A	G	0.2939	0.0422
rs7621161	LC_IGP123	IgG2 FG2S1/(FG2+FG2S1)	3	186727170	3	9.51E-31	3.47E-58	-	-0.44738	-0.6761	+	A	C	0.26052471	0.0380166	+	A	C	0.296	0.042
rs17775791	LC_IGP123	IgG2 FG2S1/(FG2+FG2S1)	3	186722362	3	1.86E-26	1.17E-56	-	-0.427663	-0.6723	+	C	T	0.243587553	0.0394792	+	C	T	0.2752	0.0424
rs13322676	LC_IGP123	IgG2 FG2S1/(FG2+FG2S1)	3	186718745	3	8.16E-26	1.38E-55	-	-0.422833	-0.6716	+	C	A	0.243973764	0.0395667	+	C	A	0.2769	0.0428
rs11923417	LC_IGP186	IgG4 SA per Gal	3	186705790	1	4.62E-08	4.36E-10	-	-0.224988	-0.3025	+	C	G	0.637440942	0.0409729	+	C	G	0.623	0.0485
rs13082825	LC_IGP186	IgG4 SA per Gal	3	186708013	1	2.66E-08	1.32E-11	-	-0.208421	-0.3101	+	C	G	0.546919217	0.0372857	+	C	G	0.5379	0.0458
rs13082825	LC_IGP189	IgG4 FG1S1/(FG1+FG1S1)	3	186708013	1	2.04E-08	5.71E-14	-	-0.209184	-0.3455	+	C	G	0.547678963	0.0371149	+	C	G	0.5381	0.046
rs11923417	LC_IGP190	IgG4 FG2S1/(FG2+FG2S1)	3	186705790	1	2.90E-08	2.00E-12	-	-0.228527	-0.3395	+	C	G	0.637574131	0.040994	+	C	G	0.6235	0.0483
rs13082825	LC_IGP190	IgG4 FG2S1/(FG2+FG2S1)	3	186708013	1	6.58E-09	6.65E-14	-	-0.21725	-0.3417	+	C	G	0.547669921	0.0372507	+	C	G	0.5381	0.0456
rs11923417	LC_IGP25	IgG1 SA per Gal	3	186705790	1	1.11E-10	1.41E-09	-	-0.267061	-0.2842	+	C	G	0.635845551	0.041125	+	C	G	0.6236	0.0469
rs13082825	LC_IGP25	IgG1 SA per Gal	3	186708013	1	1.83E-08	1.82E-10	-	-0.212024	-0.2812	+	C	G	0.545062372	0.0374828	+	C	G	0.5382	0.0441
rs11923417	LC_IGP36	IgG1 FG1S1/(FG1+FG1S1)	3	186705790	1	2.50E-08	3.92E-17	-	-0.227871	-0.4199	+	C	G	0.638478899	0.0406857	+	C	G	0.6235	0.0499
rs35832957	LC_IGP36	IgG1 FG1S1/(FG1+FG1S1)	3	186707884	1	2.38E-09	4.93E-16	-	-0.321889	-0.4806	+	T	C	0.152404188	0.0536207	+	T	C	0.1933	0.0592
rs11923417	LC_IGP37	IgG1 FG2S1/(FG2+FG2S1)	3	186705790	1	1.86E-17	1.20E-12	-	-0.347106	-0.3396	+	C	G	0.637718559	0.0403688	+	C	G	0.6235	0.0478
rs7635748	LC_IGP37	IgG1 FG2S1/(FG2+FG2S1)	3	186706893	1	9.81E-09	1.42E-08	-	-0.429144	-0.4807	+	A	C	0.082027546	0.074455	+	A	C	0.0985	0.0848
rs7622117	LC_IGP37	IgG1 FG2S1/(FG2+FG2S1)	3	186707554	1	1.43E-09	8.57E-08	-	-0.229712	-0.2444	+	A	G	0.467042715	0.0377397	+	A	G	0.4521	0.0457
rs7622133	LC_IGP37	IgG1 FG2S1/(FG2+FG2S1)	3	186707602	1	1.76E-09	1.26E-07	-	-0.228628	-0.2419	+	T	G	0.467234412	0.0377739	+	T	G	0.4521	0.0458
rs35832957	LC_IGP37	IgG1 FG2S1/(FG2+FG2S1)	3	186707884	1	1.89E-10	2.69E-12	-	-0.3457	-0.4004	+	T	C	0.152137905	0.0539262	+	T	C	0.1933	0.0572
rs13082825	LC_IGP37	IgG1 FG2S1/(FG2+FG2S1)	3	186708013	1	1.32E-16	2.02E-13	-	-0.307515	-0.3297	+	C	G	0.547775641	0.0367829	+	C	G	0.5381	0.0449
rs35832957	LC_IGP7	IgG1_G1FS1	3	186707884	1	8.58E-09	4.38E-10	-	-0.310565	-0.3736	+	T	C	0.152404188	0.0536667	+	T	C	0.1933	0.0599
rs11923417	LC_IGP93	IgG2_G1FS1	3	186705790	1	2.55E-14	1.26E-14	-	-0.312708	-0.3734	+	C	G	0.638042099	0.0406731	+	C	G	0.6235	0.0484
rs7622117	LC_IGP93	IgG2_G1FS1	3	186707554	1	5.17E-12	3.34E-07	-	-0.262317	-0.2397	+	A	G	0.467837076	0.0377312	+	A	G	0.4521	0.047
rs7622133	LC_IGP93	IgG2_G1FS1	3	186707602	1	8.92E-12	6.22E-07	-	-0.2596	-0.2347	+	T	G	0.468038743	0.0377718	+	T	G	0.4521	0.0471
rs35832957	LC_IGP93	IgG2_G1FS1	3	186707884	1	4.23E-09	1.88E-13	-	-0.319094	-0.4338	+	T	C	0.152626027	0.054019	+	T	C	0.1933	0.059
rs13082825	LC_IGP93	IgG2_G1FS1	3	186708013	1	1.24E-15	2.96E-15	-	-0.29844	-0.3593	+	C	G	0.548292556	0.036933	+	C	G	0.5381	0.0455
rs13325033	LC_IGP37	IgG1 FG2S1/(FG2+FG2S1)	3	186708571	2	3.45E-12	2.02E-10	-	-0.293628	-0.291	+	A	G	0.782990313	0.0418815	+	A	G	0.7928	0.0458
rs13325033	LC_IGP_R35	IgG1_G2FS1/IgG1_G2F	3	186708571	2	4.20E-12	1.98E-10	-	-0.292466	-0.2912	+	A	G	0.782990313	0.0418864	+	A	G	0.7928	0.0458
rs13325033	LC_IGP122	IgG2 FG1S1/(FG1+FG1S1)	3	186708571	2	2.06E-14	3.71E-15	-	-0.322208	-0.3681	+	A	G	0.783118111	0.0417533	+	A	G	0.7928	0.0468
rs35376994	LC_IGP123	IgG2 FG2S1/(FG2+FG2S1)	3	186715319	3	4.97E-23	2.89E-52	-	-0.404264	-0.6749	+	A	G	0.237520884	0.0402931	+	A	G	0.2723	0.0444
rs13325033	LC_IGP120	IgG2 FGS1/(FG+FGS1)	3	186708571	2	6.34E-10	1.16E-10	-	-0.261677	-0.2955	+	A	G	0.782985705	0.0420777	+	A	G	0.7928	0.0458
rs13325033	LC_IGP111	IgG2 SA per Gal	3	186708571	2	1.06E-10	7.51E-14	-	-0.273985	-0.35	+	A	G	0.782370279	0.0421421	+	A	G	0.7924	0.0468
rs13325033	LC_IGP93	IgG2_G1FS1	3	186708571	2	2.20E-08	3.19E-11	-	-0.237589	-0.3037	+	A	G	0.783118111	0.04225	+	A	G	0.7928	0.0458
rs13325033	LC_IGP_R74	IgG2_G1FS1/IgG2_G1F	3	186708571	2	2.06E-14	3.62E-15	-	-0.322227	-0.3682	+	A	G	0.783118111	0.041755	+	A	G	0.7928	0.0468
rs13325033	LC_IGP_R75	IgG2_G2FS1/IgG2_G2F	3	186708571	2	2.96E-09	1.90E-13	-	-0.251067	-0.3439	+	A	G	0.782814083	0.0420756	+	A	G	0.7928	0.0468
rs759602	LC_IGP37	IgG1 FG2S1/(FG2+FG2S1)	3	186708804	2	8.81E-12	1.96E-10	-	-0.284922	-0.2868	+	G	A	0.784188034	0.0414456	+	G	A	0.802	0.0451
rs759602	LC_IGP_R35	IgG1_G2FS1/IgG1_G2F	3	186708804	2	1.08E-11	1.94E-10	-	-0.283699	-0.2869	+	G	A	0.784188034	0.0414506	+	G	A	0.802	0.0451
rs759602	LC_IGP122	IgG2 FG1S1/(FG1+FG1S1)	3	186708804	2	1.77E-13	1.92E-14	-	-0.307124	-0.353	+	G	A	0.784319707	0.0413485	+	G	A	0.802	0.0461
rs55862858	LC_IGP123	IgG2 FG2S1/(FG2+FG2S1)	3	186713103	3	6.96E-22	4.45E-52	-	-0.396345	-0.6783	+	C	T	0.239142346	0.0406379	+	C	T	0.2771	0.0447
rs759602	LC_IGP120	IgG2 FGS1/(FG+FGS1)	3	186708804	2	6.21E-09	9.77E-10	-	-0.243449	-0.2764	+	G	A	0.784188034	0.0416723	+	G	A	0.802	0.0452
rs759602	LC_IGP111	IgG2 SA per Gal	3	186708804	2	1.07E-09	7.09E-13	-	-0.256081	-0.3311	+	G	A	0.783591331	0.0417382	+	G	A	0.8016	0.0461
rs759602	LC_IGP_R74	IgG2_G1FS1/IgG2_G1F	3	186708804	2	1.69E-13	1.90E-14	-	-0.307393	-0.353	+	G	A	0.784319707	0.0413491	+	G	A	0.802	0.0461
rs4012171	LC_IGP122	IgG2 FG1S1/(FG1+FG1S1)	3	186711453	2	1.22E-17	2.79E-19	-	-0.354924	-0.4471	+	C	A	0.757085723	0.0410375	+	C	A	0.7998	0.0498
rs4012171	LC_IGP_R74	IgG2_G1FS1/IgG2_G1F	3	186711453	2	1.11E-17	2.88E-19	-	-0.355364	-0.447	+	C	A	0.757085723	0.0410369	+	C	A	0.7998	0.0498
rs4012171	LC_IGP111	IgG2 SA per Gal	3	186711453	2	3.79E-14	8.20E-17	-	-0.316456	-0.415	+	C	A	0.756424505	0.0414388	+	C	A	0.7996	0.0498
rs4012171	LC_IGP_R75	IgG2_G2FS1/IgG2_G2F	3	186711453	2	2.07E-11	1.28E-15	-	-0.279521	-0.3988	+	C	A	0.756786455	0.0414213	+	C	A	0.7998	0.0499
rs16848727	LC_IGP123	IgG2 FG2S1/(FG2+FG2S1)	3	186712963	3	2.08E-21	4.86E-52	-	-0.392741	-0.679	+	G	A	0.238339159	0.0407627	+	G	A	0.2772	0.0447
rs4012171	LC_IGP_R34	IgG1_G1FS1/IgG1_G1F	3	186711453	2	6.49E-11	1.53E-15	-	-0.271409	-0.3985	+	C	A	0.757715337	0.0412739	+	C	A	0.7998	0.05
rs4012171	LC_IGP36	IgG1 FG1S1/(FG1+FG1S1)	3	186711453	2	7.16E-11	1.76E-15	-	-0.270892	-0.397	+	C	A	0.757715337	0.0412884	+	C	A	0.7998	0.0499
rs4012171	LC_IGP93	IgG2_G1FS1	3	186711453	2	1.42E-09	1.81E-13	-	-0.253533	-0.3552	+	C	A	0.757085723	0.041644	+	C	A	0.7998	0.0482
rs4012171	LC_IGP120	IgG2 FGS1/(FG+FGS1)	3	186711453	2	1.09E-11	2.98E-12	-	-0.283612	-0.3399	+	C	A	0.756937424	0.0414425	+	C	A	0.7998	0.0487
rs4012171	LC_IGP_R35	IgG1_G2FS1/IgG1_G2F	3	186711453	2	1.08E-14	3.80E-11	-	-0.321488	-0.321	+	C	A	0.756938034	0.0412071	+	C	A	0.7998	0.0486
rs4012171	LC_IGP37	IgG1 FG2S1/(FG2+FG2S1)	3	186711453	2	9.62E-15	3.81E-11	-	-0.322089	-0.3209	+	C	A	0.756938034	0.0412043	+	C	A	0.7998	0.0486
rs4012171	LC_IGP_R93	IgG4_G2FS1/IgG4_G2F	3	186711453	2	2.72E-08	4.33E-10	-	-0.232916	-0.303	+	C	A	0.756787065	0.0416963	+	C	A	0.7998	0.0486
rs4012171	LC_IGP190	IgG4 FG2S1/(FG2+FG2S1)	3	186711453	2	2.63E-08	4.39E-10	-	-0.233144	-0.3029	+	C	A	0.756787065	0.0416967	+	C	A	0.7998	0.0486
rs4012171	LC_IGP25	IgG1 SA per Gal	3	186711453	2	8.58E-10	7.47E-10	-	-0.258128	-0.2931	+	C	A	0.75569303	0.0418279	+	C	A	0.7999	0.0476
rs10937277	LC_IGP36	IgG1 FG1S1/(FG1+FG1S1)	3	186712711	3	3.50E-12	3.42E-42	-	-0.274668	-0.6249	+	A	G	0.298033028	0.039188	+	A	G	0.31	0.0459
rs10937277	LC_IGP37	IgG1 FG2S1/(FG2+FG2S1)	3	186712711	3	6.65E-20	2.81E-25	-	-0.361141	-0.4646	+	A	G	0.297487179	0.0390322	+	A	G	0.31	0.0447
rs10937277	LC_IGP34	IgG1 FGS1/(FG+FGS1)	3	186712711	3	3.23E-11	1.78E-13	-	-0.26387	-0.3212	+	A	G	0.298033028	0.0394907	+	A	G	0.31	0.0436
rs10937277	LC_IGP25	IgG1 SA per Gal	3	186712711	3	3.31E-14	8.76E-20	-	-0.303694	-0.4018	+	A	G	0.296677632	0.039669	+	A	G	0.3103	0.0441

rs10937277	LC_IGP7	IgG1_G1FS1	3	186712711	3	1.48E-09	2.65E-26	-	-0.239189	-0.4867	+	A	G	0.298033028	0.039334	+	A	G	0.31	0.0459
rs10937277	LC_IGP_R34	IgG1_G1FS1/IgG1_G1F	3	186712711	3	4.28E-12	5.00E-42	-	-0.273483	-0.6248	+	A	G	0.298033028	0.0391813	+	A	G	0.31	0.046
rs10937277	LC_IGP_R35	IgG1_G2FS1/IgG1_G2F	3	186712711	3	7.25E-20	2.65E-25	-	-0.360778	-0.4648	+	A	G	0.297487179	0.0390341	+	A	G	0.31	0.0447
rs10937277	LC_IGP122	IgG2 FG1S1/(FG1+FG1S1)	3	186712711	3	1.64E-25	5.00E-46	-	-0.411169	-0.6518	+	A	G	0.297342282	0.038727	+	A	G	0.31	0.0458
rs62292620	LC_IGP123	IgG2 FG2S1/(FG2+FG2S1)	3	186713289	3	4.60E-22	7.60E-52	-	-0.395605	-0.6758	+	G	A	0.23965476	0.0403782	+	G	A	0.2798	0.0446
rs10937277	LC_IGP120	IgG2 FGS1/(FG+FGS1)	3	186712711	3	2.09E-17	8.11E-30	-	-0.336771	-0.5086	+	A	G	0.29752381	0.0392312	+	A	G	0.31	0.0448
rs10937277	LC_IGP111	IgG2 SA per Gal	3	186712711	3	6.62E-18	2.47E-36	-	-0.343485	-0.5775	+	A	G	0.297322601	0.0393769	+	A	G	0.3102	0.0459
rs10937277	LC_IGP93	IgG2_G1FS1	3	186712711	3	7.31E-16	2.18E-30	-	-0.320915	-0.5138	+	A	G	0.297342282	0.0393889	+	A	G	0.31	0.0448
rs10937277	LC_IGP_R74	IgG2_G1FS1/IgG2_G1F	3	186712711	3	1.28E-25	4.62E-46	-	-0.412076	-0.652	+	A	G	0.297342282	0.0387228	+	A	G	0.31	0.0458
rs10937277	LC_IGP_R75	IgG2_G2FS1/IgG2_G2F	3	186712711	3	3.93E-12	2.57E-36	-	-0.276059	-0.5754	+	A	G	0.297344417	0.039481	+	A	G	0.31	0.0457
rs10937277	LC_IGP189	IgG4 FG1S1/(FG1+FG1S1)	3	186712711	3	4.38E-13	1.26E-30	-	-0.287304	-0.5221	+	A	G	0.297156707	0.0393416	+	A	G	0.31	0.0454
rs10937277	LC_IGP190	IgG4 FG2S1/(FG2+FG2S1)	3	186712711	3	8.37E-13	1.02E-27	-	-0.285168	-0.4939	+	A	G	0.297338011	0.0395386	+	A	G	0.31	0.0453
rs10937277	LC_IGP187	IgG4 FGS1/(FG+FGS1)	3	186712711	3	1.80E-10	2.32E-15	-	-0.254129	-0.3475	+	A	G	0.297338011	0.0395939	+	A	G	0.31	0.0439
rs10937277	LC_IGP186	IgG4 SA per Gal	3	186712711	3	1.40E-14	8.94E-24	-	-0.30656	-0.454	+	A	G	0.297279376	0.0394649	+	A	G	0.3098	0.0452
rs10937277	LC_IGP179	IgG4_G1FS1	3	186712711	3	1.57E-08	7.54E-19	-	-0.224876	-0.3975	+	A	G	0.297156707	0.0395738	+	A	G	0.31	0.0448
rs10937277	LC_IGP_R92	IgG4_G1FS1/IgG4_G1F	3	186712711	3	4.16E-13	1.51E-30	-	-0.287537	-0.5214	+	A	G	0.297156707	0.0393359	+	A	G	0.31	0.0454
rs10937277	LC_IGP_R93	IgG4_G2FS1/IgG4_G2F	3	186712711	3	8.41E-13	1.04E-27	-	-0.285133	-0.4939	+	A	G	0.297338011	0.0395376	+	A	G	0.31	0.0453
chr3:186712737:I	LC_IGP36	IgG1 FG1S1/(FG1+FG1S1)	3	186712737	3	1.50E-21	7.21E-51	-	-0.395101	-0.7062	+	AAAC	A	0.240221759	0.0408562	+	AAAC	A	0.2675	0.0471
chr3:186712737:I	LC_IGP37	IgG1 FG2S1/(FG2+FG2S1)	3	186712737	3	2.06E-31	4.69E-32	-	-0.4832	-0.5395	+	AAAC	A	0.239781792	0.0405801	+	AAAC	A	0.2675	0.0458
chr3:186712737:I	LC_IGP34	IgG1 FGS1/(FG+FGS1)	3	186712737	3	1.76E-14	1.14E-15	-	-0.321299	-0.3585	+	AAAC	A	0.240221759	0.0415234	+	AAAC	A	0.2675	0.0448
chr3:186712737:I	LC_IGP25	IgG1 SA per Gal	3	186712737	3	3.31E-19	1.19E-23	-	-0.378766	-0.4533	+	AAAC	A	0.237948622	0.0417454	+	AAAC	A	0.2678	0.0452
chr3:186712737:I	LC_IGP7	IgG1_G1FS1	3	186712737	3	4.99E-17	4.63E-32	-	-0.348661	-0.5557	+	AAAC	A	0.240221759	0.0411182	+	AAAC	A	0.2675	0.0472
chr3:186712737:I	LC_IGP_R34	IgG1_G1FS1/IgG1_G1F	3	186712737	3	2.18E-21	9.09E-51	-	-0.393417	-0.7068	+	AAAC	A	0.240221759	0.0408534	+	AAAC	A	0.2675	0.0472
chr3:186712737:I	LC_IGP_R35	IgG1_G2FS1/IgG1_G2F	3	186712737	3	2.25E-31	4.35E-32	-	-0.482915	-0.5398	+	AAAC	A	0.239781792	0.0405821	+	AAAC	A	0.2675	0.0458
chr3:186712737:I	LC_IGP122	IgG2 FG1S1/(FG1+FG1S1)	3	186712737	3	3.71E-40	4.16E-60	-	-0.54646	-0.7672	+	AAAC	A	0.239671187	0.0400882	+	AAAC	A	0.2675	0.0469
rs13071014	LC_IGP123	IgG2 FG2S1/(FG2+FG2S1)	3	186713556	3	2.29E-22	3.85E-51	-	-0.398752	-0.6756	+	T	C	0.238635024	0.0403921	+	T	C	0.2729	0.0449
chr3:186712737:I	LC_IGP120	IgG2 FGS1/(FG+FGS1)	3	186712737	3	5.29E-24	6.28E-38	-	-0.421581	-0.5917	+	AAAC	A	0.239817507	0.0410675	+	AAAC	A	0.2675	0.046
chr3:186712737:I	LC_IGP111	IgG2 SA per Gal	3	186712737	3	1.88E-26	2.75E-48	-	-0.445084	-0.6859	+	AAAC	A	0.240018933	0.0410808	+	AAAC	A	0.2674	0.047
chr3:186712737:I	LC_IGP93	IgG2_G1FS1	3	186712737	3	2.39E-22	4.61E-37	-	-0.406948	-0.5862	+	AAAC	A	0.239671187	0.0412429	+	AAAC	A	0.2675	0.0461
chr3:186712737:I	LC_IGP_R74	IgG2_G1FS1/IgG2_G1F	3	186712737	3	3.14E-40	3.85E-60	-	-0.546965	-0.7674	+	AAAC	A	0.239671187	0.0400858	+	AAAC	A	0.2675	0.0469
chr3:186712737:I	LC_IGP_R75	IgG2_G2FS1/IgG2_G2F	3	186712737	3	7.87E-20	4.47E-48	-	-0.381062	-0.6824	+	AAAC	A	0.239671797	0.0412707	+	AAAC	A	0.2675	0.0468
chr3:186712737:I	LC_IGP189	IgG4 FG1S1/(FG1+FG1S1)	3	186712737	3	3.88E-23	1.05E-42	-	-0.412352	-0.6358	+	AAAC	A	0.239519559	0.0409937	+	AAAC	A	0.2675	0.0464
chr3:186712737:I	LC_IGP190	IgG4 FG2S1/(FG2+FG2S1)	3	186712737	3	2.21E-24	2.09E-40	-	-0.425668	-0.6172	+	AAAC	A	0.239665696	0.041108	+	AAAC	A	0.2675	0.0464
chr3:186712737:I	LC_IGP187	IgG4 FGS1/(FG+FGS1)	3	186712737	3	8.86E-17	2.75E-21	-	-0.348725	-0.4267	+	AAAC	A	0.239665696	0.0414697	+	AAAC	A	0.2675	0.045
chr3:186712737:I	LC_IGP186	IgG4 SA per Gal	3	186712737	3	1.14E-23	1.33E-33	-	-0.419633	-0.5609	+	AAAC	A	0.239338786	0.0411946	+	AAAC	A	0.2671	0.0464
chr3:186712737:I	LC_IGP179	IgG4_G1FS1	3	186712737	3	9.33E-15	1.90E-25	-	-0.324238	-0.4799	+	AAAC	A	0.239519559	0.0414581	+	AAAC	A	0.2675	0.046
chr3:186712737:I	LC_IGP_R92	IgG4_G1FS1/IgG4_G1F	3	186712737	3	4.22E-23	1.08E-42	-	-0.411963	-0.6358	+	AAAC	A	0.239519559	0.0409911	+	AAAC	A	0.2675	0.0464
chr3:186712737:I	LC_IGP_R93	IgG4_G2FS1/IgG4_G2F	3	186712737	3	2.27E-24	2.23E-40	-	-0.425551	-0.6171	+	AAAC	A	0.239665696	0.0411075	+	AAAC	A	0.2675	0.0464
rs35653970	LC_IGP36	IgG1 FG1S1/(FG1+FG1S1)	3	186712749	3	2.46E-21	1.21E-52	-	-0.39018	-0.7116	+	T	G	0.243480048	0.0405719	+	T	G	0.2726	0.0466
rs35653970	LC_IGP37	IgG1 FG2S1/(FG2+FG2S1)	3	186712749	3	5.33E-31	1.51E-32	-	-0.476492	-0.5389	+	T	G	0.243034114	0.0403086	+	T	G	0.2726	0.0454
rs35653970	LC_IGP34	IgG1 FGS1/(FG+FGS1)	3	186712749	3	2.02E-14	1.14E-15	-	-0.318255	-0.3555	+	T	G	0.243480048	0.0412256	+	T	G	0.2726	0.0444
rs35653970	LC_IGP25	IgG1 SA per Gal	3	186712749	3	8.74E-19	5.48E-24	-	-0.371513	-0.4532	+	T	G	0.241276553	0.0414566	+	T	G	0.2728	0.0449
rs35653970	LC_IGP7	IgG1_G1FS1	3	186712749	3	7.87E-17	1.52E-33	-	-0.343954	-0.5627	+	T	G	0.243480048	0.0408312	+	T	G	0.2726	0.0466
rs35653970	LC_IGP_R34	IgG1_G1FS1/IgG1_G1F	3	186712749	3	3.52E-21	1.77E-52	-	-0.388575	-0.7117	+	T	G	0.243480048	0.0405688	+	T	G	0.2726	0.0467
rs35653970	LC_IGP_R35	IgG1_G2FS1/IgG1_G2F	3	186712749	3	5.86E-31	1.40E-32	-	-0.476167	-0.5392	+	T	G	0.243034114	0.0403108	+	T	G	0.2726	0.0454
rs35653970	LC_IGP122	IgG2 FG1S1/(FG1+FG1S1)	3	186712749	3	4.32E-40	9.59E-62	-	-0.542054	-0.7718	+	T	G	0.242916643	0.0398012	+	T	G	0.2726	0.0466
rs35653970	LC_IGP123	IgG2 FG2S1/(FG2+FG2S1)	3	186712749	3	4.67E-20	4.48E-49	-	-0.38057	-0.6842	+	T	G	0.242917254	0.0409574	+	T	G	0.2726	0.0465
rs35653970	LC_IGP121	IgG2 FGS1/(F+FG+FGS1)	3	186712749	3	3.62E-08	1.15E-13	-	-0.23116	-0.3285	+	T	G	0.243183799	0.0417647	+	T	G	0.2726	0.0443
rs35653970	LC_IGP120	IgG2 FGS1/(FG+FGS1)	3	186712749	3	3.69E-24	8.24E-39	-	-0.41994	-0.5944	+	T	G	0.243064944	0.040761	+	T	G	0.2726	0.0456
rs35653970	LC_IGP111	IgG2 SA per Gal	3	186712749	3	1.46E-26	2.36E-49	-	-0.442803	-0.6885	+	T	G	0.243278562	0.0407775	+	T	G	0.2725	0.0466
rs35653970	LC_IGP93	IgG2_G1FS1	3	186712749	3	2.26E-22	3.54E-38	-	-0.404236	-0.5889	+	T	G	0.242916643	0.0409424	+	T	G	0.2726	0.0456
rs35653970	LC_IGP_R74	IgG2_G1FS1/IgG2_G1F	3	186712749	3	3.67E-40	8.81E-62	-	-0.54255	-0.772	+	T	G	0.242916643	0.0397989	+	T	G	0.2726	0.0465
rs35653970	LC_IGP_R75	IgG2_G2FS1/IgG2_G2F	3	186712749	3	4.74E-20	4.29E-49	-	-0.380517	-0.6844	+	T	G	0.242917254	0.0409589	+	T	G	0.2726	0.0465
rs35653970	LC_IGP189	IgG4 FG1S1/(FG1+FG1S1)	3	186712749	3	4.79E-23	4.94E-44	-	-0.408508	-0.64	+	T	G	0.242767914	0.040701	+	T	G	0.2726	0.046
rs35653970	LC_IGP190	IgG4 FG2S1/(FG2+FG2S1)	3	186712749	3	2.49E-24	8.97E-41	-	-0.4221	-0.6139	+	T	G	0.242916033	0.0408123	+	T	G	0.2726	0.0459
rs35653970	LC_IGP187	IgG4 FGS1/(FG+FGS1)	3	186712749	3	7.33E-17	1.19E-21	-	-0.347105	-0.4264	+	T	G	0.242916033	0.0411637	+	T	G	0.2726	0.0446
rs35653970	LC_IGP186	IgG4 SA per Gal	3	186712749	3	1.37E-23	2.10E-34	-	-0.415861	-0.5625	+	T	G	0.242594785	0.0408983	+	T	G	0.2722	0.046
rs35653970	LC_IGP179	IgG4_G1FS1	3	186712749	3	1.34E-14	1.12E-26	-	-0.320002	-0.487	+	T	G	0.242767914	0.0411657	+	T	G	0.2726	0.0456
rs35653970	LC_IGP_R92	IgG4_G1FS1/IgG4_G1F	3	186712749	3	5.18E-23	5.08E-44	-	-0.408144	-0.6399	+	T	G	0.242767914	0.0406982	+	T	G	0.2726	0.046
rs35653970	LC_IGP_R93	IgG4_G2FS1/IgG4_G2F	3	186712749	3	2.55E-24	9.56E-41	-	-0.421999	-0.6138	+	T	G	0.242916033	0.0408118	+	T	G	0.2726	0.0459
rs16848727	LC_IGP36	IgG1 FG1S1/(FG1+FG1S1)	3	186712963	3	4.92E-23	4.88E-55	-	-0.40497	-0.7058	+	G	A	0.238891976	0.0403577	+	G	A	0.2772	0.0452
rs16848727	LC_IGP37	IgG1 FG2S1/(FG2+FG2S1)	3	186712963	3	9.90E-34	1.19E-33	-	-0.495822	-0.5286	+	G	A	0.238454445	0.0400389	+	G	A	0.2772	0.0437
rs16848727	LC_IGP34	IgG1 FGS1/(FG+FGS1)	3	186712963	3	1.76E-15	1.80E-16	-	-0.32985	-0.3509	+	G	A	0.238891976	0.0410449	+	G	A	0.2772	0.0426
rs16848727	LC_IGP29	IgG1 G2S1/(G2+G2S1)	3	186712963	3	2.71E-08	9.37E-11	-	-0.233106	-0.2865	+	G	A	0.238155184	0.0417253	+	G	A	0.2772	0.0442
rs16848727	LC_IGP25	IgG1 SA per Gal	3	186712963	3	1.62E-20	2.00E-25	-	-0.388157	-0.4497	+	G	A	0.236598275	0.0412351	+	G	A	0.2775	0.0432
rs16848727	LC_IGP7	IgG1_G1FS1	3	186712963	3	9.44E-19	3.41E-36	-	-0.363393	-0.5671	+	G	A	0.238891976	0.0406034	+	G	A	0.2772	0.0451
rs16848727	LC_IGP_R34	IgG1_G1FS1/IgG1_G1F	3	186712963	3	6.97E-23	6.74E-55	-	-0.40346	-0.7061	+	G	A	0.238891976	0.0403544	+	G	A	0.2772	0.0452
rs16848727	LC_IGP_R35	IgG1_G2FS1/IgG1_G2F	3	186712963	3	1.06E-33	1.10E-33	-	-0.495594	-0.5289	+	G	A	0.238454445	0.0400405	+	G	A	0.2772	0.0437
rs16848727	LC_IGP_R33	IgG1_G2S1/IgG1_G2	3	186712963	3	2.84E-08	9.49E-11	-	-0.232788	-0.2864	+	G	A	0.238155184	0.0417301	+	G	A	0.2772	0.0442
rs16848727	LC_IGP122	IgG2 FG1S1/(FG1+FG1S1)	3	186712963	3	4.31E-42	7.70E-66	-	-0.553654	-0.7684	+	G	A	0.238339159	0.0395756	+	G	A	0.2772	0.0448
chr3:186712737:I	LC_IGP123	IgG2 FG2S1/(FG2+FG2S1)	3	186712737	3	7.67E-20	4.58E-48	-	-0.381166	-0.6823	+	AAAC	A	0.239671797	0.0412688	+	AAAC	A	0.2675	0.0468
rs16848727	LC_IGP121	IgG2 FGS1/(F+FG+FGS1)	3	186712963	3	2.49E-08	7.24E-15	-	-0.233214	-0.3317	+	G	A	0.238600416	0.0416345	+	G	A	0.2772	0.0426
rs16848727	LC_IGP120	IgG2 FGS1/(FG+FGS1)	3	186712963	3	4.24E-25	1.63E-41	-	-0.427094	-0.5924	+	G	A	0.238484665	0.04059	+	G	A	0.2772	0.0439
rs16848727	LC_IGP111	IgG2 SA per Gal	3	186712963	3	1.84E-28	5.14E-53	-	-0.45741	-0.6874	+	G	A	0.238636149	0.0405482	+	G	A	0.2775	0.0448
rs16848727	LC_IGP93	IgG2_G1FS1	3	186712963	3	1.50E-23	1.00E-41	-	-0.414007	-0.5937	+	G	A	0.238339159	0.0407576	+	G	A	0.2772	0.0439
rs16848727	LC_IGP_R74	IgG2_G1FS1/IgG2_G1F	3	186712963	3	3.65E-42	7.45E-66	-	-0.554145	-0.7685	+	G	A	0.238339159	0.0395733	+	G	A	0.2772	0.0448
rs16848727	LC_IGP_R75	IgG2_G2FS1/IgG2_G2F	3	186712963	3	2.10E-21	4.73E-52	-	-0.39272	-0.6791	+	G	A	0.238339159	0.0407641	+	G	A	0.2772	0.0447
rs16848727	LC_IGP189	IgG4 FG1S1/(FG1+FG1S1)	3	186712963	3	7.23E-24	3.25E-47	-	-0.414829	-0.6382	+	G	A	0.238193525	0.0405376	+	G	A	0.2772	0.0442
rs16848727	LC_IGP190	IgG4 FG2S1/(FG2+FG2S1)	3	186712963	3	7.08E-27	2.45E-42	-	-0.443093	-0.6012	+	G	A	0.238338853	0.0405504	+	G	A	0.2772	0.0441
rs16848727	LC_IGP187	IgG4 FGS1/(FG+FGS1)	3	186712963	3	1.28E-17	1.07E-22	-	-0.354392	-0.4199	+	G	A	0.238338853	0.0410018	+	G	A	0.2772	0.0428
rs16848727	LC_IGP186	IgG4 SA per Gal	3	186712963	3	4.35E-25	1.21E-36	-	-0.428136	-0.5573	+	G	A	0.238058985	0.0406977	+	G	A	0.277	0.0441
rs16848727	LC_IGP179	IgG4_G1FS1	3	186712963	3	1.34E-15	9.25E-30	-	-0.33083	-0.4977	+	G	A	0.238193525	0.0409906	+	G	A	0.2772	0.0439
rs16848727	LC_IGP_R92	IgG4_G1FS1/IgG4_G1F	3	186712963	3	7.12E-24	3.27E-47	-	-0.41484	-0.6382	+	G	A	0.238193525	0.0405326	+	G	A	0.2772	0.0442
rs16848727	LC_IGP_R93	IgG4_G2FS1/IgG4_G2F	3	186712963	3	7.22E-27	2.54E-42	-	-0.443008	-0.6011	+	G	A	0.238338853	0.0405497	+	G	A	0.2772	0.0441
rs55862858	LC_IGP36	IgG1 FG1S1/(FG1+FG1S1)	3	186713103	3	2.36E-23	3.91E-55	-	-0.406914	-0.7053	+	C	T	0.239697434	0.0402429	+	C	T	0.2771	0.0451
rs55862858	LC_IGP37	IgG1 FG2S1/(FG2+FG2S1)	3	186713103	3	2.35E-34	1.32E-33	-	-0.499215	-0.528	+	C	T	0.239258428	0.039908	+	C	T	0.2771	0.0437
rs55862858	LC_IGP34	IgG1 FGS1/(FG+FGS1)	3	186713103	3	6.73E-16	1.70E-16	-	-0.333843	-0.3507	+	C	T	0.239697434	0.0409227	+	C	T	0.2771	0.0426
rs55862858	LC_IGP29	IgG1 G2S1/(G2+G2S1)	3	186713103	3	1.43E-08	1.07E-10	-	-0.237116	-0.2853	+	C	T	0.238959844	0.0416089	+	C	T	0.2771	0.0442
rs55862858	LC_IGP25	IgG1 SA per Gal	3	186713103	3	5.39E-21	2.03E-25	-	-0.391916	-0.4492	+	C	T	0.237425943	0.0411047	+	C	T	0.2774	0.0431
rs55862858	LC_IGP7	IgG1_G1FS1	3	186713103	3	3.97E-19	3.43E-36	-	-0.36637	-0.5664	+	C	T	0.239697434	0.0404847	+	C	T	0.2771	0.0451
rs55862858	LC_IGP_R34	IgG1_G1FS1/IgG1_G1F	3	186713103	3	3.27E-23	5.19E-55	-	-0.405499	-0.7057	+	C	T	0.239697434	0.0402391	+	C	T	0.2771	0.0452
rs55862858	LC_IGP_R35	IgG1_G2FS1/IgG1_G2F	3	186713103	3	2.51E-34	1.23E-33	-	-0.499004	-0.5283	+	C	T	0.239258428	0.0399095	+	C	T	0.2771	0.0437
rs55862858	LC_IGP_R33	IgG1_G2S1/IgG1_G2	3	186713103	3	1.51E-08	1.08E-10	-	-0.236764	-0.2852	+	C	T	0.238959844	0.0416139	+	C	T	0.2771	0.0442
rs55862858	LC_IGP122	IgG2 FG1S1/(FG1+FG1S1)	3	186713103	3	1.95E-42	4.94E-66	-	-0.554547	-0.7681	+	C	T	0.239142346	0.0394616	+	C	T	0.2771	0.0448
rs7617523	LC_IGP123	IgG2 FG2S1/(FG2+FG2S1)	3	186722450	3	2.07E-21	8.36E-47	-	-0.352795	-0.5818	+	G	T	0.332871263	0.0366151	+	G	T	0.3476	0.0405
rs55862858	LC_IGP121	IgG2 FGS1/(F+FG+FGS1)	3	186713103	3	1.51E-08	5.98E-15	-	-0.236217	-0.332	+	C	T	0.239404584	0.0415223	+	C	T	0.2771	0.0425
rs55862858	LC_IGP120	IgG2 FGS1/(FG+FGS1)	3	186713103	3	1.44E-25	1.33E-41	-	-0.430167	-0.592	+	C	T	0.239288342	0.0404662	+	C	T	0.2771	0.0438
rs55862858	LC_IGP111	IgG2 SA per Gal	3	186713103	3	6.42E-29	3.82E-53	-	-0.460038	-0.6871	+	C	T	0.23945282	0.0404234	+	C	T	0.2773	0.0448
rs55862858	LC_IGP93	IgG2_G1FS1	3	186713103	3	9.37E-24	6.47E-42	-	-0.414881	-0.5941	+	C	T	0.239142346	0.0406482	+	C	T	0.2771	0.0438
rs55862858	LC_IGP_R74	IgG2_G1FS1/IgG2_G1F	3	186713103	3	1.66E-42	4.77E-66	-	-0.555021	-0.7681	+	C	T	0.239142346	0.0394594	+	C	T	0.2771	0.0448
rs55862858	LC_IGP_R75	IgG2_G2FS1/IgG2_G2F	3	186713103	3	6.99E-22	4.34E-52	-	-0.396338	-0.6784	+	C	T	0.239142346	0.0406392	+	C	T	0.2771	0.0447
rs55862858	LC_IGP189	IgG4 FG1S1/(FG1+FG1S1)	3	186713103	3	5.47E-24	7.50E-47	-	-0.414923	-0.6347	+	C	T	0.238996527	0.0404334	+	C	T	0.2771	0.0442
rs55862858	LC_IGP190	IgG4 FG2S1/(FG2+FG2S1)	3	186713103	3	3.03E-27	3.46E-42	-	-0.44513	-0.599	+	C	T	0.239142346	0.0404322	+	C	T	0.2771	0.044
rs55862858	LC_IGP187	IgG4 FGS1/(FG+FGS1)	3	186713103	3	6.43E-18	1.31E-22	-	-0.356732	-0.4181	+	C	T	0.239142346	0.0408864	+	C	T	0.2771	0.0427
rs55862858	LC_IGP186	IgG4 SA per Gal	3	186713103	3	2.60E-25	1.62E-36	-	-0.429067	-0.5553	+	C	T	0.238865547	0.0405868	+	C	T	0.2768	0.044
rs55862858	LC_IGP179	IgG4_G1FS1	3	186713103	3	7.79E-16	8.52E-30	-	-0.332719	-0.4971	+	C	T	0.238996527	0.0408789	+	C	T	0.2771	0.0438
rs55862858	LC_IGP_R92	IgG4_G1FS1/IgG4_G1F	3	186713103	3	5.42E-24	7.58E-47	-	-0.414911	-0.6347	+	C	T	0.238996527	0.0404286	+	C	T	0.2771	0.0442
rs55862858	LC_IGP_R93	IgG4_G2FS1/IgG4_G2F	3	186713103	3	3.09E-27	3.56E-42	-	-0.445045	-0.599	+	C	T	0.239142346	0.0404316	+	C	T	0.2771	0.044
rs62292620	LC_IGP36	IgG1 FG1S1/(FG1+FG1S1)	3	186713289	3	1.59E-23	3.86E-54	-	-0.405946	-0.6972	+	G	A	0.240210796	0.039986	+	G	A	0.2798	0.045
rs62292620	LC_IGP37	IgG1 FG2S1/(FG2+FG2S1)	3	186713289	3	1.78E-34	2.34E-33	-	-0.497015	-0.5251	+	G	A	0.23977085	0.0396562	+	G	A	0.2798	0.0436
rs62292620	LC_IGP34	IgG1 FGS1/(FG+FGS1)	3	186713289	3	7.97E-16	5.20E-17	-	-0.330958	-0.3558	+	G	A	0.240210796	0.0406753	+	G	A	0.2798	0.0424
rs62292620	LC_IGP29	IgG1 G2S1/(G2+G2S1)	3	186713289	3	1.73E-08	8.20E-11	-	-0.234324	-0.2858	+	G	A	0.239472892	0.0413568	+	G	A	0.2798	0.044
rs62292620	LC_IGP25	IgG1 SA per Gal	3	186713289	3	8.59E-21	1.15E-25	-	-0.387525	-0.4507	+	G	A	0.238053354	0.0408635	+	G	A	0.2801	0.043
rs62292620	LC_IGP7	IgG1_G1FS1	3	186713289	3	2.39E-19	2.28E-35	-	-0.36635	-0.5578	+	G	A	0.240210796	0.0402234	+	G	A	0.2798	0.0449
rs62292620	LC_IGP_R34	IgG1_G1FS1/IgG1_G1F	3	186713289	3	2.18E-23	5.19E-54	-	-0.404596	-0.6975	+	G	A	0.240210796	0.0399819	+	G	A	0.2798	0.0451
rs62292620	LC_IGP_R35	IgG1_G2FS1/IgG1_G2F	3	186713289	3	1.90E-34	2.18E-33	-	-0.496808	-0.5254	+	G	A	0.23977085	0.0396576	+	G	A	0.2798	0.0436
rs62292620	LC_IGP_R33	IgG1_G2S1/IgG1_G2	3	186713289	3	1.83E-08	8.25E-11	-	-0.233942	-0.2858	+	G	A	0.239472892	0.0413619	+	G	A	0.2798	0.044
rs62292620	LC_IGP122	IgG2 FG1S1/(FG1+FG1S1)	3	186713289	3	1.52E-42	1.36E-65	-	-0.551832	-0.764	+	G	A	0.23965476	0.0392134	+	G	A	0.2798	0.0447
rs6788832	LC_IGP123	IgG2 FG2S1/(FG2+FG2S1)	3	186724219	3	1.52E-21	1.96E-46	-	-0.353046	-0.577	+	G	A	0.333122636	0.0365126	+	G	A	0.3464	0.0403

rs62292620	LC_IGP121	IgG2 FGS1/(F+FG+FGS1)	3	186713289	3	1.52E-08	1.64E-15	-	-0.234743	-0.3387	+	G	A	0.239917319	0.0412672	+	G	A	0.2798	0.0425
rs62292620	LC_IGP120	IgG2 FGS1/(FG+FGS1)	3	186713289	3	1.26E-25	7.69E-42	-	-0.428032	-0.5931	+	G	A	0.23980107	0.0402143	+	G	A	0.2798	0.0438
rs62292620	LC_IGP111	IgG2 SA per Gal	3	186713289	3	5.23E-29	4.79E-53	-	-0.457876	-0.686	+	G	A	0.239990806	0.0401653	+	G	A	0.28	0.0448
rs62292620	LC_IGP93	IgG2_G1FS1	3	186713289	3	1.07E-23	3.54E-42	-	-0.411814	-0.5955	+	G	A	0.23965476	0.0404018	+	G	A	0.2798	0.0438
rs62292620	LC_IGP_R74	IgG2_G1FS1/IgG2_G1F	3	186713289	3	1.30E-42	1.30E-65	-	-0.552297	-0.7641	+	G	A	0.23965476	0.0392111	+	G	A	0.2798	0.0447
rs62292620	LC_IGP_R75	IgG2_G2FS1/IgG2_G2F	3	186713289	3	4.63E-22	7.43E-52	-	-0.395593	-0.6759	+	G	A	0.23965476	0.0403796	+	G	A	0.2798	0.0446
rs62292620	LC_IGP189	IgG4 FG1S1/(FG1+FG1S1)	3	186713289	3	1.84E-24	1.68E-46	-	-0.416585	-0.6314	+	G	A	0.239508629	0.0401587	+	G	A	0.2798	0.0441
rs62292620	LC_IGP190	IgG4 FG2S1/(FG2+FG2S1)	3	186713289	3	4.91E-27	3.35E-42	-	-0.440645	-0.5987	+	G	A	0.23965476	0.0401957	+	G	A	0.2798	0.044
rs62292620	LC_IGP187	IgG4 FGS1/(FG+FGS1)	3	186713289	3	6.45E-18	2.40E-23	-	-0.354525	-0.4248	+	G	A	0.23965476	0.0406354	+	G	A	0.2798	0.0427
rs62292620	LC_IGP186	IgG4 SA per Gal	3	186713289	3	1.78E-25	7.92E-37	-	-0.427868	-0.5575	+	G	A	0.239392688	0.0403278	+	G	A	0.2795	0.044
rs62292620	LC_IGP179	IgG4_G1FS1	3	186713289	3	2.28E-16	2.39E-29	-	-0.336668	-0.4928	+	G	A	0.239508629	0.0405978	+	G	A	0.2798	0.0438
rs62292620	LC_IGP_R92	IgG4_G1FS1/IgG4_G1F	3	186713289	3	1.86E-24	1.73E-46	-	-0.416491	-0.6314	+	G	A	0.239508629	0.0401543	+	G	A	0.2798	0.0441
rs62292620	LC_IGP_R93	IgG4_G2FS1/IgG4_G2F	3	186713289	3	5.03E-27	3.45E-42	-	-0.440549	-0.5987	+	G	A	0.23965476	0.0401951	+	G	A	0.2798	0.044
rs13071014	LC_IGP36	IgG1 FG1S1/(FG1+FG1S1)	3	186713556	3	2.37E-24	1.37E-54	-	-0.413644	-0.7063	+	T	C	0.239218229	0.0399731	+	T	C	0.2729	0.0454
rs13071014	LC_IGP37	IgG1 FG2S1/(FG2+FG2S1)	3	186713556	3	2.09E-35	2.91E-33	-	-0.504122	-0.5286	+	T	C	0.2387801	0.0396377	+	T	C	0.2729	0.044
rs13071014	LC_IGP34	IgG1 FGS1/(FG+FGS1)	3	186713556	3	2.80E-16	2.16E-16	-	-0.336348	-0.3518	+	T	C	0.239218229	0.0406835	+	T	C	0.2729	0.0428
rs13071014	LC_IGP29	IgG1 G2S1/(G2+G2S1)	3	186713556	3	1.12E-08	1.28E-10	-	-0.237598	-0.2857	+	T	C	0.238480626	0.0413811	+	T	C	0.2729	0.0444
rs13071014	LC_IGP25	IgG1 SA per Gal	3	186713556	3	2.10E-21	2.79E-25	-	-0.393814	-0.4508	+	T	C	0.237050943	0.0408625	+	T	C	0.2732	0.0434
rs13071014	LC_IGP7	IgG1_G1FS1	3	186713556	3	6.73E-20	7.36E-36	-	-0.372137	-0.5665	+	T	C	0.239218229	0.0402261	+	T	C	0.2729	0.0453
rs13071014	LC_IGP_R34	IgG1_G1FS1/IgG1_G1F	3	186713556	3	3.24E-24	1.73E-54	-	-0.41232	-0.7068	+	T	C	0.239218229	0.039969	+	T	C	0.2729	0.0455
rs13071014	LC_IGP_R35	IgG1_G2FS1/IgG1_G2F	3	186713556	3	2.27E-35	2.71E-33	-	-0.503865	-0.5289	+	T	C	0.2387801	0.0396396	+	T	C	0.2729	0.044
rs13071014	LC_IGP_R33	IgG1_G2S1/IgG1_G2	3	186713556	3	1.19E-08	1.30E-10	-	-0.237191	-0.2856	+	T	C	0.238480626	0.0413862	+	T	C	0.2729	0.0444
rs13071014	LC_IGP122	IgG2 FG1S1/(FG1+FG1S1)	3	186713556	3	2.20E-43	5.10E-65	-	-0.557584	-0.7663	+	T	C	0.238635024	0.0391973	+	T	C	0.2729	0.045
rs13098740	LC_IGP123	IgG2 FG2S1/(FG2+FG2S1)	3	186723150	3	1.93E-21	5.86E-45	-	-0.352752	-0.5689	+	G	A	0.332943258	0.0365809	+	G	A	0.3429	0.0404
rs13071014	LC_IGP121	IgG2 FGS1/(F+FG+FGS1)	3	186713556	3	1.04E-08	1.45E-14	-	-0.237553	-0.3293	+	T	C	0.238925965	0.0412926	+	T	C	0.2729	0.0428
rs13071014	LC_IGP120	IgG2 FGS1/(FG+FGS1)	3	186713556	3	3.36E-26	1.08E-40	-	-0.433269	-0.5885	+	T	C	0.238780711	0.0402129	+	T	C	0.2729	0.0441
rs13071014	LC_IGP111	IgG2 SA per Gal	3	186713556	3	1.70E-29	3.88E-52	-	-0.462154	-0.6843	+	T	C	0.239300188	0.0401704	+	T	C	0.2731	0.045
rs13071014	LC_IGP93	IgG2_G1FS1	3	186713556	3	4.67E-24	1.60E-41	-	-0.415373	-0.5951	+	T	C	0.238635024	0.0404125	+	T	C	0.2729	0.0441
rs13071014	LC_IGP_R74	IgG2_G1FS1/IgG2_G1F	3	186713556	3	1.89E-43	4.95E-65	-	-0.558023	-0.7663	+	T	C	0.238635024	0.0391953	+	T	C	0.2729	0.045
rs13071014	LC_IGP_R75	IgG2_G2FS1/IgG2_G2F	3	186713556	3	2.30E-22	3.75E-51	-	-0.398741	-0.6757	+	T	C	0.238635024	0.0403934	+	T	C	0.2729	0.0449
rs13071014	LC_IGP189	IgG4 FG1S1/(FG1+FG1S1)	3	186713556	3	6.35E-25	4.80E-47	-	-0.420962	-0.6386	+	T	C	0.238489515	0.0401637	+	T	C	0.2729	0.0443
rs13071014	LC_IGP190	IgG4 FG2S1/(FG2+FG2S1)	3	186713556	3	1.77E-27	4.55E-42	-	-0.444675	-0.6008	+	T	C	0.238635024	0.0402016	+	T	C	0.2729	0.0442
rs13071014	LC_IGP187	IgG4 FGS1/(FG+FGS1)	3	186713556	3	2.46E-18	8.64E-23	-	-0.359206	-0.4216	+	T	C	0.238635024	0.0406429	+	T	C	0.2729	0.0429
rs13071014	LC_IGP186	IgG4 SA per Gal	3	186713556	3	5.48E-26	8.96E-37	-	-0.432654	-0.5597	+	T	C	0.238413589	0.0403342	+	T	C	0.2727	0.0442
rs13071014	LC_IGP179	IgG4_G1FS1	3	186713556	3	1.88E-16	4.51E-30	-	-0.337845	-0.5016	+	T	C	0.238489515	0.0406243	+	T	C	0.2729	0.044
rs13071014	LC_IGP_R92	IgG4_G1FS1/IgG4_G1F	3	186713556	3	6.54E-25	4.84E-47	-	-0.420802	-0.6386	+	T	C	0.238489515	0.0401597	+	T	C	0.2729	0.0443
rs13071014	LC_IGP_R93	IgG4_G2FS1/IgG4_G2F	3	186713556	3	1.81E-27	4.71E-42	-	-0.444594	-0.6008	+	T	C	0.238635024	0.040201	+	T	C	0.2729	0.0442
rs4686827	LC_IGP36	IgG1 FG1S1/(FG1+FG1S1)	3	186713686	3	5.57E-11	1.40E-19	-	-0.32075	-0.5007	+	A	C	0.839234695	0.0486041	+	A	C	0.8458	0.0553
rs4686827	LC_IGP37	IgG1 FG2S1/(FG2+FG2S1)	3	186713686	3	8.78E-15	2.87E-13	-	-0.381094	-0.3905	+	A	C	0.83922694	0.0486779	+	A	C	0.8458	0.0535
rs4686827	LC_IGP25	IgG1 SA per Gal	3	186713686	3	1.56E-09	2.29E-11	-	-0.299103	-0.3519	+	A	C	0.837264784	0.0492449	+	A	C	0.8457	0.0526
rs4686827	LC_IGP7	IgG1_G1FS1	3	186713686	3	5.36E-10	3.35E-17	-	-0.304024	-0.4613	+	A	C	0.839234695	0.0486746	+	A	C	0.8458	0.0547
rs4686827	LC_IGP_R34	IgG1_G1FS1/IgG1_G1F	3	186713686	3	5.05E-11	1.33E-19	-	-0.321366	-0.5018	+	A	C	0.839234695	0.048587	+	A	C	0.8458	0.0554
rs4686827	LC_IGP_R35	IgG1_G2FS1/IgG1_G2F	3	186713686	3	9.41E-15	2.83E-13	-	-0.380669	-0.3906	+	A	C	0.83922694	0.0486798	+	A	C	0.8458	0.0535
rs4686827	LC_IGP122	IgG2 FG1S1/(FG1+FG1S1)	3	186713686	3	1.84E-16	1.66E-23	-	-0.404057	-0.5493	+	A	C	0.839323223	0.0485668	+	A	C	0.8458	0.055
rs10212190	LC_IGP123	IgG2 FG2S1/(FG2+FG2S1)	3	186731157	3	1.48E-21	2.27E-44	-	-0.351065	-0.5296	+	T	A	0.361832825	0.0362988	+	T	A	0.396	0.0379
rs4686827	LC_IGP120	IgG2 FGS1/(FG+FGS1)	3	186713686	3	2.99E-11	3.27E-14	-	-0.327906	-0.4085	+	A	C	0.83922513	0.0489917	+	A	C	0.8458	0.0538
rs4686827	LC_IGP111	IgG2 SA per Gal	3	186713686	3	2.25E-12	4.25E-20	-	-0.347848	-0.5045	+	A	C	0.839576523	0.0491781	+	A	C	0.8454	0.055
rs4686827	LC_IGP93	IgG2_G1FS1	3	186713686	3	1.39E-09	6.68E-18	-	-0.299754	-0.4591	+	A	C	0.839323223	0.0492034	+	A	C	0.8458	0.0533
rs4686827	LC_IGP_R74	IgG2_G1FS1/IgG2_G1F	3	186713686	3	1.87E-16	1.75E-23	-	-0.403959	-0.549	+	A	C	0.839323223	0.0485694	+	A	C	0.8458	0.055
rs4686827	LC_IGP_R75	IgG2_G2FS1/IgG2_G2F	3	186713686	3	1.66E-11	7.05E-19	-	-0.331711	-0.488	+	A	C	0.839021623	0.0489143	+	A	C	0.8458	0.055
rs4686827	LC_IGP190	IgG4 FG2S1/(FG2+FG2S1)	3	186713686	3	1.78E-08	6.98E-11	-	-0.27869	-0.3503	+	A	C	0.839021623	0.0492348	+	A	C	0.8458	0.0537
rs4686827	LC_IGP186	IgG4 SA per Gal	3	186713686	3	1.06E-08	7.05E-10	-	-0.282996	-0.3293	+	A	C	0.839146733	0.0492186	+	A	C	0.8458	0.0534
rs4686827	LC_IGP_R93	IgG4_G2FS1/IgG4_G2F	3	186713686	3	1.86E-08	6.91E-11	-	-0.27832	-0.3504	+	A	C	0.839021623	0.0492347	+	A	C	0.8458	0.0537
rs35376994	LC_IGP36	IgG1 FG1S1/(FG1+FG1S1)	3	186715319	3	5.19E-25	6.84E-56	-	-0.418767	-0.7053	+	A	G	0.238101669	0.0398748	+	A	G	0.2723	0.0448
rs35376994	LC_IGP37	IgG1 FG2S1/(FG2+FG2S1)	3	186715319	3	2.84E-36	9.79E-34	-	-0.509484	-0.526	+	A	G	0.237665585	0.039529	+	A	G	0.2723	0.0434
rs35376994	LC_IGP34	IgG1 FGS1/(FG+FGS1)	3	186715319	3	1.17E-16	1.25E-16	-	-0.34004	-0.3498	+	A	G	0.238101669	0.0405994	+	A	G	0.2723	0.0422
rs35376994	LC_IGP29	IgG1 G2S1/(G2+G2S1)	3	186715319	3	9.00E-09	1.89E-10	-	-0.238733	-0.28	+	A	G	0.237362609	0.041313	+	A	G	0.2723	0.044
rs35376994	LC_IGP25	IgG1 SA per Gal	3	186715319	3	7.44E-22	1.50E-25	-	-0.397608	-0.4475	+	A	G	0.235912111	0.0407816	+	A	G	0.2726	0.0428
rs35376994	LC_IGP7	IgG1_G1FS1	3	186715319	3	2.47E-20	8.51E-37	-	-0.375838	-0.5674	+	A	G	0.238101669	0.0401398	+	A	G	0.2723	0.0448
rs35376994	LC_IGP_R34	IgG1_G1FS1/IgG1_G1F	3	186715319	3	7.04E-25	9.10E-56	-	-0.417496	-0.7058	+	A	G	0.238101669	0.0398705	+	A	G	0.2723	0.0449
rs35376994	LC_IGP_R35	IgG1_G2FS1/IgG1_G2F	3	186715319	3	3.09E-36	9.13E-34	-	-0.509222	-0.5263	+	A	G	0.237665585	0.0395309	+	A	G	0.2723	0.0434
rs35376994	LC_IGP_R33	IgG1_G2S1/IgG1_G2	3	186715319	3	9.67E-09	1.91E-10	-	-0.238255	-0.2799	+	A	G	0.237362609	0.0413185	+	A	G	0.2723	0.044
rs35376994	LC_IGP122	IgG2 FG1S1/(FG1+FG1S1)	3	186715319	3	7.50E-45	4.64E-66	-	-0.56589	-0.7634	+	A	G	0.237520884	0.039057	+	A	G	0.2723	0.0445
rs17776120	LC_IGP123	IgG2 FG2S1/(FG2+FG2S1)	3	186732679	3	6.87E-21	1.67E-43	-	-0.344903	-0.5215	+	A	C	0.363533335	0.0362882	+	A	C	0.3992	0.0377
rs35376994	LC_IGP121	IgG2 FGS1/(F+FG+FGS1)	3	186715319	3	4.17E-09	6.25E-15	-	-0.243501	-0.3294	+	A	G	0.237810769	0.0412067	+	A	G	0.2723	0.0422
rs35376994	LC_IGP120	IgG2 FGS1/(FG+FGS1)	3	186715319	3	4.15E-27	1.01E-41	-	-0.440264	-0.589	+	A	G	0.237665891	0.0401003	+	A	G	0.2723	0.0435
rs35376994	LC_IGP111	IgG2 SA per Gal	3	186715319	3	1.31E-30	1.88E-53	-	-0.470233	-0.6847	+	A	G	0.238166086	0.0400464	+	A	G	0.2725	0.0445
rs35376994	LC_IGP110	IgG2 Sialylation	3	186715319	3	2.61E-08	5.93E-14	-	-0.231759	-0.3168	+	A	G	0.238051716	0.0414357	+	A	G	0.2726	0.0422
rs35376994	LC_IGP93	IgG2_G1FS1	3	186715319	3	4.54E-25	4.60E-42	-	-0.423691	-0.5909	+	A	G	0.237520884	0.0402938	+	A	G	0.2723	0.0435
rs35376994	LC_IGP_R74	IgG2_G1FS1/IgG2_G1F	3	186715319	3	6.38E-45	4.51E-66	-	-0.566348	-0.7634	+	A	G	0.237520884	0.0390548	+	A	G	0.2723	0.0445
rs35376994	LC_IGP_R75	IgG2_G2FS1/IgG2_G2F	3	186715319	3	5.00E-23	2.82E-52	-	-0.404251	-0.675	+	A	G	0.237520884	0.0402944	+	A	G	0.2723	0.0444
rs35376994	LC_IGP189	IgG4 FG1S1/(FG1+FG1S1)	3	186715319	3	1.54E-25	1.01E-47	-	-0.425646	-0.6358	+	A	G	0.237376054	0.040068	+	A	G	0.2723	0.0438
rs35376994	LC_IGP190	IgG4 FG2S1/(FG2+FG2S1)	3	186715319	3	3.36E-28	2.87E-42	-	-0.449941	-0.5951	+	A	G	0.237520884	0.0400996	+	A	G	0.2723	0.0437
rs35376994	LC_IGP187	IgG4 FGS1/(FG+FGS1)	3	186715319	3	6.82E-19	7.47E-23	-	-0.364423	-0.4174	+	A	G	0.237520884	0.0405493	+	A	G	0.2723	0.0424
rs35376994	LC_IGP186	IgG4 SA per Gal	3	186715319	3	9.78E-27	6.12E-37	-	-0.438372	-0.554	+	A	G	0.23731287	0.0402321	+	A	G	0.272	0.0436
rs35376994	LC_IGP179	IgG4_G1FS1	3	186715319	3	1.45E-16	4.13E-30	-	-0.338584	-0.4966	+	A	G	0.237376054	0.0405559	+	A	G	0.2723	0.0436
rs35376994	LC_IGP_R92	IgG4_G1FS1/IgG4_G1F	3	186715319	3	1.60E-25	1.02E-47	-	-0.425466	-0.6358	+	A	G	0.237376054	0.0400642	+	A	G	0.2723	0.0438
rs35376994	LC_IGP_R93	IgG4_G2FS1/IgG4_G2F	3	186715319	3	3.42E-28	2.96E-42	-	-0.449865	-0.595	+	A	G	0.237520884	0.040099	+	A	G	0.2723	0.0437
rs3868902	LC_IGP36	IgG1 FG1S1/(FG1+FG1S1)	3	186715887	3	4.95E-16	5.06E-45	-	-0.306988	-0.6091	+	T	G	0.309773089	0.037454	+	T	G	0.3227	0.0433
rs3868902	LC_IGP37	IgG1 FG2S1/(FG2+FG2S1)	3	186715887	3	1.35E-24	3.99E-27	-	-0.387701	-0.452	+	T	G	0.309205739	0.0372604	+	T	G	0.3227	0.0419
rs3868902	LC_IGP34	IgG1 FGS1/(FG+FGS1)	3	186715887	3	7.14E-13	3.04E-15	-	-0.2739	-0.3213	+	T	G	0.309773089	0.0378585	+	T	G	0.3227	0.0407
rs3868902	LC_IGP25	IgG1 SA per Gal	3	186715887	3	7.21E-17	1.02E-21	-	-0.320783	-0.3952	+	T	G	0.308119674	0.0380216	+	T	G	0.323	0.0413
rs3868902	LC_IGP7	IgG1_G1FS1	3	186715887	3	9.41E-13	1.41E-27	-	-0.270774	-0.4699	+	T	G	0.309773089	0.0376283	+	T	G	0.3227	0.0432
rs3868902	LC_IGP_R34	IgG1_G1FS1/IgG1_G1F	3	186715887	3	5.77E-16	6.41E-45	-	-0.306207	-0.6095	+	T	G	0.309773089	0.0374465	+	T	G	0.3227	0.0433
rs3868902	LC_IGP_R35	IgG1_G2FS1/IgG1_G2F	3	186715887	3	1.51E-24	3.74E-27	-	-0.387303	-0.4523	+	T	G	0.309205739	0.0372628	+	T	G	0.3227	0.0419
rs3868902	LC_IGP122	IgG2 FG1S1/(FG1+FG1S1)	3	186715887	3	1.38E-31	8.40E-50	-	-0.440654	-0.6353	+	T	G	0.309017694	0.0368948	+	T	G	0.3227	0.0428
rs3821819	LC_IGP123	IgG2 FG2S1/(FG2+FG2S1)	3	186732725	3	7.05E-21	1.68E-43	-	-0.34478	-0.5221	+	A	G	0.363556132	0.0362865	+	A	G	0.3991	0.0378
rs3868902	LC_IGP121	IgG2 FGS1/(F+FG+FGS1)	3	186715887	3	7.01E-09	7.07E-13	-	-0.222539	-0.2928	+	T	G	0.309394624	0.0382284	+	T	G	0.3227	0.0408
rs3868902	LC_IGP120	IgG2 FGS1/(FG+FGS1)	3	186715887	3	4.30E-21	5.38E-32	-	-0.358354	-0.4939	+	T	G	0.309206349	0.0375005	+	T	G	0.3227	0.042
rs3868902	LC_IGP111	IgG2 SA per Gal	3	186715887	3	1.06E-22	2.37E-39	-	-0.374097	-0.5625	+	T	G	0.309457585	0.037576	+	T	G	0.3229	0.0429
rs3868902	LC_IGP110	IgG2 Sialylation	3	186715887	3	9.95E-09	1.25E-11	-	-0.221385	-0.276	+	T	G	0.309194873	0.0384238	+	T	G	0.3229	0.0408
rs3868902	LC_IGP93	IgG2_G1FS1	3	186715887	3	4.44E-19	6.39E-33	-	-0.340466	-0.501	+	T	G	0.309017694	0.0376763	+	T	G	0.3227	0.0419
rs3868902	LC_IGP_R74	IgG2_G1FS1/IgG2_G1F	3	186715887	3	1.12E-31	8.00E-50	-	-0.441323	-0.6354	+	T	G	0.309017694	0.0368915	+	T	G	0.3227	0.0428
rs3868902	LC_IGP_R75	IgG2_G2FS1/IgG2_G2F	3	186715887	3	5.17E-16	2.71E-38	-	-0.309038	-0.5533	+	T	G	0.309018914	0.0377302	+	T	G	0.3227	0.0428
rs3868902	LC_IGP189	IgG4 FG1S1/(FG1+FG1S1)	3	186715887	3	1.12E-15	1.46E-35	-	-0.304815	-0.5252	+	T	G	0.308830488	0.0376637	+	T	G	0.3227	0.0422
rs3868902	LC_IGP190	IgG4 FG2S1/(FG2+FG2S1)	3	186715887	3	1.43E-16	5.64E-30	-	-0.315553	-0.4785	+	T	G	0.309018914	0.0377905	+	T	G	0.3227	0.0421
rs3868902	LC_IGP187	IgG4 FGS1/(FG+FGS1)	3	186715887	3	2.66E-12	1.93E-17	-	-0.267449	-0.3473	+	T	G	0.309018914	0.0379454	+	T	G	0.3227	0.0409
rs3868902	LC_IGP186	IgG4 SA per Gal	3	186715887	3	1.74E-17	7.01E-27	-	-0.325101	-0.4508	+	T	G	0.30903213	0.0377748	+	T	G	0.3226	0.042
rs3868902	LC_IGP179	IgG4_G1FS1	3	186715887	3	1.98E-10	2.46E-21	-	-0.242849	-0.3983	+	T	G	0.308830488	0.0379232	+	T	G	0.3227	0.042
rs3868902	LC_IGP_R92	IgG4_G1FS1/IgG4_G1F	3	186715887	3	1.04E-15	1.70E-35	-	-0.305142	-0.5248	+	T	G	0.308830488	0.0376575	+	T	G	0.3227	0.0422
rs3868902	LC_IGP_R93	IgG4_G2FS1/IgG4_G2F	3	186715887	3	1.44E-16	5.66E-30	-	-0.315519	-0.4786	+	T	G	0.309018914	0.0377896	+	T	G	0.3227	0.0421
rs13322676	LC_IGP36	IgG1 FG1S1/(FG1+FG1S1)	3	186718745	3	4.92E-27	4.77E-59	-	-0.429722	-0.6993	+	C	A	0.244569419	0.0391979	+	C	A	0.2769	0.0432
rs13322676	LC_IGP37	IgG1 FG2S1/(FG2+FG2S1)	3	186718745	3	3.70E-39	1.31E-35	-	-0.521759	-0.5216	+	C	A	0.24412149	0.0388104	+	C	A	0.2769	0.0419
rs13322676	LC_IGP34	IgG1 FGS1/(FG+FGS1)	3	186718745	3	1.67E-17	5.25E-18	-	-0.34426	-0.3518	+	C	A	0.244569419	0.0399762	+	C	A	0.2769	0.0407
rs13322676	LC_IGP29	IgG1 G2S1/(G2+G2S1)	3	186718745	3	3.09E-08	9.10E-10	-	-0.22673	-0.2592	+	C	A	0.243822433	0.0407541	+	C	A	0.2769	0.0423
rs13322676	LC_IGP25	IgG1 SA per Gal	3	186718745	3	1.55E-22	2.95E-27	-	-0.398329	-0.4463	+	C	A	0.242212235	0.040166	+	C	A	0.2772	0.0413
rs13322676	LC_IGP7	IgG1_G1FS1	3	186718745	3	1.04E-20	2.58E-38	-	-0.374093	-0.5542	+	C	A	0.244569419	0.0395495	+	C	A	0.2769	0.0428
rs13322676	LC_IGP_R34	IgG1_G1FS1/IgG1_G1F	3	186718745	3	6.33E-27	6.90E-59	-	-0.428697	-0.6995	+	C	A	0.244569419	0.0391924	+	C	A	0.2769	0.0432
rs13322676	LC_IGP_R35	IgG1_G2FS1/IgG1_G2F	3	186718745	3	3.85E-39	1.24E-35	-	-0.521639	-0.5218	+	C	A	0.24412149	0.0388113	+	C	A	0.2769	0.0419
rs13322676	LC_IGP_R33	IgG1_G2S1/IgG1_G2	3	186718745	3	3.33E-08	9.18E-10	-	-0.2262	-0.2591	+	C	A	0.243822433	0.0407595	+	C	A	0.2769	0.0423
rs13322676	LC_IGP122	IgG2 FG1S1/(FG1+FG1S1)	3	186718745	3	2.62E-48	1.80E-69	-	-0.578425	-0.7543	+	C	A	0.243972544	0.038316	+	C	A	0.2769	0.0428
rs967367	LC_IGP123	IgG2 FG2S1/(FG2+FG2S1)	3	186734466	3	9.68E-21	5.04E-43	-	-0.344265	-0.5219	+	A	G	0.363388041	0.0363656	+	A	G	0.3986	0.038
rs13322676	LC_IGP121	IgG2 FGS1/(F+FG+FGS1)	3	186718745	3	1.52E-09	3.88E-16	-	-0.246694	-0.3311	+	C	A	0.244270617	0.0405971	+	C	A	0.2769	0.0407
rs13322676	LC_IGP120	IgG2 FGS1/(FG+FGS1)	3	186718745	3	2.45E-29	1.45E-44	-	-0.451916	-0.5875	+	C	A	0.24412149	0.0394078	+	C	A	0.2769	0.042
rs13322676	LC_IGP111	IgG2 SA per Gal	3	186718745	3	7.46E-34	6.89E-57	-	-0.488328	-0.6803	+	C	A	0.244253251	0.0393421	+	C	A	0.2772	0.0428
rs13322676	LC_IGP110	IgG2 Sialylation	3	186718745	3	7.40E-09	4.28E-15	-	-0.237481	-0.3185	+	C	A	0.244343422	0.0408567	+	C	A	0.2773	0.0406
rs13322676	LC_IGP93	IgG2_G1FS1	3	186718745	3	2.72E-26	3.57E-44	-	-0.428065	-0.5812	+	C	A	0.243972544	0.0396535	+	C	A	0.2769	0.0417
rs13322676	LC_IGP_R74	IgG2_G1FS1/IgG2_G1F	3	186718745	3	2.24E-48	1.75E-69	-	-0.578841	-0.7544	+	C	A	0.243972544	0.038314	+	C	A	0.2769	0.0428
rs13322676	LC_IGP_R75	IgG2_G2FS1/IgG2_G2F	3	186718745	3	8.33E-26	1.38E-55	-	-0.422771	-0.6717	+	C	A	0.243973764	0.0395683	+	C	A	0.2769	0.0428

rs13322676	LC_IGP189	IgG4 FG1S1/(FG1+FG1S1)	3	186718745	3	1.54E-26	4.19E-49	-	-0.428	-0.6202	+	C	A	0.243825	0.0394434	+	C	A	0.2769	0.0421
rs13322676	LC_IGP190	IgG4 FG2S1/(FG2+FG2S1)	3	186718745	3	8.05E-29	1.00E-43	-	-0.448507	-0.5813	+	C	A	0.243973764	0.0394957	+	C	A	0.2769	0.0419
rs13322676	LC_IGP187	IgG4 FGS1/(FG+FGS1)	3	186718745	3	6.57E-19	2.33E-23	-	-0.359406	-0.4058	+	C	A	0.243973764	0.0399723	+	C	A	0.2769	0.0408
rs13322676	LC_IGP186	IgG4 SA per Gal	3	186718745	3	3.03E-27	7.95E-38	-	-0.43633	-0.5383	+	C	A	0.243794982	0.0396302	+	C	A	0.2765	0.0419
rs13322676	LC_IGP179	IgG4_G1FS1	3	186718745	3	2.72E-16	8.23E-31	-	-0.330781	-0.483	+	C	A	0.243825	0.0399946	+	C	A	0.2769	0.0418
rs13322676	LC_IGP_R92	IgG4_G1FS1/IgG4_G1F	3	186718745	3	1.49E-26	4.28E-49	-	-0.428056	-0.6202	+	C	A	0.243825	0.0394381	+	C	A	0.2769	0.0421
rs13322676	LC_IGP_R93	IgG4_G2FS1/IgG4_G2F	3	186718745	3	8.19E-29	1.04E-43	-	-0.448437	-0.5813	+	C	A	0.243973764	0.039495	+	C	A	0.2769	0.0419
rs6808800	LC_IGP36	IgG1 FG1S1/(FG1+FG1S1)	3	186719374	3	7.43E-11	3.21E-30	-	-0.227059	-0.4461	+	G	T	0.499497859	0.0346374	+	G	T	0.4996	0.0391
rs6808800	LC_IGP37	IgG1 FG2S1/(FG2+FG2S1)	3	186719374	3	1.28E-17	1.98E-19	-	-0.298482	-0.3415	+	G	T	0.498888278	0.0345325	+	G	T	0.4996	0.0379
rs6808800	LC_IGP34	IgG1 FGS1/(FG+FGS1)	3	186719374	3	2.66E-09	9.75E-11	-	-0.209064	-0.238	+	G	T	0.499497859	0.0349331	+	G	T	0.4996	0.0368
rs6808800	LC_IGP25	IgG1 SA per Gal	3	186719374	3	6.62E-12	7.98E-16	-	-0.242301	-0.3005	+	G	T	0.49648872	0.0350259	+	G	T	0.4996	0.0373
rs6808800	LC_IGP7	IgG1_G1FS1	3	186719374	3	8.89E-09	1.71E-19	-	-0.200826	-0.3496	+	G	T	0.499497859	0.0347398	+	G	T	0.4996	0.0387
rs6808800	LC_IGP_R34	IgG1_G1FS1/IgG1_G1F	3	186719374	3	7.75E-11	3.92E-30	-	-0.226774	-0.4462	+	G	T	0.499497859	0.0346281	+	G	T	0.4996	0.0391
rs6808800	LC_IGP_R35	IgG1_G2FS1/IgG1_G2F	3	186719374	3	1.17E-17	1.89E-19	-	-0.298813	-0.3417	+	G	T	0.498888278	0.0345307	+	G	T	0.4996	0.0379
rs6808800	LC_IGP122	IgG2 FG1S1/(FG1+FG1S1)	3	186719374	3	2.41E-26	2.33E-31	-	-0.368752	-0.4512	+	G	T	0.498888956	0.0341215	+	G	T	0.4996	0.0387
rs5001409	LC_IGP123	IgG2 FG2S1/(FG2+FG2S1)	3	186735690	3	2.50E-20	1.96E-42	-	-0.340801	-0.5205	+	C	A	0.364716595	0.0364037	+	C	A	0.3995	0.0381
rs6808800	LC_IGP121	IgG2 FGS1/(F+FG+FGS1)	3	186719374	3	8.27E-09	1.96E-07	-	-0.203227	-0.1916	+	G	T	0.498887599	0.0350808	+	G	T	0.4996	0.0368
rs6808800	LC_IGP120	IgG2 FGS1/(FG+FGS1)	3	186719374	3	9.28E-19	1.01E-19	-	-0.309108	-0.3449	+	G	T	0.498888278	0.0345311	+	G	T	0.4996	0.038
rs6808800	LC_IGP111	IgG2 SA per Gal	3	186719374	3	2.53E-19	7.81E-26	-	-0.31552	-0.4076	+	G	T	0.500146749	0.0346625	+	G	T	0.5004	0.0388
rs6808800	LC_IGP110	IgG2 Sialylation	3	186719374	3	6.92E-09	5.73E-07	-	-0.205182	-0.1842	+	G	T	0.499924645	0.0352314	+	G	T	0.5004	0.0368
rs6808800	LC_IGP93	IgG2_G1FS1	3	186719374	3	5.65E-17	4.62E-21	-	-0.293631	-0.3557	+	G	T	0.498888956	0.0346914	+	G	T	0.4996	0.0378
rs6808800	LC_IGP_R74	IgG2_G1FS1/IgG2_G1F	3	186719374	3	2.01E-26	2.29E-31	-	-0.369344	-0.4512	+	G	T	0.498888956	0.034119	+	G	T	0.4996	0.0387
rs6808800	LC_IGP_R75	IgG2_G2FS1/IgG2_G2F	3	186719374	3	1.13E-13	1.23E-25	-	-0.260088	-0.405	+	G	T	0.498585722	0.0347307	+	G	T	0.4996	0.0387
rs6808800	LC_IGP189	IgG4 FG1S1/(FG1+FG1S1)	3	186719374	3	2.26E-11	2.24E-19	-	-0.234208	-0.3429	+	G	T	0.498586585	0.0347731	+	G	T	0.4996	0.0381
rs6808800	LC_IGP190	IgG4 FG2S1/(FG2+FG2S1)	3	186719374	3	2.06E-10	9.95E-20	-	-0.223855	-0.3449	+	G	T	0.498890786	0.0349916	+	G	T	0.4996	0.038
rs6808800	LC_IGP187	IgG4 FGS1/(FG+FGS1)	3	186719374	3	1.10E-08	1.79E-10	-	-0.201104	-0.2353	+	G	T	0.498890786	0.035011	+	G	T	0.4996	0.0369
rs6808800	LC_IGP186	IgG4 SA per Gal	3	186719374	3	1.30E-11	2.82E-16	-	-0.238121	-0.31	+	G	T	0.498883108	0.0349276	+	G	T	0.4998	0.0379
rs6808800	LC_IGP179	IgG4_G1FS1	3	186719374	3	1.85E-08	9.58E-11	-	-0.197298	-0.2451	+	G	T	0.498586585	0.0348994	+	G	T	0.4996	0.0379
rs6808800	LC_IGP_R92	IgG4_G1FS1/IgG4_G1F	3	186719374	3	2.11E-11	2.58E-19	-	-0.234512	-0.3423	+	G	T	0.498586585	0.0347678	+	G	T	0.4996	0.0381
rs6808800	LC_IGP_R93	IgG4_G2FS1/IgG4_G2F	3	186719374	3	2.06E-10	1.00E-19	-	-0.223835	-0.345	+	G	T	0.498890786	0.0349907	+	G	T	0.4996	0.038
rs7621438	LC_IGP36	IgG1 FG1S1/(FG1+FG1S1)	3	186720309	3	6.57E-11	5.34E-31	-	-0.227413	-0.4535	+	G	A	0.499065138	0.0345924	+	G	A	0.4961	0.0392
rs7621438	LC_IGP37	IgG1 FG2S1/(FG2+FG2S1)	3	186720309	3	9.45E-18	1.14E-19	-	-0.299296	-0.3442	+	G	A	0.498456349	0.0344841	+	G	A	0.4961	0.0379
rs7621438	LC_IGP34	IgG1 FGS1/(FG+FGS1)	3	186720309	3	2.07E-09	7.99E-11	-	-0.210237	-0.2395	+	G	A	0.499065138	0.034885	+	G	A	0.4961	0.0368
rs7621438	LC_IGP25	IgG1 SA per Gal	3	186720309	3	4.89E-12	6.16E-16	-	-0.243504	-0.3021	+	G	A	0.496033835	0.0349769	+	G	A	0.4961	0.0374
rs7621438	LC_IGP7	IgG1_G1FS1	3	186720309	3	7.94E-09	5.81E-20	-	-0.201235	-0.3549	+	G	A	0.499065138	0.0346949	+	G	A	0.4961	0.0388
rs7621438	LC_IGP_R34	IgG1_G1FS1/IgG1_G1F	3	186720309	3	6.86E-11	6.48E-31	-	-0.227126	-0.4536	+	G	A	0.499065138	0.0345831	+	G	A	0.4961	0.0392
rs7621438	LC_IGP_R35	IgG1_G2FS1/IgG1_G2F	3	186720309	3	8.67E-18	1.09E-19	-	-0.299634	-0.3445	+	G	A	0.498456349	0.0344822	+	G	A	0.4961	0.0379
rs7621438	LC_IGP122	IgG2 FG1S1/(FG1+FG1S1)	3	186720309	3	1.70E-26	1.48E-32	-	-0.369398	-0.4614	+	G	A	0.498457291	0.0340725	+	G	A	0.4961	0.0388
rs9863411	LC_IGP123	IgG2 FG2S1/(FG2+FG2S1)	3	186737820	3	5.10E-20	8.01E-42	-	-0.337954	-0.5177	+	T	C	0.364296522	0.0364091	+	T	C	0.3976	0.0382
rs7621438	LC_IGP121	IgG2 FGS1/(F+FG+FGS1)	3	186720309	3	6.59E-09	8.38E-08	-	-0.204308	-0.1978	+	G	A	0.498455406	0.0350332	+	G	A	0.4961	0.0369
rs7621438	LC_IGP120	IgG2 FGS1/(FG+FGS1)	3	186720309	3	6.46E-19	1.54E-20	-	-0.310109	-0.3535	+	G	A	0.498456349	0.0344813	+	G	A	0.4961	0.038
rs7621438	LC_IGP111	IgG2 SA per Gal	3	186720309	3	1.72E-19	1.30E-26	-	-0.316582	-0.4152	+	G	A	0.499704954	0.0346128	+	G	A	0.4969	0.0389
rs7621438	LC_IGP110	IgG2 Sialylation	3	186720309	3	5.28E-09	2.92E-07	-	-0.206516	-0.1894	+	G	A	0.499474985	0.0351825	+	G	A	0.4969	0.0369
rs7621438	LC_IGP93	IgG2_G1FS1	3	186720309	3	4.17E-17	9.38E-22	-	-0.294498	-0.3628	+	G	A	0.498457291	0.0346427	+	G	A	0.4961	0.0379
rs7621438	LC_IGP_R74	IgG2_G1FS1/IgG2_G1F	3	186720309	3	1.41E-26	1.45E-32	-	-0.369987	-0.4614	+	G	A	0.498457291	0.0340701	+	G	A	0.4961	0.0388
rs7621438	LC_IGP_R75	IgG2_G2FS1/IgG2_G2F	3	186720309	3	8.78E-14	1.71E-26	-	-0.260922	-0.4134	+	G	A	0.498154057	0.0346831	+	G	A	0.4961	0.0388
rs7621438	LC_IGP189	IgG4 FG1S1/(FG1+FG1S1)	3	186720309	3	2.60E-11	6.20E-20	-	-0.233206	-0.349	+	G	A	0.498155183	0.0347337	+	G	A	0.4961	0.0382
rs7621438	LC_IGP190	IgG4 FG2S1/(FG2+FG2S1)	3	186720309	3	2.08E-10	9.05E-20	-	-0.223529	-0.3462	+	G	A	0.498459121	0.0349491	+	G	A	0.4961	0.038
rs7621438	LC_IGP187	IgG4 FGS1/(FG+FGS1)	3	186720309	3	1.01E-08	1.56E-10	-	-0.201355	-0.2367	+	G	A	0.498459121	0.0349665	+	G	A	0.4961	0.037
rs7621438	LC_IGP186	IgG4 SA per Gal	3	186720309	3	1.19E-11	2.07E-16	-	-0.238265	-0.3121	+	G	A	0.498451346	0.0348832	+	G	A	0.4963	0.038
rs7621438	LC_IGP179	IgG4_G1FS1	3	186720309	3	2.00E-08	4.01E-11	-	-0.196606	-0.2506	+	G	A	0.498155183	0.0348584	+	G	A	0.4961	0.0379
rs7621438	LC_IGP_R92	IgG4_G1FS1/IgG4_G1F	3	186720309	3	2.44E-11	7.17E-20	-	-0.233504	-0.3484	+	G	A	0.498155183	0.0347284	+	G	A	0.4961	0.0382
rs7621438	LC_IGP_R93	IgG4_G2FS1/IgG4_G2F	3	186720309	3	2.08E-10	9.10E-20	-	-0.22351	-0.3462	+	G	A	0.498459121	0.0349482	+	G	A	0.4961	0.038
rs7643789	LC_IGP36	IgG1 FG1S1/(FG1+FG1S1)	3	186720335	3	8.10E-11	5.32E-31	-	-0.22592	-0.4536	+	C	G	0.498208563	0.0345336	+	C	G	0.496	0.0392
rs7643789	LC_IGP37	IgG1 FG2S1/(FG2+FG2S1)	3	186720335	3	1.08E-17	1.14E-19	-	-0.298225	-0.3443	+	C	G	0.497601343	0.0344243	+	C	G	0.496	0.0379
rs7643789	LC_IGP34	IgG1 FGS1/(FG+FGS1)	3	186720335	3	1.97E-09	7.88E-11	-	-0.210112	-0.2396	+	C	G	0.498208563	0.0348204	+	C	G	0.496	0.0368
rs7643789	LC_IGP25	IgG1 SA per Gal	3	186720335	3	4.62E-12	6.07E-16	-	-0.243337	-0.3022	+	C	G	0.495156015	0.0349115	+	C	G	0.496	0.0374
rs7643789	LC_IGP7	IgG1_G1FS1	3	186720335	3	8.30E-09	5.83E-20	-	-0.200608	-0.355	+	C	G	0.498208563	0.0346325	+	C	G	0.496	0.0388
rs7643789	LC_IGP_R34	IgG1_G1FS1/IgG1_G1F	3	186720335	3	8.47E-11	6.46E-31	-	-0.225622	-0.4537	+	C	G	0.498208563	0.0345244	+	C	G	0.496	0.0392
rs7643789	LC_IGP_R35	IgG1_G2FS1/IgG1_G2F	3	186720335	3	9.88E-18	1.08E-19	-	-0.298578	-0.3445	+	C	G	0.497601343	0.0344223	+	C	G	0.496	0.0379
rs7643789	LC_IGP122	IgG2 FG1S1/(FG1+FG1S1)	3	186720335	3	1.59E-26	1.45E-32	-	-0.368939	-0.4615	+	C	G	0.497602807	0.0340093	+	C	G	0.496	0.0388
rs10937280	LC_IGP123	IgG2 FG2S1/(FG2+FG2S1)	3	186738033	3	5.51E-20	1.08E-41	-	-0.337586	-0.5169	+	A	G	0.364279744	0.0364038	+	A	G	0.3975	0.0382
rs7643789	LC_IGP121	IgG2 FGS1/(F+FG+FGS1)	3	186720335	3	5.19E-09	8.23E-08	-	-0.205333	-0.1979	+	C	G	0.497599878	0.0349646	+	C	G	0.496	0.0369
rs7643789	LC_IGP120	IgG2 FGS1/(FG+FGS1)	3	186720335	3	4.83E-19	1.50E-20	-	-0.310639	-0.3536	+	C	G	0.497601343	0.0344127	+	C	G	0.496	0.038
rs7643789	LC_IGP111	IgG2 SA per Gal	3	186720335	3	1.37E-19	1.28E-26	-	-0.316842	-0.4154	+	C	G	0.498837461	0.034544	+	C	G	0.4968	0.0389
rs7643789	LC_IGP110	IgG2 Sialylation	3	186720335	3	4.27E-09	2.88E-07	-	-0.20737	-0.1895	+	C	G	0.498609636	0.0351133	+	C	G	0.4968	0.0369
rs7643789	LC_IGP93	IgG2_G1FS1	3	186720335	3	2.90E-17	9.15E-22	-	-0.295412	-0.363	+	C	G	0.497602807	0.0345723	+	C	G	0.496	0.0379
rs7643789	LC_IGP_R74	IgG2_G1FS1/IgG2_G1F	3	186720335	3	1.32E-26	1.42E-32	-	-0.369523	-0.4616	+	C	G	0.497602807	0.0340069	+	C	G	0.496	0.0388
rs7643789	LC_IGP_R75	IgG2_G2FS1/IgG2_G2F	3	186720335	3	6.12E-14	1.69E-26	-	-0.262075	-0.4135	+	C	G	0.497299573	0.0346128	+	C	G	0.496	0.0388
rs7643789	LC_IGP189	IgG4 FG1S1/(FG1+FG1S1)	3	186720335	3	2.66E-11	6.02E-20	-	-0.232664	-0.3492	+	C	G	0.49730122	0.0346713	+	C	G	0.496	0.0382
rs7643789	LC_IGP190	IgG4 FG2S1/(FG2+FG2S1)	3	186720335	3	1.67E-10	8.74E-20	-	-0.224274	-0.3464	+	C	G	0.497604637	0.0348811	+	C	G	0.496	0.038
rs7643789	LC_IGP187	IgG4 FGS1/(FG+FGS1)	3	186720335	3	8.25E-09	1.50E-10	-	-0.202188	-0.2369	+	C	G	0.497604637	0.0348988	+	C	G	0.496	0.037
rs7643789	LC_IGP186	IgG4 SA per Gal	3	186720335	3	9.47E-12	2.00E-16	-	-0.238974	-0.3123	+	C	G	0.497594246	0.0348149	+	C	G	0.4963	0.038
rs7643789	LC_IGP179	IgG4_G1FS1	3	186720335	3	1.56E-08	4.01E-11	-	-0.197742	-0.2506	+	C	G	0.49730122	0.0347901	+	C	G	0.496	0.038
rs7643789	LC_IGP_R92	IgG4_G1FS1/IgG4_G1F	3	186720335	3	2.48E-11	6.95E-20	-	-0.232989	-0.3486	+	C	G	0.49730122	0.0346658	+	C	G	0.496	0.0382
rs7643789	LC_IGP_R93	IgG4_G2FS1/IgG4_G2F	3	186720335	3	1.67E-10	8.78E-20	-	-0.224274	-0.3464	+	C	G	0.497604637	0.0348801	+	C	G	0.496	0.038
rs7643952	LC_IGP36	IgG1 FG1S1/(FG1+FG1S1)	3	186720448	3	7.29E-11	4.42E-31	-	-0.226584	-0.4543	+	C	G	0.498440673	0.0345495	+	C	G	0.497	0.0392
rs7643952	LC_IGP37	IgG1 FG2S1/(FG2+FG2S1)	3	186720448	3	8.99E-18	1.49E-19	-	-0.299102	-0.3434	+	C	G	0.497833028	0.0344384	+	C	G	0.497	0.038
rs7643952	LC_IGP34	IgG1 FGS1/(FG+FGS1)	3	186720448	3	1.73E-09	4.89E-11	-	-0.21095	-0.2423	+	C	G	0.498440673	0.0348359	+	C	G	0.497	0.0368
rs7643952	LC_IGP25	IgG1 SA per Gal	3	186720448	3	3.96E-12	4.59E-16	-	-0.24422	-0.3035	+	C	G	0.495392544	0.0349267	+	C	G	0.497	0.0374
rs7643952	LC_IGP7	IgG1_G1FS1	3	186720448	3	8.28E-09	6.37E-20	-	-0.200727	-0.3547	+	C	G	0.498440673	0.0346506	+	C	G	0.497	0.0388
rs7643952	LC_IGP_R34	IgG1_G1FS1/IgG1_G1F	3	186720448	3	7.61E-11	5.49E-31	-	-0.226291	-0.4543	+	C	G	0.498440673	0.0345403	+	C	G	0.497	0.0392
rs7643952	LC_IGP_R35	IgG1_G2FS1/IgG1_G2F	3	186720448	3	8.22E-18	1.42E-19	-	-0.299455	-0.3437	+	C	G	0.497833028	0.0344365	+	C	G	0.497	0.038
rs7643952	LC_IGP122	IgG2 FG1S1/(FG1+FG1S1)	3	186720448	3	1.32E-26	4.32E-32	-	-0.369701	-0.458	+	C	G	0.49783435	0.0340232	+	C	G	0.497	0.0388
rs7634389	LC_IGP123	IgG2 FG2S1/(FG2+FG2S1)	3	186738421	3	6.41E-20	1.85E-41	-	-0.336848	-0.5155	+	C	T	0.364245882	0.0363912	+	C	T	0.3973	0.0382
rs7643952	LC_IGP121	IgG2 FGS1/(F+FG+FGS1)	3	186720448	3	4.88E-09	9.69E-08	-	-0.205787	-0.1969	+	C	G	0.497831704	0.0349816	+	C	G	0.497	0.0369
rs7643952	LC_IGP120	IgG2 FGS1/(FG+FGS1)	3	186720448	3	4.19E-19	3.25E-20	-	-0.311341	-0.3505	+	C	G	0.497833028	0.0344277	+	C	G	0.497	0.0381
rs7643952	LC_IGP111	IgG2 SA per Gal	3	186720448	3	1.18E-19	3.33E-26	-	-0.317587	-0.4119	+	C	G	0.499071827	0.0345593	+	C	G	0.4978	0.0389
rs7643952	LC_IGP110	IgG2 Sialylation	3	186720448	3	3.98E-09	3.38E-07	-	-0.207889	-0.1884	+	C	G	0.498842187	0.0351304	+	C	G	0.4978	0.0369
rs7643952	LC_IGP93	IgG2_G1FS1	3	186720448	3	2.70E-17	1.34E-21	-	-0.295853	-0.3615	+	C	G	0.49783435	0.0345888	+	C	G	0.497	0.0379
rs7643952	LC_IGP_R74	IgG2_G1FS1/IgG2_G1F	3	186720448	3	1.10E-26	4.27E-32	-	-0.370285	-0.458	+	C	G	0.49783435	0.0340208	+	C	G	0.497	0.0388
rs7643952	LC_IGP_R75	IgG2_G2FS1/IgG2_G2F	3	186720448	3	6.03E-14	3.70E-26	-	-0.262279	-0.4108	+	C	G	0.497530811	0.0346304	+	C	G	0.497	0.0388
rs7643952	LC_IGP189	IgG4 FG1S1/(FG1+FG1S1)	3	186720448	3	2.40E-11	1.02E-19	-	-0.233305	-0.347	+	C	G	0.497532317	0.0346871	+	C	G	0.497	0.0382
rs7643952	LC_IGP190	IgG4 FG2S1/(FG2+FG2S1)	3	186720448	3	1.65E-10	1.48E-19	-	-0.224464	-0.3443	+	C	G	0.497835876	0.034899	+	C	G	0.497	0.0381
rs7643952	LC_IGP187	IgG4 FGS1/(FG+FGS1)	3	186720448	3	7.70E-09	2.20E-10	-	-0.202696	-0.2348	+	C	G	0.497835876	0.0349155	+	C	G	0.497	0.037
rs7643952	LC_IGP186	IgG4 SA per Gal	3	186720448	3	8.55E-12	3.31E-16	-	-0.239607	-0.3101	+	C	G	0.497826499	0.0348309	+	C	G	0.4972	0.038
rs7643952	LC_IGP179	IgG4_G1FS1	3	186720448	3	1.47E-08	4.01E-11	-	-0.198183	-0.2507	+	C	G	0.497532317	0.034807	+	C	G	0.497	0.038
rs7643952	LC_IGP_R92	IgG4_G1FS1/IgG4_G1F	3	186720448	3	2.24E-11	1.17E-19	-	-0.233637	-0.3465	+	C	G	0.497532317	0.0346817	+	C	G	0.497	0.0382
rs7643952	LC_IGP_R93	IgG4_G2FS1/IgG4_G2F	3	186720448	3	1.65E-10	1.49E-19	-	-0.224463	-0.3443	+	C	G	0.497835876	0.034898	+	C	G	0.497	0.0381
rs3852061	LC_IGP36	IgG1 FG1S1/(FG1+FG1S1)	3	186721820	3	5.60E-11	5.17E-31	-	-0.228093	-0.45	+	G	T	0.499279205	0.0345677	+	G	T	0.4992	0.0388
rs3852061	LC_IGP37	IgG1 FG2S1/(FG2+FG2S1)	3	186721820	3	7.74E-18	1.28E-19	-	-0.299893	-0.3412	+	G	T	0.498670024	0.0344586	+	G	T	0.4992	0.0377
rs3852061	LC_IGP34	IgG1 FGS1/(FG+FGS1)	3	186721820	3	1.79E-09	1.25E-10	-	-0.210919	-0.2353	+	G	T	0.499279205	0.0348604	+	G	T	0.4992	0.0366
rs3852061	LC_IGP25	IgG1 SA per Gal	3	186721820	3	4.21E-12	9.71E-16	-	-0.244089	-0.2978	+	G	T	0.496235276	0.0349526	+	G	T	0.4992	0.0371
rs3852061	LC_IGP7	IgG1_G1FS1	3	186721820	3	7.45E-09	5.75E-20	-	-0.201481	-0.3522	+	G	T	0.499279205	0.0346721	+	G	T	0.4992	0.0385
rs3852061	LC_IGP_R34	IgG1_G1FS1/IgG1_G1F	3	186721820	3	5.85E-11	6.22E-31	-	-0.227802	-0.4501	+	G	T	0.499279205	0.0345585	+	G	T	0.4992	0.0389
rs3852061	LC_IGP_R35	IgG1_G2FS1/IgG1_G2F	3	186721820	3	7.11E-18	1.22E-19	-	-0.300221	-0.3414	+	G	T	0.498670024	0.0344568	+	G	T	0.4992	0.0377
rs3852061	LC_IGP122	IgG2 FG1S1/(FG1+FG1S1)	3	186721820	3	1.58E-26	6.04E-32	-	-0.369391	-0.4531	+	G	T	0.498670836	0.03405	+	G	T	0.4992	0.0385
rs3868902	LC_IGP123	IgG2 FG2S1/(FG2+FG2S1)	3	186715887	3	4.87E-16	2.69E-38	-	-0.309301	-0.5533	+	T	G	0.309018914	0.0377278	+	T	G	0.3227	0.0428
rs3852061	LC_IGP121	IgG2 FGS1/(F+FG+FGS1)	3	186721820	3	6.58E-09	2.12E-07	-	-0.20419	-0.1905	+	G	T	0.498669212	0.0350115	+	G	T	0.4992	0.0367
rs3852061	LC_IGP120	IgG2 FGS1/(FG+FGS1)	3	186721820	3	5.85E-19	6.22E-20	-	-0.310293	-0.3452	+	G	T	0.498670024	0.034458	+	G	T	0.4992	0.0378
rs3852061	LC_IGP111	IgG2 SA per Gal	3	186721820	3	1.39E-19	4.50E-26	-	-0.317216	-0.4074	+	G	T	0.499914861	0.0345893	+	G	T	0.5001	0.0386
rs3852061	LC_IGP110	IgG2 Sialylation	3	186721820	3	5.10E-09	7.01E-07	-	-0.206592	-0.1823	+	G	T	0.49966924	0.0351607	+	G	T	0.5	0.0367
rs3852061	LC_IGP93	IgG2_G1FS1	3	186721820	3	4.04E-17	3.46E-21	-	-0.294445	-0.3553	+	G	T	0.498670836	0.0346206	+	G	T	0.4992	0.0376
rs3852061	LC_IGP_R74	IgG2_G1FS1/IgG2_G1F	3	186721820	3	1.32E-26	5.94E-32	-	-0.369962	-0.4531	+	G	T	0.498670836	0.0340477	+	G	T	0.4992	0.0385
rs3852061	LC_IGP_R75	IgG2_G2FS1/IgG2_G2F	3	186721820	3	7.50E-14	4.71E-26	-	-0.261474	-0.4062	+	G	T	0.498366992	0.0346583	+	G	T	0.4992	0.0385
rs3852061	LC_IGP189	IgG4 FG1S1/(FG1+FG1S1)	3	186721820	3	2.12E-11	1.48E-19	-	-0.2341	-0.3427	+	G	T	0.498367988	0.0347079	+	G	T	0.4992	0.0379
rs3852061	LC_IGP190	IgG4 FG2S1/(FG2+FG2S1)	3	186721820	3	2.03E-10	1.07E-19	-	-0.223504	-0.3429	+	G	T	0.498672056	0.034927	+	G	T	0.4992	0.0378
rs3852061	LC_IGP187	IgG4 FGS1/(FG+FGS1)	3	186721820	3	9.22E-09	2.52E-10	-	-0.201777	-0.2323	+	G	T	0.498672056	0.0349429	+	G	T	0.4992	0.0367
rs3852061	LC_IGP186	IgG4 SA per Gal	3	186721820	3	9.65E-12	3.81E-16	-	-0.239169	-0.307	+	G	T	0.498666769	0.0348573	+	G	T	0.4995	0.0377

rs3852061	LC_IGP179	IgG4_G1FS1	3	186721820	3	1.59E-08	8.69E-11	-	-0.197834	-0.2445	+	G	T	0.498367988	0.0348321	+	G	T	0.4992	0.0377
rs3852061	LC_IGP_R92	IgG4_G1FS1/IgG4_G1F	3	186721820	3	1.99E-11	1.70E-19	-	-0.234384	-0.3421	+	G	T	0.498367988	0.0347026	+	G	T	0.4992	0.0379
rs3852061	LC_IGP_R93	IgG4_G2FS1/IgG4_G2F	3	186721820	3	2.04E-10	1.07E-19	-	-0.223485	-0.3429	+	G	T	0.498672056	0.034926	+	G	T	0.4992	0.0378
rs3864109	LC_IGP36	IgG1 FG1S1/(FG1+FG1S1)	3	186721867	3	5.56E-11	3.84E-32	-	-0.228122	-0.4648	+	G	A	0.499285015	0.0345666	+	G	A	0.4935	0.0394
rs3864109	LC_IGP37	IgG1 FG2S1/(FG2+FG2S1)	3	186721867	3	7.68E-18	1.66E-20	-	-0.299913	-0.3541	+	G	A	0.498675824	0.0344575	+	G	A	0.4935	0.0382
rs3864109	LC_IGP34	IgG1 FGS1/(FG+FGS1)	3	186721867	3	1.78E-09	2.37E-11	-	-0.210941	-0.2471	+	G	A	0.499285015	0.0348593	+	G	A	0.4935	0.037
rs3864109	LC_IGP25	IgG1 SA per Gal	3	186721867	3	4.19E-12	1.79E-16	-	-0.24411	-0.309	+	G	A	0.496240915	0.0349515	+	G	A	0.4935	0.0375
rs3864109	LC_IGP7	IgG1_G1FS1	3	186721867	3	7.43E-09	3.14E-20	-	-0.201489	-0.3594	+	G	A	0.499285015	0.0346711	+	G	A	0.4935	0.039
rs3864109	LC_IGP_R34	IgG1_G1FS1/IgG1_G1F	3	186721867	3	5.81E-11	4.47E-32	-	-0.22783	-0.4651	+	G	A	0.499285015	0.0345573	+	G	A	0.4935	0.0394
rs3864109	LC_IGP_R35	IgG1_G2FS1/IgG1_G2F	3	186721867	3	7.06E-18	1.58E-20	-	-0.30024	-0.3543	+	G	A	0.498675824	0.0344557	+	G	A	0.4935	0.0382
rs3864109	LC_IGP122	IgG2 FG1S1/(FG1+FG1S1)	3	186721867	3	1.57E-26	6.82E-33	-	-0.369396	-0.4662	+	G	A	0.498676632	0.0340489	+	G	A	0.4935	0.039
rs10937277	LC_IGP123	IgG2 FG2S1/(FG2+FG2S1)	3	186712711	3	3.71E-12	2.65E-36	-	-0.27636	-0.5752	+	A	G	0.297344417	0.0394786	+	A	G	0.31	0.0457
rs3864109	LC_IGP121	IgG2 FGS1/(F+FG+FGS1)	3	186721867	3	6.58E-09	9.06E-08	-	-0.204186	-0.1982	+	G	A	0.498675015	0.0350105	+	G	A	0.4935	0.0371
rs3864109	LC_IGP120	IgG2 FGS1/(FG+FGS1)	3	186721867	3	5.82E-19	7.77E-21	-	-0.310304	-0.358	+	G	A	0.498675824	0.0344569	+	G	A	0.4935	0.0382
rs3864109	LC_IGP111	IgG2 SA per Gal	3	186721867	3	1.37E-19	5.55E-27	-	-0.317244	-0.4203	+	G	A	0.499920743	0.0345882	+	G	A	0.4943	0.0391
rs3864109	LC_IGP110	IgG2 Sialylation	3	186721867	3	5.10E-09	3.37E-07	-	-0.206593	-0.1893	+	G	A	0.499674799	0.0351597	+	G	A	0.4943	0.0371
rs3864109	LC_IGP93	IgG2_G1FS1	3	186721867	3	4.03E-17	1.38E-21	-	-0.294438	-0.3629	+	G	A	0.498676632	0.0346196	+	G	A	0.4935	0.038
rs3864109	LC_IGP_R74	IgG2_G1FS1/IgG2_G1F	3	186721867	3	1.32E-26	6.77E-33	-	-0.369966	-0.4662	+	G	A	0.498676632	0.0340466	+	G	A	0.4935	0.039
rs3864109	LC_IGP_R75	IgG2_G2FS1/IgG2_G2F	3	186721867	3	7.44E-14	4.57E-27	-	-0.261502	-0.4201	+	G	A	0.498372788	0.0346571	+	G	A	0.4935	0.039
rs3864109	LC_IGP189	IgG4 FG1S1/(FG1+FG1S1)	3	186721867	3	2.10E-11	1.38E-20	-	-0.234132	-0.357	+	G	A	0.49837378	0.0347067	+	G	A	0.4935	0.0384
rs3864109	LC_IGP190	IgG4 FG2S1/(FG2+FG2S1)	3	186721867	3	2.04E-10	1.13E-20	-	-0.223492	-0.3568	+	G	A	0.498677852	0.034926	+	G	A	0.4935	0.0383
rs3864109	LC_IGP187	IgG4 FGS1/(FG+FGS1)	3	186721867	3	9.20E-09	6.78E-11	-	-0.201785	-0.2425	+	G	A	0.498677852	0.0349418	+	G	A	0.4935	0.0372
rs3864109	LC_IGP186	IgG4 SA per Gal	3	186721867	3	9.59E-12	6.50E-17	-	-0.239195	-0.3191	+	G	A	0.498672583	0.0348562	+	G	A	0.4937	0.0382
rs3864109	LC_IGP179	IgG4_G1FS1	3	186721867	3	1.58E-08	1.80E-11	-	-0.197873	-0.2565	+	G	A	0.49837378	0.0348309	+	G	A	0.4935	0.0382
rs3864109	LC_IGP_R92	IgG4_G1FS1/IgG4_G1F	3	186721867	3	1.98E-11	1.59E-20	-	-0.234415	-0.3564	+	G	A	0.49837378	0.0347014	+	G	A	0.4935	0.0384
rs3864109	LC_IGP_R93	IgG4_G2FS1/IgG4_G2F	3	186721867	3	2.04E-10	1.14E-20	-	-0.223473	-0.3568	+	G	A	0.498677852	0.0349251	+	G	A	0.4935	0.0383
rs12490407	LC_IGP36	IgG1 FG1S1/(FG1+FG1S1)	3	186721996	3	5.48E-11	5.26E-31	-	-0.228157	-0.4496	+	A	G	0.499379817	0.0345599	+	A	G	0.4994	0.0388
rs12490407	LC_IGP37	IgG1 FG2S1/(FG2+FG2S1)	3	186721996	3	7.44E-18	1.28E-19	-	-0.299982	-0.3411	+	A	G	0.498770452	0.0344505	+	A	G	0.4994	0.0376
rs12490407	LC_IGP34	IgG1 FGS1/(FG+FGS1)	3	186721996	3	1.74E-09	1.20E-10	-	-0.211011	-0.2355	+	A	G	0.499379817	0.0348525	+	A	G	0.4994	0.0366
rs12490407	LC_IGP25	IgG1 SA per Gal	3	186721996	3	4.07E-12	9.39E-16	-	-0.244205	-0.2979	+	A	G	0.496336153	0.0349444	+	A	G	0.4994	0.0371
rs12490407	LC_IGP7	IgG1_G1FS1	3	186721996	3	7.39E-09	5.85E-20	-	-0.201489	-0.352	+	A	G	0.499379817	0.0346646	+	A	G	0.4994	0.0385
rs12490407	LC_IGP_R34	IgG1_G1FS1/IgG1_G1F	3	186721996	3	5.72E-11	6.32E-31	-	-0.227867	-0.4497	+	A	G	0.499379817	0.0345507	+	A	G	0.4994	0.0389
rs12490407	LC_IGP_R35	IgG1_G2FS1/IgG1_G2F	3	186721996	3	6.85E-18	1.22E-19	-	-0.300308	-0.3413	+	A	G	0.498770452	0.0344487	+	A	G	0.4994	0.0376
rs12490407	LC_IGP122	IgG2 FG1S1/(FG1+FG1S1)	3	186721996	3	1.50E-26	5.88E-32	-	-0.369481	-0.4531	+	A	G	0.498771202	0.0340417	+	A	G	0.4994	0.0385
rs7619989	LC_IGP123	IgG2 FG2S1/(FG2+FG2S1)	3	186731749	3	2.90E-14	1.40E-27	-	-0.272373	-0.4194	+	C	G	0.602530506	0.0355042	+	C	G	0.6482	0.0385
rs12490407	LC_IGP121	IgG2 FGS1/(F+FG+FGS1)	3	186721996	3	6.46E-09	1.97E-07	-	-0.204258	-0.1909	+	A	G	0.498769701	0.0350037	+	A	G	0.4994	0.0367
rs12490407	LC_IGP120	IgG2 FGS1/(FG+FGS1)	3	186721996	3	5.55E-19	5.76E-20	-	-0.31042	-0.3455	+	A	G	0.498770452	0.0344496	+	A	G	0.4994	0.0378
rs12490407	LC_IGP111	IgG2 SA per Gal	3	186721996	3	1.31E-19	4.20E-26	-	-0.317363	-0.4077	+	A	G	0.500013622	0.0345808	+	A	G	0.5002	0.0386
rs12490407	LC_IGP110	IgG2 Sialylation	3	186721996	3	4.97E-09	6.50E-07	-	-0.206702	-0.1828	+	A	G	0.499768376	0.0351528	+	A	G	0.5002	0.0367
rs12490407	LC_IGP93	IgG2_G1FS1	3	186721996	3	3.88E-17	3.22E-21	-	-0.294537	-0.3555	+	A	G	0.498771202	0.0346125	+	A	G	0.4994	0.0376
rs12490407	LC_IGP_R74	IgG2_G1FS1/IgG2_G1F	3	186721996	3	1.25E-26	5.78E-32	-	-0.37005	-0.4532	+	A	G	0.498771202	0.0340394	+	A	G	0.4994	0.0385
rs12490407	LC_IGP_R75	IgG2_G2FS1/IgG2_G2F	3	186721996	3	7.13E-14	4.47E-26	-	-0.261645	-0.4065	+	A	G	0.498467358	0.0346499	+	A	G	0.4994	0.0385
rs12490407	LC_IGP189	IgG4 FG1S1/(FG1+FG1S1)	3	186721996	3	2.05E-11	1.46E-19	-	-0.234201	-0.3425	+	A	G	0.498468293	0.0346999	+	A	G	0.4994	0.0379
rs12490407	LC_IGP190	IgG4 FG2S1/(FG2+FG2S1)	3	186721996	3	1.96E-10	1.03E-19	-	-0.223654	-0.3429	+	A	G	0.498772422	0.0349188	+	A	G	0.4994	0.0377
rs12490407	LC_IGP187	IgG4 FGS1/(FG+FGS1)	3	186721996	3	8.87E-09	2.39E-10	-	-0.20196	-0.2325	+	A	G	0.498772422	0.0349346	+	A	G	0.4994	0.0367
rs12490407	LC_IGP186	IgG4 SA per Gal	3	186721996	3	9.21E-12	3.61E-16	-	-0.239354	-0.3071	+	A	G	0.498767748	0.0348489	+	A	G	0.4997	0.0377
rs12490407	LC_IGP179	IgG4_G1FS1	3	186721996	3	1.55E-08	8.62E-11	-	-0.197965	-0.2444	+	A	G	0.498468293	0.0348241	+	A	G	0.4994	0.0377
rs12490407	LC_IGP_R92	IgG4_G1FS1/IgG4_G1F	3	186721996	3	1.93E-11	1.68E-19	-	-0.23448	-0.342	+	A	G	0.498468293	0.0346946	+	A	G	0.4994	0.0379
rs12490407	LC_IGP_R93	IgG4_G2FS1/IgG4_G2F	3	186721996	3	1.97E-10	1.04E-19	-	-0.223635	-0.3429	+	A	G	0.498772422	0.0349179	+	A	G	0.4994	0.0377
rs17775791	LC_IGP36	IgG1 FG1S1/(FG1+FG1S1)	3	186722362	3	1.20E-27	3.23E-61	-	-0.434121	-0.7057	+	C	T	0.244182569	0.0391125	+	C	T	0.2752	0.0428
rs17775791	LC_IGP37	IgG1 FG2S1/(FG2+FG2S1)	3	186722362	3	8.61E-40	1.23E-36	-	-0.52523	-0.5244	+	C	T	0.243735348	0.0387249	+	C	T	0.2752	0.0415
rs17775791	LC_IGP34	IgG1 FGS1/(FG+FGS1)	3	186722362	3	9.88E-18	4.47E-18	-	-0.34619	-0.3491	+	C	T	0.244182569	0.0399107	+	C	T	0.2752	0.0403
rs17775791	LC_IGP29	IgG1 G2S1/(G2+G2S1)	3	186722362	3	3.05E-08	4.45E-10	-	-0.226512	-0.2614	+	C	T	0.243431846	0.0407013	+	C	T	0.2752	0.0419
rs17775791	LC_IGP25	IgG1 SA per Gal	3	186722362	3	6.61E-23	1.47E-27	-	-0.401281	-0.4445	+	C	T	0.241797087	0.0400993	+	C	T	0.2756	0.0409
rs17775791	LC_IGP7	IgG1_G1FS1	3	186722362	3	6.32E-21	1.18E-39	-	-0.375662	-0.5585	+	C	T	0.244182569	0.0394853	+	C	T	0.2752	0.0424
rs17775791	LC_IGP_R34	IgG1_G1FS1/IgG1_G1F	3	186722362	3	1.55E-27	4.55E-61	-	-0.433096	-0.706	+	C	T	0.244182569	0.0391071	+	C	T	0.2752	0.0428
rs17775791	LC_IGP_R35	IgG1_G2FS1/IgG1_G2F	3	186722362	3	9.01E-40	1.16E-36	-	-0.5251	-0.5246	+	C	T	0.243735348	0.0387259	+	C	T	0.2752	0.0415
rs17775791	LC_IGP_R33	IgG1_G2S1/IgG1_G2	3	186722362	3	3.32E-08	4.49E-10	-	-0.225938	-0.2614	+	C	T	0.243431846	0.0407069	+	C	T	0.2752	0.0419
rs17775791	LC_IGP122	IgG2 FG1S1/(FG1+FG1S1)	3	186722362	3	6.21E-49	4.92E-71	-	-0.58128	-0.7557	+	C	T	0.243586638	0.0382321	+	C	T	0.2752	0.0424
rs3872722	LC_IGP123	IgG2 FG2S1/(FG2+FG2S1)	3	186726277	3	2.93E-14	1.77E-27	-	-0.271653	-0.4147	+	A	C	0.602277913	0.0354168	+	A	C	0.646	0.0382
rs17775791	LC_IGP121	IgG2 FGS1/(F+FG+FGS1)	3	186722362	3	1.33E-09	5.12E-16	-	-0.247246	-0.3279	+	C	T	0.243884239	0.0405403	+	C	T	0.2752	0.0404
rs17775791	LC_IGP120	IgG2 FGS1/(FG+FGS1)	3	186722362	3	7.74E-30	3.59E-45	-	-0.455226	-0.5867	+	C	T	0.243735348	0.0393283	+	C	T	0.2752	0.0416
rs17775791	LC_IGP111	IgG2 SA per Gal	3	186722362	3	1.24E-34	1.12E-57	-	-0.493404	-0.6793	+	C	T	0.243847678	0.0392559	+	C	T	0.2755	0.0424
rs17775791	LC_IGP110	IgG2 Sialylation	3	186722362	3	6.00E-09	7.26E-15	-	-0.238634	-0.3144	+	C	T	0.243914145	0.040805	+	C	T	0.2756	0.0404
rs17775791	LC_IGP93	IgG2_G1FS1	3	186722362	3	1.99E-26	1.66E-44	-	-0.428637	-0.5797	+	C	T	0.243586638	0.0395937	+	C	T	0.2752	0.0414
rs17775791	LC_IGP_R74	IgG2_G1FS1/IgG2_G1F	3	186722362	3	5.38E-49	4.78E-71	-	-0.581663	-0.7558	+	C	T	0.243586638	0.0382303	+	C	T	0.2752	0.0424
rs17775791	LC_IGP_R75	IgG2_G2FS1/IgG2_G2F	3	186722362	3	1.90E-26	1.16E-56	-	-0.427602	-0.6724	+	C	T	0.243587553	0.0394808	+	C	T	0.2752	0.0424
rs17775791	LC_IGP189	IgG4 FG1S1/(FG1+FG1S1)	3	186722362	3	4.89E-27	6.54E-50	-	-0.431545	-0.6192	+	C	T	0.243439024	0.0393641	+	C	T	0.2752	0.0417
rs17775791	LC_IGP190	IgG4 FG2S1/(FG2+FG2S1)	3	186722362	3	4.41E-29	5.40E-44	-	-0.449987	-0.578	+	C	T	0.243587553	0.0394293	+	C	T	0.2752	0.0415
rs17775791	LC_IGP187	IgG4 FGS1/(FG+FGS1)	3	186722362	3	4.11E-19	3.78E-23	-	-0.360983	-0.4005	+	C	T	0.243587553	0.0399083	+	C	T	0.2752	0.0404
rs17775791	LC_IGP186	IgG4 SA per Gal	3	186722362	3	1.19E-27	8.23E-38	-	-0.439089	-0.5333	+	C	T	0.243409425	0.0395562	+	C	T	0.2748	0.0415
rs17775791	LC_IGP179	IgG4_G1FS1	3	186722362	3	1.62E-16	3.82E-31	-	-0.33281	-0.4815	+	C	T	0.243439024	0.0399295	+	C	T	0.2752	0.0415
rs17775791	LC_IGP_R92	IgG4_G1FS1/IgG4_G1F	3	186722362	3	4.80E-27	6.68E-50	-	-0.431561	-0.6192	+	C	T	0.243439024	0.0393591	+	C	T	0.2752	0.0417
rs17775791	LC_IGP_R93	IgG4_G2FS1/IgG4_G2F	3	186722362	3	4.48E-29	5.59E-44	-	-0.449922	-0.578	+	C	T	0.243587553	0.0394285	+	C	T	0.2752	0.0416
rs7617523	LC_IGP36	IgG1 FG1S1/(FG1+FG1S1)	3	186722450	3	3.75E-20	5.13E-53	-	-0.339281	-0.6258	+	G	T	0.333679817	0.0364163	+	G	T	0.3476	0.0408
rs7617523	LC_IGP37	IgG1 FG2S1/(FG2+FG2S1)	3	186722450	3	1.05E-30	6.90E-34	-	-0.424777	-0.4806	+	G	T	0.333068681	0.0361237	+	G	T	0.3476	0.0396
rs7617523	LC_IGP34	IgG1 FGS1/(FG+FGS1)	3	186722450	3	1.90E-16	9.74E-19	-	-0.306331	-0.3399	+	G	T	0.333679817	0.0368381	+	G	T	0.3476	0.0385
rs7617523	LC_IGP25	IgG1 SA per Gal	3	186722450	3	5.68E-21	6.51E-27	-	-0.352543	-0.4193	+	G	T	0.332202694	0.0369972	+	G	T	0.348	0.039
rs7617523	LC_IGP7	IgG1_G1FS1	3	186722450	3	4.53E-15	7.53E-35	-	-0.290306	-0.4984	+	G	T	0.333679817	0.0366782	+	G	T	0.3476	0.0405
rs7617523	LC_IGP_R34	IgG1_G1FS1/IgG1_G1F	3	186722450	3	4.08E-20	6.56E-53	-	-0.338858	-0.6261	+	G	T	0.333679817	0.0364074	+	G	T	0.3476	0.0409
rs7617523	LC_IGP_R35	IgG1_G2FS1/IgG1_G2F	3	186722450	3	1.25E-30	6.45E-34	-	-0.424254	-0.4809	+	G	T	0.333068681	0.0361273	+	G	T	0.3476	0.0396
rs7617523	LC_IGP122	IgG2 FG1S1/(FG1+FG1S1)	3	186722450	3	7.84E-41	5.34E-58	-	-0.489917	-0.6508	+	G	T	0.332870043	0.0356115	+	G	T	0.3476	0.0405
rs3864109	LC_IGP123	IgG2 FG2S1/(FG2+FG2S1)	3	186721867	3	7.22E-14	4.52E-27	-	-0.261629	-0.4201	+	G	A	0.498372788	0.0346555	+	G	A	0.4935	0.039
rs7617523	LC_IGP121	IgG2 FGS1/(F+FG+FGS1)	3	186722450	3	1.06E-12	2.41E-15	-	-0.266937	-0.3059	+	G	T	0.333272144	0.0371847	+	G	T	0.3476	0.0386
rs7617523	LC_IGP120	IgG2 FGS1/(FG+FGS1)	3	186722450	3	1.01E-28	7.28E-38	-	-0.411229	-0.5113	+	G	T	0.33307326	0.0362801	+	G	T	0.3476	0.0398
rs7617523	LC_IGP111	IgG2 SA per Gal	3	186722450	3	3.55E-31	5.13E-47	-	-0.430962	-0.584	+	G	T	0.333207534	0.0363321	+	G	T	0.3479	0.0406
rs7617523	LC_IGP110	IgG2 Sialylation	3	186722450	3	1.40E-12	4.79E-14	-	-0.267002	-0.291	+	G	T	0.333059296	0.0373949	+	G	T	0.348	0.0386
rs7617523	LC_IGP93	IgG2_G1FS1	3	186722450	3	5.58E-25	2.71E-39	-	-0.38344	-0.519	+	G	T	0.332870043	0.0365376	+	G	T	0.3476	0.0396
rs7617523	LC_IGP_R74	IgG2_G1FS1/IgG2_G1F	3	186722450	3	6.84E-41	4.99E-58	-	-0.490289	-0.6509	+	G	T	0.332870043	0.03561	+	G	T	0.3476	0.0405
rs7617523	LC_IGP_R75	IgG2_G2FS1/IgG2_G2F	3	186722450	3	2.23E-21	8.45E-47	-	-0.352533	-0.5818	+	G	T	0.332871263	0.0366178	+	G	T	0.3476	0.0405
rs7617523	LC_IGP189	IgG4 FG1S1/(FG1+FG1S1)	3	186722450	3	2.16E-20	3.86E-40	-	-0.343095	-0.5282	+	G	T	0.332668293	0.0365853	+	G	T	0.3476	0.0398
rs7617523	LC_IGP190	IgG4 FG2S1/(FG2+FG2S1)	3	186722450	3	1.02E-19	2.78E-34	-	-0.338621	-0.4849	+	G	T	0.332871263	0.0367894	+	G	T	0.3476	0.0397
rs7617523	LC_IGP187	IgG4 FGS1/(FG+FGS1)	3	186722450	3	1.26E-15	2.09E-19	-	-0.298345	-0.3479	+	G	T	0.332871263	0.036932	+	G	T	0.3476	0.0386
rs7617523	LC_IGP186	IgG4 SA per Gal	3	186722450	3	2.59E-21	2.87E-30	-	-0.353097	-0.4532	+	G	T	0.332960526	0.0367369	+	G	T	0.3476	0.0396
rs7617523	LC_IGP179	IgG4_G1FS1	3	186722450	3	7.13E-13	4.84E-25	-	-0.267361	-0.4096	+	G	T	0.332668293	0.0369547	+	G	T	0.3476	0.0396
rs7617523	LC_IGP_R92	IgG4_G1FS1/IgG4_G1F	3	186722450	3	1.96E-20	4.28E-40	-	-0.343432	-0.5279	+	G	T	0.332668293	0.0365789	+	G	T	0.3476	0.0398
rs7617523	LC_IGP_R93	IgG4_G2FS1/IgG4_G2F	3	186722450	3	1.02E-19	2.81E-34	-	-0.338605	-0.485	+	G	T	0.332871263	0.0367885	+	G	T	0.3476	0.0397
rs7652995	LC_IGP36	IgG1 FG1S1/(FG1+FG1S1)	3	186722944	3	9.58E-12	5.70E-20	-	-0.328813	-0.4809	+	A	G	0.841548124	0.0479151	+	A	G	0.8505	0.0526
rs7652995	LC_IGP37	IgG1 FG2S1/(FG2+FG2S1)	3	186722944	3	3.14E-16	1.30E-12	-	-0.395677	-0.3613	+	A	G	0.841533079	0.0479425	+	A	G	0.8505	0.0509
rs7652995	LC_IGP25	IgG1 SA per Gal	3	186722944	3	2.65E-10	4.16E-11	-	-0.308855	-0.3309	+	A	G	0.839640853	0.04857	+	A	G	0.8504	0.0502
rs7652995	LC_IGP7	IgG1_G1FS1	3	186722944	3	2.34E-10	1.76E-17	-	-0.306186	-0.4427	+	A	G	0.841548124	0.0480114	+	A	G	0.8505	0.052
rs7652995	LC_IGP_R34	IgG1_G1FS1/IgG1_G1F	3	186722944	3	8.24E-12	5.00E-20	-	-0.329745	-0.4825	+	A	G	0.841548124	0.0478967	+	A	G	0.8505	0.0526
rs7652995	LC_IGP_R35	IgG1_G2FS1/IgG1_G2F	3	186722944	3	3.19E-16	1.28E-12	-	-0.395573	-0.3614	+	A	G	0.841533079	0.0479429	+	A	G	0.8505	0.0509
rs7652995	LC_IGP122	IgG2 FG1S1/(FG1+FG1S1)	3	186722944	3	2.50E-17	2.81E-23	-	-0.409908	-0.5186	+	A	G	0.841629764	0.0478717	+	A	G	0.8505	0.0522
rs7643789	LC_IGP123	IgG2 FG2S1/(FG2+FG2S1)	3	186720335	3	5.96E-14	1.68E-26	-	-0.262191	-0.4135	+	C	G	0.497299573	0.0346112	+	C	G	0.496	0.0388
rs7652995	LC_IGP120	IgG2 FGS1/(FG+FGS1)	3	186722944	3	8.71E-12	8.26E-14	-	-0.332215	-0.3821	+	A	G	0.841533079	0.0483125	+	A	G	0.8505	0.0512
rs7652995	LC_IGP111	IgG2 SA per Gal	3	186722944	3	2.89E-13	8.23E-20	-	-0.357137	-0.4759	+	A	G	0.841769448	0.0485151	+	A	G	0.8501	0.0522
rs7652995	LC_IGP93	IgG2_G1FS1	3	186722944	3	4.29E-10	1.14E-17	-	-0.304798	-0.4358	+	A	G	0.841629764	0.0485235	+	A	G	0.8505	0.0509
rs7652995	LC_IGP_R74	IgG2_G1FS1/IgG2_G1F	3	186722944	3	2.49E-17	3.04E-23	-	-0.409944	-0.5182	+	A	G	0.841629764	0.0478736	+	A	G	0.8505	0.0522
rs7652995	LC_IGP_R75	IgG2_G2FS1/IgG2_G2F	3	186722944	3	4.14E-12	1.39E-18	-	-0.336882	-0.4592	+	A	G	0.841326292	0.0482314	+	A	G	0.8505	0.0522
rs7652995	LC_IGP190	IgG4 FG2S1/(FG2+FG2S1)	3	186722944	3	8.41E-09	4.96E-11	-	-0.281207	-0.3358	+	A	G	0.841326292	0.0485661	+	A	G	0.8505	0.0511
rs7652995	LC_IGP186	IgG4 SA per Gal	3	186722944	3	7.13E-09	1.24E-09	-	-0.282572	-0.3095	+	A	G	0.841451361	0.0485634	+	A	G	0.8505	0.051
rs7652995	LC_IGP_R93	IgG4_G2FS1/IgG4_G2F	3	186722944	3	8.67E-09	4.90E-11	-	-0.28095	-0.3359	+	A	G	0.841326292	0.0485657	+	A	G	0.8505	0.0511
rs13098740	LC_IGP36	IgG1 FG1S1/(FG1+FG1S1)	3	186723150	3	3.51E-20	3.88E-52	-	-0.339235	-0.6201	+	G	A	0.333752599	0.0363824	+	G	A	0.3429	0.0408
rs13098740	LC_IGP37	IgG1 FG2S1/(FG2+FG2S1)	3	186723150	3	1.06E-30	6.76E-33	-	-0.424389	-0.4728	+	G	A	0.333141331	0.0360916	+	G	A	0.3429	0.0396
rs13098740	LC_IGP34	IgG1 FGS1/(FG+FGS1)	3	186723150	3	1.89E-16	8.03E-18	-	-0.306089	-0.3305	+	G	A	0.333752599	0.0368052	+	G	A	0.3429	0.0384
rs13098740	LC_IGP25	IgG1 SA per Gal	3	186723150	3	5.44E-21	7.40E-26	-	-0.3524	-0.4098	+	G	A	0.332270363	0.0369641	+	G	A	0.3433	0.039
rs13098740	LC_IGP7	IgG1_G1FS1	3	186723150	3	4.56E-15	4.05E-34	-	-0.29001	-0.4926	+	G	A	0.333752599	0.0366457	+	G	A	0.3429	0.0404
rs13098740	LC_IGP_R34	IgG1_G1FS1/IgG1_G1F	3	186723150	3	3.82E-20	4.70E-52	-	-0.338812	-0.6206	+	G	A	0.333752599	0.0363735	+	G	A	0.3429	0.0409

rs13098740	LC_IGP_R35	IgG1_G2FS1/IgG1_G2F	3	186723150	3	1.25E-30	6.37E-33	-	-0.42386	-0.473	+	G	A	0.333141331	0.0360953	+	G	A	0.3429	0.0396
rs13098740	LC_IGP122	IgG2 FG1S1/(FG1+FG1S1)	3	186723150	3	7.96E-41	1.77E-56	-	-0.489443	-0.6407	+	G	A	0.332942343	0.0355802	+	G	A	0.3429	0.0405
rs7621438	LC_IGP123	IgG2 FG2S1/(FG2+FG2S1)	3	186720309	3	8.53E-14	1.70E-26	-	-0.261044	-0.4134	+	G	A	0.498154057	0.0346815	+	G	A	0.4961	0.0388
rs13098740	LC_IGP121	IgG2 FGS1/(F+FG+FGS1)	3	186723150	3	1.09E-12	2.79E-14	-	-0.266555	-0.2939	+	G	A	0.333344838	0.0371523	+	G	A	0.3429	0.0386
rs13098740	LC_IGP120	IgG2 FGS1/(FG+FGS1)	3	186723150	3	9.95E-29	3.46E-36	-	-0.410903	-0.4985	+	G	A	0.333145604	0.0362476	+	G	A	0.3429	0.0397
rs13098740	LC_IGP111	IgG2 SA per Gal	3	186723150	3	3.25E-31	2.16E-45	-	-0.430861	-0.5723	+	G	A	0.333281424	0.0362991	+	G	A	0.3432	0.0405
rs13098740	LC_IGP110	IgG2 Sialylation	3	186723150	3	1.41E-12	4.50E-13	-	-0.266755	-0.2794	+	G	A	0.333125386	0.0373622	+	G	A	0.3433	0.0386
rs13098740	LC_IGP93	IgG2_G1FS1	3	186723150	3	5.85E-25	4.94E-38	-	-0.382936	-0.5098	+	G	A	0.332942343	0.0365061	+	G	A	0.3429	0.0395
rs13098740	LC_IGP_R74	IgG2_G1FS1/IgG2_G1F	3	186723150	3	6.97E-41	1.65E-56	-	-0.489802	-0.6408	+	G	A	0.332942343	0.0355788	+	G	A	0.3429	0.0405
rs13098740	LC_IGP_R75	IgG2_G2FS1/IgG2_G2F	3	186723150	3	2.08E-21	5.96E-45	-	-0.352487	-0.5688	+	G	A	0.332943258	0.0365836	+	G	A	0.3429	0.0404
rs13098740	LC_IGP189	IgG4 FG1S1/(FG1+FG1S1)	3	186723150	3	1.97E-20	1.87E-39	-	-0.343148	-0.5228	+	G	A	0.332740244	0.0365507	+	G	A	0.3429	0.0398
rs13098740	LC_IGP190	IgG4 FG2S1/(FG2+FG2S1)	3	186723150	3	1.09E-19	5.70E-33	-	-0.338055	-0.4741	+	G	A	0.332943258	0.0367582	+	G	A	0.3429	0.0396
rs13098740	LC_IGP187	IgG4 FGS1/(FG+FGS1)	3	186723150	3	1.26E-15	1.52E-18	-	-0.298091	-0.3388	+	G	A	0.332943258	0.0368991	+	G	A	0.3429	0.0386
rs13098740	LC_IGP186	IgG4 SA per Gal	3	186723150	3	2.42E-21	2.61E-29	-	-0.353049	-0.4446	+	G	A	0.333033354	0.0367028	+	G	A	0.3428	0.0396
rs13098740	LC_IGP179	IgG4_G1FS1	3	186723150	3	6.38E-13	6.90E-25	-	-0.267678	-0.4083	+	G	A	0.332740244	0.0369194	+	G	A	0.3429	0.0396
rs13098740	LC_IGP_R92	IgG4_G1FS1/IgG4_G1F	3	186723150	3	1.79E-20	2.11E-39	-	-0.343472	-0.5224	+	G	A	0.332740244	0.0365444	+	G	A	0.3429	0.0398
rs13098740	LC_IGP_R93	IgG4_G2FS1/IgG4_G2F	3	186723150	3	1.09E-19	5.76E-33	-	-0.338038	-0.4742	+	G	A	0.332943258	0.0367572	+	G	A	0.3429	0.0396
rs73189714	LC_IGP122	IgG2 FG1S1/(FG1+FG1S1)	3	186723659	3	1.50E-08	5.99E-09	-	-0.497642	-0.5047	+	C	G	0.051729542	0.087455	+	C	G	0.0549	0.0868
rs73189714	LC_IGP_R74	IgG2_G1FS1/IgG2_G1F	3	186723659	3	1.46E-08	6.00E-09	-	-0.498088	-0.5046	+	C	G	0.051729542	0.0874571	+	C	G	0.0549	0.0868
rs6764279	LC_IGP36	IgG1 FG1S1/(FG1+FG1S1)	3	186723975	3	7.61E-31	9.28E-63	-	-0.450401	-0.698	+	A	C	0.260785015	0.038205	+	A	C	0.293	0.0417
rs6764279	LC_IGP37	IgG1 FG2S1/(FG2+FG2S1)	3	186723975	3	2.85E-44	1.93E-39	-	-0.543139	-0.5323	+	A	C	0.260307387	0.0377562	+	A	C	0.293	0.0405
rs6764279	LC_IGP35	IgG1 FGS1/(F+FG+FGS1)	3	186723975	3	3.22E-08	2.86E-08	-	-0.220881	-0.2167	+	A	C	0.260785015	0.0397557	+	A	C	0.293	0.039
rs6764279	LC_IGP34	IgG1 FGS1/(FG+FGS1)	3	186723975	3	8.51E-21	3.58E-19	-	-0.3697	-0.352	+	A	C	0.260785015	0.0389926	+	A	C	0.293	0.0393
rs6764279	LC_IGP25	IgG1 SA per Gal	3	186723975	3	1.19E-26	9.13E-30	-	-0.425506	-0.452	+	A	C	0.258765038	0.0391043	+	A	C	0.2934	0.0399
rs6764279	LC_IGP7	IgG1_G1FS1	3	186723975	3	2.01E-23	8.26E-44	-	-0.39101	-0.5748	+	A	C	0.260785015	0.0386072	+	A	C	0.293	0.0414
rs6764279	LC_IGP_R34	IgG1_G1FS1/IgG1_G1F	3	186723975	3	9.20E-31	1.18E-62	-	-0.449656	-0.6986	+	A	C	0.260785015	0.0381982	+	A	C	0.293	0.0418
rs6764279	LC_IGP_R35	IgG1_G2FS1/IgG1_G2F	3	186723975	3	3.27E-44	1.81E-39	-	-0.542774	-0.5325	+	A	C	0.260307387	0.0377592	+	A	C	0.293	0.0405
rs6764279	LC_IGP122	IgG2 FG1S1/(FG1+FG1S1)	3	186723975	3	2.62E-56	6.70E-73	-	-0.610202	-0.7483	+	A	C	0.260152532	0.0371244	+	A	C	0.293	0.0414
rs4012172	LC_IGP123	IgG2 FG2S1/(FG2+FG2S1)	3	186741511	3	1.16E-11	2.85E-26	-	-0.250309	-0.4117	+	T	C	0.351939902	0.0366233	+	T	C	0.3739	0.0388
rs6764279	LC_IGP121	IgG2 FGS1/(F+FG+FGS1)	3	186723975	3	4.71E-13	1.86E-16	-	-0.288703	-0.3254	+	A	C	0.260466402	0.0395869	+	A	C	0.293	0.0395
rs6764279	LC_IGP120	IgG2 FGS1/(FG+FGS1)	3	186723975	3	3.45E-36	5.19E-46	-	-0.492307	-0.5786	+	A	C	0.260311355	0.0382451	+	A	C	0.293	0.0406
rs6764279	LC_IGP111	IgG2 SA per Gal	3	186723975	3	5.43E-41	8.05E-60	-	-0.526377	-0.6762	+	A	C	0.260647368	0.0381637	+	A	C	0.2934	0.0415
rs6764279	LC_IGP110	IgG2 Sialylation	3	186723975	3	3.94E-12	1.44E-15	-	-0.278665	-0.3153	+	A	C	0.260662755	0.0398529	+	A	C	0.2935	0.0395
rs6764279	LC_IGP93	IgG2_G1FS1	3	186723975	3	8.88E-32	8.91E-48	-	-0.462043	-0.5881	+	A	C	0.260152532	0.0385568	+	A	C	0.293	0.0405
rs6764279	LC_IGP_R74	IgG2_G1FS1/IgG2_G1F	3	186723975	3	2.48E-56	6.45E-73	-	-0.610349	-0.7484	+	A	C	0.260152532	0.0371247	+	A	C	0.293	0.0414
rs6764279	LC_IGP_R75	IgG2_G2FS1/IgG2_G2F	3	186723975	3	4.73E-30	3.48E-59	-	-0.447863	-0.6717	+	A	C	0.260153447	0.0385408	+	A	C	0.293	0.0414
rs6764279	LC_IGP189	IgG4 FG1S1/(FG1+FG1S1)	3	186723975	3	1.34E-31	1.33E-51	-	-0.458434	-0.6151	+	A	C	0.259994817	0.0383748	+	A	C	0.293	0.0407
rs6764279	LC_IGP190	IgG4 FG2S1/(FG2+FG2S1)	3	186723975	3	1.72E-32	6.78E-45	-	-0.467042	-0.5707	+	A	C	0.260153447	0.0385008	+	A	C	0.293	0.0406
rs6764279	LC_IGP187	IgG4 FGS1/(FG+FGS1)	3	186723975	3	5.92E-23	8.69E-24	-	-0.39003	-0.3969	+	A	C	0.260153447	0.0389457	+	A	C	0.293	0.0395
rs6764279	LC_IGP186	IgG4 SA per Gal	3	186723975	3	2.97E-31	3.81E-39	-	-0.458482	-0.5303	+	A	C	0.260022644	0.0386089	+	A	C	0.2927	0.0405
rs6764279	LC_IGP179	IgG4_G1FS1	3	186723975	3	4.01E-19	7.59E-34	-	-0.353182	-0.4918	+	A	C	0.259994817	0.039033	+	A	C	0.293	0.0406
rs6764279	LC_IGP_R92	IgG4_G1FS1/IgG4_G1F	3	186723975	3	1.36E-31	1.29E-51	-	-0.458334	-0.6152	+	A	C	0.259994817	0.0383707	+	A	C	0.293	0.0407
rs6764279	LC_IGP_R93	IgG4_G2FS1/IgG4_G2F	3	186723975	3	1.74E-32	7.03E-45	-	-0.466995	-0.5707	+	A	C	0.260153447	0.0384999	+	A	C	0.293	0.0406
rs6788832	LC_IGP36	IgG1 FG1S1/(FG1+FG1S1)	3	186724219	3	2.83E-20	2.00E-52	-	-0.339482	-0.6196	+	G	A	0.333933028	0.036315	+	G	A	0.3464	0.0407
rs6788832	LC_IGP37	IgG1 FG2S1/(FG2+FG2S1)	3	186724219	3	9.38E-31	1.99E-33	-	-0.424018	-0.4754	+	G	A	0.333321429	0.036027	+	G	A	0.3464	0.0395
rs6788832	LC_IGP34	IgG1 FGS1/(FG+FGS1)	3	186724219	3	1.78E-16	9.43E-19	-	-0.305819	-0.3388	+	G	A	0.333933028	0.0367405	+	G	A	0.3464	0.0383
rs6788832	LC_IGP25	IgG1 SA per Gal	3	186724219	3	4.75E-21	8.18E-27	-	-0.352328	-0.4166	+	G	A	0.332443609	0.0368989	+	G	A	0.3468	0.0389
rs6788832	LC_IGP7	IgG1_G1FS1	3	186724219	3	4.31E-15	2.65E-34	-	-0.289777	-0.4926	+	G	A	0.333933028	0.0365813	+	G	A	0.3464	0.0403
rs6788832	LC_IGP_R34	IgG1_G1FS1/IgG1_G1F	3	186724219	3	3.07E-20	2.60E-52	-	-0.339062	-0.6199	+	G	A	0.333933028	0.0363061	+	G	A	0.3464	0.0407
rs6788832	LC_IGP_R35	IgG1_G2FS1/IgG1_G2F	3	186724219	3	1.12E-30	1.85E-33	-	-0.42348	-0.4756	+	G	A	0.333321429	0.0360307	+	G	A	0.3464	0.0395
rs6788832	LC_IGP122	IgG2 FG1S1/(FG1+FG1S1)	3	186724219	3	7.27E-41	1.47E-57	-	-0.488834	-0.6455	+	G	A	0.333122026	0.035517	+	G	A	0.3464	0.0404
rs3872723	LC_IGP123	IgG2 FG2S1/(FG2+FG2S1)	3	186741131	3	1.08E-11	2.88E-26	-	-0.250535	-0.4115	+	T	C	0.351615009	0.0366052	+	T	C	0.3749	0.0388
rs6788832	LC_IGP121	IgG2 FGS1/(F+FG+FGS1)	3	186724219	3	1.12E-12	2.25E-15	-	-0.265968	-0.3052	+	G	A	0.333525046	0.0370889	+	G	A	0.3464	0.0385
rs6788832	LC_IGP120	IgG2 FGS1/(FG+FGS1)	3	186724219	3	8.95E-29	9.97E-38	-	-0.410529	-0.5081	+	G	A	0.333325397	0.0361829	+	G	A	0.3464	0.0396
rs6788832	LC_IGP111	IgG2 SA per Gal	3	186724219	3	2.62E-31	1.10E-46	-	-0.430795	-0.5793	+	G	A	0.333457895	0.0362336	+	G	A	0.3467	0.0404
rs6788832	LC_IGP110	IgG2 Sialylation	3	186724219	3	1.39E-12	4.47E-14	-	-0.266365	-0.2904	+	G	A	0.333293391	0.0372988	+	G	A	0.3468	0.0385
rs6788832	LC_IGP93	IgG2_G1FS1	3	186724219	3	5.93E-25	5.66E-39	-	-0.382229	-0.5152	+	G	A	0.333122026	0.0364437	+	G	A	0.3464	0.0394
rs6788832	LC_IGP_R74	IgG2_G1FS1/IgG2_G1F	3	186724219	3	6.42E-41	1.37E-57	-	-0.489174	-0.6456	+	G	A	0.333122026	0.0355158	+	G	A	0.3464	0.0404
rs6788832	LC_IGP_R75	IgG2_G2FS1/IgG2_G2F	3	186724219	3	1.63E-21	1.99E-46	-	-0.352777	-0.577	+	G	A	0.333122636	0.0365153	+	G	A	0.3464	0.0403
rs6788832	LC_IGP189	IgG4 FG1S1/(FG1+FG1S1)	3	186724219	3	1.62E-20	8.25E-40	-	-0.343299	-0.5235	+	G	A	0.332919512	0.0364835	+	G	A	0.3464	0.0396
rs6788832	LC_IGP190	IgG4 FG2S1/(FG2+FG2S1)	3	186724219	3	1.10E-19	3.33E-34	-	-0.337437	-0.4822	+	G	A	0.333122636	0.0366951	+	G	A	0.3464	0.0395
rs6788832	LC_IGP187	IgG4 FGS1/(FG+FGS1)	3	186724219	3	1.20E-15	1.77E-19	-	-0.297798	-0.3471	+	G	A	0.333122636	0.0368345	+	G	A	0.3464	0.0384
rs6788832	LC_IGP186	IgG4 SA per Gal	3	186724219	3	2.04E-21	2.94E-30	-	-0.353072	-0.451	+	G	A	0.333215422	0.036636	+	G	A	0.3464	0.0394
rs6788832	LC_IGP179	IgG4_G1FS1	3	186724219	3	5.31E-13	8.73E-25	-	-0.268135	-0.4061	+	G	A	0.332919512	0.0368518	+	G	A	0.3464	0.0395
rs6788832	LC_IGP_R92	IgG4_G1FS1/IgG4_G1F	3	186724219	3	1.48E-20	9.03E-40	-	-0.3436	-0.5233	+	G	A	0.332919512	0.0364774	+	G	A	0.3464	0.0396
rs6788832	LC_IGP_R93	IgG4_G2FS1/IgG4_G2F	3	186724219	3	1.10E-19	3.36E-34	-	-0.33742	-0.4822	+	G	A	0.333122636	0.0366941	+	G	A	0.3464	0.0396
rs3872721	LC_IGP36	IgG1 FG1S1/(FG1+FG1S1)	3	186725491	3	6.96E-14	2.38E-19	-	-0.308804	-0.4133	+	A	G	0.768166972	0.040877	+	A	G	0.7979	0.046
rs3872721	LC_IGP37	IgG1 FG2S1/(FG2+FG2S1)	3	186725491	3	9.05E-18	5.54E-12	-	-0.355548	-0.307	+	A	G	0.768286325	0.0409409	+	A	G	0.7979	0.0446
rs3872721	LC_IGP25	IgG1 SA per Gal	3	186725491	3	1.49E-09	1.10E-09	-	-0.253613	-0.2674	+	A	G	0.765671679	0.041707	+	A	G	0.7976	0.0439
rs3872721	LC_IGP7	IgG1_G1FS1	3	186725491	3	2.46E-12	2.18E-18	-	-0.289221	-0.3983	+	A	G	0.768166972	0.0409686	+	A	G	0.7979	0.0455
rs3872721	LC_IGP_R34	IgG1_G1FS1/IgG1_G1F	3	186725491	3	6.29E-14	2.03E-19	-	-0.309256	-0.4148	+	A	G	0.768166972	0.0408625	+	A	G	0.7979	0.046
rs3872721	LC_IGP_R35	IgG1_G2FS1/IgG1_G2F	3	186725491	3	8.58E-18	5.49E-12	-	-0.355796	-0.307	+	A	G	0.768286325	0.0409395	+	A	G	0.7979	0.0446
rs3872721	LC_IGP122	IgG2 FG1S1/(FG1+FG1S1)	3	186725491	3	3.37E-18	6.70E-26	-	-0.360108	-0.4799	+	A	G	0.7684277	0.0409158	+	A	G	0.7979	0.0456
rs7643952	LC_IGP123	IgG2 FG2S1/(FG2+FG2S1)	3	186720448	3	5.87E-14	3.67E-26	-	-0.262395	-0.4108	+	C	G	0.497530811	0.0346289	+	C	G	0.497	0.0388
rs3872721	LC_IGP120	IgG2 FGS1/(FG+FGS1)	3	186725491	3	6.50E-11	7.91E-15	-	-0.272196	-0.3473	+	A	G	0.768286325	0.0413951	+	A	G	0.7979	0.0447
rs3872721	LC_IGP111	IgG2 SA per Gal	3	186725491	3	5.11E-13	2.80E-22	-	-0.303036	-0.4428	+	A	G	0.768708978	0.0416127	+	A	G	0.7974	0.0456
rs3872721	LC_IGP93	IgG2_G1FS1	3	186725491	3	9.90E-10	2.49E-18	-	-0.255369	-0.3887	+	A	G	0.7684277	0.0415437	+	A	G	0.7979	0.0445
rs3872721	LC_IGP_R74	IgG2_G1FS1/IgG2_G1F	3	186725491	3	3.55E-18	7.44E-26	-	-0.359877	-0.4794	+	A	G	0.7684277	0.0409187	+	A	G	0.7979	0.0456
rs3872721	LC_IGP_R75	IgG2_G2FS1/IgG2_G2F	3	186725491	3	9.14E-13	5.15E-21	-	-0.297006	-0.4285	+	A	G	0.768122941	0.041251	+	A	G	0.7979	0.0456
rs3872721	LC_IGP189	IgG4 FG1S1/(FG1+FG1S1)	3	186725491	3	2.98E-10	2.65E-13	-	-0.261831	-0.3274	+	A	G	0.768264329	0.0413055	+	A	G	0.7979	0.0448
rs3872721	LC_IGP190	IgG4 FG2S1/(FG2+FG2S1)	3	186725491	3	9.83E-13	1.00E-13	-	-0.2973	-0.3322	+	A	G	0.768122941	0.0413507	+	A	G	0.7979	0.0446
rs3872721	LC_IGP187	IgG4 FGS1/(FG+FGS1)	3	186725491	3	2.96E-08	1.54E-06	-	-0.231344	-0.2087	+	A	G	0.768122941	0.0415305	+	A	G	0.7979	0.0434
rs3872721	LC_IGP186	IgG4 SA per Gal	3	186725491	3	1.47E-10	7.94E-12	-	-0.267404	-0.3045	+	A	G	0.768025092	0.0414614	+	A	G	0.7976	0.0445
rs3872721	LC_IGP179	IgG4_G1FS1	3	186725491	3	1.79E-08	1.01E-11	-	-0.234232	-0.3034	+	A	G	0.768264329	0.0413908	+	A	G	0.7979	0.0446
rs3872721	LC_IGP_R92	IgG4_G1FS1/IgG4_G1F	3	186725491	3	3.08E-10	2.69E-13	-	-0.261607	-0.3273	+	A	G	0.768264329	0.0413015	+	A	G	0.7979	0.0448
rs3872721	LC_IGP_R93	IgG4_G2FS1/IgG4_G2F	3	186725491	3	1.03E-12	1.01E-13	-	-0.297052	-0.3323	+	A	G	0.768122941	0.0413506	+	A	G	0.7979	0.0447
rs11710456	LC_IGP_R35	IgG1_G2FS1/IgG1_G2F	3	186725887	3	1.40E-44	8.01E-39	-	-0.542066	-0.5327	+	A	G	0.260389805	0.0375381	+	A	G	0.2939	0.0409
rs11710456	LC_IGP_R74	IgG2_G1FS1/IgG2_G1F	3	186725887	3	1.17E-56	6.30E-73	-	-0.608772	-0.7558	+	A	G	0.260233374	0.0369092	+	A	G	0.2939	0.0418
rs11710456	LC_IGP_R75	IgG2_G2FS1/IgG2_G2F	3	186725887	3	1.34E-30	4.66E-59	-	-0.449571	-0.678	+	A	G	0.260233374	0.0383053	+	A	G	0.2939	0.0418
rs11710456	LC_IGP_R92	IgG4_G1FS1/IgG4_G1F	3	186725887	3	6.04E-32	1.55E-50	-	-0.458446	-0.6133	+	A	G	0.260074695	0.0381467	+	A	G	0.2939	0.041
rs11710456	LC_IGP_R93	IgG4_G2FS1/IgG4_G2F	3	186725887	3	1.63E-32	4.94E-45	-	-0.464688	-0.576	+	A	G	0.260233374	0.0382925	+	A	G	0.2939	0.0409
rs11710456	LC_IGP110	IgG2 Sialylation	3	186725887	3	3.92E-12	8.49E-16	-	-0.277255	-0.3211	+	A	G	0.260698579	0.0396464	+	A	G	0.2944	0.0399
rs12490407	LC_IGP123	IgG2 FG2S1/(FG2+FG2S1)	3	186721996	3	6.92E-14	4.44E-26	-	-0.261772	-0.4065	+	A	G	0.498467358	0.0346483	+	A	G	0.4994	0.0385
rs11710456	LC_IGP111	IgG2 SA per Gal	3	186725887	3	1.74E-41	8.14E-60	-	-0.526793	-0.6833	+	A	G	0.260708669	0.0379417	+	A	G	0.2942	0.0419
rs11710456	LC_IGP120	IgG2 FGS1/(FG+FGS1)	3	186725887	3	1.82E-36	1.24E-46	-	-0.491541	-0.5886	+	A	G	0.260392247	0.0380257	+	A	G	0.2939	0.0411
rs11710456	LC_IGP121	IgG2 FGS1/(F+FG+FGS1)	3	186725887	3	5.17E-13	8.72E-17	-	-0.286657	-0.3321	+	A	G	0.26054887	0.0393774	+	A	G	0.2939	0.0399
rs11710456	LC_IGP122	IgG2 FG1S1/(FG1+FG1S1)	3	186725887	3	1.20E-56	6.51E-73	-	-0.608686	-0.7558	+	A	G	0.260233374	0.0369083	+	A	G	0.2939	0.0418
rs11710456	LC_IGP123	IgG2 FG2S1/(FG2+FG2S1)	3	186725887	3	1.33E-30	4.70E-59	-	-0.44958	-0.678	+	A	G	0.260233374	0.038304	+	A	G	0.2939	0.0418
rs11710456	LC_IGP179	IgG4_G1FS1	3	186725887	3	2.86E-19	1.65E-32	-	-0.352719	-0.4848	+	A	G	0.260074695	0.0388164	+	A	G	0.2939	0.0408
rs11710456	LC_IGP186	IgG4 SA per Gal	3	186725887	3	1.78E-31	2.10E-39	-	-0.457675	-0.5365	+	A	G	0.260107099	0.0383907	+	A	G	0.2936	0.0408
rs11710456	LC_IGP187	IgG4 FGS1/(FG+FGS1)	3	186725887	3	5.58E-23	2.24E-24	-	-0.388174	-0.4054	+	A	G	0.260233374	0.038736	+	A	G	0.2939	0.0398
rs11710456	LC_IGP189	IgG4 FG1S1/(FG1+FG1S1)	3	186725887	3	5.87E-32	1.63E-50	-	-0.458588	-0.6132	+	A	G	0.260074695	0.0381505	+	A	G	0.2939	0.041
rs11710456	LC_IGP190	IgG4 FG2S1/(FG2+FG2S1)	3	186725887	3	1.62E-32	4.76E-45	-	-0.464733	-0.576	+	A	G	0.260233374	0.0382933	+	A	G	0.2939	0.0409
rs11710456	LC_IGP25	IgG1 SA per Gal	3	186725887	3	7.08E-27	1.14E-29	-	-0.425158	-0.4558	+	A	G	0.258808271	0.0388903	+	A	G	0.2942	0.0403
rs11710456	LC_IGP34	IgG1 FGS1/(FG+FGS1)	3	186725887	3	7.18E-21	1.68E-19	-	-0.368408	-0.3586	+	A	G	0.260867584	0.0387799	+	A	G	0.2939	0.0397
rs11710456	LC_IGP35	IgG1 FGS1/(F+FG+FGS1)	3	186725887	3	3.71E-08	1.38E-08	-	-0.218706	-0.2236	+	A	G	0.260867584	0.0395462	+	A	G	0.2939	0.0394
rs11710456	LC_IGP36	IgG1 FG1S1/(FG1+FG1S1)	3	186725887	3	1.96E-31	9.68E-62	-	-0.452302	-0.6987	+	A	G	0.260867584	0.0379692	+	A	G	0.2939	0.0421
rs11710456	LC_IGP37	IgG1 FG2S1/(FG2+FG2S1)	3	186725887	3	1.21E-44	8.55E-39	-	-0.542453	-0.5325	+	A	G	0.260389805	0.0375348	+	A	G	0.2939	0.0409
rs11710456	LC_IGP7	IgG1_G1FS1	3	186725887	3	1.29E-23	2.07E-42	-	-0.390577	-0.5699	+	A	G	0.260867584	0.0383903	+	A	G	0.2939	0.0418
rs11710456	LC_IGP93	IgG2_G1FS1	3	186725887	3	9.49E-32	7.03E-47	-	-0.459361	-0.5876	+	A	G	0.260233374	0.0383521	+	A	G	0.2939	0.0409
rs3872722	LC_IGP36	IgG1 FG1S1/(FG1+FG1S1)	3	186726277	3	1.33E-17	1.48E-25	-	-0.302527	-0.4023	+	A	C	0.602219878	0.0350198	+	A	C	0.646	0.0385
rs3872722	LC_IGP37	IgG1 FG2S1/(FG2+FG2S1)	3	186726277	3	1.48E-21	1.05E-15	-	-0.338819	-0.2997	+	A	C	0.602337912	0.0350313	+	A	C	0.646	0.0374
rs3872722	LC_IGP34	IgG1 FGS1/(FG+FGS1)	3	186726277	3	1.98E-09	2.13E-07	-	-0.215055	-0.1882	+	A	C	0.602219878	0.0356445	+	A	C	0.646	0.0363
rs3872722	LC_IGP29	IgG1 G2S1/(G2+G2S1)	3	186726277	3	1.87E-08	2.51E-08	-	-0.202545	-0.2104	+	A	C	0.601853606	0.0358389	+	A	C	0.646	0.0378
rs3872722	LC_IGP25	IgG1 SA per Gal	3	186726277	3	1.86E-12	1.10E-12	-	-0.254474	-0.2618	+	A	C	0.60128916	0.0358352	+	A	C	0.6461	0.0368
rs3872722	LC_IGP7	IgG1_G1FS1	3	186726277	3	2.40E-14	5.83E-23	-	-0.270789	-0.3768	+	A	C	0.602219878	0.035182	+	A	C	0.646	0.0382
rs3872722	LC_IGP_R34	IgG1_G1FS1/IgG1_G1F	3	186726277	3	1.22E-17	1.25E-25	-	-0.302792	-0.4036	+	A	C	0.602219878	0.0350077	+	A	C	0.646	0.0386
rs3872722	LC_IGP_R35	IgG1_G2FS1/IgG1_G2F	3	186726277	3	1.77E-21	1.03E-15	-	-0.33817	-0.2997	+	A	C	0.602337912	0.035035	+	A	C	0.646	0.0374
rs3872722	LC_IGP_R33	IgG1_G2S1/IgG1_G2	3	186726277	3	2.16E-08	2.56E-08	-	-0.201676	-0.2103	+	A	C	0.601853606	0.0358451	+	A	C	0.646	0.0378

rs3872722	LC_IGP122	IgG2 FG1S1/(FG1+FG1S1)	3	186726277	3	1.00E-20	1.43E-34	-	-0.331927	-0.4687	+	A	C	0.602277913	0.035077	+	A	C	0.646	0.0382
rs3852061	LC_IGP123	IgG2 FG2S1/(FG2+FG2S1)	3	186721820	3	7.28E-14	4.68E-26	-	-0.261601	-0.4062	+	G	T	0.498366992	0.0346567	+	G	T	0.4992	0.0385
rs3872722	LC_IGP120	IgG2 FGS1/(FG+FGS1)	3	186726277	3	9.40E-13	2.28E-21	-	-0.25558	-0.3557	+	A	C	0.602340354	0.0355168	+	A	C	0.646	0.0375
rs3872722	LC_IGP111	IgG2 SA per Gal	3	186726277	3	1.21E-15	4.55E-29	-	-0.28741	-0.428	+	A	C	0.601673684	0.0355505	+	A	C	0.645	0.0382
rs3872722	LC_IGP93	IgG2_G1FS1	3	186726277	3	7.20E-11	8.53E-25	-	-0.234091	-0.384	+	A	C	0.602277913	0.0356848	+	A	C	0.646	0.0374
rs3872722	LC_IGP_R74	IgG2_G1FS1/IgG2_G1F	3	186726277	3	1.14E-20	1.52E-34	-	-0.331485	-0.4685	+	A	C	0.602277913	0.035081	+	A	C	0.646	0.0382
rs3872722	LC_IGP_R75	IgG2_G2FS1/IgG2_G2F	3	186726277	3	2.95E-14	1.80E-27	-	-0.271627	-0.4147	+	A	C	0.602277913	0.035418	+	A	C	0.646	0.0382
rs3872722	LC_IGP189	IgG4 FG1S1/(FG1+FG1S1)	3	186726277	3	1.32E-13	7.29E-22	-	-0.264201	-0.3607	+	A	C	0.602215549	0.0353798	+	A	C	0.646	0.0375
rs3872722	LC_IGP190	IgG4 FG2S1/(FG2+FG2S1)	3	186726277	3	7.78E-17	2.64E-18	-	-0.298154	-0.3267	+	A	C	0.601972849	0.0353889	+	A	C	0.646	0.0374
rs3872722	LC_IGP187	IgG4 FGS1/(FG+FGS1)	3	186726277	3	6.43E-11	3.98E-10	-	-0.23425	-0.2277	+	A	C	0.601972849	0.0356149	+	A	C	0.646	0.0364
rs3872722	LC_IGP186	IgG4 SA per Gal	3	186726277	3	3.65E-14	3.67E-17	-	-0.271167	-0.3143	+	A	C	0.60196634	0.0354887	+	A	C	0.6454	0.0373
rs3872722	LC_IGP179	IgG4_G1FS1	3	186726277	3	3.51E-09	1.37E-19	-	-0.2113	-0.3387	+	A	C	0.602215549	0.0355825	+	A	C	0.646	0.0374
rs3872722	LC_IGP_R92	IgG4_G1FS1/IgG4_G1F	3	186726277	3	1.37E-13	6.84E-22	-	-0.263998	-0.361	+	A	C	0.602215549	0.0353765	+	A	C	0.646	0.0375
rs3872722	LC_IGP_R93	IgG4_G2FS1/IgG4_G2F	3	186726277	3	8.10E-17	2.66E-18	-	-0.297977	-0.3267	+	A	C	0.601972849	0.0353888	+	A	C	0.646	0.0374
rs7643068	LC_IGP36	IgG1 FG1S1/(FG1+FG1S1)	3	186727083	3	1.91E-11	4.21E-19	-	-0.319631	-0.4649	+	G	A	0.840978593	0.0472827	+	G	A	0.8473	0.052
rs7643068	LC_IGP37	IgG1 FG2S1/(FG2+FG2S1)	3	186727083	3	3.31E-16	1.05E-11	-	-0.390001	-0.3429	+	G	A	0.840964591	0.0472926	+	G	A	0.8473	0.0504
rs7643068	LC_IGP25	IgG1 SA per Gal	3	186727083	3	2.56E-10	2.66E-10	-	-0.304886	-0.3136	+	G	A	0.838972431	0.0479044	+	G	A	0.8472	0.0496
rs7643068	LC_IGP7	IgG1_G1FS1	3	186727083	3	3.06E-10	4.36E-17	-	-0.300075	-0.4336	+	G	A	0.840978593	0.0473656	+	G	A	0.8473	0.0516
rs7643068	LC_IGP_R34	IgG1_G1FS1/IgG1_G1F	3	186727083	3	1.63E-11	3.77E-19	-	-0.320621	-0.4664	+	G	A	0.840978593	0.0472643	+	G	A	0.8473	0.0521
rs7643068	LC_IGP_R35	IgG1_G2FS1/IgG1_G2F	3	186727083	3	3.27E-16	1.03E-11	-	-0.390068	-0.3431	+	G	A	0.840964591	0.0472921	+	G	A	0.8473	0.0504
rs7643068	LC_IGP122	IgG2 FG1S1/(FG1+FG1S1)	3	186727083	3	6.12E-17	1.51E-22	-	-0.399442	-0.5047	+	G	A	0.841061623	0.0472468	+	G	A	0.8473	0.0516
rs6808800	LC_IGP123	IgG2 FG2S1/(FG2+FG2S1)	3	186719374	3	1.10E-13	1.22E-25	-	-0.260204	-0.405	+	G	T	0.498585722	0.0347291	+	G	T	0.4996	0.0387
rs7643068	LC_IGP120	IgG2 FGS1/(FG+FGS1)	3	186727083	3	1.21E-11	2.19E-13	-	-0.325438	-0.3712	+	G	A	0.840964591	0.0476656	+	G	A	0.8473	0.0506
rs7643068	LC_IGP111	IgG2 SA per Gal	3	186727083	3	8.60E-13	3.34E-19	-	-0.345262	-0.4622	+	G	A	0.841176471	0.0478904	+	G	A	0.8469	0.0516
rs7643068	LC_IGP93	IgG2_G1FS1	3	186727083	3	3.40E-10	4.55E-17	-	-0.302388	-0.4237	+	G	A	0.841061623	0.0478577	+	G	A	0.8473	0.0505
rs7643068	LC_IGP_R74	IgG2_G1FS1/IgG2_G1F	3	186727083	3	6.03E-17	1.66E-22	-	-0.39954	-0.5042	+	G	A	0.841061623	0.0472483	+	G	A	0.8473	0.0516
rs7643068	LC_IGP_R75	IgG2_G2FS1/IgG2_G2F	3	186727083	3	1.10E-11	5.75E-18	-	-0.325693	-0.4451	+	G	A	0.840756559	0.0476046	+	G	A	0.8473	0.0515
rs7643068	LC_IGP190	IgG4 FG2S1/(FG2+FG2S1)	3	186727083	3	3.62E-09	1.71E-10	-	-0.284084	-0.3228	+	G	A	0.840756559	0.0478829	+	G	A	0.8473	0.0506
rs7643068	LC_IGP186	IgG4 SA per Gal	3	186727083	3	7.81E-09	6.27E-09	-	-0.278011	-0.2929	+	G	A	0.840881273	0.0479081	+	G	A	0.8473	0.0504
rs7643068	LC_IGP_R93	IgG4_G2FS1/IgG4_G2F	3	186727083	3	3.78E-09	1.70E-10	-	-0.28375	-0.3229	+	G	A	0.840756559	0.0478827	+	G	A	0.8473	0.0506
rs7621161	LC_IGP36	IgG1 FG1S1/(FG1+FG1S1)	3	186727170	3	1.04E-31	1.26E-61	-	-0.450981	-0.7013	+	A	C	0.26116208	0.0376774	+	A	C	0.296	0.0423
rs7621161	LC_IGP37	IgG1 FG2S1/(FG2+FG2S1)	3	186727170	3	9.44E-45	2.88E-38	-	-0.53913	-0.5311	+	A	C	0.260683761	0.0372553	+	A	C	0.296	0.0411
rs7621161	LC_IGP34	IgG1 FGS1/(FG+FGS1)	3	186727170	3	8.08E-21	3.63E-19	-	-0.365241	-0.3573	+	A	C	0.26116208	0.0384994	+	A	C	0.296	0.0399
rs7621161	LC_IGP25	IgG1 SA per Gal	3	186727170	3	6.32E-27	3.57E-29	-	-0.422449	-0.454	+	A	C	0.259085213	0.0386036	+	A	C	0.2964	0.0405
rs7621161	LC_IGP7	IgG1_G1FS1	3	186727170	3	1.55E-23	3.82E-42	-	-0.387033	-0.5708	+	A	C	0.26116208	0.0381142	+	A	C	0.296	0.042
rs7621161	LC_IGP_R34	IgG1_G1FS1/IgG1_G1F	3	186727170	3	1.23E-31	1.62E-61	-	-0.450339	-0.7019	+	A	C	0.26116208	0.03767	+	A	C	0.296	0.0424
rs7621161	LC_IGP_R35	IgG1_G2FS1/IgG1_G2F	3	186727170	3	1.09E-44	2.70E-38	-	-0.53876	-0.5313	+	A	C	0.260683761	0.0372584	+	A	C	0.296	0.0411
rs7621161	LC_IGP122	IgG2 FG1S1/(FG1+FG1S1)	3	186727170	3	1.26E-56	4.68E-72	-	-0.604133	-0.755	+	A	C	0.26052471	0.03664	+	A	C	0.296	0.0421
rs62292644	LC_IGP123	IgG2 FG2S1/(FG2+FG2S1)	3	186738896	3	7.05E-09	9.51E-25	-	-0.366226	-0.6346	+	C	A	0.125241305	0.0629206	+	C	A	0.1672	0.0618
rs7621161	LC_IGP121	IgG2 FGS1/(F+FG+FGS1)	3	186727170	3	8.14E-13	1.59E-16	-	-0.282162	-0.3312	+	A	C	0.260843005	0.0391006	+	A	C	0.296	0.0401
rs7621161	LC_IGP120	IgG2 FGS1/(FG+FGS1)	3	186727170	3	1.88E-36	1.12E-45	-	-0.487861	-0.5853	+	A	C	0.260683761	0.0377488	+	A	C	0.296	0.0413
rs7621161	LC_IGP111	IgG2 SA per Gal	3	186727170	3	1.83E-41	6.36E-59	-	-0.522867	-0.6814	+	A	C	0.260990712	0.0376705	+	A	C	0.2964	0.0421
rs7621161	LC_IGP110	IgG2 Sialylation	3	186727170	3	6.20E-12	1.25E-15	-	-0.272705	-0.321	+	A	C	0.260963558	0.0393713	+	A	C	0.2965	0.0401
rs7621161	LC_IGP93	IgG2_G1FS1	3	186727170	3	2.30E-31	4.45E-47	-	-0.453209	-0.5921	+	A	C	0.26052471	0.0380926	+	A	C	0.296	0.0411
rs7621161	LC_IGP_R74	IgG2_G1FS1/IgG2_G1F	3	186727170	3	1.24E-56	4.53E-72	-	-0.604189	-0.7551	+	A	C	0.26052471	0.0366411	+	A	C	0.296	0.0421
rs7621161	LC_IGP_R75	IgG2_G2FS1/IgG2_G2F	3	186727170	3	9.60E-31	3.42E-58	-	-0.447366	-0.6762	+	A	C	0.26052471	0.0380179	+	A	C	0.296	0.042
rs7621161	LC_IGP189	IgG4 FG1S1/(FG1+FG1S1)	3	186727170	3	4.00E-32	1.57E-50	-	-0.456443	-0.6178	+	A	C	0.260365854	0.0378632	+	A	C	0.296	0.0413
rs7621161	LC_IGP190	IgG4 FG2S1/(FG2+FG2S1)	3	186727170	3	9.77E-33	3.66E-44	-	-0.462907	-0.5744	+	A	C	0.26052471	0.0380022	+	A	C	0.296	0.0412
rs7621161	LC_IGP187	IgG4 FGS1/(FG+FGS1)	3	186727170	3	4.89E-23	1.05E-23	-	-0.38584	-0.4029	+	A	C	0.26052471	0.0384501	+	A	C	0.296	0.0401
rs7621161	LC_IGP186	IgG4 SA per Gal	3	186727170	3	1.82E-31	1.03E-38	-	-0.454252	-0.5358	+	A	C	0.260403917	0.0381105	+	A	C	0.2958	0.0412
rs7621161	LC_IGP179	IgG4_G1FS1	3	186727170	3	4.36E-19	1.47E-32	-	-0.348376	-0.4887	+	A	C	0.260365854	0.0385429	+	A	C	0.296	0.0411
rs7621161	LC_IGP_R92	IgG4_G1FS1/IgG4_G1F	3	186727170	3	4.09E-32	1.53E-50	-	-0.456319	-0.618	+	A	C	0.260365854	0.0378594	+	A	C	0.296	0.0413
rs7621161	LC_IGP_R93	IgG4_G2FS1/IgG4_G2F	3	186727170	3	9.84E-33	3.81E-44	-	-0.46287	-0.5743	+	A	C	0.26052471	0.0380013	+	A	C	0.296	0.0412
rs12633034	LC_IGP36	IgG1 FG1S1/(FG1+FG1S1)	3	186727597	3	5.72E-14	1.10E-18	-	-0.308896	-0.4087	+	G	C	0.768652905	0.0407462	+	G	C	0.7886	0.0463
rs12633034	LC_IGP37	IgG1 FG2S1/(FG2+FG2S1)	3	186727597	3	5.62E-18	1.02E-11	-	-0.35666	-0.3051	+	G	C	0.768771368	0.0408032	+	G	C	0.7886	0.0448
rs12633034	LC_IGP25	IgG1 SA per Gal	3	186727597	3	1.17E-09	1.95E-09	-	-0.254474	-0.2648	+	G	C	0.766132519	0.0415721	+	G	C	0.7884	0.0441
rs12633034	LC_IGP7	IgG1_G1FS1	3	186727597	3	2.37E-12	3.72E-18	-	-0.288542	-0.3987	+	G	C	0.768652905	0.0408414	+	G	C	0.7886	0.0459
rs12633034	LC_IGP_R34	IgG1_G1FS1/IgG1_G1F	3	186727597	3	5.13E-14	9.10E-19	-	-0.309375	-0.4104	+	G	C	0.768652905	0.0407316	+	G	C	0.7886	0.0464
rs12633034	LC_IGP_R35	IgG1_G2FS1/IgG1_G2F	3	186727597	3	5.32E-18	1.01E-11	-	-0.356908	-0.3052	+	G	C	0.768771368	0.0408018	+	G	C	0.7886	0.0448
rs12633034	LC_IGP122	IgG2 FG1S1/(FG1+FG1S1)	3	186727597	3	3.01E-18	4.89E-25	-	-0.359509	-0.475	+	G	C	0.768912447	0.0407872	+	G	C	0.7886	0.046
rs3872724	LC_IGP123	IgG2 FG2S1/(FG2+FG2S1)	3	186741221	3	1.29E-10	2.91E-24	-	-0.235169	-0.3929	+	T	C	0.35661989	0.0363493	+	T	C	0.381	0.0387
rs12633034	LC_IGP120	IgG2 FGS1/(FG+FGS1)	3	186727597	3	5.92E-11	1.85E-14	-	-0.271934	-0.3451	+	G	C	0.768771368	0.0412654	+	G	C	0.7886	0.045
rs12633034	LC_IGP111	IgG2 SA per Gal	3	186727597	3	4.31E-13	1.06E-21	-	-0.303062	-0.4402	+	G	C	0.769193498	0.0414824	+	G	C	0.7882	0.046
rs12633034	LC_IGP93	IgG2_G1FS1	3	186727597	3	1.09E-09	1.15E-17	-	-0.253955	-0.3847	+	G	C	0.768912447	0.0414184	+	G	C	0.7886	0.045
rs12633034	LC_IGP_R74	IgG2_G1FS1/IgG2_G1F	3	186727597	3	3.18E-18	5.51E-25	-	-0.359282	-0.4744	+	G	C	0.768912447	0.0407902	+	G	C	0.7886	0.046
rs12633034	LC_IGP_R75	IgG2_G2FS1/IgG2_G2F	3	186727597	3	9.31E-13	2.20E-20	-	-0.295988	-0.4252	+	G	C	0.768607383	0.0411246	+	G	C	0.7886	0.046
rs12633034	LC_IGP189	IgG4 FG1S1/(FG1+FG1S1)	3	186727597	3	2.39E-10	9.97E-13	-	-0.262439	-0.3214	+	G	C	0.768748476	0.041173	+	G	C	0.7886	0.0451
rs12633034	LC_IGP190	IgG4 FG2S1/(FG2+FG2S1)	3	186727597	3	6.99E-13	9.19E-14	-	-0.298296	-0.3348	+	G	C	0.768607383	0.041215	+	G	C	0.7886	0.0449
rs12633034	LC_IGP187	IgG4 FGS1/(FG+FGS1)	3	186727597	3	2.31E-08	1.75E-06	-	-0.232413	-0.2088	+	G	C	0.768607383	0.0413966	+	G	C	0.7886	0.0437
rs12633034	LC_IGP186	IgG4 SA per Gal	3	186727597	3	9.66E-11	1.33E-11	-	-0.269223	-0.3033	+	G	C	0.768511322	0.041324	+	G	C	0.7883	0.0448
rs12633034	LC_IGP179	IgG4_G1FS1	3	186727597	3	2.49E-08	6.42E-11	-	-0.231181	-0.293	+	G	C	0.768748476	0.0412715	+	G	C	0.7886	0.0448
rs12633034	LC_IGP_R92	IgG4_G1FS1/IgG4_G1F	3	186727597	3	2.50E-10	1.02E-12	-	-0.262126	-0.3213	+	G	C	0.768748476	0.0411694	+	G	C	0.7886	0.0451
rs12633034	LC_IGP_R93	IgG4_G2FS1/IgG4_G2F	3	186727597	3	7.29E-13	9.24E-14	-	-0.298052	-0.3348	+	G	C	0.768607383	0.0412149	+	G	C	0.7886	0.0449
rs10937278	LC_IGP36	IgG1 FG1S1/(FG1+FG1S1)	3	186727815	3	5.93E-14	3.20E-19	-	-0.308733	-0.412	+	A	T	0.768722324	0.040751	+	A	T	0.799	0.046
rs10937278	LC_IGP37	IgG1 FG2S1/(FG2+FG2S1)	3	186727815	3	6.37E-18	7.05E-12	-	-0.356114	-0.3058	+	A	T	0.768840659	0.0408102	+	A	T	0.799	0.0446
rs10937278	LC_IGP25	IgG1 SA per Gal	3	186727815	3	1.26E-09	1.55E-09	-	-0.25399	-0.2652	+	A	T	0.766203634	0.041578	+	A	T	0.7988	0.0439
rs10937278	LC_IGP7	IgG1_G1FS1	3	186727815	3	2.48E-12	3.86E-18	-	-0.288303	-0.3957	+	A	T	0.768722324	0.0408465	+	A	T	0.799	0.0456
rs10937278	LC_IGP_R34	IgG1_G1FS1/IgG1_G1F	3	186727815	3	5.33E-14	2.71E-19	-	-0.30921	-0.4136	+	A	T	0.768722324	0.0407364	+	A	T	0.799	0.046
rs10937278	LC_IGP_R35	IgG1_G2FS1/IgG1_G2F	3	186727815	3	6.04E-18	6.98E-12	-	-0.356358	-0.3059	+	A	T	0.768840659	0.0408088	+	A	T	0.799	0.0446
rs10937278	LC_IGP122	IgG2 FG1S1/(FG1+FG1S1)	3	186727815	3	3.26E-18	1.51E-25	-	-0.35919	-0.4767	+	A	T	0.768981696	0.040793	+	A	T	0.799	0.0456
rs77397204	LC_IGP123	IgG2 FG2S1/(FG2+FG2S1)	3	186738899	3	7.05E-09	9.15E-22	-	-0.366216	-0.6025	+	C	A	0.125240565	0.0629206	+	C	A	0.1609	0.0628
rs10937278	LC_IGP120	IgG2 FGS1/(FG+FGS1)	3	186727815	3	6.26E-11	1.31E-14	-	-0.27162	-0.3448	+	A	T	0.768840659	0.0412707	+	A	T	0.799	0.0448
rs10937278	LC_IGP111	IgG2 SA per Gal	3	186727815	3	4.41E-13	4.63E-22	-	-0.302965	-0.4408	+	A	T	0.769263777	0.041487	+	A	T	0.7986	0.0457
rs10937278	LC_IGP93	IgG2_G1FS1	3	186727815	3	1.21E-09	6.26E-18	-	-0.253304	-0.3844	+	A	T	0.768981696	0.0414248	+	A	T	0.799	0.0446
rs10937278	LC_IGP_R74	IgG2_G1FS1/IgG2_G1F	3	186727815	3	3.43E-18	1.67E-25	-	-0.358961	-0.4763	+	A	T	0.768981696	0.040796	+	A	T	0.799	0.0456
rs10937278	LC_IGP_R75	IgG2_G2FS1/IgG2_G2F	3	186727815	3	9.67E-13	6.23E-21	-	-0.295807	-0.428	+	A	T	0.768676632	0.0411294	+	A	T	0.799	0.0456
rs10937278	LC_IGP189	IgG4 FG1S1/(FG1+FG1S1)	3	186727815	3	2.54E-10	3.23E-13	-	-0.262079	-0.3266	+	A	T	0.768817683	0.0411784	+	A	T	0.799	0.0448
rs10937278	LC_IGP190	IgG4 FG2S1/(FG2+FG2S1)	3	186727815	3	7.57E-13	1.02E-13	-	-0.297882	-0.3326	+	A	T	0.768676632	0.0412209	+	A	T	0.799	0.0447
rs10937278	LC_IGP187	IgG4 FGS1/(FG+FGS1)	3	186727815	3	2.50E-08	1.88E-06	-	-0.231873	-0.2072	+	A	T	0.768676632	0.0414025	+	A	T	0.799	0.0435
rs10937278	LC_IGP186	IgG4 SA per Gal	3	186727815	3	1.00E-10	1.04E-11	-	-0.269018	-0.3032	+	A	T	0.768580783	0.0413288	+	A	T	0.7987	0.0446
rs10937278	LC_IGP179	IgG4_G1FS1	3	186727815	3	2.68E-08	1.61E-11	-	-0.230661	-0.3009	+	A	T	0.768817683	0.0412773	+	A	T	0.799	0.0446
rs10937278	LC_IGP_R92	IgG4_G1FS1/IgG4_G1F	3	186727815	3	2.66E-10	3.29E-13	-	-0.261749	-0.3265	+	A	T	0.768817683	0.0411749	+	A	T	0.799	0.0448
rs10937278	LC_IGP_R93	IgG4_G2FS1/IgG4_G2F	3	186727815	3	7.89E-13	1.02E-13	-	-0.29764	-0.3326	+	A	T	0.768676632	0.0412208	+	A	T	0.799	0.0447
rs10937279	LC_IGP36	IgG1 FG1S1/(FG1+FG1S1)	3	186727836	3	6.02E-14	1.23E-18	-	-0.308652	-0.4051	+	G	A	0.768714373	0.0407514	+	G	A	0.7981	0.046
rs10937279	LC_IGP37	IgG1 FG2S1/(FG2+FG2S1)	3	186727836	3	6.54E-18	1.26E-11	-	-0.355995	-0.3024	+	G	A	0.768832723	0.0408109	+	G	A	0.7981	0.0446
rs10937279	LC_IGP25	IgG1 SA per Gal	3	186727836	3	1.28E-09	2.43E-09	-	-0.253866	-0.2621	+	G	A	0.766195489	0.0415785	+	G	A	0.7979	0.0439
rs10937279	LC_IGP7	IgG1_G1FS1	3	186727836	3	2.52E-12	1.47E-17	-	-0.288211	-0.3887	+	G	A	0.768714373	0.0408469	+	G	A	0.7981	0.0456
rs10937279	LC_IGP_R34	IgG1_G1FS1/IgG1_G1F	3	186727836	3	5.41E-14	1.09E-18	-	-0.309127	-0.4064	+	G	A	0.768714373	0.0407368	+	G	A	0.7981	0.046
rs10937279	LC_IGP_R35	IgG1_G2FS1/IgG1_G2F	3	186727836	3	6.20E-18	1.25E-11	-	-0.356238	-0.3025	+	G	A	0.768832723	0.0408095	+	G	A	0.7981	0.0446
rs10937279	LC_IGP122	IgG2 FG1S1/(FG1+FG1S1)	3	186727836	3	3.30E-18	6.14E-25	-	-0.359129	-0.4705	+	G	A	0.768973764	0.0407933	+	G	A	0.7981	0.0456
rs77530527	LC_IGP123	IgG2 FG2S1/(FG2+FG2S1)	3	186738900	3	7.06E-09	1.73E-21	-	-0.366213	-0.6007	+	C	T	0.12524039	0.0629206	+	C	T	0.1594	0.0631
rs10937279	LC_IGP120	IgG2 FGS1/(FG+FGS1)	3	186727836	3	6.36E-11	2.20E-14	-	-0.271515	-0.3416	+	G	A	0.768832723	0.0412711	+	G	A	0.7981	0.0447
rs10937279	LC_IGP111	IgG2 SA per Gal	3	186727836	3	4.46E-13	1.14E-21	-	-0.302913	-0.4365	+	G	A	0.769256656	0.0414873	+	G	A	0.7977	0.0456
rs10937279	LC_IGP93	IgG2_G1FS1	3	186727836	3	1.23E-09	1.88E-17	-	-0.253194	-0.3788	+	G	A	0.768973764	0.0414253	+	G	A	0.7981	0.0446
rs10937279	LC_IGP_R74	IgG2_G1FS1/IgG2_G1F	3	186727836	3	3.48E-18	6.79E-25	-	-0.358901	-0.47	+	G	A	0.768973764	0.0407963	+	G	A	0.7981	0.0456
rs10937279	LC_IGP_R75	IgG2_G2FS1/IgG2_G2F	3	186727836	3	9.86E-13	1.39E-20	-	-0.295697	-0.4242	+	G	A	0.7686687	0.0411299	+	G	A	0.7981	0.0456
rs10937279	LC_IGP189	IgG4 FG1S1/(FG1+FG1S1)	3	186727836	3	2.59E-10	6.96E-13	-	-0.261947	-0.3222	+	G	A	0.768809756	0.041179	+	G	A	0.7981	0.0449
rs10937279	LC_IGP190	IgG4 FG2S1/(FG2+FG2S1)	3	186727836	3	7.76E-13	1.42E-13	-	-0.297742	-0.331	+	G	A	0.7686687	0.0412215	+	G	A	0.7981	0.0448
rs10937279	LC_IGP187	IgG4 FGS1/(FG+FGS1)	3	186727836	3	2.56E-08	1.95E-06	-	-0.231719	-0.2069	+	G	A	0.7686687	0.041403	+	G	A	0.7981	0.0435
rs10937279	LC_IGP186	IgG4 SA per Gal	3	186727836	3	1.02E-10	1.40E-11	-	-0.268885	-0.3014	+	G	A	0.768572827	0.0413293	+	G	A	0.7978	0.0446
rs10937279	LC_IGP179	IgG4_G1FS1	3	186727836	3	2.74E-08	4.11E-11	-	-0.230522	-0.2949	+	G	A	0.768809756	0.0412778	+	G	A	0.7981	0.0447
rs10937279	LC_IGP_R92	IgG4_G1FS1/IgG4_G1F	3	186727836	3	2.72E-10	7.08E-13	-	-0.261611	-0.3221	+	G	A	0.768809756	0.0411754	+	G	A	0.7981	0.0449
rs10937279	LC_IGP_R93	IgG4_G2FS1/IgG4_G2F	3	186727836	3	8.09E-13	1.42E-13	-	-0.2975	-0.331	+	G	A	0.7686687	0.0412214	+	G	A	0.7981	0.0448
rs7619468	LC_IGP36	IgG1 FG1S1/(FG1+FG1S1)	3	186728178	3	2.18E-13	3.26E-19	-	-0.302482	-0.4119	+	C	T	0.76941896	0.0408813	+	C	T	0.7991	0.046
rs7619468	LC_IGP37	IgG1 FG2S1/(FG2+FG2S1)	3	186728178	3	2.51E-17	7.18E-12	-	-0.350556	-0.3057	+	C	T	0.76953602	0.0409424	+	C	T	0.7991	0.0446
rs7619468	LC_IGP25	IgG1 SA per Gal	3	186728178	3	3.15E-09	1.60E-09	-	-0.248427	-0.265	+	C	T	0.766917293	0.0417027	+	C	T	0.7988	0.0439
rs7619468	LC_IGP7	IgG1_G1FS1	3	186728178	3	1.00E-11	4.33E-18	-	-0.280941	-0.3951	+	C	T	0.76941896	0.0409792	+	C	T	0.7991	0.0456
rs7619468	LC_IGP_R34	IgG1_G1FS1/IgG1_G1F	3	186728178	3	1.96E-13	2.76E-19	-	-0.302989	-0.4134	+	C	T	0.76941896	0.0408666	+	C	T	0.7991	0.046
rs7619468	LC_IGP_R35	IgG1_G2FS1/IgG1_G2F	3	186728178	3	2.39E-17	7.10E-12	-	-0.350783	-0.3058	+	C	T	0.76953602	0.0409411	+	C	T	0.7991	0.0446

rs7619468	LC_IGP122	IgG2 FG1S1/(FG1+FG1S1)	3	186728178	3	4.58E-18	1.53E-25	-	-0.358487	-0.4766	+	C	T	0.769676632	0.0408996	+	C	T	0.7991	0.0456
rs3872721	LC_IGP123	IgG2 FG2S1/(FG2+FG2S1)	3	186725491	3	9.33E-13	5.07E-21	-	-0.296886	-0.4285	+	A	G	0.768122941	0.0412503	+	A	G	0.7979	0.0456
rs7619468	LC_IGP120	IgG2 FGS1/(FG+FGS1)	3	186728178	3	8.53E-11	1.33E-14	-	-0.27036	-0.3447	+	C	T	0.76953602	0.0413777	+	C	T	0.7991	0.0448
rs7619468	LC_IGP111	IgG2 SA per Gal	3	186728178	3	6.22E-13	4.68E-22	-	-0.301785	-0.4408	+	C	T	0.76996904	0.0415978	+	C	T	0.7986	0.0457
rs7619468	LC_IGP93	IgG2_G1FS1	3	186728178	3	2.53E-09	7.08E-18	-	-0.248954	-0.3838	+	C	T	0.769676632	0.0415429	+	C	T	0.7991	0.0446
rs7619468	LC_IGP_R74	IgG2_G1FS1/IgG2_G1F	3	186728178	3	4.81E-18	1.70E-25	-	-0.358271	-0.4762	+	C	T	0.769676632	0.0409025	+	C	T	0.7991	0.0456
rs7619468	LC_IGP_R75	IgG2_G2FS1/IgG2_G2F	3	186728178	3	1.87E-12	6.25E-21	-	-0.292795	-0.428	+	C	T	0.769371568	0.0412445	+	C	T	0.7991	0.0456
rs7619468	LC_IGP189	IgG4 FG1S1/(FG1+FG1S1)	3	186728178	3	5.01E-10	3.15E-13	-	-0.258366	-0.3267	+	C	T	0.769512195	0.0412942	+	C	T	0.7991	0.0448
rs7619468	LC_IGP190	IgG4 FG2S1/(FG2+FG2S1)	3	186728178	3	2.18E-12	1.02E-13	-	-0.292588	-0.3326	+	C	T	0.769371568	0.0413463	+	C	T	0.7991	0.0447
rs7619468	LC_IGP186	IgG4 SA per Gal	3	186728178	3	2.21E-10	1.02E-11	-	-0.264707	-0.3033	+	C	T	0.769277846	0.041448	+	C	T	0.7988	0.0446
rs7619468	LC_IGP_R92	IgG4_G1FS1/IgG4_G1F	3	186728178	3	5.34E-10	3.20E-13	-	-0.257917	-0.3266	+	C	T	0.769512195	0.041291	+	C	T	0.7991	0.0448
rs7619468	LC_IGP_R93	IgG4_G2FS1/IgG4_G2F	3	186728178	3	2.27E-12	1.02E-13	-	-0.292353	-0.3326	+	C	T	0.769371568	0.0413461	+	C	T	0.7991	0.0447
rs4686828	LC_IGP36	IgG1 FG1S1/(FG1+FG1S1)	3	186728255	3	6.30E-14	3.28E-19	-	-0.308421	-0.4118	+	G	T	0.76877737	0.0407532	+	G	T	0.7991	0.046
rs4686828	LC_IGP37	IgG1 FG2S1/(FG2+FG2S1)	3	186728255	3	7.44E-18	7.21E-12	-	-0.355401	-0.3057	+	G	T	0.768895604	0.0408147	+	G	T	0.7991	0.0446
rs4686828	LC_IGP25	IgG1 SA per Gal	3	186728255	3	1.39E-09	1.61E-09	-	-0.253353	-0.265	+	G	T	0.766260025	0.0415811	+	G	T	0.7989	0.0439
rs4686828	LC_IGP7	IgG1_G1FS1	3	186728255	3	2.67E-12	4.40E-18	-	-0.287908	-0.3951	+	G	T	0.76877737	0.0408489	+	G	T	0.7991	0.0456
rs4686828	LC_IGP_R34	IgG1_G1FS1/IgG1_G1F	3	186728255	3	5.66E-14	2.78E-19	-	-0.308893	-0.4134	+	G	T	0.76877737	0.0407386	+	G	T	0.7991	0.046
rs4686828	LC_IGP_R35	IgG1_G2FS1/IgG1_G2F	3	186728255	3	7.06E-18	7.13E-12	-	-0.355641	-0.3058	+	G	T	0.768895604	0.0408133	+	G	T	0.7991	0.0446
rs4686828	LC_IGP122	IgG2 FG1S1/(FG1+FG1S1)	3	186728255	3	3.61E-18	1.55E-25	-	-0.358727	-0.4766	+	G	T	0.769036608	0.0407962	+	G	T	0.7991	0.0456
rs4686830	LC_IGP123	IgG2 FG2S1/(FG2+FG2S1)	3	186728565	3	9.32E-13	6.05E-21	-	-0.295342	-0.4281	+	G	A	0.766173276	0.0410354	+	G	A	0.7991	0.0456
rs4686828	LC_IGP120	IgG2 FGS1/(FG+FGS1)	3	186728255	3	6.78E-11	1.33E-14	-	-0.271129	-0.3447	+	G	T	0.768895604	0.0412734	+	G	T	0.7991	0.0448
rs4686828	LC_IGP111	IgG2 SA per Gal	3	186728255	3	4.60E-13	4.69E-22	-	-0.302737	-0.4407	+	G	T	0.769320433	0.0414889	+	G	T	0.7986	0.0457
rs4686828	LC_IGP93	IgG2_G1FS1	3	186728255	3	1.37E-09	7.19E-18	-	-0.252485	-0.3838	+	G	T	0.769036608	0.0414286	+	G	T	0.7991	0.0446
rs4686828	LC_IGP_R74	IgG2_G1FS1/IgG2_G1F	3	186728255	3	3.80E-18	1.72E-25	-	-0.358497	-0.4762	+	G	T	0.769036608	0.0407992	+	G	T	0.7991	0.0456
rs4686828	LC_IGP_R75	IgG2_G2FS1/IgG2_G2F	3	186728255	3	1.04E-12	6.22E-21	-	-0.295425	-0.428	+	G	T	0.768731544	0.0411317	+	G	T	0.7991	0.0456
rs4686828	LC_IGP189	IgG4 FG1S1/(FG1+FG1S1)	3	186728255	3	2.76E-10	3.15E-13	-	-0.261543	-0.3267	+	G	T	0.768872561	0.0411812	+	G	T	0.7991	0.0448
rs4686828	LC_IGP190	IgG4 FG2S1/(FG2+FG2S1)	3	186728255	3	8.45E-13	1.01E-13	-	-0.297273	-0.3326	+	G	T	0.768731544	0.0412243	+	G	T	0.7991	0.0447
rs4686828	LC_IGP187	IgG4 FGS1/(FG+FGS1)	3	186728255	3	2.77E-08	1.88E-06	-	-0.231149	-0.2072	+	G	T	0.768731544	0.0414056	+	G	T	0.7991	0.0435
rs4686828	LC_IGP186	IgG4 SA per Gal	3	186728255	3	1.07E-10	1.02E-11	-	-0.268635	-0.3033	+	G	T	0.768635863	0.041331	+	G	T	0.7988	0.0446
rs4686828	LC_IGP179	IgG4_G1FS1	3	186728255	3	2.96E-08	1.68E-11	-	-0.229959	-0.3006	+	G	T	0.768872561	0.0412804	+	G	T	0.7991	0.0446
rs4686828	LC_IGP_R92	IgG4_G1FS1/IgG4_G1F	3	186728255	3	2.91E-10	3.20E-13	-	-0.261189	-0.3266	+	G	T	0.768872561	0.0411777	+	G	T	0.7991	0.0448
rs4686828	LC_IGP_R93	IgG4_G2FS1/IgG4_G2F	3	186728255	3	8.80E-13	1.01E-13	-	-0.297032	-0.3326	+	G	T	0.768731544	0.0412241	+	G	T	0.7991	0.0447
rs4686829	LC_IGP36	IgG1 FG1S1/(FG1+FG1S1)	3	186728319	3	1.83E-11	2.68E-17	-	-0.320672	-0.436	+	G	T	0.841931885	0.0473911	+	G	T	0.8418	0.0515
rs4686829	LC_IGP37	IgG1 FG2S1/(FG2+FG2S1)	3	186728319	3	4.03E-16	2.59E-10	-	-0.389788	-0.3146	+	G	T	0.841916137	0.0474077	+	G	T	0.8418	0.0498
rs4686829	LC_IGP25	IgG1 SA per Gal	3	186728319	3	2.76E-10	2.97E-09	-	-0.305037	-0.2908	+	G	T	0.839949023	0.048018	+	G	T	0.8416	0.049
rs4686829	LC_IGP7	IgG1_G1FS1	3	186728319	3	3.99E-10	7.73E-15	-	-0.298817	-0.3989	+	G	T	0.841931885	0.0474831	+	G	T	0.8418	0.0513
rs4686829	LC_IGP_R34	IgG1_G1FS1/IgG1_G1F	3	186728319	3	1.56E-11	2.49E-17	-	-0.321688	-0.437	+	G	T	0.841931885	0.0473726	+	G	T	0.8418	0.0516
rs4686829	LC_IGP_R35	IgG1_G2FS1/IgG1_G2F	3	186728319	3	4.04E-16	2.56E-10	-	-0.389771	-0.3147	+	G	T	0.841916137	0.0474077	+	G	T	0.8418	0.0498
rs4686829	LC_IGP122	IgG2 FG1S1/(FG1+FG1S1)	3	186728319	3	4.56E-17	6.60E-20	-	-0.401988	-0.4673	+	G	T	0.842012588	0.0473479	+	G	T	0.8418	0.0512
rs4686828	LC_IGP123	IgG2 FG2S1/(FG2+FG2S1)	3	186728255	3	1.05E-12	6.12E-21	-	-0.295314	-0.428	+	G	T	0.768731544	0.041131	+	G	T	0.7991	0.0456
rs4686829	LC_IGP120	IgG2 FGS1/(FG+FGS1)	3	186728319	3	1.20E-11	1.39E-11	-	-0.326251	-0.3386	+	G	T	0.841916137	0.0477757	+	G	T	0.8418	0.0501
rs4686829	LC_IGP111	IgG2 SA per Gal	3	186728319	3	4.36E-13	6.34E-17	-	-0.350497	-0.4271	+	G	T	0.842142496	0.0479838	+	G	T	0.8413	0.0511
rs4686829	LC_IGP93	IgG2_G1FS1	3	186728319	3	5.39E-10	2.25E-14	-	-0.299652	-0.3819	+	G	T	0.842012588	0.0479818	+	G	T	0.8418	0.05
rs4686829	LC_IGP_R74	IgG2_G1FS1/IgG2_G1F	3	186728319	3	4.51E-17	7.07E-20	-	-0.402067	-0.4669	+	G	T	0.842012588	0.0473495	+	G	T	0.8418	0.0512
rs4686829	LC_IGP_R75	IgG2_G2FS1/IgG2_G2F	3	186728319	3	9.09E-12	1.11E-15	-	-0.327762	-0.4091	+	G	T	0.841707525	0.0477091	+	G	T	0.8418	0.051
rs4686829	LC_IGP190	IgG4 FG2S1/(FG2+FG2S1)	3	186728319	3	7.49E-09	1.04E-08	-	-0.278961	-0.2859	+	G	T	0.841707525	0.0480142	+	G	T	0.8418	0.05
rs4686829	LC_IGP186	IgG4 SA per Gal	3	186728319	3	7.74E-09	7.67E-08	-	-0.278729	-0.2676	+	G	T	0.841835149	0.048019	+	G	T	0.8417	0.0498
rs4686829	LC_IGP_R93	IgG4_G2FS1/IgG4_G2F	3	186728319	3	7.75E-09	1.03E-08	-	-0.278682	-0.2861	+	G	T	0.841707525	0.0480139	+	G	T	0.8418	0.05
rs4686830	LC_IGP36	IgG1 FG1S1/(FG1+FG1S1)	3	186728565	3	4.65E-14	3.40E-19	-	-0.309321	-0.4117	+	G	A	0.766213456	0.0406538	+	G	A	0.7991	0.046
rs4686830	LC_IGP37	IgG1 FG2S1/(FG2+FG2S1)	3	186728565	3	6.00E-18	7.32E-12	-	-0.355587	-0.3056	+	G	A	0.766336386	0.0407173	+	G	A	0.7991	0.0446
rs4686830	LC_IGP25	IgG1 SA per Gal	3	186728565	3	9.19E-10	1.66E-09	-	-0.255507	-0.2648	+	G	A	0.763930451	0.0414782	+	G	A	0.7989	0.0439
rs4686830	LC_IGP7	IgG1_G1FS1	3	186728565	3	2.25E-12	4.73E-18	-	-0.288221	-0.3948	+	G	A	0.766213456	0.0407525	+	G	A	0.7991	0.0456
rs4686830	LC_IGP_R34	IgG1_G1FS1/IgG1_G1F	3	186728565	3	4.20E-14	2.87E-19	-	-0.309771	-0.4132	+	G	A	0.766213456	0.0406393	+	G	A	0.7991	0.046
rs4686830	LC_IGP_R35	IgG1_G2FS1/IgG1_G2F	3	186728565	3	5.70E-18	7.24E-12	-	-0.355828	-0.3057	+	G	A	0.766336386	0.0407159	+	G	A	0.7991	0.0446
rs4686830	LC_IGP122	IgG2 FG1S1/(FG1+FG1S1)	3	186728565	3	3.36E-18	1.64E-25	-	-0.358244	-0.4764	+	G	A	0.76647834	0.0407023	+	G	A	0.7991	0.0456
rs10937278	LC_IGP123	IgG2 FG2S1/(FG2+FG2S1)	3	186727815	3	9.85E-13	6.13E-21	-	-0.295695	-0.428	+	A	T	0.768676632	0.0411286	+	A	T	0.799	0.0456
rs4686830	LC_IGP120	IgG2 FGS1/(FG+FGS1)	3	186728565	3	7.25E-11	1.36E-14	-	-0.27011	-0.3446	+	G	A	0.766335775	0.0411819	+	G	A	0.7991	0.0448
rs4686830	LC_IGP111	IgG2 SA per Gal	3	186728565	3	5.81E-13	4.79E-22	-	-0.300729	-0.4406	+	G	A	0.766729721	0.0413981	+	G	A	0.7987	0.0457
rs4686830	LC_IGP93	IgG2_G1FS1	3	186728565	3	1.98E-09	7.80E-18	-	-0.249444	-0.3835	+	G	A	0.76647834	0.0413442	+	G	A	0.7991	0.0446
rs4686830	LC_IGP_R74	IgG2_G1FS1/IgG2_G1F	3	186728565	3	3.56E-18	1.82E-25	-	-0.357995	-0.4759	+	G	A	0.76647834	0.0407054	+	G	A	0.7991	0.0456
rs4686830	LC_IGP_R75	IgG2_G2FS1/IgG2_G2F	3	186728565	3	9.17E-13	6.15E-21	-	-0.295441	-0.428	+	G	A	0.766173276	0.0410362	+	G	A	0.7991	0.0456
rs4686830	LC_IGP189	IgG4 FG1S1/(FG1+FG1S1)	3	186728565	3	2.96E-10	3.14E-13	-	-0.260528	-0.3267	+	G	A	0.766316463	0.04109	+	G	A	0.7991	0.0448
rs4686830	LC_IGP190	IgG4 FG2S1/(FG2+FG2S1)	3	186728565	3	2.11E-12	9.94E-14	-	-0.291429	-0.3327	+	G	A	0.766173887	0.041154	+	G	A	0.7991	0.0447
rs4686830	LC_IGP186	IgG4 SA per Gal	3	186728565	3	2.58E-10	1.03E-11	-	-0.262494	-0.3032	+	G	A	0.766070379	0.0412597	+	G	A	0.7988	0.0446
rs4686830	LC_IGP179	IgG4_G1FS1	3	186728565	3	2.89E-08	1.70E-11	-	-0.229625	-0.3005	+	G	A	0.766316463	0.0411867	+	G	A	0.7991	0.0446
rs4686830	LC_IGP_R92	IgG4_G1FS1/IgG4_G1F	3	186728565	3	3.14E-10	3.19E-13	-	-0.260108	-0.3267	+	G	A	0.766316463	0.0410868	+	G	A	0.7991	0.0448
rs4686830	LC_IGP_R93	IgG4_G2FS1/IgG4_G2F	3	186728565	3	2.20E-12	9.99E-14	-	-0.291193	-0.3327	+	G	A	0.766173887	0.0411539	+	G	A	0.7991	0.0447
rs4686831	LC_IGP36	IgG1 FG1S1/(FG1+FG1S1)	3	186728647	3	3.57E-14	4.66E-18	-	-0.31062	-0.4007	+	T	C	0.765250459	0.0406362	+	T	C	0.777	0.0463
rs4686831	LC_IGP37	IgG1 FG2S1/(FG2+FG2S1)	3	186728647	3	3.21E-18	3.21E-11	-	-0.358362	-0.2964	+	T	C	0.765375153	0.0406908	+	T	C	0.777	0.0447
rs4686831	LC_IGP25	IgG1 SA per Gal	3	186728647	3	7.77E-10	8.75E-09	-	-0.256528	-0.2517	+	T	C	0.762966792	0.041461	+	T	C	0.7768	0.0438
rs4686831	LC_IGP7	IgG1_G1FS1	3	186728647	3	2.80E-12	8.00E-17	-	-0.286898	-0.3821	+	T	C	0.765250459	0.0407468	+	T	C	0.777	0.0459
rs4686831	LC_IGP_R34	IgG1_G1FS1/IgG1_G1F	3	186728647	3	3.20E-14	3.68E-18	-	-0.311099	-0.4026	+	T	C	0.765250459	0.0406216	+	T	C	0.777	0.0463
rs4686831	LC_IGP_R35	IgG1_G2FS1/IgG1_G2F	3	186728647	3	3.04E-18	3.20E-11	-	-0.358604	-0.2964	+	T	C	0.765375153	0.0406894	+	T	C	0.777	0.0447
rs4686831	LC_IGP122	IgG2 FG1S1/(FG1+FG1S1)	3	186728647	3	3.73E-18	7.10E-24	-	-0.357666	-0.4622	+	T	C	0.765517389	0.0406937	+	T	C	0.777	0.0459
rs7619468	LC_IGP123	IgG2 FG2S1/(FG2+FG2S1)	3	186728178	3	1.90E-12	6.15E-21	-	-0.292682	-0.428	+	C	T	0.769371568	0.0412438	+	C	T	0.7991	0.0456
rs4686831	LC_IGP120	IgG2 FGS1/(FG+FGS1)	3	186728647	3	8.18E-11	1.92E-13	-	-0.269289	-0.3293	+	T	C	0.765374237	0.0411737	+	T	C	0.777	0.0448
rs4686831	LC_IGP111	IgG2 SA per Gal	3	186728647	3	6.52E-13	1.11E-20	-	-0.299999	-0.4271	+	T	C	0.765758204	0.0413889	+	T	C	0.7767	0.0458
rs4686831	LC_IGP93	IgG2_G1FS1	3	186728647	3	2.21E-09	1.99E-16	-	-0.248662	-0.3679	+	T	C	0.765517389	0.0413355	+	T	C	0.777	0.0447
rs4686831	LC_IGP_R74	IgG2_G1FS1/IgG2_G1F	3	186728647	3	3.93E-18	7.86E-24	-	-0.357439	-0.4618	+	T	C	0.765517389	0.0406967	+	T	C	0.777	0.0459
rs4686831	LC_IGP_R75	IgG2_G2FS1/IgG2_G2F	3	186728647	3	7.65E-13	1.71E-19	-	-0.296371	-0.4123	+	T	C	0.765212325	0.0410206	+	T	C	0.777	0.0457
rs4686831	LC_IGP189	IgG4 FG1S1/(FG1+FG1S1)	3	186728647	3	1.65E-10	1.22E-13	-	-0.264131	-0.3336	+	T	C	0.765356402	0.0410644	+	T	C	0.777	0.045
rs4686831	LC_IGP190	IgG4 FG2S1/(FG2+FG2S1)	3	186728647	3	3.70E-12	1.64E-13	-	-0.288125	-0.3305	+	T	C	0.76521324	0.0411567	+	T	C	0.777	0.0448
rs4686831	LC_IGP186	IgG4 SA per Gal	3	186728647	3	1.80E-10	8.65E-12	-	-0.264696	-0.3049	+	T	C	0.765106793	0.0412396	+	T	C	0.7768	0.0447
rs4686831	LC_IGP179	IgG4_G1FS1	3	186728647	3	1.55E-08	5.33E-12	-	-0.23399	-0.3091	+	T	C	0.765356402	0.0411602	+	T	C	0.777	0.0448
rs4686831	LC_IGP_R92	IgG4_G1FS1/IgG4_G1F	3	186728647	3	1.77E-10	1.24E-13	-	-0.263643	-0.3335	+	T	C	0.765356402	0.0410615	+	T	C	0.777	0.045
rs4686831	LC_IGP_R93	IgG4_G2FS1/IgG4_G2F	3	186728647	3	3.84E-12	1.64E-13	-	-0.287901	-0.3306	+	T	C	0.76521324	0.0411565	+	T	C	0.777	0.0448
rs10804908	LC_IGP36	IgG1 FG1S1/(FG1+FG1S1)	3	186728842	3	4.21E-14	4.91E-19	-	-0.309797	-0.4098	+	A	T	0.765981346	0.0406458	+	A	T	0.7985	0.046
rs10804908	LC_IGP37	IgG1 FG2S1/(FG2+FG2S1)	3	186728842	3	5.31E-18	9.19E-12	-	-0.356115	-0.3042	+	A	T	0.766093407	0.04071	+	A	T	0.7985	0.0446
rs10804908	LC_IGP25	IgG1 SA per Gal	3	186728842	3	8.05E-10	2.06E-09	-	-0.256338	-0.2632	+	A	T	0.763692669	0.0414693	+	A	T	0.7983	0.0439
rs10804908	LC_IGP7	IgG1_G1FS1	3	186728842	3	1.96E-12	7.38E-18	-	-0.288964	-0.3925	+	A	T	0.765981346	0.0407436	+	A	T	0.7985	0.0456
rs10804908	LC_IGP_R34	IgG1_G1FS1/IgG1_G1F	3	186728842	3	3.82E-14	4.08E-19	-	-0.310228	-0.4114	+	A	T	0.765981346	0.0406315	+	A	T	0.7985	0.046
rs10804908	LC_IGP_R35	IgG1_G2FS1/IgG1_G2F	3	186728842	3	5.03E-18	9.09E-12	-	-0.356358	-0.3042	+	A	T	0.766093407	0.0407085	+	A	T	0.7985	0.0446
rs10804908	LC_IGP122	IgG2 FG1S1/(FG1+FG1S1)	3	186728842	3	2.47E-18	2.38E-25	-	-0.359607	-0.4747	+	A	T	0.766235204	0.0406904	+	A	T	0.7985	0.0456
rs4686836	LC_IGP123	IgG2 FG2S1/(FG2+FG2S1)	3	186729365	3	9.43E-13	8.00E-21	-	-0.295399	-0.4266	+	T	C	0.766118365	0.0410524	+	T	C	0.7985	0.0456
rs10804908	LC_IGP120	IgG2 FGS1/(FG+FGS1)	3	186728842	3	6.25E-11	1.95E-14	-	-0.270988	-0.3424	+	A	T	0.766092491	0.0411739	+	A	T	0.7985	0.0447
rs10804908	LC_IGP111	IgG2 SA per Gal	3	186728842	3	4.86E-13	7.35E-22	-	-0.301685	-0.4386	+	A	T	0.766495356	0.0413877	+	A	T	0.7981	0.0456
rs10804908	LC_IGP93	IgG2_G1FS1	3	186728842	3	1.61E-09	1.02E-17	-	-0.250781	-0.3821	+	A	T	0.766235204	0.0413347	+	A	T	0.7985	0.0446
rs10804908	LC_IGP_R74	IgG2_G1FS1/IgG2_G1F	3	186728842	3	2.61E-18	2.65E-25	-	-0.359371	-0.4743	+	A	T	0.766235204	0.0406934	+	A	T	0.7985	0.0456
rs10804908	LC_IGP_R75	IgG2_G2FS1/IgG2_G2F	3	186728842	3	8.73E-13	1.04E-20	-	-0.295683	-0.4255	+	A	T	0.76593014	0.0410307	+	A	T	0.7985	0.0456
rs10804908	LC_IGP189	IgG4 FG1S1/(FG1+FG1S1)	3	186728842	3	2.60E-10	3.28E-13	-	-0.26131	-0.3264	+	A	T	0.76607378	0.0410825	+	A	T	0.7985	0.0448
rs10804908	LC_IGP190	IgG4 FG2S1/(FG2+FG2S1)	3	186728842	3	1.95E-12	1.27E-13	-	-0.291833	-0.3312	+	A	T	0.765942343	0.0411465	+	A	T	0.7985	0.0447
rs10804908	LC_IGP186	IgG4 SA per Gal	3	186728842	3	2.39E-10	1.25E-11	-	-0.262945	-0.3019	+	A	T	0.765838127	0.0412522	+	A	T	0.7983	0.0446
rs10804908	LC_IGP179	IgG4_G1FS1	3	186728842	3	2.49E-08	2.06E-11	-	-0.230665	-0.2992	+	A	T	0.76607378	0.0411786	+	A	T	0.7985	0.0446
rs10804908	LC_IGP_R92	IgG4_G1FS1/IgG4_G1F	3	186728842	3	2.77E-10	3.33E-13	-	-0.260891	-0.3263	+	A	T	0.76607378	0.0410792	+	A	T	0.7985	0.0448
rs10804908	LC_IGP_R93	IgG4_G2FS1/IgG4_G2F	3	186728842	3	2.03E-12	1.27E-13	-	-0.291592	-0.3313	+	A	T	0.765942343	0.0411464	+	A	T	0.7985	0.0447
rs4686832	LC_IGP36	IgG1 FG1S1/(FG1+FG1S1)	3	186729000	3	4.82E-14	1.51E-18	-	-0.309261	-0.4029	+	T	C	0.764814679	0.0406713	+	T	C	0.7752	0.0458
rs4686832	LC_IGP37	IgG1 FG2S1/(FG2+FG2S1)	3	186729000	3	7.55E-18	1.57E-11	-	-0.354673	-0.2988	+	T	C	0.764940171	0.0407396	+	T	C	0.7752	0.0443
rs4686832	LC_IGP25	IgG1 SA per Gal	3	186729000	3	9.82E-10	1.32E-09	-	-0.255172	-0.2645	+	T	C	0.762516291	0.0414967	+	T	C	0.7752	0.0436
rs4686832	LC_IGP7	IgG1_G1FS1	3	186729000	3	1.36E-12	1.77E-17	-	-0.291169	-0.3852	+	T	C	0.764814679	0.0407569	+	T	C	0.7752	0.0453
rs4686832	LC_IGP_R34	IgG1_G1FS1/IgG1_G1F	3	186729000	3	4.35E-14	1.28E-18	-	-0.309711	-0.4045	+	T	C	0.764814679	0.0406569	+	T	C	0.7752	0.0459
rs4686832	LC_IGP_R35	IgG1_G2FS1/IgG1_G2F	3	186729000	3	7.14E-18	1.55E-11	-	-0.354933	-0.2988	+	T	C	0.764940171	0.0407381	+	T	C	0.7752	0.0443
rs4686832	LC_IGP122	IgG2 FG1S1/(FG1+FG1S1)	3	186729000	3	2.35E-18	3.58E-24	-	-0.36001	-0.4619	+	T	C	0.765082367	0.0407101	+	T	C	0.7752	0.0455
rs4012257	LC_IGP123	IgG2 FG2S1/(FG2+FG2S1)	3	186730396	3	9.38E-13	9.85E-21	-	-0.295438	-0.426	+	C	A	0.766148613	0.0410535	+	C	A	0.7986	0.0456
rs4686832	LC_IGP120	IgG2 FGS1/(FG+FGS1)	3	186729000	3	5.61E-11	8.25E-14	-	-0.27179	-0.333	+	T	C	0.76493895	0.0411925	+	T	C	0.7752	0.0446
rs4686832	LC_IGP111	IgG2 SA per Gal	3	186729000	3	3.98E-13	1.00E-20	-	-0.302948	-0.4247	+	T	C	0.76533808	0.0414029	+	T	C	0.7747	0.0455
rs4686832	LC_IGP93	IgG2_G1FS1	3	186729000	3	1.23E-09	1.55E-16	-	-0.252712	-0.3664	+	T	C	0.765082367	0.0413492	+	T	C	0.7752	0.0444
rs4686832	LC_IGP_R74	IgG2_G1FS1/IgG2_G1F	3	186729000	3	2.48E-18	3.91E-24	-	-0.359776	-0.4615	+	T	C	0.765082367	0.0407131	+	T	C	0.7752	0.0455
rs4686832	LC_IGP_R75	IgG2_G2FS1/IgG2_G2F	3	186729000	3	6.89E-13	9.84E-20	-	-0.297164	-0.4129	+	T	C	0.764777303	0.041046	+	T	C	0.7752	0.0454
rs4686832	LC_IGP189	IgG4 FG1S1/(FG1+FG1S1)	3	186729000	3	3.10E-10	5.58E-13	-	-0.260328	-0.3211	+	T	C	0.764921951	0.0411079	+	T	C	0.7752	0.0445

rs4686832	LC_IGP190	IgG4 FG2S1/(FG2+FG2S1)	3	186729000	3	4.44E-12	3.94E-13	-	-0.287279	-0.3219	+	T	C	0.764778523	0.0411892	+	T	C	0.7752	0.0444
rs4686832	LC_IGP186	IgG4 SA per Gal	3	186729000	3	3.30E-10	2.69E-11	-	-0.261032	-0.2946	+	T	C	0.764670747	0.0412828	+	T	C	0.775	0.0442
rs4686832	LC_IGP179	IgG4_G1FS1	3	186729000	3	2.08E-08	2.28E-11	-	-0.232073	-0.2959	+	T	C	0.764921951	0.0411954	+	T	C	0.7752	0.0442
rs4686832	LC_IGP_R92	IgG4_G1FS1/IgG4_G1F	3	186729000	3	3.29E-10	5.54E-13	-	-0.259931	-0.3212	+	T	C	0.764921951	0.0411046	+	T	C	0.7752	0.0445
rs4686832	LC_IGP_R93	IgG4_G2FS1/IgG4_G2F	3	186729000	3	4.61E-12	3.97E-13	-	-0.287051	-0.3219	+	T	C	0.764778523	0.041189	+	T	C	0.7752	0.0444
rs4686833	LC_IGP36	IgG1 FG1S1/(FG1+FG1S1)	3	186729012	3	9.41E-14	2.72E-18	-	-0.305175	-0.4072	+	C	A	0.764783698	0.0406152	+	C	A	0.768	0.0467
rs4686833	LC_IGP37	IgG1 FG2S1/(FG2+FG2S1)	3	186729012	3	1.14E-17	8.76E-12	-	-0.352149	-0.3062	+	C	A	0.764909247	0.0406771	+	C	A	0.768	0.0449
rs4686833	LC_IGP25	IgG1 SA per Gal	3	186729012	3	1.12E-09	1.15E-09	-	-0.253847	-0.2674	+	C	A	0.762474842	0.0414256	+	C	A	0.7679	0.0439
rs4686833	LC_IGP7	IgG1_G1FS1	3	186729012	3	3.15E-12	1.77E-16	-	-0.285907	-0.3794	+	C	A	0.764783698	0.0407049	+	C	A	0.768	0.0461
rs4686833	LC_IGP_R34	IgG1_G1FS1/IgG1_G1F	3	186729012	3	8.58E-14	2.40E-18	-	-0.305576	-0.4085	+	C	A	0.764783698	0.040601	+	C	A	0.768	0.0468
rs4686833	LC_IGP_R35	IgG1_G2FS1/IgG1_G2F	3	186729012	3	1.08E-17	8.64E-12	-	-0.3524	-0.3063	+	C	A	0.764909247	0.0406756	+	C	A	0.768	0.0449
rs4686833	LC_IGP122	IgG2 FG1S1/(FG1+FG1S1)	3	186729012	3	3.43E-18	2.03E-23	-	-0.357652	-0.4599	+	C	A	0.765051462	0.0406469	+	C	A	0.768	0.0461
rs4012256	LC_IGP123	IgG2 FG2S1/(FG2+FG2S1)	3	186730341	3	9.37E-13	9.86E-21	-	-0.295441	-0.426	+	C	T	0.766149481	0.0410534	+	C	T	0.7986	0.0456
rs4686833	LC_IGP120	IgG2 FGS1/(FG+FGS1)	3	186729012	3	5.29E-11	6.70E-14	-	-0.271663	-0.3372	+	C	A	0.764908026	0.0411177	+	C	A	0.768	0.045
rs4686833	LC_IGP111	IgG2 SA per Gal	3	186729012	3	4.70E-13	1.24E-20	-	-0.301493	-0.4282	+	C	A	0.765287212	0.041334	+	C	A	0.7675	0.046
rs4686833	LC_IGP93	IgG2_G1FS1	3	186729012	3	1.48E-09	5.35E-16	-	-0.251048	-0.3634	+	C	A	0.765051462	0.0412802	+	C	A	0.768	0.0448
rs4686833	LC_IGP_R74	IgG2_G1FS1/IgG2_G1F	3	186729012	3	3.64E-18	2.27E-23	-	-0.357399	-0.4594	+	C	A	0.765051462	0.04065	+	C	A	0.768	0.0461
rs4686833	LC_IGP_R75	IgG2_G2FS1/IgG2_G2F	3	186729012	3	7.05E-13	7.50E-20	-	-0.296506	-0.4191	+	C	A	0.764746398	0.0409736	+	C	A	0.768	0.046
rs4686833	LC_IGP189	IgG4 FG1S1/(FG1+FG1S1)	3	186729012	3	3.83E-10	1.38E-12	-	-0.258522	-0.3201	+	C	A	0.764891065	0.04104	+	C	A	0.768	0.0452
rs4686833	LC_IGP190	IgG4 FG2S1/(FG2+FG2S1)	3	186729012	3	2.42E-12	4.17E-13	-	-0.290248	-0.326	+	C	A	0.764747619	0.041101	+	C	A	0.768	0.045
rs4686833	LC_IGP186	IgG4 SA per Gal	3	186729012	3	2.84E-10	2.82E-11	-	-0.261526	-0.2976	+	C	A	0.764639747	0.0412055	+	C	A	0.7678	0.0447
rs4686833	LC_IGP179	IgG4_G1FS1	3	186729012	3	2.48E-08	5.91E-11	-	-0.230403	-0.2939	+	C	A	0.764891065	0.0411264	+	C	A	0.768	0.0449
rs4686833	LC_IGP_R92	IgG4_G1FS1/IgG4_G1F	3	186729012	3	4.05E-10	1.39E-12	-	-0.258146	-0.3201	+	C	A	0.764891065	0.0410366	+	C	A	0.768	0.0452
rs4686833	LC_IGP_R93	IgG4_G2FS1/IgG4_G2F	3	186729012	3	2.50E-12	4.20E-13	-	-0.290046	-0.326	+	C	A	0.764747619	0.0411007	+	C	A	0.768	0.045
rs4686834	LC_IGP36	IgG1 FG1S1/(FG1+FG1S1)	3	186729154	3	4.70E-14	4.98E-19	-	-0.30937	-0.4096	+	G	A	0.766178593	0.0406678	+	G	A	0.7986	0.046
rs4686834	LC_IGP37	IgG1 FG2S1/(FG2+FG2S1)	3	186729154	3	5.92E-18	9.26E-12	-	-0.355773	-0.304	+	G	A	0.766301587	0.0407307	+	G	A	0.7986	0.0446
rs4686834	LC_IGP25	IgG1 SA per Gal	3	186729154	3	9.12E-10	2.10E-09	-	-0.255645	-0.263	+	G	A	0.763894424	0.0414922	+	G	A	0.7984	0.0439
rs4686834	LC_IGP7	IgG1_G1FS1	3	186729154	3	2.27E-12	7.37E-18	-	-0.288258	-0.3924	+	G	A	0.766178593	0.0407666	+	G	A	0.7986	0.0456
rs4686834	LC_IGP_R34	IgG1_G1FS1/IgG1_G1F	3	186729154	3	4.24E-14	4.13E-19	-	-0.30982	-0.4113	+	G	A	0.766178593	0.0406533	+	G	A	0.7986	0.046
rs4686834	LC_IGP_R35	IgG1_G2FS1/IgG1_G2F	3	186729154	3	5.62E-18	9.16E-12	-	-0.356012	-0.3041	+	G	A	0.766301587	0.0407293	+	G	A	0.7986	0.0446
rs4686834	LC_IGP122	IgG2 FG1S1/(FG1+FG1S1)	3	186729154	3	3.39E-18	2.55E-25	-	-0.35832	-0.4743	+	G	A	0.766442953	0.0407163	+	G	A	0.7986	0.0456
rs3954170	LC_IGP123	IgG2 FG2S1/(FG2+FG2S1)	3	186730368	3	9.37E-13	9.86E-21	-	-0.295443	-0.426	+	G	A	0.766148871	0.0410535	+	G	A	0.7986	0.0456
rs4686834	LC_IGP120	IgG2 FGS1/(FG+FGS1)	3	186729154	3	7.31E-11	2.04E-14	-	-0.270152	-0.3421	+	G	A	0.766300366	0.0411961	+	G	A	0.7986	0.0447
rs4686834	LC_IGP111	IgG2 SA per Gal	3	186729154	3	5.86E-13	7.54E-22	-	-0.300782	-0.4384	+	G	A	0.766695046	0.0414125	+	G	A	0.7982	0.0456
rs4686834	LC_IGP93	IgG2_G1FS1	3	186729154	3	2.00E-09	1.04E-17	-	-0.249452	-0.3819	+	G	A	0.766442953	0.0413585	+	G	A	0.7986	0.0446
rs4686834	LC_IGP_R74	IgG2_G1FS1/IgG2_G1F	3	186729154	3	3.59E-18	2.83E-25	-	-0.35807	-0.4739	+	G	A	0.766442953	0.0407194	+	G	A	0.7986	0.0456
rs4686834	LC_IGP_R75	IgG2_G2FS1/IgG2_G2F	3	186729154	3	9.35E-13	1.04E-20	-	-0.295433	-0.4254	+	G	A	0.766137889	0.0410506	+	G	A	0.7986	0.0456
rs4686834	LC_IGP189	IgG4 FG1S1/(FG1+FG1S1)	3	186729154	3	2.94E-10	3.31E-13	-	-0.260645	-0.3263	+	G	A	0.766281707	0.0411038	+	G	A	0.7986	0.0448
rs4686834	LC_IGP190	IgG4 FG2S1/(FG2+FG2S1)	3	186729154	3	2.11E-12	1.26E-13	-	-0.291526	-0.3312	+	G	A	0.766139109	0.0411679	+	G	A	0.7986	0.0447
rs4686834	LC_IGP186	IgG4 SA per Gal	3	186729154	3	2.57E-10	1.26E-11	-	-0.262617	-0.3018	+	G	A	0.766035496	0.0412735	+	G	A	0.7983	0.0446
rs4686834	LC_IGP179	IgG4_G1FS1	3	186729154	3	2.89E-08	2.04E-11	-	-0.229697	-0.2992	+	G	A	0.766281707	0.0412006	+	G	A	0.7986	0.0446
rs4686834	LC_IGP_R92	IgG4_G1FS1/IgG4_G1F	3	186729154	3	3.13E-10	3.36E-13	-	-0.260222	-0.3262	+	G	A	0.766281707	0.0411005	+	G	A	0.7986	0.0448
rs4686834	LC_IGP_R93	IgG4_G2FS1/IgG4_G2F	3	186729154	3	2.20E-12	1.27E-13	-	-0.29129	-0.3312	+	G	A	0.766139109	0.0411678	+	G	A	0.7986	0.0447
rs4686835	LC_IGP36	IgG1 FG1S1/(FG1+FG1S1)	3	186729218	3	4.70E-14	4.98E-19	-	-0.309381	-0.4096	+	T	C	0.766175535	0.0406684	+	T	C	0.7986	0.046
rs4686835	LC_IGP37	IgG1 FG2S1/(FG2+FG2S1)	3	186729218	3	5.92E-18	9.27E-12	-	-0.355782	-0.304	+	T	C	0.76629884	0.0407314	+	T	C	0.7986	0.0446
rs4686835	LC_IGP25	IgG1 SA per Gal	3	186729218	3	9.11E-10	2.11E-09	-	-0.255655	-0.263	+	T	C	0.763891291	0.0414929	+	T	C	0.7984	0.0439
rs4686835	LC_IGP7	IgG1_G1FS1	3	186729218	3	2.27E-12	7.39E-18	-	-0.28826	-0.3924	+	T	C	0.766175535	0.0407673	+	T	C	0.7986	0.0456
rs4686835	LC_IGP_R34	IgG1_G1FS1/IgG1_G1F	3	186729218	3	4.24E-14	4.14E-19	-	-0.30983	-0.4113	+	T	C	0.766175535	0.040654	+	T	C	0.7986	0.046
rs4686835	LC_IGP_R35	IgG1_G2FS1/IgG1_G2F	3	186729218	3	5.61E-18	9.17E-12	-	-0.356022	-0.3041	+	T	C	0.76629884	0.04073	+	T	C	0.7986	0.0446
rs4686835	LC_IGP122	IgG2 FG1S1/(FG1+FG1S1)	3	186729218	3	3.40E-18	2.56E-25	-	-0.358318	-0.4743	+	T	C	0.766440207	0.0407171	+	T	C	0.7986	0.0456
rs6444193	LC_IGP123	IgG2 FG2S1/(FG2+FG2S1)	3	186734188	3	7.24E-13	1.01E-20	-	-0.296938	-0.4278	+	A	G	0.764193974	0.0410542	+	A	G	0.7963	0.0458
rs4686835	LC_IGP120	IgG2 FGS1/(FG+FGS1)	3	186729218	3	7.32E-11	2.04E-14	-	-0.270143	-0.3421	+	T	C	0.766297619	0.0411969	+	T	C	0.7986	0.0447
rs4686835	LC_IGP111	IgG2 SA per Gal	3	186729218	3	5.88E-13	7.55E-22	-	-0.300772	-0.4384	+	T	C	0.76669226	0.0414133	+	T	C	0.7982	0.0456
rs4686835	LC_IGP93	IgG2_G1FS1	3	186729218	3	2.01E-09	1.05E-17	-	-0.249451	-0.3819	+	T	C	0.766440207	0.0413592	+	T	C	0.7986	0.0446
rs4686835	LC_IGP_R74	IgG2_G1FS1/IgG2_G1F	3	186729218	3	3.60E-18	2.84E-25	-	-0.358068	-0.4739	+	T	C	0.766440207	0.0407202	+	T	C	0.7986	0.0456
rs4686835	LC_IGP_R75	IgG2_G2FS1/IgG2_G2F	3	186729218	3	9.37E-13	1.04E-20	-	-0.295427	-0.4254	+	T	C	0.766135143	0.0410514	+	T	C	0.7986	0.0456
rs4686835	LC_IGP189	IgG4 FG1S1/(FG1+FG1S1)	3	186729218	3	2.94E-10	3.31E-13	-	-0.260654	-0.3263	+	T	C	0.766278963	0.0411044	+	T	C	0.7986	0.0448
rs4686835	LC_IGP190	IgG4 FG2S1/(FG2+FG2S1)	3	186729218	3	2.11E-12	1.26E-13	-	-0.291536	-0.3312	+	T	C	0.766136364	0.0411686	+	T	C	0.7986	0.0447
rs4686835	LC_IGP186	IgG4 SA per Gal	3	186729218	3	2.57E-10	1.26E-11	-	-0.262622	-0.3018	+	T	C	0.766032742	0.0412742	+	T	C	0.7983	0.0446
rs4686835	LC_IGP179	IgG4_G1FS1	3	186729218	3	2.89E-08	2.05E-11	-	-0.229705	-0.2992	+	T	C	0.766278963	0.0412013	+	T	C	0.7986	0.0446
rs4686835	LC_IGP_R92	IgG4_G1FS1/IgG4_G1F	3	186729218	3	3.13E-10	3.37E-13	-	-0.260231	-0.3262	+	T	C	0.766278963	0.0411012	+	T	C	0.7986	0.0448
rs4686835	LC_IGP_R93	IgG4_G2FS1/IgG4_G2F	3	186729218	3	2.19E-12	1.27E-13	-	-0.2913	-0.3312	+	T	C	0.766136364	0.0411685	+	T	C	0.7986	0.0447
rs4686836	LC_IGP36	IgG1 FG1S1/(FG1+FG1S1)	3	186729365	3	4.62E-14	3.18E-19	-	-0.309487	-0.4119	+	T	C	0.766158716	0.04067	+	T	C	0.7985	0.046
rs4686836	LC_IGP37	IgG1 FG2S1/(FG2+FG2S1)	3	186729365	3	5.75E-18	6.90E-12	-	-0.355934	-0.3059	+	T	C	0.766282051	0.0407327	+	T	C	0.7985	0.0446
rs4686836	LC_IGP25	IgG1 SA per Gal	3	186729365	3	9.02E-10	2.21E-09	-	-0.255734	-0.2626	+	T	C	0.76387406	0.0414948	+	T	C	0.7983	0.0439
rs4686836	LC_IGP7	IgG1_G1FS1	3	186729365	3	2.24E-12	1.71E-18	-	-0.288369	-0.4	+	T	C	0.766158716	0.0407689	+	T	C	0.7985	0.0456
rs4686836	LC_IGP_R34	IgG1_G1FS1/IgG1_G1F	3	186729365	3	4.17E-14	2.65E-19	-	-0.309934	-0.4135	+	T	C	0.766158716	0.0406556	+	T	C	0.7985	0.046
rs4686836	LC_IGP_R35	IgG1_G2FS1/IgG1_G2F	3	186729365	3	5.45E-18	6.84E-12	-	-0.356174	-0.306	+	T	C	0.766282051	0.0407314	+	T	C	0.7985	0.0446
rs4686836	LC_IGP122	IgG2 FG1S1/(FG1+FG1S1)	3	186729365	3	3.34E-18	2.09E-25	-	-0.358419	-0.4752	+	T	C	0.766423429	0.0407187	+	T	C	0.7985	0.0456
rs4396886	LC_IGP123	IgG2 FG2S1/(FG2+FG2S1)	3	186730522	3	9.38E-13	1.01E-20	-	-0.295441	-0.4258	+	C	T	0.766147956	0.0410541	+	C	T	0.7986	0.0456
rs4686836	LC_IGP120	IgG2 FGS1/(FG+FGS1)	3	186729365	3	7.27E-11	2.56E-14	-	-0.270197	-0.3408	+	T	C	0.76628083	0.0411988	+	T	C	0.7985	0.0447
rs4686836	LC_IGP111	IgG2 SA per Gal	3	186729365	3	5.80E-13	6.29E-22	-	-0.300864	-0.4392	+	T	C	0.766675542	0.0414151	+	T	C	0.798	0.0456
rs4686836	LC_IGP93	IgG2_G1FS1	3	186729365	3	1.99E-09	3.92E-18	-	-0.249518	-0.3871	+	T	C	0.766423429	0.0413611	+	T	C	0.7985	0.0446
rs4686836	LC_IGP_R74	IgG2_G1FS1/IgG2_G1F	3	186729365	3	3.53E-18	2.30E-25	-	-0.358169	-0.4748	+	T	C	0.766423429	0.0407218	+	T	C	0.7985	0.0456
rs4686836	LC_IGP_R75	IgG2_G2FS1/IgG2_G2F	3	186729365	3	9.28E-13	8.12E-21	-	-0.295498	-0.4266	+	T	C	0.766118365	0.0410532	+	T	C	0.7985	0.0456
rs4686836	LC_IGP189	IgG4 FG1S1/(FG1+FG1S1)	3	186729365	3	2.93E-10	2.27E-13	-	-0.260679	-0.3285	+	T	C	0.766262195	0.0411065	+	T	C	0.7985	0.0448
rs4686836	LC_IGP190	IgG4 FG2S1/(FG2+FG2S1)	3	186729365	3	2.09E-12	1.71E-13	-	-0.29159	-0.3294	+	T	C	0.766119585	0.0411705	+	T	C	0.7985	0.0447
rs4686836	LC_IGP186	IgG4 SA per Gal	3	186729365	3	2.57E-10	1.72E-11	-	-0.262632	-0.2998	+	T	C	0.766015912	0.0412763	+	T	C	0.7982	0.0446
rs4686836	LC_IGP179	IgG4_G1FS1	3	186729365	3	2.86E-08	8.18E-12	-	-0.229773	-0.3052	+	T	C	0.766262195	0.0412032	+	T	C	0.7985	0.0446
rs4686836	LC_IGP_R92	IgG4_G1FS1/IgG4_G1F	3	186729365	3	3.12E-10	2.30E-13	-	-0.260256	-0.3285	+	T	C	0.766262195	0.0411032	+	T	C	0.7985	0.0448
rs4686836	LC_IGP_R93	IgG4_G2FS1/IgG4_G2F	3	186729365	3	2.18E-12	1.72E-13	-	-0.291353	-0.3294	+	T	C	0.766119585	0.0411704	+	T	C	0.7985	0.0447
rs4012256	LC_IGP36	IgG1 FG1S1/(FG1+FG1S1)	3	186730341	3	4.66E-14	4.80E-19	-	-0.309445	-0.4102	+	C	T	0.766190214	0.0406714	+	C	T	0.7986	0.046
rs4012256	LC_IGP37	IgG1 FG2S1/(FG2+FG2S1)	3	186730341	3	5.86E-18	8.79E-12	-	-0.355855	-0.3047	+	C	T	0.766313492	0.0407344	+	C	T	0.7986	0.0446
rs4012256	LC_IGP25	IgG1 SA per Gal	3	186730341	3	9.03E-10	2.01E-09	-	-0.255735	-0.2636	+	C	T	0.763906328	0.041496	+	C	T	0.7984	0.044
rs4012256	LC_IGP7	IgG1_G1FS1	3	186730341	3	2.25E-12	6.39E-18	-	-0.288336	-0.3935	+	C	T	0.766190214	0.0407702	+	C	T	0.7986	0.0456
rs4012256	LC_IGP_R34	IgG1_G1FS1/IgG1_G1F	3	186730341	3	4.20E-14	3.98E-19	-	-0.309896	-0.4119	+	C	T	0.766190214	0.040657	+	C	T	0.7986	0.0461
rs4012256	LC_IGP_R35	IgG1_G2FS1/IgG1_G2F	3	186730341	3	5.56E-18	8.70E-12	-	-0.356094	-0.3047	+	C	T	0.766313492	0.040733	+	C	T	0.7986	0.0446
rs4012256	LC_IGP122	IgG2 FG1S1/(FG1+FG1S1)	3	186730341	3	3.38E-18	2.52E-25	-	-0.358371	-0.4748	+	C	T	0.766454545	0.0407202	+	C	T	0.7986	0.0457
rs4686835	LC_IGP123	IgG2 FG2S1/(FG2+FG2S1)	3	186729218	3	9.52E-13	1.02E-20	-	-0.295327	-0.4254	+	T	C	0.766135143	0.0410506	+	T	C	0.7986	0.0456
rs4012256	LC_IGP120	IgG2 FGS1/(FG+FGS1)	3	186730341	3	7.22E-11	2.01E-14	-	-0.270253	-0.3425	+	C	T	0.766311966	0.0411998	+	C	T	0.7986	0.0448
rs4012256	LC_IGP111	IgG2 SA per Gal	3	186730341	3	5.84E-13	7.43E-22	-	-0.300832	-0.4388	+	C	T	0.766706192	0.0414163	+	C	T	0.7982	0.0457
rs4012256	LC_IGP93	IgG2_G1FS1	3	186730341	3	1.99E-09	9.80E-18	-	-0.24952	-0.3825	+	C	T	0.766454545	0.0413623	+	C	T	0.7986	0.0446
rs4012256	LC_IGP_R74	IgG2_G1FS1/IgG2_G1F	3	186730341	3	3.58E-18	2.80E-25	-	-0.358121	-0.4744	+	C	T	0.766454545	0.0407233	+	C	T	0.7986	0.0457
rs4012256	LC_IGP_R75	IgG2_G2FS1/IgG2_G2F	3	186730341	3	9.22E-13	1.00E-20	-	-0.29554	-0.4259	+	C	T	0.766149481	0.0410542	+	C	T	0.7986	0.0456
rs4012256	LC_IGP189	IgG4 FG1S1/(FG1+FG1S1)	3	186730341	3	2.89E-10	3.03E-13	-	-0.260781	-0.3271	+	C	T	0.766293598	0.0411073	+	C	T	0.7986	0.0449
rs4012256	LC_IGP190	IgG4 FG2S1/(FG2+FG2S1)	3	186730341	3	2.07E-12	1.19E-13	-	-0.291662	-0.3319	+	C	T	0.766151007	0.0411714	+	C	T	0.7986	0.0447
rs4012256	LC_IGP186	IgG4 SA per Gal	3	186730341	3	2.52E-10	1.19E-11	-	-0.262764	-0.3025	+	C	T	0.76604743	0.041277	+	C	T	0.7983	0.0446
rs4012256	LC_IGP179	IgG4_G1FS1	3	186730341	3	2.84E-08	1.82E-11	-	-0.229832	-0.3002	+	C	T	0.766293598	0.0412041	+	C	T	0.7986	0.0447
rs4012256	LC_IGP_R92	IgG4_G1FS1/IgG4_G1F	3	186730341	3	3.07E-10	3.08E-13	-	-0.260361	-0.3271	+	C	T	0.766293598	0.041104	+	C	T	0.7986	0.0449
rs4012256	LC_IGP_R93	IgG4_G2FS1/IgG4_G2F	3	186730341	3	2.16E-12	1.20E-13	-	-0.291425	-0.3319	+	C	T	0.766151007	0.0411713	+	C	T	0.7986	0.0448
rs3954170	LC_IGP36	IgG1 FG1S1/(FG1+FG1S1)	3	186730368	3	4.66E-14	4.80E-19	-	-0.309443	-0.4102	+	G	A	0.766189602	0.0406715	+	G	A	0.7986	0.046
rs3954170	LC_IGP37	IgG1 FG2S1/(FG2+FG2S1)	3	186730368	3	5.86E-18	8.79E-12	-	-0.355853	-0.3047	+	G	A	0.766312882	0.0407345	+	G	A	0.7986	0.0446
rs3954170	LC_IGP25	IgG1 SA per Gal	3	186730368	3	9.03E-10	2.01E-09	-	-0.255732	-0.2636	+	G	A	0.763905702	0.041496	+	G	A	0.7984	0.044
rs3954170	LC_IGP7	IgG1_G1FS1	3	186730368	3	2.25E-12	6.39E-18	-	-0.288336	-0.3935	+	G	A	0.766189602	0.0407703	+	G	A	0.7986	0.0456
rs3954170	LC_IGP_R34	IgG1_G1FS1/IgG1_G1F	3	186730368	3	4.21E-14	3.98E-19	-	-0.309894	-0.4119	+	G	A	0.766189602	0.040657	+	G	A	0.7986	0.0461
rs3954170	LC_IGP_R35	IgG1_G2FS1/IgG1_G2F	3	186730368	3	5.56E-18	8.70E-12	-	-0.356092	-0.3047	+	G	A	0.766312882	0.0407331	+	G	A	0.7986	0.0446
rs3954170	LC_IGP122	IgG2 FG1S1/(FG1+FG1S1)	3	186730368	3	3.38E-18	2.52E-25	-	-0.358369	-0.4748	+	G	A	0.766453935	0.0407203	+	G	A	0.7986	0.0457
rs4686834	LC_IGP123	IgG2 FG2S1/(FG2+FG2S1)	3	186729154	3	9.50E-13	1.02E-20	-	-0.295334	-0.4254	+	G	A	0.766137889	0.0410498	+	G	A	0.7986	0.0456
rs3954170	LC_IGP120	IgG2 FGS1/(FG+FGS1)	3	186730368	3	7.22E-11	2.01E-14	-	-0.270252	-0.3425	+	G	A	0.766311355	0.0411998	+	G	A	0.7986	0.0448
rs3954170	LC_IGP111	IgG2 SA per Gal	3	186730368	3	5.84E-13	7.43E-22	-	-0.300833	-0.4388	+	G	A	0.766705882	0.0414164	+	G	A	0.7982	0.0457
rs3954170	LC_IGP93	IgG2_G1FS1	3	186730368	3	1.99E-09	9.80E-18	-	-0.249518	-0.3825	+	G	A	0.766453935	0.0413624	+	G	A	0.7986	0.0446
rs3954170	LC_IGP_R74	IgG2_G1FS1/IgG2_G1F	3	186730368	3	3.58E-18	2.80E-25	-	-0.358119	-0.4744	+	G	A	0.766453935	0.0407233	+	G	A	0.7986	0.0457
rs3954170	LC_IGP_R75	IgG2_G2FS1/IgG2_G2F	3	186730368	3	9.22E-13	1.00E-20	-	-0.295542	-0.4259	+	G	A	0.766148871	0.0410543	+	G	A	0.7986	0.0456
rs3954170	LC_IGP189	IgG4 FG1S1/(FG1+FG1S1)	3	186730368	3	2.89E-10	3.03E-13	-	-0.260779	-0.3271	+	G	A	0.766292988	0.0411073	+	G	A	0.7986	0.0449
rs3954170	LC_IGP190	IgG4 FG2S1/(FG2+FG2S1)	3	186730368	3	2.07E-12	1.19E-13	-	-0.291661	-0.3319	+	G	A	0.766150397	0.0411715	+	G	A	0.7986	0.0447
rs3954170	LC_IGP186	IgG4 SA per Gal	3	186730368	3	2.52E-10	1.19E-11	-	-0.262762	-0.3025	+	G	A	0.766046818	0.0412771	+	G	A	0.7983	0.0446
rs3954170	LC_IGP179	IgG4_G1FS1	3	186730368	3	2.84E-08	1.82E-11	-	-0.229832	-0.3002	+	G	A	0.766292988	0.0412042	+	G	A	0.7986	0.0447
rs3954170	LC_IGP_R92	IgG4_G1FS1/IgG4_G1F	3	186730368	3	3.08E-10	3.08E-13	-	-0.260359	-0.3271	+	G	A	0.766292988	0.0411041	+	G	A	0.7986	0.0449
rs3954170	LC_IGP_R93	IgG4_G2FS1/IgG4_G2F	3	186730368	3	2.16E-12	1.20E-13	-	-0.291424	-0.3319	+	G	A	0.766150397	0.0411713	+	G	A	0.7986	0.0448

rs4012257	LC_IGP36	IgG1 FG1S1/(FG1+FG1S1)	3	186730396	3	4.66E-14	4.80E-19	-	-0.309442	-0.4102	+	C	A	0.766189343	0.0406715	+	C	A	0.7986	0.046
rs4012257	LC_IGP37	IgG1 FG2S1/(FG2+FG2S1)	3	186730396	3	5.86E-18	8.79E-12	-	-0.355856	-0.3047	+	C	A	0.766312623	0.0407345	+	C	A	0.7986	0.0446
rs4012257	LC_IGP25	IgG1 SA per Gal	3	186730396	3	9.03E-10	2.01E-09	-	-0.255735	-0.2636	+	C	A	0.763905436	0.0414961	+	C	A	0.7984	0.044
rs4012257	LC_IGP7	IgG1_G1FS1	3	186730396	3	2.25E-12	6.38E-18	-	-0.288336	-0.3935	+	C	A	0.766189343	0.0407703	+	C	A	0.7986	0.0456
rs4012257	LC_IGP_R34	IgG1_G1FS1/IgG1_G1F	3	186730396	3	4.21E-14	3.98E-19	-	-0.309893	-0.4119	+	C	A	0.766189343	0.0406571	+	C	A	0.7986	0.0461
rs4012257	LC_IGP_R35	IgG1_G2FS1/IgG1_G2F	3	186730396	3	5.56E-18	8.70E-12	-	-0.356095	-0.3047	+	C	A	0.766312623	0.0407331	+	C	A	0.7986	0.0446
rs4012257	LC_IGP122	IgG2 FG1S1/(FG1+FG1S1)	3	186730396	3	3.38E-18	2.52E-25	-	-0.358367	-0.4748	+	C	A	0.766453677	0.0407203	+	C	A	0.7986	0.0457
rs10804908	LC_IGP123	IgG2 FG2S1/(FG2+FG2S1)	3	186728842	3	8.88E-13	1.02E-20	-	-0.295584	-0.4255	+	A	T	0.76593014	0.0410299	+	A	T	0.7985	0.0456
rs4012257	LC_IGP120	IgG2 FGS1/(FG+FGS1)	3	186730396	3	7.22E-11	2.01E-14	-	-0.27025	-0.3425	+	C	A	0.766311097	0.0411999	+	C	A	0.7986	0.0448
rs4012257	LC_IGP111	IgG2 SA per Gal	3	186730396	3	5.84E-13	7.43E-22	-	-0.30083	-0.4388	+	C	A	0.76670562	0.0414164	+	C	A	0.7982	0.0457
rs4012257	LC_IGP93	IgG2_G1FS1	3	186730396	3	1.99E-09	9.79E-18	-	-0.249517	-0.3825	+	C	A	0.766453677	0.0413624	+	C	A	0.7986	0.0446
rs4012257	LC_IGP_R74	IgG2_G1FS1/IgG2_G1F	3	186730396	3	3.58E-18	2.80E-25	-	-0.358117	-0.4744	+	C	A	0.766453677	0.0407234	+	C	A	0.7986	0.0457
rs4012257	LC_IGP_R75	IgG2_G2FS1/IgG2_G2F	3	186730396	3	9.23E-13	1.00E-20	-	-0.295537	-0.4259	+	C	A	0.766148613	0.0410543	+	C	A	0.7986	0.0456
rs4012257	LC_IGP189	IgG4 FG1S1/(FG1+FG1S1)	3	186730396	3	2.89E-10	3.03E-13	-	-0.260779	-0.3271	+	C	A	0.76629273	0.0411074	+	C	A	0.7986	0.0449
rs4012257	LC_IGP190	IgG4 FG2S1/(FG2+FG2S1)	3	186730396	3	2.07E-12	1.19E-13	-	-0.291663	-0.3319	+	C	A	0.766150138	0.0411715	+	C	A	0.7986	0.0447
rs4012257	LC_IGP186	IgG4 SA per Gal	3	186730396	3	2.52E-10	1.19E-11	-	-0.262763	-0.3025	+	C	A	0.766046559	0.0412771	+	C	A	0.7983	0.0446
rs4012257	LC_IGP179	IgG4_G1FS1	3	186730396	3	2.84E-08	1.81E-11	-	-0.229832	-0.3003	+	C	A	0.76629273	0.0412042	+	C	A	0.7986	0.0447
rs4012257	LC_IGP_R92	IgG4_G1FS1/IgG4_G1F	3	186730396	3	3.08E-10	3.08E-13	-	-0.260359	-0.3271	+	C	A	0.76629273	0.0411041	+	C	A	0.7986	0.0449
rs4012257	LC_IGP_R93	IgG4_G2FS1/IgG4_G2F	3	186730396	3	2.16E-12	1.20E-13	-	-0.291426	-0.3319	+	C	A	0.766150138	0.0411713	+	C	A	0.7986	0.0448
rs4396886	LC_IGP36	IgG1 FG1S1/(FG1+FG1S1)	3	186730522	3	4.66E-14	4.79E-19	-	-0.309449	-0.4103	+	C	T	0.766188685	0.0406721	+	C	T	0.7986	0.046
rs4396886	LC_IGP37	IgG1 FG2S1/(FG2+FG2S1)	3	186730522	3	5.85E-18	9.06E-12	-	-0.355864	-0.3045	+	C	T	0.766311966	0.040735	+	C	T	0.7986	0.0446
rs4396886	LC_IGP25	IgG1 SA per Gal	3	186730522	3	9.03E-10	2.05E-09	-	-0.255739	-0.2635	+	C	T	0.763904762	0.0414966	+	C	T	0.7983	0.044
rs4396886	LC_IGP7	IgG1_G1FS1	3	186730522	3	2.25E-12	6.40E-18	-	-0.288347	-0.3935	+	C	T	0.766188685	0.0407708	+	C	T	0.7986	0.0456
rs4396886	LC_IGP_R34	IgG1_G1FS1/IgG1_G1F	3	186730522	3	4.20E-14	3.97E-19	-	-0.3099	-0.4119	+	C	T	0.766188685	0.0406576	+	C	T	0.7986	0.0461
rs4396886	LC_IGP_R35	IgG1_G2FS1/IgG1_G2F	3	186730522	3	5.56E-18	8.96E-12	-	-0.356103	-0.3045	+	C	T	0.766311966	0.0407336	+	C	T	0.7986	0.0446
rs4396886	LC_IGP122	IgG2 FG1S1/(FG1+FG1S1)	3	186730522	3	3.38E-18	2.56E-25	-	-0.35837	-0.4748	+	C	T	0.76645302	0.0407208	+	C	T	0.7986	0.0457
rs10937279	LC_IGP123	IgG2 FG2S1/(FG2+FG2S1)	3	186727836	3	1.00E-12	1.37E-20	-	-0.295585	-0.4243	+	G	A	0.7686687	0.0411291	+	G	A	0.7981	0.0456
rs4396886	LC_IGP120	IgG2 FGS1/(FG+FGS1)	3	186730522	3	7.23E-11	2.05E-14	-	-0.270249	-0.3424	+	C	T	0.76631044	0.0412004	+	C	T	0.7986	0.0448
rs4396886	LC_IGP111	IgG2 SA per Gal	3	186730522	3	5.84E-13	7.68E-22	-	-0.300835	-0.4387	+	C	T	0.766704954	0.041417	+	C	T	0.7981	0.0457
rs4396886	LC_IGP93	IgG2_G1FS1	3	186730522	3	1.99E-09	9.87E-18	-	-0.249519	-0.3825	+	C	T	0.76645302	0.041363	+	C	T	0.7986	0.0446
rs4396886	LC_IGP_R74	IgG2_G1FS1/IgG2_G1F	3	186730522	3	3.59E-18	2.84E-25	-	-0.358119	-0.4743	+	C	T	0.76645302	0.0407239	+	C	T	0.7986	0.0457
rs4396886	LC_IGP_R75	IgG2_G2FS1/IgG2_G2F	3	186730522	3	9.23E-13	1.03E-20	-	-0.295541	-0.4258	+	C	T	0.766147956	0.0410549	+	C	T	0.7986	0.0456
rs4396886	LC_IGP189	IgG4 FG1S1/(FG1+FG1S1)	3	186730522	3	2.89E-10	3.06E-13	-	-0.260784	-0.3271	+	C	T	0.766292073	0.0411079	+	C	T	0.7986	0.0449
rs4396886	LC_IGP190	IgG4 FG2S1/(FG2+FG2S1)	3	186730522	3	2.07E-12	1.22E-13	-	-0.291667	-0.3317	+	C	T	0.766149481	0.041172	+	C	T	0.7986	0.0447
rs4396886	LC_IGP186	IgG4 SA per Gal	3	186730522	3	2.52E-10	1.21E-11	-	-0.262767	-0.3024	+	C	T	0.7660459	0.0412777	+	C	T	0.7983	0.0446
rs4396886	LC_IGP179	IgG4_G1FS1	3	186730522	3	2.84E-08	1.82E-11	-	-0.229836	-0.3002	+	C	T	0.766292073	0.0412048	+	C	T	0.7986	0.0447
rs4396886	LC_IGP_R92	IgG4_G1FS1/IgG4_G1F	3	186730522	3	3.08E-10	3.11E-13	-	-0.260364	-0.327	+	C	T	0.766292073	0.0411047	+	C	T	0.7986	0.0449
rs4396886	LC_IGP_R93	IgG4_G2FS1/IgG4_G2F	3	186730522	3	2.16E-12	1.23E-13	-	-0.29143	-0.3317	+	C	T	0.766149481	0.0411719	+	C	T	0.7986	0.0447
rs10212190	LC_IGP36	IgG1 FG1S1/(FG1+FG1S1)	3	186731157	3	4.02E-24	5.91E-43	-	-0.369883	-0.5245	+	T	A	0.361797859	0.0359321	+	T	A	0.396	0.0382
rs10212190	LC_IGP37	IgG1 FG2S1/(FG2+FG2S1)	3	186731157	3	1.52E-26	3.23E-26	-	-0.390891	-0.3925	+	T	A	0.361757631	0.0360185	+	T	A	0.396	0.0371
rs10212190	LC_IGP34	IgG1 FGS1/(FG+FGS1)	3	186731157	3	1.29E-09	1.26E-10	-	-0.225233	-0.2316	+	T	A	0.361797859	0.0368975	+	T	A	0.396	0.036
rs10212190	LC_IGP25	IgG1 SA per Gal	3	186731157	3	1.05E-14	7.76E-19	-	-0.288702	-0.3237	+	T	A	0.359552005	0.0369758	+	T	A	0.3962	0.0365
rs10212190	LC_IGP7	IgG1_G1FS1	3	186731157	3	4.00E-21	1.85E-34	-	-0.345133	-0.4641	+	T	A	0.361797859	0.0360861	+	T	A	0.396	0.0379
rs10212190	LC_IGP_R34	IgG1_G1FS1/IgG1_G1F	3	186731157	3	5.05E-24	9.01E-43	-	-0.368984	-0.5242	+	T	A	0.361797859	0.0359265	+	T	A	0.396	0.0382
rs10212190	LC_IGP_R35	IgG1_G2FS1/IgG1_G2F	3	186731157	3	1.41E-26	3.13E-26	-	-0.391143	-0.3926	+	T	A	0.361757631	0.0360167	+	T	A	0.396	0.0371
rs10212190	LC_IGP122	IgG2 FG1S1/(FG1+FG1S1)	3	186731157	3	1.70E-35	2.03E-49	-	-0.452945	-0.5606	+	T	A	0.361540574	0.0355641	+	T	A	0.396	0.0379
rs4686837	LC_IGP123	IgG2 FG2S1/(FG2+FG2S1)	3	186739677	3	5.42E-10	1.68E-20	-	-0.248067	-0.3678	+	A	G	0.299521965	0.039728	+	A	G	0.3189	0.0396
rs10212190	LC_IGP120	IgG2 FGS1/(FG+FGS1)	3	186731157	3	6.92E-21	3.23E-33	-	-0.345552	-0.4467	+	T	A	0.361761294	0.0363595	+	T	A	0.396	0.0372
rs10212190	LC_IGP111	IgG2 SA per Gal	3	186731157	3	7.21E-26	5.36E-43	-	-0.38816	-0.5217	+	T	A	0.361869969	0.036271	+	T	A	0.3958	0.038
rs10212190	LC_IGP93	IgG2_G1FS1	3	186731157	3	3.38E-17	6.71E-32	-	-0.312161	-0.4357	+	T	A	0.361540574	0.036612	+	T	A	0.396	0.0371
rs10212190	LC_IGP_R74	IgG2_G1FS1/IgG2_G1F	3	186731157	3	1.66E-35	2.02E-49	-	-0.453018	-0.5606	+	T	A	0.361540574	0.0355652	+	T	A	0.396	0.0379
rs10212190	LC_IGP_R75	IgG2_G2FS1/IgG2_G2F	3	186731157	3	1.55E-21	2.27E-44	-	-0.350924	-0.5297	+	T	A	0.361832825	0.0363008	+	T	A	0.396	0.0379
rs10212190	LC_IGP189	IgG4 FG1S1/(FG1+FG1S1)	3	186731157	3	1.03E-21	3.08E-36	-	-0.351616	-0.4683	+	T	A	0.361608537	0.0362083	+	T	A	0.396	0.0372
rs10212190	LC_IGP190	IgG4 FG2S1/(FG2+FG2S1)	3	186731157	3	5.36E-21	2.77E-34	-	-0.347077	-0.4538	+	T	A	0.361817572	0.0364131	+	T	A	0.396	0.0372
rs10212190	LC_IGP187	IgG4 FGS1/(FG+FGS1)	3	186731157	3	5.01E-14	2.30E-16	-	-0.278865	-0.2967	+	T	A	0.361817572	0.0367004	+	T	A	0.396	0.0362
rs10212190	LC_IGP186	IgG4 SA per Gal	3	186731157	3	8.04E-21	9.51E-28	-	-0.345857	-0.405	+	T	A	0.362007344	0.0364534	+	T	A	0.3954	0.0371
rs10212190	LC_IGP179	IgG4_G1FS1	3	186731157	3	6.10E-16	2.73E-26	-	-0.298089	-0.3937	+	T	A	0.361608537	0.0364861	+	T	A	0.396	0.0371
rs10212190	LC_IGP_R92	IgG4_G1FS1/IgG4_G1F	3	186731157	3	8.73E-22	3.41E-36	-	-0.352182	-0.468	+	T	A	0.361608537	0.0362006	+	T	A	0.396	0.0373
rs10212190	LC_IGP_R93	IgG4_G2FS1/IgG4_G2F	3	186731157	3	5.45E-21	2.96E-34	-	-0.347007	-0.4536	+	T	A	0.361817572	0.0364124	+	T	A	0.396	0.0372
rs56291008	LC_IGP122	IgG2 FG1S1/(FG1+FG1S1)	3	186731458	3	2.40E-08	1.51E-08	-	-0.487195	-0.4875	+	T	C	0.05108034	0.08688	+	T	C	0.0564	0.0861
rs56291008	LC_IGP_R74	IgG2_G1FS1/IgG2_G1F	3	186731458	3	2.35E-08	1.51E-08	-	-0.487565	-0.4874	+	T	C	0.05108034	0.0868824	+	T	C	0.0564	0.0861
rs7619989	LC_IGP36	IgG1 FG1S1/(FG1+FG1S1)	3	186731749	3	3.80E-18	2.08E-25	-	-0.308245	-0.4049	+	C	G	0.602489297	0.0350782	+	C	G	0.6482	0.0389
rs7619989	LC_IGP37	IgG1 FG2S1/(FG2+FG2S1)	3	186731749	3	5.40E-22	5.13E-16	-	-0.343239	-0.3056	+	C	G	0.602607143	0.0350946	+	C	G	0.6482	0.0377
rs7619989	LC_IGP34	IgG1 FGS1/(FG+FGS1)	3	186731749	3	2.29E-09	1.53E-07	-	-0.21475	-0.1922	+	C	G	0.602489297	0.035734	+	C	G	0.6482	0.0366
rs7619989	LC_IGP29	IgG1 G2S1/(G2+G2S1)	3	186731749	3	1.40E-08	2.06E-08	-	-0.204816	-0.2136	+	C	G	0.602121638	0.0359197	+	C	G	0.6482	0.0381
rs7619989	LC_IGP25	IgG1 SA per Gal	3	186731749	3	1.31E-12	8.28E-13	-	-0.256778	-0.2656	+	C	G	0.601467732	0.0359092	+	C	G	0.6484	0.0371
rs7619989	LC_IGP7	IgG1_G1FS1	3	186731749	3	6.87E-15	1.36E-22	-	-0.277025	-0.377	+	C	G	0.602489297	0.0352407	+	C	G	0.6482	0.0385
rs7619989	LC_IGP_R34	IgG1_G1FS1/IgG1_G1F	3	186731749	3	3.48E-18	1.71E-25	-	-0.308492	-0.4063	+	C	G	0.602489297	0.0350661	+	C	G	0.6482	0.0389
rs7619989	LC_IGP_R35	IgG1_G2FS1/IgG1_G2F	3	186731749	3	6.50E-22	5.07E-16	-	-0.34258	-0.3057	+	C	G	0.602607143	0.0350984	+	C	G	0.6482	0.0377
rs7619989	LC_IGP_R33	IgG1_G2S1/IgG1_G2	3	186731749	3	1.61E-08	2.11E-08	-	-0.203998	-0.2135	+	C	G	0.602121638	0.0359258	+	C	G	0.6482	0.0381
rs7619989	LC_IGP122	IgG2 FG1S1/(FG1+FG1S1)	3	186731749	3	1.06E-20	2.75E-34	-	-0.332552	-0.4711	+	C	G	0.602530506	0.0351649	+	C	G	0.6482	0.0386
rs12633034	LC_IGP123	IgG2 FG2S1/(FG2+FG2S1)	3	186727597	3	9.49E-13	2.15E-20	-	-0.295875	-0.4252	+	G	C	0.768607383	0.0411238	+	G	C	0.7886	0.046
rs7619989	LC_IGP120	IgG2 FGS1/(FG+FGS1)	3	186731749	3	1.35E-12	2.35E-21	-	-0.25445	-0.3589	+	C	G	0.602593101	0.0356124	+	C	G	0.6482	0.0378
rs7619989	LC_IGP111	IgG2 SA per Gal	3	186731749	3	2.03E-15	3.84E-29	-	-0.285866	-0.4325	+	C	G	0.601984211	0.035648	+	C	G	0.6473	0.0386
rs7619989	LC_IGP93	IgG2_G1FS1	3	186731749	3	1.29E-10	7.04E-25	-	-0.231544	-0.3882	+	C	G	0.602530506	0.0357855	+	C	G	0.6482	0.0377
rs7619989	LC_IGP_R74	IgG2_G1FS1/IgG2_G1F	3	186731749	3	1.21E-20	2.95E-34	-	-0.332087	-0.4709	+	C	G	0.602530506	0.0351691	+	C	G	0.6482	0.0386
rs7619989	LC_IGP_R75	IgG2_G2FS1/IgG2_G2F	3	186731749	3	2.93E-14	1.42E-27	-	-0.272328	-0.4193	+	C	G	0.602530506	0.0355054	+	C	G	0.6482	0.0385
rs7619989	LC_IGP189	IgG4 FG1S1/(FG1+FG1S1)	3	186731749	3	6.93E-14	5.49E-22	-	-0.267832	-0.3651	+	C	G	0.602484756	0.0354516	+	C	G	0.6482	0.0379
rs7619989	LC_IGP190	IgG4 FG2S1/(FG2+FG2S1)	3	186731749	3	1.03E-16	1.52E-18	-	-0.297726	-0.332	+	C	G	0.602242221	0.0354804	+	C	G	0.6482	0.0378
rs7619989	LC_IGP187	IgG4 FGS1/(FG+FGS1)	3	186731749	3	1.13E-10	4.23E-10	-	-0.231786	-0.2295	+	C	G	0.602242221	0.0357132	+	C	G	0.6482	0.0367
rs7619989	LC_IGP186	IgG4 SA per Gal	3	186731749	3	3.29E-14	3.17E-17	-	-0.272312	-0.3179	+	C	G	0.6022347	0.0355725	+	C	G	0.6477	0.0377
rs7619989	LC_IGP179	IgG4_G1FS1	3	186731749	3	2.88E-09	4.12E-19	-	-0.212963	-0.337	+	C	G	0.602484756	0.0356644	+	C	G	0.6482	0.0377
rs7619989	LC_IGP_R92	IgG4_G1FS1/IgG4_G1F	3	186731749	3	7.48E-14	5.20E-22	-	-0.267453	-0.3653	+	C	G	0.602484756	0.0354491	+	C	G	0.6482	0.0379
rs7619989	LC_IGP_R93	IgG4_G2FS1/IgG4_G2F	3	186731749	3	1.07E-16	1.53E-18	-	-0.297555	-0.332	+	C	G	0.602242221	0.0354803	+	C	G	0.6482	0.0378
rs17776120	LC_IGP36	IgG1 FG1S1/(FG1+FG1S1)	3	186732679	3	5.28E-23	1.81E-41	-	-0.360547	-0.5128	+	A	C	0.363804893	0.0359576	+	A	C	0.3992	0.038
rs17776120	LC_IGP37	IgG1 FG2S1/(FG2+FG2S1)	3	186732679	3	5.41E-26	2.65E-25	-	-0.386344	-0.3834	+	A	C	0.363749389	0.0360142	+	A	C	0.3992	0.0369
rs17776120	LC_IGP34	IgG1 FGS1/(FG+FGS1)	3	186732679	3	2.35E-09	3.04E-10	-	-0.221461	-0.2256	+	A	C	0.363804893	0.0368794	+	A	C	0.3992	0.0358
rs17776120	LC_IGP25	IgG1 SA per Gal	3	186732679	3	2.65E-14	2.71E-18	-	-0.284065	-0.3169	+	A	C	0.361620614	0.0369614	+	A	C	0.3994	0.0363
rs17776120	LC_IGP7	IgG1_G1FS1	3	186732679	3	6.65E-21	1.48E-33	-	-0.342932	-0.4552	+	A	C	0.363804893	0.0360665	+	A	C	0.3992	0.0377
rs17776120	LC_IGP_R34	IgG1_G1FS1/IgG1_G1F	3	186732679	3	6.30E-23	2.65E-41	-	-0.359817	-0.5126	+	A	C	0.363804893	0.0359509	+	A	C	0.3992	0.0381
rs17776120	LC_IGP_R35	IgG1_G2FS1/IgG1_G2F	3	186732679	3	4.85E-26	2.57E-25	-	-0.386706	-0.3836	+	A	C	0.363749389	0.0360118	+	A	C	0.3992	0.0369
rs17776120	LC_IGP122	IgG2 FG1S1/(FG1+FG1S1)	3	186732679	3	4.39E-34	1.89E-48	-	-0.443414	-0.552	+	A	C	0.363533335	0.0356026	+	A	C	0.3992	0.0377
rs7622548	LC_IGP123	IgG2 FG2S1/(FG2+FG2S1)	3	186738118	3	2.05E-12	2.56E-20	-	-0.293289	-0.4273	+	C	G	0.763952596	0.0413942	+	C	G	0.7968	0.0463
rs17776120	LC_IGP120	IgG2 FGS1/(FG+FGS1)	3	186732679	3	2.34E-20	1.24E-32	-	-0.340609	-0.4402	+	A	C	0.363755272	0.0363539	+	A	C	0.3992	0.037
rs17776120	LC_IGP111	IgG2 SA per Gal	3	186732679	3	6.98E-25	3.04E-42	-	-0.380121	-0.5142	+	A	C	0.363890402	0.0362906	+	A	C	0.399	0.0378
rs17776120	LC_IGP93	IgG2_G1FS1	3	186732679	3	1.03E-16	1.85E-31	-	-0.307153	-0.4304	+	A	C	0.363533335	0.0366038	+	A	C	0.3992	0.0369
rs17776120	LC_IGP_R74	IgG2_G1FS1/IgG2_G1F	3	186732679	3	4.31E-34	1.89E-48	-	-0.443483	-0.552	+	A	C	0.363533335	0.0356036	+	A	C	0.3992	0.0377
rs17776120	LC_IGP_R75	IgG2_G2FS1/IgG2_G2F	3	186732679	3	7.11E-21	1.67E-43	-	-0.344781	-0.5215	+	A	C	0.363533335	0.03629	+	A	C	0.3992	0.0377
rs17776120	LC_IGP189	IgG4 FG1S1/(FG1+FG1S1)	3	186732679	3	1.48E-20	1.76E-35	-	-0.3412	-0.4608	+	A	C	0.363305793	0.0362226	+	A	C	0.3992	0.0371
rs17776120	LC_IGP190	IgG4 FG2S1/(FG2+FG2S1)	3	186732679	3	6.70E-20	1.26E-33	-	-0.33699	-0.4468	+	A	C	0.363527456	0.0364257	+	A	C	0.3992	0.037
rs17776120	LC_IGP187	IgG4 FGS1/(FG+FGS1)	3	186732679	3	1.45E-13	4.21E-16	-	-0.273447	-0.2923	+	A	C	0.363527456	0.0366806	+	A	C	0.3992	0.036
rs17776120	LC_IGP186	IgG4 SA per Gal	3	186732679	3	5.67E-20	3.14E-27	-	-0.337943	-0.3986	+	A	C	0.363722766	0.0364534	+	A	C	0.3987	0.0369
rs17776120	LC_IGP179	IgG4_G1FS1	3	186732679	3	6.97E-16	4.47E-26	-	-0.297144	-0.3901	+	A	C	0.363305793	0.0364446	+	A	C	0.3992	0.0369
rs17776120	LC_IGP_R92	IgG4_G1FS1/IgG4_G1F	3	186732679	3	1.18E-20	1.93E-35	-	-0.342039	-0.4606	+	A	C	0.363305793	0.0362133	+	A	C	0.3992	0.0371
rs17776120	LC_IGP_R93	IgG4_G2FS1/IgG4_G2F	3	186732679	3	6.78E-20	1.35E-33	-	-0.336934	-0.4466	+	A	C	0.363527456	0.0364249	+	A	C	0.3992	0.037
rs3821819	LC_IGP36	IgG1 FG1S1/(FG1+FG1S1)	3	186732725	3	5.34E-23	1.65E-41	-	-0.360485	-0.5136	+	A	G	0.363827523	0.0359556	+	A	G	0.3991	0.038
rs3821819	LC_IGP37	IgG1 FG2S1/(FG2+FG2S1)	3	186732725	3	5.43E-26	2.50E-25	-	-0.386304	-0.3841	+	A	G	0.363771978	0.0360121	+	A	G	0.3991	0.0369
rs3821819	LC_IGP34	IgG1 FGS1/(FG+FGS1)	3	186732725	3	2.35E-09	2.97E-10	-	-0.221452	-0.2259	+	A	G	0.363827523	0.0368771	+	A	G	0.3991	0.0359
rs3821819	LC_IGP25	IgG1 SA per Gal	3	186732725	3	2.62E-14	2.62E-18	-	-0.284106	-0.3174	+	A	G	0.361635338	0.0369606	+	A	G	0.3993	0.0364
rs3821819	LC_IGP7	IgG1_G1FS1	3	186732725	3	6.60E-21	1.39E-33	-	-0.34294	-0.4559	+	A	G	0.363827523	0.0360641	+	A	G	0.3991	0.0378
rs3821819	LC_IGP_R34	IgG1_G1FS1/IgG1_G1F	3	186732725	3	6.37E-23	2.41E-41	-	-0.359754	-0.5133	+	A	G	0.363827523	0.0359489	+	A	G	0.3991	0.0381
rs3821819	LC_IGP_R35	IgG1_G2FS1/IgG1_G2F	3	186732725	3	4.87E-26	2.43E-25	-	-0.386667	-0.3842	+	A	G	0.363771978	0.0360096	+	A	G	0.3991	0.0369
rs3821819	LC_IGP122	IgG2 FG1S1/(FG1+FG1S1)	3	186732725	3	4.57E-34	1.74E-48	-	-0.443271	-0.5528	+	A	G	0.363556132	0.0356012	+	A	G	0.3991	0.0378
rs4686833	LC_IGP123	IgG2 FG2S1/(FG2+FG2S1)	3	186729012	3	7.17E-13	7.40E-20	-	-0.296402	-0.4191	+	C	A	0.764746398	0.0409728	+	C	A	0.768	0.046
rs3821819	LC_IGP120	IgG2 FGS1/(FG+FGS1)	3	186732725	3	2.40E-20	1.21E-32	-	-0.340493	-0.4408	+	A	G	0.363778083	0.0363522	+	A	G	0.3991	0.037
rs3821819	LC_IGP111	IgG2 SA per Gal	3	186732725	3	7.15E-25	2.99E-42	-	-0.380016	-0.5149	+	A	G	0.363913313	0.0362889	+	A	G	0.3989	0.0378
rs3821819	LC_IGP93	IgG2_G1FS1	3	186732725	3	1.04E-16	1.88E-31	-	-0.30708	-0.4308	+	A	G	0.363556132	0.0366018	+	A	G	0.3991	0.0369
rs3821819	LC_IGP_R74	IgG2_G1FS1/IgG2_G1F	3	186732725	3	4.49E-34	1.74E-48	-	-0.44334	-0.5528	+	A	G	0.363556132	0.0356023	+	A	G	0.3991	0.0378
rs3821819	LC_IGP_R75	IgG2_G2FS1/IgG2_G2F	3	186732725	3	7.31E-21	1.68E-43	-	-0.344657	-0.5221	+	A	G	0.363556132	0.0362883	+	A	G	0.3991	0.0378

rs3821819	LC_IGP189	IgG4 FG1S1/(FG1+FG1S1)	3	186732725	3	1.49E-20	1.60E-35	-	-0.341159	-0.4616	+	A	G	0.363328354	0.0362204	+	A	G	0.3991	0.0371
rs3821819	LC_IGP190	IgG4 FG2S1/(FG2+FG2S1)	3	186732725	3	6.74E-20	1.12E-33	-	-0.336947	-0.4477	+	A	G	0.363550031	0.0364235	+	A	G	0.3991	0.037
rs3821819	LC_IGP187	IgG4 FGS1/(FG+FGS1)	3	186732725	3	1.45E-13	3.80E-16	-	-0.27342	-0.2931	+	A	G	0.363550031	0.0366783	+	A	G	0.3991	0.036
rs3821819	LC_IGP186	IgG4 SA per Gal	3	186732725	3	5.68E-20	2.81E-27	-	-0.337914	-0.3995	+	A	G	0.36374541	0.0364512	+	A	G	0.3986	0.0369
rs3821819	LC_IGP179	IgG4_G1FS1	3	186732725	3	6.92E-16	4.47E-26	-	-0.297152	-0.3905	+	A	G	0.363328354	0.0364422	+	A	G	0.3991	0.037
rs3821819	LC_IGP_R92	IgG4_G1FS1/IgG4_G1F	3	186732725	3	1.18E-20	1.75E-35	-	-0.341997	-0.4614	+	A	G	0.363328354	0.0362112	+	A	G	0.3991	0.0371
rs3821819	LC_IGP_R93	IgG4_G2FS1/IgG4_G2F	3	186732725	3	6.82E-20	1.19E-33	-	-0.336891	-0.4476	+	A	G	0.363550031	0.0364228	+	A	G	0.3991	0.037
rs6444193	LC_IGP36	IgG1 FG1S1/(FG1+FG1S1)	3	186734188	3	5.58E-14	3.49E-19	-	-0.308541	-0.4137	+	A	G	0.764233592	0.0406821	+	A	G	0.7963	0.0462
rs6444193	LC_IGP37	IgG1 FG2S1/(FG2+FG2S1)	3	186734188	3	6.68E-18	6.23E-12	-	-0.355304	-0.3073	+	A	G	0.76436198	0.040744	+	A	G	0.7963	0.0447
rs6444193	LC_IGP25	IgG1 SA per Gal	3	186734188	3	1.35E-09	1.23E-09	-	-0.253107	-0.2668	+	A	G	0.761908787	0.0415143	+	A	G	0.7961	0.0439
rs6444193	LC_IGP7	IgG1_G1FS1	3	186734188	3	2.47E-12	1.23E-17	-	-0.287866	-0.3921	+	A	G	0.764233592	0.0407788	+	A	G	0.7963	0.0459
rs6444193	LC_IGP_R34	IgG1_G1FS1/IgG1_G1F	3	186734188	3	4.96E-14	2.94E-19	-	-0.309074	-0.4153	+	A	G	0.764233592	0.0406673	+	A	G	0.7963	0.0463
rs6444193	LC_IGP_R35	IgG1_G2FS1/IgG1_G2F	3	186734188	3	6.37E-18	6.18E-12	-	-0.355522	-0.3073	+	A	G	0.76436198	0.0407427	+	A	G	0.7963	0.0447
rs6444193	LC_IGP122	IgG2 FG1S1/(FG1+FG1S1)	3	186734188	3	2.12E-18	2.31E-25	-	-0.360554	-0.4774	+	A	G	0.764498733	0.0407155	+	A	G	0.7963	0.0459
rs4686832	LC_IGP123	IgG2 FG2S1/(FG2+FG2S1)	3	186729000	3	7.01E-13	9.79E-20	-	-0.297052	-0.4129	+	T	C	0.764777303	0.0410453	+	T	C	0.7752	0.0454
rs6444193	LC_IGP120	IgG2 FGS1/(FG+FGS1)	3	186734188	3	4.90E-11	1.43E-14	-	-0.272665	-0.3454	+	A	G	0.764354959	0.0411973	+	A	G	0.7963	0.0449
rs6444193	LC_IGP111	IgG2 SA per Gal	3	186734188	3	3.90E-13	6.54E-22	-	-0.303138	-0.4412	+	A	G	0.765034937	0.0414126	+	A	G	0.7959	0.0459
rs6444193	LC_IGP93	IgG2_G1FS1	3	186734188	3	1.62E-09	8.42E-18	-	-0.250924	-0.3844	+	A	G	0.764498733	0.0413642	+	A	G	0.7963	0.0447
rs6444193	LC_IGP_R74	IgG2_G1FS1/IgG2_G1F	3	186734188	3	2.27E-18	2.58E-25	-	-0.360258	-0.4769	+	A	G	0.764498733	0.0407189	+	A	G	0.7963	0.0459
rs6444193	LC_IGP_R75	IgG2_G2FS1/IgG2_G2F	3	186734188	3	7.14E-13	1.03E-20	-	-0.297016	-0.4277	+	A	G	0.764193974	0.0410551	+	A	G	0.7963	0.0458
rs6444193	LC_IGP189	IgG4 FG1S1/(FG1+FG1S1)	3	186734188	3	3.46E-10	2.39E-13	-	-0.259691	-0.33	+	A	G	0.76434477	0.0411181	+	A	G	0.7963	0.045
rs6444193	LC_IGP190	IgG4 FG2S1/(FG2+FG2S1)	3	186734188	3	2.43E-12	1.64E-13	-	-0.290795	-0.3317	+	A	G	0.764200991	0.041182	+	A	G	0.7963	0.045
rs6444193	LC_IGP186	IgG4 SA per Gal	3	186734188	3	2.40E-10	1.16E-11	-	-0.26311	-0.3039	+	A	G	0.76410338	0.041281	+	A	G	0.796	0.0448
rs6444193	LC_IGP179	IgG4_G1FS1	3	186734188	3	3.69E-08	1.99E-11	-	-0.227986	-0.3008	+	A	G	0.76434477	0.041217	+	A	G	0.7963	0.0449
rs6444193	LC_IGP_R92	IgG4_G1FS1/IgG4_G1F	3	186734188	3	3.64E-10	2.44E-13	-	-0.259337	-0.3299	+	A	G	0.76434477	0.0411146	+	A	G	0.7963	0.045
rs6444193	LC_IGP_R93	IgG4_G2FS1/IgG4_G2F	3	186734188	3	2.53E-12	1.65E-13	-	-0.290563	-0.3317	+	A	G	0.764200991	0.0411818	+	A	G	0.7963	0.045
rs967367	LC_IGP36	IgG1 FG1S1/(FG1+FG1S1)	3	186734466	3	8.41E-23	7.79E-41	-	-0.35957	-0.5125	+	A	G	0.363689297	0.0360357	+	A	G	0.3986	0.0383
rs967367	LC_IGP37	IgG1 FG2S1/(FG2+FG2S1)	3	186734466	3	9.98E-26	3.53E-25	-	-0.385025	-0.385	+	A	G	0.363635836	0.0360957	+	A	G	0.3986	0.0371
rs967367	LC_IGP34	IgG1 FGS1/(FG+FGS1)	3	186734466	3	3.23E-09	3.84E-10	-	-0.219967	-0.2256	+	A	G	0.363689297	0.0369561	+	A	G	0.3986	0.036
rs967367	LC_IGP25	IgG1 SA per Gal	3	186734466	3	3.47E-14	3.64E-18	-	-0.283369	-0.3176	+	A	G	0.361470865	0.0370446	+	A	G	0.3988	0.0366
rs967367	LC_IGP7	IgG1_G1FS1	3	186734466	3	8.87E-21	3.54E-33	-	-0.342499	-0.4559	+	A	G	0.363689297	0.036141	+	A	G	0.3986	0.038
rs967367	LC_IGP_R34	IgG1_G1FS1/IgG1_G1F	3	186734466	3	1.01E-22	1.15E-40	-	-0.358827	-0.5122	+	A	G	0.363689297	0.0360291	+	A	G	0.3986	0.0384
rs967367	LC_IGP_R35	IgG1_G2FS1/IgG1_G2F	3	186734466	3	8.99E-26	3.43E-25	-	-0.385372	-0.3852	+	A	G	0.363635836	0.0360933	+	A	G	0.3986	0.0371
rs967367	LC_IGP122	IgG2 FG1S1/(FG1+FG1S1)	3	186734466	3	6.76E-34	7.64E-48	-	-0.443035	-0.5514	+	A	G	0.363388041	0.0356805	+	A	G	0.3986	0.0379
rs57679165	LC_IGP_R34	IgG1_G1FS1/IgG1_G1F	3	186763145	4	2.50E-10	3.25E-19	-	-0.281884	-0.4284	+	G	C	0.185890192	0.0442738	+	G	C	0.2028	0.0478
rs967367	LC_IGP120	IgG2 FGS1/(FG+FGS1)	3	186734466	3	2.64E-20	2.91E-32	-	-0.340801	-0.4397	+	A	G	0.36360989	0.0364266	+	A	G	0.3986	0.0372
rs967367	LC_IGP111	IgG2 SA per Gal	3	186734466	3	9.31E-25	1.13E-41	-	-0.379786	-0.5137	+	A	G	0.363818266	0.0363599	+	A	G	0.3984	0.038
rs967367	LC_IGP93	IgG2_G1FS1	3	186734466	3	1.22E-16	6.88E-31	-	-0.306989	-0.429	+	A	G	0.363388041	0.0366781	+	A	G	0.3986	0.0371
rs967367	LC_IGP_R74	IgG2_G1FS1/IgG2_G1F	3	186734466	3	6.64E-34	7.62E-48	-	-0.443105	-0.5514	+	A	G	0.363388041	0.0356815	+	A	G	0.3986	0.0379
rs967367	LC_IGP_R75	IgG2_G2FS1/IgG2_G2F	3	186734466	3	1.00E-20	5.05E-43	-	-0.34415	-0.5219	+	A	G	0.363388041	0.0363673	+	A	G	0.3986	0.038
rs967367	LC_IGP189	IgG4 FG1S1/(FG1+FG1S1)	3	186734466	3	2.12E-20	3.46E-35	-	-0.340486	-0.4616	+	A	G	0.363192378	0.0362987	+	A	G	0.3986	0.0373
rs967367	LC_IGP190	IgG4 FG2S1/(FG2+FG2S1)	3	186734466	3	8.45E-20	2.61E-33	-	-0.33672	-0.4477	+	A	G	0.363413972	0.0364996	+	A	G	0.3986	0.0372
rs967367	LC_IGP187	IgG4 FGS1/(FG+FGS1)	3	186734466	3	1.75E-13	5.77E-16	-	-0.273064	-0.2928	+	A	G	0.363413972	0.0367539	+	A	G	0.3986	0.0362
rs967367	LC_IGP186	IgG4 SA per Gal	3	186734466	3	8.07E-20	5.20E-27	-	-0.337202	-0.3994	+	A	G	0.363609853	0.0365302	+	A	G	0.398	0.0371
rs967367	LC_IGP179	IgG4_G1FS1	3	186734466	3	8.72E-16	9.40E-26	-	-0.296711	-0.3901	+	A	G	0.363192378	0.0365183	+	A	G	0.3986	0.0372
rs967367	LC_IGP_R92	IgG4_G1FS1/IgG4_G1F	3	186734466	3	1.68E-20	3.82E-35	-	-0.341326	-0.4613	+	A	G	0.363192378	0.0362895	+	A	G	0.3986	0.0373
rs967367	LC_IGP_R93	IgG4_G2FS1/IgG4_G2F	3	186734466	3	8.57E-20	2.78E-33	-	-0.336655	-0.4475	+	A	G	0.363413972	0.0364989	+	A	G	0.3986	0.0372
rs1009640	LC_IGP36	IgG1 FG1S1/(FG1+FG1S1)	3	186735096	3	6.59E-12	2.50E-19	-	-0.327268	-0.4743	+	T	C	0.838786791	0.0473126	+	T	C	0.8507	0.0528
rs1009640	LC_IGP37	IgG1 FG2S1/(FG2+FG2S1)	3	186735096	3	9.67E-17	3.10E-12	-	-0.397405	-0.3559	+	T	C	0.838779649	0.0473183	+	T	C	0.8507	0.051
rs1009640	LC_IGP25	IgG1 SA per Gal	3	186735096	3	9.64E-11	5.47E-11	-	-0.312379	-0.3288	+	T	C	0.836727706	0.0479386	+	T	C	0.8506	0.0501
rs1009640	LC_IGP7	IgG1_G1FS1	3	186735096	3	1.48E-10	5.20E-17	-	-0.305728	-0.4376	+	T	C	0.838786791	0.0474053	+	T	C	0.8507	0.0522
rs1009640	LC_IGP_R34	IgG1_G1FS1/IgG1_G1F	3	186735096	3	5.61E-12	2.24E-19	-	-0.328257	-0.4758	+	T	C	0.838786791	0.0472941	+	T	C	0.8507	0.0529
rs1009640	LC_IGP_R35	IgG1_G2FS1/IgG1_G2F	3	186735096	3	9.87E-17	3.04E-12	-	-0.397286	-0.3561	+	T	C	0.838779649	0.0473188	+	T	C	0.8507	0.051
rs1009640	LC_IGP122	IgG2 FG1S1/(FG1+FG1S1)	3	186735096	3	4.41E-17	1.03E-22	-	-0.401756	-0.5138	+	T	C	0.838877807	0.0472982	+	T	C	0.8507	0.0524
chr3:186738103:I	LC_IGP123	IgG2 FG2S1/(FG2+FG2S1)	3	186738103	3	4.96E-10	1.03E-19	-	-0.467127	-0.8005	+	AC	A	0.086374786	0.0746435	+	AC	A	0.0906	0.0881
rs1009640	LC_IGP120	IgG2 FGS1/(FG+FGS1)	3	186735096	3	1.09E-11	1.39E-13	-	-0.326619	-0.3797	+	T	C	0.838779442	0.0477237	+	T	C	0.8507	0.0513
rs1009640	LC_IGP111	IgG2 SA per Gal	3	186735096	3	6.58E-13	2.84E-19	-	-0.347571	-0.4703	+	T	C	0.839270638	0.0479612	+	T	C	0.8503	0.0524
rs1009640	LC_IGP93	IgG2_G1FS1	3	186735096	3	5.49E-10	2.21E-17	-	-0.299192	-0.4339	+	T	C	0.838877807	0.0479328	+	T	C	0.8507	0.0512
rs1009640	LC_IGP_R74	IgG2_G1FS1/IgG2_G1F	3	186735096	3	4.40E-17	1.12E-22	-	-0.401781	-0.5134	+	T	C	0.838877807	0.0473	+	T	C	0.8507	0.0524
rs1009640	LC_IGP_R75	IgG2_G2FS1/IgG2_G2F	3	186735096	3	1.02E-11	4.15E-18	-	-0.326616	-0.4542	+	T	C	0.838573166	0.0476643	+	T	C	0.8507	0.0524
rs1009640	LC_IGP190	IgG4 FG2S1/(FG2+FG2S1)	3	186735096	3	9.78E-09	5.21E-11	-	-0.276534	-0.3369	+	T	C	0.838573166	0.0479733	+	T	C	0.8507	0.0513
rs1009640	LC_IGP186	IgG4 SA per Gal	3	186735096	3	1.53E-08	1.17E-09	-	-0.27288	-0.3109	+	T	C	0.838703232	0.0479876	+	T	C	0.8506	0.0511
rs1009640	LC_IGP_R93	IgG4_G2FS1/IgG4_G2F	3	186735096	3	1.01E-08	5.15E-11	-	-0.276278	-0.3371	+	T	C	0.838573166	0.0479728	+	T	C	0.8507	0.0513
rs5001409	LC_IGP36	IgG1 FG1S1/(FG1+FG1S1)	3	186735690	3	1.24E-22	2.67E-40	-	-0.358354	-0.5109	+	C	A	0.36504526	0.0360608	+	C	A	0.3995	0.0384
rs5001409	LC_IGP37	IgG1 FG2S1/(FG2+FG2S1)	3	186735690	3	1.74E-25	6.03E-25	-	-0.383338	-0.3849	+	C	A	0.364990232	0.0361245	+	C	A	0.3995	0.0373
rs5001409	LC_IGP34	IgG1 FGS1/(FG+FGS1)	3	186735690	3	4.86E-09	4.94E-10	-	-0.217585	-0.2252	+	C	A	0.36504526	0.0369822	+	C	A	0.3995	0.0362
rs5001409	LC_IGP25	IgG1 SA per Gal	3	186735690	3	6.40E-14	4.98E-18	-	-0.280587	-0.3177	+	C	A	0.362838972	0.0370785	+	C	A	0.3997	0.0367
rs5001409	LC_IGP7	IgG1_G1FS1	3	186735690	3	1.11E-20	4.49E-33	-	-0.341796	-0.4566	+	C	A	0.36504526	0.0361627	+	C	A	0.3995	0.0381
rs5001409	LC_IGP_R34	IgG1_G1FS1/IgG1_G1F	3	186735690	3	1.47E-22	3.94E-40	-	-0.357622	-0.5105	+	C	A	0.36504526	0.0360541	+	C	A	0.3995	0.0385
rs5001409	LC_IGP_R35	IgG1_G2FS1/IgG1_G2F	3	186735690	3	1.58E-25	5.86E-25	-	-0.383651	-0.385	+	C	A	0.364990232	0.0361224	+	C	A	0.3995	0.0373
rs5001409	LC_IGP122	IgG2 FG1S1/(FG1+FG1S1)	3	186735690	3	2.51E-33	3.57E-47	-	-0.439474	-0.5496	+	C	A	0.364716595	0.0357259	+	C	A	0.3995	0.0381
rs6780488	LC_IGP123	IgG2 FG2S1/(FG2+FG2S1)	3	186763942	4	2.67E-11	1.06E-19	-	-0.298979	-0.4317	+	A	G	0.185517175	0.0445577	+	A	G	0.2029	0.0475
rs5001409	LC_IGP120	IgG2 FGS1/(FG+FGS1)	3	186735690	3	6.51E-20	8.02E-32	-	-0.337465	-0.4384	+	C	A	0.364939255	0.0364639	+	C	A	0.3995	0.0373
rs5001409	LC_IGP111	IgG2 SA per Gal	3	186735690	3	2.89E-24	4.97E-41	-	-0.375989	-0.5117	+	C	A	0.365221053	0.0363977	+	C	A	0.3993	0.0381
rs5001409	LC_IGP93	IgG2_G1FS1	3	186735690	3	3.47E-16	1.47E-30	-	-0.302587	-0.4285	+	C	A	0.364716595	0.0367186	+	C	A	0.3995	0.0373
rs5001409	LC_IGP_R74	IgG2_G1FS1/IgG2_G1F	3	186735690	3	2.47E-33	3.56E-47	-	-0.439547	-0.5496	+	C	A	0.364716595	0.035727	+	C	A	0.3995	0.0381
rs5001409	LC_IGP_R75	IgG2_G2FS1/IgG2_G2F	3	186735690	3	2.58E-20	1.96E-42	-	-0.340684	-0.5206	+	C	A	0.364716595	0.0364055	+	C	A	0.3995	0.0381
rs5001409	LC_IGP189	IgG4 FG1S1/(FG1+FG1S1)	3	186735690	3	5.58E-20	8.41E-35	-	-0.336909	-0.4606	+	C	A	0.364545122	0.0363366	+	C	A	0.3995	0.0374
rs5001409	LC_IGP190	IgG4 FG2S1/(FG2+FG2S1)	3	186735690	3	2.09E-19	6.10E-33	-	-0.333315	-0.4468	+	C	A	0.364767541	0.0365363	+	C	A	0.3995	0.0374
rs5001409	LC_IGP187	IgG4 FGS1/(FG+FGS1)	3	186735690	3	3.70E-13	8.10E-16	-	-0.269515	-0.2925	+	C	A	0.364767541	0.0367874	+	C	A	0.3995	0.0363
rs5001409	LC_IGP186	IgG4 SA per Gal	3	186735690	3	2.29E-19	1.02E-26	-	-0.333246	-0.3988	+	C	A	0.36496787	0.0365701	+	C	A	0.3989	0.0373
rs5001409	LC_IGP179	IgG4_G1FS1	3	186735690	3	1.56E-15	1.71E-25	-	-0.29425	-0.3896	+	C	A	0.364545122	0.0365478	+	C	A	0.3995	0.0373
rs5001409	LC_IGP_R92	IgG4_G1FS1/IgG4_G1F	3	186735690	3	4.48E-20	9.44E-35	-	-0.337721	-0.4603	+	C	A	0.364545122	0.0363275	+	C	A	0.3995	0.0374
rs5001409	LC_IGP_R93	IgG4_G2FS1/IgG4_G2F	3	186735690	3	2.13E-19	6.50E-33	-	-0.333225	-0.4467	+	C	A	0.364767541	0.0365358	+	C	A	0.3995	0.0374
rs75572438	LC_IGP122	IgG2 FG1S1/(FG1+FG1S1)	3	186735962	3	7.06E-11	3.18E-13	-	-0.568128	-0.6966	+	A	G	0.05283075	0.0865655	+	A	G	0.0526	0.0956
rs75572438	LC_IGP_R74	IgG2_G1FS1/IgG2_G1F	3	186735962	3	7.00E-11	3.18E-13	-	-0.568252	-0.6963	+	A	G	0.05283075	0.0865687	+	A	G	0.0526	0.0956
rs9863411	LC_IGP36	IgG1 FG1S1/(FG1+FG1S1)	3	186737820	3	3.18E-22	5.22E-39	-	-0.354837	-0.5032	+	T	C	0.364662997	0.0360706	+	T	C	0.3976	0.0385
rs9863411	LC_IGP37	IgG1 FG2S1/(FG2+FG2S1)	3	186737820	3	5.98E-25	1.06E-24	-	-0.379031	-0.3837	+	T	C	0.364610806	0.036141	+	T	C	0.3976	0.0374
rs9863411	LC_IGP34	IgG1 FGS1/(FG+FGS1)	3	186737820	3	8.80E-09	6.97E-10	-	-0.213863	-0.2237	+	T	C	0.364662997	0.0369842	+	T	C	0.3976	0.0363
rs9863411	LC_IGP25	IgG1 SA per Gal	3	186737820	3	1.18E-13	9.89E-18	-	-0.277573	-0.3154	+	T	C	0.362419173	0.0370875	+	T	C	0.3978	0.0368
rs9863411	LC_IGP7	IgG1_G1FS1	3	186737820	3	2.21E-20	5.32E-32	-	-0.339087	-0.4499	+	T	C	0.364662997	0.0361669	+	T	C	0.3976	0.0382
rs9863411	LC_IGP_R34	IgG1_G1FS1/IgG1_G1F	3	186737820	3	3.79E-22	7.79E-39	-	-0.354092	-0.5028	+	T	C	0.364662997	0.0360639	+	T	C	0.3976	0.0386
rs9863411	LC_IGP_R35	IgG1_G2FS1/IgG1_G2F	3	186737820	3	5.48E-25	1.03E-24	-	-0.379324	-0.3838	+	T	C	0.364610806	0.0361391	+	T	C	0.3976	0.0374
rs9863411	LC_IGP122	IgG2 FG1S1/(FG1+FG1S1)	3	186737820	3	7.20E-33	1.09E-45	-	-0.436303	-0.5422	+	T	C	0.364296522	0.0357386	+	T	C	0.3976	0.0382
rs4686831	LC_IGP123	IgG2 FG2S1/(FG2+FG2S1)	3	186728647	3	7.78E-13	1.67E-19	-	-0.296272	-0.4124	+	T	C	0.765212325	0.0410199	+	T	C	0.777	0.0457
rs9863411	LC_IGP120	IgG2 FGS1/(FG+FGS1)	3	186737820	3	1.04E-19	3.95E-31	-	-0.335537	-0.4344	+	T	C	0.364518926	0.0364639	+	T	C	0.3976	0.0374
rs9863411	LC_IGP111	IgG2 SA per Gal	3	186737820	3	5.97E-24	5.31E-40	-	-0.37326	-0.5063	+	T	C	0.364899381	0.0363962	+	T	C	0.3974	0.0382
rs9863411	LC_IGP93	IgG2_G1FS1	3	186737820	3	5.78E-16	2.21E-29	-	-0.30025	-0.421	+	T	C	0.364296522	0.0367195	+	T	C	0.3976	0.0374
rs9863411	LC_IGP_R74	IgG2_G1FS1/IgG2_G1F	3	186737820	3	7.08E-33	1.08E-45	-	-0.436371	-0.5423	+	T	C	0.364296522	0.0357397	+	T	C	0.3976	0.0382
rs9863411	LC_IGP_R75	IgG2_G2FS1/IgG2_G2F	3	186737820	3	5.25E-20	8.01E-42	-	-0.337848	-0.5177	+	T	C	0.364296522	0.0364108	+	T	C	0.3976	0.0382
rs9863411	LC_IGP189	IgG4 FG1S1/(FG1+FG1S1)	3	186737820	3	1.24E-19	6.25E-34	-	-0.333716	-0.4556	+	T	C	0.364166159	0.0363434	+	T	C	0.3976	0.0375
rs9863411	LC_IGP190	IgG4 FG2S1/(FG2+FG2S1)	3	186737820	3	4.05E-19	5.36E-32	-	-0.330584	-0.4414	+	T	C	0.364388347	0.0365403	+	T	C	0.3976	0.0375
rs9863411	LC_IGP187	IgG4 FGS1/(FG+FGS1)	3	186737820	3	6.16E-13	2.59E-15	-	-0.266908	-0.2883	+	T	C	0.364388347	0.0367879	+	T	C	0.3976	0.0365
rs9863411	LC_IGP186	IgG4 SA per Gal	3	186737820	3	4.96E-19	6.07E-26	-	-0.330068	-0.3939	+	T	C	0.364589045	0.0365763	+	T	C	0.3968	0.0374
rs9863411	LC_IGP179	IgG4_G1FS1	3	186737820	3	2.61E-15	1.08E-24	-	-0.291855	-0.3842	+	T	C	0.364166159	0.0365485	+	T	C	0.3976	0.0374
rs9863411	LC_IGP_R92	IgG4_G1FS1/IgG4_G1F	3	186737820	3	9.95E-20	7.03E-34	-	-0.334525	-0.4552	+	T	C	0.364166159	0.0363345	+	T	C	0.3976	0.0375
rs9863411	LC_IGP_R93	IgG4_G2FS1/IgG4_G2F	3	186737820	3	4.14E-19	5.71E-32	-	-0.330483	-0.4412	+	T	C	0.364388347	0.0365398	+	T	C	0.3976	0.0375
rs10937280	LC_IGP36	IgG1 FG1S1/(FG1+FG1S1)	3	186738033	3	3.52E-22	7.16E-39	-	-0.354398	-0.5023	+	A	G	0.364650459	0.0360658	+	A	G	0.3975	0.0385
rs10937280	LC_IGP37	IgG1 FG2S1/(FG2+FG2S1)	3	186738033	3	6.82E-25	1.20E-24	-	-0.378506	-0.3833	+	A	G	0.364598596	0.036137	+	A	G	0.3975	0.0374
rs10937280	LC_IGP34	IgG1 FGS1/(FG+FGS1)	3	186738033	3	9.34E-09	7.51E-10	-	-0.213452	-0.2233	+	A	G	0.364650459	0.0369783	+	A	G	0.3975	0.0363
rs10937280	LC_IGP25	IgG1 SA per Gal	3	186738033	3	1.26E-13	1.11E-17	-	-0.2772	-0.315	+	A	G	0.362403423	0.0370824	+	A	G	0.3977	0.0368
rs10937280	LC_IGP7	IgG1_G1FS1	3	186738033	3	2.39E-20	6.62E-32	-	-0.338726	-0.4492	+	A	G	0.364650459	0.0361615	+	A	G	0.3975	0.0382
rs10937280	LC_IGP_R34	IgG1_G1FS1/IgG1_G1F	3	186738033	3	4.20E-22	1.07E-38	-	-0.353652	-0.5019	+	A	G	0.364650459	0.0360591	+	A	G	0.3975	0.0386
rs10937280	LC_IGP_R35	IgG1_G2FS1/IgG1_G2F	3	186738033	3	6.26E-25	1.17E-24	-	-0.378798	-0.3834	+	A	G	0.364598596	0.036135	+	A	G	0.3975	0.0374
rs10937280	LC_IGP122	IgG2 FG1S1/(FG1+FG1S1)	3	186738033	3	8.13E-33	1.65E-45	-	-0.435864	-0.5412	+	A	G	0.364279744	0.0357343	+	A	G	0.3975	0.0382
rs13082825	LC_IGP123	IgG2 FG2S1/(FG2+FG2S1)	3	186708013	1	5.43E-11	5.69E-19	-	-0.244676	-0.4154	+	C	G	0.547688835	0.0370557	+	C	G	0.5381	0.0467
rs10937280	LC_IGP120	IgG2 FGS1/(FG+FGS1)	3	186738033	3	1.10E-19	5.18E-31	-	-0.335264	-0.4336	+	A	G	0.364502137	0.036458	+	A	G	0.3975	0.0374
rs10937280	LC_IGP111	IgG2 SA per Gal	3	186738033	3	6.50E-24	7.52E-40	-	-0.372884	-0.5054	+	A	G	0.36489226	0.0363905	+	A	G	0.3973	0.0382
rs10937280	LC_IGP93	IgG2_G1FS1	3	186738033	3	6.13E-16	2.94E-29	-	-0.299933	-0.4201	+	A	G	0.364279744	0.0367137	+	A	G	0.3975	0.0374

rs10937280	LC_IGP_R74	IgG2_G1FS1/IgG2_G1F	3	186738033	3	7.99E-33	1.63E-45	-	-0.435932	-0.5413	+	A	G	0.364279744	0.0357354	+	A	G	0.3975	0.0382
rs10937280	LC_IGP_R75	IgG2_G2FS1/IgG2_G2F	3	186738033	3	5.67E-20	1.08E-41	-	-0.337481	-0.5169	+	A	G	0.364279744	0.0364055	+	A	G	0.3975	0.0382
rs10937280	LC_IGP189	IgG4 FG1S1/(FG1+FG1S1)	3	186738033	3	1.35E-19	8.01E-34	-	-0.333316	-0.4549	+	A	G	0.364153963	0.0363383	+	A	G	0.3975	0.0375
rs10937280	LC_IGP190	IgG4 FG2S1/(FG2+FG2S1)	3	186738033	3	4.36E-19	6.81E-32	-	-0.330224	-0.4406	+	A	G	0.364376144	0.0365349	+	A	G	0.3975	0.0375
rs10937280	LC_IGP187	IgG4 FGS1/(FG+FGS1)	3	186738033	3	6.49E-13	2.93E-15	-	-0.2666	-0.2877	+	A	G	0.364376144	0.036782	+	A	G	0.3975	0.0365
rs10937280	LC_IGP186	IgG4 SA per Gal	3	186738033	3	5.42E-19	7.40E-26	-	-0.329653	-0.3933	+	A	G	0.364577111	0.0365711	+	A	G	0.3968	0.0374
rs10937280	LC_IGP179	IgG4_G1FS1	3	186738033	3	2.77E-15	1.32E-24	-	-0.291532	-0.3835	+	A	G	0.364153963	0.0365428	+	A	G	0.3975	0.0374
rs10937280	LC_IGP_R92	IgG4_G1FS1/IgG4_G1F	3	186738033	3	1.08E-19	9.02E-34	-	-0.334125	-0.4545	+	A	G	0.364153963	0.0363294	+	A	G	0.3975	0.0375
rs10937280	LC_IGP_R93	IgG4_G2FS1/IgG4_G2F	3	186738033	3	4.46E-19	7.25E-32	-	-0.330123	-0.4405	+	A	G	0.364376144	0.0365344	+	A	G	0.3975	0.0375
chr3:186738103:I	LC_IGP36	IgG1 FG1S1/(FG1+FG1S1)	3	186738103	3	3.65E-12	1.80E-21	-	-0.516985	-0.8613	+	AC	A	0.086585342	0.0738252	+	AC	A	0.0906	0.0905
chr3:186738103:I	LC_IGP37	IgG1 FG2S1/(FG2+FG2S1)	3	186738103	3	9.17E-16	1.64E-10	-	-0.600779	-0.5701	+	AC	A	0.08643236	0.0739994	+	AC	A	0.0906	0.0892
chr3:186738103:I	LC_IGP25	IgG1 SA per Gal	3	186738103	3	1.76E-08	8.62E-08	-	-0.425879	-0.4722	+	AC	A	0.086716972	0.0752075	+	AC	A	0.0907	0.0882
chr3:186738103:I	LC_IGP7	IgG1_G1FS1	3	186738103	3	7.28E-11	5.64E-13	-	-0.485089	-0.668	+	AC	A	0.086585342	0.0739648	+	AC	A	0.0906	0.0927
chr3:186738103:I	LC_IGP_R34	IgG1_G1FS1/IgG1_G1F	3	186738103	3	4.19E-12	2.94E-21	-	-0.515419	-0.86	+	AC	A	0.086585342	0.0738096	+	AC	A	0.0906	0.0909
chr3:186738103:I	LC_IGP_R35	IgG1_G2FS1/IgG1_G2F	3	186738103	3	1.01E-15	1.65E-10	-	-0.599872	-0.57	+	AC	A	0.08643236	0.0740037	+	AC	A	0.0906	0.0892
chr3:186738103:I	LC_IGP122	IgG2 FG1S1/(FG1+FG1S1)	3	186738103	3	2.05E-18	1.19E-25	-	-0.653252	-0.9032	+	AC	A	0.086374786	0.0737338	+	AC	A	0.0906	0.0863
rs4686827	LC_IGP123	IgG2 FG2S1/(FG2+FG2S1)	3	186713686	3	1.60E-11	6.83E-19	-	-0.331922	-0.4882	+	A	C	0.839021623	0.048912	+	A	C	0.8458	0.055
chr3:186738103:I	LC_IGP120	IgG2 FGS1/(FG+FGS1)	3	186738103	3	2.65E-11	1.86E-15	-	-0.50048	-0.6838	+	AC	A	0.086427518	0.0745722	+	AC	A	0.0906	0.086
chr3:186738103:I	LC_IGP111	IgG2 SA per Gal	3	186738103	3	6.41E-13	2.94E-20	-	-0.539204	-0.8117	+	AC	A	0.087072723	0.0743656	+	AC	A	0.0907	0.088
chr3:186738103:I	LC_IGP93	IgG2_G1FS1	3	186738103	3	1.85E-09	7.83E-15	-	-0.452848	-0.6997	+	AC	A	0.086374786	0.0749158	+	AC	A	0.0906	0.09
chr3:186738103:I	LC_IGP_R74	IgG2_G1FS1/IgG2_G1F	3	186738103	3	2.27E-18	1.22E-25	-	-0.652411	-0.903	+	AC	A	0.086374786	0.0737414	+	AC	A	0.0906	0.0863
chr3:186738103:I	LC_IGP_R75	IgG2_G2FS1/IgG2_G2F	3	186738103	3	4.81E-10	9.94E-20	-	-0.467503	-0.8008	+	AC	A	0.086374786	0.0746442	+	AC	A	0.0906	0.0881
chr3:186738103:I	LC_IGP189	IgG4 FG1S1/(FG1+FG1S1)	3	186738103	3	5.16E-15	1.52E-12	-	-0.584312	-0.6274	+	AC	A	0.086326955	0.0739832	+	AC	A	0.0906	0.0887
chr3:186738103:I	LC_IGP190	IgG4 FG2S1/(FG2+FG2S1)	3	186738103	3	4.20E-10	1.02E-11	-	-0.470196	-0.6121	+	AC	A	0.086379625	0.0748151	+	AC	A	0.0906	0.09
chr3:186738103:I	LC_IGP186	IgG4 SA per Gal	3	186738103	3	5.21E-12	3.71E-10	-	-0.518179	-0.5599	+	AC	A	0.086617418	0.0745432	+	AC	A	0.0902	0.0894
chr3:186738103:I	LC_IGP179	IgG4_G1FS1	3	186738103	3	9.74E-10	1.61E-07	-	-0.458112	-0.4803	+	AC	A	0.086326955	0.0744955	+	AC	A	0.0906	0.0916
chr3:186738103:I	LC_IGP_R92	IgG4_G1FS1/IgG4_G1F	3	186738103	3	5.04E-15	1.96E-12	-	-0.584448	-0.6245	+	AC	A	0.086326955	0.0739737	+	AC	A	0.0906	0.0887
chr3:186738103:I	LC_IGP_R93	IgG4_G2FS1/IgG4_G2F	3	186738103	3	4.22E-10	1.01E-11	-	-0.470121	-0.6121	+	AC	A	0.086379625	0.0748133	+	AC	A	0.0906	0.0899
rs7622548	LC_IGP36	IgG1 FG1S1/(FG1+FG1S1)	3	186738118	3	4.30E-14	3.02E-18	-	-0.312276	-0.4058	+	C	G	0.764004162	0.0409851	+	C	G	0.7968	0.0466
rs7622548	LC_IGP37	IgG1 FG2S1/(FG2+FG2S1)	3	186738118	3	2.08E-18	1.11E-11	-	-0.363396	-0.3067	+	C	G	0.764136633	0.041025	+	C	G	0.7968	0.0452
rs7622548	LC_IGP25	IgG1 SA per Gal	3	186738118	3	4.74E-10	2.72E-09	-	-0.261997	-0.2639	+	C	G	0.761680329	0.041808	+	C	G	0.7966	0.0444
rs7622548	LC_IGP7	IgG1_G1FS1	3	186738118	3	1.82E-12	3.97E-17	-	-0.291772	-0.3892	+	C	G	0.764004162	0.0410815	+	C	G	0.7968	0.0463
rs7622548	LC_IGP_R34	IgG1_G1FS1/IgG1_G1F	3	186738118	3	3.89E-14	2.59E-18	-	-0.312716	-0.4074	+	C	G	0.764004162	0.0409706	+	C	G	0.7968	0.0467
rs7622548	LC_IGP_R35	IgG1_G2FS1/IgG1_G2F	3	186738118	3	2.00E-18	1.10E-11	-	-0.363561	-0.3068	+	C	G	0.764136633	0.041024	+	C	G	0.7968	0.0452
rs7622548	LC_IGP122	IgG2 FG1S1/(FG1+FG1S1)	3	186738118	3	3.85E-18	3.81E-24	-	-0.360567	-0.4692	+	C	G	0.764256135	0.0410417	+	C	G	0.7968	0.0463
rs7652995	LC_IGP123	IgG2 FG2S1/(FG2+FG2S1)	3	186722944	3	3.96E-12	1.34E-18	-	-0.337161	-0.4594	+	A	G	0.841326292	0.0482288	+	A	G	0.8505	0.0522
rs7622548	LC_IGP120	IgG2 FGS1/(FG+FGS1)	3	186738118	3	7.43E-11	5.50E-14	-	-0.272189	-0.3406	+	C	G	0.764112213	0.0415228	+	C	G	0.7968	0.0453
rs7622548	LC_IGP111	IgG2 SA per Gal	3	186738118	3	7.22E-13	4.12E-21	-	-0.302004	-0.4368	+	C	G	0.764819694	0.041748	+	C	G	0.7963	0.0463
rs7622548	LC_IGP93	IgG2_G1FS1	3	186738118	3	2.00E-09	5.04E-17	-	-0.251439	-0.3788	+	C	G	0.764256135	0.0416857	+	C	G	0.7968	0.0452
rs7622548	LC_IGP_R74	IgG2_G1FS1/IgG2_G1F	3	186738118	3	4.11E-18	4.20E-24	-	-0.360277	-0.4688	+	C	G	0.764256135	0.041045	+	C	G	0.7968	0.0463
rs7622548	LC_IGP_R75	IgG2_G2FS1/IgG2_G2F	3	186738118	3	2.01E-12	2.60E-20	-	-0.29342	-0.4272	+	C	G	0.763952596	0.0413949	+	C	G	0.7968	0.0463
rs7622548	LC_IGP189	IgG4 FG1S1/(FG1+FG1S1)	3	186738118	3	2.66E-10	4.01E-13	-	-0.263339	-0.3298	+	C	G	0.764120003	0.0414244	+	C	G	0.7968	0.0454
rs7622548	LC_IGP190	IgG4 FG2S1/(FG2+FG2S1)	3	186738118	3	1.48E-12	4.27E-13	-	-0.295859	-0.329	+	C	G	0.763976086	0.041483	+	C	G	0.7968	0.0454
rs7622548	LC_IGP186	IgG4 SA per Gal	3	186738118	3	1.86E-10	2.13E-11	-	-0.266717	-0.3027	+	C	G	0.763876564	0.041589	+	C	G	0.7963	0.0452
rs7622548	LC_IGP179	IgG4_G1FS1	3	186738118	3	3.95E-08	2.96E-11	-	-0.22922	-0.301	+	C	G	0.764120003	0.0415322	+	C	G	0.7968	0.0453
rs7622548	LC_IGP_R92	IgG4_G1FS1/IgG4_G1F	3	186738118	3	2.86E-10	4.07E-13	-	-0.262838	-0.3297	+	C	G	0.764120003	0.0414214	+	C	G	0.7968	0.0455
rs7622548	LC_IGP_R93	IgG4_G2FS1/IgG4_G2F	3	186738118	3	1.54E-12	4.30E-13	-	-0.295636	-0.329	+	C	G	0.763976086	0.0414828	+	C	G	0.7968	0.0454
rs7634389	LC_IGP36	IgG1 FG1S1/(FG1+FG1S1)	3	186738421	3	4.29E-22	1.34E-38	-	-0.353523	-0.5005	+	C	T	0.364623853	0.0360544	+	C	T	0.3973	0.0385
rs7634389	LC_IGP37	IgG1 FG2S1/(FG2+FG2S1)	3	186738421	3	8.74E-25	1.50E-24	-	-0.377491	-0.3825	+	C	T	0.364572344	0.0361267	+	C	T	0.3973	0.0374
rs7634389	LC_IGP34	IgG1 FGS1/(FG+FGS1)	3	186738421	3	1.04E-08	8.22E-10	-	-0.212713	-0.2228	+	C	T	0.364623853	0.0369643	+	C	T	0.3973	0.0363
rs7634389	LC_IGP25	IgG1 SA per Gal	3	186738421	3	1.43E-13	1.32E-17	-	-0.276493	-0.3143	+	C	T	0.362371554	0.0370697	+	C	T	0.3975	0.0368
rs7634389	LC_IGP7	IgG1_G1FS1	3	186738421	3	2.79E-20	1.09E-31	-	-0.337983	-0.4477	+	C	T	0.364623853	0.0361491	+	C	T	0.3973	0.0382
rs7634389	LC_IGP_R34	IgG1_G1FS1/IgG1_G1F	3	186738421	3	5.12E-22	2.03E-38	-	-0.352776	-0.5	+	C	T	0.364623853	0.0360476	+	C	T	0.3973	0.0386
rs7634389	LC_IGP_R35	IgG1_G2FS1/IgG1_G2F	3	186738421	3	8.04E-25	1.46E-24	-	-0.377778	-0.3826	+	C	T	0.364572344	0.0361247	+	C	T	0.3973	0.0374
rs7634389	LC_IGP122	IgG2 FG1S1/(FG1+FG1S1)	3	186738421	3	1.02E-32	3.47E-45	-	-0.435017	-0.5394	+	C	T	0.364245882	0.0357236	+	C	T	0.3973	0.0382
rs11923417	LC_IGP123	IgG2 FG2S1/(FG2+FG2S1)	3	186705790	1	1.82E-10	2.58E-18	-	-0.261633	-0.432	+	C	G	0.63759701	0.0407726	+	C	G	0.6235	0.0495
rs7634389	LC_IGP120	IgG2 FGS1/(FG+FGS1)	3	186738421	3	1.21E-19	7.98E-31	-	-0.334749	-0.4324	+	C	T	0.364468254	0.0364441	+	C	T	0.3973	0.0375
rs7634389	LC_IGP111	IgG2 SA per Gal	3	186738421	3	7.56E-24	1.40E-39	-	-0.372176	-0.5037	+	C	T	0.364876161	0.0363768	+	C	T	0.3971	0.0383
rs7634389	LC_IGP93	IgG2_G1FS1	3	186738421	3	6.74E-16	5.28E-29	-	-0.299379	-0.4183	+	C	T	0.364245882	0.0366998	+	C	T	0.3973	0.0374
rs7634389	LC_IGP_R74	IgG2_G1FS1/IgG2_G1F	3	186738421	3	1.00E-32	3.43E-45	-	-0.435085	-0.5395	+	C	T	0.364245882	0.0357247	+	C	T	0.3973	0.0382
rs7634389	LC_IGP_R75	IgG2_G2FS1/IgG2_G2F	3	186738421	3	6.60E-20	1.85E-41	-	-0.336745	-0.5155	+	C	T	0.364245882	0.0363929	+	C	T	0.3973	0.0382
rs7634389	LC_IGP189	IgG4 FG1S1/(FG1+FG1S1)	3	186738421	3	1.57E-19	1.25E-33	-	-0.33256	-0.4535	+	C	T	0.364127744	0.036326	+	C	T	0.3973	0.0375
rs7634389	LC_IGP190	IgG4 FG2S1/(FG2+FG2S1)	3	186738421	3	4.95E-19	1.07E-31	-	-0.32957	-0.4393	+	C	T	0.364349908	0.0365218	+	C	T	0.3973	0.0375
rs7634389	LC_IGP187	IgG4 FGS1/(FG+FGS1)	3	186738421	3	7.06E-13	3.58E-15	-	-0.266064	-0.2869	+	C	T	0.364349908	0.0367678	+	C	T	0.3973	0.0365
rs7634389	LC_IGP186	IgG4 SA per Gal	3	186738421	3	6.30E-19	1.10E-25	-	-0.328905	-0.392	+	C	T	0.364550796	0.0365584	+	C	T	0.3965	0.0374
rs7634389	LC_IGP179	IgG4_G1FS1	3	186738421	3	3.10E-15	1.98E-24	-	-0.290909	-0.3821	+	C	T	0.364127744	0.0365293	+	C	T	0.3973	0.0375
rs7634389	LC_IGP_R92	IgG4_G1FS1/IgG4_G1F	3	186738421	3	1.27E-19	1.41E-33	-	-0.333367	-0.4532	+	C	T	0.364127744	0.036317	+	C	T	0.3973	0.0375
rs7634389	LC_IGP_R93	IgG4_G2FS1/IgG4_G2F	3	186738421	3	5.07E-19	1.14E-31	-	-0.329467	-0.4391	+	C	T	0.364349908	0.0365213	+	C	T	0.3973	0.0375
rs62292644	LC_IGP36	IgG1 FG1S1/(FG1+FG1S1)	3	186738896	3	4.85E-10	7.40E-21	-	-0.390286	-0.5867	+	C	A	0.125629281	0.0623275	+	C	A	0.1672	0.0626
rs62292644	LC_IGP37	IgG1 FG2S1/(FG2+FG2S1)	3	186738896	3	3.30E-13	1.22E-16	-	-0.458913	-0.5004	+	C	A	0.125405525	0.062505	+	C	A	0.1672	0.0604
rs62292644	LC_IGP7	IgG1_G1FS1	3	186738896	3	9.39E-10	1.74E-17	-	-0.383851	-0.5403	+	C	A	0.125629281	0.0623579	+	C	A	0.1672	0.0635
rs62292644	LC_IGP_R34	IgG1_G1FS1/IgG1_G1F	3	186738896	3	4.80E-10	1.13E-20	-	-0.390287	-0.5852	+	C	A	0.125629281	0.0623087	+	C	A	0.1672	0.0628
rs62292644	LC_IGP_R35	IgG1_G2FS1/IgG1_G2F	3	186738896	3	3.10E-13	1.24E-16	-	-0.459446	-0.5004	+	C	A	0.125405525	0.0625025	+	C	A	0.1672	0.0604
rs62292644	LC_IGP122	IgG2 FG1S1/(FG1+FG1S1)	3	186738896	3	2.52E-14	9.54E-26	-	-0.47993	-0.6598	+	C	A	0.125241305	0.0624087	+	C	A	0.1672	0.0629
rs56343190	LC_IGP123	IgG2 FG2S1/(FG2+FG2S1)	3	186768043	4	2.17E-09	3.57E-18	-	-0.266009	-0.3981	+	A	G	0.198620612	0.0442008	+	A	G	0.2117	0.0458
rs62292644	LC_IGP120	IgG2 FGS1/(FG+FGS1)	3	186738896	3	5.61E-10	6.80E-19	-	-0.392278	-0.5458	+	C	A	0.125317765	0.0628785	+	C	A	0.1672	0.0615
rs62292644	LC_IGP111	IgG2 SA per Gal	3	186738896	3	4.26E-11	2.17E-23	-	-0.418755	-0.6235	+	C	A	0.125104597	0.0630609	+	C	A	0.1672	0.0626
rs62292644	LC_IGP93	IgG2_G1FS1	3	186738896	3	1.44E-08	3.68E-17	-	-0.359659	-0.5335	+	C	A	0.125241305	0.0631275	+	C	A	0.1672	0.0633
rs62292644	LC_IGP_R74	IgG2_G1FS1/IgG2_G1F	3	186738896	3	2.55E-14	9.25E-26	-	-0.479843	-0.66	+	C	A	0.125241305	0.0624118	+	C	A	0.1672	0.0629
rs62292644	LC_IGP_R75	IgG2_G2FS1/IgG2_G2F	3	186738896	3	6.94E-09	8.84E-25	-	-0.366396	-0.635	+	C	A	0.125241305	0.0629219	+	C	A	0.1672	0.0618
rs62292644	LC_IGP189	IgG4 FG1S1/(FG1+FG1S1)	3	186738896	3	4.03E-09	7.18E-19	-	-0.371295	-0.5479	+	C	A	0.125252591	0.0627729	+	C	A	0.1672	0.0618
rs62292644	LC_IGP190	IgG4 FG2S1/(FG2+FG2S1)	3	186738896	3	4.73E-08	1.53E-17	-	-0.346488	-0.5254	+	C	A	0.125329011	0.0631451	+	C	A	0.1672	0.0616
rs62292644	LC_IGP179	IgG4_G1FS1	3	186738896	3	4.52E-11	4.35E-15	-	-0.414972	-0.4953	+	C	A	0.125252591	0.0625816	+	C	A	0.1672	0.0632
rs62292644	LC_IGP_R92	IgG4_G1FS1/IgG4_G1F	3	186738896	3	2.92E-09	7.32E-19	-	-0.374583	-0.5479	+	C	A	0.125252591	0.0627536	+	C	A	0.1672	0.0618
rs62292644	LC_IGP_R93	IgG4_G2FS1/IgG4_G2F	3	186738896	3	4.84E-08	1.56E-17	-	-0.346205	-0.5252	+	C	A	0.125329011	0.0631443	+	C	A	0.1672	0.0616
rs77397204	LC_IGP36	IgG1 FG1S1/(FG1+FG1S1)	3	186738899	3	4.86E-10	9.37E-19	-	-0.390269	-0.5641	+	C	A	0.125628539	0.0623275	+	C	A	0.1609	0.0638
rs77397204	LC_IGP37	IgG1 FG2S1/(FG2+FG2S1)	3	186738899	3	3.31E-13	5.24E-15	-	-0.458894	-0.4829	+	C	A	0.125404784	0.0625051	+	C	A	0.1609	0.0617
rs77397204	LC_IGP7	IgG1_G1FS1	3	186738899	3	9.40E-10	7.71E-16	-	-0.383845	-0.5234	+	C	A	0.125628539	0.0623579	+	C	A	0.1609	0.0649
rs77397204	LC_IGP_R34	IgG1_G1FS1/IgG1_G1F	3	186738899	3	4.80E-10	1.48E-18	-	-0.39027	-0.5621	+	C	A	0.125628539	0.0623088	+	C	A	0.1609	0.0639
rs77397204	LC_IGP_R35	IgG1_G2FS1/IgG1_G2F	3	186738899	3	3.10E-13	5.30E-15	-	-0.459427	-0.4828	+	C	A	0.125404784	0.0625025	+	C	A	0.1609	0.0617
rs77397204	LC_IGP122	IgG2 FG1S1/(FG1+FG1S1)	3	186738899	3	2.52E-14	1.35E-21	-	-0.479925	-0.6114	+	C	A	0.125240565	0.0624088	+	C	A	0.1609	0.064
rs1009640	LC_IGP123	IgG2 FG2S1/(FG2+FG2S1)	3	186735096	3	9.79E-12	4.00E-18	-	-0.32692	-0.4544	+	T	C	0.838573166	0.0476616	+	T	C	0.8507	0.0524
rs77397204	LC_IGP120	IgG2 FGS1/(FG+FGS1)	3	186738899	3	5.61E-10	6.90E-16	-	-0.392276	-0.5061	+	C	A	0.125317024	0.0628786	+	C	A	0.1609	0.0627
rs77397204	LC_IGP111	IgG2 SA per Gal	3	186738899	3	4.26E-11	5.76E-20	-	-0.418755	-0.5826	+	C	A	0.125104048	0.0630607	+	C	A	0.161	0.0637
rs77397204	LC_IGP93	IgG2_G1FS1	3	186738899	3	1.44E-08	1.31E-13	-	-0.359658	-0.4786	+	C	A	0.125240565	0.0631275	+	C	A	0.1609	0.0646
rs77397204	LC_IGP_R74	IgG2_G1FS1/IgG2_G1F	3	186738899	3	2.56E-14	1.32E-21	-	-0.479839	-0.6115	+	C	A	0.125240565	0.0624118	+	C	A	0.1609	0.064
rs77397204	LC_IGP_R75	IgG2_G2FS1/IgG2_G2F	3	186738899	3	6.95E-09	8.51E-22	-	-0.366386	-0.6029	+	C	A	0.125240565	0.0629219	+	C	A	0.1609	0.0628
rs77397204	LC_IGP189	IgG4 FG1S1/(FG1+FG1S1)	3	186738899	3	4.04E-09	1.96E-17	-	-0.371277	-0.5331	+	C	A	0.125251852	0.062773	+	C	A	0.1609	0.0628
rs77397204	LC_IGP190	IgG4 FG2S1/(FG2+FG2S1)	3	186738899	3	4.73E-08	9.53E-16	-	-0.346481	-0.5026	+	C	A	0.125328271	0.0631451	+	C	A	0.1609	0.0626
rs77397204	LC_IGP179	IgG4_G1FS1	3	186738899	3	4.52E-11	1.88E-14	-	-0.414964	-0.4942	+	C	A	0.125251852	0.0625817	+	C	A	0.1609	0.0645
rs77397204	LC_IGP_R92	IgG4_G1FS1/IgG4_G1F	3	186738899	3	2.92E-09	1.99E-17	-	-0.374566	-0.533	+	C	A	0.125251852	0.0627536	+	C	A	0.1609	0.0628
rs77397204	LC_IGP_R93	IgG4_G2FS1/IgG4_G2F	3	186738899	3	4.85E-08	9.68E-16	-	-0.346198	-0.5024	+	C	A	0.125328271	0.0631443	+	C	A	0.1609	0.0626
rs77530527	LC_IGP36	IgG1 FG1S1/(FG1+FG1S1)	3	186738900	3	4.86E-10	9.90E-19	-	-0.390266	-0.5666	+	C	T	0.125628364	0.0623275	+	C	T	0.1594	0.0641
rs77530527	LC_IGP37	IgG1 FG2S1/(FG2+FG2S1)	3	186738900	3	3.31E-13	9.29E-15	-	-0.458891	-0.4805	+	C	T	0.125404609	0.0625051	+	C	T	0.1594	0.062
rs77530527	LC_IGP7	IgG1_G1FS1	3	186738900	3	9.40E-10	7.85E-16	-	-0.383842	-0.526	+	C	T	0.125628364	0.0623579	+	C	T	0.1594	0.0653
rs77530527	LC_IGP_R34	IgG1_G1FS1/IgG1_G1F	3	186738900	3	4.81E-10	1.57E-18	-	-0.390267	-0.5646	+	C	T	0.125628364	0.0623088	+	C	T	0.1594	0.0643
rs77530527	LC_IGP_R35	IgG1_G2FS1/IgG1_G2F	3	186738900	3	3.11E-13	9.44E-15	-	-0.459423	-0.4804	+	C	T	0.125404609	0.0625026	+	C	T	0.1594	0.062
rs77530527	LC_IGP122	IgG2 FG1S1/(FG1+FG1S1)	3	186738900	3	2.52E-14	2.13E-21	-	-0.479921	-0.6111	+	C	T	0.12524039	0.0624088	+	C	T	0.1594	0.0643
rs257102	LC_IGP123	IgG2 FG2S1/(FG2+FG2S1)	3	186775948	4	3.76E-09	4.81E-18	+	0.262298	0.3951	+	A	G	0.799692411	0.0442574	+	A	G	0.7864	0.0456
rs77530527	LC_IGP120	IgG2 FGS1/(FG+FGS1)	3	186738900	3	5.61E-10	1.19E-15	-	-0.392271	-0.5039	+	C	T	0.125316849	0.0628786	+	C	T	0.1594	0.0629
rs77530527	LC_IGP111	IgG2 SA per Gal	3	186738900	3	4.26E-11	9.62E-20	-	-0.418749	-0.5814	+	C	T	0.125104102	0.0630607	+	C	T	0.1594	0.0639
rs77530527	LC_IGP93	IgG2_G1FS1	3	186738900	3	1.44E-08	2.04E-13	-	-0.359653	-0.4768	+	C	T	0.12524039	0.0631275	+	C	T	0.1594	0.0649
rs77530527	LC_IGP_R74	IgG2_G1FS1/IgG2_G1F	3	186738900	3	2.56E-14	2.09E-21	-	-0.479834	-0.6113	+	C	T	0.12524039	0.0624118	+	C	T	0.1594	0.0643
rs77530527	LC_IGP_R75	IgG2_G2FS1/IgG2_G2F	3	186738900	3	6.95E-09	1.61E-21	-	-0.366384	-0.6011	+	C	T	0.12524039	0.0629219	+	C	T	0.1594	0.0631
rs77530527	LC_IGP189	IgG4 FG1S1/(FG1+FG1S1)	3	186738900	3	4.04E-09	2.64E-17	-	-0.371275	-0.5335	+	C	T	0.125251677	0.062773	+	C	T	0.1594	0.063
rs77530527	LC_IGP190	IgG4 FG2S1/(FG2+FG2S1)	3	186738900	3	4.73E-08	1.54E-15	-	-0.346478	-0.5016	+	C	T	0.125328096	0.0631451	+	C	T	0.1594	0.0629
rs77530527	LC_IGP179	IgG4_G1FS1	3	186738900	3	4.52E-11	2.05E-14	-	-0.414963	-0.4957	+	C	T	0.125251677	0.0625817	+	C	T	0.1594	0.0648
rs77530527	LC_IGP_R92	IgG4_G1FS1/IgG4_G1F	3	186738900	3	2.92E-09	2.68E-17	-	-0.374564	-0.5334	+	C	T	0.125251677	0.0627536	+	C	T	0.1594	0.063
rs77530527	LC_IGP_R93	IgG4_G2FS1/IgG4_G2F	3	186738900	3	4.85E-08	1.56E-15	-	-0.346195	-0.5014	+	C	T	0.125328096	0.0631443	+	C	T	0.1594	0.0629

rs4686837	LC_IGP36	IgG1 FG1S1/(FG1+FG1S1)	3	186739677	3	1.46E-10	7.36E-18	-	-0.254627	-0.344	+	A	G	0.300196942	0.039468	+	A	G	0.3189	0.04
rs4686837	LC_IGP37	IgG1 FG2S1/(FG2+FG2S1)	3	186739677	3	2.12E-14	6.06E-10	-	-0.304462	-0.24	+	A	G	0.300264652	0.0394731	+	A	G	0.3189	0.0388
rs4686837	LC_IGP7	IgG1_G1FS1	3	186739677	3	5.83E-11	3.99E-17	-	-0.26007	-0.3334	+	A	G	0.300196942	0.03945	+	A	G	0.3189	0.0396
rs4686837	LC_IGP_R34	IgG1_G1FS1/IgG1_G1F	3	186739677	3	1.57E-10	1.25E-17	-	-0.254111	-0.3421	+	A	G	0.300196942	0.0394582	+	A	G	0.3189	0.04
rs4686837	LC_IGP_R35	IgG1_G2FS1/IgG1_G2F	3	186739677	3	1.88E-14	6.11E-10	-	-0.305067	-0.2399	+	A	G	0.300264652	0.0394701	+	A	G	0.3189	0.0388
rs4686837	LC_IGP122	IgG2 FG1S1/(FG1+FG1S1)	3	186739677	3	1.27E-16	4.88E-21	-	-0.329135	-0.3733	+	A	G	0.299827029	0.0393459	+	A	G	0.3189	0.0397
rs7643068	LC_IGP123	IgG2 FG2S1/(FG2+FG2S1)	3	186727083	3	1.06E-11	5.51E-18	-	-0.32596	-0.4453	+	G	A	0.840756559	0.0476021	+	G	A	0.8473	0.0515
rs4686837	LC_IGP120	IgG2 FGS1/(FG+FGS1)	3	186739677	3	1.16E-09	6.65E-15	-	-0.243515	-0.303	+	A	G	0.299704823	0.039786	+	A	G	0.3189	0.0389
rs4686837	LC_IGP111	IgG2 SA per Gal	3	186739677	3	1.49E-11	1.07E-18	-	-0.270734	-0.3506	+	A	G	0.300703592	0.0398235	+	A	G	0.3188	0.0397
rs4686837	LC_IGP_R74	IgG2_G1FS1/IgG2_G1F	3	186739677	3	1.23E-16	4.92E-21	-	-0.329295	-0.3733	+	A	G	0.299827029	0.0393467	+	A	G	0.3189	0.0397
rs4686837	LC_IGP_R75	IgG2_G2FS1/IgG2_G2F	3	186739677	3	5.46E-10	1.63E-20	-	-0.248032	-0.368	+	A	G	0.299521965	0.0397293	+	A	G	0.3189	0.0396
rs4686837	LC_IGP189	IgG4 FG1S1/(FG1+FG1S1)	3	186739677	3	2.39E-12	4.60E-15	-	-0.279138	-0.3053	+	A	G	0.299898476	0.0395191	+	A	G	0.3189	0.039
rs4686837	LC_IGP190	IgG4 FG2S1/(FG2+FG2S1)	3	186739677	3	2.32E-11	1.84E-12	-	-0.267613	-0.2738	+	A	G	0.300081452	0.0397558	+	A	G	0.3189	0.0388
rs4686837	LC_IGP186	IgG4 SA per Gal	3	186739677	3	9.44E-13	9.02E-10	-	-0.285452	-0.238	+	A	G	0.300385863	0.0396698	+	A	G	0.3179	0.0388
rs4686837	LC_IGP179	IgG4_G1FS1	3	186739677	3	6.59E-12	1.42E-13	-	-0.273421	-0.2871	+	A	G	0.299898476	0.0395283	+	A	G	0.3189	0.0388
rs4686837	LC_IGP_R92	IgG4_G1FS1/IgG4_G1F	3	186739677	3	1.72E-12	5.80E-15	-	-0.280929	-0.3042	+	A	G	0.299898476	0.0395066	+	A	G	0.3189	0.039
rs4686837	LC_IGP_R93	IgG4_G2FS1/IgG4_G2F	3	186739677	3	2.35E-11	1.89E-12	-	-0.267518	-0.2737	+	A	G	0.300081452	0.039755	+	A	G	0.3189	0.0389
rs3872723	LC_IGP36	IgG1 FG1S1/(FG1+FG1S1)	3	186741131	3	6.27E-13	1.30E-22	-	-0.263546	-0.3822	+	T	C	0.352474924	0.0363363	+	T	C	0.3749	0.0391
rs3872723	LC_IGP37	IgG1 FG2S1/(FG2+FG2S1)	3	186741131	3	1.82E-16	3.44E-15	-	-0.302603	-0.2988	+	T	C	0.352439866	0.0363667	+	T	C	0.3749	0.038
rs3872723	LC_IGP25	IgG1 SA per Gal	3	186741131	3	4.81E-09	9.74E-11	-	-0.218248	-0.2415	+	T	C	0.350128133	0.0370776	+	T	C	0.3751	0.0373
rs3872723	LC_IGP7	IgG1_G1FS1	3	186741131	3	2.41E-14	1.15E-20	-	-0.279137	-0.3618	+	T	C	0.352474924	0.0362685	+	T	C	0.3749	0.0388
rs3872723	LC_IGP_R34	IgG1_G1FS1/IgG1_G1F	3	186741131	3	6.98E-13	1.53E-22	-	-0.262942	-0.3822	+	T	C	0.352474924	0.036328	+	T	C	0.3749	0.0391
rs3872723	LC_IGP_R35	IgG1_G2FS1/IgG1_G2F	3	186741131	3	1.64E-16	3.38E-15	-	-0.303051	-0.2989	+	T	C	0.352439866	0.0363643	+	T	C	0.3749	0.038
rs3872723	LC_IGP122	IgG2 FG1S1/(FG1+FG1S1)	3	186741131	3	8.46E-22	2.16E-28	-	-0.351155	-0.4292	+	T	C	0.351920073	0.0360817	+	T	C	0.3749	0.0388
rs28674898	LC_IGP123	IgG2 FG2S1/(FG2+FG2S1)	3	186744563	3	5.24E-09	5.62E-18	-	-0.211623	-0.3398	+	A	G	0.382915497	0.0360467	+	A	G	0.3918	0.0393
rs3872723	LC_IGP120	IgG2 FGS1/(FG+FGS1)	3	186741131	3	9.84E-12	2.04E-19	-	-0.251302	-0.3431	+	T	C	0.35182967	0.0366415	+	T	C	0.3749	0.0381
rs3872723	LC_IGP111	IgG2 SA per Gal	3	186741131	3	1.50E-15	1.66E-25	-	-0.29499	-0.4056	+	T	C	0.352507121	0.0366111	+	T	C	0.3749	0.0389
rs3872723	LC_IGP93	IgG2_G1FS1	3	186741131	3	5.82E-11	1.54E-18	-	-0.242317	-0.3338	+	T	C	0.351920073	0.0367572	+	T	C	0.3749	0.038
rs3872723	LC_IGP_R74	IgG2_G1FS1/IgG2_G1F	3	186741131	3	7.98E-22	2.17E-28	-	-0.351383	-0.4292	+	T	C	0.351920073	0.0360819	+	T	C	0.3749	0.0388
rs3872723	LC_IGP_R75	IgG2_G2FS1/IgG2_G2F	3	186741131	3	1.11E-11	2.85E-26	-	-0.250427	-0.4116	+	T	C	0.351615009	0.0366067	+	T	C	0.3749	0.0388
rs3872723	LC_IGP189	IgG4 FG1S1/(FG1+FG1S1)	3	186741131	3	8.12E-14	5.40E-20	-	-0.27449	-0.3486	+	T	C	0.352010061	0.0364358	+	T	C	0.3749	0.0381
rs3872723	LC_IGP190	IgG4 FG2S1/(FG2+FG2S1)	3	186741131	3	6.00E-14	2.16E-19	-	-0.277219	-0.3429	+	T	C	0.352224832	0.0365994	+	T	C	0.3749	0.0381
rs3872723	LC_IGP187	IgG4 FGS1/(FG+FGS1)	3	186741131	3	1.27E-08	3.35E-09	-	-0.21048	-0.2194	+	T	C	0.352224832	0.0368018	+	T	C	0.3749	0.0371
rs3872723	LC_IGP186	IgG4 SA per Gal	3	186741131	3	1.07E-13	6.59E-16	-	-0.274313	-0.3073	+	T	C	0.352384639	0.036593	+	T	C	0.3743	0.038
rs3872723	LC_IGP179	IgG4_G1FS1	3	186741131	3	2.96E-12	4.60E-18	-	-0.256706	-0.3284	+	T	C	0.352010061	0.0365008	+	T	C	0.3749	0.0379
rs3872723	LC_IGP_R92	IgG4_G1FS1/IgG4_G1F	3	186741131	3	6.64E-14	5.98E-20	-	-0.275414	-0.3482	+	T	C	0.352010061	0.0364272	+	T	C	0.3749	0.0381
rs3872723	LC_IGP_R93	IgG4_G2FS1/IgG4_G2F	3	186741131	3	6.29E-14	2.26E-19	-	-0.276986	-0.3428	+	T	C	0.352224832	0.0365994	+	T	C	0.3749	0.0381
rs3872724	LC_IGP36	IgG1 FG1S1/(FG1+FG1S1)	3	186741221	3	2.12E-12	3.10E-21	-	-0.255307	-0.3681	+	T	C	0.357492355	0.036055	+	T	C	0.381	0.0389
rs3872724	LC_IGP37	IgG1 FG2S1/(FG2+FG2S1)	3	186741221	3	6.58E-16	2.12E-14	-	-0.294498	-0.2891	+	T	C	0.357448107	0.0360877	+	T	C	0.381	0.0378
rs3872724	LC_IGP25	IgG1 SA per Gal	3	186741221	3	9.56E-09	2.85E-10	-	-0.212065	-0.2346	+	T	C	0.354949875	0.0367586	+	T	C	0.3812	0.0372
rs3872724	LC_IGP7	IgG1_G1FS1	3	186741221	3	4.36E-13	6.01E-20	-	-0.263109	-0.3536	+	T	C	0.357492355	0.0360241	+	T	C	0.381	0.0387
rs3872724	LC_IGP_R34	IgG1_G1FS1/IgG1_G1F	3	186741221	3	2.38E-12	3.68E-21	-	-0.25464	-0.3681	+	T	C	0.357492355	0.036047	+	T	C	0.381	0.039
rs3872724	LC_IGP_R35	IgG1_G2FS1/IgG1_G2F	3	186741221	3	6.00E-16	2.09E-14	-	-0.294895	-0.2892	+	T	C	0.357448107	0.0360856	+	T	C	0.381	0.0378
rs3872724	LC_IGP122	IgG2 FG1S1/(FG1+FG1S1)	3	186741221	3	4.20E-21	3.29E-26	-	-0.342302	-0.4096	+	T	C	0.356924954	0.0358114	+	T	C	0.381	0.0387
rs28679865	LC_IGP123	IgG2 FG2S1/(FG2+FG2S1)	3	186768348	4	7.29E-09	6.29E-16	-	-0.279905	-0.4276	+	C	G	0.170329019	0.0481368	+	C	G	0.1657	0.0529
rs3872724	LC_IGP120	IgG2 FGS1/(FG+FGS1)	3	186741221	3	2.63E-11	5.04E-18	-	-0.244014	-0.3282	+	T	C	0.356837607	0.036353	+	T	C	0.381	0.0379
rs3872724	LC_IGP111	IgG2 SA per Gal	3	186741221	3	1.04E-14	1.65E-23	-	-0.283735	-0.3867	+	T	C	0.357585139	0.0363416	+	T	C	0.381	0.0387
rs3872724	LC_IGP93	IgG2_G1FS1	3	186741221	3	2.65E-10	1.15E-17	-	-0.231932	-0.3238	+	T	C	0.356924954	0.0364795	+	T	C	0.381	0.0378
rs3872724	LC_IGP_R74	IgG2_G1FS1/IgG2_G1F	3	186741221	3	3.95E-21	3.33E-26	-	-0.342542	-0.4096	+	T	C	0.356924954	0.0358115	+	T	C	0.381	0.0387
rs3872724	LC_IGP_R75	IgG2_G2FS1/IgG2_G2F	3	186741221	3	1.33E-10	2.89E-24	-	-0.235036	-0.3929	+	T	C	0.35661989	0.0363509	+	T	C	0.381	0.0387
rs3872724	LC_IGP189	IgG4 FG1S1/(FG1+FG1S1)	3	186741221	3	1.27E-13	7.54E-19	-	-0.270047	-0.3363	+	T	C	0.357012195	0.0361377	+	T	C	0.381	0.0379
rs3872724	LC_IGP190	IgG4 FG2S1/(FG2+FG2S1)	3	186741221	3	1.02E-13	8.19E-19	-	-0.272366	-0.336	+	T	C	0.357230018	0.0363019	+	T	C	0.381	0.0379
rs3872724	LC_IGP187	IgG4 FGS1/(FG+FGS1)	3	186741221	3	1.09E-08	6.80E-09	-	-0.209643	-0.2142	+	T	C	0.357230018	0.0364878	+	T	C	0.381	0.037
rs3872724	LC_IGP186	IgG4 SA per Gal	3	186741221	3	1.05E-13	2.83E-15	-	-0.272375	-0.2992	+	T	C	0.357099143	0.0363221	+	T	C	0.3804	0.0379
rs3872724	LC_IGP179	IgG4_G1FS1	3	186741221	3	1.64E-11	4.25E-17	-	-0.245747	-0.3175	+	T	C	0.357012195	0.0362295	+	T	C	0.381	0.0378
rs3872724	LC_IGP_R92	IgG4_G1FS1/IgG4_G1F	3	186741221	3	1.08E-13	8.50E-19	-	-0.270795	-0.3359	+	T	C	0.357012195	0.03613	+	T	C	0.381	0.0379
rs3872724	LC_IGP_R93	IgG4_G2FS1/IgG4_G2F	3	186741221	3	1.06E-13	8.57E-19	-	-0.272175	-0.3358	+	T	C	0.357230018	0.0363018	+	T	C	0.381	0.0379
rs4012172	LC_IGP36	IgG1 FG1S1/(FG1+FG1S1)	3	186741511	3	7.11E-13	1.50E-22	-	-0.263036	-0.382	+	T	C	0.352785933	0.0363542	+	T	C	0.3739	0.0391
rs4012172	LC_IGP37	IgG1 FG2S1/(FG2+FG2S1)	3	186741511	3	1.97E-16	4.46E-15	-	-0.302372	-0.2979	+	T	C	0.352750305	0.0363836	+	T	C	0.3739	0.038
rs4012172	LC_IGP25	IgG1 SA per Gal	3	186741511	3	5.00E-09	1.11E-10	-	-0.218079	-0.2409	+	T	C	0.350381266	0.0370898	+	T	C	0.3741	0.0373
rs4012172	LC_IGP7	IgG1_G1FS1	3	186741511	3	2.83E-14	1.09E-20	-	-0.278503	-0.3625	+	T	C	0.352785933	0.0362871	+	T	C	0.3739	0.0389
rs4012172	LC_IGP_R34	IgG1_G1FS1/IgG1_G1F	3	186741511	3	7.92E-13	1.75E-22	-	-0.26243	-0.3821	+	T	C	0.352785933	0.0363459	+	T	C	0.3739	0.0392
rs4012172	LC_IGP_R35	IgG1_G2FS1/IgG1_G2F	3	186741511	3	1.78E-16	4.39E-15	-	-0.302819	-0.298	+	T	C	0.352750305	0.0363812	+	T	C	0.3739	0.038
rs4012172	LC_IGP122	IgG2 FG1S1/(FG1+FG1S1)	3	186741511	3	9.20E-22	1.95E-28	-	-0.351005	-0.4296	+	T	C	0.352244966	0.0361001	+	T	C	0.3739	0.0388
rs4686829	LC_IGP123	IgG2 FG2S1/(FG2+FG2S1)	3	186728319	3	8.69E-12	1.07E-15	-	-0.328058	-0.4092	+	G	T	0.841707525	0.0477064	+	G	T	0.8418	0.051
rs4012172	LC_IGP120	IgG2 FGS1/(FG+FGS1)	3	186741511	3	1.06E-11	2.04E-19	-	-0.251017	-0.3431	+	T	C	0.352154762	0.03666	+	T	C	0.3739	0.0381
rs4012172	LC_IGP111	IgG2 SA per Gal	3	186741511	3	1.45E-15	1.56E-25	-	-0.295263	-0.406	+	T	C	0.352811455	0.0366263	+	T	C	0.3738	0.0389
rs4012172	LC_IGP93	IgG2_G1FS1	3	186741511	3	6.09E-11	1.23E-18	-	-0.242185	-0.3348	+	T	C	0.352244966	0.0367751	+	T	C	0.3739	0.038
rs4012172	LC_IGP_R74	IgG2_G1FS1/IgG2_G1F	3	186741511	3	8.68E-22	1.96E-28	-	-0.351235	-0.4296	+	T	C	0.352244966	0.0361003	+	T	C	0.3739	0.0388
rs4012172	LC_IGP_R75	IgG2_G2FS1/IgG2_G2F	3	186741511	3	1.18E-11	2.82E-26	-	-0.250203	-0.4117	+	T	C	0.351939902	0.0366249	+	T	C	0.3739	0.0388
rs4012172	LC_IGP189	IgG4 FG1S1/(FG1+FG1S1)	3	186741511	3	7.94E-14	5.76E-20	-	-0.274711	-0.3488	+	T	C	0.352320122	0.0364504	+	T	C	0.3739	0.0381
rs4012172	LC_IGP190	IgG4 FG2S1/(FG2+FG2S1)	3	186741511	3	6.58E-14	2.48E-19	-	-0.276893	-0.3426	+	T	C	0.352535082	0.0366166	+	T	C	0.3739	0.0381
rs4012172	LC_IGP187	IgG4 FGS1/(FG+FGS1)	3	186741511	3	1.29E-08	3.47E-09	-	-0.210451	-0.2193	+	T	C	0.352535082	0.0368175	+	T	C	0.3739	0.0371
rs4012172	LC_IGP186	IgG4 SA per Gal	3	186741511	3	1.13E-13	7.01E-16	-	-0.274156	-0.3072	+	T	C	0.352695838	0.0366095	+	T	C	0.3732	0.0381
rs4012172	LC_IGP179	IgG4_G1FS1	3	186741511	3	3.15E-12	4.00E-18	-	-0.256494	-0.3294	+	T	C	0.352320122	0.0365172	+	T	C	0.3739	0.038
rs4012172	LC_IGP_R92	IgG4_G1FS1/IgG4_G1F	3	186741511	3	6.48E-14	6.36E-20	-	-0.275645	-0.3484	+	T	C	0.352320122	0.0364417	+	T	C	0.3739	0.0381
rs4012172	LC_IGP_R93	IgG4_G2FS1/IgG4_G2F	3	186741511	3	6.90E-14	2.60E-19	-	-0.276661	-0.3424	+	T	C	0.352535082	0.0366166	+	T	C	0.3739	0.0381
rs9941987	LC_IGP36	IgG1 FG1S1/(FG1+FG1S1)	3	186742527	3	1.44E-10	3.33E-15	-	-0.309624	-0.432	+	A	G	0.837196127	0.0479808	+	A	G	0.853	0.0548
rs9941987	LC_IGP37	IgG1 FG2S1/(FG2+FG2S1)	3	186742527	3	3.89E-15	1.84E-09	-	-0.38087	-0.3205	+	A	G	0.837189052	0.048003	+	A	G	0.853	0.0533
rs9941987	LC_IGP25	IgG1 SA per Gal	3	186742527	3	1.18E-09	5.74E-09	-	-0.297294	-0.3045	+	A	G	0.834871552	0.0485769	+	A	G	0.8528	0.0523
rs9941987	LC_IGP7	IgG1_G1FS1	3	186742527	3	8.58E-10	1.20E-13	-	-0.296393	-0.4024	+	A	G	0.837196127	0.0480365	+	A	G	0.853	0.0542
rs9941987	LC_IGP_R34	IgG1_G1FS1/IgG1_G1F	3	186742527	3	1.27E-10	2.61E-15	-	-0.310439	-0.4345	+	A	G	0.837196127	0.0479631	+	A	G	0.853	0.055
rs9941987	LC_IGP_R35	IgG1_G2FS1/IgG1_G2F	3	186742527	3	4.00E-15	1.81E-09	-	-0.380715	-0.3206	+	A	G	0.837189052	0.0480036	+	A	G	0.853	0.0533
rs9941987	LC_IGP122	IgG2 FG1S1/(FG1+FG1S1)	3	186742527	3	3.26E-15	3.16E-17	-	-0.381949	-0.4635	+	A	G	0.837287359	0.048	+	A	G	0.853	0.0549
rs4012171	LC_IGP123	IgG2 FG2S1/(FG2+FG2S1)	3	186711453	2	2.03E-11	1.28E-15	-	-0.279633	-0.3988	+	C	A	0.756786455	0.0414196	+	C	A	0.7998	0.0499
rs9941987	LC_IGP120	IgG2 FGS1/(FG+FGS1)	3	186742527	3	3.41E-10	2.13E-10	-	-0.305818	-0.3412	+	A	G	0.837188023	0.0484065	+	A	G	0.853	0.0537
rs9941987	LC_IGP111	IgG2 SA per Gal	3	186742527	3	4.01E-11	1.22E-14	-	-0.323632	-0.4237	+	A	G	0.837644903	0.0486681	+	A	G	0.8525	0.0549
rs9941987	LC_IGP93	IgG2_G1FS1	3	186742527	3	1.55E-09	4.28E-14	-	-0.294858	-0.4026	+	A	G	0.837287359	0.0485483	+	A	G	0.853	0.0533
rs9941987	LC_IGP_R74	IgG2_G1FS1/IgG2_G1F	3	186742527	3	3.18E-15	3.38E-17	-	-0.382104	-0.4631	+	A	G	0.837287359	0.0480012	+	A	G	0.853	0.0549
rs9941987	LC_IGP_R75	IgG2_G2FS1/IgG2_G2F	3	186742527	3	4.17E-09	1.36E-13	-	-0.286146	-0.4066	+	A	G	0.836989617	0.0484212	+	A	G	0.853	0.0549
rs4686838	LC_IGP122	IgG2 FG1S1/(FG1+FG1S1)	3	186743053	3	1.70E-12	1.02E-11	-	-0.243542	-0.2558	+	G	A	0.461866992	0.0342448	+	G	A	0.4434	0.0376
rs4686838	LC_IGP111	IgG2 SA per Gal	3	186743053	3	7.23E-10	6.57E-12	-	-0.214514	-0.2582	+	G	A	0.462229102	0.0346085	+	G	A	0.4434	0.0376
rs4686838	LC_IGP_R74	IgG2_G1FS1/IgG2_G1F	3	186743053	3	1.57E-12	1.03E-11	-	-0.243932	-0.2557	+	G	A	0.461866992	0.0342445	+	G	A	0.4434	0.0376
rs28674898	LC_IGP36	IgG1 FG1S1/(FG1+FG1S1)	3	186744563	3	1.97E-10	3.65E-12	-	-0.22904	-0.2748	+	A	G	0.383563303	0.0357618	+	A	G	0.3918	0.0395
rs28674898	LC_IGP37	IgG1 FG2S1/(FG2+FG2S1)	3	186744563	3	8.65E-12	2.29E-10	-	-0.24691	-0.2439	+	A	G	0.383471306	0.035902	+	A	G	0.3918	0.0385
rs28674898	LC_IGP25	IgG1 SA per Gal	3	186744563	3	4.46E-08	6.44E-08	-	-0.200445	-0.2042	+	A	G	0.380322055	0.0364556	+	A	G	0.3917	0.0378
rs28674898	LC_IGP7	IgG1_G1FS1	3	186744563	3	6.04E-13	2.54E-12	-	-0.25869	-0.2752	+	A	G	0.383563303	0.0356412	+	A	G	0.3918	0.0393
rs28674898	LC_IGP_R34	IgG1_G1FS1/IgG1_G1F	3	186744563	3	2.06E-10	4.13E-12	-	-0.228722	-0.2746	+	A	G	0.383563303	0.0357523	+	A	G	0.3918	0.0396
rs28674898	LC_IGP_R35	IgG1_G2FS1/IgG1_G2F	3	186744563	3	7.52E-12	2.25E-10	-	-0.247626	-0.244	+	A	G	0.383471306	0.0358989	+	A	G	0.3918	0.0385
rs28674898	LC_IGP122	IgG2 FG1S1/(FG1+FG1S1)	3	186744563	3	7.55E-18	4.44E-18	-	-0.309886	-0.3405	+	A	G	0.383194936	0.0355952	+	A	G	0.3918	0.0393
rs35832957	LC_IGP123	IgG2 FG2S1/(FG2+FG2S1)	3	186707884	1	1.62E-09	2.27E-15	-	-0.326724	-0.4728	+	T	C	0.152346511	0.0538599	+	T	C	0.1933	0.0596
rs28674898	LC_IGP120	IgG2 FGS1/(FG+FGS1)	3	186744563	3	3.22E-10	2.78E-13	-	-0.227924	-0.2816	+	A	G	0.383123626	0.0360236	+	A	G	0.3918	0.0386
rs28674898	LC_IGP111	IgG2 SA per Gal	3	186744563	3	1.31E-13	8.11E-18	-	-0.269104	-0.3381	+	A	G	0.383704025	0.0360279	+	A	G	0.3917	0.0393
rs28674898	LC_IGP93	IgG2_G1FS1	3	186744563	3	1.43E-09	3.08E-11	-	-0.219922	-0.2562	+	A	G	0.383194936	0.0361319	+	A	G	0.3918	0.0386
rs28674898	LC_IGP_R74	IgG2_G1FS1/IgG2_G1F	3	186744563	3	7.21E-18	4.61E-18	-	-0.310083	-0.3404	+	A	G	0.383194936	0.0355957	+	A	G	0.3918	0.0393
rs28674898	LC_IGP_R75	IgG2_G2FS1/IgG2_G2F	3	186744563	3	5.29E-09	5.50E-18	-	-0.211568	-0.3399	+	A	G	0.382915497	0.036048	+	A	G	0.3918	0.0393
rs28674898	LC_IGP190	IgG4 FG2S1/(FG2+FG2S1)	3	186744563	3	8.60E-10	6.02E-13	-	-0.222765	-0.2781	+	A	G	0.383262965	0.0361061	+	A	G	0.3918	0.0386
rs28674898	LC_IGP_R93	IgG4_G2FS1/IgG4_G2F	3	186744563	3	9.03E-10	6.32E-13	-	-0.222483	-0.2779	+	A	G	0.383262965	0.0361061	+	A	G	0.3918	0.0386
rs57679165	LC_IGP_R35	IgG1_G2FS1/IgG1_G2F	3	186763145	4	1.48E-14	1.98E-14	-	-0.344129	-0.3551	+	G	C	0.185549926	0.044345	+	G	C	0.2028	0.0464
rs6780488	LC_IGP_R34	IgG1_G1FS1/IgG1_G1F	3	186763942	4	3.26E-10	3.42E-19	-	-0.279813	-0.4279	+	A	G	0.186582471	0.0442399	+	A	G	0.2029	0.0478
rs56343190	LC_IGP_R34	IgG1_G1FS1/IgG1_G1F	3	186768043	4	4.03E-08	1.26E-16	-	-0.242177	-0.3817	+	A	G	0.199717419	0.0439043	+	A	G	0.2117	0.0461
rs257102	LC_IGP_R34	IgG1_G1FS1/IgG1_G1F	3	186775948	4	4.19E-08	1.27E-16	+	0.242111	0.3813	+	A	G	0.798591839	0.0439483	+	A	G	0.7864	0.0461
rs57679165	LC_IGP_R74	IgG2_G1FS1/IgG2_G1F	3	186763145	4	3.72E-20	4.60E-23	-	-0.41011	-0.4698	+	G	C	0.185131652	0.0440163	+	G	C	0.2028	0.0475
rs6780488	LC_IGP_R35	IgG1_G2FS1/IgG1_G2F	3	186763942	4	1.71E-14	2.07E-14	-	-0.342996	-0.3547	+	A	G	0.186240935	0.0443077	+	A	G	0.2029	0.0464
rs56343190	LC_IGP_R35	IgG1_G2FS1/IgG1_G2F	3	186768043	4	8.62E-13	5.19E-13	-	-0.316781	-0.3231	+	A	G	0.199351635	0.0439466	+	A	G	0.2117	0.0448
rs28679865	LC_IGP_R35	IgG1_G2FS1/IgG1_G2F	3	186768348	4	1.38E-11	2.35E-11	-	-0.326041	-0.3468	+	C	G	0.171041459	0.0478879	+	C	G	0.1657	0.0519
rs257102	LC_IGP_R35	IgG1_G2FS1/IgG1_G2F	3	186775948	4	2.68E-12	3.78E-13	+	0.310207	0.3241	+	A	G	0.798960719	0.0440192	+	A	G	0.7864	0.0446
rs257101	LC_IGP_R35	IgG1_G2FS1/IgG1_G2F	3	186778828	4	3.76E-11	5.48E-13	+	0.290935	0.3186	+	A	C	0.792970801	0.0436915	+	A	C	0.786	0.0442
rs257100	LC_IGP_R35	IgG1_G2FS1/IgG1_G2F	3	186782999	4	1.87E-09	6.30E-13	+	0.29583	0.3527	+	G	A	0.794616634	0.0489514	+	G	A	0.7805	0.049

rs16861533	LC_IGP_R74	IgG2_G1FS1/IgG2_G1F	3	186754722	4	5.05E-11	1.13E-14	-	-0.350032	-0.4164	+	A	G	0.122297444	0.052922	+	A	G	0.139	0.0539
rs58909335	LC_IGP_R74	IgG2_G1FS1/IgG2_G1F	3	186754838	4	2.36E-11	1.19E-14	-	-0.357395	-0.4149	+	G	T	0.1216944	0.0531149	+	G	T	0.1399	0.0538
rs59688792	LC_IGP_R74	IgG2_G1FS1/IgG2_G1F	3	186755323	4	1.46E-11	6.48E-15	-	-0.357754	-0.4182	+	C	A	0.12368273	0.0526119	+	C	A	0.1398	0.0536
rs2268536	LC_IGP_R74	IgG2_G1FS1/IgG2_G1F	3	186756908	4	3.07E-10	2.47E-12	-	-0.325061	-0.3681	+	G	T	0.129652227	0.0513179	+	G	T	0.1459	0.0526
rs2268534	LC_IGP_R74	IgG2_G1FS1/IgG2_G1F	3	186757452	4	2.48E-10	1.37E-12	-	-0.326764	-0.3731	+	C	T	0.129652227	0.0513113	+	C	T	0.146	0.0526
rs4686842	LC_IGP_R74	IgG2_G1FS1/IgG2_G1F	3	186761377	4	4.10E-11	2.85E-12	-	-0.258221	-0.3044	+	A	G	0.264607849	0.0388558	+	A	G	0.2626	0.0436
rs57679165	LC_IGP_R75	IgG2_G2FS1/IgG2_G2F	3	186763145	4	2.27E-11	9.86E-20	-	-0.300347	-0.4322	+	G	C	0.184826588	0.0445962	+	G	C	0.2028	0.0475
rs6780488	LC_IGP_R74	IgG2_G1FS1/IgG2_G1F	3	186763942	4	5.25E-20	4.97E-23	-	-0.408122	-0.4693	+	A	G	0.185822239	0.0439844	+	A	G	0.2029	0.0475
rs56343190	LC_IGP_R74	IgG2_G1FS1/IgG2_G1F	3	186768043	4	1.30E-17	1.46E-20	-	-0.377314	-0.4255	+	A	G	0.198925676	0.0436653	+	A	G	0.2117	0.0458
rs28679865	LC_IGP_R74	IgG2_G1FS1/IgG2_G1F	3	186768348	4	3.70E-16	3.27E-17	-	-0.391938	-0.4475	+	C	G	0.170634084	0.0476072	+	C	G	0.1657	0.053
rs4686845	LC_IGP_R74	IgG2_G1FS1/IgG2_G1F	3	186771204	4	9.79E-10	2.75E-13	-	-0.248856	-0.3161	+	T	G	0.262618365	0.0404724	+	T	G	0.2562	0.0433
rs7615919	LC_IGP_R74	IgG2_G1FS1/IgG2_G1F	3	186773305	4	4.15E-10	1.48E-11	+	0.243208	0.2901	+	G	A	0.7158072	0.0386873	+	G	A	0.714	0.043
rs7638437	LC_IGP_R74	IgG2_G1FS1/IgG2_G1F	3	186773443	4	1.47E-10	1.99E-13	+	0.254158	0.3164	+	A	G	0.72195363	0.0394066	+	A	G	0.7224	0.043
rs147013120	LC_IGP_R74	IgG2_G1FS1/IgG2_G1F	3	186774366	4	6.78E-10	3.79E-13	+	0.244965	0.3153	+	T	C	0.717797132	0.0394591	+	T	C	0.7008	0.0434
rs142892049	LC_IGP_R74	IgG2_G1FS1/IgG2_G1F	3	186774729	4	1.25E-10	1.74E-13	+	0.256756	0.3145	+	A	G	0.728509762	0.0396527	+	A	G	0.7356	0.0427
rs257102	LC_IGP_R74	IgG2_G1FS1/IgG2_G1F	3	186775948	4	3.65E-17	1.07E-20	+	0.372489	0.4256	+	A	G	0.799387347	0.0437344	+	A	G	0.7864	0.0456
rs257101	LC_IGP_R74	IgG2_G1FS1/IgG2_G1F	3	186778828	4	1.32E-15	1.59E-20	+	0.350602	0.4196	+	A	C	0.7933959	0.0434325	+	A	C	0.786	0.0452
rs257100	LC_IGP_R74	IgG2_G1FS1/IgG2_G1F	3	186782999	4	2.66E-15	1.66E-19	+	0.387672	0.4527	+	G	A	0.794733691	0.04856	+	G	A	0.7805	0.0501
rs57679165	LC_IGP_R92	IgG4_G1FS1/IgG4_G1F	3	186763145	4	1.63E-09	7.79E-16	-	-0.270449	-0.3745	+	G	C	0.185323645	0.0445879	+	G	C	0.2028	0.0465
rs6780488	LC_IGP_R75	IgG2_G2FS1/IgG2_G2F	3	186763942	4	2.75E-11	1.04E-19	-	-0.298802	-0.4318	+	A	G	0.185517175	0.0445598	+	A	G	0.2029	0.0475
rs56343190	LC_IGP_R75	IgG2_G2FS1/IgG2_G2F	3	186768043	4	2.19E-09	3.47E-18	-	-0.265947	-0.3983	+	A	G	0.198620612	0.0442023	+	A	G	0.2117	0.0458
rs28679865	LC_IGP_R75	IgG2_G2FS1/IgG2_G2F	3	186768348	4	7.28E-09	6.24E-16	-	-0.279922	-0.4276	+	C	G	0.170329019	0.0481381	+	C	G	0.1657	0.0529
rs257102	LC_IGP_R75	IgG2_G2FS1/IgG2_G2F	3	186775948	4	3.80E-09	4.68E-18	+	0.262229	0.3953	+	A	G	0.799692411	0.0442589	+	A	G	0.7864	0.0456
rs57679165	LC_IGP_R93	IgG4_G2FS1/IgG4_G2F	3	186763145	4	1.41E-11	4.14E-15	-	-0.303948	-0.3656	+	G	C	0.185436716	0.0446645	+	G	C	0.2028	0.0466
rs6780488	LC_IGP_R92	IgG4_G1FS1/IgG4_G1F	3	186763942	4	3.45E-09	7.88E-16	-	-0.264785	-0.3743	+	A	G	0.186013812	0.0445664	+	A	G	0.2029	0.0465
rs56343190	LC_IGP_R92	IgG4_G1FS1/IgG4_G1F	3	186768043	4	9.93E-09	3.44E-15	-	-0.254237	-0.3542	+	A	G	0.199109257	0.0441256	+	A	G	0.2117	0.045
rs28679865	LC_IGP_R92	IgG4_G1FS1/IgG4_G1F	3	186768348	4	9.27E-09	1.45E-12	-	-0.277141	-0.3721	+	C	G	0.170834716	0.048003	+	C	G	0.1657	0.0526
rs257102	LC_IGP_R92	IgG4_G1FS1/IgG4_G1F	3	186775948	4	1.83E-08	1.62E-15	+	0.249896	0.3569	+	A	G	0.799204977	0.0441841	+	A	G	0.7864	0.0448
rs257101	LC_IGP_R92	IgG4_G1FS1/IgG4_G1F	3	186778828	4	1.54E-08	2.56E-15	+	0.248915	0.3511	+	A	C	0.793216463	0.0437816	+	A	C	0.786	0.0444
rs57679165	LC_IGP111	IgG2 SA per Gal	3	186763145	4	6.42E-14	1.22E-19	-	-0.338034	-0.431	+	G	C	0.185337748	0.0446766	+	G	C	0.2034	0.0475
rs6780488	LC_IGP_R93	IgG4_G2FS1/IgG4_G2F	3	186763942	4	1.45E-11	4.25E-15	-	-0.303481	-0.3654	+	A	G	0.186127304	0.0446238	+	A	G	0.2029	0.0466
rs56343190	LC_IGP_R93	IgG4_G2FS1/IgG4_G2F	3	186768043	4	2.97E-11	7.76E-14	-	-0.295711	-0.3357	+	A	G	0.199230739	0.0441744	+	A	G	0.2117	0.0449
rs28679865	LC_IGP_R93	IgG4_G2FS1/IgG4_G2F	3	186768348	4	2.21E-10	2.07E-11	-	-0.307282	-0.3508	+	C	G	0.170938948	0.0481146	+	C	G	0.1657	0.0524
rs257102	LC_IGP_R93	IgG4_G2FS1/IgG4_G2F	3	186775948	4	5.37E-11	3.90E-14	+	0.292147	0.3383	+	A	G	0.799082466	0.0442327	+	A	G	0.7864	0.0447
rs257101	LC_IGP_R93	IgG4_G2FS1/IgG4_G2F	3	186778828	4	3.76E-10	5.25E-14	+	0.276589	0.3331	+	A	C	0.793090299	0.0438854	+	A	C	0.786	0.0443
rs257100	LC_IGP_R93	IgG4_G2FS1/IgG4_G2F	3	186782999	4	5.13E-10	4.42E-14	+	0.306805	0.3672	+	G	A	0.794709106	0.0490653	+	G	A	0.7805	0.0486
rs16861533	LC_IGP111	IgG2 SA per Gal	3	186754722	4	2.19E-08	1.17E-12	-	-0.300437	-0.3838	+	A	G	0.122257282	0.0534189	+	A	G	0.1392	0.054
rs58909335	LC_IGP111	IgG2 SA per Gal	3	186754838	4	1.19E-08	1.14E-12	-	-0.307317	-0.3831	+	G	T	0.121645277	0.0536211	+	G	T	0.1401	0.0539
rs59688792	LC_IGP111	IgG2 SA per Gal	3	186755323	4	1.21E-08	8.11E-13	-	-0.304315	-0.3844	+	C	A	0.123663155	0.0531225	+	C	A	0.14	0.0537
rs4686842	LC_IGP111	IgG2 SA per Gal	3	186761377	4	3.73E-08	2.29E-10	-	-0.217199	-0.2769	+	A	G	0.264693972	0.0392771	+	A	G	0.2631	0.0437
rs57679165	LC_IGP120	IgG2 FGS1/(FG+FGS1)	3	186763145	4	6.34E-13	9.23E-16	-	-0.322955	-0.3739	+	G	C	0.184939425	0.0445373	+	G	C	0.2028	0.0465
rs6780488	LC_IGP111	IgG2 SA per Gal	3	186763942	4	1.19E-13	1.30E-19	-	-0.334112	-0.4306	+	A	G	0.186061048	0.0446501	+	A	G	0.2035	0.0475
rs56343190	LC_IGP111	IgG2 SA per Gal	3	186768043	4	9.16E-12	5.89E-18	-	-0.304217	-0.3958	+	A	G	0.199565314	0.0442847	+	A	G	0.2124	0.0458
rs28679865	LC_IGP111	IgG2 SA per Gal	3	186768348	4	7.04E-11	2.10E-15	-	-0.316793	-0.4213	+	C	G	0.171110788	0.0482615	+	C	G	0.1662	0.0531
rs257102	LC_IGP111	IgG2 SA per Gal	3	186775948	4	2.44E-11	8.71E-18	+	0.298311	0.3923	+	A	G	0.798776133	0.0443611	+	A	G	0.7856	0.0457
rs257101	LC_IGP111	IgG2 SA per Gal	3	186778828	4	2.17E-10	9.30E-18	+	0.28123	0.3879	+	A	C	0.79269969	0.0440131	+	A	C	0.7852	0.0452
rs257100	LC_IGP111	IgG2 SA per Gal	3	186782999	4	1.58E-10	2.17E-17	+	0.316558	0.4256	+	G	A	0.793979468	0.0491595	+	G	A	0.7796	0.0502
rs57679165	LC_IGP122	IgG2 FG1S1/(FG1+FG1S1)	3	186763145	4	4.15E-20	4.72E-23	-	-0.409587	-0.4696	+	G	C	0.185131652	0.0440174	+	G	C	0.2028	0.0475
rs6780488	LC_IGP120	IgG2 FGS1/(FG+FGS1)	3	186763942	4	1.14E-12	9.84E-16	-	-0.319103	-0.3735	+	A	G	0.185630433	0.0445114	+	A	G	0.2029	0.0465
rs56343190	LC_IGP120	IgG2 FGS1/(FG+FGS1)	3	186768043	4	5.87E-11	3.08E-14	-	-0.290962	-0.3414	+	A	G	0.19874187	0.044144	+	A	G	0.2117	0.0449
rs28679865	LC_IGP120	IgG2 FGS1/(FG+FGS1)	3	186768348	4	6.90E-10	9.34E-12	-	-0.298544	-0.3548	+	C	G	0.170433005	0.0481101	+	C	G	0.1657	0.052
rs257102	LC_IGP120	IgG2 FGS1/(FG+FGS1)	3	186775948	4	1.58E-10	4.88E-14	+	0.284651	0.3373	+	A	G	0.799570123	0.0442124	+	A	G	0.7864	0.0448
rs257101	LC_IGP120	IgG2 FGS1/(FG+FGS1)	3	186778828	4	1.20E-09	4.96E-14	+	0.268261	0.3337	+	A	C	0.793575019	0.0438645	+	A	C	0.786	0.0443
rs257100	LC_IGP120	IgG2 FGS1/(FG+FGS1)	3	186782999	4	1.28E-09	1.58E-13	+	0.299316	0.3623	+	G	A	0.794913627	0.0490286	+	G	A	0.7805	0.0491
rs16861533	LC_IGP122	IgG2 FG1S1/(FG1+FG1S1)	3	186754722	4	5.24E-11	1.15E-14	-	-0.349727	-0.4162	+	A	G	0.122297444	0.052921	+	A	G	0.139	0.0539
rs58909335	LC_IGP122	IgG2 FG1S1/(FG1+FG1S1)	3	186754838	4	2.45E-11	1.22E-14	-	-0.357094	-0.4147	+	G	T	0.1216944	0.0531139	+	G	T	0.1399	0.0538
rs59688792	LC_IGP122	IgG2 FG1S1/(FG1+FG1S1)	3	186755323	4	1.51E-11	6.64E-15	-	-0.357518	-0.418	+	C	A	0.12368273	0.0526107	+	C	A	0.1398	0.0536
rs2268536	LC_IGP122	IgG2 FG1S1/(FG1+FG1S1)	3	186756908	4	3.16E-10	2.50E-12	-	-0.324826	-0.368	+	G	T	0.129652227	0.0513167	+	G	T	0.1459	0.0526
rs2268534	LC_IGP122	IgG2 FG1S1/(FG1+FG1S1)	3	186757452	4	2.55E-10	1.39E-12	-	-0.326523	-0.373	+	C	T	0.129652227	0.0513101	+	C	T	0.146	0.0526
rs4686842	LC_IGP122	IgG2 FG1S1/(FG1+FG1S1)	3	186761377	4	4.42E-11	2.89E-12	-	-0.257782	-0.3043	+	A	G	0.264607849	0.038856	+	A	G	0.2626	0.0436
rs57679165	LC_IGP123	IgG2 FG2S1/(FG2+FG2S1)	3	186763145	4	2.20E-11	1.00E-19	-	-0.300519	-0.4321	+	G	C	0.184826588	0.0445942	+	G	C	0.2028	0.0475
rs6780488	LC_IGP122	IgG2 FG1S1/(FG1+FG1S1)	3	186763942	4	5.74E-20	5.11E-23	-	-0.407689	-0.4691	+	A	G	0.185822239	0.043985	+	A	G	0.2029	0.0475
rs56343190	LC_IGP122	IgG2 FG1S1/(FG1+FG1S1)	3	186768043	4	1.42E-17	1.56E-20	-	-0.376847	-0.4252	+	A	G	0.198925676	0.0436659	+	A	G	0.2117	0.0458
rs28679865	LC_IGP122	IgG2 FG1S1/(FG1+FG1S1)	3	186768348	4	3.73E-16	3.45E-17	-	-0.391862	-0.4472	+	C	G	0.170634084	0.0476055	+	C	G	0.1657	0.053
rs4686845	LC_IGP122	IgG2 FG1S1/(FG1+FG1S1)	3	186771204	4	1.01E-09	2.87E-13	-	-0.248644	-0.3158	+	T	G	0.262618365	0.0404715	+	T	G	0.2562	0.0433
rs7615919	LC_IGP122	IgG2 FG1S1/(FG1+FG1S1)	3	186773305	4	4.38E-10	1.53E-11	+	0.242881	0.2899	+	G	A	0.7158072	0.0386869	+	G	A	0.714	0.043
rs7638437	LC_IGP122	IgG2 FG1S1/(FG1+FG1S1)	3	186773443	4	1.55E-10	2.06E-13	+	0.25383	0.3162	+	A	G	0.72195363	0.0394062	+	A	G	0.7224	0.043
rs147013120	LC_IGP122	IgG2 FG1S1/(FG1+FG1S1)	3	186774366	4	7.14E-10	3.90E-13	+	0.244637	0.3152	+	T	C	0.717797132	0.0394587	+	T	C	0.7008	0.0434
rs142892049	LC_IGP122	IgG2 FG1S1/(FG1+FG1S1)	3	186774729	4	1.33E-10	1.82E-13	+	0.25637	0.3143	+	A	G	0.728509762	0.0396526	+	A	G	0.7356	0.0427
rs257102	LC_IGP122	IgG2 FG1S1/(FG1+FG1S1)	3	186775948	4	3.89E-17	1.15E-20	+	0.372152	0.4253	+	A	G	0.799387347	0.0437343	+	A	G	0.7864	0.0456
rs257101	LC_IGP122	IgG2 FG1S1/(FG1+FG1S1)	3	186778828	4	1.40E-15	1.71E-20	+	0.350287	0.4193	+	A	C	0.7933959	0.0434322	+	A	C	0.786	0.0452
rs257100	LC_IGP122	IgG2 FG1S1/(FG1+FG1S1)	3	186782999	4	2.79E-15	1.76E-19	+	0.387366	0.4523	+	G	A	0.794733691	0.0485594	+	G	A	0.7805	0.0501
rs9941987	LC_IGP123	IgG2 FG2S1/(FG2+FG2S1)	3	186742527	3	3.96E-09	1.29E-13	-	-0.286535	-0.4069	+	A	G	0.836989617	0.0484183	+	A	G	0.853	0.0549
rs13325033	LC_IGP123	IgG2 FG2S1/(FG2+FG2S1)	3	186708571	2	2.87E-09	1.89E-13	-	-0.251259	-0.3439	+	A	G	0.782814083	0.0420737	+	A	G	0.7928	0.0468
rs759602	LC_IGP123	IgG2 FG2S1/(FG2+FG2S1)	3	186708804	2	4.94E-08	1.42E-12	-	-0.228378	-0.3263	+	G	A	0.784014643	0.041682	+	G	A	0.802	0.0461
rs7622133	LC_IGP123	IgG2 FG2S1/(FG2+FG2S1)	3	186707602	1	1.85E-08	1.76E-08	-	-0.21395	-0.2684	+	T	G	0.467437767	0.0378446	+	T	G	0.4521	0.0476
rs7622117	LC_IGP123	IgG2 FG2S1/(FG2+FG2S1)	3	186707554	1	1.46E-08	1.82E-08	-	-0.215313	-0.2675	+	A	G	0.467236405	0.037811	+	A	G	0.4521	0.0475
rs57679165	LC_IGP186	IgG4 SA per Gal	3	186763145	4	4.55E-10	6.55E-13	-	-0.280625	-0.3338	+	G	C	0.185698151	0.0447403	+	G	C	0.203	0.0464
rs6780488	LC_IGP186	IgG4 SA per Gal	3	186763942	4	9.51E-10	6.63E-13	-	-0.275184	-0.3336	+	A	G	0.186390852	0.0447186	+	A	G	0.2032	0.0464
rs56343190	LC_IGP186	IgG4 SA per Gal	3	186768043	4	3.27E-09	1.94E-12	-	-0.263493	-0.3158	+	A	G	0.199525754	0.0442822	+	A	G	0.212	0.0449
rs28679865	LC_IGP186	IgG4 SA per Gal	3	186768348	4	1.29E-08	6.13E-10	-	-0.275666	-0.3243	+	C	G	0.171148651	0.0482172	+	C	G	0.1658	0.0524
rs257102	LC_IGP186	IgG4 SA per Gal	3	186775948	4	8.28E-09	1.25E-12	+	0.256916	0.317	+	A	G	0.798783756	0.0443497	+	A	G	0.786	0.0447
rs257101	LC_IGP186	IgG4 SA per Gal	3	186778828	4	7.52E-09	1.89E-12	+	0.25531	0.3113	+	A	C	0.792780801	0.0439474	+	A	C	0.7856	0.0442
rs57679165	LC_IGP189	IgG4 FG1S1/(FG1+FG1S1)	3	186763145	4	1.29E-09	8.07E-16	-	-0.272176	-0.3742	+	G	C	0.185323645	0.0445867	+	G	C	0.2028	0.0465
rs6780488	LC_IGP189	IgG4 FG1S1/(FG1+FG1S1)	3	186763942	4	2.76E-09	8.17E-16	-	-0.266422	-0.3741	+	A	G	0.186013812	0.0445656	+	A	G	0.2029	0.0465
rs56343190	LC_IGP189	IgG4 FG1S1/(FG1+FG1S1)	3	186768043	4	8.72E-09	3.67E-15	-	-0.25523	-0.3538	+	A	G	0.199109257	0.0441273	+	A	G	0.2117	0.045
rs28679865	LC_IGP189	IgG4 FG1S1/(FG1+FG1S1)	3	186768348	4	8.62E-09	1.49E-12	-	-0.277763	-0.3718	+	C	G	0.170834716	0.0480064	+	C	G	0.1657	0.0526
rs257102	LC_IGP189	IgG4 FG1S1/(FG1+FG1S1)	3	186775948	4	1.59E-08	1.73E-15	+	0.25097	0.3566	+	A	G	0.799204977	0.0441855	+	A	G	0.7864	0.0448
rs257101	LC_IGP189	IgG4 FG1S1/(FG1+FG1S1)	3	186778828	4	1.37E-08	2.74E-15	+	0.249805	0.3507	+	A	C	0.793216463	0.0437836	+	A	C	0.786	0.0444
rs57679165	LC_IGP190	IgG4 FG2S1/(FG2+FG2S1)	3	186763145	4	1.37E-11	4.09E-15	-	-0.304137	-0.3656	+	G	C	0.185436716	0.044665	+	G	C	0.2028	0.0466
rs6780488	LC_IGP190	IgG4 FG2S1/(FG2+FG2S1)	3	186763942	4	1.41E-11	4.19E-15	-	-0.303681	-0.3654	+	A	G	0.186127304	0.0446243	+	A	G	0.2029	0.0466
rs56343190	LC_IGP190	IgG4 FG2S1/(FG2+FG2S1)	3	186768043	4	2.93E-11	7.66E-14	-	-0.295806	-0.3357	+	A	G	0.199230739	0.0441752	+	A	G	0.2117	0.0449
rs28679865	LC_IGP190	IgG4 FG2S1/(FG2+FG2S1)	3	186768348	4	2.12E-10	2.06E-11	-	-0.307594	-0.3508	+	C	G	0.170938948	0.0481148	+	C	G	0.1657	0.0523
rs257102	LC_IGP190	IgG4 FG2S1/(FG2+FG2S1)	3	186775948	4	5.32E-11	3.86E-14	+	0.292213	0.3384	+	A	G	0.799082466	0.0442337	+	A	G	0.7864	0.0447
rs257101	LC_IGP190	IgG4 FG2S1/(FG2+FG2S1)	3	186778828	4	3.72E-10	5.19E-14	+	0.276655	0.3332	+	A	C	0.793090299	0.0438864	+	A	C	0.786	0.0443
rs257100	LC_IGP190	IgG4 FG2S1/(FG2+FG2S1)	3	186782999	4	5.04E-10	4.36E-14	+	0.306949	0.3673	+	G	A	0.794709106	0.0490662	+	G	A	0.7805	0.0486
rs57679165	LC_IGP25	IgG1 SA per Gal	3	186763145	4	1.51E-08	8.66E-12	-	-0.257655	-0.3115	+	G	C	0.18343288	0.0452819	+	G	C	0.203	0.0456
rs6780488	LC_IGP25	IgG1 SA per Gal	3	186763942	4	2.01E-08	9.15E-12	-	-0.25516	-0.3111	+	A	G	0.184158035	0.0452452	+	A	G	0.2031	0.0456
rs57679165	LC_IGP36	IgG1 FG1S1/(FG1+FG1S1)	3	186763145	4	2.35E-10	2.91E-19	-	-0.282402	-0.4282	+	G	C	0.185890192	0.0442851	+	G	C	0.2028	0.0477
rs6780488	LC_IGP36	IgG1 FG1S1/(FG1+FG1S1)	3	186763942	4	3.08E-10	3.07E-19	-	-0.280282	-0.4278	+	A	G	0.186582471	0.0442514	+	A	G	0.2029	0.0477
rs56343190	LC_IGP36	IgG1 FG1S1/(FG1+FG1S1)	3	186768043	4	3.92E-08	1.23E-16	-	-0.242451	-0.3812	+	A	G	0.199717419	0.0439165	+	A	G	0.2117	0.046
rs257102	LC_IGP36	IgG1 FG1S1/(FG1+FG1S1)	3	186775948	4	4.08E-08	1.25E-16	+	0.242373	0.3808	+	A	G	0.798591839	0.0439605	+	A	G	0.7864	0.046
rs57679165	LC_IGP37	IgG1 FG2S1/(FG2+FG2S1)	3	186763145	4	1.46E-14	2.06E-14	-	-0.344232	-0.3548	+	G	C	0.185549926	0.0443446	+	G	C	0.2028	0.0464
rs6780488	LC_IGP37	IgG1 FG2S1/(FG2+FG2S1)	3	186763942	4	1.68E-14	2.16E-14	-	-0.343115	-0.3545	+	A	G	0.186240935	0.0443073	+	A	G	0.2029	0.0464
rs56343190	LC_IGP37	IgG1 FG2S1/(FG2+FG2S1)	3	186768043	4	9.18E-13	5.36E-13	-	-0.316405	-0.3229	+	A	G	0.199351635	0.0439484	+	A	G	0.2117	0.0448
rs28679865	LC_IGP37	IgG1 FG2S1/(FG2+FG2S1)	3	186768348	4	1.44E-11	2.41E-11	-	-0.325731	-0.3466	+	C	G	0.171041459	0.0478893	+	C	G	0.1657	0.0519
rs257102	LC_IGP37	IgG1 FG2S1/(FG2+FG2S1)	3	186775948	4	2.84E-12	3.91E-13	+	0.309863	0.3239	+	A	G	0.798960719	0.0440208	+	A	G	0.7864	0.0446
rs257101	LC_IGP37	IgG1 FG2S1/(FG2+FG2S1)	3	186778828	4	3.93E-11	5.65E-13	+	0.290639	0.3184	+	A	C	0.792970801	0.0436929	+	A	C	0.786	0.0442
rs257100	LC_IGP37	IgG1 FG2S1/(FG2+FG2S1)	3	186782999	4	1.84E-09	6.47E-13	+	0.295933	0.3525	+	G	A	0.794616634	0.0489512	+	G	A	0.7805	0.049
rs57679165	LC_IGP93	IgG2_G1FS1	3	186763145	4	1.21E-10	5.41E-15	-	-0.290035	-0.3637	+	G	C	0.185131652	0.0447555	+	G	C	0.2028	0.0465
rs6780488	LC_IGP93	IgG2_G1FS1	3	186763942	4	9.37E-11	5.82E-15	-	-0.291467	-0.3632	+	A	G	0.185822239	0.0447071	+	A	G	0.2029	0.0465
rs56343190	LC_IGP93	IgG2_G1FS1	3	186768043	4	3.09E-09	1.20E-13	-	-0.264169	-0.3324	+	A	G	0.198925676	0.0443276	+	A	G	0.2117	0.0448
rs28679865	LC_IGP93	IgG2_G1FS1	3	186768348	4	3.87E-08	1.65E-10	-	-0.266781	-0.3364	+	C	G	0.170634084	0.0483043	+	C	G	0.1657	0.0526
rs257102	LC_IGP93	IgG2_G1FS1	3	186775948	4	4.15E-09	1.20E-13	+	0.262281	0.331	+	A	G	0.799387347	0.0443779	+	A	G	0.7864	0.0446
rs257101	LC_IGP93	IgG2_G1FS1	3	186778828	4	4.20E-08	1.67E-13	+	0.242572	0.3256	+	A	C	0.7933959	0.0440368	+	A	C	0.786	0.0442
rs257100	LC_IGP93	IgG2_G1FS1	3	186782999	4	1.06E-08	5.10E-13	+	0.282771	0.3546	+	G	A	0.794733691	0.0491749	+	G	A	0.7805	0.0491
rs9886239	LC_IGP78	IgG1 BG0n/G0n	7	50336551	1	1.58E-08	6.27E-08	-	-0.220371	-0.216	+	C	A	0.288692689	0.0387893	+	C	A	0.3215	0.0399
rs9886239	LC_IGP77	IgG1 Bn	7	50336551	1	8.16E-09	1.00E-07	-	-0.225293	-0.2133	+	C	A	0.288692689	0.0388718	+	C	A	0.3215	0.04
rs9886239	LC_IGP86	IgG1 Bn/Fn total	7	50336551	1	1.71E-08	2.91E-07	-	-0.220378	-0.2054	+	C	A	0.288692689	0.0388806	+	C	A	0.3215	0.04

rs9886239	LC_IGP81	IgG1 Fn/Bn	7	50336551	1	1.49E-08	6.03E-08	+	0.22119	0.2162	+	C	A	0.288692689	0.0388646	+	C	A	0.3215	0.0399
rs1993444	LC_IGP78	IgG1 BG0n/G0n	7	50338499	1	1.38E-08	5.97E-08	-	-0.221024	-0.216	+	A	G	0.288991326	0.0387413	+	A	G	0.3213	0.0398
rs1993444	LC_IGP77	IgG1 Bn	7	50338499	1	6.12E-09	9.44E-08	-	-0.226897	-0.2134	+	A	G	0.288991326	0.0388201	+	A	G	0.3213	0.04
rs1993444	LC_IGP86	IgG1 Bn/Fn total	7	50338499	1	1.31E-08	2.74E-07	-	-0.221877	-0.2055	+	A	G	0.288991326	0.0388295	+	A	G	0.3213	0.04
rs1993444	LC_IGP81	IgG1 Fn/Bn	7	50338499	1	1.12E-08	5.71E-08	+	0.222857	0.2161	+	A	G	0.288991326	0.0388128	+	A	G	0.3213	0.0398
rs11765436	LC_IGP78	IgG1 BG0n/G0n	7	50344077	1	2.00E-08	3.18E-07	-	-0.216758	-0.2033	+	A	T	0.296285936	0.0384279	+	A	T	0.332	0.0398
rs11765436	LC_IGP77	IgG1 Bn	7	50344077	1	8.73E-09	6.05E-07	-	-0.222727	-0.1989	+	A	T	0.296285936	0.0385058	+	A	T	0.332	0.0399
rs11765436	LC_IGP86	IgG1 Bn/Fn total	7	50344077	1	1.66E-08	1.53E-06	-	-0.218499	-0.1918	+	A	T	0.296285936	0.0385123	+	A	T	0.332	0.0399
rs11765436	LC_IGP81	IgG1 Fn/Bn	7	50344077	1	1.64E-08	3.80E-07	+	0.218485	0.2016	+	A	T	0.296285936	0.0384993	+	A	T	0.332	0.0397
rs7782210	LC_IGP56	IgG1_G1Nn	7	50348745	1	2.76E-09	2.38E-13	-	-0.220405	-0.2853	+	G	A	0.357290582	0.0368626	+	G	A	0.4071	0.0389
rs7782210	LC_IGP15	IgG1_G1N	7	50348745	1	2.81E-09	4.27E-13	-	-0.219912	-0.2826	+	G	A	0.356809348	0.0368003	+	G	A	0.4071	0.039
rs7782210	LC_IGP81	IgG1 Fn/Bn	7	50348745	1	2.59E-11	7.88E-13	+	0.246715	0.2789	+	G	A	0.357290582	0.036739	+	G	A	0.4071	0.0389
rs7782210	LC_IGP78	IgG1 BG0n/G0n	7	50348745	1	5.46E-10	9.52E-13	-	-0.229347	-0.2786	+	G	A	0.357290582	0.0367344	+	G	A	0.4071	0.039
rs7782210	LC_IGP79	IgG1 BG1n/G1n	7	50348745	1	2.60E-09	9.90E-13	-	-0.220713	-0.2777	+	G	A	0.357290582	0.0368546	+	G	A	0.4071	0.0389
rs7782210	LC_IGP77	IgG1 Bn	7	50348745	1	3.22E-11	1.03E-12	-	-0.245679	-0.2789	+	G	A	0.357290582	0.0367641	+	G	A	0.4071	0.0391
rs7782210	LC_IGP86	IgG1 Bn/Fn total	7	50348745	1	7.32E-11	4.24E-12	-	-0.241173	-0.2713	+	G	A	0.357290582	0.0367744	+	G	A	0.4071	0.0392
rs7782210	LC_IGP55	IgG1_G0Nn	7	50348745	1	1.03E-09	1.17E-10	-	-0.225866	-0.2507	+	G	A	0.357290582	0.0367806	+	G	A	0.4071	0.0389
rs7782210	LC_IGP14	IgG1_G0N	7	50348745	1	1.20E-08	3.76E-10	-	-0.210226	-0.2433	+	G	A	0.35742131	0.0366898	+	G	A	0.4071	0.0388
rs7782210	LC_IGP_R16	IgG1_G1N/IgG1_G1	7	50348745	1	5.17E-09	2.21E-08	-	-0.215565	-0.2186	+	G	A	0.356940579	0.0367017	+	G	A	0.4071	0.0391
rs6583437	LC_IGP77	IgG1 Bn	7	50350267	1	4.81E-09	1.10E-11	-	-0.220174	-0.2669	+	A	G	0.332713755	0.0374087	+	A	G	0.386	0.0393
rs6583437	LC_IGP86	IgG1 Bn/Fn total	7	50350267	1	9.22E-09	3.19E-11	-	-0.216072	-0.2609	+	A	G	0.332713755	0.0374153	+	A	G	0.386	0.0393
rs6583437	LC_IGP81	IgG1 Fn/Bn	7	50350267	1	5.15E-09	9.80E-12	+	0.219641	0.2662	+	A	G	0.332713755	0.0373897	+	A	G	0.386	0.0391
rs7797255	LC_IGP78	IgG1 BG0n/G0n	7	50351604	1	7.62E-10	3.05E-12	-	-0.226974	-0.2723	+	G	A	0.356907063	0.0366679	+	G	A	0.4016	0.039
rs7797255	LC_IGP79	IgG1 BG1n/G1n	7	50351604	1	5.19E-09	4.01E-12	-	-0.216094	-0.2701	+	G	A	0.356907063	0.0367958	+	G	A	0.4016	0.0389
rs7797255	LC_IGP77	IgG1 Bn	7	50351604	1	6.68E-11	3.86E-12	-	-0.241235	-0.2718	+	G	A	0.356907063	0.0367063	+	G	A	0.4016	0.0392
rs7797255	LC_IGP86	IgG1 Bn/Fn total	7	50351604	1	1.50E-10	1.53E-11	-	-0.23671	-0.2642	+	G	A	0.356907063	0.0367164	+	G	A	0.4016	0.0392
rs7797255	LC_IGP81	IgG1 Fn/Bn	7	50351604	1	5.73E-11	2.62E-12	+	0.241941	0.2725	+	G	A	0.356907063	0.0366827	+	G	A	0.4016	0.039
rs7797255	LC_IGP14	IgG1_G0N	7	50351604	1	1.62E-08	1.07E-09	-	-0.207919	-0.2369	+	G	A	0.357045622	0.0366229	+	G	A	0.4016	0.0388
rs7797255	LC_IGP55	IgG1_G0Nn	7	50351604	1	1.43E-09	3.25E-10	-	-0.223482	-0.2445	+	G	A	0.356907063	0.0367139	+	G	A	0.4016	0.0389
rs7797255	LC_IGP15	IgG1_G1N	7	50351604	1	5.13E-09	1.92E-12	-	-0.215829	-0.2746	+	G	A	0.356432042	0.0367397	+	G	A	0.4016	0.039
rs7797255	LC_IGP_R16	IgG1_G1N/IgG1_G1	7	50351604	1	9.24E-09	1.13E-08	-	-0.211577	-0.2227	+	G	A	0.3565625	0.0366407	+	G	A	0.4016	0.039
rs7797255	LC_IGP56	IgG1_G1Nn	7	50351604	1	5.07E-09	1.06E-12	-	-0.216277	-0.2774	+	G	A	0.356907063	0.036802	+	G	A	0.4016	0.039
rs7789913	LC_IGP78	IgG1 BG0n/G0n	7	50352695	1	5.74E-10	4.11E-12	-	-0.22877	-0.2701	+	T	C	0.357532528	0.0366885	+	T	C	0.4039	0.039
rs7789913	LC_IGP79	IgG1 BG1n/G1n	7	50352695	1	3.93E-09	4.02E-12	-	-0.217952	-0.2694	+	T	C	0.357532528	0.0368165	+	T	C	0.4039	0.0388
rs7789913	LC_IGP77	IgG1 Bn	7	50352695	1	4.80E-11	4.67E-12	-	-0.243215	-0.2702	+	T	C	0.357532528	0.0367258	+	T	C	0.4039	0.0391
rs7789913	LC_IGP86	IgG1 Bn/Fn total	7	50352695	1	1.09E-10	1.78E-11	-	-0.238664	-0.2628	+	T	C	0.357532528	0.0367361	+	T	C	0.4039	0.0391
rs7789913	LC_IGP81	IgG1 Fn/Bn	7	50352695	1	4.07E-11	3.08E-12	+	0.24397	0.271	+	T	C	0.357532528	0.036702	+	T	C	0.4039	0.0389
rs7789913	LC_IGP14	IgG1_G0N	7	50352695	1	1.26E-08	1.93E-09	-	-0.209621	-0.233	+	T	C	0.357666565	0.036644	+	T	C	0.4039	0.0388
rs7789913	LC_IGP55	IgG1_G0Nn	7	50352695	1	1.10E-09	5.91E-10	-	-0.22521	-0.2407	+	T	C	0.357532528	0.0367348	+	T	C	0.4039	0.0389
rs7789913	LC_IGP15	IgG1_G1N	7	50352695	1	3.87E-09	1.74E-12	-	-0.217702	-0.2744	+	T	C	0.357055966	0.03676	+	T	C	0.4039	0.0389
rs7789913	LC_IGP_R16	IgG1_G1N/IgG1_G1	7	50352695	1	7.47E-09	1.48E-08	-	-0.213039	-0.2202	+	T	C	0.357187192	0.0366626	+	T	C	0.4039	0.0389
rs7789913	LC_IGP56	IgG1_G1Nn	7	50352695	1	3.80E-09	9.63E-13	-	-0.218192	-0.2772	+	T	C	0.357532528	0.0368225	+	T	C	0.4039	0.0388
rs6583438	LC_IGP77	IgG1 Bn	7	50352892	1	1.39E-08	4.71E-11	-	-0.217834	-0.2655	+	T	C	0.321919565	0.038192	+	T	C	0.3706	0.0403
rs6583438	LC_IGP86	IgG1 Bn/Fn total	7	50352892	1	2.60E-08	1.16E-10	-	-0.213697	-0.2601	+	T	C	0.321919565	0.038198	+	T	C	0.3706	0.0404
rs6583438	LC_IGP81	IgG1 Fn/Bn	7	50352892	1	1.50E-08	4.10E-11	+	0.217248	0.265	+	T	C	0.321919565	0.0381727	+	T	C	0.3706	0.0402
rs7804185	LC_IGP78	IgG1 BG0n/G0n	7	50353144	1	3.31E-10	4.89E-12	-	-0.232166	-0.2696	+	C	T	0.35866202	0.0367161	+	C	T	0.4049	0.039
rs7804185	LC_IGP79	IgG1 BG1n/G1n	7	50353144	1	2.00E-09	5.09E-12	-	-0.222233	-0.2686	+	C	T	0.35866202	0.0368416	+	C	T	0.4049	0.0389
rs7804185	LC_IGP77	IgG1 Bn	7	50353144	1	2.24E-11	5.81E-12	-	-0.247591	-0.2694	+	C	T	0.35866202	0.0367487	+	C	T	0.4049	0.0391
rs7804185	LC_IGP86	IgG1 Bn/Fn total	7	50353144	1	5.19E-11	2.21E-11	-	-0.242999	-0.262	+	C	T	0.35866202	0.0367595	+	C	T	0.4049	0.0392
rs7804185	LC_IGP84	IgG1 FBn/Bn total	7	50353144	1	4.46E-08	1.69E-09	+	0.203336	0.2374	+	C	T	0.35866202	0.0369849	+	C	T	0.4049	0.0394
rs7804185	LC_IGP81	IgG1 Fn/Bn	7	50353144	1	1.81E-11	3.86E-12	+	0.248592	0.2703	+	C	T	0.35866202	0.0367238	+	C	T	0.4049	0.0389
rs7804185	LC_IGP14	IgG1_G0N	7	50353144	1	6.44E-09	2.30E-09	-	-0.214002	-0.2322	+	C	T	0.358783833	0.0366689	+	C	T	0.4049	0.0389
rs7804185	LC_IGP55	IgG1_G0Nn	7	50353144	1	5.44E-10	7.03E-10	-	-0.229523	-0.24	+	C	T	0.35866202	0.0367592	+	C	T	0.4049	0.0389
rs7804185	LC_IGP15	IgG1_G1N	7	50353144	1	2.14E-09	2.25E-12	-	-0.221488	-0.2735	+	C	T	0.358178659	0.0367866	+	C	T	0.4049	0.039
rs7804185	LC_IGP_R16	IgG1_G1N/IgG1_G1	7	50353144	1	4.35E-09	1.28E-08	-	-0.216564	-0.2217	+	C	T	0.358311576	0.0366903	+	C	T	0.4049	0.039
rs7804185	LC_IGP56	IgG1_G1Nn	7	50353144	1	2.16E-09	1.24E-12	-	-0.221829	-0.2763	+	C	T	0.35866202	0.03685	+	C	T	0.4049	0.0389
rs7791218	LC_IGP77	IgG1 Bn	7	50353627	1	4.54E-09	1.75E-11	-	-0.221913	-0.2641	+	A	G	0.334369269	0.0376421	+	A	G	0.3896	0.0393
rs7791218	LC_IGP86	IgG1 Bn/Fn total	7	50353627	1	8.85E-09	4.77E-11	-	-0.217682	-0.2584	+	A	G	0.334369269	0.0376491	+	A	G	0.3896	0.0393
rs7791218	LC_IGP81	IgG1 Fn/Bn	7	50353627	1	4.81E-09	1.51E-11	+	0.221438	0.2636	+	A	G	0.334369269	0.0376227	+	A	G	0.3896	0.0391
rs7791218	LC_IGP15	IgG1_G1N	7	50353627	1	4.53E-08	5.53E-12	-	-0.206722	-0.2695	+	A	G	0.333756458	0.0376193	+	A	G	0.3896	0.0391
rs10269380	LC_IGP77	IgG1 Bn	7	50354512	1	4.35E-09	1.84E-11	-	-0.222287	-0.2638	+	A	G	0.33471675	0.0376583	+	A	G	0.3898	0.0393
rs10269380	LC_IGP86	IgG1 Bn/Fn total	7	50354512	1	8.48E-09	5.00E-11	-	-0.218057	-0.2581	+	A	G	0.33471675	0.0376653	+	A	G	0.3898	0.0393
rs10269380	LC_IGP81	IgG1 Fn/Bn	7	50354512	1	4.64E-09	1.60E-11	+	0.221762	0.2633	+	A	G	0.33471675	0.0376391	+	A	G	0.3898	0.0391
rs10269380	LC_IGP15	IgG1_G1N	7	50354512	1	4.24E-08	5.83E-12	-	-0.207253	-0.2692	+	A	G	0.334104449	0.0376348	+	A	G	0.3898	0.0391
rs6421315	LC_IGP78	IgG1 BG0n/G0n	7	50355207	1	8.42E-10	4.73E-12	-	-0.227161	-0.2698	+	C	G	0.359231103	0.0367948	+	C	G	0.4048	0.039
rs6421315	LC_IGP79	IgG1 BG1n/G1n	7	50355207	1	3.62E-09	4.46E-12	-	-0.219035	-0.2694	+	C	G	0.359231103	0.0369128	+	C	G	0.4048	0.0389
rs6421315	LC_IGP77	IgG1 Bn	7	50355207	1	5.34E-11	5.23E-12	-	-0.24328	-0.27	+	C	G	0.359231103	0.0368261	+	C	G	0.4048	0.0391
rs6421315	LC_IGP86	IgG1 Bn/Fn total	7	50355207	1	1.22E-10	1.95E-11	-	-0.238697	-0.2628	+	C	G	0.359231103	0.0368365	+	C	G	0.4048	0.0392
rs6421315	LC_IGP81	IgG1 Fn/Bn	7	50355207	1	4.47E-11	3.55E-12	+	0.244115	0.2708	+	C	G	0.359231103	0.0368019	+	C	G	0.4048	0.0389
rs6421315	LC_IGP14	IgG1_G0N	7	50355207	1	1.75E-08	2.11E-09	-	-0.208116	-0.2329	+	C	G	0.359358236	0.0367476	+	C	G	0.4048	0.0389
rs6421315	LC_IGP55	IgG1_G0Nn	7	50355207	1	1.60E-09	6.50E-10	-	-0.223593	-0.2405	+	C	G	0.359231103	0.0368412	+	C	G	0.4048	0.0389
rs6421315	LC_IGP15	IgG1_G1N	7	50355207	1	3.65E-09	1.92E-12	-	-0.218634	-0.2743	+	C	G	0.358752153	0.0368556	+	C	G	0.4048	0.039
rs6421315	LC_IGP_R16	IgG1_G1N/IgG1_G1	7	50355207	1	6.91E-09	1.74E-08	-	-0.214077	-0.2196	+	C	G	0.358885468	0.0367577	+	C	G	0.4048	0.039
rs6421315	LC_IGP56	IgG1_G1Nn	7	50355207	1	3.61E-09	1.07E-12	-	-0.21908	-0.2771	+	C	G	0.359231103	0.0369196	+	C	G	0.4048	0.0389
rs7019909	LC_IGP_R89	IgG4_G2FS1/IgG4_G1FS1	9	33113322	1	2.90E-08	1.52E-07	-	-0.30427	-0.3075	+	T	C	0.124582681	0.0545816	+	T	C	0.1046	0.0586
rs73484569	LC_IGP_R20	IgG1_G1/IgG1_G0	9	33133191	2	1.94E-08	5.26E-07	+	0.747111	0.674	+	A	G	0.017114619	0.132335	+	A	G	0.0173	0.1344
rs3824459	LC_IGP_R20	IgG1_G1/IgG1_G0	9	33134617	2	1.94E-08	3.86E-07	+	0.747111	0.6225	+	T	C	0.017114619	0.132335	+	T	C	0.0204	0.1226
rs73472094	LC_IGP_R20	IgG1_G1/IgG1_G0	9	33135537	2	1.93E-08	5.32E-07	+	0.747148	0.674	+	A	G	0.017114914	0.132335	+	A	G	0.0173	0.1344
rs12346594	LC_IGP_R20	IgG1_G1/IgG1_G0	9	33136536	2	1.93E-08	1.60E-06	+	0.747148	0.6273	+	A	G	0.017114914	0.132335	+	A	G	0.0184	0.1307
rs3780485	LC_IGP_R20	IgG1_G1/IgG1_G0	9	33139618	2	1.83E-08	5.58E-07	+	0.748377	0.6774	+	C	T	0.01712773	0.132333	+	C	T	0.0173	0.1353
rs10121006	LC_IGP_R20	IgG1_G1/IgG1_G0	9	33141250	2	1.80E-08	5.60E-07	+	0.748795	0.6776	+	T	C	0.017131081	0.132339	+	T	C	0.0173	0.1354
rs7020500	LC_IGP_R20	IgG1_G1/IgG1_G0	9	33143733	2	1.69E-08	5.72E-07	+	0.754561	0.6821	+	C	G	0.017066921	0.133087	+	C	G	0.0172	0.1364
rs113197944	LC_IGP_R20	IgG1_G1/IgG1_G0	9	33149340	2	1.55E-08	5.77E-07	+	0.763066	0.6833	+	A	G	0.016971261	0.134245	+	A	G	0.0171	0.1367
rs1328899	LC_IGP_R20	IgG1_G1/IgG1_G0	9	33151415	2	1.30E-08	3.97E-07	+	0.739893	0.6914	+	T	C	0.018947069	0.129466	+	T	C	0.0176	0.1364
rs12343079	LC_IGP_R20	IgG1_G1/IgG1_G0	9	33158557	2	1.55E-08	6.77E-07	+	0.763315	0.701	+	A	G	0.016968447	0.134284	+	A	G	0.0162	0.1411
rs3780481	LC_IGP_R20	IgG1_G1/IgG1_G0	9	33163298	2	1.55E-08	7.08E-07	+	0.763513	0.6992	+	T	C	0.016966721	0.134304	+	T	C	0.0162	0.141
rs3780478	LC_IGP_R20	IgG1_G1/IgG1_G0	9	33164116	2	1.55E-08	6.79E-07	+	0.76367	0.7012	+	A	T	0.016964836	0.134336	+	A	T	0.0162	0.1412
rs73472938	LC_IGP_R20	IgG1_G1/IgG1_G0	9	33164527	2	1.55E-08	6.83E-07	+	0.763739	0.7011	+	A	T	0.016964887	0.134347	+	A	T	0.0162	0.1412
rs3758273	LC_IGP_R20	IgG1_G1/IgG1_G0	9	33168309	2	1.02E-08	7.09E-07	+	0.803103	0.7026	+	A	C	0.016043122	0.139512	+	A	C	0.0161	0.1417
rs28429368	LC_IGP_R22	IgG1_G1F/IgG1_G0F	9	33125359	2	4.19E-10	3.15E-08	+	0.83498	0.7766	+	T	C	0.017061266	0.132853	+	T	C	0.0158	0.1404
rs3780494	LC_IGP_R22	IgG1_G1F/IgG1_G0F	9	33126224	2	4.38E-10	3.35E-08	+	0.831918	0.7775	+	C	T	0.017083588	0.13251	+	C	T	0.0157	0.1408
rs73484569	LC_IGP_R22	IgG1_G1F/IgG1_G0F	9	33133191	2	4.37E-10	4.62E-09	+	0.831991	0.7882	+	A	G	0.017083293	0.132515	+	A	G	0.0173	0.1345
rs3824459	LC_IGP_R22	IgG1_G1F/IgG1_G0F	9	33134617	2	4.37E-10	6.60E-09	+	0.831991	0.7116	+	T	C	0.017083293	0.132515	+	T	C	0.0204	0.1227
rs73472094	LC_IGP_R22	IgG1_G1F/IgG1_G0F	9	33135537	2	4.36E-10	4.58E-09	+	0.832029	0.7885	+	A	G	0.017083588	0.132514	+	A	G	0.0173	0.1345
rs12346594	LC_IGP_R22	IgG1_G1F/IgG1_G0F	9	33136536	2	4.36E-10	2.39E-08	+	0.832029	0.7412	+	A	G	0.017083588	0.132514	+	A	G	0.0184	0.1328
rs3780485	LC_IGP_R22	IgG1_G1F/IgG1_G0F	9	33139618	2	4.24E-10	5.27E-09	+	0.832605	0.7892	+	C	T	0.017096379	0.132515	+	C	T	0.0173	0.1352
rs10121006	LC_IGP_R22	IgG1_G1F/IgG1_G0F	9	33141250	2	4.22E-10	5.27E-09	+	0.832761	0.7895	+	T	C	0.017099725	0.132521	+	T	C	0.0173	0.1352
rs7020500	LC_IGP_R22	IgG1_G1F/IgG1_G0F	9	33143733	2	4.10E-10	5.09E-09	+	0.838123	0.7946	+	C	G	0.017035682	0.133273	+	C	G	0.0172	0.136
rs113197944	LC_IGP_R22	IgG1_G1F/IgG1_G0F	9	33149340	2	3.79E-10	5.10E-09	+	0.84708	0.7958	+	A	G	0.016940197	0.134433	+	A	G	0.0171	0.1362
rs1328899	LC_IGP_R22	IgG1_G1F/IgG1_G0F	9	33151415	2	1.92E-09	3.07E-09	+	0.783761	0.8052	+	T	C	0.018912389	0.129786	+	T	C	0.0176	0.1358
rs12343079	LC_IGP_R22	IgG1_G1F/IgG1_G0F	9	33158557	2	3.80E-10	1.00E-08	+	0.847278	0.7978	+	A	G	0.016937389	0.134472	+	A	G	0.0162	0.1392
rs3780481	LC_IGP_R22	IgG1_G1F/IgG1_G0F	9	33163298	2	3.79E-10	1.03E-08	+	0.847462	0.7971	+	T	C	0.016935665	0.134492	+	T	C	0.0162	0.1392
rs3780478	LC_IGP_R22	IgG1_G1F/IgG1_G0F	9	33164116	2	3.80E-10	1.00E-08	+	0.847622	0.798	+	A	T	0.016933783	0.134525	+	A	T	0.0162	0.1393
rs73472938	LC_IGP_R22	IgG1_G1F/IgG1_G0F	9	33164527	2	3.80E-10	1.01E-08	+	0.84765	0.798	+	A	T	0.016933835	0.134536	+	A	T	0.0162	0.1393
rs3758273	LC_IGP_R22	IgG1_G1F/IgG1_G0F	9	33168309	2	1.15E-09	1.07E-08	+	0.85614	0.7992	+	A	C	0.016014052	0.139835	+	A	C	0.0161	0.1397
rs12337458	LC_IGP_R22	IgG1_G1F/IgG1_G0F	9	33180566	2	1.27E-10	1.10E-06	+	1.03009	0.7797	+	C	T	0.013489372	0.159144	+	C	T	0.0132	0.16
rs28429368	LC_IGP_R23	IgG1_G1FN/IgG1_G0FN	9	33125359	2	1.59E-13	2.21E-09	+	0.982926	0.8364	+	T	C	0.01708211	0.132078	+	T	C	0.0158	0.1398
rs3780494	LC_IGP_R23	IgG1_G1FN/IgG1_G0FN	9	33126224	2	1.63E-13	2.55E-09	+	0.979989	0.8359	+	C	T	0.017104459	0.131735	+	C	T	0.0157	0.1403
rs73484569	LC_IGP_R23	IgG1_G1FN/IgG1_G0FN	9	33133191	2	1.63E-13	4.66E-10	+	0.980052	0.8337	+	A	G	0.017104164	0.13174	+	A	G	0.0173	0.1338
rs3824459	LC_IGP_R23	IgG1_G1FN/IgG1_G0FN	9	33134617	2	1.63E-13	7.68E-10	+	0.980052	0.7589	+	T	C	0.017104164	0.13174	+	T	C	0.0204	0.1234
rs73472094	LC_IGP_R23	IgG1_G1FN/IgG1_G0FN	9	33135537	2	1.62E-13	4.70E-10	+	0.980091	0.8336	+	A	G	0.017104459	0.13174	+	A	G	0.0173	0.1338
rs12346594	LC_IGP_R23	IgG1_G1FN/IgG1_G0FN	9	33136536	2	1.62E-13	2.64E-09	+	0.980091	0.7875	+	A	G	0.017104459	0.13174	+	A	G	0.0184	0.1323
rs3780485	LC_IGP_R23	IgG1_G1FN/IgG1_G0FN	9	33139618	2	1.57E-13	4.90E-10	+	0.98067	0.8362	+	C	T	0.017117267	0.131739	+	C	T	0.0173	0.1344
rs10121006	LC_IGP_R23	IgG1_G1FN/IgG1_G0FN	9	33141250	2	1.56E-13	4.90E-10	+	0.980855	0.8364	+	T	C	0.017120616	0.131746	+	T	C	0.0173	0.1344
rs7020500	LC_IGP_R23	IgG1_G1FN/IgG1_G0FN	9	33143733	2	1.47E-13	4.60E-10	+	0.987431	0.841	+	C	G	0.017056495	0.132491	+	C	G	0.0172	0.1349
rs113197944	LC_IGP_R23	IgG1_G1FN/IgG1_G0FN	9	33149340	2	1.34E-13	4.58E-10	+	0.997723	0.8421	+	A	G	0.016960894	0.133643	+	A	G	0.0171	0.1351
rs1328899	LC_IGP_R23	IgG1_G1FN/IgG1_G0FN	9	33151415	2	3.75E-13	2.68E-10	+	0.944685	0.8511	+	T	C	0.018935495	0.128978	+	T	C	0.0176	0.1348
rs12343079	LC_IGP_R23	IgG1_G1FN/IgG1_G0FN	9	33158557	2	1.34E-13	8.13E-10	+	0.997999	0.8441	+	A	G	0.016958082	0.133682	+	A	G	0.0162	0.1374
rs3780481	LC_IGP_R23	IgG1_G1FN/IgG1_G0FN	9	33163298	2	1.34E-13	8.12E-10	+	0.998218	0.8437	+	T	C	0.016956357	0.133701	+	T	C	0.0162	0.1374

rs3780478	LC_IGP_R23	IgG1_G1FN/IgG1_G0FN	9	33164116	2	1.34E-13	8.17E-10	+	0.998378	0.8442	+	A	T	0.016954472	0.133734	+	A	T	0.0162	0.1375
rs73472938	LC_IGP_R23	IgG1_G1FN/IgG1_G0FN	9	33164527	2	1.34E-13	8.22E-10	+	0.998456	0.8442	+	A	T	0.016954524	0.133745	+	A	T	0.0162	0.1375
rs3758273	LC_IGP_R23	IgG1_G1FN/IgG1_G0FN	9	33168309	2	3.63E-13	8.83E-10	+	1.01873	0.845	+	A	C	0.016033322	0.139004	+	A	C	0.0161	0.1379
rs12337458	LC_IGP_R23	IgG1_G1FN/IgG1_G0FN	9	33180566	2	7.45E-14	4.17E-07	+	1.19408	0.8003	+	C	T	0.013504961	0.158256	+	C	T	0.0132	0.1581
rs1969977	LC_IGP_R28	IgG1_G2F/IgG1_G1F	9	33120203	2	1.94E-08	7.27E-07	-	-0.232444	-0.2179	+	G	A	0.235256716	0.041175	+	G	A	0.2419	0.044
rs10971417	LC_IGP_R28	IgG1_G2F/IgG1_G1F	9	33121480	2	3.03E-08	2.55E-07	-	-0.216646	-0.2145	+	T	C	0.26016514	0.0389175	+	T	C	0.2577	0.0416
rs12342831	LC_IGP_R28	IgG1_G2F/IgG1_G1F	9	33124872	2	2.16E-08	1.99E-07	-	-0.217267	-0.2134	+	C	T	0.261285714	0.0386162	+	C	T	0.2631	0.041
rs10813951	LC_IGP_R28	IgG1_G2F/IgG1_G1F	9	33128021	2	2.01E-08	4.40E-07	-	-0.21766	-0.2077	+	G	A	0.261289377	0.038599	+	G	A	0.2647	0.0411
rs3780486	LC_IGP_R28	IgG1_G2F/IgG1_G1F	9	33139453	2	1.99E-08	5.08E-07	-	-0.217731	-0.2074	+	T	C	0.261294261	0.0385959	+	T	C	0.2662	0.0413
chr9:33152337:D	LC_IGP_R28	IgG1_G2F/IgG1_G1F	9	33152337	2	1.41E-08	5.09E-07	-	-0.221475	-0.2085	+	C	CAT	0.259763126	0.0388451	+	C	CAT	0.2654	0.0415
rs10813957	LC_IGP_R28	IgG1_G2F/IgG1_G1F	9	33153527	2	2.20E-08	7.70E-07	-	-0.21783	-0.2048	+	T	G	0.26173779	0.038735	+	T	G	0.2668	0.0414
rs3780480	LC_IGP_R28	IgG1_G2F/IgG1_G1F	9	33163486	2	3.08E-08	4.55E-07	-	-0.215906	-0.2096	+	T	G	0.26325641	0.0388069	+	T	G	0.2654	0.0415
rs10971434	LC_IGP_R28	IgG1_G2F/IgG1_G1F	9	33168891	2	2.16E-08	5.37E-07	-	-0.218788	-0.2098	+	A	C	0.263001221	0.0388882	+	A	C	0.2682	0.0418
rs10758195	LC_IGP_R28	IgG1_G2F/IgG1_G1F	9	33174587	2	1.22E-08	6.59E-07	-	-0.225396	-0.2113	+	T	C	0.265822182	0.0393637	+	T	C	0.2691	0.0425
rs1969977	LC_IGP_R29	IgG1_G2FS1/IgG1_G1FS1	9	33120203	2	3.72E-08	2.93E-09	-	-0.227989	-0.2594	+	G	A	0.235382569	0.041225	+	G	A	0.2419	0.0437
rs10971417	LC_IGP_R29	IgG1_G2FS1/IgG1_G1FS1	9	33121480	2	3.53E-08	1.46E-09	-	-0.215773	-0.2506	+	T	C	0.260336697	0.0389522	+	T	C	0.2577	0.0414
rs12342831	LC_IGP_R29	IgG1_G2FS1/IgG1_G1FS1	9	33124872	2	2.66E-08	8.88E-10	-	-0.216051	-0.2505	+	C	T	0.261459327	0.0386516	+	C	T	0.2631	0.0409
rs10813951	LC_IGP_R29	IgG1_G2FS1/IgG1_G1FS1	9	33128021	2	2.52E-08	2.82E-09	-	-0.216329	-0.2435	+	G	A	0.261462997	0.0386348	+	G	A	0.2647	0.041
rs3780486	LC_IGP_R29	IgG1_G2FS1/IgG1_G1FS1	9	33139453	2	2.50E-08	3.63E-09	-	-0.216364	-0.2425	+	T	C	0.26146789	0.0386317	+	T	C	0.2662	0.0411
chr9:33152337:D	LC_IGP_R29	IgG1_G2FS1/IgG1_G1FS1	9	33152337	2	1.59E-08	3.76E-09	-	-0.220834	-0.2437	+	C	CAT	0.259935474	0.0388785	+	C	CAT	0.2654	0.0414
rs10813957	LC_IGP_R29	IgG1_G2FS1/IgG1_G1FS1	9	33153527	2	2.86E-08	6.24E-09	-	-0.216215	-0.2396	+	T	G	0.261913761	0.0387716	+	T	G	0.2668	0.0412
rs3780480	LC_IGP_R29	IgG1_G2FS1/IgG1_G1FS1	9	33163486	2	4.52E-08	4.22E-09	-	-0.213466	-0.2434	+	T	G	0.263435474	0.0388465	+	T	G	0.2654	0.0414
rs10971434	LC_IGP_R29	IgG1_G2FS1/IgG1_G1FS1	9	33168891	2	2.43E-08	6.29E-09	-	-0.218189	-0.2424	+	A	C	0.263180734	0.0389214	+	A	C	0.2682	0.0417
rs10758195	LC_IGP_R29	IgG1_G2FS1/IgG1_G1FS1	9	33174587	2	1.76E-08	8.21E-09	-	-0.223073	-0.2442	+	T	C	0.265925525	0.039397	+	T	C	0.2691	0.0424
rs28429368	LC_IGP_R62	IgG2_G1F/IgG2_G0F	9	33125359	2	2.11E-08	3.82E-12	+	0.750567	0.9433	+	T	C	0.017061266	0.133297	+	T	C	0.0158	0.1358
rs3780494	LC_IGP_R62	IgG2_G1F/IgG2_G0F	9	33126224	2	2.14E-08	3.90E-12	+	0.748275	0.946	+	C	T	0.017083588	0.132951	+	C	T	0.0157	0.1363
rs73484569	LC_IGP_R62	IgG2_G1F/IgG2_G0F	9	33133191	2	2.13E-08	1.28E-13	+	0.748343	0.9683	+	A	G	0.017083293	0.132955	+	A	G	0.0173	0.1307
rs3824459	LC_IGP_R62	IgG2_G1F/IgG2_G0F	9	33134617	2	2.13E-08	7.99E-14	+	0.748343	0.9179	+	T	C	0.017083293	0.132955	+	T	C	0.0204	0.1229
rs73472094	LC_IGP_R62	IgG2_G1F/IgG2_G0F	9	33135537	2	2.13E-08	1.28E-13	+	0.748369	0.9684	+	A	G	0.017083588	0.132955	+	A	G	0.0173	0.1307
rs12346594	LC_IGP_R62	IgG2_G1F/IgG2_G0F	9	33136536	2	2.13E-08	1.55E-13	+	0.748369	0.961	+	A	G	0.017083588	0.132955	+	A	G	0.0184	0.1302
rs3780485	LC_IGP_R62	IgG2_G1F/IgG2_G0F	9	33139618	2	2.03E-08	1.57E-13	+	0.749495	0.9681	+	C	T	0.017096379	0.132954	+	C	T	0.0173	0.1312
rs10121006	LC_IGP_R62	IgG2_G1F/IgG2_G0F	9	33141250	2	2.01E-08	1.55E-13	+	0.749826	0.9688	+	T	C	0.017099725	0.13296	+	T	C	0.0173	0.1312
rs7020500	LC_IGP_R62	IgG2_G1F/IgG2_G0F	9	33143733	2	1.95E-08	1.29E-13	+	0.75472	0.9764	+	C	G	0.017035682	0.133714	+	C	G	0.0172	0.1318
rs113197944	LC_IGP_R62	IgG2_G1F/IgG2_G0F	9	33149340	2	1.88E-08	1.24E-13	+	0.762245	0.9784	+	A	G	0.016940197	0.134881	+	A	G	0.0171	0.132
rs1328899	LC_IGP_R62	IgG2_G1F/IgG2_G0F	9	33151415	2	2.39E-08	1.28E-13	+	0.729709	0.9782	+	T	C	0.018912389	0.130112	+	T	C	0.0176	0.132
rs12343079	LC_IGP_R62	IgG2_G1F/IgG2_G0F	9	33158557	2	1.87E-08	2.41E-13	+	0.762491	0.982	+	A	G	0.016937389	0.13492	+	A	G	0.0162	0.1341
rs3780481	LC_IGP_R62	IgG2_G1F/IgG2_G0F	9	33163298	2	1.87E-08	2.53E-13	+	0.762639	0.9813	+	T	C	0.016935665	0.13494	+	T	C	0.0162	0.1341
rs3780478	LC_IGP_R62	IgG2_G1F/IgG2_G0F	9	33164116	2	1.87E-08	2.42E-13	+	0.762801	0.9823	+	A	T	0.016933783	0.134973	+	A	T	0.0162	0.1341
rs73472938	LC_IGP_R62	IgG2_G1F/IgG2_G0F	9	33164527	2	1.87E-08	2.42E-13	+	0.762863	0.9824	+	A	T	0.016933835	0.134984	+	A	T	0.0162	0.1341
rs12337458	LC_IGP_R62	IgG2_G1F/IgG2_G0F	9	33180566	2	1.11E-08	4.77E-11	+	0.917189	1.0077	+	C	T	0.013488482	0.159729	+	C	T	0.0132	0.1532
rs28429368	LC_IGP_R63	IgG2_G1FN/IgG2_G0FN	9	33125359	2	6.25E-09	3.71E-12	+	0.777529	0.9432	+	T	C	0.017071682	0.133117	+	T	C	0.0158	0.1358
rs3780494	LC_IGP_R63	IgG2_G1FN/IgG2_G0FN	9	33126224	2	6.18E-09	3.95E-12	+	0.775753	0.9462	+	C	T	0.017094017	0.132769	+	C	T	0.0157	0.1364
rs73484569	LC_IGP_R63	IgG2_G1FN/IgG2_G0FN	9	33133191	2	6.18E-09	8.23E-14	+	0.775791	0.9711	+	A	G	0.017093722	0.132774	+	A	G	0.0173	0.1301
rs3824459	LC_IGP_R63	IgG2_G1FN/IgG2_G0FN	9	33134617	2	6.18E-09	3.52E-14	+	0.775791	0.9243	+	T	C	0.017093722	0.132774	+	T	C	0.0204	0.122
rs73472094	LC_IGP_R63	IgG2_G1FN/IgG2_G0FN	9	33135537	2	6.17E-09	8.39E-14	+	0.775825	0.9712	+	A	G	0.017094017	0.132774	+	A	G	0.0173	0.1301
rs12346594	LC_IGP_R63	IgG2_G1FN/IgG2_G0FN	9	33136536	2	6.17E-09	1.00E-13	+	0.775825	0.9639	+	A	G	0.017094017	0.132774	+	A	G	0.0184	0.1295
rs3780485	LC_IGP_R63	IgG2_G1FN/IgG2_G0FN	9	33139618	2	5.84E-09	9.97E-14	+	0.777051	0.9726	+	C	T	0.017106817	0.132772	+	C	T	0.0173	0.1307
rs10121006	LC_IGP_R63	IgG2_G1FN/IgG2_G0FN	9	33141250	2	5.74E-09	9.86E-14	+	0.777449	0.9733	+	T	C	0.017110164	0.132778	+	T	C	0.0173	0.1308
rs7020500	LC_IGP_R63	IgG2_G1FN/IgG2_G0FN	9	33143733	2	5.49E-09	8.58E-14	+	0.782879	0.982	+	C	G	0.017046082	0.13353	+	C	G	0.0172	0.1316
rs113197944	LC_IGP_R63	IgG2_G1FN/IgG2_G0FN	9	33149340	2	4.98E-09	8.32E-14	+	0.791891	0.9843	+	A	G	0.016950539	0.13469	+	A	G	0.0171	0.1319
rs1328899	LC_IGP_R63	IgG2_G1FN/IgG2_G0FN	9	33151415	2	9.79E-09	1.12E-13	+	0.749117	0.9788	+	T	C	0.018923935	0.129961	+	T	C	0.0176	0.1318
rs12343079	LC_IGP_R63	IgG2_G1FN/IgG2_G0FN	9	33158557	2	4.97E-09	1.27E-13	+	0.792157	1.0054	+	A	G	0.016947729	0.134729	+	A	G	0.0162	0.1357
rs3780481	LC_IGP_R63	IgG2_G1FN/IgG2_G0FN	9	33163298	2	4.96E-09	1.31E-13	+	0.792331	1.0048	+	T	C	0.016946005	0.134749	+	T	C	0.0162	0.1357
rs3780478	LC_IGP_R63	IgG2_G1FN/IgG2_G0FN	9	33164116	2	4.97E-09	1.28E-13	+	0.792501	1.0058	+	A	T	0.016944122	0.134782	+	A	T	0.0162	0.1358
rs73472938	LC_IGP_R63	IgG2_G1FN/IgG2_G0FN	9	33164527	2	4.96E-09	1.28E-13	+	0.792609	1.0058	+	A	T	0.016944173	0.134793	+	A	T	0.0162	0.1358
rs3758273	LC_IGP_R63	IgG2_G1FN/IgG2_G0FN	9	33168309	2	2.29E-08	1.33E-13	+	0.786996	1.0086	+	A	C	0.016023534	0.140137	+	A	C	0.0161	0.1362
rs12337458	LC_IGP_R63	IgG2_G1FN/IgG2_G0FN	9	33180566	2	5.26E-09	1.37E-11	+	0.936613	1.0341	+	C	T	0.013496716	0.159558	+	C	T	0.0132	0.153
chr9:33152337:D	LC_IGP_R68	IgG2_G2F/IgG2_G1F	9	33152337	2	4.90E-08	7.89E-07	-	-0.213323	-0.2059	+	C	CAT	0.259909701	0.0389227	+	C	CAT	0.2654	0.0417
rs10971417	LC_IGP_R69	IgG2_G2FS1/IgG2_G1FS1	9	33121480	2	1.03E-08	1.47E-09	-	-0.224201	-0.2512	+	T	C	0.260466728	0.0389535	+	T	C	0.2577	0.0415
rs12342831	LC_IGP_R69	IgG2_G2FS1/IgG2_G1FS1	9	33124872	2	8.63E-09	1.38E-09	-	-0.223653	-0.2483	+	C	T	0.261590965	0.0386554	+	C	T	0.2631	0.041
rs10813951	LC_IGP_R69	IgG2_G2FS1/IgG2_G1FS1	9	33128021	2	8.27E-09	4.47E-09	-	-0.223843	-0.2405	+	G	A	0.261594628	0.0386389	+	G	A	0.2647	0.041
rs3780486	LC_IGP_R69	IgG2_G2FS1/IgG2_G1FS1	9	33139453	2	8.21E-09	8.16E-09	-	-0.22387	-0.2375	+	T	C	0.261599512	0.0386359	+	T	C	0.2662	0.0412
chr9:33152337:D	LC_IGP_R69	IgG2_G2FS1/IgG2_G1FS1	9	33152337	2	8.32E-09	7.69E-09	-	-0.225272	-0.2392	+	C	CAT	0.260068376	0.0388934	+	C	CAT	0.2654	0.0414
rs10813957	LC_IGP_R69	IgG2_G2FS1/IgG2_G1FS1	9	33153527	2	1.05E-08	1.22E-08	-	-0.223079	-0.2354	+	T	G	0.26204304	0.0387785	+	T	G	0.2668	0.0413
rs3780480	LC_IGP_R69	IgG2_G2FS1/IgG2_G1FS1	9	33163486	2	7.82E-09	1.31E-08	-	-0.225352	-0.2358	+	T	G	0.263561355	0.0388362	+	T	G	0.2654	0.0415
rs10971434	LC_IGP_R69	IgG2_G2FS1/IgG2_G1FS1	9	33168891	2	8.39E-09	1.03E-08	-	-0.225415	-0.239	+	A	C	0.263306471	0.0389272	+	A	C	0.2682	0.0418
rs10758195	LC_IGP_R69	IgG2_G2FS1/IgG2_G1FS1	9	33174587	2	6.14E-09	1.43E-08	-	-0.230308	-0.2399	+	T	C	0.266129569	0.0394095	+	T	C	0.2691	0.0423
rs6476398	LC_IGP_R88	IgG4_G2F/IgG4_G1F	9	33119241	2	9.80E-12	4.68E-12	-	-0.25037	-0.2742	+	T	C	0.318972993	0.0365032	+	T	C	0.3236	0.0396
rs7859454	LC_IGP_R88	IgG4_G2F/IgG4_G1F	9	33119583	2	1.19E-09	4.26E-13	-	-0.261235	-0.3412	+	A	T	0.198480304	0.0427024	+	A	T	0.1798	0.0471
rs10121987	LC_IGP_R88	IgG4_G2F/IgG4_G1F	9	33119818	2	9.22E-12	2.03E-12	-	-0.250226	-0.2792	+	G	A	0.319139549	0.0364342	+	G	A	0.3275	0.0397
rs1969977	LC_IGP_R88	IgG4_G2F/IgG4_G1F	9	33120203	2	3.01E-14	2.20E-13	-	-0.312186	-0.3273	+	G	A	0.235428093	0.0407199	+	G	A	0.2419	0.0446
rs76344840	LC_IGP_R88	IgG4_G2F/IgG4_G1F	9	33120204	2	1.47E-11	9.66E-12	-	-0.256867	-0.2841	+	G	A	0.297118525	0.0377773	+	G	A	0.3144	0.0417
rs3818593	LC_IGP_R88	IgG4_G2F/IgG4_G1F	9	33120706	2	1.05E-09	4.13E-13	-	-0.261139	-0.3388	+	G	A	0.198720935	0.0425511	+	G	A	0.1808	0.0467
rs10971417	LC_IGP_R88	IgG4_G2F/IgG4_G1F	9	33121480	2	3.37E-14	2.46E-14	-	-0.294457	-0.3219	+	T	C	0.260295247	0.0384836	+	T	C	0.2577	0.0422
rs10813948	LC_IGP_R88	IgG4_G2F/IgG4_G1F	9	33121570	2	8.66E-12	3.74E-12	-	-0.249972	-0.2742	+	A	G	0.319353748	0.0363484	+	A	G	0.3244	0.0395
rs10971418	LC_IGP_R88	IgG4_G2F/IgG4_G1F	9	33122024	2	1.04E-09	3.95E-13	-	-0.261148	-0.3395	+	A	C	0.19873059	0.0425441	+	A	C	0.1807	0.0468
rs10116966	LC_IGP_R88	IgG4_G2F/IgG4_G1F	9	33122370	2	7.87E-12	3.21E-12	-	-0.250078	-0.275	+	A	T	0.319719378	0.0362898	+	A	T	0.3246	0.0395
rs10113903	LC_IGP_R88	IgG4_G2F/IgG4_G1F	9	33122645	2	7.78E-12	3.90E-12	-	-0.250139	-0.2739	+	C	G	0.319810786	0.0362899	+	C	G	0.3254	0.0395
rs6476400	LC_IGP_R88	IgG4_G2F/IgG4_G1F	9	33123106	2	3.23E-11	2.39E-12	-	-0.242055	-0.2779	+	A	T	0.322416101	0.0362279	+	A	T	0.3275	0.0396
rs10813949	LC_IGP_R88	IgG4_G2F/IgG4_G1F	9	33123464	2	7.84E-12	3.33E-12	-	-0.250077	-0.2748	+	A	G	0.3197273	0.0362867	+	A	G	0.3249	0.0395
rs10738905	LC_IGP_R88	IgG4_G2F/IgG4_G1F	9	33123627	2	7.97E-12	3.83E-12	-	-0.250015	-0.2741	+	G	A	0.319855881	0.0362908	+	G	A	0.3257	0.0395
rs10971419	LC_IGP_R88	IgG4_G2F/IgG4_G1F	9	33124161	2	6.38E-12	2.91E-12	-	-0.250021	-0.2734	+	C	T	0.320183425	0.0361216	+	C	T	0.3271	0.0392
rs12342831	LC_IGP_R88	IgG4_G2F/IgG4_G1F	9	33124872	2	2.19E-14	1.34E-14	-	-0.294347	-0.3203	+	C	T	0.261417428	0.0381835	+	C	T	0.2631	0.0416
rs10971420	LC_IGP_R88	IgG4_G2F/IgG4_G1F	9	33125000	2	6.38E-12	2.91E-12	-	-0.250025	-0.2734	+	C	T	0.320184644	0.0361214	+	C	T	0.3271	0.0392
rs10758189	LC_IGP_R88	IgG4_G2F/IgG4_G1F	9	33125804	2	5.99E-12	4.49E-12	-	-0.250292	-0.271	+	C	T	0.320195003	0.0361129	+	C	T	0.328	0.0392
rs3780495	LC_IGP_R88	IgG4_G2F/IgG4_G1F	9	33125942	2	5.90E-12	5.26E-12	-	-0.250358	-0.2703	+	C	A	0.320197745	0.0361106	+	C	A	0.3281	0.0392
rs7850878	LC_IGP_R88	IgG4_G2F/IgG4_G1F	9	33126570	2	1.08E-11	5.62E-12	-	-0.247785	-0.2712	+	G	A	0.318818099	0.0362011	+	G	A	0.327	0.0394
rs10813950	LC_IGP_R88	IgG4_G2F/IgG4_G1F	9	33127640	2	5.70E-12	7.67E-12	-	-0.250505	-0.2682	+	A	G	0.320202925	0.0361054	+	A	G	0.3295	0.0392
rs10813951	LC_IGP_R88	IgG4_G2F/IgG4_G1F	9	33128021	2	1.98E-14	3.91E-14	-	-0.294724	-0.3151	+	G	A	0.261421085	0.0381658	+	G	A	0.2647	0.0417
rs3780490	LC_IGP_R88	IgG4_G2F/IgG4_G1F	9	33129839	2	5.68E-12	6.81E-12	-	-0.250568	-0.2684	+	G	A	0.320132846	0.0361115	+	G	A	0.3286	0.0391
rs3780489	LC_IGP_R88	IgG4_G2F/IgG4_G1F	9	33129844	2	5.69E-12	6.81E-12	-	-0.250512	-0.2684	+	T	G	0.320203534	0.0361051	+	T	G	0.3286	0.0391
rs10758192	LC_IGP_R88	IgG4_G2F/IgG4_G1F	9	33131651	2	5.68E-12	2.40E-11	-	-0.250518	-0.2611	+	A	G	0.32020323	0.0361049	+	A	G	0.3303	0.0391
rs12554995	LC_IGP_R88	IgG4_G2F/IgG4_G1F	9	33132044	2	5.68E-12	2.49E-11	-	-0.25052	-0.2609	+	G	A	0.320203839	0.0361048	+	G	A	0.3303	0.0391
rs56687083	LC_IGP_R88	IgG4_G2F/IgG4_G1F	9	33132134	2	5.68E-12	2.57E-11	-	-0.25052	-0.2614	+	G	A	0.320203839	0.0361048	+	G	A	0.3296	0.0392
chr9:33132215:I	LC_IGP_R88	IgG4_G2F/IgG4_G1F	9	33132215	2	7.81E-10	4.31E-13	-	-0.260482	-0.34	+	TG	T	0.199873796	0.0421126	+	TG	T	0.1853	0.0469
rs73645257	LC_IGP_R88	IgG4_G2F/IgG4_G1F	9	33132354	2	7.81E-10	4.17E-13	-	-0.260482	-0.3393	+	T	C	0.199873796	0.0421126	+	T	C	0.1848	0.0468
rs73484568	LC_IGP_R88	IgG4_G2F/IgG4_G1F	9	33132492	2	5.67E-12	2.51E-11	-	-0.250523	-0.2609	+	A	G	0.320203839	0.0361047	+	A	G	0.3303	0.0391
rs3780488	LC_IGP_R88	IgG4_G2F/IgG4_G1F	9	33133812	2	5.67E-12	2.58E-11	-	-0.250528	-0.2607	+	C	T	0.32020323	0.0361045	+	C	T	0.3303	0.0391
rs913214	LC_IGP_R88	IgG4_G2F/IgG4_G1F	9	33135085	2	5.65E-12	2.61E-11	-	-0.250539	-0.2607	+	A	G	0.320203534	0.036104	+	A	G	0.3304	0.0391
rs10738906	LC_IGP_R88	IgG4_G2F/IgG4_G1F	9	33135634	2	5.64E-12	2.88E-11	-	-0.250549	-0.2601	+	C	T	0.320204449	0.0361037	+	C	T	0.3305	0.0391
rs10738907	LC_IGP_R88	IgG4_G2F/IgG4_G1F	9	33135890	2	1.41E-11	2.75E-10	-	-0.248399	-0.2594	+	A	T	0.313928093	0.0365018	+	A	T	0.3167	0.0411
rs10124479	LC_IGP_R88	IgG4_G2F/IgG4_G1F	9	33136233	2	5.62E-12	4.07E-11	-	-0.250564	-0.2586	+	G	T	0.320204753	0.0361032	+	G	T	0.3317	0.0392
rs10813954	LC_IGP_R88	IgG4_G2F/IgG4_G1F	9	33137596	2	5.58E-12	3.60E-11	-	-0.250595	-0.2588	+	C	A	0.320206886	0.0361022	+	C	A	0.3308	0.0391
rs2209948	LC_IGP_R88	IgG4_G2F/IgG4_G1F	9	33138247	2	5.58E-12	3.59E-11	-	-0.250591	-0.259	+	A	G	0.320214808	0.0361024	+	A	G	0.3308	0.0391
rs7849148	LC_IGP_R88	IgG4_G2F/IgG4_G1F	9	33138352	2	5.58E-12	3.58E-11	-	-0.250592	-0.2589	+	T	A	0.320218769	0.0361024	+	T	A	0.3308	0.0391
rs10971424	LC_IGP_R88	IgG4_G2F/IgG4_G1F	9	33138775	2	5.58E-12	3.59E-11	-	-0.250593	-0.2589	+	C	T	0.320221816	0.0361025	+	C	T	0.3308	0.0391
rs3780487	LC_IGP_R88	IgG4_G2F/IgG4_G1F	9	33139237	2	5.58E-12	3.59E-11	-	-0.250593	-0.2589	+	C	T	0.320226996	0.0361025	+	C	T	0.3308	0.0391
rs3780486	LC_IGP_R88	IgG4_G2F/IgG4_G1F	9	33139453	2	1.94E-14	5.55E-14	-	-0.294801	-0.3145	+	T	C	0.26142596	0.0381625	+	T	C	0.2662	0.0418
rs7864705	LC_IGP_R88	IgG4_G2F/IgG4_G1F	9	33140352	2	6.28E-12	3.59E-11	-	-0.250043	-0.259	+	C	A	0.320349482	0.0361127	+	C	A	0.3307	0.0391
rs7865745	LC_IGP_R88	IgG4_G2F/IgG4_G1F	9	33140976	2	6.60E-12	3.57E-11	-	-0.249801	-0.2591	+	A	G	0.3203961	0.0361149	+	A	G	0.3307	0.0391
rs7868781	LC_IGP_R88	IgG4_G2F/IgG4_G1F	9	33141320	2	6.29E-12	4.10E-11	-	-0.251052	-0.2624	+	G	A	0.317100853	0.0362599	+	G	A	0.3242	0.0398
rs10813955	LC_IGP_R88	IgG4_G2F/IgG4_G1F	9	33141791	2	1.06E-11	3.58E-11	-	-0.247383	-0.2591	+	T	C	0.320840951	0.0361285	+	T	C	0.3307	0.0391
rs7873182	LC_IGP_R88	IgG4_G2F/IgG4_G1F	9	33142170	2	7.64E-12	2.03E-11	-	-0.249151	-0.2632	+	A	G	0.320572821	0.0361325	+	A	G	0.3318	0.0393
rs7873077	LC_IGP_R88	IgG4_G2F/IgG4_G1F	9	33142258	2	7.67E-12	3.55E-11	-	-0.249163	-0.2595	+	A	C	0.320592626	0.0361378	+	A	C	0.3306	0.0392
rs7873903	LC_IGP_R88	IgG4_G2F/IgG4_G1F	9	33142728	2	7.99E-12	3.52E-11	-	-0.249104	-0.2598	+	A	G	0.320687995	0.0361605	+	A	G	0.3305	0.0392
rs7036812	LC_IGP_R88	IgG4_G2F/IgG4_G1F	9	33143822	2	8.35E-12	3.36E-11	-	-0.249007	-0.2604	+	C	T	0.320811091	0.0361801	+	C	T	0.3303	0.0393
rs3824458	LC_IGP_R88	IgG4_G2F/IgG4_G1F	9	33144809	2	8.83E-12	6.33E-11	-	-0.248815	-0.2571	+	T	C	0.320969531	0.0361953	+	T	C	0.3311	0.0393
chr9:33145647:I	LC_IGP_R88	IgG4_G2F/IgG4_G1F	9	33145647	2	4.48E-11	4.29E-10	-	-0.23875	-0.2452	+	CA	C	0.329182511	0.0359982	+	CA	C	0.3351	0.0393
rs4584234	LC_IGP_R88	IgG4_G2F/IgG4_G1F	9	33147479	2	5.13E-11	4.74E-10	-	-0.238134	-0.2452	+	A	G	0.328226082	0.036017	+	A	G	0.3348	0.0394

rs6476402	LC_IGP_R88	IgG4_G2F/IgG4_G1F	9	33148552	2	5.65E-11	4.61E-10	-	-0.237659	-0.2455	+	A	G	0.328088361	0.0360261	+	A	G	0.3338	0.0394
chr9:33152337:D	LC_IGP_R88	IgG4_G2F/IgG4_G1F	9	33152337	2	1.75E-14	4.68E-14	-	-0.297269	-0.317	+	C	CAT	0.259901889	0.0384145	+	C	CAT	0.2654	0.042
rs112906036	LC_IGP_R88	IgG4_G2F/IgG4_G1F	9	33152622	2	1.52E-09	3.74E-13	-	-0.262577	-0.3544	+	T	C	0.194435376	0.0432084	+	T	C	0.1801	0.0488
rs10813957	LC_IGP_R88	IgG4_G2F/IgG4_G1F	9	33153527	2	2.66E-14	9.62E-14	-	-0.294297	-0.3124	+	T	G	0.261872943	0.0383054	+	T	G	0.2668	0.042
rs3780480	LC_IGP_R88	IgG4_G2F/IgG4_G1F	9	33163486	2	6.63E-14	5.28E-14	-	-0.290257	-0.317	+	T	G	0.263389092	0.0383898	+	T	G	0.2654	0.0421
rs10971434	LC_IGP_R88	IgG4_G2F/IgG4_G1F	9	33168891	2	3.12E-14	2.84E-14	-	-0.29468	-0.3227	+	A	C	0.263136502	0.038461	+	A	C	0.2682	0.0424
rs10758195	LC_IGP_R88	IgG4_G2F/IgG4_G1F	9	33174587	2	9.59E-14	4.11E-14	-	-0.292712	-0.325	+	T	C	0.265958704	0.0389706	+	T	C	0.2691	0.043
rs28517717	LC_IGP_R88	IgG4_G2F/IgG4_G1F	9	33179451	2	2.16E-10	7.48E-11	-	-0.248812	-0.2758	+	T	C	0.282707495	0.0389379	+	T	C	0.2849	0.0424
rs10971438	LC_IGP_R88	IgG4_G2F/IgG4_G1F	9	33180308	2	3.88E-10	1.70E-10	-	-0.244423	-0.2702	+	G	A	0.284714199	0.0388127	+	G	A	0.2854	0.0423
rs10813960	LC_IGP_R88	IgG4_G2F/IgG4_G1F	9	33180362	2	2.40E-10	8.94E-11	-	-0.248571	-0.2754	+	T	C	0.27963376	0.039001	+	T	C	0.2839	0.0425
rs10971439	LC_IGP_R88	IgG4_G2F/IgG4_G1F	9	33180813	2	5.01E-10	1.30E-12	-	-0.269335	-0.3509	+	C	T	0.207957412	0.0430477	+	C	T	0.1883	0.0495
rs6476398	LC_IGP_R89	IgG4_G2FS1/IgG4_G1FS1	9	33119241	2	1.40E-14	1.33E-17	-	-0.282422	-0.3367	+	T	C	0.318754534	0.0363602	+	T	C	0.3236	0.0394
rs7859454	LC_IGP_R89	IgG4_G2FS1/IgG4_G1FS1	9	33119583	2	1.06E-11	6.70E-15	-	-0.291685	-0.3646	+	A	T	0.198419936	0.0425955	+	A	T	0.1798	0.0468
rs10121987	LC_IGP_R89	IgG4_G2FS1/IgG4_G1FS1	9	33119818	2	1.35E-14	5.02E-18	-	-0.282082	-0.3415	+	G	A	0.318920683	0.0362919	+	G	A	0.3275	0.0395
rs1969977	LC_IGP_R89	IgG4_G2FS1/IgG4_G1FS1	9	33120203	2	8.19E-17	3.60E-19	-	-0.341694	-0.3954	+	G	A	0.235113179	0.040587	+	G	A	0.2419	0.0442
rs76344840	LC_IGP_R89	IgG4_G2FS1/IgG4_G1FS1	9	33120204	2	8.59E-14	4.96E-17	-	-0.283452	-0.3464	+	G	A	0.29687889	0.0376632	+	G	A	0.3144	0.0413
rs3818593	LC_IGP_R89	IgG4_G2FS1/IgG4_G1FS1	9	33120706	2	9.85E-12	5.37E-15	-	-0.291101	-0.3633	+	G	A	0.198659281	0.0424457	+	G	A	0.1808	0.0465
rs10971417	LC_IGP_R89	IgG4_G2FS1/IgG4_G1FS1	9	33121480	2	1.86E-17	4.52E-20	-	-0.329451	-0.3848	+	T	C	0.260006406	0.0383167	+	T	C	0.2577	0.0419
rs10813948	LC_IGP_R89	IgG4_G2FS1/IgG4_G1FS1	9	33121570	2	1.30E-14	1.05E-17	-	-0.281603	-0.3363	+	A	G	0.319134838	0.036207	+	A	G	0.3244	0.0392
rs10971418	LC_IGP_R89	IgG4_G2FS1/IgG4_G1FS1	9	33122024	2	9.79E-12	5.07E-15	-	-0.291095	-0.3641	+	A	C	0.198668947	0.0424387	+	A	C	0.1807	0.0465
rs10116966	LC_IGP_R89	IgG4_G2FS1/IgG4_G1FS1	9	33122370	2	1.39E-14	8.93E-18	-	-0.280863	-0.337	+	A	T	0.319500915	0.0361521	+	A	T	0.3246	0.0392
rs10113903	LC_IGP_R89	IgG4_G2FS1/IgG4_G1FS1	9	33122645	2	1.37E-14	1.04E-17	-	-0.280909	-0.336	+	C	G	0.319592434	0.0361522	+	C	G	0.3254	0.0392
rs6476400	LC_IGP_R89	IgG4_G2FS1/IgG4_G1FS1	9	33123106	2	4.70E-14	7.98E-18	-	-0.27451	-0.339	+	A	T	0.322200929	0.0360862	+	A	T	0.3275	0.0394
rs10813949	LC_IGP_R89	IgG4_G2FS1/IgG4_G1FS1	9	33123464	2	1.38E-14	9.04E-18	-	-0.280857	-0.337	+	A	G	0.319508847	0.036149	+	A	G	0.3249	0.0392
rs10738905	LC_IGP_R89	IgG4_G2FS1/IgG4_G1FS1	9	33123627	2	1.44E-14	1.06E-17	-	-0.280695	-0.3361	+	G	A	0.319637584	0.0361536	+	G	A	0.3257	0.0392
rs10971419	LC_IGP_R89	IgG4_G2FS1/IgG4_G1FS1	9	33124161	2	1.14E-14	9.40E-18	-	-0.280504	-0.3341	+	C	T	0.319964002	0.0359847	+	C	T	0.3271	0.0389
rs12342831	LC_IGP_R89	IgG4_G2FS1/IgG4_G1FS1	9	33124872	2	1.79E-17	2.96E-20	-	-0.327143	-0.3813	+	C	T	0.261126297	0.0380268	+	C	T	0.2631	0.0414
rs10971420	LC_IGP_R89	IgG4_G2FS1/IgG4_G1FS1	9	33125000	2	1.13E-14	9.40E-18	-	-0.280508	-0.3341	+	C	T	0.319965223	0.0359845	+	C	T	0.3271	0.0389
rs10758189	LC_IGP_R89	IgG4_G2FS1/IgG4_G1FS1	9	33125804	2	1.06E-14	1.55E-17	-	-0.280746	-0.332	+	C	T	0.319975595	0.0359759	+	C	T	0.328	0.039
rs3780495	LC_IGP_R89	IgG4_G2FS1/IgG4_G1FS1	9	33125942	2	1.05E-14	1.88E-17	-	-0.280805	-0.3314	+	C	A	0.31997834	0.0359736	+	C	A	0.3281	0.039
rs7850878	LC_IGP_R89	IgG4_G2FS1/IgG4_G1FS1	9	33126570	2	2.17E-14	2.53E-17	-	-0.278063	-0.3315	+	G	A	0.31859701	0.0360668	+	G	A	0.327	0.0392
rs10813950	LC_IGP_R89	IgG4_G2FS1/IgG4_G1FS1	9	33127640	2	1.01E-14	2.93E-17	-	-0.280931	-0.3293	+	A	G	0.319983527	0.0359684	+	A	G	0.3295	0.039
rs10813951	LC_IGP_R89	IgG4_G2FS1/IgG4_G1FS1	9	33128021	2	1.67E-17	8.52E-20	-	-0.327324	-0.377	+	G	A	0.261129957	0.0380099	+	G	A	0.2647	0.0414
rs13297246	LC_IGP_R89	IgG4_G2FS1/IgG4_G1FS1	9	33128617	2	8.92E-10	1.34E-17	+	0.278293	0.4298	+	A	G	0.164831404	0.0451494	+	A	G	0.1602	0.0503
rs3780490	LC_IGP_R89	IgG4_G2FS1/IgG4_G1FS1	9	33129839	2	1.00E-14	2.63E-17	-	-0.281009	-0.3293	+	G	A	0.319913362	0.0359745	+	G	A	0.3286	0.0389
rs3780489	LC_IGP_R89	IgG4_G2FS1/IgG4_G1FS1	9	33129844	2	1.01E-14	2.63E-17	-	-0.280938	-0.3293	+	T	G	0.319984137	0.0359681	+	T	G	0.3286	0.0389
rs10758192	LC_IGP_R89	IgG4_G2FS1/IgG4_G1FS1	9	33131651	2	1.01E-14	1.11E-16	-	-0.280943	-0.3223	+	A	G	0.319983832	0.0359679	+	A	G	0.3303	0.0389
rs12554995	LC_IGP_R89	IgG4_G2FS1/IgG4_G1FS1	9	33132044	2	1.01E-14	1.16E-16	-	-0.280945	-0.3222	+	G	A	0.319984442	0.0359678	+	G	A	0.3303	0.0389
rs56687083	LC_IGP_R89	IgG4_G2FS1/IgG4_G1FS1	9	33132134	2	1.01E-14	1.14E-16	-	-0.280945	-0.3231	+	G	A	0.319984442	0.0359678	+	G	A	0.3296	0.039
chr9:33132215:I	LC_IGP_R89	IgG4_G2FS1/IgG4_G1FS1	9	33132215	2	8.83E-12	1.09E-14	-	-0.28881	-0.36	+	TG	T	0.199812629	0.0420128	+	TG	T	0.1853	0.0466
rs73645257	LC_IGP_R89	IgG4_G2FS1/IgG4_G1FS1	9	33132354	2	8.83E-12	4.98E-15	-	-0.28881	-0.3639	+	T	C	0.199812629	0.0420128	+	T	C	0.1848	0.0465
rs73484568	LC_IGP_R89	IgG4_G2FS1/IgG4_G1FS1	9	33132492	2	1.01E-14	1.17E-16	-	-0.280946	-0.3222	+	A	G	0.319984442	0.0359677	+	A	G	0.3303	0.0389
rs3780488	LC_IGP_R89	IgG4_G2FS1/IgG4_G1FS1	9	33133812	2	1.00E-14	1.21E-16	-	-0.280952	-0.322	+	C	T	0.319983832	0.0359675	+	C	T	0.3303	0.0389
rs913214	LC_IGP_R89	IgG4_G2FS1/IgG4_G1FS1	9	33135085	2	1.00E-14	1.22E-16	-	-0.280962	-0.3219	+	A	G	0.319984137	0.0359671	+	A	G	0.3304	0.0389
rs10738906	LC_IGP_R89	IgG4_G2FS1/IgG4_G1FS1	9	33135634	2	9.99E-15	1.41E-16	-	-0.280972	-0.3213	+	C	T	0.319985052	0.0359667	+	C	T	0.3305	0.0389
rs10738907	LC_IGP_R89	IgG4_G2FS1/IgG4_G1FS1	9	33135890	2	1.74E-14	2.55E-15	-	-0.281369	-0.3227	+	A	T	0.313705003	0.0363558	+	A	T	0.3167	0.0408
rs10124479	LC_IGP_R89	IgG4_G2FS1/IgG4_G1FS1	9	33136233	2	9.95E-15	2.07E-16	-	-0.280985	-0.32	+	G	T	0.319985357	0.0359662	+	G	T	0.3317	0.0389
rs10813954	LC_IGP_R89	IgG4_G2FS1/IgG4_G1FS1	9	33137596	2	9.87E-15	1.92E-16	-	-0.281015	-0.3198	+	C	A	0.319987492	0.0359652	+	C	A	0.3308	0.0389
rs2209948	LC_IGP_R89	IgG4_G2FS1/IgG4_G1FS1	9	33138247	2	9.88E-15	1.95E-16	-	-0.281013	-0.3199	+	A	G	0.319995424	0.0359654	+	A	G	0.3308	0.0389
rs7849148	LC_IGP_R89	IgG4_G2FS1/IgG4_G1FS1	9	33138352	2	9.87E-15	1.90E-16	-	-0.281016	-0.3199	+	T	A	0.31999939	0.0359654	+	T	A	0.3308	0.0389
rs10971424	LC_IGP_R89	IgG4_G2FS1/IgG4_G1FS1	9	33138775	2	9.87E-15	1.91E-16	-	-0.281017	-0.3199	+	C	T	0.320002441	0.0359655	+	C	T	0.3308	0.0389
rs3780487	LC_IGP_R89	IgG4_G2FS1/IgG4_G1FS1	9	33139237	2	9.87E-15	1.91E-16	-	-0.281017	-0.32	+	C	T	0.320007627	0.0359655	+	C	T	0.3308	0.0389
rs3780486	LC_IGP_R89	IgG4_G2FS1/IgG4_G1FS1	9	33139453	2	1.64E-17	1.81E-19	-	-0.327357	-0.3752	+	T	C	0.261134838	0.0380068	+	T	C	0.2662	0.0416
rs7864705	LC_IGP_R89	IgG4_G2FS1/IgG4_G1FS1	9	33140352	2	1.22E-14	1.90E-16	-	-0.280129	-0.3201	+	C	A	0.320130262	0.0359776	+	C	A	0.3307	0.0389
rs7865745	LC_IGP_R89	IgG4_G2FS1/IgG4_G1FS1	9	33140976	2	1.32E-14	1.89E-16	-	-0.279748	-0.3202	+	A	G	0.320176937	0.0359806	+	A	G	0.3307	0.0389
rs7868781	LC_IGP_R89	IgG4_G2FS1/IgG4_G1FS1	9	33141320	2	8.07E-15	1.83E-16	-	-0.283139	-0.3249	+	G	A	0.316877669	0.0361156	+	G	A	0.3242	0.0395
rs10813955	LC_IGP_R89	IgG4_G2FS1/IgG4_G1FS1	9	33141791	2	2.27E-14	1.90E-16	-	-0.277319	-0.3203	+	T	C	0.320622331	0.0359955	+	T	C	0.3307	0.0389
rs7873182	LC_IGP_R89	IgG4_G2FS1/IgG4_G1FS1	9	33142170	2	1.70E-14	7.39E-17	-	-0.278713	-0.3257	+	A	G	0.320353874	0.0360005	+	A	G	0.3318	0.039
rs7873077	LC_IGP_R89	IgG4_G2FS1/IgG4_G1FS1	9	33142258	2	1.73E-14	1.87E-16	-	-0.278691	-0.3206	+	A	C	0.320373703	0.0360058	+	A	C	0.3306	0.039
rs7873903	LC_IGP_R89	IgG4_G2FS1/IgG4_G1FS1	9	33142728	2	1.83E-14	1.86E-16	-	-0.278585	-0.3211	+	A	G	0.320469189	0.0360289	+	A	G	0.3305	0.039
rs7036812	LC_IGP_R89	IgG4_G2FS1/IgG4_G1FS1	9	33143822	2	1.98E-14	1.71E-16	-	-0.278373	-0.3218	+	C	T	0.320592739	0.0360491	+	C	T	0.3303	0.039
rs3824458	LC_IGP_R89	IgG4_G2FS1/IgG4_G1FS1	9	33144809	2	2.10E-14	3.45E-16	-	-0.2782	-0.3191	+	T	C	0.320751373	0.0360645	+	T	C	0.3311	0.0391
chr9:33145647:I	LC_IGP_R89	IgG4_G2FS1/IgG4_G1FS1	9	33145647	2	6.46E-14	3.42E-15	-	-0.271233	-0.3078	+	CA	C	0.32897712	0.0358566	+	CA	C	0.3351	0.0391
rs4584234	LC_IGP_R89	IgG4_G2FS1/IgG4_G1FS1	9	33147479	2	5.97E-14	3.30E-15	-	-0.271725	-0.3084	+	A	G	0.328016473	0.035871	+	A	G	0.3348	0.0391
rs6476402	LC_IGP_R89	IgG4_G2FS1/IgG4_G1FS1	9	33148552	2	6.58E-14	3.40E-15	-	-0.271324	-0.3085	+	A	G	0.327878585	0.03588	+	A	G	0.3338	0.0392
chr9:33152337:D	LC_IGP_R89	IgG4_G2FS1/IgG4_G1FS1	9	33152337	2	2.46E-17	1.48E-19	-	-0.327776	-0.3781	+	C	CAT	0.259608908	0.0382697	+	C	CAT	0.2654	0.0418
rs112906036	LC_IGP_R89	IgG4_G2FS1/IgG4_G1FS1	9	33152622	2	4.79E-11	1.32E-14	-	-0.285638	-0.3725	+	T	C	0.194369827	0.0431334	+	T	C	0.1801	0.0484
rs10813957	LC_IGP_R89	IgG4_G2FS1/IgG4_G1FS1	9	33153527	2	2.47E-17	2.74E-19	-	-0.326728	-0.3748	+	T	G	0.261582367	0.0381513	+	T	G	0.2668	0.0417
rs3780480	LC_IGP_R89	IgG4_G2FS1/IgG4_G1FS1	9	33163486	2	1.07E-16	3.07E-19	-	-0.320746	-0.3757	+	T	G	0.263100061	0.0382481	+	T	G	0.2654	0.0419
rs10971434	LC_IGP_R89	IgG4_G2FS1/IgG4_G1FS1	9	33168891	2	3.17E-17	1.19E-19	-	-0.326925	-0.3822	+	A	C	0.262847468	0.0383082	+	A	C	0.2682	0.0421
rs10758195	LC_IGP_R89	IgG4_G2FS1/IgG4_G1FS1	9	33174587	2	8.86E-17	1.54E-19	-	-0.326394	-0.386	+	T	C	0.265670979	0.038814	+	T	C	0.2691	0.0427
rs28517717	LC_IGP_R89	IgG4_G2FS1/IgG4_G1FS1	9	33179451	2	3.12E-12	1.63E-16	-	-0.272903	-0.3468	+	T	C	0.282439292	0.0388445	+	T	C	0.2849	0.042
rs10971438	LC_IGP_R89	IgG4_G2FS1/IgG4_G1FS1	9	33180308	2	1.06E-11	3.91E-16	-	-0.265218	-0.3419	+	G	A	0.284448444	0.0387342	+	G	A	0.2854	0.042
rs10813960	LC_IGP_R89	IgG4_G2FS1/IgG4_G1FS1	9	33180362	2	2.26E-12	1.84E-16	-	-0.275074	-0.3471	+	T	C	0.279362111	0.0388978	+	T	C	0.2839	0.0422
rs10971439	LC_IGP_R89	IgG4_G2FS1/IgG4_G1FS1	9	33180813	2	2.78E-11	5.54E-15	-	-0.288198	-0.3827	+	C	T	0.207906261	0.0429873	+	C	T	0.1883	0.049
rs12337458	LC_IGP109	IgG2 Galactosylation	9	33180566	2	4.19E-08	1.84E-10	+	0.888025	0.9873	+	C	T	0.013415195	0.161192	+	C	T	0.0133	0.1549
rs28429368	LC_IGP133	IgG2_G1Fn	9	33125359	2	9.81E-09	3.73E-13	+	0.767787	0.9992	+	T	C	0.017218851	0.133202	+	T	C	0.0158	0.1375
rs3780494	LC_IGP133	IgG2_G1Fn	9	33126224	2	1.01E-08	3.99E-13	+	0.765103	1.0013	+	C	T	0.017241379	0.132857	+	C	T	0.0157	0.138
rs73484569	LC_IGP133	IgG2_G1Fn	9	33133191	2	1.01E-08	7.57E-14	+	0.765131	0.9872	+	A	G	0.017241082	0.132861	+	A	G	0.0173	0.132
rs3824459	LC_IGP133	IgG2_G1Fn	9	33134617	2	1.01E-08	1.84E-12	+	0.765131	0.8623	+	T	C	0.017241082	0.132861	+	T	C	0.0205	0.1224
rs73472094	LC_IGP133	IgG2_G1Fn	9	33135537	2	1.01E-08	7.70E-14	+	0.765149	0.9872	+	A	G	0.017241379	0.132861	+	A	G	0.0173	0.1321
rs12346594	LC_IGP133	IgG2_G1Fn	9	33136536	2	1.01E-08	1.29E-13	+	0.765149	0.9727	+	A	G	0.017241379	0.132861	+	A	G	0.0184	0.1313
rs3780485	LC_IGP133	IgG2_G1Fn	9	33139618	2	9.77E-09	9.11E-14	+	0.765919	0.9863	+	C	T	0.017254289	0.132861	+	C	T	0.0173	0.1323
rs10121006	LC_IGP133	IgG2_G1Fn	9	33141250	2	9.70E-09	8.96E-14	+	0.766111	0.9869	+	T	C	0.017257665	0.132867	+	T	C	0.0173	0.1324
rs7020500	LC_IGP133	IgG2_G1Fn	9	33143733	2	9.60E-09	6.71E-14	+	0.770712	0.9952	+	C	G	0.017193031	0.133623	+	C	G	0.0172	0.1328
rs113197944	LC_IGP133	IgG2_G1Fn	9	33149340	2	8.40E-09	6.30E-14	+	0.780493	0.9974	+	A	G	0.017096664	0.134782	+	A	G	0.0172	0.133
rs1328899	LC_IGP133	IgG2_G1Fn	9	33151415	2	1.26E-08	4.13E-14	+	0.743891	1.0023	+	T	C	0.01907298	0.13003	+	T	C	0.0176	0.1326
rs12343079	LC_IGP133	IgG2_G1Fn	9	33158557	2	8.41E-09	1.16E-13	+	0.780682	1.0004	+	A	G	0.01709383	0.134821	+	A	G	0.0162	0.1348
rs3780481	LC_IGP133	IgG2_G1Fn	9	33163298	2	8.40E-09	1.22E-13	+	0.780825	0.9997	+	T	C	0.017092091	0.134841	+	T	C	0.0162	0.1348
rs3780478	LC_IGP133	IgG2_G1Fn	9	33164116	2	8.40E-09	1.15E-13	+	0.781018	1.0007	+	A	T	0.017090191	0.134873	+	A	T	0.0162	0.1348
rs73472938	LC_IGP133	IgG2_G1Fn	9	33164527	2	8.40E-09	1.16E-13	+	0.781086	1.0008	+	A	T	0.017090243	0.134884	+	A	T	0.0162	0.1348
rs3758273	LC_IGP133	IgG2_G1Fn	9	33168309	2	3.11E-08	1.14E-13	+	0.779868	1.003	+	A	C	0.016161668	0.140214	+	A	C	0.0162	0.1351
rs12337458	LC_IGP133	IgG2_G1Fn	9	33180566	2	2.23E-08	3.81E-11	+	0.897921	1.0022	+	C	T	0.013596775	0.159751	+	C	T	0.0132	0.1516
rs12337458	LC_IGP134	IgG2_G2Fn	9	33180566	2	2.27E-08	1.34E-09	+	0.893606	0.9584	+	C	T	0.013596775	0.159068	+	C	T	0.0132	0.1581
rs28429368	LC_IGP144	IgG2 G0n	9	33125359	2	3.15E-08	4.18E-12	-	-0.741297	-0.9407	+	T	C	0.017218851	0.133321	+	T	C	0.0158	0.1357
rs3780494	LC_IGP144	IgG2 G0n	9	33126224	2	3.19E-08	4.36E-12	-	-0.739051	-0.9433	+	C	T	0.017241379	0.132975	+	C	T	0.0157	0.1362
rs73484569	LC_IGP144	IgG2 G0n	9	33133191	2	3.18E-08	1.27E-13	-	-0.739125	-0.967	+	A	G	0.017241082	0.132979	+	A	G	0.0173	0.1305
rs3824459	LC_IGP144	IgG2 G0n	9	33134617	2	3.18E-08	4.94E-14	-	-0.739125	-0.924	+	T	C	0.017241082	0.132979	+	T	C	0.0205	0.1226
rs73472094	LC_IGP144	IgG2 G0n	9	33135537	2	3.18E-08	1.28E-13	-	-0.739157	-0.9671	+	A	G	0.017241379	0.132979	+	A	G	0.0173	0.1305
rs12346594	LC_IGP144	IgG2 G0n	9	33136536	2	3.18E-08	1.87E-13	-	-0.739157	-0.9612	+	A	G	0.017241379	0.132979	+	A	G	0.0184	0.1306
rs3780485	LC_IGP144	IgG2 G0n	9	33139618	2	3.02E-08	1.59E-13	-	-0.740313	-0.9674	+	C	T	0.017254289	0.132978	+	C	T	0.0173	0.1311
rs10121006	LC_IGP144	IgG2 G0n	9	33141250	2	2.98E-08	1.57E-13	-	-0.740659	-0.9681	+	T	C	0.017257665	0.132984	+	T	C	0.0173	0.1312
rs7020500	LC_IGP144	IgG2 G0n	9	33143733	2	2.87E-08	1.35E-13	-	-0.745776	-0.9764	+	C	G	0.017193031	0.133738	+	C	G	0.0172	0.1319
rs113197944	LC_IGP144	IgG2 G0n	9	33149340	2	2.72E-08	1.31E-13	-	-0.753609	-0.9787	+	A	G	0.017096664	0.134904	+	A	G	0.0172	0.1322
rs1328899	LC_IGP144	IgG2 G0n	9	33151415	2	3.04E-08	1.21E-13	-	-0.724291	-0.9792	+	T	C	0.01907298	0.130125	+	T	C	0.0176	0.132
rs12343079	LC_IGP144	IgG2 G0n	9	33158557	2	2.71E-08	2.16E-13	-	-0.753853	-0.9939	+	A	G	0.01709383	0.134943	+	A	G	0.0162	0.1354
rs3780481	LC_IGP144	IgG2 G0n	9	33163298	2	2.71E-08	2.28E-13	-	-0.75402	-0.9931	+	T	C	0.017092091	0.134962	+	T	C	0.0162	0.1355
rs3780478	LC_IGP144	IgG2 G0n	9	33164116	2	2.71E-08	2.17E-13	-	-0.754177	-0.9942	+	A	T	0.017090191	0.134995	+	A	T	0.0162	0.1355
rs73472938	LC_IGP144	IgG2 G0n	9	33164527	2	2.71E-08	2.17E-13	-	-0.754246	-0.9943	+	A	T	0.017090243	0.135006	+	A	T	0.0162	0.1355
rs12337458	LC_IGP144	IgG2 G0n	9	33180566	2	1.50E-08	1.60E-11	-	-0.909041	-1.0324	+	C	T	0.013596775	0.159745	+	C	T	0.0132	0.1532
rs28429368	LC_IGP145	IgG2 G1n	9	33125359	2	8.84E-09	1.33E-13	+	0.771309	1.0111	+	T	C	0.017218851	0.133397	+	T	C	0.0158	0.1366
rs3780494	LC_IGP145	IgG2 G1n	9	33126224	2	8.89E-09	1.30E-13	+	0.769156	1.0143	+	C	T	0.017241379	0.13305	+	C	T	0.0157	0.137
rs73484569	LC_IGP145	IgG2 G1n	9	33133191	2	8.88E-09	9.71E-15	+	0.769211	1.0157	+	A	G	0.017241082	0.133055	+	A	G	0.0173	0.1312
rs3824459	LC_IGP145	IgG2 G1n	9	33134617	2	8.88E-09	3.67E-14	+	0.769211	0.929	+	T	C	0.017241082	0.133055	+	T	C	0.0205	0.1227
rs73472094	LC_IGP145	IgG2 G1n	9	33135537	2	8.87E-09	9.83E-15	+	0.769234	1.0158	+	A	G	0.017241379	0.133055	+	A	G	0.0173	0.1312
rs12346594	LC_IGP145	IgG2 G1n	9	33136536	2	8.87E-09	1.31E-14	+	0.769234	1.0015	+	A	G	0.017241379	0.133055	+	A	G	0.0184	0.13
rs3780485	LC_IGP145	IgG2 G1n	9	33139618	2	8.46E-09	1.19E-14	+	0.770303	1.0151	+	C	T	0.017254289	0.133053	+	C	T	0.0173	0.1315
rs10121006	LC_IGP145	IgG2 G1n	9	33141250	2	8.35E-09	1.17E-14	+	0.770632	1.0157	+	T	C	0.017257665	0.133059	+	T	C	0.0173	0.1316
rs7020500	LC_IGP145	IgG2 G1n	9	33143733	2	8.16E-09	8.58E-15	+	0.775541	1.0232	+	C	G	0.017193031	0.133815	+	C	G	0.0172	0.1319

rs113197944	LC_IGP145	IgG2 G1n	9	33149340	2	7.61E-09	8.03E-15	+	0.783915	1.0251	+	A	G	0.017096664	0.13498	+	A	G	0.0172	0.132
rs1328899	LC_IGP145	IgG2 G1n	9	33151415	2	9.44E-09	1.03E-14	+	0.751383	1.0225	+	T	C	0.01907298	0.130207	+	T	C	0.0176	0.1322
rs12343079	LC_IGP145	IgG2 G1n	9	33158557	2	7.60E-09	1.71E-14	+	0.78416	1.0194	+	A	G	0.01709383	0.135019	+	A	G	0.0162	0.1329
rs3780481	LC_IGP145	IgG2 G1n	9	33163298	2	7.59E-09	1.80E-14	+	0.784308	1.0187	+	T	C	0.017092091	0.135039	+	T	C	0.0162	0.1329
rs3780478	LC_IGP145	IgG2 G1n	9	33164116	2	7.60E-09	1.71E-14	+	0.784466	1.0197	+	A	T	0.017090191	0.135072	+	A	T	0.0162	0.1329
rs73472938	LC_IGP145	IgG2 G1n	9	33164527	2	7.60E-09	1.72E-14	+	0.784551	1.0198	+	A	T	0.017090243	0.135083	+	A	T	0.0162	0.133
rs3758273	LC_IGP145	IgG2 G1n	9	33168309	2	3.35E-08	1.81E-14	+	0.779276	1.0216	+	A	C	0.016161668	0.140435	+	A	C	0.0162	0.1333
rs12337458	LC_IGP145	IgG2 G1n	9	33180566	2	1.15E-08	2.64E-11	+	0.91755	1.0133	+	C	T	0.013596775	0.159932	+	C	T	0.0132	0.152
rs1969977	LC_IGP175	IgG4_G2F	9	33120203	2	2.99E-08	1.67E-07	-	-0.228542	-0.2378	+	G	A	0.235428093	0.0410406	+	G	A	0.2419	0.0454
rs12342831	LC_IGP175	IgG4_G2F	9	33124872	2	4.77E-08	3.24E-08	-	-0.211195	-0.2336	+	C	T	0.261417428	0.0385021	+	C	T	0.2631	0.0422
rs10813951	LC_IGP175	IgG4_G2F	9	33128021	2	4.54E-08	4.50E-08	-	-0.211457	-0.2318	+	G	A	0.261421085	0.0384855	+	G	A	0.2647	0.0424
rs3780486	LC_IGP175	IgG4_G2F	9	33139453	2	4.50E-08	4.23E-08	-	-0.211497	-0.2331	+	T	C	0.26142596	0.0384824	+	T	C	0.2662	0.0425
chr9:33152337:D	LC_IGP175	IgG4_G2F	9	33152337	2	4.97E-08	3.37E-08	-	-0.212232	-0.236	+	C	CAT	0.259901889	0.0387412	+	C	CAT	0.2654	0.0428
rs6476398	LC_IGP180	IgG4_G2FS1	9	33119241	2	1.01E-09	3.20E-14	-	-0.225941	-0.2971	+	T	C	0.318754534	0.0367771	+	T	C	0.3236	0.0392
rs7859454	LC_IGP180	IgG4_G2FS1	9	33119583	2	2.97E-08	3.06E-10	-	-0.239517	-0.2929	+	A	T	0.198419936	0.0429993	+	A	T	0.1798	0.0465
rs10121987	LC_IGP180	IgG4_G2FS1	9	33119818	2	1.01E-09	1.40E-14	-	-0.225533	-0.3012	+	G	A	0.318920683	0.0367089	+	G	A	0.3275	0.0391
rs1969977	LC_IGP180	IgG4_G2FS1	9	33120203	2	3.47E-11	3.35E-15	-	-0.274142	-0.3466	+	G	A	0.235113179	0.0410969	+	G	A	0.2419	0.044
rs76344840	LC_IGP180	IgG4_G2FS1	9	33120204	2	1.21E-09	1.15E-13	-	-0.232682	-0.3054	+	G	A	0.29687889	0.0380577	+	G	A	0.3144	0.0412
rs3818593	LC_IGP180	IgG4_G2FS1	9	33120706	2	2.90E-08	2.49E-10	-	-0.238847	-0.2924	+	G	A	0.198659281	0.0428493	+	G	A	0.1808	0.0462
rs10971417	LC_IGP180	IgG4_G2FS1	9	33121480	2	7.31E-11	1.30E-15	-	-0.254763	-0.3333	+	T	C	0.260006406	0.03885	+	T	C	0.2577	0.0417
rs10813948	LC_IGP180	IgG4_G2FS1	9	33121570	2	1.02E-09	2.65E-14	-	-0.224958	-0.2969	+	A	G	0.319134838	0.036624	+	A	G	0.3244	0.039
rs10971418	LC_IGP180	IgG4_G2FS1	9	33122024	2	2.89E-08	2.36E-10	-	-0.238843	-0.2931	+	A	C	0.198668947	0.0428424	+	A	C	0.1807	0.0463
rs10116966	LC_IGP180	IgG4_G2FS1	9	33122370	2	1.06E-09	2.26E-14	-	-0.224391	-0.2977	+	A	T	0.319500915	0.0365678	+	A	T	0.3246	0.039
rs10113903	LC_IGP180	IgG4_G2FS1	9	33122645	2	1.02E-09	2.43E-14	-	-0.224602	-0.2974	+	C	G	0.319592434	0.0365674	+	C	G	0.3254	0.039
rs6476400	LC_IGP180	IgG4_G2FS1	9	33123106	2	2.43E-09	2.55E-14	-	-0.218932	-0.2985	+	A	T	0.322200929	0.0364926	+	A	T	0.3275	0.0392
rs10813949	LC_IGP180	IgG4_G2FS1	9	33123464	2	1.06E-09	2.30E-14	-	-0.224381	-0.2975	+	A	G	0.319508847	0.0365648	+	A	G	0.3249	0.039
rs10738905	LC_IGP180	IgG4_G2FS1	9	33123627	2	1.04E-09	2.59E-14	-	-0.224464	-0.2972	+	G	A	0.319637584	0.0365682	+	G	A	0.3257	0.039
rs10971419	LC_IGP180	IgG4_G2FS1	9	33124161	2	9.37E-10	1.60E-14	-	-0.224075	-0.2972	+	C	T	0.319964002	0.0364003	+	C	T	0.3271	0.0387
rs12342831	LC_IGP180	IgG4_G2FS1	9	33124872	2	7.13E-11	4.01E-16	-	-0.252986	-0.3346	+	C	T	0.261126297	0.0385565	+	C	T	0.2631	0.0411
rs10971420	LC_IGP180	IgG4_G2FS1	9	33125000	2	9.37E-10	1.59E-14	-	-0.224078	-0.2973	+	C	T	0.319965223	0.0364001	+	C	T	0.3271	0.0387
rs10758189	LC_IGP180	IgG4_G2FS1	9	33125804	2	8.99E-10	2.12E-14	-	-0.224271	-0.296	+	C	T	0.319975595	0.0363919	+	C	T	0.328	0.0387
rs3780495	LC_IGP180	IgG4_G2FS1	9	33125942	2	8.90E-10	2.42E-14	-	-0.224318	-0.2955	+	C	A	0.31997834	0.0363897	+	C	A	0.3281	0.0388
rs7850878	LC_IGP180	IgG4_G2FS1	9	33126570	2	1.39E-09	2.84E-14	-	-0.222201	-0.2962	+	G	A	0.31859701	0.0364777	+	G	A	0.327	0.0389
rs10813950	LC_IGP180	IgG4_G2FS1	9	33127640	2	8.70E-10	3.30E-14	-	-0.224419	-0.2939	+	A	G	0.319983527	0.0363847	+	A	G	0.3295	0.0388
rs10813951	LC_IGP180	IgG4_G2FS1	9	33128021	2	6.97E-11	7.00E-16	-	-0.253014	-0.3322	+	G	A	0.261129957	0.0385404	+	G	A	0.2647	0.0412
rs3780490	LC_IGP180	IgG4_G2FS1	9	33129839	2	8.81E-10	2.84E-14	-	-0.224387	-0.2943	+	G	A	0.319913362	0.0363913	+	G	A	0.3286	0.0387
rs3780489	LC_IGP180	IgG4_G2FS1	9	33129844	2	8.69E-10	2.84E-14	-	-0.224425	-0.2943	+	T	G	0.319984137	0.0363845	+	T	G	0.3286	0.0387
rs10758192	LC_IGP180	IgG4_G2FS1	9	33131651	2	8.68E-10	1.25E-13	-	-0.224429	-0.2864	+	A	G	0.319983832	0.0363843	+	A	G	0.3303	0.0386
rs12554995	LC_IGP180	IgG4_G2FS1	9	33132044	2	8.68E-10	1.27E-13	-	-0.22443	-0.2864	+	G	A	0.319984442	0.0363842	+	G	A	0.3303	0.0386
rs56687083	LC_IGP180	IgG4_G2FS1	9	33132134	2	8.68E-10	1.29E-13	-	-0.22443	-0.287	+	G	A	0.319984442	0.0363842	+	G	A	0.3296	0.0388
chr9:33132215:I	LC_IGP180	IgG4_G2FS1	9	33132215	2	3.14E-08	9.45E-11	-	-0.235839	-0.2998	+	TG	T	0.199812629	0.0424171	+	TG	T	0.1853	0.0463
rs73645257	LC_IGP180	IgG4_G2FS1	9	33132354	2	3.14E-08	1.05E-10	-	-0.235839	-0.2983	+	T	C	0.199812629	0.0424171	+	T	C	0.1848	0.0462
rs73484568	LC_IGP180	IgG4_G2FS1	9	33132492	2	8.68E-10	1.27E-13	-	-0.224431	-0.2864	+	A	G	0.319984442	0.0363841	+	A	G	0.3303	0.0386
rs3780488	LC_IGP180	IgG4_G2FS1	9	33133812	2	8.67E-10	1.33E-13	-	-0.224434	-0.2861	+	C	T	0.319983832	0.0363839	+	C	T	0.3303	0.0386
rs913214	LC_IGP180	IgG4_G2FS1	9	33135085	2	8.65E-10	1.35E-13	-	-0.224443	-0.286	+	A	G	0.319984137	0.0363835	+	A	G	0.3304	0.0386
rs10738906	LC_IGP180	IgG4_G2FS1	9	33135634	2	8.64E-10	1.54E-13	-	-0.224451	-0.2853	+	C	T	0.319985052	0.0363832	+	C	T	0.3305	0.0386
rs10738907	LC_IGP180	IgG4_G2FS1	9	33135890	2	8.12E-10	1.15E-12	-	-0.227162	-0.2883	+	A	T	0.313705003	0.0367632	+	A	T	0.3167	0.0405
rs10124479	LC_IGP180	IgG4_G2FS1	9	33136233	2	8.62E-10	2.10E-13	-	-0.224463	-0.2842	+	G	T	0.319985357	0.0363826	+	G	T	0.3317	0.0387
rs10813954	LC_IGP180	IgG4_G2FS1	9	33137596	2	8.57E-10	2.12E-13	-	-0.224492	-0.2836	+	C	A	0.319987492	0.0363817	+	C	A	0.3308	0.0386
rs2209948	LC_IGP180	IgG4_G2FS1	9	33138247	2	8.56E-10	2.13E-13	-	-0.224497	-0.2837	+	A	G	0.319995424	0.0363819	+	A	G	0.3308	0.0386
rs7849148	LC_IGP180	IgG4_G2FS1	9	33138352	2	8.55E-10	2.10E-13	-	-0.224503	-0.2837	+	T	A	0.31999939	0.0363819	+	T	A	0.3308	0.0386
rs10971424	LC_IGP180	IgG4_G2FS1	9	33138775	2	8.54E-10	2.11E-13	-	-0.224508	-0.2837	+	C	T	0.320002441	0.0363819	+	C	T	0.3308	0.0386
rs3780487	LC_IGP180	IgG4_G2FS1	9	33139237	2	8.54E-10	2.11E-13	-	-0.224513	-0.2837	+	C	T	0.320007627	0.0363819	+	C	T	0.3308	0.0386
rs3780486	LC_IGP180	IgG4_G2FS1	9	33139453	2	6.95E-11	1.11E-15	-	-0.253011	-0.3311	+	T	C	0.261134838	0.0385375	+	T	C	0.2662	0.0413
rs7864705	LC_IGP180	IgG4_G2FS1	9	33140352	2	1.00E-09	2.09E-13	-	-0.22362	-0.2839	+	C	A	0.320130262	0.0363931	+	C	A	0.3307	0.0387
rs7865745	LC_IGP180	IgG4_G2FS1	9	33140976	2	1.07E-09	2.08E-13	-	-0.223241	-0.2839	+	A	G	0.320176937	0.0363957	+	A	G	0.3307	0.0387
rs7868781	LC_IGP180	IgG4_G2FS1	9	33141320	2	6.17E-10	2.07E-13	-	-0.227345	-0.2883	+	G	A	0.316877669	0.0365311	+	G	A	0.3242	0.0392
rs10813955	LC_IGP180	IgG4_G2FS1	9	33141791	2	1.49E-09	2.05E-13	-	-0.221378	-0.2841	+	T	C	0.320622331	0.0364061	+	T	C	0.3307	0.0387
rs7873182	LC_IGP180	IgG4_G2FS1	9	33142170	2	1.40E-09	1.10E-13	-	-0.221796	-0.2882	+	A	G	0.320353874	0.0364161	+	A	G	0.3318	0.0388
rs7873077	LC_IGP180	IgG4_G2FS1	9	33142258	2	1.44E-09	2.05E-13	-	-0.221668	-0.2844	+	A	C	0.320373703	0.0364218	+	A	C	0.3306	0.0387
rs7873903	LC_IGP180	IgG4_G2FS1	9	33142728	2	1.65E-09	2.02E-13	-	-0.221013	-0.2848	+	A	G	0.320469189	0.0364467	+	A	G	0.3305	0.0388
rs7036812	LC_IGP180	IgG4_G2FS1	9	33143822	2	1.93E-09	1.87E-13	-	-0.220195	-0.2855	+	C	T	0.320592739	0.036469	+	C	T	0.3303	0.0388
rs3824458	LC_IGP180	IgG4_G2FS1	9	33144809	2	2.08E-09	3.75E-13	-	-0.219844	-0.2825	+	T	C	0.320751373	0.0364847	+	T	C	0.3311	0.0389
chr9:33145647:I	LC_IGP180	IgG4_G2FS1	9	33145647	2	8.90E-09	1.70E-12	-	-0.209728	-0.2742	+	CA	C	0.32897712	0.0362814	+	CA	C	0.3351	0.0389
rs4584234	LC_IGP180	IgG4_G2FS1	9	33147479	2	6.29E-09	1.52E-12	-	-0.211924	-0.275	+	A	G	0.328016473	0.0362902	+	A	G	0.3348	0.0389
rs6476402	LC_IGP180	IgG4_G2FS1	9	33148552	2	6.94E-09	9.60E-13	-	-0.211375	-0.2776	+	A	G	0.327878585	0.0362994	+	A	G	0.3338	0.0389
chr9:33152337:D	LC_IGP180	IgG4_G2FS1	9	33152337	2	7.72E-11	8.83E-16	-	-0.254092	-0.334	+	C	CAT	0.259608908	0.0387971	+	C	CAT	0.2654	0.0415
rs10813957	LC_IGP180	IgG4_G2FS1	9	33153527	2	1.09E-10	2.20E-15	-	-0.251289	-0.3288	+	T	G	0.261582367	0.0386849	+	T	G	0.2668	0.0415
rs3780480	LC_IGP180	IgG4_G2FS1	9	33163486	2	3.74E-10	1.27E-15	-	-0.244428	-0.3331	+	T	G	0.263100061	0.0387773	+	T	G	0.2654	0.0416
rs10971434	LC_IGP180	IgG4_G2FS1	9	33168891	2	1.73E-10	5.11E-16	-	-0.249592	-0.3399	+	A	C	0.262847468	0.0388489	+	A	C	0.2682	0.0419
rs10758195	LC_IGP180	IgG4_G2FS1	9	33174587	2	4.73E-10	4.32E-15	-	-0.246631	-0.3329	+	T	C	0.265670979	0.0393611	+	T	C	0.2691	0.0424
rs10971439	LC_IGP180	IgG4_G2FS1	9	33180813	2	3.92E-08	4.40E-11	-	-0.239461	-0.3209	+	C	T	0.207906261	0.0433769	+	C	T	0.1883	0.0487
rs10971420	LC_IGP187	IgG4 FGS1/(FG+FGS1)	9	33125000	2	1.34E-09	2.44E-12	-	-0.221813	-0.2689	+	C	T	0.319965223	0.0363802	+	C	T	0.3271	0.0384
rs10971419	LC_IGP187	IgG4 FGS1/(FG+FGS1)	9	33124161	2	1.34E-09	2.46E-12	-	-0.221813	-0.2689	+	C	T	0.319964002	0.0363804	+	C	T	0.3271	0.0384
rs10113903	LC_IGP187	IgG4 FGS1/(FG+FGS1)	9	33122645	2	1.46E-09	3.38E-12	-	-0.222356	-0.2695	+	C	G	0.319592434	0.0365473	+	C	G	0.3254	0.0387
rs10121987	LC_IGP187	IgG4 FGS1/(FG+FGS1)	9	33119818	2	1.67E-09	3.44E-12	-	-0.222414	-0.2701	+	G	A	0.318920683	0.036692	+	G	A	0.3275	0.0388
rs10758189	LC_IGP187	IgG4 FGS1/(FG+FGS1)	9	33125804	2	1.33E-09	3.62E-12	-	-0.221823	-0.2671	+	C	T	0.319975595	0.0363728	+	C	T	0.328	0.0384
rs10738905	LC_IGP187	IgG4 FGS1/(FG+FGS1)	9	33123627	2	1.49E-09	3.66E-12	-	-0.222214	-0.2692	+	G	A	0.319637584	0.0365482	+	G	A	0.3257	0.0387
rs3780495	LC_IGP187	IgG4 FGS1/(FG+FGS1)	9	33125942	2	1.33E-09	4.28E-12	-	-0.221821	-0.2664	+	C	A	0.31997834	0.0363708	+	C	A	0.3281	0.0385
rs10813949	LC_IGP187	IgG4 FGS1/(FG+FGS1)	9	33123464	2	1.50E-09	4.32E-12	-	-0.222149	-0.2678	+	A	G	0.319508847	0.0365447	+	A	G	0.3249	0.0387
rs10116966	LC_IGP187	IgG4 FGS1/(FG+FGS1)	9	33122370	2	1.51E-09	4.38E-12	-	-0.222157	-0.2679	+	A	T	0.319500915	0.0365477	+	A	T	0.3246	0.0387
rs7850878	LC_IGP187	IgG4 FGS1/(FG+FGS1)	9	33126570	2	1.75E-09	4.74E-12	-	-0.220684	-0.2672	+	G	A	0.31859701	0.0364549	+	G	A	0.327	0.0386
rs6476398	LC_IGP187	IgG4 FGS1/(FG+FGS1)	9	33119241	2	1.70E-09	4.84E-12	-	-0.222719	-0.2684	+	T	C	0.318754534	0.0367605	+	T	C	0.3236	0.0388
rs10813948	LC_IGP187	IgG4 FGS1/(FG+FGS1)	9	33121570	2	1.64E-09	5.17E-12	-	-0.222006	-0.267	+	A	G	0.319134838	0.0366065	+	A	G	0.3244	0.0387
rs6476400	LC_IGP187	IgG4 FGS1/(FG+FGS1)	9	33123106	2	2.72E-09	5.26E-12	-	-0.2181	-0.268	+	A	T	0.322200929	0.0364673	+	A	T	0.3275	0.0388
rs3780490	LC_IGP187	IgG4 FGS1/(FG+FGS1)	9	33129839	2	1.31E-09	5.45E-12	-	-0.221923	-0.2649	+	G	A	0.319913362	0.0363723	+	G	A	0.3286	0.0384
rs3780489	LC_IGP187	IgG4 FGS1/(FG+FGS1)	9	33129844	2	1.32E-09	5.45E-12	-	-0.221822	-0.2649	+	T	G	0.319984137	0.036366	+	T	G	0.3286	0.0384
rs10813950	LC_IGP187	IgG4 FGS1/(FG+FGS1)	9	33127640	2	1.32E-09	6.15E-12	-	-0.221821	-0.2646	+	A	G	0.319983527	0.0363662	+	A	G	0.3295	0.0385
rs76344840	LC_IGP187	IgG4 FGS1/(FG+FGS1)	9	33120204	2	4.41E-09	9.24E-12	-	-0.224536	-0.2771	+	G	A	0.29687889	0.0380581	+	G	A	0.3144	0.0406
rs7873182	LC_IGP187	IgG4 FGS1/(FG+FGS1)	9	33142170	2	1.70E-09	1.14E-11	-	-0.220491	-0.2618	+	A	G	0.320353874	0.0363926	+	A	G	0.3318	0.0386
rs12554995	LC_IGP187	IgG4 FGS1/(FG+FGS1)	9	33132044	2	1.32E-09	1.23E-11	-	-0.221821	-0.2601	+	G	A	0.319984442	0.0363657	+	G	A	0.3303	0.0384
rs73484568	LC_IGP187	IgG4 FGS1/(FG+FGS1)	9	33132492	2	1.32E-09	1.23E-11	-	-0.221821	-0.2601	+	A	G	0.319984442	0.0363656	+	A	G	0.3303	0.0384
rs913214	LC_IGP187	IgG4 FGS1/(FG+FGS1)	9	33135085	2	1.32E-09	1.24E-11	-	-0.221816	-0.26	+	A	G	0.319984137	0.036365	+	A	G	0.3304	0.0384
rs10758192	LC_IGP187	IgG4 FGS1/(FG+FGS1)	9	33131651	2	1.32E-09	1.26E-11	-	-0.22182	-0.2599	+	A	G	0.319983832	0.0363657	+	A	G	0.3303	0.0384
rs3780488	LC_IGP187	IgG4 FGS1/(FG+FGS1)	9	33133812	2	1.32E-09	1.27E-11	-	-0.221817	-0.2599	+	C	T	0.319983832	0.0363654	+	C	T	0.3303	0.0384
rs7868781	LC_IGP187	IgG4 FGS1/(FG+FGS1)	9	33141320	2	8.27E-10	1.28E-11	-	-0.225486	-0.2636	+	G	A	0.316877669	0.0365097	+	G	A	0.3242	0.0389
rs56687083	LC_IGP187	IgG4 FGS1/(FG+FGS1)	9	33132134	2	1.32E-09	1.28E-11	-	-0.221821	-0.2606	+	G	A	0.319984442	0.0363657	+	G	A	0.3296	0.0385
rs10738906	LC_IGP187	IgG4 FGS1/(FG+FGS1)	9	33135634	2	1.32E-09	1.43E-11	-	-0.221817	-0.2592	+	C	T	0.319985052	0.0363647	+	C	T	0.3305	0.0384
rs7036812	LC_IGP187	IgG4 FGS1/(FG+FGS1)	9	33143822	2	2.58E-09	1.64E-11	-	-0.218318	-0.2596	+	C	T	0.320592739	0.0364475	+	C	T	0.3303	0.0385
rs7873903	LC_IGP187	IgG4 FGS1/(FG+FGS1)	9	33142728	2	2.10E-09	1.81E-11	-	-0.219418	-0.2588	+	A	G	0.320469189	0.0364243	+	A	G	0.3305	0.0385
rs7873077	LC_IGP187	IgG4 FGS1/(FG+FGS1)	9	33142258	2	1.76E-09	1.81E-11	-	-0.220332	-0.2585	+	A	C	0.320373703	0.0363984	+	A	C	0.3306	0.0385
rs7865745	LC_IGP187	IgG4 FGS1/(FG+FGS1)	9	33140976	2	1.40E-09	1.83E-11	-	-0.221515	-0.2581	+	A	G	0.320176937	0.0363739	+	A	G	0.3307	0.0384
rs10124479	LC_IGP187	IgG4 FGS1/(FG+FGS1)	9	33136233	2	1.32E-09	1.83E-11	-	-0.221819	-0.2584	+	G	T	0.319985357	0.0363642	+	G	T	0.3317	0.0384
rs7864705	LC_IGP187	IgG4 FGS1/(FG+FGS1)	9	33140352	2	1.38E-09	1.83E-11	-	-0.22163	-0.258	+	C	A	0.320130262	0.0363723	+	C	A	0.3307	0.0384
rs7849148	LC_IGP187	IgG4 FGS1/(FG+FGS1)	9	33138352	2	1.32E-09	1.84E-11	-	-0.221843	-0.2579	+	T	A	0.31999939	0.0363636	+	T	A	0.3308	0.0384
rs10813955	LC_IGP187	IgG4 FGS1/(FG+FGS1)	9	33141791	2	1.66E-09	1.84E-11	-	-0.220572	-0.2581	+	T	C	0.320622331	0.0363808	+	T	C	0.3307	0.0384
rs3780487	LC_IGP187	IgG4 FGS1/(FG+FGS1)	9	33139237	2	1.31E-09	1.84E-11	-	-0.221857	-0.2579	+	C	T	0.320007627	0.0363636	+	C	T	0.3308	0.0384
rs10971424	LC_IGP187	IgG4 FGS1/(FG+FGS1)	9	33138775	2	1.31E-09	1.84E-11	-	-0.22185	-0.2579	+	C	T	0.320002441	0.0363636	+	C	T	0.3308	0.0384
rs10813954	LC_IGP187	IgG4 FGS1/(FG+FGS1)	9	33137596	2	1.32E-09	1.85E-11	-	-0.221826	-0.2578	+	C	A	0.319987492	0.0363634	+	C	A	0.3308	0.0384
rs2209948	LC_IGP187	IgG4 FGS1/(FG+FGS1)	9	33138247	2	1.32E-09	1.89E-11	-	-0.221837	-0.2578	+	A	G	0.319995424	0.0363635	+	A	G	0.3308	0.0384
rs3824458	LC_IGP187	IgG4 FGS1/(FG+FGS1)	9	33144809	2	2.85E-09	2.25E-11	-	-0.217807	-0.2582	+	T	C	0.320751373	0.0364638	+	T	C	0.3311	0.0386
rs6476402	LC_IGP187	IgG4 FGS1/(FG+FGS1)	9	33148552	2	8.97E-09	2.28E-11	-	-0.209652	-0.2584	+	A	G	0.327878585	0.0362773	+	A	G	0.3338	0.0386
rs4584234	LC_IGP187	IgG4 FGS1/(FG+FGS1)	9	33147479	2	8.15E-09	2.96E-11	-	-0.210193	-0.2567	+	A	G	0.328016473	0.0362682	+	A	G	0.3348	0.0386
chr9:33145647:I	LC_IGP187	IgG4 FGS1/(FG+FGS1)	9	33145647	2	1.35E-08	4.38E-11	-	-0.207019	-0.2544	+	CA	C	0.32897712	0.0362628	+	CA	C	0.3351	0.0386
rs10738907	LC_IGP187	IgG4 FGS1/(FG+FGS1)	9	33135890	2	8.12E-10	6.22E-11	-	-0.22699	-0.2629	+	A	T	0.313705003	0.0367352	+	A	T	0.3167	0.0402
rs1969977	LC_IGP199	IgG4_G2Fn	9	33120203	2	6.42E-09	1.35E-08	-	-0.2397	-0.2566	+	G	A	0.234789554	0.0410696	+	G	A	0.2419	0.0452
rs10971417	LC_IGP199	IgG4_G2Fn	9	33121480	2	1.67E-08	4.13E-09	-	-0.220182	-0.2507	+	T	C	0.259713195	0.038824	+	T	C	0.2577	0.0426
rs12342831	LC_IGP199	IgG4_G2Fn	9	33124872	2	1.43E-08	2.20E-09	-	-0.219551	-0.2513	+	C	T	0.260834453	0.0385273	+	C	T	0.2631	0.042
rs10813951	LC_IGP199	IgG4_G2Fn	9	33128021	2	1.36E-08	3.22E-09	-	-0.219774	-0.2494	+	G	A	0.260838119	0.0385107	+	G	A	0.2647	0.0421

rs3780486	LC_IGP199	IgG4_G2Fn	9	33139453	2	1.35E-08	3.19E-09	-	-0.219806	-0.2502	+	T	C	0.260843005	0.0385077	+	T	C	0.2662	0.0423
chr9:33152337:D	LC_IGP199	IgG4_G2Fn	9	33152337	2	1.44E-08	2.51E-09	-	-0.220887	-0.2532	+	C	CAT	0.25931796	0.0387661	+	C	CAT	0.2654	0.0425
rs10813957	LC_IGP199	IgG4_G2Fn	9	33153527	2	1.94E-08	4.46E-09	-	-0.218201	-0.2487	+	T	G	0.26129383	0.0386526	+	T	G	0.2668	0.0424
rs10971434	LC_IGP199	IgG4_G2Fn	9	33168891	2	1.96E-08	7.97E-10	-	-0.219012	-0.2631	+	A	C	0.262562309	0.0388059	+	A	C	0.2682	0.0428
rs10758195	LC_IGP199	IgG4_G2Fn	9	33174587	2	4.31E-08	1.33E-09	-	-0.216355	-0.2631	+	T	C	0.265392019	0.039312	+	T	C	0.2691	0.0434
rs1969977	LC_IGP205	IgG4_G2n	9	33120203	2	4.28E-09	1.63E-08	-	-0.242839	-0.2545	+	G	A	0.234789554	0.0411249	+	G	A	0.2419	0.0451
rs10971417	LC_IGP205	IgG4_G2n	9	33121480	2	1.12E-08	4.58E-09	-	-0.223208	-0.2495	+	T	C	0.259713195	0.0388764	+	T	C	0.2577	0.0426
rs12342831	LC_IGP205	IgG4_G2n	9	33124872	2	9.06E-09	2.30E-09	-	-0.222883	-0.2505	+	C	T	0.260834453	0.038578	+	C	T	0.2631	0.0419
rs10813951	LC_IGP205	IgG4_G2n	9	33128021	2	8.66E-09	4.32E-09	-	-0.223086	-0.2469	+	G	A	0.260838119	0.0385615	+	G	A	0.2647	0.042
rs3780486	LC_IGP205	IgG4_G2n	9	33139453	2	8.60E-09	4.38E-09	-	-0.223117	-0.2476	+	T	C	0.260843005	0.0385584	+	T	C	0.2662	0.0422
chr9:33152337:D	LC_IGP205	IgG4_G2n	9	33152337	2	1.04E-08	3.58E-09	-	-0.223361	-0.2504	+	C	CAT	0.25931796	0.0388203	+	C	CAT	0.2654	0.0424
rs10813957	LC_IGP205	IgG4_G2n	9	33153527	2	1.32E-08	6.44E-09	-	-0.221079	-0.2456	+	T	G	0.26129383	0.0387053	+	T	G	0.2668	0.0423
rs3780480	LC_IGP205	IgG4_G2n	9	33163486	2	4.08E-08	3.51E-09	-	-0.213894	-0.2509	+	T	G	0.262813684	0.0387949	+	T	G	0.2654	0.0425
rs10971434	LC_IGP205	IgG4_G2n	9	33168891	2	1.60E-08	1.49E-09	-	-0.220697	-0.2585	+	A	C	0.262562309	0.0388629	+	A	C	0.2682	0.0428
rs10758195	LC_IGP205	IgG4_G2n	9	33174587	2	2.72E-08	2.84E-09	-	-0.219868	-0.2576	+	T	C	0.265392019	0.0393637	+	T	C	0.2691	0.0434
rs28429368	LC_IGP23	IgG1 Galactosylation	9	33125359	2	1.72E-10	5.08E-07	+	0.852723	0.6984	+	T	C	0.017520935	0.132697	+	T	C	0.0158	0.139
rs3780494	LC_IGP23	IgG1 Galactosylation	9	33126224	2	1.80E-10	5.53E-07	+	0.849587	0.6989	+	C	T	0.01754386	0.132355	+	C	T	0.0157	0.1396
rs73484569	LC_IGP23	IgG1 Galactosylation	9	33133191	2	1.80E-10	4.32E-08	+	0.849669	0.7319	+	A	G	0.017543557	0.132359	+	A	G	0.0173	0.1336
rs3824459	LC_IGP23	IgG1 Galactosylation	9	33134617	2	1.80E-10	2.98E-08	+	0.849669	0.6793	+	T	C	0.017543557	0.132359	+	T	C	0.0204	0.1226
rs73472094	LC_IGP23	IgG1 Galactosylation	9	33135537	2	1.80E-10	4.33E-08	+	0.849707	0.732	+	A	G	0.01754386	0.132359	+	A	G	0.0173	0.1336
rs12346594	LC_IGP23	IgG1 Galactosylation	9	33136536	2	1.80E-10	1.66E-07	+	0.849707	0.7007	+	A	G	0.01754386	0.132359	+	A	G	0.0184	0.1339
rs3780485	LC_IGP23	IgG1 Galactosylation	9	33139618	2	1.73E-10	4.93E-08	+	0.850492	0.7332	+	C	T	0.017556996	0.132358	+	C	T	0.0173	0.1344
rs10121006	LC_IGP23	IgG1 Galactosylation	9	33141250	2	1.71E-10	4.94E-08	+	0.850741	0.7335	+	T	C	0.017560431	0.132365	+	T	C	0.0173	0.1345
rs7020500	LC_IGP23	IgG1 Galactosylation	9	33143733	2	1.63E-10	4.99E-08	+	0.856612	0.7382	+	C	G	0.017494361	0.133114	+	C	G	0.0172	0.1354
rs113197944	LC_IGP23	IgG1 Galactosylation	9	33149340	2	1.45E-10	5.03E-08	+	0.866508	0.7394	+	A	G	0.017396001	0.134269	+	A	G	0.0172	0.1357
rs1328899	LC_IGP23	IgG1 Galactosylation	9	33151415	2	2.94E-10	4.00E-08	+	0.821769	0.7441	+	T	C	0.019405966	0.129567	+	T	C	0.0176	0.1355
rs12343079	LC_IGP23	IgG1 Galactosylation	9	33158557	2	1.45E-10	8.42E-08	+	0.866715	0.7496	+	A	G	0.017392814	0.134308	+	A	G	0.0162	0.1399
rs3780481	LC_IGP23	IgG1 Galactosylation	9	33163298	2	1.44E-10	8.74E-08	+	0.86691	0.7484	+	T	C	0.017390937	0.134328	+	T	C	0.0162	0.1399
rs3780478	LC_IGP23	IgG1 Galactosylation	9	33164116	2	1.45E-10	8.44E-08	+	0.867079	0.7498	+	A	T	0.017389112	0.134361	+	A	T	0.0162	0.14
rs73472938	LC_IGP23	IgG1 Galactosylation	9	33164527	2	1.45E-10	8.48E-08	+	0.867127	0.7498	+	A	T	0.017389165	0.134372	+	A	T	0.0162	0.14
rs3758273	LC_IGP23	IgG1 Galactosylation	9	33168309	2	3.74E-10	8.96E-08	+	0.880388	0.7509	+	A	C	0.016444903	0.139652	+	A	C	0.0162	0.1404
rs12337458	LC_IGP3	IgG1_G2F	9	33180566	2	9.18E-11	1.10E-06	+	1.03476	0.792	+	C	T	0.013489372	0.158642	+	C	T	0.0132	0.1625
rs3780478	LC_IGP3	IgG1_G2F	9	33164116	2	1.67E-10	7.39E-08	+	0.862026	0.7691	+	A	T	0.016933783	0.134061	+	A	T	0.0162	0.1429
rs3780481	LC_IGP3	IgG1_G2F	9	33163298	2	1.67E-10	7.79E-08	+	0.861802	0.7673	+	T	C	0.016935665	0.134028	+	T	C	0.0162	0.1428
rs73472938	LC_IGP3	IgG1_G2F	9	33164527	2	1.67E-10	7.41E-08	+	0.862069	0.7692	+	A	T	0.016933835	0.134071	+	A	T	0.0162	0.1429
rs113197944	LC_IGP3	IgG1_G2F	9	33149340	2	1.67E-10	6.44E-08	+	0.861384	0.7452	+	A	G	0.016940197	0.13397	+	A	G	0.0171	0.1378
rs12343079	LC_IGP3	IgG1_G2F	9	33158557	2	1.68E-10	7.36E-08	+	0.861604	0.7689	+	A	G	0.016937389	0.134008	+	A	G	0.0162	0.1429
rs7020500	LC_IGP3	IgG1_G2F	9	33143733	2	1.78E-10	6.38E-08	+	0.852682	0.7438	+	C	G	0.017035682	0.132812	+	C	G	0.0172	0.1375
rs28429368	LC_IGP3	IgG1_G2F	9	33125359	2	1.93E-10	5.01E-07	+	0.84837	0.7105	+	T	C	0.017061266	0.132398	+	T	C	0.0158	0.1414
rs10121006	LC_IGP3	IgG1_G2F	9	33141250	2	1.93E-10	6.33E-08	+	0.84624	0.7379	+	T	C	0.017099725	0.132067	+	T	C	0.0173	0.1364
rs3780485	LC_IGP3	IgG1_G2F	9	33139618	2	1.95E-10	6.32E-08	+	0.845932	0.7375	+	C	T	0.017096379	0.132061	+	C	T	0.0173	0.1363
rs73472094	LC_IGP3	IgG1_G2F	9	33135537	2	2.05E-10	4.67E-08	+	0.844963	0.7404	+	A	G	0.017083588	0.132062	+	A	G	0.0173	0.1355
rs12346594	LC_IGP3	IgG1_G2F	9	33136536	2	2.05E-10	1.48E-07	+	0.844963	0.7158	+	A	G	0.017083588	0.132062	+	A	G	0.0184	0.1362
rs73484569	LC_IGP3	IgG1_G2F	9	33133191	2	2.05E-10	4.63E-08	+	0.844919	0.7404	+	A	G	0.017083293	0.132062	+	A	G	0.0173	0.1355
rs3824459	LC_IGP3	IgG1_G2F	9	33134617	2	2.05E-10	3.08E-08	+	0.844919	0.6865	+	T	C	0.017083293	0.132062	+	T	C	0.0204	0.124
rs3780494	LC_IGP3	IgG1_G2F	9	33126224	2	2.05E-10	5.50E-07	+	0.844854	0.7105	+	C	T	0.017083588	0.132058	+	C	T	0.0157	0.1419
rs3758273	LC_IGP3	IgG1_G2F	9	33168309	2	2.97E-10	7.72E-08	+	0.883197	0.7709	+	A	C	0.016014052	0.139308	+	A	C	0.0161	0.1435
rs1328899	LC_IGP3	IgG1_G2F	9	33151415	2	4.29E-10	4.39E-08	+	0.81211	0.7522	+	T	C	0.018912389	0.129286	+	T	C	0.0176	0.1374
rs3780481	LC_IGP4	IgG1_G0FN	9	33163298	2	2.44E-11	1.06E-08	-	-0.901079	-0.7935	+	T	C	0.016925339	0.134019	+	T	C	0.0162	0.1387
rs73472938	LC_IGP4	IgG1_G0FN	9	33164527	2	2.45E-11	1.07E-08	-	-0.901331	-0.7939	+	A	T	0.016923509	0.134063	+	A	T	0.0162	0.1388
rs12343079	LC_IGP4	IgG1_G0FN	9	33158557	2	2.45E-11	1.07E-08	-	-0.900881	-0.7938	+	A	G	0.016927061	0.134	+	A	G	0.0162	0.1388
rs113197944	LC_IGP4	IgG1_G0FN	9	33149340	2	2.45E-11	8.12E-09	-	-0.900612	-0.7809	+	A	G	0.016929868	0.133961	+	A	G	0.0171	0.1354
rs3758273	LC_IGP4	IgG1_G0FN	9	33168309	2	2.46E-11	1.11E-08	-	-0.936147	-0.7949	+	A	C	0.016003993	0.139252	+	A	C	0.0161	0.1392
rs3780478	LC_IGP4	IgG1_G0FN	9	33164116	2	2.46E-11	1.07E-08	-	-0.90119	-0.7939	+	A	T	0.016923458	0.134052	+	A	T	0.0162	0.1388
rs7020500	LC_IGP4	IgG1_G0FN	9	33143733	2	3.16E-11	8.21E-09	-	-0.887845	-0.7797	+	C	G	0.017025294	0.132819	+	C	G	0.0172	0.1353
rs28429368	LC_IGP4	IgG1_G0FN	9	33125359	2	3.32E-11	1.77E-08	-	-0.884086	-0.7974	+	T	C	0.017050863	0.132403	+	T	C	0.0158	0.1416
rs3780485	LC_IGP4	IgG1_G0FN	9	33139618	2	3.41E-11	8.84E-09	-	-0.881337	-0.7746	+	C	T	0.017085955	0.132066	+	C	T	0.0173	0.1347
rs73472094	LC_IGP4	IgG1_G0FN	9	33135537	2	3.41E-11	7.78E-09	-	-0.881306	-0.774	+	A	G	0.017073171	0.132064	+	A	G	0.0173	0.1341
rs12346594	LC_IGP4	IgG1_G0FN	9	33136536	2	3.41E-11	3.29E-08	-	-0.881306	-0.7418	+	A	G	0.017073171	0.132064	+	A	G	0.0184	0.1343
rs10121006	LC_IGP4	IgG1_G0FN	9	33141250	2	3.42E-11	8.83E-09	-	-0.881337	-0.7748	+	T	C	0.017089298	0.132073	+	T	C	0.0173	0.1347
rs73484569	LC_IGP4	IgG1_G0FN	9	33133191	2	3.42E-11	7.65E-09	-	-0.881271	-0.7743	+	A	G	0.017072876	0.132064	+	A	G	0.0173	0.134
rs3824459	LC_IGP4	IgG1_G0FN	9	33134617	2	3.42E-11	1.14E-07	-	-0.881271	-0.655	+	T	C	0.017072876	0.132064	+	T	C	0.0204	0.1235
rs3780494	LC_IGP4	IgG1_G0FN	9	33126224	2	3.42E-11	1.90E-08	-	-0.88121	-0.7964	+	C	T	0.017073171	0.13206	+	C	T	0.0157	0.1417
rs1328899	LC_IGP4	IgG1_G0FN	9	33151415	2	5.10E-11	5.32E-09	-	-0.854727	-0.7875	+	T	C	0.018900857	0.129261	+	T	C	0.0176	0.1349
rs28429368	LC_IGP48	IgG1_G2Fn	9	33125359	2	4.27E-10	7.46E-07	+	0.831683	0.6934	+	T	C	0.017325535	0.132372	+	T	C	0.0158	0.1401
rs3780494	LC_IGP48	IgG1_G2Fn	9	33126224	2	4.54E-10	8.22E-07	+	0.828253	0.6934	+	C	T	0.017348203	0.132031	+	C	T	0.0157	0.1406
rs73484569	LC_IGP48	IgG1_G2Fn	9	33133191	2	4.53E-10	6.55E-08	+	0.828323	0.7259	+	A	G	0.017347904	0.132036	+	A	G	0.0173	0.1343
rs3824459	LC_IGP48	IgG1_G2Fn	9	33134617	2	4.53E-10	3.46E-08	+	0.828323	0.6786	+	T	C	0.017347904	0.132036	+	T	C	0.0204	0.123
rs73472094	LC_IGP48	IgG1_G2Fn	9	33135537	2	4.52E-10	6.61E-08	+	0.828372	0.7259	+	A	G	0.017348203	0.132036	+	A	G	0.0173	0.1344
rs12346594	LC_IGP48	IgG1_G2Fn	9	33136536	2	4.52E-10	2.03E-07	+	0.828372	0.7023	+	A	G	0.017348203	0.132036	+	A	G	0.0184	0.1351
rs3780485	LC_IGP48	IgG1_G2Fn	9	33139618	2	4.32E-10	8.60E-08	+	0.829294	0.7243	+	C	T	0.017361193	0.132034	+	C	T	0.0173	0.1353
rs10121006	LC_IGP48	IgG1_G2Fn	9	33141250	2	4.27E-10	8.62E-08	+	0.829587	0.7247	+	T	C	0.01736459	0.132041	+	T	C	0.0173	0.1354
rs7020500	LC_IGP48	IgG1_G2Fn	9	33143733	2	3.95E-10	8.80E-08	+	0.835914	0.73	+	C	G	0.017299556	0.132786	+	C	G	0.0172	0.1364
rs113197944	LC_IGP48	IgG1_G2Fn	9	33149340	2	3.62E-10	8.91E-08	+	0.845049	0.7313	+	A	G	0.017202592	0.133941	+	A	G	0.0171	0.1368
rs1328899	LC_IGP48	IgG1_G2Fn	9	33151415	2	7.95E-10	5.74E-08	+	0.799162	0.7391	+	T	C	0.019190556	0.129251	+	T	C	0.0176	0.1362
rs12343079	LC_IGP48	IgG1_G2Fn	9	33158557	2	3.62E-10	1.10E-07	+	0.845255	0.7525	+	A	G	0.017199739	0.13398	+	A	G	0.0162	0.1417
rs3780481	LC_IGP48	IgG1_G2Fn	9	33163298	2	3.61E-10	1.16E-07	+	0.845455	0.751	+	T	C	0.01719799	0.134	+	T	C	0.0162	0.1417
rs3780478	LC_IGP48	IgG1_G2Fn	9	33164116	2	3.61E-10	1.10E-07	+	0.845669	0.7527	+	A	T	0.017196078	0.134032	+	A	T	0.0162	0.1418
rs73472938	LC_IGP48	IgG1_G2Fn	9	33164527	2	3.61E-10	1.11E-07	+	0.845714	0.7527	+	A	T	0.017196131	0.134043	+	A	T	0.0162	0.1418
rs3758273	LC_IGP48	IgG1_G2Fn	9	33168309	2	6.04E-10	1.16E-07	+	0.867296	0.7542	+	A	C	0.016261802	0.139273	+	A	C	0.0161	0.1423
rs12337458	LC_IGP48	IgG1_G2Fn	9	33180566	2	2.16E-10	2.04E-06	+	1.01354	0.7685	+	C	T	0.013681319	0.158611	+	C	T	0.0132	0.1618
rs28429368	LC_IGP49	IgG1_G0FNn	9	33125359	2	2.35E-11	2.17E-08	-	-0.890188	-0.7964	+	T	C	0.017325535	0.132277	+	T	C	0.0158	0.1423
rs3780494	LC_IGP49	IgG1_G0FNn	9	33126224	2	2.43E-11	2.34E-08	-	-0.887256	-0.7956	+	C	T	0.017348203	0.131935	+	C	T	0.0157	0.1424
rs73484569	LC_IGP49	IgG1_G0FNn	9	33133191	2	2.43E-11	1.04E-08	-	-0.887315	-0.7706	+	A	G	0.017347904	0.131939	+	A	G	0.0173	0.1346
rs3824459	LC_IGP49	IgG1_G0FNn	9	33134617	2	2.43E-11	2.11E-07	-	-0.887315	-0.6428	+	T	C	0.017347904	0.131939	+	T	C	0.0204	0.1239
rs73472094	LC_IGP49	IgG1_G0FNn	9	33135537	2	2.42E-11	1.06E-08	-	-0.887348	-0.7703	+	A	G	0.017348203	0.131939	+	A	G	0.0173	0.1346
rs12346594	LC_IGP49	IgG1_G0FNn	9	33136536	2	2.42E-11	4.64E-08	-	-0.887348	-0.7369	+	A	G	0.017348203	0.131939	+	A	G	0.0184	0.1349
rs3780485	LC_IGP49	IgG1_G0FNn	9	33139618	2	2.42E-11	1.24E-08	-	-0.88736	-0.7703	+	C	T	0.017361193	0.131941	+	C	T	0.0173	0.1353
rs10121006	LC_IGP49	IgG1_G0FNn	9	33141250	2	2.43E-11	1.24E-08	-	-0.887349	-0.7705	+	T	C	0.01736459	0.131949	+	T	C	0.0173	0.1353
rs7020500	LC_IGP49	IgG1_G0FNn	9	33143733	2	2.25E-11	1.17E-08	-	-0.893863	-0.7754	+	C	G	0.017299556	0.132694	+	C	G	0.0172	0.1359
rs113197944	LC_IGP49	IgG1_G0FNn	9	33149340	2	1.75E-11	1.16E-08	-	-0.906613	-0.7766	+	A	G	0.017202592	0.133834	+	A	G	0.0171	0.1361
rs1328899	LC_IGP49	IgG1_G0FNn	9	33151415	2	3.99E-11	7.86E-09	-	-0.858907	-0.7825	+	T	C	0.019190556	0.129152	+	T	C	0.0176	0.1356
rs12343079	LC_IGP49	IgG1_G0FNn	9	33158557	2	1.75E-11	1.49E-08	-	-0.906893	-0.7888	+	A	G	0.017199739	0.133873	+	A	G	0.0162	0.1393
rs3780481	LC_IGP49	IgG1_G0FNn	9	33163298	2	1.74E-11	1.48E-08	-	-0.907088	-0.7886	+	T	C	0.01719799	0.133893	+	T	C	0.0162	0.1393
rs3780478	LC_IGP49	IgG1_G0FNn	9	33164116	2	1.75E-11	1.49E-08	-	-0.907199	-0.789	+	A	T	0.017196078	0.133926	+	A	T	0.0162	0.1393
rs73472938	LC_IGP49	IgG1_G0FNn	9	33164527	2	1.75E-11	1.50E-08	-	-0.90734	-0.789	+	A	T	0.017196131	0.133936	+	A	T	0.0162	0.1394
rs3758273	LC_IGP49	IgG1_G0FNn	9	33168309	2	1.45E-11	1.56E-08	-	-0.946184	-0.79	+	A	C	0.016261802	0.139103	+	A	C	0.0161	0.1397
rs28429368	LC_IGP58	IgG1 G0n	9	33125359	2	1.64E-11	1.35E-08	-	-0.899117	-0.7937	+	T	C	0.017325535	0.132545	+	T	C	0.0158	0.1398
rs3780494	LC_IGP58	IgG1 G0n	9	33126224	2	1.71E-11	1.49E-08	-	-0.896013	-0.7942	+	C	T	0.017348203	0.132202	+	C	T	0.0157	0.1403
rs73484569	LC_IGP58	IgG1 G0n	9	33133191	2	1.70E-11	2.04E-09	-	-0.896093	-0.8034	+	A	G	0.017347904	0.132207	+	A	G	0.0173	0.134
rs3824459	LC_IGP58	IgG1 G0n	9	33134617	2	1.70E-11	2.28E-09	-	-0.896093	-0.7347	+	T	C	0.017347904	0.132207	+	T	C	0.0204	0.1229
rs73472094	LC_IGP58	IgG1 G0n	9	33135537	2	1.70E-11	2.04E-09	-	-0.896137	-0.8035	+	A	G	0.017348203	0.132206	+	A	G	0.0173	0.134
rs12346594	LC_IGP58	IgG1 G0n	9	33136536	2	1.70E-11	1.02E-08	-	-0.896137	-0.7613	+	A	G	0.017348203	0.132206	+	A	G	0.0184	0.1329
rs3780485	LC_IGP58	IgG1 G0n	9	33139618	2	1.64E-11	2.35E-09	-	-0.896822	-0.8046	+	C	T	0.017361193	0.132206	+	C	T	0.0173	0.1347
rs10121006	LC_IGP58	IgG1 G0n	9	33141250	2	1.63E-11	2.35E-09	-	-0.897031	-0.8049	+	T	C	0.01736459	0.132212	+	T	C	0.0173	0.1348
rs7020500	LC_IGP58	IgG1 G0n	9	33143733	2	1.54E-11	2.27E-09	-	-0.903228	-0.8102	+	C	G	0.017299556	0.13296	+	C	G	0.0172	0.1356
rs113197944	LC_IGP58	IgG1 G0n	9	33149340	2	1.41E-11	2.27E-09	-	-0.912848	-0.8115	+	A	G	0.017202592	0.134117	+	A	G	0.0171	0.1358
rs1328899	LC_IGP58	IgG1 G0n	9	33151415	2	4.12E-11	1.45E-09	-	-0.860211	-0.8198	+	T	C	0.019190556	0.129444	+	T	C	0.0176	0.1355
rs12343079	LC_IGP58	IgG1 G0n	9	33158557	2	1.41E-11	3.78E-09	-	-0.913072	-0.8215	+	A	G	0.017199739	0.134156	+	A	G	0.0162	0.1394
rs3780481	LC_IGP58	IgG1 G0n	9	33163298	2	1.40E-11	3.92E-09	-	-0.91329	-0.8205	+	T	C	0.01719799	0.134175	+	T	C	0.0162	0.1394
rs3780478	LC_IGP58	IgG1 G0n	9	33164116	2	1.41E-11	3.80E-09	-	-0.913465	-0.8217	+	A	T	0.017196078	0.134208	+	A	T	0.0162	0.1394
rs73472938	LC_IGP58	IgG1 G0n	9	33164527	2	1.41E-11	3.81E-09	-	-0.913507	-0.8217	+	A	T	0.017196131	0.134219	+	A	T	0.0162	0.1395
rs3758273	LC_IGP58	IgG1 G0n	9	33168309	2	4.20E-11	4.02E-09	-	-0.926677	-0.823	+	A	C	0.016261802	0.139505	+	A	C	0.0161	0.1399
rs12337458	LC_IGP58	IgG1 G0n	9	33180566	2	4.77E-12	3.87E-07	-	-1.10579	-0.8096	+	C	T	0.013681319	0.158764	+	C	T	0.0132	0.1595
rs28429368	LC_IGP59	IgG1 G1n	9	33125359	2	5.65E-09	2.38E-07	+	0.779082	0.7438	+	T	C	0.017325535	0.132979	+	T	C	0.0158	0.144
rs3780494	LC_IGP59	IgG1 G1n	9	33126224	2	5.73E-09	2.38E-07	+	0.776741	0.7454	+	C	T	0.017348203	0.132634	+	C	T	0.0157	0.1443
rs73484569	LC_IGP59	IgG1 G1n	9	33133191	2	5.72E-09	1.85E-07	+	0.776774	0.7095	+	A	G	0.017347904	0.132638	+	A	G	0.0173	0.1361
rs3824459	LC_IGP59	IgG1 G1n	9	33134617	2	5.72E-09	6.12E-07	+	0.776774	0.6136	+	T	C	0.017347904	0.132638	+	T	C	0.0204	0.123
rs73472094	LC_IGP59	IgG1 G1n	9	33135537	2	5.72E-09	1.84E-07	+	0.776779	0.7097	+	A	G	0.017348203	0.132638	+	A	G	0.0173	0.1361

rs12346594	LC_IGP59	IgG1 G1n	9	33136536	2	5.72E-09	7.09E-07	+	0.776779	0.6587	+	A	G	0.017348203	0.132638	+	A	G	0.0184	0.1328
rs3780485	LC_IGP59	IgG1 G1n	9	33139618	2	5.65E-09	1.87E-07	+	0.777082	0.7135	+	C	T	0.017361193	0.13264	+	C	T	0.0173	0.1369
rs10121006	LC_IGP59	IgG1 G1n	9	33141250	2	5.64E-09	1.88E-07	+	0.777145	0.7139	+	T	C	0.01736459	0.132647	+	T	C	0.0173	0.137
rs7020500	LC_IGP59	IgG1 G1n	9	33143733	2	5.68E-09	1.92E-07	+	0.781426	0.7196	+	C	G	0.017299556	0.133402	+	C	G	0.0172	0.1382
rs113197944	LC_IGP59	IgG1 G1n	9	33149340	2	5.71E-09	1.95E-07	+	0.788126	0.7211	+	A	G	0.017202592	0.13457	+	A	G	0.0171	0.1386
rs1328899	LC_IGP59	IgG1 G1n	9	33151415	2	2.47E-08	1.16E-07	+	0.7279	0.7322	+	T	C	0.019190556	0.129911	+	T	C	0.0176	0.1382
rs12343079	LC_IGP59	IgG1 G1n	9	33158557	2	5.73E-09	5.40E-07	+	0.788293	0.7198	+	A	G	0.017199739	0.13461	+	A	G	0.0162	0.1436
rs3780481	LC_IGP59	IgG1 G1n	9	33163298	2	5.72E-09	5.36E-07	+	0.78846	0.7196	+	T	C	0.01719799	0.134629	+	T	C	0.0162	0.1436
rs3780478	LC_IGP59	IgG1 G1n	9	33164116	2	5.73E-09	5.43E-07	+	0.788593	0.7199	+	A	T	0.017196078	0.134662	+	A	T	0.0162	0.1437
rs73472938	LC_IGP59	IgG1 G1n	9	33164527	2	5.74E-09	5.44E-07	+	0.78861	0.72	+	A	T	0.017196131	0.134673	+	A	T	0.0162	0.1437
rs3758273	LC_IGP59	IgG1 G1n	9	33168309	2	1.76E-08	5.67E-07	+	0.792697	0.7217	+	A	C	0.016261802	0.139979	+	A	C	0.0161	0.1443
rs73484569	LC_IGP60	IgG1 G2n	9	33133191	2	1.20E-09	6.24E-07	+	0.809435	0.6679	+	A	G	0.017347904	0.132328	+	A	G	0.0173	0.134
rs3824459	LC_IGP60	IgG1 G2n	9	33134617	2	1.20E-09	3.04E-07	+	0.809435	0.6286	+	T	C	0.017347904	0.132328	+	T	C	0.0204	0.1228
rs73472094	LC_IGP60	IgG1 G2n	9	33135537	2	1.19E-09	6.26E-07	+	0.809489	0.668	+	A	G	0.017348203	0.132327	+	A	G	0.0173	0.134
rs12346594	LC_IGP60	IgG1 G2n	9	33136536	2	1.19E-09	1.47E-06	+	0.809489	0.6486	+	A	G	0.017348203	0.132327	+	A	G	0.0184	0.1347
rs3780485	LC_IGP60	IgG1 G2n	9	33139618	2	1.15E-09	7.55E-07	+	0.810329	0.6677	+	C	T	0.017361193	0.132327	+	C	T	0.0173	0.135
rs10121006	LC_IGP60	IgG1 G2n	9	33141250	2	1.13E-09	7.57E-07	+	0.8106	0.668	+	T	C	0.01736459	0.132333	+	T	C	0.0173	0.135
rs7020500	LC_IGP60	IgG1 G2n	9	33143733	2	1.05E-09	7.88E-07	+	0.816793	0.6723	+	C	G	0.017299556	0.13308	+	C	G	0.0172	0.1361
rs113197944	LC_IGP60	IgG1 G2n	9	33149340	2	9.54E-10	8.01E-07	+	0.826039	0.6733	+	A	G	0.017202592	0.134237	+	A	G	0.0171	0.1364
rs1328899	LC_IGP60	IgG1 G2n	9	33151415	2	1.26E-09	6.29E-07	+	0.791047	0.6781	+	T	C	0.019190556	0.129497	+	T	C	0.0176	0.1361
rs12343079	LC_IGP60	IgG1 G2n	9	33158557	2	9.55E-10	1.16E-06	+	0.82625	0.6873	+	A	G	0.017199739	0.134276	+	A	G	0.0162	0.1413
rs3780481	LC_IGP60	IgG1 G2n	9	33163298	2	9.51E-10	1.21E-06	+	0.826465	0.6859	+	T	C	0.01719799	0.134295	+	T	C	0.0162	0.1413
rs3780478	LC_IGP60	IgG1 G2n	9	33164116	2	9.52E-10	1.16E-06	+	0.826632	0.6875	+	A	T	0.017196078	0.134328	+	A	T	0.0162	0.1414
rs73472938	LC_IGP60	IgG1 G2n	9	33164527	2	9.54E-10	1.16E-06	+	0.826672	0.6875	+	A	T	0.017196131	0.134339	+	A	T	0.0162	0.1414
rs3758273	LC_IGP60	IgG1 G2n	9	33168309	2	1.63E-09	1.23E-06	+	0.846695	0.6886	+	A	C	0.016261802	0.139583	+	A	C	0.0161	0.142
rs113197944	LC_IGP88	IgG2_G1F	9	33149340	2	4.40E-08	1.25E-10	+	0.741794	0.8764	+	A	G	0.016929868	0.134868	+	A	G	0.0171	0.1362
rs7020500	LC_IGP88	IgG2_G1F	9	33143733	2	4.97E-08	1.28E-10	+	0.73246	0.8745	+	C	G	0.017025294	0.133707	+	C	G	0.0172	0.136
rs10121006	LC_IGP88	IgG2_G1F	9	33141250	2	4.99E-08	1.46E-10	+	0.728235	0.8672	+	T	C	0.017089298	0.132951	+	T	C	0.0173	0.1353
rs12343079	LC_IGP88	IgG2_G1F	9	33158557	2	4.40E-08	4.20E-10	+	0.741962	0.8674	+	A	G	0.016927061	0.134907	+	A	G	0.0162	0.1389
rs3780478	LC_IGP88	IgG2_G1F	9	33164116	2	4.40E-08	4.22E-10	+	0.742281	0.8676	+	A	T	0.016923458	0.13496	+	A	T	0.0162	0.1389
rs73472938	LC_IGP88	IgG2_G1F	9	33164527	2	4.40E-08	4.23E-10	+	0.74234	0.8676	+	A	T	0.016923509	0.134971	+	A	T	0.0162	0.1389
rs3780481	LC_IGP88	IgG2_G1F	9	33163298	2	4.40E-08	4.35E-10	+	0.742112	0.8667	+	T	C	0.016925339	0.134927	+	T	C	0.0162	0.1389
rs12337458	LC_IGP89	IgG2_G2F	9	33180566	2	2.38E-08	5.95E-10	+	0.892519	0.9829	+	C	T	0.013481147	0.15912	+	C	T	0.0132	0.1587
rs72712459	LC_IGP78	IgG1 BG0n/G0n	14	65734600	1	6.15E-10	8.73E-07	+	0.231428	0.208	+	A	G	0.33679399	0.03718	+	A	G	0.3201	0.0423
rs72712459	LC_IGP79	IgG1 BG1n/G1n	14	65734600	1	4.98E-11	1.31E-06	+	0.246215	0.205	+	A	G	0.33679399	0.0372097	+	A	G	0.3201	0.0424
rs72712459	LC_IGP77	IgG1 Bn	14	65734600	1	2.36E-10	8.87E-07	+	0.237532	0.209	+	A	G	0.33679399	0.0372523	+	A	G	0.3201	0.0425
rs72712459	LC_IGP86	IgG1 Bn/Fn total	14	65734600	1	8.79E-11	3.39E-07	+	0.243057	0.2169	+	A	G	0.33679399	0.0372219	+	A	G	0.3201	0.0425
rs72712459	LC_IGP84	IgG1 FBn/Bn total	14	65734600	1	2.97E-11	1.64E-09	-	-0.249534	-0.2567	+	A	G	0.33679399	0.0372729	+	A	G	0.3201	0.0426
rs72712459	LC_IGP62	IgG1 FG0n total/G0n	14	65734600	1	5.10E-12	1.94E-08	-	-0.257537	-0.2382	+	A	G	0.33679399	0.0370278	+	A	G	0.3201	0.0424
rs72712459	LC_IGP63	IgG1 FG1n total/G1n	14	65734600	1	5.39E-12	1.85E-08	-	-0.258004	-0.2388	+	A	G	0.33679399	0.0371382	+	A	G	0.3201	0.0424
rs72712459	LC_IGP64	IgG1 FG2n total/G2n	14	65734600	1	5.30E-09	5.32E-09	-	-0.219113	-0.2463	+	A	G	0.33679399	0.0373325	+	A	G	0.3201	0.0422
rs72712459	LC_IGP61	IgG1 Fn total	14	65734600	1	2.13E-11	1.44E-08	-	-0.250511	-0.2406	+	A	G	0.33679399	0.0371432	+	A	G	0.3201	0.0424
rs72712459	LC_IGP21	IgG1 Fucosylation	14	65734600	1	2.83E-10	3.64E-08	-	-0.235046	-0.2341	+	A	G	0.337581381	0.0370276	+	A	G	0.3201	0.0425
rs72712459	LC_IGP11	IgG1_G0	14	65734600	1	3.97E-13	1.12E-09	+	0.268559	0.2572	+	A	G	0.336981368	0.0367058	+	A	G	0.3201	0.0422
rs72712459	LC_IGP_R1	IgG1_G0F/IgG1_G0	14	65734600	1	1.26E-11	5.02E-08	-	-0.250811	-0.2306	+	A	G	0.336981368	0.0367656	+	A	G	0.3201	0.0423
rs72712459	LC_IGP_R4	IgG1_G0FN/IgG1_G0N	14	65734600	1	2.19E-10	6.27E-09	-	-0.23611	-0.2468	+	A	G	0.337335579	0.0369646	+	A	G	0.3201	0.0425
rs72712459	LC_IGP52	IgG1_G0n	14	65734600	1	2.95E-13	1.31E-09	+	0.271792	0.2566	+	A	G	0.33679399	0.0369369	+	A	G	0.3201	0.0423
rs72712459	LC_IGP14	IgG1_G0N	14	65734600	1	6.06E-12	1.09E-07	+	0.255527	0.2244	+	A	G	0.337335579	0.0368758	+	A	G	0.3201	0.0423
rs72712459	LC_IGP55	IgG1_G0Nn	14	65734600	1	3.83E-12	1.38E-07	+	0.259566	0.2229	+	A	G	0.33679399	0.0370979	+	A	G	0.3201	0.0423
rs72712459	LC_IGP12	IgG1_G1	14	65734600	1	4.75E-10	1.40E-08	+	0.231308	0.2401	+	A	G	0.336677153	0.0369185	+	A	G	0.3201	0.0423
rs72712459	LC_IGP_R2	IgG1_G1F/IgG1_G1	14	65734600	1	3.20E-11	5.85E-08	-	-0.246433	-0.2299	+	A	G	0.336677153	0.0368741	+	A	G	0.3201	0.0424
rs72712459	LC_IGP_R5	IgG1_G1FN/IgG1_G1N	14	65734600	1	1.38E-11	1.43E-09	-	-0.252588	-0.2567	+	A	G	0.336161439	0.0370964	+	A	G	0.3201	0.0424
rs72712459	LC_IGP53	IgG1_G1n	14	65734600	1	1.36E-09	2.69E-08	+	0.226975	0.2352	+	A	G	0.33679399	0.0372339	+	A	G	0.3201	0.0423
rs72712459	LC_IGP15	IgG1_G1N	14	65734600	1	3.56E-09	2.00E-06	+	0.220833	0.2012	+	A	G	0.336161439	0.0372012	+	A	G	0.3201	0.0423
rs72712459	LC_IGP_R3	IgG1_G2F/IgG1_G2	14	65734600	1	7.31E-10	6.04E-09	-	-0.229049	-0.2449	+	A	G	0.336936125	0.0369665	+	A	G	0.3201	0.0421
rs72712459	LC_IGP_R26	IgG1_G2N/IgG1_G1N	14	65734600	1	3.97E-09	4.36E-07	-	-0.21976	-0.2068	+	A	G	0.336471605	0.0371344	+	A	G	0.3201	0.0409
rs1256519	LC_IGP78	IgG1 BG0n/G0n	14	65736324	1	1.67E-09	1.78E-06	+	0.213341	0.1814	+	A	G	0.540872341	0.0351907	+	A	G	0.5293	0.038
rs1256519	LC_IGP79	IgG1 BG1n/G1n	14	65736324	1	1.35E-10	5.77E-07	+	0.227641	0.1899	+	A	G	0.540872341	0.0352189	+	A	G	0.5293	0.038
rs1256519	LC_IGP32	IgG1 BG1S1/(BG1+BG1S1)	14	65736324	1	4.93E-09	6.16E-08	-	-0.205906	-0.2017	+	A	G	0.541360666	0.0350098	+	A	G	0.5293	0.0372
rs1256519	LC_IGP77	IgG1 Bn	14	65736324	1	5.49E-10	4.82E-07	+	0.220094	0.192	+	A	G	0.540872341	0.0352559	+	A	G	0.5293	0.0382
rs1256519	LC_IGP86	IgG1 Bn/Fn total	14	65736324	1	2.55E-10	1.82E-07	+	0.22423	0.199	+	A	G	0.540872341	0.0352319	+	A	G	0.5293	0.0382
rs1256519	LC_IGP84	IgG1 FBn/Bn total	14	65736324	1	3.27E-10	5.11E-11	-	-0.223327	-0.2521	+	A	G	0.540872341	0.0353087	+	A	G	0.5293	0.0384
rs1256519	LC_IGP62	IgG1 FG0n total/G0n	14	65736324	1	1.75E-09	8.30E-08	-	-0.212816	-0.2048	+	A	G	0.540872341	0.0351498	+	A	G	0.5293	0.0382
rs1256519	LC_IGP63	IgG1 FG1n total/G1n	14	65736324	1	3.58E-10	6.98E-09	-	-0.222257	-0.2213	+	A	G	0.540872341	0.0352196	+	A	G	0.5293	0.0382
rs1256519	LC_IGP61	IgG1 Fn total	14	65736324	1	8.35E-10	1.16E-08	-	-0.217443	-0.2182	+	A	G	0.540872341	0.0352129	+	A	G	0.5293	0.0382
rs1256519	LC_IGP21	IgG1 Fucosylation	14	65736324	1	1.15E-08	4.83E-08	-	-0.202033	-0.2088	+	A	G	0.542850493	0.0352138	+	A	G	0.5293	0.0383
rs1256519	LC_IGP11	IgG1_G0	14	65736324	1	1.65E-08	2.42E-09	+	0.198451	0.2273	+	A	G	0.542246793	0.0349801	+	A	G	0.5293	0.0381
rs1256519	LC_IGP_R1	IgG1_G0F/IgG1_G0	14	65736324	1	9.09E-09	3.33E-07	-	-0.201915	-0.1945	+	A	G	0.542246793	0.034952	+	A	G	0.5293	0.0381
rs1256519	LC_IGP_R4	IgG1_G0FN/IgG1_G0N	14	65736324	1	1.09E-09	1.95E-09	-	-0.214843	-0.23	+	A	G	0.542186773	0.0350373	+	A	G	0.5293	0.0383
rs1256519	LC_IGP52	IgG1_G0n	14	65736324	1	1.86E-08	2.86E-09	+	0.198832	0.2266	+	A	G	0.540872341	0.0351746	+	A	G	0.5293	0.0382
rs1256519	LC_IGP14	IgG1_G0N	14	65736324	1	8.37E-11	9.21E-08	+	0.22863	0.2028	+	A	G	0.542186773	0.0349748	+	A	G	0.5293	0.038
rs1256519	LC_IGP55	IgG1_G0Nn	14	65736324	1	1.48E-10	1.11E-07	+	0.226839	0.2017	+	A	G	0.540872341	0.0351698	+	A	G	0.5293	0.038
rs1256519	LC_IGP12	IgG1_G1	14	65736324	1	9.43E-09	1.33E-08	+	0.202144	0.217	+	A	G	0.542246793	0.0350303	+	A	G	0.5293	0.0382
rs1256519	LC_IGP_R2	IgG1_G1F/IgG1_G1	14	65736324	1	8.64E-10	3.82E-08	-	-0.215884	-0.2097	+	A	G	0.542246793	0.0349948	+	A	G	0.5293	0.0381
rs1256519	LC_IGP_R5	IgG1_G1FN/IgG1_G1N	14	65736324	1	3.40E-11	2.18E-11	-	-0.234423	-0.2557	+	A	G	0.54096433	0.0351241	+	A	G	0.5293	0.0382
rs1256519	LC_IGP53	IgG1_G1n	14	65736324	1	4.35E-08	2.19E-08	+	0.19421	0.2136	+	A	G	0.540872341	0.0352942	+	A	G	0.5293	0.0382
rs1256519	LC_IGP15	IgG1_G1N	14	65736324	1	8.93E-10	2.17E-06	+	0.216817	0.1799	+	A	G	0.54096433	0.0351749	+	A	G	0.5293	0.038
rs1256519	LC_IGP_R36	IgG1_G1NS1/IgG1_G1N	14	65736324	1	6.99E-09	5.72E-08	-	-0.203922	-0.2024	+	A	G	0.541360666	0.0350254	+	A	G	0.5293	0.0373
rs1256519	LC_IGP_R3	IgG1_G2F/IgG1_G2	14	65736324	1	1.13E-08	1.04E-08	-	-0.201249	-0.2172	+	A	G	0.542286064	0.0350621	+	A	G	0.5293	0.038
rs76922762	LC_IGP84	IgG1 FBn/Bn total	14	65737580	1	1.20E-08	5.86E-07	-	-0.212076	-0.2121	+	G	C	0.324156335	0.0370162	+	G	C	0.2955	0.0425
rs76922762	LC_IGP11	IgG1_G0	14	65737580	1	1.50E-09	8.46E-07	+	0.222019	0.2068	+	G	C	0.323922922	0.0365222	+	G	C	0.2955	0.042
rs76922762	LC_IGP52	IgG1_G0n	14	65737580	1	2.13E-09	1.39E-06	+	0.221277	0.2031	+	G	C	0.324156335	0.0367472	+	G	C	0.2955	0.0421
rs76922762	LC_IGP_R5	IgG1_G1FN/IgG1_G1N	14	65737580	1	1.91E-08	2.92E-07	-	-0.208416	-0.2165	+	G	C	0.323037407	0.0369005	+	G	C	0.2955	0.0422
rs1256527	LC_IGP78	IgG1 BG0n/G0n	14	65740296	1	3.37E-09	8.37E-07	+	0.210909	0.1869	+	G	A	0.543703222	0.0354717	+	G	A	0.5291	0.038
rs1256527	LC_IGP79	IgG1 BG1n/G1n	14	65740296	1	2.81E-10	2.28E-07	+	0.225404	0.1965	+	G	A	0.543703222	0.0355008	+	G	A	0.5291	0.038
rs1256527	LC_IGP32	IgG1 BG1S1/(BG1+BG1S1)	14	65740296	1	8.48E-09	3.06E-08	-	-0.204267	-0.2065	+	G	A	0.544252774	0.0352842	+	G	A	0.5291	0.0373
rs1256527	LC_IGP77	IgG1 Bn	14	65740296	1	1.31E-09	2.01E-07	+	0.216862	0.1982	+	G	A	0.543703222	0.0355411	+	G	A	0.5291	0.0381
rs1256527	LC_IGP86	IgG1 Bn/Fn total	14	65740296	1	6.08E-10	7.49E-08	+	0.22114	0.2052	+	G	A	0.543703222	0.0355168	+	G	A	0.5291	0.0381
rs1256527	LC_IGP84	IgG1 FBn/Bn total	14	65740296	1	6.68E-10	2.09E-11	-	-0.221056	-0.2576	+	G	A	0.543703222	0.0355909	+	G	A	0.5291	0.0384
rs1256527	LC_IGP62	IgG1 FG0n total/G0n	14	65740296	1	3.60E-09	3.64E-08	-	-0.210259	-0.2105	+	G	A	0.543703222	0.0354308	+	G	A	0.5291	0.0382
rs1256527	LC_IGP63	IgG1 FG1n total/G1n	14	65740296	1	6.55E-10	2.55E-09	-	-0.220602	-0.2279	+	G	A	0.543703222	0.0354987	+	G	A	0.5291	0.0382
rs1256527	LC_IGP61	IgG1 Fn total	14	65740296	1	1.72E-09	4.30E-09	-	-0.214989	-0.2247	+	G	A	0.543703222	0.0354945	+	G	A	0.5291	0.0383
rs1256527	LC_IGP81	IgG1 Fn/Bn	14	65740296	1	1.42E-09	1.50E-06	-	-0.216276	-0.1827	+	G	A	0.543703222	0.0355233	+	G	A	0.5291	0.038
rs1256527	LC_IGP21	IgG1 Fucosylation	14	65740296	1	2.58E-08	1.90E-08	-	-0.198599	-0.215	+	G	A	0.545682799	0.0354919	+	G	A	0.5291	0.0382
rs1256527	LC_IGP11	IgG1_G0	14	65740296	1	2.73E-08	1.25E-09	+	0.196873	0.2314	+	G	A	0.545073916	0.0352507	+	G	A	0.5291	0.0381
rs1256527	LC_IGP_R1	IgG1_G0F/IgG1_G0	14	65740296	1	1.90E-08	1.47E-07	-	-0.199012	-0.2003	+	G	A	0.545073916	0.0352273	+	G	A	0.5291	0.0381
rs1256527	LC_IGP_R4	IgG1_G0FN/IgG1_G0N	14	65740296	1	1.81E-09	8.84E-10	-	-0.213577	-0.2351	+	G	A	0.545080527	0.035313	+	G	A	0.5291	0.0384
rs1256527	LC_IGP52	IgG1_G0n	14	65740296	1	3.16E-08	1.42E-09	+	0.197098	0.231	+	G	A	0.543703222	0.0354516	+	G	A	0.5291	0.0382
rs1256527	LC_IGP14	IgG1_G0N	14	65740296	1	1.51E-10	5.02E-08	+	0.227214	0.2066	+	G	A	0.545080527	0.0352518	+	G	A	0.5291	0.0379
rs1256527	LC_IGP55	IgG1_G0Nn	14	65740296	1	2.92E-10	5.83E-08	+	0.224855	0.2058	+	G	A	0.543703222	0.0354502	+	G	A	0.5291	0.0379
rs1256527	LC_IGP12	IgG1_G1	14	65740296	1	1.73E-08	5.29E-09	+	0.200012	0.2233	+	G	A	0.545073916	0.0353036	+	G	A	0.5291	0.0382
rs1256527	LC_IGP_R2	IgG1_G1F/IgG1_G1	14	65740296	1	1.54E-09	1.44E-08	-	-0.214233	-0.2163	+	G	A	0.545073916	0.0352673	+	G	A	0.5291	0.0382
rs1256527	LC_IGP_R5	IgG1_G1FN/IgG1_G1N	14	65740296	1	5.65E-11	8.41E-12	-	-0.233527	-0.2614	+	G	A	0.543851476	0.0353974	+	G	A	0.5291	0.0383
rs1256527	LC_IGP15	IgG1_G1N	14	65740296	1	1.95E-09	9.26E-07	+	0.214011	0.1864	+	G	A	0.543851476	0.0354544	+	G	A	0.5291	0.038
rs1256527	LC_IGP56	IgG1_G1Nn	14	65740296	1	2.67E-09	1.77E-06	+	0.212791	0.1813	+	G	A	0.543703222	0.0355557	+	G	A	0.5291	0.0379
rs1256527	LC_IGP_R36	IgG1_G1NS1/IgG1_G1N	14	65740296	1	1.21E-08	2.84E-08	-	-0.202181	-0.2071	+	G	A	0.544252774	0.0353001	+	G	A	0.5291	0.0373
rs1256527	LC_IGP_R3	IgG1_G2F/IgG1_G2	14	65740296	1	2.00E-08	3.92E-09	-	-0.199296	-0.2236	+	G	A	0.545098411	0.0353346	+	G	A	0.5291	0.038
rs79230737	LC_IGP84	IgG1 FBn/Bn total	14	65742463	1	3.03E-09	3.16E-07	-	-0.223394	-0.218	+	T	C	0.323598823	0.037461	+	T	C	0.295	0.0426
rs79230737	LC_IGP11	IgG1_G0	14	65742463	1	4.98E-10	4.57E-07	+	0.231298	0.2126	+	T	C	0.323370189	0.0369615	+	T	C	0.295	0.0421
rs79230737	LC_IGP52	IgG1_G0n	14	65742463	1	7.04E-10	7.66E-07	+	0.230706	0.2088	+	T	C	0.323598823	0.0371948	+	T	C	0.295	0.0422
rs79230737	LC_IGP_R5	IgG1_G1FN/IgG1_G1N	14	65742463	1	6.01E-09	1.71E-07	-	-0.218398	-0.2217	+	T	C	0.32248278	0.0373469	+	T	C	0.295	0.0424
rs79230737	LC_IGP_R3	IgG1_G2F/IgG1_G2	14	65742463	1	2.94E-08	7.98E-07	-	-0.206993	-0.2074	+	T	C	0.323262225	0.0371496	+	T	C	0.295	0.042
rs9783622	LC_IGP84	IgG1 FBn/Bn total	14	65749045	1	9.94E-09	3.58E-09	-	-0.21726	-0.2574	+	G	C	0.318644981	0.0377053	+	G	C	0.2775	0.0436
rs9783622	LC_IGP62	IgG1 FG0n total/G0n	14	65749045	1	4.27E-09	8.98E-07	-	-0.221337	-0.2134	+	G	C	0.318644981	0.0374784	+	G	C	0.2775	0.0434
rs9783622	LC_IGP63	IgG1 FG1n total/G1n	14	65749045	1	1.02E-08	6.64E-08	-	-0.216539	-0.2348	+	G	C	0.318644981	0.0376086	+	G	C	0.2775	0.0435
rs9783622	LC_IGP61	IgG1 Fn total	14	65749045	1	6.56E-09	7.73E-08	-	-0.219163	-0.2337	+	G	C	0.318644981	0.0375722	+	G	C	0.2775	0.0435
rs9783622	LC_IGP_R2	IgG1_G1F/IgG1_G1	14	65749045	1	3.11E-08	2.00E-07	-	-0.207576	-0.2257	+	G	C	0.318620648	0.0373215	+	G	C	0.2775	0.0434
rs9783622	LC_IGP_R5	IgG1_G1FN/IgG1_G1N	14	65749045	1	4.86E-08	1.27E-09	-	-0.206057	-0.2638	+	G	C	0.317707872	0.0375856	+	G	C	0.2775	0.0434
rs12147994	LC_IGP84	IgG1 FBn/Bn total	14	65751627	1	4.82E-09	9.63E-09	-	-0.22457	-0.2568	+	A	C	0.300618526	0.0381572	+	A	C	0.2665	0.0448

rs12147994	LC_IGP62	IgG1 FG0n total/G0n	14	65751627	1	4.65E-10	1.72E-06	-	-0.237565	-0.2129	+	A	C	0.300618526	0.0378933 +	A	C	0.2665	0.0445
rs12147994	LC_IGP63	IgG1 FG1n total/G1n	14	65751627	1	1.58E-09	1.91E-07	-	-0.230908	-0.2317	+	A	C	0.300618526	0.0380332 +	A	C	0.2665	0.0445
rs12147994	LC_IGP61	IgG1 Fn total	14	65751627	1	1.81E-09	1.89E-07	-	-0.229915	-0.232	+	A	C	0.300618526	0.0380096 +	A	C	0.2665	0.0445
rs12147994	LC_IGP21	IgG1 Fucosylation	14	65751627	1	1.41E-08	5.77E-07	-	-0.216339	-0.2222	+	A	C	0.300673737	0.0379425 +	A	C	0.2665	0.0444
rs12147994	LC_IGP11	IgG1_G0	14	65751627	1	6.89E-10	1.58E-07	+	0.233538	0.2324	+	A	C	0.30066573	0.0376329 +	A	C	0.2665	0.0443
rs12147994	LC_IGP_R4	IgG1_G0FN/IgG1_G0N	14	65751627	1	3.16E-08	1.69E-07	-	-0.210661	-0.2336	+	A	C	0.30048365	0.0378919 +	A	C	0.2665	0.0447
rs12147994	LC_IGP52	IgG1_G0n	14	65751627	1	8.05E-10	1.91E-07	+	0.234132	0.2314	+	A	C	0.300618526	0.0378785 +	A	C	0.2665	0.0444
rs12147994	LC_IGP12	IgG1_G1	14	65751627	1	1.44E-08	1.91E-07	+	0.215186	0.2316	+	A	C	0.30066573	0.0377679 +	A	C	0.2665	0.0445
rs12147994	LC_IGP_R2	IgG1_G1F/IgG1_G1	14	65751627	1	3.62E-09	5.35E-07	-	-0.223987	-0.2225	+	A	C	0.30066573	0.0377524 +	A	C	0.2665	0.0444
rs12147994	LC_IGP_R5	IgG1_G1FN/IgG1_G1N	14	65751627	1	1.35E-08	4.02E-09	-	-0.217204	-0.262	+	A	C	0.299629951	0.0380495 +	A	C	0.2665	0.0445
rs12147994	LC_IGP53	IgG1_G1n	14	65751627	1	3.74E-08	3.37E-07	+	0.210538	0.2266	+	A	C	0.300618526	0.0380761 +	A	C	0.2665	0.0444
rs28666010	LC_IGP86	IgG1 Bn/Fn total	14	65758148	1	1.80E-08	1.80E-06	+	0.231371	0.2251	+	T	C	0.249311648	0.040887 +	T	C	0.2128	0.0472
rs28666010	LC_IGP84	IgG1 FBn/Bn total	14	65758148	1	3.46E-09	3.78E-10	-	-0.243161	-0.2974	+	T	C	0.249311648	0.0409293 +	T	C	0.2128	0.0475
rs28666010	LC_IGP62	IgG1 FG0n total/G0n	14	65758148	1	6.69E-10	1.34E-07	-	-0.252551	-0.2491	+	T	C	0.249311648	0.0406632 +	T	C	0.2128	0.0472
rs28666010	LC_IGP63	IgG1 FG1n total/G1n	14	65758148	1	1.17E-08	1.16E-08	-	-0.234239	-0.2692	+	T	C	0.249311648	0.0408537 +	T	C	0.2128	0.0472
rs28666010	LC_IGP61	IgG1 Fn total	14	65758148	1	3.58E-09	1.73E-08	-	-0.242141	-0.2663	+	T	C	0.249311648	0.0407958 +	T	C	0.2128	0.0472
rs28666010	LC_IGP21	IgG1 Fucosylation	14	65758148	1	1.22E-08	1.80E-08	-	-0.232954	-0.2656	+	T	C	0.249623921	0.0406817 +	T	C	0.2128	0.0472
rs28666010	LC_IGP11	IgG1_G0	14	65758148	1	6.47E-10	2.45E-08	+	0.251046	0.2619	+	T	C	0.249777337	0.0403896 +	T	C	0.2128	0.047
rs28666010	LC_IGP_R1	IgG1_G0F/IgG1_G0	14	65758148	1	1.29E-09	4.40E-07	-	-0.24653	-0.2378	+	T	C	0.249777337	0.0403881 +	T	C	0.2128	0.0471
rs28666010	LC_IGP_R4	IgG1_G0FN/IgG1_G0N	14	65758148	1	4.10E-09	1.12E-08	-	-0.23986	-0.2709	+	T	C	0.249777097	0.0405699 +	T	C	0.2128	0.0474
rs28666010	LC_IGP52	IgG1_G0n	14	65758148	1	9.28E-10	2.55E-08	+	0.250278	0.2621	+	T	C	0.249311648	0.0406419 +	T	C	0.2128	0.0471
rs28666010	LC_IGP12	IgG1_G1	14	65758148	1	3.95E-08	8.05E-09	+	0.223875	0.2721	+	T	C	0.249777337	0.0405602 +	T	C	0.2128	0.0472
rs28666010	LC_IGP_R2	IgG1_G1F/IgG1_G1	14	65758148	1	1.89E-08	3.87E-08	-	-0.229169	-0.2588	+	T	C	0.249777337	0.040559 +	T	C	0.2128	0.0471
rs28666010	LC_IGP_R5	IgG1_G1FN/IgG1_G1N	14	65758148	1	2.96E-08	1.03E-10	-	-0.227537	-0.3052	+	T	C	0.248701415	0.0408433 +	T	C	0.2128	0.0472
rs76824828	LC_IGP77	IgG1 Bn	14	65758236	1	3.73E-08	1.78E-06	+	0.227722	0.226	+	A	G	0.245258984	0.0411809 +	A	G	0.209	0.0473
rs76824828	LC_IGP86	IgG1 Bn/Fn total	14	65758236	1	1.77E-08	7.50E-07	+	0.233018	0.2341	+	A	G	0.245258984	0.0411534 +	A	G	0.209	0.0473
rs76824828	LC_IGP84	IgG1 FBn/Bn total	14	65758236	1	3.83E-09	1.66E-10	-	-0.244071	-0.3044	+	A	G	0.245258984	0.0411989 +	A	G	0.209	0.0476
rs76824828	LC_IGP62	IgG1 FG0n total/G0n	14	65758236	1	4.07E-10	7.05E-08	-	-0.257372	-0.2554	+	A	G	0.245258984	0.0409163 +	A	G	0.209	0.0474
rs76824828	LC_IGP63	IgG1 FG1n total/G1n	14	65758236	1	7.70E-09	4.56E-09	-	-0.238679	-0.2776	+	A	G	0.245258984	0.0411099 +	A	G	0.209	0.0474
rs76824828	LC_IGP61	IgG1 Fn total	14	65758236	1	2.07E-09	7.12E-09	-	-0.247378	-0.2743	+	A	G	0.245258984	0.0410485 +	A	G	0.209	0.0474
rs76824828	LC_IGP21	IgG1 Fucosylation	14	65758236	1	6.08E-09	7.44E-09	-	-0.23924	-0.2737	+	A	G	0.245865906	0.0409239 +	A	G	0.209	0.0474
rs76824828	LC_IGP11	IgG1_G0	14	65758236	1	1.79E-10	1.29E-08	+	0.260751	0.268	+	A	G	0.245781613	0.0406176 +	A	G	0.209	0.0471
rs76824828	LC_IGP_R1	IgG1_G0F/IgG1_G0	14	65758236	1	5.61E-10	2.44E-07	-	-0.253453	-0.2438	+	A	G	0.245781613	0.0406272 +	A	G	0.209	0.0472
rs76824828	LC_IGP_R4	IgG1_G0FN/IgG1_G0N	14	65758236	1	3.41E-09	5.75E-09	-	-0.242645	-0.2771	+	A	G	0.245771586	0.0408263 +	A	G	0.209	0.0476
rs76824828	LC_IGP52	IgG1_G0n	14	65758236	1	2.70E-10	1.37E-08	+	0.259775	0.268	+	A	G	0.245258984	0.0408766 +	A	G	0.209	0.0472
rs76824828	LC_IGP14	IgG1_G0N	14	65758236	1	1.13E-09	1.46E-06	+	0.249851	0.2264	+	A	G	0.245771586	0.0407892 +	A	G	0.209	0.047
rs76824828	LC_IGP55	IgG1_G0Nn	14	65758236	1	3.06E-09	1.44E-06	+	0.244734	0.2267	+	A	G	0.245258984	0.041051 +	A	G	0.209	0.047
rs76824828	LC_IGP12	IgG1_G1	14	65758236	1	2.34E-08	3.40E-09	+	0.229026	0.2798	+	A	G	0.245781613	0.0408079 +	A	G	0.209	0.0473
rs76824828	LC_IGP_R2	IgG1_G1F/IgG1_G1	14	65758236	1	9.61E-09	1.57E-08	-	-0.235329	-0.2671	+	A	G	0.245781613	0.040803 +	A	G	0.209	0.0472
rs76824828	LC_IGP_R5	IgG1_G1FN/IgG1_G1N	14	65758236	1	2.39E-08	3.94E-11	-	-0.230553	-0.313	+	A	G	0.244678659	0.0411044 +	A	G	0.209	0.0474
rs76824828	LC_IGP_R3	IgG1_G2F/IgG1_G2	14	65758236	1	4.34E-08	5.56E-10	-	-0.224853	-0.2915	+	A	G	0.245628056	0.0408639 +	A	G	0.209	0.047
rs7159888	LC_IGP78	IgG1 BG0n/G0n	14	65758642	1	3.18E-10	5.17E-11	+	0.225457	0.2508	+	A	G	0.420082094	0.0356196 +	A	G	0.3973	0.0382
rs7159888	LC_IGP79	IgG1 BG1n/G1n	14	65758642	1	7.43E-10	3.38E-11	+	0.221257	0.2529	+	A	G	0.420082094	0.0357208 +	A	G	0.3973	0.0382
rs7159888	LC_IGP77	IgG1 Bn	14	65758642	1	2.78E-10	3.57E-11	+	0.226768	0.2538	+	A	G	0.420082094	0.0357067 +	A	G	0.3973	0.0383
rs7159888	LC_IGP86	IgG1 Bn/Fn total	14	65758642	1	9.73E-11	9.62E-12	+	0.23241	0.2612	+	A	G	0.420082094	0.0356762 +	A	G	0.3973	0.0384
rs7159888	LC_IGP84	IgG1 FBn/Bn total	14	65758642	1	5.12E-11	5.62E-16	-	-0.236303	-0.3129	+	A	G	0.420082094	0.0357347 +	A	G	0.3973	0.0386
rs7159888	LC_IGP62	IgG1 FG0n total/G0n	14	65758642	1	2.84E-11	6.12E-13	-	-0.238075	-0.2767	+	A	G	0.420082094	0.035525 +	A	G	0.3973	0.0384
rs7159888	LC_IGP63	IgG1 FG1n total/G1n	14	65758642	1	2.72E-11	5.74E-14	-	-0.23901	-0.2886	+	A	G	0.420082094	0.0356288 +	A	G	0.3973	0.0384
rs7159888	LC_IGP64	IgG1 FG2n total/G2n	14	65758642	1	3.36E-09	1.05E-13	-	-0.212695	-0.284	+	A	G	0.420082094	0.0357701 +	A	G	0.3973	0.0382
rs7159888	LC_IGP61	IgG1 Fn total	14	65758642	1	2.50E-11	1.05E-13	-	-0.239283	-0.2858	+	A	G	0.420082094	0.0356019 +	A	G	0.3973	0.0384
rs7159888	LC_IGP81	IgG1 Fn/Bn	14	65758642	1	2.31E-10	4.20E-10	-	-0.227659	-0.2384	+	A	G	0.420082094	0.035683 +	A	G	0.3973	0.0382
rs7159888	LC_IGP21	IgG1 Fucosylation	14	65758642	1	3.86E-11	6.30E-14	-	-0.23581	-0.288	+	A	G	0.421790382	0.0354322 +	A	G	0.3973	0.0384
rs7159888	LC_IGP11	IgG1_G0	14	65758642	1	8.37E-12	1.38E-13	+	0.242475	0.2829	+	A	G	0.421771839	0.0352325 +	A	G	0.3973	0.0382
rs7159888	LC_IGP_R1	IgG1_G0F/IgG1_G0	14	65758642	1	9.26E-11	3.14E-12	-	-0.229989	-0.2672	+	A	G	0.421771839	0.0352674 +	A	G	0.3973	0.0383
rs7159888	LC_IGP_R4	IgG1_G0FN/IgG1_G0N	14	65758642	1	1.86E-11	1.73E-14	-	-0.239086	-0.296	+	A	G	0.421747704	0.035343 +	A	G	0.3973	0.0386
rs7159888	LC_IGP52	IgG1_G0n	14	65758642	1	1.03E-11	6.98E-14	+	0.243109	0.2869	+	A	G	0.420082094	0.0354775 +	A	G	0.3973	0.0383
rs7159888	LC_IGP14	IgG1_G0N	14	65758642	1	5.15E-13	1.38E-11	+	0.256697	0.2575	+	A	G	0.421747704	0.0352581 +	A	G	0.3973	0.0381
rs7159888	LC_IGP55	IgG1_G0Nn	14	65758642	1	4.62E-12	7.66E-12	+	0.247828	0.261	+	A	G	0.420082094	0.0355592 +	A	G	0.3973	0.0381
rs7159888	LC_IGP12	IgG1_G1	14	65758642	1	9.75E-10	1.65E-13	+	0.217847	0.2832	+	A	G	0.421466402	0.0354252 +	A	G	0.3973	0.0384
rs7159888	LC_IGP_R2	IgG1_G1F/IgG1_G1	14	65758642	1	6.63E-11	4.52E-13	-	-0.232553	-0.2774	+	A	G	0.421466402	0.0353828 +	A	G	0.3973	0.0383
rs7159888	LC_IGP_R5	IgG1_G1FN/IgG1_G1N	14	65758642	1	4.25E-11	2.23E-16	-	-0.236056	-0.3155	+	A	G	0.420314883	0.0355481 +	A	G	0.3973	0.0384
rs7159888	LC_IGP53	IgG1_G1n	14	65758642	1	2.99E-09	1.54E-13	+	0.212983	0.2834	+	A	G	0.420082094	0.0357025 +	A	G	0.3973	0.0384
rs7159888	LC_IGP15	IgG1_G1N	14	65758642	1	2.88E-09	2.19E-10	+	0.212699	0.2424	+	A	G	0.420314883	0.0356198 +	A	G	0.3973	0.0382
rs7159888	LC_IGP56	IgG1_G1Nn	14	65758642	1	7.22E-09	3.04E-10	+	0.208107	0.2401	+	A	G	0.420082094	0.0357764 +	A	G	0.3973	0.0381
rs7159888	LC_IGP_R3	IgG1_G2F/IgG1_G2	14	65758642	1	5.61E-10	1.34E-13	-	-0.220923	-0.2822	+	A	G	0.4217228	0.0354123 +	A	G	0.3973	0.0381
rs10047879	LC_IGP78	IgG1 BG0n/G0n	14	65760543	1	4.42E-09	4.76E-08	+	0.208617	0.2116	+	A	C	0.381189901	0.0353597 +	A	C	0.3532	0.0388
rs10047879	LC_IGP77	IgG1 Bn	14	65760543	1	1.85E-08	7.84E-09	+	0.200607	0.2245	+	A	C	0.381189901	0.0354796 +	A	C	0.3532	0.0389
rs10047879	LC_IGP86	IgG1 Bn/Fn total	14	65760543	1	7.82E-09	3.00E-09	+	0.205744	0.2308	+	A	C	0.381189901	0.0354535 +	A	C	0.3532	0.0389
rs10047879	LC_IGP84	IgG1 FBn/Bn total	14	65760543	1	2.43E-09	1.55E-13	-	-0.213012	-0.2894	+	A	C	0.381189901	0.0355003 +	A	C	0.3532	0.0392
rs10047879	LC_IGP62	IgG1 FG0n total/G0n	14	65760543	1	1.03E-09	1.65E-09	-	-0.216709	-0.2351	+	A	C	0.381189901	0.0352863 +	A	C	0.3532	0.039
rs10047879	LC_IGP63	IgG1 FG1n total/G1n	14	65760543	1	1.26E-09	1.15E-11	-	-0.216188	-0.2644	+	A	C	0.381189901	0.0353948 +	A	C	0.3532	0.039
rs10047879	LC_IGP61	IgG1 Fn total	14	65760543	1	6.63E-10	3.95E-11	-	-0.219647	-0.2576	+	A	C	0.381189901	0.0353561 +	A	C	0.3532	0.039
rs10047879	LC_IGP81	IgG1 Fn/Bn	14	65760543	1	2.03E-08	7.04E-08	-	-0.199929	-0.2087	+	A	C	0.381189901	0.0354621 +	A	C	0.3532	0.0387
rs10047879	LC_IGP21	IgG1 Fucosylation	14	65760543	1	4.93E-09	6.39E-11	-	-0.207398	-0.2544	+	A	C	0.382053946	0.0352624 +	A	C	0.3532	0.0389
rs10047879	LC_IGP_R1	IgG1_G0F/IgG1_G0	14	65760543	1	7.39E-09	8.52E-09	-	-0.20387	-0.2238	+	A	C	0.382243433	0.0350746 +	A	C	0.3532	0.0389
rs10047879	LC_IGP_R4	IgG1_G0FN/IgG1_G0N	14	65760543	1	1.28E-09	2.32E-11	-	-0.21486	-0.2618	+	A	C	0.381955297	0.0351912 +	A	C	0.3532	0.0392
rs10047879	LC_IGP14	IgG1_G0N	14	65760543	1	5.47E-09	1.06E-08	+	0.206477	0.221	+	A	C	0.381955297	0.0352129 +	A	C	0.3532	0.0386
rs10047879	LC_IGP55	IgG1_G0Nn	14	65760543	1	1.16E-08	7.13E-09	+	0.203092	0.2238	+	A	C	0.381189901	0.0354111 +	A	C	0.3532	0.0387
rs10047879	LC_IGP12	IgG1_G1	14	65760543	1	1.01E-08	3.95E-11	+	0.202679	0.2574	+	A	C	0.381937996	0.0351915 +	A	C	0.3532	0.039
rs10047879	LC_IGP_R2	IgG1_G1F/IgG1_G1	14	65760543	1	5.67E-09	6.61E-11	-	-0.206151	-0.2538	+	A	C	0.381937996	0.0351938 +	A	C	0.3532	0.0389
rs10047879	LC_IGP_R5	IgG1_G1FN/IgG1_G1N	14	65760543	1	7.04E-09	2.53E-14	-	-0.205781	-0.2971	+	A	C	0.381140221	0.0353527 +	A	C	0.3532	0.039
rs10047879	LC_IGP53	IgG1_G1n	14	65760543	1	5.97E-09	4.52E-11	+	0.207063	0.2564	+	A	C	0.381189901	0.0354003 +	A	C	0.3532	0.0389
rs1256536	LC_IGP78	IgG1 BG0n/G0n	14	65762476	1	3.57E-09	2.23E-08	+	0.210034	0.2183	+	G	A	0.380284077	0.0353844 +	G	A	0.3478	0.039
rs1256536	LC_IGP77	IgG1 Bn	14	65762476	1	1.43E-08	3.82E-09	+	0.202339	0.2306	+	G	A	0.380284077	0.0355034 +	G	A	0.3478	0.0391
rs1256536	LC_IGP86	IgG1 Bn/Fn total	14	65762476	1	5.96E-09	1.37E-09	+	0.207518	0.2372	+	G	A	0.380284077	0.035477 +	G	A	0.3478	0.0392
rs1256536	LC_IGP84	IgG1 FBn/Bn total	14	65762476	1	1.76E-09	5.44E-14	-	-0.215029	-0.2969	+	G	A	0.380284077	0.0355228 +	G	A	0.3478	0.0395
rs1256536	LC_IGP62	IgG1 FG0n total/G0n	14	65762476	1	8.74E-10	5.82E-10	-	-0.217793	-0.2433	+	G	A	0.380284077	0.0353121 +	G	A	0.3478	0.0393
rs1256536	LC_IGP63	IgG1 FG1n total/G1n	14	65762476	1	8.56E-10	3.30E-12	-	-0.218558	-0.2729	+	G	A	0.380284077	0.0354157 +	G	A	0.3478	0.0392
rs1256536	LC_IGP61	IgG1 Fn total	14	65762476	1	4.81E-10	1.40E-11	-	-0.2216	-0.2652	+	G	A	0.380284077	0.0353785 +	G	A	0.3478	0.0392
rs1256536	LC_IGP81	IgG1 Fn/Bn	14	65762476	1	1.56E-08	3.65E-08	-	-0.201685	-0.2146	+	G	A	0.380284077	0.0354859 +	G	A	0.3478	0.039
rs1256536	LC_IGP21	IgG1 Fucosylation	14	65762476	1	3.48E-09	1.91E-11	-	-0.209587	-0.2631	+	G	A	0.381159063	0.035283 +	G	A	0.3478	0.0392
rs1256536	LC_IGP_R1	IgG1_G0F/IgG1_G0	14	65762476	1	6.56E-09	2.93E-09	-	-0.204729	-0.2323	+	G	A	0.381354307	0.0350998 +	G	A	0.3478	0.0391
rs1256536	LC_IGP_R4	IgG1_G0FN/IgG1_G0N	14	65762476	1	1.03E-09	8.81E-12	-	-0.216253	-0.2692	+	G	A	0.381063686	0.0352145 +	G	A	0.3478	0.0394
rs1256536	LC_IGP14	IgG1_G0N	14	65762476	1	5.42E-09	4.61E-09	+	0.20669	0.2279	+	G	A	0.381063686	0.0352407 +	G	A	0.3478	0.0389
rs1256536	LC_IGP55	IgG1_G0Nn	14	65762476	1	1.14E-08	3.13E-09	+	0.203369	0.2306	+	G	A	0.380284077	0.03544 +	G	A	0.3478	0.0389
rs1256536	LC_IGP12	IgG1_G1	14	65762476	1	6.98E-09	1.13E-11	+	0.205005	0.2661	+	G	A	0.38104887	0.0352118 +	G	A	0.3478	0.0392
rs1256536	LC_IGP_R2	IgG1_G1F/IgG1_G1	14	65762476	1	4.00E-09	2.00E-11	-	-0.208346	-0.2622	+	G	A	0.38104887	0.0352145 +	G	A	0.3478	0.0391
rs1256536	LC_IGP_R5	IgG1_G1FN/IgG1_G1N	14	65762476	1	4.64E-09	6.48E-15	-	-0.208395	-0.3058	+	G	A	0.380244465	0.0353723 +	G	A	0.3478	0.0392
rs1256536	LC_IGP53	IgG1_G1n	14	65762476	1	3.96E-09	1.40E-11	+	0.209643	0.2648	+	G	A	0.380284077	0.0354208 +	G	A	0.3478	0.0392
rs1256540	LC_IGP78	IgG1 BG0n/G0n	14	65764069	1	9.16E-09	4.39E-08	+	0.202688	0.2118	+	C	T	0.382874226	0.0350917 +	C	T	0.3567	0.0387
rs1256540	LC_IGP77	IgG1 Bn	14	65764069	1	2.65E-08	8.25E-09	+	0.196804	0.2238	+	C	T	0.382874226	0.0352028 +	C	T	0.3567	0.0388
rs1256540	LC_IGP86	IgG1 Bn/Fn total	14	65764069	1	1.14E-08	2.92E-09	+	0.201845	0.2305	+	C	T	0.382874226	0.0351773 +	C	T	0.3567	0.0388
rs1256540	LC_IGP84	IgG1 FBn/Bn total	14	65764069	1	7.98E-09	2.03E-13	-	-0.204388	-0.2874	+	C	T	0.382874226	0.0352411 +	C	T	0.3567	0.0391
rs1256540	LC_IGP62	IgG1 FG0n total/G0n	14	65764069	1	2.56E-09	1.30E-09	-	-0.209831	-0.2362	+	C	T	0.382874226	0.0350228 +	C	T	0.3567	0.0389
rs1256540	LC_IGP63	IgG1 FG1n total/G1n	14	65764069	1	2.53E-09	1.02E-11	-	-0.21052	-0.2646	+	C	T	0.382874226	0.0351258 +	C	T	0.3567	0.0389
rs1256540	LC_IGP61	IgG1 Fn total	14	65764069	1	1.47E-09	3.40E-11	-	-0.213455	-0.258	+	C	T	0.382874226	0.0350896 +	C	T	0.3567	0.0389
rs1256540	LC_IGP81	IgG1 Fn/Bn	14	65764069	1	2.45E-08	7.32E-08	-	-0.197174	-0.2081	+	C	T	0.382874226	0.0351818 +	C	T	0.3567	0.0386
rs1256540	LC_IGP21	IgG1 Fucosylation	14	65764069	1	1.18E-08	4.95E-11	-	-0.200618	-0.2555	+	C	T	0.383740136	0.0349996 +	C	T	0.3567	0.0389
rs1256540	LC_IGP_R1	IgG1_G0F/IgG1_G0	14	65764069	1	1.46E-08	6.34E-09	-	-0.198237	-0.2254	+	C	T	0.38390898	0.0348109 +	C	T	0.3567	0.0388
rs1256540	LC_IGP_R4	IgG1_G0FN/IgG1_G0N	14	65764069	1	5.25E-09	2.44E-11	-	-0.205122	-0.2609	+	C	T	0.383624617	0.034941 +	C	T	0.3567	0.0391
rs1256540	LC_IGP14	IgG1_G0N	14	65764069	1	1.37E-08	8.20E-09	+	0.199431	0.2223	+	C	T	0.383624617	0.0349523 +	C	T	0.3567	0.0386
rs1256540	LC_IGP55	IgG1_G0Nn	14	65764069	1	2.73E-08	5.68E-09	+	0.196313	0.2249	+	C	T	0.382874226	0.0351453 +	C	T	0.3567	0.0386
rs1256540	LC_IGP12	IgG1_G1	14	65764069	1	2.17E-08	3.63E-11	+	0.196497	0.2574	+	C	T	0.383603543	0.034928 +	C	T	0.3567	0.0389
rs1256540	LC_IGP_R2	IgG1_G1F/IgG1_G1	14	65764069	1	9.56E-09	5.24E-11	-	-0.201455	-0.2548	+	C	T	0.383603543	0.0349253 +	C	T	0.3567	0.0388
rs1256540	LC_IGP_R5	IgG1_G1FN/IgG1_G1N	14	65764069	1	1.68E-08	3.58E-14	-	-0.198992	-0.2948	+	C	T	0.382816113	0.035089 +	C	T	0.3567	0.0389
rs1256540	LC_IGP53	IgG1_G1n	14	65764069	1	1.29E-08	4.16E-11	+	0.200864	0.2564	+	C	T	0.382874226	0.0351328 +	C	T	0.3567	0.0389
rs1269068	LC_IGP78	IgG1 BG0n/G0n	14	65767333	1	6.17E-09	2.28E-08	+	0.204275	0.2149	+	T	C	0.383828996	0.034958 +	T	C	0.3584	0.0384
rs1269068	LC_IGP77	IgG1 Bn	14	65767333	1	2.01E-08	4.77E-09	+	0.19777	0.2259	+	T	C	0.383828996	0.0350712 +	T	C	0.3584	0.0386
rs1269068	LC_IGP86	IgG1 Bn/Fn total	14	65767333	1	8.64E-09	1.65E-09	+	0.202776	0.2327	+	T	C	0.383828996	0.0350457 +	T	C	0.3584	0.0386

rs1269068	LC_IGP84	IgG1 FBn/Bn total	14	65767333	1	5.91E-09	1.02E-13	-	-0.205419	-0.2893	+	T	C	0.383828996	0.0351088	+	T	C	0.3584	0.0389
rs1269068	LC_IGP62	IgG1 FG0n total/G0n	14	65767333	1	2.11E-09	6.33E-10	-	-0.210171	-0.2392	+	T	C	0.383828996	0.0348936	+	T	C	0.3584	0.0387
rs1269068	LC_IGP63	IgG1 FG1n total/G1n	14	65767333	1	2.47E-09	5.63E-12	-	-0.20991	-0.2663	+	T	C	0.383828996	0.0349998	+	T	C	0.3584	0.0387
rs1269068	LC_IGP61	IgG1 Fn total	14	65767333	1	1.34E-09	1.90E-11	-	-0.213224	-0.2598	+	T	C	0.383828996	0.0349622	+	T	C	0.3584	0.0387
rs1269068	LC_IGP81	IgG1 Fn/Bn	14	65767333	1	1.91E-08	4.39E-08	-	-0.197997	-0.2103	+	T	C	0.383828996	0.0350508	+	T	C	0.3584	0.0384
rs1269068	LC_IGP21	IgG1 Fucosylation	14	65767333	1	1.04E-08	2.34E-11	-	-0.200646	-0.2582	+	T	C	0.384710234	0.0348723	+	T	C	0.3584	0.0386
rs1269068	LC_IGP_R1	IgG1_G0F/IgG1_G0	14	65767333	1	1.37E-08	3.18E-09	-	-0.197944	-0.2284	+	T	C	0.384850336	0.034687	+	T	C	0.3584	0.0386
rs1269068	LC_IGP_R4	IgG1_G0FN/IgG1_G0N	14	65767333	1	3.70E-09	1.09E-11	-	-0.206413	-0.2639	+	T	C	0.384568279	0.0348103	+	T	C	0.3584	0.0388
rs1269068	LC_IGP14	IgG1_G0N	14	65767333	1	1.06E-08	3.28E-09	+	0.20023	0.2268	+	T	C	0.384568279	0.0348236	+	T	C	0.3584	0.0383
rs1269068	LC_IGP55	IgG1_G0Nn	14	65767333	1	2.07E-08	2.32E-09	+	0.197287	0.2292	+	T	C	0.383828996	0.0350139	+	T	C	0.3584	0.0384
rs1269068	LC_IGP12	IgG1_G1	14	65767333	1	2.20E-08	1.87E-11	+	0.195711	0.2596	+	T	C	0.384544899	0.0348051	+	T	C	0.3584	0.0386
rs1269068	LC_IGP_R2	IgG1_G1F/IgG1_G1	14	65767333	1	1.01E-08	2.89E-11	-	-0.200445	-0.2566	+	T	C	0.384544899	0.0348031	+	T	C	0.3584	0.0386
rs1269068	LC_IGP_R5	IgG1_G1FN/IgG1_G1N	14	65767333	1	1.52E-08	1.79E-14	-	-0.198884	-0.2965	+	T	C	0.383763838	0.0349625	+	T	C	0.3584	0.0387
rs1269068	LC_IGP53	IgG1_G1n	14	65767333	1	1.28E-08	2.31E-11	+	0.200168	0.2582	+	T	C	0.383828996	0.0350072	+	T	C	0.3584	0.0386
rs10135194	LC_IGP62	IgG1 FG0n total/G0n	14	65770978	1	2.80E-11	4.83E-10	-	-0.380451	-0.4257	+	T	C	0.107195123	0.05675	+	T	C	0.0852	0.0684
rs10135194	LC_IGP63	IgG1 FG1n total/G1n	14	65770978	1	2.75E-09	7.13E-10	-	-0.341287	-0.4223	+	T	C	0.107195123	0.0570758	+	T	C	0.0852	0.0685
rs10135194	LC_IGP64	IgG1 FG2n total/G2n	14	65770978	1	1.40E-08	5.32E-10	-	-0.326126	-0.4236	+	T	C	0.107195123	0.0571913	+	T	C	0.0852	0.0682
rs10135194	LC_IGP61	IgG1 Fn total	14	65770978	1	4.79E-10	4.20E-10	-	-0.356921	-0.4282	+	T	C	0.107195123	0.0569754	+	T	C	0.0852	0.0686
rs10135194	LC_IGP21	IgG1 Fucosylation	14	65770978	1	5.71E-10	1.46E-10	-	-0.353389	-0.4391	+	T	C	0.108215916	0.0566678	+	T	C	0.0852	0.0685
rs10135194	LC_IGP11	IgG1_G0	14	65770978	1	3.40E-12	1.23E-10	+	0.394113	0.4383	+	T	C	0.107834717	0.0561958	+	T	C	0.0852	0.0681
rs10135194	LC_IGP_R1	IgG1_G0F/IgG1_G0	14	65770978	1	7.19E-12	1.71E-09	-	-0.388005	-0.4107	+	T	C	0.107834717	0.0561959	+	T	C	0.0852	0.0682
rs10135194	LC_IGP_R4	IgG1_G0FN/IgG1_G0N	14	65770978	1	3.48E-08	4.09E-12	-	-0.31433	-0.4753	+	T	C	0.107792671	0.0567189	+	T	C	0.0852	0.0686
rs10135194	LC_IGP52	IgG1_G0n	14	65770978	1	4.26E-12	6.46E-11	+	0.39545	0.4458	+	T	C	0.107195123	0.0566442	+	T	C	0.0852	0.0682
rs10135194	LC_IGP14	IgG1_G0N	14	65770978	1	1.57E-09	3.56E-10	+	0.343648	0.4242	+	T	C	0.107792671	0.0566001	+	T	C	0.0852	0.0676
rs10135194	LC_IGP55	IgG1_G0Nn	14	65770978	1	5.56E-09	2.73E-10	+	0.334388	0.4276	+	T	C	0.107195123	0.0570499	+	T	C	0.0852	0.0677
rs10135194	LC_IGP12	IgG1_G1	14	65770978	1	5.28E-10	1.64E-09	+	0.352482	0.4131	+	T	C	0.108140154	0.0564136	+	T	C	0.0852	0.0685
rs10135194	LC_IGP_R2	IgG1_G1F/IgG1_G1	14	65770978	1	2.57E-10	2.94E-09	-	-0.358928	-0.4057	+	T	C	0.108140154	0.0564124	+	T	C	0.0852	0.0684
rs10135194	LC_IGP53	IgG1_G1n	14	65770978	1	1.04E-08	1.84E-09	+	0.328391	0.4118	+	T	C	0.107195123	0.0570771	+	T	C	0.0852	0.0685
rs10135194	LC_IGP_R3	IgG1_G2F/IgG1_G2	14	65770978	1	2.20E-09	4.57E-10	-	-0.340622	-0.4242	+	T	C	0.107595006	0.056615	+	T	C	0.0852	0.0681
rs1760978	LC_IGP78	IgG1 BG0n/G0n	14	65771047	1	2.66E-11	1.48E-12	+	0.237287	0.2748	+	G	T	0.401438042	0.0353561	+	G	T	0.3855	0.0388
rs1760978	LC_IGP79	IgG1 BG1n/G1n	14	65771047	1	4.79E-11	3.63E-13	+	0.234787	0.2823	+	G	T	0.401438042	0.0354509	+	G	T	0.3855	0.0388
rs1760978	LC_IGP77	IgG1 Bn	14	65771047	1	2.72E-11	3.64E-13	+	0.237783	0.2836	+	G	T	0.401438042	0.0354458	+	G	T	0.3855	0.039
rs1760978	LC_IGP86	IgG1 Bn/Fn total	14	65771047	1	7.42E-12	1.13E-13	+	0.244351	0.2898	+	G	T	0.401438042	0.0354102	+	G	T	0.3855	0.039
rs1760978	LC_IGP84	IgG1 FBn/Bn total	14	65771047	1	7.86E-13	1.01E-18	-	-0.255903	-0.3467	+	G	T	0.401438042	0.0354336	+	G	T	0.3855	0.0392
rs1760978	LC_IGP62	IgG1 FG0n total/G0n	14	65771047	1	1.66E-12	8.66E-15	-	-0.250878	-0.303	+	G	T	0.401438042	0.0352544	+	G	T	0.3855	0.0391
rs1760978	LC_IGP63	IgG1 FG1n total/G1n	14	65771047	1	1.10E-13	4.50E-16	-	-0.264528	-0.3172	+	G	T	0.401438042	0.0352998	+	G	T	0.3855	0.039
rs1760978	LC_IGP64	IgG1 FG2n total/G2n	14	65771047	1	5.12E-11	7.19E-16	-	-0.234542	-0.3131	+	G	T	0.401438042	0.0354689	+	G	T	0.3855	0.0388
rs1760978	LC_IGP61	IgG1 Fn total	14	65771047	1	1.36E-13	5.68E-16	-	-0.263337	-0.3164	+	G	T	0.401438042	0.0352797	+	G	T	0.3855	0.0391
rs1760978	LC_IGP81	IgG1 Fn/Bn	14	65771047	1	2.85E-11	6.11E-12	-	-0.237395	-0.2674	+	G	T	0.401438042	0.0354274	+	G	T	0.3855	0.0389
rs1760978	LC_IGP21	IgG1 Fucosylation	14	65771047	1	2.90E-12	1.48E-15	-	-0.247646	-0.3116	+	G	T	0.402808878	0.035195	+	G	T	0.3855	0.039
rs1760978	LC_IGP28	IgG1 G1S1/(G1+G1S1)	14	65771047	1	4.26E-08	1.18E-10	-	-0.194141	-0.2413	+	G	T	0.402363914	0.0352615	+	G	T	0.3855	0.0374
rs1760978	LC_IGP11	IgG1_G0	14	65771047	1	1.64E-10	6.89E-16	+	0.225817	0.3137	+	G	T	0.402788638	0.0351022	+	G	T	0.3855	0.0389
rs1760978	LC_IGP_R1	IgG1_G0F/IgG1_G0	14	65771047	1	1.81E-11	6.66E-14	-	-0.237164	-0.2919	+	G	T	0.402788638	0.0350403	+	G	T	0.3855	0.039
rs1760978	LC_IGP_R4	IgG1_G0FN/IgG1_G0N	14	65771047	1	3.68E-12	6.35E-17	-	-0.246172	-0.3274	+	G	T	0.402550827	0.0351583	+	G	T	0.3855	0.0392
rs1760978	LC_IGP52	IgG1_G0n	14	65771047	1	1.34E-10	2.85E-16	+	0.228377	0.3185	+	G	T	0.401438042	0.035323	+	G	T	0.3855	0.0389
rs1760978	LC_IGP14	IgG1_G0N	14	65771047	1	9.91E-12	1.71E-13	+	0.241181	0.2857	+	G	T	0.402550827	0.0351704	+	G	T	0.3855	0.0388
rs1760978	LC_IGP55	IgG1_G0Nn	14	65771047	1	3.83E-11	9.93E-14	+	0.235607	0.2887	+	G	T	0.401438042	0.0353947	+	G	T	0.3855	0.0388
rs1760978	LC_IGP12	IgG1_G1	14	65771047	1	2.34E-12	2.34E-15	+	0.248074	0.3089	+	G	T	0.402483201	0.0351059	+	G	T	0.3855	0.039
rs1760978	LC_IGP_R2	IgG1_G1F/IgG1_G1	14	65771047	1	9.75E-13	4.62E-15	-	-0.252418	-0.3054	+	G	T	0.402483201	0.0351018	+	G	T	0.3855	0.039
rs1760978	LC_IGP_R5	IgG1_G1FN/IgG1_G1N	14	65771047	1	7.37E-13	3.91E-19	-	-0.255066	-0.3491	+	G	T	0.4015123	0.0352757	+	G	T	0.3855	0.0391
rs1760978	LC_IGP53	IgG1_G1n	14	65771047	1	1.81E-12	2.50E-15	+	0.251012	0.3084	+	G	T	0.401438042	0.0353325	+	G	T	0.3855	0.039
rs1760978	LC_IGP15	IgG1_G1N	14	65771047	1	7.52E-11	4.21E-12	+	0.231707	0.2693	+	G	T	0.4015123	0.0353559	+	G	T	0.3855	0.0389
rs1760978	LC_IGP_SC15	IgG1_G1N(20)/IgG2_G1N(20)	14	65771047	1	3.40E-09	4.26E-07	+	0.20972	0.1881	+	G	T	0.401698892	0.0352822	+	G	T	0.3855	0.0372
rs1760978	LC_IGP56	IgG1_G1Nn	14	65771047	1	1.19E-10	6.08E-12	+	0.229995	0.2669	+	G	T	0.401438042	0.035477	+	G	T	0.3855	0.0388
rs1760978	LC_IGP13	IgG1_G2	14	65771047	1	1.08E-09	2.10E-11	+	0.216128	0.2574	+	G	T	0.402788638	0.0352437	+	G	T	0.3855	0.0384
rs1760978	LC_IGP_R3	IgG1_G2F/IgG1_G2	14	65771047	1	1.56E-11	9.78E-16	-	-0.238648	-0.3111	+	G	T	0.402729218	0.0351434	+	G	T	0.3855	0.0388
rs1760978	LC_IGP54	IgG1_G2n	14	65771047	1	5.33E-09	4.52E-11	+	0.20863	0.2523	+	G	T	0.401438042	0.0355507	+	G	T	0.3855	0.0383
rs10131385	LC_IGP78	IgG1 BG0n/G0n	14	65773555	1	3.88E-08	1.92E-09	+	0.312012	0.3987	+	C	T	0.108717512	0.0564947	+	C	T	0.0887	0.0664
rs10131385	LC_IGP62	IgG1 FG0n total/G0n	14	65773555	1	2.80E-11	4.76E-10	-	-0.376631	-0.4158	+	C	T	0.108717512	0.0561808	+	C	T	0.0887	0.0668
rs10131385	LC_IGP63	IgG1 FG1n total/G1n	14	65773555	1	3.79E-09	5.11E-10	-	-0.334904	-0.4146	+	C	T	0.108717512	0.0565143	+	C	T	0.0887	0.0667
rs10131385	LC_IGP64	IgG1 FG2n total/G2n	14	65773555	1	2.14E-08	2.77E-10	-	-0.318754	-0.4185	+	C	T	0.108717512	0.0566321	+	C	T	0.0887	0.0663
rs10131385	LC_IGP61	IgG1 Fn total	14	65773555	1	6.11E-10	3.22E-10	-	-0.351189	-0.4199	+	C	T	0.108717512	0.0564123	+	C	T	0.0887	0.0668
rs10131385	LC_IGP21	IgG1 Fucosylation	14	65773555	1	6.58E-10	1.07E-10	-	-0.347243	-0.4306	+	C	T	0.110030863	0.0558868	+	C	T	0.0887	0.0667
rs10131385	LC_IGP11	IgG1_G0	14	65773555	1	3.93E-12	1.13E-10	+	0.387549	0.4276	+	C	T	0.109633513	0.0554248	+	C	T	0.0887	0.0663
rs10131385	LC_IGP_R1	IgG1_G0F/IgG1_G0	14	65773555	1	6.85E-12	1.66E-09	-	-0.38302	-0.4013	+	C	T	0.109633513	0.0554185	+	C	T	0.0887	0.0666
rs10131385	LC_IGP_R4	IgG1_G0FN/IgG1_G0N	14	65773555	1	1.77E-08	4.08E-12	-	-0.316568	-0.465	+	C	T	0.109595874	0.0559113	+	C	T	0.0887	0.0671
rs10131385	LC_IGP52	IgG1_G0n	14	65773555	1	4.90E-12	6.00E-11	+	0.39038	0.4349	+	C	T	0.108717512	0.0560808	+	C	T	0.0887	0.0664
rs10131385	LC_IGP14	IgG1_G0N	14	65773555	1	5.55E-10	2.92E-10	+	0.348106	0.417	+	C	T	0.109595874	0.055782	+	C	T	0.0887	0.0662
rs10131385	LC_IGP55	IgG1_G0Nn	14	65773555	1	3.85E-09	2.24E-10	+	0.334469	0.4202	+	C	T	0.108717512	0.0564652	+	C	T	0.0887	0.0662
rs10131385	LC_IGP12	IgG1_G1	14	65773555	1	4.45E-10	1.31E-09	+	0.349125	0.4046	+	C	T	0.109938949	0.0556311	+	C	T	0.0887	0.0667
rs10131385	LC_IGP_R2	IgG1_G1F/IgG1_G1	14	65773555	1	2.24E-10	2.08E-09	-	-0.355149	-0.3987	+	C	T	0.109938949	0.0556311	+	C	T	0.0887	0.0666
rs10131385	LC_IGP53	IgG1_G1n	14	65773555	1	1.43E-08	1.47E-09	+	0.32205	0.4031	+	C	T	0.108717512	0.0565155	+	C	T	0.0887	0.0667
rs10131385	LC_IGP_R3	IgG1_G2F/IgG1_G2	14	65773555	1	3.39E-09	2.40E-10	-	-0.331959	-0.4191	+	C	T	0.109394904	0.0558458	+	C	T	0.0887	0.0662
rs10142617	LC_IGP78	IgG1 BG0n/G0n	14	65773671	1	1.32E-08	2.70E-16	+	0.260297	0.403	+	T	G	0.184315333	0.0455653	+	T	G	0.1697	0.0492
rs10142617	LC_IGP86	IgG1 Bn/Fn total	14	65773671	1	3.39E-08	1.10E-16	+	0.253482	0.4099	+	T	G	0.184315333	0.0456964	+	T	G	0.1697	0.0494
rs10142617	LC_IGP84	IgG1 FBn/Bn total	14	65773671	1	1.28E-08	2.11E-20	-	-0.261651	-0.4607	+	T	G	0.184315333	0.0457623	+	T	G	0.1697	0.0498
rs10142617	LC_IGP62	IgG1 FG0n total/G0n	14	65773671	1	2.21E-10	3.41E-17	-	-0.289946	-0.4176	+	T	G	0.184315333	0.0453985	+	T	G	0.1697	0.0495
rs10142617	LC_IGP63	IgG1 FG1n total/G1n	14	65773671	1	1.43E-09	4.04E-18	-	-0.277496	-0.4293	+	T	G	0.184315333	0.0455838	+	T	G	0.1697	0.0495
rs10142617	LC_IGP64	IgG1 FG2n total/G2n	14	65773671	1	1.85E-08	1.22E-16	-	-0.258407	-0.4074	+	T	G	0.184315333	0.0457018	+	T	G	0.1697	0.0492
rs10142617	LC_IGP61	IgG1 Fn total	14	65773671	1	3.42E-10	8.45E-18	-	-0.287549	-0.4256	+	T	G	0.184315333	0.0455124	+	T	G	0.1697	0.0495
rs10142617	LC_IGP21	IgG1 Fucosylation	14	65773671	1	4.50E-10	3.17E-18	-	-0.284014	-0.4305	+	T	G	0.185872344	0.0452663	+	T	G	0.1697	0.0495
rs10142617	LC_IGP11	IgG1_G0	14	65773671	1	7.78E-10	8.06E-18	+	0.278493	0.4234	+	T	G	0.185696361	0.0450193	+	T	G	0.1697	0.0492
rs10142617	LC_IGP_R1	IgG1_G0F/IgG1_G0	14	65773671	1	1.88E-10	1.63E-16	-	-0.288281	-0.4072	+	T	G	0.185696361	0.0449609	+	T	G	0.1697	0.0494
rs10142617	LC_IGP_R4	IgG1_G0FN/IgG1_G0N	14	65773671	1	9.15E-10	1.93E-19	-	-0.278347	-0.4484	+	T	G	0.185538851	0.0451871	+	T	G	0.1697	0.0497
rs10142617	LC_IGP52	IgG1_G0n	14	65773671	1	2.14E-09	2.10E-18	+	0.273528	0.4318	+	T	G	0.184315333	0.0454281	+	T	G	0.1697	0.0493
rs10142617	LC_IGP14	IgG1_G0N	14	65773671	1	6.63E-10	2.01E-17	+	0.280577	0.4168	+	T	G	0.185538851	0.0451671	+	T	G	0.1697	0.0491
rs10142617	LC_IGP55	IgG1_G0Nn	14	65773671	1	6.13E-09	6.81E-18	+	0.266427	0.4233	+	T	G	0.184315333	0.0455839	+	T	G	0.1697	0.0491
rs10142617	LC_IGP12	IgG1_G1	14	65773671	1	6.12E-10	1.28E-16	+	0.280681	0.4093	+	T	G	0.185696361	0.0450926	+	T	G	0.1697	0.0495
rs10142617	LC_IGP_R2	IgG1_G1F/IgG1_G1	14	65773671	1	3.33E-10	4.22E-17	-	-0.28507	-0.4149	+	T	G	0.185696361	0.0450947	+	T	G	0.1697	0.0494
rs10142617	LC_IGP53	IgG1_G1n	14	65773671	1	4.48E-09	1.01E-16	+	0.268822	0.4104	+	T	G	0.184315333	0.0455796	+	T	G	0.1697	0.0494
rs10142617	LC_IGP_SC15	IgG1_G1N(20)/IgG2_G1N(20)	14	65773671	1	3.22E-08	1.38E-09	+	0.252248	0.2858	+	T	G	0.184104032	0.0454017	+	T	G	0.1697	0.0472
rs10142617	LC_IGP_R3	IgG1_G2F/IgG1_G2	14	65773671	1	3.31E-09	1.35E-16	-	-0.268817	-0.406	+	T	G	0.185504244	0.0451921	+	T	G	0.1697	0.0491
rs10144975	LC_IGP78	IgG1 BG0n/G0n	14	65773982	1	1.32E-08	2.93E-16	+	0.260282	0.4012	+	T	C	0.184324659	0.0455626	+	T	C	0.1691	0.0491
rs10144975	LC_IGP86	IgG1 Bn/Fn total	14	65773982	1	3.39E-08	1.17E-16	+	0.253473	0.4082	+	T	C	0.184324659	0.0456936	+	T	C	0.1691	0.0493
rs10144975	LC_IGP84	IgG1 FBn/Bn total	14	65773982	1	1.28E-08	2.51E-20	-	-0.261651	-0.4585	+	T	C	0.184324659	0.0457596	+	T	C	0.1691	0.0496
rs10144975	LC_IGP62	IgG1 FG0n total/G0n	14	65773982	1	2.21E-10	3.68E-17	-	-0.289944	-0.4159	+	T	C	0.184324659	0.0453957	+	T	C	0.1691	0.0494
rs10144975	LC_IGP63	IgG1 FG1n total/G1n	14	65773982	1	1.42E-09	4.77E-18	-	-0.277507	-0.4272	+	T	C	0.184324659	0.045581	+	T	C	0.1691	0.0493
rs10144975	LC_IGP64	IgG1 FG2n total/G2n	14	65773982	1	1.84E-08	1.26E-16	-	-0.258408	-0.4062	+	T	C	0.184324659	0.045699	+	T	C	0.1691	0.0491
rs10144975	LC_IGP61	IgG1 Fn total	14	65773982	1	3.41E-10	9.52E-18	-	-0.287549	-0.4237	+	T	C	0.184324659	0.0455096	+	T	C	0.1691	0.0494
rs10144975	LC_IGP21	IgG1 Fucosylation	14	65773982	1	4.50E-10	3.17E-18	-	-0.283993	-0.4292	+	T	C	0.185881628	0.0452636	+	T	C	0.1691	0.0493
rs10144975	LC_IGP11	IgG1_G0	14	65773982	1	7.74E-10	8.16E-18	+	0.278508	0.4223	+	T	C	0.185705559	0.0450166	+	T	C	0.1691	0.0491
rs10144975	LC_IGP_R1	IgG1_G0F/IgG1_G0	14	65773982	1	1.87E-10	1.66E-16	-	-0.288278	-0.4058	+	T	C	0.185705559	0.0449583	+	T	C	0.1691	0.0492
rs10144975	LC_IGP_R4	IgG1_G0FN/IgG1_G0N	14	65773982	1	9.13E-10	2.31E-19	-	-0.278346	-0.446	+	T	C	0.185548071	0.0451844	+	T	C	0.1691	0.0496
rs10144975	LC_IGP52	IgG1_G0n	14	65773982	1	2.13E-09	1.95E-18	+	0.273542	0.4311	+	T	C	0.184324659	0.0454253	+	T	C	0.1691	0.0492
rs10144975	LC_IGP14	IgG1_G0N	14	65773982	1	6.61E-10	2.05E-17	+	0.280574	0.4154	+	T	C	0.185548071	0.0451644	+	T	C	0.1691	0.0489
rs10144975	LC_IGP55	IgG1_G0Nn	14	65773982	1	6.12E-09	6.89E-18	+	0.266425	0.422	+	T	C	0.184324659	0.0455811	+	T	C	0.1691	0.049
rs10144975	LC_IGP12	IgG1_G1	14	65773982	1	6.10E-10	1.66E-16	+	0.280696	0.4067	+	T	C	0.185705559	0.0450899	+	T	C	0.1691	0.0493
rs10144975	LC_IGP_R2	IgG1_G1F/IgG1_G1	14	65773982	1	3.32E-10	4.73E-17	-	-0.285081	-0.4132	+	T	C	0.185705559	0.0450919	+	T	C	0.1691	0.0492
rs10144975	LC_IGP53	IgG1_G1n	14	65773982	1	4.46E-09	1.30E-16	+	0.268834	0.4079	+	T	C	0.184324659	0.0455768	+	T	C	0.1691	0.0493
rs10144975	LC_IGP_SC15	IgG1_G1N(20)/IgG2_G1N(20)	14	65773982	1	3.20E-08	1.10E-09	+	0.252279	0.2868	+	T	C	0.1841133	0.0453988	+	T	C	0.1691	0.0471
rs10144975	LC_IGP_R3	IgG1_G2F/IgG1_G2	14	65773982	1	3.30E-09	1.41E-16	-	-0.268818	-0.4048	+	T	C	0.185513447	0.0451894	+	T	C	0.1691	0.049
rs17102587	LC_IGP78	IgG1 BG0n/G0n	14	65774477	1	1.33E-08	3.45E-16	+	0.260267	0.4024	+	C	T	0.184324072	0.0455632	+	C	T	0.1686	0.0493
rs17102587	LC_IGP86	IgG1 Bn/Fn total	14	65774477	1	3.39E-08	1.27E-16	+	0.253458	0.4099	+	C	T	0.184324072	0.0456943	+	C	T	0.1686	0.0495
rs17102587	LC_IGP84	IgG1 FBn/Bn total	14	65774477	1	1.29E-08	2.47E-20	-	-0.261636	-0.4611	+	C	T	0.184324072	0.0457602	+	C	T	0.1686	0.0499
rs17102587	LC_IGP62	IgG1 FG0n total/G0n	14	65774477	1	2.21E-10	4.39E-17	-	-0.289934	-0.4172	+	C	T	0.184324072	0.0453963	+	C	T	0.1686	0.0496
rs17102587	LC_IGP63	IgG1 FG1n total/G1n	14	65774477	1	1.43E-09	4.59E-18	-	-0.277503	-0.4297	+	C	T	0.184324072	0.0455816	+	C	T	0.1686	0.0496
rs17102587	LC_IGP64	IgG1 FG2n total/G2n	14	65774477	1	1.85E-08	9.99E-17	-	-0.258407	-0.4095	+	C	T	0.184324072	0.0456996	+	C	T	0.1686	0.0493
rs17102587	LC_IGP61	IgG1 Fn total	14	65774477	1	3.42E-10	9.71E-18	-	-0.287543	-0.4259	+	C	T	0.184324072	0.0455102	+	C	T	0.1686	0.0497
rs17102587	LC_IGP21	IgG1 Fucosylation	14	65774477	1	4.50E-10	3.46E-18	-	-0.28399	-0.4312	+	C	T	0.185881047	0.0452642	+	C	T	0.1686	0.0496

rs17102587	LC_IGP11	IgG1_G0	14	65774477	1	7.76E-10	9.58E-18	+	0.2785	0.4236	+	C	T	0.185704983	0.0450171	+	C	T	0.1686	0.0494
rs17102587	LC_IGP_R1	IgG1_G0F/IgG1_G0	14	65774477	1	1.88E-10	2.00E-16	-	-0.28827	-0.407	+	C	T	0.185704983	0.0449589	+	C	T	0.1686	0.0495
rs17102587	LC_IGP_R4	IgG1_G0FN/IgG1_G0N	14	65774477	1	9.15E-10	2.70E-19	-	-0.278332	-0.4477	+	C	T	0.185547493	0.045185	+	C	T	0.1686	0.0498
rs17102587	LC_IGP52	IgG1_G0n	14	65774477	1	2.14E-09	2.34E-18	+	0.273533	0.4324	+	C	T	0.184324072	0.0454259	+	C	T	0.1686	0.0495
rs17102587	LC_IGP14	IgG1_G0N	14	65774477	1	6.63E-10	2.38E-17	+	0.280561	0.4167	+	C	T	0.185547493	0.045165	+	C	T	0.1686	0.0492
rs17102587	LC_IGP55	IgG1_G0Nn	14	65774477	1	6.13E-09	8.09E-18	+	0.266411	0.4233	+	C	T	0.184324072	0.0455817	+	C	T	0.1686	0.0492
rs17102587	LC_IGP12	IgG1_G1	14	65774477	1	6.10E-10	1.61E-16	+	0.280692	0.4091	+	C	T	0.185704983	0.0450904	+	C	T	0.1686	0.0496
rs17102587	LC_IGP_R2	IgG1_G1F/IgG1_G1	14	65774477	1	3.32E-10	4.75E-17	-	-0.28508	-0.4153	+	C	T	0.185704983	0.0450925	+	C	T	0.1686	0.0495
rs17102587	LC_IGP53	IgG1_G1n	14	65774477	1	4.47E-09	1.28E-16	+	0.26883	0.4102	+	C	T	0.184324072	0.0455774	+	C	T	0.1686	0.0496
rs17102587	LC_IGP_SC15	IgG1_G1N(20)/IgG2_G1N(20)	14	65774477	1	3.21E-08	1.03E-09	+	0.252264	0.2887	+	C	T	0.184112717	0.0453994	+	C	T	0.1686	0.0473
rs17102587	LC_IGP_R3	IgG1_G2F/IgG1_G2	14	65774477	1	3.30E-09	1.08E-16	-	-0.268817	-0.4082	+	C	T	0.185512871	0.04519	+	C	T	0.1686	0.0492
rs8012054	LC_IGP78	IgG1 BG0n/G0n	14	65774762	1	1.33E-08	1.37E-15	+	0.260262	0.3929	+	C	A	0.184324268	0.0455634	+	C	A	0.17	0.0492
rs8012054	LC_IGP86	IgG1 Bn/Fn total	14	65774762	1	3.40E-08	3.95E-16	+	0.25345	0.4025	+	C	A	0.184324268	0.0456944	+	C	A	0.17	0.0494
rs8012054	LC_IGP84	IgG1 FBn/Bn total	14	65774762	1	1.29E-08	8.47E-20	-	-0.26163	-0.4542	+	C	A	0.184324268	0.0457604	+	C	A	0.17	0.0499
rs8012054	LC_IGP62	IgG1 FG0n total/G0n	14	65774762	1	2.21E-10	1.87E-16	-	-0.289931	-0.4075	+	C	A	0.184324268	0.0453965	+	C	A	0.17	0.0495
rs8012054	LC_IGP63	IgG1 FG1n total/G1n	14	65774762	1	1.43E-09	1.24E-17	-	-0.277501	-0.4231	+	C	A	0.184324268	0.0455817	+	C	A	0.17	0.0495
rs8012054	LC_IGP64	IgG1 FG2n total/G2n	14	65774762	1	1.85E-08	2.18E-16	-	-0.258405	-0.404	+	C	A	0.184324268	0.0456997	+	C	A	0.17	0.0492
rs8012054	LC_IGP61	IgG1 Fn total	14	65774762	1	3.42E-10	2.92E-17	-	-0.28754	-0.4186	+	C	A	0.184324268	0.0455104	+	C	A	0.17	0.0495
rs8012054	LC_IGP21	IgG1 Fucosylation	14	65774762	1	4.51E-10	1.17E-17	-	-0.283989	-0.4232	+	C	A	0.18588124	0.0452644	+	C	A	0.17	0.0495
rs8012054	LC_IGP11	IgG1_G0	14	65774762	1	7.76E-10	5.82E-17	+	0.278498	0.4122	+	C	A	0.185705175	0.0450173	+	C	A	0.17	0.0493
rs8012054	LC_IGP_R1	IgG1_G0F/IgG1_G0	14	65774762	1	1.88E-10	8.17E-16	-	-0.288268	-0.3974	+	C	A	0.185705175	0.044959	+	C	A	0.17	0.0494
rs8012054	LC_IGP_R4	IgG1_G0FN/IgG1_G0N	14	65774762	1	9.16E-10	1.29E-18	-	-0.278327	-0.4379	+	C	A	0.185547686	0.0451851	+	C	A	0.17	0.0497
rs8012054	LC_IGP52	IgG1_G0n	14	65774762	1	2.14E-09	1.46E-17	+	0.273532	0.421	+	C	A	0.184324268	0.045426	+	C	A	0.17	0.0494
rs8012054	LC_IGP14	IgG1_G0N	14	65774762	1	6.63E-10	1.37E-16	+	0.280557	0.4056	+	C	A	0.185547686	0.0451652	+	C	A	0.17	0.0491
rs8012054	LC_IGP55	IgG1_G0Nn	14	65774762	1	6.13E-09	4.76E-17	+	0.266407	0.4121	+	C	A	0.184324268	0.0455819	+	C	A	0.17	0.0491
rs8012054	LC_IGP12	IgG1_G1	14	65774762	1	6.11E-10	4.00E-16	+	0.28069	0.4026	+	C	A	0.185705175	0.0450906	+	C	A	0.17	0.0495
rs8012054	LC_IGP_R2	IgG1_G1F/IgG1_G1	14	65774762	1	3.32E-10	1.22E-16	-	-0.285079	-0.4087	+	C	A	0.185705175	0.0450926	+	C	A	0.17	0.0494
rs8012054	LC_IGP53	IgG1_G1n	14	65774762	1	4.47E-09	3.14E-16	+	0.268828	0.4039	+	C	A	0.184324268	0.0455776	+	C	A	0.17	0.0494
rs8012054	LC_IGP_SC15	IgG1_G1N(20)/IgG2_G1N(20)	14	65774762	1	3.21E-08	1.57E-09	+	0.252257	0.2856	+	C	A	0.184112911	0.0453996	+	C	A	0.17	0.0473
rs8012054	LC_IGP_R3	IgG1_G2F/IgG1_G2	14	65774762	1	3.30E-09	2.37E-16	-	-0.268816	-0.4027	+	C	A	0.185513063	0.0451901	+	C	A	0.17	0.0491
rs8013568	LC_IGP78	IgG1 BG0n/G0n	14	65774875	1	1.33E-08	3.86E-16	+	0.260256	0.4017	+	A	T	0.184324464	0.0455635	+	A	T	0.1685	0.0493
rs8013568	LC_IGP86	IgG1 Bn/Fn total	14	65774875	1	3.40E-08	1.31E-16	+	0.253443	0.4097	+	A	T	0.184324464	0.0456946	+	A	T	0.1685	0.0495
rs8013568	LC_IGP84	IgG1 FBn/Bn total	14	65774875	1	1.29E-08	2.39E-20	-	-0.261623	-0.4613	+	A	T	0.184324464	0.0457605	+	A	T	0.1685	0.0499
rs8013568	LC_IGP62	IgG1 FG0n total/G0n	14	65774875	1	2.21E-10	4.82E-17	-	-0.289928	-0.4166	+	A	T	0.184324464	0.0453966	+	A	T	0.1685	0.0496
rs8013568	LC_IGP63	IgG1 FG1n total/G1n	14	65774875	1	1.43E-09	4.37E-18	-	-0.277499	-0.43	+	A	T	0.184324464	0.0455819	+	A	T	0.1685	0.0496
rs8013568	LC_IGP64	IgG1 FG2n total/G2n	14	65774875	1	1.85E-08	8.67E-17	-	-0.258403	-0.4103	+	A	T	0.184324464	0.0456999	+	A	T	0.1685	0.0493
rs8013568	LC_IGP61	IgG1 Fn total	14	65774875	1	3.42E-10	9.58E-18	-	-0.287538	-0.426	+	A	T	0.184324464	0.0455105	+	A	T	0.1685	0.0497
rs8013568	LC_IGP21	IgG1 Fucosylation	14	65774875	1	4.51E-10	3.63E-18	-	-0.283987	-0.4309	+	A	T	0.185881434	0.0452645	+	A	T	0.1685	0.0496
rs8013568	LC_IGP11	IgG1_G0	14	65774875	1	7.76E-10	1.05E-17	+	0.278497	0.4231	+	A	T	0.185705367	0.0450174	+	A	T	0.1685	0.0494
rs8013568	LC_IGP_R1	IgG1_G0F/IgG1_G0	14	65774875	1	1.88E-10	2.18E-16	-	-0.288266	-0.4064	+	A	T	0.185705367	0.0449591	+	A	T	0.1685	0.0495
rs8013568	LC_IGP_R4	IgG1_G0FN/IgG1_G0N	14	65774875	1	9.17E-10	3.01E-19	-	-0.278322	-0.4471	+	A	T	0.185547878	0.0451853	+	A	T	0.1685	0.0498
rs8013568	LC_IGP52	IgG1_G0n	14	65774875	1	2.14E-09	2.60E-18	+	0.27353	0.4318	+	A	T	0.184324464	0.0454262	+	A	T	0.1685	0.0495
rs8013568	LC_IGP14	IgG1_G0N	14	65774875	1	6.64E-10	2.68E-17	+	0.280553	0.416	+	A	T	0.185547878	0.0451653	+	A	T	0.1685	0.0492
rs8013568	LC_IGP55	IgG1_G0Nn	14	65774875	1	6.14E-09	9.21E-18	+	0.266403	0.4225	+	A	T	0.184324464	0.045582	+	A	T	0.1685	0.0492
rs8013568	LC_IGP12	IgG1_G1	14	65774875	1	6.11E-10	1.54E-16	+	0.280688	0.4093	+	A	T	0.185705367	0.0450907	+	A	T	0.1685	0.0496
rs8013568	LC_IGP_R2	IgG1_G1F/IgG1_G1	14	65774875	1	3.32E-10	4.64E-17	-	-0.285078	-0.4155	+	A	T	0.185705367	0.0450928	+	A	T	0.1685	0.0495
rs8013568	LC_IGP53	IgG1_G1n	14	65774875	1	4.47E-09	1.24E-16	+	0.268825	0.4103	+	A	T	0.184324464	0.0455777	+	A	T	0.1685	0.0496
rs8013568	LC_IGP_SC15	IgG1_G1N(20)/IgG2_G1N(20)	14	65774875	1	3.22E-08	9.03E-10	+	0.25225	0.2899	+	A	T	0.184113106	0.0453998	+	A	T	0.1685	0.0473
rs8013568	LC_IGP_R3	IgG1_G2F/IgG1_G2	14	65774875	1	3.30E-09	9.32E-17	-	-0.268815	-0.4091	+	A	T	0.185513255	0.0451903	+	A	T	0.1685	0.0492
rs8017974	LC_IGP78	IgG1 BG0n/G0n	14	65775187	1	1.33E-08	4.25E-16	+	0.260251	0.401	+	C	T	0.184324659	0.0455637	+	C	T	0.1685	0.0493
rs8017974	LC_IGP86	IgG1 Bn/Fn total	14	65775187	1	3.40E-08	1.37E-16	+	0.253436	0.4093	+	C	T	0.184324659	0.0456947	+	C	T	0.1685	0.0495
rs8017974	LC_IGP84	IgG1 FBn/Bn total	14	65775187	1	1.29E-08	2.37E-20	-	-0.261617	-0.4612	+	C	T	0.184324659	0.0457607	+	C	T	0.1685	0.0499
rs8017974	LC_IGP62	IgG1 FG0n total/G0n	14	65775187	1	2.22E-10	5.34E-17	-	-0.289925	-0.4159	+	C	T	0.184324659	0.0453968	+	C	T	0.1685	0.0496
rs8017974	LC_IGP63	IgG1 FG1n total/G1n	14	65775187	1	1.43E-09	4.40E-18	-	-0.277498	-0.4298	+	C	T	0.184324659	0.045582	+	C	T	0.1685	0.0496
rs8017974	LC_IGP64	IgG1 FG2n total/G2n	14	65775187	1	1.85E-08	8.04E-17	-	-0.258401	-0.4107	+	C	T	0.184324659	0.0457	+	C	T	0.1685	0.0493
rs8017974	LC_IGP61	IgG1 Fn total	14	65775187	1	3.42E-10	9.81E-18	-	-0.287535	-0.4257	+	C	T	0.184324659	0.0455106	+	C	T	0.1685	0.0496
rs8017974	LC_IGP21	IgG1 Fucosylation	14	65775187	1	4.51E-10	3.86E-18	-	-0.283985	-0.4304	+	C	T	0.185881628	0.0452646	+	C	T	0.1685	0.0496
rs8017974	LC_IGP11	IgG1_G0	14	65775187	1	7.76E-10	1.19E-17	+	0.278496	0.4223	+	C	T	0.185705559	0.0450176	+	C	T	0.1685	0.0494
rs8017974	LC_IGP_R1	IgG1_G0F/IgG1_G0	14	65775187	1	1.88E-10	2.43E-16	-	-0.288264	-0.4057	+	C	T	0.185705559	0.0449593	+	C	T	0.1685	0.0495
rs8017974	LC_IGP_R4	IgG1_G0FN/IgG1_G0N	14	65775187	1	9.18E-10	3.26E-19	-	-0.278317	-0.4465	+	C	T	0.185548071	0.0451854	+	C	T	0.1685	0.0498
rs8017974	LC_IGP52	IgG1_G0n	14	65775187	1	2.14E-09	2.98E-18	+	0.273529	0.4309	+	C	T	0.184324659	0.0454263	+	C	T	0.1685	0.0495
rs8017974	LC_IGP14	IgG1_G0N	14	65775187	1	6.64E-10	2.99E-17	+	0.280549	0.4152	+	C	T	0.185548071	0.0451655	+	C	T	0.1685	0.0492
rs8017974	LC_IGP55	IgG1_G0Nn	14	65775187	1	6.14E-09	1.04E-17	+	0.266399	0.4217	+	C	T	0.184324659	0.0455822	+	C	T	0.1685	0.0492
rs8017974	LC_IGP12	IgG1_G1	14	65775187	1	6.11E-10	1.55E-16	+	0.280686	0.4092	+	C	T	0.185705559	0.0450908	+	C	T	0.1685	0.0496
rs8017974	LC_IGP_R2	IgG1_G1F/IgG1_G1	14	65775187	1	3.32E-10	4.75E-17	-	-0.285077	-0.4152	+	C	T	0.185705559	0.0450929	+	C	T	0.1685	0.0495
rs8017974	LC_IGP53	IgG1_G1n	14	65775187	1	4.47E-09	1.25E-16	+	0.268823	0.4101	+	C	T	0.184324659	0.0455778	+	C	T	0.1685	0.0495
rs8017974	LC_IGP_SC15	IgG1_G1N(20)/IgG2_G1N(20)	14	65775187	1	3.22E-08	8.59E-10	+	0.252243	0.2902	+	C	T	0.1841133	0.0453999	+	C	T	0.1685	0.0473
rs8017974	LC_IGP_R3	IgG1_G2F/IgG1_G2	14	65775187	1	3.30E-09	8.58E-17	-	-0.268813	-0.4095	+	C	T	0.185513447	0.0451904	+	C	T	0.1685	0.0492
rs11847263	LC_IGP78	IgG1 BG0n/G0n	14	65775695	1	1.33E-12	9.27E-16	+	0.259894	0.3209	+	G	T	0.356953532	0.0363604	+	G	T	0.3303	0.0399
rs11847263	LC_IGP79	IgG1 BG1n/G1n	14	65775695	1	1.20E-12	7.12E-17	+	0.261003	0.3328	+	G	T	0.356953532	0.0364426	+	G	T	0.3303	0.0399
rs11847263	LC_IGP77	IgG1 Bn	14	65775695	1	8.30E-13	1.61E-16	+	0.262906	0.3305	+	G	T	0.356953532	0.0364417	+	G	T	0.3303	0.0401
rs11847263	LC_IGP86	IgG1 Bn/Fn total	14	65775695	1	1.87E-13	3.25E-17	+	0.270126	0.3382	+	G	T	0.356953532	0.0364008	+	G	T	0.3303	0.0401
rs11847263	LC_IGP84	IgG1 FBn/Bn total	14	65775695	1	5.80E-15	4.19E-23	-	-0.286956	-0.3998	+	G	T	0.356953532	0.0363973	+	G	T	0.3303	0.0404
rs11847263	LC_IGP62	IgG1 FG0n total/G0n	14	65775695	1	1.35E-14	1.22E-18	-	-0.281519	-0.3541	+	G	T	0.356953532	0.0362155	+	G	T	0.3303	0.0402
rs11847263	LC_IGP63	IgG1 FG1n total/G1n	14	65775695	1	5.09E-15	8.79E-21	-	-0.286802	-0.3754	+	G	T	0.356953532	0.0363006	+	G	T	0.3303	0.0402
rs11847263	LC_IGP64	IgG1 FG2n total/G2n	14	65775695	1	9.68E-13	1.61E-20	-	-0.262212	-0.3708	+	G	T	0.356953532	0.0364548	+	G	T	0.3303	0.0399
rs11847263	LC_IGP68	IgG1 FG2n/G2n	14	65775695	1	1.29E-09	1.97E-12	-	-0.22343	-0.2798	+	G	T	0.356953532	0.0366026	+	G	T	0.3303	0.0398
rs11847263	LC_IGP65	IgG1 Fn	14	65775695	1	4.11E-08	1.86E-06	-	-0.201297	-0.1873	+	G	T	0.356953532	0.0365158	+	G	T	0.3303	0.0393
rs11847263	LC_IGP61	IgG1 Fn total	14	65775695	1	2.76E-15	3.11E-20	-	-0.28936	-0.3703	+	G	T	0.356953532	0.0362615	+	G	T	0.3303	0.0402
rs11847263	LC_IGP81	IgG1 Fn/Bn	14	65775695	1	1.02E-12	3.26E-15	-	-0.261735	-0.3144	+	G	T	0.356953532	0.0364263	+	G	T	0.3303	0.0399
rs11847263	LC_IGP21	IgG1 Fucosylation	14	65775695	1	1.53E-14	1.96E-20	-	-0.280045	-0.3718	+	G	T	0.358275586	0.0361043	+	G	T	0.3303	0.0401
rs11847263	LC_IGP11	IgG1_G0	14	65775695	1	3.35E-14	5.40E-20	+	0.275174	0.366	+	G	T	0.358047037	0.0359587	+	G	T	0.3303	0.04
rs11847263	LC_IGP_R1	IgG1_G0F/IgG1_G0	14	65775695	1	6.44E-14	1.02E-17	-	-0.272007	-0.3434	+	G	T	0.358047037	0.0359568	+	G	T	0.3303	0.0401
rs11847263	LC_IGP_R4	IgG1_G0FN/IgG1_G0N	14	65775695	1	1.32E-14	1.45E-20	-	-0.280272	-0.375	+	G	T	0.358005511	0.0360468	+	G	T	0.3303	0.0403
rs11847263	LC_IGP52	IgG1_G0n	14	65775695	1	8.72E-14	2.71E-20	+	0.272635	0.3697	+	G	T	0.356953532	0.0362305	+	G	T	0.3303	0.04
rs11847263	LC_IGP14	IgG1_G0N	14	65775695	1	2.59E-14	7.96E-17	+	0.27712	0.3317	+	G	T	0.358005511	0.0360525	+	G	T	0.3303	0.0398
rs11847263	LC_IGP55	IgG1_G0Nn	14	65775695	1	1.55E-13	4.75E-17	+	0.270654	0.3345	+	G	T	0.356953532	0.0363444	+	G	T	0.3303	0.0398
rs11847263	LC_IGP12	IgG1_G1	14	65775695	1	1.67E-13	6.02E-20	+	0.268401	0.3671	+	G	T	0.3577416	0.0360951	+	G	T	0.3303	0.0402
rs11847263	LC_IGP_R2	IgG1_G1F/IgG1_G1	14	65775695	1	3.89E-14	1.61E-19	-	-0.275362	-0.362	+	G	T	0.3577416	0.0360784	+	G	T	0.3303	0.0401
rs11847263	LC_IGP_R5	IgG1_G1FN/IgG1_G1N	14	65775695	1	9.13E-15	2.36E-24	-	-0.283345	-0.4091	+	G	T	0.356779213	0.0362138	+	G	T	0.3303	0.0402
rs11847263	LC_IGP53	IgG1_G1n	14	65775695	1	5.71E-13	9.78E-20	+	0.264348	0.3647	+	G	T	0.356953532	0.0363768	+	G	T	0.3303	0.0401
rs11847263	LC_IGP15	IgG1_G1N	14	65775695	1	5.72E-12	9.20E-16	+	0.252104	0.3208	+	G	T	0.356779213	0.0363362	+	G	T	0.3303	0.0399
rs11847263	LC_IGP_SC15	IgG1_G1N(20)/IgG2_G1N(20)	14	65775695	1	2.73E-09	1.05E-09	+	0.21716	0.2337	+	G	T	0.356910714	0.0363107	+	G	T	0.3303	0.0383
rs11847263	LC_IGP56	IgG1_G1Nn	14	65775695	1	6.86E-12	2.46E-15	+	0.252207	0.3155	+	G	T	0.356953532	0.0364879	+	G	T	0.3303	0.0399
rs11847263	LC_IGP13	IgG1_G2	14	65775695	1	2.57E-09	6.58E-15	+	0.217486	0.3079	+	G	T	0.358047037	0.0363068	+	G	T	0.3303	0.0395
rs11847263	LC_IGP_R3	IgG1_G2F/IgG1_G2	14	65775695	1	1.42E-13	2.30E-20	-	-0.269101	-0.3686	+	G	T	0.357960269	0.0360841	+	G	T	0.3303	0.0399
rs11847263	LC_IGP_R8	IgG1_G2FS1/IgG1_G2S1	14	65775695	1	5.75E-09	9.79E-17	-	-0.212674	-0.3304	+	G	T	0.358047037	0.0363222	+	G	T	0.3303	0.0398
rs11847263	LC_IGP54	IgG1_G2n	14	65775695	1	2.70E-08	2.85E-14	+	0.204847	0.2997	+	G	T	0.356953532	0.036663	+	G	T	0.3303	0.0394
rs10132229	LC_IGP62	IgG1 FG0n total/G0n	14	65777560	1	3.57E-11	3.00E-10	-	-0.380266	-0.4182	+	G	A	0.106312999	0.057036	+	G	A	0.0882	0.0664
rs10132229	LC_IGP63	IgG1 FG1n total/G1n	14	65777560	1	1.67E-09	1.53E-10	-	-0.34761	-0.4247	+	G	A	0.106312999	0.0573375	+	G	A	0.0882	0.0663
rs10132229	LC_IGP64	IgG1 FG2n total/G2n	14	65777560	1	9.26E-09	5.16E-11	-	-0.331768	-0.4333	+	G	A	0.106312999	0.0574566	+	G	A	0.0882	0.066
rs10132229	LC_IGP61	IgG1 Fn total	14	65777560	1	3.83E-10	1.10E-10	-	-0.360666	-0.4285	+	G	A	0.106312999	0.0572463	+	G	A	0.0882	0.0664
rs10132229	LC_IGP21	IgG1 Fucosylation	14	65777560	1	2.95E-10	6.71E-11	-	-0.359428	-0.4328	+	G	A	0.10763821	0.0566821	+	G	A	0.0882	0.0663
rs10132229	LC_IGP11	IgG1_G0	14	65777560	1	4.89E-12	1.15E-10	+	0.391496	0.4257	+	G	A	0.107262784	0.0562444	+	G	A	0.0882	0.066
rs10132229	LC_IGP_R1	IgG1_G0F/IgG1_G0	14	65777560	1	7.87E-12	1.10E-09	-	-0.387529	-0.4034	+	G	A	0.107262784	0.0562352	+	G	A	0.0882	0.0662
rs10132229	LC_IGP_R4	IgG1_G0FN/IgG1_G0N	14	65777560	1	2.40E-08	2.29E-12	-	-0.318216	-0.4676	+	G	A	0.107219338	0.056743	+	G	A	0.0882	0.0666
rs10132229	LC_IGP52	IgG1_G0n	14	65777560	1	5.84E-12	6.20E-11	+	0.394847	0.4327	+	G	A	0.106312999	0.0569322	+	G	A	0.0882	0.0662
rs10132229	LC_IGP14	IgG1_G0N	14	65777560	1	1.06E-09	3.33E-10	+	0.347426	0.4133	+	G	A	0.107219338	0.0566234	+	G	A	0.0882	0.0658
rs10132229	LC_IGP55	IgG1_G0Nn	14	65777560	1	7.47E-09	2.60E-10	+	0.333201	0.4163	+	G	A	0.106312999	0.0573391	+	G	A	0.0882	0.0659
rs10132229	LC_IGP12	IgG1_G1	14	65777560	1	2.17E-10	3.47E-10	+	0.360492	0.4163	+	G	A	0.107568221	0.0564196	+	G	A	0.0882	0.0663
rs10132229	LC_IGP_R2	IgG1_G1F/IgG1_G1	14	65777560	1	8.89E-11	6.95E-10	-	-0.368245	-0.4082	+	G	A	0.107568221	0.0564127	+	G	A	0.0882	0.0662
rs10132229	LC_IGP53	IgG1_G1n	14	65777560	1	7.09E-09	3.99E-10	+	0.333732	0.4146	+	G	A	0.106312999	0.0573429	+	G	A	0.0882	0.0663
rs10132229	LC_IGP_R3	IgG1_G2F/IgG1_G2	14	65777560	1	1.46E-09	3.99E-11	-	-0.344585	-0.4349	+	G	A	0.107022726	0.0566379	+	G	A	0.0882	0.0659
rs4902385	LC_IGP78	IgG1 BG0n/G0n	14	65778179	1	1.75E-08	1.56E-15	-	-0.256912	-0.3942	+	G	A	0.815054442	0.0453575	+	G	A	0.8286	0.0494
rs4902385	LC_IGP86	IgG1 Bn/Fn total	14	65778179	1	4.64E-08	4.88E-16	-	-0.249774	-0.4026	+	G	A	0.815054442	0.0454889	+	G	A	0.8286	0.0496
rs4902385	LC_IGP84	IgG1 FBn/Bn total	14	65778179	1	1.85E-08	1.04E-19	+	0.257591	0.4535	+	G	A	0.815054442	0.0455559	+	G	A	0.8286	0.0499
rs4902385	LC_IGP62	IgG1 FG0n total/G0n	14	65778179	1	2.96E-10	2.56E-16	+	0.286564	0.4074	+	G	A	0.815054442	0.0451918	+	G	A	0.8286	0.0497
rs4902385	LC_IGP63	IgG1 FG1n total/G1n	14	65778179	1	2.15E-09	1.96E-17	+	0.273213	0.4217	+	G	A	0.815054442	0.0453794	+	G	A	0.8286	0.0496
rs4902385	LC_IGP64	IgG1 FG2n total/G2n	14	65778179	1	3.63E-08	2.96E-16	+	0.251851	0.4032	+	G	A	0.815054442	0.0455042	+	G	A	0.8286	0.0493

rs4902385	LC_IGP61	IgG1 Fn total	14	65778179	1	5.08E-10	4.42E-17	+	0.283404	0.4175	+	G	A	0.815054442	0.045308	+	G	A	0.8286	0.0497
rs4902385	LC_IGP21	IgG1 Fucosylation	14	65778179	1	6.25E-10	1.81E-17	+	0.280371	0.4221	+	G	A	0.813500538	0.0450641	+	G	A	0.8286	0.0496
rs4902385	LC_IGP11	IgG1_G0	14	65778179	1	1.40E-09	5.73E-17	-	-0.273013	-0.4143	+	G	A	0.813682268	0.0448264	+	G	A	0.8286	0.0495
rs4902385	LC_IGP_R1	IgG1_G0F/IgG1_G0	14	65778179	1	2.44E-10	1.18E-15	+	0.285141	0.397	+	G	A	0.813682268	0.0447596	+	G	A	0.8286	0.0496
rs4902385	LC_IGP_R4	IgG1_G0FN/IgG1_G0N	14	65778179	1	1.37E-09	1.48E-18	+	0.27415	0.4388	+	G	A	0.813838256	0.0449881	+	G	A	0.8286	0.0499
rs4902385	LC_IGP52	IgG1_G0n	14	65778179	1	3.77E-09	1.53E-17	-	-0.268068	-0.4227	+	G	A	0.815054442	0.0452287	+	G	A	0.8286	0.0496
rs4902385	LC_IGP14	IgG1_G0N	14	65778179	1	1.19E-09	1.08E-16	-	-0.275113	-0.4092	+	G	A	0.813838256	0.044973	+	G	A	0.8286	0.0493
rs4902385	LC_IGP55	IgG1_G0Nn	14	65778179	1	1.05E-08	3.90E-17	-	-0.26106	-0.4156	+	G	A	0.815054442	0.0453831	+	G	A	0.8286	0.0494
rs4902385	LC_IGP12	IgG1_G1	14	65778179	1	8.26E-10	6.28E-16	-	-0.277266	-0.401	+	G	A	0.813682268	0.0448917	+	G	A	0.8286	0.0496
rs4902385	LC_IGP_R2	IgG1_G1F/IgG1_G1	14	65778179	1	5.45E-10	2.14E-16	+	0.280321	0.4068	+	G	A	0.813682268	0.0448989	+	G	A	0.8286	0.0495
rs4902385	LC_IGP53	IgG1_G1n	14	65778179	1	5.64E-09	5.16E-16	-	-0.265819	-0.4019	+	G	A	0.815054442	0.0453704	+	G	A	0.8286	0.0496
rs4902385	LC_IGP_SC15	IgG1_G1N(20)/IgG2_G1N(20)	14	65778179	1	2.45E-08	1.33E-09	-	-0.253222	-0.2874	+	G	A	0.815269624	0.0451804	+	G	A	0.8286	0.0474
rs4902385	LC_IGP_R3	IgG1_G2F/IgG1_G2	14	65778179	1	6.67E-09	3.16E-16	+	0.262358	0.4019	+	G	A	0.813874005	0.0450013	+	G	A	0.8286	0.0492
rs4902386	LC_IGP78	IgG1 BG0n/G0n	14	65778290	1	1.75E-08	6.15E-16	-	-0.256895	-0.3984	+	C	A	0.815051344	0.0453571	+	C	A	0.8305	0.0493
rs4902386	LC_IGP86	IgG1 Bn/Fn total	14	65778290	1	4.65E-08	2.16E-16	-	-0.249757	-0.4061	+	C	A	0.815051344	0.0454885	+	C	A	0.8305	0.0494
rs4902386	LC_IGP84	IgG1 FBn/Bn total	14	65778290	1	1.85E-08	3.14E-20	+	0.257581	0.4589	+	C	A	0.815051344	0.0455555	+	C	A	0.8305	0.0498
rs4902386	LC_IGP62	IgG1 FG0n total/G0n	14	65778290	1	2.96E-10	9.24E-17	+	0.286555	0.4121	+	C	A	0.815051344	0.0451914	+	C	A	0.8305	0.0496
rs4902386	LC_IGP63	IgG1 FG1n total/G1n	14	65778290	1	2.15E-09	7.06E-18	+	0.273202	0.4265	+	C	A	0.815051344	0.045379	+	C	A	0.8305	0.0495
rs4902386	LC_IGP64	IgG1 FG2n total/G2n	14	65778290	1	3.63E-08	1.19E-16	+	0.25185	0.4077	+	C	A	0.815051344	0.0455038	+	C	A	0.8305	0.0492
rs4902386	LC_IGP61	IgG1 Fn total	14	65778290	1	5.09E-10	1.59E-17	+	0.283394	0.4223	+	C	A	0.815051344	0.0453077	+	C	A	0.8305	0.0496
rs4902386	LC_IGP21	IgG1 Fucosylation	14	65778290	1	6.26E-10	6.19E-18	+	0.28036	0.4272	+	C	A	0.813497456	0.0450637	+	C	A	0.8305	0.0495
rs4902386	LC_IGP11	IgG1_G0	14	65778290	1	1.40E-09	2.56E-17	-	-0.273012	-0.4173	+	C	A	0.813679214	0.044826	+	C	A	0.8305	0.0493
rs4902386	LC_IGP_R1	IgG1_G0F/IgG1_G0	14	65778290	1	2.45E-10	4.50E-16	+	0.285134	0.4015	+	C	A	0.813679214	0.0447593	+	C	A	0.8305	0.0494
rs4902386	LC_IGP_R4	IgG1_G0FN/IgG1_G0N	14	65778290	1	1.37E-09	4.52E-19	+	0.274144	0.444	+	C	A	0.813835194	0.0449877	+	C	A	0.8305	0.0498
rs4902386	LC_IGP52	IgG1_G0n	14	65778290	1	3.77E-09	6.55E-18	-	-0.268066	-0.4259	+	C	A	0.815051344	0.0452283	+	C	A	0.8305	0.0494
rs4902386	LC_IGP14	IgG1_G0N	14	65778290	1	1.19E-09	5.08E-17	-	-0.275102	-0.4117	+	C	A	0.813835194	0.0449726	+	C	A	0.8305	0.0491
rs4902386	LC_IGP55	IgG1_G0Nn	14	65778290	1	1.05E-08	1.77E-17	-	-0.261048	-0.4182	+	C	A	0.815051344	0.0453827	+	C	A	0.8305	0.0492
rs4902386	LC_IGP12	IgG1_G1	14	65778290	1	8.26E-10	2.28E-16	-	-0.277261	-0.4062	+	C	A	0.813679214	0.0448913	+	C	A	0.8305	0.0495
rs4902386	LC_IGP_R2	IgG1_G1F/IgG1_G1	14	65778290	1	5.45E-10	7.91E-17	+	0.280311	0.4117	+	C	A	0.813679214	0.0448985	+	C	A	0.8305	0.0494
rs4902386	LC_IGP53	IgG1_G1n	14	65778290	1	5.64E-09	1.84E-16	-	-0.265812	-0.4072	+	C	A	0.815051344	0.04537	+	C	A	0.8305	0.0495
rs4902386	LC_IGP_SC15	IgG1_G1N(20)/IgG2_G1N(20)	14	65778290	1	2.45E-08	1.00E-09	-	-0.253198	-0.2887	+	C	A	0.815266545	0.0451801	+	C	A	0.8305	0.0473
rs4902386	LC_IGP_R3	IgG1_G2F/IgG1_G2	14	65778290	1	6.66E-09	1.28E-16	+	0.262357	0.4065	+	C	A	0.813870949	0.0450009	+	C	A	0.8305	0.0491
rs10147958	LC_IGP78	IgG1 BG0n/G0n	14	65778833	1	3.92E-08	3.19E-09	+	0.311874	0.3917	+	C	T	0.108726416	0.0564867	+	C	T	0.0886	0.0662
rs10147958	LC_IGP62	IgG1 FG0n total/G0n	14	65778833	1	2.84E-11	8.00E-10	-	-0.376451	-0.409	+	C	T	0.108726416	0.0561731	+	C	T	0.0886	0.0666
rs10147958	LC_IGP63	IgG1 FG1n total/G1n	14	65778833	1	3.84E-09	4.98E-10	-	-0.334717	-0.4136	+	C	T	0.108726416	0.0565065	+	C	T	0.0886	0.0665
rs10147958	LC_IGP64	IgG1 FG2n total/G2n	14	65778833	1	2.18E-08	1.72E-10	-	-0.318546	-0.4222	+	C	T	0.108726416	0.0566244	+	C	T	0.0886	0.0661
rs10147958	LC_IGP61	IgG1 Fn total	14	65778833	1	6.21E-10	3.49E-10	-	-0.351001	-0.4177	+	C	T	0.108726416	0.0564046	+	C	T	0.0886	0.0666
rs10147958	LC_IGP21	IgG1 Fucosylation	14	65778833	1	6.70E-10	1.35E-10	-	-0.347039	-0.427	+	C	T	0.110039729	0.0558794	+	C	T	0.0886	0.0665
rs10147958	LC_IGP11	IgG1_G0	14	65778833	1	4.01E-12	2.40E-10	+	0.387336	0.4189	+	C	T	0.109642296	0.0554176	+	C	T	0.0886	0.0662
rs10147958	LC_IGP_R1	IgG1_G0F/IgG1_G0	14	65778833	1	6.97E-12	2.80E-09	-	-0.382837	-0.3944	+	C	T	0.109642296	0.0554111	+	C	T	0.0886	0.0664
rs10147958	LC_IGP_R4	IgG1_G0FN/IgG1_G0N	14	65778833	1	1.78E-08	6.31E-12	-	-0.316434	-0.4591	+	C	T	0.109604678	0.0559035	+	C	T	0.0886	0.0668
rs10147958	LC_IGP52	IgG1_G0n	14	65778833	1	5.00E-12	1.33E-10	+	0.390158	0.4258	+	C	T	0.108726416	0.0560733	+	C	T	0.0886	0.0663
rs10147958	LC_IGP14	IgG1_G0N	14	65778833	1	5.62E-10	5.82E-10	+	0.347941	0.4082	+	C	T	0.109604678	0.0557744	+	C	T	0.0886	0.0659
rs10147958	LC_IGP55	IgG1_G0Nn	14	65778833	1	3.89E-09	4.58E-10	+	0.334302	0.4112	+	C	T	0.108726416	0.0564573	+	C	T	0.0886	0.066
rs10147958	LC_IGP12	IgG1_G1	14	65778833	1	4.51E-10	1.27E-09	+	0.348958	0.4037	+	C	T	0.109947734	0.0556236	+	C	T	0.0886	0.0665
rs10147958	LC_IGP_R2	IgG1_G1F/IgG1_G1	14	65778833	1	2.28E-10	2.20E-09	-	-0.354951	-0.397	+	C	T	0.109947734	0.0556237	+	C	T	0.0886	0.0664
rs10147958	LC_IGP53	IgG1_G1n	14	65778833	1	1.45E-08	1.46E-09	+	0.321884	0.402	+	C	T	0.108726416	0.0565076	+	C	T	0.0886	0.0664
rs10147958	LC_IGP_R3	IgG1_G2F/IgG1_G2	14	65778833	1	3.45E-09	1.40E-10	-	-0.331758	-0.4234	+	C	T	0.10940369	0.0558384	+	C	T	0.0886	0.066
rs8019473	LC_IGP78	IgG1 BG0n/G0n	14	65779128	1	1.35E-08	6.80E-16	+	0.260156	0.4009	+	A	G	0.184328148	0.0455665	+	A	G	0.167	0.0496
rs8019473	LC_IGP86	IgG1 Bn/Fn total	14	65779128	1	3.46E-08	2.14E-16	+	0.25331	0.4096	+	A	G	0.184328148	0.0456977	+	A	G	0.167	0.0499
rs8019473	LC_IGP84	IgG1 FBn/Bn total	14	65779128	1	1.31E-08	4.21E-20	-	-0.261505	-0.461	+	A	G	0.184328148	0.0457636	+	A	G	0.167	0.0502
rs8019473	LC_IGP62	IgG1 FG0n total/G0n	14	65779128	1	2.24E-10	1.36E-16	-	-0.289869	-0.4127	+	A	G	0.184328148	0.0453995	+	A	G	0.167	0.0499
rs8019473	LC_IGP63	IgG1 FG1n total/G1n	14	65779128	1	1.44E-09	1.02E-17	-	-0.27746	-0.4281	+	A	G	0.184328148	0.0455847	+	A	G	0.167	0.05
rs8019473	LC_IGP64	IgG1 FG2n total/G2n	14	65779128	1	1.86E-08	1.34E-16	-	-0.258359	-0.4111	+	A	G	0.184328148	0.0457027	+	A	G	0.167	0.0497
rs8019473	LC_IGP61	IgG1 Fn total	14	65779128	1	3.45E-10	2.22E-17	-	-0.287484	-0.4239	+	A	G	0.184328148	0.0455134	+	A	G	0.167	0.05
rs8019473	LC_IGP21	IgG1 Fucosylation	14	65779128	1	4.54E-10	8.68E-18	-	-0.283949	-0.4287	+	A	G	0.185885098	0.0452673	+	A	G	0.167	0.0499
rs8019473	LC_IGP11	IgG1_G0	14	65779128	1	7.81E-10	3.65E-17	+	0.278471	0.4181	+	A	G	0.185708997	0.0450201	+	A	G	0.167	0.0496
rs8019473	LC_IGP_R1	IgG1_G0F/IgG1_G0	14	65779128	1	1.89E-10	6.46E-16	-	-0.288227	-0.4021	+	A	G	0.185708997	0.0449619	+	A	G	0.167	0.0498
rs8019473	LC_IGP_R4	IgG1_G0FN/IgG1_G0N	14	65779128	1	9.30E-10	6.51E-19	-	-0.278235	-0.445	+	A	G	0.185551518	0.0451882	+	A	G	0.167	0.0501
rs8019473	LC_IGP52	IgG1_G0n	14	65779128	1	2.15E-09	9.70E-18	+	0.273502	0.4266	+	A	G	0.184328148	0.0454289	+	A	G	0.167	0.0497
rs8019473	LC_IGP14	IgG1_G0N	14	65779128	1	6.72E-10	4.90E-17	+	0.280481	0.4152	+	A	G	0.185551518	0.0451682	+	A	G	0.167	0.0495
rs8019473	LC_IGP55	IgG1_G0Nn	14	65779128	1	6.21E-09	1.80E-17	+	0.266326	0.4215	+	A	G	0.184328148	0.0455849	+	A	G	0.167	0.0495
rs8019473	LC_IGP12	IgG1_G1	14	65779128	1	6.16E-10	3.58E-16	+	0.280646	0.4069	+	A	G	0.185708997	0.0450935	+	A	G	0.167	0.0499
rs8019473	LC_IGP_R2	IgG1_G1F/IgG1_G1	14	65779128	1	3.34E-10	1.18E-16	-	-0.285055	-0.4129	+	A	G	0.185708997	0.0450954	+	A	G	0.167	0.0498
rs8019473	LC_IGP53	IgG1_G1n	14	65779128	1	4.51E-09	2.98E-16	+	0.268778	0.4077	+	A	G	0.184328148	0.0455805	+	A	G	0.167	0.0499
rs8019473	LC_IGP_SC15	IgG1_G1N(20)/IgG2_G1N(20)	14	65779128	1	3.28E-08	9.07E-10	+	0.252118	0.2918	+	A	G	0.184116768	0.0454028	+	A	G	0.167	0.0476
rs8019473	LC_IGP_R3	IgG1_G2F/IgG1_G2	14	65779128	1	3.32E-09	1.45E-16	-	-0.268782	-0.4099	+	A	G	0.185516888	0.045193	+	A	G	0.167	0.0496
rs10138662	LC_IGP78	IgG1 BG0n/G0n	14	65779482	1	1.35E-08	5.01E-16	+	0.260145	0.3995	+	G	A	0.184328539	0.0455668	+	G	A	0.1689	0.0493
rs10138662	LC_IGP86	IgG1 Bn/Fn total	14	65779482	1	3.47E-08	1.62E-16	+	0.253295	0.4077	+	G	A	0.184328539	0.045698	+	G	A	0.1689	0.0494
rs10138662	LC_IGP84	IgG1 FBn/Bn total	14	65779482	1	1.31E-08	2.32E-20	-	-0.261492	-0.4605	+	G	A	0.184328539	0.0457639	+	G	A	0.1689	0.0498
rs10138662	LC_IGP62	IgG1 FG0n total/G0n	14	65779482	1	2.24E-10	6.81E-17	-	-0.289862	-0.4139	+	G	A	0.184328539	0.0453998	+	G	A	0.1689	0.0496
rs10138662	LC_IGP63	IgG1 FG1n total/G1n	14	65779482	1	1.44E-09	5.01E-18	-	-0.277457	-0.4285	+	G	A	0.184328539	0.0455849	+	G	A	0.1689	0.0495
rs10138662	LC_IGP64	IgG1 FG2n total/G2n	14	65779482	1	1.86E-08	8.35E-17	-	-0.258354	-0.4099	+	G	A	0.184328539	0.0457029	+	G	A	0.1689	0.0492
rs10138662	LC_IGP61	IgG1 Fn total	14	65779482	1	3.46E-10	1.14E-17	-	-0.287479	-0.4242	+	G	A	0.184328539	0.0455136	+	G	A	0.1689	0.0496
rs10138662	LC_IGP21	IgG1 Fucosylation	14	65779482	1	4.55E-10	4.51E-18	-	-0.283945	-0.429	+	G	A	0.185885485	0.0452675	+	G	A	0.1689	0.0495
rs10138662	LC_IGP11	IgG1_G0	14	65779482	1	7.81E-10	1.71E-17	+	0.278469	0.4196	+	G	A	0.185709381	0.0450204	+	G	A	0.1689	0.0493
rs10138662	LC_IGP_R1	IgG1_G0F/IgG1_G0	14	65779482	1	1.89E-10	3.28E-16	-	-0.288223	-0.4033	+	G	A	0.185709381	0.0449621	+	G	A	0.1689	0.0494
rs10138662	LC_IGP_R4	IgG1_G0FN/IgG1_G0N	14	65779482	1	9.32E-10	3.53E-19	-	-0.278225	-0.4453	+	G	A	0.185551903	0.0451885	+	G	A	0.1689	0.0498
rs10138662	LC_IGP52	IgG1_G0n	14	65779482	1	2.15E-09	4.42E-18	+	0.273499	0.4281	+	G	A	0.184328539	0.0454292	+	G	A	0.1689	0.0494
rs10138662	LC_IGP14	IgG1_G0N	14	65779482	1	6.73E-10	3.82E-17	+	0.280473	0.4133	+	G	A	0.185551903	0.0451685	+	G	A	0.1689	0.0491
rs10138662	LC_IGP55	IgG1_G0Nn	14	65779482	1	6.22E-09	1.35E-17	+	0.266317	0.4197	+	G	A	0.184328539	0.0455852	+	G	A	0.1689	0.0492
rs10138662	LC_IGP12	IgG1_G1	14	65779482	1	6.16E-10	1.67E-16	+	0.280642	0.4081	+	G	A	0.185709381	0.0450937	+	G	A	0.1689	0.0495
rs10138662	LC_IGP_R2	IgG1_G1F/IgG1_G1	14	65779482	1	3.34E-10	5.65E-17	-	-0.285053	-0.4137	+	G	A	0.185709381	0.0450957	+	G	A	0.1689	0.0494
rs10138662	LC_IGP53	IgG1_G1n	14	65779482	1	4.51E-09	1.37E-16	+	0.268773	0.409	+	G	A	0.184328539	0.0455808	+	G	A	0.1689	0.0495
rs10138662	LC_IGP_SC15	IgG1_G1N(20)/IgG2_G1N(20)	14	65779482	1	3.28E-08	8.93E-10	+	0.252104	0.2895	+	G	A	0.184117157	0.0454031	+	G	A	0.1689	0.0472
rs10138662	LC_IGP_R3	IgG1_G2F/IgG1_G2	14	65779482	1	3.33E-09	8.93E-17	-	-0.268779	-0.4088	+	G	A	0.185517273	0.0451932	+	G	A	0.1689	0.0492
rs10134589	LC_IGP78	IgG1 BG0n/G0n	14	65781234	1	8.15E-09	1.87E-16	+	0.265781	0.4089	+	T	A	0.181984708	0.0458553	+	T	A	0.1676	0.0497
rs10134589	LC_IGP86	IgG1 Bn/Fn total	14	65781234	1	4.14E-08	6.20E-17	+	0.253553	0.4171	+	T	A	0.181984708	0.0460062	+	T	A	0.1676	0.0499
rs10134589	LC_IGP84	IgG1 FBn/Bn total	14	65781234	1	1.89E-08	7.09E-21	-	-0.26034	-0.4709	+	T	A	0.181984708	0.0460778	+	T	A	0.1676	0.0502
rs10134589	LC_IGP62	IgG1 FG0n total/G0n	14	65781234	1	1.31E-10	2.45E-17	-	-0.29552	-0.4234	+	T	A	0.181984708	0.0456862	+	T	A	0.1676	0.05
rs10134589	LC_IGP63	IgG1 FG1n total/G1n	14	65781234	1	2.38E-09	1.84E-18	-	-0.275586	-0.438	+	T	A	0.181984708	0.0459014	+	T	A	0.1676	0.05
rs10134589	LC_IGP64	IgG1 FG2n total/G2n	14	65781234	1	2.99E-08	3.66E-17	-	-0.256306	-0.4186	+	T	A	0.181984708	0.0460194	+	T	A	0.1676	0.0497
rs10134589	LC_IGP61	IgG1 Fn total	14	65781234	1	4.91E-10	4.21E-18	-	-0.286885	-0.4337	+	T	A	0.181984708	0.0458255	+	T	A	0.1676	0.05
rs10134589	LC_IGP21	IgG1 Fucosylation	14	65781234	1	5.01E-10	2.02E-18	-	-0.285127	-0.4377	+	T	A	0.183538583	0.0455678	+	T	A	0.1676	0.05
rs10134589	LC_IGP11	IgG1_G0	14	65781234	1	2.59E-10	6.86E-18	+	0.288076	0.4285	+	T	A	0.183383985	0.0452846	+	T	A	0.1676	0.0497
rs10134589	LC_IGP_R1	IgG1_G0F/IgG1_G0	14	65781234	1	1.05E-10	1.23E-16	-	-0.294155	-0.4125	+	T	A	0.183383985	0.0452399	+	T	A	0.1676	0.0498
rs10134589	LC_IGP_R4	IgG1_G0FN/IgG1_G0N	14	65781234	1	5.88E-10	1.13E-19	-	-0.283348	-0.4554	+	T	A	0.183220811	0.0454721	+	T	A	0.1676	0.0502
rs10134589	LC_IGP52	IgG1_G0n	14	65781234	1	7.79E-10	1.74E-18	+	0.282733	0.4371	+	T	A	0.181984708	0.0457026	+	T	A	0.1676	0.0498
rs10134589	LC_IGP14	IgG1_G0N	14	65781234	1	2.06E-10	1.49E-17	+	0.290656	0.4224	+	T	A	0.183220811	0.0454322	+	T	A	0.1676	0.0495
rs10134589	LC_IGP55	IgG1_G0Nn	14	65781234	1	2.17E-09	5.17E-18	+	0.27603	0.4289	+	T	A	0.181984708	0.0458585	+	T	A	0.1676	0.0496
rs10134589	LC_IGP12	IgG1_G1	14	65781234	1	1.14E-09	6.42E-17	+	0.278054	0.4174	+	T	A	0.183383985	0.045405	+	T	A	0.1676	0.0499
rs10134589	LC_IGP_R2	IgG1_G1F/IgG1_G1	14	65781234	1	5.16E-10	2.24E-17	-	-0.283859	-0.4227	+	T	A	0.183383985	0.0454021	+	T	A	0.1676	0.0498
rs10134589	LC_IGP53	IgG1_G1n	14	65781234	1	9.19E-09	5.25E-17	+	0.265113	0.4182	+	T	A	0.181984708	0.045903	+	T	A	0.1676	0.0499
rs10134589	LC_IGP_SC15	IgG1_G1N(20)/IgG2_G1N(20)	14	65781234	1	3.31E-08	3.57E-10	+	0.253723	0.2982	+	T	A	0.181773147	0.0457064	+	T	A	0.1676	0.0475
rs10134589	LC_IGP_R3	IgG1_G2F/IgG1_G2	14	65781234	1	6.12E-09	3.90E-17	-	-0.265964	-0.4174	+	T	A	0.183190455	0.0455055	+	T	A	0.1676	0.0496
rs7151301	LC_IGP78	IgG1 BG0n/G0n	14	65781496	1	1.36E-08	5.02E-16	+	0.260094	0.3996	+	A	G	0.184330855	0.0455683	+	A	G	0.1689	0.0493
rs7151301	LC_IGP86	IgG1 Bn/Fn total	14	65781496	1	3.50E-08	1.62E-16	+	0.253228	0.4078	+	A	G	0.184330855	0.0456995	+	A	G	0.1689	0.0494
rs7151301	LC_IGP84	IgG1 FBn/Bn total	14	65781496	1	1.32E-08	2.33E-20	-	-0.261433	-0.4605	+	A	G	0.184330855	0.0457654	+	A	G	0.1689	0.0498
rs7151301	LC_IGP62	IgG1 FG0n total/G0n	14	65781496	1	2.25E-10	6.86E-17	-	-0.289833	-0.4139	+	A	G	0.184330855	0.0454012	+	A	G	0.1689	0.0496
rs7151301	LC_IGP63	IgG1 FG1n total/G1n	14	65781496	1	1.44E-09	5.04E-18	-	-0.277437	-0.4285	+	A	G	0.184330855	0.0455863	+	A	G	0.1689	0.0495
rs7151301	LC_IGP64	IgG1 FG2n total/G2n	14	65781496	1	1.87E-08	8.42E-17	-	-0.258332	-0.4099	+	A	G	0.184330855	0.0457043	+	A	G	0.1689	0.0492
rs7151301	LC_IGP61	IgG1 Fn total	14	65781496	1	3.47E-10	1.14E-17	-	-0.287452	-0.4242	+	A	G	0.184330855	0.0455151	+	A	G	0.1689	0.0496
rs7151301	LC_IGP21	IgG1 Fucosylation	14	65781496	1	4.56E-10	4.53E-18	-	-0.283926	-0.429	+	A	G	0.185887793	0.0452689	+	A	G	0.1689	0.0495
rs7151301	LC_IGP11	IgG1_G0	14	65781496	1	7.83E-10	1.72E-17	+	0.278456	0.4196	+	A	G	0.185711668	0.0450217	+	A	G	0.1689	0.0493
rs7151301	LC_IGP_R1	IgG1_G0F/IgG1_G0	14	65781496	1	1.90E-10	3.30E-16	-	-0.288203	-0.4034	+	A	G	0.185711668	0.0449635	+	A	G	0.1689	0.0494
rs7151301	LC_IGP_R4	IgG1_G0FN/IgG1_G0N	14	65781496	1	9.38E-10	3.56E-19	-	-0.278181	-0.4454	+	A	G	0.185554195	0.0451899	+	A	G	0.1689	0.0498
rs7151301	LC_IGP52	IgG1_G0n	14	65781496	1	2.16E-09	4.44E-18	+	0.273485	0.4281	+	A	G	0.184330855	0.0454305	+	A	G	0.1689	0.0494
rs7151301	LC_IGP14	IgG1_G0N	14	65781496	1	6.77E-10	3.81E-17	+	0.280436	0.4134	+	A	G	0.185554195	0.0451699	+	A	G	0.1689	0.0491
rs7151301	LC_IGP55	IgG1_G0Nn	14	65781496	1	6.26E-09	1.35E-17	+	0.266278	0.4198	+	A	G	0.184330855	0.0455867	+	A	G	0.1689	0.0492

rs7151301	LC_IGP12	IgG1_G1	14	65781496	1	6.19E-10	1.68E-16	+	0.280621	0.4081	+	A	G	0.185711668	0.0450951	+	A	G	0.1689	0.0495
rs7151301	LC_IGP_R2	IgG1_G1F/IgG1_G1	14	65781496	1	3.35E-10	5.68E-17	-	-0.28504	-0.4137	+	A	G	0.185711668	0.045097	+	A	G	0.1689	0.0494
rs7151301	LC_IGP53	IgG1_G1n	14	65781496	1	4.53E-09	1.39E-16	+	0.268749	0.409	+	A	G	0.184330855	0.0455822	+	A	G	0.1689	0.0495
rs7151301	LC_IGP_SC15	IgG1_G1N(20)/IgG2_G1N(20)	14	65781496	1	3.31E-08	8.90E-10	+	0.252038	0.2896	+	A	G	0.184119458	0.0454047	+	A	G	0.1689	0.0473
rs7151301	LC_IGP_R3	IgG1_G2F/IgG1_G2	14	65781496	1	3.34E-09	9.01E-17	-	-0.268762	-0.4088	+	A	G	0.18551956	0.0451946	+	A	G	0.1689	0.0492
rs7151212	LC_IGP78	IgG1 BG0n/G0n	14	65781622	1	1.36E-08	2.83E-15	+	0.260094	0.3876	+	G	C	0.184330659	0.0455684	+	G	C	0.1713	0.0491
rs7151212	LC_IGP86	IgG1 Bn/Fn total	14	65781622	1	3.50E-08	9.25E-16	+	0.253227	0.3957	+	G	C	0.184330659	0.0456996	+	G	C	0.1713	0.0492
rs7151212	LC_IGP84	IgG1 FBn/Bn total	14	65781622	1	1.32E-08	2.56E-19	-	-0.261432	-0.4458	+	G	C	0.184330659	0.0457655	+	G	C	0.1713	0.0496
rs7151212	LC_IGP62	IgG1 FG0n total/G0n	14	65781622	1	2.25E-10	4.92E-16	-	-0.289832	-0.4006	+	G	C	0.184330659	0.0454013	+	G	C	0.1713	0.0494
rs7151212	LC_IGP63	IgG1 FG1n total/G1n	14	65781622	1	1.44E-09	3.88E-17	-	-0.277435	-0.4151	+	G	C	0.184330659	0.0455864	+	G	C	0.1713	0.0493
rs7151212	LC_IGP64	IgG1 FG2n total/G2n	14	65781622	1	1.87E-08	5.02E-16	-	-0.25833	-0.3977	+	G	C	0.184330659	0.0457044	+	G	C	0.1713	0.049
rs7151212	LC_IGP61	IgG1 Fn total	14	65781622	1	3.47E-10	8.55E-17	-	-0.28745	-0.4109	+	G	C	0.184330659	0.0455151	+	G	C	0.1713	0.0494
rs7151212	LC_IGP21	IgG1 Fucosylation	14	65781622	1	4.56E-10	2.49E-17	-	-0.283924	-0.4175	+	G	C	0.185887599	0.045269	+	G	C	0.1713	0.0493
rs7151212	LC_IGP11	IgG1_G0	14	65781622	1	7.84E-10	1.21E-16	+	0.278455	0.4065	+	G	C	0.185711476	0.0450217	+	G	C	0.1713	0.0491
rs7151212	LC_IGP_R1	IgG1_G0F/IgG1_G0	14	65781622	1	1.90E-10	2.22E-15	-	-0.288202	-0.3903	+	G	C	0.185711476	0.0449635	+	G	C	0.1713	0.0492
rs7151212	LC_IGP_R4	IgG1_G0FN/IgG1_G0N	14	65781622	1	9.38E-10	3.15E-18	-	-0.278181	-0.4316	+	G	C	0.185554002	0.04519	+	G	C	0.1713	0.0496
rs7151212	LC_IGP52	IgG1_G0n	14	65781622	1	2.16E-09	3.39E-17	+	0.273483	0.4147	+	G	C	0.184330659	0.0454306	+	G	C	0.1713	0.0492
rs7151212	LC_IGP14	IgG1_G0N	14	65781622	1	6.77E-10	2.27E-16	+	0.280436	0.4014	+	G	C	0.185554002	0.0451699	+	G	C	0.1713	0.0489
rs7151212	LC_IGP55	IgG1_G0Nn	14	65781622	1	6.26E-09	8.41E-17	+	0.266278	0.4077	+	G	C	0.184330659	0.0455867	+	G	C	0.1713	0.049
rs7151212	LC_IGP12	IgG1_G1	14	65781622	1	6.19E-10	1.11E-15	+	0.280619	0.3951	+	G	C	0.185711476	0.0450951	+	G	C	0.1713	0.0493
rs7151212	LC_IGP_R2	IgG1_G1F/IgG1_G1	14	65781622	1	3.35E-10	3.77E-16	-	-0.285039	-0.4008	+	G	C	0.185711476	0.0450971	+	G	C	0.1713	0.0492
rs7151212	LC_IGP53	IgG1_G1n	14	65781622	1	4.53E-09	9.24E-16	+	0.268747	0.396	+	G	C	0.184330659	0.0455823	+	G	C	0.1713	0.0493
rs7151212	LC_IGP_SC15	IgG1_G1N(20)/IgG2_G1N(20)	14	65781622	1	3.32E-08	2.14E-09	+	0.252036	0.2818	+	G	C	0.184119264	0.0454047	+	G	C	0.1713	0.0471
rs7151212	LC_IGP_R3	IgG1_G2F/IgG1_G2	14	65781622	1	3.34E-09	5.34E-16	-	-0.26876	-0.3966	+	G	C	0.185519368	0.0451946	+	G	C	0.1713	0.0489
rs11158587	LC_IGP78	IgG1 BG0n/G0n	14	65782712	1	1.37E-08	5.01E-16	+	0.260066	0.3996	+	A	G	0.184331442	0.0455691	+	A	G	0.1689	0.0493
rs11158587	LC_IGP86	IgG1 Bn/Fn total	14	65782712	1	3.52E-08	1.62E-16	+	0.25319	0.4078	+	A	G	0.184331442	0.0457004	+	A	G	0.1689	0.0494
rs11158587	LC_IGP84	IgG1 FBn/Bn total	14	65782712	1	1.33E-08	2.33E-20	-	-0.261398	-0.4605	+	A	G	0.184331442	0.0457663	+	A	G	0.1689	0.0498
rs11158587	LC_IGP62	IgG1 FG0n total/G0n	14	65782712	1	2.26E-10	6.84E-17	-	-0.289815	-0.4139	+	A	G	0.184331442	0.045402	+	A	G	0.1689	0.0496
rs11158587	LC_IGP63	IgG1 FG1n total/G1n	14	65782712	1	1.45E-09	5.03E-18	-	-0.277424	-0.4285	+	A	G	0.184331442	0.0455871	+	A	G	0.1689	0.0495
rs11158587	LC_IGP64	IgG1 FG2n total/G2n	14	65782712	1	1.87E-08	8.41E-17	-	-0.258318	-0.41	+	A	G	0.184331442	0.0457051	+	A	G	0.1689	0.0492
rs11158587	LC_IGP61	IgG1 Fn total	14	65782712	1	3.48E-10	1.14E-17	-	-0.287435	-0.4243	+	A	G	0.184331442	0.0455159	+	A	G	0.1689	0.0496
rs11158587	LC_IGP21	IgG1 Fucosylation	14	65782712	1	4.57E-10	4.52E-18	-	-0.283913	-0.4291	+	A	G	0.185888374	0.0452697	+	A	G	0.1689	0.0495
rs11158587	LC_IGP11	IgG1_G0	14	65782712	1	7.85E-10	1.71E-17	+	0.278447	0.4196	+	A	G	0.185712244	0.0450224	+	A	G	0.1689	0.0493
rs11158587	LC_IGP_R1	IgG1_G0F/IgG1_G0	14	65782712	1	1.91E-10	3.29E-16	-	-0.288191	-0.4034	+	A	G	0.185712244	0.0449642	+	A	G	0.1689	0.0494
rs11158587	LC_IGP_R4	IgG1_G0FN/IgG1_G0N	14	65782712	1	9.42E-10	3.55E-19	-	-0.278156	-0.4454	+	A	G	0.185554772	0.0451908	+	A	G	0.1689	0.0498
rs11158587	LC_IGP52	IgG1_G0n	14	65782712	1	2.16E-09	4.43E-18	+	0.273475	0.4282	+	A	G	0.184331442	0.0454313	+	A	G	0.1689	0.0494
rs11158587	LC_IGP14	IgG1_G0N	14	65782712	1	6.80E-10	3.80E-17	+	0.280415	0.4134	+	A	G	0.185554772	0.0451707	+	A	G	0.1689	0.0491
rs11158587	LC_IGP55	IgG1_G0Nn	14	65782712	1	6.28E-09	1.34E-17	+	0.266257	0.4198	+	A	G	0.184331442	0.0455875	+	A	G	0.1689	0.0492
rs11158587	LC_IGP12	IgG1_G1	14	65782712	1	6.20E-10	1.68E-16	+	0.280607	0.4081	+	A	G	0.185712244	0.0450958	+	A	G	0.1689	0.0495
rs11158587	LC_IGP_R2	IgG1_G1F/IgG1_G1	14	65782712	1	3.36E-10	5.67E-17	-	-0.285032	-0.4137	+	A	G	0.185712244	0.0450978	+	A	G	0.1689	0.0494
rs11158587	LC_IGP53	IgG1_G1n	14	65782712	1	4.54E-09	1.38E-16	+	0.268733	0.409	+	A	G	0.184331442	0.045583	+	A	G	0.1689	0.0495
rs11158587	LC_IGP_SC15	IgG1_G1N(20)/IgG2_G1N(20)	14	65782712	1	3.33E-08	8.89E-10	+	0.251998	0.2896	+	A	G	0.184120042	0.0454055	+	A	G	0.1689	0.0473
rs11158587	LC_IGP_R3	IgG1_G2F/IgG1_G2	14	65782712	1	3.34E-09	9.00E-17	-	-0.268751	-0.4088	+	A	G	0.185520137	0.0451953	+	A	G	0.1689	0.0492
rs8019767	LC_IGP78	IgG1 BG0n/G0n	14	65782785	1	1.37E-08	5.01E-16	+	0.26006	0.3996	+	T	G	0.184331833	0.0455692	+	T	G	0.1689	0.0493
rs8019767	LC_IGP86	IgG1 Bn/Fn total	14	65782785	1	3.53E-08	1.62E-16	+	0.253183	0.4078	+	T	G	0.184331833	0.0457005	+	T	G	0.1689	0.0494
rs8019767	LC_IGP84	IgG1 FBn/Bn total	14	65782785	1	1.33E-08	2.33E-20	-	-0.261393	-0.4605	+	T	G	0.184331833	0.0457664	+	T	G	0.1689	0.0498
rs8019767	LC_IGP62	IgG1 FG0n total/G0n	14	65782785	1	2.26E-10	6.84E-17	-	-0.289813	-0.4139	+	T	G	0.184331833	0.0454021	+	T	G	0.1689	0.0496
rs8019767	LC_IGP63	IgG1 FG1n total/G1n	14	65782785	1	1.45E-09	5.03E-18	-	-0.277424	-0.4285	+	T	G	0.184331833	0.0455872	+	T	G	0.1689	0.0495
rs8019767	LC_IGP64	IgG1 FG2n total/G2n	14	65782785	1	1.87E-08	8.41E-17	-	-0.258317	-0.41	+	T	G	0.184331833	0.0457052	+	T	G	0.1689	0.0492
rs8019767	LC_IGP61	IgG1 Fn total	14	65782785	1	3.49E-10	1.14E-17	-	-0.287433	-0.4243	+	T	G	0.184331833	0.0455159	+	T	G	0.1689	0.0496
rs8019767	LC_IGP21	IgG1 Fucosylation	14	65782785	1	4.57E-10	4.52E-18	-	-0.283913	-0.4291	+	T	G	0.185888761	0.0452697	+	T	G	0.1689	0.0495
rs8019767	LC_IGP11	IgG1_G0	14	65782785	1	7.85E-10	1.71E-17	+	0.278447	0.4196	+	T	G	0.185712628	0.0450225	+	T	G	0.1689	0.0493
rs8019767	LC_IGP_R1	IgG1_G0F/IgG1_G0	14	65782785	1	1.91E-10	3.29E-16	-	-0.28819	-0.4034	+	T	G	0.185712628	0.0449643	+	T	G	0.1689	0.0494
rs8019767	LC_IGP_R4	IgG1_G0FN/IgG1_G0N	14	65782785	1	9.43E-10	3.54E-19	-	-0.278151	-0.4454	+	T	G	0.185555158	0.0451908	+	T	G	0.1689	0.0498
rs8019767	LC_IGP52	IgG1_G0n	14	65782785	1	2.16E-09	4.43E-18	+	0.273475	0.4282	+	T	G	0.184331833	0.0454314	+	T	G	0.1689	0.0494
rs8019767	LC_IGP14	IgG1_G0N	14	65782785	1	6.80E-10	3.80E-17	+	0.280411	0.4134	+	T	G	0.185555158	0.0451708	+	T	G	0.1689	0.0491
rs8019767	LC_IGP55	IgG1_G0Nn	14	65782785	1	6.28E-09	1.34E-17	+	0.266252	0.4198	+	T	G	0.184331833	0.0455876	+	T	G	0.1689	0.0492
rs8019767	LC_IGP12	IgG1_G1	14	65782785	1	6.20E-10	1.68E-16	+	0.280607	0.4081	+	T	G	0.185712628	0.0450959	+	T	G	0.1689	0.0495
rs8019767	LC_IGP_R2	IgG1_G1F/IgG1_G1	14	65782785	1	3.36E-10	5.67E-17	-	-0.285033	-0.4137	+	T	G	0.185712628	0.0450978	+	T	G	0.1689	0.0494
rs8019767	LC_IGP53	IgG1_G1n	14	65782785	1	4.54E-09	1.38E-16	+	0.268733	0.409	+	T	G	0.184331833	0.045583	+	T	G	0.1689	0.0495
rs8019767	LC_IGP_SC15	IgG1_G1N(20)/IgG2_G1N(20)	14	65782785	1	3.33E-08	8.88E-10	+	0.251994	0.2896	+	T	G	0.184120431	0.0454056	+	T	G	0.1689	0.0473
rs8019767	LC_IGP_R3	IgG1_G2F/IgG1_G2	14	65782785	1	3.34E-09	8.99E-17	-	-0.268751	-0.4088	+	T	G	0.185520521	0.0451954	+	T	G	0.1689	0.0492
rs6573598	LC_IGP78	IgG1 BG0n/G0n	14	65783019	1	1.37E-08	5.01E-16	+	0.260054	0.3996	+	C	T	0.184332975	0.0455694	+	C	T	0.1689	0.0493
rs6573598	LC_IGP86	IgG1 Bn/Fn total	14	65783019	1	3.53E-08	1.62E-16	+	0.253175	0.4078	+	C	T	0.184332975	0.0457007	+	C	T	0.1689	0.0494
rs6573598	LC_IGP84	IgG1 FBn/Bn total	14	65783019	1	1.33E-08	2.33E-20	-	-0.261385	-0.4605	+	C	T	0.184332975	0.0457666	+	C	T	0.1689	0.0498
rs6573598	LC_IGP62	IgG1 FG0n total/G0n	14	65783019	1	2.26E-10	6.85E-17	-	-0.289808	-0.4139	+	C	T	0.184332975	0.0454023	+	C	T	0.1689	0.0496
rs6573598	LC_IGP63	IgG1 FG1n total/G1n	14	65783019	1	1.45E-09	5.03E-18	-	-0.27742	-0.4285	+	C	T	0.184332975	0.0455874	+	C	T	0.1689	0.0495
rs6573598	LC_IGP64	IgG1 FG2n total/G2n	14	65783019	1	1.88E-08	8.40E-17	-	-0.258313	-0.41	+	C	T	0.184332975	0.0457054	+	C	T	0.1689	0.0492
rs6573598	LC_IGP61	IgG1 Fn total	14	65783019	1	3.49E-10	1.14E-17	-	-0.287429	-0.4243	+	C	T	0.184332975	0.0455161	+	C	T	0.1689	0.0496
rs6573598	LC_IGP21	IgG1 Fucosylation	14	65783019	1	4.58E-10	4.52E-18	-	-0.283909	-0.429	+	C	T	0.185889907	0.0452699	+	C	T	0.1689	0.0495
rs6573598	LC_IGP11	IgG1_G0	14	65783019	1	7.85E-10	1.71E-17	+	0.278444	0.4196	+	C	T	0.185713762	0.0450227	+	C	T	0.1689	0.0493
rs6573598	LC_IGP_R1	IgG1_G0F/IgG1_G0	14	65783019	1	1.91E-10	3.29E-16	-	-0.288187	-0.4034	+	C	T	0.185713762	0.0449645	+	C	T	0.1689	0.0494
rs6573598	LC_IGP_R4	IgG1_G0FN/IgG1_G0N	14	65783019	1	9.44E-10	3.55E-19	-	-0.278146	-0.4454	+	C	T	0.185556294	0.045191	+	C	T	0.1689	0.0498
rs6573598	LC_IGP52	IgG1_G0n	14	65783019	1	2.16E-09	4.43E-18	+	0.273472	0.4282	+	C	T	0.184332975	0.0454316	+	C	T	0.1689	0.0494
rs6573598	LC_IGP14	IgG1_G0N	14	65783019	1	6.81E-10	3.80E-17	+	0.280407	0.4134	+	C	T	0.185556294	0.0451709	+	C	T	0.1689	0.0491
rs6573598	LC_IGP55	IgG1_G0Nn	14	65783019	1	6.29E-09	1.34E-17	+	0.266248	0.4198	+	C	T	0.184332975	0.0455878	+	C	T	0.1689	0.0492
rs6573598	LC_IGP12	IgG1_G1	14	65783019	1	6.21E-10	1.68E-16	+	0.280603	0.4081	+	C	T	0.185713762	0.0450961	+	C	T	0.1689	0.0495
rs6573598	LC_IGP_R2	IgG1_G1F/IgG1_G1	14	65783019	1	3.36E-10	5.67E-17	-	-0.28503	-0.4137	+	C	T	0.185713762	0.045098	+	C	T	0.1689	0.0494
rs6573598	LC_IGP53	IgG1_G1n	14	65783019	1	4.55E-09	1.38E-16	+	0.268728	0.409	+	C	T	0.184332975	0.0455832	+	C	T	0.1689	0.0495
rs6573598	LC_IGP_SC15	IgG1_G1N(20)/IgG2_G1N(20)	14	65783019	1	3.34E-08	8.89E-10	+	0.251984	0.2896	+	C	T	0.184121564	0.0454058	+	C	T	0.1689	0.0473
rs6573598	LC_IGP_R3	IgG1_G2F/IgG1_G2	14	65783019	1	3.35E-09	8.99E-17	-	-0.268748	-0.4088	+	C	T	0.185521655	0.0451956	+	C	T	0.1689	0.0492
rs6573599	LC_IGP78	IgG1 BG0n/G0n	14	65783127	1	1.37E-08	5.02E-16	+	0.260054	0.3996	+	A	C	0.184332975	0.0455694	+	A	C	0.1689	0.0493
rs6573599	LC_IGP86	IgG1 Bn/Fn total	14	65783127	1	3.53E-08	1.62E-16	+	0.253175	0.4078	+	A	C	0.184332975	0.0457007	+	A	C	0.1689	0.0494
rs6573599	LC_IGP84	IgG1 FBn/Bn total	14	65783127	1	1.33E-08	2.33E-20	-	-0.261385	-0.4605	+	A	C	0.184332975	0.0457666	+	A	C	0.1689	0.0498
rs6573599	LC_IGP62	IgG1 FG0n total/G0n	14	65783127	1	2.26E-10	6.85E-17	-	-0.289808	-0.4139	+	A	C	0.184332975	0.0454023	+	A	C	0.1689	0.0496
rs6573599	LC_IGP63	IgG1 FG1n total/G1n	14	65783127	1	1.45E-09	5.04E-18	-	-0.27742	-0.4285	+	A	C	0.184332975	0.0455874	+	A	C	0.1689	0.0495
rs6573599	LC_IGP64	IgG1 FG2n total/G2n	14	65783127	1	1.88E-08	8.41E-17	-	-0.258313	-0.4099	+	A	C	0.184332975	0.0457054	+	A	C	0.1689	0.0492
rs6573599	LC_IGP61	IgG1 Fn total	14	65783127	1	3.49E-10	1.14E-17	-	-0.287429	-0.4243	+	A	C	0.184332975	0.0455161	+	A	C	0.1689	0.0496
rs6573599	LC_IGP21	IgG1 Fucosylation	14	65783127	1	4.58E-10	4.52E-18	-	-0.283909	-0.429	+	A	C	0.185889907	0.0452699	+	A	C	0.1689	0.0495
rs6573599	LC_IGP11	IgG1_G0	14	65783127	1	7.85E-10	1.72E-17	+	0.278444	0.4196	+	A	C	0.185713762	0.0450227	+	A	C	0.1689	0.0493
rs6573599	LC_IGP_R1	IgG1_G0F/IgG1_G0	14	65783127	1	1.91E-10	3.30E-16	-	-0.288186	-0.4034	+	A	C	0.185713762	0.0449645	+	A	C	0.1689	0.0494
rs6573599	LC_IGP_R4	IgG1_G0FN/IgG1_G0N	14	65783127	1	9.44E-10	3.55E-19	-	-0.278146	-0.4454	+	A	C	0.185556294	0.045191	+	A	C	0.1689	0.0498
rs6573599	LC_IGP52	IgG1_G0n	14	65783127	1	2.16E-09	4.43E-18	+	0.273472	0.4281	+	A	C	0.184332975	0.0454316	+	A	C	0.1689	0.0494
rs6573599	LC_IGP14	IgG1_G0N	14	65783127	1	6.81E-10	3.80E-17	+	0.280407	0.4134	+	A	C	0.185556294	0.0451709	+	A	C	0.1689	0.0491
rs6573599	LC_IGP55	IgG1_G0Nn	14	65783127	1	6.29E-09	1.35E-17	+	0.266248	0.4198	+	A	C	0.184332975	0.0455878	+	A	C	0.1689	0.0492
rs6573599	LC_IGP12	IgG1_G1	14	65783127	1	6.21E-10	1.68E-16	+	0.280603	0.4081	+	A	C	0.185713762	0.0450961	+	A	C	0.1689	0.0495
rs6573599	LC_IGP_R2	IgG1_G1F/IgG1_G1	14	65783127	1	3.36E-10	5.67E-17	-	-0.28503	-0.4137	+	A	C	0.185713762	0.045098	+	A	C	0.1689	0.0494
rs6573599	LC_IGP53	IgG1_G1n	14	65783127	1	4.55E-09	1.39E-16	+	0.268728	0.409	+	A	C	0.184332975	0.0455832	+	A	C	0.1689	0.0495
rs6573599	LC_IGP_SC15	IgG1_G1N(20)/IgG2_G1N(20)	14	65783127	1	3.34E-08	8.89E-10	+	0.251984	0.2896	+	A	C	0.184121564	0.0454058	+	A	C	0.1689	0.0473
rs6573599	LC_IGP_R3	IgG1_G2F/IgG1_G2	14	65783127	1	3.35E-09	9.00E-17	-	-0.268748	-0.4088	+	A	C	0.185521655	0.0451956	+	A	C	0.1689	0.0492
rs6573600	LC_IGP78	IgG1 BG0n/G0n	14	65783270	1	1.39E-08	3.90E-16	+	0.260039	0.4048	+	G	T	0.184262669	0.0455884	+	G	T	0.1676	0.0497
rs6573600	LC_IGP86	IgG1 Bn/Fn total	14	65783270	1	3.60E-08	1.50E-16	+	0.253127	0.4121	+	G	T	0.184262669	0.0457198	+	G	T	0.1676	0.0499
rs6573600	LC_IGP84	IgG1 FBn/Bn total	14	65783270	1	1.35E-08	2.00E-20	-	-0.261371	-0.4657	+	G	T	0.184262669	0.0457856	+	G	T	0.1676	0.0503
rs6573600	LC_IGP62	IgG1 FG0n total/G0n	14	65783270	1	2.33E-10	5.55E-17	-	-0.289711	-0.419	+	G	T	0.184262669	0.0454216	+	G	T	0.1676	0.05
rs6573600	LC_IGP63	IgG1 FG1n total/G1n	14	65783270	1	1.51E-09	4.98E-18	-	-0.277245	-0.4327	+	G	T	0.184262669	0.045607	+	G	T	0.1676	0.05
rs6573600	LC_IGP64	IgG1 FG2n total/G2n	14	65783270	1	1.93E-08	9.88E-17	-	-0.2582	-0.4129	+	G	T	0.184262669	0.0457247	+	G	T	0.1676	0.0497
rs6573600	LC_IGP61	IgG1 Fn total	14	65783270	1	3.61E-10	1.10E-17	-	-0.287305	-0.4286	+	G	T	0.184262669	0.0455356	+	G	T	0.1676	0.05
rs6573600	LC_IGP21	IgG1 Fucosylation	14	65783270	1	4.74E-10	4.55E-18	-	-0.283781	-0.4333	+	G	T	0.18581994	0.045289	+	G	T	0.1676	0.05
rs6573600	LC_IGP11	IgG1_G0	14	65783270	1	8.24E-10	1.56E-17	+	0.278215	0.4243	+	G	T	0.185644437	0.0450419	+	G	T	0.1676	0.0498
rs6573600	LC_IGP_R1	IgG1_G0F/IgG1_G0	14	65783270	1	1.97E-10	2.69E-16	-	-0.288071	-0.4083	+	G	T	0.185644437	0.0449833	+	G	T	0.1676	0.0499
rs6573600	LC_IGP_R4	IgG1_G0FN/IgG1_G0N	14	65783270	1	9.52E-10	2.81E-19	-	-0.278195	-0.4508	+	G	T	0.185486799	0.0452093	+	G	T	0.1676	0.0502
rs6573600	LC_IGP52	IgG1_G0n	14	65783270	1	2.27E-09	4.09E-18	+	0.273235	0.4328	+	G	T	0.184262669	0.0454513	+	G	T	0.1676	0.0499
rs6573600	LC_IGP14	IgG1_G0N	14	65783270	1	6.99E-10	3.13E-17	+	0.280332	0.4185	+	G	T	0.185486799	0.0451897	+	G	T	0.1676	0.0496
rs6573600	LC_IGP55	IgG1_G0Nn	14	65783270	1	6.46E-09	1.14E-17	+	0.266147	0.4248	+	G	T	0.184262669	0.0456071	+	G	T	0.1676	0.0496
rs6573600	LC_IGP12	IgG1_G1	14	65783270	1	6.42E-10	1.56E-16	+	0.280474	0.4125	+	G	T	0.185644437	0.045115	+	G	T	0.1676	0.05
rs6573600	LC_IGP_R2	IgG1_G1F/IgG1_G1	14	65783270	1	3.51E-10	5.66E-17	-	-0.284847	-0.4177	+	G	T	0.185644437	0.0451171	+	G	T	0.1676	0.0499
rs6573600	LC_IGP53	IgG1_G1n	14	65783270	1	4.69E-09	1.33E-16	+	0.268595	0.4132	+	G	T	0.184262669	0.0456027	+	G	T	0.1676	0.05
rs6573600	LC_IGP_SC15	IgG1_G1N(20)/IgG2_G1N(20)	14	65783270	1	3.49E-08	9.96E-10	+	0.251729	0.291	+	G	T	0.184051692	0.0454254	+	G	T	0.1676	0.0476
rs6573600	LC_IGP_R3	IgG1_G2F/IgG1_G2	14	65783270	1	3.45E-09	1.08E-16	-	-0.268622	-0.4117	+	G	T	0.185452288	0.0452145	+	G	T	0.1676	0.0496

rs8004239	LC_IGP78	IgG1 BG0n/G0n	14	65783460	1	1.37E-08	3.59E-16	+	0.260037	0.4022	+	G	A	0.184333562	0.0455699	+	G	A	0.1713	0.0493
rs8004239	LC_IGP86	IgG1 Bn/Fn total	14	65783460	1	3.54E-08	1.05E-16	+	0.253153	0.4108	+	G	A	0.184333562	0.0457011	+	G	A	0.1713	0.0495
rs8004239	LC_IGP84	IgG1 FBn/Bn total	14	65783460	1	1.34E-08	1.16E-20	-	-0.261366	-0.4651	+	G	A	0.184333562	0.045767	+	G	A	0.1713	0.0499
rs8004239	LC_IGP62	IgG1 FG0n total/G0n	14	65783460	1	2.27E-10	4.54E-17	-	-0.289799	-0.4169	+	G	A	0.184333562	0.0454027	+	G	A	0.1713	0.0496
rs8004239	LC_IGP63	IgG1 FG1n total/G1n	14	65783460	1	1.45E-09	2.98E-18	-	-0.277414	-0.4319	+	G	A	0.184333562	0.0455878	+	G	A	0.1713	0.0496
rs8004239	LC_IGP64	IgG1 FG2n total/G2n	14	65783460	1	1.88E-08	5.10E-17	-	-0.258307	-0.4133	+	G	A	0.184333562	0.0457058	+	G	A	0.1713	0.0493
rs8004239	LC_IGP61	IgG1 Fn total	14	65783460	1	3.49E-10	7.10E-18	-	-0.287421	-0.4275	+	G	A	0.184333562	0.0455165	+	G	A	0.1713	0.0496
rs8004239	LC_IGP21	IgG1 Fucosylation	14	65783460	1	4.58E-10	2.93E-18	-	-0.283904	-0.432	+	G	A	0.185890488	0.0452703	+	G	A	0.1713	0.0496
rs8004239	LC_IGP11	IgG1_G0	14	65783460	1	7.86E-10	1.05E-17	+	0.27844	0.423	+	G	A	0.185714338	0.045023	+	G	A	0.1713	0.0494
rs8004239	LC_IGP_R1	IgG1_G0F/IgG1_G0	14	65783460	1	1.91E-10	2.31E-16	-	-0.28818	-0.406	+	G	A	0.185714338	0.0449649	+	G	A	0.1713	0.0495
rs8004239	LC_IGP_R4	IgG1_G0FN/IgG1_G0N	14	65783460	1	9.46E-10	1.92E-19	-	-0.278131	-0.4496	+	G	A	0.185556871	0.0451914	+	G	A	0.1713	0.0499
rs8004239	LC_IGP52	IgG1_G0n	14	65783460	1	2.16E-09	2.91E-18	+	0.273468	0.4312	+	G	A	0.184333562	0.0454319	+	G	A	0.1713	0.0495
rs8004239	LC_IGP14	IgG1_G0N	14	65783460	1	6.82E-10	2.59E-17	+	0.280395	0.4161	+	G	A	0.185556871	0.0451713	+	G	A	0.1713	0.0492
rs8004239	LC_IGP55	IgG1_G0Nn	14	65783460	1	6.30E-09	9.66E-18	+	0.266235	0.4223	+	G	A	0.184333562	0.0455882	+	G	A	0.1713	0.0492
rs8004239	LC_IGP12	IgG1_G1	14	65783460	1	6.22E-10	9.61E-17	+	0.280597	0.4119	+	G	A	0.185714338	0.0450965	+	G	A	0.1713	0.0496
rs8004239	LC_IGP_R2	IgG1_G1F/IgG1_G1	14	65783460	1	3.36E-10	3.58E-17	-	-0.285027	-0.4167	+	G	A	0.185714338	0.0450984	+	G	A	0.1713	0.0495
rs8004239	LC_IGP53	IgG1_G1n	14	65783460	1	4.55E-09	8.53E-17	+	0.268721	0.4124	+	G	A	0.184333562	0.0455836	+	G	A	0.1713	0.0495
rs8004239	LC_IGP_SC15	IgG1_G1N(20)/IgG2_G1N(20)	14	65783460	1	3.35E-08	6.79E-10	+	0.251963	0.2921	+	G	A	0.184122148	0.0454062	+	G	A	0.1713	0.0473
rs8004239	LC_IGP_R3	IgG1_G2F/IgG1_G2	14	65783460	1	3.35E-09	5.46E-17	-	-0.268743	-0.4121	+	G	A	0.185522232	0.045196	+	G	A	0.1713	0.0492
rs28740895	LC_IGP78	IgG1 BG0n/G0n	14	65783523	1	1.83E-11	7.86E-12	+	0.244596	0.2698	+	A	G	0.361578686	0.0361413	+	A	G	0.3335	0.0394
rs28740895	LC_IGP79	IgG1 BG1n/G1n	14	65783523	1	1.10E-10	2.86E-13	+	0.235546	0.2875	+	A	G	0.361578686	0.0362649	+	A	G	0.3335	0.0394
rs28740895	LC_IGP77	IgG1 Bn	14	65783523	1	4.04E-11	1.31E-12	+	0.241006	0.2807	+	A	G	0.361578686	0.0362501	+	A	G	0.3335	0.0396
rs28740895	LC_IGP86	IgG1 Bn/Fn total	14	65783523	1	1.10E-11	3.24E-13	+	0.247814	0.2883	+	A	G	0.361578686	0.0362135	+	A	G	0.3335	0.0396
rs28740895	LC_IGP84	IgG1 FBn/Bn total	14	65783523	1	3.08E-13	5.75E-19	-	-0.266223	-0.3548	+	A	G	0.361578686	0.0362082	+	A	G	0.3335	0.0399
rs28740895	LC_IGP62	IgG1 FG0n total/G0n	14	65783523	1	6.58E-13	5.35E-14	-	-0.261073	-0.2986	+	A	G	0.361578686	0.0360253	+	A	G	0.3335	0.0397
rs28740895	LC_IGP63	IgG1 FG1n total/G1n	14	65783523	1	4.55E-13	2.74E-16	-	-0.263647	-0.3244	+	A	G	0.361578686	0.0361234	+	A	G	0.3335	0.0396
rs28740895	LC_IGP64	IgG1 FG2n total/G2n	14	65783523	1	3.21E-11	2.11E-16	-	-0.242293	-0.3238	+	A	G	0.361578686	0.0362547	+	A	G	0.3335	0.0394
rs28740895	LC_IGP68	IgG1 FG2n/G2n	14	65783523	1	3.66E-08	2.88E-09	-	-0.201393	-0.2332	+	A	G	0.361578686	0.0363976	+	A	G	0.3335	0.0393
rs28740895	LC_IGP61	IgG1 Fn total	14	65783523	1	2.34E-13	1.36E-15	-	-0.266676	-0.317	+	A	G	0.361578686	0.0360834	+	A	G	0.3335	0.0397
rs28740895	LC_IGP81	IgG1 Fn/Bn	14	65783523	1	5.07E-11	2.25E-11	-	-0.239666	-0.2635	+	A	G	0.361578686	0.0362352	+	A	G	0.3335	0.0394
rs28740895	LC_IGP21	IgG1 Fucosylation	14	65783523	1	1.77E-12	1.23E-15	-	-0.255499	-0.317	+	A	G	0.36318619	0.0359486	+	A	G	0.3335	0.0396
rs28740895	LC_IGP11	IgG1_G0	14	65783523	1	4.47E-12	8.95E-15	+	0.249775	0.3061	+	A	G	0.363218082	0.0358172	+	A	G	0.3335	0.0395
rs28740895	LC_IGP_R1	IgG1_G0F/IgG1_G0	14	65783523	1	7.22E-12	4.45E-13	-	-0.247235	-0.2865	+	A	G	0.363218082	0.0358115	+	A	G	0.3335	0.0396
rs28740895	LC_IGP_R4	IgG1_G0FN/IgG1_G0N	14	65783523	1	7.10E-14	2.32E-16	-	-0.270626	-0.327	+	A	G	0.363189222	0.0358355	+	A	G	0.3335	0.0398
rs28740895	LC_IGP52	IgG1_G0n	14	65783523	1	4.57E-12	5.20E-15	+	0.251249	0.3094	+	A	G	0.361578686	0.0360418	+	A	G	0.3335	0.0396
rs28740895	LC_IGP14	IgG1_G0N	14	65783523	1	3.79E-13	1.97E-12	+	0.262631	0.2766	+	A	G	0.363189222	0.0358628	+	A	G	0.3335	0.0393
rs28740895	LC_IGP55	IgG1_G0Nn	14	65783523	1	3.07E-12	1.31E-12	+	0.253974	0.279	+	A	G	0.361578686	0.0361336	+	A	G	0.3335	0.0393
rs28740895	LC_IGP12	IgG1_G1	14	65783523	1	1.10E-11	1.29E-15	+	0.245863	0.317	+	A	G	0.362912645	0.0359369	+	A	G	0.3335	0.0396
rs28740895	LC_IGP_R2	IgG1_G1F/IgG1_G1	14	65783523	1	4.65E-12	4.43E-15	-	-0.25038	-0.3101	+	A	G	0.362912645	0.0359329	+	A	G	0.3335	0.0396
rs28740895	LC_IGP_R5	IgG1_G1FN/IgG1_G1N	14	65783523	1	4.09E-13	3.18E-20	-	-0.263589	-0.3653	+	A	G	0.36198524	0.0360447	+	A	G	0.3335	0.0396
rs28740895	LC_IGP53	IgG1_G1n	14	65783523	1	1.65E-11	1.84E-15	+	0.245375	0.315	+	A	G	0.361578686	0.0361741	+	A	G	0.3335	0.0396
rs28740895	LC_IGP15	IgG1_G1N	14	65783523	1	1.41E-10	2.54E-12	+	0.233424	0.2759	+	A	G	0.36198524	0.0361532	+	A	G	0.3335	0.0394
rs28740895	LC_IGP_SC15	IgG1_G1N(20)/IgG2_G1N(20)	14	65783523	1	2.43E-08	1.65E-07	+	0.202393	0.1985	+	A	G	0.362123153	0.0361055	+	A	G	0.3335	0.0379
rs28740895	LC_IGP56	IgG1_G1Nn	14	65783523	1	4.26E-10	5.10E-12	+	0.228085	0.2718	+	A	G	0.361578686	0.0363013	+	A	G	0.3335	0.0394
rs28740895	LC_IGP13	IgG1_G2	14	65783523	1	3.42E-08	3.86E-12	+	0.200261	0.2712	+	A	G	0.363218082	0.0361133	+	A	G	0.3335	0.0391
rs28740895	LC_IGP_R3	IgG1_G2F/IgG1_G2	14	65783523	1	9.16E-12	2.99E-16	-	-0.246777	-0.3215	+	A	G	0.363134474	0.0359261	+	A	G	0.3335	0.0393
rs10144503	LC_IGP78	IgG1 BG0n/G0n	14	65784109	1	3.95E-08	3.25E-09	+	0.311757	0.3915	+	A	G	0.10873214	0.0564811	+	A	G	0.0886	0.0661
rs10144503	LC_IGP62	IgG1 FG0n total/G0n	14	65784109	1	2.88E-11	8.16E-10	-	-0.376312	-0.4088	+	A	G	0.10873214	0.0561676	+	A	G	0.0886	0.0666
rs10144503	LC_IGP63	IgG1 FG1n total/G1n	14	65784109	1	3.89E-09	5.09E-10	-	-0.334564	-0.4134	+	A	G	0.10873214	0.056501	+	A	G	0.0886	0.0665
rs10144503	LC_IGP64	IgG1 FG2n total/G2n	14	65784109	1	2.21E-08	1.75E-10	-	-0.318372	-0.422	+	A	G	0.10873214	0.0566189	+	A	G	0.0886	0.0661
rs10144503	LC_IGP61	IgG1 Fn total	14	65784109	1	6.29E-10	3.56E-10	-	-0.35085	-0.4174	+	A	G	0.10873214	0.0563991	+	A	G	0.0886	0.0666
rs10144503	LC_IGP21	IgG1 Fucosylation	14	65784109	1	6.80E-10	1.37E-10	-	-0.346873	-0.4268	+	A	G	0.110045419	0.0558741	+	A	G	0.0886	0.0665
rs10144503	LC_IGP11	IgG1_G0	14	65784109	1	4.07E-12	2.41E-10	+	0.387171	0.4189	+	A	G	0.109647936	0.0554124	+	A	G	0.0886	0.0662
rs10144503	LC_IGP_R1	IgG1_G0F/IgG1_G0	14	65784109	1	7.05E-12	2.85E-09	-	-0.3827	-0.3941	+	A	G	0.109647936	0.0554059	+	A	G	0.0886	0.0664
rs10144503	LC_IGP_R4	IgG1_G0FN/IgG1_G0N	14	65784109	1	1.80E-08	6.44E-12	-	-0.316317	-0.4588	+	A	G	0.109610331	0.0558981	+	A	G	0.0886	0.0668
rs10144503	LC_IGP52	IgG1_G0n	14	65784109	1	5.08E-12	1.34E-10	+	0.389987	0.4257	+	A	G	0.10873214	0.056068	+	A	G	0.0886	0.0663
rs10144503	LC_IGP14	IgG1_G0N	14	65784109	1	5.69E-10	5.84E-10	+	0.347799	0.4082	+	A	G	0.109610331	0.0557691	+	A	G	0.0886	0.0659
rs10144503	LC_IGP55	IgG1_G0Nn	14	65784109	1	3.94E-09	4.59E-10	+	0.334157	0.4112	+	A	G	0.10873214	0.0564518	+	A	G	0.0886	0.066
rs10144503	LC_IGP12	IgG1_G1	14	65784109	1	4.56E-10	1.30E-09	+	0.348821	0.4034	+	A	G	0.109953372	0.0556183	+	A	G	0.0886	0.0665
rs10144503	LC_IGP_R2	IgG1_G1F/IgG1_G1	14	65784109	1	2.31E-10	2.24E-09	-	-0.354791	-0.3967	+	A	G	0.109953372	0.0556185	+	A	G	0.0886	0.0664
rs10144503	LC_IGP53	IgG1_G1n	14	65784109	1	1.47E-08	1.49E-09	+	0.321748	0.4016	+	A	G	0.10873214	0.0565021	+	A	G	0.0886	0.0664
rs10144503	LC_IGP_R3	IgG1_G2F/IgG1_G2	14	65784109	1	3.50E-09	1.42E-10	-	-0.331594	-0.4233	+	A	G	0.109409335	0.0558331	+	A	G	0.0886	0.066
rs8010759	LC_IGP78	IgG1 BG0n/G0n	14	65784559	1	4.67E-08	2.26E-09	+	0.312453	0.3966	+	A	G	0.10761834	0.0569153	+	A	G	0.0884	0.0664
rs8010759	LC_IGP62	IgG1 FG0n total/G0n	14	65784559	1	3.50E-11	5.20E-10	-	-0.377537	-0.4147	+	A	G	0.10761834	0.0566005	+	A	G	0.0884	0.0668
rs8010759	LC_IGP63	IgG1 FG1n total/G1n	14	65784559	1	2.34E-09	2.91E-10	-	-0.341826	-0.4199	+	A	G	0.10761834	0.0569122	+	A	G	0.0884	0.0666
rs8010759	LC_IGP64	IgG1 FG2n total/G2n	14	65784559	1	1.38E-08	1.04E-10	-	-0.325358	-0.4279	+	A	G	0.10761834	0.0570325	+	A	G	0.0884	0.0662
rs8010759	LC_IGP61	IgG1 Fn total	14	65784559	1	4.86E-10	2.09E-10	-	-0.355799	-0.4238	+	A	G	0.10761834	0.0568182	+	A	G	0.0884	0.0667
rs8010759	LC_IGP21	IgG1 Fucosylation	14	65784559	1	4.49E-10	1.19E-10	-	-0.353105	-0.4296	+	A	G	0.108937115	0.0562744	+	A	G	0.0884	0.0667
rs8010759	LC_IGP11	IgG1_G0	14	65784559	1	5.43E-12	1.79E-10	+	0.38776	0.4237	+	A	G	0.108549786	0.0558311	+	A	G	0.0884	0.0664
rs8010759	LC_IGP_R1	IgG1_G0F/IgG1_G0	14	65784559	1	8.30E-12	1.85E-09	-	-0.384232	-0.3999	+	A	G	0.108549786	0.0558203	+	A	G	0.0884	0.0665
rs8010759	LC_IGP_R4	IgG1_G0FN/IgG1_G0N	14	65784559	1	2.03E-08	4.10E-12	-	-0.317474	-0.4647	+	A	G	0.108509492	0.0563159	+	A	G	0.0884	0.067
rs8010759	LC_IGP52	IgG1_G0n	14	65784559	1	6.66E-12	9.91E-11	+	0.390795	0.4306	+	A	G	0.10761834	0.0565027	+	A	G	0.0884	0.0666
rs8010759	LC_IGP14	IgG1_G0N	14	65784559	1	7.25E-10	4.71E-10	+	0.34824	0.4124	+	A	G	0.108509492	0.0561899	+	A	G	0.0884	0.0662
rs8010759	LC_IGP55	IgG1_G0Nn	14	65784559	1	5.05E-09	3.75E-10	+	0.334368	0.4152	+	A	G	0.10761834	0.0568886	+	A	G	0.0884	0.0663
rs8010759	LC_IGP12	IgG1_G1	14	65784559	1	3.00E-10	6.68E-10	+	0.355025	0.411	+	A	G	0.108855223	0.0560137	+	A	G	0.0884	0.0666
rs8010759	LC_IGP_R2	IgG1_G1F/IgG1_G1	14	65784559	1	1.34E-10	1.30E-09	-	-0.362066	-0.4033	+	A	G	0.108855223	0.0560096	+	A	G	0.0884	0.0665
rs8010759	LC_IGP53	IgG1_G1n	14	65784559	1	9.85E-09	7.76E-10	+	0.328044	0.4091	+	A	G	0.10761834	0.0569171	+	A	G	0.0884	0.0665
rs8010759	LC_IGP_R3	IgG1_G2F/IgG1_G2	14	65784559	1	2.13E-09	8.15E-11	-	-0.338579	-0.4295	+	A	G	0.108310513	0.0562298	+	A	G	0.0884	0.0661
rs6573602	LC_IGP78	IgG1 BG0n/G0n	14	65784610	1	1.38E-08	5.02E-16	+	0.259991	0.3996	+	C	G	0.18433454	0.0455711	+	C	G	0.1689	0.0493
rs6573602	LC_IGP86	IgG1 Bn/Fn total	14	65784610	1	3.57E-08	1.62E-16	+	0.253093	0.4078	+	C	G	0.18433454	0.0457025	+	C	G	0.1689	0.0494
rs6573602	LC_IGP84	IgG1 FBn/Bn total	14	65784610	1	1.35E-08	2.33E-20	-	-0.261312	-0.4605	+	C	G	0.18433454	0.0457683	+	C	G	0.1689	0.0498
rs6573602	LC_IGP62	IgG1 FG0n total/G0n	14	65784610	1	2.28E-10	6.86E-17	-	-0.289771	-0.4139	+	C	G	0.18433454	0.0454039	+	C	G	0.1689	0.0496
rs6573602	LC_IGP63	IgG1 FG1n total/G1n	14	65784610	1	1.46E-09	5.05E-18	-	-0.277395	-0.4285	+	C	G	0.18433454	0.045589	+	C	G	0.1689	0.0495
rs6573602	LC_IGP64	IgG1 FG2n total/G2n	14	65784610	1	1.88E-08	8.42E-17	-	-0.258286	-0.41	+	C	G	0.18433454	0.045707	+	C	G	0.1689	0.0492
rs6573602	LC_IGP61	IgG1 Fn total	14	65784610	1	3.51E-10	1.15E-17	-	-0.287396	-0.4243	+	C	G	0.18433454	0.0455178	+	C	G	0.1689	0.0496
rs6573602	LC_IGP21	IgG1 Fucosylation	14	65784610	1	4.60E-10	4.53E-18	-	-0.283886	-0.4291	+	C	G	0.185891456	0.0452715	+	C	G	0.1689	0.0495
rs6573602	LC_IGP11	IgG1_G0	14	65784610	1	7.88E-10	1.71E-17	+	0.278428	0.4196	+	C	G	0.185715298	0.0450242	+	C	G	0.1689	0.0493
rs6573602	LC_IGP_R1	IgG1_G0F/IgG1_G0	14	65784610	1	1.92E-10	3.30E-16	-	-0.288162	-0.4034	+	C	G	0.185715298	0.0449661	+	C	G	0.1689	0.0494
rs6573602	LC_IGP_R4	IgG1_G0FN/IgG1_G0N	14	65784610	1	9.52E-10	3.55E-19	-	-0.278092	-0.4454	+	C	G	0.185557834	0.0451927	+	C	G	0.1689	0.0498
rs6573602	LC_IGP52	IgG1_G0n	14	65784610	1	2.17E-09	4.43E-18	+	0.273454	0.4282	+	C	G	0.18433454	0.0454331	+	C	G	0.1689	0.0494
rs6573602	LC_IGP14	IgG1_G0N	14	65784610	1	6.86E-10	3.80E-17	+	0.280361	0.4134	+	C	G	0.185557834	0.0451726	+	C	G	0.1689	0.0491
rs6573602	LC_IGP55	IgG1_G0Nn	14	65784610	1	6.34E-09	1.35E-17	+	0.266199	0.4198	+	C	G	0.18433454	0.0455894	+	C	G	0.1689	0.0492
rs6573602	LC_IGP12	IgG1_G1	14	65784610	1	6.24E-10	1.68E-16	+	0.280577	0.4081	+	C	G	0.185715298	0.0450976	+	C	G	0.1689	0.0495
rs6573602	LC_IGP_R2	IgG1_G1F/IgG1_G1	14	65784610	1	3.37E-10	5.69E-17	-	-0.285015	-0.4137	+	C	G	0.185715298	0.0450995	+	C	G	0.1689	0.0494
rs6573602	LC_IGP53	IgG1_G1n	14	65784610	1	4.57E-09	1.39E-16	+	0.268699	0.409	+	C	G	0.18433454	0.0455848	+	C	G	0.1689	0.0495
rs6573602	LC_IGP_SC15	IgG1_G1N(20)/IgG2_G1N(20)	14	65784610	1	3.38E-08	8.91E-10	+	0.251904	0.2896	+	C	G	0.18412312	0.0454076	+	C	G	0.1689	0.0473
rs6573602	LC_IGP_R3	IgG1_G2F/IgG1_G2	14	65784610	1	3.36E-09	9.01E-17	-	-0.268727	-0.4088	+	C	G	0.185523193	0.0451971	+	C	G	0.1689	0.0492
rs17102598	LC_IGP78	IgG1 BG0n/G0n	14	65784860	1	1.38E-08	5.03E-16	+	0.25998	0.3996	+	T	G	0.184334735	0.0455715	+	T	G	0.1689	0.0493
rs17102598	LC_IGP86	IgG1 Bn/Fn total	14	65784860	1	3.58E-08	1.62E-16	+	0.253077	0.4078	+	T	G	0.184334735	0.0457028	+	T	G	0.1689	0.0494
rs17102598	LC_IGP84	IgG1 FBn/Bn total	14	65784860	1	1.35E-08	2.33E-20	-	-0.261298	-0.4605	+	T	G	0.184334735	0.0457687	+	T	G	0.1689	0.0498
rs17102598	LC_IGP62	IgG1 FG0n total/G0n	14	65784860	1	2.28E-10	6.86E-17	-	-0.289764	-0.4139	+	T	G	0.184334735	0.0454043	+	T	G	0.1689	0.0496
rs17102598	LC_IGP63	IgG1 FG1n total/G1n	14	65784860	1	1.46E-09	5.05E-18	-	-0.27739	-0.4285	+	T	G	0.184334735	0.0455893	+	T	G	0.1689	0.0495
rs17102598	LC_IGP64	IgG1 FG2n total/G2n	14	65784860	1	1.89E-08	8.42E-17	-	-0.25828	-0.41	+	T	G	0.184334735	0.0457073	+	T	G	0.1689	0.0492
rs17102598	LC_IGP61	IgG1 Fn total	14	65784860	1	3.51E-10	1.15E-17	-	-0.287388	-0.4243	+	T	G	0.184334735	0.0455181	+	T	G	0.1689	0.0496
rs17102598	LC_IGP21	IgG1 Fucosylation	14	65784860	1	4.60E-10	4.53E-18	-	-0.28388	-0.4291	+	T	G	0.18589165	0.0452718	+	T	G	0.1689	0.0495
rs17102598	LC_IGP11	IgG1_G0	14	65784860	1	7.89E-10	1.71E-17	+	0.278424	0.4196	+	T	G	0.18571549	0.0450245	+	T	G	0.1689	0.0493
rs17102598	LC_IGP_R1	IgG1_G0F/IgG1_G0	14	65784860	1	1.92E-10	3.30E-16	-	-0.288156	-0.4034	+	T	G	0.18571549	0.0449664	+	T	G	0.1689	0.0494
rs17102598	LC_IGP_R4	IgG1_G0FN/IgG1_G0N	14	65784860	1	9.54E-10	3.55E-19	-	-0.278081	-0.4454	+	T	G	0.185558027	0.045193	+	T	G	0.1689	0.0498
rs17102598	LC_IGP52	IgG1_G0n	14	65784860	1	2.17E-09	4.43E-18	+	0.27345	0.4282	+	T	G	0.184334735	0.0454334	+	T	G	0.1689	0.0494
rs17102598	LC_IGP14	IgG1_G0N	14	65784860	1	6.87E-10	3.80E-17	+	0.280353	0.4134	+	T	G	0.185558027	0.0451729	+	T	G	0.1689	0.0491
rs17102598	LC_IGP55	IgG1_G0Nn	14	65784860	1	6.34E-09	1.35E-17	+	0.266191	0.4198	+	T	G	0.184334735	0.0455898	+	T	G	0.1689	0.0492
rs17102598	LC_IGP12	IgG1_G1	14	65784860	1	6.25E-10	1.68E-16	+	0.280571	0.4081	+	T	G	0.18571549	0.045098	+	T	G	0.1689	0.0495
rs17102598	LC_IGP_R2	IgG1_G1F/IgG1_G1	14	65784860	1	3.37E-10	5.69E-17	-	-0.285011	-0.4137	+	T	G	0.18571549	0.0450998	+	T	G	0.1689	0.0494
rs17102598	LC_IGP53	IgG1_G1n	14	65784860	1	4.57E-09	1.39E-16	+	0.268692	0.409	+	T	G	0.184334735	0.0455852	+	T	G	0.1689	0.0495
rs17102598	LC_IGP_SC15	IgG1_G1N(20)/IgG2_G1N(20)	14	65784860	1	3.38E-08	8.90E-10	+	0.251888	0.2896	+	T	G	0.184123315	0.0454079	+	T	G	0.1689	0.0473
rs17102598	LC_IGP_R3	IgG1_G2F/IgG1_G2	14	65784860	1	3.36E-09	9.01E-17	-	-0.268722	-0.4088	+	T	G	0.185523385	0.0451974	+	T	G	0.1689	0.0492
rs12436299	LC_IGP78	IgG1 BG0n/G0n	14	65785194	1	3.97E-08	3.25E-09	+	0.311687	0.3915	+	A	G	0.108736059	0.0564783	+	A	G	0.0886	0.0662
rs12436299	LC_IGP62	IgG1 FG0n total/G0n	14	65785194	1	2.90E-11	8.15E-10	-	-0.376234	-0.4088	+	A	G	0.108736059	0.0561649	+	A	G	0.0886	0.0666
rs12436299	LC_IGP63	IgG1 FG1n total/G1n	14	65785194	1	3.92E-09	5.08E-10	-	-0.334476	-0.4134	+	A	G	0.108736059	0.0564982	+	A	G	0.0886	0.0665

rs12436299	LC_IGP64	IgG1 FG2n total/G2n	14	65785194	1	2.22E-08	1.74E-10	-	-0.318277	-0.422	+	A	G	0.108736059	0.0566162	+	A	G	0.0886	0.0661
rs12436299	LC_IGP61	IgG1 Fn total	14	65785194	1	6.34E-10	3.56E-10	-	-0.350762	-0.4175	+	A	G	0.108736059	0.0563964	+	A	G	0.0886	0.0666
rs12436299	LC_IGP21	IgG1 Fucosylation	14	65785194	1	6.86E-10	1.37E-10	-	-0.346775	-0.4268	+	A	G	0.110049322	0.0558715	+	A	G	0.0886	0.0665
rs12436299	LC_IGP11	IgG1_G0	14	65785194	1	4.10E-12	2.41E-10	+	0.387087	0.4189	+	A	G	0.109651802	0.0554099	+	A	G	0.0886	0.0662
rs12436299	LC_IGP_R1	IgG1_G0F/IgG1_G0	14	65785194	1	7.10E-12	2.85E-09	-	-0.382624	-0.3942	+	A	G	0.109651802	0.0554033	+	A	G	0.0886	0.0664
rs12436299	LC_IGP_R4	IgG1_G0FN/IgG1_G0N	14	65785194	1	1.81E-08	6.43E-12	-	-0.316249	-0.4589	+	A	G	0.109614207	0.0558954	+	A	G	0.0886	0.0668
rs12436299	LC_IGP52	IgG1_G0n	14	65785194	1	5.13E-12	1.34E-10	+	0.3899	0.4258	+	A	G	0.108736059	0.0560653	+	A	G	0.0886	0.0663
rs12436299	LC_IGP14	IgG1_G0N	14	65785194	1	5.73E-10	5.82E-10	+	0.347722	0.4083	+	A	G	0.109614207	0.0557664	+	A	G	0.0886	0.0659
rs12436299	LC_IGP55	IgG1_G0Nn	14	65785194	1	3.97E-09	4.58E-10	+	0.334077	0.4112	+	A	G	0.108736059	0.056449	+	A	G	0.0886	0.066
rs12436299	LC_IGP12	IgG1_G1	14	65785194	1	4.59E-10	1.30E-09	+	0.348747	0.4034	+	A	G	0.109957239	0.0556157	+	A	G	0.0886	0.0665
rs12436299	LC_IGP_R2	IgG1_G1F/IgG1_G1	14	65785194	1	2.33E-10	2.24E-09	-	-0.354701	-0.3967	+	A	G	0.109957239	0.055616	+	A	G	0.0886	0.0664
rs12436299	LC_IGP53	IgG1_G1n	14	65785194	1	1.48E-08	1.49E-09	+	0.321672	0.4017	+	A	G	0.108736059	0.0564993	+	A	G	0.0886	0.0664
rs12436299	LC_IGP_R3	IgG1_G2F/IgG1_G2	14	65785194	1	3.52E-09	1.42E-10	-	-0.331503	-0.4233	+	A	G	0.109413203	0.0558305	+	A	G	0.0886	0.066
rs10137464	LC_IGP78	IgG1 BG0n/G0n	14	65785829	1	1.40E-08	5.04E-16	+	0.259917	0.3996	+	C	T	0.184337638	0.0455731	+	C	T	0.169	0.0493
rs10137464	LC_IGP86	IgG1 Bn/Fn total	14	65785829	1	3.62E-08	1.63E-16	+	0.252994	0.4078	+	C	T	0.184337638	0.0457045	+	C	T	0.169	0.0494
rs10137464	LC_IGP84	IgG1 FBn/Bn total	14	65785829	1	1.36E-08	2.34E-20	-	-0.261224	-0.4605	+	C	T	0.184337638	0.0457703	+	C	T	0.169	0.0498
rs10137464	LC_IGP62	IgG1 FG0n total/G0n	14	65785829	1	2.30E-10	6.89E-17	-	-0.289726	-0.4139	+	C	T	0.184337638	0.0454058	+	C	T	0.169	0.0496
rs10137464	LC_IGP63	IgG1 FG1n total/G1n	14	65785829	1	1.46E-09	5.07E-18	-	-0.277364	-0.4285	+	C	T	0.184337638	0.0455908	+	C	T	0.169	0.0495
rs10137464	LC_IGP64	IgG1 FG2n total/G2n	14	65785829	1	1.89E-08	8.47E-17	-	-0.258251	-0.4099	+	C	T	0.184337638	0.0457088	+	C	T	0.169	0.0492
rs10137464	LC_IGP61	IgG1 Fn total	14	65785829	1	3.54E-10	1.15E-17	-	-0.287354	-0.4242	+	C	T	0.184337638	0.0455196	+	C	T	0.169	0.0496
rs10137464	LC_IGP21	IgG1 Fucosylation	14	65785829	1	4.63E-10	4.55E-18	-	-0.283855	-0.429	+	C	T	0.185894539	0.0452733	+	C	T	0.169	0.0495
rs10137464	LC_IGP11	IgG1_G0	14	65785829	1	7.92E-10	1.72E-17	+	0.278407	0.4196	+	C	T	0.185718352	0.0450259	+	C	T	0.169	0.0493
rs10137464	LC_IGP_R1	IgG1_G0F/IgG1_G0	14	65785829	1	1.93E-10	3.32E-16	-	-0.288131	-0.4033	+	C	T	0.185718352	0.0449678	+	C	T	0.169	0.0494
rs10137464	LC_IGP_R4	IgG1_G0FN/IgG1_G0N	14	65785829	1	9.62E-10	3.56E-19	-	-0.278025	-0.4454	+	C	T	0.185560896	0.0451946	+	C	T	0.169	0.0498
rs10137464	LC_IGP52	IgG1_G0n	14	65785829	1	2.18E-09	4.44E-18	+	0.273431	0.4282	+	C	T	0.184337638	0.0454349	+	C	T	0.169	0.0494
rs10137464	LC_IGP14	IgG1_G0N	14	65785829	1	6.92E-10	3.81E-17	+	0.280307	0.4134	+	C	T	0.185560896	0.0451744	+	C	T	0.169	0.0491
rs10137464	LC_IGP55	IgG1_G0Nn	14	65785829	1	6.39E-09	1.35E-17	+	0.266142	0.4198	+	C	T	0.184337638	0.0455913	+	C	T	0.169	0.0492
rs10137464	LC_IGP12	IgG1_G1	14	65785829	1	6.28E-10	1.69E-16	+	0.280544	0.4081	+	C	T	0.185718352	0.0450994	+	C	T	0.169	0.0495
rs10137464	LC_IGP_R2	IgG1_G1F/IgG1_G1	14	65785829	1	3.39E-10	5.72E-17	-	-0.284995	-0.4137	+	C	T	0.185718352	0.0451013	+	C	T	0.169	0.0494
rs10137464	LC_IGP53	IgG1_G1n	14	65785829	1	4.60E-09	1.39E-16	+	0.268661	0.409	+	C	T	0.184337638	0.0455867	+	C	T	0.169	0.0495
rs10137464	LC_IGP_SC15	IgG1_G1N(20)/IgG2_G1N(20)	14	65785829	1	3.42E-08	8.96E-10	+	0.251805	0.2896	+	C	T	0.184126199	0.0454096	+	C	T	0.169	0.0473
rs10137464	LC_IGP_R3	IgG1_G2F/IgG1_G2	14	65785829	1	3.38E-09	9.07E-17	-	-0.268701	-0.4088	+	C	T	0.185526249	0.0451989	+	C	T	0.169	0.0492
rs8006316	LC_IGP78	IgG1 BG0n/G0n	14	65787115	1	1.60E-08	5.21E-16	+	0.258939	0.3995	+	T	A	0.184375203	0.0455928	+	T	A	0.1691	0.0493
rs8006316	LC_IGP86	IgG1 Bn/Fn total	14	65787115	1	4.28E-08	1.69E-16	+	0.251727	0.4077	+	T	A	0.184375203	0.0457251	+	T	A	0.1691	0.0495
rs8006316	LC_IGP84	IgG1 FBn/Bn total	14	65787115	1	1.60E-08	2.42E-20	-	-0.260091	-0.4605	+	T	A	0.184375203	0.0457906	+	T	A	0.1691	0.0498
rs8006316	LC_IGP62	IgG1 FG0n total/G0n	14	65787115	1	2.54E-10	7.10E-17	-	-0.289115	-0.4138	+	T	A	0.184375203	0.0454245	+	T	A	0.1691	0.0496
rs8006316	LC_IGP63	IgG1 FG1n total/G1n	14	65787115	1	1.57E-09	5.26E-18	-	-0.276939	-0.4284	+	T	A	0.184375203	0.0456087	+	T	A	0.1691	0.0495
rs8006316	LC_IGP64	IgG1 FG2n total/G2n	14	65787115	1	2.03E-08	8.72E-17	-	-0.257786	-0.4099	+	T	A	0.184375203	0.0457267	+	T	A	0.1691	0.0493
rs8006316	LC_IGP61	IgG1 Fn total	14	65787115	1	3.88E-10	1.19E-17	-	-0.286794	-0.4242	+	T	A	0.184375203	0.0455381	+	T	A	0.1691	0.0496
rs8006316	LC_IGP21	IgG1 Fucosylation	14	65787115	1	4.98E-10	4.68E-18	-	-0.283437	-0.429	+	T	A	0.185931918	0.0452909	+	T	A	0.1691	0.0495
rs8006316	LC_IGP11	IgG1_G0	14	65787115	1	8.38E-10	1.72E-17	+	0.278098	0.4198	+	T	A	0.185755389	0.0450429	+	T	A	0.1691	0.0493
rs8006316	LC_IGP_R1	IgG1_G0F/IgG1_G0	14	65787115	1	2.08E-10	3.43E-16	-	-0.2877	-0.4032	+	T	A	0.185755389	0.0449853	+	T	A	0.1691	0.0494
rs8006316	LC_IGP_R4	IgG1_G0FN/IgG1_G0N	14	65787115	1	1.10E-09	3.67E-19	-	-0.27716	-0.4453	+	T	A	0.185598023	0.0452138	+	T	A	0.1691	0.0498
rs8006316	LC_IGP52	IgG1_G0n	14	65787115	1	2.31E-09	4.48E-18	+	0.273099	0.4283	+	T	A	0.184375203	0.0454523	+	T	A	0.1691	0.0494
rs8006316	LC_IGP14	IgG1_G0N	14	65787115	1	7.78E-10	3.84E-17	+	0.279573	0.4135	+	T	A	0.185598023	0.0451931	+	T	A	0.1691	0.0491
rs8006316	LC_IGP55	IgG1_G0Nn	14	65787115	1	7.16E-09	1.37E-17	+	0.265372	0.4199	+	T	A	0.184375203	0.0456104	+	T	A	0.1691	0.0492
rs8006316	LC_IGP12	IgG1_G1	14	65787115	1	6.78E-10	1.74E-16	+	0.280099	0.4081	+	T	A	0.185755389	0.045117	+	T	A	0.1691	0.0495
rs8006316	LC_IGP_R2	IgG1_G1F/IgG1_G1	14	65787115	1	3.58E-10	5.91E-17	-	-0.28471	-0.4136	+	T	A	0.185755389	0.0451182	+	T	A	0.1691	0.0494
rs8006316	LC_IGP53	IgG1_G1n	14	65787115	1	4.97E-09	1.44E-16	+	0.268163	0.4089	+	T	A	0.184375203	0.0456047	+	T	A	0.1691	0.0495
rs8006316	LC_IGP_SC15	IgG1_G1N(20)/IgG2_G1N(20)	14	65787115	1	4.05E-08	9.29E-10	+	0.250559	0.2894	+	T	A	0.184163534	0.0454299	+	T	A	0.1691	0.0473
rs8006316	LC_IGP_R3	IgG1_G2F/IgG1_G2	14	65787115	1	3.59E-09	9.36E-17	-	-0.268336	-0.4087	+	T	A	0.185563308	0.0452161	+	T	A	0.1691	0.0492
rs6573604	LC_IGP78	IgG1 BG0n/G0n	14	65787941	1	2.03E-08	5.50E-16	+	0.257133	0.3993	+	C	T	0.184439281	0.045607	+	C	T	0.1693	0.0493
rs6573604	LC_IGP84	IgG1 FBn/Bn total	14	65787941	1	2.08E-08	2.56E-20	-	-0.258034	-0.4603	+	C	T	0.184439281	0.0458058	+	C	T	0.1693	0.0498
rs6573604	LC_IGP62	IgG1 FG0n total/G0n	14	65787941	1	3.06E-10	7.50E-17	-	-0.287883	-0.4136	+	C	T	0.184439281	0.0454372	+	C	T	0.1693	0.0496
rs6573604	LC_IGP63	IgG1 FG1n total/G1n	14	65787941	1	1.79E-09	5.61E-18	-	-0.276024	-0.4283	+	C	T	0.184439281	0.04562	+	C	T	0.1693	0.0496
rs6573604	LC_IGP64	IgG1 FG2n total/G2n	14	65787941	1	2.31E-08	9.24E-17	-	-0.256824	-0.4097	+	C	T	0.184439281	0.0457379	+	C	T	0.1693	0.0493
rs6573604	LC_IGP61	IgG1 Fn total	14	65787941	1	4.59E-10	1.27E-17	-	-0.28565	-0.424	+	C	T	0.184439281	0.0455505	+	C	T	0.1693	0.0496
rs6573604	LC_IGP21	IgG1 Fucosylation	14	65787941	1	5.69E-10	4.95E-18	-	-0.282532	-0.4288	+	C	T	0.185995684	0.0453023	+	C	T	0.1693	0.0495
rs6573604	LC_IGP11	IgG1_G0	14	65787941	1	9.33E-10	1.73E-17	+	0.277377	0.4199	+	C	T	0.185818571	0.0450534	+	C	T	0.1693	0.0493
rs6573604	LC_IGP_R1	IgG1_G0F/IgG1_G0	14	65787941	1	2.40E-10	3.63E-16	-	-0.286768	-0.403	+	C	T	0.185818571	0.0449967	+	C	T	0.1693	0.0494
rs6573604	LC_IGP_R4	IgG1_G0FN/IgG1_G0N	14	65787941	1	1.39E-09	3.88E-19	-	-0.275529	-0.4452	+	C	T	0.185661359	0.0452277	+	C	T	0.1693	0.0498
rs6573604	LC_IGP52	IgG1_G0n	14	65787941	1	2.58E-09	4.55E-18	+	0.27234	0.4284	+	C	T	0.184439281	0.0454631	+	C	T	0.1693	0.0494
rs6573604	LC_IGP14	IgG1_G0N	14	65787941	1	9.54E-10	3.89E-17	+	0.278158	0.4136	+	C	T	0.185661359	0.0452063	+	C	T	0.1693	0.0492
rs6573604	LC_IGP55	IgG1_G0Nn	14	65787941	1	8.72E-09	1.40E-17	+	0.263906	0.42	+	C	T	0.184439281	0.0456235	+	C	T	0.1693	0.0492
rs6573604	LC_IGP12	IgG1_G1	14	65787941	1	7.80E-10	1.83E-16	+	0.279151	0.4079	+	C	T	0.185818571	0.0451283	+	C	T	0.1693	0.0495
rs6573604	LC_IGP_R2	IgG1_G1F/IgG1_G1	14	65787941	1	3.97E-10	6.27E-17	-	-0.284021	-0.4135	+	C	T	0.185818571	0.0451287	+	C	T	0.1693	0.0495
rs6573604	LC_IGP53	IgG1_G1n	14	65787941	1	5.73E-09	1.53E-16	+	0.267133	0.4087	+	C	T	0.184439281	0.0456164	+	C	T	0.1693	0.0495
rs6573604	LC_IGP_R3	IgG1_G2F/IgG1_G2	14	65787941	1	4.03E-09	9.95E-17	-	-0.26753	-0.4085	+	C	T	0.185626528	0.0452267	+	C	T	0.1693	0.0492
rs57071950	LC_IGP78	IgG1 BG0n/G0n	14	65788437	1	2.35E-08	5.69E-16	+	0.255953	0.3992	+	T	C	0.18447587	0.045605	+	T	C	0.1694	0.0493
rs57071950	LC_IGP84	IgG1 FBn/Bn total	14	65788437	1	2.45E-08	2.64E-20	-	-0.256705	-0.4602	+	T	C	0.18447587	0.0458042	+	T	C	0.1694	0.0498
rs57071950	LC_IGP62	IgG1 FG0n total/G0n	14	65788437	1	3.43E-10	7.73E-17	-	-0.28703	-0.4135	+	T	C	0.18447587	0.0454344	+	T	C	0.1694	0.0496
rs57071950	LC_IGP63	IgG1 FG1n total/G1n	14	65788437	1	1.95E-09	5.81E-18	-	-0.27536	-0.4281	+	T	C	0.18447587	0.0456163	+	T	C	0.1694	0.0496
rs57071950	LC_IGP64	IgG1 FG2n total/G2n	14	65788437	1	2.51E-08	9.53E-17	-	-0.256139	-0.4096	+	T	C	0.18447587	0.0457341	+	T	C	0.1694	0.0493
rs57071950	LC_IGP61	IgG1 Fn total	14	65788437	1	5.12E-10	1.32E-17	-	-0.284848	-0.4238	+	T	C	0.18447587	0.0455474	+	T	C	0.1694	0.0496
rs57071950	LC_IGP21	IgG1 Fucosylation	14	65788437	1	6.22E-10	5.10E-18	-	-0.281871	-0.4287	+	T	C	0.186032095	0.0452988	+	T	C	0.1694	0.0495
rs57071950	LC_IGP11	IgG1_G0	14	65788437	1	1.00E-09	1.74E-17	+	0.276827	0.42	+	T	C	0.185854647	0.0450495	+	T	C	0.1694	0.0494
rs57071950	LC_IGP_R1	IgG1_G0F/IgG1_G0	14	65788437	1	2.64E-10	3.75E-16	-	-0.286095	-0.4028	+	T	C	0.185854647	0.0449934	+	T	C	0.1694	0.0494
rs57071950	LC_IGP_R4	IgG1_G0FN/IgG1_G0N	14	65788437	1	1.60E-09	4.00E-19	-	-0.274445	-0.4451	+	T	C	0.185697524	0.0452257	+	T	C	0.1694	0.0498
rs57071950	LC_IGP52	IgG1_G0n	14	65788437	1	2.77E-09	4.59E-18	+	0.271768	0.4284	+	T	C	0.18447587	0.045459	+	T	C	0.1694	0.0494
rs57071950	LC_IGP14	IgG1_G0N	14	65788437	1	1.09E-09	3.93E-17	+	0.277196	0.4137	+	T	C	0.185697524	0.0452039	+	T	C	0.1694	0.0492
rs57071950	LC_IGP55	IgG1_G0Nn	14	65788437	1	9.87E-09	1.42E-17	+	0.262919	0.42	+	T	C	0.18447587	0.0456209	+	T	C	0.1694	0.0492
rs57071950	LC_IGP12	IgG1_G1	14	65788437	1	8.53E-10	1.88E-16	+	0.278469	0.4078	+	T	C	0.185854647	0.0451249	+	T	C	0.1694	0.0496
rs57071950	LC_IGP_R2	IgG1_G1F/IgG1_G1	14	65788437	1	4.27E-10	6.48E-17	-	-0.283489	-0.4133	+	T	C	0.185854647	0.0451247	+	T	C	0.1694	0.0495
rs57071950	LC_IGP53	IgG1_G1n	14	65788437	1	6.28E-09	1.58E-16	+	0.266408	0.4086	+	T	C	0.18447587	0.0456129	+	T	C	0.1694	0.0495
rs57071950	LC_IGP_R3	IgG1_G2F/IgG1_G2	14	65788437	1	4.34E-09	1.03E-16	-	-0.266936	-0.4084	+	T	C	0.185662627	0.0452229	+	T	C	0.1694	0.0492
rs73278053	LC_IGP62	IgG1 FG0n total/G0n	14	65788490	1	2.99E-11	8.34E-10	-	-0.378775	-0.4082	+	G	A	0.107437799	0.0565844	+	G	A	0.0894	0.0665
rs73278053	LC_IGP63	IgG1 FG1n total/G1n	14	65788490	1	2.86E-09	4.71E-10	-	-0.339915	-0.4139	+	G	A	0.107437799	0.0569083	+	G	A	0.0894	0.0664
rs73278053	LC_IGP64	IgG1 FG2n total/G2n	14	65788490	1	1.53E-08	1.49E-10	-	-0.32431	-0.4233	+	G	A	0.107437799	0.0570252	+	G	A	0.0894	0.0661
rs73278053	LC_IGP61	IgG1 Fn total	14	65788490	1	5.08E-10	3.35E-10	-	-0.355337	-0.4178	+	G	A	0.107437799	0.056809	+	G	A	0.0894	0.0665
rs73278053	LC_IGP21	IgG1 Fucosylation	14	65788490	1	4.63E-10	1.23E-10	-	-0.352755	-0.4275	+	G	A	0.108757465	0.0562648	+	G	A	0.0894	0.0664
rs73278053	LC_IGP11	IgG1_G0	14	65788490	1	3.75E-12	2.46E-10	+	0.390607	0.4184	+	G	A	0.108371783	0.0558082	+	G	A	0.0894	0.0661
rs73278053	LC_IGP_R1	IgG1_G0F/IgG1_G0	14	65788490	1	6.81E-12	2.87E-09	-	-0.385733	-0.3937	+	G	A	0.108371783	0.0558032	+	G	A	0.0894	0.0663
rs73278053	LC_IGP_R4	IgG1_G0FN/IgG1_G0N	14	65788490	1	2.16E-08	7.52E-12	-	-0.316835	-0.457	+	G	A	0.108331052	0.0563073	+	G	A	0.0894	0.0667
rs73278053	LC_IGP52	IgG1_G0n	14	65788490	1	4.57E-12	1.37E-10	+	0.393719	0.4252	+	G	A	0.107437799	0.0564791	+	G	A	0.0894	0.0662
rs73278053	LC_IGP14	IgG1_G0N	14	65788490	1	8.50E-10	7.84E-10	+	0.346761	0.405	+	G	A	0.108331052	0.0561847	+	G	A	0.0894	0.0659
rs73278053	LC_IGP55	IgG1_G0Nn	14	65788490	1	5.94E-09	6.09E-10	+	0.332767	0.4081	+	G	A	0.107437799	0.0568834	+	G	A	0.0894	0.0659
rs73278053	LC_IGP12	IgG1_G1	14	65788490	1	3.38E-10	1.10E-09	+	0.353928	0.4048	+	G	A	0.10867722	0.0560071	+	G	A	0.0894	0.0664
rs73278053	LC_IGP_R2	IgG1_G1F/IgG1_G1	14	65788490	1	1.59E-10	2.00E-09	-	-0.360542	-0.3977	+	G	A	0.10867722	0.0560048	+	G	A	0.0894	0.0663
rs73278053	LC_IGP53	IgG1_G1n	14	65788490	1	1.08E-08	1.25E-09	+	0.327064	0.4032	+	G	A	0.107437799	0.0569096	+	G	A	0.0894	0.0664
rs73278053	LC_IGP_R3	IgG1_G2F/IgG1_G2	14	65788490	1	2.47E-09	1.21E-10	-	-0.337171	-0.4245	+	G	A	0.108132401	0.0562242	+	G	A	0.0894	0.0659
rs12893094	LC_IGP78	IgG1 BG0n/G0n	14	65789077	1	4.16E-12	8.02E-11	-	-0.241871	-0.2467	+	A	G	0.587980173	0.0346283	+	A	G	0.6089	0.038
rs12893094	LC_IGP79	IgG1 BG1n/G1n	14	65789077	1	5.55E-12	1.74E-12	-	-0.241018	-0.2674	+	A	G	0.587980173	0.0347149	+	A	G	0.6089	0.0379
rs12893094	LC_IGP77	IgG1 Bn	14	65789077	1	4.90E-12	6.01E-12	-	-0.241682	-0.262	+	A	G	0.587980173	0.0347192	+	A	G	0.6089	0.0381
rs12893094	LC_IGP86	IgG1 Bn/Fn total	14	65789077	1	1.09E-12	1.85E-12	-	-0.248869	-0.2684	+	A	G	0.587980173	0.0346799	+	A	G	0.6089	0.0381
rs12893094	LC_IGP84	IgG1 FBn/Bn total	14	65789077	1	2.55E-13	4.44E-18	+	0.256185	0.3327	+	A	G	0.587980173	0.0347196	+	A	G	0.6089	0.0384
rs12893094	LC_IGP62	IgG1 FG0n total/G0n	14	65789077	1	8.54E-14	3.27E-13	+	0.259748	0.2781	+	A	G	0.587980173	0.0345052	+	A	G	0.6089	0.0382
rs12893094	LC_IGP63	IgG1 FG1n total/G1n	14	65789077	1	1.08E-14	9.02E-16	+	0.269711	0.3068	+	A	G	0.587980173	0.0345632	+	A	G	0.6089	0.0382
rs12893094	LC_IGP64	IgG1 FG2n total/G2n	14	65789077	1	3.91E-11	4.57E-16	+	0.231356	0.3081	+	A	G	0.587980173	0.0347723	+	A	G	0.6089	0.0379
rs12893094	LC_IGP68	IgG1 FG2n/G2n	14	65789077	1	1.52E-08	4.02E-09	+	0.19845	0.2224	+	A	G	0.587980173	0.0348867	+	A	G	0.6089	0.0378
rs12893094	LC_IGP61	IgG1 Fn total	14	65789077	1	1.43E-14	3.19E-15	+	0.268265	0.301	+	A	G	0.587980173	0.034545	+	A	G	0.6089	0.0382
rs12893094	LC_IGP81	IgG1 Fn/Bn	14	65789077	1	3.95E-12	7.86E-11	+	0.242597	0.2466	+	A	G	0.587980173	0.0346956	+	A	G	0.6089	0.0379
rs12893094	LC_IGP21	IgG1 Fucosylation	14	65789077	1	4.19E-13	6.70E-15	+	0.252028	0.297	+	A	G	0.586621455	0.0344782	+	A	G	0.6089	0.0381
rs12893094	LC_IGP11	IgG1_G0	14	65789077	1	4.17E-12	1.86E-14	-	-0.239928	-0.291	+	A	G	0.586744044	0.0343551	+	A	G	0.6089	0.038
rs12893094	LC_IGP_R1	IgG1_G0F/IgG1_G0	14	65789077	1	1.08E-12	2.18E-12	+	0.246259	0.2673	+	A	G	0.586744044	0.0343118	+	A	G	0.6089	0.0381
rs12893094	LC_IGP_R4	IgG1_G0FN/IgG1_G0N	14	65789077	1	5.24E-13	2.52E-15	+	0.250679	0.3034	+	A	G	0.586956522	0.0344429	+	A	G	0.6089	0.0384
rs12893094	LC_IGP52	IgG1_G0n	14	65789077	1	3.28E-12	1.09E-14	-	-0.242553	-0.2942	+	A	G	0.587980173	0.034557	+	A	G	0.6089	0.0381
rs12893094	LC_IGP14	IgG1_G0N	14	65789077	1	7.11E-13	1.07E-11	-	-0.249195	-0.2572	+	A	G	0.586956522	0.0344406	+	A	G	0.6089	0.0378
rs12893094	LC_IGP55	IgG1_G0Nn	14	65789077	1	2.23E-12	7.65E-12	-	-0.2451	-0.2593	+	A	G	0.587980173	0.0346453	+	A	G	0.6089	0.0379
rs12893094	LC_IGP12	IgG1_G1	14	65789077	1	4.57E-13	3.77E-15	-	-0.251006	-0.2999	+	A	G	0.587049481	0.0343985	+	A	G	0.6089	0.0382

rs12893094	LC_IGP_R2	IgG1_G1F/IgG1_G1	14	65789077	1	1.17E-13	9.78E-15	+	0.257355	0.2947	+	A	G	0.587049481	0.0343844	+	A	G	0.6089	0.0381
rs12893094	LC_IGP_R5	IgG1_G1FN/IgG1_G1N	14	65789077	1	1.43E-13	3.32E-19	+	0.257706	0.342	+	A	G	0.587945879	0.0345586	+	A	G	0.6089	0.0382
rs12893094	LC_IGP53	IgG1_G1n	14	65789077	1	4.36E-13	4.99E-15	-	-0.252832	-0.2984	+	A	G	0.587980173	0.0346143	+	A	G	0.6089	0.0381
rs12893094	LC_IGP15	IgG1_G1N	14	65789077	1	1.11E-11	2.05E-11	-	-0.236932	-0.2543	+	A	G	0.587945879	0.0346315	+	A	G	0.6089	0.0379
rs12893094	LC_IGP56	IgG1_G1Nn	14	65789077	1	1.92E-11	3.52E-11	-	-0.234899	-0.2509	+	A	G	0.587980173	0.0347474	+	A	G	0.6089	0.0379
rs12893094	LC_IGP13	IgG1_G2	14	65789077	1	3.89E-09	4.80E-12	-	-0.204851	-0.2595	+	A	G	0.586744044	0.0345958	+	A	G	0.6089	0.0375
rs12893094	LC_IGP_R3	IgG1_G2F/IgG1_G2	14	65789077	1	7.86E-12	5.95E-16	+	0.237457	0.3063	+	A	G	0.586797066	0.0344568	+	A	G	0.6089	0.0379
rs12893094	LC_IGP_R8	IgG1_G2FS1/IgG1_G2S1	14	65789077	1	2.75E-08	3.02E-13	+	0.193241	0.2756	+	A	G	0.586438607	0.0346058	+	A	G	0.6089	0.0378
rs12893094	LC_IGP54	IgG1_G2n	14	65789077	1	1.98E-08	1.32E-11	-	-0.196831	-0.2533	+	A	G	0.587980173	0.0348858	+	A	G	0.6089	0.0374
rs7156013	LC_IGP62	IgG1 FG0n total/G0n	14	65789201	1	6.41E-09	4.50E-16	-	-0.260366	-0.3978	+	T	A	0.190766227	0.0446063	+	T	A	0.1768	0.049
rs7156013	LC_IGP63	IgG1 FG1n total/G1n	14	65789201	1	1.72E-08	1.09E-16	-	-0.253692	-0.4061	+	T	A	0.190766227	0.0447644	+	T	A	0.1768	0.049
rs7156013	LC_IGP61	IgG1 Fn total	14	65789201	1	6.01E-09	1.87E-16	-	-0.26143	-0.4032	+	T	A	0.190766227	0.0447047	+	T	A	0.1768	0.049
rs7156013	LC_IGP21	IgG1 Fucosylation	14	65789201	1	5.60E-09	4.64E-17	-	-0.26058	-0.4106	+	T	A	0.192291426	0.0444683	+	T	A	0.1768	0.0489
rs7156013	LC_IGP11	IgG1_G0	14	65789201	1	1.24E-08	7.41E-17	+	0.253165	0.4061	+	T	A	0.192056623	0.0442366	+	T	A	0.1768	0.0487
rs7156013	LC_IGP_R1	IgG1_G0F/IgG1_G0	14	65789201	1	4.12E-09	1.81E-15	-	-0.261216	-0.3882	+	T	A	0.192056623	0.0441878	+	T	A	0.1768	0.0488
rs7156013	LC_IGP_R4	IgG1_G0FN/IgG1_G0N	14	65789201	1	3.42E-08	3.90E-18	-	-0.246322	-0.427	+	T	A	0.191914692	0.0444217	+	T	A	0.1768	0.0492
rs7156013	LC_IGP52	IgG1_G0n	14	65789201	1	3.20E-08	1.92E-17	+	0.247961	0.4146	+	T	A	0.190766227	0.0446173	+	T	A	0.1768	0.0488
rs7156013	LC_IGP14	IgG1_G0N	14	65789201	1	1.31E-08	1.30E-16	+	0.253592	0.4015	+	T	A	0.191914692	0.0443854	+	T	A	0.1768	0.0485
rs7156013	LC_IGP12	IgG1_G1	14	65789201	1	1.02E-08	3.03E-15	+	0.255067	0.386	+	T	A	0.192056623	0.0443099	+	T	A	0.1768	0.0489
rs7156013	LC_IGP_R2	IgG1_G1F/IgG1_G1	14	65789201	1	3.73E-09	9.12E-16	-	-0.262633	-0.3926	+	T	A	0.192056623	0.0443014	+	T	A	0.1768	0.0488
rs7156013	LC_IGP_R3	IgG1_G2F/IgG1_G2	14	65789201	1	4.24E-08	4.42E-15	-	-0.244494	-0.381	+	T	A	0.191868394	0.0443992	+	T	A	0.1768	0.0486
rs10138151	LC_IGP62	IgG1 FG0n total/G0n	14	65789729	1	3.19E-11	3.77E-10	-	-0.378459	-0.4184	+	T	C	0.107423482	0.0566183	+	T	C	0.089	0.0668
rs10138151	LC_IGP63	IgG1 FG1n total/G1n	14	65789729	1	3.03E-09	2.91E-10	-	-0.339578	-0.421	+	T	C	0.107423482	0.0569421	+	T	C	0.089	0.0668
rs10138151	LC_IGP64	IgG1 FG2n total/G2n	14	65789729	1	1.58E-08	1.16E-10	-	-0.3242	-0.428	+	T	C	0.107423482	0.0570582	+	T	C	0.089	0.0664
rs10138151	LC_IGP61	IgG1 Fn total	14	65789729	1	5.40E-10	1.92E-10	-	-0.354996	-0.4256	+	T	C	0.107423482	0.0568429	+	T	C	0.089	0.0668
rs10138151	LC_IGP21	IgG1 Fucosylation	14	65789729	1	4.97E-10	7.29E-11	-	-0.352331	-0.435	+	T	C	0.108743218	0.0562981	+	T	C	0.089	0.0668
rs10138151	LC_IGP11	IgG1_G0	14	65789729	1	3.86E-12	1.04E-10	+	0.390589	0.4291	+	T	C	0.108357666	0.0558397	+	T	C	0.089	0.0664
rs10138151	LC_IGP_R1	IgG1_G0F/IgG1_G0	14	65789729	1	7.12E-12	1.37E-09	-	-0.385585	-0.4034	+	T	C	0.108357666	0.0558352	+	T	C	0.089	0.0666
rs10138151	LC_IGP_R4	IgG1_G0FN/IgG1_G0N	14	65789729	1	2.34E-08	2.75E-12	-	-0.316227	-0.4686	+	T	C	0.108316901	0.0563408	+	T	C	0.089	0.067
rs10138151	LC_IGP52	IgG1_G0n	14	65789729	1	4.72E-12	5.75E-11	+	0.393681	0.436	+	T	C	0.107423482	0.0565118	+	T	C	0.089	0.0666
rs10138151	LC_IGP14	IgG1_G0N	14	65789729	1	9.16E-10	2.35E-10	+	0.346288	0.4191	+	T	C	0.108316901	0.056218	+	T	C	0.089	0.0661
rs10138151	LC_IGP55	IgG1_G0Nn	14	65789729	1	6.43E-09	1.87E-10	+	0.332202	0.4219	+	T	C	0.107423482	0.056918	+	T	C	0.089	0.0662
rs10138151	LC_IGP12	IgG1_G1	14	65789729	1	3.50E-10	7.88E-10	+	0.353813	0.4105	+	T	C	0.108663103	0.0560388	+	T	C	0.089	0.0668
rs10138151	LC_IGP_R2	IgG1_G1F/IgG1_G1	14	65789729	1	1.64E-10	1.33E-09	-	-0.360454	-0.4041	+	T	C	0.108663103	0.0560365	+	T	C	0.089	0.0666
rs10138151	LC_IGP53	IgG1_G1n	14	65789729	1	1.12E-08	9.25E-10	+	0.326907	0.4086	+	T	C	0.107423482	0.0569427	+	T	C	0.089	0.0667
rs10138151	LC_IGP_R3	IgG1_G2F/IgG1_G2	14	65789729	1	2.51E-09	9.44E-11	-	-0.337197	-0.4292	+	T	C	0.108118276	0.0562556	+	T	C	0.089	0.0663
rs28557275	LC_IGP62	IgG1 FG0n total/G0n	14	65790415	1	3.34E-11	7.64E-10	-	-0.37834	-0.4103	+	G	A	0.10736294	0.0566603	+	G	A	0.0893	0.0667
rs28557275	LC_IGP63	IgG1 FG1n total/G1n	14	65790415	1	3.20E-09	4.24E-10	-	-0.339304	-0.4162	+	G	A	0.10736294	0.0569846	+	G	A	0.0893	0.0666
rs28557275	LC_IGP64	IgG1 FG2n total/G2n	14	65790415	1	1.63E-08	1.35E-10	-	-0.324094	-0.4255	+	G	A	0.10736294	0.0571	+	G	A	0.0893	0.0663
rs28557275	LC_IGP61	IgG1 Fn total	14	65790415	1	5.69E-10	3.04E-10	-	-0.354794	-0.42	+	G	A	0.10736294	0.0568852	+	G	A	0.0893	0.0667
rs28557275	LC_IGP21	IgG1 Fucosylation	14	65790415	1	5.27E-10	1.11E-10	-	-0.35207	-0.4298	+	G	A	0.108682973	0.0563393	+	G	A	0.0893	0.0666
rs28557275	LC_IGP11	IgG1_G0	14	65790415	1	4.06E-12	2.19E-10	+	0.390454	0.4209	+	G	A	0.108297974	0.0558802	+	G	A	0.0893	0.0663
rs28557275	LC_IGP_R1	IgG1_G0F/IgG1_G0	14	65790415	1	7.46E-12	2.63E-09	-	-0.385487	-0.3958	+	G	A	0.108297974	0.0558755	+	G	A	0.0893	0.0665
rs28557275	LC_IGP_R4	IgG1_G0FN/IgG1_G0N	14	65790415	1	2.40E-08	6.93E-12	-	-0.316193	-0.4592	+	G	A	0.108257063	0.0563809	+	G	A	0.0893	0.0669
rs28557275	LC_IGP52	IgG1_G0n	14	65790415	1	4.98E-12	1.22E-10	+	0.393535	0.4277	+	G	A	0.10736294	0.0565539	+	G	A	0.0893	0.0664
rs28557275	LC_IGP14	IgG1_G0N	14	65790415	1	9.51E-10	7.03E-10	+	0.346196	0.4073	+	G	A	0.108257063	0.0562584	+	G	A	0.0893	0.0661
rs28557275	LC_IGP55	IgG1_G0Nn	14	65790415	1	6.71E-09	5.50E-10	+	0.332042	0.4104	+	G	A	0.10736294	0.05696	+	G	A	0.0893	0.0661
rs28557275	LC_IGP12	IgG1_G1	14	65790415	1	3.67E-10	1.01E-09	+	0.353645	0.4069	+	G	A	0.10860341	0.0560792	+	G	A	0.0893	0.0666
rs28557275	LC_IGP_R2	IgG1_G1F/IgG1_G1	14	65790415	1	1.74E-10	1.80E-09	-	-0.360239	-0.4	+	G	A	0.10860341	0.0560771	+	G	A	0.0893	0.0665
rs28557275	LC_IGP53	IgG1_G1n	14	65790415	1	1.18E-08	1.15E-09	+	0.326696	0.4052	+	G	A	0.10736294	0.0569848	+	G	A	0.0893	0.0666
rs28557275	LC_IGP_R3	IgG1_G2F/IgG1_G2	14	65790415	1	2.60E-09	1.10E-10	-	-0.337113	-0.4267	+	G	A	0.108058548	0.0562959	+	G	A	0.0893	0.0661
rs10150168	LC_IGP62	IgG1 FG0n total/G0n	14	65791249	1	3.31E-11	6.89E-10	-	-0.378262	-0.4114	+	A	G	0.107415294	0.0566376	+	A	G	0.0887	0.0667
rs10150168	LC_IGP63	IgG1 FG1n total/G1n	14	65791249	1	3.13E-09	4.25E-10	-	-0.339363	-0.4163	+	A	G	0.107415294	0.0569614	+	A	G	0.0887	0.0667
rs10150168	LC_IGP64	IgG1 FG2n total/G2n	14	65791249	1	1.61E-08	1.38E-10	-	-0.324115	-0.4254	+	A	G	0.107415294	0.0570769	+	A	G	0.0887	0.0663
rs10150168	LC_IGP61	IgG1 Fn total	14	65791249	1	5.61E-10	2.94E-10	-	-0.354779	-0.4204	+	A	G	0.107415294	0.0568622	+	A	G	0.0887	0.0667
rs10150168	LC_IGP21	IgG1 Fucosylation	14	65791249	1	5.19E-10	1.14E-10	-	-0.352062	-0.4296	+	A	G	0.108735069	0.0563171	+	A	G	0.0887	0.0666
rs10150168	LC_IGP11	IgG1_G0	14	65791249	1	3.93E-12	1.85E-10	+	0.390567	0.4227	+	A	G	0.108349593	0.0558576	+	A	G	0.0887	0.0663
rs10150168	LC_IGP_R1	IgG1_G0F/IgG1_G0	14	65791249	1	7.32E-12	2.38E-09	-	-0.385488	-0.3969	+	A	G	0.108349593	0.0558534	+	A	G	0.0887	0.0665
rs10150168	LC_IGP_R4	IgG1_G0FN/IgG1_G0N	14	65791249	1	2.45E-08	5.73E-12	-	-0.315855	-0.4611	+	A	G	0.108308808	0.0563599	+	A	G	0.0887	0.067
rs10150168	LC_IGP52	IgG1_G0n	14	65791249	1	4.82E-12	1.04E-10	+	0.393647	0.4294	+	A	G	0.107415294	0.0565304	+	A	G	0.0887	0.0665
rs10150168	LC_IGP14	IgG1_G0N	14	65791249	1	9.58E-10	4.81E-10	+	0.345997	0.4114	+	A	G	0.108308808	0.0562369	+	A	G	0.0887	0.0661
rs10150168	LC_IGP55	IgG1_G0Nn	14	65791249	1	6.75E-09	3.83E-10	+	0.331856	0.4142	+	A	G	0.107415294	0.0569377	+	A	G	0.0887	0.0662
rs10150168	LC_IGP12	IgG1_G1	14	65791249	1	3.58E-10	1.11E-09	+	0.353731	0.406	+	A	G	0.108655029	0.0560569	+	A	G	0.0887	0.0666
rs10150168	LC_IGP_R2	IgG1_G1F/IgG1_G1	14	65791249	1	1.68E-10	1.86E-09	-	-0.360387	-0.3997	+	A	G	0.108655029	0.0560545	+	A	G	0.0887	0.0665
rs10150168	LC_IGP53	IgG1_G1n	14	65791249	1	1.15E-08	1.29E-09	+	0.326801	0.4041	+	A	G	0.107415294	0.0569615	+	A	G	0.0887	0.0666
rs10150168	LC_IGP_R3	IgG1_G2F/IgG1_G2	14	65791249	1	2.54E-09	1.12E-10	-	-0.337194	-0.4267	+	A	G	0.108110198	0.0562734	+	A	G	0.0887	0.0662
rs899959	LC_IGP78	IgG1 BG0n/G0n	14	65791928	1	1.48E-12	3.14E-10	-	-0.248015	-0.2435	+	G	A	0.591212515	0.0347716	+	G	A	0.6087	0.0387
rs899959	LC_IGP79	IgG1 BG1n/G1n	14	65791928	1	4.81E-12	6.48E-12	-	-0.242878	-0.2654	+	G	A	0.591212515	0.0348774	+	G	A	0.6087	0.0386
rs899959	LC_IGP32	IgG1 BG1S1/(BG1+BG1S1)	14	65791928	1	4.66E-08	1.56E-11	+	0.190842	0.2548	+	G	A	0.5910709	0.0347627	+	G	A	0.6087	0.0378
rs899959	LC_IGP77	IgG1 Bn	14	65791928	1	3.06E-12	2.40E-11	-	-0.245137	-0.2594	+	G	A	0.591212515	0.0348748	+	G	A	0.6087	0.0388
rs899959	LC_IGP86	IgG1 Bn/Fn total	14	65791928	1	6.41E-13	8.09E-12	-	-0.252571	-0.2656	+	G	A	0.591212515	0.0348341	+	G	A	0.6087	0.0388
rs899959	LC_IGP84	IgG1 FBn/Bn total	14	65791928	1	6.12E-14	1.05E-17	+	0.263945	0.3352	+	G	A	0.591212515	0.0348549	+	G	A	0.6087	0.0391
rs899959	LC_IGP62	IgG1 FG0n total/G0n	14	65791928	1	2.30E-14	1.38E-12	+	0.266858	0.2758	+	G	A	0.591212515	0.0346421	+	G	A	0.6087	0.0389
rs899959	LC_IGP63	IgG1 FG1n total/G1n	14	65791928	1	3.76E-15	3.95E-15	+	0.275556	0.3054	+	G	A	0.591212515	0.0347058	+	G	A	0.6087	0.0389
rs899959	LC_IGP64	IgG1 FG2n total/G2n	14	65791928	1	1.29E-11	1.34E-15	+	0.238109	0.3087	+	G	A	0.591212515	0.0349146	+	G	A	0.6087	0.0386
rs899959	LC_IGP68	IgG1 FG2n/G2n	14	65791928	1	2.16E-08	1.85E-08	+	0.197292	0.2168	+	G	A	0.591212515	0.0350605	+	G	A	0.6087	0.0385
rs899959	LC_IGP61	IgG1 Fn total	14	65791928	1	4.45E-15	1.43E-14	+	0.274641	0.2995	+	G	A	0.591212515	0.034685	+	G	A	0.6087	0.0389
rs899959	LC_IGP81	IgG1 Fn/Bn	14	65791928	1	2.83E-12	3.18E-10	+	0.245375	0.2433	+	G	A	0.591212515	0.0348541	+	G	A	0.6087	0.0387
rs899959	LC_IGP21	IgG1 Fucosylation	14	65791928	1	1.53E-13	3.89E-14	+	0.257858	0.2938	+	G	A	0.589837855	0.034619	+	G	A	0.6087	0.0388
rs899959	LC_IGP28	IgG1 G1S1/(G1+G1S1)	14	65791928	1	1.64E-08	6.09E-10	+	0.197063	0.2308	+	G	A	0.590346789	0.0347272	+	G	A	0.6087	0.0373
rs899959	LC_IGP11	IgG1_G0	14	65791928	1	1.65E-12	5.25E-14	-	-0.245495	-0.2912	+	G	A	0.589930971	0.0344966	+	G	A	0.6087	0.0387
rs899959	LC_IGP_R1	IgG1_G0F/IgG1_G0	14	65791928	1	3.82E-13	9.41E-12	+	0.252246	0.2644	+	G	A	0.589930971	0.0344508	+	G	A	0.6087	0.0388
rs899959	LC_IGP_R4	IgG1_G0FN/IgG1_G0N	14	65791928	1	9.22E-14	7.45E-15	+	0.25984	0.304	+	G	A	0.590151255	0.0345683	+	G	A	0.6087	0.0391
rs899959	LC_IGP52	IgG1_G0n	14	65791928	1	1.29E-12	3.33E-14	-	-0.248202	-0.2941	+	G	A	0.591212515	0.0347021	+	G	A	0.6087	0.0388
rs899959	LC_IGP14	IgG1_G0N	14	65791928	1	3.10E-13	3.27E-11	-	-0.254229	-0.2558	+	G	A	0.590151255	0.0345849	+	G	A	0.6087	0.0386
rs899959	LC_IGP55	IgG1_G0Nn	14	65791928	1	1.03E-12	2.50E-11	-	-0.249962	-0.2576	+	G	A	0.591212515	0.0347943	+	G	A	0.6087	0.0386
rs899959	LC_IGP12	IgG1_G1	14	65791928	1	1.16E-13	1.39E-14	-	-0.258479	-0.2992	+	G	A	0.590236408	0.0345314	+	G	A	0.6087	0.0389
rs899959	LC_IGP_R2	IgG1_G1F/IgG1_G1	14	65791928	1	4.43E-14	4.94E-14	+	0.262916	0.2923	+	G	A	0.590236408	0.0345256	+	G	A	0.6087	0.0388
rs899959	LC_IGP_R5	IgG1_G1FN/IgG1_G1N	14	65791928	1	5.84E-14	6.09E-19	+	0.262998	0.3458	+	G	A	0.591154367	0.0347034	+	G	A	0.6087	0.0389
rs899959	LC_IGP53	IgG1_G1n	14	65791928	1	1.08E-13	2.02E-14	-	-0.260488	-0.2971	+	G	A	0.591212515	0.0347498	+	G	A	0.6087	0.0388
rs899959	LC_IGP15	IgG1_G1N	14	65791928	1	8.74E-12	6.70E-11	-	-0.239228	-0.2525	+	G	A	0.591154367	0.0347905	+	G	A	0.6087	0.0387
rs899959	LC_IGP56	IgG1_G1Nn	14	65791928	1	1.51E-11	1.18E-10	-	-0.237225	-0.2488	+	G	A	0.591212515	0.0349081	+	G	A	0.6087	0.0386
rs899959	LC_IGP_R32	IgG1_G1S1/IgG1_G1	14	65791928	1	2.09E-08	6.67E-10	+	0.195645	0.2305	+	G	A	0.590346789	0.0347374	+	G	A	0.6087	0.0373
rs899959	LC_IGP13	IgG1_G2	14	65791928	1	1.23E-09	1.27E-11	-	-0.212282	-0.2588	+	G	A	0.589930971	0.0347337	+	G	A	0.6087	0.0382
rs899959	LC_IGP_R3	IgG1_G2F/IgG1_G2	14	65791928	1	3.14E-12	1.82E-15	+	0.243044	0.3066	+	G	A	0.589985941	0.0345989	+	G	A	0.6087	0.0386
rs899959	LC_IGP_R8	IgG1_G2FS1/IgG1_G2S1	14	65791928	1	1.65E-08	5.61E-13	+	0.1972	0.2772	+	G	A	0.589625535	0.0347565	+	G	A	0.6087	0.0384
rs899959	LC_IGP54	IgG1_G2n	14	65791928	1	6.61E-09	3.59E-11	-	-0.204285	-0.2522	+	G	A	0.591212515	0.035029	+	G	A	0.6087	0.0381
rs28595734	LC_IGP62	IgG1 FG0n total/G0n	14	65792269	1	3.40E-11	6.84E-10	-	-0.378113	-0.4115	+	C	A	0.107409718	0.0566497	+	C	A	0.0887	0.0667
rs28595734	LC_IGP63	IgG1 FG1n total/G1n	14	65792269	1	3.21E-09	4.22E-10	-	-0.339207	-0.4164	+	C	A	0.107409718	0.0569735	+	C	A	0.0887	0.0667
rs28595734	LC_IGP64	IgG1 FG2n total/G2n	14	65792269	1	1.63E-08	1.37E-10	-	-0.324049	-0.4256	+	C	A	0.107409718	0.0570887	+	C	A	0.0887	0.0663
rs28595734	LC_IGP61	IgG1 Fn total	14	65792269	1	5.75E-10	2.92E-10	-	-0.35462	-0.4205	+	C	A	0.107409718	0.0568743	+	C	A	0.0887	0.0667
rs28595734	LC_IGP21	IgG1 Fucosylation	14	65792269	1	5.35E-10	1.13E-10	-	-0.351869	-0.4298	+	C	A	0.108729521	0.056329	+	C	A	0.0887	0.0666
rs28595734	LC_IGP11	IgG1_G0	14	65792269	1	3.98E-12	1.82E-10	+	0.390539	0.4229	+	C	A	0.108344095	0.0558688	+	C	A	0.0887	0.0663
rs28595734	LC_IGP_R1	IgG1_G0F/IgG1_G0	14	65792269	1	7.47E-12	2.36E-09	-	-0.385406	-0.397	+	C	A	0.108344095	0.0558648	+	C	A	0.0887	0.0665
rs28595734	LC_IGP_R4	IgG1_G0FN/IgG1_G0N	14	65792269	1	2.53E-08	5.71E-12	-	-0.315597	-0.4612	+	C	A	0.108303297	0.0563719	+	C	A	0.0887	0.067
rs28595734	LC_IGP52	IgG1_G0n	14	65792269	1	4.89E-12	1.02E-10	+	0.39361	0.4296	+	C	A	0.107409718	0.0565421	+	C	A	0.0887	0.0665
rs28595734	LC_IGP14	IgG1_G0N	14	65792269	1	9.87E-10	4.78E-10	+	0.345793	0.4115	+	C	A	0.108303297	0.0562488	+	C	A	0.0887	0.0661
rs28595734	LC_IGP55	IgG1_G0Nn	14	65792269	1	6.97E-09	3.81E-10	+	0.331617	0.4144	+	C	A	0.107409718	0.0569501	+	C	A	0.0887	0.0662
rs28595734	LC_IGP12	IgG1_G1	14	65792269	1	3.64E-10	1.11E-09	+	0.353661	0.4061	+	C	A	0.108649531	0.0560682	+	C	A	0.0887	0.0666
rs28595734	LC_IGP_R2	IgG1_G1F/IgG1_G1	14	65792269	1	1.70E-10	1.85E-09	-	-0.360329	-0.3999	+	C	A	0.108649531	0.0560657	+	C	A	0.0887	0.0665
rs28595734	LC_IGP53	IgG1_G1n	14	65792269	1	1.17E-08	1.29E-09	+	0.326714	0.4042	+	C	A	0.107409718	0.0569733	+	C	A	0.0887	0.0666
rs28595734	LC_IGP_R3	IgG1_G2F/IgG1_G2	14	65792269	1	2.57E-09	1.11E-10	-	-0.337182	-0.4268	+	C	A	0.108104697	0.0562845	+	C	A	0.0887	0.0662
rs11627598	LC_IGP78	IgG1 BG0n/G0n	14	65797766	1	5.28E-12	7.75E-11	-	-0.2417	-0.2471	+	T	G	0.588060409	0.0347764	+	T	G	0.6088	0.038
rs11627598	LC_IGP79	IgG1 BG1n/G1n	14	65797766	1	8.32E-12	1.68E-12	-	-0.240015	-0.2679	+	T	G	0.588060409	0.0348669	+	T	G	0.6088	0.038
rs11627598	LC_IGP77	IgG1 Bn	14	65797766	1	6.59E-12	5.69E-12	-	-0.241221	-0.2625	+	T	G	0.588060409	0.0348689	+	T	G	0.6088	0.0381
rs11627598	LC_IGP86	IgG1 Bn/Fn total	14	65797766	1	1.48E-12	1.74E-12	-	-0.248434	-0.269	+	T	G	0.588060409	0.0348297	+	T	G	0.6088	0.0381
rs11627598	LC_IGP84	IgG1 FBn/Bn total	14	65797766	1	3.62E-13	4.60E-18	+	0.255601	0.3328	+	T	G	0.588060409	0.0348705	+	T	G	0.6088	0.0384
rs11627598	LC_IGP62	IgG1 FG0n total/G0n	14	65797766	1	1.31E-13	3.11E-13	+	0.258884	0.2786	+	T	G	0.588060409	0.0346567	+	T	G	0.6088	0.0382
rs11627598	LC_IGP63	IgG1 FG1n total/G1n	14	65797766	1	1.69E-14	8.79E-16	+	0.268834	0.3071	+	T	G	0.588060409	0.0347156	+	T	G	0.6088	0.0382

rs11627598	LC_IGP64	IgG1 FG2n total/G2n	14	65797766	1	5.84E-11	4.49E-16	+	0.23024	0.3085	+	T	G	0.588060409	0.0349245	+	T	G	0.6088	0.038
rs11627598	LC_IGP68	IgG1 FG2n/G2n	14	65797766	1	2.18E-08	3.67E-09	+	0.197111	0.2231	+	T	G	0.588060409	0.0350384	+	T	G	0.6088	0.0378
rs11627598	LC_IGP61	IgG1 Fn total	14	65797766	1	2.43E-14	3.12E-15	+	0.26705	0.3014	+	T	G	0.588060409	0.0346988	+	T	G	0.6088	0.0382
rs11627598	LC_IGP81	IgG1 Fn/Bn	14	65797766	1	5.22E-12	7.35E-11	+	0.242232	0.2472	+	T	G	0.588060409	0.0348448	+	T	G	0.6088	0.038
rs11627598	LC_IGP21	IgG1 Fucosylation	14	65797766	1	8.48E-13	6.82E-15	+	0.2498	0.2971	+	T	G	0.586741677	0.0346405	+	T	G	0.6088	0.0382
rs11627598	LC_IGP28	IgG1 G1S1/(G1+G1S1)	14	65797766	1	4.91E-08	1.85E-10	+	0.190326	0.2337	+	T	G	0.587235168	0.0347287	+	T	G	0.6088	0.0367
rs11627598	LC_IGP11	IgG1_G0	14	65797766	1	5.95E-12	1.45E-14	-	-0.239219	-0.2923	+	T	G	0.586862553	0.0345086	+	T	G	0.6088	0.038
rs11627598	LC_IGP_R1	IgG1_G0F/IgG1_G0	14	65797766	1	1.72E-12	2.05E-12	+	0.245084	0.2678	+	T	G	0.586862553	0.0344675	+	T	G	0.6088	0.0381
rs11627598	LC_IGP_R4	IgG1_G0FN/IgG1_G0N	14	65797766	1	7.76E-13	2.56E-15	+	0.249906	0.3036	+	T	G	0.587075321	0.034598	+	T	G	0.6088	0.0384
rs11627598	LC_IGP52	IgG1_G0n	14	65797766	1	4.37E-12	8.65E-15	-	-0.242164	-0.2954	+	T	G	0.588060409	0.0347058	+	T	G	0.6088	0.0381
rs11627598	LC_IGP14	IgG1_G0N	14	65797766	1	8.83E-13	8.69E-12	-	-0.249238	-0.2584	+	T	G	0.587075321	0.034592	+	T	G	0.6088	0.0379
rs11627598	LC_IGP55	IgG1_G0Nn	14	65797766	1	2.65E-12	6.34E-12	-	-0.245273	-0.2604	+	T	G	0.588060409	0.0347921	+	T	G	0.6088	0.0379
rs11627598	LC_IGP12	IgG1_G1	14	65797766	1	6.56E-13	3.76E-15	-	-0.250345	-0.3002	+	T	G	0.587127978	0.0345464	+	T	G	0.6088	0.0382
rs11627598	LC_IGP_R2	IgG1_G1F/IgG1_G1	14	65797766	1	1.67E-13	9.38E-15	+	0.256772	0.2952	+	T	G	0.587127978	0.0345322	+	T	G	0.6088	0.0381
rs11627598	LC_IGP_R5	IgG1_G1FN/IgG1_G1N	14	65797766	1	2.59E-13	3.42E-19	+	0.256084	0.3422	+	T	G	0.588065806	0.0347191	+	T	G	0.6088	0.0382
rs11627598	LC_IGP53	IgG1_G1n	14	65797766	1	6.36E-13	5.09E-15	-	-0.25211	-0.2985	+	T	G	0.588060409	0.0347652	+	T	G	0.6088	0.0382
rs11627598	LC_IGP15	IgG1_G1N	14	65797766	1	1.82E-11	1.99E-11	-	-0.235461	-0.2547	+	T	G	0.588065806	0.0347902	+	T	G	0.6088	0.038
rs11627598	LC_IGP56	IgG1_G1Nn	14	65797766	1	2.87E-11	3.52E-11	-	-0.233832	-0.2512	+	T	G	0.588060409	0.0348995	+	T	G	0.6088	0.0379
rs11627598	LC_IGP13	IgG1_G2	14	65797766	1	7.68E-09	5.22E-12	-	-0.201796	-0.2592	+	T	G	0.586862553	0.0347571	+	T	G	0.6088	0.0376
rs11627598	LC_IGP_R3	IgG1_G2F/IgG1_G2	14	65797766	1	1.34E-11	5.88E-16	+	0.235831	0.3067	+	T	G	0.586916259	0.0346145	+	T	G	0.6088	0.0379
rs11627598	LC_IGP_R8	IgG1_G2FS1/IgG1_G2S1	14	65797766	1	4.55E-08	2.93E-13	+	0.190989	0.2759	+	T	G	0.586557728	0.0347632	+	T	G	0.6088	0.0378
rs11627598	LC_IGP54	IgG1_G2n	14	65797766	1	3.45E-08	1.46E-11	-	-0.194263	-0.253	+	T	G	0.588060409	0.0350416	+	T	G	0.6088	0.0375
rs9635250	LC_IGP62	IgG1 FG0n total/G0n	14	65799348	1	6.88E-11	6.35E-10	-	-0.373642	-0.4133	+	T	A	0.107454748	0.0568911	+	T	A	0.0887	0.0669
rs9635250	LC_IGP63	IgG1 FG1n total/G1n	14	65799348	1	5.21E-09	4.10E-10	-	-0.335947	-0.4178	+	T	A	0.107454748	0.0572085	+	T	A	0.0887	0.0669
rs9635250	LC_IGP64	IgG1 FG2n total/G2n	14	65799348	1	1.90E-08	1.30E-10	-	-0.323764	-0.4272	+	T	A	0.107454748	0.0573128	+	T	A	0.0887	0.0665
rs9635250	LC_IGP61	IgG1 Fn total	14	65799348	1	1.03E-09	2.78E-10	-	-0.350727	-0.4221	+	T	A	0.107454748	0.0571124	+	T	A	0.0887	0.0669
rs9635250	LC_IGP21	IgG1 Fucosylation	14	65799348	1	8.99E-10	1.07E-10	-	-0.348573	-0.4314	+	T	A	0.10877312	0.0565593	+	T	A	0.0887	0.0668
rs9635250	LC_IGP11	IgG1_G0	14	65799348	1	6.15E-12	1.56E-10	+	0.388563	0.4257	+	T	A	0.10838821	0.0560925	+	T	A	0.0887	0.0665
rs9635250	LC_IGP_R1	IgG1_G0F/IgG1_G0	14	65799348	1	1.51E-11	2.17E-09	-	-0.381182	-0.3988	+	T	A	0.10838821	0.0560976	+	T	A	0.0887	0.0666
rs9635250	LC_IGP_R4	IgG1_G0FN/IgG1_G0N	14	65799348	1	4.43E-08	5.48E-12	-	-0.311252	-0.4628	+	T	A	0.10834752	0.0566022	+	T	A	0.0887	0.0672
rs9635250	LC_IGP52	IgG1_G0n	14	65799348	1	7.90E-12	8.82E-11	+	0.391259	0.4324	+	T	A	0.107454748	0.0567753	+	T	A	0.0887	0.0667
rs9635250	LC_IGP14	IgG1_G0N	14	65799348	1	1.47E-09	4.21E-10	+	0.343512	0.4142	+	T	A	0.10834752	0.0564733	+	T	A	0.0887	0.0663
rs9635250	LC_IGP55	IgG1_G0Nn	14	65799348	1	1.08E-08	3.39E-10	+	0.328707	0.4169	+	T	A	0.107454748	0.0571833	+	T	A	0.0887	0.0664
rs9635250	LC_IGP12	IgG1_G1	14	65799348	1	5.52E-10	1.10E-09	+	0.351333	0.4071	+	T	A	0.108693647	0.0562917	+	T	A	0.0887	0.0668
rs9635250	LC_IGP_R2	IgG1_G1F/IgG1_G1	14	65799348	1	2.46E-10	1.77E-09	-	-0.358535	-0.4014	+	T	A	0.108693647	0.0562875	+	T	A	0.0887	0.0667
rs9635250	LC_IGP53	IgG1_G1n	14	65799348	1	1.78E-08	1.29E-09	+	0.323813	0.4052	+	T	A	0.107454748	0.0572063	+	T	A	0.0887	0.0668
rs9635250	LC_IGP_R3	IgG1_G2F/IgG1_G2	14	65799348	1	2.89E-09	1.04E-10	-	-0.337359	-0.4286	+	T	A	0.108147922	0.0565	+	T	A	0.0887	0.0663
rs12880725	LC_IGP78	IgG1 BG0n/G0n	14	65799805	1	5.16E-12	7.32E-11	-	-0.242709	-0.2474	+	A	G	0.588726146	0.0349043	+	A	G	0.6088	0.038
rs12880725	LC_IGP79	IgG1 BG1n/G1n	14	65799805	1	6.99E-12	1.51E-12	-	-0.24177	-0.2685	+	A	G	0.588726146	0.0349919	+	A	G	0.6088	0.038
rs12880725	LC_IGP77	IgG1 Bn	14	65799805	1	5.89E-12	5.11E-12	-	-0.242669	-0.2632	+	A	G	0.588726146	0.0349953	+	A	G	0.6088	0.0381
rs12880725	LC_IGP86	IgG1 Bn/Fn total	14	65799805	1	1.28E-12	1.55E-12	-	-0.250059	-0.2696	+	A	G	0.588726146	0.0349552	+	A	G	0.6088	0.0381
rs12880725	LC_IGP84	IgG1 FBn/Bn total	14	65799805	1	3.35E-13	4.67E-18	+	0.256914	0.3327	+	A	G	0.588726146	0.0349976	+	A	G	0.6088	0.0384
rs12880725	LC_IGP62	IgG1 FG0n total/G0n	14	65799805	1	1.14E-13	2.90E-13	+	0.260468	0.2789	+	A	G	0.588726146	0.0347818	+	A	G	0.6088	0.0382
rs12880725	LC_IGP63	IgG1 FG1n total/G1n	14	65799805	1	1.31E-14	8.00E-16	+	0.270973	0.3076	+	A	G	0.588726146	0.0348383	+	A	G	0.6088	0.0382
rs12880725	LC_IGP64	IgG1 FG2n total/G2n	14	65799805	1	4.42E-11	4.06E-16	+	0.232542	0.309	+	A	G	0.588726146	0.0350475	+	A	G	0.6088	0.038
rs12880725	LC_IGP68	IgG1 FG2n/G2n	14	65799805	1	1.54E-08	3.04E-09	+	0.199919	0.2243	+	A	G	0.588726146	0.0351605	+	A	G	0.6088	0.0378
rs12880725	LC_IGP61	IgG1 Fn total	14	65799805	1	2.16E-14	2.90E-15	+	0.268565	0.3018	+	A	G	0.588726146	0.0348245	+	A	G	0.6088	0.0382
rs12880725	LC_IGP81	IgG1 Fn/Bn	14	65799805	1	4.47E-12	6.53E-11	+	0.243896	0.2479	+	A	G	0.588726146	0.0349702	+	A	G	0.6088	0.038
rs12880725	LC_IGP21	IgG1 Fucosylation	14	65799805	1	9.81E-13	6.68E-15	+	0.250103	0.2973	+	A	G	0.587494143	0.0347816	+	A	G	0.6088	0.0382
rs12880725	LC_IGP28	IgG1 G1S1/(G1+G1S1)	14	65799805	1	4.10E-08	1.72E-10	+	0.192132	0.2342	+	A	G	0.587892966	0.0348516	+	A	G	0.6088	0.0367
rs12880725	LC_IGP11	IgG1_G0	14	65799805	1	5.03E-12	1.09E-14	-	-0.240981	-0.2936	+	A	G	0.587607819	0.0346419	+	A	G	0.6088	0.038
rs12880725	LC_IGP_R1	IgG1_G0F/IgG1_G0	14	65799805	1	1.70E-12	1.88E-12	+	0.246105	0.2683	+	A	G	0.587607819	0.0346039	+	A	G	0.6088	0.0381
rs12880725	LC_IGP_R4	IgG1_G0FN/IgG1_G0N	14	65799805	1	9.79E-13	2.56E-15	+	0.249803	0.3036	+	A	G	0.587822413	0.0347403	+	A	G	0.6088	0.0384
rs12880725	LC_IGP52	IgG1_G0n	14	65799805	1	3.08E-12	6.71E-15	-	-0.244764	-0.2966	+	A	G	0.588726146	0.0348266	+	A	G	0.6088	0.0381
rs12880725	LC_IGP14	IgG1_G0N	14	65799805	1	8.63E-13	6.81E-12	-	-0.250338	-0.2598	+	A	G	0.587822413	0.034729	+	A	G	0.6088	0.0379
rs12880725	LC_IGP55	IgG1_G0Nn	14	65799805	1	2.30E-12	5.10E-12	-	-0.246856	-0.2616	+	A	G	0.588726146	0.0349176	+	A	G	0.6088	0.0379
rs12880725	LC_IGP12	IgG1_G1	14	65799805	1	5.10E-13	3.49E-15	-	-0.252434	-0.3006	+	A	G	0.587784667	0.0346672	+	A	G	0.6088	0.0382
rs12880725	LC_IGP_R2	IgG1_G1F/IgG1_G1	14	65799805	1	1.23E-13	8.40E-15	+	0.259107	0.2957	+	A	G	0.587784667	0.0346517	+	A	G	0.6088	0.0381
rs12880725	LC_IGP_R5	IgG1_G1FN/IgG1_G1N	14	65799805	1	2.86E-13	3.40E-19	+	0.256648	0.3422	+	A	G	0.588815806	0.0348598	+	A	G	0.6088	0.0382
rs12880725	LC_IGP53	IgG1_G1n	14	65799805	1	5.08E-13	4.91E-15	-	-0.254104	-0.2987	+	A	G	0.588726146	0.0348888	+	A	G	0.6088	0.0382
rs12880725	LC_IGP15	IgG1_G1N	14	65799805	1	1.97E-11	1.81E-11	-	-0.235985	-0.2552	+	A	G	0.588815806	0.0349309	+	A	G	0.6088	0.038
rs12880725	LC_IGP56	IgG1_G1Nn	14	65799805	1	2.53E-11	3.31E-11	-	-0.235335	-0.2516	+	A	G	0.588726146	0.0350257	+	A	G	0.6088	0.0379
rs12880725	LC_IGP13	IgG1_G2	14	65799805	1	1.09E-08	5.44E-12	-	-0.200523	-0.2591	+	A	G	0.587607819	0.0349021	+	A	G	0.6088	0.0376
rs12880725	LC_IGP_R3	IgG1_G2F/IgG1_G2	14	65799805	1	1.20E-11	5.36E-16	+	0.237323	0.3072	+	A	G	0.587662286	0.0347493	+	A	G	0.6088	0.0379
rs12880725	LC_IGP_R8	IgG1_G2FS1/IgG1_G2S1	14	65799805	1	4.63E-08	2.68E-13	+	0.191637	0.2764	+	A	G	0.587303299	0.034901	+	A	G	0.6088	0.0378
rs12880725	LC_IGP54	IgG1_G2n	14	65799805	1	4.06E-08	1.55E-11	-	-0.193988	-0.2527	+	A	G	0.588726146	0.0351745	+	A	G	0.6088	0.0375
rs12881755	LC_IGP84	IgG1 FBn/Bn total	14	65801811	1	2.14E-10	3.98E-09	+	0.233923	0.2404	+	G	A	0.661371747	0.0365985	+	G	A	0.6802	0.0408
rs12881755	LC_IGP63	IgG1 FG1n total/G1n	14	65801811	1	3.87E-12	1.37E-07	+	0.254726	0.2141	+	G	A	0.661371747	0.0364152	+	G	A	0.6802	0.0406
rs12881755	LC_IGP64	IgG1 FG2n total/G2n	14	65801811	1	7.69E-10	4.01E-09	+	0.226314	0.2377	+	G	A	0.661371747	0.0365703	+	G	A	0.6802	0.0404
rs12881755	LC_IGP61	IgG1 Fn total	14	65801811	1	9.67E-12	2.24E-07	+	0.249836	0.2105	+	G	A	0.661371747	0.0364099	+	G	A	0.6802	0.0406
rs12881755	LC_IGP21	IgG1 Fucosylation	14	65801811	1	2.61E-10	7.16E-07	+	0.231289	0.2012	+	G	A	0.660512947	0.0363641	+	G	A	0.6802	0.0406
rs12881755	LC_IGP11	IgG1_G0	14	65801811	1	1.33E-10	8.41E-07	-	-0.23357	-0.1989	+	G	A	0.660872938	0.0361273	+	G	A	0.6802	0.0404
rs12881755	LC_IGP_R4	IgG1_G0FN/IgG1_G0N	14	65801811	1	2.36E-09	3.30E-07	+	0.218254	0.2083	+	G	A	0.660960808	0.036348	+	G	A	0.6802	0.0408
rs12881755	LC_IGP52	IgG1_G0n	14	65801811	1	3.26E-11	9.51E-07	-	-0.242711	-0.1984	+	G	A	0.661371747	0.0363289	+	G	A	0.6802	0.0405
rs12881755	LC_IGP12	IgG1_G1	14	65801811	1	5.20E-11	9.10E-08	-	-0.239092	-0.217	+	G	A	0.660630727	0.0361738	+	G	A	0.6802	0.0406
rs12881755	LC_IGP_R2	IgG1_G1F/IgG1_G1	14	65801811	1	1.33E-11	4.87E-07	+	0.246387	0.2039	+	G	A	0.660630727	0.0361591	+	G	A	0.6802	0.0405
rs12881755	LC_IGP_R5	IgG1_G1FN/IgG1_G1N	14	65801811	1	1.73E-10	6.25E-10	+	0.234409	0.2513	+	G	A	0.661962177	0.0364851	+	G	A	0.6802	0.0406
rs12881755	LC_IGP53	IgG1_G1n	14	65801811	1	1.00E-10	1.49E-07	-	-0.237353	-0.2131	+	G	A	0.661371747	0.0364587	+	G	A	0.6802	0.0406
rs12881755	LC_IGP_R3	IgG1_G2F/IgG1_G2	14	65801811	1	3.15E-10	4.47E-09	+	0.229419	0.2365	+	G	A	0.660973105	0.0362411	+	G	A	0.6802	0.0403
rs72712500	LC_IGP84	IgG1 FBn/Bn total	14	65804328	1	4.10E-08	1.02E-06	-	-0.237905	-0.2505	+	A	G	0.207208666	0.043152	+	A	G	0.1722	0.0513
rs72712500	LC_IGP12	IgG1_G1	14	65804328	1	3.95E-08	7.98E-07	+	0.235528	0.2513	+	A	G	0.207350511	0.0426732	+	A	G	0.1722	0.0509
rs12878872	LC_IGP78	IgG1 BG0n/G0n	14	65804665	1	4.51E-13	2.06E-11	-	-0.254101	-0.2577	+	A	G	0.575292751	0.0348096	+	A	G	0.6141	0.0385
rs12878872	LC_IGP79	IgG1 BG1n/G1n	14	65804665	1	1.27E-12	4.70E-14	-	-0.24977	-0.2898	+	A	G	0.575292751	0.0349126	+	A	G	0.6141	0.0384
rs12878872	LC_IGP77	IgG1 Bn	14	65804665	1	1.58E-12	2.88E-13	-	-0.248796	-0.2818	+	A	G	0.575292751	0.0349241	+	A	G	0.6141	0.0386
rs12878872	LC_IGP86	IgG1 Bn/Fn total	14	65804665	1	2.41E-13	1.04E-13	-	-0.257602	-0.2872	+	A	G	0.575292751	0.0348767	+	A	G	0.6141	0.0386
rs12878872	LC_IGP84	IgG1 FBn/Bn total	14	65804665	1	1.16E-13	2.72E-20	+	0.261526	0.359	+	A	G	0.575292751	0.0349318	+	A	G	0.6141	0.0389
rs12878872	LC_IGP62	IgG1 FG0n total/G0n	14	65804665	1	1.19E-15	4.02E-14	+	0.280164	0.2926	+	A	G	0.575292751	0.0346422	+	A	G	0.6141	0.0387
rs12878872	LC_IGP63	IgG1 FG1n total/G1n	14	65804665	1	3.28E-16	1.53E-17	+	0.286381	0.3298	+	A	G	0.575292751	0.0347171	+	A	G	0.6141	0.0387
rs12878872	LC_IGP64	IgG1 FG2n total/G2n	14	65804665	1	1.13E-12	1.13E-18	+	0.250446	0.3394	+	A	G	0.575292751	0.0349264	+	A	G	0.6141	0.0385
rs12878872	LC_IGP68	IgG1 FG2n/G2n	14	65804665	1	7.58E-10	5.59E-10	+	0.217009	0.2378	+	A	G	0.575292751	0.0350533	+	A	G	0.6141	0.0384
rs12878872	LC_IGP61	IgG1 Fn total	14	65804665	1	1.09E-15	5.96E-17	+	0.281163	0.3239	+	A	G	0.575292751	0.0347181	+	A	G	0.6141	0.0387
rs12878872	LC_IGP81	IgG1 Fn/Bn	14	65804665	1	6.29E-13	4.25E-12	+	0.253038	0.2663	+	A	G	0.575292751	0.0348854	+	A	G	0.6141	0.0384
rs12878872	LC_IGP21	IgG1 Fucosylation	14	65804665	1	1.02E-13	4.12E-16	+	0.260613	0.3143	+	A	G	0.574595869	0.0347342	+	A	G	0.6141	0.0386
rs12878872	LC_IGP28	IgG1 G1S1/(G1+G1S1)	14	65804665	1	2.57E-10	9.63E-12	+	0.220818	0.2524	+	A	G	0.57463211	0.0347069	+	A	G	0.6141	0.037
rs12878872	LC_IGP11	IgG1_G0	14	65804665	1	8.61E-16	1.36E-15	-	-0.279834	-0.3076	+	A	G	0.574523519	0.034434	+	A	G	0.6141	0.0385
rs12878872	LC_IGP_R1	IgG1_G0F/IgG1_G0	14	65804665	1	7.61E-15	2.75E-13	+	0.270457	0.2819	+	A	G	0.574523519	0.0344637	+	A	G	0.6141	0.0386
rs12878872	LC_IGP_R4	IgG1_G0FN/IgG1_G0N	14	65804665	1	2.32E-13	2.46E-16	+	0.25665	0.3185	+	A	G	0.57470545	0.0347243	+	A	G	0.6141	0.0388
rs12878872	LC_IGP52	IgG1_G0n	14	65804665	1	5.90E-16	9.55E-16	-	-0.282891	-0.3098	+	A	G	0.575292751	0.0346024	+	A	G	0.6141	0.0386
rs12878872	LC_IGP14	IgG1_G0N	14	65804665	1	4.50E-15	1.92E-12	-	-0.274147	-0.27	+	A	G	0.57470545	0.0346332	+	A	G	0.6141	0.0384
rs12878872	LC_IGP55	IgG1_G0Nn	14	65804665	1	1.17E-14	1.65E-12	-	-0.270865	-0.2711	+	A	G	0.575292751	0.0347625	+	A	G	0.6141	0.0384
rs12878872	LC_IGP12	IgG1_G1	14	65804665	1	8.23E-15	7.61E-17	-	-0.270719	-0.3224	+	A	G	0.574540929	0.0345423	+	A	G	0.6141	0.0387
rs12878872	LC_IGP_R2	IgG1_G1F/IgG1_G1	14	65804665	1	8.44E-16	2.78E-16	+	0.280533	0.3157	+	A	G	0.574540929	0.0345091	+	A	G	0.6141	0.0386
rs12878872	LC_IGP_R5	IgG1_G1FN/IgG1_G1N	14	65804665	1	6.39E-14	7.64E-22	+	0.263278	0.3718	+	A	G	0.575642681	0.0347957	+	A	G	0.6141	0.0387
rs12878872	LC_IGP53	IgG1_G1n	14	65804665	1	2.07E-14	1.35E-16	-	-0.268383	-0.3195	+	A	G	0.575292751	0.0347779	+	A	G	0.6141	0.0386
rs12878872	LC_IGP15	IgG1_G1N	14	65804665	1	8.78E-12	6.24E-13	-	-0.239827	-0.2767	+	A	G	0.575642681	0.0348812	+	A	G	0.6141	0.0385
rs12878872	LC_IGP56	IgG1_G1Nn	14	65804665	1	1.17E-11	1.34E-12	-	-0.238954	-0.2723	+	A	G	0.575292751	0.0349661	+	A	G	0.6141	0.0384
rs12878872	LC_IGP_R32	IgG1_G1S1/IgG1_G1	14	65804665	1	3.23E-10	1.34E-11	+	0.219632	0.251	+	A	G	0.57463211	0.0347169	+	A	G	0.6141	0.0371
rs12878872	LC_IGP13	IgG1_G2	14	65804665	1	2.95E-08	3.66E-14	-	-0.194408	-0.2879	+	A	G	0.574523519	0.0348946	+	A	G	0.6141	0.038
rs12878872	LC_IGP_R3	IgG1_G2F/IgG1_G2	14	65804665	1	1.81E-13	2.15E-18	+	0.257147	0.3359	+	A	G	0.574569071	0.0346338	+	A	G	0.6141	0.0384
rs12878872	LC_IGP_R8	IgG1_G2FS1/IgG1_G2S1	14	65804665	1	6.80E-09	9.23E-16	+	0.202968	0.3076	+	A	G	0.574218082	0.0348344	+	A	G	0.6141	0.0383
rs12878872	LC_IGP_R26	IgG1_G2N/IgG1_G1N	14	65804665	1	3.93E-09	8.46E-11	+	0.206506	0.2439	+	A	G	0.575614198	0.0348836	+	A	G	0.6141	0.0376
rs747541	LC_IGP78	IgG1 BG0n/G0n	14	65805410	1	1.47E-12	4.32E-11	-	-0.247957	-0.2514	+	A	C	0.571962825	0.0347592	+	A	C	0.6026	0.0381
rs747541	LC_IGP79	IgG1 BG1n/G1n	14	65805410	1	2.04E-12	1.41E-13	-	-0.246953	-0.2818	+	A	C	0.571962825	0.0348469	+	A	C	0.6026	0.0381
rs747541	LC_IGP77	IgG1 Bn	14	65805410	1	3.13E-12	6.86E-13	-	-0.244941	-0.275	+	A	C	0.571962825	0.034863	+	A	C	0.6026	0.0383
rs747541	LC_IGP86	IgG1 Bn/Fn total	14	65805410	1	5.19E-13	2.38E-13	-	-0.253471	-0.2806	+	A	C	0.571962825	0.0348173	+	A	C	0.6026	0.0383
rs747541	LC_IGP84	IgG1 FBn/Bn total	14	65805410	1	5.98E-13	1.70E-19	+	0.25332	0.3485	+	A	C	0.571962825	0.034891	+	A	C	0.6026	0.0386
rs747541	LC_IGP62	IgG1 FG0n total/G0n	14	65805410	1	6.57E-15	7.60E-14	+	0.27225	0.2869	+	A	C	0.571962825	0.0346033	+	A	C	0.6026	0.0384
rs747541	LC_IGP63	IgG1 FG1n total/G1n	14	65805410	1	1.46E-15	4.03E-17	+	0.279509	0.3227	+	A	C	0.571962825	0.0346734	+	A	C	0.6026	0.0384
rs747541	LC_IGP64	IgG1 FG2n total/G2n	14	65805410	1	5.37E-12	3.38E-18	+	0.242362	0.3319	+	A	C	0.571962825	0.0348837	+	A	C	0.6026	0.0382

rs747541	LC_IGP68	IgG1 FG2n/G2n	14	65805410	1	7.39E-10	6.37E-10	+	0.216673	0.2348	+	A	C	0.571962825	0.0349768	+	A	C	0.6026	0.038
rs747541	LC_IGP61	IgG1 Fn total	14	65805410	1	5.43E-15	1.47E-16	+	0.273684	0.3171	+	A	C	0.571962825	0.0346769	+	A	C	0.6026	0.0384
rs747541	LC_IGP81	IgG1 Fn/Bn	14	65805410	1	1.10E-12	8.40E-12	+	0.249855	0.2604	+	A	C	0.571962825	0.0348216	+	A	C	0.6026	0.0381
rs747541	LC_IGP21	IgG1 Fucosylation	14	65805410	1	4.57E-13	1.26E-15	+	0.253166	0.3066	+	A	C	0.571293465	0.0346917	+	A	C	0.6026	0.0383
rs747541	LC_IGP28	IgG1 G1S1/(G1+G1S1)	14	65805410	1	1.01E-09	1.67E-11	+	0.21294	0.248	+	A	C	0.571344343	0.0346614	+	A	C	0.6026	0.0368
rs747541	LC_IGP11	IgG1_G0	14	65805410	1	3.52E-15	1.69E-15	-	-0.273309	-0.304	+	A	C	0.571251069	0.0343902	+	A	C	0.6026	0.0382
rs747541	LC_IGP_R1	IgG1_G0F/IgG1_G0	14	65805410	1	3.35E-14	4.37E-13	+	0.263401	0.2771	+	A	C	0.571251069	0.0344214	+	A	C	0.6026	0.0383
rs747541	LC_IGP_R4	IgG1_G0FN/IgG1_G0N	14	65805410	1	1.53E-12	8.85E-16	+	0.247261	0.3099	+	A	C	0.571425597	0.0346897	+	A	C	0.6026	0.0385
rs747541	LC_IGP52	IgG1_G0n	14	65805410	1	2.38E-15	1.33E-15	-	-0.276402	-0.3057	+	A	C	0.571962825	0.0345569	+	A	C	0.6026	0.0382
rs747541	LC_IGP14	IgG1_G0N	14	65805410	1	1.25E-14	2.98E-12	-	-0.269143	-0.2654	+	A	C	0.571425597	0.0345807	+	A	C	0.6026	0.038
rs747541	LC_IGP55	IgG1_G0Nn	14	65805410	1	3.02E-14	2.82E-12	-	-0.266101	-0.266	+	A	C	0.571962825	0.0347072	+	A	C	0.6026	0.0381
rs747541	LC_IGP12	IgG1_G1	14	65805410	1	4.84E-14	1.71E-16	-	-0.262353	-0.3161	+	A	C	0.571257178	0.0345056	+	A	C	0.6026	0.0384
rs747541	LC_IGP_R2	IgG1_G1F/IgG1_G1	14	65805410	1	3.40E-15	5.65E-16	+	0.274054	0.3099	+	A	C	0.571257178	0.0344646	+	A	C	0.6026	0.0383
rs747541	LC_IGP_R5	IgG1_G1FN/IgG1_G1N	14	65805410	1	2.33E-13	5.82E-21	+	0.256811	0.3606	+	A	C	0.572348093	0.0347477	+	A	C	0.6026	0.0384
rs747541	LC_IGP53	IgG1_G1n	14	65805410	1	1.22E-13	3.15E-16	-	-0.259843	-0.313	+	A	C	0.571962825	0.0347401	+	A	C	0.6026	0.0383
rs747541	LC_IGP15	IgG1_G1N	14	65805410	1	1.54E-11	1.71E-12	-	-0.236489	-0.2691	+	A	C	0.572348093	0.0348177	+	A	C	0.6026	0.0381
rs747541	LC_IGP56	IgG1_G1Nn	14	65805410	1	2.01E-11	3.77E-12	-	-0.235715	-0.2645	+	A	C	0.571962825	0.0349017	+	A	C	0.6026	0.0381
rs747541	LC_IGP_R32	IgG1_G1S1/IgG1_G1	14	65805410	1	1.36E-09	2.26E-11	+	0.211336	0.2467	+	A	C	0.571344343	0.0346727	+	A	C	0.6026	0.0369
rs747541	LC_IGP_R3	IgG1_G2F/IgG1_G2	14	65805410	1	7.51E-13	6.22E-18	+	0.250006	0.3286	+	A	C	0.571294621	0.03459	+	A	C	0.6026	0.0381
rs747541	LC_IGP_R8	IgG1_G2FS1/IgG1_G2S1	14	65805410	1	1.68E-08	3.34E-15	+	0.197229	0.2994	+	A	C	0.570945632	0.0347796	+	A	C	0.6026	0.038
rs747541	LC_IGP_R26	IgG1_G2N/IgG1_G1N	14	65805410	1	2.03E-09	9.01E-11	+	0.209804	0.2413	+	A	C	0.572307407	0.0347943	+	A	C	0.6026	0.0372
rs1954052	LC_IGP78	IgG1 BG0n/G0n	14	65805709	1	4.82E-13	3.28E-11	-	-0.253562	-0.2537	+	A	T	0.57544083	0.0347805	+	A	T	0.608	0.0382
rs1954052	LC_IGP79	IgG1 BG1n/G1n	14	65805709	1	1.33E-12	5.35E-14	-	-0.249328	-0.2874	+	A	T	0.57544083	0.034883	+	A	T	0.608	0.0382
rs1954052	LC_IGP77	IgG1 Bn	14	65805709	1	1.66E-12	3.45E-13	-	-0.248317	-0.2792	+	A	T	0.57544083	0.0348947	+	A	T	0.608	0.0384
rs1954052	LC_IGP86	IgG1 Bn/Fn total	14	65805709	1	2.54E-13	1.31E-13	-	-0.257133	-0.2844	+	A	T	0.57544083	0.0348472	+	A	T	0.608	0.0384
rs1954052	LC_IGP84	IgG1 FBn/Bn total	14	65805709	1	1.23E-13	7.24E-20	+	0.261022	0.3534	+	A	T	0.57544083	0.0349024	+	A	T	0.608	0.0387
rs1954052	LC_IGP62	IgG1 FG0n total/G0n	14	65805709	1	1.25E-15	4.26E-14	+	0.279701	0.2908	+	A	T	0.57544083	0.034613	+	A	T	0.608	0.0385
rs1954052	LC_IGP63	IgG1 FG1n total/G1n	14	65805709	1	3.43E-16	1.45E-17	+	0.285948	0.3284	+	A	T	0.57544083	0.0346876	+	A	T	0.608	0.0385
rs1954052	LC_IGP64	IgG1 FG2n total/G2n	14	65805709	1	1.15E-12	1.35E-18	+	0.250147	0.337	+	A	T	0.57544083	0.0348962	+	A	T	0.608	0.0383
rs1954052	LC_IGP68	IgG1 FG2n/G2n	14	65805709	1	7.53E-10	4.03E-10	+	0.216854	0.2382	+	A	T	0.57544083	0.0350224	+	A	T	0.608	0.0381
rs1954052	LC_IGP61	IgG1 Fn total	14	65805709	1	1.15E-15	5.66E-17	+	0.280667	0.3226	+	A	T	0.57544083	0.034689	+	A	T	0.608	0.0385
rs1954052	LC_IGP81	IgG1 Fn/Bn	14	65805709	1	6.60E-13	4.10E-12	+	0.252585	0.2649	+	A	T	0.57544083	0.0348559	+	A	T	0.608	0.0382
rs1954052	LC_IGP21	IgG1 Fucosylation	14	65805709	1	1.12E-13	5.74E-16	+	0.25998	0.3114	+	A	T	0.574756165	0.0347062	+	A	T	0.608	0.0385
rs1954052	LC_IGP28	IgG1 G1S1/(G1+G1S1)	14	65805709	1	2.63E-10	6.80E-12	+	0.220501	0.2539	+	A	T	0.574777676	0.0346772	+	A	T	0.608	0.037
rs1954052	LC_IGP11	IgG1_G0	14	65805709	1	9.03E-16	1.44E-15	-	-0.279399	-0.3059	+	A	T	0.57468204	0.0344053	+	A	T	0.608	0.0383
rs1954052	LC_IGP_R1	IgG1_G0F/IgG1_G0	14	65805709	1	8.12E-15	2.44E-13	+	0.269943	0.2811	+	A	T	0.57468204	0.0344354	+	A	T	0.608	0.0384
rs1954052	LC_IGP_R4	IgG1_G0FN/IgG1_G0N	14	65805709	1	2.57E-13	6.17E-16	+	0.255951	0.3127	+	A	T	0.574864972	0.0346965	+	A	T	0.608	0.0387
rs1954052	LC_IGP52	IgG1_G0n	14	65805709	1	5.99E-16	1.07E-15	-	-0.282581	-0.3078	+	A	T	0.57544083	0.0345724	+	A	T	0.608	0.0384
rs1954052	LC_IGP14	IgG1_G0N	14	65805709	1	4.90E-15	3.97E-12	-	-0.273551	-0.2646	+	A	T	0.574864972	0.0346051	+	A	T	0.608	0.0381
rs1954052	LC_IGP55	IgG1_G0Nn	14	65805709	1	1.25E-14	3.53E-12	-	-0.270358	-0.2655	+	A	T	0.57544083	0.0347334	+	A	T	0.608	0.0382
rs1954052	LC_IGP12	IgG1_G1	14	65805709	1	8.46E-15	5.17E-17	-	-0.270362	-0.3227	+	A	T	0.574686316	0.0345128	+	A	T	0.608	0.0385
rs1954052	LC_IGP_R2	IgG1_G1F/IgG1_G1	14	65805709	1	8.68E-16	2.09E-16	+	0.280171	0.3156	+	A	T	0.574686316	0.0344796	+	A	T	0.608	0.0384
rs1954052	LC_IGP_R5	IgG1_G1FN/IgG1_G1N	14	65805709	1	7.00E-14	1.77E-21	+	0.26264	0.3667	+	A	T	0.575802276	0.0347675	+	A	T	0.608	0.0385
rs1954052	LC_IGP53	IgG1_G1n	14	65805709	1	2.14E-14	1.02E-16	-	-0.268005	-0.3194	+	A	T	0.57544083	0.0347482	+	A	T	0.608	0.0385
rs1954052	LC_IGP15	IgG1_G1N	14	65805709	1	9.51E-12	5.46E-13	-	-0.23922	-0.2758	+	A	T	0.575802276	0.0348528	+	A	T	0.608	0.0382
rs1954052	LC_IGP56	IgG1_G1Nn	14	65805709	1	1.23E-11	1.23E-12	-	-0.238503	-0.2712	+	A	T	0.57544083	0.0349365	+	A	T	0.608	0.0382
rs1954052	LC_IGP_R32	IgG1_G1S1/IgG1_G1	14	65805709	1	3.31E-10	9.35E-12	+	0.219309	0.2525	+	A	T	0.574777676	0.0346872	+	A	T	0.608	0.037
rs1954052	LC_IGP13	IgG1_G2	14	65805709	1	3.22E-08	4.55E-14	-	-0.19372	-0.286	+	A	T	0.57468204	0.0348664	+	A	T	0.608	0.0379
rs1954052	LC_IGP_R3	IgG1_G2F/IgG1_G2	14	65805709	1	1.90E-13	2.45E-18	+	0.256708	0.3338	+	A	T	0.574727689	0.034605	+	A	T	0.608	0.0382
rs1954052	LC_IGP_R8	IgG1_G2FS1/IgG1_G2S1	14	65805709	1	7.09E-09	8.96E-16	+	0.202552	0.3065	+	A	T	0.574376604	0.0348053	+	A	T	0.608	0.0381
rs1954052	LC_IGP_R26	IgG1_G2N/IgG1_G1N	14	65805709	1	4.06E-09	8.75E-11	+	0.206139	0.2424	+	A	T	0.575774383	0.0348541	+	A	T	0.608	0.0374
rs12895337	LC_IGP84	IgG1 FBn/Bn total	14	65807442	1	4.53E-11	1.43E-09	+	0.238592	0.2415	+	T	C	0.634543061	0.0359798	+	T	C	0.6546	0.0399
rs12895337	LC_IGP63	IgG1 FG1n total/G1n	14	65807442	1	8.85E-13	5.87E-08	+	0.257966	0.2151	+	T	C	0.634543061	0.0358012	+	T	C	0.6546	0.0397
rs12895337	LC_IGP64	IgG1 FG2n total/G2n	14	65807442	1	3.74E-10	1.52E-09	+	0.226746	0.2382	+	T	C	0.634543061	0.0359704	+	T	C	0.6546	0.0394
rs12895337	LC_IGP61	IgG1 Fn total	14	65807442	1	1.03E-12	8.23E-08	+	0.25706	0.2128	+	T	C	0.634543061	0.0357793	+	T	C	0.6546	0.0397
rs12895337	LC_IGP21	IgG1 Fucosylation	14	65807442	1	1.12E-10	3.24E-07	+	0.232247	0.2025	+	T	C	0.63387947	0.0357686	+	T	C	0.6546	0.0396
rs12895337	LC_IGP11	IgG1_G0	14	65807442	1	7.37E-13	2.18E-07	-	-0.256016	-0.2046	+	T	C	0.634179902	0.0354086	+	T	C	0.6546	0.0395
rs12895337	LC_IGP_R4	IgG1_G0FN/IgG1_G0N	14	65807442	1	8.77E-11	1.42E-07	+	0.233179	0.2097	+	T	C	0.634202388	0.0357094	+	T	C	0.6546	0.0398
rs12895337	LC_IGP52	IgG1_G0n	14	65807442	1	1.82E-13	2.47E-07	-	-0.264602	-0.2041	+	T	C	0.634543061	0.0356358	+	T	C	0.6546	0.0396
rs12895337	LC_IGP12	IgG1_G1	14	65807442	1	5.00E-12	4.13E-08	-	-0.247147	-0.2175	+	T	C	0.633874465	0.0355227	+	T	C	0.6546	0.0397
rs12895337	LC_IGP_R2	IgG1_G1F/IgG1_G1	14	65807442	1	1.72E-12	2.05E-07	+	0.252529	0.2056	+	T	C	0.633874465	0.0355146	+	T	C	0.6546	0.0396
rs12895337	LC_IGP_R5	IgG1_G1FN/IgG1_G1N	14	65807442	1	7.05E-11	2.07E-10	+	0.235217	0.2523	+	T	C	0.635087638	0.0358374	+	T	C	0.6546	0.0397
rs12895337	LC_IGP53	IgG1_G1n	14	65807442	1	1.35E-11	7.00E-08	-	-0.244106	-0.2136	+	T	C	0.634543061	0.0358326	+	T	C	0.6546	0.0396
rs12895337	LC_IGP_R3	IgG1_G2F/IgG1_G2	14	65807442	1	1.25E-10	1.58E-09	+	0.23056	0.2375	+	T	C	0.634261919	0.0356104	+	T	C	0.6546	0.0394
rs73278083	LC_IGP52	IgG1_G0n	14	65807694	1	2.80E-08	9.21E-09	+	0.371931	0.424	+	A	G	0.076646411	0.0666409	+	A	G	0.0735	0.0738
rs74825628	LC_IGP84	IgG1 FBn/Bn total	14	65807781	1	4.68E-08	9.29E-07	-	-0.236579	-0.2512	+	A	G	0.20678384	0.0430977	+	A	G	0.1722	0.0512
rs74825628	LC_IGP61	IgG1 Fn total	14	65807781	1	1.28E-08	2.20E-06	-	-0.245418	-0.2411	+	A	G	0.20678384	0.042923	+	A	G	0.1722	0.0509
rs74825628	LC_IGP12	IgG1_G1	14	65807781	1	3.98E-08	7.30E-07	+	0.235157	0.2519	+	A	G	0.206932875	0.0426166	+	A	G	0.1722	0.0509
rs74351765	LC_IGP61	IgG1 Fn total	14	65808468	1	1.41E-08	7.39E-07	-	-0.244681	-0.2509	+	T	C	0.206810227	0.0429166	+	T	C	0.1751	0.0507
rs74351765	LC_IGP12	IgG1_G1	14	65808468	1	4.31E-08	2.33E-07	+	0.234519	0.2617	+	T	C	0.206958892	0.04261	+	T	C	0.1751	0.0506
rs12886005	LC_IGP78	IgG1 BG0n/G0n	14	65809247	1	6.84E-13	4.42E-11	-	-0.251796	-0.251	+	T	C	0.575629492	0.0347708	+	T	C	0.6053	0.0381
rs12886005	LC_IGP79	IgG1 BG1n/G1n	14	65809247	1	1.88E-12	9.64E-14	-	-0.247554	-0.2833	+	T	C	0.575629492	0.0348731	+	T	C	0.6053	0.038
rs12886005	LC_IGP77	IgG1 Bn	14	65809247	1	2.34E-12	5.91E-13	-	-0.246555	-0.2753	+	T	C	0.575629492	0.0348847	+	T	C	0.6053	0.0382
rs12886005	LC_IGP86	IgG1 Bn/Fn total	14	65809247	1	3.63E-13	2.16E-13	-	-0.255359	-0.2807	+	T	C	0.575629492	0.0348375	+	T	C	0.6053	0.0382
rs12886005	LC_IGP84	IgG1 FBn/Bn total	14	65809247	1	1.75E-13	9.70E-20	+	0.259253	0.3504	+	T	C	0.575629492	0.0348928	+	T	C	0.6053	0.0385
rs12886005	LC_IGP62	IgG1 FG0n total/G0n	14	65809247	1	1.80E-15	8.20E-14	+	0.278014	0.2862	+	T	C	0.575629492	0.0346036	+	T	C	0.6053	0.0383
rs12886005	LC_IGP63	IgG1 FG1n total/G1n	14	65809247	1	4.83E-16	3.11E-17	+	0.284376	0.3234	+	T	C	0.575629492	0.0346777	+	T	C	0.6053	0.0383
rs12886005	LC_IGP64	IgG1 FG2n total/G2n	14	65809247	1	1.42E-12	1.86E-18	+	0.249026	0.334	+	T	C	0.575629492	0.0348834	+	T	C	0.6053	0.0381
rs12886005	LC_IGP68	IgG1 FG2n/G2n	14	65809247	1	8.68E-10	5.74E-10	+	0.215961	0.2352	+	T	C	0.575629492	0.0350082	+	T	C	0.6053	0.038
rs12886005	LC_IGP61	IgG1 Fn total	14	65809247	1	1.64E-15	1.24E-16	+	0.279037	0.3174	+	T	C	0.575629492	0.0346793	+	T	C	0.6053	0.0383
rs12886005	LC_IGP81	IgG1 Fn/Bn	14	65809247	1	9.26E-13	7.58E-12	+	0.250859	0.2606	+	T	C	0.575629492	0.0348459	+	T	C	0.6053	0.0381
rs12886005	LC_IGP21	IgG1 Fucosylation	14	65809247	1	1.53E-13	1.16E-15	+	0.25843	0.3067	+	T	C	0.574948212	0.0346957	+	T	C	0.6053	0.0383
rs12886005	LC_IGP28	IgG1 G1S1/(G1+G1S1)	14	65809247	1	2.46E-10	1.43E-11	+	0.220765	0.2486	+	T	C	0.574963914	0.0346588	+	T	C	0.6053	0.0368
rs12886005	LC_IGP11	IgG1_G0	14	65809247	1	1.19E-15	3.12E-15	-	-0.278114	-0.3006	+	T	C	0.574872327	0.0343943	+	T	C	0.6053	0.0381
rs12886005	LC_IGP_R1	IgG1_G0F/IgG1_G0	14	65809247	1	1.15E-14	4.99E-13	+	0.268304	0.276	+	T	C	0.574872327	0.0344259	+	T	C	0.6053	0.0382
rs12886005	LC_IGP_R4	IgG1_G0FN/IgG1_G0N	14	65809247	1	3.63E-13	7.56E-16	+	0.254225	0.3102	+	T	C	0.575055726	0.0346868	+	T	C	0.6053	0.0385
rs12886005	LC_IGP52	IgG1_G0n	14	65809247	1	7.94E-16	2.38E-15	-	-0.281265	-0.3024	+	T	C	0.575629492	0.0345613	+	T	C	0.6053	0.0382
rs12886005	LC_IGP14	IgG1_G0N	14	65809247	1	6.67E-15	4.79E-12	-	-0.27208	-0.2624	+	T	C	0.575055726	0.0345947	+	T	C	0.6053	0.038
rs12886005	LC_IGP55	IgG1_G0Nn	14	65809247	1	1.73E-14	4.35E-12	-	-0.268796	-0.2632	+	T	C	0.575629492	0.0347231	+	T	C	0.6053	0.038
rs12886005	LC_IGP12	IgG1_G1	14	65809247	1	1.11E-14	1.23E-16	-	-0.269041	-0.3172	+	T	C	0.574872327	0.0345016	+	T	C	0.6053	0.0383
rs12886005	LC_IGP_R2	IgG1_G1F/IgG1_G1	14	65809247	1	1.17E-15	5.02E-16	+	0.278784	0.31	+	T	C	0.574872327	0.034469	+	T	C	0.6053	0.0382
rs12886005	LC_IGP_R5	IgG1_G1FN/IgG1_G1N	14	65809247	1	1.01E-13	2.67E-21	+	0.260828	0.3631	+	T	C	0.57599385	0.0347584	+	T	C	0.6053	0.0383
rs12886005	LC_IGP53	IgG1_G1n	14	65809247	1	2.87E-14	2.28E-16	-	-0.266577	-0.3141	+	T	C	0.575629492	0.0347372	+	T	C	0.6053	0.0383
rs12886005	LC_IGP15	IgG1_G1N	14	65809247	1	1.30E-11	1.06E-12	-	-0.237527	-0.2713	+	T	C	0.57599385	0.0348424	+	T	C	0.6053	0.0381
rs12886005	LC_IGP56	IgG1_G1Nn	14	65809247	1	1.70E-11	2.25E-12	-	-0.236733	-0.2669	+	T	C	0.575629492	0.0349262	+	T	C	0.6053	0.038
rs12886005	LC_IGP_R32	IgG1_G1S1/IgG1_G1	14	65809247	1	3.10E-10	1.92E-11	+	0.219564	0.2472	+	T	C	0.574963914	0.0346688	+	T	C	0.6053	0.0368
rs12886005	LC_IGP13	IgG1_G2	14	65809247	1	3.99E-08	6.55E-14	-	-0.192302	-0.2826	+	T	C	0.574872327	0.0348539	+	T	C	0.6053	0.0377
rs12886005	LC_IGP_R3	IgG1_G2F/IgG1_G2	14	65809247	1	2.35E-13	3.49E-18	+	0.255597	0.3306	+	T	C	0.574918093	0.0345927	+	T	C	0.6053	0.038
rs12886005	LC_IGP_R8	IgG1_G2FS1/IgG1_G2S1	14	65809247	1	8.56E-09	1.24E-15	+	0.201349	0.3035	+	T	C	0.574566891	0.0347923	+	T	C	0.6053	0.0379
rs12886005	LC_IGP_R26	IgG1_G2N/IgG1_G1N	14	65809247	1	4.61E-09	1.22E-10	+	0.205305	0.2392	+	T	C	0.575966667	0.0348398	+	T	C	0.6053	0.0372
rs12886168	LC_IGP78	IgG1 BG0n/G0n	14	65809286	1	2.10E-12	4.16E-11	-	-0.246091	-0.2512	+	A	C	0.572173482	0.0347448	+	A	C	0.6027	0.0381
rs12886168	LC_IGP79	IgG1 BG1n/G1n	14	65809286	1	2.91E-12	1.32E-13	-	-0.245084	-0.2815	+	A	C	0.572173482	0.0348324	+	A	C	0.6027	0.038
rs12886168	LC_IGP77	IgG1 Bn	14	65809286	1	4.44E-12	6.40E-13	-	-0.243082	-0.2747	+	A	C	0.572173482	0.0348484	+	A	C	0.6027	0.0382
rs12886168	LC_IGP86	IgG1 Bn/Fn total	14	65809286	1	7.47E-13	2.24E-13	-	-0.251602	-0.2803	+	A	C	0.572173482	0.034803	+	A	C	0.6027	0.0382
rs12886168	LC_IGP84	IgG1 FBn/Bn total	14	65809286	1	8.57E-13	1.63E-19	+	0.251456	0.3479	+	A	C	0.572173482	0.0348766	+	A	C	0.6027	0.0385
rs12886168	LC_IGP62	IgG1 FG0n total/G0n	14	65809286	1	9.50E-15	7.23E-14	+	0.270476	0.2867	+	A	C	0.572173482	0.0345892	+	A	C	0.6027	0.0383
rs12886168	LC_IGP63	IgG1 FG1n total/G1n	14	65809286	1	2.07E-15	3.90E-17	+	0.277854	0.3221	+	A	C	0.572173482	0.0346589	+	A	C	0.6027	0.0383
rs12886168	LC_IGP64	IgG1 FG2n total/G2n	14	65809286	1	6.61E-12	3.27E-18	+	0.241185	0.3313	+	A	C	0.572173482	0.0348661	+	A	C	0.6027	0.0381
rs12886168	LC_IGP68	IgG1 FG2n/G2n	14	65809286	1	8.55E-10	6.10E-10	+	0.215738	0.2346	+	A	C	0.572173482	0.0349577	+	A	C	0.6027	0.0379
rs12886168	LC_IGP61	IgG1 Fn total	14	65809286	1	7.76E-15	1.39E-16	+	0.271963	0.3167	+	A	C	0.572173482	0.0346626	+	A	C	0.6027	0.0383
rs12886168	LC_IGP81	IgG1 Fn/Bn	14	65809286	1	1.55E-12	7.74E-12	+	0.248035	0.2603	+	A	C	0.572173482	0.034807	+	A	C	0.6027	0.038
rs12886168	LC_IGP21	IgG1 Fucosylation	14	65809286	1	6.29E-13	1.20E-15	+	0.251508	0.3062	+	A	C	0.571509248	0.0346767	+	A	C	0.6027	0.0382
rs12886168	LC_IGP28	IgG1 G1S1/(G1+G1S1)	14	65809286	1	9.49E-10	1.64E-11	+	0.213164	0.2477	+	A	C	0.571552294	0.0346383	+	A	C	0.6027	0.0368
rs12886168	LC_IGP11	IgG1_G0	14	65809286	1	4.66E-15	1.50E-15	-	-0.271946	-0.304	+	A	C	0.571464875	0.0343746	+	A	C	0.6027	0.0381
rs12886168	LC_IGP_R1	IgG1_G0F/IgG1_G0	14	65809286	1	4.78E-14	4.11E-13	+	0.261667	0.2769	+	A	C	0.571464875	0.0344074	+	A	C	0.6027	0.0382
rs12886168	LC_IGP_R4	IgG1_G0FN/IgG1_G0N	14	65809286	1	2.17E-12	8.66E-16	+	0.24542	0.3094	+	A	C	0.571639927	0.0346753	+	A	C	0.6027	0.0385
rs12886168	LC_IGP52	IgG1_G0n	14	65809286	1	3.16E-15	1.19E-15	-	-0.275025	-0.3057	+	A	C	0.572173482	0.034541	+	A	C	0.6027	0.0382
rs12886168	LC_IGP14	IgG1_G0N	14	65809286	1	1.72E-14	2.68E-12	-	-0.267567	-0.2655	+	A	C	0.571639927	0.0345658	+	A	C	0.6027	0.038
rs12886168	LC_IGP55	IgG1_G0Nn	14	65809286	1	4.22E-14	2.54E-12	-	-0.264445	-0.266	+	A	C	0.572173482	0.0346923	+	A	C	0.6027	0.038

rs12886168	LC_IGP12	IgG1_G1	14	65809286	1	6.36E-14	1.69E-16	-	-0.260969	-0.3154	+	A	C	0.571464875	0.0344897	+	A	C	0.6027	0.0383
rs12886168	LC_IGP_R2	IgG1_G1F/IgG1_G1	14	65809286	1	4.59E-15	5.49E-16	+	0.272598	0.3093	+	A	C	0.571464875	0.0344493	+	A	C	0.6027	0.0382
rs12886168	LC_IGP_R5	IgG1_G1FN/IgG1_G1N	14	65809286	1	3.41E-13	5.52E-21	+	0.254883	0.36	+	A	C	0.572563346	0.034734	+	A	C	0.6027	0.0383
rs12886168	LC_IGP53	IgG1_G1n	14	65809286	1	1.63E-13	3.14E-16	-	-0.258346	-0.3124	+	A	C	0.572173482	0.0347243	+	A	C	0.6027	0.0382
rs12886168	LC_IGP15	IgG1_G1N	14	65809286	1	2.15E-11	1.61E-12	-	-0.234682	-0.2688	+	A	C	0.572563346	0.0348029	+	A	C	0.6027	0.038
rs12886168	LC_IGP56	IgG1_G1Nn	14	65809286	1	2.81E-11	3.58E-12	-	-0.233851	-0.2642	+	A	C	0.572173482	0.0348869	+	A	C	0.6027	0.038
rs12886168	LC_IGP_R32	IgG1_G1S1/IgG1_G1	14	65809286	1	1.28E-09	2.23E-11	+	0.211551	0.2463	+	A	C	0.571552294	0.0346497	+	A	C	0.6027	0.0368
rs12886168	LC_IGP_R3	IgG1_G2F/IgG1_G2	14	65809286	1	9.35E-13	6.04E-18	+	0.248821	0.328	+	A	C	0.571508557	0.034573	+	A	C	0.6027	0.038
rs12886168	LC_IGP_R8	IgG1_G2FS1/IgG1_G2S1	14	65809286	1	2.03E-08	3.19E-15	+	0.195953	0.2989	+	A	C	0.571159438	0.034762	+	A	C	0.6027	0.0379
rs12886168	LC_IGP_R26	IgG1_G2N/IgG1_G1N	14	65809286	1	2.33E-09	8.05E-11	+	0.208901	0.2415	+	A	C	0.572523457	0.0347755	+	A	C	0.6027	0.0372
rs7143666	LC_IGP79	IgG1 BG1n/G1n	14	65809977	1	3.83E-10	8.97E-07	-	-0.226569	-0.1929	+	C	A	0.633526332	0.035963	+	C	A	0.6526	0.0393
rs7143666	LC_IGP86	IgG1 Bn/Fn total	14	65809977	1	7.65E-11	2.08E-06	-	-0.23537	-0.1872	+	C	A	0.633526332	0.0359274	+	C	A	0.6526	0.0395
rs7143666	LC_IGP84	IgG1 FBn/Bn total	14	65809977	1	6.71E-11	1.16E-10	+	0.236556	0.2562	+	C	A	0.633526332	0.0359974	+	C	A	0.6526	0.0398
rs7143666	LC_IGP62	IgG1 FG0n total/G0n	14	65809977	1	6.05E-13	5.18E-07	+	0.259115	0.1986	+	C	A	0.633526332	0.0356966	+	C	A	0.6526	0.0396
rs7143666	LC_IGP63	IgG1 FG1n total/G1n	14	65809977	1	1.79E-12	5.41E-09	+	0.254556	0.2305	+	C	A	0.633526332	0.0358256	+	C	A	0.6526	0.0395
rs7143666	LC_IGP64	IgG1 FG2n total/G2n	14	65809977	1	6.42E-10	1.18E-10	+	0.223778	0.2532	+	C	A	0.633526332	0.0359911	+	C	A	0.6526	0.0393
rs7143666	LC_IGP61	IgG1 Fn total	14	65809977	1	1.98E-12	9.17E-09	+	0.253877	0.2273	+	C	A	0.633526332	0.0358026	+	C	A	0.6526	0.0396
rs7143666	LC_IGP21	IgG1 Fucosylation	14	65809977	1	1.85E-10	3.98E-08	+	0.229578	0.2169	+	C	A	0.632867756	0.0357885	+	C	A	0.6526	0.0395
rs7143666	LC_IGP11	IgG1_G0	14	65809977	1	1.04E-12	3.78E-08	-	-0.254414	-0.2165	+	C	A	0.633177459	0.0354247	+	C	A	0.6526	0.0394
rs7143666	LC_IGP_R1	IgG1_G0F/IgG1_G0	14	65809977	1	5.74E-12	1.47E-06	+	0.24589	0.1899	+	C	A	0.633177459	0.0354452	+	C	A	0.6526	0.0394
rs7143666	LC_IGP_R4	IgG1_G0FN/IgG1_G0N	14	65809977	1	1.15E-10	2.22E-08	+	0.23177	0.2223	+	C	A	0.633197489	0.0357242	+	C	A	0.6526	0.0397
rs7143666	LC_IGP52	IgG1_G0n	14	65809977	1	2.61E-13	4.20E-08	-	-0.262945	-0.2162	+	C	A	0.633526332	0.0356526	+	C	A	0.6526	0.0394
rs7143666	LC_IGP12	IgG1_G1	14	65809977	1	9.80E-12	4.51E-09	-	-0.243812	-0.2318	+	C	A	0.632872022	0.035546	+	C	A	0.6526	0.0395
rs7143666	LC_IGP_R2	IgG1_G1F/IgG1_G1	14	65809977	1	3.59E-12	2.24E-08	+	0.248964	0.2205	+	C	A	0.632872022	0.0355391	+	C	A	0.6526	0.0394
rs7143666	LC_IGP_R5	IgG1_G1FN/IgG1_G1N	14	65809977	1	1.09E-10	1.09E-11	+	0.232927	0.2685	+	C	A	0.634078413	0.0358556	+	C	A	0.6526	0.0395
rs7143666	LC_IGP53	IgG1_G1n	14	65809977	1	2.63E-11	7.89E-09	-	-0.240716	-0.2279	+	C	A	0.633526332	0.0358561	+	C	A	0.6526	0.0395
rs7143666	LC_IGP15	IgG1_G1N	14	65809977	1	2.31E-09	2.21E-06	-	-0.215723	-0.186	+	C	A	0.634078413	0.0359026	+	C	A	0.6526	0.0393
rs7143666	LC_IGP_R3	IgG1_G2F/IgG1_G2	14	65809977	1	2.20E-10	1.24E-10	+	0.227587	0.2524	+	C	A	0.633258863	0.0356312	+	C	A	0.6526	0.0392
rs59136469	LC_IGP11	IgG1_G0	14	65811352	1	4.41E-08	1.59E-08	+	0.358942	0.4144	+	A	G	0.077664162	0.0652681	+	A	G	0.074	0.0733
rs59136469	LC_IGP52	IgG1_G0n	14	65811352	1	2.51E-08	1.01E-08	+	0.368974	0.421	+	A	G	0.077841533	0.0658797	+	A	G	0.074	0.0735
rs4899174	LC_IGP78	IgG1 BG0n/G0n	14	65811400	1	9.18E-13	4.38E-11	-	-0.250294	-0.251	+	T	A	0.575748761	0.0347614	+	T	A	0.6053	0.0381
rs4899174	LC_IGP79	IgG1 BG1n/G1n	14	65811400	1	2.53E-12	9.51E-14	-	-0.246013	-0.2834	+	T	A	0.575748761	0.0348637	+	T	A	0.6053	0.0381
rs4899174	LC_IGP77	IgG1 Bn	14	65811400	1	3.12E-12	5.85E-13	-	-0.245039	-0.2754	+	T	A	0.575748761	0.0348752	+	T	A	0.6053	0.0382
rs4899174	LC_IGP86	IgG1 Bn/Fn total	14	65811400	1	4.91E-13	2.14E-13	-	-0.253824	-0.2808	+	T	A	0.575748761	0.0348283	+	T	A	0.6053	0.0382
rs4899174	LC_IGP84	IgG1 FBn/Bn total	14	65811400	1	2.38E-13	9.63E-20	+	0.257731	0.3504	+	T	A	0.575748761	0.0348836	+	T	A	0.6053	0.0385
rs4899174	LC_IGP62	IgG1 FG0n total/G0n	14	65811400	1	2.47E-15	8.17E-14	+	0.276548	0.2862	+	T	A	0.575748761	0.0345947	+	T	A	0.6053	0.0383
rs4899174	LC_IGP63	IgG1 FG1n total/G1n	14	65811400	1	6.53E-16	3.09E-17	+	0.282989	0.3235	+	T	A	0.575748761	0.0346686	+	T	A	0.6053	0.0383
rs4899174	LC_IGP64	IgG1 FG2n total/G2n	14	65811400	1	1.71E-12	1.85E-18	+	0.24801	0.3341	+	T	A	0.575748761	0.0348717	+	T	A	0.6053	0.0381
rs4899174	LC_IGP68	IgG1 FG2n/G2n	14	65811400	1	9.94E-10	5.69E-10	+	0.215113	0.2353	+	T	A	0.575748761	0.0349953	+	T	A	0.6053	0.038
rs4899174	LC_IGP61	IgG1 Fn total	14	65811400	1	2.22E-15	1.24E-16	+	0.277625	0.3175	+	T	A	0.575748761	0.0346701	+	T	A	0.6053	0.0384
rs4899174	LC_IGP81	IgG1 Fn/Bn	14	65811400	1	1.24E-12	7.51E-12	+	0.249363	0.2607	+	T	A	0.575748761	0.0348364	+	T	A	0.6053	0.0381
rs4899174	LC_IGP21	IgG1 Fucosylation	14	65811400	1	1.97E-13	1.15E-15	+	0.257141	0.3067	+	T	A	0.575066893	0.0346856	+	T	A	0.6053	0.0383
rs4899174	LC_IGP28	IgG1 G1S1/(G1+G1S1)	14	65811400	1	2.33E-10	1.43E-11	+	0.22096	0.2486	+	T	A	0.575081651	0.0346422	+	T	A	0.6053	0.0368
rs4899174	LC_IGP11	IgG1_G0	14	65811400	1	1.51E-15	3.13E-15	-	-0.276988	-0.3006	+	T	A	0.574989921	0.0343839	+	T	A	0.6053	0.0381
rs4899174	LC_IGP_R1	IgG1_G0F/IgG1_G0	14	65811400	1	1.54E-14	4.98E-13	+	0.266901	0.2761	+	T	A	0.574989921	0.0344167	+	T	A	0.6053	0.0382
rs4899174	LC_IGP_R4	IgG1_G0FN/IgG1_G0N	14	65811400	1	4.81E-13	7.52E-16	+	0.252801	0.3102	+	T	A	0.575173607	0.0346772	+	T	A	0.6053	0.0385
rs4899174	LC_IGP52	IgG1_G0n	14	65811400	1	1.02E-15	2.38E-15	-	-0.280074	-0.3025	+	T	A	0.575748761	0.0345512	+	T	A	0.6053	0.0382
rs4899174	LC_IGP14	IgG1_G0N	14	65811400	1	8.64E-15	4.77E-12	-	-0.270837	-0.2625	+	T	A	0.575173607	0.0345846	+	T	A	0.6053	0.038
rs4899174	LC_IGP55	IgG1_G0Nn	14	65811400	1	2.28E-14	4.33E-12	-	-0.267451	-0.2633	+	T	A	0.575748761	0.0347134	+	T	A	0.6053	0.038
rs4899174	LC_IGP12	IgG1_G1	14	65811400	1	1.42E-14	1.22E-16	-	-0.267862	-0.3172	+	T	A	0.574989921	0.0344913	+	T	A	0.6053	0.0383
rs4899174	LC_IGP_R2	IgG1_G1F/IgG1_G1	14	65811400	1	1.53E-15	4.98E-16	+	0.277542	0.3101	+	T	A	0.574989921	0.0344592	+	T	A	0.6053	0.0382
rs4899174	LC_IGP_R5	IgG1_G1FN/IgG1_G1N	14	65811400	1	1.38E-13	2.64E-21	+	0.259307	0.3632	+	T	A	0.576112239	0.0347494	+	T	A	0.6053	0.0383
rs4899174	LC_IGP53	IgG1_G1n	14	65811400	1	3.71E-14	2.27E-16	-	-0.26531	-0.3142	+	T	A	0.575748761	0.034727	+	T	A	0.6053	0.0383
rs4899174	LC_IGP15	IgG1_G1N	14	65811400	1	1.69E-11	1.04E-12	-	-0.236111	-0.2714	+	T	A	0.576112239	0.0348324	+	T	A	0.6053	0.0381
rs4899174	LC_IGP56	IgG1_G1Nn	14	65811400	1	2.26E-11	2.22E-12	-	-0.235204	-0.267	+	T	A	0.575748761	0.0349165	+	T	A	0.6053	0.038
rs4899174	LC_IGP_R32	IgG1_G1S1/IgG1_G1	14	65811400	1	2.93E-10	1.93E-11	+	0.219755	0.2473	+	T	A	0.575081651	0.0346523	+	T	A	0.6053	0.0368
rs4899174	LC_IGP13	IgG1_G2	14	65811400	1	4.73E-08	6.49E-14	-	-0.191174	-0.2827	+	T	A	0.574989921	0.0348419	+	T	A	0.6053	0.0377
rs4899174	LC_IGP_R3	IgG1_G2F/IgG1_G2	14	65811400	1	2.83E-13	3.46E-18	+	0.254627	0.3307	+	T	A	0.575035758	0.0345812	+	T	A	0.6053	0.038
rs4899174	LC_IGP_R8	IgG1_G2FS1/IgG1_G2S1	14	65811400	1	1.01E-08	1.24E-15	+	0.200317	0.3035	+	T	A	0.574684484	0.0347802	+	T	A	0.6053	0.0379
rs4899174	LC_IGP_R26	IgG1_G2N/IgG1_G1N	14	65811400	1	5.15E-09	1.22E-10	+	0.204579	0.2393	+	T	A	0.576085494	0.0348265	+	T	A	0.6053	0.0372
rs8014475	LC_IGP84	IgG1 FBn/Bn total	14	65811537	1	7.62E-11	1.07E-09	+	0.235681	0.243	+	C	T	0.634758364	0.0359713	+	C	T	0.6569	0.0398
rs8014475	LC_IGP62	IgG1 FG0n total/G0n	14	65811537	1	6.31E-13	2.26E-06	+	0.258706	0.1874	+	C	T	0.634758364	0.0356689	+	C	T	0.6569	0.0396
rs8014475	LC_IGP63	IgG1 FG1n total/G1n	14	65811537	1	1.47E-12	4.27E-08	+	0.255337	0.217	+	C	T	0.634758364	0.0357925	+	C	T	0.6569	0.0396
rs8014475	LC_IGP64	IgG1 FG2n total/G2n	14	65811537	1	5.15E-10	1.04E-09	+	0.224841	0.2404	+	C	T	0.634758364	0.0359574	+	C	T	0.6569	0.0394
rs8014475	LC_IGP61	IgG1 Fn total	14	65811537	1	1.72E-12	6.25E-08	+	0.254374	0.2145	+	C	T	0.634758364	0.0357709	+	C	T	0.6569	0.0396
rs8014475	LC_IGP21	IgG1 Fucosylation	14	65811537	1	1.71E-10	2.41E-07	+	0.229816	0.2045	+	C	T	0.634093711	0.0357581	+	C	T	0.6569	0.0396
rs8014475	LC_IGP11	IgG1_G0	14	65811537	1	1.11E-12	2.08E-07	-	-0.253921	-0.2046	+	C	T	0.634392181	0.0353979	+	C	T	0.6569	0.0394
rs8014475	LC_IGP_R4	IgG1_G0FN/IgG1_G0N	14	65811537	1	1.41E-10	1.18E-07	+	0.230484	0.2108	+	C	T	0.634415187	0.0357001	+	C	T	0.6569	0.0398
rs8014475	LC_IGP52	IgG1_G0n	14	65811537	1	2.83E-13	2.32E-07	-	-0.26236	-0.2042	+	C	T	0.634758364	0.0356257	+	C	T	0.6569	0.0395
rs8014475	LC_IGP12	IgG1_G1	14	65811537	1	7.60E-12	3.11E-08	-	-0.244906	-0.2192	+	C	T	0.634086744	0.0355123	+	C	T	0.6569	0.0396
rs8014475	LC_IGP_R2	IgG1_G1F/IgG1_G1	14	65811537	1	2.69E-12	1.53E-07	+	0.250198	0.2075	+	C	T	0.634086744	0.0355047	+	C	T	0.6569	0.0395
rs8014475	LC_IGP_R5	IgG1_G1FN/IgG1_G1N	14	65811537	1	1.18E-10	1.43E-10	+	0.232317	0.2541	+	C	T	0.635301353	0.0358289	+	C	T	0.6569	0.0396
rs8014475	LC_IGP53	IgG1_G1n	14	65811537	1	2.10E-11	5.21E-08	-	-0.241687	-0.2154	+	C	T	0.634758364	0.0358225	+	C	T	0.6569	0.0396
rs8014475	LC_IGP_R3	IgG1_G2F/IgG1_G2	14	65811537	1	1.71E-10	1.08E-09	+	0.228774	0.2397	+	C	T	0.634474328	0.0355976	+	C	T	0.6569	0.0393
rs4902391	LC_IGP78	IgG1 BG0n/G0n	14	65815979	1	2.67E-12	5.89E-11	-	-0.24488	-0.2493	+	T	G	0.572430297	0.0347418	+	T	G	0.601	0.0381
rs4902391	LC_IGP79	IgG1 BG1n/G1n	14	65815979	1	3.57E-12	1.45E-13	-	-0.244035	-0.2813	+	T	G	0.572430297	0.0348288	+	T	G	0.601	0.0381
rs4902391	LC_IGP77	IgG1 Bn	14	65815979	1	5.45E-12	7.94E-13	-	-0.242017	-0.2738	+	T	G	0.572430297	0.0348447	+	T	G	0.601	0.0382
rs4902391	LC_IGP86	IgG1 Bn/Fn total	14	65815979	1	9.21E-13	2.70E-13	-	-0.250549	-0.2796	+	T	G	0.572430297	0.0347995	+	T	G	0.601	0.0382
rs4902391	LC_IGP84	IgG1 FBn/Bn total	14	65815979	1	1.03E-12	2.78E-19	+	0.250522	0.346	+	T	G	0.572430297	0.0348725	+	T	G	0.601	0.0385
rs4902391	LC_IGP62	IgG1 FG0n total/G0n	14	65815979	1	1.17E-14	1.01E-13	+	0.269505	0.2851	+	T	G	0.572430297	0.0345857	+	T	G	0.601	0.0383
rs4902391	LC_IGP63	IgG1 FG1n total/G1n	14	65815979	1	2.32E-15	5.05E-17	+	0.277289	0.3212	+	T	G	0.572430297	0.0346534	+	T	G	0.601	0.0383
rs4902391	LC_IGP64	IgG1 FG2n total/G2n	14	65815979	1	6.88E-12	4.43E-18	+	0.240934	0.3303	+	T	G	0.572430297	0.0348589	+	T	G	0.601	0.0381
rs4902391	LC_IGP68	IgG1 FG2n/G2n	14	65815979	1	8.81E-10	6.52E-10	+	0.215522	0.2344	+	T	G	0.572430297	0.0349503	+	T	G	0.601	0.038
rs4902391	LC_IGP61	IgG1 Fn total	14	65815979	1	9.24E-15	1.95E-16	+	0.271142	0.3154	+	T	G	0.572430297	0.0346583	+	T	G	0.601	0.0383
rs4902391	LC_IGP81	IgG1 Fn/Bn	14	65815979	1	1.90E-12	9.59E-12	+	0.247	0.2593	+	T	G	0.572430297	0.0348033	+	T	G	0.601	0.0381
rs4902391	LC_IGP21	IgG1 Fucosylation	14	65815979	1	6.98E-13	1.71E-15	+	0.250961	0.3048	+	T	G	0.571764797	0.0346709	+	T	G	0.601	0.0383
rs4902391	LC_IGP28	IgG1 G1S1/(G1+G1S1)	14	65815979	1	8.19E-10	2.22E-11	+	0.213919	0.2463	+	T	G	0.57180581	0.0346274	+	T	G	0.601	0.0368
rs4902391	LC_IGP11	IgG1_G0	14	65815979	1	4.95E-15	1.92E-15	-	-0.27162	-0.303	+	T	G	0.571718082	0.034368	+	T	G	0.601	0.0381
rs4902391	LC_IGP_R1	IgG1_G0F/IgG1_G0	14	65815979	1	5.78E-14	5.39E-13	+	0.260759	0.2756	+	T	G	0.571718082	0.0344034	+	T	G	0.601	0.0382
rs4902391	LC_IGP_R4	IgG1_G0FN/IgG1_G0N	14	65815979	1	2.61E-12	1.67E-15	+	0.244475	0.3065	+	T	G	0.571893754	0.0346712	+	T	G	0.601	0.0385
rs4902391	LC_IGP52	IgG1_G0n	14	65815979	1	3.41E-15	1.47E-15	-	-0.274634	-0.3048	+	T	G	0.572430297	0.0345347	+	T	G	0.601	0.0382
rs4902391	LC_IGP14	IgG1_G0N	14	65815979	1	1.96E-14	3.65E-12	-	-0.266936	-0.264	+	T	G	0.571893754	0.0345606	+	T	G	0.601	0.038
rs4902391	LC_IGP55	IgG1_G0Nn	14	65815979	1	4.94E-14	3.40E-12	-	-0.263682	-0.2646	+	T	G	0.572430297	0.0346877	+	T	G	0.601	0.038
rs4902391	LC_IGP12	IgG1_G1	14	65815979	1	6.76E-14	2.37E-16	-	-0.260634	-0.3141	+	T	G	0.571718082	0.0344831	+	T	G	0.601	0.0383
rs4902391	LC_IGP_R2	IgG1_G1F/IgG1_G1	14	65815979	1	4.98E-15	6.98E-16	+	0.272187	0.3085	+	T	G	0.571718082	0.0344431	+	T	G	0.601	0.0382
rs4902391	LC_IGP_R5	IgG1_G1FN/IgG1_G1N	14	65815979	1	4.05E-13	8.28E-21	+	0.254025	0.3587	+	T	G	0.572818266	0.0347297	+	T	G	0.601	0.0383
rs4902391	LC_IGP53	IgG1_G1n	14	65815979	1	1.77E-13	4.24E-16	-	-0.257899	-0.3112	+	T	G	0.572430297	0.0347181	+	T	G	0.601	0.0383
rs4902391	LC_IGP15	IgG1_G1N	14	65815979	1	2.57E-11	1.87E-12	-	-0.233711	-0.2683	+	T	G	0.572818266	0.0347988	+	T	G	0.601	0.0381
rs4902391	LC_IGP56	IgG1_G1Nn	14	65815979	1	3.46E-11	4.00E-12	-	-0.232742	-0.2638	+	T	G	0.572430297	0.0348832	+	T	G	0.601	0.038
rs4902391	LC_IGP_R32	IgG1_G1S1/IgG1_G1	14	65815979	1	1.11E-09	2.99E-11	+	0.212305	0.245	+	T	G	0.57180581	0.0346388	+	T	G	0.601	0.0369
rs4902391	LC_IGP_R3	IgG1_G2F/IgG1_G2	14	65815979	1	9.71E-13	8.49E-18	+	0.248583	0.3268	+	T	G	0.571761919	0.0345659	+	T	G	0.601	0.038
rs4902391	LC_IGP_R8	IgG1_G2FS1/IgG1_G2S1	14	65815979	1	2.10E-08	5.55E-15	+	0.195716	0.2966	+	T	G	0.571412645	0.0347547	+	T	G	0.601	0.038
rs4902391	LC_IGP_R26	IgG1_G2N/IgG1_G1N	14	65815979	1	2.40E-09	8.26E-11	+	0.208692	0.2415	+	T	G	0.572779321	0.0347681	+	T	G	0.601	0.0372
rs11623920	LC_IGP78	IgG1 BG0n/G0n	14	65819314	1	8.26E-13	5.31E-11	-	-0.250849	-0.2502	+	C	A	0.576063197	0.0347671	+	C	A	0.6049	0.0381
rs11623920	LC_IGP79	IgG1 BG1n/G1n	14	65819314	1	2.06E-12	1.13E-13	-	-0.247048	-0.283	+	C	A	0.576063197	0.0348673	+	C	A	0.6049	0.0381
rs11623920	LC_IGP77	IgG1 Bn	14	65819314	1	2.60E-12	7.01E-13	-	-0.245976	-0.2749	+	C	A	0.576063197	0.0348793	+	C	A	0.6049	0.0383
rs11623920	LC_IGP86	IgG1 Bn/Fn total	14	65819314	1	4.01E-13	2.57E-13	-	-0.254828	-0.2802	+	C	A	0.576063197	0.034832	+	C	A	0.6049	0.0383
rs11623920	LC_IGP84	IgG1 FBn/Bn total	14	65819314	1	1.85E-13	1.34E-19	+	0.258959	0.3494	+	C	A	0.576063197	0.0348862	+	C	A	0.6049	0.0386
rs11623920	LC_IGP62	IgG1 FG0n total/G0n	14	65819314	1	1.97E-15	1.09E-13	+	0.277581	0.2851	+	C	A	0.576063197	0.0345978	+	C	A	0.6049	0.0384
rs11623920	LC_IGP63	IgG1 FG1n total/G1n	14	65819314	1	4.34E-16	4.11E-17	+	0.284757	0.3226	+	C	A	0.576063197	0.0346678	+	C	A	0.6049	0.0384
rs11623920	LC_IGP64	IgG1 FG2n total/G2n	14	65819314	1	1.23E-12	2.37E-18	+	0.249654	0.3335	+	C	A	0.576063197	0.0348726	+	C	A	0.6049	0.0382
rs11623920	LC_IGP68	IgG1 FG2n/G2n	14	65819314	1	7.84E-10	5.99E-10	+	0.216472	0.2353	+	C	A	0.576063197	0.0349983	+	C	A	0.6049	0.038
rs11623920	LC_IGP61	IgG1 Fn total	14	65819314	1	1.68E-15	1.67E-16	+	0.278867	0.3165	+	C	A	0.576063197	0.0346722	+	C	A	0.6049	0.0384
rs11623920	LC_IGP81	IgG1 Fn/Bn	14	65819314	1	1.02E-12	8.98E-12	+	0.250324	0.2601	+	C	A	0.576063197	0.0348403	+	C	A	0.6049	0.0381
rs11623920	LC_IGP21	IgG1 Fucosylation	14	65819314	1	1.41E-13	1.61E-15	+	0.258736	0.3056	+	C	A	0.575379778	0.0346864	+	C	A	0.6049	0.0384
rs11623920	LC_IGP28	IgG1 G1S1/(G1+G1S1)	14	65819314	1	1.87E-10	1.90E-11	+	0.222151	0.2474	+	C	A	0.575392049	0.0346455	+	C	A	0.6049	0.0368
rs11623920	LC_IGP11	IgG1_G0	14	65819314	1	1.01E-15	4.16E-15	-	-0.278737	-0.2997	+	C	A	0.575299939	0.0343833	+	C	A	0.6049	0.0382
rs11623920	LC_IGP_R1	IgG1_G0F/IgG1_G0	14	65819314	1	1.23E-14	6.61E-13	+	0.267949	0.2749	+	C	A	0.575299939	0.0344197	+	C	A	0.6049	0.0382
rs11623920	LC_IGP_R4	IgG1_G0FN/IgG1_G0N	14	65819314	1	3.95E-13	1.02E-15	+	0.253769	0.3091	+	C	A	0.575484385	0.0346809	+	C	A	0.6049	0.0385

rs11623920	LC_IGP52	IgG1_G0n	14	65819314	1	6.85E-16	3.21E-15	-	-0.28182	-0.3015	+	C	A	0.576063197	0.0345506	+	C	A	0.6049	0.0382
rs11623920	LC_IGP14	IgG1_G0N	14	65819314	1	6.52E-15	5.63E-12	-	-0.27212	-0.262	+	C	A	0.575484385	0.0345866	+	C	A	0.6049	0.038
rs11623920	LC_IGP55	IgG1_G0Nn	14	65819314	1	1.76E-14	5.18E-12	-	-0.268668	-0.2627	+	C	A	0.576063197	0.0347158	+	C	A	0.6049	0.0381
rs11623920	LC_IGP12	IgG1_G1	14	65819314	1	9.48E-15	1.64E-16	-	-0.26968	-0.3162	+	C	A	0.575299939	0.0344907	+	C	A	0.6049	0.0383
rs11623920	LC_IGP_R2	IgG1_G1F/IgG1_G1	14	65819314	1	1.01E-15	6.55E-16	+	0.279335	0.3092	+	C	A	0.575299939	0.0344583	+	C	A	0.6049	0.0383
rs11623920	LC_IGP_R5	IgG1_G1FN/IgG1_G1N	14	65819314	1	1.03E-13	3.70E-21	+	0.260718	0.3623	+	C	A	0.576424354	0.0347511	+	C	A	0.6049	0.0384
rs11623920	LC_IGP53	IgG1_G1n	14	65819314	1	2.53E-14	3.08E-16	-	-0.26707	-0.3131	+	C	A	0.576063197	0.0347269	+	C	A	0.6049	0.0383
rs11623920	LC_IGP15	IgG1_G1N	14	65819314	1	1.42E-11	1.24E-12	-	-0.23704	-0.2708	+	C	A	0.576424354	0.0348366	+	C	A	0.6049	0.0381
rs11623920	LC_IGP56	IgG1_G1Nn	14	65819314	1	1.92E-11	2.64E-12	-	-0.236076	-0.2664	+	C	A	0.576063197	0.034921	+	C	A	0.6049	0.0381
rs11623920	LC_IGP_R32	IgG1_G1S1/IgG1_G1	14	65819314	1	2.36E-10	2.53E-11	+	0.220956	0.2461	+	C	A	0.575392049	0.0346555	+	C	A	0.6049	0.0369
rs11623920	LC_IGP13	IgG1_G2	14	65819314	1	4.59E-08	8.05E-14	-	-0.191407	-0.2819	+	C	A	0.575299939	0.0348491	+	C	A	0.6049	0.0377
rs11623920	LC_IGP_R3	IgG1_G2F/IgG1_G2	14	65819314	1	2.04E-13	4.48E-18	+	0.256191	0.3301	+	C	A	0.575345966	0.0345821	+	C	A	0.6049	0.0381
rs11623920	LC_IGP_R8	IgG1_G2FS1/IgG1_G2S1	14	65819314	1	7.53E-09	1.58E-15	+	0.202057	0.3028	+	C	A	0.574994502	0.0347819	+	C	A	0.6049	0.038
rs11623920	LC_IGP_R26	IgG1_G2N/IgG1_G1N	14	65819314	1	4.29E-09	1.49E-10	+	0.205675	0.2384	+	C	A	0.576398765	0.0348306	+	C	A	0.6049	0.0372
rs11621121	LC_IGP78	IgG1 BG0n/G0n	14	65822493	1	7.45E-13	5.27E-11	-	-0.251406	-0.2507	+	T	C	0.575741016	0.0347745	+	T	C	0.6042	0.0382
rs11621121	LC_IGP79	IgG1 BG1n/G1n	14	65822493	1	1.83E-12	1.10E-13	-	-0.247686	-0.2837	+	T	C	0.575741016	0.0348744	+	T	C	0.6042	0.0382
rs11621121	LC_IGP77	IgG1 Bn	14	65822493	1	2.27E-12	6.93E-13	-	-0.246706	-0.2755	+	T	C	0.575741016	0.034886	+	T	C	0.6042	0.0384
rs11621121	LC_IGP86	IgG1 Bn/Fn total	14	65822493	1	3.49E-13	2.55E-13	-	-0.255549	-0.2808	+	T	C	0.575741016	0.0348386	+	T	C	0.6042	0.0384
rs11621121	LC_IGP84	IgG1 FBn/Bn total	14	65822493	1	1.85E-13	1.35E-19	+	0.259031	0.35	+	T	C	0.575741016	0.0348958	+	T	C	0.6042	0.0386
rs11621121	LC_IGP62	IgG1 FG0n total/G0n	14	65822493	1	1.91E-15	1.10E-13	+	0.277786	0.2855	+	T	C	0.575741016	0.0346067	+	T	C	0.6042	0.0384
rs11621121	LC_IGP63	IgG1 FG1n total/G1n	14	65822493	1	3.79E-16	4.05E-17	+	0.285401	0.3233	+	T	C	0.575741016	0.0346745	+	T	C	0.6042	0.0384
rs11621121	LC_IGP64	IgG1 FG2n total/G2n	14	65822493	1	1.05E-12	2.34E-18	+	0.250462	0.3342	+	T	C	0.575741016	0.0348789	+	T	C	0.6042	0.0382
rs11621121	LC_IGP68	IgG1 FG2n/G2n	14	65822493	1	6.54E-10	6.07E-10	+	0.217531	0.2358	+	T	C	0.575741016	0.0350041	+	T	C	0.6042	0.0381
rs11621121	LC_IGP61	IgG1 Fn total	14	65822493	1	1.59E-15	1.68E-16	+	0.279191	0.3171	+	T	C	0.575741016	0.0346805	+	T	C	0.6042	0.0385
rs11621121	LC_IGP81	IgG1 Fn/Bn	14	65822493	1	8.67E-13	8.92E-12	+	0.251183	0.2607	+	T	C	0.575741016	0.0348464	+	T	C	0.6042	0.0382
rs11621121	LC_IGP21	IgG1 Fucosylation	14	65822493	1	1.22E-13	1.63E-15	+	0.259463	0.3062	+	T	C	0.575059186	0.0346929	+	T	C	0.6042	0.0384
rs11621121	LC_IGP28	IgG1 G1S1/(G1+G1S1)	14	65822493	1	1.61E-10	1.76E-11	+	0.223024	0.2482	+	T	C	0.575074006	0.0346518	+	T	C	0.6042	0.0369
rs11621121	LC_IGP11	IgG1_G0	14	65822493	1	9.09E-16	4.10E-15	-	-0.279248	-0.3004	+	T	C	0.574982285	0.0343905	+	T	C	0.6042	0.0382
rs11621121	LC_IGP_R1	IgG1_G0F/IgG1_G0	14	65822493	1	1.18E-14	6.69E-13	+	0.268197	0.2753	+	T	C	0.574982285	0.0344283	+	T	C	0.6042	0.0383
rs11621121	LC_IGP_R4	IgG1_G0FN/IgG1_G0N	14	65822493	1	4.07E-13	1.03E-15	+	0.253702	0.3096	+	T	C	0.575165952	0.0346911	+	T	C	0.6042	0.0386
rs11621121	LC_IGP52	IgG1_G0n	14	65822493	1	6.25E-16	3.18E-15	-	-0.282283	-0.3021	+	T	C	0.575741016	0.0345581	+	T	C	0.6042	0.0383
rs11621121	LC_IGP14	IgG1_G0N	14	65822493	1	5.03E-15	5.47E-12	-	-0.273315	-0.2627	+	T	C	0.575165952	0.0345906	+	T	C	0.6042	0.0381
rs11621121	LC_IGP55	IgG1_G0Nn	14	65822493	1	1.38E-14	5.04E-12	-	-0.269816	-0.2634	+	T	C	0.575741016	0.0347201	+	T	C	0.6042	0.0382
rs11621121	LC_IGP12	IgG1_G1	14	65822493	1	8.74E-15	1.64E-16	-	-0.270104	-0.3168	+	T	C	0.574982285	0.0344984	+	T	C	0.6042	0.0384
rs11621121	LC_IGP_R2	IgG1_G1F/IgG1_G1	14	65822493	1	8.70E-16	6.48E-16	+	0.28004	0.3099	+	T	C	0.574982285	0.0344646	+	T	C	0.6042	0.0384
rs11621121	LC_IGP_R5	IgG1_G1FN/IgG1_G1N	14	65822493	1	9.76E-14	3.62E-21	+	0.261019	0.3631	+	T	C	0.576104551	0.0347595	+	T	C	0.6042	0.0384
rs11621121	LC_IGP53	IgG1_G1n	14	65822493	1	2.36E-14	3.09E-16	-	-0.267465	-0.3136	+	T	C	0.575741016	0.0347349	+	T	C	0.6042	0.0384
rs11621121	LC_IGP15	IgG1_G1N	14	65822493	1	1.31E-11	1.22E-12	-	-0.237535	-0.2715	+	T	C	0.576104551	0.0348443	+	T	C	0.6042	0.0382
rs11621121	LC_IGP56	IgG1_G1Nn	14	65822493	1	1.78E-11	2.61E-12	-	-0.23653	-0.267	+	T	C	0.575741016	0.034929	+	T	C	0.6042	0.0382
rs11621121	LC_IGP_R32	IgG1_G1S1/IgG1_G1	14	65822493	1	2.02E-10	2.34E-11	+	0.221846	0.2469	+	T	C	0.575074006	0.0346618	+	T	C	0.6042	0.0369
rs11621121	LC_IGP13	IgG1_G2	14	65822493	1	4.65E-08	8.21E-14	-	-0.191377	-0.2823	+	T	C	0.574982285	0.0348589	+	T	C	0.6042	0.0378
rs11621121	LC_IGP_R3	IgG1_G2F/IgG1_G2	14	65822493	1	1.74E-13	4.46E-18	+	0.256995	0.3307	+	T	C	0.575028117	0.0345882	+	T	C	0.6042	0.0382
rs11621121	LC_IGP_R8	IgG1_G2FS1/IgG1_G2S1	14	65822493	1	6.73E-09	1.62E-15	+	0.20276	0.3032	+	T	C	0.574676848	0.0347891	+	T	C	0.6042	0.0381
rs11621121	LC_IGP_R26	IgG1_G2N/IgG1_G1N	14	65822493	1	3.87E-09	1.41E-10	+	0.206317	0.2392	+	T	C	0.576077778	0.034838	+	T	C	0.6042	0.0373
rs60488013	LC_IGP78	IgG1 BG0n/G0n	14	65825854	1	7.41E-12	1.03E-09	+	0.263832	0.2702	+	A	C	0.283396221	0.038232	+	A	C	0.2458	0.0443
rs60488013	LC_IGP79	IgG1 BG1n/G1n	14	65825854	1	3.57E-11	5.40E-11	+	0.255743	0.2902	+	A	C	0.283396221	0.0383575	+	A	C	0.2458	0.0442
rs60488013	LC_IGP31	IgG1 BGS1/(BG0+BG+BGS1)	14	65825854	1	2.09E-08	3.17E-10	-	-0.215409	-0.2756	+	A	C	0.282669264	0.0382402	+	A	C	0.2458	0.0438
rs60488013	LC_IGP77	IgG1 Bn	14	65825854	1	1.26E-11	1.51E-10	+	0.261598	0.2846	+	A	C	0.283396221	0.0383409	+	A	C	0.2458	0.0444
rs60488013	LC_IGP86	IgG1 Bn/Fn total	14	65825854	1	1.69E-12	7.33E-11	+	0.272354	0.2896	+	A	C	0.283396221	0.0382858	+	A	C	0.2458	0.0445
rs60488013	LC_IGP84	IgG1 FBn/Bn total	14	65825854	1	6.99E-14	2.64E-15	-	-0.289266	-0.354	+	A	C	0.283396221	0.0382891	+	A	C	0.2458	0.0448
rs60488013	LC_IGP62	IgG1 FG0n total/G0n	14	65825854	1	3.65E-17	4.39E-12	-	-0.322876	-0.3081	+	A	C	0.283396221	0.0379025	+	A	C	0.2458	0.0445
rs60488013	LC_IGP63	IgG1 FG1n total/G1n	14	65825854	1	1.25E-14	1.35E-13	-	-0.296943	-0.3292	+	A	C	0.283396221	0.0381502	+	A	C	0.2458	0.0445
rs60488013	LC_IGP64	IgG1 FG2n total/G2n	14	65825854	1	2.57E-13	5.37E-15	-	-0.282258	-0.3461	+	A	C	0.283396221	0.0382604	+	A	C	0.2458	0.0443
rs60488013	LC_IGP68	IgG1 FG2n/G2n	14	65825854	1	6.26E-10	8.80E-09	-	-0.239092	-0.2539	+	A	C	0.283396221	0.0384296	+	A	C	0.2458	0.0441
rs60488013	LC_IGP61	IgG1 Fn total	14	65825854	1	9.98E-16	1.60E-13	-	-0.308675	-0.3285	+	A	C	0.283396221	0.0380646	+	A	C	0.2458	0.0445
rs60488013	LC_IGP81	IgG1 Fn/Bn	14	65825854	1	7.69E-12	4.36E-10	-	-0.264147	-0.2762	+	A	C	0.283396221	0.0383084	+	A	C	0.2458	0.0442
rs60488013	LC_IGP21	IgG1 Fucosylation	14	65825854	1	5.61E-14	2.38E-13	-	-0.288518	-0.3262	+	A	C	0.283539766	0.0380423	+	A	C	0.2458	0.0445
rs60488013	LC_IGP28	IgG1 G1S1/(G1+G1S1)	14	65825854	1	1.98E-09	1.22E-11	-	-0.229634	-0.2892	+	A	C	0.283425994	0.0380579	+	A	C	0.2458	0.0427
rs60488013	LC_IGP27	IgG1 GS1/(G0+G+GS1)	14	65825854	1	1.29E-08	1.69E-08	-	-0.218059	-0.2451	+	A	C	0.283425994	0.0381467	+	A	C	0.2458	0.0434
rs60488013	LC_IGP11	IgG1_G0	14	65825854	1	2.81E-19	2.84E-13	+	0.340995	0.3234	+	A	C	0.283385156	0.0375176	+	A	C	0.2458	0.0443
rs60488013	LC_IGP_R1	IgG1_G0F/IgG1_G0	14	65825854	1	2.84E-17	1.68E-11	-	-0.321433	-0.2986	+	A	C	0.283385156	0.0376055	+	A	C	0.2458	0.0444
rs60488013	LC_IGP_R4	IgG1_G0FN/IgG1_G0N	14	65825854	1	4.52E-13	1.69E-13	-	-0.277516	-0.3295	+	A	C	0.283466932	0.038022	+	A	C	0.2458	0.0447
rs60488013	LC_IGP52	IgG1_G0n	14	65825854	1	3.21E-19	2.80E-13	+	0.342744	0.3242	+	A	C	0.283396221	0.0377655	+	A	C	0.2458	0.0444
rs60488013	LC_IGP14	IgG1_G0N	14	65825854	1	5.72E-14	2.37E-10	+	0.287819	0.2799	+	A	C	0.283466932	0.0379653	+	A	C	0.2458	0.0442
rs60488013	LC_IGP55	IgG1_G0Nn	14	65825854	1	9.85E-14	2.60E-10	+	0.286561	0.2795	+	A	C	0.283396221	0.0381647	+	A	C	0.2458	0.0442
rs60488013	LC_IGP12	IgG1_G1	14	65825854	1	3.28E-15	1.45E-13	+	0.300763	0.3285	+	A	C	0.283690593	0.0378008	+	A	C	0.2458	0.0445
rs60488013	LC_IGP_R2	IgG1_G1F/IgG1_G1	14	65825854	1	5.44E-15	6.43E-13	-	-0.298508	-0.3192	+	A	C	0.283690593	0.0378278	+	A	C	0.2458	0.0444
rs60488013	LC_IGP_R5	IgG1_G1FN/IgG1_G1N	14	65825854	1	6.81E-13	2.78E-16	-	-0.276364	-0.3644	+	A	C	0.282842251	0.0381625	+	A	C	0.2458	0.0445
rs60488013	LC_IGP53	IgG1_G1n	14	65825854	1	1.00E-13	3.13E-13	+	0.286511	0.3237	+	A	C	0.283396221	0.0381688	+	A	C	0.2458	0.0444
rs60488013	LC_IGP15	IgG1_G1N	14	65825854	1	2.18E-10	1.22E-10	+	0.244525	0.2849	+	A	C	0.282842251	0.0382756	+	A	C	0.2458	0.0443
rs60488013	LC_IGP_SC15	IgG1_G1N(20)/IgG2_G1N(20)	14	65825854	1	1.04E-08	7.15E-07	+	0.219784	0.2099	+	A	C	0.282882697	0.0381947	+	A	C	0.2458	0.0424
rs60488013	LC_IGP56	IgG1_G1Nn	14	65825854	1	1.55E-10	3.04E-10	+	0.247375	0.2783	+	A	C	0.283396221	0.0383987	+	A	C	0.2458	0.0442
rs60488013	LC_IGP_R32	IgG1_G1S1/IgG1_G1	14	65825854	1	2.57E-09	1.83E-11	-	-0.22806	-0.2871	+	A	C	0.283425994	0.0380695	+	A	C	0.2458	0.0427
rs60488013	LC_IGP_R3	IgG1_G2F/IgG1_G2	14	65825854	1	5.75E-14	4.24E-15	-	-0.287266	-0.3467	+	A	C	0.283252751	0.0378973	+	A	C	0.2458	0.0442
rs60488013	LC_IGP_R8	IgG1_G2FS1/IgG1_G2S1	14	65825854	1	1.69E-09	6.74E-14	-	-0.231103	-0.3298	+	A	C	0.283385156	0.0381371	+	A	C	0.2458	0.044
rs60488013	LC_IGP_R26	IgG1_G2N/IgG1_G1N	14	65825854	1	2.95E-08	1.58E-10	-	-0.213257	-0.2763	+	A	C	0.282963889	0.0382741	+	A	C	0.2458	0.0432
rs60488013	LC_IGP97	IgG2_G0	14	65825854	1	6.47E-10	3.37E-09	+	0.236276	0.2611	+	A	C	0.283252751	0.0380124	+	A	C	0.2458	0.0442
rs60488013	LC_IGP_R41	IgG2_G0F/IgG2_G0	14	65825854	1	4.39E-08	3.32E-09	-	-0.209545	-0.2593	+	A	C	0.283252751	0.0380952	+	A	C	0.2458	0.0438
rs60488013	LC_IGP138	IgG2_G0n	14	65825854	1	4.53E-10	3.60E-09	+	0.239132	0.2608	+	A	C	0.283190579	0.0381211	+	A	C	0.2459	0.0442
rs7152441	LC_IGP78	IgG1 BG0n/G0n	14	65827903	1	1.70E-13	3.75E-09	-	-0.26254	-0.2309	+	A	G	0.454128872	0.0353134	+	A	G	0.4706	0.0392
rs7152441	LC_IGP79	IgG1 BG1n/G1n	14	65827903	1	5.83E-14	1.56E-10	-	-0.268095	-0.2503	+	A	G	0.454128872	0.0353724	+	A	G	0.4706	0.0391
rs7152441	LC_IGP32	IgG1 BG1S1/(BG1+BG1S1)	14	65827903	1	4.56E-09	1.16E-11	+	0.208049	0.2598	+	A	G	0.454574291	0.0352941	+	A	G	0.4706	0.0383
rs7152441	LC_IGP31	IgG1 BGS1/(BG0+BG+BGS1)	14	65827903	1	4.29E-08	4.55E-11	+	0.194925	0.2552	+	A	G	0.455182668	0.0354085	+	A	G	0.4706	0.0388
rs7152441	LC_IGP77	IgG1 Bn	14	65827903	1	1.45E-13	2.13E-10	-	-0.263937	-0.2498	+	A	G	0.454128872	0.0353992	+	A	G	0.4706	0.0393
rs7152441	LC_IGP86	IgG1 Bn/Fn total	14	65827903	1	1.97E-14	1.07E-10	-	-0.273034	-0.254	+	A	G	0.454128872	0.0353484	+	A	G	0.4706	0.0393
rs7152441	LC_IGP84	IgG1 FBn/Bn total	14	65827903	1	5.82E-15	9.76E-16	+	0.279025	0.3174	+	A	G	0.454128872	0.0353938	+	A	G	0.4706	0.0395
rs7152441	LC_IGP62	IgG1 FG0n total/G0n	14	65827903	1	2.68E-17	6.68E-12	+	0.300173	0.2708	+	A	G	0.454128872	0.035083	+	A	G	0.4706	0.0394
rs7152441	LC_IGP63	IgG1 FG1n total/G1n	14	65827903	1	1.14E-18	1.28E-13	+	0.313584	0.2916	+	A	G	0.454128872	0.0351185	+	A	G	0.4706	0.0394
rs7152441	LC_IGP64	IgG1 FG2n total/G2n	14	65827903	1	6.72E-16	1.15E-14	+	0.287958	0.302	+	A	G	0.454128872	0.0352926	+	A	G	0.4706	0.0391
rs7152441	LC_IGP68	IgG1 FG2n/G2n	14	65827903	1	3.43E-12	1.05E-07	+	0.248699	0.2078	+	A	G	0.454128872	0.0354654	+	A	G	0.4706	0.0391
rs7152441	LC_IGP61	IgG1 Fn total	14	65827903	1	1.82E-17	1.85E-13	+	0.302409	0.2901	+	A	G	0.454128872	0.0351535	+	A	G	0.4706	0.0394
rs7152441	LC_IGP81	IgG1 Fn/Bn	14	65827903	1	5.66E-14	1.59E-09	+	0.268137	0.2366	+	A	G	0.454128872	0.0353595	+	A	G	0.4706	0.0392
rs7152441	LC_IGP21	IgG1 Fucosylation	14	65827903	1	1.12E-15	1.42E-12	+	0.284508	0.2789	+	A	G	0.453084155	0.0351491	+	A	G	0.4706	0.0394
rs7152441	LC_IGP28	IgG1 G1S1/(G1+G1S1)	14	65827903	1	8.87E-12	8.91E-10	+	0.241699	0.2311	+	A	G	0.453217431	0.0351625	+	A	G	0.4706	0.0377
rs7152441	LC_IGP26	IgG1 GS1/(G+GS1)	14	65827903	1	3.68E-09	1.47E-09	+	0.209775	0.2334	+	A	G	0.453217431	0.0353737	+	A	G	0.4706	0.0386
rs7152441	LC_IGP27	IgG1 GS1/(G0+G+GS1)	14	65827903	1	2.23E-10	3.23E-08	+	0.225224	0.212	+	A	G	0.453217431	0.0352732	+	A	G	0.4706	0.0384
rs7152441	LC_IGP11	IgG1_G0	14	65827903	1	6.52E-20	4.21E-14	-	-0.321431	-0.296	+	A	G	0.453546732	0.0347317	+	A	G	0.4706	0.0392
rs7152441	LC_IGP_R1	IgG1_G0F/IgG1_G0	14	65827903	1	1.11E-16	1.85E-11	+	0.292274	0.2644	+	A	G	0.453546732	0.0348726	+	A	G	0.4706	0.0394
rs7152441	LC_IGP_R4	IgG1_G0FN/IgG1_G0N	14	65827903	1	8.91E-14	5.01E-13	+	0.264563	0.2855	+	A	G	0.454003062	0.035175	+	A	G	0.4706	0.0395
rs7152441	LC_IGP52	IgG1_G0n	14	65827903	1	5.37E-20	4.24E-14	-	-0.324033	-0.2967	+	A	G	0.454128872	0.0349245	+	A	G	0.4706	0.0393
rs7152441	LC_IGP14	IgG1_G0N	14	65827903	1	5.67E-17	1.79E-10	-	-0.296328	-0.2488	+	A	G	0.454003062	0.0350102	+	A	G	0.4706	0.039
rs7152441	LC_IGP55	IgG1_G0Nn	14	65827903	1	5.39E-17	2.08E-10	-	-0.297936	-0.2483	+	A	G	0.454128872	0.0351707	+	A	G	0.4706	0.0391
rs7152441	LC_IGP12	IgG1_G1	14	65827903	1	1.12E-16	2.93E-13	-	-0.292906	-0.2871	+	A	G	0.453572694	0.03495	+	A	G	0.4706	0.0394
rs7152441	LC_IGP_R2	IgG1_G1F/IgG1_G1	14	65827903	1	2.15E-18	1.06E-12	+	0.308825	0.2799	+	A	G	0.453572694	0.0348811	+	A	G	0.4706	0.0393
rs7152441	LC_IGP_R5	IgG1_G1FN/IgG1_G1N	14	65827903	1	2.21E-15	1.16E-16	+	0.282083	0.3258	+	A	G	0.454680504	0.0352279	+	A	G	0.4706	0.0393
rs7152441	LC_IGP53	IgG1_G1n	14	65827903	1	6.91E-16	5.71E-13	-	-0.287316	-0.2834	+	A	G	0.454128872	0.035229	+	A	G	0.4706	0.0393
rs7152441	LC_IGP15	IgG1_G1N	14	65827903	1	2.15E-12	1.01E-09	-	-0.250285	-0.2392	+	A	G	0.454680504	0.0353565	+	A	G	0.4706	0.0392
rs7152441	LC_IGP56	IgG1_G1Nn	14	65827903	1	3.44E-12	2.11E-09	-	-0.248701	-0.2343	+	A	G	0.454128872	0.0354669	+	A	G	0.4706	0.0391
rs7152441	LC_IGP_R36	IgG1_G1NS1/IgG1_G1N	14	65827903	1	4.69E-09	1.30E-11	+	0.207929	0.2594	+	A	G	0.454574291	0.035303	+	A	G	0.4706	0.0383
rs7152441	LC_IGP_R32	IgG1_G1S1/IgG1_G1	14	65827903	1	1.64E-11	1.33E-09	+	0.238626	0.2288	+	A	G	0.453217431	0.0351808	+	A	G	0.4706	0.0377
rs7152441	LC_IGP13	IgG1_G2	14	65827903	1	3.49E-08	2.10E-10	-	-0.196181	-0.2457	+	A	G	0.453546732	0.0354022	+	A	G	0.4706	0.0387
rs7152441	LC_IGP_R3	IgG1_G2F/IgG1_G2	14	65827903	1	3.40E-17	3.06E-14	+	0.29822	0.2966	+	A	G	0.453212714	0.0349789	+	A	G	0.4706	0.039
rs7152441	LC_IGP_R8	IgG1_G2FS1/IgG1_G2S1	14	65827903	1	2.77E-10	2.29E-12	+	0.224226	0.2728	+	A	G	0.452935858	0.0353067	+	A	G	0.4706	0.0389
rs7152441	LC_IGP_R26	IgG1_G2N/IgG1_G1N	14	65827903	1	1.42E-10	3.77E-08	+	0.228026	0.2097	+	A	G	0.454868827	0.035319	+	A	G	0.4706	0.0381
rs7152441	LC_IGP148	IgG2 FG0n total/G0n	14	65827903	1	1.32E-08	1.54E-13	+	0.202629	0.2876	+	A	G	0.45269766	0.0354692	+	A	G	0.4706	0.039
rs7152441	LC_IGP97	IgG2_G0	14	65827903	1	3.25E-10	2.41E-13	-	-0.22285	-0.2857	+	A	G	0.453183374	0.0352302	+	A	G	0.4706	0.039
rs7152441	LC_IGP138	IgG2_G0n	14	65827903	1	2.18E-10	1.76E-13	-	-0.225827	-0.2878	+	A	G	0.45269766	0.0353489	+	A	G	0.4706	0.0391
rs7152441	LC_IGP_R64	IgG2_G2/IgG2_G1	14	65827903	1	3.30E-08	1.63E-11	+	0.197434	0.2572	+	A	G	0.452546512	0.035565	+	A	G	0.4706	0.0382
rs7157006	LC_IGP78	IgG1 BG0n/G0n	14	65828207	1	2.02E-12	5.48E-11	-	-0.24565	-0.2506	+	T	C	0.580822181	0.0346563	+	T	C	0.6054	0.0382

rs7157006	LC_IGP79	IgG1 BG1n/G1n	14	65828207	1	5.29E-12	1.13E-13	-	-0.241561	-0.2837	+	T	C	0.580822181	0.0347572	+	T	C	0.6054	0.0382
rs7157006	LC_IGP77	IgG1 Bn	14	65828207	1	6.28E-12	7.09E-13	-	-0.240763	-0.2755	+	T	C	0.580822181	0.0347678	+	T	C	0.6054	0.0384
rs7157006	LC_IGP86	IgG1 Bn/Fn total	14	65828207	1	9.35E-13	2.67E-13	-	-0.249905	-0.2807	+	T	C	0.580822181	0.03472	+	T	C	0.6054	0.0384
rs7157006	LC_IGP84	IgG1 FBn/Bn total	14	65828207	1	2.57E-13	1.56E-19	+	0.256466	0.3495	+	T	C	0.580822181	0.0347631	+	T	C	0.6054	0.0387
rs7157006	LC_IGP62	IgG1 FG0n total/G0n	14	65828207	1	3.64E-15	1.33E-13	+	0.273931	0.2846	+	T	C	0.580822181	0.0344817	+	T	C	0.6054	0.0384
rs7157006	LC_IGP63	IgG1 FG1n total/G1n	14	65828207	1	4.90E-16	5.12E-17	+	0.283195	0.3222	+	T	C	0.580822181	0.0345411	+	T	C	0.6054	0.0384
rs7157006	LC_IGP64	IgG1 FG2n total/G2n	14	65828207	1	8.08E-13	2.76E-18	+	0.250714	0.3335	+	T	C	0.580822181	0.0347337	+	T	C	0.6054	0.0382
rs7157006	LC_IGP68	IgG1 FG2n/G2n	14	65828207	1	1.09E-09	5.95E-10	+	0.213867	0.2359	+	T	C	0.580822181	0.0348746	+	T	C	0.6054	0.0381
rs7157006	LC_IGP61	IgG1 Fn total	14	65828207	1	2.03E-15	2.08E-16	+	0.277036	0.3161	+	T	C	0.580822181	0.0345468	+	T	C	0.6054	0.0385
rs7157006	LC_IGP81	IgG1 Fn/Bn	14	65828207	1	2.62E-12	9.08E-12	+	0.244888	0.2607	+	T	C	0.580822181	0.0347302	+	T	C	0.6054	0.0382
rs7157006	LC_IGP21	IgG1 Fucosylation	14	65828207	1	1.44E-13	1.87E-15	+	0.257681	0.3055	+	T	C	0.58011529	0.034557	+	T	C	0.6054	0.0384
rs7157006	LC_IGP28	IgG1 G1S1/(G1+G1S1)	14	65828207	1	4.43E-10	3.06E-11	+	0.216756	0.2451	+	T	C	0.580089908	0.0345346	+	T	C	0.6054	0.0369
rs7157006	LC_IGP11	IgG1_G0	14	65828207	1	3.27E-15	5.64E-15	-	-0.272644	-0.2989	+	T	C	0.580297495	0.0342655	+	T	C	0.6054	0.0383
rs7157006	LC_IGP_R1	IgG1_G0F/IgG1_G0	14	65828207	1	5.22E-14	8.25E-13	+	0.260504	0.2743	+	T	C	0.580297495	0.0343073	+	T	C	0.6054	0.0383
rs7157006	LC_IGP_R4	IgG1_G0FN/IgG1_G0N	14	65828207	1	6.87E-13	1.13E-15	+	0.250143	0.3092	+	T	C	0.580494182	0.0345487	+	T	C	0.6054	0.0386
rs7157006	LC_IGP52	IgG1_G0n	14	65828207	1	1.25E-15	4.32E-15	-	-0.278248	-0.3007	+	T	C	0.580822181	0.0344344	+	T	C	0.6054	0.0383
rs7157006	LC_IGP14	IgG1_G0N	14	65828207	1	3.30E-14	6.00E-12	-	-0.263902	-0.2624	+	T	C	0.580494182	0.034477	+	T	C	0.6054	0.0381
rs7157006	LC_IGP55	IgG1_G0Nn	14	65828207	1	4.26E-14	5.46E-12	-	-0.263739	-0.2631	+	T	C	0.580822181	0.034605	+	T	C	0.6054	0.0382
rs7157006	LC_IGP12	IgG1_G1	14	65828207	1	1.79E-14	2.21E-16	-	-0.265797	-0.3154	+	T	C	0.580297495	0.0343614	+	T	C	0.6054	0.0384
rs7157006	LC_IGP_R2	IgG1_G1F/IgG1_G1	14	65828207	1	2.66E-15	8.18E-16	+	0.27412	0.3088	+	T	C	0.580297495	0.034336	+	T	C	0.6054	0.0384
rs7157006	LC_IGP_R5	IgG1_G1FN/IgG1_G1N	14	65828207	1	1.44E-13	4.41E-21	+	0.258216	0.3624	+	T	C	0.581148216	0.0346297	+	T	C	0.6054	0.0385
rs7157006	LC_IGP53	IgG1_G1n	14	65828207	1	2.14E-14	4.06E-16	-	-0.266827	-0.3124	+	T	C	0.580822181	0.0345938	+	T	C	0.6054	0.0384
rs7157006	LC_IGP15	IgG1_G1N	14	65828207	1	3.38E-11	1.27E-12	-	-0.231803	-0.2714	+	T	C	0.581148216	0.034726	+	T	C	0.6054	0.0382
rs7157006	LC_IGP56	IgG1_G1Nn	14	65828207	1	4.71E-11	2.68E-12	-	-0.230628	-0.267	+	T	C	0.580822181	0.0348097	+	T	C	0.6054	0.0382
rs7157006	LC_IGP_R32	IgG1_G1S1/IgG1_G1	14	65828207	1	5.54E-10	4.09E-11	+	0.215579	0.2438	+	T	C	0.580089908	0.0345444	+	T	C	0.6054	0.0369
rs7157006	LC_IGP13	IgG1_G2	14	65828207	1	4.56E-08	9.44E-14	-	-0.190654	-0.2816	+	T	C	0.580297495	0.034705	+	T	C	0.6054	0.0378
rs7157006	LC_IGP_R3	IgG1_G2F/IgG1_G2	14	65828207	1	1.17E-13	5.29E-18	+	0.25777	0.33	+	T	C	0.580040954	0.0344421	+	T	C	0.6054	0.0382
rs7157006	LC_IGP_R8	IgG1_G2FS1/IgG1_G2S1	14	65828207	1	4.30E-09	1.98E-15	+	0.204524	0.3023	+	T	C	0.579686622	0.0346409	+	T	C	0.6054	0.0381
rs7157006	LC_IGP_R26	IgG1_G2N/IgG1_G1N	14	65828207	1	7.59E-09	1.69E-10	+	0.201624	0.2383	+	T	C	0.581140123	0.0347136	+	T	C	0.6054	0.0373
rs7157006	LC_IGP138	IgG2_G0n	14	65828207	1	4.59E-08	2.88E-16	-	-0.1907	-0.3121	+	T	C	0.580016626	0.0347204	+	T	C	0.6053	0.0382
rs11628611	LC_IGP79	IgG1 BG1n/G1n	14	65828525	1	2.09E-10	3.32E-07	-	-0.284189	-0.297	+	T	C	0.294709418	0.044436	+	T	C	0.2863	0.0582
rs11628611	LC_IGP77	IgG1 Bn	14	65828525	1	2.52E-09	8.43E-07	-	-0.266818	-0.2888	+	T	C	0.294709418	0.0445119	+	T	C	0.2863	0.0586
rs11628611	LC_IGP86	IgG1 Bn/Fn total	14	65828525	1	9.24E-10	6.37E-07	-	-0.273912	-0.2918	+	T	C	0.294709418	0.0444753	+	T	C	0.2863	0.0586
rs11628611	LC_IGP84	IgG1 FBn/Bn total	14	65828525	1	5.81E-10	6.82E-10	+	0.277729	0.3625	+	T	C	0.294709418	0.044553	+	T	C	0.2863	0.0588
rs11628611	LC_IGP62	IgG1 FG0n total/G0n	14	65828525	1	3.24E-10	6.60E-07	+	0.280211	0.2944	+	T	C	0.294709418	0.0442918	+	T	C	0.2863	0.0592
rs11628611	LC_IGP63	IgG1 FG1n total/G1n	14	65828525	1	1.56E-11	1.37E-08	+	0.301136	0.3343	+	T	C	0.294709418	0.0443406	+	T	C	0.2863	0.0589
rs11628611	LC_IGP64	IgG1 FG2n total/G2n	14	65828525	1	3.78E-09	6.87E-10	+	0.263933	0.3616	+	T	C	0.294709418	0.0445347	+	T	C	0.2863	0.0586
rs11628611	LC_IGP61	IgG1 Fn total	14	65828525	1	4.56E-10	2.33E-08	+	0.278487	0.3299	+	T	C	0.294709418	0.0444004	+	T	C	0.2863	0.0591
rs11628611	LC_IGP81	IgG1 Fn/Bn	14	65828525	1	1.53E-09	1.79E-06	+	0.270262	0.2803	+	T	C	0.294709418	0.044474	+	T	C	0.2863	0.0587
rs11628611	LC_IGP21	IgG1 Fucosylation	14	65828525	1	1.02E-08	8.77E-08	+	0.255334	0.3164	+	T	C	0.293918002	0.044351	+	T	C	0.2863	0.0591
rs11628611	LC_IGP11	IgG1_G0	14	65828525	1	1.42E-12	1.36E-08	-	-0.313163	-0.3346	+	T	C	0.294120953	0.0438747	+	T	C	0.2863	0.0589
rs11628611	LC_IGP_R1	IgG1_G0F/IgG1_G0	14	65828525	1	4.27E-10	1.16E-06	+	0.276444	0.2875	+	T	C	0.294120953	0.0440052	+	T	C	0.2863	0.0591
rs11628611	LC_IGP_R4	IgG1_G0FN/IgG1_G0N	14	65828525	1	7.96E-09	1.02E-07	+	0.257126	0.3149	+	T	C	0.293931108	0.0443344	+	T	C	0.2863	0.0592
rs11628611	LC_IGP52	IgG1_G0n	14	65828525	1	3.17E-12	1.45E-08	-	-0.309997	-0.3346	+	T	C	0.294709418	0.0441357	+	T	C	0.2863	0.059
rs11628611	LC_IGP14	IgG1_G0N	14	65828525	1	1.70E-11	1.83E-06	-	-0.299293	-0.2792	+	T	C	0.293931108	0.0441605	+	T	C	0.2863	0.0585
rs11628611	LC_IGP55	IgG1_G0Nn	14	65828525	1	1.50E-11	2.11E-06	-	-0.301119	-0.2776	+	T	C	0.294709418	0.0443006	+	T	C	0.2863	0.0585
rs11628611	LC_IGP12	IgG1_G1	14	65828525	1	1.67E-09	3.05E-08	-	-0.267521	-0.326	+	T	C	0.294223274	0.0441342	+	T	C	0.2863	0.0589
rs11628611	LC_IGP_R2	IgG1_G1F/IgG1_G1	14	65828525	1	1.07E-10	6.14E-08	+	0.286458	0.3193	+	T	C	0.294223274	0.04408	+	T	C	0.2863	0.059
rs11628611	LC_IGP_R5	IgG1_G1FN/IgG1_G1N	14	65828525	1	1.39E-11	8.52E-11	+	0.301643	0.3785	+	T	C	0.294398831	0.0443116	+	T	C	0.2863	0.0583
rs11628611	LC_IGP53	IgG1_G1n	14	65828525	1	6.41E-09	5.32E-08	-	-0.25956	-0.3198	+	T	C	0.294709418	0.0444679	+	T	C	0.2863	0.0588
rs11628611	LC_IGP15	IgG1_G1N	14	65828525	1	3.27E-09	1.00E-06	-	-0.264397	-0.285	+	T	C	0.294398831	0.0444342	+	T	C	0.2863	0.0583
rs11628611	LC_IGP56	IgG1_G1Nn	14	65828525	1	8.35E-09	1.77E-06	-	-0.257991	-0.2778	+	T	C	0.294709418	0.0445434	+	T	C	0.2863	0.0581
rs11628611	LC_IGP_R3	IgG1_G2F/IgG1_G2	14	65828525	1	1.28E-09	1.15E-09	+	0.26975	0.3556	+	T	C	0.293944071	0.0441812	+	T	C	0.2863	0.0584
rs7158347	LC_IGP78	IgG1 BG0n/G0n	14	65828955	1	2.08E-12	9.76E-11	-	-0.245391	-0.2475	+	T	C	0.580855019	0.0346401	+	T	C	0.6061	0.0382
rs7158347	LC_IGP79	IgG1 BG1n/G1n	14	65828955	1	5.57E-12	1.93E-13	-	-0.241184	-0.2813	+	T	C	0.580855019	0.0347414	+	T	C	0.6061	0.0382
rs7158347	LC_IGP77	IgG1 Bn	14	65828955	1	6.55E-12	1.21E-12	-	-0.240437	-0.273	+	T	C	0.580855019	0.0347518	+	T	C	0.6061	0.0384
rs7158347	LC_IGP86	IgG1 Bn/Fn total	14	65828955	1	9.76E-13	4.87E-13	-	-0.24958	-0.2778	+	T	C	0.580855019	0.034704	+	T	C	0.6061	0.0384
rs7158347	LC_IGP84	IgG1 FBn/Bn total	14	65828955	1	2.64E-13	4.50E-19	+	0.256221	0.3449	+	T	C	0.580855019	0.0347467	+	T	C	0.6061	0.0386
rs7158347	LC_IGP62	IgG1 FG0n total/G0n	14	65828955	1	3.76E-15	3.31E-13	+	0.273654	0.2798	+	T	C	0.580855019	0.0344657	+	T	C	0.6061	0.0384
rs7158347	LC_IGP63	IgG1 FG1n total/G1n	14	65828955	1	5.09E-16	1.30E-16	+	0.282903	0.3181	+	T	C	0.580855019	0.034525	+	T	C	0.6061	0.0384
rs7158347	LC_IGP64	IgG1 FG2n total/G2n	14	65828955	1	8.09E-13	6.97E-18	+	0.250585	0.3295	+	T	C	0.580855019	0.0347168	+	T	C	0.6061	0.0382
rs7158347	LC_IGP68	IgG1 FG2n/G2n	14	65828955	1	1.10E-09	9.56E-10	+	0.213676	0.2333	+	T	C	0.580855019	0.034858	+	T	C	0.6061	0.0381
rs7158347	LC_IGP61	IgG1 Fn total	14	65828955	1	2.09E-15	5.30E-16	+	0.276764	0.3118	+	T	C	0.580855019	0.0345307	+	T	C	0.6061	0.0385
rs7158347	LC_IGP81	IgG1 Fn/Bn	14	65828955	1	2.74E-12	1.41E-11	+	0.244553	0.2586	+	T	C	0.580855019	0.0347143	+	T	C	0.6061	0.0383
rs7158347	LC_IGP21	IgG1 Fucosylation	14	65828955	1	1.45E-13	4.29E-15	+	0.257509	0.3016	+	T	C	0.580147965	0.0345405	+	T	C	0.6061	0.0384
rs7158347	LC_IGP28	IgG1 G1S1/(G1+G1S1)	14	65828955	1	4.66E-10	5.35E-11	+	0.216372	0.2423	+	T	C	0.580122324	0.0345191	+	T	C	0.6061	0.0369
rs7158347	LC_IGP11	IgG1_G0	14	65828955	1	3.39E-15	1.32E-14	-	-0.272362	-0.2948	+	T	C	0.580329872	0.0342498	+	T	C	0.6061	0.0383
rs7158347	LC_IGP_R1	IgG1_G0F/IgG1_G0	14	65828955	1	5.40E-14	1.94E-12	+	0.260228	0.2697	+	T	C	0.580329872	0.0342915	+	T	C	0.6061	0.0383
rs7158347	LC_IGP_R4	IgG1_G0FN/IgG1_G0N	14	65828955	1	6.94E-13	3.12E-15	+	0.249972	0.3042	+	T	C	0.580526638	0.0345322	+	T	C	0.6061	0.0386
rs7158347	LC_IGP52	IgG1_G0n	14	65828955	1	1.30E-15	1.01E-14	-	-0.277961	-0.2967	+	T	C	0.580855019	0.0344184	+	T	C	0.6061	0.0383
rs7158347	LC_IGP14	IgG1_G0N	14	65828955	1	3.42E-14	9.51E-12	-	-0.263621	-0.2602	+	T	C	0.580526638	0.034461	+	T	C	0.6061	0.0382
rs7158347	LC_IGP55	IgG1_G0Nn	14	65828955	1	4.40E-14	8.60E-12	-	-0.263463	-0.261	+	T	C	0.580855019	0.0345888	+	T	C	0.6061	0.0382
rs7158347	LC_IGP12	IgG1_G1	14	65828955	1	1.83E-14	6.30E-16	-	-0.265583	-0.3104	+	T	C	0.580329872	0.0343453	+	T	C	0.6061	0.0384
rs7158347	LC_IGP_R2	IgG1_G1F/IgG1_G1	14	65828955	1	2.75E-15	1.95E-15	+	0.273847	0.3048	+	T	C	0.580329872	0.0343202	+	T	C	0.6061	0.0384
rs7158347	LC_IGP_R5	IgG1_G1FN/IgG1_G1N	14	65828955	1	1.49E-13	1.24E-20	+	0.257932	0.3582	+	T	C	0.581180812	0.0346136	+	T	C	0.6061	0.0385
rs7158347	LC_IGP53	IgG1_G1n	14	65828955	1	2.18E-14	1.14E-15	-	-0.266616	-0.3074	+	T	C	0.580855019	0.0345773	+	T	C	0.6061	0.0384
rs7158347	LC_IGP15	IgG1_G1N	14	65828955	1	3.54E-11	2.27E-12	-	-0.231451	-0.2685	+	T	C	0.581180812	0.0347101	+	T	C	0.6061	0.0383
rs7158347	LC_IGP56	IgG1_G1Nn	14	65828955	1	4.93E-11	4.72E-12	-	-0.230274	-0.2642	+	T	C	0.580855019	0.0347938	+	T	C	0.6061	0.0382
rs7158347	LC_IGP_R32	IgG1_G1S1/IgG1_G1	14	65828955	1	5.83E-10	7.42E-11	+	0.215204	0.2408	+	T	C	0.580122324	0.0345288	+	T	C	0.6061	0.037
rs7158347	LC_IGP13	IgG1_G2	14	65828955	1	4.59E-08	2.11E-13	-	-0.19053	-0.2776	+	T	C	0.580329872	0.0346884	+	T	C	0.6061	0.0378
rs7158347	LC_IGP_R3	IgG1_G2F/IgG1_G2	14	65828955	1	1.17E-13	1.33E-17	+	0.25765	0.326	+	T	C	0.58007335	0.0344255	+	T	C	0.6061	0.0382
rs7158347	LC_IGP_R8	IgG1_G2FS1/IgG1_G2S1	14	65828955	1	4.25E-09	4.52E-15	+	0.204502	0.2984	+	T	C	0.579718998	0.034624	+	T	C	0.6061	0.0381
rs7158347	LC_IGP_R26	IgG1_G2N/IgG1_G1N	14	65828955	1	7.82E-09	3.10E-10	+	0.201347	0.235	+	T	C	0.58117284	0.0346974	+	T	C	0.6061	0.0373
rs7158347	LC_IGP138	IgG2_G0n	14	65828955	1	4.62E-08	5.52E-16	-	-0.190572	-0.3094	+	T	C	0.580049261	0.0347037	+	T	C	0.606	0.0382
rs139251867	LC_IGP84	IgG1 FBn/Bn total	14	65829552	1	4.58E-08	1.73E-06	-	-0.238896	-0.2458	+	C	T	0.201385442	0.0434884	+	C	T	0.1706	0.0514
rs139251867	LC_IGP12	IgG1_G1	14	65829552	1	4.07E-08	1.69E-06	+	0.237133	0.2443	+	C	T	0.201304889	0.0430058	+	C	T	0.1706	0.051
rs74655860	LC_IGP84	IgG1 FBn/Bn total	14	65831141	1	4.68E-08	1.61E-06	-	-0.238671	-0.247	+	A	G	0.201406515	0.043479	+	A	G	0.1704	0.0515
rs74655860	LC_IGP12	IgG1_G1	14	65831141	1	4.19E-08	1.56E-06	+	0.236862	0.2455	+	A	G	0.201325666	0.0429969	+	A	G	0.1704	0.0511
rs12881911	LC_IGP78	IgG1 BG0n/G0n	14	65831221	1	2.04E-12	8.93E-11	-	-0.245529	-0.2487	+	C	T	0.580877014	0.034643	+	C	T	0.6055	0.0384
rs12881911	LC_IGP79	IgG1 BG1n/G1n	14	65831221	1	5.46E-12	1.69E-13	-	-0.241307	-0.2828	+	C	T	0.580877014	0.0347444	+	C	T	0.6055	0.0384
rs12881911	LC_IGP77	IgG1 Bn	14	65831221	1	6.40E-12	1.06E-12	-	-0.240574	-0.2744	+	C	T	0.580877014	0.0347547	+	C	T	0.6055	0.0385
rs12881911	LC_IGP86	IgG1 Bn/Fn total	14	65831221	1	9.52E-13	4.28E-13	-	-0.24972	-0.2793	+	C	T	0.580877014	0.0347069	+	C	T	0.6055	0.0385
rs12881911	LC_IGP84	IgG1 FBn/Bn total	14	65831221	1	2.58E-13	4.32E-19	+	0.256355	0.3461	+	C	T	0.580877014	0.0347496	+	C	T	0.6055	0.0388
rs12881911	LC_IGP62	IgG1 FG0n total/G0n	14	65831221	1	3.67E-15	3.25E-13	+	0.273781	0.2808	+	C	T	0.580877014	0.0344685	+	C	T	0.6055	0.0386
rs12881911	LC_IGP63	IgG1 FG1n total/G1n	14	65831221	1	4.95E-16	1.27E-16	+	0.283039	0.3191	+	C	T	0.580877014	0.0345279	+	C	T	0.6055	0.0386
rs12881911	LC_IGP64	IgG1 FG2n total/G2n	14	65831221	1	7.91E-13	6.88E-18	+	0.250721	0.3304	+	C	T	0.580877014	0.0347197	+	C	T	0.6055	0.0384
rs12881911	LC_IGP68	IgG1 FG2n/G2n	14	65831221	1	1.08E-09	8.67E-10	+	0.213809	0.2346	+	C	T	0.580877014	0.034861	+	C	T	0.6055	0.0382
rs12881911	LC_IGP61	IgG1 Fn total	14	65831221	1	2.04E-15	5.38E-16	+	0.276906	0.3126	+	C	T	0.580877014	0.0345335	+	C	T	0.6055	0.0386
rs12881911	LC_IGP81	IgG1 Fn/Bn	14	65831221	1	2.68E-12	1.25E-11	+	0.244692	0.26	+	C	T	0.580877014	0.0347172	+	C	T	0.6055	0.0384
rs12881911	LC_IGP21	IgG1 Fucosylation	14	65831221	1	1.41E-13	4.21E-15	+	0.257668	0.3027	+	C	T	0.580169852	0.0345433	+	C	T	0.6055	0.0386
rs12881911	LC_IGP28	IgG1 G1S1/(G1+G1S1)	14	65831221	1	4.57E-10	5.84E-11	+	0.216504	0.2425	+	C	T	0.580144037	0.034522	+	C	T	0.6055	0.037
rs12881911	LC_IGP11	IgG1_G0	14	65831221	1	3.33E-15	1.29E-14	-	-0.272462	-0.2956	+	C	T	0.580351558	0.0342527	+	C	T	0.6055	0.0384
rs12881911	LC_IGP_R1	IgG1_G0F/IgG1_G0	14	65831221	1	5.28E-14	1.91E-12	+	0.260347	0.2706	+	C	T	0.580351558	0.0342944	+	C	T	0.6055	0.0384
rs12881911	LC_IGP_R4	IgG1_G0FN/IgG1_G0N	14	65831221	1	6.80E-13	3.04E-15	+	0.250096	0.3052	+	C	T	0.580548377	0.0345351	+	C	T	0.6055	0.0387
rs12881911	LC_IGP52	IgG1_G0n	14	65831221	1	1.27E-15	9.80E-15	-	-0.278067	-0.2976	+	C	T	0.580877014	0.0344214	+	C	T	0.6055	0.0384
rs12881911	LC_IGP14	IgG1_G0N	14	65831221	1	3.35E-14	8.36E-12	-	-0.263741	-0.2615	+	C	T	0.580548377	0.034464	+	C	T	0.6055	0.0383
rs12881911	LC_IGP55	IgG1_G0Nn	14	65831221	1	4.30E-14	7.47E-12	-	-0.263589	-0.2624	+	C	T	0.580877014	0.0345918	+	C	T	0.6055	0.0383
rs12881911	LC_IGP12	IgG1_G1	14	65831221	1	1.79E-14	6.47E-16	-	-0.2657	-0.3112	+	C	T	0.580351558	0.0343482	+	C	T	0.6055	0.0385
rs12881911	LC_IGP_R2	IgG1_G1F/IgG1_G1	14	65831221	1	2.68E-15	1.90E-15	+	0.273984	0.3059	+	C	T	0.580351558	0.0343229	+	C	T	0.6055	0.0385
rs12881911	LC_IGP_R5	IgG1_G1FN/IgG1_G1N	14	65831221	1	1.46E-13	1.20E-20	+	0.258046	0.3594	+	C	T	0.581202645	0.0346166	+	C	T	0.6055	0.0386
rs12881911	LC_IGP53	IgG1_G1n	14	65831221	1	2.13E-14	1.16E-15	-	-0.266743	-0.3083	+	C	T	0.580877014	0.0345802	+	C	T	0.6055	0.0385
rs12881911	LC_IGP15	IgG1_G1N	14	65831221	1	3.47E-11	2.02E-12	-	-0.231571	-0.2699	+	C	T	0.581202645	0.0347131	+	C	T	0.6055	0.0384
rs12881911	LC_IGP56	IgG1_G1Nn	14	65831221	1	4.84E-11	4.19E-12	-	-0.230396	-0.2656	+	C	T	0.580877014	0.0347968	+	C	T	0.6055	0.0383
rs12881911	LC_IGP_R32	IgG1_G1S1/IgG1_G1	14	65831221	1	5.71E-10	8.14E-11	+	0.215334	0.2409	+	C	T	0.580144037	0.0345318	+	C	T	0.6055	0.0371
rs12881911	LC_IGP13	IgG1_G2	14	65831221	1	4.51E-08	2.32E-13	-	-0.190652	-0.2779	+	C	T	0.580351558	0.0346914	+	C	T	0.6055	0.0379
rs12881911	LC_IGP_R3	IgG1_G2F/IgG1_G2	14	65831221	1	1.14E-13	1.38E-17	+	0.257789	0.3267	+	C	T	0.580095049	0.0344283	+	C	T	0.6055	0.0383
rs12881911	LC_IGP_R8	IgG1_G2FS1/IgG1_G2S1	14	65831221	1	4.17E-09	4.97E-15	+	0.204622	0.2987	+	C	T	0.579740684	0.0346269	+	C	T	0.6055	0.0382
rs12881911	LC_IGP_R26	IgG1_G2N/IgG1_G1N	14	65831221	1	7.73E-09	3.30E-10	+	0.201435	0.2353	+	C	T	0.581194753	0.0347006	+	C	T	0.6055	0.0374
rs12881911	LC_IGP138	IgG2_G0n	14	65831221	1	4.58E-08	5.20E-16	-	-0.190652	-0.3105	+	C	T	0.580071121	0.0347069	+	C	T	0.6054	0.0383

rs80001898	LC_IGP11	IgG1_G0	14	65832786	1	3.20E-08	3.13E-09	+	0.368535	0.4356	+	T	C	0.076216415	0.0663214	+	T	C	0.0749	0.0735
rs80001898	LC_IGP52	IgG1_G0n	14	65832786	1	1.76E-08	1.83E-09	+	0.379187	0.4429	+	T	C	0.076373154	0.0669635	+	T	C	0.0749	0.0737
rs10148907	LC_IGP78	IgG1 BG0n/G0n	14	65833372	1	8.00E-12	1.49E-09	+	0.26309	0.267	+	G	C	0.28345632	0.038187	+	G	C	0.244	0.0442
rs10148907	LC_IGP79	IgG1 BG1n/G1n	14	65833372	1	3.77E-11	5.79E-11	+	0.255116	0.2894	+	G	C	0.28345632	0.0383118	+	G	C	0.244	0.0442
rs10148907	LC_IGP31	IgG1 BGS1/(BG0+BG+BGS1)	14	65833372	1	2.11E-08	3.96E-10	-	-0.215082	-0.2737	+	G	C	0.282729738	0.0381933	+	G	C	0.244	0.0438
rs10148907	LC_IGP77	IgG1 Bn	14	65833372	1	1.32E-11	1.70E-10	+	0.260998	0.2834	+	G	C	0.28345632	0.0382951	+	G	C	0.244	0.0444
rs10148907	LC_IGP86	IgG1 Bn/Fn total	14	65833372	1	1.78E-12	8.65E-11	+	0.271767	0.288	+	G	C	0.28345632	0.0382401	+	G	C	0.244	0.0444
rs10148907	LC_IGP84	IgG1 FBn/Bn total	14	65833372	1	7.26E-14	4.06E-15	-	-0.288722	-0.3502	+	G	C	0.28345632	0.0382431	+	G	C	0.244	0.0446
rs10148907	LC_IGP62	IgG1 FG0n total/G0n	14	65833372	1	3.98E-17	8.79E-12	-	-0.322099	-0.3026	+	G	C	0.28345632	0.0378582	+	G	C	0.244	0.0443
rs10148907	LC_IGP63	IgG1 FG1n total/G1n	14	65833372	1	1.29E-14	2.08E-13	-	-0.296425	-0.3258	+	G	C	0.28345632	0.0381043	+	G	C	0.244	0.0444
rs10148907	LC_IGP64	IgG1 FG2n total/G2n	14	65833372	1	2.40E-13	6.35E-15	-	-0.282262	-0.3444	+	G	C	0.28345632	0.038212	+	G	C	0.244	0.0442
rs10148907	LC_IGP68	IgG1 FG2n/G2n	14	65833372	1	6.09E-10	9.13E-09	-	-0.238968	-0.2533	+	G	C	0.28345632	0.0383819	+	G	C	0.244	0.0441
rs10148907	LC_IGP61	IgG1 Fn total	14	65833372	1	1.06E-15	2.62E-13	-	-0.308012	-0.3247	+	G	C	0.28345632	0.0380194	+	G	C	0.244	0.0444
rs10148907	LC_IGP81	IgG1 Fn/Bn	14	65833372	1	8.09E-12	4.66E-10	-	-0.263548	-0.2754	+	G	C	0.28345632	0.0382626	+	G	C	0.244	0.0442
rs10148907	LC_IGP21	IgG1 Fucosylation	14	65833372	1	5.88E-14	3.68E-13	-	-0.28793	-0.3228	+	G	C	0.283599568	0.0379971	+	G	C	0.244	0.0444
rs10148907	LC_IGP28	IgG1 G1S1/(G1+G1S1)	14	65833372	1	2.08E-09	1.44E-11	-	-0.229035	-0.2879	+	G	C	0.283485321	0.0380131	+	G	C	0.244	0.0426
rs10148907	LC_IGP27	IgG1 GS1/(G0+G+GS1)	14	65833372	1	1.34E-08	2.32E-08	-	-0.217562	-0.242	+	G	C	0.283485321	0.0381014	+	G	C	0.244	0.0433
rs10148907	LC_IGP11	IgG1_G0	14	65833372	1	2.81E-19	4.17E-13	+	0.340593	0.32	+	G	C	0.283444411	0.0374723	+	G	C	0.244	0.0441
rs10148907	LC_IGP_R1	IgG1_G0F/IgG1_G0	14	65833372	1	3.09E-17	3.35E-11	-	-0.320682	-0.2932	+	G	C	0.283444411	0.037562	+	G	C	0.244	0.0442
rs10148907	LC_IGP_R4	IgG1_G0FN/IgG1_G0N	14	65833372	1	4.77E-13	3.14E-13	-	-0.276899	-0.3243	+	G	C	0.283526332	0.0379772	+	G	C	0.244	0.0445
rs10148907	LC_IGP52	IgG1_G0n	14	65833372	1	3.23E-19	4.17E-13	+	0.342299	0.3206	+	G	C	0.28345632	0.0377194	+	G	C	0.244	0.0442
rs10148907	LC_IGP14	IgG1_G0N	14	65833372	1	5.91E-14	2.43E-10	+	0.287307	0.2791	+	G	C	0.283526332	0.03792	+	G	C	0.244	0.0441
rs10148907	LC_IGP55	IgG1_G0Nn	14	65833372	1	1.02E-13	2.70E-10	+	0.286034	0.2787	+	G	C	0.28345632	0.0381188	+	G	C	0.244	0.0441
rs10148907	LC_IGP12	IgG1_G1	14	65833372	1	3.34E-15	2.68E-13	+	0.300311	0.3237	+	G	C	0.283749847	0.0377556	+	G	C	0.244	0.0443
rs10148907	LC_IGP_R2	IgG1_G1F/IgG1_G1	14	65833372	1	5.56E-15	9.77E-13	-	-0.298041	-0.3159	+	G	C	0.283749847	0.0377827	+	G	C	0.244	0.0443
rs10148907	LC_IGP_R5	IgG1_G1FN/IgG1_G1N	14	65833372	1	7.10E-13	3.72E-16	-	-0.275812	-0.3617	+	G	C	0.282901907	0.0381171	+	G	C	0.244	0.0444
rs10148907	LC_IGP53	IgG1_G1n	14	65833372	1	1.02E-13	5.85E-13	+	0.28606	0.3189	+	G	C	0.28345632	0.0381225	+	G	C	0.244	0.0443
rs10148907	LC_IGP15	IgG1_G1N	14	65833372	1	2.31E-10	1.43E-10	+	0.243887	0.2834	+	G	C	0.282901907	0.0382305	+	G	C	0.244	0.0442
rs10148907	LC_IGP_SC15	IgG1_G1N(20)/IgG2_G1N(20)	14	65833372	1	9.89E-09	7.96E-07	+	0.21983	0.2088	+	G	C	0.282942426	0.0381472	+	G	C	0.244	0.0423
rs10148907	LC_IGP56	IgG1_G1Nn	14	65833372	1	1.65E-10	3.64E-10	+	0.246696	0.2766	+	G	C	0.28345632	0.0383532	+	G	C	0.244	0.0441
rs10148907	LC_IGP_R32	IgG1_G1S1/IgG1_G1	14	65833372	1	2.70E-09	2.29E-11	-	-0.22747	-0.2853	+	G	C	0.283485321	0.0380247	+	G	C	0.244	0.0427
rs10148907	LC_IGP_R3	IgG1_G2F/IgG1_G2	14	65833372	1	5.46E-14	5.15E-15	-	-0.287174	-0.3448	+	G	C	0.283312042	0.0378504	+	G	C	0.244	0.0441
rs10148907	LC_IGP_R8	IgG1_G2FS1/IgG1_G2S1	14	65833372	1	1.67E-09	8.03E-14	-	-0.230896	-0.3281	+	G	C	0.283444411	0.0380908	+	G	C	0.244	0.0439
rs10148907	LC_IGP_R26	IgG1_G2N/IgG1_G1N	14	65833372	1	3.06E-08	2.34E-10	-	-0.212738	-0.2731	+	G	C	0.283023765	0.0382284	+	G	C	0.244	0.0431
rs10148907	LC_IGP97	IgG2_G0	14	65833372	1	6.33E-10	6.39E-09	+	0.236119	0.2561	+	G	C	0.283312042	0.0379661	+	G	C	0.244	0.0441
rs10148907	LC_IGP_R41	IgG2_G0F/IgG2_G0	14	65833372	1	4.68E-08	6.59E-09	-	-0.208857	-0.2541	+	G	C	0.283312042	0.0380507	+	G	C	0.244	0.0438
rs10148907	LC_IGP138	IgG2_G0n	14	65833372	1	4.50E-10	6.80E-09	+	0.238887	0.2559	+	G	C	0.283250308	0.0380746	+	G	C	0.2441	0.0442
rs10138052	LC_IGP78	IgG1 BG0n/G0n	14	65833440	1	8.01E-12	1.49E-09	+	0.263081	0.267	+	C	T	0.283457249	0.0381868	+	C	T	0.244	0.0442
rs10138052	LC_IGP79	IgG1 BG1n/G1n	14	65833440	1	3.78E-11	5.80E-11	+	0.255107	0.2894	+	C	T	0.283457249	0.0383116	+	C	T	0.244	0.0442
rs10138052	LC_IGP31	IgG1 BGS1/(BG0+BG+BGS1)	14	65833440	1	2.11E-08	3.96E-10	-	-0.215076	-0.2737	+	C	T	0.282730673	0.038193	+	C	T	0.244	0.0438
rs10138052	LC_IGP77	IgG1 Bn	14	65833440	1	1.33E-11	1.71E-10	+	0.26099	0.2834	+	C	T	0.283457249	0.0382949	+	C	T	0.244	0.0444
rs10138052	LC_IGP86	IgG1 Bn/Fn total	14	65833440	1	1.78E-12	8.67E-11	+	0.271758	0.288	+	C	T	0.283457249	0.0382398	+	C	T	0.244	0.0444
rs10138052	LC_IGP84	IgG1 FBn/Bn total	14	65833440	1	7.27E-14	4.07E-15	-	-0.288713	-0.3502	+	C	T	0.283457249	0.0382429	+	C	T	0.244	0.0446
rs10138052	LC_IGP62	IgG1 FG0n total/G0n	14	65833440	1	3.99E-17	8.82E-12	-	-0.322088	-0.3026	+	C	T	0.283457249	0.037858	+	C	T	0.244	0.0443
rs10138052	LC_IGP63	IgG1 FG1n total/G1n	14	65833440	1	1.30E-14	2.09E-13	-	-0.296414	-0.3258	+	C	T	0.283457249	0.0381041	+	C	T	0.244	0.0444
rs10138052	LC_IGP64	IgG1 FG2n total/G2n	14	65833440	1	2.40E-13	6.36E-15	-	-0.282256	-0.3443	+	C	T	0.283457249	0.0382117	+	C	T	0.244	0.0442
rs10138052	LC_IGP68	IgG1 FG2n/G2n	14	65833440	1	6.09E-10	9.15E-09	-	-0.238963	-0.2533	+	C	T	0.283457249	0.0383817	+	C	T	0.244	0.0441
rs10138052	LC_IGP61	IgG1 Fn total	14	65833440	1	1.06E-15	2.62E-13	-	-0.308002	-0.3246	+	C	T	0.283457249	0.0380192	+	C	T	0.244	0.0444
rs10138052	LC_IGP81	IgG1 Fn/Bn	14	65833440	1	8.10E-12	4.68E-10	-	-0.263539	-0.2754	+	C	T	0.283457249	0.0382624	+	C	T	0.244	0.0442
rs10138052	LC_IGP21	IgG1 Fucosylation	14	65833440	1	5.90E-14	3.69E-13	-	-0.287918	-0.3228	+	C	T	0.283600493	0.0379969	+	C	T	0.244	0.0444
rs10138052	LC_IGP28	IgG1 G1S1/(G1+G1S1)	14	65833440	1	2.09E-09	1.44E-11	-	-0.229025	-0.288	+	C	T	0.283486239	0.0380129	+	C	T	0.244	0.0426
rs10138052	LC_IGP27	IgG1 GS1/(G0+G+GS1)	14	65833440	1	1.34E-08	2.32E-08	-	-0.217554	-0.242	+	C	T	0.283486239	0.0381012	+	C	T	0.244	0.0433
rs10138052	LC_IGP11	IgG1_G0	14	65833440	1	2.81E-19	4.19E-13	+	0.340581	0.3199	+	C	T	0.283445327	0.0374721	+	C	T	0.244	0.0441
rs10138052	LC_IGP_R1	IgG1_G0F/IgG1_G0	14	65833440	1	3.09E-17	3.36E-11	-	-0.320671	-0.2931	+	C	T	0.283445327	0.0375619	+	C	T	0.244	0.0442
rs10138052	LC_IGP_R4	IgG1_G0FN/IgG1_G0N	14	65833440	1	4.78E-13	3.15E-13	-	-0.27689	-0.3243	+	C	T	0.28352725	0.037977	+	C	T	0.244	0.0445
rs10138052	LC_IGP52	IgG1_G0n	14	65833440	1	3.23E-19	4.18E-13	+	0.342286	0.3206	+	C	T	0.283457249	0.0377192	+	C	T	0.244	0.0442
rs10138052	LC_IGP14	IgG1_G0N	14	65833440	1	5.92E-14	2.44E-10	+	0.287296	0.2791	+	C	T	0.28352725	0.0379198	+	C	T	0.244	0.0441
rs10138052	LC_IGP55	IgG1_G0Nn	14	65833440	1	1.02E-13	2.71E-10	+	0.286024	0.2787	+	C	T	0.283457249	0.0381186	+	C	T	0.244	0.0441
rs10138052	LC_IGP12	IgG1_G1	14	65833440	1	3.35E-15	2.69E-13	+	0.300301	0.3237	+	C	T	0.283750764	0.0377555	+	C	T	0.244	0.0443
rs10138052	LC_IGP_R2	IgG1_G1F/IgG1_G1	14	65833440	1	5.57E-15	9.79E-13	-	-0.29803	-0.3159	+	C	T	0.283750764	0.0377825	+	C	T	0.244	0.0443
rs10138052	LC_IGP_R5	IgG1_G1FN/IgG1_G1N	14	65833440	1	7.11E-13	3.73E-16	-	-0.275802	-0.3617	+	C	T	0.282902829	0.0381169	+	C	T	0.244	0.0444
rs10138052	LC_IGP53	IgG1_G1n	14	65833440	1	1.02E-13	5.86E-13	+	0.28605	0.3188	+	C	T	0.283457249	0.0381223	+	C	T	0.244	0.0443
rs10138052	LC_IGP15	IgG1_G1N	14	65833440	1	2.32E-10	1.43E-10	+	0.243879	0.2833	+	C	T	0.282902829	0.0382302	+	C	T	0.244	0.0442
rs10138052	LC_IGP_SC15	IgG1_G1N(20)/IgG2_G1N(20)	14	65833440	1	9.90E-09	7.97E-07	+	0.219823	0.2088	+	C	T	0.28294335	0.038147	+	C	T	0.244	0.0423
rs10138052	LC_IGP56	IgG1_G1Nn	14	65833440	1	1.65E-10	3.65E-10	+	0.246688	0.2766	+	C	T	0.283457249	0.038353	+	C	T	0.244	0.0441
rs10138052	LC_IGP_R32	IgG1_G1S1/IgG1_G1	14	65833440	1	2.70E-09	2.29E-11	-	-0.227461	-0.2853	+	C	T	0.283486239	0.0380245	+	C	T	0.244	0.0427
rs10138052	LC_IGP_R3	IgG1_G2F/IgG1_G2	14	65833440	1	5.47E-14	5.16E-15	-	-0.287167	-0.3448	+	C	T	0.283312958	0.0378502	+	C	T	0.244	0.0441
rs10138052	LC_IGP_R8	IgG1_G2FS1/IgG1_G2S1	14	65833440	1	1.67E-09	8.04E-14	-	-0.230887	-0.3281	+	C	T	0.283445327	0.0380906	+	C	T	0.244	0.0439
rs10138052	LC_IGP_R26	IgG1_G2N/IgG1_G1N	14	65833440	1	3.07E-08	2.34E-10	-	-0.212728	-0.2731	+	C	T	0.283024691	0.0382282	+	C	T	0.244	0.0431
rs10138052	LC_IGP97	IgG2_G0	14	65833440	1	6.34E-10	6.39E-09	+	0.236111	0.2561	+	C	T	0.283312958	0.0379659	+	C	T	0.244	0.0441
rs10138052	LC_IGP_R41	IgG2_G0F/IgG2_G0	14	65833440	1	4.69E-08	6.61E-09	-	-0.208849	-0.2541	+	C	T	0.283312958	0.0380504	+	C	T	0.244	0.0438
rs10138052	LC_IGP138	IgG2_G0n	14	65833440	1	4.50E-10	6.81E-09	+	0.238879	0.2558	+	C	T	0.283251232	0.0380743	+	C	T	0.2441	0.0442
rs66492349	LC_IGP78	IgG1 BG0n/G0n	14	65839489	1	8.76E-12	1.89E-09	+	0.262767	0.2658	+	C	A	0.28367658	0.0382138	+	C	A	0.246	0.0442
rs66492349	LC_IGP79	IgG1 BG1n/G1n	14	65839489	1	4.27E-11	7.59E-11	+	0.254574	0.2882	+	C	A	0.28367658	0.0383395	+	C	A	0.246	0.0443
rs66492349	LC_IGP31	IgG1 BGS1/(BG0+BG+BGS1)	14	65839489	1	1.74E-08	4.74E-10	-	-0.216468	-0.2728	+	C	A	0.282951372	0.0382137	+	C	A	0.246	0.0438
rs66492349	LC_IGP77	IgG1 Bn	14	65839489	1	1.50E-11	2.18E-10	+	0.260494	0.2823	+	C	A	0.28367658	0.0383228	+	C	A	0.246	0.0445
rs66492349	LC_IGP86	IgG1 Bn/Fn total	14	65839489	1	2.03E-12	1.11E-10	+	0.271226	0.2869	+	C	A	0.28367658	0.038268	+	C	A	0.246	0.0445
rs66492349	LC_IGP84	IgG1 FBn/Bn total	14	65839489	1	9.17E-14	6.33E-15	-	-0.287746	-0.3488	+	C	A	0.28367658	0.0382733	+	C	A	0.246	0.0447
rs66492349	LC_IGP62	IgG1 FG0n total/G0n	14	65839489	1	5.49E-17	1.12E-11	-	-0.320888	-0.3017	+	C	A	0.28367658	0.0378901	+	C	A	0.246	0.0444
rs66492349	LC_IGP63	IgG1 FG1n total/G1n	14	65839489	1	1.83E-14	3.14E-13	-	-0.294926	-0.324	+	C	A	0.28367658	0.0381371	+	C	A	0.246	0.0445
rs66492349	LC_IGP64	IgG1 FG2n total/G2n	14	65839489	1	4.05E-13	1.18E-14	-	-0.279777	-0.3415	+	C	A	0.28367658	0.0382489	+	C	A	0.246	0.0442
rs66492349	LC_IGP68	IgG1 FG2n/G2n	14	65839489	1	8.28E-10	1.35E-08	-	-0.237263	-0.2507	+	C	A	0.28367658	0.0384139	+	C	A	0.246	0.0441
rs66492349	LC_IGP61	IgG1 Fn total	14	65839489	1	1.61E-15	3.85E-13	-	-0.30627	-0.323	+	C	A	0.28367658	0.0380538	+	C	A	0.246	0.0445
rs66492349	LC_IGP81	IgG1 Fn/Bn	14	65839489	1	9.08E-12	5.85E-10	-	-0.263088	-0.2743	+	C	A	0.28367658	0.0382901	+	C	A	0.246	0.0443
rs66492349	LC_IGP21	IgG1 Fucosylation	14	65839489	1	1.22E-13	6.00E-13	-	-0.284851	-0.3205	+	C	A	0.283550555	0.0380879	+	C	A	0.246	0.0445
rs66492349	LC_IGP28	IgG1 G1S1/(G1+G1S1)	14	65839489	1	4.23E-09	2.11E-11	-	-0.225073	-0.2856	+	C	A	0.283432722	0.0381028	+	C	A	0.246	0.0426
rs66492349	LC_IGP27	IgG1 GS1/(G0+G+GS1)	14	65839489	1	1.36E-08	2.67E-08	-	-0.217896	-0.241	+	C	A	0.283432722	0.0381755	+	C	A	0.246	0.0433
rs66492349	LC_IGP11	IgG1_G0	14	65839489	1	4.04E-19	4.97E-13	+	0.339766	0.3194	+	C	A	0.283391875	0.0375531	+	C	A	0.246	0.0442
rs66492349	LC_IGP_R1	IgG1_G0F/IgG1_G0	14	65839489	1	5.16E-17	4.19E-11	-	-0.319065	-0.2923	+	C	A	0.283391875	0.0376464	+	C	A	0.246	0.0443
rs66492349	LC_IGP_R4	IgG1_G0FN/IgG1_G0N	14	65839489	1	9.29E-13	4.54E-13	-	-0.273999	-0.3228	+	C	A	0.283473668	0.0380664	+	C	A	0.246	0.0446
rs66492349	LC_IGP52	IgG1_G0n	14	65839489	1	4.55E-19	5.06E-13	+	0.341107	0.32	+	C	A	0.28367658	0.0377518	+	C	A	0.246	0.0443
rs66492349	LC_IGP14	IgG1_G0N	14	65839489	1	1.07E-13	2.97E-10	+	0.284924	0.2781	+	C	A	0.283473668	0.0380073	+	C	A	0.246	0.0442
rs66492349	LC_IGP55	IgG1_G0Nn	14	65839489	1	1.05E-13	3.30E-10	+	0.286057	0.2777	+	C	A	0.28367658	0.0381443	+	C	A	0.246	0.0442
rs66492349	LC_IGP12	IgG1_G1	14	65839489	1	8.16E-15	4.05E-13	+	0.296675	0.322	+	C	A	0.283697312	0.0378491	+	C	A	0.246	0.0444
rs66492349	LC_IGP_R2	IgG1_G1F/IgG1_G1	14	65839489	1	1.26E-14	1.42E-12	-	-0.294713	-0.3142	+	C	A	0.283697312	0.0378746	+	C	A	0.246	0.0444
rs66492349	LC_IGP_R5	IgG1_G1FN/IgG1_G1N	14	65839489	1	8.83E-13	6.29E-16	-	-0.274861	-0.3599	+	C	A	0.283120849	0.0381466	+	C	A	0.246	0.0445
rs66492349	LC_IGP53	IgG1_G1n	14	65839489	1	1.58E-13	8.80E-13	+	0.284039	0.3171	+	C	A	0.28367658	0.0381574	+	C	A	0.246	0.0444
rs66492349	LC_IGP15	IgG1_G1N	14	65839489	1	2.66E-10	1.83E-10	+	0.243231	0.2822	+	C	A	0.283120849	0.0382582	+	C	A	0.246	0.0443
rs66492349	LC_IGP_SC15	IgG1_G1N(20)/IgG2_G1N(20)	14	65839489	1	1.04E-08	1.14E-06	+	0.219657	0.2061	+	C	A	0.283161638	0.0381728	+	C	A	0.246	0.0424
rs66492349	LC_IGP56	IgG1_G1Nn	14	65839489	1	1.91E-10	4.65E-10	+	0.246011	0.2754	+	C	A	0.28367658	0.0383814	+	C	A	0.246	0.0442
rs66492349	LC_IGP_R32	IgG1_G1S1/IgG1_G1	14	65839489	1	5.30E-09	3.42E-11	-	-0.223689	-0.2828	+	C	A	0.283432722	0.0381137	+	C	A	0.246	0.0427
rs66492349	LC_IGP_R3	IgG1_G2F/IgG1_G2	14	65839489	1	1.05E-13	9.47E-15	-	-0.284503	-0.3419	+	C	A	0.283259474	0.0379386	+	C	A	0.246	0.0441
rs66492349	LC_IGP_R8	IgG1_G2FS1/IgG1_G2S1	14	65839489	1	3.27E-09	1.06E-13	-	-0.227181	-0.327	+	C	A	0.283391875	0.0381799	+	C	A	0.246	0.044
rs66492349	LC_IGP_R26	IgG1_G2N/IgG1_G1N	14	65839489	1	3.31E-08	2.14E-10	-	-0.212354	-0.2741	+	C	A	0.28324321	0.0382548	+	C	A	0.246	0.0432
rs66492349	LC_IGP97	IgG2_G0	14	65839489	1	7.24E-10	7.90E-09	+	0.235776	0.2546	+	C	A	0.283259474	0.0380426	+	C	A	0.246	0.0441
rs66492349	LC_IGP138	IgG2_G0n	14	65839489	1	5.31E-10	8.80E-09	+	0.23833	0.254	+	C	A	0.283221983	0.038147	+	C	A	0.2461	0.0442
rs4902393	LC_IGP78	IgG1 BG0n/G0n	14	65839514	1	9.12E-13	1.03E-10	-	-0.249715	-0.2474	+	C	G	0.576556382	0.0346766	+	C	G	0.6035	0.0383
rs4902393	LC_IGP79	IgG1 BG1n/G1n	14	65839514	1	2.79E-12	1.97E-13	-	-0.244943	-0.2815	+	C	G	0.576556382	0.0347809	+	C	G	0.6035	0.0383
rs4902393	LC_IGP77	IgG1 Bn	14	65839514	1	3.20E-12	1.24E-12	-	-0.244318	-0.2731	+	C	G	0.576556382	0.0347908	+	C	G	0.6035	0.0385
rs4902393	LC_IGP86	IgG1 Bn/Fn total	14	65839514	1	4.86E-13	4.85E-13	-	-0.253252	-0.2782	+	C	G	0.576556382	0.0347434	+	C	G	0.6035	0.0385
rs4902393	LC_IGP84	IgG1 FBn/Bn total	14	65839514	1	1.86E-13	5.48E-19	+	0.258242	0.3447	+	C	G	0.576556382	0.0347934	+	C	G	0.6035	0.0387
rs4902393	LC_IGP62	IgG1 FG0n total/G0n	14	65839514	1	2.10E-15	3.26E-13	+	0.276559	0.2804	+	C	G	0.576556382	0.0345071	+	C	G	0.6035	0.0385
rs4902393	LC_IGP63	IgG1 FG1n total/G1n	14	65839514	1	4.37E-16	1.40E-16	+	0.283975	0.3182	+	C	G	0.576556382	0.0345757	+	C	G	0.6035	0.0385
rs4902393	LC_IGP64	IgG1 FG2n total/G2n	14	65839514	1	9.54E-13	1.00E-17	+	0.250195	0.3282	+	C	G	0.576556382	0.0347744	+	C	G	0.6035	0.0383
rs4902393	LC_IGP68	IgG1 FG2n/G2n	14	65839514	1	7.42E-10	1.17E-09	+	0.216202	0.2321	+	C	G	0.576556382	0.034904	+	C	G	0.6035	0.0382
rs4902393	LC_IGP61	IgG1 Fn total	14	65839514	1	1.73E-15	5.86E-16	+	0.278003	0.3118	+	C	G	0.576556382	0.0345805	+	C	G	0.6035	0.0385
rs4902393	LC_IGP81	IgG1 Fn/Bn	14	65839514	1	1.27E-12	1.44E-11	+	0.248617	0.2587	+	C	G	0.576556382	0.0347522	+	C	G	0.6035	0.0383
rs4902393	LC_IGP21	IgG1 Fucosylation	14	65839514	1	1.19E-13	5.52E-15	+	0.258844	0.3009	+	C	G	0.575866523	0.0345906	+	C	G	0.6035	0.0385
rs4902393	LC_IGP28	IgG1 G1S1/(G1+G1S1)	14	65839514	1	2.55E-10	5.16E-11	+	0.219943	0.2427	+	C	G	0.575882875	0.0345607	+	C	G	0.6035	0.037

rs4902393	LC_IGP11	IgG1_G0	14	65839514	1	1.03E-15	1.14E-14	-	-0.277933	-0.2956	+	C	G	0.575790165	0.0342931 +	C	G	0.6035	0.0383
rs4902393	LC_IGP_R1	IgG1_G0F/IgG1_G0	14	65839514	1	1.34E-14	1.86E-12	+	0.266875	0.2704	+	C	G	0.575790165	0.0343308 +	C	G	0.6035	0.0384
rs4902393	LC_IGP_R4	IgG1_G0FN/IgG1_G0N	14	65839514	1	3.54E-13	3.77E-15	+	0.253608	0.3037	+	C	G	0.575975811	0.0345869 +	C	G	0.6035	0.0386
rs4902393	LC_IGP52	IgG1_G0n	14	65839514	1	6.99E-16	8.93E-15	-	-0.280988	-0.2974	+	C	G	0.576556382	0.034459 +	C	G	0.6035	0.0384
rs4902393	LC_IGP14	IgG1_G0N	14	65839514	1	6.94E-15	9.33E-12	-	-0.271128	-0.2603	+	C	G	0.575975811	0.0344964 +	C	G	0.6035	0.0382
rs4902393	LC_IGP55	IgG1_G0Nn	14	65839514	1	1.85E-14	8.49E-12	-	-0.267727	-0.2611	+	C	G	0.576556382	0.0346244 +	C	G	0.6035	0.0382
rs4902393	LC_IGP12	IgG1_G1	14	65839514	1	8.17E-15	6.64E-16	-	-0.269613	-0.3106	+	C	G	0.575790165	0.0343968 +	C	G	0.6035	0.0385
rs4902393	LC_IGP_R2	IgG1_G1F/IgG1_G1	14	65839514	1	9.83E-16	2.03E-15	+	0.278718	0.305	+	C	G	0.575790165	0.034367 +	C	G	0.6035	0.0384
rs4902393	LC_IGP_R5	IgG1_G1FN/IgG1_G1N	14	65839514	1	1.11E-13	1.57E-20	+	0.259662	0.3579	+	C	G	0.576917897	0.0346609 +	C	G	0.6035	0.0385
rs4902393	LC_IGP53	IgG1_G1n	14	65839514	1	2.18E-14	1.22E-15	-	-0.267016	-0.3076	+	C	G	0.576556382	0.0346313 +	C	G	0.6035	0.0384
rs4902393	LC_IGP15	IgG1_G1N	14	65839514	1	1.86E-11	2.24E-12	-	-0.235062	-0.2689	+	C	G	0.576917897	0.0347502 +	C	G	0.6035	0.0383
rs4902393	LC_IGP56	IgG1_G1Nn	14	65839514	1	2.51E-11	4.73E-12	-	-0.23409	-0.2645	+	C	G	0.576556382	0.0348337 +	C	G	0.6035	0.0382
rs4902393	LC_IGP_R32	IgG1_G1S1/IgG1_G1	14	65839514	1	3.15E-10	7.08E-11	+	0.218854	0.2412	+	C	G	0.575882875	0.0345702 +	C	G	0.6035	0.037
rs4902393	LC_IGP13	IgG1_G2	14	65839514	1	4.41E-08	2.77E-13	-	-0.191142	-0.2765	+	C	G	0.575790165	0.0347565 +	C	G	0.6035	0.0378
rs4902393	LC_IGP_R3	IgG1_G2F/IgG1_G2	14	65839514	1	1.53E-13	1.94E-17	+	0.256835	0.3246	+	C	G	0.575836491	0.0344851 +	C	G	0.6035	0.0382
rs4902393	LC_IGP_R8	IgG1_G2FS1/IgG1_G2S1	14	65839514	1	5.33E-09	5.39E-15	+	0.203518	0.2977	+	C	G	0.575484728	0.0346833 +	C	G	0.6035	0.0381
rs4902393	LC_IGP_R26	IgG1_G2N/IgG1_G1N	14	65839514	1	4.73E-09	2.49E-10	+	0.204566	0.2364	+	C	G	0.576890123	0.0347404 +	C	G	0.6035	0.0374
rs11621604	LC_IGP78	IgG1 BG0n/G0n	14	65840774	1	1.12E-12	1.63E-10	-	-0.249813	-0.2458	+	G	A	0.570422243	0.034828 +	G	A	0.593	0.0384
rs11621604	LC_IGP79	IgG1 BG1n/G1n	14	65840774	1	4.83E-12	3.21E-13	-	-0.243289	-0.2802	+	G	A	0.570422243	0.0349401 +	G	A	0.593	0.0385
rs11621604	LC_IGP77	IgG1 Bn	14	65840774	1	6.09E-12	2.26E-12	-	-0.242195	-0.271	+	G	A	0.570422243	0.0349521 +	G	A	0.593	0.0386
rs11621604	LC_IGP86	IgG1 Bn/Fn total	14	65840774	1	9.41E-13	1.01E-12	-	-0.251203	-0.2754	+	G	A	0.570422243	0.0349048 +	G	A	0.593	0.0386
rs11621604	LC_IGP84	IgG1 FBn/Bn total	14	65840774	1	3.45E-13	2.43E-18	+	0.25645	0.3397	+	G	A	0.570422243	0.0349542 +	G	A	0.593	0.0389
rs11621604	LC_IGP62	IgG1 FG0n total/G0n	14	65840774	1	2.81E-15	9.59E-13	+	0.276499	0.2759	+	G	A	0.570422243	0.0346596 +	G	A	0.593	0.0387
rs11621604	LC_IGP63	IgG1 FG1n total/G1n	14	65840774	1	9.11E-16	3.58E-16	+	0.282101	0.3151	+	G	A	0.570422243	0.0347379 +	G	A	0.593	0.0386
rs11621604	LC_IGP64	IgG1 FG2n total/G2n	14	65840774	1	2.77E-12	5.53E-17	+	0.246134	0.3219	+	G	A	0.570422243	0.0349446 +	G	A	0.593	0.0384
rs11621604	LC_IGP68	IgG1 FG2n/G2n	14	65840774	1	1.14E-09	2.16E-09	+	0.214738	0.2293	+	G	A	0.570422243	0.0350611 +	G	A	0.593	0.0383
rs11621604	LC_IGP61	IgG1 Fn total	14	65840774	1	3.84E-15	1.69E-15	+	0.275774	0.308	+	G	A	0.570422243	0.0347441 +	G	A	0.593	0.0387
rs11621604	LC_IGP81	IgG1 Fn/Bn	14	65840774	1	2.64E-12	2.54E-11	+	0.24615	0.2565	+	G	A	0.570422243	0.0349151 +	G	A	0.593	0.0384
rs11621604	LC_IGP21	IgG1 Fucosylation	14	65840774	1	1.83E-13	1.43E-14	+	0.257968	0.2975	+	G	A	0.569747226	0.0347486 +	G	A	0.593	0.0387
rs11621604	LC_IGP28	IgG1 G1S1/(G1+G1S1)	14	65840774	1	7.93E-10	7.57E-11	+	0.214743	0.2417	+	G	A	0.569820795	0.0347312 +	G	A	0.593	0.0371
rs11621604	LC_IGP11	IgG1_G0	14	65840774	1	1.67E-15	1.29E-13	-	-0.277067	-0.2848	+	G	A	0.569735492	0.0344491 +	G	A	0.593	0.0384
rs11621604	LC_IGP_R1	IgG1_G0F/IgG1_G0	14	65840774	1	2.01E-14	5.11E-12	+	0.266242	0.266	+	G	A	0.569735492	0.0344853 +	G	A	0.593	0.0385
rs11621604	LC_IGP_R4	IgG1_G0FN/IgG1_G0N	14	65840774	1	3.60E-13	1.33E-14	+	0.25462	0.2989	+	G	A	0.569897122	0.0347359 +	G	A	0.593	0.0388
rs11621604	LC_IGP52	IgG1_G0n	14	65840774	1	1.19E-15	9.59E-14	-	-0.279952	-0.2869	+	G	A	0.570422243	0.0346164 +	G	A	0.593	0.0385
rs11621604	LC_IGP14	IgG1_G0N	14	65840774	1	1.25E-14	4.21E-11	-	-0.269729	-0.2527	+	G	A	0.569897122	0.034657 +	G	A	0.593	0.0383
rs11621604	LC_IGP55	IgG1_G0Nn	14	65840774	1	3.17E-14	3.67E-11	-	-0.266458	-0.2537	+	G	A	0.570422243	0.0347828 +	G	A	0.593	0.0384
rs11621604	LC_IGP12	IgG1_G1	14	65840774	1	1.52E-14	1.37E-15	-	-0.268039	-0.3085	+	G	A	0.569735492	0.0345561 +	G	A	0.593	0.0386
rs11621604	LC_IGP_R2	IgG1_G1F/IgG1_G1	14	65840774	1	1.94E-15	4.87E-15	+	0.277031	0.302	+	G	A	0.569735492	0.0345274 +	G	A	0.593	0.0386
rs11621604	LC_IGP_R5	IgG1_G1FN/IgG1_G1N	14	65840774	1	1.87E-13	7.51E-20	+	0.258379	0.353	+	G	A	0.570813038	0.0348194 +	G	A	0.593	0.0387
rs11621604	LC_IGP53	IgG1_G1n	14	65840774	1	3.92E-14	2.19E-15	-	-0.265534	-0.306	+	G	A	0.570422243	0.0347907 +	G	A	0.593	0.0386
rs11621604	LC_IGP15	IgG1_G1N	14	65840774	1	3.08E-11	2.55E-12	-	-0.233503	-0.2694	+	G	A	0.570813038	0.0349087 +	G	A	0.593	0.0385
rs11621604	LC_IGP56	IgG1_G1Nn	14	65840774	1	4.01E-11	5.13E-12	-	-0.232681	-0.2651	+	G	A	0.570422243	0.0349915 +	G	A	0.593	0.0384
rs11621604	LC_IGP_R32	IgG1_G1S1/IgG1_G1	14	65840774	1	8.34E-10	1.03E-10	+	0.2145	0.2402	+	G	A	0.569820795	0.0347374 +	G	A	0.593	0.0372
rs11621604	LC_IGP_R3	IgG1_G2F/IgG1_G2	14	65840774	1	4.13E-13	8.80E-17	+	0.253345	0.319	+	G	A	0.569778117	0.0346524 +	G	A	0.593	0.0384
rs11621604	LC_IGP_R8	IgG1_G2FS1/IgG1_G2S1	14	65840774	1	2.17E-09	1.71E-14	+	0.209516	0.2931	+	G	A	0.569430055	0.0348125 +	G	A	0.593	0.0382
rs11621604	LC_IGP_R26	IgG1_G2N/IgG1_G1N	14	65840774	1	3.90E-09	7.02E-10	+	0.206541	0.2314	+	G	A	0.570769444	0.0348836 +	G	A	0.593	0.0375
rs11621604	LC_IGP97	IgG2_G0	14	65840774	1	3.45E-08	9.48E-16	-	-0.192923	-0.3085	+	G	A	0.569768949	0.0347999 +	G	A	0.593	0.0384
rs11621604	LC_IGP138	IgG2_G0n	14	65840774	1	3.25E-08	8.71E-16	-	-0.19374	-0.3091	+	G	A	0.569953202	0.0348822 +	G	A	0.5929	0.0384
rs11627402	LC_IGP78	IgG1 BG0n/G0n	14	65841322	1	6.86E-12	2.92E-10	-	-0.244014	-0.2466	+	G	T	0.548864962	0.0353025 +	G	T	0.5755	0.0391
rs11627402	LC_IGP79	IgG1 BG1n/G1n	14	65841322	1	9.11E-11	4.25E-13	-	-0.231228	-0.2829	+	G	T	0.548864962	0.0354402 +	G	T	0.5755	0.039
rs11627402	LC_IGP77	IgG1 Bn	14	65841322	1	8.15E-11	3.28E-12	-	-0.231869	-0.2734	+	G	T	0.548864962	0.0354449 +	G	T	0.5755	0.0392
rs11627402	LC_IGP86	IgG1 Bn/Fn total	14	65841322	1	1.41E-11	1.52E-12	-	-0.240948	-0.2776	+	G	T	0.548864962	0.0353995 +	G	T	0.5755	0.0392
rs11627402	LC_IGP84	IgG1 FBn/Bn total	14	65841322	1	4.17E-12	8.71E-18	+	0.247566	0.3394	+	G	T	0.548864962	0.0354451 +	G	T	0.5755	0.0395
rs11627402	LC_IGP62	IgG1 FG0n total/G0n	14	65841322	1	1.46E-14	3.00E-12	+	0.272714	0.2746	+	G	T	0.548864962	0.0351284 +	G	T	0.5755	0.0394
rs11627402	LC_IGP63	IgG1 FG1n total/G1n	14	65841322	1	3.20E-14	1.04E-15	+	0.269982	0.3149	+	G	T	0.548864962	0.0352489 +	G	T	0.5755	0.0393
rs11627402	LC_IGP64	IgG1 FG2n total/G2n	14	65841322	1	4.49E-11	1.08E-16	+	0.235065	0.3233	+	G	T	0.548864962	0.0354416 +	G	T	0.5755	0.039
rs11627402	LC_IGP68	IgG1 FG2n/G2n	14	65841322	1	1.05E-08	1.59E-09	+	0.204486	0.2348	+	G	T	0.548864962	0.0355472 +	G	T	0.5755	0.0389
rs11627402	LC_IGP61	IgG1 Fn total	14	65841322	1	6.91E-14	4.74E-15	+	0.266289	0.3081	+	G	T	0.548864962	0.0352407 +	G	T	0.5755	0.0393
rs11627402	LC_IGP81	IgG1 Fn/Bn	14	65841322	1	3.84E-11	3.37E-11	+	0.235697	0.2592	+	G	T	0.548864962	0.0354093 +	G	T	0.5755	0.0391
rs11627402	LC_IGP21	IgG1 Fucosylation	14	65841322	1	1.50E-12	2.18E-14	+	0.251242	0.3002	+	G	T	0.548237391	0.0352352 +	G	T	0.5755	0.0393
rs11627402	LC_IGP28	IgG1 G1S1/(G1+G1S1)	14	65841322	1	1.96E-09	2.97E-10	+	0.212421	0.2375	+	G	T	0.548401253	0.0351946 +	G	T	0.5755	0.0377
rs11627402	LC_IGP11	IgG1_G0	14	65841322	1	7.16E-15	4.35E-13	-	-0.274314	-0.2835	+	G	T	0.548306993	0.0349202 +	G	T	0.5755	0.0391
rs11627402	LC_IGP_R1	IgG1_G0F/IgG1_G0	14	65841322	1	6.41E-14	1.53E-11	+	0.264422	0.2647	+	G	T	0.548306993	0.0349507 +	G	T	0.5755	0.0392
rs11627402	LC_IGP_R4	IgG1_G0FN/IgG1_G0N	14	65841322	1	2.02E-12	4.93E-14	+	0.249622	0.2975	+	G	T	0.548413992	0.0352181 +	G	T	0.5755	0.0395
rs11627402	LC_IGP52	IgG1_G0n	14	65841322	1	5.63E-15	3.26E-13	-	-0.27672	-0.2856	+	G	T	0.548864962	0.0350827 +	G	T	0.5755	0.0392
rs11627402	LC_IGP14	IgG1_G0N	14	65841322	1	1.38E-13	6.87E-11	-	-0.262316	-0.2542	+	G	T	0.548413992	0.0351524 +	G	T	0.5755	0.039
rs11627402	LC_IGP55	IgG1_G0Nn	14	65841322	1	3.41E-13	5.90E-11	-	-0.258823	-0.2554	+	G	T	0.548864962	0.0352688 +	G	T	0.5755	0.039
rs11627402	LC_IGP12	IgG1_G1	14	65841322	1	2.20E-13	4.67E-15	-	-0.259324	-0.3075	+	G	T	0.548306993	0.0350543 +	G	T	0.5755	0.0392
rs11627402	LC_IGP_R2	IgG1_G1F/IgG1_G1	14	65841322	1	4.86E-14	1.35E-14	+	0.266371	0.3017	+	G	T	0.548306993	0.0350368 +	G	T	0.5755	0.0392
rs11627402	LC_IGP_R5	IgG1_G1FN/IgG1_G1N	14	65841322	1	3.52E-12	2.30E-19	+	0.247576	0.3534	+	G	T	0.549287546	0.0353246 +	G	T	0.5755	0.0393
rs11627402	LC_IGP53	IgG1_G1n	14	65841322	1	5.87E-13	7.47E-15	-	-0.256269	-0.305	+	G	T	0.548864962	0.0352839 +	G	T	0.5755	0.0392
rs11627402	LC_IGP15	IgG1_G1N	14	65841322	1	3.72E-10	3.42E-12	-	-0.223203	-0.2719	+	G	T	0.549287546	0.0354056 +	G	T	0.5755	0.0391
rs11627402	LC_IGP56	IgG1_G1Nn	14	65841322	1	5.18E-10	6.63E-12	-	-0.221842	-0.2678	+	G	T	0.548864962	0.0354841 +	G	T	0.5755	0.039
rs11627402	LC_IGP_R32	IgG1_G1S1/IgG1_G1	14	65841322	1	2.10E-09	3.88E-10	+	0.212055	0.2361	+	G	T	0.548401253	0.0352014 +	G	T	0.5755	0.0377
rs11627402	LC_IGP_R3	IgG1_G2F/IgG1_G2	14	65841322	1	6.09E-12	1.68E-16	+	0.243566	0.3206	+	G	T	0.548371668	0.0351528 +	G	T	0.5755	0.0389
rs11627402	LC_IGP_R8	IgG1_G2FS1/IgG1_G2S1	14	65841322	1	3.54E-08	2.15E-14	+	0.195627	0.2961	+	G	T	0.548036682	0.0353183 +	G	T	0.5755	0.0387
rs11627402	LC_IGP_R26	IgG1_G2N/IgG1_G1N	14	65841322	1	9.49E-09	9.17E-10	+	0.203875	0.2337	+	G	T	0.549291388	0.0353344 +	G	T	0.5755	0.0382
rs111950176	LC_IGP78	IgG1 BG0n/G0n	14	65843359	1	5.80E-09	6.43E-07	+	0.305075	0.3266	+	G	A	0.166170642	0.0521139 +	G	A	0.1443	0.0656
rs111950176	LC_IGP84	IgG1 FBn/Bn total	14	65843359	1	7.35E-09	1.95E-09	-	-0.304338	-0.4005	+	G	A	0.166170642	0.0523476 +	G	A	0.1443	0.0667
rs111950176	LC_IGP62	IgG1 FG0n total/G0n	14	65843359	1	9.50E-13	7.35E-08	-	-0.372562	-0.3537	+	G	A	0.166170642	0.0517771 +	G	A	0.1443	0.0657
rs111950176	LC_IGP63	IgG1 FG1n total/G1n	14	65843359	1	6.52E-09	1.97E-08	-	-0.3046	-0.3691	+	G	A	0.166170642	0.0522088 +	G	A	0.1443	0.0657
rs111950176	LC_IGP61	IgG1 Fn total	14	65843359	1	3.35E-10	2.60E-08	-	-0.329196	-0.3665	+	G	A	0.166170642	0.0520786 +	G	A	0.1443	0.0658
rs111950176	LC_IGP21	IgG1 Fucosylation	14	65843359	1	1.51E-08	5.94E-08	-	-0.296461	-0.3571	+	G	A	0.165994707	0.0521115 +	G	A	0.1443	0.0659
rs111950176	LC_IGP28	IgG1 G1S1/(G1+G1S1)	14	65843359	1	2.29E-08	2.46E-07	-	-0.291326	-0.3264	+	G	A	0.16553532	0.0518719 +	G	A	0.1443	0.0632
rs111950176	LC_IGP11	IgG1_G0	14	65843359	1	4.45E-13	2.73E-08	+	0.375852	0.3616	+	G	A	0.165638514	0.0514824 +	G	A	0.1443	0.0651
rs111950176	LC_IGP_R1	IgG1_G0F/IgG1_G0	14	65843359	1	1.01E-12	1.95E-07	-	-0.369937	-0.3399	+	G	A	0.165638514	0.0514848 +	G	A	0.1443	0.0653
rs111950176	LC_IGP_R4	IgG1_G0FN/IgG1_G0N	14	65843359	1	5.10E-09	5.91E-09	-	-0.305254	-0.3846	+	G	A	0.165762657	0.0519538 +	G	A	0.1443	0.0661
rs111950176	LC_IGP52	IgG1_G0n	14	65843359	1	4.00E-13	2.56E-08	+	0.378354	0.3633	+	G	A	0.166170642	0.0517124 +	G	A	0.1443	0.0652
rs111950176	LC_IGP14	IgG1_G0N	14	65843359	1	6.93E-09	4.00E-07	+	0.302535	0.3312	+	G	A	0.165762657	0.0519503 +	G	A	0.1443	0.0653
rs111950176	LC_IGP55	IgG1_G0Nn	14	65843359	1	3.07E-09	4.54E-07	+	0.310811	0.3301	+	G	A	0.166170642	0.0521392 +	G	A	0.1443	0.0654
rs111950176	LC_IGP12	IgG1_G1	14	65843359	1	6.21E-10	2.01E-08	+	0.322151	0.3683	+	G	A	0.165943951	0.0517739 +	G	A	0.1443	0.0656
rs111950176	LC_IGP_R2	IgG1_G1F/IgG1_G1	14	65843359	1	2.03E-09	7.40E-08	-	-0.312498	-0.3523	+	G	A	0.165943951	0.0518318 +	G	A	0.1443	0.0655
rs111950176	LC_IGP53	IgG1_G1n	14	65843359	1	6.34E-09	3.18E-08	+	0.304596	0.363	+	G	A	0.166170642	0.0521671 +	G	A	0.1443	0.0656
rs111950176	LC_IGP_R32	IgG1_G1S1/IgG1_G1	14	65843359	1	3.00E-08	2.57E-07	-	-0.288946	-0.3266	+	G	A	0.16553532	0.0518879 +	G	A	0.1443	0.0634
rs57507878	LC_IGP84	IgG1 FBn/Bn total	14	65843372	1	7.35E-09	6.50E-09	-	-0.304338	-0.3815	+	T	C	0.166170435	0.0523476 +	T	C	0.1497	0.0657
rs57507878	LC_IGP62	IgG1 FG0n total/G0n	14	65843372	1	9.50E-13	4.82E-07	-	-0.372562	-0.3255	+	T	C	0.166170435	0.0517771 +	T	C	0.1497	0.0647
rs57507878	LC_IGP63	IgG1 FG1n total/G1n	14	65843372	1	6.52E-09	7.29E-08	-	-0.3046	-0.3488	+	T	C	0.166170435	0.0522088 +	T	C	0.1497	0.0648
rs57507878	LC_IGP61	IgG1 Fn total	14	65843372	1	3.35E-10	1.15E-07	-	-0.329196	-0.3434	+	T	C	0.166170435	0.0520786 +	T	C	0.1497	0.0648
rs57507878	LC_IGP21	IgG1 Fucosylation	14	65843372	1	1.51E-08	2.17E-07	-	-0.296462	-0.336	+	T	C	0.165994501	0.0521115 +	T	C	0.1497	0.0648
rs57507878	LC_IGP28	IgG1 G1S1/(G1+G1S1)	14	65843372	1	2.29E-08	5.20E-07	-	-0.291326	-0.3137	+	T	C	0.165535115	0.0518719 +	T	C	0.1497	0.0625
rs57507878	LC_IGP11	IgG1_G0	14	65843372	1	4.45E-13	2.30E-07	+	0.375852	0.332	+	T	C	0.16563831	0.0514824 +	T	C	0.1497	0.0642
rs57507878	LC_IGP_R1	IgG1_G0F/IgG1_G0	14	65843372	1	1.01E-12	1.24E-06	-	-0.369937	-0.3119	+	T	C	0.16563831	0.0514848 +	T	C	0.1497	0.0643
rs57507878	LC_IGP_R4	IgG1_G0FN/IgG1_G0N	14	65843372	1	5.10E-09	3.89E-08	-	-0.305254	-0.3574	+	T	C	0.165762453	0.0519538 +	T	C	0.1497	0.065
rs57507878	LC_IGP52	IgG1_G0n	14	65843372	1	4.00E-13	2.13E-07	+	0.378354	0.3336	+	T	C	0.166170435	0.0517124 +	T	C	0.1497	0.0643
rs57507878	LC_IGP12	IgG1_G1	14	65843372	1	6.21E-10	6.77E-08	+	0.322151	0.3488	+	T	C	0.165943747	0.0517739 +	T	C	0.1497	0.0646
rs57507878	LC_IGP_R2	IgG1_G1F/IgG1_G1	14	65843372	1	2.03E-09	2.68E-07	-	-0.312498	-0.3319	+	T	C	0.165943747	0.0518318 +	T	C	0.1497	0.0645
rs57507878	LC_IGP53	IgG1_G1n	14	65843372	1	6.34E-09	1.00E-07	+	0.304596	0.3441	+	T	C	0.166170435	0.0521671 +	T	C	0.1497	0.0646
rs57507878	LC_IGP_R32	IgG1_G1S1/IgG1_G1	14	65843372	1	3.00E-08	5.55E-07	-	-0.288946	-0.3135	+	T	C	0.165535115	0.0518879 +	T	C	0.1497	0.0626
rs61161789	LC_IGP84	IgG1 FBn/Bn total	14	65843373	1	7.35E-09	3.29E-08	-	-0.304338	-0.3662	+	G	A	0.166170435	0.0523476 +	G	A	0.1502	0.0663
rs61161789	LC_IGP62	IgG1 FG0n total/G0n	14	65843373	1	9.50E-13	1.68E-06	-	-0.372562	-0.3121	+	G	A	0.166170435	0.0517771 +	G	A	0.1502	0.0652
rs61161789	LC_IGP63	IgG1 FG1n total/G1n	14	65843373	1	6.52E-09	2.67E-07	-	-0.3046	-0.3356	+	G	A	0.166170435	0.0522088 +	G	A	0.1502	0.0652
rs61161789	LC_IGP61	IgG1 Fn total	14	65843373	1	3.35E-10	4.28E-07	-	-0.329196	-0.3299	+	G	A	0.166170435	0.0520786 +	G	A	0.1502	0.0652
rs61161789	LC_IGP21	IgG1 Fucosylation	14	65843373	1	1.51E-08	8.01E-07	-	-0.296462	-0.322	+	G	A	0.165994501	0.0521115 +	G	A	0.1502	0.0652
rs61161789	LC_IGP28	IgG1 G1S1/(G1+G1S1)	14	65843373	1	2.29E-08	1.05E-06	-	-0.291326	-0.3072	+	G	A	0.165535115	0.0518719 +	G	A	0.1502	0.0629
rs61161789	LC_IGP11	IgG1_G0	14	65843373	1	4.45E-13	8.02E-07	+	0.375852	0.319	+	G	A	0.16563831	0.0514824 +	G	A	0.1502	0.0646
rs61161789	LC_IGP_R4	IgG1_G0FN/IgG1_G0N	14	65843373	1	5.10E-09	1.91E-07	-	-0.305254	-0.3419	+	G	A	0.165762453	0.0519538 +	G	A	0.1502	0.0656
rs61161789	LC_IGP52	IgG1_G0n	14	65843373	1	4.00E-13	7.42E-07	+	0.378354	0.3206	+	G	A	0.166170435	0.0517124 +	G	A	0.1502	0.0648
rs61161789	LC_IGP12	IgG1_G1	14	65843373	1	6.21E-10	2.26E-07	+	0.322151	0.337	+	G	A	0.165943747	0.0517739 +	G	A	0.1502	0.0651
rs61161789	LC_IGP_R2	IgG1_G1F/IgG1_G1	14	65843373	1	2.03E-09	9.07E-07	-	-0.312498	-0.319	+	G	A	0.165943747	0.0518318 +	G	A	0.1502	0.0649

rs61161789	LC_IGP53	IgG1_G1n	14	65843373	1	6.34E-09	3.21E-07	+	0.304596	0.3325	+	G	A	0.166170435	0.0521671	+	G	A	0.1502	0.0651
rs61161789	LC_IGP_R32	IgG1_G1S1/IgG1_G1	14	65843373	1	3.00E-08	1.05E-06	-	-0.288946	-0.3076	+	G	A	0.165535115	0.0518879	+	G	A	0.1502	0.063
rs8022094	LC_IGP78	IgG1 BG0n/G0n	14	65846841	1	1.97E-13	1.87E-10	-	-0.25921	-0.2453	+	T	G	0.567458178	0.0349623	+	T	G	0.5905	0.0385
rs8022094	LC_IGP79	IgG1 BG1n/G1n	14	65846841	1	1.60E-12	2.88E-13	-	-0.249876	-0.2808	+	T	G	0.567458178	0.0350884	+	T	G	0.5905	0.0385
rs8022094	LC_IGP77	IgG1 Bn	14	65846841	1	1.72E-12	2.24E-12	-	-0.249597	-0.2714	+	T	G	0.567458178	0.0350969	+	T	G	0.5905	0.0387
rs8022094	LC_IGP86	IgG1 Bn/Fn total	14	65846841	1	2.62E-13	1.08E-12	-	-0.258489	-0.2755	+	T	G	0.567458178	0.0350491	+	T	G	0.5905	0.0387
rs8022094	LC_IGP84	IgG1 FBn/Bn total	14	65846841	1	6.74E-14	3.05E-18	+	0.26528	0.3393	+	T	G	0.567458178	0.0350912	+	T	G	0.5905	0.039
rs8022094	LC_IGP62	IgG1 FG0n total/G0n	14	65846841	1	7.07E-16	1.18E-12	+	0.28372	0.2753	+	T	G	0.567458178	0.0348008	+	T	G	0.5905	0.0387
rs8022094	LC_IGP63	IgG1 FG1n total/G1n	14	65846841	1	4.14E-16	4.26E-16	+	0.286812	0.3145	+	T	G	0.567458178	0.0348919	+	T	G	0.5905	0.0387
rs8022094	LC_IGP64	IgG1 FG2n total/G2n	14	65846841	1	3.31E-12	7.21E-17	+	0.246466	0.3211	+	T	G	0.567458178	0.0351202	+	T	G	0.5905	0.0385
rs8022094	LC_IGP68	IgG1 FG2n/G2n	14	65846841	1	1.95E-09	1.98E-09	+	0.212748	0.2299	+	T	G	0.567458178	0.035245	+	T	G	0.5905	0.0383
rs8022094	LC_IGP61	IgG1 Fn total	14	65846841	1	1.37E-15	2.10E-15	+	0.281543	0.3074	+	T	G	0.567458178	0.034893	+	T	G	0.5905	0.0387
rs8022094	LC_IGP81	IgG1 Fn/Bn	14	65846841	1	8.12E-13	2.25E-11	+	0.253057	0.2574	+	T	G	0.567458178	0.0350616	+	T	G	0.5905	0.0385
rs8022094	LC_IGP21	IgG1 Fucosylation	14	65846841	1	1.26E-13	1.47E-14	+	0.261175	0.2977	+	T	G	0.567062885	0.034939	+	T	G	0.5905	0.0387
rs8022094	LC_IGP28	IgG1 G1S1/(G1+G1S1)	14	65846841	1	9.81E-10	6.25E-11	+	0.214618	0.2428	+	T	G	0.566937615	0.0349055	+	T	G	0.5905	0.0371
rs8022094	LC_IGP11	IgG1_G0	14	65846841	1	1.47E-15	1.51E-13	-	-0.278971	-0.2843	+	T	G	0.566855834	0.034615	+	T	G	0.5905	0.0385
rs8022094	LC_IGP_R1	IgG1_G0F/IgG1_G0	14	65846841	1	7.64E-15	5.93E-12	+	0.271774	0.2656	+	T	G	0.566855834	0.0346339	+	T	G	0.5905	0.0386
rs8022094	LC_IGP_R4	IgG1_G0FN/IgG1_G0N	14	65846841	1	8.74E-14	1.78E-14	+	0.26241	0.298	+	T	G	0.56701041	0.0348768	+	T	G	0.5905	0.0389
rs8022094	LC_IGP52	IgG1_G0n	14	65846841	1	1.04E-15	1.08E-13	-	-0.281893	-0.2867	+	T	G	0.567458178	0.0347838	+	T	G	0.5905	0.0386
rs8022094	LC_IGP14	IgG1_G0N	14	65846841	1	4.14E-15	4.42E-11	-	-0.275878	-0.2527	+	T	G	0.56701041	0.0348041	+	T	G	0.5905	0.0384
rs8022094	LC_IGP55	IgG1_G0Nn	14	65846841	1	8.91E-15	3.71E-11	-	-0.273409	-0.254	+	T	G	0.567458178	0.0349267	+	T	G	0.5905	0.0384
rs8022094	LC_IGP12	IgG1_G1	14	65846841	1	6.05E-15	2.07E-15	-	-0.273387	-0.3069	+	T	G	0.566855834	0.0347059	+	T	G	0.5905	0.0387
rs8022094	LC_IGP_R2	IgG1_G1F/IgG1_G1	14	65846841	1	1.02E-15	5.67E-15	+	0.281112	0.3015	+	T	G	0.566855834	0.0346829	+	T	G	0.5905	0.0386
rs8022094	LC_IGP_R5	IgG1_G1FN/IgG1_G1N	14	65846841	1	6.83E-14	7.21E-20	+	0.264272	0.3536	+	T	G	0.567913899	0.0349685	+	T	G	0.5905	0.0388
rs8022094	LC_IGP53	IgG1_G1n	14	65846841	1	1.57E-14	3.15E-15	-	-0.270947	-0.3047	+	T	G	0.567458178	0.0349421	+	T	G	0.5905	0.0386
rs8022094	LC_IGP15	IgG1_G1N	14	65846841	1	1.02E-11	2.65E-12	-	-0.240262	-0.2694	+	T	G	0.567913899	0.0350562	+	T	G	0.5905	0.0385
rs8022094	LC_IGP56	IgG1_G1Nn	14	65846841	1	1.14E-11	5.14E-12	-	-0.240262	-0.2654	+	T	G	0.567458178	0.0351366	+	T	G	0.5905	0.0385
rs8022094	LC_IGP_R32	IgG1_G1S1/IgG1_G1	14	65846841	1	1.08E-09	8.92E-11	+	0.214126	0.241	+	T	G	0.566937615	0.0349127	+	T	G	0.5905	0.0372
rs8022094	LC_IGP13	IgG1_G2	14	65846841	1	3.27E-08	3.70E-13	-	-0.194732	-0.2762	+	T	G	0.566855834	0.0350689	+	T	G	0.5905	0.038
rs8022094	LC_IGP_R3	IgG1_G2F/IgG1_G2	14	65846841	1	4.21E-13	1.14E-16	+	0.254502	0.3183	+	T	G	0.566896699	0.0348223	+	T	G	0.5905	0.0384
rs8022094	LC_IGP_R8	IgG1_G2FS1/IgG1_G2S1	14	65846841	1	5.89E-09	3.39E-14	+	0.20481	0.29	+	T	G	0.566550397	0.0350036	+	T	G	0.5905	0.0382
rs8022094	LC_IGP_R26	IgG1_G2N/IgG1_G1N	14	65846841	1	9.39E-10	5.54E-10	+	0.21561	0.2328	+	T	G	0.567816358	0.035024	+	T	G	0.5905	0.0375
rs8022094	LC_IGP97	IgG2_G0	14	65846841	1	3.76E-08	9.73E-16	-	-0.193326	-0.3087	+	T	G	0.566887531	0.0349719	+	T	G	0.5905	0.0384
rs8022094	LC_IGP138	IgG2_G0n	14	65846841	1	3.56E-08	8.53E-16	-	-0.194022	-0.3095	+	T	G	0.566815887	0.035035	+	T	G	0.5904	0.0385
rs12882269	LC_IGP78	IgG1 BG0n/G0n	14	65847144	1	1.06E-13	1.34E-10	-	-0.262039	-0.2472	+	G	T	0.568508984	0.0349454	+	G	T	0.5934	0.0385
rs12882269	LC_IGP79	IgG1 BG1n/G1n	14	65847144	1	1.58E-12	2.19E-13	-	-0.249936	-0.2824	+	G	T	0.568508984	0.0350843	+	G	T	0.5934	0.0385
rs12882269	LC_IGP77	IgG1 Bn	14	65847144	1	1.38E-12	1.67E-12	-	-0.250644	-0.273	+	G	T	0.568508984	0.0350884	+	G	T	0.5934	0.0387
rs12882269	LC_IGP86	IgG1 Bn/Fn total	14	65847144	1	2.07E-13	7.94E-13	-	-0.25956	-0.2771	+	G	T	0.568508984	0.0350403	+	G	T	0.5934	0.0387
rs12882269	LC_IGP84	IgG1 FBn/Bn total	14	65847144	1	4.92E-14	2.29E-18	+	0.266685	0.3408	+	G	T	0.568508984	0.0350807	+	G	T	0.5934	0.039
rs12882269	LC_IGP62	IgG1 FG0n total/G0n	14	65847144	1	4.69E-16	1.02E-12	+	0.285412	0.2761	+	G	T	0.568508984	0.0347883	+	G	T	0.5934	0.0387
rs12882269	LC_IGP63	IgG1 FG1n total/G1n	14	65847144	1	3.45E-16	3.69E-16	+	0.287537	0.3153	+	G	T	0.568508984	0.0348843	+	G	T	0.5934	0.0387
rs12882269	LC_IGP64	IgG1 FG2n total/G2n	14	65847144	1	4.68E-12	6.77E-17	+	0.244727	0.3213	+	G	T	0.568508984	0.0351239	+	G	T	0.5934	0.0385
rs12882269	LC_IGP68	IgG1 FG2n/G2n	14	65847144	1	2.33E-09	2.10E-09	+	0.211708	0.2294	+	G	T	0.568508984	0.0352451	+	G	T	0.5934	0.0383
rs12882269	LC_IGP61	IgG1 Fn total	14	65847144	1	1.02E-15	1.81E-15	+	0.282797	0.3081	+	G	T	0.568508984	0.0348829	+	G	T	0.5934	0.0387
rs12882269	LC_IGP81	IgG1 Fn/Bn	14	65847144	1	6.53E-13	1.75E-11	+	0.254067	0.2589	+	G	T	0.568508984	0.0350532	+	G	T	0.5934	0.0385
rs12882269	LC_IGP21	IgG1 Fucosylation	14	65847144	1	1.03E-13	1.52E-14	+	0.262041	0.2975	+	G	T	0.568102343	0.0349311	+	G	T	0.5934	0.0387
rs12882269	LC_IGP28	IgG1 G1S1/(G1+G1S1)	14	65847144	1	1.24E-09	8.73E-11	+	0.213313	0.241	+	G	T	0.567980734	0.0349064	+	G	T	0.5934	0.0371
rs12882269	LC_IGP11	IgG1_G0	14	65847144	1	1.20E-15	1.63E-13	-	-0.279784	-0.284	+	G	T	0.567897679	0.0346069	+	G	T	0.5934	0.0385
rs12882269	LC_IGP_R1	IgG1_G0F/IgG1_G0	14	65847144	1	5.71E-15	5.37E-12	+	0.273006	0.2661	+	G	T	0.567897679	0.0346239	+	G	T	0.5934	0.0386
rs12882269	LC_IGP_R4	IgG1_G0FN/IgG1_G0N	14	65847144	1	4.35E-14	1.32E-14	+	0.265536	0.2995	+	G	T	0.568054807	0.0348583	+	G	T	0.5934	0.0389
rs12882269	LC_IGP52	IgG1_G0n	14	65847144	1	8.69E-16	1.19E-13	-	-0.282617	-0.2862	+	G	T	0.568508984	0.0347763	+	G	T	0.5934	0.0386
rs12882269	LC_IGP14	IgG1_G0N	14	65847144	1	2.21E-15	3.55E-11	-	-0.278546	-0.2541	+	G	T	0.568054807	0.034787	+	G	T	0.5934	0.0384
rs12882269	LC_IGP55	IgG1_G0Nn	14	65847144	1	4.88E-15	3.03E-11	-	-0.276007	-0.2553	+	G	T	0.568508984	0.0349102	+	G	T	0.5934	0.0384
rs12882269	LC_IGP12	IgG1_G1	14	65847144	1	4.08E-15	1.77E-15	-	-0.275059	-0.3077	+	G	T	0.567897679	0.0346938	+	G	T	0.5934	0.0387
rs12882269	LC_IGP_R2	IgG1_G1F/IgG1_G1	14	65847144	1	8.34E-16	5.13E-15	+	0.281934	0.302	+	G	T	0.567897679	0.0346748	+	G	T	0.5934	0.0386
rs12882269	LC_IGP_R5	IgG1_G1FN/IgG1_G1N	14	65847144	1	6.63E-14	6.04E-20	+	0.264385	0.3546	+	G	T	0.568962792	0.0349643	+	G	T	0.5934	0.0388
rs12882269	LC_IGP53	IgG1_G1n	14	65847144	1	1.11E-14	2.77E-15	-	-0.272435	-0.3053	+	G	T	0.568508984	0.0349311	+	G	T	0.5934	0.0386
rs12882269	LC_IGP15	IgG1_G1N	14	65847144	1	9.11E-12	1.97E-12	-	-0.240799	-0.2711	+	G	T	0.568962792	0.0350502	+	G	T	0.5934	0.0385
rs12882269	LC_IGP56	IgG1_G1Nn	14	65847144	1	1.01E-11	3.89E-12	-	-0.240849	-0.267	+	G	T	0.568508984	0.0351303	+	G	T	0.5934	0.0385
rs12882269	LC_IGP_R32	IgG1_G1S1/IgG1_G1	14	65847144	1	1.37E-09	1.20E-10	+	0.212777	0.2394	+	G	T	0.567980734	0.0349138	+	G	T	0.5934	0.0372
rs12882269	LC_IGP13	IgG1_G2	14	65847144	1	3.17E-08	3.10E-13	-	-0.19491	-0.277	+	G	T	0.567897679	0.0350644	+	G	T	0.5934	0.038
rs12882269	LC_IGP_R3	IgG1_G2F/IgG1_G2	14	65847144	1	5.46E-13	1.10E-16	+	0.253245	0.3184	+	G	T	0.567939181	0.0348239	+	G	T	0.5934	0.0384
rs12882269	LC_IGP_R8	IgG1_G2FS1/IgG1_G2S1	14	65847144	1	4.55E-09	2.39E-14	+	0.206275	0.2919	+	G	T	0.567592242	0.0349943	+	G	T	0.5934	0.0383
rs12882269	LC_IGP_R26	IgG1_G2N/IgG1_G1N	14	65847144	1	6.14E-10	5.18E-10	+	0.217926	0.2333	+	G	T	0.568863272	0.0350114	+	G	T	0.5934	0.0376
rs12882269	LC_IGP97	IgG2_G0	14	65847144	1	3.54E-08	1.05E-15	-	-0.193677	-0.3084	+	G	T	0.567930012	0.0349668	+	G	T	0.5934	0.0384
rs12882269	LC_IGP138	IgG2_G0n	14	65847144	1	3.32E-08	9.32E-16	-	-0.194423	-0.3092	+	G	T	0.567827278	0.0350278	+	G	T	0.5933	0.0385
rs73282213	LC_IGP62	IgG1 FG0n total/G0n	14	65852139	1	4.26E-08	1.07E-07	-	-0.368253	-0.3966	+	G	C	0.076793943	0.0668779	+	G	C	0.0738	0.0746
rs73282213	LC_IGP11	IgG1_G0	14	65852139	1	3.11E-08	6.58E-08	+	0.370194	0.4012	+	G	C	0.076325854	0.0665568	+	G	C	0.0738	0.0743
rs73282213	LC_IGP52	IgG1_G0n	14	65852139	1	1.77E-08	4.07E-08	+	0.378163	0.4084	+	G	C	0.076793943	0.0667944	+	G	C	0.0738	0.0744
rs1815654	LC_IGP78	IgG1 BG0n/G0n	14	65855232	1	1.58E-13	5.07E-11	-	-0.260249	-0.2528	+	T	C	0.5765998	0.0349597	+	T	C	0.6031	0.0385
rs1815654	LC_IGP79	IgG1 BG1n/G1n	14	65855232	1	6.06E-13	9.84E-14	-	-0.254556	-0.2866	+	T	C	0.5765998	0.0350697	+	T	C	0.6031	0.0385
rs1815654	LC_IGP77	IgG1 Bn	14	65855232	1	6.75E-13	6.34E-13	-	-0.254095	-0.2782	+	T	C	0.5765998	0.0350791	+	T	C	0.6031	0.0387
rs1815654	LC_IGP86	IgG1 Bn/Fn total	14	65855232	1	1.02E-13	2.68E-13	-	-0.262871	-0.2829	+	T	C	0.5765998	0.0350311	+	T	C	0.6031	0.0387
rs1815654	LC_IGP84	IgG1 FBn/Bn total	14	65855232	1	3.61E-14	3.76E-19	+	0.268129	0.3487	+	T	C	0.5765998	0.0350799	+	T	C	0.6031	0.039
rs1815654	LC_IGP62	IgG1 FG0n total/G0n	14	65855232	1	7.21E-16	1.91E-13	+	0.28366	0.2847	+	T	C	0.5765998	0.0348033	+	T	C	0.6031	0.0387
rs1815654	LC_IGP63	IgG1 FG1n total/G1n	14	65855232	1	1.51E-16	8.13E-17	+	0.291002	0.3223	+	T	C	0.5765998	0.0348726	+	T	C	0.6031	0.0387
rs1815654	LC_IGP64	IgG1 FG2n total/G2n	14	65855232	1	1.14E-12	1.26E-17	+	0.251653	0.3289	+	T	C	0.5765998	0.0350996	+	T	C	0.6031	0.0385
rs1815654	LC_IGP68	IgG1 FG2n/G2n	14	65855232	1	8.25E-10	1.24E-09	+	0.217611	0.2326	+	T	C	0.5765998	0.0352288	+	T	C	0.6031	0.0383
rs1815654	LC_IGP61	IgG1 Fn total	14	65855232	1	5.93E-16	3.77E-16	+	0.285117	0.3154	+	T	C	0.5765998	0.0348772	+	T	C	0.6031	0.0387
rs1815654	LC_IGP81	IgG1 Fn/Bn	14	65855232	1	2.77E-13	6.50E-12	+	0.258153	0.2643	+	T	C	0.5765998	0.0350408	+	T	C	0.6031	0.0385
rs1815654	LC_IGP21	IgG1 Fucosylation	14	65855232	1	8.33E-14	4.73E-15	+	0.263047	0.3032	+	T	C	0.57615726	0.0349297	+	T	C	0.6031	0.0387
rs1815654	LC_IGP28	IgG1 G1S1/(G1+G1S1)	14	65855232	1	4.78E-10	2.89E-11	+	0.218565	0.2472	+	T	C	0.575987508	0.0348899	+	T	C	0.6031	0.0372
rs1815654	LC_IGP11	IgG1_G0	14	65855232	1	9.20E-16	2.14E-14	-	-0.280931	-0.2942	+	T	C	0.575894671	0.0346045	+	T	C	0.6031	0.0385
rs1815654	LC_IGP_R1	IgG1_G0F/IgG1_G0	14	65855232	1	6.76E-15	1.05E-12	+	0.272297	0.2748	+	T	C	0.575894671	0.0346306	+	T	C	0.6031	0.0386
rs1815654	LC_IGP_R4	IgG1_G0FN/IgG1_G0N	14	65855232	1	9.66E-14	2.81E-15	+	0.261941	0.307	+	T	C	0.576080573	0.0348776	+	T	C	0.6031	0.0389
rs1815654	LC_IGP52	IgG1_G0n	14	65855232	1	6.73E-16	1.33E-14	-	-0.283742	-0.2973	+	T	C	0.5765998	0.0347767	+	T	C	0.6031	0.0386
rs1815654	LC_IGP14	IgG1_G0N	14	65855232	1	1.49E-15	9.41E-12	-	-0.280255	-0.2617	+	T	C	0.576080573	0.0347812	+	T	C	0.6031	0.0384
rs1815654	LC_IGP55	IgG1_G0Nn	14	65855232	1	3.64E-15	7.12E-12	-	-0.277324	-0.2636	+	T	C	0.5765998	0.0349098	+	T	C	0.6031	0.0384
rs1815654	LC_IGP12	IgG1_G1	14	65855232	1	2.88E-15	5.10E-16	-	-0.276581	-0.3134	+	T	C	0.575894671	0.0346896	+	T	C	0.6031	0.0386
rs1815654	LC_IGP_R2	IgG1_G1F/IgG1_G1	14	65855232	1	3.91E-16	1.21E-15	+	0.285123	0.3089	+	T	C	0.575894671	0.0346621	+	T	C	0.6031	0.0386
rs1815654	LC_IGP_R5	IgG1_G1FN/IgG1_G1N	14	65855232	1	3.28E-14	9.48E-21	+	0.2676	0.3624	+	T	C	0.57702311	0.034954	+	T	C	0.6031	0.0388
rs1815654	LC_IGP53	IgG1_G1n	14	65855232	1	8.65E-15	7.30E-16	-	-0.273582	-0.3116	+	T	C	0.5765998	0.0349316	+	T	C	0.6031	0.0386
rs1815654	LC_IGP15	IgG1_G1N	14	65855232	1	4.54E-12	1.27E-12	-	-0.244282	-0.2734	+	T	C	0.57702311	0.0350401	+	T	C	0.6031	0.0385
rs1815654	LC_IGP56	IgG1_G1Nn	14	65855232	1	5.26E-12	2.18E-12	-	-0.244123	-0.2701	+	T	C	0.5765998	0.0351223	+	T	C	0.6031	0.0385
rs1815654	LC_IGP_R32	IgG1_G1S1/IgG1_G1	14	65855232	1	5.97E-10	4.05E-11	+	0.21738	0.2455	+	T	C	0.575987508	0.0348998	+	T	C	0.6031	0.0372
rs1815654	LC_IGP13	IgG1_G2	14	65855232	1	2.62E-08	2.27E-13	-	-0.196088	-0.2785	+	T	C	0.575894671	0.0350636	+	T	C	0.6031	0.038
rs1815654	LC_IGP_SC13	IgG1_G2(20)/IgG2_G2(20)	14	65855232	1	4.62E-08	9.10E-07	-	-0.19282	-0.1825	+	T	C	0.575375887	0.0351144	+	T	C	0.6031	0.0372
rs1815654	LC_IGP_R3	IgG1_G2F/IgG1_G2	14	65855232	1	1.60E-13	2.38E-17	+	0.258984	0.3253	+	T	C	0.575941061	0.0348014	+	T	C	0.6031	0.0384
rs1815654	LC_IGP_R8	IgG1_G2FS1/IgG1_G2S1	14	65855232	1	7.00E-09	1.18E-14	+	0.203794	0.2953	+	T	C	0.575589234	0.0350057	+	T	C	0.6031	0.0383
rs1815654	LC_IGP_R26	IgG1_G2N/IgG1_G1N	14	65855232	1	7.65E-10	1.33E-10	+	0.216753	0.2412	+	T	C	0.576933381	0.0350216	+	T	C	0.6031	0.0376
rs11158591	LC_IGP78	IgG1 BG0n/G0n	14	65855762	1	1.08E-13	1.39E-10	-	-0.262124	-0.2485	+	T	C	0.576890954	0.0349683	+	T	C	0.5986	0.0387
rs11158591	LC_IGP79	IgG1 BG1n/G1n	14	65855762	1	2.39E-13	2.06E-13	-	-0.259056	-0.2845	+	T	C	0.576890954	0.0350666	+	T	C	0.5986	0.0387
rs11158591	LC_IGP32	IgG1 BG1S1/(BG1+BG1S1)	14	65855762	1	3.89E-08	4.18E-13	+	0.193498	0.2756	+	T	C	0.577195746	0.0350401	+	T	C	0.5987	0.038
rs11158591	LC_IGP77	IgG1 Bn	14	65855762	1	3.42E-13	1.67E-12	-	-0.257433	-0.2747	+	T	C	0.576890954	0.0350813	+	T	C	0.5986	0.0389
rs11158591	LC_IGP86	IgG1 Bn/Fn total	14	65855762	1	4.93E-14	7.40E-13	-	-0.266311	-0.2792	+	T	C	0.576890954	0.0350323	+	T	C	0.5986	0.0389
rs11158591	LC_IGP84	IgG1 FBn/Bn total	14	65855762	1	1.74E-14	1.40E-18	+	0.27153	0.3453	+	T	C	0.576890954	0.0350811	+	T	C	0.5986	0.0393
rs11158591	LC_IGP62	IgG1 FG0n total/G0n	14	65855762	1	4.02E-16	7.03E-13	+	0.286236	0.2798	+	T	C	0.576890954	0.0348075	+	T	C	0.5986	0.039
rs11158591	LC_IGP63	IgG1 FG1n total/G1n	14	65855762	1	4.59E-17	2.18E-16	+	0.296021	0.3198	+	T	C	0.576890954	0.0348638	+	T	C	0.5986	0.0389
rs11158591	LC_IGP64	IgG1 FG2n total/G2n	14	65855762	1	4.34E-13	4.00E-17	+	0.256376	0.326	+	T	C	0.576890954	0.0350957	+	T	C	0.5986	0.0387
rs11158591	LC_IGP68	IgG1 FG2n/G2n	14	65855762	1	3.94E-10	1.97E-09	+	0.221785	0.2311	+	T	C	0.576890954	0.0352299	+	T	C	0.5986	0.0385
rs11158591	LC_IGP61	IgG1 Fn total	14	65855762	1	2.38E-16	1.14E-15	+	0.289069	0.3123	+	T	C	0.576890954	0.0348744	+	T	C	0.5986	0.039
rs11158591	LC_IGP81	IgG1 Fn/Bn	14	65855762	1	1.37E-13	1.84E-11	+	0.261555	0.26	+	T	C	0.576890954	0.0350424	+	T	C	0.5986	0.0387
rs11158591	LC_IGP21	IgG1 Fucosylation	14	65855762	1	3.07E-14	1.77E-14	+	0.267692	0.2988	+	T	C	0.576446054	0.0349251	+	T	C	0.5986	0.039
rs11158591	LC_IGP28	IgG1 G1S1/(G1+G1S1)	14	65855762	1	3.54E-10	7.25E-11	+	0.220291	0.2438	+	T	C	0.576276147	0.0349001	+	T	C	0.5986	0.0374
rs11158591	LC_IGP11	IgG1_G0	14	65855762	1	5.58E-16	9.34E-14	-	-0.283155	-0.2887	+	T	C	0.576182957	0.0346103	+	T	C	0.5986	0.0388
rs11158591	LC_IGP_R1	IgG1_G0F/IgG1_G0	14	65855762	1	3.81E-15	3.84E-12	+	0.274905	0.2696	+	T	C	0.576182957	0.0346349	+	T	C	0.5986	0.0388
rs11158591	LC_IGP_R4	IgG1_G0FN/IgG1_G0N	14	65855762	1	6.16E-14	9.12E-15	+	0.264112	0.3033	+	T	C	0.576369565	0.0348846	+	T	C	0.5986	0.0391
rs11158591	LC_IGP52	IgG1_G0n	14	65855762	1	3.97E-16	7.01E-14	-	-0.286091	-0.2908	+	T	C	0.576890954	0.0347821	+	T	C	0.5986	0.0388
rs11158591	LC_IGP14	IgG1_G0N	14	65855762	1	1.04E-15	3.52E-11	-	-0.281891	-0.2557	+	T	C	0.576369565	0.0347902	+	T	C	0.5986	0.0386

rs11158591	LC_IGP55	IgG1_G0Nn	14	65855762	1	2.51E-15	3.08E-11	-	-0.279065	-0.2567	+	T	C	0.576890954	0.0349185	+	T	C	0.5986	0.0386
rs11158591	LC_IGP12	IgG1_G1	14	65855762	1	1.04E-15	9.70E-16	-	-0.281052	-0.3126	+	T	C	0.576182957	0.0346846	+	T	C	0.5986	0.0389
rs11158591	LC_IGP_R2	IgG1_G1F/IgG1_G1	14	65855762	1	1.25E-16	3.19E-15	+	0.289964	0.3062	+	T	C	0.576182957	0.0346545	+	T	C	0.5986	0.0388
rs11158591	LC_IGP_R5	IgG1_G1FN/IgG1_G1N	14	65855762	1	1.24E-14	3.66E-20	+	0.272066	0.3593	+	T	C	0.577313346	0.03495	+	T	C	0.5986	0.0391
rs11158591	LC_IGP53	IgG1_G1n	14	65855762	1	3.11E-15	1.61E-15	-	-0.27817	-0.3099	+	T	C	0.576890954	0.0349265	+	T	C	0.5986	0.0389
rs11158591	LC_IGP15	IgG1_G1N	14	65855762	1	1.94E-12	1.88E-12	-	-0.248562	-0.273	+	T	C	0.577313346	0.0350388	+	T	C	0.5986	0.0388
rs11158591	LC_IGP56	IgG1_G1Nn	14	65855762	1	2.20E-12	3.80E-12	-	-0.24852	-0.2687	+	T	C	0.576890954	0.0351205	+	T	C	0.5986	0.0387
rs11158591	LC_IGP_R36	IgG1_G1NS1/IgG1_G1N	14	65855762	1	2.69E-08	4.63E-13	+	0.195804	0.2754	+	T	C	0.577195746	0.0350406	+	T	C	0.5987	0.0381
rs11158591	LC_IGP_R32	IgG1_G1S1/IgG1_G1	14	65855762	1	3.90E-10	9.48E-11	+	0.219805	0.2425	+	T	C	0.576276147	0.0349073	+	T	C	0.5986	0.0375
rs11158591	LC_IGP13	IgG1_G2	14	65855762	1	1.45E-08	2.10E-13	-	-0.199733	-0.2808	+	T	C	0.576182957	0.0350678	+	T	C	0.5986	0.0382
rs11158591	LC_IGP_SC13	IgG1_G2(20)/IgG2_G2(20)	14	65855762	1	2.31E-08	8.70E-07	-	-0.197164	-0.1839	+	T	C	0.575664526	0.0351165	+	T	C	0.5986	0.0374
rs11158591	LC_IGP_R3	IgG1_G2F/IgG1_G2	14	65855762	1	5.80E-14	6.41E-17	+	0.263726	0.323	+	T	C	0.576229523	0.0347965	+	T	C	0.5986	0.0386
rs11158591	LC_IGP_R8	IgG1_G2FS1/IgG1_G2S1	14	65855762	1	1.99E-09	1.32E-14	+	0.211114	0.2968	+	T	C	0.57587752	0.0349959	+	T	C	0.5986	0.0385
rs11158591	LC_IGP_R26	IgG1_G2N/IgG1_G1N	14	65855762	1	3.78E-10	3.52E-10	+	0.220714	0.2371	+	T	C	0.577223457	0.0350234	+	T	C	0.5986	0.0378
chr14:65855901:I	LC_IGP62	IgG1 FG0n total/G0n	14	65855901	1	1.74E-09	2.13E-06	-	-0.380741	-0.3354	+	AT	A	0.088626649	0.062879	+	AT	A	0.0842	0.0707
chr14:65855901:I	LC_IGP63	IgG1 FG1n total/G1n	14	65855901	1	3.33E-08	7.09E-07	-	-0.350659	-0.3518	+	AT	A	0.088626649	0.0631767	+	AT	A	0.0842	0.071
chr14:65855901:I	LC_IGP61	IgG1 Fn total	14	65855901	1	2.19E-08	8.24E-07	-	-0.354985	-0.3494	+	AT	A	0.088626649	0.0631165	+	AT	A	0.0842	0.0709
chr14:65855901:I	LC_IGP11	IgG1_G0	14	65855901	1	8.64E-10	2.21E-06	+	0.386088	0.3342	+	AT	A	0.08799231	0.0625843	+	AT	A	0.0842	0.0706
chr14:65855901:I	LC_IGP52	IgG1_G0n	14	65855901	1	4.49E-10	1.46E-06	+	0.393931	0.3407	+	AT	A	0.088626649	0.0627821	+	AT	A	0.0842	0.0707
chr14:65855901:I	LC_IGP_R2	IgG1_G1F/IgG1_G1	14	65855901	1	4.38E-08	1.82E-06	-	-0.345374	-0.3376	+	AT	A	0.088297747	0.0627861	+	AT	A	0.0842	0.0707
rs73282250	LC_IGP62	IgG1 FG0n total/G0n	14	65857146	1	3.41E-08	1.36E-07	-	-0.373765	-0.394	+	G	A	0.076325042	0.0673917	+	G	A	0.0737	0.0748
rs73282250	LC_IGP11	IgG1_G0	14	65857146	1	2.95E-08	9.15E-08	+	0.373691	0.3978	+	G	A	0.075863543	0.0670708	+	G	A	0.0737	0.0744
rs73282250	LC_IGP52	IgG1_G0n	14	65857146	1	1.67E-08	5.63E-08	+	0.381776	0.4051	+	G	A	0.076325042	0.0673142	+	G	A	0.0737	0.0746
rs28668545	LC_IGP62	IgG1 FG0n total/G0n	14	65858308	1	3.12E-08	1.75E-07	-	-0.3761	-0.3908	+	A	G	0.075949073	0.0676194	+	A	G	0.0737	0.0748
rs28668545	LC_IGP11	IgG1_G0	14	65858308	1	3.10E-08	1.26E-07	+	0.374374	0.3939	+	A	G	0.075492854	0.0673014	+	A	G	0.0737	0.0745
rs28668545	LC_IGP52	IgG1_G0n	14	65858308	1	1.76E-08	7.76E-08	+	0.382487	0.4013	+	A	G	0.075949073	0.0675474	+	A	G	0.0737	0.0747
rs73282260	LC_IGP62	IgG1 FG0n total/G0n	14	65858964	1	3.53E-08	9.63E-08	-	-0.37654	-0.4002	+	G	A	0.07478386	0.0679713	+	G	A	0.071	0.075
rs73282260	LC_IGP11	IgG1_G0	14	65858964	1	4.28E-08	8.51E-08	+	0.372445	0.4013	+	G	A	0.074344013	0.0676575	+	G	A	0.071	0.0749
rs73282260	LC_IGP52	IgG1_G0n	14	65858964	1	2.42E-08	4.90E-08	+	0.380742	0.4093	+	G	A	0.07478386	0.0679067	+	G	A	0.071	0.075
rs11158592	LC_IGP_R1	IgG1_G0F/IgG1_G0	14	65859968	1	2.08E-21	2.23E-16	+	0.324194	0.3134	+	T	G	0.497141723	0.0336465	+	T	G	0.531	0.0382
rs11158592	LC_IGP_R2	IgG1_G1F/IgG1_G1	14	65859968	1	7.07E-21	8.94E-18	+	0.320773	0.328	+	T	G	0.497141723	0.03376	+	T	G	0.531	0.0382
rs11158592	LC_IGP_R26	IgG1_G2N/IgG1_G1N	14	65859968	1	9.99E-13	4.66E-12	+	0.246383	0.2572	+	T	G	0.497759568	0.0342762	+	T	G	0.531	0.0372
rs11158592	LC_IGP_R3	IgG1_G2F/IgG1_G2	14	65859968	1	8.75E-18	1.40E-18	+	0.294702	0.3344	+	T	G	0.497139976	0.0339183	+	T	G	0.531	0.038
rs11158592	LC_IGP_R32	IgG1_G1S1/IgG1_G1	14	65859968	1	6.86E-13	2.72E-13	+	0.246729	0.269	+	T	G	0.497138226	0.034077	+	T	G	0.531	0.0368
rs11158592	LC_IGP_R36	IgG1_G1NS1/IgG1_G1N	14	65859968	1	7.21E-09	3.56E-14	+	0.199567	0.2833	+	T	G	0.498356042	0.0343078	+	T	G	0.531	0.0374
rs11158592	LC_IGP_R4	IgG1_G0FN/IgG1_G0N	14	65859968	1	1.64E-15	8.17E-18	+	0.274369	0.331	+	T	G	0.4977624	0.0341028	+	T	G	0.531	0.0385
rs11158592	LC_IGP_R41	IgG2_G0F/IgG2_G0	14	65859968	1	3.56E-08	2.37E-14	+	0.189797	0.288	+	T	G	0.497138447	0.0342724	+	T	G	0.531	0.0378
rs11158592	LC_IGP_R5	IgG1_G1FN/IgG1_G1N	14	65859968	1	6.65E-16	1.47E-21	+	0.279097	0.3657	+	T	G	0.498360701	0.034204	+	T	G	0.531	0.0384
rs11158592	LC_IGP_R64	IgG2_G2/IgG2_G1	14	65859968	1	7.67E-10	8.10E-11	+	0.212925	0.2423	+	T	G	0.496219706	0.034407	+	T	G	0.531	0.0373
rs11158592	LC_IGP_R8	IgG1_G2FS1/IgG1_G2S1	14	65859968	1	3.73E-11	1.45E-17	+	0.227905	0.3233	+	T	G	0.496836286	0.0342208	+	T	G	0.531	0.0379
rs11158592	LC_IGP_SC13	IgG1_G2(20)/IgG2_G2(20)	14	65859968	1	1.15E-08	1.46E-06	-	-0.197377	-0.1778	+	T	G	0.496526606	0.0344094	+	T	G	0.531	0.0369
rs11158592	LC_IGP_SC15	IgG1_G1N(20)/IgG2_G1N(20)	14	65859968	1	8.20E-10	9.65E-07	-	-0.212228	-0.1789	+	T	G	0.498358682	0.034353	+	T	G	0.531	0.0365
rs11158592	LC_IGP11	IgG1_G0	14	65859968	1	1.32E-24	2.39E-19	-	-0.348762	-0.3429	+	T	G	0.497141723	0.0335119	+	T	G	0.531	0.0381
rs11158592	LC_IGP12	IgG1_G1	14	65859968	1	1.18E-19	1.30E-17	-	-0.310578	-0.327	+	T	G	0.497141723	0.0338037	+	T	G	0.531	0.0383
rs11158592	LC_IGP13	IgG1_G2	14	65859968	1	3.58E-09	1.86E-13	-	-0.203782	-0.277	+	T	G	0.497141723	0.0343365	+	T	G	0.531	0.0377
rs11158592	LC_IGP138	IgG2_G0n	14	65859968	1	1.93E-11	6.04E-15	-	-0.231462	-0.2972	+	T	G	0.49626447	0.0342461	+	T	G	0.531	0.0381
rs11158592	LC_IGP14	IgG1_G0N	14	65859968	1	5.32E-18	1.12E-15	-	-0.297213	-0.3044	+	T	G	0.4977624	0.0339764	+	T	G	0.531	0.038
rs11158592	LC_IGP148	IgG2 FG0n total/G0n	14	65859968	1	1.62E-08	7.24E-15	+	0.195396	0.2952	+	T	G	0.49626447	0.0344171	+	T	G	0.531	0.038
rs11158592	LC_IGP15	IgG1_G1N	14	65859968	1	1.23E-12	1.14E-14	-	-0.24586	-0.2941	+	T	G	0.498360701	0.0343422	+	T	G	0.531	0.0381
rs11158592	LC_IGP21	IgG1 Fucosylation	14	65859968	1	1.90E-17	3.40E-18	+	0.292968	0.3327	+	T	G	0.49715074	0.0340795	+	T	G	0.531	0.0383
rs11158592	LC_IGP26	IgG1 GS1/(G+GS1)	14	65859968	1	7.83E-10	1.47E-11	+	0.212133	0.2543	+	T	G	0.497138226	0.0342975	+	T	G	0.531	0.0377
rs11158592	LC_IGP27	IgG1 GS1/(G0+G+GS1)	14	65859968	1	5.17E-12	2.47E-10	+	0.237465	0.237	+	T	G	0.497138226	0.0341546	+	T	G	0.531	0.0374
rs11158592	LC_IGP28	IgG1 G1S1/(G1+G1S1)	14	65859968	1	3.14E-13	1.90E-13	+	0.250281	0.2704	+	T	G	0.497138226	0.0340559	+	T	G	0.531	0.0368
rs11158592	LC_IGP31	IgG1 BGS1/(BG0+BG+BGS1)	14	65859968	1	2.43E-08	3.82E-15	+	0.193245	0.2968	+	T	G	0.498353803	0.0344691	+	T	G	0.5308	0.0378
rs11158592	LC_IGP32	IgG1 BG1S1/(BG1+BG1S1)	14	65859968	1	9.05E-09	3.17E-14	+	0.198209	0.2837	+	T	G	0.498356042	0.0343044	+	T	G	0.531	0.0374
rs11158592	LC_IGP47	IgG1_G1Fn	14	65859968	1	8.33E-09	2.23E-06	+	0.200079	0.1725	+	T	G	0.49713166	0.0345414	+	T	G	0.531	0.0365
rs11158592	LC_IGP52	IgG1_G0n	14	65859968	1	6.63E-24	1.84E-19	-	-0.346241	-0.3446	+	T	G	0.49713166	0.033797	+	T	G	0.531	0.0382
rs11158592	LC_IGP53	IgG1_G1n	14	65859968	1	7.53E-18	2.68E-17	-	-0.297683	-0.3235	+	T	G	0.49713166	0.0341858	+	T	G	0.531	0.0382
rs11158592	LC_IGP54	IgG1_G2n	14	65859968	1	4.56E-08	6.54E-13	-	-0.190653	-0.2699	+	T	G	0.49713166	0.0347031	+	T	G	0.531	0.0375
rs11158592	LC_IGP55	IgG1_G0Nn	14	65859968	1	2.15E-18	1.04E-15	-	-0.302461	-0.305	+	T	G	0.49713166	0.0341563	+	T	G	0.531	0.038
rs11158592	LC_IGP56	IgG1_G1Nn	14	65859968	1	4.70E-13	3.01E-14	-	-0.251424	-0.2889	+	T	G	0.49713166	0.0344702	+	T	G	0.531	0.038
rs11158592	LC_IGP61	IgG1 Fn total	14	65859968	1	8.25E-20	1.14E-18	+	0.314684	0.338	+	T	G	0.49713166	0.0340941	+	T	G	0.531	0.0383
rs11158592	LC_IGP62	IgG1 FG0n total/G0n	14	65859968	1	4.98E-21	6.75E-17	+	0.324032	0.3199	+	T	G	0.49713166	0.0339592	+	T	G	0.531	0.0383
rs11158592	LC_IGP63	IgG1 FG1n total/G1n	14	65859968	1	4.95E-20	1.01E-18	+	0.316763	0.3383	+	T	G	0.49713166	0.0341074	+	T	G	0.531	0.0383
rs11158592	LC_IGP64	IgG1 FG2n total/G2n	14	65859968	1	4.45E-16	4.97E-19	+	0.281908	0.3396	+	T	G	0.49713166	0.0343337	+	T	G	0.531	0.0381
rs11158592	LC_IGP65	IgG1 Fn	14	65859968	1	7.42E-11	1.20E-06	+	0.225663	0.1817	+	T	G	0.49713166	0.0344212	+	T	G	0.531	0.0374
rs11158592	LC_IGP68	IgG1 FG2n/G2n	14	65859968	1	1.62E-11	6.27E-12	+	0.234398	0.2606	+	T	G	0.49713166	0.0345431	+	T	G	0.531	0.0379
rs11158592	LC_IGP77	IgG1 Bn	14	65859968	1	1.99E-14	6.87E-16	-	-0.265679	-0.3086	+	T	G	0.49713166	0.0344044	+	T	G	0.531	0.0382
rs11158592	LC_IGP78	IgG1 BG0n/G0n	14	65859968	1	8.33E-15	5.61E-14	-	-0.2688	-0.2862	+	T	G	0.49713166	0.0342995	+	T	G	0.531	0.0381
rs11158592	LC_IGP79	IgG1 BG1n/G1n	14	65859968	1	1.54E-14	6.74E-16	-	-0.266746	-0.3074	+	T	G	0.49713166	0.0343922	+	T	G	0.531	0.0381
rs11158592	LC_IGP81	IgG1 Fn/Bn	14	65859968	1	8.99E-15	4.72E-15	+	0.268998	0.2981	+	T	G	0.49713166	0.0343688	+	T	G	0.531	0.038
rs11158592	LC_IGP84	IgG1 FBn/Bn total	14	65859968	1	6.90E-17	1.11E-20	+	0.289935	0.3594	+	T	G	0.49713166	0.0343476	+	T	G	0.531	0.0386
rs11158592	LC_IGP86	IgG1 Bn/Fn total	14	65859968	1	1.97E-15	3.04E-16	-	-0.275574	-0.3124	+	T	G	0.49713166	0.0343484	+	T	G	0.531	0.0382
rs11158592	LC_IGP97	IgG2_G0	14	65859968	1	2.29E-11	1.19E-14	-	-0.229822	-0.2936	+	T	G	0.497138447	0.0341339	+	T	G	0.531	0.038
rs11158593	LC_IGP78	IgG1 BG0n/G0n	14	65859984	1	8.68E-15	6.25E-14	-	-0.268627	-0.2862	+	A	G	0.497079618	0.0343009	+	A	G	0.532	0.0381
rs11158593	LC_IGP79	IgG1 BG1n/G1n	14	65859984	1	1.62E-14	7.60E-16	-	-0.266537	-0.3074	+	A	G	0.497079618	0.0343938	+	A	G	0.532	0.0381
rs11158593	LC_IGP32	IgG1 BG1S1/(BG1+BG1S1)	14	65859984	1	9.38E-09	3.55E-14	+	0.198006	0.2836	+	A	G	0.498304255	0.0343057	+	A	G	0.532	0.0374
rs11158593	LC_IGP31	IgG1 BGS1/(BG0+BG+BGS1)	14	65859984	1	2.50E-08	4.59E-15	+	0.193086	0.2965	+	A	G	0.498301747	0.0344702	+	A	G	0.5317	0.0378
rs11158593	LC_IGP77	IgG1 Bn	14	65859984	1	2.10E-14	7.77E-16	-	-0.265463	-0.3086	+	A	G	0.497079618	0.0344061	+	A	G	0.532	0.0383
rs11158593	LC_IGP86	IgG1 Bn/Fn total	14	65859984	1	2.07E-15	3.42E-16	-	-0.275369	-0.3125	+	A	G	0.497079618	0.03435	+	A	G	0.532	0.0383
rs11158593	LC_IGP84	IgG1 FBn/Bn total	14	65859984	1	7.12E-17	1.26E-20	+	0.289811	0.3596	+	A	G	0.497079618	0.0343489	+	A	G	0.532	0.0386
rs11158593	LC_IGP62	IgG1 FG0n total/G0n	14	65859984	1	5.10E-21	7.81E-17	+	0.323953	0.3199	+	A	G	0.497079618	0.0339603	+	A	G	0.532	0.0384
rs11158593	LC_IGP63	IgG1 FG1n total/G1n	14	65859984	1	5.04E-20	1.16E-18	+	0.316704	0.3384	+	A	G	0.497079618	0.0341083	+	A	G	0.532	0.0384
rs11158593	LC_IGP64	IgG1 FG2n total/G2n	14	65859984	1	4.39E-16	5.66E-19	+	0.281968	0.3397	+	A	G	0.497079618	0.0343341	+	A	G	0.532	0.0382
rs11158593	LC_IGP68	IgG1 FG2n/G2n	14	65859984	1	1.61E-11	6.67E-12	+	0.234418	0.2608	+	A	G	0.497079618	0.0345436	+	A	G	0.532	0.038
rs11158593	LC_IGP65	IgG1 Fn	14	65859984	1	7.71E-11	1.26E-06	+	0.225472	0.1816	+	A	G	0.497079618	0.0344226	+	A	G	0.532	0.0375
rs11158593	LC_IGP61	IgG1 Fn total	14	65859984	1	8.31E-20	1.33E-18	+	0.31466	0.338	+	A	G	0.497079618	0.0340949	+	A	G	0.532	0.0384
rs11158593	LC_IGP81	IgG1 Fn/Bn	14	65859984	1	9.46E-15	5.30E-15	+	0.268785	0.2981	+	A	G	0.497079618	0.0343705	+	A	G	0.532	0.0381
rs11158593	LC_IGP21	IgG1 Fucosylation	14	65859984	1	1.87E-17	3.88E-18	+	0.293032	0.3328	+	A	G	0.497098645	0.0340799	+	A	G	0.532	0.0383
rs11158593	LC_IGP28	IgG1 G1S1/(G1+G1S1)	14	65859984	1	3.21E-13	1.99E-13	+	0.25018	0.2707	+	A	G	0.497087158	0.0340569	+	A	G	0.532	0.0368
rs11158593	LC_IGP26	IgG1 GS1/(G+GS1)	14	65859984	1	8.15E-10	1.67E-11	+	0.211915	0.254	+	A	G	0.497087158	0.0342989	+	A	G	0.532	0.0377
rs11158593	LC_IGP27	IgG1 GS1/(G0+G+GS1)	14	65859984	1	5.44E-12	2.78E-10	+	0.237219	0.2367	+	A	G	0.497087158	0.0341562	+	A	G	0.532	0.0375
rs11158593	LC_IGP11	IgG1_G0	14	65859984	1	1.37E-24	2.82E-19	-	-0.348668	-0.3428	+	A	G	0.497090716	0.0335131	+	A	G	0.532	0.0382
rs11158593	LC_IGP_R1	IgG1_G0F/IgG1_G0	14	65859984	1	2.12E-21	2.60E-16	+	0.324127	0.3133	+	A	G	0.497090716	0.0336475	+	A	G	0.532	0.0382
rs11158593	LC_IGP_R4	IgG1_G0FN/IgG1_G0N	14	65859984	1	1.68E-15	9.04E-18	+	0.274276	0.3311	+	A	G	0.497710963	0.0341039	+	A	G	0.532	0.0386
rs11158593	LC_IGP52	IgG1_G0n	14	65859984	1	6.82E-24	2.17E-19	-	-0.346156	-0.3445	+	A	G	0.497079618	0.0337981	+	A	G	0.532	0.0383
rs11158593	LC_IGP14	IgG1_G0N	14	65859984	1	5.62E-18	1.27E-15	-	-0.297013	-0.3043	+	A	G	0.497710963	0.0339781	+	A	G	0.532	0.038
rs11158593	LC_IGP55	IgG1_G0Nn	14	65859984	1	2.27E-18	1.18E-15	-	-0.302268	-0.3049	+	A	G	0.497079618	0.034158	+	A	G	0.532	0.0381
rs11158593	LC_IGP12	IgG1_G1	14	65859984	1	1.19E-19	1.49E-17	-	-0.310563	-0.327	+	A	G	0.497090716	0.0338044	+	A	G	0.532	0.0384
rs11158593	LC_IGP_R2	IgG1_G1F/IgG1_G1	14	65859984	1	7.15E-21	1.02E-17	+	0.320739	0.3281	+	A	G	0.497090716	0.0337608	+	A	G	0.532	0.0383
rs11158593	LC_IGP47	IgG1_G1Fn	14	65859984	1	8.57E-09	2.19E-06	+	0.199918	0.1729	+	A	G	0.497079618	0.0345426	+	A	G	0.532	0.0365
rs11158593	LC_IGP_R5	IgG1_G1FN/IgG1_G1N	14	65859984	1	6.89E-16	1.70E-21	+	0.278955	0.3658	+	A	G	0.498309042	0.0342053	+	A	G	0.532	0.0384
rs11158593	LC_IGP53	IgG1_G1n	14	65859984	1	7.54E-18	3.05E-17	-	-0.297684	-0.3236	+	A	G	0.497079618	0.0341864	+	A	G	0.532	0.0383
rs11158593	LC_IGP15	IgG1_G1N	14	65859984	1	1.28E-12	1.29E-14	-	-0.245641	-0.294	+	A	G	0.498309042	0.0343438	+	A	G	0.532	0.0382
rs11158593	LC_IGP_SC15	IgG1_G1N(20)/IgG2_G1N(20)	14	65859984	1	8.31E-10	8.73E-07	-	-0.212156	-0.1798	+	A	G	0.498306959	0.0343539	+	A	G	0.532	0.0366
rs11158593	LC_IGP56	IgG1_G1Nn	14	65859984	1	4.92E-13	3.38E-14	-	-0.251213	-0.2889	+	A	G	0.497079618	0.0344718	+	A	G	0.532	0.0381
rs11158593	LC_IGP_R36	IgG1_G1NS1/IgG1_G1N	14	65859984	1	7.47E-09	3.98E-14	+	0.199365	0.2833	+	A	G	0.498304255	0.0343091	+	A	G	0.532	0.0375
rs11158593	LC_IGP_R32	IgG1_G1S1/IgG1_G1	14	65859984	1	7.02E-13	2.85E-13	+	0.246629	0.2692	+	A	G	0.497087158	0.034078	+	A	G	0.532	0.0369
rs11158593	LC_IGP13	IgG1_G2	14	65859984	1	3.49E-09	2.05E-13	-	-0.20394	-0.2771	+	A	G	0.497090716	0.0343365	+	A	G	0.532	0.0377
rs11158593	LC_IGP_SC13	IgG1_G2(20)/IgG2_G2(20)	14	65859984	1	1.15E-08	1.41E-06	-	-0.197407	-0.1784	+	A	G	0.496475537	0.0344099	+	A	G	0.532	0.037
rs11158593	LC_IGP_R3	IgG1_G2F/IgG1_G2	14	65859984	1	8.55E-18	1.59E-18	+	0.294796	0.3345	+	A	G	0.497088938	0.0339184	+	A	G	0.532	0.0381
rs11158593	LC_IGP_R8	IgG1_G2FS1/IgG1_G2S1	14	65859984	1	3.57E-11	1.63E-17	+	0.228129	0.3235	+	A	G	0.49678528	0.0342205	+	A	G	0.532	0.038
rs11158593	LC_IGP54	IgG1_G2n	14	65859984	1	4.43E-08	7.15E-13	-	-0.190834	-0.2699	+	A	G	0.497079618	0.0347031	+	A	G	0.532	0.0376
rs11158593	LC_IGP_R26	IgG1_G2N/IgG1_G1N	14	65859984	1	1.03E-12	5.35E-12	+	0.246236	0.2568	+	A	G	0.497707717	0.0342775	+	A	G	0.532	0.0372
rs11158593	LC_IGP148	IgG2 FG0n total/G0n	14	65859984	1	1.64E-08	8.67E-15	+	0.195311	0.2948	+	A	G	0.496211825	0.0344182	+	A	G	0.532	0.038
rs11158593	LC_IGP97	IgG2_G0	14	65859984	1	2.35E-11	1.41E-14	-	-0.229714	-0.2932	+	A	G	0.49708741	0.0341349	+	A	G	0.532	0.0381
rs11158593	LC_IGP_R41	IgG2_G0F/IgG2_G0	14	65859984	1	3.60E-08	2.76E-14	+	0.189734	0.2877	+	A	G	0.49708741	0.0342732	+	A	G	0.532	0.0378
rs11158593	LC_IGP138	IgG2_G0n	14	65859984	1	1.97E-11	7.15E-15	-	-0.231368	-0.2969	+	A	G	0.496211825	0.0342472	+	A	G	0.532	0.0382
rs11158593	LC_IGP_R64	IgG2_G2/IgG2_G1	14	65859984	1	7.95E-10	8.67E-11	+	0.212735	0.2423	+	A	G	0.496168607	0.0344083	+	A	G	0.532	0.0373

rs10138570	LC_IGP78	IgG1 BG0n/G0n	14	65860038	1	9.98E-15	7.13E-14	-	-0.267926	-0.2848	+	G	A	0.496490397	0.0342913	+	G	A	0.5305	0.038
rs10138570	LC_IGP79	IgG1 BG1n/G1n	14	65860038	1	1.88E-14	7.99E-16	-	-0.265799	-0.3066	+	G	A	0.496490397	0.0343842	+	G	A	0.5305	0.0381
rs10138570	LC_IGP32	IgG1 BG1S1/(BG1+BG1S1)	14	65860038	1	6.43E-09	3.34E-14	+	0.200105	0.2836	+	G	A	0.497717633	0.0342852	+	G	A	0.5306	0.0374
rs10138570	LC_IGP31	IgG1 BGS1/(BG0+BG+BGS1)	14	65860038	1	1.83E-08	4.08E-15	+	0.194872	0.2967	+	G	A	0.497709788	0.0344508	+	G	A	0.5303	0.0378
rs10138570	LC_IGP77	IgG1 Bn	14	65860038	1	2.57E-14	8.40E-16	-	-0.264471	-0.3075	+	G	A	0.496490397	0.0343977	+	G	A	0.5305	0.0382
rs10138570	LC_IGP86	IgG1 Bn/Fn total	14	65860038	1	2.54E-15	3.74E-16	-	-0.274403	-0.3114	+	G	A	0.496490397	0.0343417	+	G	A	0.5305	0.0382
rs10138570	LC_IGP84	IgG1 FBn/Bn total	14	65860038	1	9.04E-17	1.32E-20	+	0.288743	0.3589	+	G	A	0.496490397	0.0343413	+	G	A	0.5305	0.0386
rs10138570	LC_IGP62	IgG1 FG0n total/G0n	14	65860038	1	5.87E-21	8.96E-17	+	0.323343	0.3184	+	G	A	0.496490397	0.0339507	+	G	A	0.5305	0.0383
rs10138570	LC_IGP63	IgG1 FG1n total/G1n	14	65860038	1	6.82E-20	1.28E-18	+	0.315487	0.3372	+	G	A	0.496490397	0.0341021	+	G	A	0.5305	0.0383
rs10138570	LC_IGP64	IgG1 FG2n total/G2n	14	65860038	1	8.01E-16	6.07E-19	+	0.279379	0.3388	+	G	A	0.496490397	0.0343341	+	G	A	0.5305	0.0381
rs10138570	LC_IGP68	IgG1 FG2n/G2n	14	65860038	1	2.80E-11	7.76E-12	+	0.23157	0.2593	+	G	A	0.496490397	0.0345425	+	G	A	0.5305	0.0379
rs10138570	LC_IGP65	IgG1 Fn	14	65860038	1	8.51E-11	1.46E-06	+	0.224884	0.18	+	G	A	0.496490397	0.034412	+	G	A	0.5305	0.0374
rs10138570	LC_IGP61	IgG1 Fn total	14	65860038	1	1.08E-19	1.49E-18	+	0.3136	0.3367	+	G	A	0.496490397	0.0340877	+	G	A	0.5305	0.0383
rs10138570	LC_IGP81	IgG1 Fn/Bn	14	65860038	1	1.16E-14	5.81E-15	+	0.26782	0.2969	+	G	A	0.496490397	0.0343621	+	G	A	0.5305	0.038
rs10138570	LC_IGP21	IgG1 Fucosylation	14	65860038	1	2.21E-17	4.51E-18	+	0.292284	0.3313	+	G	A	0.496509864	0.0340711	+	G	A	0.5305	0.0382
rs10138570	LC_IGP28	IgG1 G1S1/(G1+G1S1)	14	65860038	1	2.95E-13	2.33E-13	+	0.250478	0.2693	+	G	A	0.496505199	0.0340426	+	G	A	0.5305	0.0368
rs10138570	LC_IGP26	IgG1 GS1/(G+GS1)	14	65860038	1	7.97E-10	1.64E-11	+	0.211963	0.2539	+	G	A	0.496505199	0.0342858	+	G	A	0.5305	0.0377
rs10138570	LC_IGP27	IgG1 GS1/(G0+G+GS1)	14	65860038	1	5.85E-12	2.64E-10	+	0.236778	0.2368	+	G	A	0.496505199	0.0341452	+	G	A	0.5305	0.0375
rs10138570	LC_IGP11	IgG1_G0	14	65860038	1	2.43E-24	2.95E-19	-	-0.346693	-0.342	+	G	A	0.496509469	0.0335125	+	G	A	0.5305	0.0381
rs10138570	LC_IGP_R1	IgG1_G0F/IgG1_G0	14	65860038	1	2.39E-21	2.99E-16	+	0.323594	0.3118	+	G	A	0.496509469	0.0336376	+	G	A	0.5305	0.0381
rs10138570	LC_IGP_R4	IgG1_G0FN/IgG1_G0N	14	65860038	1	2.09E-15	1.02E-17	+	0.273265	0.33	+	G	A	0.497127373	0.0340958	+	G	A	0.5305	0.0385
rs10138570	LC_IGP52	IgG1_G0n	14	65860038	1	1.19E-23	2.30E-19	-	-0.344197	-0.3437	+	G	A	0.496490397	0.0337973	+	G	A	0.5305	0.0382
rs10138570	LC_IGP14	IgG1_G0N	14	65860038	1	8.83E-18	1.31E-15	-	-0.29516	-0.3036	+	G	A	0.497127373	0.0339749	+	G	A	0.5305	0.038
rs10138570	LC_IGP55	IgG1_G0Nn	14	65860038	1	3.58E-18	1.24E-15	-	-0.300416	-0.3042	+	G	A	0.496490397	0.034155	+	G	A	0.5305	0.038
rs10138570	LC_IGP12	IgG1_G1	14	65860038	1	1.39E-19	1.69E-17	-	-0.309863	-0.3258	+	G	A	0.496509469	0.0337953	+	G	A	0.5305	0.0383
rs10138570	LC_IGP_R2	IgG1_G1F/IgG1_G1	14	65860038	1	1.06E-20	1.15E-17	+	0.319249	0.3268	+	G	A	0.496509469	0.0337564	+	G	A	0.5305	0.0382
rs10138570	LC_IGP47	IgG1_G1Fn	14	65860038	1	1.46E-08	2.27E-06	+	0.196733	0.1724	+	G	A	0.496490397	0.034541	+	G	A	0.5305	0.0365
rs10138570	LC_IGP_R5	IgG1_G1FN/IgG1_G1N	14	65860038	1	8.34E-16	1.67E-21	+	0.278064	0.3654	+	G	A	0.497723862	0.0341966	+	G	A	0.5305	0.0384
rs10138570	LC_IGP53	IgG1_G1n	14	65860038	1	8.30E-18	3.52E-17	-	-0.297198	-0.3222	+	G	A	0.496490397	0.0341759	+	G	A	0.5305	0.0382
rs10138570	LC_IGP15	IgG1_G1N	14	65860038	1	1.33E-12	1.37E-14	-	-0.245372	-0.2931	+	G	A	0.497723862	0.0343319	+	G	A	0.5305	0.0381
rs10138570	LC_IGP_SC15	IgG1_G1N(20)/IgG2_G1N(20)	14	65860038	1	5.84E-10	1.05E-06	-	-0.213983	-0.1783	+	G	A	0.497721059	0.0343339	+	G	A	0.5305	0.0365
rs10138570	LC_IGP56	IgG1_G1Nn	14	65860038	1	5.03E-13	3.65E-14	-	-0.251022	-0.2879	+	G	A	0.496490397	0.0344595	+	G	A	0.5305	0.038
rs10138570	LC_IGP_R36	IgG1_G1NS1/IgG1_G1N	14	65860038	1	5.18E-09	3.75E-14	+	0.201381	0.2832	+	G	A	0.497717633	0.0342889	+	G	A	0.5306	0.0374
rs10138570	LC_IGP_R32	IgG1_G1S1/IgG1_G1	14	65860038	1	6.53E-13	3.34E-13	+	0.246873	0.2679	+	G	A	0.496505199	0.034064	+	G	A	0.5305	0.0368
rs10138570	LC_IGP13	IgG1_G2	14	65860038	1	3.67E-09	2.39E-13	-	-0.203582	-0.2757	+	G	A	0.496509469	0.0343249	+	G	A	0.5305	0.0376
rs10138570	LC_IGP_SC13	IgG1_G2(20)/IgG2_G2(20)	14	65860038	1	8.64E-09	1.68E-06	-	-0.198977	-0.1768	+	G	A	0.495893578	0.0343915	+	G	A	0.5305	0.0369
rs10138570	LC_IGP_R3	IgG1_G2F/IgG1_G2	14	65860038	1	1.58E-17	1.74E-18	+	0.292305	0.3335	+	G	A	0.496507335	0.0339185	+	G	A	0.5305	0.038
rs10138570	LC_IGP_R8	IgG1_G2FS1/IgG1_G2S1	14	65860038	1	4.92E-11	1.86E-17	+	0.226436	0.3222	+	G	A	0.496204032	0.0342145	+	G	A	0.5305	0.0379
rs10138570	LC_IGP54	IgG1_G2n	14	65860038	1	4.56E-08	8.44E-13	-	-0.190586	-0.2685	+	G	A	0.496490397	0.034691	+	G	A	0.5305	0.0375
rs10138570	LC_IGP_R26	IgG1_G2N/IgG1_G1N	14	65860038	1	8.28E-13	5.23E-12	+	0.247174	0.2567	+	G	A	0.497120679	0.0342603	+	G	A	0.5305	0.0372
rs10138570	LC_IGP148	IgG2 FG0n total/G0n	14	65860038	1	1.63E-08	8.88E-15	+	0.1953	0.2941	+	G	A	0.495621305	0.0344059	+	G	A	0.5306	0.0379
rs10138570	LC_IGP97	IgG2_G0	14	65860038	1	2.59E-11	1.34E-14	-	-0.229142	-0.293	+	G	A	0.496505807	0.0341244	+	G	A	0.5305	0.038
rs10138570	LC_IGP_R41	IgG2_G0F/IgG2_G0	14	65860038	1	3.51E-08	2.85E-14	+	0.189812	0.287	+	G	A	0.496505807	0.0342601	+	G	A	0.5305	0.0377
rs10138570	LC_IGP138	IgG2_G0n	14	65860038	1	2.13E-11	6.90E-15	-	-0.230896	-0.2965	+	G	A	0.495621305	0.0342368	+	G	A	0.5306	0.0381
rs10138570	LC_IGP_R64	IgG2_G2/IgG2_G1	14	65860038	1	7.42E-10	7.94E-11	+	0.213027	0.2426	+	G	A	0.495585985	0.0343944	+	G	A	0.5305	0.0373
rs10138671	LC_IGP78	IgG1 BG0n/G0n	14	65860092	1	2.91E-08	1.26E-06	-	-0.198312	-0.1865	+	G	A	0.571274473	0.0355775	+	G	A	0.6016	0.0385
rs10138671	LC_IGP79	IgG1 BG1n/G1n	14	65860092	1	1.99E-08	5.07E-08	-	-0.201117	-0.2094	+	G	A	0.571274473	0.0356521	+	G	A	0.6016	0.0384
rs10138671	LC_IGP77	IgG1 Bn	14	65860092	1	1.84E-08	6.54E-08	-	-0.201641	-0.2087	+	G	A	0.571274473	0.0356575	+	G	A	0.6016	0.0386
rs10138671	LC_IGP86	IgG1 Bn/Fn total	14	65860092	1	6.41E-09	4.10E-08	-	-0.207961	-0.212	+	G	A	0.571274473	0.0356271	+	G	A	0.6016	0.0386
rs10138671	LC_IGP84	IgG1 FBn/Bn total	14	65860092	1	8.39E-10	1.93E-10	+	0.220151	0.2478	+	G	A	0.571274473	0.0356555	+	G	A	0.6016	0.0389
rs10138671	LC_IGP62	IgG1 FG0n total/G0n	14	65860092	1	1.32E-11	1.25E-08	+	0.24106	0.2205	+	G	A	0.571274473	0.0353699	+	G	A	0.6016	0.0387
rs10138671	LC_IGP63	IgG1 FG1n total/G1n	14	65860092	1	1.62E-11	4.39E-10	+	0.240736	0.2414	+	G	A	0.571274473	0.0354786	+	G	A	0.6016	0.0387
rs10138671	LC_IGP64	IgG1 FG2n total/G2n	14	65860092	1	6.24E-09	3.09E-10	+	0.20822	0.2422	+	G	A	0.571274473	0.0356437	+	G	A	0.6016	0.0385
rs10138671	LC_IGP61	IgG1 Fn total	14	65860092	1	3.15E-11	6.43E-10	+	0.237147	0.2393	+	G	A	0.571274473	0.0354679	+	G	A	0.6016	0.0387
rs10138671	LC_IGP81	IgG1 Fn/Bn	14	65860092	1	1.42E-08	1.85E-07	+	0.203132	0.2005	+	G	A	0.571274473	0.0356323	+	G	A	0.6016	0.0384
rs10138671	LC_IGP21	IgG1 Fucosylation	14	65860092	1	5.04E-10	2.05E-09	+	0.221356	0.2318	+	G	A	0.570925401	0.0353819	+	G	A	0.6016	0.0387
rs10138671	LC_IGP28	IgG1 G1S1/(G1+G1S1)	14	65860092	1	4.43E-08	2.28E-07	+	0.193773	0.1924	+	G	A	0.570940673	0.0352383	+	G	A	0.6016	0.0372
rs10138671	LC_IGP11	IgG1_G0	14	65860092	1	2.54E-14	2.28E-10	-	-0.268544	-0.2443	+	G	A	0.570854001	0.0349257	+	G	A	0.6016	0.0385
rs10138671	LC_IGP_R1	IgG1_G0F/IgG1_G0	14	65860092	1	2.93E-12	1.72E-08	+	0.246269	0.2176	+	G	A	0.570854001	0.0350098	+	G	A	0.6016	0.0386
rs10138671	LC_IGP_R4	IgG1_G0FN/IgG1_G0N	14	65860092	1	5.33E-09	1.35E-08	+	0.207574	0.2208	+	G	A	0.571653705	0.0353739	+	G	A	0.6016	0.0389
rs10138671	LC_IGP52	IgG1_G0n	14	65860092	1	1.40E-13	2.62E-10	-	-0.26298	-0.2439	+	G	A	0.571274473	0.0352465	+	G	A	0.6016	0.0386
rs10138671	LC_IGP14	IgG1_G0N	14	65860092	1	1.91E-10	1.74E-07	-	-0.226224	-0.2005	+	G	A	0.571653705	0.0352947	+	G	A	0.6016	0.0384
rs10138671	LC_IGP55	IgG1_G0Nn	14	65860092	1	1.74E-10	1.82E-07	-	-0.228062	-0.2004	+	G	A	0.571274473	0.0354985	+	G	A	0.6016	0.0384
rs10138671	LC_IGP12	IgG1_G1	14	65860092	1	2.89E-11	1.11E-09	-	-0.235251	-0.2357	+	G	A	0.571159438	0.0351197	+	G	A	0.6016	0.0387
rs10138671	LC_IGP_R2	IgG1_G1F/IgG1_G1	14	65860092	1	6.02E-12	1.18E-09	+	0.24326	0.2348	+	G	A	0.571159438	0.0351011	+	G	A	0.6016	0.0386
rs10138671	LC_IGP_R5	IgG1_G1FN/IgG1_G1N	14	65860092	1	2.04E-09	5.82E-11	+	0.21385	0.2535	+	G	A	0.57226353	0.035469	+	G	A	0.6016	0.0387
rs10138671	LC_IGP53	IgG1_G1n	14	65860092	1	2.08E-10	1.98E-09	-	-0.227117	-0.2319	+	G	A	0.571274473	0.0355057	+	G	A	0.6016	0.0387
rs10138671	LC_IGP_R3	IgG1_G2F/IgG1_G2	14	65860092	1	7.36E-10	6.45E-10	+	0.21826	0.2374	+	G	A	0.570897311	0.0352319	+	G	A	0.6016	0.0384
rs10138765	LC_IGP52	IgG1_G0n	14	65860239	1	4.08E-08	1.64E-07	+	0.372208	0.3914	+	G	A	0.075367287	0.0675043	+	G	A	0.0714	0.0748
rs6573609	LC_IGP78	IgG1 BG0n/G0n	14	65861402	1	1.61E-09	1.27E-06	-	-0.216397	-0.1878	+	C	A	0.581700434	0.0356592	+	C	A	0.6103	0.0388
rs6573609	LC_IGP79	IgG1 BG1n/G1n	14	65861402	1	2.18E-09	7.64E-08	-	-0.215135	-0.2084	+	C	A	0.581700434	0.0357487	+	C	A	0.6103	0.0388
rs6573609	LC_IGP77	IgG1 Bn	14	65861402	1	1.36E-09	8.80E-08	-	-0.217902	-0.2084	+	C	A	0.581700434	0.0357456	+	C	A	0.6103	0.039
rs6573609	LC_IGP86	IgG1 Bn/Fn total	14	65861402	1	4.15E-10	5.64E-08	-	-0.224539	-0.2116	+	C	A	0.581700434	0.0357122	+	C	A	0.6103	0.039
rs6573609	LC_IGP84	IgG1 FBn/Bn total	14	65861402	1	3.79E-11	2.25E-10	+	0.237916	0.249	+	C	A	0.581700434	0.0357328	+	C	A	0.6103	0.0392
rs6573609	LC_IGP62	IgG1 FG0n total/G0n	14	65861402	1	8.16E-13	1.20E-08	+	0.255859	0.2226	+	C	A	0.581700434	0.035453	+	C	A	0.6103	0.039
rs6573609	LC_IGP63	IgG1 FG1n total/G1n	14	65861402	1	1.17E-12	4.97E-10	+	0.254867	0.2427	+	C	A	0.581700434	0.0355653	+	C	A	0.6103	0.039
rs6573609	LC_IGP64	IgG1 FG2n total/G2n	14	65861402	1	5.51E-10	3.21E-10	+	0.223063	0.244	+	C	A	0.581700434	0.0357356	+	C	A	0.6103	0.0388
rs6573609	LC_IGP61	IgG1 Fn total	14	65861402	1	2.16E-12	6.75E-10	+	0.251685	0.241	+	C	A	0.581700434	0.0355536	+	C	A	0.6103	0.039
rs6573609	LC_IGP81	IgG1 Fn/Bn	14	65861402	1	1.07E-09	2.31E-07	+	0.219155	0.2006	+	C	A	0.581700434	0.0357208	+	C	A	0.6103	0.0388
rs6573609	LC_IGP21	IgG1 Fucosylation	14	65861402	1	1.72E-11	3.36E-09	+	0.240254	0.2306	+	C	A	0.581319051	0.0354531	+	C	A	0.6103	0.039
rs6573609	LC_IGP28	IgG1 G1S1/(G1+G1S1)	14	65861402	1	2.70E-08	4.39E-07	+	0.197613	0.1891	+	C	A	0.581252294	0.0353694	+	C	A	0.6103	0.0374
rs6573609	LC_IGP11	IgG1_G0	14	65861402	1	1.75E-15	1.78E-10	-	-0.281373	-0.2476	+	C	A	0.581153024	0.0350091	+	C	A	0.6103	0.0388
rs6573609	LC_IGP_R1	IgG1_G0F/IgG1_G0	14	65861402	1	2.49E-13	1.58E-08	+	0.259063	0.2201	+	C	A	0.581153024	0.035098	+	C	A	0.6103	0.0389
rs6573609	LC_IGP_R4	IgG1_G0FN/IgG1_G0N	14	65861402	1	2.28E-10	1.29E-08	+	0.226214	0.2228	+	C	A	0.581958971	0.0354496	+	C	A	0.6103	0.0392
rs6573609	LC_IGP52	IgG1_G0n	14	65861402	1	9.97E-15	2.02E-10	-	-0.276064	-0.2474	+	C	A	0.581700434	0.0353323	+	C	A	0.6103	0.0389
rs6573609	LC_IGP14	IgG1_G0N	14	65861402	1	5.99E-12	1.48E-07	-	-0.24511	-0.2031	+	C	A	0.581958971	0.0353634	+	C	A	0.6103	0.0386
rs6573609	LC_IGP55	IgG1_G0Nn	14	65861402	1	5.63E-12	1.56E-07	-	-0.246874	-0.203	+	C	A	0.581700434	0.0355681	+	C	A	0.6103	0.0387
rs6573609	LC_IGP12	IgG1_G1	14	65861402	1	2.15E-12	1.27E-09	-	-0.249209	-0.2368	+	C	A	0.581458461	0.0352064	+	C	A	0.6103	0.039
rs6573609	LC_IGP_R2	IgG1_G1F/IgG1_G1	14	65861402	1	5.05E-13	1.28E-09	+	0.256296	0.2362	+	C	A	0.581458461	0.0351902	+	C	A	0.6103	0.0389
rs6573609	LC_IGP_R5	IgG1_G1FN/IgG1_G1N	14	65861402	1	1.23E-10	6.48E-11	+	0.230299	0.2551	+	C	A	0.582612854	0.0355541	+	C	A	0.6103	0.039
rs6573609	LC_IGP53	IgG1_G1n	14	65861402	1	1.91E-11	2.22E-09	-	-0.240689	-0.2331	+	C	A	0.581700434	0.0355979	+	C	A	0.6103	0.039
rs6573609	LC_IGP15	IgG1_G1N	14	65861402	1	2.78E-08	3.21E-07	-	-0.199009	-0.1983	+	C	A	0.582612854	0.0356513	+	C	A	0.6103	0.0388
rs6573609	LC_IGP56	IgG1_G1Nn	14	65861402	1	1.66E-08	6.10E-07	-	-0.203092	-0.1932	+	C	A	0.581700434	0.035799	+	C	A	0.6103	0.0387
rs6573609	LC_IGP_R32	IgG1_G1S1/IgG1_G1	14	65861402	1	3.40E-08	4.95E-07	+	0.196226	0.1885	+	C	A	0.581252294	0.0353796	+	C	A	0.6103	0.0375
rs6573609	LC_IGP_R3	IgG1_G2F/IgG1_G2	14	65861402	1	5.73E-11	6.73E-10	+	0.232926	0.239	+	C	A	0.581202628	0.0353193	+	C	A	0.6103	0.0387
rs4899178	LC_IGP78	IgG1 BG0n/G0n	14	65862987	1	1.61E-09	1.41E-06	-	-0.216357	-0.1872	+	T	C	0.581700434	0.0356571	+	T	C	0.6102	0.0388
rs4899178	LC_IGP79	IgG1 BG1n/G1n	14	65862987	1	2.19E-09	8.44E-08	-	-0.21509	-0.2078	+	T	C	0.581700434	0.0357466	+	T	C	0.6102	0.0388
rs4899178	LC_IGP77	IgG1 Bn	14	65862987	1	1.37E-09	9.92E-08	-	-0.217855	-0.2076	+	T	C	0.581700434	0.0357436	+	T	C	0.6102	0.039
rs4899178	LC_IGP86	IgG1 Bn/Fn total	14	65862987	1	4.17E-10	6.45E-08	-	-0.224492	-0.2108	+	T	C	0.581700434	0.0357102	+	T	C	0.6102	0.039
rs4899178	LC_IGP84	IgG1 FBn/Bn total	14	65862987	1	3.80E-11	2.55E-10	+	0.237881	0.2484	+	T	C	0.581700434	0.0357307	+	T	C	0.6102	0.0393
rs4899178	LC_IGP62	IgG1 FG0n total/G0n	14	65862987	1	8.21E-13	1.34E-08	+	0.255816	0.222	+	T	C	0.581700434	0.035451	+	T	C	0.6102	0.0391
rs4899178	LC_IGP63	IgG1 FG1n total/G1n	14	65862987	1	1.18E-12	5.49E-10	+	0.254826	0.2422	+	T	C	0.581700434	0.0355633	+	T	C	0.6102	0.039
rs4899178	LC_IGP64	IgG1 FG2n total/G2n	14	65862987	1	5.53E-10	3.55E-10	+	0.22303	0.2436	+	T	C	0.581700434	0.0357335	+	T	C	0.6102	0.0388
rs4899178	LC_IGP61	IgG1 Fn total	14	65862987	1	2.17E-12	7.47E-10	+	0.251643	0.2406	+	T	C	0.581700434	0.0355516	+	T	C	0.6102	0.0391
rs4899178	LC_IGP81	IgG1 Fn/Bn	14	65862987	1	1.07E-09	2.56E-07	+	0.219106	0.2	+	T	C	0.581700434	0.0357188	+	T	C	0.6102	0.0388
rs4899178	LC_IGP21	IgG1 Fucosylation	14	65862987	1	1.72E-11	3.68E-09	+	0.240217	0.2302	+	T	C	0.581318742	0.035451	+	T	C	0.6102	0.039
rs4899178	LC_IGP28	IgG1 G1S1/(G1+G1S1)	14	65862987	1	2.71E-08	4.56E-07	+	0.197587	0.189	+	T	C	0.581251988	0.0353673	+	T	C	0.6102	0.0375
rs4899178	LC_IGP11	IgG1_G0	14	65862987	1	1.76E-15	2.12E-10	-	-0.281332	-0.2467	+	T	C	0.581152718	0.0350071	+	T	C	0.6102	0.0388
rs4899178	LC_IGP_R1	IgG1_G0F/IgG1_G0	14	65862987	1	2.50E-13	1.76E-08	+	0.259017	0.2195	+	T	C	0.581152718	0.035096	+	T	C	0.6102	0.039
rs4899178	LC_IGP_R4	IgG1_G0FN/IgG1_G0N	14	65862987	1	2.29E-10	1.43E-08	+	0.226188	0.2222	+	T	C	0.581958971	0.0354475	+	T	C	0.6102	0.0392
rs4899178	LC_IGP52	IgG1_G0n	14	65862987	1	1.00E-14	2.40E-10	-	-0.276022	-0.2465	+	T	C	0.581700434	0.0353303	+	T	C	0.6102	0.0389
rs4899178	LC_IGP14	IgG1_G0N	14	65862987	1	6.02E-12	1.74E-07	-	-0.245073	-0.2021	+	T	C	0.581958971	0.0353614	+	T	C	0.6102	0.0387
rs4899178	LC_IGP55	IgG1_G0Nn	14	65862987	1	5.65E-12	1.82E-07	-	-0.246837	-0.202	+	T	C	0.581700434	0.035566	+	T	C	0.6102	0.0387
rs4899178	LC_IGP12	IgG1_G1	14	65862987	1	2.17E-12	1.38E-09	-	-0.24917	-0.2364	+	T	C	0.581458155	0.0352044	+	T	C	0.6102	0.039
rs4899178	LC_IGP_R2	IgG1_G1F/IgG1_G1	14	65862987	1	5.08E-13	1.41E-09	+	0.256253	0.2358	+	T	C	0.581458155	0.0351882	+	T	C	0.6102	0.039
rs4899178	LC_IGP_R5	IgG1_G1FN/IgG1_G1N	14	65862987	1	1.24E-10	7.26E-11	+	0.230266	0.2546	+	T	C	0.582612854	0.0355521	+	T	C	0.6102	0.0391
rs4899178	LC_IGP53	IgG1_G1n	14	65862987	1	1.91E-11	2.40E-09	-	-0.24065	-0.2328	+	T	C	0.581700434	0.0355958	+	T	C	0.6102	0.039
rs4899178	LC_IGP15	IgG1_G1N	14	65862987	1	2.79E-08	3.52E-07	-	-0.198965	-0.1977	+	T	C	0.582612854	0.0356493	+	T	C	0.6102	0.0388
rs4899178	LC_IGP56	IgG1_G1Nn	14	65862987	1	1.67E-08	6.64E-07	-	-0.203046	-0.1927	+	T	C	0.581700434	0.035797	+	T	C	0.6102	0.0388

rs4899178	LC_IGP_R32	IgG1_G1S1/IgG1_G1	14	65862987	1	3.40E-08	5.13E-07	+	0.196202	0.1884	+	T	C	0.581251988	0.0353774	+	T	C	0.6102	0.0375
rs4899178	LC_IGP_R3	IgG1_G2F/IgG1_G2	14	65862987	1	5.75E-11	7.30E-10	+	0.232895	0.2387	+	T	C	0.581202323	0.0353172	+	T	C	0.6102	0.0388
rs72714423	LC_IGP61	IgG1 Fn total	14	65863561	1	4.99E-08	2.11E-06	-	-0.235814	-0.2436	+	A	G	0.206383209	0.0430508	+	A	G	0.1687	0.0514
rs4359350	LC_IGP78	IgG1 BG0n/G0n	14	65863748	1	3.53E-10	1.31E-06	-	-0.224892	-0.1863	+	C	T	0.565850991	0.0356245	+	C	T	0.5883	0.0385
rs4359350	LC_IGP79	IgG1 BG1n/G1n	14	65863748	1	7.16E-10	1.72E-07	-	-0.221481	-0.2015	+	C	T	0.565850991	0.0357226	+	C	T	0.5883	0.0386
rs4359350	LC_IGP77	IgG1 Bn	14	65863748	1	5.09E-10	1.45E-07	-	-0.223435	-0.2035	+	C	T	0.565850991	0.0357224	+	C	T	0.5883	0.0387
rs4359350	LC_IGP86	IgG1 Bn/Fn total	14	65863748	1	1.73E-10	8.96E-08	-	-0.229323	-0.207	+	C	T	0.565850991	0.0356912	+	C	T	0.5883	0.0387
rs4359350	LC_IGP84	IgG1 FBn/Bn total	14	65863748	1	1.40E-11	1.46E-10	+	0.243088	0.2504	+	C	T	0.565850991	0.0357091	+	C	T	0.5883	0.0391
rs4359350	LC_IGP62	IgG1 FG0n total/G0n	14	65863748	1	9.64E-13	1.03E-08	+	0.255041	0.2221	+	C	T	0.565850991	0.0354545	+	C	T	0.5883	0.0388
rs4359350	LC_IGP63	IgG1 FG1n total/G1n	14	65863748	1	5.59E-13	8.31E-10	+	0.258421	0.2381	+	C	T	0.565850991	0.0355473	+	C	T	0.5883	0.0388
rs4359350	LC_IGP64	IgG1 FG2n total/G2n	14	65863748	1	7.93E-10	7.13E-10	+	0.22101	0.2379	+	C	T	0.565850991	0.0357414	+	C	T	0.5883	0.0386
rs4359350	LC_IGP61	IgG1 Fn total	14	65863748	1	2.45E-12	9.60E-10	+	0.251046	0.2374	+	C	T	0.565850991	0.0355543	+	C	T	0.5883	0.0388
rs4359350	LC_IGP81	IgG1 Fn/Bn	14	65863748	1	4.64E-10	5.68E-07	+	0.223823	0.1927	+	C	T	0.565850991	0.0357007	+	C	T	0.5883	0.0385
rs4359350	LC_IGP21	IgG1 Fucosylation	14	65863748	1	1.92E-11	5.93E-09	+	0.239606	0.2255	+	C	T	0.564930949	0.0354431	+	C	T	0.5883	0.0388
rs4359350	LC_IGP11	IgG1_G0	14	65863748	1	6.17E-15	1.07E-10	-	-0.275805	-0.2492	+	C	T	0.564915394	0.0350241	+	C	T	0.5883	0.0386
rs4359350	LC_IGP_R1	IgG1_G0F/IgG1_G0	14	65863748	1	6.99E-13	1.50E-08	+	0.254105	0.2189	+	C	T	0.564915394	0.0351081	+	C	T	0.5883	0.0387
rs4359350	LC_IGP_R4	IgG1_G0FN/IgG1_G0N	14	65863748	1	2.96E-11	1.09E-08	+	0.236934	0.2227	+	C	T	0.565681568	0.0353906	+	C	T	0.5883	0.039
rs4359350	LC_IGP52	IgG1_G0n	14	65863748	1	2.56E-14	1.24E-10	-	-0.271823	-0.2489	+	C	T	0.565850991	0.0353506	+	C	T	0.5883	0.0387
rs4359350	LC_IGP14	IgG1_G0N	14	65863748	1	5.19E-13	1.08E-07	-	-0.256934	-0.2041	+	C	T	0.565681568	0.0352959	+	C	T	0.5883	0.0384
rs4359350	LC_IGP55	IgG1_G0Nn	14	65863748	1	1.17E-12	1.19E-07	-	-0.254622	-0.2037	+	C	T	0.565850991	0.0355321	+	C	T	0.5883	0.0385
rs4359350	LC_IGP12	IgG1_G1	14	65863748	1	4.40E-12	1.78E-09	-	-0.245605	-0.2333	+	C	T	0.565220831	0.0352083	+	C	T	0.5883	0.0388
rs4359350	LC_IGP_R2	IgG1_G1F/IgG1_G1	14	65863748	1	5.85E-13	2.91E-09	+	0.255503	0.2297	+	C	T	0.565220831	0.0351801	+	C	T	0.5883	0.0387
rs4359350	LC_IGP_R5	IgG1_G1FN/IgG1_G1N	14	65863748	1	5.65E-11	5.21E-11	+	0.234404	0.2552	+	C	T	0.566880381	0.0355309	+	C	T	0.5883	0.0389
rs4359350	LC_IGP53	IgG1_G1n	14	65863748	1	2.35E-11	3.22E-09	-	-0.239586	-0.2294	+	C	T	0.565850991	0.0356004	+	C	T	0.5883	0.0387
rs4359350	LC_IGP15	IgG1_G1N	14	65863748	1	1.56E-08	6.68E-07	-	-0.202525	-0.1917	+	C	T	0.566880381	0.0356324	+	C	T	0.5883	0.0386
rs4359350	LC_IGP56	IgG1_G1Nn	14	65863748	1	7.46E-09	1.32E-06	-	-0.207925	-0.1862	+	C	T	0.565850991	0.0357797	+	C	T	0.5883	0.0385
rs4359350	LC_IGP_R3	IgG1_G2F/IgG1_G2	14	65863748	1	1.04E-10	1.80E-09	+	0.229701	0.2316	+	C	T	0.564955073	0.0353199	+	C	T	0.5883	0.0385
rs4587890	LC_IGP78	IgG1 BG0n/G0n	14	65863784	1	1.62E-09	1.30E-06	-	-0.21636	-0.1878	+	A	T	0.581671623	0.0356589	+	A	T	0.6104	0.0388
rs4587890	LC_IGP79	IgG1 BG1n/G1n	14	65863784	1	2.19E-09	7.66E-08	-	-0.215107	-0.2084	+	A	T	0.581671623	0.0357484	+	A	T	0.6104	0.0388
rs4587890	LC_IGP77	IgG1 Bn	14	65863784	1	1.37E-09	8.91E-08	-	-0.217855	-0.2084	+	A	T	0.581671623	0.0357454	+	A	T	0.6104	0.039
rs4587890	LC_IGP86	IgG1 Bn/Fn total	14	65863784	1	4.17E-10	5.71E-08	-	-0.224498	-0.2116	+	A	T	0.581671623	0.0357119	+	A	T	0.6104	0.039
rs4587890	LC_IGP84	IgG1 FBn/Bn total	14	65863784	1	3.79E-11	2.24E-10	+	0.237912	0.2491	+	A	T	0.581671623	0.0357324	+	A	T	0.6104	0.0393
rs4587890	LC_IGP62	IgG1 FG0n total/G0n	14	65863784	1	8.16E-13	1.20E-08	+	0.255854	0.2226	+	A	T	0.581671623	0.0354526	+	A	T	0.6104	0.0391
rs4587890	LC_IGP63	IgG1 FG1n total/G1n	14	65863784	1	1.17E-12	4.89E-10	+	0.254859	0.2428	+	A	T	0.581671623	0.0355649	+	A	T	0.6104	0.039
rs4587890	LC_IGP64	IgG1 FG2n total/G2n	14	65863784	1	5.49E-10	3.17E-10	+	0.223078	0.2442	+	A	T	0.581671623	0.0357352	+	A	T	0.6104	0.0388
rs4587890	LC_IGP61	IgG1 Fn total	14	65863784	1	2.16E-12	6.68E-10	+	0.251683	0.2412	+	A	T	0.581671623	0.0355532	+	A	T	0.6104	0.0391
rs4587890	LC_IGP81	IgG1 Fn/Bn	14	65863784	1	1.08E-09	2.34E-07	+	0.219105	0.2006	+	A	T	0.581671623	0.0357206	+	A	T	0.6104	0.0388
rs4587890	LC_IGP21	IgG1 Fucosylation	14	65863784	1	1.71E-11	3.35E-09	+	0.240263	0.2307	+	A	T	0.581290074	0.0354526	+	A	T	0.6104	0.039
rs4587890	LC_IGP28	IgG1 G1S1/(G1+G1S1)	14	65863784	1	2.69E-08	4.34E-07	+	0.197641	0.1893	+	A	T	0.581223853	0.0353688	+	A	T	0.6104	0.0375
rs4587890	LC_IGP11	IgG1_G0	14	65863784	1	1.74E-15	1.76E-10	-	-0.281382	-0.2478	+	A	T	0.581124618	0.0350086	+	A	T	0.6104	0.0388
rs4587890	LC_IGP_R1	IgG1_G0F/IgG1_G0	14	65863784	1	2.49E-13	1.58E-08	+	0.259057	0.2202	+	A	T	0.581124618	0.0350976	+	A	T	0.6104	0.0389
rs4587890	LC_IGP_R4	IgG1_G0FN/IgG1_G0N	14	65863784	1	2.28E-10	1.29E-08	+	0.226221	0.2228	+	A	T	0.581930496	0.0354492	+	A	T	0.6104	0.0392
rs4587890	LC_IGP52	IgG1_G0n	14	65863784	1	9.94E-15	2.00E-10	-	-0.276075	-0.2476	+	A	T	0.581671623	0.0353319	+	A	T	0.6104	0.0389
rs4587890	LC_IGP14	IgG1_G0N	14	65863784	1	6.03E-12	1.51E-07	-	-0.245077	-0.203	+	A	T	0.581930496	0.0353631	+	A	T	0.6104	0.0387
rs4587890	LC_IGP55	IgG1_G0Nn	14	65863784	1	5.66E-12	1.60E-07	-	-0.246844	-0.2029	+	A	T	0.581671623	0.0355678	+	A	T	0.6104	0.0387
rs4587890	LC_IGP12	IgG1_G1	14	65863784	1	2.15E-12	1.25E-09	-	-0.24921	-0.237	+	A	T	0.581430055	0.035206	+	A	T	0.6104	0.039
rs4587890	LC_IGP_R2	IgG1_G1F/IgG1_G1	14	65863784	1	5.05E-13	1.26E-09	+	0.256286	0.2364	+	A	T	0.581430055	0.0351897	+	A	T	0.6104	0.0389
rs4587890	LC_IGP_R5	IgG1_G1FN/IgG1_G1N	14	65863784	1	1.23E-10	6.36E-11	+	0.230299	0.2553	+	A	T	0.582584256	0.0355537	+	A	T	0.6104	0.0391
rs4587890	LC_IGP53	IgG1_G1n	14	65863784	1	1.90E-11	2.19E-09	-	-0.240689	-0.2333	+	A	T	0.581671623	0.0355975	+	A	T	0.6104	0.039
rs4587890	LC_IGP15	IgG1_G1N	14	65863784	1	2.79E-08	3.23E-07	-	-0.198976	-0.1983	+	A	T	0.582584256	0.035651	+	A	T	0.6104	0.0388
rs4587890	LC_IGP56	IgG1_G1Nn	14	65863784	1	1.67E-08	6.15E-07	-	-0.20306	-0.1932	+	A	T	0.581671623	0.0357987	+	A	T	0.6104	0.0388
rs4587890	LC_IGP_R32	IgG1_G1S1/IgG1_G1	14	65863784	1	3.38E-08	4.88E-07	+	0.196249	0.1887	+	A	T	0.581223853	0.035379	+	A	T	0.6104	0.0375
rs4587890	LC_IGP_R3	IgG1_G2F/IgG1_G2	14	65863784	1	5.70E-11	6.65E-10	+	0.232955	0.2392	+	A	T	0.581174205	0.0353187	+	A	T	0.6104	0.0387
rs2411823	LC_IGP79	IgG1 BG1n/G1n	14	65865066	1	2.98E-08	7.29E-08	-	-0.198111	-0.208	+	T	C	0.572480173	0.0355663	+	T	C	0.6051	0.0386
rs2411823	LC_IGP77	IgG1 Bn	14	65865066	1	2.92E-08	1.12E-07	-	-0.198273	-0.206	+	T	C	0.572480173	0.035573	+	T	C	0.6051	0.0388
rs2411823	LC_IGP86	IgG1 Bn/Fn total	14	65865066	1	9.65E-09	6.86E-08	-	-0.204973	-0.2095	+	T	C	0.572480173	0.0355416	+	T	C	0.6051	0.0388
rs2411823	LC_IGP84	IgG1 FBn/Bn total	14	65865066	1	1.27E-09	1.98E-10	+	0.217223	0.2489	+	T	C	0.572480173	0.0355701	+	T	C	0.6051	0.0391
rs2411823	LC_IGP62	IgG1 FG0n total/G0n	14	65865066	1	1.56E-11	1.33E-08	+	0.239604	0.2212	+	T	C	0.572480173	0.0352797	+	T	C	0.6051	0.0389
rs2411823	LC_IGP63	IgG1 FG1n total/G1n	14	65865066	1	1.79E-11	3.29E-10	+	0.239595	0.2443	+	T	C	0.572480173	0.0353868	+	T	C	0.6051	0.0389
rs2411823	LC_IGP64	IgG1 FG2n total/G2n	14	65865066	1	5.96E-09	2.24E-10	+	0.207941	0.2453	+	T	C	0.572480173	0.0355484	+	T	C	0.6051	0.0387
rs2411823	LC_IGP61	IgG1 Fn total	14	65865066	1	3.53E-11	5.36E-10	+	0.235916	0.2416	+	T	C	0.572480173	0.0353766	+	T	C	0.6051	0.0389
rs2411823	LC_IGP81	IgG1 Fn/Bn	14	65865066	1	2.11E-08	3.12E-07	+	0.200174	0.1978	+	T	C	0.572480173	0.0355465	+	T	C	0.6051	0.0386
rs2411823	LC_IGP21	IgG1 Fucosylation	14	65865066	1	5.43E-10	2.17E-09	+	0.220369	0.2326	+	T	C	0.572119297	0.035291	+	T	C	0.6051	0.0389
rs2411823	LC_IGP28	IgG1 G1S1/(G1+G1S1)	14	65865066	1	3.58E-08	3.23E-07	+	0.194584	0.1909	+	T	C	0.572133333	0.0351411	+	T	C	0.6051	0.0374
rs2411823	LC_IGP11	IgG1_G0	14	65865066	1	2.62E-14	1.33E-10	-	-0.267699	-0.2487	+	T	C	0.572045205	0.0348343	+	T	C	0.6051	0.0387
rs2411823	LC_IGP_R1	IgG1_G0F/IgG1_G0	14	65865066	1	3.05E-12	1.73E-08	+	0.245429	0.2187	+	T	C	0.572045205	0.0349184	+	T	C	0.6051	0.0388
rs2411823	LC_IGP_R4	IgG1_G0FN/IgG1_G0N	14	65865066	1	7.30E-09	1.80E-08	+	0.205179	0.22	+	T	C	0.572842927	0.0352878	+	T	C	0.6051	0.0391
rs2411823	LC_IGP52	IgG1_G0n	14	65865066	1	1.42E-13	1.64E-10	-	-0.262193	-0.248	+	T	C	0.572480173	0.0351537	+	T	C	0.6051	0.0388
rs2411823	LC_IGP14	IgG1_G0N	14	65865066	1	2.65E-10	2.78E-07	-	-0.223855	-0.1981	+	T	C	0.572842927	0.0352092	+	T	C	0.6051	0.0386
rs2411823	LC_IGP55	IgG1_G0Nn	14	65865066	1	2.55E-10	3.11E-07	-	-0.225379	-0.1975	+	T	C	0.572480173	0.0354127	+	T	C	0.6051	0.0386
rs2411823	LC_IGP12	IgG1_G1	14	65865066	1	2.72E-11	7.28E-10	-	-0.234945	-0.2394	+	T	C	0.572350641	0.0350261	+	T	C	0.6051	0.0389
rs2411823	LC_IGP_R2	IgG1_G1F/IgG1_G1	14	65865066	1	5.45E-12	8.46E-10	+	0.243115	0.238	+	T	C	0.572350641	0.0350068	+	T	C	0.6051	0.0388
rs2411823	LC_IGP_R5	IgG1_G1FN/IgG1_G1N	14	65865066	1	2.96E-09	4.69E-11	+	0.211132	0.256	+	T	C	0.57345941	0.0353845	+	T	C	0.6051	0.0389
rs2411823	LC_IGP53	IgG1_G1n	14	65865066	1	1.96E-10	1.41E-09	-	-0.226821	-0.2351	+	T	C	0.572480173	0.0354106	+	T	C	0.6051	0.0388
rs2411823	LC_IGP_R32	IgG1_G1S1/IgG1_G1	14	65865066	1	4.62E-08	3.58E-07	+	0.193026	0.1904	+	T	C	0.572133333	0.0351517	+	T	C	0.6051	0.0374
rs2411823	LC_IGP_R3	IgG1_G2F/IgG1_G2	14	65865066	1	5.99E-10	4.72E-10	+	0.218828	0.2404	+	T	C	0.572089242	0.0351348	+	T	C	0.6051	0.0386
rs6573611	LC_IGP79	IgG1 BG1n/G1n	14	65865255	1	2.29E-08	7.40E-08	-	-0.199623	-0.208	+	T	G	0.572797088	0.0355398	+	T	G	0.6052	0.0386
rs6573611	LC_IGP77	IgG1 Bn	14	65865255	1	2.30E-08	1.16E-07	-	-0.199635	-0.2058	+	T	G	0.572797088	0.0355471	+	T	G	0.6052	0.0388
rs6573611	LC_IGP86	IgG1 Bn/Fn total	14	65865255	1	7.57E-09	7.05E-08	-	-0.206302	-0.2093	+	T	G	0.572797088	0.0355156	+	T	G	0.6052	0.0388
rs6573611	LC_IGP84	IgG1 FBn/Bn total	14	65865255	1	1.00E-09	1.98E-10	+	0.218433	0.2489	+	T	G	0.572797088	0.0355442	+	T	G	0.6052	0.0391
rs6573611	LC_IGP62	IgG1 FG0n total/G0n	14	65865255	1	1.42E-11	1.34E-08	+	0.239917	0.2212	+	T	G	0.572797088	0.0352572	+	T	G	0.6052	0.0389
rs6573611	LC_IGP63	IgG1 FG1n total/G1n	14	65865255	1	1.54E-11	3.26E-10	+	0.240209	0.2444	+	T	G	0.572797088	0.0353629	+	T	G	0.6052	0.0389
rs6573611	LC_IGP64	IgG1 FG2n total/G2n	14	65865255	1	5.47E-09	2.25E-10	+	0.208327	0.2453	+	T	G	0.572797088	0.0355258	+	T	G	0.6052	0.0387
rs6573611	LC_IGP61	IgG1 Fn total	14	65865255	1	3.15E-11	5.36E-10	+	0.236377	0.2416	+	T	G	0.572797088	0.0353534	+	T	G	0.6052	0.0389
rs6573611	LC_IGP81	IgG1 Fn/Bn	14	65865255	1	1.66E-08	3.21E-07	+	0.2015	0.1976	+	T	G	0.572797088	0.0355206	+	T	G	0.6052	0.0387
rs6573611	LC_IGP21	IgG1 Fucosylation	14	65865255	1	4.78E-10	2.17E-09	+	0.220938	0.2326	+	T	G	0.57243434	0.0352678	+	T	G	0.6052	0.0389
rs6573611	LC_IGP28	IgG1 G1S1/(G1+G1S1)	14	65865255	1	2.73E-08	3.20E-07	+	0.196127	0.191	+	T	G	0.572445872	0.0351151	+	T	G	0.6052	0.0374
rs6573611	LC_IGP11	IgG1_G0	14	65865255	1	2.49E-14	1.32E-10	-	-0.267775	-0.2488	+	T	G	0.572357361	0.0348131	+	T	G	0.6052	0.0387
rs6573611	LC_IGP_R1	IgG1_G0F/IgG1_G0	14	65865255	1	2.90E-12	1.74E-08	+	0.245543	0.2187	+	T	G	0.572357361	0.0348971	+	T	G	0.6052	0.0388
rs6573611	LC_IGP_R4	IgG1_G0FN/IgG1_G0N	14	65865255	1	6.19E-09	1.84E-08	+	0.206034	0.2199	+	T	G	0.573155542	0.0352639	+	T	G	0.6052	0.0391
rs6573611	LC_IGP52	IgG1_G0n	14	65865255	1	1.34E-13	1.63E-10	-	-0.262312	-0.248	+	T	G	0.572797088	0.0351318	+	T	G	0.6052	0.0388
rs6573611	LC_IGP14	IgG1_G0N	14	65865255	1	2.11E-10	2.90E-07	-	-0.224942	-0.1978	+	T	G	0.573155542	0.035184	+	T	G	0.6052	0.0386
rs6573611	LC_IGP55	IgG1_G0Nn	14	65865255	1	2.00E-10	3.24E-07	-	-0.226568	-0.1972	+	T	G	0.572797088	0.0353868	+	T	G	0.6052	0.0386
rs6573611	LC_IGP12	IgG1_G1	14	65865255	1	2.59E-11	7.19E-10	-	-0.235051	-0.2395	+	T	G	0.572662798	0.035005	+	T	G	0.6052	0.0389
rs6573611	LC_IGP_R2	IgG1_G1F/IgG1_G1	14	65865255	1	4.90E-12	8.40E-10	+	0.243502	0.2381	+	T	G	0.572662798	0.0349843	+	T	G	0.6052	0.0388
rs6573611	LC_IGP_R5	IgG1_G1FN/IgG1_G1N	14	65865255	1	2.27E-09	4.61E-11	+	0.212552	0.2562	+	T	G	0.573773678	0.0353584	+	T	G	0.6052	0.0389
rs6573611	LC_IGP53	IgG1_G1n	14	65865255	1	1.86E-10	1.40E-09	-	-0.226991	-0.2352	+	T	G	0.572797088	0.0353886	+	T	G	0.6052	0.0388
rs6573611	LC_IGP_R32	IgG1_G1S1/IgG1_G1	14	65865255	1	3.58E-08	3.55E-07	+	0.194498	0.1904	+	T	G	0.572445872	0.035126	+	T	G	0.6052	0.0374
rs6573611	LC_IGP_R3	IgG1_G2F/IgG1_G2	14	65865255	1	5.49E-10	4.74E-10	+	0.219173	0.2404	+	T	G	0.572401589	0.0351127	+	T	G	0.6052	0.0386
rs72714425	LC_IGP61	IgG1 Fn total	14	65877493	1	4.41E-08	2.27E-06	-	-0.239942	-0.2442	+	A	T	0.199464251	0.0436248	+	A	T	0.1658	0.0516
rs2411822	LC_IGP78	IgG1 BG0n/G0n	14	65878395	1	4.54E-13	3.65E-10	-	-0.250935	-0.2346	+	G	A	0.467507145	0.0343803	+	G	A	0.4875	0.0374
rs2411822	LC_IGP79	IgG1 BG1n/G1n	14	65878395	1	9.76E-13	2.07E-12	-	-0.247941	-0.2628	+	G	A	0.467507145	0.0344763	+	G	A	0.4875	0.0374
rs2411822	LC_IGP32	IgG1 BG1S1/(BG1+BG1S1)	14	65878395	1	2.74E-08	2.20E-12	+	0.191776	0.2574	+	G	A	0.468568762	0.0343375	+	G	A	0.4875	0.0367
rs2411822	LC_IGP77	IgG1 Bn	14	65878395	1	2.29E-12	5.15E-12	-	-0.243937	-0.2592	+	G	A	0.467507145	0.0345012	+	G	A	0.4875	0.0376
rs2411822	LC_IGP86	IgG1 Bn/Fn total	14	65878395	1	3.44E-13	2.77E-12	-	-0.25279	-0.2626	+	G	A	0.467507145	0.0344539	+	G	A	0.4875	0.0376
rs2411822	LC_IGP84	IgG1 FBn/Bn total	14	65878395	1	3.48E-14	3.18E-17	+	0.263676	0.3196	+	G	A	0.467507145	0.0344756	+	G	A	0.4875	0.0379
rs2411822	LC_IGP62	IgG1 FG0n total/G0n	14	65878395	1	8.51E-17	5.78E-13	+	0.28747	0.2714	+	G	A	0.467507145	0.0341598	+	G	A	0.4875	0.0377
rs2411822	LC_IGP63	IgG1 FG1n total/G1n	14	65878395	1	4.10E-17	2.70E-15	+	0.291236	0.2975	+	G	A	0.467507145	0.0342452	+	G	A	0.4875	0.0376
rs2411822	LC_IGP64	IgG1 FG2n total/G2n	14	65878395	1	2.38E-13	5.21E-16	+	0.254617	0.3035	+	G	A	0.467507145	0.0344628	+	G	A	0.4875	0.0374
rs2411822	LC_IGP68	IgG1 FG2n/G2n	14	65878395	1	4.76E-10	1.08E-08	+	0.216849	0.2131	+	G	A	0.467507145	0.0346111	+	G	A	0.4875	0.0373
rs2411822	LC_IGP61	IgG1 Fn total	14	65878395	1	3.38E-16	6.73E-15	+	0.282521	0.2934	+	G	A	0.467507145	0.0342654	+	G	A	0.4875	0.0377
rs2411822	LC_IGP81	IgG1 Fn/Bn	14	65878395	1	9.93E-13	3.65E-11	+	0.247774	0.2475	+	G	A	0.467507145	0.0344647	+	G	A	0.4875	0.0374
rs2411822	LC_IGP21	IgG1 Fucosylation	14	65878395	1	3.74E-14	7.40E-14	+	0.261693	0.2814	+	G	A	0.466756186	0.0342609	+	G	A	0.4875	0.0376
rs2411822	LC_IGP28	IgG1 G1S1/(G1+G1S1)	14	65878395	1	9.66E-11	1.18E-10	+	0.222778	0.233	+	G	A	0.466980143	0.0341953	+	G	A	0.4875	0.0362
rs2411822	LC_IGP26	IgG1 GS1/(G+GS1)	14	65878395	1	2.26E-08	8.94E-11	+	0.193217	0.2401	+	G	A	0.466980143	0.0343885	+	G	A	0.4875	0.037
rs2411822	LC_IGP27	IgG1 GS1/(G0+G+GS1)	14	65878395	1	9.57E-10	2.10E-09	+	0.210932	0.2204	+	G	A	0.466980143	0.0342838	+	G	A	0.4875	0.0368
rs2411822	LC_IGP11	IgG1_G0	14	65878395	1	1.11E-18	2.01E-15	-	-0.301778	-0.2976	+	G	A	0.467325921	0.0337884	+	G	A	0.4875	0.0375
rs2411822	LC_IGP_R1	IgG1_G0F/IgG1_G0	14	65878395	1	4.68E-16	1.66E-12	+	0.278069	0.2651	+	G	A	0.467325921	0.0338968	+	G	A	0.4875	0.0376
rs2411822	LC_IGP_R4	IgG1_G0FN/IgG1_G0N	14	65878395	1	1.35E-13	5.90E-14	+	0.255205	0.284	+	G	A	0.467864686	0.0341893	+	G	A	0.4875	0.0378

rs2411822	LC_IGP52	IgG1_G0n	14	65878395	1	9.83E-19	2.17E-15	-	-0.304557	-0.2978	+	G	A	0.467507145	0.034042	+	G	A	0.4875	0.0375
rs2411822	LC_IGP14	IgG1_G0N	14	65878395	1	7.92E-16	8.51E-12	-	-0.277286	-0.2549	+	G	A	0.467864686	0.0340748	+	G	A	0.4875	0.0373
rs2411822	LC_IGP55	IgG1_G0Nn	14	65878395	1	3.73E-16	1.03E-11	-	-0.282072	-0.2542	+	G	A	0.467507145	0.0342614	+	G	A	0.4875	0.0374
rs2411822	LC_IGP12	IgG1_G1	14	65878395	1	1.52E-15	1.66E-14	-	-0.273836	-0.2888	+	G	A	0.467325921	0.0339962	+	G	A	0.4875	0.0376
rs2411822	LC_IGP_R2	IgG1_G1F/IgG1_G1	14	65878395	1	6.11E-17	2.69E-14	+	0.286986	0.2859	+	G	A	0.467325921	0.0339442	+	G	A	0.4875	0.0376
rs2411822	LC_IGP_R5	IgG1_G1FN/IgG1_G1N	14	65878395	1	1.24E-13	2.03E-18	+	0.256608	0.3298	+	G	A	0.468646083	0.0343226	+	G	A	0.4875	0.0377
rs2411822	LC_IGP53	IgG1_G1n	14	65878395	1	9.61E-15	3.74E-14	-	-0.268418	-0.2847	+	G	A	0.467507145	0.0343328	+	G	A	0.4875	0.0376
rs2411822	LC_IGP15	IgG1_G1N	14	65878395	1	6.28E-11	2.59E-11	-	-0.226613	-0.2495	+	G	A	0.468646083	0.0344339	+	G	A	0.4875	0.0374
rs2411822	LC_IGP56	IgG1_G1Nn	14	65878395	1	2.60E-11	6.75E-11	-	-0.232013	-0.2438	+	G	A	0.467507145	0.0345515	+	G	A	0.4875	0.0374
rs2411822	LC_IGP_R36	IgG1_G1NS1/IgG1_G1N	14	65878395	1	2.26E-08	2.52E-12	+	0.192968	0.257	+	G	A	0.468568762	0.0343416	+	G	A	0.4875	0.0367
rs2411822	LC_IGP_R32	IgG1_G1S1/IgG1_G1	14	65878395	1	1.80E-10	1.72E-10	+	0.219583	0.2312	+	G	A	0.466980143	0.0342132	+	G	A	0.4875	0.0362
rs2411822	LC_IGP_R3	IgG1_G2F/IgG1_G2	14	65878395	1	7.21E-15	1.88E-15	+	0.267671	0.297	+	G	A	0.467000326	0.0340779	+	G	A	0.4875	0.0374
rs2411822	LC_IGP_R8	IgG1_G2FS1/IgG1_G2S1	14	65878395	1	2.38E-09	8.16E-14	+	0.206092	0.2784	+	G	A	0.466715048	0.0343303	+	G	A	0.4875	0.0373
rs2411822	LC_IGP_R26	IgG1_G2N/IgG1_G1N	14	65878395	1	4.92E-12	3.19E-09	+	0.238746	0.2164	+	G	A	0.468244773	0.0343011	+	G	A	0.4875	0.0365
rs2411822	LC_IGP148	IgG2 FG0n total/G0n	14	65878395	1	2.79E-08	1.58E-14	+	0.192363	0.2862	+	G	A	0.465862398	0.0344639	+	G	A	0.4874	0.0373
rs2411822	LC_IGP97	IgG2_G0	14	65878395	1	5.03E-10	7.15E-15	-	-0.214082	-0.2904	+	G	A	0.467000326	0.0342191	+	G	A	0.4875	0.0373
rs2411822	LC_IGP138	IgG2_G0n	14	65878395	1	3.54E-10	5.86E-15	-	-0.216781	-0.2918	+	G	A	0.465862398	0.0343416	+	G	A	0.4874	0.0374
rs2411822	LC_IGP_R64	IgG2_G2/IgG2_G1	14	65878395	1	4.23E-09	1.67E-13	+	0.203622	0.2698	+	G	A	0.466041942	0.034472	+	G	A	0.4875	0.0366
rs1953416	LC_IGP78	IgG1 BG0n/G0n	14	65878807	1	1.93E-13	1.01E-09	-	-0.255522	-0.2284	+	T	C	0.462582404	0.0344502	+	T	C	0.4906	0.0374
rs1953416	LC_IGP79	IgG1 BG1n/G1n	14	65878807	1	4.89E-13	7.83E-12	-	-0.251819	-0.2557	+	T	C	0.462582404	0.0345499	+	T	C	0.4906	0.0374
rs1953416	LC_IGP32	IgG1 BG1S1/(BG1+BG1S1)	14	65878807	1	2.60E-08	8.26E-12	+	0.192552	0.2506	+	T	C	0.463668311	0.0344222	+	T	C	0.4906	0.0367
rs1953416	LC_IGP77	IgG1 Bn	14	65878807	1	1.10E-12	1.88E-11	-	-0.248039	-0.2521	+	T	C	0.462582404	0.034574	+	T	C	0.4906	0.0376
rs1953416	LC_IGP86	IgG1 Bn/Fn total	14	65878807	1	1.68E-13	9.94E-12	-	-0.256729	-0.2557	+	T	C	0.462582404	0.0345269	+	T	C	0.4906	0.0376
rs1953416	LC_IGP84	IgG1 FBn/Bn total	14	65878807	1	2.31E-14	1.12E-16	+	0.266169	0.3137	+	T	C	0.462582404	0.034555	+	T	C	0.4906	0.0378
rs1953416	LC_IGP62	IgG1 FG0n total/G0n	14	65878807	1	4.23E-17	1.46E-12	+	0.290998	0.2664	+	T	C	0.462582404	0.0342324	+	T	C	0.4906	0.0376
rs1953416	LC_IGP63	IgG1 FG1n total/G1n	14	65878807	1	3.11E-17	9.27E-15	+	0.293073	0.2915	+	T	C	0.462582404	0.0343269	+	T	C	0.4906	0.0376
rs1953416	LC_IGP64	IgG1 FG2n total/G2n	14	65878807	1	2.55E-13	1.85E-15	+	0.254942	0.2975	+	T	C	0.462582404	0.0345523	+	T	C	0.4906	0.0374
rs1953416	LC_IGP68	IgG1 FG2n/G2n	14	65878807	1	2.96E-10	2.66E-08	+	0.219964	0.2073	+	T	C	0.462582404	0.0346896	+	T	C	0.4906	0.0373
rs1953416	LC_IGP61	IgG1 Fn total	14	65878807	1	2.51E-16	2.10E-14	+	0.284463	0.2878	+	T	C	0.462582404	0.0343467	+	T	C	0.4906	0.0377
rs1953416	LC_IGP81	IgG1 Fn/Bn	14	65878807	1	4.56E-13	1.20E-10	+	0.252056	0.2407	+	T	C	0.462582404	0.0345363	+	T	C	0.4906	0.0374
rs1953416	LC_IGP21	IgG1 Fucosylation	14	65878807	1	2.77E-14	2.73E-13	+	0.263693	0.2747	+	T	C	0.461855425	0.0343427	+	T	C	0.4906	0.0376
rs1953416	LC_IGP28	IgG1 G1S1/(G1+G1S1)	14	65878807	1	5.86E-11	1.77E-10	+	0.2259	0.2306	+	T	C	0.462118654	0.0342718	+	T	C	0.4906	0.0362
rs1953416	LC_IGP26	IgG1 GS1/(G+GS1)	14	65878807	1	2.05E-08	1.63E-10	+	0.194282	0.2367	+	T	C	0.462118654	0.0344738	+	T	C	0.4906	0.037
rs1953416	LC_IGP27	IgG1 GS1/(G0+G+GS1)	14	65878807	1	6.90E-10	4.28E-09	+	0.213242	0.2161	+	T	C	0.462118654	0.034364	+	T	C	0.4906	0.0368
rs1953416	LC_IGP11	IgG1_G0	14	65878807	1	2.63E-19	7.32E-15	-	-0.308082	-0.2914	+	T	C	0.462164936	0.0338676	+	T	C	0.4906	0.0375
rs1953416	LC_IGP_R1	IgG1_G0F/IgG1_G0	14	65878807	1	9.54E-17	3.96E-12	+	0.285381	0.2603	+	T	C	0.462164936	0.0339731	+	T	C	0.4906	0.0375
rs1953416	LC_IGP_R4	IgG1_G0FN/IgG1_G0N	14	65878807	1	6.29E-14	1.57E-13	+	0.259461	0.2789	+	T	C	0.462691059	0.0342831	+	T	C	0.4906	0.0378
rs1953416	LC_IGP52	IgG1_G0n	14	65878807	1	4.52E-19	7.97E-15	-	-0.30825	-0.2915	+	T	C	0.462582404	0.0341127	+	T	C	0.4906	0.0375
rs1953416	LC_IGP14	IgG1_G0N	14	65878807	1	1.46E-16	3.72E-11	-	-0.285085	-0.2468	+	T	C	0.462691059	0.0341492	+	T	C	0.4906	0.0373
rs1953416	LC_IGP55	IgG1_G0Nn	14	65878807	1	1.47E-16	4.55E-11	-	-0.286598	-0.2459	+	T	C	0.462582404	0.0343294	+	T	C	0.4906	0.0373
rs1953416	LC_IGP12	IgG1_G1	14	65878807	1	5.50E-16	4.50E-14	-	-0.278915	-0.2838	+	T	C	0.462164936	0.0340846	+	T	C	0.4906	0.0376
rs1953416	LC_IGP_R2	IgG1_G1F/IgG1_G1	14	65878807	1	1.80E-17	8.16E-14	+	0.292712	0.2803	+	T	C	0.462164936	0.0340283	+	T	C	0.4906	0.0375
rs1953416	LC_IGP_R5	IgG1_G1FN/IgG1_G1N	14	65878807	1	9.00E-14	8.88E-18	+	0.258703	0.3232	+	T	C	0.463757688	0.0344012	+	T	C	0.4906	0.0376
rs1953416	LC_IGP53	IgG1_G1n	14	65878807	1	8.19E-15	9.85E-14	-	-0.269798	-0.2797	+	T	C	0.462582404	0.0344172	+	T	C	0.4906	0.0376
rs1953416	LC_IGP15	IgG1_G1N	14	65878807	1	3.39E-11	8.21E-11	-	-0.230325	-0.243	+	T	C	0.463757688	0.0345067	+	T	C	0.4906	0.0374
rs1953416	LC_IGP56	IgG1_G1Nn	14	65878807	1	1.33E-11	2.09E-10	-	-0.23598	-0.2373	+	T	C	0.462582404	0.0346257	+	T	C	0.4906	0.0374
rs1953416	LC_IGP_R36	IgG1_G1NS1/IgG1_G1N	14	65878807	1	2.08E-08	9.18E-12	+	0.19394	0.2503	+	T	C	0.463668311	0.0344256	+	T	C	0.4906	0.0367
rs1953416	LC_IGP_R32	IgG1_G1S1/IgG1_G1	14	65878807	1	1.07E-10	2.65E-10	+	0.222852	0.2287	+	T	C	0.462118654	0.0342893	+	T	C	0.4906	0.0362
rs1953416	LC_IGP_R3	IgG1_G2F/IgG1_G2	14	65878807	1	8.61E-15	6.13E-15	+	0.267586	0.2914	+	T	C	0.462141809	0.034168	+	T	C	0.4906	0.0374
rs1953416	LC_IGP_R8	IgG1_G2FS1/IgG1_G2S1	14	65878807	1	2.85E-09	2.25E-13	+	0.205609	0.2732	+	T	C	0.461859499	0.0344214	+	T	C	0.4906	0.0373
rs1953416	LC_IGP_R26	IgG1_G2N/IgG1_G1N	14	65878807	1	2.95E-12	1.28E-08	+	0.241806	0.2078	+	T	C	0.463338272	0.0343766	+	T	C	0.4906	0.0365
rs1953416	LC_IGP97	IgG2_G0	14	65878807	1	1.28E-09	2.12E-14	-	-0.209573	-0.2853	+	T	C	0.462141809	0.0343249	+	T	C	0.4906	0.0373
rs1953416	LC_IGP138	IgG2_G0n	14	65878807	1	9.60E-10	1.80E-14	-	-0.211955	-0.2864	+	T	C	0.460967672	0.0344517	+	T	C	0.4905	0.0374
rs1953416	LC_IGP_R64	IgG2_G2/IgG2_G1	14	65878807	1	5.80E-09	5.20E-13	+	0.202348	0.2643	+	T	C	0.461177479	0.0345673	+	T	C	0.4906	0.0366
rs883081	LC_IGP78	IgG1 BG0n/G0n	14	65880621	1	4.28E-15	2.78E-10	-	-0.272332	-0.2361	+	A	C	0.471775713	0.0343714	+	A	C	0.4927	0.0374
rs883081	LC_IGP79	IgG1 BG1n/G1n	14	65880621	1	2.62E-14	3.22E-12	-	-0.265087	-0.2606	+	A	C	0.471775713	0.0344894	+	A	C	0.4927	0.0374
rs883081	LC_IGP32	IgG1 BG1S1/(BG1+BG1S1)	14	65880621	1	4.40E-09	1.09E-12	+	0.202918	0.2605	+	A	C	0.472816893	0.0343892	+	A	C	0.4927	0.0366
rs883081	LC_IGP31	IgG1 BGS1/(BG0+BG+BGS1)	14	65880621	1	6.22E-09	1.92E-12	+	0.201678	0.2604	+	A	C	0.47284788	0.0345197	+	A	C	0.4927	0.037
rs883081	LC_IGP77	IgG1 Bn	14	65880621	1	3.66E-14	6.59E-12	-	-0.263664	-0.258	+	A	C	0.471775713	0.0345033	+	A	C	0.4927	0.0376
rs883081	LC_IGP86	IgG1 Bn/Fn total	14	65880621	1	5.04E-15	3.72E-12	-	-0.272258	-0.2612	+	A	C	0.471775713	0.0344541	+	A	C	0.4927	0.0376
rs883081	LC_IGP84	IgG1 FBn/Bn total	14	65880621	1	4.60E-16	4.08E-17	+	0.282919	0.3185	+	A	C	0.471775713	0.0344735	+	A	C	0.4927	0.0379
rs883081	LC_IGP62	IgG1 FG0n total/G0n	14	65880621	1	1.81E-18	5.96E-13	+	0.303266	0.2713	+	A	C	0.471775713	0.0341675	+	A	C	0.4927	0.0377
rs883081	LC_IGP63	IgG1 FG1n total/G1n	14	65880621	1	1.66E-18	3.98E-15	+	0.304471	0.2957	+	A	C	0.471775713	0.0342665	+	A	C	0.4927	0.0376
rs883081	LC_IGP64	IgG1 FG2n total/G2n	14	65880621	1	1.90E-14	7.24E-16	+	0.266638	0.302	+	A	C	0.471775713	0.0344987	+	A	C	0.4927	0.0374
rs883081	LC_IGP68	IgG1 FG2n/G2n	14	65880621	1	3.33E-11	1.20E-08	+	0.231347	0.2124	+	A	C	0.471775713	0.0346448	+	A	C	0.4927	0.0373
rs883081	LC_IGP61	IgG1 Fn total	14	65880621	1	1.30E-17	8.74E-15	+	0.296319	0.2923	+	A	C	0.471775713	0.0342856	+	A	C	0.4927	0.0377
rs883081	LC_IGP81	IgG1 Fn/Bn	14	65880621	1	1.65E-14	4.22E-11	+	0.267035	0.2468	+	A	C	0.471775713	0.0344676	+	A	C	0.4927	0.0374
rs883081	LC_IGP21	IgG1 Fucosylation	14	65880621	1	6.40E-16	1.33E-13	+	0.279768	0.2785	+	A	C	0.471012022	0.0342644	+	A	C	0.4927	0.0376
rs883081	LC_IGP28	IgG1 G1S1/(G1+G1S1)	14	65880621	1	5.25E-11	1.10E-10	+	0.226462	0.2329	+	A	C	0.471202752	0.0342703	+	A	C	0.4927	0.0361
rs883081	LC_IGP26	IgG1 GS1/(G+GS1)	14	65880621	1	2.16E-08	2.10E-10	+	0.193965	0.2348	+	A	C	0.471202752	0.0344756	+	A	C	0.4927	0.037
rs883081	LC_IGP27	IgG1 GS1/(G0+G+GS1)	14	65880621	1	7.82E-10	3.87E-09	+	0.212572	0.2162	+	A	C	0.471202752	0.0343673	+	A	C	0.4927	0.0367
rs883081	LC_IGP11	IgG1_G0	14	65880621	1	1.82E-20	2.65E-15	-	-0.317747	-0.2961	+	A	C	0.471237935	0.0338138	+	A	C	0.4927	0.0374
rs883081	LC_IGP_R1	IgG1_G0F/IgG1_G0	14	65880621	1	7.93E-18	1.65E-12	+	0.295135	0.2652	+	A	C	0.471237935	0.033923	+	A	C	0.4927	0.0376
rs883081	LC_IGP_R4	IgG1_G0FN/IgG1_G0N	14	65880621	1	1.08E-15	5.04E-14	+	0.276955	0.2846	+	A	C	0.471778016	0.0342002	+	A	C	0.4927	0.0378
rs883081	LC_IGP52	IgG1_G0n	14	65880621	1	3.09E-20	2.62E-15	-	-0.318094	-0.2967	+	A	C	0.471775713	0.0340578	+	A	C	0.4927	0.0375
rs883081	LC_IGP14	IgG1_G0N	14	65880621	1	1.80E-18	7.18E-12	-	-0.302272	-0.2556	+	A	C	0.471778016	0.0340596	+	A	C	0.4927	0.0373
rs883081	LC_IGP55	IgG1_G0Nn	14	65880621	1	1.79E-18	8.14E-12	-	-0.303935	-0.2552	+	A	C	0.471775713	0.0342379	+	A	C	0.4927	0.0373
rs883081	LC_IGP12	IgG1_G1	14	65880621	1	3.91E-17	2.64E-14	-	-0.289566	-0.2866	+	A	C	0.471237935	0.0340311	+	A	C	0.4927	0.0376
rs883081	LC_IGP_R2	IgG1_G1F/IgG1_G1	14	65880621	1	1.55E-18	3.84E-14	+	0.302146	0.2842	+	A	C	0.471237935	0.0339787	+	A	C	0.4927	0.0376
rs883081	LC_IGP_R5	IgG1_G1FN/IgG1_G1N	14	65880621	1	2.61E-15	2.61E-18	+	0.274187	0.3288	+	A	C	0.472883764	0.0343313	+	A	C	0.4927	0.0377
rs883081	LC_IGP53	IgG1_G1n	14	65880621	1	7.25E-16	5.36E-14	-	-0.280079	-0.2829	+	A	C	0.471775713	0.0343673	+	A	C	0.4927	0.0376
rs883081	LC_IGP15	IgG1_G1N	14	65880621	1	2.05E-12	4.33E-11	-	-0.244118	-0.2467	+	A	C	0.472883764	0.0344523	+	A	C	0.4927	0.0374
rs883081	LC_IGP56	IgG1_G1Nn	14	65880621	1	8.25E-13	1.01E-10	-	-0.249422	-0.2416	+	A	C	0.471775713	0.0345683	+	A	C	0.4927	0.0374
rs883081	LC_IGP_R36	IgG1_G1NS1/IgG1_G1N	14	65880621	1	3.66E-09	1.28E-12	+	0.204007	0.26	+	A	C	0.472816893	0.0343934	+	A	C	0.4927	0.0366
rs883081	LC_IGP_R32	IgG1_G1S1/IgG1_G1	14	65880621	1	9.24E-11	1.72E-10	+	0.22361	0.2307	+	A	C	0.471202752	0.034287	+	A	C	0.4927	0.0361
rs883081	LC_IGP_R3	IgG1_G2F/IgG1_G2	14	65880621	1	6.69E-16	2.49E-15	+	0.278342	0.2957	+	A	C	0.471220355	0.0341162	+	A	C	0.4927	0.0374
rs883081	LC_IGP_R8	IgG1_G2FS1/IgG1_G2S1	14	65880621	1	2.61E-10	2.35E-13	+	0.218622	0.2732	+	A	C	0.470932498	0.0343724	+	A	C	0.4927	0.0373
rs883081	LC_IGP_R26	IgG1_G2N/IgG1_G1N	14	65880621	1	4.61E-13	3.51E-09	+	0.250565	0.2155	+	A	C	0.472497531	0.0343409	+	A	C	0.4927	0.0365
rs883081	LC_IGP148	IgG2 FG0n total/G0n	14	65880621	1	1.27E-08	2.22E-14	+	0.197537	0.2843	+	A	C	0.4701133	0.0345353	+	A	C	0.4927	0.0372
rs883081	LC_IGP97	IgG2_G0	14	65880621	1	3.04E-10	1.23E-14	-	-0.217308	-0.2875	+	A	C	0.471220355	0.0342963	+	A	C	0.4927	0.0373
rs883081	LC_IGP138	IgG2_G0n	14	65880621	1	1.98E-10	9.21E-15	-	-0.220389	-0.2893	+	A	C	0.4701133	0.034417	+	A	C	0.4927	0.0373
rs883081	LC_IGP_R64	IgG2_G2/IgG2_G1	14	65880621	1	6.15E-09	1.96E-13	+	0.202004	0.2688	+	A	C	0.470267136	0.034567	+	A	C	0.4927	0.0366
rs883082	LC_IGP78	IgG1 BG0n/G0n	14	65880940	1	4.28E-15	3.31E-10	-	-0.272326	-0.2353	+	G	A	0.471777881	0.0343711	+	G	A	0.4932	0.0374
rs883082	LC_IGP79	IgG1 BG1n/G1n	14	65880940	1	2.62E-14	3.68E-12	-	-0.265077	-0.2601	+	G	A	0.471777881	0.0344891	+	G	A	0.4932	0.0374
rs883082	LC_IGP32	IgG1 BG1S1/(BG1+BG1S1)	14	65880940	1	4.40E-09	1.46E-12	+	0.202912	0.2593	+	G	A	0.472819051	0.0343889	+	G	A	0.4932	0.0366
rs883082	LC_IGP31	IgG1 BGS1/(BG0+BG+BGS1)	14	65880940	1	6.22E-09	2.49E-12	+	0.201674	0.2593	+	G	A	0.472850062	0.0345194	+	G	A	0.4932	0.037
rs883082	LC_IGP77	IgG1 Bn	14	65880940	1	3.66E-14	7.57E-12	-	-0.263656	-0.2575	+	G	A	0.471777881	0.0345031	+	G	A	0.4932	0.0376
rs883082	LC_IGP86	IgG1 Bn/Fn total	14	65880940	1	5.05E-15	4.32E-12	-	-0.27225	-0.2606	+	G	A	0.471777881	0.0344539	+	G	A	0.4932	0.0376
rs883082	LC_IGP84	IgG1 FBn/Bn total	14	65880940	1	4.60E-16	5.45E-17	+	0.282914	0.3175	+	G	A	0.471777881	0.0344733	+	G	A	0.4932	0.0379
rs883082	LC_IGP62	IgG1 FG0n total/G0n	14	65880940	1	1.81E-18	7.64E-13	+	0.303261	0.2702	+	G	A	0.471777881	0.0341672	+	G	A	0.4932	0.0377
rs883082	LC_IGP63	IgG1 FG1n total/G1n	14	65880940	1	1.67E-18	5.16E-15	+	0.304463	0.2947	+	G	A	0.471777881	0.0342662	+	G	A	0.4932	0.0377
rs883082	LC_IGP64	IgG1 FG2n total/G2n	14	65880940	1	1.90E-14	9.49E-16	+	0.266633	0.301	+	G	A	0.471777881	0.0344984	+	G	A	0.4932	0.0375
rs883082	LC_IGP68	IgG1 FG2n/G2n	14	65880940	1	3.33E-11	1.36E-08	+	0.231341	0.2118	+	G	A	0.471777881	0.0346445	+	G	A	0.4932	0.0373
rs883082	LC_IGP61	IgG1 Fn total	14	65880940	1	1.30E-17	1.15E-14	+	0.296312	0.2912	+	G	A	0.471777881	0.0342853	+	G	A	0.4932	0.0377
rs883082	LC_IGP81	IgG1 Fn/Bn	14	65880940	1	1.65E-14	4.82E-11	+	0.267026	0.2463	+	G	A	0.471777881	0.0344673	+	G	A	0.4932	0.0374
rs883082	LC_IGP21	IgG1 Fucosylation	14	65880940	1	6.40E-16	1.68E-13	+	0.279764	0.2776	+	G	A	0.471013872	0.0342642	+	G	A	0.4932	0.0377
rs883082	LC_IGP28	IgG1 G1S1/(G1+G1S1)	14	65880940	1	5.26E-11	1.67E-10	+	0.226449	0.2308	+	G	A	0.471204893	0.03427	+	G	A	0.4932	0.0361
rs883082	LC_IGP26	IgG1 GS1/(G+GS1)	14	65880940	1	2.17E-08	2.67E-10	+	0.193957	0.2337	+	G	A	0.471204893	0.0344754	+	G	A	0.4932	0.037
rs883082	LC_IGP27	IgG1 GS1/(G0+G+GS1)	14	65880940	1	7.82E-10	4.41E-09	+	0.212566	0.2156	+	G	A	0.471204893	0.0343671	+	G	A	0.4932	0.0367
rs883082	LC_IGP11	IgG1_G0	14	65880940	1	1.82E-20	3.38E-15	-	-0.317746	-0.2952	+	G	A	0.471240073	0.0338135	+	G	A	0.4932	0.0375
rs883082	LC_IGP_R1	IgG1_G0F/IgG1_G0	14	65880940	1	7.93E-18	2.10E-12	+	0.295131	0.2642	+	G	A	0.471240073	0.0339227	+	G	A	0.4932	0.0376
rs883082	LC_IGP_R4	IgG1_G0FN/IgG1_G0N	14	65880940	1	1.08E-15	6.65E-14	+	0.276951	0.2835	+	G	A	0.471780159	0.0342	+	G	A	0.4932	0.0378
rs883082	LC_IGP52	IgG1_G0n	14	65880940	1	3.09E-20	3.34E-15	-	-0.318093	-0.2958	+	G	A	0.471777881	0.0340575	+	G	A	0.4932	0.0376
rs883082	LC_IGP14	IgG1_G0N	14	65880940	1	1.80E-18	8.23E-12	-	-0.30227	-0.2551	+	G	A	0.471780159	0.0340593	+	G	A	0.4932	0.0373
rs883082	LC_IGP55	IgG1_G0Nn	14	65880940	1	1.79E-18	9.32E-12	-	-0.303932	-0.2547	+	G	A	0.471777881	0.0342376	+	G	A	0.4932	0.0374
rs883082	LC_IGP12	IgG1_G1	14	65880940	1	3.91E-17	3.53E-14	-	-0.28956	-0.2854	+	G	A	0.471240073	0.0340308	+	G	A	0.4932	0.0377
rs883082	LC_IGP_R2	IgG1_G1F/IgG1_G1	14	65880940	1	1.55E-18	4.98E-14	+	0.302138	0.2831	+	G	A	0.471240073	0.0339785	+	G	A	0.4932	0.0376
rs883082	LC_IGP_R5	IgG1_G1FN/IgG1_G1N	14	65880940	1	2.61E-15	3.50E-18	+	0.274182	0.3278	+	G	A	0.472885916	0.034331	+	G	A	0.4932	0.0377
rs883082	LC_IGP53	IgG1_G1n	14	65880940	1	7.26E-16	7.10E-14	-	-0.280073	-0.2818	+	G	A	0.471777881	0.0343671	+	G	A	0.4932	0.0376

rs883082	LC_IGP15	IgG1_G1N	14	65880940	1	2.06E-12	4.97E-11	-	-0.24411	-0.2462	+	G	A	0.472885916	0.034452	+	G	A	0.4932	0.0375
rs883082	LC_IGP56	IgG1_G1Nn	14	65880940	1	8.26E-13	1.16E-10	-	-0.249413	-0.2411	+	G	A	0.471777881	0.034568	+	G	A	0.4932	0.0374
rs883082	LC_IGP_R36	IgG1_G1NS1/IgG1_G1N	14	65880940	1	3.66E-09	1.70E-12	+	0.204001	0.2588	+	G	A	0.472819051	0.0343932	+	G	A	0.4932	0.0367
rs883082	LC_IGP_R32	IgG1_G1S1/IgG1_G1	14	65880940	1	9.26E-11	2.53E-10	+	0.223597	0.2288	+	G	A	0.471204893	0.0342868	+	G	A	0.4932	0.0362
rs883082	LC_IGP_R3	IgG1_G2F/IgG1_G2	14	65880940	1	6.69E-16	3.30E-15	+	0.278337	0.2946	+	G	A	0.471222494	0.0341159	+	G	A	0.4932	0.0374
rs883082	LC_IGP_R8	IgG1_G2FS1/IgG1_G2S1	14	65880940	1	2.60E-10	3.01E-13	+	0.218621	0.2721	+	G	A	0.470934637	0.0343721	+	G	A	0.4932	0.0373
rs883082	LC_IGP_R26	IgG1_G2N/IgG1_G1N	14	65880940	1	4.61E-13	3.90E-09	+	0.250563	0.2151	+	G	A	0.472499691	0.0343406	+	G	A	0.4932	0.0365
rs883082	LC_IGP148	IgG2 FG0n total/G0n	14	65880940	1	1.27E-08	2.50E-14	+	0.197536	0.284	+	G	A	0.470115148	0.034535	+	G	A	0.4931	0.0373
rs883082	LC_IGP97	IgG2_G0	14	65880940	1	3.04E-10	1.31E-14	-	-0.217308	-0.2874	+	G	A	0.471222494	0.034296	+	G	A	0.4932	0.0373
rs883082	LC_IGP138	IgG2_G0n	14	65880940	1	1.98E-10	9.95E-15	-	-0.220391	-0.2892	+	G	A	0.470115148	0.0344167	+	G	A	0.4931	0.0374
rs883082	LC_IGP_R64	IgG2_G2/IgG2_G1	14	65880940	1	6.15E-09	2.00E-13	+	0.202003	0.2689	+	G	A	0.470269278	0.0345667	+	G	A	0.4932	0.0366
rs11628196	LC_IGP78	IgG1 BG0n/G0n	14	65891241	1	1.40E-14	3.13E-10	-	-0.266665	-0.2355	+	G	A	0.478319393	0.0343258	+	G	A	0.493	0.0374
rs11628196	LC_IGP79	IgG1 BG1n/G1n	14	65891241	1	6.12E-14	3.83E-12	-	-0.26078	-0.2596	+	G	A	0.478319393	0.0344366	+	G	A	0.493	0.0374
rs11628196	LC_IGP32	IgG1 BG1S1/(BG1+BG1S1)	14	65891241	1	3.50E-09	1.82E-12	+	0.203812	0.2579	+	G	A	0.479330456	0.0343162	+	G	A	0.493	0.0366
rs11628196	LC_IGP31	IgG1 BGS1/(BG0+BG+BGS1)	14	65891241	1	1.74E-08	3.18E-12	+	0.195284	0.2579	+	G	A	0.47943298	0.034472	+	G	A	0.493	0.037
rs11628196	LC_IGP77	IgG1 Bn	14	65891241	1	9.83E-14	8.06E-12	-	-0.258701	-0.2569	+	G	A	0.478319393	0.0344534	+	G	A	0.493	0.0376
rs11628196	LC_IGP86	IgG1 Bn/Fn total	14	65891241	1	1.36E-14	4.65E-12	-	-0.267414	-0.26	+	G	A	0.478319393	0.0344044	+	G	A	0.493	0.0376
rs11628196	LC_IGP84	IgG1 FBn/Bn total	14	65891241	1	9.71E-16	7.23E-17	+	0.279228	0.3159	+	G	A	0.478319393	0.0344187	+	G	A	0.493	0.0379
rs11628196	LC_IGP62	IgG1 FG0n total/G0n	14	65891241	1	5.94E-18	8.30E-13	+	0.298091	0.2696	+	G	A	0.478319393	0.0341224	+	G	A	0.493	0.0377
rs11628196	LC_IGP63	IgG1 FG1n total/G1n	14	65891241	1	3.14E-18	6.12E-15	+	0.301418	0.2937	+	G	A	0.478319393	0.0342096	+	G	A	0.493	0.0376
rs11628196	LC_IGP64	IgG1 FG2n total/G2n	14	65891241	1	2.42E-14	1.14E-15	+	0.265021	0.3	+	G	A	0.478319393	0.0344331	+	G	A	0.493	0.0374
rs11628196	LC_IGP68	IgG1 FG2n/G2n	14	65891241	1	6.71E-11	1.26E-08	+	0.227293	0.2122	+	G	A	0.478319393	0.0345885	+	G	A	0.493	0.0373
rs11628196	LC_IGP61	IgG1 Fn total	14	65891241	1	2.71E-17	1.33E-14	+	0.29283	0.2903	+	G	A	0.478319393	0.0342308	+	G	A	0.493	0.0377
rs11628196	LC_IGP81	IgG1 Fn/Bn	14	65891241	1	4.64E-14	4.88E-11	+	0.261921	0.246	+	G	A	0.478319393	0.0344188	+	G	A	0.493	0.0374
rs11628196	LC_IGP21	IgG1 Fucosylation	14	65891241	1	1.31E-15	1.90E-13	+	0.276202	0.2768	+	G	A	0.477523736	0.0342083	+	G	A	0.493	0.0376
rs11628196	LC_IGP28	IgG1 G1S1/(G1+G1S1)	14	65891241	1	7.14E-11	1.70E-10	+	0.224437	0.2305	+	G	A	0.477664526	0.0342061	+	G	A	0.493	0.0361
rs11628196	LC_IGP26	IgG1 GS1/(G+GS1)	14	65891241	1	1.95E-08	4.25E-10	+	0.194197	0.2309	+	G	A	0.477664526	0.0344026	+	G	A	0.493	0.037
rs11628196	LC_IGP27	IgG1 GS1/(G0+G+GS1)	14	65891241	1	8.98E-10	7.34E-09	+	0.211385	0.2124	+	G	A	0.477664526	0.0342995	+	G	A	0.493	0.0367
rs11628196	LC_IGP11	IgG1_G0	14	65891241	1	1.07E-19	5.47E-15	-	-0.310536	-0.2928	+	G	A	0.477997251	0.0337585	+	G	A	0.493	0.0375
rs11628196	LC_IGP_R1	IgG1_G0F/IgG1_G0	14	65891241	1	6.36E-17	2.17E-12	+	0.286225	0.2638	+	G	A	0.477997251	0.0338738	+	G	A	0.493	0.0376
rs11628196	LC_IGP_R4	IgG1_G0FN/IgG1_G0N	14	65891241	1	3.05E-15	7.68E-14	+	0.271876	0.2826	+	G	A	0.478553889	0.0341298	+	G	A	0.493	0.0378
rs11628196	LC_IGP52	IgG1_G0n	14	65891241	1	9.59E-20	5.16E-15	-	-0.313344	-0.2936	+	G	A	0.478319393	0.0340116	+	G	A	0.493	0.0375
rs11628196	LC_IGP14	IgG1_G0N	14	65891241	1	1.24E-17	1.03E-11	-	-0.294083	-0.2538	+	G	A	0.478553889	0.0340079	+	G	A	0.493	0.0373
rs11628196	LC_IGP55	IgG1_G0Nn	14	65891241	1	5.74E-18	1.13E-11	-	-0.29884	-0.2536	+	G	A	0.478319393	0.0341922	+	G	A	0.493	0.0374
rs11628196	LC_IGP12	IgG1_G1	14	65891241	1	1.64E-16	4.52E-14	-	-0.283083	-0.284	+	G	A	0.477997251	0.0339684	+	G	A	0.493	0.0376
rs11628196	LC_IGP_R2	IgG1_G1F/IgG1_G1	14	65891241	1	7.71E-18	5.60E-14	+	0.295219	0.2823	+	G	A	0.477997251	0.0339197	+	G	A	0.493	0.0376
rs11628196	LC_IGP_R5	IgG1_G1FN/IgG1_G1N	14	65891241	1	4.46E-15	4.87E-18	+	0.271366	0.326	+	G	A	0.479381304	0.0342746	+	G	A	0.493	0.0377
rs11628196	LC_IGP53	IgG1_G1n	14	65891241	1	1.29E-15	8.62E-14	-	-0.277101	-0.2806	+	G	A	0.478319393	0.034309	+	G	A	0.493	0.0376
rs11628196	LC_IGP15	IgG1_G1N	14	65891241	1	4.43E-12	5.30E-11	-	-0.239949	-0.2456	+	G	A	0.479381304	0.0344001	+	G	A	0.493	0.0374
rs11628196	LC_IGP56	IgG1_G1Nn	14	65891241	1	1.94E-12	1.18E-10	-	-0.244855	-0.2407	+	G	A	0.478319393	0.0345155	+	G	A	0.493	0.0374
rs11628196	LC_IGP_R36	IgG1_G1NS1/IgG1_G1N	14	65891241	1	2.98E-09	2.12E-12	+	0.204763	0.2574	+	G	A	0.479330456	0.0343209	+	G	A	0.493	0.0366
rs11628196	LC_IGP_R32	IgG1_G1S1/IgG1_G1	14	65891241	1	1.29E-10	2.59E-10	+	0.221447	0.2285	+	G	A	0.477664526	0.0342233	+	G	A	0.493	0.0361
rs11628196	LC_IGP_R3	IgG1_G2F/IgG1_G2	14	65891241	1	7.30E-16	3.81E-15	+	0.277408	0.2937	+	G	A	0.477678178	0.0340479	+	G	A	0.493	0.0374
rs11628196	LC_IGP_R8	IgG1_G2FS1/IgG1_G2S1	14	65891241	1	2.71E-10	3.57E-13	+	0.217969	0.2711	+	G	A	0.477386378	0.0343021	+	G	A	0.493	0.0373
rs11628196	LC_IGP_R26	IgG1_G2N/IgG1_G1N	14	65891241	1	6.58E-13	6.23E-09	+	0.248414	0.212	+	G	A	0.479016975	0.0342795	+	G	A	0.493	0.0365
rs11628196	LC_IGP148	IgG2 FG0n total/G0n	14	65891241	1	5.53E-09	3.39E-14	+	0.20192	0.2824	+	G	A	0.476616687	0.0344449	+	G	A	0.493	0.0372
rs11628196	LC_IGP97	IgG2_G0	14	65891241	1	1.24E-10	2.28E-14	-	-0.221538	-0.2847	+	G	A	0.477678178	0.0342075	+	G	A	0.493	0.0373
rs11628196	LC_IGP_R41	IgG2_G0F/IgG2_G0	14	65891241	1	4.30E-08	1.58E-13	+	0.188871	0.2735	+	G	A	0.477678178	0.0343154	+	G	A	0.493	0.0371
rs11628196	LC_IGP138	IgG2_G0n	14	65891241	1	7.74E-11	1.70E-14	-	-0.224798	-0.2865	+	G	A	0.476616687	0.0343246	+	G	A	0.493	0.0374
rs11628196	LC_IGP_R64	IgG2_G2/IgG2_G1	14	65891241	1	3.47E-09	6.08E-13	+	0.204838	0.2633	+	G	A	0.476732864	0.0344824	+	G	A	0.493	0.0366
rs867972	LC_IGP78	IgG1 BG0n/G0n	14	65895761	1	1.38E-13	2.12E-10	-	-0.2573	-0.2381	+	C	T	0.464810431	0.034478	+	C	T	0.4875	0.0375
rs867972	LC_IGP79	IgG1 BG1n/G1n	14	65895761	1	4.59E-13	1.26E-12	-	-0.252369	-0.2657	+	C	T	0.464810431	0.0345835	+	C	T	0.4875	0.0374
rs867972	LC_IGP32	IgG1 BG1S1/(BG1+BG1S1)	14	65895761	1	1.79E-08	1.17E-12	+	0.19496	0.2607	+	C	T	0.465888739	0.0344496	+	C	T	0.4875	0.0367
rs867972	LC_IGP77	IgG1 Bn	14	65895761	1	9.64E-13	3.36E-12	-	-0.248934	-0.2619	+	C	T	0.464810431	0.0346061	+	C	T	0.4875	0.0376
rs867972	LC_IGP86	IgG1 Bn/Fn total	14	65895761	1	1.47E-13	1.83E-12	-	-0.257606	-0.2653	+	C	T	0.464810431	0.0345589	+	C	T	0.4875	0.0376
rs867972	LC_IGP84	IgG1 FBn/Bn total	14	65895761	1	2.11E-14	2.04E-17	+	0.266836	0.322	+	C	T	0.464810431	0.0345881	+	C	T	0.4875	0.0379
rs867972	LC_IGP62	IgG1 FG0n total/G0n	14	65895761	1	3.85E-17	3.31E-13	+	0.291663	0.2747	+	C	T	0.464810431	0.0342651	+	C	T	0.4875	0.0377
rs867972	LC_IGP63	IgG1 FG1n total/G1n	14	65895761	1	2.63E-17	1.38E-15	+	0.294034	0.3009	+	C	T	0.464810431	0.0343581	+	C	T	0.4875	0.0377
rs867972	LC_IGP64	IgG1 FG2n total/G2n	14	65895761	1	3.10E-13	2.28E-16	+	0.254306	0.3075	+	C	T	0.464810431	0.0345914	+	C	T	0.4875	0.0375
rs867972	LC_IGP68	IgG1 FG2n/G2n	14	65895761	1	3.96E-10	5.57E-09	+	0.218616	0.2175	+	C	T	0.464810431	0.0347308	+	C	T	0.4875	0.0373
rs867972	LC_IGP61	IgG1 Fn total	14	65895761	1	2.37E-16	3.37E-15	+	0.284996	0.297	+	C	T	0.464810431	0.0343802	+	C	T	0.4875	0.0377
rs867972	LC_IGP81	IgG1 Fn/Bn	14	65895761	1	3.99E-13	2.21E-11	+	0.25293	0.2506	+	C	T	0.464810431	0.0345685	+	C	T	0.4875	0.0374
rs867972	LC_IGP21	IgG1 Fucosylation	14	65895761	1	2.54E-14	3.89E-14	+	0.264335	0.2849	+	C	T	0.46407308	0.0343753	+	C	T	0.4875	0.0377
rs867972	LC_IGP28	IgG1 G1S1/(G1+G1S1)	14	65895761	1	4.21E-11	5.02E-11	+	0.227799	0.2376	+	C	T	0.46432204	0.0342985	+	C	T	0.4875	0.0362
rs867972	LC_IGP26	IgG1 GS1/(G+GS1)	14	65895761	1	1.52E-08	7.78E-11	+	0.196232	0.2408	+	C	T	0.46432204	0.0345014	+	C	T	0.4875	0.037
rs867972	LC_IGP27	IgG1 GS1/(G0+G+GS1)	14	65895761	1	5.41E-10	2.35E-09	+	0.214766	0.2198	+	C	T	0.46432204	0.0343926	+	C	T	0.4875	0.0368
rs867972	LC_IGP11	IgG1_G0	14	65895761	1	2.05E-19	1.66E-15	-	-0.309304	-0.2989	+	C	T	0.46436563	0.0338956	+	C	T	0.4875	0.0375
rs867972	LC_IGP_R1	IgG1_G0F/IgG1_G0	14	65895761	1	9.04E-17	8.81E-13	+	0.285877	0.2687	+	C	T	0.46436563	0.0340053	+	C	T	0.4875	0.0376
rs867972	LC_IGP_R4	IgG1_G0FN/IgG1_G0N	14	65895761	1	3.95E-14	3.73E-14	+	0.261786	0.2867	+	C	T	0.464896531	0.0343075	+	C	T	0.4875	0.0379
rs867972	LC_IGP52	IgG1_G0n	14	65895761	1	3.65E-19	1.70E-15	-	-0.30936	-0.2993	+	C	T	0.464810431	0.0341428	+	C	T	0.4875	0.0376
rs867972	LC_IGP14	IgG1_G0N	14	65895761	1	7.81E-17	6.74E-12	-	-0.28788	-0.2566	+	C	T	0.464896531	0.0341703	+	C	T	0.4875	0.0374
rs867972	LC_IGP55	IgG1_G0Nn	14	65895761	1	8.19E-17	7.88E-12	-	-0.28925	-0.256	+	C	T	0.464810431	0.0343518	+	C	T	0.4875	0.0374
rs867972	LC_IGP12	IgG1_G1	14	65895761	1	4.66E-16	9.33E-15	-	-0.279869	-0.2918	+	C	T	0.46436563	0.0341145	+	C	T	0.4875	0.0377
rs867972	LC_IGP_R2	IgG1_G1F/IgG1_G1	14	65895761	1	1.48E-17	1.32E-14	+	0.293766	0.2896	+	C	T	0.46436563	0.0340576	+	C	T	0.4875	0.0376
rs867972	LC_IGP_R5	IgG1_G1FN/IgG1_G1N	14	65895761	1	8.39E-14	1.19E-18	+	0.259284	0.3324	+	C	T	0.465972653	0.0344347	+	C	T	0.4875	0.0377
rs867972	LC_IGP53	IgG1_G1n	14	65895761	1	7.32E-15	2.01E-14	-	-0.270554	-0.2879	+	C	T	0.464810431	0.0344496	+	C	T	0.4875	0.0376
rs867972	LC_IGP15	IgG1_G1N	14	65895761	1	3.05E-11	1.71E-11	-	-0.231085	-0.2521	+	C	T	0.465972653	0.0345397	+	C	T	0.4875	0.0375
rs867972	LC_IGP56	IgG1_G1Nn	14	65895761	1	1.22E-11	4.26E-11	-	-0.236644	-0.2467	+	C	T	0.464810431	0.0346589	+	C	T	0.4875	0.0374
rs867972	LC_IGP_R36	IgG1_G1NS1/IgG1_G1N	14	65895761	1	1.40E-08	1.37E-12	+	0.196466	0.2602	+	C	T	0.465888739	0.0344525	+	C	T	0.4875	0.0367
rs867972	LC_IGP_R32	IgG1_G1S1/IgG1_G1	14	65895761	1	7.53E-11	7.65E-11	+	0.224874	0.2356	+	C	T	0.46432204	0.0343156	+	C	T	0.4875	0.0362
rs867972	LC_IGP_R3	IgG1_G2F/IgG1_G2	14	65895761	1	9.28E-15	7.83E-16	+	0.267525	0.3013	+	C	T	0.464343848	0.034203	+	C	T	0.4875	0.0374
rs867972	LC_IGP_R8	IgG1_G2FS1/IgG1_G2S1	14	65895761	1	2.75E-09	3.87E-14	+	0.205998	0.2823	+	C	T	0.464060193	0.0344541	+	C	T	0.4875	0.0373
rs867972	LC_IGP_R26	IgG1_G2N/IgG1_G1N	14	65895761	1	1.64E-12	3.53E-09	+	0.244845	0.2158	+	C	T	0.465558046	0.0343995	+	C	T	0.4875	0.0366
rs867972	LC_IGP97	IgG2_G0	14	65895761	1	1.20E-09	1.37E-14	-	-0.210136	-0.2878	+	C	T	0.464343848	0.0343572	+	C	T	0.4875	0.0374
rs867972	LC_IGP138	IgG2_G0n	14	65895761	1	9.10E-10	1.07E-14	-	-0.212457	-0.2894	+	C	T	0.463183212	0.0344843	+	C	T	0.4875	0.0374
rs867972	LC_IGP_R64	IgG2_G2/IgG2_G1	14	65895761	1	5.47E-09	7.38E-13	+	0.202877	0.2628	+	C	T	0.463382213	0.0345994	+	C	T	0.4875	0.0366
rs111568962	LC_IGP78	IgG1 BG0n/G0n	14	65897898	1	2.35E-13	1.97E-09	-	-0.255041	-0.2318	+	T	A	0.464245663	0.0345124	+	T	A	0.4651	0.0386
rs111568962	LC_IGP79	IgG1 BG1n/G1n	14	65897898	1	5.30E-13	1.48E-11	-	-0.251867	-0.2602	+	T	A	0.464245663	0.0346098	+	T	A	0.4651	0.0386
rs111568962	LC_IGP32	IgG1 BG1S1/(BG1+BG1S1)	14	65897898	1	1.94E-08	3.19E-12	+	0.194637	0.2636	+	T	A	0.465327374	0.0344739	+	T	A	0.4651	0.0378
rs111568962	LC_IGP77	IgG1 Bn	14	65897898	1	1.29E-12	3.86E-11	-	-0.247715	-0.2562	+	T	A	0.464245663	0.0346356	+	T	A	0.4651	0.0388
rs111568962	LC_IGP86	IgG1 Bn/Fn total	14	65897898	1	1.99E-13	2.09E-11	-	-0.256382	-0.2599	+	T	A	0.464245663	0.0345886	+	T	A	0.4651	0.0388
rs111568962	LC_IGP84	IgG1 FBn/Bn total	14	65897898	1	2.89E-14	2.37E-16	+	0.265626	0.3204	+	T	A	0.464245663	0.034618	+	T	A	0.4651	0.0391
rs111568962	LC_IGP62	IgG1 FG0n total/G0n	14	65897898	1	6.03E-17	2.20E-12	+	0.290072	0.2732	+	T	A	0.464245663	0.0342976	+	T	A	0.4651	0.0389
rs111568962	LC_IGP63	IgG1 FG1n total/G1n	14	65897898	1	4.09E-17	1.48E-14	+	0.292487	0.2987	+	T	A	0.464245663	0.0343905	+	T	A	0.4651	0.0388
rs111568962	LC_IGP64	IgG1 FG2n total/G2n	14	65897898	1	3.28E-13	3.30E-15	+	0.254214	0.3047	+	T	A	0.464245663	0.0346159	+	T	A	0.4651	0.0387
rs111568962	LC_IGP68	IgG1 FG2n/G2n	14	65897898	1	3.49E-10	4.09E-08	+	0.219446	0.2114	+	T	A	0.464245663	0.0347515	+	T	A	0.4651	0.0385
rs111568962	LC_IGP61	IgG1 Fn total	14	65897898	1	3.54E-16	3.93E-14	+	0.283534	0.2942	+	T	A	0.464245663	0.0344118	+	T	A	0.4651	0.0389
rs111568962	LC_IGP81	IgG1 Fn/Bn	14	65897898	1	5.32E-13	2.16E-10	+	0.251755	0.2451	+	T	A	0.464245663	0.0345978	+	T	A	0.4651	0.0386
rs111568962	LC_IGP21	IgG1 Fucosylation	14	65897898	1	3.82E-14	4.18E-13	+	0.262712	0.2818	+	T	A	0.463510789	0.034407	+	T	A	0.4651	0.0389
rs111568962	LC_IGP28	IgG1 G1S1/(G1+G1S1)	14	65897898	1	4.65E-11	1.68E-10	+	0.227451	0.2382	+	T	A	0.463765443	0.0343232	+	T	A	0.4651	0.0373
rs111568962	LC_IGP26	IgG1 GS1/(G+GS1)	14	65897898	1	1.58E-08	4.18E-10	+	0.196133	0.2388	+	T	A	0.463765443	0.034525	+	T	A	0.4651	0.0382
rs111568962	LC_IGP27	IgG1 GS1/(G0+G+GS1)	14	65897898	1	5.29E-10	7.61E-09	+	0.215031	0.2194	+	T	A	0.463765443	0.0344149	+	T	A	0.4651	0.038
rs111568962	LC_IGP11	IgG1_G0	14	65897898	1	3.01E-19	8.08E-15	-	-0.308084	-0.3011	+	T	A	0.463809713	0.033926	+	T	A	0.4651	0.0388
rs111568962	LC_IGP_R1	IgG1_G0F/IgG1_G0	14	65897898	1	1.34E-16	5.21E-12	+	0.284489	0.2678	+	T	A	0.463809713	0.0340359	+	T	A	0.4651	0.0388
rs111568962	LC_IGP_R4	IgG1_G0FN/IgG1_G0N	14	65897898	1	7.17E-14	3.28E-13	+	0.259308	0.2841	+	T	A	0.464338947	0.0343426	+	T	A	0.4651	0.039
rs111568962	LC_IGP52	IgG1_G0n	14	65897898	1	5.41E-19	7.76E-15	-	-0.308094	-0.3018	+	T	A	0.464245663	0.034174	+	T	A	0.4651	0.0388
rs111568962	LC_IGP14	IgG1_G0N	14	65897898	1	1.47E-16	5.58E-11	-	-0.285515	-0.2525	+	T	A	0.464338947	0.0342061	+	T	A	0.4651	0.0385
rs111568962	LC_IGP55	IgG1_G0Nn	14	65897898	1	1.56E-16	6.37E-11	-	-0.286842	-0.252	+	T	A	0.464245663	0.0343885	+	T	A	0.4651	0.0386
rs111568962	LC_IGP12	IgG1_G1	14	65897898	1	7.41E-16	8.60E-14	-	-0.278145	-0.2898	+	T	A	0.463809713	0.0341466	+	T	A	0.4651	0.0388
rs111568962	LC_IGP_R2	IgG1_G1F/IgG1_G1	14	65897898	1	2.37E-17	1.19E-13	+	0.292119	0.2877	+	T	A	0.463809713	0.0340898	+	T	A	0.4651	0.0388
rs111568962	LC_IGP_R5	IgG1_G1FN/IgG1_G1N	14	65897898	1	1.02E-13	1.22E-17	+	0.258567	0.3322	+	T	A	0.465412669	0.0344616	+	T	A	0.4651	0.0388
rs111568962	LC_IGP53	IgG1_G1n	14	65897898	1	1.15E-14	1.76E-13	-	-0.268779	-0.2859	+	T	A	0.464245663	0.0344823	+	T	A	0.4651	0.0388
rs111568962	LC_IGP15	IgG1_G1N	14	65897898	1	3.68E-11	2.00E-10	-	-0.230296	-0.2455	+	T	A	0.465412669	0.0345663	+	T	A	0.4651	0.0386
rs111568962	LC_IGP56	IgG1_G1Nn	14	65897898	1	1.48E-11	4.53E-10	-	-0.235823	-0.2403	+	T	A	0.464245663	0.0346864	+	T	A	0.4651	0.0386
rs111568962	LC_IGP_R36	IgG1_G1NS1/IgG1_G1N	14	65897898	1	1.53E-08	3.78E-12	+	0.196088	0.2629	+	T	A	0.465327374	0.0344771	+	T	A	0.4651	0.0378
rs111568962	LC_IGP_R32	IgG1_G1S1/IgG1_G1	14	65897898	1	8.49E-11	2.76E-10	+	0.224409	0.2357	+	T	A	0.463765443	0.0343408	+	T	A	0.4651	0.0373
rs111568962	LC_IGP_R3	IgG1_G2F/IgG1_G2	14	65897898	1	1.06E-14	1.08E-14	+	0.267134	0.2983	+	T	A	0.463787592	0.0342283	+	T	A	0.4651	0.0386
rs111568962	LC_IGP_R8	IgG1_G2FS1/IgG1_G2S1	14	65897898	1	3.41E-09	6.42E-13	+	0.20493	0.2767	+	T	A	0.463504276	0.0344813	+	T	A	0.4651	0.0385
rs111568962	LC_IGP_R26	IgG1_G2N/IgG1_G1N	14	65897898	1	2.35E-12	1.15E-08	+	0.243294	0.2151	+	T	A	0.46499537	0.0344299	+	T	A	0.4651	0.0377

rs111568962	LC_IGP97	IgG2_G0	14	65897898	1	1.29E-09	1.14E-14	-	-0.209861	-0.2981	+	T	A	0.463787592	0.0343815	+	T	A	0.4651	0.0386
rs111568962	LC_IGP138	IgG2_G0n	14	65897898	1	9.97E-10	9.49E-15	-	-0.212103	-0.2994	+	T	A	0.462621921	0.0345095	+	T	A	0.4651	0.0386
rs111568962	LC_IGP_R64	IgG2_G2/IgG2_G1	14	65897898	1	4.20E-09	5.13E-13	+	0.204522	0.2727	+	T	A	0.462825275	0.0346171	+	T	A	0.4651	0.0378
rs4456409	LC_IGP78	IgG1 BG0n/G0n	14	65897967	1	2.35E-13	6.58E-10	-	-0.255036	-0.2369	+	C	T	0.464246592	0.0345123	+	C	T	0.4804	0.0384
rs4456409	LC_IGP79	IgG1 BG1n/G1n	14	65897967	1	5.30E-13	5.95E-12	-	-0.251861	-0.2637	+	C	T	0.464246592	0.0346097	+	C	T	0.4804	0.0383
rs4456409	LC_IGP32	IgG1 BG1S1/(BG1+BG1S1)	14	65897967	1	1.94E-08	4.54E-12	+	0.194635	0.2593	+	C	T	0.465328298	0.0344738	+	C	T	0.4804	0.0375
rs4456409	LC_IGP77	IgG1 Bn	14	65897967	1	1.29E-12	1.32E-11	-	-0.24771	-0.2606	+	C	T	0.464246592	0.0346354	+	C	T	0.4804	0.0385
rs4456409	LC_IGP86	IgG1 Bn/Fn total	14	65897967	1	2.00E-13	7.72E-12	-	-0.256377	-0.2636	+	C	T	0.464246592	0.0345885	+	C	T	0.4804	0.0385
rs4456409	LC_IGP84	IgG1 FBn/Bn total	14	65897967	1	2.89E-14	7.64E-17	+	0.265622	0.323	+	C	T	0.464246592	0.0346179	+	C	T	0.4804	0.0387
rs4456409	LC_IGP62	IgG1 FG0n total/G0n	14	65897967	1	6.03E-17	1.23E-12	+	0.290071	0.2739	+	C	T	0.464246592	0.0342974	+	C	T	0.4804	0.0386
rs4456409	LC_IGP63	IgG1 FG1n total/G1n	14	65897967	1	4.09E-17	9.40E-15	+	0.292484	0.2985	+	C	T	0.464246592	0.0343904	+	C	T	0.4804	0.0385
rs4456409	LC_IGP64	IgG1 FG2n total/G2n	14	65897967	1	3.28E-13	1.92E-15	+	0.254211	0.305	+	C	T	0.464246592	0.0346157	+	C	T	0.4804	0.0384
rs4456409	LC_IGP68	IgG1 FG2n/G2n	14	65897967	1	3.49E-10	2.85E-08	+	0.219443	0.2125	+	C	T	0.464246592	0.0347514	+	C	T	0.4804	0.0383
rs4456409	LC_IGP61	IgG1 Fn total	14	65897967	1	3.54E-16	1.99E-14	+	0.283531	0.2952	+	C	T	0.464246592	0.0344117	+	C	T	0.4804	0.0386
rs4456409	LC_IGP81	IgG1 Fn/Bn	14	65897967	1	5.33E-13	9.27E-11	+	0.251749	0.2486	+	C	T	0.464246592	0.0345977	+	C	T	0.4804	0.0384
rs4456409	LC_IGP21	IgG1 Fucosylation	14	65897967	1	3.83E-14	2.59E-13	+	0.262707	0.2819	+	C	T	0.463511714	0.0344069	+	C	T	0.4804	0.0385
rs4456409	LC_IGP28	IgG1 G1S1/(G1+G1S1)	14	65897967	1	4.65E-11	2.10E-10	+	0.227449	0.2349	+	C	T	0.463766361	0.0343231	+	C	T	0.4804	0.037
rs4456409	LC_IGP26	IgG1 GS1/(G+GS1)	14	65897967	1	1.58E-08	1.20E-10	+	0.196132	0.2441	+	C	T	0.463766361	0.0345249	+	C	T	0.4804	0.0379
rs4456409	LC_IGP27	IgG1 GS1/(G0+G+GS1)	14	65897967	1	5.28E-10	2.75E-09	+	0.215032	0.2239	+	C	T	0.463766361	0.0344147	+	C	T	0.4804	0.0377
rs4456409	LC_IGP11	IgG1_G0	14	65897967	1	3.01E-19	4.96E-15	-	-0.308087	-0.3004	+	C	T	0.463810629	0.0339258	+	C	T	0.4804	0.0384
rs4456409	LC_IGP_R1	IgG1_G0F/IgG1_G0	14	65897967	1	1.34E-16	3.58E-12	+	0.284489	0.2674	+	C	T	0.463810629	0.0340357	+	C	T	0.4804	0.0385
rs4456409	LC_IGP_R4	IgG1_G0FN/IgG1_G0N	14	65897967	1	7.17E-14	9.43E-14	+	0.259305	0.2884	+	C	T	0.464339865	0.0343425	+	C	T	0.4804	0.0387
rs4456409	LC_IGP52	IgG1_G0n	14	65897967	1	5.41E-19	5.63E-15	-	-0.308097	-0.3003	+	C	T	0.464246592	0.0341738	+	C	T	0.4804	0.0384
rs4456409	LC_IGP14	IgG1_G0N	14	65897967	1	1.47E-16	1.75E-11	-	-0.285514	-0.2574	+	C	T	0.464339865	0.0342059	+	C	T	0.4804	0.0383
rs4456409	LC_IGP55	IgG1_G0Nn	14	65897967	1	1.56E-16	2.25E-11	-	-0.286841	-0.2562	+	C	T	0.464246592	0.0343883	+	C	T	0.4804	0.0383
rs4456409	LC_IGP12	IgG1_G1	14	65897967	1	7.42E-16	5.01E-14	-	-0.278142	-0.2901	+	C	T	0.463810629	0.0341465	+	C	T	0.4804	0.0385
rs4456409	LC_IGP_R2	IgG1_G1F/IgG1_G1	14	65897967	1	2.37E-17	9.19E-14	+	0.292116	0.2866	+	C	T	0.463810629	0.0340896	+	C	T	0.4804	0.0385
rs4456409	LC_IGP_R5	IgG1_G1FN/IgG1_G1N	14	65897967	1	1.02E-13	6.27E-18	+	0.258562	0.3325	+	C	T	0.465413592	0.0344614	+	C	T	0.4804	0.0385
rs4456409	LC_IGP53	IgG1_G1n	14	65897967	1	1.15E-14	1.15E-13	-	-0.268776	-0.2857	+	C	T	0.464246592	0.0344822	+	C	T	0.4804	0.0385
rs4456409	LC_IGP15	IgG1_G1N	14	65897967	1	3.68E-11	6.48E-11	-	-0.230291	-0.2506	+	C	T	0.465413592	0.0345662	+	C	T	0.4804	0.0384
rs4456409	LC_IGP56	IgG1_G1Nn	14	65897967	1	1.48E-11	1.66E-10	-	-0.235817	-0.2447	+	C	T	0.464246592	0.0346862	+	C	T	0.4804	0.0383
rs4456409	LC_IGP_R36	IgG1_G1NS1/IgG1_G1N	14	65897967	1	1.53E-08	5.28E-12	+	0.196086	0.2587	+	C	T	0.465328298	0.034477	+	C	T	0.4804	0.0375
rs4456409	LC_IGP_R32	IgG1_G1S1/IgG1_G1	14	65897967	1	8.49E-11	3.25E-10	+	0.224407	0.2327	+	C	T	0.463766361	0.0343406	+	C	T	0.4804	0.037
rs4456409	LC_IGP_R3	IgG1_G2F/IgG1_G2	14	65897967	1	1.06E-14	6.42E-15	+	0.267131	0.2986	+	C	T	0.463788509	0.0342282	+	C	T	0.4804	0.0383
rs4456409	LC_IGP_R8	IgG1_G2FS1/IgG1_G2S1	14	65897967	1	3.41E-09	2.38E-13	+	0.20493	0.28	+	C	T	0.463505192	0.0344812	+	C	T	0.4804	0.0382
rs4456409	LC_IGP_R26	IgG1_G2N/IgG1_G1N	14	65897967	1	2.35E-12	6.14E-09	+	0.243294	0.2176	+	C	T	0.464996296	0.0344297	+	C	T	0.4804	0.0374
rs4456409	LC_IGP97	IgG2_G0	14	65897967	1	1.29E-09	1.35E-14	-	-0.209863	-0.2937	+	C	T	0.463788509	0.0343813	+	C	T	0.4804	0.0381
rs4456409	LC_IGP138	IgG2_G0n	14	65897967	1	9.96E-10	1.14E-14	-	-0.212105	-0.2949	+	C	T	0.462622845	0.0345093	+	C	T	0.4804	0.0382
rs4456409	LC_IGP_R64	IgG2_G2/IgG2_G1	14	65897967	1	4.20E-09	7.55E-13	+	0.204528	0.2692	+	C	T	0.462826193	0.0346169	+	C	T	0.4804	0.0376
rs2002692	LC_IGP78	IgG1 BG0n/G0n	14	65899454	1	5.59E-13	3.26E-10	-	-0.250379	-0.2358	+	G	A	0.469192999	0.0344405	+	G	A	0.487	0.0375
rs2002692	LC_IGP79	IgG1 BG1n/G1n	14	65899454	1	1.08E-12	2.11E-12	-	-0.247868	-0.2633	+	G	A	0.469192999	0.0345344	+	G	A	0.487	0.0375
rs2002692	LC_IGP32	IgG1 BG1S1/(BG1+BG1S1)	14	65899454	1	2.06E-08	2.34E-12	+	0.193776	0.2574	+	G	A	0.470250617	0.0343871	+	G	A	0.487	0.0367
rs2002692	LC_IGP77	IgG1 Bn	14	65899454	1	2.72E-12	5.40E-12	-	-0.243518	-0.2596	+	G	A	0.469192999	0.0345607	+	G	A	0.487	0.0377
rs2002692	LC_IGP86	IgG1 Bn/Fn total	14	65899454	1	4.14E-13	2.92E-12	-	-0.252349	-0.263	+	G	A	0.469192999	0.0345136	+	G	A	0.487	0.0377
rs2002692	LC_IGP84	IgG1 FBn/Bn total	14	65899454	1	4.39E-14	3.72E-17	+	0.263069	0.3195	+	G	A	0.469192999	0.0345364	+	G	A	0.487	0.0379
rs2002692	LC_IGP62	IgG1 FG0n total/G0n	14	65899454	1	1.22E-16	5.72E-13	+	0.286489	0.2721	+	G	A	0.469192999	0.0342227	+	G	A	0.487	0.0378
rs2002692	LC_IGP63	IgG1 FG1n total/G1n	14	65899454	1	5.47E-17	2.86E-15	+	0.29056	0.2978	+	G	A	0.469192999	0.0343068	+	G	A	0.487	0.0377
rs2002692	LC_IGP64	IgG1 FG2n total/G2n	14	65899454	1	3.09E-13	5.34E-16	+	0.253819	0.304	+	G	A	0.469192999	0.0345242	+	G	A	0.487	0.0375
rs2002692	LC_IGP68	IgG1 FG2n/G2n	14	65899454	1	5.70E-10	9.70E-09	+	0.216227	0.2144	+	G	A	0.469192999	0.0346711	+	G	A	0.487	0.0374
rs2002692	LC_IGP61	IgG1 Fn total	14	65899454	1	4.84E-16	6.96E-15	+	0.281508	0.294	+	G	A	0.469192999	0.0343285	+	G	A	0.487	0.0378
rs2002692	LC_IGP81	IgG1 Fn/Bn	14	65899454	1	1.18E-12	3.62E-11	+	0.247371	0.2482	+	G	A	0.469192999	0.0345242	+	G	A	0.487	0.0375
rs2002692	LC_IGP21	IgG1 Fucosylation	14	65899454	1	5.23E-14	7.72E-14	+	0.260632	0.2819	+	G	A	0.468433107	0.0343232	+	G	A	0.487	0.0377
rs2002692	LC_IGP28	IgG1 G1S1/(G1+G1S1)	14	65899454	1	7.75E-11	9.05E-11	+	0.224257	0.2346	+	G	A	0.468649235	0.0342447	+	G	A	0.487	0.0362
rs2002692	LC_IGP26	IgG1 GS1/(G+GS1)	14	65899454	1	1.74E-08	1.30E-10	+	0.195069	0.2382	+	G	A	0.468649235	0.0344373	+	G	A	0.487	0.0371
rs2002692	LC_IGP27	IgG1 GS1/(G0+G+GS1)	14	65899454	1	7.29E-10	3.46E-09	+	0.212744	0.2176	+	G	A	0.468649235	0.0343322	+	G	A	0.487	0.0368
rs2002692	LC_IGP11	IgG1_G0	14	65899454	1	1.25E-18	2.96E-15	-	-0.301778	-0.2964	+	G	A	0.468992975	0.0338443	+	G	A	0.487	0.0376
rs2002692	LC_IGP_R1	IgG1_G0F/IgG1_G0	14	65899454	1	6.56E-16	1.57E-12	+	0.277122	0.266	+	G	A	0.468992975	0.0339572	+	G	A	0.487	0.0376
rs2002692	LC_IGP_R4	IgG1_G0FN/IgG1_G0N	14	65899454	1	1.55E-13	5.96E-14	+	0.255001	0.2845	+	G	A	0.469534905	0.0342466	+	G	A	0.487	0.0379
rs2002692	LC_IGP52	IgG1_G0n	14	65899454	1	1.17E-18	3.05E-15	-	-0.304397	-0.2968	+	G	A	0.469192999	0.0341008	+	G	A	0.487	0.0376
rs2002692	LC_IGP14	IgG1_G0N	14	65899454	1	8.00E-16	1.03E-11	-	-0.277683	-0.2544	+	G	A	0.469534905	0.0341294	+	G	A	0.487	0.0374
rs2002692	LC_IGP55	IgG1_G0Nn	14	65899454	1	3.96E-16	1.20E-11	-	-0.282286	-0.2538	+	G	A	0.469192999	0.0343182	+	G	A	0.487	0.0374
rs2002692	LC_IGP12	IgG1_G1	14	65899454	1	2.06E-15	1.87E-14	-	-0.272991	-0.2888	+	G	A	0.468992975	0.034056	+	G	A	0.487	0.0377
rs2002692	LC_IGP_R2	IgG1_G1F/IgG1_G1	14	65899454	1	8.15E-17	2.68E-14	+	0.286295	0.2865	+	G	A	0.468992975	0.0340037	+	G	A	0.487	0.0376
rs2002692	LC_IGP_R5	IgG1_G1FN/IgG1_G1N	14	65899454	1	1.43E-13	2.44E-18	+	0.256396	0.3296	+	G	A	0.470323801	0.0343808	+	G	A	0.487	0.0377
rs2002692	LC_IGP53	IgG1_G1n	14	65899454	1	1.37E-14	3.99E-14	-	-0.267319	-0.2849	+	G	A	0.469192999	0.0343959	+	G	A	0.487	0.0377
rs2002692	LC_IGP15	IgG1_G1N	14	65899454	1	6.93E-11	2.71E-11	-	-0.226472	-0.2498	+	G	A	0.470323801	0.0344915	+	G	A	0.487	0.0375
rs2002692	LC_IGP56	IgG1_G1Nn	14	65899454	1	2.96E-11	6.71E-11	-	-0.231738	-0.2444	+	G	A	0.469192999	0.0346103	+	G	A	0.487	0.0374
rs2002692	LC_IGP_R36	IgG1_G1NS1/IgG1_G1N	14	65899454	1	1.68E-08	2.69E-12	+	0.195031	0.2569	+	G	A	0.470250617	0.034391	+	G	A	0.487	0.0367
rs2002692	LC_IGP_R32	IgG1_G1S1/IgG1_G1	14	65899454	1	1.45E-10	1.36E-10	+	0.221068	0.2327	+	G	A	0.468649235	0.0342626	+	G	A	0.487	0.0362
rs2002692	LC_IGP_R3	IgG1_G2F/IgG1_G2	14	65899454	1	8.99E-15	1.83E-15	+	0.267144	0.2977	+	G	A	0.468668399	0.0341362	+	G	A	0.487	0.0374
rs2002692	LC_IGP_R8	IgG1_G2FS1/IgG1_G2S1	14	65899454	1	2.88E-09	8.12E-14	+	0.205354	0.279	+	G	A	0.468382101	0.0343881	+	G	A	0.487	0.0374
rs2002692	LC_IGP_R26	IgG1_G2N/IgG1_G1N	14	65899454	1	3.96E-12	5.04E-09	+	0.240181	0.2139	+	G	A	0.469924383	0.0343522	+	G	A	0.487	0.0366
rs2002692	LC_IGP148	IgG2 FG0n total/G0n	14	65899454	1	3.05E-08	1.93E-14	+	0.192128	0.2859	+	G	A	0.467538485	0.0345206	+	G	A	0.487	0.0374
rs2002692	LC_IGP97	IgG2_G0	14	65899454	1	5.07E-10	1.14E-14	-	-0.214373	-0.2888	+	G	A	0.468668399	0.0342732	+	G	A	0.487	0.0374
rs2002692	LC_IGP138	IgG2_G0n	14	65899454	1	3.67E-10	9.21E-15	-	-0.216934	-0.2902	+	G	A	0.467538485	0.034397	+	G	A	0.487	0.0374
rs2002692	LC_IGP_R64	IgG2_G2/IgG2_G1	14	65899454	1	3.05E-09	4.92E-13	+	0.205808	0.265	+	G	A	0.467712056	0.0345192	+	G	A	0.487	0.0367
rs11620749	LC_IGP78	IgG1 BG0n/G0n	14	65899804	1	5.59E-13	3.26E-10	-	-0.250373	-0.2358	+	G	A	0.469195167	0.0344399	+	G	A	0.487	0.0375
rs11620749	LC_IGP79	IgG1 BG1n/G1n	14	65899804	1	1.08E-12	2.11E-12	-	-0.247857	-0.2633	+	G	A	0.469195167	0.0345338	+	G	A	0.487	0.0375
rs11620749	LC_IGP32	IgG1 BG1S1/(BG1+BG1S1)	14	65899804	1	2.06E-08	2.34E-12	+	0.193774	0.2574	+	G	A	0.470252774	0.0343865	+	G	A	0.487	0.0367
rs11620749	LC_IGP77	IgG1 Bn	14	65899804	1	2.72E-12	5.40E-12	-	-0.243512	-0.2596	+	G	A	0.469195167	0.0345601	+	G	A	0.487	0.0377
rs11620749	LC_IGP86	IgG1 Bn/Fn total	14	65899804	1	4.14E-13	2.92E-12	-	-0.252342	-0.263	+	G	A	0.469195167	0.034513	+	G	A	0.487	0.0377
rs11620749	LC_IGP84	IgG1 FBn/Bn total	14	65899804	1	4.40E-14	3.72E-17	+	0.263063	0.3195	+	G	A	0.469195167	0.0345358	+	G	A	0.487	0.0379
rs11620749	LC_IGP62	IgG1 FG0n total/G0n	14	65899804	1	1.22E-16	5.72E-13	+	0.286485	0.2721	+	G	A	0.469195167	0.0342221	+	G	A	0.487	0.0378
rs11620749	LC_IGP63	IgG1 FG1n total/G1n	14	65899804	1	5.48E-17	2.86E-15	+	0.290549	0.2978	+	G	A	0.469195167	0.0343062	+	G	A	0.487	0.0377
rs11620749	LC_IGP64	IgG1 FG2n total/G2n	14	65899804	1	3.09E-13	5.33E-16	+	0.253813	0.304	+	G	A	0.469195167	0.0345236	+	G	A	0.487	0.0375
rs11620749	LC_IGP68	IgG1 FG2n/G2n	14	65899804	1	5.71E-10	9.70E-09	+	0.216218	0.2144	+	G	A	0.469195167	0.0346705	+	G	A	0.487	0.0374
rs11620749	LC_IGP61	IgG1 Fn total	14	65899804	1	4.84E-16	6.96E-15	+	0.281498	0.294	+	G	A	0.469195167	0.0343279	+	G	A	0.487	0.0378
rs11620749	LC_IGP81	IgG1 Fn/Bn	14	65899804	1	1.18E-12	3.62E-11	+	0.247364	0.2482	+	G	A	0.469195167	0.0345236	+	G	A	0.487	0.0375
rs11620749	LC_IGP21	IgG1 Fucosylation	14	65899804	1	5.24E-14	7.72E-14	+	0.260621	0.2819	+	G	A	0.468435265	0.0343226	+	G	A	0.487	0.0377
rs11620749	LC_IGP28	IgG1 G1S1/(G1+G1S1)	14	65899804	1	7.76E-11	9.04E-11	+	0.224252	0.2346	+	G	A	0.468651376	0.0342441	+	G	A	0.487	0.0362
rs11620749	LC_IGP26	IgG1 GS1/(G+GS1)	14	65899804	1	1.74E-08	1.30E-10	+	0.195074	0.2382	+	G	A	0.468651376	0.0344367	+	G	A	0.487	0.0371
rs11620749	LC_IGP27	IgG1 GS1/(G0+G+GS1)	14	65899804	1	7.27E-10	3.46E-09	+	0.212753	0.2176	+	G	A	0.468651376	0.0343315	+	G	A	0.487	0.0368
rs11620749	LC_IGP11	IgG1_G0	14	65899804	1	1.25E-18	2.96E-15	-	-0.301784	-0.2964	+	G	A	0.468995113	0.0338436	+	G	A	0.487	0.0376
rs11620749	LC_IGP_R1	IgG1_G0F/IgG1_G0	14	65899804	1	6.56E-16	1.57E-12	+	0.277119	0.266	+	G	A	0.468995113	0.0339566	+	G	A	0.487	0.0376
rs11620749	LC_IGP_R4	IgG1_G0FN/IgG1_G0N	14	65899804	1	1.55E-13	5.96E-14	+	0.254997	0.2845	+	G	A	0.469537048	0.034246	+	G	A	0.487	0.0379
rs11620749	LC_IGP52	IgG1_G0n	14	65899804	1	1.17E-18	3.05E-15	-	-0.304402	-0.2968	+	G	A	0.469195167	0.0341002	+	G	A	0.487	0.0376
rs11620749	LC_IGP14	IgG1_G0N	14	65899804	1	7.99E-16	1.03E-11	-	-0.277686	-0.2544	+	G	A	0.469537048	0.0341288	+	G	A	0.487	0.0374
rs11620749	LC_IGP55	IgG1_G0Nn	14	65899804	1	3.95E-16	1.20E-11	-	-0.282288	-0.2538	+	G	A	0.469195167	0.0343175	+	G	A	0.487	0.0374
rs11620749	LC_IGP12	IgG1_G1	14	65899804	1	2.06E-15	1.87E-14	-	-0.272982	-0.2888	+	G	A	0.468995113	0.0340555	+	G	A	0.487	0.0377
rs11620749	LC_IGP_R2	IgG1_G1F/IgG1_G1	14	65899804	1	8.16E-17	2.68E-14	+	0.286284	0.2865	+	G	A	0.468995113	0.0340031	+	G	A	0.487	0.0376
rs11620749	LC_IGP_R5	IgG1_G1FN/IgG1_G1N	14	65899804	1	1.43E-13	2.44E-18	+	0.25639	0.3296	+	G	A	0.470325953	0.0343802	+	G	A	0.487	0.0377
rs11620749	LC_IGP53	IgG1_G1n	14	65899804	1	1.37E-14	3.99E-14	-	-0.267308	-0.2849	+	G	A	0.469195167	0.0343953	+	G	A	0.487	0.0377
rs11620749	LC_IGP15	IgG1_G1N	14	65899804	1	6.94E-11	2.71E-11	-	-0.226463	-0.2498	+	G	A	0.470325953	0.0344909	+	G	A	0.487	0.0375
rs11620749	LC_IGP56	IgG1_G1Nn	14	65899804	1	2.96E-11	6.71E-11	-	-0.231728	-0.2444	+	G	A	0.469195167	0.0346097	+	G	A	0.487	0.0374
rs11620749	LC_IGP_R36	IgG1_G1NS1/IgG1_G1N	14	65899804	1	1.68E-08	2.69E-12	+	0.195028	0.2569	+	G	A	0.470252774	0.0343904	+	G	A	0.487	0.0367
rs11620749	LC_IGP_R32	IgG1_G1S1/IgG1_G1	14	65899804	1	1.45E-10	1.36E-10	+	0.221064	0.2327	+	G	A	0.468651376	0.034262	+	G	A	0.487	0.0362
rs11620749	LC_IGP_R3	IgG1_G2F/IgG1_G2	14	65899804	1	9.00E-15	1.82E-15	+	0.267136	0.2978	+	G	A	0.468670538	0.0341356	+	G	A	0.487	0.0374
rs11620749	LC_IGP_R8	IgG1_G2FS1/IgG1_G2S1	14	65899804	1	2.88E-09	8.10E-14	+	0.205348	0.279	+	G	A	0.468384239	0.0343875	+	G	A	0.487	0.0374
rs11620749	LC_IGP_R26	IgG1_G2N/IgG1_G1N	14	65899804	1	3.96E-12	5.05E-09	+	0.240175	0.2139	+	G	A	0.469926543	0.0343516	+	G	A	0.487	0.0366
rs11620749	LC_IGP148	IgG2 FG0n total/G0n	14	65899804	1	3.05E-08	1.93E-14	+	0.192127	0.2859	+	G	A	0.46754064	0.03452	+	G	A	0.487	0.0374
rs11620749	LC_IGP97	IgG2_G0	14	65899804	1	5.06E-10	1.15E-14	-	-0.214375	-0.2888	+	G	A	0.468670538	0.0342725	+	G	A	0.487	0.0374
rs11620749	LC_IGP138	IgG2_G0n	14	65899804	1	3.66E-10	9.23E-15	-	-0.216935	-0.2902	+	G	A	0.46754064	0.0343963	+	G	A	0.487	0.0374
rs11620749	LC_IGP_R64	IgG2_G2/IgG2_G1	14	65899804	1	3.04E-09	4.93E-13	+	0.20581	0.265	+	G	A	0.467714198	0.0345186	+	G	A	0.487	0.0367
rs11851576	LC_IGP84	IgG1 FBn/Bn total	14	65900283	1	7.10E-13	5.03E-08	+	0.252973	0.2065	+	C	T	0.532516729	0.034959	+	C	T	0.5448	0.0379
rs11851576	LC_IGP63	IgG1 FG1n total/G1n	14	65900283	1	3.55E-14	6.36E-07	+	0.266106	0.1875	+	C	T	0.532516729	0.0348052	+	C	T	0.5448	0.0377
rs11851576	LC_IGP64	IgG1 FG2n total/G2n	14	65900283	1	1.63E-11	4.95E-08	+	0.237283	0.2043	+	C	T	0.532516729	0.0349716	+	C	T	0.5448	0.0375
rs11851576	LC_IGP61	IgG1 Fn total	14	65900283	1	8.86E-14	5.83E-07	+	0.261794	0.1882	+	C	T	0.532516729	0.0348002	+	C	T	0.5448	0.0377
rs11851576	LC_IGP11	IgG1_G0	14	65900283	1	1.35E-16	1.19E-07	-	-0.2864	-0.1983	+	C	T	0.532350641	0.0342681	+	C	T	0.5448	0.0375
rs11851576	LC_IGP_R4	IgG1_G0FN/IgG1_G0N	14	65900283	1	1.92E-12	1.27E-06	+	0.245919	0.183	+	C	T	0.532734538	0.03466	+	C	T	0.5448	0.0378

rs11851576	LC_IGP52	IgG1_G0n	14	65900283	1	7.94E-17	1.51E-07	-	-0.29104	-0.1971	+	C	T	0.532516729	0.0345487 +	C	T	0.5448	0.0375
rs11851576	LC_IGP12	IgG1_G1	14	65900283	1	3.20E-13	7.44E-07	-	-0.253509	-0.1863	+	C	T	0.532045205	0.0345082 +	C	T	0.5448	0.0376
rs11851576	LC_IGP_R2	IgG1_G1F/IgG1_G1	14	65900283	1	3.19E-14	1.60E-06	+	0.264039	0.1803	+	C	T	0.532045205	0.0344745 +	C	T	0.5448	0.0376
rs11851576	LC_IGP_R5	IgG1_G1FN/IgG1_G1N	14	65900283	1	6.74E-12	1.31E-08	+	0.240607	0.2143	+	C	T	0.53379551	0.0347988 +	C	T	0.5448	0.0377
rs11851576	LC_IGP53	IgG1_G1n	14	65900283	1	5.39E-12	1.25E-06	-	-0.242344	-0.1823	+	C	T	0.532516729	0.0348846 +	C	T	0.5448	0.0376
rs11851576	LC_IGP_R3	IgG1_G2F/IgG1_G2	14	65900283	1	1.66E-12	7.68E-08	+	0.245867	0.2009	+	C	T	0.532370416	0.0345511 +	C	T	0.5448	0.0374
rs11851576	LC_IGP_R8	IgG1_G2FS1/IgG1_G2S1	14	65900283	1	2.60E-08	3.23E-07	+	0.194303	0.1904	+	C	T	0.532045205	0.0347368 +	C	T	0.5448	0.0373
rs11851576	LC_IGP148	IgG2 FG0n total/G0n	14	65900283	1	3.65E-08	1.31E-06	+	0.192771	0.18	+	C	T	0.530787254	0.0348346 +	C	T	0.5446	0.0372
rs11851576	LC_IGP97	IgG2_G0	14	65900283	1	3.39E-09	7.31E-07	-	-0.205654	-0.1848	+	C	T	0.532064792	0.0345964 +	C	T	0.5448	0.0373
rs11851576	LC_IGP138	IgG2_G0n	14	65900283	1	9.86E-10	9.37E-07	-	-0.213501	-0.1831	+	C	T	0.530787254	0.0347268 +	C	T	0.5446	0.0373
rs12879971	LC_IGP78	IgG1 BG0n/G0n	14	65901604	1	5.40E-13	3.40E-10	-	-0.249991	-0.2355	+	A	G	0.467192379	0.0343643 +	A	G	0.487	0.0375
rs12879971	LC_IGP79	IgG1 BG1n/G1n	14	65901604	1	1.12E-12	2.22E-12	-	-0.247134	-0.263	+	A	G	0.467192379	0.0344596 +	A	G	0.487	0.0375
rs12879971	LC_IGP32	IgG1 BG1S1/(BG1+BG1S1)	14	65901604	1	2.71E-08	2.42E-12	+	0.191729	0.2572	+	A	G	0.468259864	0.034317 +	A	G	0.487	0.0367
rs12879971	LC_IGP77	IgG1 Bn	14	65901604	1	2.56E-12	5.69E-12	-	-0.243264	-0.2593	+	A	G	0.467192379	0.0344838 +	A	G	0.487	0.0377
rs12879971	LC_IGP86	IgG1 Bn/Fn total	14	65901604	1	3.87E-13	3.07E-12	-	-0.252105	-0.2627	+	A	G	0.467192379	0.0344366 +	A	G	0.487	0.0377
rs12879971	LC_IGP84	IgG1 FBn/Bn total	14	65901604	1	3.86E-14	3.89E-17	+	0.263067	0.3193	+	A	G	0.467192379	0.034458 +	A	G	0.487	0.0379
rs12879971	LC_IGP62	IgG1 FG0n total/G0n	14	65901604	1	8.96E-17	6.01E-13	+	0.287102	0.2718	+	A	G	0.467192379	0.0341413 +	A	G	0.487	0.0378
rs12879971	LC_IGP63	IgG1 FG1n total/G1n	14	65901604	1	4.69E-17	3.01E-15	+	0.290536	0.2976	+	A	G	0.467192379	0.0342284 +	A	G	0.487	0.0377
rs12879971	LC_IGP64	IgG1 FG2n total/G2n	14	65901604	1	2.17E-13	5.50E-16	+	0.254899	0.3038	+	A	G	0.467192379	0.0344411 +	A	G	0.487	0.0375
rs12879971	LC_IGP68	IgG1 FG2n/G2n	14	65901604	1	4.44E-10	1.00E-08	+	0.217107	0.2141	+	A	G	0.467192379	0.0345898 +	A	G	0.487	0.0374
rs12879971	LC_IGP61	IgG1 Fn total	14	65901604	1	3.77E-16	7.31E-15	+	0.281918	0.2937	+	A	G	0.467192379	0.034248 +	A	G	0.487	0.0378
rs12879971	LC_IGP81	IgG1 Fn/Bn	14	65901604	1	1.12E-12	3.82E-11	+	0.247064	0.2478	+	A	G	0.467192379	0.0344475 +	A	G	0.487	0.0375
rs12879971	LC_IGP21	IgG1 Fucosylation	14	65901604	1	4.50E-14	8.14E-14	+	0.260734	0.2816	+	A	G	0.466440506	0.0342457 +	A	G	0.487	0.0377
rs12879971	LC_IGP28	IgG1 G1S1/(G1+G1S1)	14	65901604	1	9.21E-11	9.25E-11	+	0.222892	0.2345	+	A	G	0.466674312	0.0341743 +	A	G	0.487	0.0362
rs12879971	LC_IGP26	IgG1 GS1/(G+GS1)	14	65901604	1	1.72E-08	1.36E-10	+	0.1947	0.238	+	A	G	0.466674312	0.0343628 +	A	G	0.487	0.0371
rs12879971	LC_IGP27	IgG1 GS1/(G0+G+GS1)	14	65901604	1	6.98E-10	3.63E-09	+	0.212517	0.2174	+	A	G	0.466674312	0.0342572 +	A	G	0.487	0.0368
rs12879971	LC_IGP11	IgG1_G0	14	65901604	1	8.64E-19	3.12E-15	-	-0.302519	-0.2961	+	A	G	0.467020464	0.0337635 +	A	G	0.487	0.0376
rs12879971	LC_IGP_R1	IgG1_G0F/IgG1_G0	14	65901604	1	4.68E-16	1.65E-12	+	0.277908	0.2658	+	A	G	0.467020464	0.0338769 +	A	G	0.487	0.0376
rs12879971	LC_IGP_R4	IgG1_G0FN/IgG1_G0N	14	65901604	1	1.55E-13	6.22E-14	+	0.254459	0.2843	+	A	G	0.467557257	0.034172 +	A	G	0.487	0.0379
rs12879971	LC_IGP52	IgG1_G0n	14	65901604	1	7.97E-19	3.22E-15	-	-0.30517	-0.2965	+	A	G	0.467192379	0.0340181 +	A	G	0.487	0.0376
rs12879971	LC_IGP14	IgG1_G0N	14	65901604	1	8.00E-16	1.08E-11	-	-0.277081	-0.2541	+	A	G	0.467557257	0.0340551 +	A	G	0.487	0.0374
rs12879971	LC_IGP55	IgG1_G0Nn	14	65901604	1	3.83E-16	1.26E-11	-	-0.281801	-0.2535	+	A	G	0.467192379	0.0342423 +	A	G	0.487	0.0374
rs12879971	LC_IGP12	IgG1_G1	14	65901604	1	1.66E-15	1.95E-14	-	-0.273301	-0.2886	+	A	G	0.467020464	0.033978 +	A	G	0.487	0.0377
rs12879971	LC_IGP_R2	IgG1_G1F/IgG1_G1	14	65901604	1	6.90E-17	2.81E-14	+	0.286335	0.2863	+	A	G	0.467020464	0.0339268 +	A	G	0.487	0.0376
rs12879971	LC_IGP_R5	IgG1_G1FN/IgG1_G1N	14	65901604	1	1.40E-13	2.55E-18	+	0.25591	0.3294	+	A	G	0.468337946	0.0343049 +	A	G	0.487	0.0377
rs12879971	LC_IGP53	IgG1_G1n	14	65901604	1	1.09E-14	4.17E-14	-	-0.267728	-0.2847	+	A	G	0.467192379	0.0343157 +	A	G	0.487	0.0377
rs12879971	LC_IGP15	IgG1_G1N	14	65901604	1	7.02E-11	2.84E-11	-	-0.225915	-0.2495	+	A	G	0.468337946	0.034416 +	A	G	0.487	0.0375
rs12879971	LC_IGP56	IgG1_G1Nn	14	65901604	1	2.96E-11	7.03E-11	-	-0.231229	-0.2441	+	A	G	0.467192379	0.0345344 +	A	G	0.487	0.0374
rs12879971	LC_IGP_R36	IgG1_G1NS1/IgG1_G1N	14	65901604	1	2.22E-08	2.78E-12	+	0.192962	0.2567	+	A	G	0.468259864	0.034321 +	A	G	0.487	0.0367
rs12879971	LC_IGP_R32	IgG1_G1S1/IgG1_G1	14	65901604	1	1.70E-10	1.39E-10	+	0.219773	0.2326	+	A	G	0.466674312	0.0341919 +	A	G	0.487	0.0362
rs12879971	LC_IGP_R3	IgG1_G2F/IgG1_G2	14	65901604	1	6.78E-15	1.87E-15	+	0.267773	0.2976	+	A	G	0.466694682	0.0340567 +	A	G	0.487	0.0374
rs12879971	LC_IGP_R8	IgG1_G2FS1/IgG1_G2S1	14	65901604	1	2.79E-09	8.16E-14	+	0.205086	0.2789	+	A	G	0.466409591	0.0343135 +	A	G	0.487	0.0374
rs12879971	LC_IGP_R26	IgG1_G2N/IgG1_G1N	14	65901604	1	5.65E-12	5.27E-09	+	0.237938	0.2136	+	A	G	0.467931173	0.0342843 +	A	G	0.487	0.0366
rs12879971	LC_IGP148	IgG2 FG0n total/G0n	14	65901604	1	3.71E-08	2.03E-14	+	0.190537	0.2857	+	A	G	0.465548645	0.0344503 +	A	G	0.4869	0.0374
rs12879971	LC_IGP97	IgG2_G0	14	65901604	1	6.02E-10	1.21E-14	-	-0.21299	-0.2885	+	A	G	0.466694682	0.0342027 +	A	G	0.487	0.0374
rs12879971	LC_IGP138	IgG2_G0n	14	65901604	1	4.36E-10	9.77E-15	-	-0.215541	-0.2899	+	A	G	0.465548645	0.0343265 +	A	G	0.4869	0.0374
rs12879971	LC_IGP_R64	IgG2_G2/IgG2_G1	14	65901604	1	4.88E-09	5.16E-13	+	0.20269	0.2648	+	A	G	0.465735924	0.0344548 +	A	G	0.487	0.0367
rs72714433	LC_IGP61	IgG1 Fn total	14	65902814	1	4.59E-08	2.15E-06	-	-0.236083	-0.2437	+	G	A	0.20598759	0.0429792 +	G	A	0.1658	0.0514
rs12892058	LC_IGP78	IgG1 BG0n/G0n	14	65903441	1	2.17E-13	2.89E-10	-	-0.254728	-0.2363	+	C	T	0.462730506	0.034419 +	C	T	0.4865	0.0375
rs12892058	LC_IGP79	IgG1 BG1n/G1n	14	65903441	1	5.97E-13	1.95E-12	-	-0.25064	-0.2635	+	C	T	0.462730506	0.0345202 +	C	T	0.4865	0.0374
rs12892058	LC_IGP32	IgG1 BG1S1/(BG1+BG1S1)	14	65903441	1	2.84E-08	1.85E-12	+	0.191848	0.2584	+	C	T	0.463820306	0.0343899 +	C	T	0.4865	0.0367
rs12892058	LC_IGP77	IgG1 Bn	14	65903441	1	1.28E-12	5.00E-12	-	-0.247091	-0.2598	+	C	T	0.462730506	0.0345433 +	C	T	0.4865	0.0376
rs12892058	LC_IGP86	IgG1 Bn/Fn total	14	65903441	1	1.96E-13	2.67E-12	-	-0.255768	-0.2632	+	C	T	0.462730506	0.0344963 +	C	T	0.4865	0.0376
rs12892058	LC_IGP84	IgG1 FBn/Bn total	14	65903441	1	2.56E-14	2.97E-17	+	0.265457	0.3203	+	C	T	0.462730506	0.0345233 +	C	T	0.4865	0.0379
rs12892058	LC_IGP62	IgG1 FG0n total/G0n	14	65903441	1	4.64E-17	4.78E-13	+	0.290347	0.2728	+	C	T	0.462730506	0.0342008 +	C	T	0.4865	0.0377
rs12892058	LC_IGP63	IgG1 FG1n total/G1n	14	65903441	1	3.74E-17	2.36E-15	+	0.292064	0.2985	+	C	T	0.462730506	0.0342971 +	C	T	0.4865	0.0377
rs12892058	LC_IGP64	IgG1 FG2n total/G2n	14	65903441	1	2.88E-13	4.25E-16	+	0.254133	0.3048	+	C	T	0.462730506	0.034521 +	C	T	0.4865	0.0375
rs12892058	LC_IGP68	IgG1 FG2n/G2n	14	65903441	1	3.41E-10	8.89E-09	+	0.218985	0.2148	+	C	T	0.462730506	0.0346586 +	C	T	0.4865	0.0373
rs12892058	LC_IGP61	IgG1 Fn total	14	65903441	1	2.97E-16	5.67E-15	+	0.283497	0.2947	+	C	T	0.462730506	0.0343166 +	C	T	0.4865	0.0377
rs12892058	LC_IGP81	IgG1 Fn/Bn	14	65903441	1	5.37E-13	3.38E-11	+	0.251045	0.2483	+	C	T	0.462730506	0.034506 +	C	T	0.4865	0.0375
rs12892058	LC_IGP21	IgG1 Fucosylation	14	65903441	1	3.23E-14	6.52E-14	+	0.262768	0.2824	+	C	T	0.462000948	0.0343128 +	C	T	0.4865	0.0377
rs12892058	LC_IGP28	IgG1 G1S1/(G1+G1S1)	14	65903441	1	6.48E-11	6.23E-11	+	0.225164	0.2364	+	C	T	0.462270054	0.0342399 +	C	T	0.4865	0.0362
rs12892058	LC_IGP26	IgG1 GS1/(G+GS1)	14	65903441	1	1.99E-08	1.10E-10	+	0.194253	0.239	+	C	T	0.462270054	0.0344391 +	C	T	0.4865	0.037
rs12892058	LC_IGP27	IgG1 GS1/(G0+G+GS1)	14	65903441	1	6.38E-10	3.27E-09	+	0.213457	0.2178	+	C	T	0.462270054	0.0343283 +	C	T	0.4865	0.0368
rs12892058	LC_IGP11	IgG1_G0	14	65903441	1	2.44E-19	2.52E-15	-	-0.308044	-0.2969	+	C	T	0.46231615	0.0338326 +	C	T	0.4865	0.0375
rs12892058	LC_IGP_R1	IgG1_G0F/IgG1_G0	14	65903441	1	1.03E-16	1.32E-12	+	0.284803	0.2667	+	C	T	0.46231615	0.033941 +	C	T	0.4865	0.0376
rs12892058	LC_IGP_R4	IgG1_G0FN/IgG1_G0N	14	65903441	1	6.76E-14	4.81E-14	+	0.258887	0.2853	+	C	T	0.462841419	0.0342506 +	C	T	0.4865	0.0379
rs12892058	LC_IGP52	IgG1_G0n	14	65903441	1	4.29E-19	2.59E-15	-	-0.308139	-0.2973	+	C	T	0.462730506	0.0340783 +	C	T	0.4865	0.0376
rs12892058	LC_IGP14	IgG1_G0N	14	65903441	1	1.46E-16	9.56E-12	-	-0.2848	-0.2546	+	C	T	0.462841419	0.0341154 +	C	T	0.4865	0.0374
rs12892058	LC_IGP55	IgG1_G0Nn	14	65903441	1	1.49E-16	1.12E-11	-	-0.286273	-0.254	+	C	T	0.462730506	0.034296 +	C	T	0.4865	0.0374
rs12892058	LC_IGP12	IgG1_G1	14	65903441	1	6.27E-16	1.49E-14	-	-0.278101	-0.2897	+	C	T	0.46231615	0.0340535 +	C	T	0.4865	0.0377
rs12892058	LC_IGP_R2	IgG1_G1F/IgG1_G1	14	65903441	1	2.18E-17	2.20E-14	+	0.29169	0.2872	+	C	T	0.46231615	0.0339984 +	C	T	0.4865	0.0376
rs12892058	LC_IGP_R5	IgG1_G1FN/IgG1_G1N	14	65903441	1	1.02E-13	1.94E-18	+	0.257891	0.3303	+	C	T	0.463909309	0.0343698 +	C	T	0.4865	0.0377
rs12892058	LC_IGP53	IgG1_G1n	14	65903441	1	9.57E-15	3.21E-14	-	-0.268859	-0.2858	+	C	T	0.462730506	0.0343867 +	C	T	0.4865	0.0377
rs12892058	LC_IGP15	IgG1_G1N	14	65903441	1	4.03E-11	2.50E-11	-	-0.229222	-0.25	+	C	T	0.463909309	0.0344762 +	C	T	0.4865	0.0375
rs12892058	LC_IGP56	IgG1_G1Nn	14	65903441	1	1.59E-11	6.18E-11	-	-0.234831	-0.2447	+	C	T	0.462730506	0.0345956 +	C	T	0.4865	0.0374
rs12892058	LC_IGP_R36	IgG1_G1NS1/IgG1_G1N	14	65903441	1	2.26E-08	2.14E-12	+	0.193238	0.2578	+	C	T	0.463820306	0.0343933 +	C	T	0.4865	0.0367
rs12892058	LC_IGP_R32	IgG1_G1S1/IgG1_G1	14	65903441	1	1.18E-10	9.70E-11	+	0.222128	0.2343	+	C	T	0.462270054	0.0342573 +	C	T	0.4865	0.0362
rs12892058	LC_IGP_R3	IgG1_G2F/IgG1_G2	14	65903441	1	9.95E-15	1.43E-15	+	0.2667	0.2986	+	C	T	0.462293116	0.0341371 +	C	T	0.4865	0.0374
rs12892058	LC_IGP_R8	IgG1_G2FS1/IgG1_G2S1	14	65903441	1	3.11E-09	6.40E-14	+	0.204915	0.2799	+	C	T	0.462010713	0.0343891 +	C	T	0.4865	0.0373
rs12892058	LC_IGP_R26	IgG1_G2N/IgG1_G1N	14	65903441	1	3.13E-12	4.82E-09	+	0.241281	0.214	+	C	T	0.463485825	0.0343444 +	C	T	0.4865	0.0366
rs12892058	LC_IGP97	IgG2_G0	14	65903441	1	1.28E-09	1.15E-14	-	-0.20937	-0.2885	+	C	T	0.462293116	0.0342909 +	C	T	0.4865	0.0374
rs12892058	LC_IGP138	IgG2_G0n	14	65903441	1	9.68E-10	9.21E-15	-	-0.211706	-0.29	+	C	T	0.461114556	0.0344181 +	C	T	0.4865	0.0374
rs12892058	LC_IGP_R64	IgG2_G2/IgG2_G1	14	65903441	1	5.50E-09	5.15E-13	+	0.202454	0.2646	+	C	T	0.461328971	0.0345319 +	C	T	0.4865	0.0366
rs72714436	LC_IGP61	IgG1 Fn total	14	65907478	1	4.60E-08	2.26E-06	-	-0.236053	-0.2432	+	G	A	0.20599231	0.0429775 +	G	A	0.1656	0.0514
rs74897779	LC_IGP61	IgG1 Fn total	14	65909005	1	4.60E-08	2.26E-06	-	-0.236043	-0.2432	+	G	T	0.205993112	0.0429769 +	G	T	0.1656	0.0514
rs11158596	LC_IGP78	IgG1 BG0n/G0n	14	65911975	1	5.02E-13	4.81E-10	-	-0.250025	-0.234	+	A	T	0.468689591	0.0343215 +	A	T	0.4862	0.0376
rs11158596	LC_IGP79	IgG1 BG1n/G1n	14	65911975	1	9.75E-13	3.36E-12	-	-0.247505	-0.2615	+	A	T	0.468689591	0.0344152 +	A	T	0.4862	0.0376
rs11158596	LC_IGP32	IgG1 BG1S1/(BG1+BG1S1)	14	65911975	1	2.87E-08	3.45E-12	+	0.191146	0.2561	+	A	T	0.469751233	0.0342775 +	A	T	0.4862	0.0368
rs11158596	LC_IGP77	IgG1 Bn	14	65911975	1	2.39E-12	8.59E-12	-	-0.243311	-0.2577	+	A	T	0.468689591	0.0344409 +	A	T	0.4862	0.0377
rs11158596	LC_IGP86	IgG1 Bn/Fn total	14	65911975	1	3.53E-13	4.60E-12	-	-0.252227	-0.2612	+	A	T	0.468689591	0.0343933 +	A	T	0.4862	0.0378
rs11158596	LC_IGP84	IgG1 FBn/Bn total	14	65911975	1	3.43E-14	5.95E-17	+	0.263273	0.3183	+	A	T	0.468689591	0.0344142 +	A	T	0.4862	0.038
rs11158596	LC_IGP62	IgG1 FG0n total/G0n	14	65911975	1	7.69E-17	8.80E-13	+	0.287366	0.2706	+	A	T	0.468689591	0.0340971 +	A	T	0.4862	0.0379
rs11158596	LC_IGP63	IgG1 FG1n total/G1n	14	65911975	1	3.95E-17	4.53E-15	+	0.290871	0.2965	+	A	T	0.468689591	0.0341837 +	A	T	0.4862	0.0378
rs11158596	LC_IGP64	IgG1 FG2n total/G2n	14	65911975	1	1.96E-13	7.83E-16	+	0.255058	0.303	+	A	T	0.468689591	0.0343976 +	A	T	0.4862	0.0376
rs11158596	LC_IGP68	IgG1 FG2n/G2n	14	65911975	1	3.99E-10	1.34E-08	+	0.217421	0.2128	+	A	T	0.468689591	0.034546 +	A	T	0.4862	0.0375
rs11158596	LC_IGP61	IgG1 Fn total	14	65911975	1	3.24E-16	1.10E-14	+	0.282197	0.2925	+	A	T	0.468689591	0.0342037 +	A	T	0.4862	0.0379
rs11158596	LC_IGP81	IgG1 Fn/Bn	14	65911975	1	1.05E-12	5.76E-11	+	0.24709	0.2462	+	A	T	0.468689591	0.0344046 +	A	T	0.4862	0.0376
rs11158596	LC_IGP21	IgG1 Fucosylation	14	65911975	1	4.02E-14	1.19E-13	+	0.260914	0.2805	+	A	T	0.467930025	0.0342022 +	A	T	0.4862	0.0378
rs11158596	LC_IGP28	IgG1 G1S1/(G1+G1S1)	14	65911975	1	9.93E-11	1.25E-10	+	0.222241	0.2334	+	A	T	0.468153823	0.0341349 +	A	T	0.4862	0.0363
rs11158596	LC_IGP26	IgG1 GS1/(G+GS1)	14	65911975	1	2.17E-08	1.60E-10	+	0.193113	0.2378	+	A	T	0.468153823	0.0343264 +	A	T	0.4862	0.0372
rs11158596	LC_IGP27	IgG1 GS1/(G0+G+GS1)	14	65911975	1	8.26E-10	4.04E-09	+	0.211352	0.2173	+	A	T	0.468153823	0.0342196 +	A	T	0.4862	0.0369
rs11158596	LC_IGP11	IgG1_G0	14	65911975	1	7.58E-19	4.36E-15	-	-0.302645	-0.2954	+	A	T	0.468498167	0.0337206 +	A	T	0.4862	0.0377
rs11158596	LC_IGP_R1	IgG1_G0F/IgG1_G0	14	65911975	1	3.94E-16	2.39E-12	+	0.278271	0.2646	+	A	T	0.468498167	0.033833 +	A	T	0.4862	0.0377
rs11158596	LC_IGP_R4	IgG1_G0FN/IgG1_G0N	14	65911975	1	1.42E-13	9.11E-14	+	0.254551	0.2831	+	A	T	0.469038273	0.0341295 +	A	T	0.4862	0.038
rs11158596	LC_IGP52	IgG1_G0n	14	65911975	1	6.93E-19	4.58E-15	-	-0.305327	-0.2958	+	A	T	0.468689591	0.0339744 +	A	T	0.4862	0.0377
rs11158596	LC_IGP14	IgG1_G0N	14	65911975	1	7.19E-16	1.51E-11	-	-0.277191	-0.253	+	A	T	0.469038273	0.0340124 +	A	T	0.4862	0.0375
rs11158596	LC_IGP55	IgG1_G0Nn	14	65911975	1	3.50E-16	1.79E-11	-	-0.281826	-0.2523	+	A	T	0.468689591	0.0341993 +	A	T	0.4862	0.0375
rs11158596	LC_IGP12	IgG1_G1	14	65911975	1	1.50E-15	2.88E-14	-	-0.273393	-0.2875	+	A	T	0.468498167	0.0339355 +	A	T	0.4862	0.0378
rs11158596	LC_IGP_R2	IgG1_G1F/IgG1_G1	14	65911975	1	6.07E-17	4.21E-14	+	0.286503	0.2851	+	A	T	0.468498167	0.0338837 +	A	T	0.4862	0.0377
rs11158596	LC_IGP_R5	IgG1_G1FN/IgG1_G1N	14	65911975	1	1.13E-13	3.86E-18	+	0.256581	0.3285	+	A	T	0.469825646	0.0342598 +	A	T	0.4862	0.0378
rs11158596	LC_IGP53	IgG1_G1n	14	65911975	1	9.83E-15	6.22E-14	-	-0.267844	-0.2835	+	A	T	0.468689591	0.0342724 +	A	T	0.4862	0.0378
rs11158596	LC_IGP15	IgG1_G1N	14	65911975	1	6.32E-11	4.21E-11	-	-0.226185	-0.2479	+	A	T	0.469825646	0.0343731 +	A	T	0.4862	0.0376
rs11158596	LC_IGP56	IgG1_G1Nn	14	65911975	1	2.64E-11	1.05E-10	-	-0.231524	-0.2425	+	A	T	0.468689591	0.0344906 +	A	T	0.4862	0.0375
rs11158596	LC_IGP_R36	IgG1_G1NS1/IgG1_G1N	14	65911975	1	2.37E-08	3.95E-12	+	0.192333	0.2556	+	A	T	0.469751233	0.0342816 +	A	T	0.4862	0.0368
rs11158596	LC_IGP_R32	IgG1_G1S1/IgG1_G1	14	65911975	1	1.84E-10	1.87E-10	+	0.219079	0.2316	+	A	T	0.468153823	0.0341527 +	A	T	0.4862	0.0363
rs11158596	LC_IGP_R3	IgG1_G2F/IgG1_G2	14	65911975	1	5.60E-15	2.64E-15	+	0.268267	0.2968	+	A	T	0.468173289	0.034012 +	A	T	0.4862	0.0375
rs11158596	LC_IGP_R8	IgG1_G2FS1/IgG1_G2S1	14	65911975	1	2.14E-09	1.07E-13	+	0.206316	0.2783	+	A	T	0.467887294	0.0342669 +	A	T	0.4862	0.0374
rs11158596	LC_IGP_R26	IgG1_G2N/IgG1_G1N	14	65911975	1	4.17E-12	7.08E-09	+	0.239116	0.2124	+	A	T	0.46942284	0.0342374 +	A	T	0.4862	0.0367

rs11158596	LC_IGP148	IgG2 FG0n total/G0n	14	65911975	1	1.97E-08	2.97E-14	+	0.194094	0.2847	+	A	T	0.467036638	0.0343954	+	A	T	0.4862	0.0375
rs11158596	LC_IGP97	IgG2_G0	14	65911975	1	3.68E-10	1.71E-14	-	-0.215361	-0.2878	+	A	T	0.468173289	0.0341517	+	A	T	0.4862	0.0375
rs11158596	LC_IGP138	IgG2_G0n	14	65911975	1	2.53E-10	1.39E-14	-	-0.21817	-0.2892	+	A	T	0.467036638	0.0342735	+	A	T	0.4862	0.0376
rs11158596	LC_IGP_R64	IgG2_G2/IgG2_G1	14	65911975	1	2.42E-09	7.03E-13	+	0.206397	0.2641	+	A	T	0.46721634	0.0343987	+	A	T	0.4862	0.0368
rs72714439	LC_IGP61	IgG1 Fn total	14	65916969	1	4.62E-08	2.16E-06	-	-0.235998	-0.2433	+	T	C	0.205999799	0.0429741	+	T	C	0.1659	0.0514
rs72714440	LC_IGP61	IgG1 Fn total	14	65918591	1	4.62E-08	2.15E-06	-	-0.235993	-0.2433	+	T	C	0.205999617	0.0429741	+	T	C	0.1659	0.0514
rs72714442	LC_IGP61	IgG1 Fn total	14	65919046	1	4.62E-08	2.15E-06	-	-0.235993	-0.2433	+	C	T	0.205999617	0.0429741	+	C	T	0.1659	0.0514
rs12589698	LC_IGP78	IgG1 BG0n/G0n	14	65920435	1	2.28E-13	4.12E-10	-	-0.254394	-0.2348	+	A	G	0.462803594	0.0344052	+	A	G	0.4858	0.0376
rs12589698	LC_IGP79	IgG1 BG1n/G1n	14	65920435	1	6.44E-13	2.97E-12	-	-0.25018	-0.262	+	A	G	0.462803594	0.034507	+	A	G	0.4858	0.0375
rs12589698	LC_IGP32	IgG1 BG1S1/(BG1+BG1S1)	14	65920435	1	2.95E-08	2.61E-12	+	0.191521	0.2573	+	A	G	0.463894883	0.0343759	+	A	G	0.4858	0.0368
rs12589698	LC_IGP77	IgG1 Bn	14	65920435	1	1.36E-12	7.60E-12	-	-0.246711	-0.2582	+	A	G	0.462803594	0.0345297	+	A	G	0.4858	0.0377
rs12589698	LC_IGP86	IgG1 Bn/Fn total	14	65920435	1	2.09E-13	4.04E-12	-	-0.255383	-0.2617	+	A	G	0.462803594	0.0344828	+	A	G	0.4858	0.0377
rs12589698	LC_IGP84	IgG1 FBn/Bn total	14	65920435	1	2.68E-14	4.58E-17	+	0.265147	0.3192	+	A	G	0.462803594	0.0345095	+	A	G	0.4858	0.038
rs12589698	LC_IGP62	IgG1 FG0n total/G0n	14	65920435	1	4.85E-17	7.02E-13	+	0.290049	0.2716	+	A	G	0.462803594	0.0341871	+	A	G	0.4858	0.0378
rs12589698	LC_IGP63	IgG1 FG1n total/G1n	14	65920435	1	4.01E-17	3.54E-15	+	0.291654	0.2974	+	A	G	0.462803594	0.0342839	+	A	G	0.4858	0.0378
rs12589698	LC_IGP64	IgG1 FG2n total/G2n	14	65920435	1	3.04E-13	6.00E-16	+	0.253782	0.304	+	A	G	0.462803594	0.0345074	+	A	G	0.4858	0.0376
rs12589698	LC_IGP68	IgG1 FG2n/G2n	14	65920435	1	3.61E-10	1.18E-08	+	0.218585	0.2135	+	A	G	0.462803594	0.0346449	+	A	G	0.4858	0.0374
rs12589698	LC_IGP61	IgG1 Fn total	14	65920435	1	3.18E-16	8.53E-15	+	0.2831	0.2935	+	A	G	0.462803594	0.0343033	+	A	G	0.4858	0.0378
rs12589698	LC_IGP81	IgG1 Fn/Bn	14	65920435	1	5.72E-13	5.12E-11	+	0.250643	0.2466	+	A	G	0.462803594	0.0344925	+	A	G	0.4858	0.0376
rs12589698	LC_IGP21	IgG1 Fucosylation	14	65920435	1	3.42E-14	9.62E-14	+	0.262394	0.2813	+	A	G	0.462073058	0.0342995	+	A	G	0.4858	0.0378
rs12589698	LC_IGP28	IgG1 G1S1/(G1+G1S1)	14	65920435	1	6.80E-11	8.47E-11	+	0.224827	0.2354	+	A	G	0.462344343	0.034226	+	A	G	0.4858	0.0363
rs12589698	LC_IGP26	IgG1 GS1/(G+GS1)	14	65920435	1	2.00E-08	1.33E-10	+	0.19416	0.2386	+	A	G	0.462344343	0.0344242	+	A	G	0.4858	0.0371
rs12589698	LC_IGP27	IgG1 GS1/(G0+G+GS1)	14	65920435	1	6.31E-10	3.76E-09	+	0.21342	0.2175	+	A	G	0.462344343	0.0343133	+	A	G	0.4858	0.0369
rs12589698	LC_IGP11	IgG1_G0	14	65920435	1	2.43E-19	3.57E-15	-	-0.307936	-0.2961	+	A	G	0.462390348	0.0338178	+	A	G	0.4858	0.0376
rs12589698	LC_IGP_R1	IgG1_G0F/IgG1_G0	14	65920435	1	1.07E-16	1.91E-12	+	0.284535	0.2655	+	A	G	0.462390348	0.033927	+	A	G	0.4858	0.0377
rs12589698	LC_IGP_R4	IgG1_G0FN/IgG1_G0N	14	65920435	1	7.01E-14	7.10E-14	+	0.258614	0.2842	+	A	G	0.462915187	0.0342366	+	A	G	0.4858	0.038
rs12589698	LC_IGP52	IgG1_G0n	14	65920435	1	4.30E-19	3.73E-15	-	-0.308004	-0.2965	+	A	G	0.462803594	0.0340637	+	A	G	0.4858	0.0377
rs12589698	LC_IGP14	IgG1_G0N	14	65920435	1	1.48E-16	1.37E-11	-	-0.284617	-0.2533	+	A	G	0.462915187	0.034101	+	A	G	0.4858	0.0375
rs12589698	LC_IGP55	IgG1_G0Nn	14	65920435	1	1.51E-16	1.62E-11	-	-0.286075	-0.2526	+	A	G	0.462803594	0.0342817	+	A	G	0.4858	0.0375
rs12589698	LC_IGP12	IgG1_G1	14	65920435	1	6.60E-16	2.18E-14	-	-0.27777	-0.2886	+	A	G	0.462390348	0.0340398	+	A	G	0.4858	0.0378
rs12589698	LC_IGP_R2	IgG1_G1F/IgG1_G1	14	65920435	1	2.34E-17	3.28E-14	+	0.291277	0.2861	+	A	G	0.462390348	0.0339852	+	A	G	0.4858	0.0377
rs12589698	LC_IGP_R5	IgG1_G1FN/IgG1_G1N	14	65920435	1	1.07E-13	2.95E-18	+	0.257552	0.3294	+	A	G	0.463983702	0.0343561	+	A	G	0.4858	0.0378
rs12589698	LC_IGP53	IgG1_G1n	14	65920435	1	1.02E-14	4.74E-14	-	-0.268486	-0.2846	+	A	G	0.462803594	0.0343732	+	A	G	0.4858	0.0378
rs12589698	LC_IGP15	IgG1_G1N	14	65920435	1	4.29E-11	3.70E-11	-	-0.228799	-0.2485	+	A	G	0.463983702	0.0344627	+	A	G	0.4858	0.0376
rs12589698	LC_IGP56	IgG1_G1Nn	14	65920435	1	1.71E-11	9.16E-11	-	-0.234393	-0.243	+	A	G	0.462803594	0.0345822	+	A	G	0.4858	0.0375
rs12589698	LC_IGP_R36	IgG1_G1NS1/IgG1_G1N	14	65920435	1	2.36E-08	3.02E-12	+	0.192915	0.2568	+	A	G	0.463894883	0.0343793	+	A	G	0.4858	0.0368
rs12589698	LC_IGP_R32	IgG1_G1S1/IgG1_G1	14	65920435	1	1.23E-10	1.31E-10	+	0.221796	0.2333	+	A	G	0.462344343	0.0342434	+	A	G	0.4858	0.0363
rs12589698	LC_IGP_R3	IgG1_G2F/IgG1_G2	14	65920435	1	1.06E-14	1.99E-15	+	0.266319	0.2979	+	A	G	0.462367359	0.0341236	+	A	G	0.4858	0.0375
rs12589698	LC_IGP_R8	IgG1_G2FS1/IgG1_G2S1	14	65920435	1	3.21E-09	8.33E-14	+	0.204644	0.2793	+	A	G	0.462084911	0.0343748	+	A	G	0.4858	0.0374
rs12589698	LC_IGP_R26	IgG1_G2N/IgG1_G1N	14	65920435	1	3.26E-12	6.54E-09	+	0.240991	0.2127	+	A	G	0.463558642	0.0343306	+	A	G	0.4858	0.0366
rs12589698	LC_IGP97	IgG2_G0	14	65920435	1	1.28E-09	1.62E-14	-	-0.209279	-0.2878	+	A	G	0.462367359	0.0342761	+	A	G	0.4858	0.0375
rs12589698	LC_IGP138	IgG2_G0n	14	65920435	1	9.70E-10	1.30E-14	-	-0.211605	-0.2893	+	A	G	0.461186884	0.0344035	+	A	G	0.4857	0.0375
rs12589698	LC_IGP_R64	IgG2_G2/IgG2_G1	14	65920435	1	5.48E-09	7.00E-13	+	0.202388	0.264	+	A	G	0.461403305	0.0345168	+	A	G	0.4858	0.0368
rs12883815	LC_IGP78	IgG1 BG0n/G0n	14	65923367	1	2.29E-13	3.72E-10	-	-0.254376	-0.2354	+	T	C	0.462807311	0.0344048	+	T	C	0.4861	0.0376
rs12883815	LC_IGP79	IgG1 BG1n/G1n	14	65923367	1	6.46E-13	2.71E-12	-	-0.250161	-0.2626	+	T	C	0.462807311	0.0345066	+	T	C	0.4861	0.0376
rs12883815	LC_IGP32	IgG1 BG1S1/(BG1+BG1S1)	14	65923367	1	2.96E-08	2.39E-12	+	0.191508	0.2579	+	T	C	0.463898582	0.0343754	+	T	C	0.4861	0.0368
rs12883815	LC_IGP77	IgG1 Bn	14	65923367	1	1.36E-12	6.86E-12	-	-0.246693	-0.2588	+	T	C	0.462807311	0.0345293	+	T	C	0.4861	0.0377
rs12883815	LC_IGP86	IgG1 Bn/Fn total	14	65923367	1	2.09E-13	3.64E-12	-	-0.255364	-0.2624	+	T	C	0.462807311	0.0344824	+	T	C	0.4861	0.0378
rs12883815	LC_IGP84	IgG1 FBn/Bn total	14	65923367	1	2.68E-14	4.07E-17	+	0.265129	0.3198	+	T	C	0.462807311	0.0345091	+	T	C	0.4861	0.038
rs12883815	LC_IGP62	IgG1 FG0n total/G0n	14	65923367	1	4.86E-17	6.23E-13	+	0.290031	0.2722	+	T	C	0.462807311	0.0341867	+	T	C	0.4861	0.0378
rs12883815	LC_IGP63	IgG1 FG1n total/G1n	14	65923367	1	4.03E-17	3.14E-15	+	0.291637	0.2981	+	T	C	0.462807311	0.0342835	+	T	C	0.4861	0.0378
rs12883815	LC_IGP64	IgG1 FG2n total/G2n	14	65923367	1	3.05E-13	5.35E-16	+	0.253767	0.3047	+	T	C	0.462807311	0.0345069	+	T	C	0.4861	0.0376
rs12883815	LC_IGP68	IgG1 FG2n/G2n	14	65923367	1	3.62E-10	1.11E-08	+	0.218572	0.214	+	T	C	0.462807311	0.0346445	+	T	C	0.4861	0.0375
rs12883815	LC_IGP61	IgG1 Fn total	14	65923367	1	3.19E-16	7.46E-15	+	0.283081	0.2943	+	T	C	0.462807311	0.0343029	+	T	C	0.4861	0.0378
rs12883815	LC_IGP81	IgG1 Fn/Bn	14	65923367	1	5.74E-13	4.62E-11	+	0.250624	0.2473	+	T	C	0.462807311	0.0344921	+	T	C	0.4861	0.0376
rs12883815	LC_IGP21	IgG1 Fucosylation	14	65923367	1	3.44E-14	8.15E-14	+	0.262377	0.2821	+	T	C	0.462076449	0.0342991	+	T	C	0.4861	0.0378
rs12883815	LC_IGP28	IgG1 G1S1/(G1+G1S1)	14	65923367	1	6.81E-11	7.35E-11	+	0.224816	0.2362	+	T	C	0.462348012	0.0342256	+	T	C	0.4861	0.0363
rs12883815	LC_IGP26	IgG1 GS1/(G+GS1)	14	65923367	1	2.00E-08	1.33E-10	+	0.194158	0.2387	+	T	C	0.462348012	0.0344237	+	T	C	0.4861	0.0372
rs12883815	LC_IGP27	IgG1 GS1/(G0+G+GS1)	14	65923367	1	6.31E-10	3.85E-09	+	0.21342	0.2174	+	T	C	0.462348012	0.0343128	+	T	C	0.4861	0.0369
rs12883815	LC_IGP11	IgG1_G0	14	65923367	1	2.43E-19	3.38E-15	-	-0.307927	-0.2965	+	T	C	0.462394013	0.0338173	+	T	C	0.4861	0.0376
rs12883815	LC_IGP_R1	IgG1_G0F/IgG1_G0	14	65923367	1	1.07E-16	1.68E-12	+	0.284519	0.2662	+	T	C	0.462394013	0.0339267	+	T	C	0.4861	0.0377
rs12883815	LC_IGP_R4	IgG1_G0FN/IgG1_G0N	14	65923367	1	7.03E-14	6.54E-14	+	0.258598	0.2847	+	T	C	0.462918861	0.0342362	+	T	C	0.4861	0.038
rs12883815	LC_IGP52	IgG1_G0n	14	65923367	1	4.30E-19	3.47E-15	-	-0.307994	-0.2969	+	T	C	0.462807311	0.0340632	+	T	C	0.4861	0.0377
rs12883815	LC_IGP14	IgG1_G0N	14	65923367	1	1.49E-16	1.30E-11	-	-0.284606	-0.2536	+	T	C	0.462918861	0.0341006	+	T	C	0.4861	0.0375
rs12883815	LC_IGP55	IgG1_G0Nn	14	65923367	1	1.52E-16	1.52E-11	-	-0.286063	-0.2531	+	T	C	0.462807311	0.0342812	+	T	C	0.4861	0.0375
rs12883815	LC_IGP12	IgG1_G1	14	65923367	1	6.62E-16	1.94E-14	-	-0.277756	-0.2893	+	T	C	0.462394013	0.0340394	+	T	C	0.4861	0.0378
rs12883815	LC_IGP_R2	IgG1_G1F/IgG1_G1	14	65923367	1	2.35E-17	2.90E-14	+	0.291263	0.2868	+	T	C	0.462394013	0.0339848	+	T	C	0.4861	0.0377
rs12883815	LC_IGP_R5	IgG1_G1FN/IgG1_G1N	14	65923367	1	1.07E-13	2.60E-18	+	0.257535	0.3301	+	T	C	0.463987392	0.0343557	+	T	C	0.4861	0.0378
rs12883815	LC_IGP53	IgG1_G1n	14	65923367	1	1.02E-14	4.13E-14	-	-0.268471	-0.2854	+	T	C	0.462807311	0.0343728	+	T	C	0.4861	0.0378
rs12883815	LC_IGP15	IgG1_G1N	14	65923367	1	4.30E-11	3.38E-11	-	-0.228781	-0.2491	+	T	C	0.463987392	0.0344623	+	T	C	0.4861	0.0376
rs12883815	LC_IGP56	IgG1_G1Nn	14	65923367	1	1.71E-11	8.28E-11	-	-0.234374	-0.2437	+	T	C	0.462807311	0.0345818	+	T	C	0.4861	0.0375
rs12883815	LC_IGP_R36	IgG1_G1NS1/IgG1_G1N	14	65923367	1	2.36E-08	2.77E-12	+	0.192902	0.2574	+	T	C	0.463898582	0.0343789	+	T	C	0.4861	0.0368
rs12883815	LC_IGP_R32	IgG1_G1S1/IgG1_G1	14	65923367	1	1.24E-10	1.14E-10	+	0.221785	0.2342	+	T	C	0.462348012	0.034243	+	T	C	0.4861	0.0363
rs12883815	LC_IGP_R3	IgG1_G2F/IgG1_G2	14	65923367	1	1.06E-14	1.77E-15	+	0.266304	0.2986	+	T	C	0.462371027	0.0341232	+	T	C	0.4861	0.0375
rs12883815	LC_IGP_R8	IgG1_G2FS1/IgG1_G2S1	14	65923367	1	3.21E-09	7.48E-14	+	0.204632	0.2799	+	T	C	0.462088577	0.0343744	+	T	C	0.4861	0.0374
rs12883815	LC_IGP_R26	IgG1_G2N/IgG1_G1N	14	65923367	1	3.26E-12	6.42E-09	+	0.240982	0.2129	+	T	C	0.463562346	0.0343302	+	T	C	0.4861	0.0367
rs12883815	LC_IGP97	IgG2_G0	14	65923367	1	1.28E-09	1.54E-14	-	-0.209269	-0.2882	+	T	C	0.462371027	0.0342756	+	T	C	0.4861	0.0375
rs12883815	LC_IGP138	IgG2_G0n	14	65923367	1	9.71E-10	1.22E-14	-	-0.211594	-0.2897	+	T	C	0.461190579	0.034403	+	T	C	0.4861	0.0376
rs12883815	LC_IGP_R64	IgG2_G2/IgG2_G1	14	65923367	1	5.48E-09	7.07E-13	+	0.202386	0.2641	+	T	C	0.461406977	0.0345163	+	T	C	0.4861	0.0368
rs4899179	LC_IGP78	IgG1 BG0n/G0n	14	65926748	1	5.38E-13	5.12E-10	-	-0.249787	-0.2337	+	G	T	0.46775062	0.0343342	+	G	T	0.4865	0.0376
rs4899179	LC_IGP79	IgG1 BG1n/G1n	14	65926748	1	1.29E-12	3.54E-12	-	-0.246278	-0.2613	+	G	T	0.46775062	0.0344323	+	G	T	0.4865	0.0376
rs4899179	LC_IGP32	IgG1 BG1S1/(BG1+BG1S1)	14	65926748	1	3.12E-08	3.45E-12	+	0.190712	0.2561	+	G	T	0.468817509	0.0342901	+	G	T	0.4865	0.0368
rs4899179	LC_IGP77	IgG1 Bn	14	65926748	1	2.82E-12	9.14E-12	-	-0.242587	-0.2575	+	G	T	0.46775062	0.0344557	+	G	T	0.4865	0.0378
rs4899179	LC_IGP86	IgG1 Bn/Fn total	14	65926748	1	4.28E-13	4.90E-12	-	-0.25142	-0.261	+	G	T	0.46775062	0.0344087	+	G	T	0.4865	0.0378
rs4899179	LC_IGP84	IgG1 FBn/Bn total	14	65926748	1	4.05E-14	6.43E-17	+	0.262629	0.318	+	G	T	0.46775062	0.034429	+	G	T	0.4865	0.038
rs4899179	LC_IGP62	IgG1 FG0n total/G0n	14	65926748	1	9.79E-17	9.51E-13	+	0.286495	0.2703	+	G	T	0.46775062	0.0341134	+	G	T	0.4865	0.0379
rs4899179	LC_IGP63	IgG1 FG1n total/G1n	14	65926748	1	5.32E-17	4.83E-15	+	0.289784	0.2963	+	G	T	0.46775062	0.0342012	+	G	T	0.4865	0.0378
rs4899179	LC_IGP64	IgG1 FG2n total/G2n	14	65926748	1	2.85E-13	8.29E-16	+	0.253424	0.3029	+	G	T	0.46775062	0.0344168	+	G	T	0.4865	0.0376
rs4899179	LC_IGP68	IgG1 FG2n/G2n	14	65926748	1	5.83E-10	1.39E-08	+	0.215443	0.2127	+	G	T	0.46775062	0.0345654	+	G	T	0.4865	0.0375
rs4899179	LC_IGP61	IgG1 Fn total	14	65926748	1	4.31E-16	1.18E-14	+	0.281122	0.2923	+	G	T	0.46775062	0.0342209	+	G	T	0.4865	0.0379
rs4899179	LC_IGP81	IgG1 Fn/Bn	14	65926748	1	1.25E-12	6.09E-11	+	0.246338	0.2459	+	G	T	0.46775062	0.0344197	+	G	T	0.4865	0.0376
rs4899179	LC_IGP21	IgG1 Fucosylation	14	65926748	1	4.65E-14	1.28E-13	+	0.260365	0.2802	+	G	T	0.466995376	0.0342167	+	G	T	0.4865	0.0378
rs4899179	LC_IGP28	IgG1 G1S1/(G1+G1S1)	14	65926748	1	1.12E-10	1.26E-10	+	0.221698	0.2335	+	G	T	0.467227829	0.0341484	+	G	T	0.4865	0.0363
rs4899179	LC_IGP26	IgG1 GS1/(G+GS1)	14	65926748	1	2.20E-08	1.80E-10	+	0.193088	0.2371	+	G	T	0.467227829	0.0343378	+	G	T	0.4865	0.0372
rs4899179	LC_IGP27	IgG1 GS1/(G0+G+GS1)	14	65926748	1	8.74E-10	4.54E-09	+	0.211115	0.2166	+	G	T	0.467227829	0.0342319	+	G	T	0.4865	0.0369
rs4899179	LC_IGP11	IgG1_G0	14	65926748	1	1.02E-18	4.90E-15	-	-0.301636	-0.295	+	G	T	0.467573305	0.0337375	+	G	T	0.4865	0.0377
rs4899179	LC_IGP_R1	IgG1_G0F/IgG1_G0	14	65926748	1	5.23E-16	2.57E-12	+	0.277212	0.2643	+	G	T	0.467573305	0.0338497	+	G	T	0.4865	0.0378
rs4899179	LC_IGP_R4	IgG1_G0FN/IgG1_G0N	14	65926748	1	1.51E-13	9.93E-14	+	0.254343	0.2828	+	G	T	0.468110839	0.0341418	+	G	T	0.4865	0.038
rs4899179	LC_IGP52	IgG1_G0n	14	65926748	1	9.36E-19	5.11E-15	-	-0.304304	-0.2953	+	G	T	0.46775062	0.0339918	+	G	T	0.4865	0.0377
rs4899179	LC_IGP14	IgG1_G0N	14	65926748	1	8.02E-16	1.65E-11	-	-0.276827	-0.2525	+	G	T	0.468110839	0.0340255	+	G	T	0.4865	0.0375
rs4899179	LC_IGP55	IgG1_G0Nn	14	65926748	1	3.84E-16	1.94E-11	-	-0.281544	-0.2519	+	G	T	0.46775062	0.0342125	+	G	T	0.4865	0.0375
rs4899179	LC_IGP12	IgG1_G1	14	65926748	1	1.83E-15	3.08E-14	-	-0.27267	-0.2873	+	G	T	0.467573305	0.0339504	+	G	T	0.4865	0.0378
rs4899179	LC_IGP_R2	IgG1_G1F/IgG1_G1	14	65926748	1	7.93E-17	4.47E-14	+	0.285534	0.2849	+	G	T	0.467573305	0.0339001	+	G	T	0.4865	0.0378
rs4899179	LC_IGP_R5	IgG1_G1FN/IgG1_G1N	14	65926748	1	1.48E-13	4.10E-18	+	0.255435	0.3283	+	G	T	0.468894219	0.0342763	+	G	T	0.4865	0.0378
rs4899179	LC_IGP53	IgG1_G1n	14	65926748	1	1.20E-14	6.59E-14	-	-0.267068	-0.2833	+	G	T	0.46775062	0.0342879	+	G	T	0.4865	0.0378
rs4899179	LC_IGP15	IgG1_G1N	14	65926748	1	7.97E-11	4.45E-11	-	-0.225064	-0.2477	+	G	T	0.468894219	0.0343887	+	G	T	0.4865	0.0376
rs4899179	LC_IGP56	IgG1_G1Nn	14	65926748	1	3.35E-11	1.09E-10	-	-0.2304	-0.2423	+	G	T	0.46775062	0.0345069	+	G	T	0.4865	0.0375
rs4899179	LC_IGP_R36	IgG1_G1NS1/IgG1_G1N	14	65926748	1	2.57E-08	3.96E-12	+	0.191911	0.2556	+	G	T	0.468817509	0.0342942	+	G	T	0.4865	0.0368
rs4899179	LC_IGP_R32	IgG1_G1S1/IgG1_G1	14	65926748	1	2.08E-10	1.88E-10	+	0.218519	0.2316	+	G	T	0.467227829	0.0341662	+	G	T	0.4865	0.0364
rs4899179	LC_IGP_R3	IgG1_G2F/IgG1_G2	14	65926748	1	8.92E-15	2.78E-15	+	0.26637	0.2967	+	G	T	0.467247861	0.0340326	+	G	T	0.4865	0.0376
rs4899179	LC_IGP_R8	IgG1_G2FS1/IgG1_G2S1	14	65926748	1	2.69E-09	1.13E-13	+	0.205104	0.2781	+	G	T	0.466962431	0.0342828	+	G	T	0.4865	0.0375
rs4899179	LC_IGP_R26	IgG1_G2N/IgG1_G1N	14	65926748	1	5.43E-12	7.50E-09	+	0.237926	0.212	+	G	T	0.468487346	0.0342539	+	G	T	0.4865	0.0367
rs4899179	LC_IGP148	IgG2 FG0n total/G0n	14	65926748	1	3.00E-08	3.18E-14	+	0.191649	0.2844	+	G	T	0.466103448	0.0344157	+	G	T	0.4865	0.0375
rs4899179	LC_IGP97	IgG2_G0	14	65926748	1	5.03E-10	1.85E-14	-	-0.213763	-0.2874	+	G	T	0.467247861	0.0341692	+	G	T	0.4865	0.0375
rs4899179	LC_IGP138	IgG2_G0n	14	65926748	1	3.58E-10	1.50E-14	-	-0.216406	-0.2889	+	G	T	0.466103448	0.0342923	+	G	T	0.4865	0.0376
rs4899179	LC_IGP_R64	IgG2_G2/IgG2_G1	14	65926748	1	3.98E-09	7.42E-13	+	0.203669	0.2639	+	G	T	0.46628978	0.0344203	+	G	T	0.4865	0.0368
rs2184603	LC_IGP78	IgG1 BG0n/G0n	14	65930670	1	1.38E-14	5.10E-10	-	-0.266005	-0.233	+	C	A	0.47692596	0.0342311	+	C	A	0.4924	0.0375
rs2184603	LC_IGP79	IgG1 BG1n/G1n	14	65930670	1	7.76E-14	6.60E-12	-	-0.259002	-0.2571	+	C	A	0.47692596	0.0343468	+	C	A	0.4924	0.0374
rs2184603	LC_IGP32	IgG1 BG1S1/(BG1+BG1S1)	14	65930670	1	5.51E-09	2.82E-12	+	0.200677	0.2562	+	C	A	0.477947596	0.0342307	+	C	A	0.4924	0.0367

rs2184603	LC_IGP31	IgG1 BGS1/(BG0+BG+BGS1)	14	65930670	1	2.27E-08	5.63E-12	+	0.193179	0.2553	+	C	A	0.47803086	0.0343818	+	C	A	0.4924	0.0371
rs2184603	LC_IGP77	IgG1 Bn	14	65930670	1	1.06E-13	1.41E-11	-	-0.257635	-0.2543	+	C	A	0.47692596	0.0343603	+	C	A	0.4924	0.0376
rs2184603	LC_IGP86	IgG1 Bn/Fn total	14	65930670	1	1.46E-14	8.03E-12	-	-0.266358	-0.2575	+	C	A	0.47692596	0.0343113	+	C	A	0.4924	0.0377
rs2184603	LC_IGP84	IgG1 FBn/Bn total	14	65930670	1	9.06E-16	1.28E-16	+	0.278752	0.3139	+	C	A	0.47692596	0.0343225	+	C	A	0.4924	0.0379
rs2184603	LC_IGP62	IgG1 FG0n total/G0n	14	65930670	1	4.73E-18	1.42E-12	+	0.298147	0.2673	+	C	A	0.47692596	0.0340238	+	C	A	0.4924	0.0378
rs2184603	LC_IGP63	IgG1 FG1n total/G1n	14	65930670	1	3.16E-18	1.05E-14	+	0.300573	0.2916	+	C	A	0.47692596	0.0341156	+	C	A	0.4924	0.0377
rs2184603	LC_IGP64	IgG1 FG2n total/G2n	14	65930670	1	2.27E-14	1.78E-15	+	0.264568	0.2984	+	C	A	0.47692596	0.0343371	+	C	A	0.4924	0.0375
rs2184603	LC_IGP68	IgG1 FG2n/G2n	14	65930670	1	7.14E-11	1.84E-08	+	0.226352	0.2101	+	C	A	0.47692596	0.0344947	+	C	A	0.4924	0.0373
rs2184603	LC_IGP61	IgG1 Fn total	14	65930670	1	2.48E-17	2.30E-14	+	0.292373	0.2881	+	C	A	0.47692596	0.0341348	+	C	A	0.4924	0.0378
rs2184603	LC_IGP81	IgG1 Fn/Bn	14	65930670	1	5.15E-14	8.48E-11	+	0.260741	0.2432	+	C	A	0.47692596	0.0343263	+	C	A	0.4924	0.0375
rs2184603	LC_IGP21	IgG1 Fucosylation	14	65930670	1	1.20E-15	3.22E-13	+	0.27583	0.2746	+	C	A	0.476131628	0.0341133	+	C	A	0.4924	0.0377
rs2184603	LC_IGP28	IgG1 G1S1/(G1+G1S1)	14	65930670	1	1.04E-10	2.41E-10	+	0.221889	0.229	+	C	A	0.476291437	0.0341203	+	C	A	0.4924	0.0362
rs2184603	LC_IGP26	IgG1 GS1/(G+GS1)	14	65930670	1	2.40E-08	6.19E-10	+	0.192436	0.2292	+	C	A	0.476291437	0.0343127	+	C	A	0.4924	0.0371
rs2184603	LC_IGP27	IgG1 GS1/(G0+G+GS1)	14	65930670	1	1.03E-09	1.03E-08	+	0.210085	0.2107	+	C	A	0.476291437	0.0342084	+	C	A	0.4924	0.0368
rs2184603	LC_IGP11	IgG1_G0	14	65930670	1	8.12E-20	9.56E-15	-	-0.310682	-0.2907	+	C	A	0.47662584	0.0336605	+	C	A	0.4924	0.0375
rs2184603	LC_IGP_R1	IgG1_G0F/IgG1_G0	14	65930670	1	4.99E-17	3.65E-12	+	0.286396	0.2615	+	C	A	0.47662584	0.0337762	+	C	A	0.4924	0.0376
rs2184603	LC_IGP_R4	IgG1_G0FN/IgG1_G0N	14	65930670	1	2.98E-15	1.34E-13	+	0.271221	0.2803	+	C	A	0.477179424	0.0340357	+	C	A	0.4924	0.0379
rs2184603	LC_IGP52	IgG1_G0n	14	65930670	1	7.21E-20	9.13E-15	-	-0.313518	-0.2915	+	C	A	0.47692596	0.0339121	+	C	A	0.4924	0.0376
rs2184603	LC_IGP14	IgG1_G0N	14	65930670	1	1.21E-17	1.75E-11	-	-0.293356	-0.2513	+	C	A	0.477179424	0.0339141	+	C	A	0.4924	0.0374
rs2184603	LC_IGP55	IgG1_G0Nn	14	65930670	1	5.44E-18	1.93E-11	-	-0.298218	-0.2511	+	C	A	0.47692596	0.0340971	+	C	A	0.4924	0.0374
rs2184603	LC_IGP12	IgG1_G1	14	65930670	1	1.47E-16	7.57E-14	-	-0.282747	-0.282	+	C	A	0.47662584	0.0338732	+	C	A	0.4924	0.0377
rs2184603	LC_IGP_R2	IgG1_G1F/IgG1_G1	14	65930670	1	7.75E-18	9.48E-14	+	0.294394	0.2802	+	C	A	0.47662584	0.033827	+	C	A	0.4924	0.0376
rs2184603	LC_IGP_R5	IgG1_G1FN/IgG1_G1N	14	65930670	1	4.76E-15	8.32E-18	+	0.270334	0.3242	+	C	A	0.478001845	0.0341814	+	C	A	0.4924	0.0377
rs2184603	LC_IGP53	IgG1_G1n	14	65930670	1	1.16E-15	1.45E-13	-	-0.276787	-0.2785	+	C	A	0.47692596	0.0342124	+	C	A	0.4924	0.0377
rs2184603	LC_IGP15	IgG1_G1N	14	65930670	1	5.38E-12	8.88E-11	-	-0.238346	-0.243	+	C	A	0.478001845	0.0343092	+	C	A	0.4924	0.0375
rs2184603	LC_IGP56	IgG1_G1Nn	14	65930670	1	2.33E-12	1.97E-10	-	-0.243318	-0.2381	+	C	A	0.47692596	0.0344244	+	C	A	0.4924	0.0374
rs2184603	LC_IGP_R36	IgG1_G1NS1/IgG1_G1N	14	65930670	1	4.75E-09	3.27E-12	+	0.201576	0.2557	+	C	A	0.477947596	0.0342356	+	C	A	0.4924	0.0367
rs2184603	LC_IGP_R32	IgG1_G1S1/IgG1_G1	14	65930670	1	1.87E-10	3.63E-10	+	0.218907	0.227	+	C	A	0.476291437	0.0341373	+	C	A	0.4924	0.0362
rs2184603	LC_IGP_R3	IgG1_G2F/IgG1_G2	14	65930670	1	7.43E-16	5.82E-15	+	0.276576	0.2922	+	C	A	0.476305929	0.0339551	+	C	A	0.4924	0.0374
rs2184603	LC_IGP_R8	IgG1_G2FS1/IgG1_G2S1	14	65930670	1	2.57E-10	4.84E-13	+	0.217643	0.2699	+	C	A	0.476014966	0.0342074	+	C	A	0.4924	0.0373
rs2184603	LC_IGP_R26	IgG1_G2N/IgG1_G1N	14	65930670	1	9.06E-13	9.74E-09	+	0.246248	0.2097	+	C	A	0.477628704	0.0341921	+	C	A	0.4924	0.0366
rs2184603	LC_IGP148	IgG2 FG0n total/G0n	14	65930670	1	5.45E-09	5.73E-14	+	0.201453	0.2805	+	C	A	0.475228448	0.0343508	+	C	A	0.4924	0.0373
rs2184603	LC_IGP97	IgG2_G0	14	65930670	1	1.21E-10	3.89E-14	-	-0.221076	-0.2828	+	C	A	0.476305929	0.0341134	+	C	A	0.4924	0.0374
rs2184603	LC_IGP_R41	IgG2_G0F/IgG2_G0	14	65930670	1	4.06E-08	2.62E-13	+	0.188703	0.2716	+	C	A	0.476305929	0.0342204	+	C	A	0.4924	0.0371
rs2184603	LC_IGP138	IgG2_G0n	14	65930670	1	7.41E-11	2.91E-14	-	-0.224407	-0.2846	+	C	A	0.475228448	0.0342301	+	C	A	0.4924	0.0374
rs2184603	LC_IGP_R64	IgG2_G2/IgG2_G1	14	65930670	1	4.36E-09	9.80E-13	+	0.20298	0.2617	+	C	A	0.475358935	0.034393	+	C	A	0.4924	0.0367
rs4902400	LC_IGP78	IgG1 BG0n/G0n	14	65957115	1	6.22E-13	8.00E-10	-	-0.249062	-0.2309	+	G	A	0.467819703	0.0343304	+	G	A	0.4872	0.0376
rs4902400	LC_IGP79	IgG1 BG1n/G1n	14	65957115	1	1.47E-12	5.39E-12	-	-0.245587	-0.2588	+	G	A	0.467819703	0.0344283	+	G	A	0.4872	0.0375
rs4902400	LC_IGP32	IgG1 BG1S1/(BG1+BG1S1)	14	65957115	1	3.66E-08	5.22E-12	+	0.189708	0.2537	+	G	A	0.468888718	0.0342863	+	G	A	0.4872	0.0368
rs4902400	LC_IGP77	IgG1 Bn	14	65957115	1	3.21E-12	1.48E-11	-	-0.241929	-0.2546	+	G	A	0.467819703	0.0344515	+	G	A	0.4872	0.0377
rs4902400	LC_IGP86	IgG1 Bn/Fn total	14	65957115	1	4.89E-13	8.08E-12	-	-0.250755	-0.258	+	G	A	0.467819703	0.0344046	+	G	A	0.4872	0.0377
rs4902400	LC_IGP84	IgG1 FBn/Bn total	14	65957115	1	4.50E-14	1.28E-16	+	0.262106	0.3146	+	G	A	0.467819703	0.0344243	+	G	A	0.4872	0.038
rs4902400	LC_IGP62	IgG1 FG0n total/G0n	14	65957115	1	1.14E-16	1.65E-12	+	0.285818	0.2672	+	G	A	0.467819703	0.0341098	+	G	A	0.4872	0.0378
rs4902400	LC_IGP63	IgG1 FG1n total/G1n	14	65957115	1	5.89E-17	8.16E-15	+	0.289315	0.2935	+	G	A	0.467819703	0.0341965	+	G	A	0.4872	0.0378
rs4902400	LC_IGP64	IgG1 FG2n total/G2n	14	65957115	1	3.01E-13	1.46E-15	+	0.253119	0.3	+	G	A	0.467819703	0.034411	+	G	A	0.4872	0.0376
rs4902400	LC_IGP68	IgG1 FG2n/G2n	14	65957115	1	6.22E-10	1.95E-08	+	0.21505	0.2103	+	G	A	0.467819703	0.0345598	+	G	A	0.4872	0.0374
rs4902400	LC_IGP61	IgG1 Fn total	14	65957115	1	4.85E-16	2.06E-14	+	0.280581	0.2894	+	G	A	0.467819703	0.0342166	+	G	A	0.4872	0.0378
rs4902400	LC_IGP81	IgG1 Fn/Bn	14	65957115	1	1.43E-12	9.37E-11	+	0.245653	0.2433	+	G	A	0.467819703	0.0344156	+	G	A	0.4872	0.0376
rs4902400	LC_IGP21	IgG1 Fucosylation	14	65957115	1	5.04E-14	2.35E-13	+	0.259957	0.2769	+	G	A	0.467062885	0.0342118	+	G	A	0.4872	0.0378
rs4902400	LC_IGP28	IgG1 G1S1/(G1+G1S1)	14	65957115	1	1.25E-10	1.84E-10	+	0.221078	0.2312	+	G	A	0.467298777	0.0341435	+	G	A	0.4872	0.0363
rs4902400	LC_IGP26	IgG1 GS1/(G+GS1)	14	65957115	1	2.34E-08	3.72E-10	+	0.192694	0.2328	+	G	A	0.467298777	0.0343318	+	G	A	0.4872	0.0372
rs4902400	LC_IGP27	IgG1 GS1/(G0+G+GS1)	14	65957115	1	9.48E-10	9.35E-09	+	0.210635	0.2119	+	G	A	0.467298777	0.0342263	+	G	A	0.4872	0.0369
rs4902400	LC_IGP11	IgG1_G0	14	65957115	1	1.19E-18	1.07E-14	-	-0.301002	-0.2909	+	G	A	0.467644166	0.0337336	+	G	A	0.4872	0.0376
rs4902400	LC_IGP_R1	IgG1_G0F/IgG1_G0	14	65957115	1	6.02E-16	4.30E-12	+	0.276574	0.2613	+	G	A	0.467644166	0.0338455	+	G	A	0.4872	0.0377
rs4902400	LC_IGP_R4	IgG1_G0FN/IgG1_G0N	14	65957115	1	1.72E-13	1.90E-13	+	0.253708	0.2792	+	G	A	0.468181261	0.0341373	+	G	A	0.4872	0.038
rs4902400	LC_IGP52	IgG1_G0n	14	65957115	1	1.09E-18	1.11E-14	-	-0.303659	-0.2913	+	G	A	0.467819703	0.0339882	+	G	A	0.4872	0.0377
rs4902400	LC_IGP14	IgG1_G0N	14	65957115	1	9.63E-16	3.06E-11	-	-0.276017	-0.2488	+	G	A	0.468181261	0.0340222	+	G	A	0.4872	0.0375
rs4902400	LC_IGP55	IgG1_G0Nn	14	65957115	1	4.62E-16	3.55E-11	-	-0.280733	-0.2483	+	G	A	0.467819703	0.0342095	+	G	A	0.4872	0.0375
rs4902400	LC_IGP12	IgG1_G1	14	65957115	1	1.99E-15	5.30E-14	-	-0.272248	-0.2844	+	G	A	0.467644166	0.0339451	+	G	A	0.4872	0.0378
rs4902400	LC_IGP_R2	IgG1_G1F/IgG1_G1	14	65957115	1	8.72E-17	7.30E-14	+	0.285098	0.2822	+	G	A	0.467644166	0.0338949	+	G	A	0.4872	0.0377
rs4902400	LC_IGP_R5	IgG1_G1FN/IgG1_G1N	14	65957115	1	1.67E-13	7.64E-18	+	0.254844	0.3254	+	G	A	0.468965252	0.0342717	+	G	A	0.4872	0.0378
rs4902400	LC_IGP53	IgG1_G1n	14	65957115	1	1.31E-14	1.09E-13	-	-0.266643	-0.2806	+	G	A	0.467819703	0.0342828	+	G	A	0.4872	0.0378
rs4902400	LC_IGP15	IgG1_G1N	14	65957115	1	9.08E-11	6.73E-11	-	-0.224347	-0.2452	+	G	A	0.468965252	0.0343843	+	G	A	0.4872	0.0376
rs4902400	LC_IGP56	IgG1_G1Nn	14	65957115	1	3.79E-11	1.60E-10	-	-0.229719	-0.2399	+	G	A	0.467819703	0.0345027	+	G	A	0.4872	0.0375
rs4902400	LC_IGP_R36	IgG1_G1NS1/IgG1_G1N	14	65957115	1	3.03E-08	5.96E-12	+	0.190883	0.2532	+	G	A	0.468888718	0.0342905	+	G	A	0.4872	0.0368
rs4902400	LC_IGP_R32	IgG1_G1S1/IgG1_G1	14	65957115	1	2.33E-10	2.74E-10	+	0.21789	0.2293	+	G	A	0.467298777	0.0341613	+	G	A	0.4872	0.0363
rs4902400	LC_IGP_R3	IgG1_G2F/IgG1_G2	14	65957115	1	9.50E-15	4.74E-15	+	0.266041	0.2939	+	G	A	0.467318765	0.0340267	+	G	A	0.4872	0.0375
rs4902400	LC_IGP_R8	IgG1_G2FS1/IgG1_G2S1	14	65957115	1	2.90E-09	1.70E-13	+	0.20464	0.2758	+	G	A	0.467033293	0.0342771	+	G	A	0.4872	0.0374
rs4902400	LC_IGP_R26	IgG1_G2N/IgG1_G1N	14	65957115	1	6.42E-12	1.24E-08	+	0.237067	0.2087	+	G	A	0.468556173	0.0342506	+	G	A	0.4872	0.0366
rs4902400	LC_IGP148	IgG2 FG0n total/G0n	14	65957115	1	2.99E-08	5.25E-14	+	0.191636	0.2818	+	G	A	0.466171798	0.0344087	+	G	A	0.4872	0.0374
rs4902400	LC_IGP97	IgG2_G0	14	65957115	1	5.13E-10	3.02E-14	-	-0.21362	-0.2849	+	G	A	0.467318765	0.0341623	+	G	A	0.4872	0.0375
rs4902400	LC_IGP138	IgG2_G0n	14	65957115	1	3.62E-10	2.44E-14	-	-0.216298	-0.2863	+	G	A	0.466171798	0.0342857	+	G	A	0.4872	0.0376
rs4902400	LC_IGP_R64	IgG2_G2/IgG2_G1	14	65957115	1	4.23E-09	1.27E-12	+	0.203287	0.261	+	G	A	0.466360771	0.0344143	+	G	A	0.4872	0.0368
rs3825640	LC_IGP78	IgG1 BG0n/G0n	14	65961204	1	6.86E-15	6.63E-10	-	-0.269833	-0.2311	+	G	C	0.47211803	0.0343205	+	G	C	0.4925	0.0374
rs3825640	LC_IGP79	IgG1 BG1n/G1n	14	65961204	1	4.57E-14	8.68E-12	-	-0.262151	-0.2553	+	G	C	0.47211803	0.0344402	+	G	C	0.4925	0.0374
rs3825640	LC_IGP32	IgG1 BG1S1/(BG1+BG1S1)	14	65961204	1	6.69E-09	3.15E-12	+	0.200165	0.2552	+	G	C	0.473167386	0.0343362	+	G	C	0.4925	0.0366
rs3825640	LC_IGP31	IgG1 BGS1/(BG0+BG+BGS1)	14	65961204	1	9.71E-09	7.78E-12	+	0.198753	0.2532	+	G	C	0.473193267	0.0344681	+	G	C	0.4925	0.037
rs3825640	LC_IGP77	IgG1 Bn	14	65961204	1	5.91E-14	1.97E-11	-	-0.261062	-0.2521	+	G	C	0.47211803	0.0344525	+	G	C	0.4925	0.0376
rs3825640	LC_IGP86	IgG1 Bn/Fn total	14	65961204	1	8.31E-15	1.13E-11	-	-0.269628	-0.2553	+	G	C	0.47211803	0.0344038	+	G	C	0.4925	0.0376
rs3825640	LC_IGP84	IgG1 FBn/Bn total	14	65961204	1	6.72E-16	1.88E-16	+	0.280846	0.3117	+	G	C	0.47211803	0.0344206	+	G	C	0.4925	0.0379
rs3825640	LC_IGP62	IgG1 FG0n total/G0n	14	65961204	1	2.80E-18	1.88E-12	+	0.301059	0.2654	+	G	C	0.47211803	0.0341163	+	G	C	0.4925	0.0377
rs3825640	LC_IGP63	IgG1 FG1n total/G1n	14	65961204	1	2.66E-18	1.34E-14	+	0.302144	0.29	+	G	C	0.47211803	0.0342158	+	G	C	0.4925	0.0377
rs3825640	LC_IGP64	IgG1 FG2n total/G2n	14	65961204	1	2.57E-14	2.34E-15	+	0.26483	0.2967	+	G	C	0.47211803	0.0344441	+	G	C	0.4925	0.0374
rs3825640	LC_IGP68	IgG1 FG2n/G2n	14	65961204	1	4.68E-11	2.26E-08	+	0.229202	0.2085	+	G	C	0.47211803	0.0345907	+	G	C	0.4925	0.0373
rs3825640	LC_IGP61	IgG1 Fn total	14	65961204	1	2.10E-17	2.98E-14	+	0.293923	0.2864	+	G	C	0.47211803	0.0342351	+	G	C	0.4925	0.0377
rs3825640	LC_IGP81	IgG1 Fn/Bn	14	65961204	1	2.75E-14	1.13E-10	+	0.264311	0.2413	+	G	C	0.47211803	0.0344175	+	G	C	0.4925	0.0374
rs3825640	LC_IGP21	IgG1 Fucosylation	14	65961204	1	9.50E-16	4.55E-13	+	0.277641	0.2725	+	G	C	0.47134402	0.0342128	+	G	C	0.4925	0.0376
rs3825640	LC_IGP28	IgG1 G1S1/(G1+G1S1)	14	65961204	1	7.47E-11	2.28E-10	+	0.224262	0.2289	+	G	C	0.471548318	0.0342154	+	G	C	0.4925	0.0361
rs3825640	LC_IGP26	IgG1 GS1/(G+GS1)	14	65961204	1	2.37E-08	9.82E-10	+	0.193077	0.2262	+	G	C	0.471548318	0.0344151	+	G	C	0.4925	0.037
rs3825640	LC_IGP27	IgG1 GS1/(G0+G+GS1)	14	65961204	1	8.32E-10	1.82E-08	+	0.211847	0.2068	+	G	C	0.471548318	0.0343064	+	G	C	0.4925	0.0367
rs3825640	LC_IGP11	IgG1_G0	14	65961204	1	2.26E-20	1.59E-14	-	-0.31641	-0.2878	+	G	C	0.471583079	0.033757	+	G	C	0.4925	0.0375
rs3825640	LC_IGP_R1	IgG1_G0F/IgG1_G0	14	65961204	1	1.17E-17	4.66E-12	+	0.293117	0.2598	+	G	C	0.471583079	0.0338695	+	G	C	0.4925	0.0376
rs3825640	LC_IGP_R4	IgG1_G0FN/IgG1_G0N	14	65961204	1	1.57E-15	1.91E-13	+	0.274907	0.2781	+	G	C	0.472124617	0.0341466	+	G	C	0.4925	0.0378
rs3825640	LC_IGP52	IgG1_G0n	14	65961204	1	3.96E-20	1.50E-14	-	-0.316641	-0.2887	+	G	C	0.47211803	0.0340028	+	G	C	0.4925	0.0376
rs3825640	LC_IGP14	IgG1_G0N	14	65961204	1	2.65E-18	2.83E-11	-	-0.300265	-0.2483	+	G	C	0.472124617	0.0340066	+	G	C	0.4925	0.0373
rs3825640	LC_IGP55	IgG1_G0Nn	14	65961204	1	2.67E-18	3.07E-11	-	-0.301868	-0.2481	+	G	C	0.47211803	0.0341858	+	G	C	0.4925	0.0374
rs3825640	LC_IGP12	IgG1_G1	14	65961204	1	5.60E-17	9.48E-14	-	-0.287621	-0.2804	+	G	C	0.471583079	0.0339768	+	G	C	0.4925	0.0377
rs3825640	LC_IGP_R2	IgG1_G1F/IgG1_G1	14	65961204	1	2.44E-18	1.17E-13	+	0.299882	0.2788	+	G	C	0.471583079	0.0339265	+	G	C	0.4925	0.0376
rs3825640	LC_IGP_R5	IgG1_G1FN/IgG1_G1N	14	65961204	1	3.98E-15	1.14E-17	+	0.271899	0.3224	+	G	C	0.473233395	0.0342788	+	G	C	0.4925	0.0377
rs3825640	LC_IGP53	IgG1_G1n	14	65961204	1	1.07E-15	1.76E-13	-	-0.277968	-0.2771	+	G	C	0.47211803	0.0343148	+	G	C	0.4925	0.0376
rs3825640	LC_IGP15	IgG1_G1N	14	65961204	1	3.40E-12	1.15E-10	-	-0.241267	-0.2412	+	G	C	0.473233395	0.0344012	+	G	C	0.4925	0.0374
rs3825640	LC_IGP56	IgG1_G1Nn	14	65961204	1	1.38E-12	2.47E-10	-	-0.246564	-0.2365	+	G	C	0.47211803	0.034518	+	G	C	0.4925	0.0374
rs3825640	LC_IGP_R36	IgG1_G1NS1/IgG1_G1N	14	65961204	1	5.61E-09	3.67E-12	+	0.201217	0.2546	+	G	C	0.473167386	0.0343407	+	G	C	0.4925	0.0366
rs3825640	LC_IGP_R32	IgG1_G1S1/IgG1_G1	14	65961204	1	1.31E-10	3.55E-10	+	0.221402	0.2268	+	G	C	0.471548318	0.0342321	+	G	C	0.4925	0.0362
rs3825640	LC_IGP_R3	IgG1_G2F/IgG1_G2	14	65961204	1	9.44E-16	7.32E-15	+	0.276417	0.2908	+	G	C	0.471565709	0.0340615	+	G	C	0.4925	0.0374
rs3825640	LC_IGP_R8	IgG1_G2FS1/IgG1_G2S1	14	65961204	1	3.42E-10	5.64E-13	+	0.216789	0.2688	+	G	C	0.471277642	0.0343158	+	G	C	0.4925	0.0373
rs3825640	LC_IGP_R26	IgG1_G2N/IgG1_G1N	14	65961204	1	6.80E-13	1.42E-08	+	0.248323	0.207	+	G	C	0.47283858	0.0342887	+	G	C	0.4925	0.0365
rs3825640	LC_IGP148	IgG2 FG0n total/G0n	14	65961204	1	1.35E-08	8.29E-14	+	0.196824	0.2783	+	G	C	0.470447968	0.0344752	+	G	C	0.4925	0.0373
rs3825640	LC_IGP97	IgG2_G0	14	65961204	1	3.11E-10	5.85E-14	-	-0.216787	-0.2804	+	G	C	0.471565709	0.0342347	+	G	C	0.4925	0.0373
rs3825640	LC_IGP138	IgG2_G0n	14	65961204	1	2.03E-10	4.33E-14	-	-0.21987	-0.2823	+	G	C	0.470447968	0.0343563	+	G	C	0.4925	0.0374
rs3825640	LC_IGP_R64	IgG2_G2/IgG2_G1	14	65961204	1	6.38E-09	1.62E-12	+	0.201426	0.2588	+	G	C	0.470612913	0.0345049	+	G	C	0.4925	0.0366
rs72714461	LC_IGP61	IgG1 Fn total	14	65968132	1	3.68E-08	2.08E-06	-	-0.237884	-0.2439	+	G	A	0.20572015	0.0429996	+	G	A	0.1658	0.0514
rs12895074	LC_IGP78	IgG1 BG0n/G0n	14	65976793	1	4.47E-13	2.07E-09	-	-0.252413	-0.2273	+	T	G	0.459752788	0.0345733	+	T	G	0.4793	0.0379
rs12895074	LC_IGP79	IgG1 BG1n/G1n	14	65976793	1	8.89E-13	1.70E-11	-	-0.249775	-0.2549	+	T	G	0.459752788	0.0346682	+	T	G	0.4793	0.0379
rs12895074	LC_IGP32	IgG1 BG1S1/(BG1+BG1S1)	14	65976793	1	4.79E-08	4.97E-12	+	0.189431	0.2569	+	T	G	0.460864057	0.034537	+	T	G	0.4793	0.0372
rs12895074	LC_IGP77	IgG1 Bn	14	65976793	1	2.17E-12	4.69E-11	-	-0.245564	-0.2505	+	T	G	0.459752788	0.034694	+	T	G	0.4793	0.0381
rs12895074	LC_IGP86	IgG1 Bn/Fn total	14	65976793	1	3.55E-13	2.49E-11	-	-0.254069	-0.2542	+	T	G	0.459752788	0.0346482	+	T	G	0.4793	0.0381
rs12895074	LC_IGP84	IgG1 FBn/Bn total	14	65976793	1	4.55E-14	2.80E-16	+	0.263965	0.314	+	T	G	0.459752788	0.0346751	+	T	G	0.4793	0.0384
rs12895074	LC_IGP62	IgG1 FG0n total/G0n	14	65976793	1	1.19E-16	3.02E-12	+	0.287729	0.2666	+	T	G	0.459752788	0.0343589	+	T	G	0.4793	0.0382

rs12895074	LC_IGP63	IgG1 FG1n total/G1n	14	65976793	1	7.64E-17	1.74E-14	+	0.290376	0.2924	+	T	G	0.459752788	0.0344508	+	T	G	0.4793	0.0381
rs12895074	LC_IGP64	IgG1 FG2n total/G2n	14	65976793	1	3.59E-13	2.71E-15	+	0.254138	0.2998	+	T	G	0.459752788	0.0346652	+	T	G	0.4793	0.0379
rs12895074	LC_IGP68	IgG1 FG2n/G2n	14	65976793	1	4.52E-10	4.21E-08	+	0.218357	0.2071	+	T	G	0.459752788	0.0348046	+	T	G	0.4793	0.0378
rs12895074	LC_IGP61	IgG1 Fn total	14	65976793	1	6.72E-16	4.20E-14	+	0.281268	0.2884	+	T	G	0.459752788	0.0344725	+	T	G	0.4793	0.0382
rs12895074	LC_IGP81	IgG1 Fn/Bn	14	65976793	1	9.57E-13	2.88E-10	+	0.24934	0.239	+	T	G	0.459752788	0.0346576	+	T	G	0.4793	0.0379
rs12895074	LC_IGP21	IgG1 Fucosylation	14	65976793	1	5.26E-14	3.95E-13	+	0.261659	0.277	+	T	G	0.459034217	0.0344621	+	T	G	0.4793	0.0382
rs12895074	LC_IGP28	IgG1 G1S1/(G1+G1S1)	14	65976793	1	9.83E-11	1.95E-10	+	0.223917	0.2331	+	T	G	0.459338226	0.0343838	+	T	G	0.4793	0.0366
rs12895074	LC_IGP26	IgG1 GS1/(G+GS1)	14	65976793	1	2.39E-08	3.59E-10	+	0.193943	0.235	+	T	G	0.459338226	0.0345789	+	T	G	0.4793	0.0375
rs12895074	LC_IGP27	IgG1 GS1/(G0+G+GS1)	14	65976793	1	8.16E-10	9.70E-09	+	0.212962	0.2138	+	T	G	0.459338226	0.0344691	+	T	G	0.4793	0.0373
rs12895074	LC_IGP11	IgG1_G0	14	65976793	1	6.39E-19	1.42E-14	-	-0.305699	-0.2926	+	T	G	0.459387905	0.033986	+	T	G	0.4793	0.038
rs12895074	LC_IGP_R1	IgG1_G0F/IgG1_G0	14	65976793	1	2.67E-16	7.46E-12	+	0.282081	0.2609	+	T	G	0.459387905	0.0340947	+	T	G	0.4793	0.0381
rs12895074	LC_IGP_R4	IgG1_G0FN/IgG1_G0N	14	65976793	1	1.45E-13	3.94E-13	+	0.256466	0.2783	+	T	G	0.459904164	0.0344023	+	T	G	0.4793	0.0383
rs12895074	LC_IGP52	IgG1_G0n	14	65976793	1	1.11E-18	1.52E-14	-	-0.305809	-0.2929	+	T	G	0.459752788	0.0342359	+	T	G	0.4793	0.0381
rs12895074	LC_IGP14	IgG1_G0N	14	65976793	1	3.51E-16	7.36E-11	-	-0.282363	-0.2464	+	T	G	0.459904164	0.0342688	+	T	G	0.4793	0.0378
rs12895074	LC_IGP55	IgG1_G0Nn	14	65976793	1	3.25E-16	8.79E-11	-	-0.284219	-0.2457	+	T	G	0.459752788	0.0344511	+	T	G	0.4793	0.0379
rs12895074	LC_IGP12	IgG1_G1	14	65976793	1	1.39E-15	9.50E-14	-	-0.275884	-0.284	+	T	G	0.459387905	0.0342045	+	T	G	0.4793	0.0381
rs12895074	LC_IGP_R2	IgG1_G1F/IgG1_G1	14	65976793	1	4.86E-17	1.51E-13	+	0.289671	0.2811	+	T	G	0.459387905	0.0341493	+	T	G	0.4793	0.0381
rs12895074	LC_IGP_R5	IgG1_G1FN/IgG1_G1N	14	65976793	1	1.59E-13	1.62E-17	+	0.256891	0.3251	+	T	G	0.460960332	0.0345149	+	T	G	0.4793	0.0382
rs12895074	LC_IGP53	IgG1_G1n	14	65976793	1	2.04E-14	2.01E-13	-	-0.266635	-0.28	+	T	G	0.459752788	0.0345417	+	T	G	0.4793	0.0381
rs12895074	LC_IGP15	IgG1_G1N	14	65976793	1	6.20E-11	1.99E-10	-	-0.227914	-0.2412	+	T	G	0.460960332	0.0346214	+	T	G	0.4793	0.0379
rs12895074	LC_IGP56	IgG1_G1Nn	14	65976793	1	2.34E-11	4.76E-10	-	-0.233855	-0.2358	+	T	G	0.459752788	0.0347435	+	T	G	0.4793	0.0379
rs12895074	LC_IGP_R36	IgG1_G1NS1/IgG1_G1N	14	65976793	1	3.86E-08	5.76E-12	+	0.190801	0.2564	+	T	G	0.460864057	0.0345406	+	T	G	0.4793	0.0372
rs12895074	LC_IGP_R32	IgG1_G1S1/IgG1_G1	14	65976793	1	1.79E-10	3.03E-10	+	0.22084	0.2308	+	T	G	0.459338226	0.0344013	+	T	G	0.4793	0.0367
rs12895074	LC_IGP_R3	IgG1_G2F/IgG1_G2	14	65976793	1	1.29E-14	8.33E-15	+	0.266614	0.2939	+	T	G	0.459363081	0.0342775	+	T	G	0.4793	0.0379
rs12895074	LC_IGP_R8	IgG1_G2FS1/IgG1_G2S1	14	65976793	1	4.34E-09	3.02E-13	+	0.203834	0.2756	+	T	G	0.459082468	0.0345319	+	T	G	0.4793	0.0378
rs12895074	LC_IGP_R26	IgG1_G2N/IgG1_G1N	14	65976793	1	4.55E-12	2.63E-08	+	0.240449	0.2061	+	T	G	0.460519136	0.0344897	+	T	G	0.4793	0.037
rs12895074	LC_IGP97	IgG2_G0	14	65976793	1	2.39E-09	4.62E-14	-	-0.20671	-0.2862	+	T	G	0.459363081	0.0344394	+	T	G	0.4793	0.038
rs12895074	LC_IGP138	IgG2_G0n	14	65976793	1	1.81E-09	3.81E-14	-	-0.209109	-0.2875	+	T	G	0.458154249	0.0345706	+	T	G	0.4793	0.038
rs12895074	LC_IGP_R64	IgG2_G2/IgG2_G1	14	65976793	1	6.13E-09	1.66E-12	+	0.202635	0.2632	+	T	G	0.458395349	0.0346716	+	T	G	0.4793	0.0373
rs12879202	LC_IGP78	IgG1 BG0n/G0n	14	65977182	1	3.16E-13	6.92E-10	-	-0.252742	-0.2316	+	T	C	0.462991016	0.0343922	+	T	C	0.4866	0.0376
rs12879202	LC_IGP79	IgG1 BG1n/G1n	14	65977182	1	8.78E-13	4.89E-12	-	-0.248582	-0.2592	+	T	C	0.462991016	0.0344936	+	T	C	0.4866	0.0375
rs12879202	LC_IGP32	IgG1 BG1S1/(BG1+BG1S1)	14	65977182	1	4.19E-08	4.00E-12	+	0.189309	0.2549	+	T	C	0.464086621	0.0343626	+	T	C	0.4866	0.0368
rs12879202	LC_IGP77	IgG1 Bn	14	65977182	1	1.82E-12	1.34E-11	-	-0.245184	-0.255	+	T	C	0.462991016	0.0345158	+	T	C	0.4866	0.0377
rs12879202	LC_IGP86	IgG1 Bn/Fn total	14	65977182	1	2.83E-13	7.23E-12	-	-0.253842	-0.2585	+	T	C	0.462991016	0.0344693	+	T	C	0.4866	0.0377
rs12879202	LC_IGP84	IgG1 FBn/Bn total	14	65977182	1	3.43E-14	9.88E-17	+	0.263894	0.3156	+	T	C	0.462991016	0.0344948	+	T	C	0.4866	0.038
rs12879202	LC_IGP62	IgG1 FG0n total/G0n	14	65977182	1	6.81E-17	1.31E-12	+	0.288526	0.2682	+	T	C	0.462991016	0.0341745	+	T	C	0.4866	0.0378
rs12879202	LC_IGP63	IgG1 FG1n total/G1n	14	65977182	1	5.08E-17	6.42E-15	+	0.290546	0.2945	+	T	C	0.462991016	0.0342691	+	T	C	0.4866	0.0378
rs12879202	LC_IGP64	IgG1 FG2n total/G2n	14	65977182	1	3.48E-13	1.13E-15	+	0.253004	0.3011	+	T	C	0.462991016	0.0344903	+	T	C	0.4866	0.0376
rs12879202	LC_IGP68	IgG1 FG2n/G2n	14	65977182	1	4.20E-10	1.75E-08	+	0.217656	0.2109	+	T	C	0.462991016	0.0346281	+	T	C	0.4866	0.0374
rs12879202	LC_IGP61	IgG1 Fn total	14	65977182	1	4.14E-16	1.60E-14	+	0.281854	0.2904	+	T	C	0.462991016	0.034289	+	T	C	0.4866	0.0378
rs12879202	LC_IGP81	IgG1 Fn/Bn	14	65977182	1	7.77E-13	8.44E-11	+	0.249066	0.2437	+	T	C	0.462991016	0.034479	+	T	C	0.4866	0.0375
rs12879202	LC_IGP21	IgG1 Fucosylation	14	65977182	1	4.12E-14	1.89E-13	+	0.261428	0.2778	+	T	C	0.462256473	0.0342841	+	T	C	0.4866	0.0378
rs12879202	LC_IGP28	IgG1 G1S1/(G1+G1S1)	14	65977182	1	8.81E-11	1.22E-10	+	0.223367	0.2333	+	T	C	0.462535474	0.0342108	+	T	C	0.4866	0.0362
rs12879202	LC_IGP26	IgG1 GS1/(G+GS1)	14	65977182	1	2.30E-08	2.97E-10	+	0.193198	0.2339	+	T	C	0.462535474	0.0344064	+	T	C	0.4866	0.0371
rs12879202	LC_IGP27	IgG1 GS1/(G0+G+GS1)	14	65977182	1	7.60E-10	8.36E-09	+	0.212288	0.2124	+	T	C	0.462535474	0.0342965	+	T	C	0.4866	0.0369
rs12879202	LC_IGP11	IgG1_G0	14	65977182	1	3.41E-19	8.53E-15	-	-0.3065	-0.2918	+	T	C	0.462581246	0.0338044	+	T	C	0.4866	0.0376
rs12879202	LC_IGP_R1	IgG1_G0F/IgG1_G0	14	65977182	1	1.46E-16	3.41E-12	+	0.283112	0.2624	+	T	C	0.462581246	0.0339131	+	T	C	0.4866	0.0377
rs12879202	LC_IGP_R4	IgG1_G0FN/IgG1_G0N	14	65977182	1	9.37E-14	1.48E-13	+	0.257163	0.2803	+	T	C	0.463105021	0.0342222	+	T	C	0.4866	0.0379
rs12879202	LC_IGP52	IgG1_G0n	14	65977182	1	6.06E-19	8.78E-15	-	-0.306547	-0.2922	+	T	C	0.462991016	0.0340512	+	T	C	0.4866	0.0377
rs12879202	LC_IGP14	IgG1_G0N	14	65977182	1	2.25E-16	2.78E-11	-	-0.282769	-0.2492	+	T	C	0.463105021	0.0340893	+	T	C	0.4866	0.0374
rs12879202	LC_IGP55	IgG1_G0Nn	14	65977182	1	2.29E-16	3.22E-11	-	-0.284224	-0.2486	+	T	C	0.462991016	0.0342706	+	T	C	0.4866	0.0375
rs12879202	LC_IGP12	IgG1_G1	14	65977182	1	8.03E-16	3.98E-14	-	-0.276798	-0.2856	+	T	C	0.462581246	0.0340234	+	T	C	0.4866	0.0378
rs12879202	LC_IGP_R2	IgG1_G1F/IgG1_G1	14	65977182	1	2.91E-17	5.70E-14	+	0.290259	0.2833	+	T	C	0.462581246	0.0339693	+	T	C	0.4866	0.0377
rs12879202	LC_IGP_R5	IgG1_G1FN/IgG1_G1N	14	65977182	1	1.41E-13	5.89E-18	+	0.25616	0.3263	+	T	C	0.464174969	0.0343414	+	T	C	0.4866	0.0378
rs12879202	LC_IGP53	IgG1_G1n	14	65977182	1	1.23E-14	8.26E-14	-	-0.267514	-0.2818	+	T	C	0.462991016	0.0343573	+	T	C	0.4866	0.0378
rs12879202	LC_IGP15	IgG1_G1N	14	65977182	1	5.76E-11	6.02E-11	-	-0.227169	-0.2457	+	T	C	0.464174969	0.0344484	+	T	C	0.4866	0.0376
rs12879202	LC_IGP56	IgG1_G1Nn	14	65977182	1	2.27E-11	1.43E-10	-	-0.232835	-0.2404	+	T	C	0.462991016	0.0345682	+	T	C	0.4866	0.0375
rs12879202	LC_IGP_R36	IgG1_G1NS1/IgG1_G1N	14	65977182	1	3.38E-08	4.61E-12	+	0.190648	0.2544	+	T	C	0.464086621	0.0343663	+	T	C	0.4866	0.0368
rs12879202	LC_IGP_R32	IgG1_G1S1/IgG1_G1	14	65977182	1	1.60E-10	1.88E-10	+	0.22031	0.2312	+	T	C	0.462535474	0.0342282	+	T	C	0.4866	0.0363
rs12879202	LC_IGP_R3	IgG1_G2F/IgG1_G2	14	65977182	1	1.23E-14	3.60E-15	+	0.265509	0.2951	+	T	C	0.462558374	0.0341063	+	T	C	0.4866	0.0375
rs12879202	LC_IGP_R8	IgG1_G2FS1/IgG1_G2S1	14	65977182	1	3.79E-09	1.36E-13	+	0.20359	0.2768	+	T	C	0.462275809	0.0343576	+	T	C	0.4866	0.0374
rs12879202	LC_IGP_R26	IgG1_G2N/IgG1_G1N	14	65977182	1	4.68E-12	1.13E-08	+	0.239115	0.2091	+	T	C	0.46374537	0.0343184	+	T	C	0.4866	0.0366
rs12879202	LC_IGP97	IgG2_G0	14	65977182	1	1.32E-09	2.90E-14	-	-0.208986	-0.2848	+	T	C	0.462558374	0.0342562	+	T	C	0.4866	0.0375
rs12879202	LC_IGP138	IgG2_G0n	14	65977182	1	9.88E-10	2.32E-14	-	-0.21138	-0.2863	+	T	C	0.461372537	0.0343839	+	T	C	0.4866	0.0375
rs12879202	LC_IGP_R64	IgG2_G2/IgG2_G1	14	65977182	1	6.18E-09	1.26E-12	+	0.201575	0.2609	+	T	C	0.461594553	0.0344985	+	T	C	0.4866	0.0368
rs11627084	LC_IGP78	IgG1 BG0n/G0n	14	65978836	1	1.92E-14	8.24E-10	-	-0.264445	-0.23	+	G	T	0.477159232	0.0342233	+	G	T	0.4932	0.0375
rs11627084	LC_IGP79	IgG1 BG1n/G1n	14	65978836	1	1.05E-13	1.03E-11	-	-0.257517	-0.2546	+	G	T	0.477159232	0.0343385	+	G	T	0.4932	0.0374
rs11627084	LC_IGP32	IgG1 BG1S1/(BG1+BG1S1)	14	65978836	1	7.80E-09	4.44E-12	+	0.198612	0.2537	+	G	T	0.478184957	0.0342225	+	G	T	0.4932	0.0366
rs11627084	LC_IGP31	IgG1 BGS1/(BG0+BG+BGS1)	14	65978836	1	3.41E-08	1.06E-11	+	0.190652	0.2517	+	G	T	0.478265586	0.0343757	+	G	T	0.4932	0.037
rs11627084	LC_IGP77	IgG1 Bn	14	65978836	1	1.42E-13	2.35E-11	-	-0.256219	-0.2513	+	G	T	0.477159232	0.0343516	+	G	T	0.4932	0.0376
rs11627084	LC_IGP86	IgG1 Bn/Fn total	14	65978836	1	1.98E-14	1.36E-11	-	-0.264922	-0.2545	+	G	T	0.477159232	0.0343029	+	G	T	0.4932	0.0376
rs11627084	LC_IGP84	IgG1 FBn/Bn total	14	65978836	1	1.16E-15	2.64E-16	+	0.277595	0.3105	+	G	T	0.477159232	0.034313	+	G	T	0.4932	0.0379
rs11627084	LC_IGP62	IgG1 FG0n total/G0n	14	65978836	1	6.80E-18	2.53E-12	+	0.296623	0.2642	+	G	T	0.477159232	0.0340167	+	G	T	0.4932	0.0377
rs11627084	LC_IGP63	IgG1 FG1n total/G1n	14	65978836	1	4.05E-18	1.80E-14	+	0.299492	0.2889	+	G	T	0.477159232	0.0341062	+	G	T	0.4932	0.0377
rs11627084	LC_IGP64	IgG1 FG2n total/G2n	14	65978836	1	2.64E-14	3.10E-15	+	0.263799	0.2957	+	G	T	0.477159232	0.0343255	+	G	T	0.4932	0.0375
rs11627084	LC_IGP68	IgG1 FG2n/G2n	14	65978836	1	8.33E-11	2.56E-08	+	0.225456	0.2079	+	G	T	0.477159232	0.0344831	+	G	T	0.4932	0.0373
rs11627084	LC_IGP61	IgG1 Fn total	14	65978836	1	3.28E-17	4.05E-14	+	0.291141	0.2852	+	G	T	0.477159232	0.0341259	+	G	T	0.4932	0.0377
rs11627084	LC_IGP81	IgG1 Fn/Bn	14	65978836	1	6.98E-14	1.33E-10	+	0.259271	0.2406	+	G	T	0.477159232	0.0343179	+	G	T	0.4932	0.0374
rs11627084	LC_IGP21	IgG1 Fucosylation	14	65978836	1	1.47E-15	5.91E-13	+	0.274863	0.2714	+	G	T	0.476359125	0.0341031	+	G	T	0.4932	0.0377
rs11627084	LC_IGP28	IgG1 G1S1/(G1+G1S1)	14	65978836	1	1.33E-10	3.48E-10	+	0.220525	0.2268	+	G	T	0.476525994	0.0341096	+	G	T	0.4932	0.0361
rs11627084	LC_IGP26	IgG1 GS1/(G+GS1)	14	65978836	1	2.75E-08	1.25E-09	+	0.191522	0.225	+	G	T	0.476525994	0.0342997	+	G	T	0.4932	0.037
rs11627084	LC_IGP27	IgG1 GS1/(G0+G+GS1)	14	65978836	1	1.23E-09	2.10E-08	+	0.208983	0.2061	+	G	T	0.476525994	0.0341964	+	G	T	0.4932	0.0368
rs11627084	LC_IGP11	IgG1_G0	14	65978836	1	1.18E-19	2.12E-14	-	-0.309204	-0.2867	+	G	T	0.47686011	0.0336526	+	G	T	0.4932	0.0375
rs11627084	LC_IGP_R1	IgG1_G0F/IgG1_G0	14	65978836	1	6.96E-17	6.22E-12	+	0.284955	0.2586	+	G	T	0.47686011	0.0337674	+	G	T	0.4932	0.0376
rs11627084	LC_IGP_R4	IgG1_G0FN/IgG1_G0N	14	65978836	1	4.01E-15	2.65E-13	+	0.269851	0.2767	+	G	T	0.477414574	0.0340266	+	G	T	0.4932	0.0378
rs11627084	LC_IGP52	IgG1_G0n	14	65978836	1	1.05E-19	2.01E-14	-	-0.312013	-0.2876	+	G	T	0.477159232	0.0339054	+	G	T	0.4932	0.0376
rs11627084	LC_IGP14	IgG1_G0N	14	65978836	1	1.85E-17	3.38E-11	-	-0.291592	-0.2475	+	G	T	0.477414574	0.0339076	+	G	T	0.4932	0.0373
rs11627084	LC_IGP55	IgG1_G0Nn	14	65978836	1	8.34E-18	3.67E-11	-	-0.296445	-0.2473	+	G	T	0.477159232	0.0340913	+	G	T	0.4932	0.0374
rs11627084	LC_IGP12	IgG1_G1	14	65978836	1	1.81E-16	1.30E-13	-	-0.28178	-0.2792	+	G	T	0.47686011	0.0338619	+	G	T	0.4932	0.0377
rs11627084	LC_IGP_R2	IgG1_G1F/IgG1_G1	14	65978836	1	9.72E-18	1.55E-13	+	0.293388	0.2777	+	G	T	0.47686011	0.0338161	+	G	T	0.4932	0.0376
rs11627084	LC_IGP_R5	IgG1_G1FN/IgG1_G1N	14	65978836	1	6.28E-15	1.60E-17	+	0.269035	0.3213	+	G	T	0.478238622	0.0341719	+	G	T	0.4932	0.0377
rs11627084	LC_IGP53	IgG1_G1n	14	65978836	1	1.42E-15	2.39E-13	-	-0.275815	-0.2759	+	G	T	0.477159232	0.034202	+	G	T	0.4932	0.0377
rs11627084	LC_IGP15	IgG1_G1N	14	65978836	1	7.20E-12	1.37E-10	-	-0.236829	-0.2405	+	G	T	0.478238622	0.0343	+	G	T	0.4932	0.0375
rs11627084	LC_IGP56	IgG1_G1Nn	14	65978836	1	3.08E-12	2.93E-10	-	-0.241877	-0.2357	+	G	T	0.477159232	0.0344154	+	G	T	0.4932	0.0374
rs11627084	LC_IGP_R36	IgG1_G1NS1/IgG1_G1N	14	65978836	1	6.78E-09	5.13E-12	+	0.199457	0.2532	+	G	T	0.478184957	0.0342277	+	G	T	0.4932	0.0367
rs11627084	LC_IGP_R32	IgG1_G1S1/IgG1_G1	14	65978836	1	2.40E-10	5.24E-10	+	0.217513	0.2248	+	G	T	0.476525994	0.0341267	+	G	T	0.4932	0.0362
rs11627084	LC_IGP_R3	IgG1_G2F/IgG1_G2	14	65978836	1	8.78E-16	9.79E-15	+	0.275765	0.2897	+	G	T	0.476540342	0.0339429	+	G	T	0.4932	0.0374
rs11627084	LC_IGP_R8	IgG1_G2FS1/IgG1_G2S1	14	65978836	1	3.10E-10	7.12E-13	+	0.216557	0.2678	+	G	T	0.476249236	0.0341955	+	G	T	0.4932	0.0373
rs11627084	LC_IGP_R26	IgG1_G2N/IgG1_G1N	14	65978836	1	1.30E-12	1.65E-08	+	0.244445	0.2062	+	G	T	0.477861111	0.0341851	+	G	T	0.4932	0.0365
rs11627084	LC_IGP148	IgG2 FG0n total/G0n	14	65978836	1	5.36E-09	9.88E-14	+	0.201453	0.2777	+	G	T	0.47545782	0.0343354	+	G	T	0.4932	0.0373
rs11627084	LC_IGP97	IgG2_G0	14	65978836	1	1.25E-10	6.61E-14	-	-0.220782	-0.2801	+	G	T	0.476540342	0.0340984	+	G	T	0.4932	0.0374
rs11627084	LC_IGP_R41	IgG2_G0F/IgG2_G0	14	65978836	1	4.19E-08	4.34E-13	+	0.188437	0.269	+	G	T	0.476540342	0.0342052	+	G	T	0.4932	0.0371
rs11627084	LC_IGP138	IgG2_G0n	14	65978836	1	7.59E-11	4.94E-14	-	-0.224191	-0.2819	+	G	T	0.47545782	0.0342156	+	G	T	0.4932	0.0374
rs11627084	LC_IGP_R64	IgG2_G2/IgG2_G1	14	65978836	1	4.92E-09	1.73E-12	+	0.202201	0.2587	+	G	T	0.475593635	0.0343795	+	G	T	0.4932	0.0367
rs10483780	LC_IGP78	IgG1 BG0n/G0n	14	65980170	1	4.39E-14	8.34E-10	-	-0.260242	-0.2299	+	C	T	0.482896221	0.0341648	+	C	T	0.493	0.0374
rs10483780	LC_IGP79	IgG1 BG1n/G1n	14	65980170	1	3.71E-13	1.05E-11	-	-0.251232	-0.2545	+	C	T	0.482896221	0.0342888	+	C	T	0.493	0.0374
rs10483780	LC_IGP32	IgG1 BG1S1/(BG1+BG1S1)	14	65980170	1	9.47E-09	4.41E-12	+	0.197065	0.2536	+	C	T	0.48389365	0.0341528	+	C	T	0.493	0.0366
rs10483780	LC_IGP77	IgG1 Bn	14	65980170	1	3.61E-13	2.40E-11	-	-0.251403	-0.2512	+	C	T	0.482896221	0.0342951	+	C	T	0.493	0.0376
rs10483780	LC_IGP86	IgG1 Bn/Fn total	14	65980170	1	5.07E-14	1.39E-11	-	-0.260202	-0.2543	+	C	T	0.482896221	0.0342467	+	C	T	0.493	0.0376
rs10483780	LC_IGP84	IgG1 FBn/Bn total	14	65980170	1	2.67E-15	2.62E-16	+	0.273483	0.3104	+	C	T	0.482896221	0.0342545	+	C	T	0.493	0.0379
rs10483780	LC_IGP62	IgG1 FG0n total/G0n	14	65980170	1	8.81E-18	2.53E-12	+	0.294969	0.2641	+	C	T	0.482896221	0.0339468	+	C	T	0.493	0.0377
rs10483780	LC_IGP63	IgG1 FG1n total/G1n	14	65980170	1	1.71E-17	1.82E-14	+	0.293265	0.2888	+	C	T	0.482896221	0.0340607	+	C	T	0.493	0.0377
rs10483780	LC_IGP64	IgG1 FG2n total/G2n	14	65980170	1	6.46E-14	3.16E-15	+	0.259252	0.2955	+	C	T	0.482896221	0.0342683	+	C	T	0.493	0.0375
rs10483780	LC_IGP68	IgG1 FG2n/G2n	14	65980170	1	2.29E-10	2.65E-08	+	0.219704	0.2076	+	C	T	0.482896221	0.0344279	+	C	T	0.493	0.0373
rs10483780	LC_IGP61	IgG1 Fn total	14	65980170	1	5.74E-17	4.09E-14	+	0.288284	0.2851	+	C	T	0.482896221	0.0340621	+	C	T	0.493	0.0377
rs10483780	LC_IGP81	IgG1 Fn/Bn	14	65980170	1	1.72E-13	1.36E-10	+	0.254654	0.2404	+	C	T	0.482896221	0.0342608	+	C	T	0.493	0.0374
rs10483780	LC_IGP21	IgG1 Fucosylation	14	65980170	1	1.93E-15	5.99E-13	+	0.273275	0.2712	+	C	T	0.482376079	0.0340514	+	C	T	0.493	0.0377
rs10483780	LC_IGP28	IgG1 G1S1/(G1+G1S1)	14	65980170	1	2.04E-10	3.45E-10	+	0.21795	0.2268	+	C	T	0.482495107	0.0340618	+	C	T	0.493	0.0361
rs10483780	LC_IGP27	IgG1 GS1/(G0+G+GS1)	14	65980170	1	3.50E-09	2.08E-08	+	0.202871	0.2061	+	C	T	0.482495107	0.034161	+	C	T	0.493	0.0368

rs10483780	LC_IGP11	IgG1_G0	14	65980170	1	2.17E-19	2.10E-14	-	-0.306471	-0.2867	+	C	T	0.48282193	0.0336098	+	C	T	0.493	0.0375
rs10483780	LC_IGP_R1	IgG1_G0F/IgG1_G0	14	65980170	1	6.74E-17	6.24E-12	+	0.284615	0.2585	+	C	T	0.48282193	0.0337114	+	C	T	0.493	0.0376
rs10483780	LC_IGP_R4	IgG1_G0FN/IgG1_G0N	14	65980170	1	1.20E-14	2.64E-13	+	0.264755	0.2766	+	C	T	0.483390998	0.0339939	+	C	T	0.493	0.0378
rs10483780	LC_IGP52	IgG1_G0n	14	65980170	1	2.15E-19	1.99E-14	-	-0.308709	-0.2875	+	C	T	0.482896221	0.0338453	+	C	T	0.493	0.0376
rs10483780	LC_IGP14	IgG1_G0N	14	65980170	1	1.66E-16	3.41E-11	-	-0.28244	-0.2474	+	C	T	0.483390998	0.0338977	+	C	T	0.493	0.0373
rs10483780	LC_IGP55	IgG1_G0Nn	14	65980170	1	4.24E-17	3.70E-11	-	-0.289432	-0.2472	+	C	T	0.482896221	0.0340498	+	C	T	0.493	0.0374
rs10483780	LC_IGP12	IgG1_G1	14	65980170	1	2.50E-16	1.31E-13	-	-0.280029	-0.2791	+	C	T	0.48282193	0.033813	+	C	T	0.493	0.0377
rs10483780	LC_IGP_R2	IgG1_G1F/IgG1_G1	14	65980170	1	2.70E-17	1.57E-13	+	0.288958	0.2776	+	C	T	0.48282193	0.0337814	+	C	T	0.493	0.0376
rs10483780	LC_IGP_R5	IgG1_G1FN/IgG1_G1N	14	65980170	1	1.79E-14	1.59E-17	+	0.263956	0.3212	+	C	T	0.483933272	0.0341202	+	C	T	0.493	0.0377
rs10483780	LC_IGP53	IgG1_G1n	14	65980170	1	2.36E-15	2.41E-13	-	-0.273087	-0.2758	+	C	T	0.482896221	0.0341369	+	C	T	0.493	0.0377
rs10483780	LC_IGP15	IgG1_G1N	14	65980170	1	1.46E-11	1.39E-10	-	-0.232847	-0.2403	+	C	T	0.483933272	0.034241	+	C	T	0.493	0.0374
rs10483780	LC_IGP56	IgG1_G1Nn	14	65980170	1	6.39E-12	2.99E-10	-	-0.237814	-0.2356	+	C	T	0.482896221	0.0343545	+	C	T	0.493	0.0374
rs10483780	LC_IGP_R36	IgG1_G1NS1/IgG1_G1N	14	65980170	1	8.21E-09	5.10E-12	+	0.197932	0.2531	+	C	T	0.48389365	0.0341579	+	C	T	0.493	0.0367
rs10483780	LC_IGP_R32	IgG1_G1S1/IgG1_G1	14	65980170	1	3.66E-10	5.19E-10	+	0.21493	0.2248	+	C	T	0.482495107	0.0340788	+	C	T	0.493	0.0362
rs10483780	LC_IGP_R3	IgG1_G2F/IgG1_G2	14	65980170	1	1.93E-15	9.99E-15	+	0.272055	0.2895	+	C	T	0.482505807	0.0339027	+	C	T	0.493	0.0374
rs10483780	LC_IGP_R8	IgG1_G2FS1/IgG1_G2S1	14	65980170	1	6.61E-10	7.29E-13	+	0.212173	0.2677	+	C	T	0.482211057	0.0341539	+	C	T	0.493	0.0373
rs10483780	LC_IGP_R26	IgG1_G2N/IgG1_G1N	14	65980170	1	3.52E-12	1.63E-08	+	0.239218	0.2063	+	C	T	0.483576852	0.0341314	+	C	T	0.493	0.0365
rs10483780	LC_IGP148	IgG2 FG0n total/G0n	14	65980170	1	8.02E-09	9.95E-14	+	0.198802	0.2776	+	C	T	0.481467365	0.0342846	+	C	T	0.493	0.0373
rs10483780	LC_IGP97	IgG2_G0	14	65980170	1	1.07E-10	6.59E-14	-	-0.221232	-0.28	+	C	T	0.482505807	0.0340387	+	C	T	0.493	0.0374
rs10483780	LC_IGP_R41	IgG2_G0F/IgG2_G0	14	65980170	1	4.52E-08	4.36E-13	+	0.187656	0.2689	+	C	T	0.482505807	0.0341502	+	C	T	0.493	0.0371
rs10483780	LC_IGP138	IgG2_G0n	14	65980170	1	6.85E-11	4.92E-14	-	-0.224326	-0.2818	+	C	T	0.481467365	0.0341546	+	C	T	0.493	0.0374
rs10483780	LC_IGP_R64	IgG2_G2/IgG2_G1	14	65980170	1	3.02E-09	1.72E-12	+	0.204616	0.2587	+	C	T	0.481566401	0.034311	+	C	T	0.493	0.0367
rs12890978	LC_IGP78	IgG1 BG0n/G0n	14	65982890	1	3.20E-13	7.24E-10	-	-0.252692	-0.2304	+	T	C	0.462996902	0.0343919	+	T	C	0.4926	0.0374
rs12890978	LC_IGP79	IgG1 BG1n/G1n	14	65982890	1	8.85E-13	9.27E-12	-	-0.248536	-0.2548	+	T	C	0.462996902	0.0344932	+	T	C	0.4926	0.0374
rs12890978	LC_IGP32	IgG1 BG1S1/(BG1+BG1S1)	14	65982890	1	4.23E-08	3.45E-12	+	0.18925	0.2546	+	T	C	0.464092478	0.0343622	+	T	C	0.4926	0.0366
rs12890978	LC_IGP77	IgG1 Bn	14	65982890	1	1.83E-12	2.13E-11	-	-0.245141	-0.2515	+	T	C	0.462996902	0.0345154	+	T	C	0.4926	0.0376
rs12890978	LC_IGP86	IgG1 Bn/Fn total	14	65982890	1	2.85E-13	1.22E-11	-	-0.253797	-0.2546	+	T	C	0.462996902	0.0344689	+	T	C	0.4926	0.0376
rs12890978	LC_IGP84	IgG1 FBn/Bn total	14	65982890	1	3.46E-14	2.08E-16	+	0.263854	0.311	+	T	C	0.462996902	0.0344944	+	T	C	0.4926	0.0378
rs12890978	LC_IGP62	IgG1 FG0n total/G0n	14	65982890	1	6.88E-17	2.02E-12	+	0.288479	0.2648	+	T	C	0.462996902	0.0341741	+	T	C	0.4926	0.0377
rs12890978	LC_IGP63	IgG1 FG1n total/G1n	14	65982890	1	5.12E-17	1.43E-14	+	0.290508	0.2895	+	T	C	0.462996902	0.0342687	+	T	C	0.4926	0.0376
rs12890978	LC_IGP64	IgG1 FG2n total/G2n	14	65982890	1	3.50E-13	2.46E-15	+	0.252979	0.2962	+	T	C	0.462996902	0.0344898	+	T	C	0.4926	0.0374
rs12890978	LC_IGP68	IgG1 FG2n/G2n	14	65982890	1	4.22E-10	2.33E-08	+	0.217628	0.2082	+	T	C	0.462996902	0.0346276	+	T	C	0.4926	0.0373
rs12890978	LC_IGP61	IgG1 Fn total	14	65982890	1	4.17E-16	3.18E-14	+	0.281814	0.2859	+	T	C	0.462996902	0.0342886	+	T	C	0.4926	0.0377
rs12890978	LC_IGP81	IgG1 Fn/Bn	14	65982890	1	7.84E-13	1.20E-10	+	0.249021	0.2407	+	T	C	0.462996902	0.0344786	+	T	C	0.4926	0.0374
rs12890978	LC_IGP21	IgG1 Fucosylation	14	65982890	1	4.15E-14	4.84E-13	+	0.261397	0.2719	+	T	C	0.46226233	0.0342837	+	T	C	0.4926	0.0376
rs12890978	LC_IGP28	IgG1 G1S1/(G1+G1S1)	14	65982890	1	8.87E-11	2.35E-10	+	0.223329	0.2286	+	T	C	0.462541284	0.0342104	+	T	C	0.4926	0.0361
rs12890978	LC_IGP26	IgG1 GS1/(G+GS1)	14	65982890	1	2.31E-08	1.04E-09	+	0.19317	0.2257	+	T	C	0.462541284	0.034406	+	T	C	0.4926	0.037
rs12890978	LC_IGP27	IgG1 GS1/(G0+G+GS1)	14	65982890	1	7.64E-10	1.94E-08	+	0.21226	0.2063	+	T	C	0.462541284	0.034296	+	T	C	0.4926	0.0367
rs12890978	LC_IGP11	IgG1_G0	14	65982890	1	3.45E-19	1.74E-14	-	-0.306459	-0.2872	+	T	C	0.462587049	0.0338041	+	T	C	0.4926	0.0374
rs12890978	LC_IGP_R1	IgG1_G0F/IgG1_G0	14	65982890	1	1.47E-16	4.95E-12	+	0.28307	0.2593	+	T	C	0.462587049	0.0339128	+	T	C	0.4926	0.0375
rs12890978	LC_IGP_R4	IgG1_G0FN/IgG1_G0N	14	65982890	1	9.45E-14	2.14E-13	+	0.257119	0.2773	+	T	C	0.463110839	0.0342218	+	T	C	0.4926	0.0378
rs12890978	LC_IGP52	IgG1_G0n	14	65982890	1	6.11E-19	1.63E-14	-	-0.306506	-0.2881	+	T	C	0.462996902	0.0340508	+	T	C	0.4926	0.0375
rs12890978	LC_IGP14	IgG1_G0N	14	65982890	1	2.27E-16	3.16E-11	-	-0.282718	-0.2475	+	T	C	0.463110839	0.0340889	+	T	C	0.4926	0.0373
rs12890978	LC_IGP55	IgG1_G0Nn	14	65982890	1	2.32E-16	3.41E-11	-	-0.284172	-0.2474	+	T	C	0.462996902	0.0342702	+	T	C	0.4926	0.0373
rs12890978	LC_IGP12	IgG1_G1	14	65982890	1	8.09E-16	1.00E-13	-	-0.276763	-0.28	+	T	C	0.462587049	0.034023	+	T	C	0.4926	0.0376
rs12890978	LC_IGP_R2	IgG1_G1F/IgG1_G1	14	65982890	1	2.93E-17	1.23E-13	+	0.290224	0.2784	+	T	C	0.462587049	0.0339688	+	T	C	0.4926	0.0376
rs12890978	LC_IGP_R5	IgG1_G1FN/IgG1_G1N	14	65982890	1	1.42E-13	1.25E-17	+	0.256119	0.3217	+	T	C	0.464180812	0.034341	+	T	C	0.4926	0.0376
rs12890978	LC_IGP53	IgG1_G1n	14	65982890	1	1.24E-14	1.84E-13	-	-0.267479	-0.2767	+	T	C	0.462996902	0.0343569	+	T	C	0.4926	0.0376
rs12890978	LC_IGP15	IgG1_G1N	14	65982890	1	5.80E-11	1.22E-10	-	-0.227123	-0.2407	+	T	C	0.464180812	0.034448	+	T	C	0.4926	0.0374
rs12890978	LC_IGP56	IgG1_G1Nn	14	65982890	1	2.28E-11	2.61E-10	-	-0.23279	-0.236	+	T	C	0.462996902	0.0345678	+	T	C	0.4926	0.0373
rs12890978	LC_IGP_R36	IgG1_G1NS1/IgG1_G1N	14	65982890	1	3.41E-08	4.03E-12	+	0.190589	0.254	+	T	C	0.464092478	0.0343659	+	T	C	0.4926	0.0366
rs12890978	LC_IGP_R32	IgG1_G1S1/IgG1_G1	14	65982890	1	1.61E-10	3.67E-10	+	0.22027	0.2264	+	T	C	0.462541284	0.0342278	+	T	C	0.4926	0.0361
rs12890978	LC_IGP_R3	IgG1_G2F/IgG1_G2	14	65982890	1	1.24E-14	7.65E-15	+	0.265481	0.2903	+	T	C	0.462564181	0.0341058	+	T	C	0.4926	0.0374
rs12890978	LC_IGP_R8	IgG1_G2FS1/IgG1_G2S1	14	65982890	1	3.80E-09	5.99E-13	+	0.203561	0.2683	+	T	C	0.462281613	0.0343572	+	T	C	0.4926	0.0373
rs12890978	LC_IGP_R26	IgG1_G2N/IgG1_G1N	14	65982890	1	4.73E-12	1.48E-08	+	0.239061	0.2066	+	T	C	0.463751235	0.0343181	+	T	C	0.4926	0.0365
rs12890978	LC_IGP97	IgG2_G0	14	65982890	1	1.32E-09	6.14E-14	-	-0.208971	-0.2799	+	T	C	0.462564181	0.0342556	+	T	C	0.4926	0.0373
rs12890978	LC_IGP138	IgG2_G0n	14	65982890	1	9.90E-10	4.52E-14	-	-0.211366	-0.2818	+	T	C	0.461378387	0.0343834	+	T	C	0.4926	0.0374
rs12890978	LC_IGP_R64	IgG2_G2/IgG2_G1	14	65982890	1	6.20E-09	1.65E-12	+	0.201551	0.2586	+	T	C	0.461600367	0.034498	+	T	C	0.4926	0.0366
rs7154536	LC_IGP84	IgG1 FBn/Bn total	14	65991737	1	1.13E-11	4.11E-08	+	0.239417	0.2099	+	G	A	0.527369269	0.0350085	+	G	A	0.5379	0.0382
rs7154536	LC_IGP63	IgG1 FG1n total/G1n	14	65991737	1	1.21E-13	4.10E-07	+	0.260485	0.1922	+	G	A	0.527369269	0.0348219	+	G	A	0.5379	0.038
rs7154536	LC_IGP64	IgG1 FG2n total/G2n	14	65991737	1	7.83E-11	2.53E-08	+	0.22914	0.2105	+	G	A	0.527369269	0.0349956	+	G	A	0.5379	0.0378
rs7154536	LC_IGP61	IgG1 Fn total	14	65991737	1	4.83E-13	4.08E-07	+	0.253889	0.1926	+	G	A	0.527369269	0.0348268	+	G	A	0.5379	0.038
rs7154536	LC_IGP21	IgG1 Fucosylation	14	65991737	1	2.63E-11	2.24E-06	+	0.233092	0.1795	+	G	A	0.526687423	0.0347224	+	G	A	0.5379	0.038
rs7154536	LC_IGP11	IgG1_G0	14	65991737	1	1.66E-15	1.08E-07	-	-0.275824	-0.2009	+	G	A	0.527577886	0.0342914	+	G	A	0.5379	0.0378
rs7154536	LC_IGP_R4	IgG1_G0FN/IgG1_G0N	14	65991737	1	9.90E-11	1.25E-06	+	0.226027	0.1852	+	G	A	0.527950704	0.0347134	+	G	A	0.5379	0.0382
rs7154536	LC_IGP52	IgG1_G0n	14	65991737	1	5.08E-16	1.35E-07	-	-0.283344	-0.1998	+	G	A	0.527369269	0.0345787	+	G	A	0.5379	0.0379
rs7154536	LC_IGP12	IgG1_G1	14	65991737	1	2.07E-12	5.46E-07	-	-0.244544	-0.1904	+	G	A	0.52727245	0.0345185	+	G	A	0.5379	0.038
rs7154536	LC_IGP_R2	IgG1_G1F/IgG1_G1	14	65991737	1	1.80E-13	1.01E-06	+	0.256044	0.1852	+	G	A	0.52727245	0.0344821	+	G	A	0.5379	0.0379
rs7154536	LC_IGP_R5	IgG1_G1FN/IgG1_G1N	14	65991737	1	4.82E-11	8.21E-09	+	0.230621	0.2192	+	G	A	0.528684809	0.0348289	+	G	A	0.5379	0.038
rs7154536	LC_IGP53	IgG1_G1n	14	65991737	1	1.45E-11	8.85E-07	-	-0.237378	-0.1866	+	G	A	0.527369269	0.0348961	+	G	A	0.5379	0.038
rs7154536	LC_IGP_R3	IgG1_G2F/IgG1_G2	14	65991737	1	9.19E-12	3.74E-08	+	0.237492	0.2075	+	G	A	0.52728912	0.0345767	+	G	A	0.5379	0.0377
rs7154536	LC_IGP_R8	IgG1_G2FS1/IgG1_G2S1	14	65991737	1	3.62E-08	1.97E-07	+	0.19225	0.1955	+	G	A	0.526967013	0.0347341	+	G	A	0.5379	0.0376
rs7154536	LC_IGP97	IgG2_G0	14	65991737	1	4.10E-09	4.27E-07	-	-0.204513	-0.1901	+	G	A	0.526983496	0.0345908	+	G	A	0.5379	0.0376
rs7154536	LC_IGP138	IgG2_G0n	14	65991737	1	1.10E-09	5.52E-07	-	-0.212797	-0.1884	+	G	A	0.525711207	0.0347166	+	G	A	0.5377	0.0376
rs2411816	LC_IGP78	IgG1 BG0n/G0n	14	65994880	1	7.91E-13	8.51E-10	-	-0.247826	-0.2302	+	A	G	0.467964064	0.0343193	+	A	G	0.4876	0.0375
rs2411816	LC_IGP79	IgG1 BG1n/G1n	14	65994880	1	1.85E-12	5.45E-12	-	-0.244397	-0.2585	+	A	G	0.467964064	0.0344168	+	A	G	0.4876	0.0375
rs2411816	LC_IGP32	IgG1 BG1S1/(BG1+BG1S1)	14	65994880	1	4.75E-08	5.31E-12	+	0.188056	0.2534	+	A	G	0.469036067	0.034275	+	A	G	0.4875	0.0367
rs2411816	LC_IGP77	IgG1 Bn	14	65994880	1	3.96E-12	1.53E-11	-	-0.240793	-0.2541	+	A	G	0.467964064	0.0344397	+	A	G	0.4876	0.0377
rs2411816	LC_IGP86	IgG1 Bn/Fn total	14	65994880	1	6.11E-13	8.41E-12	-	-0.249604	-0.2575	+	A	G	0.467964064	0.034393	+	A	G	0.4876	0.0377
rs2411816	LC_IGP84	IgG1 FBn/Bn total	14	65994880	1	5.45E-14	1.34E-16	+	0.261132	0.3141	+	A	G	0.467964064	0.0344121	+	A	G	0.4876	0.038
rs2411816	LC_IGP62	IgG1 FG0n total/G0n	14	65994880	1	1.47E-16	1.72E-12	+	0.284666	0.2666	+	A	G	0.467964064	0.034099	+	A	G	0.4876	0.0378
rs2411816	LC_IGP63	IgG1 FG1n total/G1n	14	65994880	1	7.06E-17	8.30E-15	+	0.288457	0.2931	+	A	G	0.467964064	0.0341841	+	A	G	0.4876	0.0378
rs2411816	LC_IGP64	IgG1 FG2n total/G2n	14	65994880	1	3.34E-13	1.47E-15	+	0.252516	0.2996	+	A	G	0.467964064	0.034397	+	A	G	0.4876	0.0376
rs2411816	LC_IGP68	IgG1 FG2n/G2n	14	65994880	1	6.92E-10	1.97E-08	+	0.21437	0.2099	+	A	G	0.467964064	0.0345458	+	A	G	0.4876	0.0374
rs2411816	LC_IGP61	IgG1 Fn total	14	65994880	1	5.92E-16	2.10E-14	+	0.279628	0.2889	+	A	G	0.467964064	0.0342046	+	A	G	0.4876	0.0378
rs2411816	LC_IGP81	IgG1 Fn/Bn	14	65994880	1	1.78E-12	9.58E-11	+	0.244486	0.2428	+	A	G	0.467964064	0.034404	+	A	G	0.4876	0.0375
rs2411816	LC_IGP21	IgG1 Fucosylation	14	65994880	1	5.81E-14	2.40E-13	+	0.259211	0.2763	+	A	G	0.467204069	0.034199	+	A	G	0.4876	0.0377
rs2411816	LC_IGP28	IgG1 G1S1/(G1+G1S1)	14	65994880	1	1.51E-10	1.71E-10	+	0.219994	0.2314	+	A	G	0.46744526	0.0341308	+	A	G	0.4876	0.0362
rs2411816	LC_IGP26	IgG1 GS1/(G+GS1)	14	65994880	1	2.59E-08	3.64E-10	+	0.191987	0.2327	+	A	G	0.46744526	0.0343172	+	A	G	0.4876	0.0371
rs2411816	LC_IGP27	IgG1 GS1/(G0+G+GS1)	14	65994880	1	1.08E-09	9.50E-09	+	0.20982	0.2115	+	A	G	0.46744526	0.0342124	+	A	G	0.4876	0.0369
rs2411816	LC_IGP11	IgG1_G0	14	65994880	1	1.52E-18	1.14E-14	-	-0.299938	-0.2902	+	A	G	0.46779047	0.0337222	+	A	G	0.4876	0.0376
rs2411816	LC_IGP_R1	IgG1_G0F/IgG1_G0	14	65994880	1	7.56E-16	4.44E-12	+	0.275511	0.2608	+	A	G	0.46779047	0.0338337	+	A	G	0.4876	0.0377
rs2411816	LC_IGP_R4	IgG1_G0FN/IgG1_G0N	14	65994880	1	2.14E-13	2.03E-13	+	0.252599	0.2785	+	A	G	0.468327006	0.0341253	+	A	G	0.4876	0.0379
rs2411816	LC_IGP52	IgG1_G0n	14	65994880	1	1.41E-18	1.17E-14	-	-0.302571	-0.2906	+	A	G	0.467964064	0.0339774	+	A	G	0.4876	0.0377
rs2411816	LC_IGP14	IgG1_G0N	14	65994880	1	1.29E-15	3.35E-11	-	-0.27466	-0.248	+	A	G	0.468327006	0.0340119	+	A	G	0.4876	0.0374
rs2411816	LC_IGP55	IgG1_G0Nn	14	65994880	1	6.23E-16	3.85E-11	-	-0.279363	-0.2474	+	A	G	0.467964064	0.0341997	+	A	G	0.4876	0.0374
rs2411816	LC_IGP12	IgG1_G1	14	65994880	1	2.32E-15	5.31E-14	-	-0.271484	-0.2841	+	A	G	0.46779047	0.0339317	+	A	G	0.4876	0.0378
rs2411816	LC_IGP_R2	IgG1_G1F/IgG1_G1	14	65994880	1	1.02E-16	7.37E-14	+	0.284318	0.2818	+	A	G	0.46779047	0.0338818	+	A	G	0.4876	0.0377
rs2411816	LC_IGP_R5	IgG1_G1FN/IgG1_G1N	14	65994880	1	2.06E-13	7.84E-18	+	0.253774	0.325	+	A	G	0.469112239	0.0342595	+	A	G	0.4876	0.0378
rs2411816	LC_IGP53	IgG1_G1n	14	65994880	1	1.52E-14	1.08E-13	-	-0.265872	-0.2803	+	A	G	0.467964064	0.0342698	+	A	G	0.4876	0.0377
rs2411816	LC_IGP15	IgG1_G1N	14	65994880	1	1.13E-10	6.72E-11	-	-0.223131	-0.245	+	A	G	0.469112239	0.0343722	+	A	G	0.4876	0.0375
rs2411816	LC_IGP56	IgG1_G1Nn	14	65994880	1	4.67E-11	1.59E-10	-	-0.228551	-0.2397	+	A	G	0.467964064	0.0344908	+	A	G	0.4876	0.0375
rs2411816	LC_IGP_R36	IgG1_G1NS1/IgG1_G1N	14	65994880	1	3.96E-08	6.09E-12	+	0.18919	0.2529	+	A	G	0.469036067	0.0342794	+	A	G	0.4875	0.0368
rs2411816	LC_IGP_R32	IgG1_G1S1/IgG1_G1	14	65994880	1	2.81E-10	2.54E-10	+	0.216785	0.2295	+	A	G	0.46744526	0.0341486	+	A	G	0.4876	0.0363
rs2411816	LC_IGP_R3	IgG1_G2F/IgG1_G2	14	65994880	1	1.07E-14	4.71E-15	+	0.265411	0.2936	+	A	G	0.467465159	0.0340126	+	A	G	0.4876	0.0375
rs2411816	LC_IGP_R8	IgG1_G2FS1/IgG1_G2S1	14	65994880	1	3.29E-09	1.76E-13	+	0.203833	0.2753	+	A	G	0.467179597	0.0342631	+	A	G	0.4876	0.0374
rs2411816	LC_IGP_R26	IgG1_G2N/IgG1_G1N	14	65994880	1	8.38E-12	1.16E-08	+	0.235658	0.2089	+	A	G	0.4687	0.0342401	+	A	G	0.4876	0.0366
rs2411816	LC_IGP148	IgG2 FG0n total/G0n	14	65994880	1	2.98E-08	5.82E-14	+	0.191556	0.2808	+	A	G	0.466314655	0.0343924	+	A	G	0.4875	0.0374
rs2411816	LC_IGP97	IgG2_G0	14	65994880	1	5.25E-10	3.31E-14	-	-0.213393	-0.284	+	A	G	0.467465159	0.0341462	+	A	G	0.4876	0.0374
rs2411816	LC_IGP138	IgG2_G0n	14	65994880	1	3.68E-10	2.67E-14	-	-0.216115	-0.2854	+	A	G	0.466314655	0.0342698	+	A	G	0.4875	0.0375
rs2411816	LC_IGP_R64	IgG2_G2/IgG2_G1	14	65994880	1	4.61E-09	1.38E-12	+	0.202701	0.2602	+	A	G	0.466507344	0.0343993	+	A	G	0.4876	0.0367
rs12890123	LC_IGP78	IgG1 BG0n/G0n	14	65998069	1	3.53E-13	7.14E-10	-	-0.25218	-0.2311	+	T	C	0.463059789	0.0343864	+	T	C	0.487	0.0375
rs12890123	LC_IGP79	IgG1 BG1n/G1n	14	65998069	1	9.75E-13	4.62E-12	-	-0.248029	-0.2592	+	T	C	0.463059789	0.0344876	+	T	C	0.487	0.0375
rs12890123	LC_IGP32	IgG1 BG1S1/(BG1+BG1S1)	14	65998069	1	4.70E-08	3.75E-12	+	0.188558	0.255	+	T	C	0.464156597	0.0343567	+	T	C	0.487	0.0367
rs12890123	LC_IGP77	IgG1 Bn	14	65998069	1	2.00E-12	1.31E-11	-	-0.244665	-0.2548	+	T	C	0.463059789	0.0345097	+	T	C	0.487	0.0377
rs12890123	LC_IGP86	IgG1 Bn/Fn total	14	65998069	1	3.13E-13	7.14E-12	-	-0.253314	-0.2582	+	T	C	0.463059789	0.0344632	+	T	C	0.487	0.0377
rs12890123	LC_IGP84	IgG1 FBn/Bn total	14	65998069	1	3.75E-14	1.01E-16	+	0.263434	0.3152	+	T	C	0.463059789	0.0344885	+	T	C	0.487	0.038
rs12890123	LC_IGP62	IgG1 FG0n total/G0n	14	65998069	1	7.64E-17	1.34E-12	+	0.287998	0.2677	+	T	C	0.463059789	0.0341687	+	T	C	0.487	0.0378
rs12890123	LC_IGP63	IgG1 FG1n total/G1n	14	65998069	1	5.54E-17	6.25E-15	+	0.290134	0.2943	+	T	C	0.463059789	0.0342627	+	T	C	0.487	0.0377

rs12890123	LC_IGP64	IgG1 FG2n total/G2n	14	65998069	1	3.66E-13	1.09E-15	+	0.252716	0.3008	+	T	C	0.463059789	0.0344831	+	T	C	0.487	0.0375
rs12890123	LC_IGP68	IgG1 FG2n/G2n	14	65998069	1	4.41E-10	1.70E-08	+	0.217338	0.2108	+	T	C	0.463059789	0.0346209	+	T	C	0.487	0.0374
rs12890123	LC_IGP61	IgG1 Fn total	14	65998069	1	4.54E-16	1.58E-14	+	0.281406	0.2901	+	T	C	0.463059789	0.0342828	+	T	C	0.487	0.0378
rs12890123	LC_IGP81	IgG1 Fn/Bn	14	65998069	1	8.60E-13	8.21E-11	+	0.248533	0.2435	+	T	C	0.463059789	0.034473	+	T	C	0.487	0.0375
rs12890123	LC_IGP21	IgG1 Fucosylation	14	65998069	1	4.41E-14	1.90E-13	+	0.261071	0.2773	+	T	C	0.462323674	0.0342775	+	T	C	0.487	0.0377
rs12890123	LC_IGP28	IgG1 G1S1/(G1+G1S1)	14	65998069	1	9.61E-11	1.07E-10	+	0.222868	0.2337	+	T	C	0.462604893	0.0342043	+	T	C	0.487	0.0362
rs12890123	LC_IGP26	IgG1 GS1/(G+GS1)	14	65998069	1	2.41E-08	2.81E-10	+	0.19289	0.234	+	T	C	0.462604893	0.034399	+	T	C	0.487	0.0371
rs12890123	LC_IGP27	IgG1 GS1/(G0+G+GS1)	14	65998069	1	8.03E-10	8.15E-09	+	0.211944	0.2123	+	T	C	0.462604893	0.0342893	+	T	C	0.487	0.0368
rs12890123	LC_IGP11	IgG1_G0	14	65998069	1	3.81E-19	8.71E-15	-	-0.306023	-0.2913	+	T	C	0.46265058	0.0337985	+	T	C	0.487	0.0376
rs12890123	LC_IGP_R1	IgG1_G0F/IgG1_G0	14	65998069	1	1.62E-16	3.45E-12	+	0.282625	0.2619	+	T	C	0.46265058	0.033907	+	T	C	0.487	0.0376
rs12890123	LC_IGP_R4	IgG1_G0FN/IgG1_G0N	14	65998069	1	1.04E-13	1.56E-13	+	0.25665	0.2797	+	T	C	0.463174219	0.034216	+	T	C	0.487	0.0379
rs12890123	LC_IGP52	IgG1_G0n	14	65998069	1	6.77E-19	8.94E-15	-	-0.306059	-0.2917	+	T	C	0.463059789	0.0340454	+	T	C	0.487	0.0376
rs12890123	LC_IGP14	IgG1_G0N	14	65998069	1	2.56E-16	2.89E-11	-	-0.282164	-0.2486	+	T	C	0.463174219	0.0340837	+	T	C	0.487	0.0374
rs12890123	LC_IGP55	IgG1_G0Nn	14	65998069	1	2.62E-16	3.33E-11	-	-0.283613	-0.2481	+	T	C	0.463059789	0.0342652	+	T	C	0.487	0.0374
rs12890123	LC_IGP12	IgG1_G1	14	65998069	1	8.65E-16	3.92E-14	-	-0.276425	-0.2854	+	T	C	0.46265058	0.0340167	+	T	C	0.487	0.0377
rs12890123	LC_IGP_R2	IgG1_G1F/IgG1_G1	14	65998069	1	3.14E-17	5.53E-14	+	0.289881	0.2831	+	T	C	0.46265058	0.0339626	+	T	C	0.487	0.0377
rs12890123	LC_IGP_R5	IgG1_G1FN/IgG1_G1N	14	65998069	1	1.56E-13	5.75E-18	+	0.255655	0.3261	+	T	C	0.464244772	0.0343352	+	T	C	0.487	0.0378
rs12890123	LC_IGP53	IgG1_G1n	14	65998069	1	1.32E-14	8.08E-14	-	-0.267137	-0.2816	+	T	C	0.463059789	0.0343507	+	T	C	0.487	0.0377
rs12890123	LC_IGP15	IgG1_G1N	14	65998069	1	6.36E-11	5.72E-11	-	-0.226607	-0.2457	+	T	C	0.464244772	0.0344421	+	T	C	0.487	0.0375
rs12890123	LC_IGP56	IgG1_G1Nn	14	65998069	1	2.50E-11	1.36E-10	-	-0.232291	-0.2405	+	T	C	0.463059789	0.034562	+	T	C	0.487	0.0374
rs12890123	LC_IGP_R36	IgG1_G1NS1/IgG1_G1N	14	65998069	1	3.81E-08	4.33E-12	+	0.189878	0.2545	+	T	C	0.464156597	0.0343605	+	T	C	0.487	0.0367
rs12890123	LC_IGP_R32	IgG1_G1S1/IgG1_G1	14	65998069	1	1.75E-10	1.65E-10	+	0.2198	0.2316	+	T	C	0.462604893	0.0342217	+	T	C	0.487	0.0362
rs12890123	LC_IGP_R3	IgG1_G2F/IgG1_G2	14	65998069	1	1.30E-14	3.47E-15	+	0.265207	0.2949	+	T	C	0.462627751	0.0340991	+	T	C	0.487	0.0374
rs12890123	LC_IGP_R8	IgG1_G2FS1/IgG1_G2S1	14	65998069	1	4.02E-09	1.39E-13	+	0.203207	0.2764	+	T	C	0.462345144	0.0343505	+	T	C	0.487	0.0374
rs12890123	LC_IGP_R26	IgG1_G2N/IgG1_G1N	14	65998069	1	5.28E-12	9.94E-09	+	0.238469	0.2097	+	T	C	0.463813889	0.0343129	+	T	C	0.487	0.0366
rs12890123	LC_IGP97	IgG2_G0	14	65998069	1	1.33E-09	3.06E-14	-	-0.208869	-0.2841	+	T	C	0.462627751	0.0342481	+	T	C	0.487	0.0374
rs12890123	LC_IGP138	IgG2_G0n	14	65998069	1	9.97E-10	2.41E-14	-	-0.211279	-0.2857	+	T	C	0.461440579	0.0343759	+	T	C	0.487	0.0375
rs12890123	LC_IGP_R64	IgG2_G2/IgG2_G1	14	65998069	1	6.43E-09	1.27E-12	+	0.201294	0.2605	+	T	C	0.461664015	0.0344909	+	T	C	0.487	0.0367
rs7153297	LC_IGP78	IgG1 BG0n/G0n	14	66003466	1	3.85E-13	5.05E-10	-	-0.251726	-0.236	+	A	T	0.463111834	0.0343809	+	A	T	0.4825	0.038
rs7153297	LC_IGP79	IgG1 BG1n/G1n	14	66003466	1	1.06E-12	2.49E-12	-	-0.247586	-0.2657	+	A	T	0.463111834	0.0344821	+	A	T	0.4825	0.0379
rs7153297	LC_IGP77	IgG1 Bn	14	66003466	1	2.16E-12	7.24E-12	-	-0.244246	-0.2612	+	A	T	0.463111834	0.034504	+	A	T	0.4825	0.0381
rs7153297	LC_IGP86	IgG1 Bn/Fn total	14	66003466	1	3.39E-13	4.01E-12	-	-0.252889	-0.2645	+	A	T	0.463111834	0.0344576	+	A	T	0.4825	0.0381
rs7153297	LC_IGP84	IgG1 FBn/Bn total	14	66003466	1	4.03E-14	6.83E-17	+	0.263062	0.3206	+	A	T	0.463111834	0.0344827	+	A	T	0.4825	0.0384
rs7153297	LC_IGP62	IgG1 FG0n total/G0n	14	66003466	1	8.37E-17	8.80E-13	+	0.287572	0.2733	+	A	T	0.463111834	0.0341634	+	A	T	0.4825	0.0382
rs7153297	LC_IGP63	IgG1 FG1n total/G1n	14	66003466	1	5.92E-17	3.41E-15	+	0.289805	0.3009	+	A	T	0.463111834	0.0342569	+	A	T	0.4825	0.0382
rs7153297	LC_IGP64	IgG1 FG2n total/G2n	14	66003466	1	3.80E-13	6.35E-16	+	0.252483	0.3071	+	A	T	0.463111834	0.0344767	+	A	T	0.4825	0.038
rs7153297	LC_IGP68	IgG1 FG2n/G2n	14	66003466	1	4.58E-10	8.42E-09	+	0.217084	0.2181	+	A	T	0.463111834	0.0346144	+	A	T	0.4825	0.0379
rs7153297	LC_IGP61	IgG1 Fn total	14	66003466	1	4.88E-16	8.90E-15	+	0.281047	0.2966	+	A	T	0.463111834	0.0342771	+	A	T	0.4825	0.0382
rs7153297	LC_IGP81	IgG1 Fn/Bn	14	66003466	1	9.31E-13	4.41E-11	+	0.248105	0.2501	+	A	T	0.463111834	0.0344674	+	A	T	0.4825	0.038
rs7153297	LC_IGP21	IgG1 Fucosylation	14	66003466	1	4.65E-14	1.06E-13	+	0.260783	0.2838	+	A	T	0.462374538	0.0342716	+	A	T	0.4825	0.0382
rs7153297	LC_IGP28	IgG1 G1S1/(G1+G1S1)	14	66003466	1	1.03E-10	1.25E-10	+	0.222461	0.2352	+	A	T	0.462657798	0.0341984	+	A	T	0.4825	0.0366
rs7153297	LC_IGP26	IgG1 GS1/(G+GS1)	14	66003466	1	2.50E-08	2.64E-10	+	0.192635	0.2368	+	A	T	0.462657798	0.0343923	+	A	T	0.4825	0.0375
rs7153297	LC_IGP27	IgG1 GS1/(G0+G+GS1)	14	66003466	1	8.40E-10	8.02E-09	+	0.211652	0.2146	+	A	T	0.462657798	0.0342829	+	A	T	0.4825	0.0372
rs7153297	LC_IGP11	IgG1_G0	14	66003466	1	4.16E-19	5.60E-15	-	-0.305633	-0.2966	+	A	T	0.462703421	0.033793	+	A	T	0.4825	0.038
rs7153297	LC_IGP_R1	IgG1_G0F/IgG1_G0	14	66003466	1	1.76E-16	2.14E-12	+	0.282229	0.2678	+	A	T	0.462703421	0.0339015	+	A	T	0.4825	0.0381
rs7153297	LC_IGP_R4	IgG1_G0FN/IgG1_G0N	14	66003466	1	1.12E-13	1.20E-13	+	0.256232	0.2842	+	A	T	0.463226883	0.0342103	+	A	T	0.4825	0.0383
rs7153297	LC_IGP52	IgG1_G0n	14	66003466	1	7.39E-19	5.39E-15	-	-0.305663	-0.2974	+	A	T	0.463111834	0.03404	+	A	T	0.4825	0.038
rs7153297	LC_IGP14	IgG1_G0N	14	66003466	1	2.85E-16	2.08E-11	-	-0.281672	-0.2532	+	A	T	0.463226883	0.0340786	+	A	T	0.4825	0.0378
rs7153297	LC_IGP55	IgG1_G0Nn	14	66003466	1	2.91E-16	2.29E-11	-	-0.283117	-0.2529	+	A	T	0.463111834	0.0342602	+	A	T	0.4825	0.0378
rs7153297	LC_IGP12	IgG1_G1	14	66003466	1	9.16E-16	2.15E-14	-	-0.276128	-0.292	+	A	T	0.462703421	0.0340106	+	A	T	0.4825	0.0382
rs7153297	LC_IGP_R2	IgG1_G1F/IgG1_G1	14	66003466	1	3.34E-17	2.94E-14	+	0.289581	0.2898	+	A	T	0.462703421	0.0339566	+	A	T	0.4825	0.0381
rs7153297	LC_IGP_R5	IgG1_G1FN/IgG1_G1N	14	66003466	1	1.68E-13	3.83E-18	+	0.255249	0.3317	+	A	T	0.464297663	0.0343295	+	A	T	0.4825	0.0382
rs7153297	LC_IGP53	IgG1_G1n	14	66003466	1	1.39E-14	4.25E-14	-	-0.266839	-0.2885	+	A	T	0.463111834	0.0343446	+	A	T	0.4825	0.0382
rs7153297	LC_IGP15	IgG1_G1N	14	66003466	1	6.87E-11	3.06E-11	-	-0.226156	-0.2524	+	A	T	0.464297663	0.0344364	+	A	T	0.4825	0.038
rs7153297	LC_IGP56	IgG1_G1Nn	14	66003466	1	2.70E-11	6.90E-11	-	-0.231853	-0.2474	+	A	T	0.463111834	0.0345563	+	A	T	0.4825	0.0379
rs7153297	LC_IGP_R36	IgG1_G1NS1/IgG1_G1N	14	66003466	1	4.20E-08	4.48E-12	+	0.189254	0.257	+	A	T	0.464209618	0.0343551	+	A	T	0.4825	0.0371
rs7153297	LC_IGP_R32	IgG1_G1S1/IgG1_G1	14	66003466	1	1.88E-10	2.04E-10	+	0.219386	0.2328	+	A	T	0.462657798	0.0342158	+	A	T	0.4825	0.0366
rs7153297	LC_IGP_R3	IgG1_G2F/IgG1_G2	14	66003466	1	1.36E-14	2.06E-15	+	0.264961	0.301	+	A	T	0.462680623	0.0340927	+	A	T	0.4825	0.0379
rs7153297	LC_IGP_R8	IgG1_G2FS1/IgG1_G2S1	14	66003466	1	4.20E-09	1.29E-13	+	0.202912	0.2802	+	A	T	0.462397984	0.034344	+	A	T	0.4825	0.0378
rs7153297	LC_IGP_R26	IgG1_G2N/IgG1_G1N	14	66003466	1	5.83E-12	6.39E-09	+	0.237945	0.2146	+	A	T	0.463865741	0.0343077	+	A	T	0.4825	0.0369
rs7153297	LC_IGP97	IgG2_G0	14	66003466	1	1.34E-09	3.45E-14	-	-0.208777	-0.287	+	A	T	0.462680623	0.0342409	+	A	T	0.4825	0.0379
rs7153297	LC_IGP138	IgG2_G0n	14	66003466	1	1.00E-09	2.76E-14	-	-0.211199	-0.2885	+	A	T	0.461492303	0.0343688	+	A	T	0.4824	0.0379
rs7153297	LC_IGP_R64	IgG2_G2/IgG2_G1	14	66003466	1	6.64E-09	1.08E-12	+	0.201067	0.2638	+	A	T	0.461716952	0.0344842	+	A	T	0.4825	0.037
rs2149841	LC_IGP78	IgG1 BG0n/G0n	14	66010319	1	1.03E-14	4.20E-10	-	-0.267846	-0.2349	+	T	C	0.472343556	0.0342983	+	T	C	0.492	0.0376
rs2149841	LC_IGP79	IgG1 BG1n/G1n	14	66010319	1	6.73E-14	5.30E-12	-	-0.260196	-0.2593	+	T	C	0.472343556	0.0344175	+	T	C	0.492	0.0376
rs2149841	LC_IGP32	IgG1 BG1S1/(BG1+BG1S1)	14	66010319	1	1.00E-08	1.78E-12	+	0.197671	0.2593	+	T	C	0.473397349	0.0343133	+	T	C	0.492	0.0368
rs2149841	LC_IGP31	IgG1 BGS1/(BG0+BG+BGS1)	14	66010319	1	1.57E-08	4.08E-12	+	0.195753	0.2577	+	T	C	0.473419264	0.0344475	+	T	C	0.492	0.0372
rs2149841	LC_IGP77	IgG1 Bn	14	66010319	1	8.52E-14	1.17E-11	-	-0.259186	-0.2562	+	T	C	0.472343556	0.0344294	+	T	C	0.492	0.0378
rs2149841	LC_IGP86	IgG1 Bn/Fn total	14	66010319	1	1.22E-14	6.93E-12	-	-0.267729	-0.2591	+	T	C	0.472343556	0.034381	+	T	C	0.492	0.0378
rs2149841	LC_IGP84	IgG1 FBn/Bn total	14	66010319	1	9.31E-16	1.24E-16	+	0.279236	0.315	+	T	C	0.472343556	0.0343967	+	T	C	0.492	0.038
rs2149841	LC_IGP62	IgG1 FG0n total/G0n	14	66010319	1	4.24E-18	1.12E-12	+	0.299198	0.2695	+	T	C	0.472343556	0.0340945	+	T	C	0.492	0.0379
rs2149841	LC_IGP63	IgG1 FG1n total/G1n	14	66010319	1	3.63E-18	7.66E-15	+	0.300684	0.2942	+	T	C	0.472343556	0.0341917	+	T	C	0.492	0.0378
rs2149841	LC_IGP64	IgG1 FG2n total/G2n	14	66010319	1	3.12E-14	1.50E-15	+	0.263737	0.3002	+	T	C	0.472343556	0.0344174	+	T	C	0.492	0.0376
rs2149841	LC_IGP68	IgG1 FG2n/G2n	14	66010319	1	5.73E-11	1.49E-08	+	0.227971	0.2123	+	T	C	0.472343556	0.034564	+	T	C	0.492	0.0375
rs2149841	LC_IGP61	IgG1 Fn total	14	66010319	1	2.94E-17	1.69E-14	+	0.292329	0.2908	+	T	C	0.472343556	0.0342115	+	T	C	0.492	0.0379
rs2149841	LC_IGP81	IgG1 Fn/Bn	14	66010319	1	4.04E-14	6.41E-11	+	0.262378	0.2457	+	T	C	0.472343556	0.0343948	+	T	C	0.492	0.0376
rs2149841	LC_IGP21	IgG1 Fucosylation	14	66010319	1	1.22E-15	2.21E-13	+	0.27635	0.2774	+	T	C	0.471562577	0.0341878	+	T	C	0.492	0.0378
rs2149841	LC_IGP28	IgG1 G1S1/(G1+G1S1)	14	66010319	1	1.00E-10	1.24E-10	+	0.222544	0.2332	+	T	C	0.471775229	0.0341901	+	T	C	0.492	0.0362
rs2149841	LC_IGP26	IgG1 GS1/(G+GS1)	14	66010319	1	2.78E-08	5.76E-10	+	0.191954	0.2302	+	T	C	0.471775229	0.0343867	+	T	C	0.492	0.0372
rs2149841	LC_IGP27	IgG1 GS1/(G0+G+GS1)	14	66010319	1	1.02E-09	1.21E-08	+	0.210558	0.2101	+	T	C	0.471775229	0.034279	+	T	C	0.492	0.0369
rs2149841	LC_IGP11	IgG1_G0	14	66010319	1	3.38E-20	1.03E-14	-	-0.314676	-0.2911	+	T	C	0.471809713	0.0337342	+	T	C	0.492	0.0376
rs2149841	LC_IGP_R1	IgG1_G0F/IgG1_G0	14	66010319	1	1.70E-17	2.65E-12	+	0.291383	0.2642	+	T	C	0.471809713	0.0338461	+	T	C	0.492	0.0378
rs2149841	LC_IGP_R4	IgG1_G0FN/IgG1_G0N	14	66010319	1	2.26E-15	1.22E-13	+	0.273133	0.2817	+	T	C	0.472352419	0.0341231	+	T	C	0.492	0.038
rs2149841	LC_IGP52	IgG1_G0n	14	66010319	1	5.97E-20	9.01E-15	-	-0.314876	-0.2924	+	T	C	0.472343556	0.0339809	+	T	C	0.492	0.0377
rs2149841	LC_IGP14	IgG1_G0N	14	66010319	1	4.39E-18	1.86E-11	-	-0.298069	-0.2516	+	T	C	0.472352419	0.033986	+	T	C	0.492	0.0374
rs2149841	LC_IGP55	IgG1_G0Nn	14	66010319	1	4.42E-18	1.94E-11	-	-0.299661	-0.2516	+	T	C	0.472343556	0.0341658	+	T	C	0.492	0.0375
rs2149841	LC_IGP12	IgG1_G1	14	66010319	1	7.26E-17	5.23E-14	-	-0.286329	-0.2849	+	T	C	0.471809713	0.033951	+	T	C	0.492	0.0379
rs2149841	LC_IGP_R2	IgG1_G1F/IgG1_G1	14	66010319	1	3.24E-18	6.46E-14	+	0.298531	0.2832	+	T	C	0.471809713	0.0339012	+	T	C	0.492	0.0378
rs2149841	LC_IGP_R5	IgG1_G1FN/IgG1_G1N	14	66010319	1	5.66E-15	7.81E-18	+	0.270153	0.3256	+	T	C	0.473462792	0.034255	+	T	C	0.492	0.0378
rs2149841	LC_IGP53	IgG1_G1n	14	66010319	1	1.37E-15	9.28E-14	-	-0.27667	-0.2819	+	T	C	0.472343556	0.0342894	+	T	C	0.492	0.0378
rs2149841	LC_IGP15	IgG1_G1N	14	66010319	1	4.89E-12	7.02E-11	-	-0.239297	-0.2454	+	T	C	0.473462792	0.0343775	+	T	C	0.492	0.0376
rs2149841	LC_IGP56	IgG1_G1Nn	14	66010319	1	1.96E-12	1.45E-10	-	-0.244661	-0.2409	+	T	C	0.472343556	0.0344946	+	T	C	0.492	0.0376
rs2149841	LC_IGP_R36	IgG1_G1NS1/IgG1_G1N	14	66010319	1	8.49E-09	2.08E-12	+	0.198662	0.2588	+	T	C	0.473397349	0.034318	+	T	C	0.492	0.0368
rs2149841	LC_IGP_R32	IgG1_G1S1/IgG1_G1	14	66010319	1	1.77E-10	1.98E-10	+	0.219653	0.231	+	T	C	0.471775229	0.0342068	+	T	C	0.492	0.0363
rs2149841	LC_IGP_R3	IgG1_G2F/IgG1_G2	14	66010319	1	1.16E-15	4.85E-15	+	0.275305	0.2942	+	T	C	0.471792482	0.0340345	+	T	C	0.492	0.0376
rs2149841	LC_IGP_R8	IgG1_G2FS1/IgG1_G2S1	14	66010319	1	4.26E-10	6.44E-13	+	0.215424	0.2694	+	T	C	0.471504276	0.0342888	+	T	C	0.492	0.0375
rs2149841	LC_IGP_R26	IgG1_G2N/IgG1_G1N	14	66010319	1	1.02E-12	9.19E-09	+	0.246221	0.2106	+	T	C	0.473063272	0.0342666	+	T	C	0.492	0.0366
rs2149841	LC_IGP148	IgG2 FG0n total/G0n	14	66010319	1	1.37E-08	5.34E-14	+	0.196562	0.2819	+	T	C	0.470669643	0.0344445	+	T	C	0.492	0.0375
rs2149841	LC_IGP97	IgG2_G0	14	66010319	1	3.28E-10	4.55E-14	-	-0.216319	-0.283	+	T	C	0.471792482	0.0342043	+	T	C	0.492	0.0375
rs2149841	LC_IGP138	IgG2_G0n	14	66010319	1	2.12E-10	3.19E-14	-	-0.219462	-0.2852	+	T	C	0.470669643	0.0343262	+	T	C	0.492	0.0376
rs2149841	LC_IGP_R64	IgG2_G2/IgG2_G1	14	66010319	1	7.32E-09	1.13E-12	+	0.20045	0.2612	+	T	C	0.470839963	0.034476	+	T	C	0.492	0.0367
rs72714483	LC_IGP61	IgG1 Fn total	14	66012624	1	3.56E-08	1.14E-06	-	-0.239989	-0.2508	+	A	G	0.200788226	0.0433336	+	A	G	0.1648	0.0515
rs11621680	LC_IGP78	IgG1 BG0n/G0n	14	66014681	1	8.63E-12	1.10E-09	-	-0.2349	-0.2324	+	G	T	0.484728005	0.03415	+	G	T	0.5087	0.0381
rs11621680	LC_IGP79	IgG1 BG1n/G1n	14	66014681	1	1.24E-11	2.54E-12	-	-0.233673	-0.2668	+	G	T	0.484728005	0.0342369	+	G	T	0.5087	0.0381
rs11621680	LC_IGP77	IgG1 Bn	14	66014681	1	2.07E-11	1.07E-11	-	-0.231189	-0.2602	+	G	T	0.484728005	0.0342544	+	G	T	0.5087	0.0383
rs11621680	LC_IGP86	IgG1 Bn/Fn total	14	66014681	1	3.00E-12	6.62E-12	-	-0.240532	-0.263	+	G	T	0.484728005	0.0342069	+	G	T	0.5087	0.0383
rs11621680	LC_IGP84	IgG1 FBn/Bn total	14	66014681	1	2.87E-13	6.99E-16	+	0.251995	0.3115	+	G	T	0.484728005	0.0342273	+	G	T	0.5087	0.0386
rs11621680	LC_IGP62	IgG1 FG0n total/G0n	14	66014681	1	3.08E-16	2.82E-12	+	0.279876	0.2684	+	G	T	0.484728005	0.0338968	+	G	T	0.5087	0.0384
rs11621680	LC_IGP63	IgG1 FG1n total/G1n	14	66014681	1	2.78E-16	7.63E-15	+	0.281117	0.2984	+	G	T	0.484728005	0.0339946	+	G	T	0.5087	0.0384
rs11621680	LC_IGP64	IgG1 FG2n total/G2n	14	66014681	1	2.24E-13	1.28E-15	+	0.252737	0.3052	+	G	T	0.484728005	0.0341693	+	G	T	0.5087	0.0382
rs11621680	LC_IGP68	IgG1 FG2n/G2n	14	66014681	1	8.72E-10	1.55E-09	+	0.211754	0.2295	+	G	T	0.484728005	0.0343302	+	G	T	0.5087	0.038
rs11621680	LC_IGP61	IgG1 Fn total	14	66014681	1	9.70E-16	2.22E-14	+	0.275812	0.2935	+	G	T	0.484728005	0.0339967	+	G	T	0.5087	0.0384
rs11621680	LC_IGP81	IgG1 Fn/Bn	14	66014681	1	8.31E-12	3.39E-11	+	0.235544	0.2527	+	G	T	0.484728005	0.0342167	+	G	T	0.5087	0.0381
rs11621680	LC_IGP21	IgG1 Fucosylation	14	66014681	1	8.86E-14	2.05E-13	+	0.255656	0.2818	+	G	T	0.484496301	0.0339855	+	G	T	0.5087	0.0384
rs11621680	LC_IGP28	IgG1 G1S1/(G1+G1S1)	14	66014681	1	3.01E-11	2.36E-10	+	0.22671	0.2331	+	G	T	0.484614373	0.033877	+	G	T	0.5087	0.0368
rs11621680	LC_IGP26	IgG1 GS1/(G+GS1)	14	66014681	1	1.04E-08	2.05E-09	+	0.19607	0.226	+	G	T	0.484614373	0.0340765	+	G	T	0.5087	0.0377
rs11621680	LC_IGP27	IgG1 GS1/(G0+G+GS1)	14	66014681	1	3.92E-10	6.45E-08	+	0.21387	0.2024	+	G	T	0.484614373	0.0339702	+	G	T	0.5087	0.0374
rs11621680	LC_IGP11	IgG1_G0	14	66014681	1	7.02E-19	4.55E-14	-	-0.300871	-0.288	+	G	T	0.484938607	0.03349	+	G	T	0.5087	0.0382
rs11621680	LC_IGP_R1	IgG1_G0F/IgG1_G0	14	66014681	1	6.19E-16	5.38E-12	+	0.274558	0.2642	+	G	T	0.484938607	0.0336123	+	G	T	0.5087	0.0383

rs11621680	LC_IGP_R4	IgG1_G0FN/IgG1_G0N	14	66014681	1	3.53E-12	5.90E-13	+	0.238023	0.2775	+	G	T	0.485515003	0.0339652	+	G	T	0.5087	0.0385
rs11621680	LC_IGP52	IgG1_G0n	14	66014681	1	8.97E-19	4.03E-14	-	-0.302319	-0.2892	+	G	T	0.484728005	0.0337515	+	G	T	0.5087	0.0383
rs11621680	LC_IGP14	IgG1_G0N	14	66014681	1	3.34E-14	9.39E-11	-	-0.25914	-0.246	+	G	T	0.485515003	0.0338616	+	G	T	0.5087	0.038
rs11621680	LC_IGP55	IgG1_G0Nn	14	66014681	1	6.69E-15	9.38E-11	-	-0.267665	-0.2463	+	G	T	0.484728005	0.0340309	+	G	T	0.5087	0.038
rs11621680	LC_IGP12	IgG1_G1	14	66014681	1	1.95E-15	5.87E-14	-	-0.270453	-0.2884	+	G	T	0.484938607	0.0337102	+	G	T	0.5087	0.0384
rs11621680	LC_IGP_R2	IgG1_G1F/IgG1_G1	14	66014681	1	1.37E-16	4.11E-14	+	0.281349	0.2895	+	G	T	0.484938607	0.03367	+	G	T	0.5087	0.0383
rs11621680	LC_IGP_R5	IgG1_G1FN/IgG1_G1N	14	66014681	1	8.98E-13	3.18E-17	+	0.245456	0.324	+	G	T	0.485760148	0.034077	+	G	T	0.5087	0.0384
rs11621680	LC_IGP53	IgG1_G1n	14	66014681	1	2.23E-14	1.02E-13	-	-0.262514	-0.2854	+	G	T	0.484728005	0.0340592	+	G	T	0.5087	0.0384
rs11621680	LC_IGP15	IgG1_G1N	14	66014681	1	4.31E-10	3.22E-11	-	-0.214717	-0.2531	+	G	T	0.485760148	0.0341863	+	G	T	0.5087	0.0382
rs11621680	LC_IGP56	IgG1_G1Nn	14	66014681	1	2.31E-10	6.40E-11	-	-0.218846	-0.2489	+	G	T	0.484728005	0.0343041	+	G	T	0.5087	0.0381
rs11621680	LC_IGP_R32	IgG1_G1S1/IgG1_G1	14	66014681	1	5.77E-11	3.70E-10	+	0.223501	0.2308	+	G	T	0.484614373	0.0338953	+	G	T	0.5087	0.0368
rs11621680	LC_IGP_R3	IgG1_G2F/IgG1_G2	14	66014681	1	5.79E-15	3.04E-15	+	0.266293	0.3006	+	G	T	0.484623778	0.0337799	+	G	T	0.5087	0.0381
rs11621680	LC_IGP_R8	IgG1_G2FS1/IgG1_G2S1	14	66014681	1	7.70E-09	7.71E-14	+	0.197716	0.284	+	G	T	0.484327734	0.0340573	+	G	T	0.5087	0.038
rs11621680	LC_IGP_R26	IgG1_G2N/IgG1_G1N	14	66014681	1	2.64E-11	8.48E-09	+	0.228565	0.2142	+	G	T	0.485401852	0.0340495	+	G	T	0.5087	0.0372
rs11621680	LC_IGP97	IgG2_G0	14	66014681	1	2.49E-10	5.29E-14	-	-0.215923	-0.2865	+	G	T	0.484623778	0.03391	+	G	T	0.5087	0.0381
rs11621680	LC_IGP138	IgG2_G0n	14	66014681	1	2.00E-10	3.58E-14	-	-0.217856	-0.2889	+	G	T	0.483590209	0.0340299	+	G	T	0.5087	0.0381
rs11621680	LC_IGP_R64	IgG2_G2/IgG2_G1	14	66014681	1	6.26E-10	4.81E-12	+	0.212337	0.2574	+	G	T	0.483686965	0.0341311	+	G	T	0.5087	0.0372
rs72714484	LC_IGP61	IgG1 Fn total	14	66016247	1	3.78E-08	1.15E-06	-	-0.239517	-0.2506	+	G	A	0.200797522	0.0433323	+	G	A	0.1647	0.0515
rs78230848	LC_IGP78	IgG1 BG0n/G0n	14	66016384	1	1.49E-12	9.06E-10	-	-0.244391	-0.2316	+	A	G	0.468363073	0.0342671	+	A	G	0.4851	0.0378
rs78230848	LC_IGP79	IgG1 BG1n/G1n	14	66016384	1	3.41E-12	3.98E-12	-	-0.24101	-0.2622	+	A	G	0.468363073	0.0343641	+	A	G	0.4851	0.0378
rs78230848	LC_IGP77	IgG1 Bn	14	66016384	1	6.99E-12	1.25E-11	-	-0.237585	-0.2571	+	A	G	0.468363073	0.034386	+	A	G	0.4851	0.038
rs78230848	LC_IGP86	IgG1 Bn/Fn total	14	66016384	1	1.11E-12	7.07E-12	-	-0.246329	-0.2604	+	A	G	0.468363073	0.0343401	+	A	G	0.4851	0.038
rs78230848	LC_IGP84	IgG1 FBn/Bn total	14	66016384	1	9.42E-14	1.88E-16	+	0.258185	0.3148	+	A	G	0.468363073	0.034358	+	A	G	0.4851	0.0382
rs78230848	LC_IGP62	IgG1 FG0n total/G0n	14	66016384	1	2.95E-16	1.97E-12	+	0.281317	0.268	+	A	G	0.468363073	0.0340485	+	A	G	0.4851	0.0381
rs78230848	LC_IGP63	IgG1 FG1n total/G1n	14	66016384	1	1.21E-16	8.17E-15	+	0.285735	0.2955	+	A	G	0.468363073	0.0341304	+	A	G	0.4851	0.0381
rs78230848	LC_IGP64	IgG1 FG2n total/G2n	14	66016384	1	4.70E-13	1.55E-15	+	0.25046	0.3017	+	A	G	0.468363073	0.0343387	+	A	G	0.4851	0.0378
rs78230848	LC_IGP68	IgG1 FG2n/G2n	14	66016384	1	9.47E-10	1.20E-08	+	0.212257	0.2149	+	A	G	0.468363073	0.0344865	+	A	G	0.4851	0.0377
rs78230848	LC_IGP61	IgG1 Fn total	14	66016384	1	1.06E-15	2.14E-14	+	0.276692	0.2911	+	A	G	0.468363073	0.0341516	+	A	G	0.4851	0.0381
rs78230848	LC_IGP81	IgG1 Fn/Bn	14	66016384	1	3.22E-12	7.27E-11	+	0.241197	0.2462	+	A	G	0.468363073	0.0343509	+	A	G	0.4851	0.0378
rs78230848	LC_IGP21	IgG1 Fucosylation	14	66016384	1	8.94E-14	2.67E-13	+	0.256808	0.2779	+	A	G	0.467595253	0.034144	+	A	G	0.4851	0.038
rs78230848	LC_IGP28	IgG1 G1S1/(G1+G1S1)	14	66016384	1	2.53E-10	3.02E-10	+	0.216901	0.2296	+	A	G	0.467849541	0.0340755	+	A	G	0.4851	0.0365
rs78230848	LC_IGP26	IgG1 GS1/(G+GS1)	14	66016384	1	3.41E-08	6.38E-10	+	0.18998	0.2308	+	A	G	0.467849541	0.0342567	+	A	G	0.4851	0.0374
rs78230848	LC_IGP27	IgG1 GS1/(G0+G+GS1)	14	66016384	1	1.51E-09	1.76E-08	+	0.207607	0.2089	+	A	G	0.467849541	0.0341532	+	A	G	0.4851	0.0371
rs78230848	LC_IGP11	IgG1_G0	14	66016384	1	2.93E-18	1.72E-14	-	-0.296899	-0.2901	+	A	G	0.468194258	0.0336707	+	A	G	0.4851	0.0378
rs78230848	LC_IGP_R1	IgG1_G0F/IgG1_G0	14	66016384	1	1.41E-15	4.66E-12	+	0.272414	0.2626	+	A	G	0.468194258	0.0337814	+	A	G	0.4851	0.038
rs78230848	LC_IGP_R4	IgG1_G0FN/IgG1_G0N	14	66016384	1	3.88E-13	3.00E-13	+	0.249404	0.2785	+	A	G	0.468729333	0.0340721	+	A	G	0.4851	0.0382
rs78230848	LC_IGP52	IgG1_G0n	14	66016384	1	2.75E-18	1.65E-14	-	-0.299454	-0.291	+	A	G	0.468363073	0.0339267	+	A	G	0.4851	0.0379
rs78230848	LC_IGP14	IgG1_G0N	14	66016384	1	2.74E-15	4.30E-11	-	-0.27101	-0.2482	+	A	G	0.468729333	0.033962	+	A	G	0.4851	0.0376
rs78230848	LC_IGP55	IgG1_G0Nn	14	66016384	1	1.34E-15	4.75E-11	-	-0.275658	-0.2479	+	A	G	0.468363073	0.0341505	+	A	G	0.4851	0.0377
rs78230848	LC_IGP12	IgG1_G1	14	66016384	1	3.71E-15	5.22E-14	-	-0.268999	-0.2865	+	A	G	0.468194258	0.033876	+	A	G	0.4851	0.0381
rs78230848	LC_IGP_R2	IgG1_G1F/IgG1_G1	14	66016384	1	1.67E-16	6.80E-14	+	0.281816	0.2845	+	A	G	0.468194258	0.0338267	+	A	G	0.4851	0.038
rs78230848	LC_IGP_R5	IgG1_G1FN/IgG1_G1N	14	66016384	1	3.71E-13	1.08E-17	+	0.250603	0.3259	+	A	G	0.46951722	0.0342059	+	A	G	0.4851	0.038
rs78230848	LC_IGP53	IgG1_G1n	14	66016384	1	2.41E-14	1.00E-13	-	-0.263352	-0.283	+	A	G	0.468363073	0.0342142	+	A	G	0.4851	0.038
rs78230848	LC_IGP15	IgG1_G1N	14	66016384	1	2.00E-10	4.67E-11	-	-0.219708	-0.2489	+	A	G	0.46951722	0.0343181	+	A	G	0.4851	0.0378
rs78230848	LC_IGP56	IgG1_G1Nn	14	66016384	1	8.24E-11	1.05E-10	-	-0.225214	-0.244	+	A	G	0.468363073	0.0344369	+	A	G	0.4851	0.0378
rs78230848	LC_IGP_R32	IgG1_G1S1/IgG1_G1	14	66016384	1	4.72E-10	4.58E-10	+	0.213635	0.2276	+	A	G	0.467849541	0.0340934	+	A	G	0.4851	0.0365
rs78230848	LC_IGP_R3	IgG1_G2F/IgG1_G2	14	66016384	1	1.56E-14	5.01E-15	+	0.263276	0.2956	+	A	G	0.467869193	0.0339548	+	A	G	0.4851	0.0378
rs78230848	LC_IGP_R8	IgG1_G2FS1/IgG1_G2S1	14	66016384	1	4.72E-09	2.50E-13	+	0.201413	0.2757	+	A	G	0.467583384	0.0342044	+	A	G	0.4851	0.0377
rs78230848	LC_IGP_R26	IgG1_G2N/IgG1_G1N	14	66016384	1	1.66E-11	1.26E-08	+	0.231876	0.2097	+	A	G	0.469097531	0.0341894	+	A	G	0.4851	0.0368
rs78230848	LC_IGP148	IgG2 FG0n total/G0n	14	66016384	1	3.17E-08	1.09E-13	+	0.190833	0.2799	+	A	G	0.466710283	0.0343283	+	A	G	0.4851	0.0377
rs78230848	LC_IGP97	IgG2_G0	14	66016384	1	5.82E-10	7.30E-14	-	-0.212428	-0.2822	+	A	G	0.467869193	0.0340826	+	A	G	0.4851	0.0377
rs78230848	LC_IGP138	IgG2_G0n	14	66016384	1	4.02E-10	5.86E-14	-	-0.215231	-0.2836	+	A	G	0.466710283	0.0342065	+	A	G	0.4851	0.0378
rs78230848	LC_IGP_R64	IgG2_G2/IgG2_G1	14	66016384	1	5.90E-09	2.15E-12	+	0.200902	0.2593	+	A	G	0.466911873	0.0343379	+	A	G	0.4851	0.0369
rs11623662	LC_IGP79	IgG1 BG1n/G1n	14	66021192	1	5.73E-09	4.54E-07	-	-0.208129	-0.1961	+	G	T	0.588595725	0.03554	+	G	T	0.6138	0.0389
rs11623662	LC_IGP77	IgG1 Bn	14	66021192	1	3.81E-09	7.40E-07	-	-0.21056	-0.1933	+	G	T	0.588595725	0.0355385	+	G	T	0.6138	0.039
rs11623662	LC_IGP86	IgG1 Bn/Fn total	14	66021192	1	1.12E-09	5.41E-07	-	-0.217568	-0.1958	+	G	T	0.588595725	0.0355044	+	G	T	0.6138	0.0391
rs11623662	LC_IGP84	IgG1 FBn/Bn total	14	66021192	1	6.45E-11	3.39E-09	+	0.233597	0.2326	+	G	T	0.588595725	0.035515	+	G	T	0.6138	0.0394
rs11623662	LC_IGP62	IgG1 FG0n total/G0n	14	66021192	1	2.66E-12	1.21E-07	+	0.248491	0.2073	+	G	T	0.588595725	0.0352509	+	G	T	0.6138	0.0392
rs11623662	LC_IGP63	IgG1 FG1n total/G1n	14	66021192	1	2.03E-12	4.34E-09	+	0.250548	0.2297	+	G	T	0.588595725	0.035349	+	G	T	0.6138	0.0391
rs11623662	LC_IGP64	IgG1 FG2n total/G2n	14	66021192	1	6.70E-10	2.76E-09	+	0.220541	0.2314	+	G	T	0.588595725	0.0355106	+	G	T	0.6138	0.0389
rs11623662	LC_IGP61	IgG1 Fn total	14	66021192	1	3.95E-12	6.72E-09	+	0.247091	0.2271	+	G	T	0.588595725	0.0353385	+	G	T	0.6138	0.0392
rs11623662	LC_IGP81	IgG1 Fn/Bn	14	66021192	1	3.13E-09	1.45E-06	+	0.211596	0.1874	+	G	T	0.588595725	0.0355147	+	G	T	0.6138	0.0389
rs11623662	LC_IGP21	IgG1 Fucosylation	14	66021192	1	2.38E-11	3.32E-08	+	0.23704	0.2161	+	G	T	0.588158755	0.0352312	+	G	T	0.6138	0.0391
rs11623662	LC_IGP11	IgG1_G0	14	66021192	1	1.74E-14	2.89E-09	-	-0.269438	-0.2314	+	G	T	0.588267257	0.0348132	+	G	T	0.6138	0.039
rs11623662	LC_IGP_R1	IgG1_G0F/IgG1_G0	14	66021192	1	1.82E-12	1.34E-07	+	0.24785	0.2059	+	G	T	0.588267257	0.034895	+	G	T	0.6138	0.039
rs11623662	LC_IGP_R4	IgG1_G0FN/IgG1_G0N	14	66021192	1	5.73E-10	1.52E-07	+	0.219649	0.2064	+	G	T	0.589095836	0.0352265	+	G	T	0.6138	0.0393
rs11623662	LC_IGP52	IgG1_G0n	14	66021192	1	5.55E-14	3.05E-09	-	-0.266589	-0.2316	+	G	T	0.588595725	0.0351424	+	G	T	0.6138	0.039
rs11623662	LC_IGP14	IgG1_G0N	14	66021192	1	5.78E-11	1.64E-06	-	-0.231891	-0.186	+	G	T	0.589095836	0.0351693	+	G	T	0.6138	0.0388
rs11623662	LC_IGP55	IgG1_G0Nn	14	66021192	1	2.91E-11	1.64E-06	-	-0.236941	-0.1862	+	G	T	0.588595725	0.0353752	+	G	T	0.6138	0.0388
rs11623662	LC_IGP12	IgG1_G1	14	66021192	1	6.16E-12	1.17E-08	-	-0.242334	-0.2231	+	G	T	0.588572694	0.0349839	+	G	T	0.6138	0.0391
rs11623662	LC_IGP_R2	IgG1_G1F/IgG1_G1	14	66021192	1	2.00E-12	9.90E-09	+	0.247943	0.2238	+	G	T	0.588572694	0.0349746	+	G	T	0.6138	0.039
rs11623662	LC_IGP_R5	IgG1_G1FN/IgG1_G1N	14	66021192	1	2.05E-10	7.11E-10	+	0.226099	0.2412	+	G	T	0.589479397	0.0353383	+	G	T	0.6138	0.0391
rs11623662	LC_IGP53	IgG1_G1n	14	66021192	1	2.52E-11	1.80E-08	-	-0.2377	-0.2201	+	G	T	0.588595725	0.0353755	+	G	T	0.6138	0.0391
rs11623662	LC_IGP_R3	IgG1_G2F/IgG1_G2	14	66021192	1	6.25E-11	5.15E-09	+	0.230944	0.2269	+	G	T	0.588015587	0.0350893	+	G	T	0.6138	0.0388
rs9972106	LC_IGP79	IgG1 BG1n/G1n	14	66023131	1	6.66E-09	2.77E-07	-	-0.207409	-0.1998	+	T	A	0.587977695	0.0355727	+	T	A	0.6175	0.0389
rs9972106	LC_IGP77	IgG1 Bn	14	66023131	1	3.99E-09	4.41E-07	-	-0.210464	-0.1973	+	T	A	0.587977695	0.0355689	+	T	A	0.6175	0.0391
rs9972106	LC_IGP86	IgG1 Bn/Fn total	14	66023131	1	1.17E-09	3.08E-07	-	-0.217477	-0.2001	+	T	A	0.587977695	0.0355348	+	T	A	0.6175	0.0391
rs9972106	LC_IGP84	IgG1 FBn/Bn total	14	66023131	1	5.74E-11	1.71E-09	+	0.234408	0.2372	+	T	A	0.587977695	0.0355419	+	T	A	0.6175	0.0394
rs9972106	LC_IGP62	IgG1 FG0n total/G0n	14	66023131	1	2.42E-12	6.15E-08	+	0.249158	0.2122	+	T	A	0.587977695	0.035278	+	T	A	0.6175	0.0392
rs9972106	LC_IGP63	IgG1 FG1n total/G1n	14	66023131	1	2.06E-12	2.11E-09	+	0.250685	0.2345	+	T	A	0.587977695	0.0353785	+	T	A	0.6175	0.0392
rs9972106	LC_IGP64	IgG1 FG2n total/G2n	14	66023131	1	6.82E-10	1.33E-09	+	0.220623	0.2362	+	T	A	0.587977695	0.0355403	+	T	A	0.6175	0.0389
rs9972106	LC_IGP61	IgG1 Fn total	14	66023131	1	3.91E-12	3.16E-09	+	0.247346	0.2322	+	T	A	0.587977695	0.0353675	+	T	A	0.6175	0.0392
rs9972106	LC_IGP81	IgG1 Fn/Bn	14	66023131	1	3.38E-09	9.32E-07	+	0.21133	0.1908	+	T	A	0.587977695	0.0355457	+	T	A	0.6175	0.0389
rs9972106	LC_IGP21	IgG1 Fucosylation	14	66023131	1	2.47E-11	1.58E-08	+	0.237034	0.2212	+	T	A	0.587543773	0.0352611	+	T	A	0.6175	0.0391
rs9972106	LC_IGP11	IgG1_G0	14	66023131	1	1.34E-14	1.66E-09	-	-0.270815	-0.2351	+	T	A	0.587657911	0.034836	+	T	A	0.6175	0.039
rs9972106	LC_IGP_R1	IgG1_G0F/IgG1_G0	14	66023131	1	1.72E-12	6.66E-08	+	0.248321	0.211	+	T	A	0.587657911	0.0349222	+	T	A	0.6175	0.0391
rs9972106	LC_IGP_R4	IgG1_G0FN/IgG1_G0N	14	66023131	1	4.64E-10	9.10E-08	+	0.220989	0.2102	+	T	A	0.588484997	0.0352507	+	T	A	0.6175	0.0393
rs9972106	LC_IGP52	IgG1_G0n	14	66023131	1	4.33E-14	1.73E-09	-	-0.267939	-0.2353	+	T	A	0.587977695	0.0351662	+	T	A	0.6175	0.0391
rs9972106	LC_IGP14	IgG1_G0N	14	66023131	1	4.74E-11	1.29E-06	-	-0.233112	-0.188	+	T	A	0.588484997	0.0351938	+	T	A	0.6175	0.0388
rs9972106	LC_IGP55	IgG1_G0Nn	14	66023131	1	2.39E-11	1.27E-06	-	-0.238163	-0.1883	+	T	A	0.587977695	0.0354002	+	T	A	0.6175	0.0389
rs9972106	LC_IGP12	IgG1_G1	14	66023131	1	5.96E-12	4.94E-09	-	-0.24269	-0.229	+	T	A	0.587963348	0.0350116	+	T	A	0.6175	0.0392
rs9972106	LC_IGP_R2	IgG1_G1F/IgG1_G1	14	66023131	1	1.99E-12	4.71E-09	+	0.248173	0.2288	+	T	A	0.587963348	0.0350028	+	T	A	0.6175	0.0391
rs9972106	LC_IGP_R5	IgG1_G1FN/IgG1_G1N	14	66023131	1	2.12E-10	3.80E-10	+	0.226101	0.2453	+	T	A	0.588865929	0.0353678	+	T	A	0.6175	0.0392
rs9972106	LC_IGP53	IgG1_G1n	14	66023131	1	2.49E-11	7.80E-09	-	-0.23797	-0.2258	+	T	A	0.587977695	0.0354045	+	T	A	0.6175	0.0391
rs9972106	LC_IGP56	IgG1_G1Nn	14	66023131	1	4.24E-08	1.85E-06	-	-0.196153	-0.1853	+	T	A	0.587977695	0.0356189	+	T	A	0.6175	0.0389
rs9972106	LC_IGP_R3	IgG1_G2F/IgG1_G2	14	66023131	1	6.41E-11	2.44E-09	+	0.231	0.2319	+	T	A	0.587405868	0.0351184	+	T	A	0.6175	0.0389
rs11158601	LC_IGP78	IgG1 BG0n/G0n	14	66025363	1	5.73E-14	7.55E-10	-	-0.258878	-0.2311	+	G	A	0.477695167	0.0341458	+	G	A	0.4936	0.0376
rs11158601	LC_IGP79	IgG1 BG1n/G1n	14	66025363	1	2.95E-13	8.28E-12	-	-0.252093	-0.2565	+	G	A	0.477695167	0.0342594	+	G	A	0.4936	0.0375
rs11158601	LC_IGP32	IgG1 BG1S1/(BG1+BG1S1)	14	66025363	1	2.10E-08	3.11E-12	+	0.192284	0.2561	+	G	A	0.478729963	0.0341417	+	G	A	0.4936	0.0367
rs11158601	LC_IGP77	IgG1 Bn	14	66025363	1	3.82E-13	2.01E-11	-	-0.250963	-0.2528	+	G	A	0.477695167	0.0342717	+	G	A	0.4936	0.0377
rs11158601	LC_IGP86	IgG1 Bn/Fn total	14	66025363	1	5.67E-14	1.21E-11	-	-0.259526	-0.2557	+	G	A	0.477695167	0.0342244	+	G	A	0.4936	0.0377
rs11158601	LC_IGP84	IgG1 FBn/Bn total	14	66025363	1	3.03E-15	3.22E-16	+	0.272753	0.3102	+	G	A	0.477695167	0.0342326	+	G	A	0.4936	0.038
rs11158601	LC_IGP62	IgG1 FG0n total/G0n	14	66025363	1	2.24E-17	2.38E-12	+	0.291133	0.2651	+	G	A	0.477695167	0.0339419	+	G	A	0.4936	0.0378
rs11158601	LC_IGP63	IgG1 FG1n total/G1n	14	66025363	1	1.05E-17	1.55E-14	+	0.294933	0.2903	+	G	A	0.477695167	0.0340262	+	G	A	0.4936	0.0378
rs11158601	LC_IGP64	IgG1 FG2n total/G2n	14	66025363	1	5.10E-14	2.86E-15	+	0.260121	0.2967	+	G	A	0.477695167	0.0342387	+	G	A	0.4936	0.0376
rs11158601	LC_IGP68	IgG1 FG2n/G2n	14	66025363	1	1.51E-10	1.84E-08	+	0.221721	0.2106	+	G	A	0.477695167	0.0343945	+	G	A	0.4936	0.0374
rs11158601	LC_IGP61	IgG1 Fn total	14	66025363	1	9.26E-17	3.59E-14	+	0.286174	0.2866	+	G	A	0.477695167	0.0340476	+	G	A	0.4936	0.0378
rs11158601	LC_IGP81	IgG1 Fn/Bn	14	66025363	1	1.97E-13	1.04E-10	+	0.253846	0.2426	+	G	A	0.477695167	0.0342391	+	G	A	0.4936	0.0375
rs11158601	LC_IGP21	IgG1 Fucosylation	14	66025363	1	3.29E-15	4.68E-13	+	0.270693	0.2731	+	G	A	0.476880395	0.0340209	+	G	A	0.4936	0.0378
rs11158601	LC_IGP28	IgG1 G1S1/(G1+G1S1)	14	66025363	1	2.90E-10	2.36E-10	+	0.215839	0.2294	+	G	A	0.47706422	0.0340243	+	G	A	0.4936	0.0362
rs11158601	LC_IGP26	IgG1 GS1/(G+GS1)	14	66025363	1	4.27E-08	1.08E-09	+	0.188325	0.2262	+	G	A	0.47706422	0.034207	+	G	A	0.4936	0.0371
rs11158601	LC_IGP27	IgG1 GS1/(G0+G+GS1)	14	66025363	1	2.07E-09	2.07E-08	+	0.205533	0.2065	+	G	A	0.47706422	0.0341055	+	G	A	0.4936	0.0368
rs11158601	LC_IGP11	IgG1_G0	14	66025363	1	3.46E-19	2.27E-14	-	-0.304361	-0.2868	+	G	A	0.477397679	0.0335749	+	G	A	0.4936	0.0376
rs11158601	LC_IGP_R1	IgG1_G0F/IgG1_G0	14	66025363	1	2.03E-16	5.45E-12	+	0.279862	0.2599	+	G	A	0.477397679	0.0336893	+	G	A	0.4936	0.0377
rs11158601	LC_IGP_R4	IgG1_G0FN/IgG1_G0N	14	66025363	1	1.11E-14	2.75E-13	+	0.264732	0.2771	+	G	A	0.477954685	0.0339473	+	G	A	0.4936	0.0379
rs11158601	LC_IGP52	IgG1_G0n	14	66025363	1	3.18E-19	2.00E-14	-	-0.307051	-0.288	+	G	A	0.477695167	0.0338291	+	G	A	0.4936	0.0377
rs11158601	LC_IGP14	IgG1_G0N	14	66025363	1	6.46E-17	3.39E-11	-	-0.285829	-0.2479	+	G	A	0.477954685	0.0338334	+	G	A	0.4936	0.0374
rs11158601	LC_IGP55	IgG1_G0Nn	14	66025363	1	3.00E-17	3.51E-11	-	-0.290596	-0.248	+	G	A	0.477695167	0.0340181	+	G	A	0.4936	0.0374
rs11158601	LC_IGP12	IgG1_G1	14	66025363	1	4.21E-16	1.06E-13	-	-0.27755	-0.2809	+	G	A	0.477397679	0.0337789	+	G	A	0.4936	0.0378

rs11158601	LC_IGP_R2	IgG1_G1F/IgG1_G1	14	66025363	1	2.33E-17	1.26E-13	+	0.289141	0.2794	+	G	A	0.477397679	0.0337338	+	G	A	0.4936	0.0377
rs11158601	LC_IGP_R5	IgG1_G1FN/IgG1_G1N	14	66025363	1	1.69E-14	1.84E-17	+	0.263997	0.3213	+	G	A	0.478782288	0.0340917	+	G	A	0.4936	0.0378
rs11158601	LC_IGP53	IgG1_G1n	14	66025363	1	3.27E-15	1.84E-13	-	-0.271511	-0.2779	+	G	A	0.477695167	0.0341192	+	G	A	0.4936	0.0378
rs11158601	LC_IGP15	IgG1_G1N	14	66025363	1	1.91E-11	1.06E-10	-	-0.231348	-0.2426	+	G	A	0.478782288	0.0342191	+	G	A	0.4936	0.0376
rs11158601	LC_IGP56	IgG1_G1Nn	14	66025363	1	8.09E-12	2.16E-10	-	-0.236497	-0.2382	+	G	A	0.477695167	0.0343348	+	G	A	0.4936	0.0375
rs11158601	LC_IGP_R36	IgG1_G1NS1/IgG1_G1N	14	66025363	1	1.88E-08	3.59E-12	+	0.192981	0.2556	+	G	A	0.478729963	0.0341475	+	G	A	0.4936	0.0368
rs11158601	LC_IGP_R32	IgG1_G1S1/IgG1_G1	14	66025363	1	5.24E-10	3.59E-10	+	0.212749	0.2274	+	G	A	0.47706422	0.0340414	+	G	A	0.4936	0.0363
rs11158601	LC_IGP_R3	IgG1_G2F/IgG1_G2	14	66025363	1	1.76E-15	8.89E-15	+	0.272065	0.2908	+	G	A	0.47707824	0.0338565	+	G	A	0.4936	0.0375
rs11158601	LC_IGP_R8	IgG1_G2FS1/IgG1_G2S1	14	66025363	1	5.86E-10	1.17E-12	+	0.212545	0.2659	+	G	A	0.476786805	0.0341071	+	G	A	0.4936	0.0374
rs11158601	LC_IGP_R26	IgG1_G2N/IgG1_G1N	14	66025363	1	3.45E-12	1.09E-08	+	0.239138	0.2093	+	G	A	0.478395062	0.0341057	+	G	A	0.4936	0.0366
rs11158601	LC_IGP148	IgG2 FG0n total/G0n	14	66025363	1	6.69E-09	1.19E-13	+	0.199597	0.2775	+	G	A	0.475985222	0.0342391	+	G	A	0.4935	0.0374
rs11158601	LC_IGP97	IgG2_G0	14	66025363	1	1.61E-10	8.88E-14	-	-0.218836	-0.2793	+	G	A	0.47707824	0.0340024	+	G	A	0.4936	0.0374
rs11158601	LC_IGP138	IgG2_G0n	14	66025363	1	9.58E-11	6.27E-14	-	-0.222343	-0.2814	+	G	A	0.475985222	0.0341198	+	G	A	0.4935	0.0375
rs11158601	LC_IGP_R64	IgG2_G2/IgG2_G1	14	66025363	1	7.30E-09	1.65E-12	+	0.199357	0.2589	+	G	A	0.476132191	0.0342853	+	G	A	0.4936	0.0367
rs7146742	LC_IGP84	IgG1 FBn/Bn total	14	66032934	1	6.00E-12	9.24E-07	+	0.240124	0.1875	+	A	G	0.552316295	0.0346424	+	A	G	0.5703	0.0382
rs7146742	LC_IGP64	IgG1 FG2n total/G2n	14	66032934	1	1.53E-11	2.64E-07	+	0.235136	0.1943	+	A	G	0.552316295	0.0346086	+	A	G	0.5703	0.0378
rs7146742	LC_IGP11	IgG1_G0	14	66032934	1	3.60E-16	1.74E-06	-	-0.27942	-0.1808	+	A	G	0.552802993	0.0339265	+	A	G	0.5703	0.0378
rs7146742	LC_IGP52	IgG1_G0n	14	66032934	1	1.62E-16	2.04E-06	-	-0.285148	-0.1799	+	A	G	0.552316295	0.0342064	+	A	G	0.5703	0.0379
rs7146742	LC_IGP_R5	IgG1_G1FN/IgG1_G1N	14	66032934	1	4.65E-11	2.35E-07	+	0.228544	0.1964	+	A	G	0.553467712	0.0344866	+	A	G	0.5703	0.038
rs7146742	LC_IGP_R3	IgG1_G2F/IgG1_G2	14	66032934	1	1.23E-12	3.30E-07	+	0.244787	0.1923	+	A	G	0.552529645	0.0341967	+	A	G	0.5703	0.0377
rs1958561	LC_IGP78	IgG1 BG0n/G0n	14	66036761	1	4.93E-14	7.67E-10	-	-0.259614	-0.231	+	G	T	0.477682156	0.0341513	+	G	T	0.4935	0.0376
rs1958561	LC_IGP79	IgG1 BG1n/G1n	14	66036761	1	2.70E-13	8.35E-12	-	-0.252563	-0.2564	+	G	T	0.477682156	0.0342662	+	G	T	0.4935	0.0375
rs1958561	LC_IGP32	IgG1 BG1S1/(BG1+BG1S1)	14	66036761	1	2.02E-08	3.19E-12	+	0.192547	0.256	+	G	T	0.478717016	0.0341496	+	G	T	0.4935	0.0367
rs1958561	LC_IGP77	IgG1 Bn	14	66036761	1	3.45E-13	2.03E-11	-	-0.251493	-0.2527	+	G	T	0.477682156	0.0342782	+	G	T	0.4935	0.0377
rs1958561	LC_IGP86	IgG1 Bn/Fn total	14	66036761	1	5.03E-14	1.23E-11	-	-0.260117	-0.2556	+	G	T	0.477682156	0.0342306	+	G	T	0.4935	0.0377
rs1958561	LC_IGP84	IgG1 FBn/Bn total	14	66036761	1	2.61E-15	3.36E-16	+	0.273461	0.31	+	G	T	0.477682156	0.0342381	+	G	T	0.4935	0.038
rs1958561	LC_IGP62	IgG1 FG0n total/G0n	14	66036761	1	1.83E-17	2.46E-12	+	0.29199	0.2649	+	G	T	0.477682156	0.0339463	+	G	T	0.4935	0.0378
rs1958561	LC_IGP63	IgG1 FG1n total/G1n	14	66036761	1	9.28E-18	1.59E-14	+	0.295497	0.2902	+	G	T	0.477682156	0.0340322	+	G	T	0.4935	0.0378
rs1958561	LC_IGP64	IgG1 FG2n total/G2n	14	66036761	1	4.65E-14	2.92E-15	+	0.260597	0.2966	+	G	T	0.477682156	0.0342455	+	G	T	0.4935	0.0376
rs1958561	LC_IGP68	IgG1 FG2n/G2n	14	66036761	1	1.45E-10	1.83E-08	+	0.221971	0.2107	+	G	T	0.477682156	0.0344024	+	G	T	0.4935	0.0374
rs1958561	LC_IGP61	IgG1 Fn total	14	66036761	1	7.82E-17	3.67E-14	+	0.286921	0.2864	+	G	T	0.477682156	0.0340527	+	G	T	0.4935	0.0378
rs1958561	LC_IGP81	IgG1 Fn/Bn	14	66036761	1	1.77E-13	1.05E-10	+	0.254402	0.2425	+	G	T	0.477682156	0.0342454	+	G	T	0.4935	0.0375
rs1958561	LC_IGP21	IgG1 Fucosylation	14	66036761	1	2.87E-15	4.79E-13	+	0.271332	0.273	+	G	T	0.476867448	0.0340267	+	G	T	0.4935	0.0378
rs1958561	LC_IGP28	IgG1 G1S1/(G1+G1S1)	14	66036761	1	2.69E-10	2.53E-10	+	0.21629	0.229	+	G	T	0.477051376	0.0340312	+	G	T	0.4935	0.0362
rs1958561	LC_IGP26	IgG1 GS1/(G+GS1)	14	66036761	1	4.28E-08	1.16E-09	+	0.188357	0.2258	+	G	T	0.477051376	0.0342155	+	G	T	0.4935	0.0371
rs1958561	LC_IGP27	IgG1 GS1/(G0+G+GS1)	14	66036761	1	2.08E-09	2.24E-08	+	0.20555	0.206	+	G	T	0.477051376	0.0341142	+	G	T	0.4935	0.0368
rs1958561	LC_IGP11	IgG1_G0	14	66036761	1	3.11E-19	2.44E-14	-	-0.30483	-0.2865	+	G	T	0.47738485	0.033581	+	G	T	0.4935	0.0376
rs1958561	LC_IGP_R1	IgG1_G0F/IgG1_G0	14	66036761	1	1.70E-16	5.62E-12	+	0.280635	0.2598	+	G	T	0.47738485	0.0336941	+	G	T	0.4935	0.0377
rs1958561	LC_IGP_R4	IgG1_G0FN/IgG1_G0N	14	66036761	1	9.36E-15	2.86E-13	+	0.265533	0.2769	+	G	T	0.477941825	0.0339523	+	G	T	0.4935	0.0379
rs1958561	LC_IGP52	IgG1_G0n	14	66036761	1	2.84E-19	2.14E-14	-	-0.307544	-0.2877	+	G	T	0.477682156	0.0338353	+	G	T	0.4935	0.0377
rs1958561	LC_IGP14	IgG1_G0N	14	66036761	1	5.90E-17	3.52E-11	-	-0.286255	-0.2477	+	G	T	0.477941825	0.03384	+	G	T	0.4935	0.0374
rs1958561	LC_IGP55	IgG1_G0Nn	14	66036761	1	2.72E-17	3.64E-11	-	-0.291051	-0.2478	+	G	T	0.477682156	0.0340247	+	G	T	0.4935	0.0374
rs1958561	LC_IGP12	IgG1_G1	14	66036761	1	3.62E-16	1.09E-13	-	-0.278224	-0.2808	+	G	T	0.47738485	0.0337842	+	G	T	0.4935	0.0378
rs1958561	LC_IGP_R2	IgG1_G1F/IgG1_G1	14	66036761	1	2.08E-17	1.28E-13	+	0.28966	0.2793	+	G	T	0.47738485	0.0337398	+	G	T	0.4935	0.0377
rs1958561	LC_IGP_R5	IgG1_G1FN/IgG1_G1N	14	66036761	1	1.50E-14	1.93E-17	+	0.264575	0.3211	+	G	T	0.478769373	0.0340978	+	G	T	0.4935	0.0378
rs1958561	LC_IGP53	IgG1_G1n	14	66036761	1	2.81E-15	1.88E-13	-	-0.272233	-0.2779	+	G	T	0.477682156	0.0341246	+	G	T	0.4935	0.0378
rs1958561	LC_IGP15	IgG1_G1N	14	66036761	1	1.73E-11	1.06E-10	-	-0.231897	-0.2426	+	G	T	0.478769373	0.0342256	+	G	T	0.4935	0.0376
rs1958561	LC_IGP56	IgG1_G1Nn	14	66036761	1	7.27E-12	2.15E-10	-	-0.237079	-0.2382	+	G	T	0.477682156	0.0343412	+	G	T	0.4935	0.0375
rs1958561	LC_IGP_R36	IgG1_G1NS1/IgG1_G1N	14	66036761	1	1.81E-08	3.67E-12	+	0.193253	0.2555	+	G	T	0.478717016	0.0341553	+	G	T	0.4935	0.0368
rs1958561	LC_IGP_R32	IgG1_G1S1/IgG1_G1	14	66036761	1	4.85E-10	3.85E-10	+	0.213212	0.227	+	G	T	0.477051376	0.0340483	+	G	T	0.4935	0.0363
rs1958561	LC_IGP_R3	IgG1_G2F/IgG1_G2	14	66036761	1	1.61E-15	9.03E-15	+	0.272507	0.2908	+	G	T	0.477065403	0.0338631	+	G	T	0.4935	0.0375
rs1958561	LC_IGP_R8	IgG1_G2FS1/IgG1_G2S1	14	66036761	1	5.29E-10	1.17E-12	+	0.213141	0.2659	+	G	T	0.476773977	0.0341135	+	G	T	0.4935	0.0374
rs1958561	LC_IGP_R26	IgG1_G2N/IgG1_G1N	14	66036761	1	3.26E-12	1.12E-08	+	0.239464	0.2092	+	G	T	0.478382099	0.0341131	+	G	T	0.4935	0.0366
rs1958561	LC_IGP148	IgG2 FG0n total/G0n	14	66036761	1	5.96E-09	1.24E-13	+	0.200313	0.2773	+	G	T	0.475972291	0.0342452	+	G	T	0.4935	0.0374
rs1958561	LC_IGP97	IgG2_G0	14	66036761	1	1.51E-10	9.29E-14	-	-0.219219	-0.279	+	G	T	0.477065403	0.0340096	+	G	T	0.4935	0.0374
rs1958561	LC_IGP138	IgG2_G0n	14	66036761	1	8.96E-11	6.59E-14	-	-0.222738	-0.2811	+	G	T	0.475972291	0.034127	+	G	T	0.4935	0.0375
rs1958561	LC_IGP_R64	IgG2_G2/IgG2_G1	14	66036761	1	7.07E-09	1.80E-12	+	0.199585	0.2584	+	G	T	0.476119339	0.0342932	+	G	T	0.4935	0.0367
rs1958560	LC_IGP84	IgG1 FBn/Bn total	14	66036795	1	1.97E-08	1.33E-07	+	0.199204	0.2039	+	A	G	0.570546778	0.0352985	+	A	G	0.5913	0.0386
rs1958560	LC_IGP63	IgG1 FG1n total/G1n	14	66036795	1	4.07E-10	9.97E-08	+	0.220952	0.2046	+	A	G	0.570546778	0.0351251	+	A	G	0.5913	0.0384
rs1958560	LC_IGP64	IgG1 FG2n total/G2n	14	66036795	1	3.03E-08	3.14E-08	+	0.196249	0.2114	+	A	G	0.570546778	0.0352533	+	A	G	0.5913	0.0382
rs1958560	LC_IGP61	IgG1 Fn total	14	66036795	1	1.32E-09	2.56E-07	+	0.214308	0.1982	+	A	G	0.570546778	0.0351254	+	A	G	0.5913	0.0385
rs1958560	LC_IGP21	IgG1 Fucosylation	14	66036795	1	6.49E-09	1.99E-06	+	0.20442	0.1826	+	A	G	0.569892109	0.0350343	+	A	G	0.5913	0.0384
rs1958560	LC_IGP11	IgG1_G0	14	66036795	1	1.92E-11	6.78E-07	-	-0.234203	-0.1901	+	A	G	0.570168296	0.0346497	+	A	G	0.5913	0.0383
rs1958560	LC_IGP52	IgG1_G0n	14	66036795	1	3.38E-11	7.35E-07	-	-0.233272	-0.1899	+	A	G	0.570546778	0.0349437	+	A	G	0.5913	0.0383
rs1958560	LC_IGP12	IgG1_G1	14	66036795	1	1.47E-09	2.13E-07	-	-0.211593	-0.1993	+	A	G	0.570473732	0.0347856	+	A	G	0.5913	0.0384
rs1958560	LC_IGP_R2	IgG1_G1F/IgG1_G1	14	66036795	1	4.12E-10	1.96E-07	+	0.218657	0.1994	+	A	G	0.570473732	0.0347735	+	A	G	0.5913	0.0383
rs1958560	LC_IGP_R5	IgG1_G1FN/IgG1_G1N	14	66036795	1	2.47E-08	1.96E-08	+	0.196751	0.2158	+	A	G	0.571257995	0.0351166	+	A	G	0.5913	0.0384
rs1958560	LC_IGP53	IgG1_G1n	14	66036795	1	3.72E-09	2.90E-07	-	-0.208374	-0.1969	+	A	G	0.570546778	0.0351446	+	A	G	0.5913	0.0384
rs1958560	LC_IGP_R3	IgG1_G2F/IgG1_G2	14	66036795	1	3.56E-09	5.83E-08	+	0.206941	0.2068	+	A	G	0.569905562	0.0348629	+	A	G	0.5913	0.0381
rs11622014	LC_IGP78	IgG1 BG0n/G0n	14	66041645	1	1.20E-14	6.49E-10	-	-0.266509	-0.232	+	T	C	0.473771685	0.0342173	+	T	C	0.4931	0.0376
rs11622014	LC_IGP79	IgG1 BG1n/G1n	14	66041645	1	8.58E-14	7.42E-12	-	-0.258466	-0.257	+	T	C	0.473771685	0.034338	+	T	C	0.4931	0.0375
rs11622014	LC_IGP32	IgG1 BG1S1/(BG1+BG1S1)	14	66041645	1	1.70E-08	2.47E-12	+	0.194088	0.2572	+	T	C	0.474825832	0.0342398	+	T	C	0.4931	0.0367
rs11622014	LC_IGP31	IgG1 BGS1/(BG0+BG+BGS1)	14	66041645	1	2.28E-08	6.67E-12	+	0.193087	0.2547	+	T	C	0.47485505	0.0343707	+	T	C	0.4931	0.0371
rs11622014	LC_IGP77	IgG1 Bn	14	66041645	1	9.55E-14	1.79E-11	-	-0.258037	-0.2534	+	T	C	0.473771685	0.0343471	+	T	C	0.4931	0.0377
rs11622014	LC_IGP86	IgG1 Bn/Fn total	14	66041645	1	1.40E-14	1.07E-11	-	-0.266456	-0.2563	+	T	C	0.473771685	0.0342995	+	T	C	0.4931	0.0377
rs11622014	LC_IGP84	IgG1 FBn/Bn total	14	66041645	1	7.94E-16	2.46E-16	+	0.279208	0.3113	+	T	C	0.473771685	0.0343088	+	T	C	0.4931	0.038
rs11622014	LC_IGP62	IgG1 FG0n total/G0n	14	66041645	1	5.60E-18	1.89E-12	+	0.297398	0.2663	+	T	C	0.473771685	0.0340165	+	T	C	0.4931	0.0378
rs11622014	LC_IGP63	IgG1 FG1n total/G1n	14	66041645	1	4.77E-18	1.19E-14	+	0.298893	0.2915	+	T	C	0.473771685	0.0341134	+	T	C	0.4931	0.0378
rs11622014	LC_IGP64	IgG1 FG2n total/G2n	14	66041645	1	3.22E-14	2.16E-15	+	0.262955	0.2979	+	T	C	0.473771685	0.0343334	+	T	C	0.4931	0.0376
rs11622014	LC_IGP68	IgG1 FG2n/G2n	14	66041645	1	7.27E-11	1.66E-08	+	0.226184	0.2112	+	T	C	0.473771685	0.0344841	+	T	C	0.4931	0.0374
rs11622014	LC_IGP61	IgG1 Fn total	14	66041645	1	3.52E-17	2.73E-14	+	0.290897	0.2878	+	T	C	0.473771685	0.0341312	+	T	C	0.4931	0.0378
rs11622014	LC_IGP81	IgG1 Fn/Bn	14	66041645	1	4.86E-14	9.11E-11	+	0.260916	0.2433	+	T	C	0.473771685	0.0343141	+	T	C	0.4931	0.0375
rs11622014	LC_IGP21	IgG1 Fucosylation	14	66041645	1	1.44E-15	3.63E-13	+	0.275002	0.2743	+	T	C	0.472976264	0.0341077	+	T	C	0.4931	0.0377
rs11622014	LC_IGP28	IgG1 G1S1/(G1+G1S1)	14	66041645	1	3.17E-10	1.55E-10	+	0.216024	0.2316	+	T	C	0.473191131	0.0341288	+	T	C	0.4931	0.0362
rs11622014	LC_IGP27	IgG1 GS1/(G0+G+GS1)	14	66041645	1	2.62E-09	1.88E-08	+	0.204848	0.207	+	T	C	0.473191131	0.0342134	+	T	C	0.4931	0.0368
rs11622014	LC_IGP11	IgG1_G0	14	66041645	1	6.42E-20	1.83E-14	-	-0.311602	-0.2878	+	T	C	0.473223885	0.0336637	+	T	C	0.4931	0.0376
rs11622014	LC_IGP_R1	IgG1_G0F/IgG1_G0	14	66041645	1	2.31E-17	4.28E-12	+	0.289482	0.2612	+	T	C	0.473223885	0.0337685	+	T	C	0.4931	0.0377
rs11622014	LC_IGP_R4	IgG1_G0FN/IgG1_G0N	14	66041645	1	2.39E-15	2.17E-13	+	0.272225	0.2782	+	T	C	0.473770667	0.0340402	+	T	C	0.4931	0.0379
rs11622014	LC_IGP52	IgG1_G0n	14	66041645	1	1.09E-19	1.61E-14	-	-0.311907	-0.289	+	T	C	0.473771685	0.03391	+	T	C	0.4931	0.0376
rs11622014	LC_IGP14	IgG1_G0N	14	66041645	1	7.11E-18	3.04E-11	-	-0.295488	-0.2485	+	T	C	0.473770667	0.0339122	+	T	C	0.4931	0.0374
rs11622014	LC_IGP55	IgG1_G0Nn	14	66041645	1	6.77E-18	3.15E-11	-	-0.297285	-0.2486	+	T	C	0.473771685	0.0340907	+	T	C	0.4931	0.0374
rs11622014	LC_IGP12	IgG1_G1	14	66041645	1	9.07E-17	7.84E-14	-	-0.284735	-0.2824	+	T	C	0.473223885	0.0338714	+	T	C	0.4931	0.0378
rs11622014	LC_IGP_R2	IgG1_G1F/IgG1_G1	14	66041645	1	4.64E-18	9.53E-14	+	0.296427	0.2807	+	T	C	0.473223885	0.0338245	+	T	C	0.4931	0.0377
rs11622014	LC_IGP_R5	IgG1_G1FN/IgG1_G1N	14	66041645	1	5.58E-15	1.39E-17	+	0.269547	0.3224	+	T	C	0.474887761	0.0341707	+	T	C	0.4931	0.0378
rs11622014	LC_IGP53	IgG1_G1n	14	66041645	1	1.66E-15	1.37E-13	-	-0.275191	-0.2794	+	T	C	0.473771685	0.0342091	+	T	C	0.4931	0.0378
rs11622014	LC_IGP15	IgG1_G1N	14	66041645	1	5.96E-12	9.43E-11	-	-0.237752	-0.2433	+	T	C	0.474887761	0.0342973	+	T	C	0.4931	0.0376
rs11622014	LC_IGP56	IgG1_G1Nn	14	66041645	1	2.33E-12	1.91E-10	-	-0.243233	-0.2389	+	T	C	0.473771685	0.0344134	+	T	C	0.4931	0.0375
rs11622014	LC_IGP_R36	IgG1_G1NS1/IgG1_G1N	14	66041645	1	1.48E-08	2.86E-12	+	0.194943	0.2567	+	T	C	0.474825832	0.034245	+	T	C	0.4931	0.0368
rs11622014	LC_IGP_R32	IgG1_G1S1/IgG1_G1	14	66041645	1	5.47E-10	2.50E-10	+	0.213162	0.2292	+	T	C	0.473191131	0.0341451	+	T	C	0.4931	0.0362
rs11622014	LC_IGP_R3	IgG1_G2F/IgG1_G2	14	66041645	1	1.32E-15	6.60E-15	+	0.274086	0.2921	+	T	C	0.473207518	0.0339528	+	T	C	0.4931	0.0375
rs11622014	LC_IGP_R8	IgG1_G2FS1/IgG1_G2S1	14	66041645	1	5.33E-10	8.94E-13	+	0.213697	0.2672	+	T	C	0.472918448	0.0342084	+	T	C	0.4931	0.0374
rs11622014	LC_IGP_R26	IgG1_G2N/IgG1_G1N	14	66041645	1	2.86E-12	1.01E-08	+	0.240746	0.2098	+	T	C	0.474486111	0.0342045	+	T	C	0.4931	0.0366
rs11622014	LC_IGP148	IgG2 FG0n total/G0n	14	66041645	1	1.07E-08	1.10E-13	+	0.197521	0.2779	+	T	C	0.472085899	0.0343541	+	T	C	0.4931	0.0374
rs11622014	LC_IGP97	IgG2_G0	14	66041645	1	3.08E-10	8.58E-14	-	-0.216099	-0.2794	+	T	C	0.473207518	0.0341184	+	T	C	0.4931	0.0374
rs11622014	LC_IGP138	IgG2_G0n	14	66041645	1	1.90E-10	6.04E-14	-	-0.219477	-0.2815	+	T	C	0.472085899	0.0342391	+	T	C	0.4931	0.0375
rs11622014	LC_IGP_R64	IgG2_G2/IgG2_G1	14	66041645	1	1.29E-08	1.73E-12	+	0.196666	0.2586	+	T	C	0.472256732	0.0344017	+	T	C	0.4931	0.0367
rs72714499	LC_IGP61	IgG1 Fn total	14	66042459	1	2.95E-08	9.80E-07	-	-0.239888	-0.2503	+	T	C	0.206755555	0.0430524	+	T	C	0.1661	0.0511
rs12887134	LC_IGP78	IgG1 BG0n/G0n	14	66045543	1	1.90E-14	6.55E-10	-	-0.264685	-0.2319	+	C	G	0.472730483	0.0342483	+	C	G	0.4931	0.0376
rs12887134	LC_IGP79	IgG1 BG1n/G1n	14	66045543	1	1.27E-13	7.41E-12	-	-0.256872	-0.257	+	C	G	0.472730483	0.0343676	+	C	G	0.4931	0.0375
rs12887134	LC_IGP32	IgG1 BG1S1/(BG1+BG1S1)	14	66045543	1	1.87E-08	2.45E-12	+	0.193663	0.2573	+	C	G	0.473789766	0.0342627	+	C	G	0.4931	0.0367
rs12887134	LC_IGP31	IgG1 BGS1/(BG0+BG+BGS1)	14	66045543	1	3.28E-08	6.67E-12	+	0.191033	0.2547	+	C	G	0.473807357	0.0343996	+	C	G	0.4931	0.0371
rs12887134	LC_IGP77	IgG1 Bn	14	66045543	1	1.54E-13	1.79E-11	-	-0.256048	-0.2534	+	C	G	0.472730483	0.0343786	+	C	G	0.4931	0.0377
rs12887134	LC_IGP86	IgG1 Bn/Fn total	14	66045543	1	2.27E-14	1.07E-11	-	-0.264527	-0.2563	+	C	G	0.472730483	0.034331	+	C	G	0.4931	0.0377
rs12887134	LC_IGP84	IgG1 FBn/Bn total	14	66045543	1	1.59E-15	2.47E-16	+	0.276492	0.3113	+	C	G	0.472730483	0.0343448	+	C	G	0.4931	0.038
rs12887134	LC_IGP62	IgG1 FG0n total/G0n	14	66045543	1	8.33E-18	1.90E-12	+	0.296057	0.2662	+	C	G	0.472730483	0.034046	+	C	G	0.4931	0.0378
rs12887134	LC_IGP63	IgG1 FG1n total/G1n	14	66045543	1	6.51E-18	1.19E-14	+	0.297883	0.2915	+	C	G	0.472730483	0.0341412	+	C	G	0.4931	0.0378
rs12887134	LC_IGP64	IgG1 FG2n total/G2n	14	66045543	1	4.71E-14	2.17E-15	+	0.261431	0.2979	+	C	G	0.472730483	0.0343629	+	C	G	0.4931	0.0376
rs12887134	LC_IGP68	IgG1 FG2n/G2n	14	66045543	1	8.56E-11	1.66E-08	+	0.225484	0.2112	+	C	G	0.472730483	0.034509	+	C	G	0.4931	0.0374
rs12887134	LC_IGP61	IgG1 Fn total	14	66045543	1	5.37E-17	2.74E-14	+	0.289407	0.2878	+	C	G	0.472730483	0.0341614	+	C	G	0.4931	0.0378

rs12887134	LC_IGP81	IgG1 Fn/Bn	14	66045543	1	7.51E-14	9.12E-11	+	0.259135	0.2432	+	C	G	0.472730483	0.0343447	+	C	G	0.4931	0.0375
rs12887134	LC_IGP21	IgG1 Fucosylation	14	66045543	1	1.96E-15	3.65E-13	+	0.273867	0.2743	+	C	G	0.471940197	0.0341356	+	C	G	0.4931	0.0377
rs12887134	LC_IGP28	IgG1 G1S1/(G1+G1S1)	14	66045543	1	1.61E-10	1.54E-10	+	0.219699	0.2316	+	C	G	0.472163303	0.0341362	+	C	G	0.4931	0.0362
rs12887134	LC_IGP26	IgG1 GS1/(G+GS1)	14	66045543	1	3.82E-08	8.57E-10	+	0.189677	0.2274	+	C	G	0.472163303	0.0343293	+	C	G	0.4931	0.0371
rs12887134	LC_IGP27	IgG1 GS1/(G0+G+GS1)	14	66045543	1	1.48E-09	1.88E-08	+	0.208125	0.207	+	C	G	0.472163303	0.0342229	+	C	G	0.4931	0.0368
rs12887134	LC_IGP11	IgG1_G0	14	66045543	1	6.75E-20	1.85E-14	-	-0.311615	-0.2877	+	C	G	0.472197312	0.0336856	+	C	G	0.4931	0.0376
rs12887134	LC_IGP_R1	IgG1_G0F/IgG1_G0	14	66045543	1	3.19E-17	4.29E-12	+	0.288398	0.2611	+	C	G	0.472197312	0.033796	+	C	G	0.4931	0.0377
rs12887134	LC_IGP_R4	IgG1_G0FN/IgG1_G0N	14	66045543	1	3.92E-15	2.20E-13	+	0.270308	0.2782	+	C	G	0.47274158	0.0340714	+	C	G	0.4931	0.0379
rs12887134	LC_IGP52	IgG1_G0n	14	66045543	1	1.20E-19	1.62E-14	-	-0.311767	-0.289	+	C	G	0.472730483	0.0339331	+	C	G	0.4931	0.0376
rs12887134	LC_IGP14	IgG1_G0N	14	66045543	1	9.58E-18	3.08E-11	-	-0.294524	-0.2484	+	C	G	0.47274158	0.0339393	+	C	G	0.4931	0.0374
rs12887134	LC_IGP55	IgG1_G0Nn	14	66045543	1	9.66E-18	3.18E-11	-	-0.296095	-0.2485	+	C	G	0.472730483	0.0341195	+	C	G	0.4931	0.0374
rs12887134	LC_IGP12	IgG1_G1	14	66045543	1	1.20E-16	7.85E-14	-	-0.283795	-0.2823	+	C	G	0.472197312	0.0338982	+	C	G	0.4931	0.0378
rs12887134	LC_IGP_R2	IgG1_G1F/IgG1_G1	14	66045543	1	5.64E-18	9.53E-14	+	0.295861	0.2807	+	C	G	0.472197312	0.0338495	+	C	G	0.4931	0.0377
rs12887134	LC_IGP_R5	IgG1_G1FN/IgG1_G1N	14	66045543	1	1.01E-14	1.39E-17	+	0.267169	0.3224	+	C	G	0.473854244	0.0342038	+	C	G	0.4931	0.0378
rs12887134	LC_IGP53	IgG1_G1n	14	66045543	1	2.22E-15	1.37E-13	-	-0.274144	-0.2794	+	C	G	0.472730483	0.0342365	+	C	G	0.4931	0.0378
rs12887134	LC_IGP15	IgG1_G1N	14	66045543	1	8.78E-12	9.42E-11	-	-0.236013	-0.2433	+	C	G	0.473854244	0.0343262	+	C	G	0.4931	0.0376
rs12887134	LC_IGP56	IgG1_G1Nn	14	66045543	1	3.48E-12	1.91E-10	-	-0.241463	-0.2389	+	C	G	0.472730483	0.0344434	+	C	G	0.4931	0.0375
rs12887134	LC_IGP_R36	IgG1_G1NS1/IgG1_G1N	14	66045543	1	1.61E-08	2.85E-12	+	0.194565	0.2567	+	C	G	0.473789766	0.0342677	+	C	G	0.4931	0.0368
rs12887134	LC_IGP_R32	IgG1_G1S1/IgG1_G1	14	66045543	1	2.84E-10	2.50E-10	+	0.216767	0.2292	+	C	G	0.472163303	0.0341529	+	C	G	0.4931	0.0362
rs12887134	LC_IGP_R3	IgG1_G2F/IgG1_G2	14	66045543	1	1.81E-15	6.62E-15	+	0.272943	0.2921	+	C	G	0.472180318	0.0339804	+	C	G	0.4931	0.0375
rs12887134	LC_IGP_R8	IgG1_G2FS1/IgG1_G2S1	14	66045543	1	6.10E-10	9.00E-13	+	0.2131	0.2672	+	C	G	0.471891875	0.0342324	+	C	G	0.4931	0.0374
rs12887134	LC_IGP_R26	IgG1_G2N/IgG1_G1N	14	66045543	1	1.87E-12	1.00E-08	+	0.242916	0.2098	+	C	G	0.473448765	0.0342168	+	C	G	0.4931	0.0366
rs12887134	LC_IGP148	IgG2 FG0n total/G0n	14	66045543	1	1.49E-08	1.09E-13	+	0.195716	0.2779	+	C	G	0.471051108	0.0343827	+	C	G	0.493	0.0374
rs12887134	LC_IGP97	IgG2_G0	14	66045543	1	3.79E-10	8.58E-14	-	-0.215149	-0.2794	+	C	G	0.472180318	0.0341437	+	C	G	0.4931	0.0374
rs12887134	LC_IGP138	IgG2_G0n	14	66045543	1	2.42E-10	6.04E-14	-	-0.218363	-0.2815	+	C	G	0.471051108	0.0342658	+	C	G	0.493	0.0375
rs12887134	LC_IGP_R64	IgG2_G2/IgG2_G1	14	66045543	1	9.40E-09	1.73E-12	+	0.198628	0.2586	+	C	G	0.471228274	0.0344169	+	C	G	0.4931	0.0367
rs7155541	LC_IGP78	IgG1 BG0n/G0n	14	66046242	1	1.90E-14	5.86E-10	-	-0.264684	-0.2329	+	C	G	0.472731103	0.0342483	+	C	G	0.4935	0.0376
rs7155541	LC_IGP79	IgG1 BG1n/G1n	14	66046242	1	1.27E-13	6.31E-12	-	-0.25687	-0.2582	+	C	G	0.472731103	0.0343676	+	C	G	0.4935	0.0376
rs7155541	LC_IGP32	IgG1 BG1S1/(BG1+BG1S1)	14	66046242	1	1.87E-08	1.99E-12	+	0.193661	0.2586	+	C	G	0.473790382	0.0342627	+	C	G	0.4935	0.0368
rs7155541	LC_IGP31	IgG1 BGS1/(BG0+BG+BGS1)	14	66046242	1	3.28E-08	5.71E-12	+	0.19103	0.2558	+	C	G	0.47380798	0.0343996	+	C	G	0.4935	0.0371
rs7155541	LC_IGP77	IgG1 Bn	14	66046242	1	1.54E-13	1.58E-11	-	-0.256046	-0.2544	+	C	G	0.472731103	0.0343786	+	C	G	0.4935	0.0377
rs7155541	LC_IGP86	IgG1 Bn/Fn total	14	66046242	1	2.27E-14	9.51E-12	-	-0.264526	-0.2573	+	C	G	0.472731103	0.034331	+	C	G	0.4935	0.0378
rs7155541	LC_IGP84	IgG1 FBn/Bn total	14	66046242	1	1.59E-15	2.02E-16	+	0.276491	0.3126	+	C	G	0.472731103	0.0343448	+	C	G	0.4935	0.038
rs7155541	LC_IGP62	IgG1 FG0n total/G0n	14	66046242	1	8.33E-18	1.67E-12	+	0.296056	0.2672	+	C	G	0.472731103	0.034046	+	C	G	0.4935	0.0379
rs7155541	LC_IGP63	IgG1 FG1n total/G1n	14	66046242	1	6.51E-18	9.95E-15	+	0.297881	0.2927	+	C	G	0.472731103	0.0341412	+	C	G	0.4935	0.0378
rs7155541	LC_IGP64	IgG1 FG2n total/G2n	14	66046242	1	4.71E-14	1.84E-15	+	0.261428	0.299	+	C	G	0.472731103	0.0343629	+	C	G	0.4935	0.0376
rs7155541	LC_IGP68	IgG1 FG2n/G2n	14	66046242	1	8.57E-11	1.53E-08	+	0.225481	0.212	+	C	G	0.472731103	0.034509	+	C	G	0.4935	0.0375
rs7155541	LC_IGP61	IgG1 Fn total	14	66046242	1	5.37E-17	2.33E-14	+	0.289405	0.289	+	C	G	0.472731103	0.0341614	+	C	G	0.4935	0.0379
rs7155541	LC_IGP81	IgG1 Fn/Bn	14	66046242	1	7.52E-14	8.09E-11	+	0.259133	0.2442	+	C	G	0.472731103	0.0343447	+	C	G	0.4935	0.0376
rs7155541	LC_IGP21	IgG1 Fucosylation	14	66046242	1	1.96E-15	3.22E-13	+	0.273865	0.2753	+	C	G	0.471940814	0.0341356	+	C	G	0.4935	0.0378
rs7155541	LC_IGP28	IgG1 G1S1/(G1+G1S1)	14	66046242	1	1.61E-10	1.21E-10	+	0.219698	0.2333	+	C	G	0.472163914	0.0341362	+	C	G	0.4935	0.0362
rs7155541	LC_IGP26	IgG1 GS1/(G+GS1)	14	66046242	1	3.83E-08	7.46E-10	+	0.189673	0.2285	+	C	G	0.472163914	0.0343293	+	C	G	0.4935	0.0371
rs7155541	LC_IGP27	IgG1 GS1/(G0+G+GS1)	14	66046242	1	1.48E-09	1.75E-08	+	0.208122	0.2077	+	C	G	0.472163914	0.0342229	+	C	G	0.4935	0.0369
rs7155541	LC_IGP11	IgG1_G0	14	66046242	1	6.75E-20	1.75E-14	-	-0.311613	-0.2884	+	C	G	0.472197923	0.0336856	+	C	G	0.4935	0.0376
rs7155541	LC_IGP_R1	IgG1_G0F/IgG1_G0	14	66046242	1	3.19E-17	3.82E-12	+	0.288397	0.2621	+	C	G	0.472197923	0.033796	+	C	G	0.4935	0.0378
rs7155541	LC_IGP_R4	IgG1_G0FN/IgG1_G0N	14	66046242	1	3.92E-15	1.89E-13	+	0.270308	0.2793	+	C	G	0.472742192	0.0340714	+	C	G	0.4935	0.038
rs7155541	LC_IGP52	IgG1_G0n	14	66046242	1	1.20E-19	1.52E-14	-	-0.311765	-0.2896	+	C	G	0.472731103	0.0339331	+	C	G	0.4935	0.0377
rs7155541	LC_IGP14	IgG1_G0N	14	66046242	1	9.58E-18	2.93E-11	-	-0.294522	-0.249	+	C	G	0.472742192	0.0339393	+	C	G	0.4935	0.0374
rs7155541	LC_IGP55	IgG1_G0Nn	14	66046242	1	9.66E-18	3.01E-11	-	-0.296093	-0.2491	+	C	G	0.472731103	0.0341195	+	C	G	0.4935	0.0375
rs7155541	LC_IGP12	IgG1_G1	14	66046242	1	1.20E-16	6.61E-14	-	-0.283793	-0.2835	+	C	G	0.472197923	0.0338981	+	C	G	0.4935	0.0378
rs7155541	LC_IGP_R2	IgG1_G1F/IgG1_G1	14	66046242	1	5.65E-18	8.13E-14	+	0.295859	0.2818	+	C	G	0.472197923	0.0338495	+	C	G	0.4935	0.0377
rs7155541	LC_IGP_R5	IgG1_G1FN/IgG1_G1N	14	66046242	1	1.01E-14	1.09E-17	+	0.267168	0.3238	+	C	G	0.473854859	0.0342038	+	C	G	0.4935	0.0378
rs7155541	LC_IGP53	IgG1_G1n	14	66046242	1	2.22E-15	1.15E-13	-	-0.274142	-0.2806	+	C	G	0.472731103	0.0342365	+	C	G	0.4935	0.0378
rs7155541	LC_IGP15	IgG1_G1N	14	66046242	1	8.78E-12	8.10E-11	-	-0.236012	-0.2444	+	C	G	0.473854859	0.0343262	+	C	G	0.4935	0.0376
rs7155541	LC_IGP56	IgG1_G1Nn	14	66046242	1	3.48E-12	1.64E-10	-	-0.241461	-0.24	+	C	G	0.472731103	0.0344433	+	C	G	0.4935	0.0376
rs7155541	LC_IGP_R36	IgG1_G1NS1/IgG1_G1N	14	66046242	1	1.61E-08	2.32E-12	+	0.194562	0.258	+	C	G	0.473790382	0.0342677	+	C	G	0.4935	0.0368
rs7155541	LC_IGP_R32	IgG1_G1S1/IgG1_G1	14	66046242	1	2.84E-10	1.97E-10	+	0.216766	0.2309	+	C	G	0.472163914	0.0341529	+	C	G	0.4935	0.0363
rs7155541	LC_IGP_R3	IgG1_G2F/IgG1_G2	14	66046242	1	1.81E-15	5.61E-15	+	0.272939	0.2933	+	C	G	0.472180929	0.0339804	+	C	G	0.4935	0.0375
rs7155541	LC_IGP_R8	IgG1_G2FS1/IgG1_G2S1	14	66046242	1	6.10E-10	7.98E-13	+	0.213099	0.2681	+	C	G	0.471892486	0.0342324	+	C	G	0.4935	0.0374
rs7155541	LC_IGP_R26	IgG1_G2N/IgG1_G1N	14	66046242	1	1.87E-12	8.79E-09	+	0.242915	0.2108	+	C	G	0.473449383	0.0342168	+	C	G	0.4935	0.0366
rs7155541	LC_IGP148	IgG2 FG0n total/G0n	14	66046242	1	1.49E-08	9.22E-14	+	0.195715	0.2791	+	C	G	0.471051724	0.0343827	+	C	G	0.4935	0.0375
rs7155541	LC_IGP97	IgG2_G0	14	66046242	1	3.79E-10	7.74E-14	-	-0.215148	-0.2803	+	C	G	0.472180929	0.0341437	+	C	G	0.4935	0.0375
rs7155541	LC_IGP138	IgG2_G0n	14	66046242	1	2.42E-10	5.35E-14	-	-0.218362	-0.2825	+	C	G	0.471051724	0.0342658	+	C	G	0.4935	0.0376
rs7155541	LC_IGP_R64	IgG2_G2/IgG2_G1	14	66046242	1	9.40E-09	1.76E-12	+	0.198627	0.2589	+	C	G	0.471228886	0.0344168	+	C	G	0.4935	0.0367
rs6573615	LC_IGP79	IgG1 BG1n/G1n	14	66046534	1	9.08E-09	2.10E-07	-	-0.205259	-0.2017	+	A	G	0.585935874	0.0355269	+	A	G	0.6175	0.0389
rs6573615	LC_IGP77	IgG1 Bn	14	66046534	1	5.29E-09	3.16E-07	-	-0.208503	-0.1997	+	A	G	0.585935874	0.0355224	+	A	G	0.6175	0.039
rs6573615	LC_IGP86	IgG1 Bn/Fn total	14	66046534	1	1.56E-09	2.29E-07	-	-0.21555	-0.2021	+	A	G	0.585935874	0.0354884	+	A	G	0.6175	0.0391
rs6573615	LC_IGP84	IgG1 FBn/Bn total	14	66046534	1	5.85E-11	1.38E-09	+	0.233958	0.2384	+	A	G	0.585935874	0.0354898	+	A	G	0.6175	0.0394
rs6573615	LC_IGP62	IgG1 FG0n total/G0n	14	66046534	1	3.19E-12	4.68E-08	+	0.247438	0.214	+	A	G	0.585935874	0.0352319	+	A	G	0.6175	0.0392
rs6573615	LC_IGP63	IgG1 FG1n total/G1n	14	66046534	1	2.29E-12	1.81E-09	+	0.249789	0.2353	+	A	G	0.585935874	0.0353287	+	A	G	0.6175	0.0391
rs6573615	LC_IGP64	IgG1 FG2n total/G2n	14	66046534	1	6.11E-10	1.26E-09	+	0.220914	0.2363	+	A	G	0.585935874	0.0354855	+	A	G	0.6175	0.0389
rs6573615	LC_IGP61	IgG1 Fn total	14	66046534	1	4.38E-12	2.61E-09	+	0.246426	0.2332	+	A	G	0.585935874	0.0353178	+	A	G	0.6175	0.0392
rs6573615	LC_IGP81	IgG1 Fn/Bn	14	66046534	1	4.68E-09	6.67E-07	+	0.20911	0.1932	+	A	G	0.585935874	0.0355003	+	A	G	0.6175	0.0389
rs6573615	LC_IGP21	IgG1 Fucosylation	14	66046534	1	1.82E-11	1.40E-08	+	0.238261	0.2219	+	A	G	0.585512022	0.0352032	+	A	G	0.6175	0.0391
rs6573615	LC_IGP11	IgG1_G0	14	66046534	1	1.75E-14	1.35E-09	-	-0.269237	-0.2362	+	A	G	0.585628589	0.0347933	+	A	G	0.6175	0.039
rs6573615	LC_IGP_R1	IgG1_G0F/IgG1_G0	14	66046534	1	2.25E-12	5.15E-08	+	0.246674	0.2126	+	A	G	0.585628589	0.0348793	+	A	G	0.6175	0.039
rs6573615	LC_IGP_R4	IgG1_G0FN/IgG1_G0N	14	66046534	1	6.07E-10	6.49E-08	+	0.219201	0.2125	+	A	G	0.586450704	0.0352066	+	A	G	0.6175	0.0393
rs6573615	LC_IGP52	IgG1_G0n	14	66046534	1	5.64E-14	1.35E-09	-	-0.266343	-0.2367	+	A	G	0.585935874	0.03512	+	A	G	0.6175	0.039
rs6573615	LC_IGP14	IgG1_G0N	14	66046534	1	7.91E-11	9.13E-07	-	-0.230112	-0.1905	+	A	G	0.586450704	0.0351549	+	A	G	0.6175	0.0388
rs6573615	LC_IGP55	IgG1_G0Nn	14	66046534	1	3.97E-11	8.86E-07	-	-0.235172	-0.1909	+	A	G	0.585935874	0.0353589	+	A	G	0.6175	0.0388
rs6573615	LC_IGP12	IgG1_G1	14	66046534	1	5.77E-12	4.36E-09	-	-0.242511	-0.2296	+	A	G	0.585934026	0.0349621	+	A	G	0.6175	0.0391
rs6573615	LC_IGP_R2	IgG1_G1F/IgG1_G1	14	66046534	1	2.12E-12	4.12E-09	+	0.247512	0.2295	+	A	G	0.585934026	0.0349554	+	A	G	0.6175	0.039
rs6573615	LC_IGP_R5	IgG1_G1FN/IgG1_G1N	14	66046534	1	2.40E-10	3.37E-10	+	0.225105	0.2458	+	A	G	0.586839176	0.0353182	+	A	G	0.6175	0.0391
rs6573615	LC_IGP53	IgG1_G1n	14	66046534	1	2.46E-11	6.59E-09	-	-0.237687	-0.2268	+	A	G	0.585935874	0.035352	+	A	G	0.6175	0.0391
rs6573615	LC_IGP_R3	IgG1_G2F/IgG1_G2	14	66046534	1	5.73E-11	2.38E-09	+	0.23125	0.2319	+	A	G	0.585391504	0.0350655	+	A	G	0.6175	0.0388
rs12879805	LC_IGP78	IgG1 BG0n/G0n	14	66047795	1	3.13E-14	7.32E-10	-	-0.261454	-0.2314	+	C	T	0.478738228	0.0341215	+	C	T	0.4939	0.0376
rs12879805	LC_IGP79	IgG1 BG1n/G1n	14	66047795	1	1.88E-13	7.78E-12	-	-0.254056	-0.2569	+	C	T	0.478738228	0.0342383	+	C	T	0.4939	0.0375
rs12879805	LC_IGP32	IgG1 BG1S1/(BG1+BG1S1)	14	66047795	1	1.89E-08	3.18E-12	+	0.192826	0.2562	+	C	T	0.479768187	0.0341283	+	C	T	0.4939	0.0368
rs12879805	LC_IGP77	IgG1 Bn	14	66047795	1	2.19E-13	1.91E-11	-	-0.25342	-0.2532	+	C	T	0.478738228	0.0342483	+	C	T	0.4939	0.0377
rs12879805	LC_IGP86	IgG1 Bn/Fn total	14	66047795	1	3.19E-14	1.15E-11	-	-0.261979	-0.256	+	C	T	0.478738228	0.0342006	+	C	T	0.4939	0.0377
rs12879805	LC_IGP84	IgG1 FBn/Bn total	14	66047795	1	1.32E-15	3.06E-16	+	0.276161	0.3105	+	C	T	0.478738228	0.0342034	+	C	T	0.4939	0.038
rs12879805	LC_IGP62	IgG1 FG0n total/G0n	14	66047795	1	1.25E-17	2.29E-12	+	0.293305	0.2654	+	C	T	0.478738228	0.0339181	+	C	T	0.4939	0.0378
rs12879805	LC_IGP63	IgG1 FG1n total/G1n	14	66047795	1	7.07E-18	1.45E-14	+	0.296372	0.2908	+	C	T	0.478738228	0.0340063	+	C	T	0.4939	0.0378
rs12879805	LC_IGP64	IgG1 FG2n total/G2n	14	66047795	1	3.28E-14	2.59E-15	+	0.261973	0.2973	+	C	T	0.478738228	0.0342178	+	C	T	0.4939	0.0376
rs12879805	LC_IGP68	IgG1 FG2n/G2n	14	66047795	1	1.29E-10	1.69E-08	+	0.222481	0.2112	+	C	T	0.478738228	0.0343793	+	C	T	0.4939	0.0374
rs12879805	LC_IGP61	IgG1 Fn total	14	66047795	1	5.28E-17	3.32E-14	+	0.28831	0.287	+	C	T	0.478738228	0.0340243	+	C	T	0.4939	0.0378
rs12879805	LC_IGP81	IgG1 Fn/Bn	14	66047795	1	1.17E-13	9.81E-11	+	0.256111	0.243	+	C	T	0.478738228	0.0342164	+	C	T	0.4939	0.0376
rs12879805	LC_IGP21	IgG1 Fucosylation	14	66047795	1	2.16E-15	4.48E-13	+	0.272369	0.2734	+	C	T	0.477918311	0.0340004	+	C	T	0.4939	0.0378
rs12879805	LC_IGP28	IgG1 G1S1/(G1+G1S1)	14	66047795	1	5.27E-10	2.25E-10	+	0.212613	0.2298	+	C	T	0.47809419	0.0340248	+	C	T	0.4939	0.0362
rs12879805	LC_IGP27	IgG1 GS1/(G0+G+GS1)	14	66047795	1	3.77E-09	2.30E-08	+	0.20213	0.2059	+	C	T	0.47809419	0.0341061	+	C	T	0.4939	0.0368
rs12879805	LC_IGP11	IgG1_G0	14	66047795	1	3.04E-19	2.38E-14	-	-0.304734	-0.2867	+	C	T	0.47842639	0.0335609	+	C	T	0.4939	0.0376
rs12879805	LC_IGP_R1	IgG1_G0F/IgG1_G0	14	66047795	1	1.26E-16	5.24E-12	+	0.28167	0.2602	+	C	T	0.47842639	0.0336681	+	C	T	0.4939	0.0377
rs12879805	LC_IGP_R4	IgG1_G0FN/IgG1_G0N	14	66047795	1	5.69E-15	2.68E-13	+	0.267494	0.2773	+	C	T	0.478985609	0.0339222	+	C	T	0.4939	0.0379
rs12879805	LC_IGP52	IgG1_G0n	14	66047795	1	2.66E-19	2.08E-14	-	-0.307599	-0.288	+	C	T	0.478738228	0.0338138	+	C	T	0.4939	0.0377
rs12879805	LC_IGP14	IgG1_G0N	14	66047795	1	4.47E-17	3.52E-11	-	-0.287186	-0.2478	+	C	T	0.478985609	0.0338145	+	C	T	0.4939	0.0374
rs12879805	LC_IGP55	IgG1_G0Nn	14	66047795	1	1.94E-17	3.62E-11	-	-0.292206	-0.248	+	C	T	0.478738228	0.0339974	+	C	T	0.4939	0.0375
rs12879805	LC_IGP12	IgG1_G1	14	66047795	1	2.84E-16	9.82E-14	-	-0.27904	-0.2814	+	C	T	0.47842639	0.0337594	+	C	T	0.4939	0.0378
rs12879805	LC_IGP_R2	IgG1_G1F/IgG1_G1	14	66047795	1	1.79E-17	1.16E-13	+	0.290075	0.2799	+	C	T	0.47842639	0.0337169	+	C	T	0.4939	0.0377
rs12879805	LC_IGP_R5	IgG1_G1FN/IgG1_G1N	14	66047795	1	8.45E-15	1.75E-17	+	0.266888	0.3216	+	C	T	0.479817958	0.0340662	+	C	T	0.4939	0.0378
rs12879805	LC_IGP53	IgG1_G1n	14	66047795	1	2.16E-15	1.69E-13	-	-0.27316	-0.2785	+	C	T	0.478738228	0.0340989	+	C	T	0.4939	0.0378
rs12879805	LC_IGP15	IgG1_G1N	14	66047795	1	1.20E-11	9.91E-11	-	-0.233545	-0.2431	+	C	T	0.479817958	0.0341983	+	C	T	0.4939	0.0376
rs12879805	LC_IGP56	IgG1_G1Nn	14	66047795	1	4.98E-12	2.00E-10	-	-0.238767	-0.2387	+	C	T	0.478738228	0.0343129	+	C	T	0.4939	0.0375
rs12879805	LC_IGP_R36	IgG1_G1NS1/IgG1_G1N	14	66047795	1	1.70E-08	3.67E-12	+	0.193486	0.2556	+	C	T	0.479768187	0.0341342	+	C	T	0.4939	0.0368
rs12879805	LC_IGP_R32	IgG1_G1S1/IgG1_G1	14	66047795	1	9.29E-10	3.41E-10	+	0.209609	0.2277	+	C	T	0.47809419	0.0340414	+	C	T	0.4939	0.0363
rs12879805	LC_IGP_R3	IgG1_G2F/IgG1_G2	14	66047795	1	1.23E-15	8.00E-15	+	0.273481	0.2914	+	C	T	0.478107579	0.0338375	+	C	T	0.4939	0.0375
rs12879805	LC_IGP_R8	IgG1_G2FS1/IgG1_G2S1	14	66047795	1	4.64E-10	1.00E-12	+	0.21371	0.2668	+	C	T	0.477815516	0.0340907	+	C	T	0.4939	0.0374
rs12879805	LC_IGP_R26	IgG1_G2N/IgG1_G1N	14	66047795	1	5.04E-12	1.13E-08	+	0.237243	0.2092	+	C	T	0.479434568	0.0341021	+	C	T	0.4939	0.0366
rs12879805	LC_IGP148	IgG2 FG0n total/G0n	14	66047795	1	4.28E-09	1.26E-13	+	0.20207	0.2773	+	C	T	0.477022167	0.0342178	+	C	T	0.4938	0.0374
rs12879805	LC_IGP97	IgG2_G0	14	66047795	1	1.25E-10	1.01E-13	-	-0.220069	-0.2788	+	C	T	0.478107579	0.0339856	+	C	T	0.4939	0.0375

rs12879805	LC_IGP_R41	IgG2_G0F/IgG2_G0	14	66047795	1	3.70E-08	5.78E-13	+	0.18855	0.2682	+	C	T	0.478107579	0.0340897 +	C	T	0.4939	0.0372
rs12879805	LC_IGP138	IgG2_G0n	14	66047795	1	7.16E-11	7.11E-14	-	-0.223752	-0.2809	+	C	T	0.477022167	0.0341018 +	C	T	0.4938	0.0375
rs12879805	LC_IGP_R64	IgG2_G2/IgG2_G1	14	66047795	1	9.89E-09	1.98E-12	+	0.197533	0.2581	+	C	T	0.477162791	0.0342796 +	C	T	0.4939	0.0367
rs2411357	LC_IGP78	IgG1 BG0n/G0n	14	66051100	1	7.45E-14	8.96E-10	-	-0.257874	-0.2307	+	T	G	0.475101301	0.0341729 +	T	G	0.4924	0.0377
rs2411357	LC_IGP79	IgG1 BG1n/G1n	14	66051100	1	4.71E-13	8.99E-12	-	-0.250104	-0.2566	+	T	G	0.475101301	0.0342909 +	T	G	0.4924	0.0376
rs2411357	LC_IGP32	IgG1 BG1S1/(BG1+BG1S1)	14	66051100	1	3.00E-08	3.67E-12	+	0.190278	0.2559	+	T	G	0.476149507	0.0341699 +	T	G	0.4924	0.0368
rs2411357	LC_IGP77	IgG1 Bn	14	66051100	1	5.01E-13	2.28E-11	-	-0.249867	-0.2527	+	T	G	0.475101301	0.034299 +	T	G	0.4924	0.0378
rs2411357	LC_IGP86	IgG1 Bn/Fn total	14	66051100	1	7.34E-14	1.37E-11	-	-0.258536	-0.2556	+	T	G	0.475101301	0.0342515 +	T	G	0.4924	0.0378
rs2411357	LC_IGP84	IgG1 FBn/Bn total	14	66051100	1	2.21E-15	3.93E-16	+	0.274252	0.3097	+	T	G	0.475101301	0.0342477 +	T	G	0.4924	0.038
rs2411357	LC_IGP62	IgG1 FG0n total/G0n	14	66051100	1	2.15E-17	2.92E-12	+	0.291475	0.2646	+	T	G	0.475101301	0.0339625 +	T	G	0.4924	0.0379
rs2411357	LC_IGP63	IgG1 FG1n total/G1n	14	66051100	1	1.08E-17	1.75E-14	+	0.295025	0.2904	+	T	G	0.475101301	0.0340482 +	T	G	0.4924	0.0379
rs2411357	LC_IGP64	IgG1 FG2n total/G2n	14	66051100	1	4.27E-14	3.04E-15	+	0.261068	0.2971	+	T	G	0.475101301	0.0342567 +	T	G	0.4924	0.0377
rs2411357	LC_IGP68	IgG1 FG2n/G2n	14	66051100	1	1.81E-10	1.82E-08	+	0.220924	0.2114	+	T	G	0.475101301	0.03442 +	T	G	0.4924	0.0376
rs2411357	LC_IGP61	IgG1 Fn total	14	66051100	1	8.70E-17	4.10E-14	+	0.286608	0.2866	+	T	G	0.475101301	0.0340678 +	T	G	0.4924	0.0379
rs2411357	LC_IGP81	IgG1 Fn/Bn	14	66051100	1	2.83E-13	1.15E-10	+	0.252357	0.2426	+	T	G	0.475101301	0.0342683 +	T	G	0.4924	0.0376
rs2411357	LC_IGP21	IgG1 Fucosylation	14	66051100	1	2.09E-15	5.47E-13	+	0.272768	0.2729	+	T	G	0.474299322	0.0340335 +	T	G	0.4924	0.0378
rs2411357	LC_IGP28	IgG1 G1S1/(G1+G1S1)	14	66051100	1	1.62E-10	2.90E-10	+	0.219016	0.2288	+	T	G	0.474504281	0.034034 +	T	G	0.4924	0.0363
rs2411357	LC_IGP26	IgG1 GS1/(G+GS1)	14	66051100	1	2.86E-08	1.43E-09	+	0.190836	0.2251	+	T	G	0.474504281	0.0342206 +	T	G	0.4924	0.0372
rs2411357	LC_IGP27	IgG1 GS1/(G0+G+GS1)	14	66051100	1	1.40E-09	2.80E-08	+	0.207815	0.2051	+	T	G	0.474504281	0.0341193 +	T	G	0.4924	0.0369
rs2411357	LC_IGP11	IgG1_G0	14	66051100	1	3.08E-19	3.02E-14	-	-0.305005	-0.2861	+	T	G	0.474824679	0.0335958 +	T	G	0.4924	0.0377
rs2411357	LC_IGP_R1	IgG1_G0F/IgG1_G0	14	66051100	1	2.09E-16	6.56E-12	+	0.279951	0.2596	+	T	G	0.474824679	0.0337132 +	T	G	0.4924	0.0378
rs2411357	LC_IGP_R4	IgG1_G0FN/IgG1_G0N	14	66051100	1	9.27E-15	3.53E-13	+	0.265686	0.2764	+	T	G	0.47537477	0.0339668 +	T	G	0.4924	0.038
rs2411357	LC_IGP52	IgG1_G0n	14	66051100	1	2.83E-19	2.63E-14	-	-0.307678	-0.2874	+	T	G	0.475101301	0.033848 +	T	G	0.4924	0.0377
rs2411357	LC_IGP14	IgG1_G0N	14	66051100	1	8.39E-17	4.29E-11	-	-0.284988	-0.2472	+	T	G	0.47537477	0.0338619 +	T	G	0.4924	0.0375
rs2411357	LC_IGP55	IgG1_G0Nn	14	66051100	1	3.84E-17	4.41E-11	-	-0.289798	-0.2473	+	T	G	0.475101301	0.0340448 +	T	G	0.4924	0.0375
rs2411357	LC_IGP12	IgG1_G1	14	66051100	1	3.36E-16	1.21E-13	-	-0.278651	-0.2809	+	T	G	0.474824679	0.0337977 +	T	G	0.4924	0.0379
rs2411357	LC_IGP_R2	IgG1_G1F/IgG1_G1	14	66051100	1	2.26E-17	1.39E-13	+	0.289459	0.2796	+	T	G	0.474824679	0.0337566 +	T	G	0.4924	0.0378
rs2411357	LC_IGP_R5	IgG1_G1FN/IgG1_G1N	14	66051100	1	1.76E-14	2.17E-17	+	0.263983	0.3211	+	T	G	0.47620818	0.0341132 +	T	G	0.4924	0.0378
rs2411357	LC_IGP53	IgG1_G1n	14	66051100	1	2.71E-15	2.07E-13	-	-0.272482	-0.278	+	T	G	0.475101301	0.0341368 +	T	G	0.4924	0.0379
rs2411357	LC_IGP15	IgG1_G1N	14	66051100	1	2.95E-11	1.15E-10	-	-0.229314	-0.2427	+	T	G	0.47620818	0.0342489 +	T	G	0.4924	0.0377
rs2411357	LC_IGP56	IgG1_G1Nn	14	66051100	1	1.22E-11	2.32E-10	-	-0.234631	-0.2383	+	T	G	0.475101301	0.0343651 +	T	G	0.4924	0.0376
rs2411357	LC_IGP_R36	IgG1_G1NS1/IgG1_G1N	14	66051100	1	2.86E-08	4.23E-12	+	0.190615	0.2553	+	T	G	0.476149507	0.0341769 +	T	G	0.4924	0.0368
rs2411357	LC_IGP_R32	IgG1_G1S1/IgG1_G1	14	66051100	1	3.10E-10	4.39E-10	+	0.215662	0.2268	+	T	G	0.474504281	0.0340524 +	T	G	0.4924	0.0364
rs2411357	LC_IGP_R3	IgG1_G2F/IgG1_G2	14	66051100	1	1.50E-15	9.42E-15	+	0.272906	0.2912	+	T	G	0.474519866	0.0338748 +	T	G	0.4924	0.0376
rs2411357	LC_IGP_R8	IgG1_G2FS1/IgG1_G2S1	14	66051100	1	4.34E-10	1.21E-12	+	0.214273	0.2664	+	T	G	0.474229994	0.0341229 +	T	G	0.4924	0.0375
rs2411357	LC_IGP_R26	IgG1_G2N/IgG1_G1N	14	66051100	1	3.97E-12	1.32E-08	+	0.238604	0.2086	+	T	G	0.47581142	0.0341297 +	T	G	0.4924	0.0367
rs2411357	LC_IGP148	IgG2 FG0n total/G0n	14	66051100	1	4.53E-09	1.39E-13	+	0.201952	0.2776	+	T	G	0.473407943	0.0342538 +	T	G	0.4924	0.0375
rs2411357	LC_IGP97	IgG2_G0	14	66051100	1	8.88E-11	1.10E-13	-	-0.222024	-0.279	+	T	G	0.474519866	0.034012 +	T	G	0.4924	0.0376
rs2411357	LC_IGP_R41	IgG2_G0F/IgG2_G0	14	66051100	1	4.03E-08	6.14E-13	+	0.188227	0.2687	+	T	G	0.474519866	0.0341248 +	T	G	0.4924	0.0373
rs2411357	LC_IGP138	IgG2_G0n	14	66051100	1	5.27E-11	7.71E-14	-	-0.225528	-0.2812	+	T	G	0.473407943	0.0341302 +	T	G	0.4924	0.0376
rs2411357	LC_IGP_R64	IgG2_G2/IgG2_G1	14	66051100	1	5.79E-09	2.03E-12	+	0.200813	0.2585	+	T	G	0.473570685	0.0343031 +	T	G	0.4924	0.0368
rs8016322	LC_IGP79	IgG1 BG1n/G1n	14	66051536	1	6.40E-09	1.80E-07	-	-0.207162	-0.203	+	A	G	0.586621437	0.0354886 +	A	G	0.6177	0.0389
rs8016322	LC_IGP77	IgG1 Bn	14	66051536	1	3.74E-09	2.71E-07	-	-0.210361	-0.2009	+	A	G	0.586621437	0.0354842 +	A	G	0.6177	0.0391
rs8016322	LC_IGP86	IgG1 Bn/Fn total	14	66051536	1	1.02E-09	2.01E-07	-	-0.217743	-0.2032	+	A	G	0.586621437	0.0354487 +	A	G	0.6177	0.0391
rs8016322	LC_IGP84	IgG1 FBn/Bn total	14	66051536	1	3.52E-11	1.09E-09	+	0.236411	0.2399	+	A	G	0.586621437	0.0354482 +	A	G	0.6177	0.0394
rs8016322	LC_IGP62	IgG1 FG0n total/G0n	14	66051536	1	1.09E-12	4.01E-08	+	0.252457	0.2152	+	A	G	0.586621437	0.0351783 +	A	G	0.6177	0.0392
rs8016322	LC_IGP63	IgG1 FG1n total/G1n	14	66051536	1	8.80E-13	1.44E-09	+	0.254216	0.2369	+	A	G	0.586621437	0.0352775 +	A	G	0.6177	0.0392
rs8016322	LC_IGP64	IgG1 FG2n total/G2n	14	66051536	1	2.23E-10	9.51E-10	+	0.226266	0.2383	+	A	G	0.586621437	0.0354331 +	A	G	0.6177	0.039
rs8016322	LC_IGP61	IgG1 Fn total	14	66051536	1	1.70E-12	2.08E-09	+	0.250865	0.2348	+	A	G	0.586621437	0.0352668 +	A	G	0.6177	0.0392
rs8016322	LC_IGP81	IgG1 Fn/Bn	14	66051536	1	3.24E-09	5.49E-07	+	0.211077	0.1949	+	A	G	0.586621437	0.0354617 +	A	G	0.6177	0.0389
rs8016322	LC_IGP21	IgG1 Fucosylation	14	66051536	1	7.06E-12	1.25E-08	+	0.242819	0.2228	+	A	G	0.586194821	0.0351527 +	A	G	0.6177	0.0391
rs8016322	LC_IGP28	IgG1 G1S1/(G1+G1S1)	14	66051536	1	3.19E-08	8.85E-07	+	0.195003	0.1849	+	A	G	0.586121101	0.0350889 +	A	G	0.6177	0.0376
rs8016322	LC_IGP11	IgG1_G0	14	66051536	1	5.25E-15	1.35E-09	-	-0.274284	-0.2363	+	A	G	0.586305131	0.0347385 +	A	G	0.6177	0.039
rs8016322	LC_IGP_R1	IgG1_G0F/IgG1_G0	14	66051536	1	7.02E-13	4.40E-08	+	0.252038	0.2139	+	A	G	0.586305131	0.0348253 +	A	G	0.6177	0.0391
rs8016322	LC_IGP_R4	IgG1_G0FN/IgG1_G0N	14	66051536	1	3.87E-10	5.49E-08	+	0.221484	0.2137	+	A	G	0.587128291	0.0351671 +	A	G	0.6177	0.0393
rs8016322	LC_IGP52	IgG1_G0n	14	66051536	1	1.61E-14	1.33E-09	-	-0.271751	-0.2368	+	A	G	0.586621437	0.0350628 +	A	G	0.6177	0.0391
rs8016322	LC_IGP14	IgG1_G0N	14	66051536	1	5.72E-11	9.09E-07	-	-0.231613	-0.1906	+	A	G	0.587128291	0.0351181 +	A	G	0.6177	0.0388
rs8016322	LC_IGP55	IgG1_G0Nn	14	66051536	1	2.82E-11	8.64E-07	-	-0.236751	-0.1912	+	A	G	0.586621437	0.0353209 +	A	G	0.6177	0.0389
rs8016322	LC_IGP12	IgG1_G1	14	66051536	1	2.07E-12	3.53E-09	-	-0.247329	-0.2312	+	A	G	0.586610568	0.034911 +	A	G	0.6177	0.0392
rs8016322	LC_IGP_R2	IgG1_G1F/IgG1_G1	14	66051536	1	7.56E-13	3.20E-09	+	0.252244	0.2314	+	A	G	0.586610568	0.0349042 +	A	G	0.6177	0.0391
rs8016322	LC_IGP_R5	IgG1_G1FN/IgG1_G1N	14	66051536	1	1.51E-10	2.71E-10	+	0.227403	0.2472	+	A	G	0.587520295	0.0352784 +	A	G	0.6177	0.0391
rs8016322	LC_IGP53	IgG1_G1n	14	66051536	1	8.63E-12	5.20E-09	-	-0.242799	-0.2285	+	A	G	0.586621437	0.035299 +	A	G	0.6177	0.0391
rs8016322	LC_IGP56	IgG1_G1Nn	14	66051536	1	4.02E-08	1.18E-06	-	-0.196035	-0.1888	+	A	G	0.586621437	0.0355344 +	A	G	0.6177	0.0389
rs8016322	LC_IGP_R32	IgG1_G1S1/IgG1_G1	14	66051536	1	4.23E-08	9.89E-07	+	0.193301	0.1843	+	A	G	0.586121101	0.0351001 +	A	G	0.6177	0.0377
rs8016322	LC_IGP_R3	IgG1_G2F/IgG1_G2	14	66051536	1	1.92E-11	1.86E-09	+	0.236664	0.2336	+	A	G	0.58606846	0.0350127 +	A	G	0.6177	0.0389
rs8016322	LC_IGP138	IgG2_G0n	14	66051536	1	3.99E-08	1.68E-10	-	-0.194075	-0.2488	+	A	G	0.585472906	0.0351729 +	A	G	0.6176	0.039
rs1958559	LC_IGP84	IgG1 FBn/Bn total	14	66052055	1	2.02E-08	2.21E-07	+	0.198955	0.2005	+	T	C	0.565784387	0.0352867 +	T	C	0.5895	0.0387
rs1958559	LC_IGP63	IgG1 FG1n total/G1n	14	66052055	1	6.83E-10	1.43E-07	+	0.21803	0.2026	+	T	C	0.565784387	0.0351237 +	T	C	0.5895	0.0385
rs1958559	LC_IGP64	IgG1 FG2n total/G2n	14	66052055	1	4.32E-08	4.67E-08	+	0.193995	0.2092	+	T	C	0.565784387	0.0352484 +	T	C	0.5895	0.0383
rs1958559	LC_IGP61	IgG1 Fn total	14	66052055	1	1.94E-09	3.77E-07	+	0.21203	0.1958	+	T	C	0.565784387	0.0351213 +	T	C	0.5895	0.0385
rs1958559	LC_IGP11	IgG1_G0	14	66052055	1	2.87E-11	8.80E-07	-	-0.232124	-0.1884	+	T	C	0.565455406	0.0346481 +	T	C	0.5895	0.0383
rs1958559	LC_IGP52	IgG1_G0n	14	66052055	1	5.08E-11	9.70E-07	-	-0.231085	-0.188	+	T	C	0.565784387	0.0349401 +	T	C	0.5895	0.0384
rs1958559	LC_IGP12	IgG1_G1	14	66052055	1	2.14E-09	3.03E-07	-	-0.209409	-0.1972	+	T	C	0.565760843	0.0347832 +	T	C	0.5895	0.0385
rs1958559	LC_IGP_R2	IgG1_G1F/IgG1_G1	14	66052055	1	6.61E-10	2.78E-07	+	0.216022	0.1974	+	T	C	0.565760843	0.0347731 +	T	C	0.5895	0.0384
rs1958559	LC_IGP_R5	IgG1_G1FN/IgG1_G1N	14	66052055	1	3.51E-08	3.07E-08	+	0.194539	0.2132	+	T	C	0.56652952	0.0351105 +	T	C	0.5895	0.0385
rs1958559	LC_IGP53	IgG1_G1n	14	66052055	1	5.49E-09	4.22E-07	-	-0.206045	-0.1946	+	T	C	0.565784387	0.0351405 +	T	C	0.5895	0.0385
rs1958559	LC_IGP_R3	IgG1_G2F/IgG1_G2	14	66052055	1	5.20E-09	8.34E-08	+	0.204702	0.2049	+	T	C	0.56520599	0.0348592 +	T	C	0.5895	0.0382
rs7160780	LC_IGP79	IgG1 BG1n/G1n	14	66052713	1	8.19E-09	1.90E-07	-	-0.205805	-0.2026	+	T	G	0.587163259	0.0355122 +	T	G	0.6176	0.0389
rs7160780	LC_IGP77	IgG1 Bn	14	66052713	1	4.95E-09	2.84E-07	-	-0.208824	-0.2006	+	T	G	0.587163259	0.0355086 +	T	G	0.6176	0.0391
rs7160780	LC_IGP86	IgG1 Bn/Fn total	14	66052713	1	1.42E-09	2.10E-07	-	-0.21599	-0.2029	+	T	G	0.587163259	0.0354741 +	T	G	0.6176	0.0391
rs7160780	LC_IGP84	IgG1 FBn/Bn total	14	66052713	1	4.53E-11	1.13E-09	+	0.235225	0.2397	+	T	G	0.587163259	0.0354719 +	T	G	0.6176	0.0394
rs7160780	LC_IGP62	IgG1 FG0n total/G0n	14	66052713	1	2.11E-12	4.13E-08	+	0.249363	0.215	+	T	G	0.587163259	0.0352108 +	T	G	0.6176	0.0392
rs7160780	LC_IGP63	IgG1 FG1n total/G1n	14	66052713	1	1.48E-12	1.52E-09	+	0.251845	0.2366	+	T	G	0.587163259	0.0353069 +	T	G	0.6176	0.0392
rs7160780	LC_IGP64	IgG1 FG2n total/G2n	14	66052713	1	3.93E-10	1.01E-09	+	0.22328	0.2379	+	T	G	0.587163259	0.0354637 +	T	G	0.6176	0.039
rs7160780	LC_IGP61	IgG1 Fn total	14	66052713	1	3.15E-12	2.18E-09	+	0.247961	0.2345	+	T	G	0.587163259	0.0352984 +	T	G	0.6176	0.0392
rs7160780	LC_IGP81	IgG1 Fn/Bn	14	66052713	1	4.45E-09	5.76E-07	+	0.209338	0.1946	+	T	G	0.587163259	0.0354868 +	T	G	0.6176	0.0389
rs7160780	LC_IGP21	IgG1 Fucosylation	14	66052713	1	1.29E-11	1.30E-08	+	0.239916	0.2225	+	T	G	0.586733354	0.0351835 +	T	G	0.6176	0.0391
rs7160780	LC_IGP28	IgG1 G1S1/(G1+G1S1)	14	66052713	1	2.65E-08	8.99E-07	+	0.196235	0.1848	+	T	G	0.586655963	0.0351028 +	T	G	0.6176	0.0376
rs7160780	LC_IGP11	IgG1_G0	14	66052713	1	8.98E-15	1.39E-09	-	-0.272087	-0.2361	+	T	G	0.58683934	0.0347674 +	T	G	0.6176	0.039
rs7160780	LC_IGP_R1	IgG1_G0F/IgG1_G0	14	66052713	1	1.41E-12	4.53E-08	+	0.248834	0.2137	+	T	G	0.58683934	0.0348576 +	T	G	0.6176	0.0391
rs7160780	LC_IGP_R4	IgG1_G0FN/IgG1_G0N	14	66052713	1	5.25E-10	5.62E-08	+	0.219926	0.2135	+	T	G	0.587663809	0.0351916 +	T	G	0.6176	0.0393
rs7160780	LC_IGP52	IgG1_G0n	14	66052713	1	2.92E-14	1.37E-09	-	-0.269234	-0.2367	+	T	G	0.587163259	0.0350936 +	T	G	0.6176	0.0391
rs7160780	LC_IGP14	IgG1_G0N	14	66052713	1	7.26E-11	9.35E-07	-	-0.230482	-0.1904	+	T	G	0.587663809	0.0351411 +	T	G	0.6176	0.0388
rs7160780	LC_IGP55	IgG1_G0Nn	14	66052713	1	3.83E-11	8.88E-07	-	-0.235279	-0.191	+	T	G	0.587163259	0.0353458 +	T	G	0.6176	0.0389
rs7160780	LC_IGP12	IgG1_G1	14	66052713	1	3.53E-12	3.70E-09	-	-0.244843	-0.2309	+	T	G	0.587144777	0.0349399 +	T	G	0.6176	0.0392
rs7160780	LC_IGP_R2	IgG1_G1F/IgG1_G1	14	66052713	1	1.35E-12	3.39E-09	+	0.249608	0.231	+	T	G	0.587144777	0.0349341 +	T	G	0.6176	0.0391
rs7160780	LC_IGP_R5	IgG1_G1FN/IgG1_G1N	14	66052713	1	1.90E-10	2.85E-10	+	0.226292	0.2469	+	T	G	0.588058118	0.0353015 +	T	G	0.6176	0.0391
rs7160780	LC_IGP53	IgG1_G1n	14	66052713	1	1.57E-11	5.43E-09	-	-0.23991	-0.2282	+	T	G	0.587163259	0.03533 +	T	G	0.6176	0.0391
rs7160780	LC_IGP_R32	IgG1_G1S1/IgG1_G1	14	66052713	1	3.64E-08	1.01E-06	+	0.194325	0.1842	+	T	G	0.586655963	0.0351147 +	T	G	0.6176	0.0377
rs7160780	LC_IGP_R3	IgG1_G2F/IgG1_G2	14	66052713	1	3.72E-11	1.98E-09	+	0.233402	0.2332	+	T	G	0.586602995	0.0350443 +	T	G	0.6176	0.0389
rs7161123	LC_IGP78	IgG1 BG0n/G0n	14	66052901	1	7.46E-14	9.03E-10	-	-0.257866	-0.2303	+	A	G	0.475106877	0.0341725 +	A	G	0.493	0.0376
rs7161123	LC_IGP79	IgG1 BG1n/G1n	14	66052901	1	4.74E-13	9.44E-12	-	-0.25007	-0.256	+	A	G	0.475106877	0.0342906 +	A	G	0.493	0.0376
rs7161123	LC_IGP32	IgG1 BG1S1/(BG1+BG1S1)	14	66052901	1	3.02E-08	3.74E-12	+	0.190237	0.2554	+	A	G	0.476155055	0.0341696 +	A	G	0.493	0.0368
rs7161123	LC_IGP77	IgG1 Bn	14	66052901	1	5.04E-13	2.36E-11	-	-0.249842	-0.2521	+	A	G	0.475106877	0.0342987 +	A	G	0.493	0.0377
rs7161123	LC_IGP86	IgG1 Bn/Fn total	14	66052901	1	7.38E-14	1.42E-11	-	-0.25851	-0.255	+	A	G	0.475106877	0.0342512 +	A	G	0.493	0.0377
rs7161123	LC_IGP84	IgG1 FBn/Bn total	14	66052901	1	2.22E-15	4.01E-16	+	0.274236	0.3092	+	A	G	0.475106877	0.0342473 +	A	G	0.493	0.038
rs7161123	LC_IGP62	IgG1 FG0n total/G0n	14	66052901	1	2.16E-17	2.92E-12	+	0.291461	0.2642	+	A	G	0.475106877	0.0339622 +	A	G	0.493	0.0378
rs7161123	LC_IGP63	IgG1 FG1n total/G1n	14	66052901	1	1.09E-17	1.78E-14	+	0.294986	0.2898	+	A	G	0.475106877	0.034048 +	A	G	0.493	0.0378
rs7161123	LC_IGP64	IgG1 FG2n total/G2n	14	66052901	1	4.31E-14	3.16E-15	+	0.261023	0.2965	+	A	G	0.475106877	0.0342565 +	A	G	0.493	0.0376
rs7161123	LC_IGP68	IgG1 FG2n/G2n	14	66052901	1	1.82E-10	1.91E-08	+	0.220872	0.2108	+	A	G	0.475106877	0.0344198 +	A	G	0.493	0.0375
rs7161123	LC_IGP61	IgG1 Fn total	14	66052901	1	8.75E-17	4.19E-14	+	0.286578	0.286	+	A	G	0.475106877	0.0340676 +	A	G	0.493	0.0379
rs7161123	LC_IGP81	IgG1 Fn/Bn	14	66052901	1	2.85E-13	1.18E-10	+	0.252329	0.2421	+	A	G	0.475106877	0.034268 +	A	G	0.493	0.0376
rs7161123	LC_IGP21	IgG1 Fucosylation	14	66052901	1	2.11E-15	5.49E-13	+	0.272738	0.2725	+	A	G	0.474304871	0.0340332 +	A	G	0.493	0.0378
rs7161123	LC_IGP28	IgG1 G1S1/(G1+G1S1)	14	66052901	1	1.62E-10	2.80E-10	+	0.218996	0.2287	+	A	G	0.474509786	0.0340337 +	A	G	0.493	0.0362
rs7161123	LC_IGP26	IgG1 GS1/(G+GS1)	14	66052901	1	2.89E-08	1.37E-09	+	0.190785	0.225	+	A	G	0.474509786	0.0342204 +	A	G	0.493	0.0371
rs7161123	LC_IGP27	IgG1 GS1/(G0+G+GS1)	14	66052901	1	1.41E-09	2.69E-08	+	0.207772	0.205	+	A	G	0.474509786	0.034119 +	A	G	0.493	0.0369
rs7161123	LC_IGP11	IgG1_G0	14	66052901	1	3.10E-19	2.95E-14	-	-0.304976	-0.2856	+	A	G	0.474830177	0.0335955 +	A	G	0.493	0.0376
rs7161123	LC_IGP_R1	IgG1_G0F/IgG1_G0	14	66052901	1	2.09E-16	6.57E-12	+	0.279934	0.2592	+	A	G	0.474830177	0.0337129 +	A	G	0.493	0.0377
rs7161123	LC_IGP_R4	IgG1_G0FN/IgG1_G0N	14	66052901	1	9.27E-15	3.52E-13	+	0.265685	0.2759	+	A	G	0.475380282	0.0339664 +	A	G	0.493	0.0379

rs7161123	LC_IGP52	IgG1_G0n	14	66052901	1	2.85E-19	2.57E-14	-	-0.307649	-0.2869	+	A	G	0.475106877	0.0338478	+	A	G	0.493	0.0377
rs7161123	LC_IGP14	IgG1_G0N	14	66052901	1	8.43E-17	4.25E-11	-	-0.284966	-0.2468	+	A	G	0.475380282	0.0338616	+	A	G	0.493	0.0374
rs7161123	LC_IGP55	IgG1_G0Nn	14	66052901	1	3.86E-17	4.37E-11	-	-0.289777	-0.2469	+	A	G	0.475106877	0.0340445	+	A	G	0.493	0.0375
rs7161123	LC_IGP12	IgG1_G1	14	66052901	1	3.39E-16	1.22E-13	-	-0.278618	-0.2804	+	A	G	0.474830177	0.0337975	+	A	G	0.493	0.0378
rs7161123	LC_IGP_R2	IgG1_G1F/IgG1_G1	14	66052901	1	2.28E-17	1.41E-13	+	0.289417	0.2791	+	A	G	0.474830177	0.0337564	+	A	G	0.493	0.0377
rs7161123	LC_IGP_R5	IgG1_G1FN/IgG1_G1N	14	66052901	1	1.77E-14	2.23E-17	+	0.263957	0.3205	+	A	G	0.476213715	0.034113	+	A	G	0.493	0.0378
rs7161123	LC_IGP53	IgG1_G1n	14	66052901	1	2.73E-15	2.11E-13	-	-0.27245	-0.2775	+	A	G	0.475106877	0.0341366	+	A	G	0.493	0.0378
rs7161123	LC_IGP15	IgG1_G1N	14	66052901	1	2.97E-11	1.21E-10	-	-0.229286	-0.242	+	A	G	0.476213715	0.0342486	+	A	G	0.493	0.0376
rs7161123	LC_IGP56	IgG1_G1Nn	14	66052901	1	1.23E-11	2.44E-10	-	-0.234604	-0.2377	+	A	G	0.475106877	0.0343648	+	A	G	0.493	0.0376
rs7161123	LC_IGP_R36	IgG1_G1NS1/IgG1_G1N	14	66052901	1	2.88E-08	4.32E-12	+	0.190574	0.2548	+	A	G	0.476155055	0.0341767	+	A	G	0.493	0.0368
rs7161123	LC_IGP_R32	IgG1_G1S1/IgG1_G1	14	66052901	1	3.11E-10	4.25E-10	+	0.215642	0.2266	+	A	G	0.474509786	0.0340521	+	A	G	0.493	0.0363
rs7161123	LC_IGP_R3	IgG1_G2F/IgG1_G2	14	66052901	1	1.52E-15	9.75E-15	+	0.272859	0.2907	+	A	G	0.474525367	0.0338746	+	A	G	0.493	0.0375
rs7161123	LC_IGP_R8	IgG1_G2FS1/IgG1_G2S1	14	66052901	1	4.36E-10	1.25E-12	+	0.214257	0.2659	+	A	G	0.474235492	0.0341226	+	A	G	0.493	0.0374
rs7161123	LC_IGP_R26	IgG1_G2N/IgG1_G1N	14	66052901	1	3.99E-12	1.31E-08	+	0.238586	0.2083	+	A	G	0.475816975	0.0341293	+	A	G	0.493	0.0366
rs7161123	LC_IGP148	IgG2 FG0n total/G0n	14	66052901	1	4.54E-09	1.37E-13	+	0.20194	0.2772	+	A	G	0.473413485	0.0342534	+	A	G	0.493	0.0375
rs7161123	LC_IGP97	IgG2_G0	14	66052901	1	8.92E-11	1.08E-13	-	-0.222001	-0.2786	+	A	G	0.474525367	0.0340117	+	A	G	0.493	0.0375
rs7161123	LC_IGP_R41	IgG2_G0F/IgG2_G0	14	66052901	1	4.04E-08	6.08E-13	+	0.188211	0.2683	+	A	G	0.474525367	0.0341244	+	A	G	0.493	0.0373
rs7161123	LC_IGP138	IgG2_G0n	14	66052901	1	5.29E-11	7.59E-14	-	-0.225505	-0.2808	+	A	G	0.473413485	0.0341299	+	A	G	0.493	0.0376
rs7161123	LC_IGP_R64	IgG2_G2/IgG2_G1	14	66052901	1	5.81E-09	1.92E-12	+	0.20079	0.2584	+	A	G	0.473576193	0.0343028	+	A	G	0.493	0.0367
rs34640387	LC_IGP61	IgG1 Fn total	14	66053006	1	4.51E-08	1.92E-06	-	-0.237813	-0.2432	+	A	G	0.203463639	0.0432703	+	A	G	0.1664	0.0511
rs34772005	LC_IGP61	IgG1 Fn total	14	66053091	1	4.51E-08	2.03E-06	-	-0.237816	-0.2423	+	A	G	0.203462195	0.0432703	+	A	G	0.167	0.051
rs2411356	LC_IGP79	IgG1 BG1n/G1n	14	66053161	1	7.97E-09	2.68E-07	-	-0.205792	-0.1997	+	A	G	0.587785626	0.0354822	+	A	G	0.616	0.0388
rs2411356	LC_IGP77	IgG1 Bn	14	66053161	1	4.80E-09	4.54E-07	-	-0.208826	-0.1966	+	A	G	0.587785626	0.0354785	+	A	G	0.616	0.039
rs2411356	LC_IGP86	IgG1 Bn/Fn total	14	66053161	1	1.36E-09	3.37E-07	-	-0.216047	-0.199	+	A	G	0.587785626	0.0354438	+	A	G	0.616	0.039
rs2411356	LC_IGP84	IgG1 FBn/Bn total	14	66053161	1	4.66E-11	1.91E-09	+	0.234881	0.236	+	A	G	0.587785626	0.0354431	+	A	G	0.616	0.0393
rs2411356	LC_IGP62	IgG1 FG0n total/G0n	14	66053161	1	1.81E-12	7.82E-08	+	0.249906	0.21	+	A	G	0.587785626	0.0351783	+	A	G	0.616	0.0391
rs2411356	LC_IGP63	IgG1 FG1n total/G1n	14	66053161	1	1.29E-12	2.55E-09	+	0.252278	0.2328	+	A	G	0.587785626	0.0352748	+	A	G	0.616	0.0391
rs2411356	LC_IGP64	IgG1 FG2n total/G2n	14	66053161	1	3.22E-10	1.58E-09	+	0.224191	0.2348	+	A	G	0.587785626	0.03543	+	A	G	0.616	0.0389
rs2411356	LC_IGP61	IgG1 Fn total	14	66053161	1	2.66E-12	3.92E-09	+	0.248594	0.2302	+	A	G	0.587785626	0.0352655	+	A	G	0.616	0.0391
rs2411356	LC_IGP81	IgG1 Fn/Bn	14	66053161	1	4.22E-09	9.16E-07	+	0.209463	0.1906	+	A	G	0.587785626	0.0354563	+	A	G	0.616	0.0388
rs2411356	LC_IGP21	IgG1 Fucosylation	14	66053161	1	1.11E-11	2.49E-08	+	0.240476	0.2176	+	A	G	0.587352651	0.0351513	+	A	G	0.616	0.039
rs2411356	LC_IGP28	IgG1 G1S1/(G1+G1S1)	14	66053161	1	3.77E-08	1.26E-06	+	0.193919	0.182	+	A	G	0.587270336	0.0350815	+	A	G	0.616	0.0376
rs2411356	LC_IGP11	IgG1_G0	14	66053161	1	8.27E-15	3.68E-09	-	-0.272223	-0.2292	+	A	G	0.587452963	0.0347373	+	A	G	0.616	0.0389
rs2411356	LC_IGP_R1	IgG1_G0F/IgG1_G0	14	66053161	1	1.15E-12	8.53E-08	+	0.249613	0.2088	+	A	G	0.587452963	0.0348249	+	A	G	0.616	0.039
rs2411356	LC_IGP_R4	IgG1_G0FN/IgG1_G0N	14	66053161	1	5.35E-10	1.02E-07	+	0.219639	0.2088	+	A	G	0.588278934	0.0351633	+	A	G	0.616	0.0392
rs2411356	LC_IGP52	IgG1_G0n	14	66053161	1	2.60E-14	3.60E-09	-	-0.26953	-0.2298	+	A	G	0.587785626	0.035062	+	A	G	0.616	0.0389
rs2411356	LC_IGP14	IgG1_G0N	14	66053161	1	7.76E-11	1.84E-06	-	-0.229949	-0.1846	+	A	G	0.588278934	0.0351138	+	A	G	0.616	0.0387
rs2411356	LC_IGP55	IgG1_G0Nn	14	66053161	1	3.98E-11	1.75E-06	-	-0.234889	-0.1852	+	A	G	0.587785626	0.0353173	+	A	G	0.616	0.0388
rs2411356	LC_IGP12	IgG1_G1	14	66053161	1	3.09E-12	6.37E-09	-	-0.245298	-0.2268	+	A	G	0.5877584	0.0349087	+	A	G	0.616	0.0391
rs2411356	LC_IGP_R2	IgG1_G1F/IgG1_G1	14	66053161	1	1.13E-12	5.82E-09	+	0.250261	0.2269	+	A	G	0.5877584	0.0349019	+	A	G	0.616	0.039
rs2411356	LC_IGP_R5	IgG1_G1FN/IgG1_G1N	14	66053161	1	1.89E-10	4.62E-10	+	0.226119	0.2436	+	A	G	0.588675892	0.0352726	+	A	G	0.616	0.0391
rs2411356	LC_IGP53	IgG1_G1n	14	66053161	1	1.31E-11	8.97E-09	-	-0.240613	-0.2244	+	A	G	0.587785626	0.0352969	+	A	G	0.616	0.039
rs2411356	LC_IGP_R32	IgG1_G1S1/IgG1_G1	14	66053161	1	4.96E-08	1.37E-06	+	0.192248	0.1816	+	A	G	0.587270336	0.0350925	+	A	G	0.616	0.0376
rs2411356	LC_IGP_R3	IgG1_G2F/IgG1_G2	14	66053161	1	2.92E-11	3.11E-09	+	0.234464	0.2301	+	A	G	0.587216993	0.0350106	+	A	G	0.616	0.0388
rs2411356	LC_IGP138	IgG2_G0n	14	66053161	1	4.73E-08	3.04E-10	-	-0.192954	-0.2448	+	A	G	0.586629926	0.0351652	+	A	G	0.6159	0.0389
rs4581615	LC_IGP78	IgG1 BG0n/G0n	14	66055943	1	7.46E-14	9.09E-10	-	-0.257868	-0.2302	+	A	C	0.475107807	0.0341725	+	A	C	0.493	0.0376
rs4581615	LC_IGP79	IgG1 BG1n/G1n	14	66055943	1	4.74E-13	9.49E-12	-	-0.250073	-0.2559	+	A	C	0.475107807	0.0342906	+	A	C	0.493	0.0376
rs4581615	LC_IGP32	IgG1 BG1S1/(BG1+BG1S1)	14	66055943	1	3.02E-08	3.75E-12	+	0.19024	0.2553	+	A	C	0.47615598	0.0341696	+	A	C	0.493	0.0368
rs4581615	LC_IGP77	IgG1 Bn	14	66055943	1	5.04E-13	2.37E-11	-	-0.249844	-0.2521	+	A	C	0.475107807	0.0342987	+	A	C	0.493	0.0377
rs4581615	LC_IGP86	IgG1 Bn/Fn total	14	66055943	1	7.38E-14	1.43E-11	-	-0.258513	-0.255	+	A	C	0.475107807	0.0342511	+	A	C	0.493	0.0377
rs4581615	LC_IGP84	IgG1 FBn/Bn total	14	66055943	1	2.22E-15	4.03E-16	+	0.274238	0.3092	+	A	C	0.475107807	0.0342473	+	A	C	0.493	0.038
rs4581615	LC_IGP62	IgG1 FG0n total/G0n	14	66055943	1	2.16E-17	2.94E-12	+	0.291462	0.2642	+	A	C	0.475107807	0.0339621	+	A	C	0.493	0.0378
rs4581615	LC_IGP63	IgG1 FG1n total/G1n	14	66055943	1	1.09E-17	1.79E-14	+	0.294988	0.2898	+	A	C	0.475107807	0.034048	+	A	C	0.493	0.0378
rs4581615	LC_IGP64	IgG1 FG2n total/G2n	14	66055943	1	4.31E-14	3.17E-15	+	0.261025	0.2965	+	A	C	0.475107807	0.0342565	+	A	C	0.493	0.0376
rs4581615	LC_IGP68	IgG1 FG2n/G2n	14	66055943	1	1.82E-10	1.91E-08	+	0.220874	0.2107	+	A	C	0.475107807	0.0344198	+	A	C	0.493	0.0375
rs4581615	LC_IGP61	IgG1 Fn total	14	66055943	1	8.75E-17	4.21E-14	+	0.286579	0.286	+	A	C	0.475107807	0.0340676	+	A	C	0.493	0.0379
rs4581615	LC_IGP81	IgG1 Fn/Bn	14	66055943	1	2.84E-13	1.19E-10	+	0.252332	0.242	+	A	C	0.475107807	0.034268	+	A	C	0.493	0.0376
rs4581615	LC_IGP21	IgG1 Fucosylation	14	66055943	1	2.11E-15	5.53E-13	+	0.27274	0.2725	+	A	C	0.474305795	0.0340332	+	A	C	0.493	0.0378
rs4581615	LC_IGP28	IgG1 G1S1/(G1+G1S1)	14	66055943	1	1.62E-10	2.81E-10	+	0.218997	0.2287	+	A	C	0.474510703	0.0340337	+	A	C	0.493	0.0362
rs4581615	LC_IGP26	IgG1 GS1/(G+GS1)	14	66055943	1	2.89E-08	1.37E-09	+	0.190781	0.2249	+	A	C	0.474510703	0.0342204	+	A	C	0.493	0.0371
rs4581615	LC_IGP27	IgG1 GS1/(G0+G+GS1)	14	66055943	1	1.41E-09	2.70E-08	+	0.207767	0.205	+	A	C	0.474510703	0.0341191	+	A	C	0.493	0.0369
rs4581615	LC_IGP11	IgG1_G0	14	66055943	1	3.10E-19	2.98E-14	-	-0.304976	-0.2856	+	A	C	0.474831093	0.0335955	+	A	C	0.493	0.0376
rs4581615	LC_IGP_R1	IgG1_G0F/IgG1_G0	14	66055943	1	2.09E-16	6.61E-12	+	0.279934	0.2591	+	A	C	0.474831093	0.0337128	+	A	C	0.493	0.0377
rs4581615	LC_IGP_R4	IgG1_G0FN/IgG1_G0N	14	66055943	1	9.27E-15	3.55E-13	+	0.265686	0.2759	+	A	C	0.4753812	0.0339664	+	A	C	0.493	0.0379
rs4581615	LC_IGP52	IgG1_G0n	14	66055943	1	2.85E-19	2.59E-14	-	-0.307649	-0.2869	+	A	C	0.475107807	0.0338478	+	A	C	0.493	0.0377
rs4581615	LC_IGP14	IgG1_G0N	14	66055943	1	8.43E-17	4.28E-11	-	-0.284968	-0.2468	+	A	C	0.4753812	0.0338616	+	A	C	0.493	0.0374
rs4581615	LC_IGP55	IgG1_G0Nn	14	66055943	1	3.86E-17	4.41E-11	-	-0.289778	-0.2469	+	A	C	0.475107807	0.0340445	+	A	C	0.493	0.0375
rs4581615	LC_IGP12	IgG1_G1	14	66055943	1	3.39E-16	1.23E-13	-	-0.278619	-0.2804	+	A	C	0.474831093	0.0337975	+	A	C	0.493	0.0378
rs4581615	LC_IGP_R2	IgG1_G1F/IgG1_G1	14	66055943	1	2.28E-17	1.42E-13	+	0.289419	0.2791	+	A	C	0.474831093	0.0337564	+	A	C	0.493	0.0377
rs4581615	LC_IGP_R5	IgG1_G1FN/IgG1_G1N	14	66055943	1	1.76E-14	2.24E-17	+	0.26396	0.3204	+	A	C	0.476214637	0.0341129	+	A	C	0.493	0.0378
rs4581615	LC_IGP53	IgG1_G1n	14	66055943	1	2.73E-15	2.12E-13	-	-0.272452	-0.2775	+	A	C	0.475107807	0.0341366	+	A	C	0.493	0.0378
rs4581615	LC_IGP15	IgG1_G1N	14	66055943	1	2.97E-11	1.22E-10	-	-0.229289	-0.242	+	A	C	0.476214637	0.0342486	+	A	C	0.493	0.0376
rs4581615	LC_IGP56	IgG1_G1Nn	14	66055943	1	1.22E-11	2.45E-10	-	-0.234607	-0.2377	+	A	C	0.475107807	0.0343648	+	A	C	0.493	0.0376
rs4581615	LC_IGP_R36	IgG1_G1NS1/IgG1_G1N	14	66055943	1	2.88E-08	4.32E-12	+	0.190576	0.2548	+	A	C	0.47615598	0.0341767	+	A	C	0.493	0.0368
rs4581615	LC_IGP_R32	IgG1_G1S1/IgG1_G1	14	66055943	1	3.11E-10	4.26E-10	+	0.215643	0.2266	+	A	C	0.474510703	0.0340521	+	A	C	0.493	0.0363
rs4581615	LC_IGP_R3	IgG1_G2F/IgG1_G2	14	66055943	1	1.52E-15	9.80E-15	+	0.272861	0.2906	+	A	C	0.474526284	0.0338746	+	A	C	0.493	0.0375
rs4581615	LC_IGP_R8	IgG1_G2FS1/IgG1_G2S1	14	66055943	1	4.35E-10	1.26E-12	+	0.21426	0.2658	+	A	C	0.474236408	0.0341226	+	A	C	0.493	0.0374
rs4581615	LC_IGP_R26	IgG1_G2N/IgG1_G1N	14	66055943	1	3.99E-12	1.32E-08	+	0.238588	0.2083	+	A	C	0.475817901	0.0341293	+	A	C	0.493	0.0366
rs4581615	LC_IGP148	IgG2 FG0n total/G0n	14	66055943	1	4.53E-09	1.37E-13	+	0.201942	0.2772	+	A	C	0.473414409	0.0342534	+	A	C	0.493	0.0375
rs4581615	LC_IGP97	IgG2_G0	14	66055943	1	8.92E-11	1.09E-13	-	-0.222002	-0.2786	+	A	C	0.474526284	0.0340116	+	A	C	0.493	0.0375
rs4581615	LC_IGP_R41	IgG2_G0F/IgG2_G0	14	66055943	1	4.04E-08	6.09E-13	+	0.188214	0.2683	+	A	C	0.474526284	0.0341244	+	A	C	0.493	0.0373
rs4581615	LC_IGP138	IgG2_G0n	14	66055943	1	5.29E-11	7.60E-14	-	-0.225506	-0.2808	+	A	C	0.473414409	0.0341299	+	A	C	0.493	0.0376
rs4581615	LC_IGP_R64	IgG2_G2/IgG2_G1	14	66055943	1	5.81E-09	1.92E-12	+	0.200789	0.2584	+	A	C	0.473577111	0.0343028	+	A	C	0.493	0.0367
rs17753508	LC_IGP61	IgG1 Fn total	14	66057452	1	4.05E-08	1.53E-06	-	-0.23896	-0.2462	+	G	A	0.200836185	0.0433265	+	G	A	0.1642	0.0512
rs3783709	LC_IGP78	IgG1 BG0n/G0n	14	66058664	1	7.46E-14	9.08E-10	-	-0.257868	-0.2302	+	A	T	0.475108426	0.0341725	+	A	T	0.493	0.0376
rs3783709	LC_IGP79	IgG1 BG1n/G1n	14	66058664	1	4.74E-13	9.48E-12	-	-0.250071	-0.2559	+	A	T	0.475108426	0.0342906	+	A	T	0.493	0.0376
rs3783709	LC_IGP32	IgG1 BG1S1/(BG1+BG1S1)	14	66058664	1	3.02E-08	3.75E-12	+	0.190238	0.2554	+	A	T	0.476156597	0.0341696	+	A	T	0.493	0.0368
rs3783709	LC_IGP77	IgG1 Bn	14	66058664	1	5.04E-13	2.37E-11	-	-0.249843	-0.2521	+	A	T	0.475108426	0.0342987	+	A	T	0.493	0.0377
rs3783709	LC_IGP86	IgG1 Bn/Fn total	14	66058664	1	7.38E-14	1.43E-11	-	-0.258511	-0.255	+	A	T	0.475108426	0.0342511	+	A	T	0.493	0.0377
rs3783709	LC_IGP84	IgG1 FBn/Bn total	14	66058664	1	2.22E-15	4.03E-16	+	0.274238	0.3092	+	A	T	0.475108426	0.0342473	+	A	T	0.493	0.038
rs3783709	LC_IGP62	IgG1 FG0n total/G0n	14	66058664	1	2.16E-17	2.94E-12	+	0.291462	0.2642	+	A	T	0.475108426	0.0339621	+	A	T	0.493	0.0378
rs3783709	LC_IGP63	IgG1 FG1n total/G1n	14	66058664	1	1.09E-17	1.79E-14	+	0.294985	0.2898	+	A	T	0.475108426	0.034048	+	A	T	0.493	0.0378
rs3783709	LC_IGP64	IgG1 FG2n total/G2n	14	66058664	1	4.31E-14	3.17E-15	+	0.261023	0.2965	+	A	T	0.475108426	0.0342565	+	A	T	0.493	0.0376
rs3783709	LC_IGP68	IgG1 FG2n/G2n	14	66058664	1	1.82E-10	1.91E-08	+	0.220871	0.2107	+	A	T	0.475108426	0.0344198	+	A	T	0.493	0.0375
rs3783709	LC_IGP61	IgG1 Fn total	14	66058664	1	8.75E-17	4.21E-14	+	0.286578	0.286	+	A	T	0.475108426	0.0340676	+	A	T	0.493	0.0379
rs3783709	LC_IGP81	IgG1 Fn/Bn	14	66058664	1	2.85E-13	1.19E-10	+	0.252331	0.242	+	A	T	0.475108426	0.034268	+	A	T	0.493	0.0376
rs3783709	LC_IGP21	IgG1 Fucosylation	14	66058664	1	2.11E-15	5.52E-13	+	0.272738	0.2725	+	A	T	0.474306412	0.0340332	+	A	T	0.493	0.0378
rs3783709	LC_IGP28	IgG1 G1S1/(G1+G1S1)	14	66058664	1	1.62E-10	2.80E-10	+	0.218996	0.2287	+	A	T	0.474511315	0.0340337	+	A	T	0.493	0.0362
rs3783709	LC_IGP26	IgG1 GS1/(G+GS1)	14	66058664	1	2.89E-08	1.37E-09	+	0.190779	0.2249	+	A	T	0.474511315	0.0342204	+	A	T	0.493	0.0371
rs3783709	LC_IGP27	IgG1 GS1/(G0+G+GS1)	14	66058664	1	1.41E-09	2.70E-08	+	0.207766	0.205	+	A	T	0.474511315	0.0341191	+	A	T	0.493	0.0369
rs3783709	LC_IGP11	IgG1_G0	14	66058664	1	3.10E-19	2.98E-14	-	-0.304975	-0.2856	+	A	T	0.474831704	0.0335955	+	A	T	0.493	0.0376
rs3783709	LC_IGP_R1	IgG1_G0F/IgG1_G0	14	66058664	1	2.09E-16	6.61E-12	+	0.279934	0.2591	+	A	T	0.474831704	0.0337128	+	A	T	0.493	0.0377
rs3783709	LC_IGP_R4	IgG1_G0FN/IgG1_G0N	14	66058664	1	9.27E-15	3.55E-13	+	0.265687	0.2759	+	A	T	0.475381813	0.0339664	+	A	T	0.493	0.0379
rs3783709	LC_IGP52	IgG1_G0n	14	66058664	1	2.85E-19	2.59E-14	-	-0.307648	-0.2869	+	A	T	0.475108426	0.0338478	+	A	T	0.493	0.0377
rs3783709	LC_IGP14	IgG1_G0N	14	66058664	1	8.43E-17	4.28E-11	-	-0.284966	-0.2468	+	A	T	0.475381813	0.0338616	+	A	T	0.493	0.0374
rs3783709	LC_IGP55	IgG1_G0Nn	14	66058664	1	3.86E-17	4.41E-11	-	-0.289777	-0.2469	+	A	T	0.475108426	0.0340445	+	A	T	0.493	0.0375
rs3783709	LC_IGP12	IgG1_G1	14	66058664	1	3.39E-16	1.23E-13	-	-0.278617	-0.2804	+	A	T	0.474831704	0.0337975	+	A	T	0.493	0.0378
rs3783709	LC_IGP_R2	IgG1_G1F/IgG1_G1	14	66058664	1	2.29E-17	1.42E-13	+	0.289416	0.2791	+	A	T	0.474831704	0.0337564	+	A	T	0.493	0.0377
rs3783709	LC_IGP_R5	IgG1_G1FN/IgG1_G1N	14	66058664	1	1.76E-14	2.24E-17	+	0.263959	0.3204	+	A	T	0.476215252	0.0341129	+	A	T	0.493	0.0378
rs3783709	LC_IGP53	IgG1_G1n	14	66058664	1	2.73E-15	2.12E-13	-	-0.27245	-0.2775	+	A	T	0.475108426	0.0341366	+	A	T	0.493	0.0378
rs3783709	LC_IGP15	IgG1_G1N	14	66058664	1	2.97E-11	1.22E-10	-	-0.229287	-0.242	+	A	T	0.476215252	0.0342486	+	A	T	0.493	0.0376
rs3783709	LC_IGP56	IgG1_G1Nn	14	66058664	1	1.22E-11	2.45E-10	-	-0.234605	-0.2377	+	A	T	0.475108426	0.0343648	+	A	T	0.493	0.0376
rs3783709	LC_IGP_R36	IgG1_G1NS1/IgG1_G1N	14	66058664	1	2.88E-08	4.32E-12	+	0.190575	0.2548	+	A	T	0.476156597	0.0341767	+	A	T	0.493	0.0368
rs3783709	LC_IGP_R32	IgG1_G1S1/IgG1_G1	14	66058664	1	3.11E-10	4.26E-10	+	0.215642	0.2266	+	A	T	0.474511315	0.034052	+	A	T	0.493	0.0363
rs3783709	LC_IGP_R3	IgG1_G2F/IgG1_G2	14	66058664	1	1.52E-15	9.79E-15	+	0.272859	0.2906	+	A	T	0.474526895	0.0338746	+	A	T	0.493	0.0375
rs3783709	LC_IGP_R8	IgG1_G2FS1/IgG1_G2S1	14	66058664	1	4.35E-10	1.26E-12	+	0.214259	0.2659	+	A	T	0.474237019	0.0341226	+	A	T	0.493	0.0374
rs3783709	LC_IGP_R26	IgG1_G2N/IgG1_G1N	14	66058664	1	3.99E-12	1.32E-08	+	0.238587	0.2083	+	A	T	0.475818519	0.0341293	+	A	T	0.493	0.0366
rs3783709	LC_IGP148	IgG2 FG0n total/G0n	14	66058664	1	4.54E-09	1.37E-13	+	0.201941	0.2772	+	A	T	0.473415025	0.0342534	+	A	T	0.493	0.0375
rs3783709	LC_IGP97	IgG2_G0	14	66058664	1	8.92E-11	1.08E-13	-	-0.222	-0.2786	+	A	T	0.474526895	0.0340116	+	A	T	0.493	0.0375

rs3783709	LC_IGP_R41	IgG2_G0F/IgG2_G0	14	66058664	1	4.04E-08	6.09E-13	+	0.188212	0.2683	+	A	T	0.474526895	0.0341244	+	A	T	0.493	0.0373
rs3783709	LC_IGP138	IgG2_G0n	14	66058664	1	5.29E-11	7.60E-14	-	-0.225505	-0.2808	+	A	T	0.473415025	0.0341299	+	A	T	0.493	0.0376
rs3783709	LC_IGP_R64	IgG2_G2/IgG2_G1	14	66058664	1	5.81E-09	1.92E-12	+	0.200786	0.2584	+	A	T	0.473577723	0.0343028	+	A	T	0.493	0.0367
rs2411355	LC_IGP78	IgG1 BG0n/G0n	14	66060707	1	7.40E-14	9.09E-10	-	-0.257911	-0.2302	+	A	C	0.475125774	0.0341735	+	A	C	0.493	0.0376
rs2411355	LC_IGP79	IgG1 BG1n/G1n	14	66060707	1	4.72E-13	9.49E-12	-	-0.250104	-0.2559	+	A	C	0.475125774	0.0342916	+	A	C	0.493	0.0376
rs2411355	LC_IGP32	IgG1 BG1S1/(BG1+BG1S1)	14	66060707	1	3.00E-08	3.75E-12	+	0.190289	0.2554	+	A	C	0.476173859	0.0341706	+	A	C	0.493	0.0368
rs2411355	LC_IGP77	IgG1 Bn	14	66060707	1	5.00E-13	2.37E-11	-	-0.249882	-0.252	+	A	C	0.475125774	0.0342997	+	A	C	0.493	0.0377
rs2411355	LC_IGP86	IgG1 Bn/Fn total	14	66060707	1	7.33E-14	1.43E-11	-	-0.258549	-0.255	+	A	C	0.475125774	0.0342522	+	A	C	0.493	0.0377
rs2411355	LC_IGP84	IgG1 FBn/Bn total	14	66060707	1	2.21E-15	4.03E-16	+	0.274264	0.3091	+	A	C	0.475125774	0.0342484	+	A	C	0.493	0.038
rs2411355	LC_IGP62	IgG1 FG0n total/G0n	14	66060707	1	2.14E-17	2.94E-12	+	0.291495	0.2642	+	A	C	0.475125774	0.0339632	+	A	C	0.493	0.0378
rs2411355	LC_IGP63	IgG1 FG1n total/G1n	14	66060707	1	1.09E-17	1.79E-14	+	0.295002	0.2898	+	A	C	0.475125774	0.0340491	+	A	C	0.493	0.0378
rs2411355	LC_IGP64	IgG1 FG2n total/G2n	14	66060707	1	4.30E-14	3.17E-15	+	0.26104	0.2965	+	A	C	0.475125774	0.0342576	+	A	C	0.493	0.0376
rs2411355	LC_IGP68	IgG1 FG2n/G2n	14	66060707	1	1.81E-10	1.92E-08	+	0.220901	0.2107	+	A	C	0.475125774	0.0344208	+	A	C	0.493	0.0375
rs2411355	LC_IGP61	IgG1 Fn total	14	66060707	1	8.72E-17	4.21E-14	+	0.286605	0.286	+	A	C	0.475125774	0.0340686	+	A	C	0.493	0.0379
rs2411355	LC_IGP81	IgG1 Fn/Bn	14	66060707	1	2.83E-13	1.19E-10	+	0.252371	0.242	+	A	C	0.475125774	0.034269	+	A	C	0.493	0.0376
rs2411355	LC_IGP21	IgG1 Fucosylation	14	66060707	1	2.10E-15	5.53E-13	+	0.272752	0.2725	+	A	C	0.474323674	0.0340343	+	A	C	0.493	0.0378
rs2411355	LC_IGP28	IgG1 G1S1/(G1+G1S1)	14	66060707	1	1.62E-10	2.80E-10	+	0.218996	0.2287	+	A	C	0.47452844	0.0340349	+	A	C	0.493	0.0362
rs2411355	LC_IGP26	IgG1 GS1/(G+GS1)	14	66060707	1	2.89E-08	1.37E-09	+	0.190779	0.2249	+	A	C	0.47452844	0.0342216	+	A	C	0.493	0.0371
rs2411355	LC_IGP27	IgG1 GS1/(G0+G+GS1)	14	66060707	1	1.41E-09	2.70E-08	+	0.207769	0.205	+	A	C	0.47452844	0.0341202	+	A	C	0.493	0.0369
rs2411355	LC_IGP11	IgG1_G0	14	66060707	1	3.09E-19	2.98E-14	-	-0.304993	-0.2856	+	A	C	0.474848809	0.0335966	+	A	C	0.493	0.0376
rs2411355	LC_IGP_R1	IgG1_G0F/IgG1_G0	14	66060707	1	2.08E-16	6.61E-12	+	0.279965	0.2591	+	A	C	0.474848809	0.0337138	+	A	C	0.493	0.0377
rs2411355	LC_IGP_R4	IgG1_G0FN/IgG1_G0N	14	66060707	1	9.22E-15	3.55E-13	+	0.265717	0.2759	+	A	C	0.475398959	0.0339674	+	A	C	0.493	0.0379
rs2411355	LC_IGP52	IgG1_G0n	14	66060707	1	2.84E-19	2.60E-14	-	-0.307668	-0.2869	+	A	C	0.475125774	0.0338488	+	A	C	0.493	0.0377
rs2411355	LC_IGP14	IgG1_G0N	14	66060707	1	8.37E-17	4.29E-11	-	-0.285003	-0.2468	+	A	C	0.475398959	0.0338626	+	A	C	0.493	0.0374
rs2411355	LC_IGP55	IgG1_G0Nn	14	66060707	1	3.83E-17	4.42E-11	-	-0.289814	-0.2469	+	A	C	0.475125774	0.0340455	+	A	C	0.493	0.0375
rs2411355	LC_IGP12	IgG1_G1	14	66060707	1	3.38E-16	1.23E-13	-	-0.278632	-0.2804	+	A	C	0.474848809	0.0337986	+	A	C	0.493	0.0378
rs2411355	LC_IGP_R2	IgG1_G1F/IgG1_G1	14	66060707	1	2.28E-17	1.42E-13	+	0.28943	0.2791	+	A	C	0.474848809	0.0337575	+	A	C	0.493	0.0377
rs2411355	LC_IGP_R5	IgG1_G1FN/IgG1_G1N	14	66060707	1	1.76E-14	2.24E-17	+	0.263977	0.3204	+	A	C	0.476232472	0.034114	+	A	C	0.493	0.0378
rs2411355	LC_IGP53	IgG1_G1n	14	66060707	1	2.72E-15	2.12E-13	-	-0.272466	-0.2775	+	A	C	0.475125774	0.0341377	+	A	C	0.493	0.0378
rs2411355	LC_IGP15	IgG1_G1N	14	66060707	1	2.95E-11	1.22E-10	-	-0.229322	-0.242	+	A	C	0.476232472	0.0342496	+	A	C	0.493	0.0376
rs2411355	LC_IGP56	IgG1_G1Nn	14	66060707	1	1.22E-11	2.45E-10	-	-0.234642	-0.2377	+	A	C	0.475125774	0.0343658	+	A	C	0.493	0.0376
rs2411355	LC_IGP_R36	IgG1_G1NS1/IgG1_G1N	14	66060707	1	2.86E-08	4.32E-12	+	0.190624	0.2548	+	A	C	0.476173859	0.0341777	+	A	C	0.493	0.0368
rs2411355	LC_IGP_R32	IgG1_G1S1/IgG1_G1	14	66060707	1	3.11E-10	4.26E-10	+	0.215641	0.2266	+	A	C	0.47452844	0.0340532	+	A	C	0.493	0.0363
rs2411355	LC_IGP_R3	IgG1_G2F/IgG1_G2	14	66060707	1	1.52E-15	9.80E-15	+	0.272874	0.2906	+	A	C	0.47454401	0.0338757	+	A	C	0.493	0.0375
rs2411355	LC_IGP_R8	IgG1_G2FS1/IgG1_G2S1	14	66060707	1	4.36E-10	1.26E-12	+	0.214255	0.2658	+	A	C	0.474254123	0.0341237	+	A	C	0.493	0.0374
rs2411355	LC_IGP_R26	IgG1_G2N/IgG1_G1N	14	66060707	1	3.98E-12	1.32E-08	+	0.238605	0.2083	+	A	C	0.475835802	0.0341304	+	A	C	0.493	0.0366
rs2411355	LC_IGP148	IgG2 FG0n total/G0n	14	66060707	1	4.49E-09	1.37E-13	+	0.201999	0.2772	+	A	C	0.473432266	0.0342543	+	A	C	0.493	0.0375
rs2411355	LC_IGP97	IgG2_G0	14	66060707	1	8.86E-11	1.09E-13	-	-0.222039	-0.2786	+	A	C	0.47454401	0.0340126	+	A	C	0.493	0.0375
rs2411355	LC_IGP_R41	IgG2_G0F/IgG2_G0	14	66060707	1	4.01E-08	6.09E-13	+	0.188262	0.2683	+	A	C	0.47454401	0.0341254	+	A	C	0.493	0.0373
rs2411355	LC_IGP138	IgG2_G0n	14	66060707	1	5.26E-11	7.60E-14	-	-0.225547	-0.2808	+	A	C	0.473432266	0.0341309	+	A	C	0.493	0.0376
rs2411355	LC_IGP_R64	IgG2_G2/IgG2_G1	14	66060707	1	5.80E-09	1.92E-12	+	0.200803	0.2584	+	A	C	0.473594859	0.0343039	+	A	C	0.493	0.0367
rs12889002	LC_IGP78	IgG1 BG0n/G0n	14	66063582	1	4.92E-14	7.99E-10	-	-0.259601	-0.2308	+	G	C	0.477738848	0.0341494	+	G	C	0.4936	0.0376
rs12889002	LC_IGP79	IgG1 BG1n/G1n	14	66063582	1	2.87E-13	8.28E-12	-	-0.252277	-0.2564	+	G	C	0.477738848	0.0342655	+	G	C	0.4936	0.0375
rs12889002	LC_IGP32	IgG1 BG1S1/(BG1+BG1S1)	14	66063582	1	2.15E-08	3.15E-12	+	0.192169	0.256	+	G	C	0.478774044	0.034149	+	G	C	0.4936	0.0367
rs12889002	LC_IGP77	IgG1 Bn	14	66063582	1	3.59E-13	2.07E-11	-	-0.251298	-0.2526	+	G	C	0.477738848	0.0342771	+	G	C	0.4936	0.0377
rs12889002	LC_IGP86	IgG1 Bn/Fn total	14	66063582	1	5.25E-14	1.24E-11	-	-0.259913	-0.2555	+	G	C	0.477738848	0.0342295	+	G	C	0.4936	0.0377
rs12889002	LC_IGP84	IgG1 FBn/Bn total	14	66063582	1	2.65E-15	3.35E-16	+	0.273374	0.31	+	G	C	0.477738848	0.0342365	+	G	C	0.4936	0.038
rs12889002	LC_IGP62	IgG1 FG0n total/G0n	14	66063582	1	1.87E-17	2.51E-12	+	0.291888	0.2648	+	G	C	0.477738848	0.0339448	+	G	C	0.4936	0.0378
rs12889002	LC_IGP63	IgG1 FG1n total/G1n	14	66063582	1	1.02E-17	1.54E-14	+	0.295131	0.2903	+	G	C	0.477738848	0.0340321	+	G	C	0.4936	0.0378
rs12889002	LC_IGP64	IgG1 FG2n total/G2n	14	66063582	1	5.07E-14	2.75E-15	+	0.260192	0.2969	+	G	C	0.477738848	0.0342454	+	G	C	0.4936	0.0376
rs12889002	LC_IGP68	IgG1 FG2n/G2n	14	66063582	1	1.60E-10	1.81E-08	+	0.22147	0.2108	+	G	C	0.477738848	0.0344024	+	G	C	0.4936	0.0374
rs12889002	LC_IGP61	IgG1 Fn total	14	66063582	1	8.34E-17	3.61E-14	+	0.286648	0.2865	+	G	C	0.477738848	0.0340521	+	G	C	0.4936	0.0378
rs12889002	LC_IGP81	IgG1 Fn/Bn	14	66063582	1	1.85E-13	1.07E-10	+	0.254184	0.2424	+	G	C	0.477738848	0.0342444	+	G	C	0.4936	0.0375
rs12889002	LC_IGP21	IgG1 Fucosylation	14	66063582	1	3.06E-15	4.74E-13	+	0.271059	0.273	+	G	C	0.476923859	0.034026	+	G	C	0.4936	0.0378
rs12889002	LC_IGP28	IgG1 G1S1/(G1+G1S1)	14	66063582	1	2.76E-10	2.48E-10	+	0.216142	0.2292	+	G	C	0.477107951	0.0340299	+	G	C	0.4936	0.0362
rs12889002	LC_IGP26	IgG1 GS1/(G+GS1)	14	66063582	1	4.67E-08	1.22E-09	+	0.187822	0.2255	+	G	C	0.477107951	0.0342155	+	G	C	0.4936	0.0371
rs12889002	LC_IGP27	IgG1 GS1/(G0+G+GS1)	14	66063582	1	2.26E-09	2.41E-08	+	0.205087	0.2055	+	G	C	0.477107951	0.034114	+	G	C	0.4936	0.0368
rs12889002	LC_IGP11	IgG1_G0	14	66063582	1	3.34E-19	2.56E-14	-	-0.304557	-0.2862	+	G	C	0.477441356	0.0335807	+	G	C	0.4936	0.0376
rs12889002	LC_IGP_R1	IgG1_G0F/IgG1_G0	14	66063582	1	1.76E-16	5.69E-12	+	0.280482	0.2597	+	G	C	0.477441356	0.033693	+	G	C	0.4936	0.0377
rs12889002	LC_IGP_R4	IgG1_G0FN/IgG1_G0N	14	66063582	1	9.20E-15	2.99E-13	+	0.265592	0.2767	+	G	C	0.477997857	0.03395	+	G	C	0.4936	0.0379
rs12889002	LC_IGP52	IgG1_G0n	14	66063582	1	3.04E-19	2.23E-14	-	-0.307277	-0.2875	+	G	C	0.477738848	0.0338348	+	G	C	0.4936	0.0377
rs12889002	LC_IGP14	IgG1_G0N	14	66063582	1	6.13E-17	3.80E-11	-	-0.286093	-0.2473	+	G	C	0.477997857	0.0338389	+	G	C	0.4936	0.0374
rs12889002	LC_IGP55	IgG1_G0Nn	14	66063582	1	2.82E-17	3.91E-11	-	-0.290894	-0.2474	+	G	C	0.477738848	0.0340235	+	G	C	0.4936	0.0374
rs12889002	LC_IGP12	IgG1_G1	14	66063582	1	3.91E-16	1.05E-13	-	-0.277897	-0.2809	+	G	C	0.477441356	0.033784	+	G	C	0.4936	0.0378
rs12889002	LC_IGP_R2	IgG1_G1F/IgG1_G1	14	66063582	1	2.30E-17	1.24E-13	+	0.289255	0.2794	+	G	C	0.477441356	0.0337401	+	G	C	0.4936	0.0377
rs12889002	LC_IGP_R5	IgG1_G1FN/IgG1_G1N	14	66063582	1	1.56E-14	1.86E-17	+	0.26438	0.3212	+	G	C	0.478826261	0.0340969	+	G	C	0.4936	0.0378
rs12889002	LC_IGP53	IgG1_G1n	14	66063582	1	3.00E-15	1.81E-13	-	-0.271928	-0.278	+	G	C	0.477738848	0.0341241	+	G	C	0.4936	0.0378
rs12889002	LC_IGP15	IgG1_G1N	14	66063582	1	1.81E-11	1.06E-10	-	-0.231653	-0.2426	+	G	C	0.478826261	0.0342248	+	G	C	0.4936	0.0376
rs12889002	LC_IGP56	IgG1_G1Nn	14	66063582	1	7.59E-12	2.14E-10	-	-0.236853	-0.2383	+	G	C	0.477738848	0.0343402	+	G	C	0.4936	0.0375
rs12889002	LC_IGP_R36	IgG1_G1NS1/IgG1_G1N	14	66063582	1	1.92E-08	3.63E-12	+	0.192873	0.2555	+	G	C	0.478774044	0.0341548	+	G	C	0.4936	0.0368
rs12889002	LC_IGP_R32	IgG1_G1S1/IgG1_G1	14	66063582	1	4.97E-10	3.77E-10	+	0.213071	0.2271	+	G	C	0.477107951	0.034047	+	G	C	0.4936	0.0363
rs12889002	LC_IGP_R3	IgG1_G2F/IgG1_G2	14	66063582	1	1.79E-15	8.50E-15	+	0.272064	0.291	+	G	C	0.477121944	0.0338634	+	G	C	0.4936	0.0375
rs12889002	LC_IGP_R8	IgG1_G2FS1/IgG1_G2S1	14	66063582	1	5.38E-10	1.11E-12	+	0.213045	0.2662	+	G	C	0.476830483	0.034112	+	G	C	0.4936	0.0374
rs12889002	LC_IGP_R26	IgG1_G2N/IgG1_G1N	14	66063582	1	3.37E-12	1.16E-08	+	0.239292	0.209	+	G	C	0.478439198	0.034112	+	G	C	0.4936	0.0366
rs12889002	LC_IGP148	IgG2 FG0n total/G0n	14	66063582	1	6.05E-09	1.26E-13	+	0.200217	0.2772	+	G	C	0.476029249	0.0342437	+	G	C	0.4936	0.0374
rs12889002	LC_IGP97	IgG2_G0	14	66063582	1	1.57E-10	9.99E-14	-	-0.219	-0.2787	+	G	C	0.477121944	0.0340086	+	G	C	0.4936	0.0375
rs12889002	LC_IGP138	IgG2_G0n	14	66063582	1	9.33E-11	6.97E-14	-	-0.222521	-0.2809	+	G	C	0.476029249	0.034126	+	G	C	0.4936	0.0375
rs12889002	LC_IGP_R64	IgG2_G2/IgG2_G1	14	66063582	1	7.37E-09	1.93E-12	+	0.199339	0.2581	+	G	C	0.476175949	0.0342922	+	G	C	0.4936	0.0367
rs113131712	LC_IGP78	IgG1 BG0n/G0n	14	66064120	1	8.01E-14	8.26E-10	-	-0.257755	-0.2332	+	A	G	0.475902726	0.0342011	+	A	G	0.4895	0.038
rs113131712	LC_IGP79	IgG1 BG1n/G1n	14	66064120	1	3.15E-13	1.22E-11	-	-0.252158	-0.2573	+	A	G	0.475902726	0.0343092	+	A	G	0.4895	0.038
rs113131712	LC_IGP32	IgG1 BG1S1/(BG1+BG1S1)	14	66064120	1	2.47E-08	4.08E-12	+	0.191591	0.2574	+	A	G	0.476946979	0.0341926	+	A	G	0.4895	0.0371
rs113131712	LC_IGP77	IgG1 Bn	14	66064120	1	4.15E-13	2.56E-11	-	-0.250939	-0.2544	+	A	G	0.475902726	0.0343219	+	A	G	0.4895	0.0381
rs113131712	LC_IGP86	IgG1 Bn/Fn total	14	66064120	1	6.20E-14	1.52E-11	-	-0.259496	-0.2574	+	A	G	0.475902726	0.0342747	+	A	G	0.4895	0.0382
rs113131712	LC_IGP84	IgG1 FBn/Bn total	14	66064120	1	2.48E-15	3.65E-16	+	0.273996	0.3129	+	A	G	0.475902726	0.0342767	+	A	G	0.4895	0.0384
rs113131712	LC_IGP62	IgG1 FG0n total/G0n	14	66064120	1	2.93E-17	2.22E-12	+	0.290492	0.2683	+	A	G	0.475902726	0.0339955	+	A	G	0.4895	0.0382
rs113131712	LC_IGP63	IgG1 FG1n total/G1n	14	66064120	1	1.29E-17	2.08E-14	+	0.294552	0.2919	+	A	G	0.475902726	0.0340786	+	A	G	0.4895	0.0382
rs113131712	LC_IGP64	IgG1 FG2n total/G2n	14	66064120	1	4.33E-14	3.77E-15	+	0.261217	0.2985	+	A	G	0.475902726	0.0342837	+	A	G	0.4895	0.038
rs113131712	LC_IGP68	IgG1 FG2n/G2n	14	66064120	1	2.06E-10	2.55E-08	+	0.220414	0.211	+	A	G	0.475902726	0.0344496	+	A	G	0.4895	0.0379
rs113131712	LC_IGP61	IgG1 Fn total	14	66064120	1	1.04E-16	4.24E-14	+	0.286117	0.2888	+	A	G	0.475902726	0.0340982	+	A	G	0.4895	0.0382
rs113131712	LC_IGP81	IgG1 Fn/Bn	14	66064120	1	2.21E-13	1.33E-10	+	0.253682	0.2439	+	A	G	0.475902726	0.0342899	+	A	G	0.4895	0.038
rs113131712	LC_IGP21	IgG1 Fucosylation	14	66064120	1	3.57E-15	5.24E-13	+	0.270738	0.2754	+	A	G	0.475096794	0.0340708	+	A	G	0.4895	0.0382
rs113131712	LC_IGP28	IgG1 G1S1/(G1+G1S1)	14	66064120	1	4.19E-10	3.43E-10	+	0.214193	0.2296	+	A	G	0.475295413	0.0340795	+	A	G	0.4895	0.0366
rs113131712	LC_IGP27	IgG1 GS1/(G0+G+GS1)	14	66064120	1	2.89E-09	2.26E-08	+	0.20397	0.2081	+	A	G	0.475295413	0.0341602	+	A	G	0.4895	0.0372
rs113131712	LC_IGP11	IgG1_G0	14	66064120	1	4.22E-19	2.27E-14	-	-0.304059	-0.29	+	A	G	0.475631032	0.0336258	+	A	G	0.4895	0.038
rs113131712	LC_IGP_R1	IgG1_G0F/IgG1_G0	14	66064120	1	2.69E-16	5.10E-12	+	0.279131	0.2631	+	A	G	0.475631032	0.033742	+	A	G	0.4895	0.0381
rs113131712	LC_IGP_R4	IgG1_G0FN/IgG1_G0N	14	66064120	1	1.18E-14	2.69E-13	+	0.264843	0.2803	+	A	G	0.476183099	0.0339958	+	A	G	0.4895	0.0384
rs113131712	LC_IGP52	IgG1_G0n	14	66064120	1	3.84E-19	2.00E-14	-	-0.30679	-0.2912	+	A	G	0.475902726	0.0338808	+	A	G	0.4895	0.0381
rs113131712	LC_IGP14	IgG1_G0N	14	66064120	1	9.73E-17	4.05E-11	-	-0.284601	-0.2498	+	A	G	0.476183099	0.033889	+	A	G	0.4895	0.0378
rs113131712	LC_IGP55	IgG1_G0Nn	14	66064120	1	4.50E-17	4.17E-11	-	-0.289405	-0.2499	+	A	G	0.475902726	0.0340747	+	A	G	0.4895	0.0379
rs113131712	LC_IGP12	IgG1_G1	14	66064120	1	4.83E-16	1.16E-13	-	-0.277373	-0.2833	+	A	G	0.475631032	0.0338289	+	A	G	0.4895	0.0382
rs113131712	LC_IGP_R2	IgG1_G1F/IgG1_G1	14	66064120	1	3.07E-17	1.64E-13	+	0.288478	0.281	+	A	G	0.475631032	0.0337865	+	A	G	0.4895	0.0381
rs113131712	LC_IGP_R5	IgG1_G1FN/IgG1_G1N	14	66064120	1	1.61E-14	2.57E-17	+	0.264559	0.3232	+	A	G	0.47700369	0.0341381	+	A	G	0.4895	0.0382
rs113131712	LC_IGP53	IgG1_G1n	14	66064120	1	3.71E-15	2.02E-13	-	-0.271362	-0.2802	+	A	G	0.475902726	0.0341701	+	A	G	0.4895	0.0381
rs113131712	LC_IGP15	IgG1_G1N	14	66064120	1	2.01E-11	1.36E-10	-	-0.231423	-0.244	+	A	G	0.47700369	0.0342677	+	A	G	0.4895	0.038
rs113131712	LC_IGP56	IgG1_G1Nn	14	66064120	1	8.43E-12	2.75E-10	-	-0.236626	-0.2395	+	A	G	0.475902726	0.0343842	+	A	G	0.4895	0.0379
rs113131712	LC_IGP_R36	IgG1_G1NS1/IgG1_G1N	14	66064120	1	2.15E-08	4.69E-12	+	0.192463	0.257	+	A	G	0.476946979	0.0341978	+	A	G	0.4895	0.0372
rs113131712	LC_IGP_R32	IgG1_G1S1/IgG1_G1	14	66064120	1	7.20E-10	5.14E-10	+	0.211343	0.2276	+	A	G	0.475295413	0.0340957	+	A	G	0.4895	0.0366
rs113131712	LC_IGP_R3	IgG1_G2F/IgG1_G2	14	66064120	1	1.78E-15	1.19E-14	+	0.27241	0.2925	+	A	G	0.475310513	0.0339042	+	A	G	0.4895	0.0379
rs113131712	LC_IGP_R8	IgG1_G2FS1/IgG1_G2S1	14	66064120	1	6.03E-10	1.38E-12	+	0.212687	0.2678	+	A	G	0.475020159	0.0341557	+	A	G	0.4895	0.0378
rs113131712	LC_IGP_R26	IgG1_G2N/IgG1_G1N	14	66064120	1	6.64E-12	1.21E-08	+	0.236321	0.2109	+	A	G	0.476609877	0.034167	+	A	G	0.4895	0.037
rs113131712	LC_IGP148	IgG2 FG0n total/G0n	14	66064120	1	7.60E-09	2.37E-13	+	0.199157	0.2769	+	A	G	0.474204433	0.0342906	+	A	G	0.4895	0.0378
rs113131712	LC_IGP97	IgG2_G0	14	66064120	1	2.00E-10	2.01E-13	-	-0.218009	-0.278	+	A	G	0.475310513	0.0340546	+	A	G	0.4895	0.0378
rs113131712	LC_IGP138	IgG2_G0n	14	66064120	1	1.18E-10	1.47E-13	-	-0.221595	-0.28	+	A	G	0.474204433	0.0341729	+	A	G	0.4895	0.0379
rs113131712	LC_IGP_R64	IgG2_G2/IgG2_G1	14	66064120	1	4.27E-09	2.88E-12	+	0.202693	0.2584	+	A	G	0.474362301	0.034323	+	A	G	0.4895	0.037
rs12895401	LC_IGP84	IgG1 FBn/Bn total	14	66064827	1	2.46E-08	1.59E-07	+	0.19763	0.2026	+	A	G	0.569031289	0.035268	+	A	G	0.5907	0.0387
rs12895401	LC_IGP63	IgG1 FG1n total/G1n	14	66064827	1	6.18E-10	1.10E-07	+	0.218444	0.2041	+	A	G	0.569031289	0.0350988	+	A	G	0.5907	0.0384
rs12895401	LC_IGP64	IgG1 FG2n total/G2n	14	66064827	1	4.66E-08	3.42E-08	+	0.1934	0.211	+	A	G	0.569031289	0.0352271	+	A	G	0.5907	0.0382
rs12895401	LC_IGP61	IgG1 Fn total	14	66064827	1	1.83E-09	2.90E-07	+	0.212244	0.1974	+	A	G	0.569031289	0.0350972	+	A	G	0.5907	0.0385
rs12895401	LC_IGP21	IgG1 Fucosylation	14	66064827	1	9.06E-09	2.17E-06	+	0.202259	0.182	+	A	G	0.568382552	0.0350065	+	A	G	0.5907	0.0384

rs12895401	LC_IGP11	IgG1_G0	14	66064827	1	3.64E-11	7.52E-07	-	-0.23076	-0.1894	+	A	G	0.568672877	0.0346287 +	A	G	0.5907	0.0383
rs12895401	LC_IGP52	IgG1_G0n	14	66064827	1	6.34E-11	8.22E-07	-	-0.229788	-0.1891	+	A	G	0.569031289	0.0349221 +	A	G	0.5907	0.0384
rs12895401	LC_IGP12	IgG1_G1	14	66064827	1	2.37E-09	2.35E-07	-	-0.208705	-0.1986	+	A	G	0.568978314	0.0347612 +	A	G	0.5907	0.0384
rs12895401	LC_IGP_R2	IgG1_G1F/IgG1_G1	14	66064827	1	6.42E-10	2.18E-07	+	0.216031	0.1988	+	A	G	0.568978314	0.0347484 +	A	G	0.5907	0.0384
rs12895401	LC_IGP_R5	IgG1_G1FN/IgG1_G1N	14	66064827	1	3.03E-08	2.19E-08	+	0.195317	0.2152	+	A	G	0.56975246	0.0350855 +	A	G	0.5907	0.0384
rs12895401	LC_IGP53	IgG1_G1n	14	66064827	1	5.78E-09	3.25E-07	-	-0.205613	-0.1962	+	A	G	0.569031289	0.0351188 +	A	G	0.5907	0.0384
rs12895401	LC_IGP_R3	IgG1_G2F/IgG1_G2	14	66064827	1	5.56E-09	6.20E-08	+	0.20418	0.2065	+	A	G	0.56840923	0.0348378 +	A	G	0.5907	0.0382
rs72716407	LC_IGP61	IgG1 Fn total	14	66065231	1	4.05E-08	1.56E-06	-	-0.238956	-0.2454	+	T	G	0.20083036	0.0433264 +	T	G	0.1658	0.0511
rs8005710	LC_IGP78	IgG1 BG0n/G0n	14	66065666	1	4.91E-14	6.39E-10	-	-0.259612	-0.2335	+	A	G	0.477736989	0.0341494 +	A	G	0.4893	0.0378
rs8005710	LC_IGP79	IgG1 BG1n/G1n	14	66065666	1	2.86E-13	7.10E-12	-	-0.252284	-0.2585	+	A	G	0.477736989	0.0342656 +	A	G	0.4893	0.0377
rs8005710	LC_IGP32	IgG1 BG1S1/(BG1+BG1S1)	14	66065666	1	2.15E-08	1.82E-12	+	0.192177	0.2606	+	A	G	0.478772195	0.0341491 +	A	G	0.4893	0.037
rs8005710	LC_IGP77	IgG1 Bn	14	66065666	1	3.58E-13	1.74E-11	-	-0.251306	-0.2549	+	A	G	0.477736989	0.0342772 +	A	G	0.4893	0.0379
rs8005710	LC_IGP86	IgG1 Bn/Fn total	14	66065666	1	5.24E-14	1.07E-11	-	-0.259922	-0.2577	+	A	G	0.477736989	0.0342296 +	A	G	0.4893	0.0379
rs8005710	LC_IGP84	IgG1 FBn/Bn total	14	66065666	1	2.65E-15	2.78E-16	+	0.273383	0.3128	+	A	G	0.477736989	0.0342366 +	A	G	0.4893	0.0382
rs8005710	LC_IGP62	IgG1 FG0n total/G0n	14	66065666	1	1.87E-17	2.35E-12	+	0.291901	0.2668	+	A	G	0.477736989	0.0339449 +	A	G	0.4893	0.038
rs8005710	LC_IGP63	IgG1 FG1n total/G1n	14	66065666	1	1.01E-17	1.55E-14	+	0.295141	0.292	+	A	G	0.477736989	0.0340322 +	A	G	0.4893	0.038
rs8005710	LC_IGP64	IgG1 FG2n total/G2n	14	66065666	1	5.07E-14	2.96E-15	+	0.260199	0.2982	+	A	G	0.477736989	0.0342454 +	A	G	0.4893	0.0378
rs8005710	LC_IGP68	IgG1 FG2n/G2n	14	66065666	1	1.60E-10	2.20E-08	+	0.221475	0.2104	+	A	G	0.477736989	0.0344025 +	A	G	0.4893	0.0376
rs8005710	LC_IGP61	IgG1 Fn total	14	66065666	1	8.32E-17	3.65E-14	+	0.28666	0.2881	+	A	G	0.477736989	0.0340522 +	A	G	0.4893	0.038
rs8005710	LC_IGP81	IgG1 Fn/Bn	14	66065666	1	1.85E-13	8.97E-11	+	0.254193	0.2446	+	A	G	0.477736989	0.0342445 +	A	G	0.4893	0.0377
rs8005710	LC_IGP21	IgG1 Fucosylation	14	66065666	1	3.05E-15	5.94E-13	+	0.271069	0.2734	+	A	G	0.47692201	0.0340261 +	A	G	0.4893	0.038
rs8005710	LC_IGP28	IgG1 G1S1/(G1+G1S1)	14	66065666	1	2.75E-10	2.13E-10	+	0.216147	0.2314	+	A	G	0.477106116	0.03403 +	A	G	0.4893	0.0364
rs8005710	LC_IGP26	IgG1 GS1/(G+GS1)	14	66065666	1	4.67E-08	8.67E-10	+	0.18782	0.229	+	A	G	0.477106116	0.0342156 +	A	G	0.4893	0.0373
rs8005710	LC_IGP27	IgG1 GS1/(G0+G+GS1)	14	66065666	1	2.26E-09	1.60E-08	+	0.205085	0.2096	+	A	G	0.477106116	0.0341141 +	A	G	0.4893	0.0371
rs8005710	LC_IGP11	IgG1_G0	14	66065666	1	3.33E-19	2.23E-14	-	-0.304564	-0.2889	+	A	G	0.477439524	0.0335807 +	A	G	0.4893	0.0378
rs8005710	LC_IGP_R1	IgG1_G0F/IgG1_G0	14	66065666	1	1.76E-16	5.68E-12	+	0.280494	0.2613	+	A	G	0.477439524	0.033693 +	A	G	0.4893	0.0379
rs8005710	LC_IGP_R4	IgG1_G0FN/IgG1_G0N	14	66065666	1	9.17E-15	2.22E-13	+	0.265602	0.2801	+	A	G	0.47799602	0.0339501 +	A	G	0.4893	0.0382
rs8005710	LC_IGP52	IgG1_G0n	14	66065666	1	3.03E-19	1.99E-14	-	-0.307284	-0.29	+	A	G	0.477736989	0.0338348 +	A	G	0.4893	0.0379
rs8005710	LC_IGP14	IgG1_G0N	14	66065666	1	6.12E-17	2.58E-11	-	-0.286099	-0.251	+	A	G	0.47799602	0.033839 +	A	G	0.4893	0.0376
rs8005710	LC_IGP55	IgG1_G0Nn	14	66065666	1	2.81E-17	2.69E-11	-	-0.290901	-0.2511	+	A	G	0.477736989	0.0340236 +	A	G	0.4893	0.0377
rs8005710	LC_IGP12	IgG1_G1	14	66065666	1	3.90E-16	1.10E-13	-	-0.277908	-0.2824	+	A	G	0.477439524	0.033784 +	A	G	0.4893	0.038
rs8005710	LC_IGP_R2	IgG1_G1F/IgG1_G1	14	66065666	1	2.29E-17	1.27E-13	+	0.289265	0.2809	+	A	G	0.477439524	0.0337401 +	A	G	0.4893	0.0379
rs8005710	LC_IGP_R5	IgG1_G1FN/IgG1_G1N	14	66065666	1	1.56E-14	1.61E-17	+	0.264387	0.3238	+	A	G	0.478824416	0.0340969 +	A	G	0.4893	0.038
rs8005710	LC_IGP53	IgG1_G1n	14	66065666	1	3.00E-15	1.90E-13	-	-0.27194	-0.2794	+	A	G	0.477736989	0.0341242 +	A	G	0.4893	0.038
rs8005710	LC_IGP15	IgG1_G1N	14	66065666	1	1.81E-11	9.09E-11	-	-0.231661	-0.2447	+	A	G	0.478824416	0.0342249 +	A	G	0.4893	0.0378
rs8005710	LC_IGP56	IgG1_G1Nn	14	66065666	1	7.58E-12	1.88E-10	-	-0.236861	-0.2402	+	A	G	0.477736989	0.0343403 +	A	G	0.4893	0.0377
rs8005710	LC_IGP_R36	IgG1_G1NS1/IgG1_G1N	14	66065666	1	1.92E-08	2.10E-12	+	0.192881	0.26	+	A	G	0.478772195	0.0341548 +	A	G	0.4893	0.037
rs8005710	LC_IGP_R32	IgG1_G1S1/IgG1_G1	14	66065666	1	4.96E-10	3.32E-10	+	0.213076	0.2293	+	A	G	0.477106116	0.0340471 +	A	G	0.4893	0.0365
rs8005710	LC_IGP_R3	IgG1_G2F/IgG1_G2	14	66065666	1	1.78E-15	9.24E-15	+	0.272071	0.2922	+	A	G	0.47712011	0.0338635 +	A	G	0.4893	0.0377
rs8005710	LC_IGP_R8	IgG1_G2FS1/IgG1_G2S1	14	66065666	1	5.36E-10	1.54E-12	+	0.213056	0.2659	+	A	G	0.47682865	0.0341121 +	A	G	0.4893	0.0376
rs8005710	LC_IGP_R26	IgG1_G2N/IgG1_G1N	14	66065666	1	3.37E-12	6.75E-09	+	0.239298	0.2136	+	A	G	0.478437346	0.034112 +	A	G	0.4893	0.0369
rs8005710	LC_IGP148	IgG2 FG0n total/G0n	14	66065666	1	6.03E-09	1.66E-13	+	0.20023	0.2775	+	A	G	0.476027401	0.0342438 +	A	G	0.4893	0.0376
rs8005710	LC_IGP97	IgG2_G0	14	66065666	1	1.57E-10	1.23E-13	-	-0.219008	-0.2793	+	A	G	0.47712011	0.0340086 +	A	G	0.4893	0.0377
rs8005710	LC_IGP138	IgG2_G0n	14	66065666	1	9.31E-11	8.83E-14	-	-0.22253	-0.2813	+	A	G	0.476027401	0.0341261 +	A	G	0.4893	0.0377
rs8005710	LC_IGP_R64	IgG2_G2/IgG2_G1	14	66065666	1	7.37E-09	1.66E-12	+	0.199342	0.2602	+	A	G	0.476174113	0.0342923 +	A	G	0.4893	0.0368
rs72716408	LC_IGP61	IgG1 Fn total	14	66066001	1	4.05E-08	1.57E-06	-	-0.238959	-0.2454	+	G	A	0.20083036	0.0433264 +	G	A	0.1657	0.0511
rs76087165	LC_IGP61	IgG1 Fn total	14	66067006	1	4.05E-08	1.56E-06	-	-0.238962	-0.2454	+	C	T	0.200829989	0.0433265 +	C	T	0.1658	0.0511
rs2064695	LC_IGP84	IgG1 FBn/Bn total	14	66067088	1	2.52E-08	1.66E-07	+	0.197327	0.2024	+	G	T	0.569651177	0.0352367 +	G	T	0.591	0.0387
rs2064695	LC_IGP63	IgG1 FG1n total/G1n	14	66067088	1	5.49E-10	1.13E-07	+	0.218893	0.204	+	G	T	0.569651177	0.0350647 +	G	T	0.591	0.0384
rs2064695	LC_IGP64	IgG1 FG2n total/G2n	14	66067088	1	3.91E-08	3.48E-08	+	0.194315	0.211	+	G	T	0.569651177	0.0351916 +	G	T	0.591	0.0382
rs2064695	LC_IGP61	IgG1 Fn total	14	66067088	1	1.57E-09	2.98E-07	+	0.212897	0.1973	+	G	T	0.569651177	0.0350624 +	G	T	0.591	0.0385
rs2064695	LC_IGP21	IgG1 Fucosylation	14	66067088	1	7.95E-09	2.14E-06	+	0.202845	0.1822	+	G	T	0.568999383	0.0349724 +	G	T	0.591	0.0384
rs2064695	LC_IGP11	IgG1_G0	14	66067088	1	3.38E-11	7.26E-07	-	-0.230924	-0.1897	+	G	T	0.569284056	0.0345965 +	G	T	0.591	0.0383
rs2064695	LC_IGP52	IgG1_G0n	14	66067088	1	5.73E-11	7.98E-07	-	-0.230107	-0.1894	+	G	T	0.569651177	0.0348885 +	G	T	0.591	0.0384
rs2064695	LC_IGP12	IgG1_G1	14	66067088	1	2.10E-09	2.39E-07	-	-0.209185	-0.1986	+	G	T	0.569589493	0.034728 +	G	T	0.591	0.0384
rs2064695	LC_IGP_R2	IgG1_G1F/IgG1_G1	14	66067088	1	5.48E-10	2.24E-07	+	0.216698	0.1987	+	G	T	0.569589493	0.0347144 +	G	T	0.591	0.0384
rs2064695	LC_IGP_R5	IgG1_G1FN/IgG1_G1N	14	66067088	1	3.02E-08	2.28E-08	+	0.195167	0.215	+	G	T	0.570367774	0.0350542 +	G	T	0.591	0.0385
rs2064695	LC_IGP53	IgG1_G1n	14	66067088	1	4.94E-09	3.34E-07	-	-0.206335	-0.1961	+	G	T	0.569651177	0.0350839 +	G	T	0.591	0.0384
rs2064695	LC_IGP_R3	IgG1_G2F/IgG1_G2	14	66067088	1	4.48E-09	6.26E-08	+	0.205243	0.2066	+	G	T	0.569020782	0.0348024 +	G	T	0.591	0.0382
rs743085	LC_IGP78	IgG1 BG0n/G0n	14	66068133	1	4.90E-14	7.97E-10	-	-0.259623	-0.2308	+	G	A	0.47773544	0.0341494 +	G	A	0.4936	0.0376
rs743085	LC_IGP79	IgG1 BG1n/G1n	14	66068133	1	2.86E-13	8.31E-12	-	-0.25229	-0.2564	+	G	A	0.47773544	0.0342656 +	G	A	0.4936	0.0375
rs743085	LC_IGP32	IgG1 BG1S1/(BG1+BG1S1)	14	66068133	1	2.15E-08	3.16E-12	+	0.192181	0.256	+	G	A	0.478770654	0.0341491 +	G	A	0.4936	0.0367
rs743085	LC_IGP77	IgG1 Bn	14	66068133	1	3.58E-13	2.07E-11	-	-0.251313	-0.2526	+	G	A	0.47773544	0.0342772 +	G	A	0.4936	0.0377
rs743085	LC_IGP86	IgG1 Bn/Fn total	14	66068133	1	5.24E-14	1.24E-11	-	-0.25993	-0.2555	+	G	A	0.47773544	0.0342296 +	G	A	0.4936	0.0377
rs743085	LC_IGP84	IgG1 FBn/Bn total	14	66068133	1	2.64E-15	3.35E-16	+	0.27339	0.3099	+	G	A	0.47773544	0.0342366 +	G	A	0.4936	0.038
rs743085	LC_IGP62	IgG1 FG0n total/G0n	14	66068133	1	1.86E-17	2.50E-12	+	0.291914	0.2648	+	G	A	0.47773544	0.0339449 +	G	A	0.4936	0.0378
rs743085	LC_IGP63	IgG1 FG1n total/G1n	14	66068133	1	1.01E-17	1.55E-14	+	0.29515	0.2903	+	G	A	0.47773544	0.0340322 +	G	A	0.4936	0.0378
rs743085	LC_IGP64	IgG1 FG2n total/G2n	14	66068133	1	5.06E-14	2.76E-15	+	0.260205	0.2968	+	G	A	0.47773544	0.0342455 +	G	A	0.4936	0.0376
rs743085	LC_IGP68	IgG1 FG2n/G2n	14	66068133	1	1.59E-10	1.81E-08	+	0.221481	0.2107	+	G	A	0.47773544	0.0344025 +	G	A	0.4936	0.0374
rs743085	LC_IGP61	IgG1 Fn total	14	66068133	1	8.30E-17	3.61E-14	+	0.286671	0.2865	+	G	A	0.47773544	0.0340522 +	G	A	0.4936	0.0378
rs743085	LC_IGP81	IgG1 Fn/Bn	14	66068133	1	1.84E-13	1.07E-10	+	0.2542	0.2424	+	G	A	0.47773544	0.0342445 +	G	A	0.4936	0.0375
rs743085	LC_IGP21	IgG1 Fucosylation	14	66068133	1	3.04E-15	4.76E-13	+	0.27108	0.273	+	G	A	0.476920469	0.0340261 +	G	A	0.4936	0.0378
rs743085	LC_IGP28	IgG1 G1S1/(G1+G1S1)	14	66068133	1	2.75E-10	2.48E-10	+	0.216151	0.2292	+	G	A	0.477104587	0.03403 +	G	A	0.4936	0.0362
rs743085	LC_IGP26	IgG1 GS1/(G+GS1)	14	66068133	1	4.67E-08	1.20E-09	+	0.187817	0.2255	+	G	A	0.477104587	0.0342156 +	G	A	0.4936	0.0371
rs743085	LC_IGP27	IgG1 GS1/(G0+G+GS1)	14	66068133	1	2.26E-09	2.38E-08	+	0.205082	0.2056	+	G	A	0.477104587	0.0341142 +	G	A	0.4936	0.0368
rs743085	LC_IGP11	IgG1_G0	14	66068133	1	3.32E-19	2.54E-14	-	-0.304571	-0.2863	+	G	A	0.477437996	0.0335808 +	G	A	0.4936	0.0376
rs743085	LC_IGP_R1	IgG1_G0F/IgG1_G0	14	66068133	1	1.75E-16	5.68E-12	+	0.280507	0.2597	+	G	A	0.477437996	0.033693 +	G	A	0.4936	0.0377
rs743085	LC_IGP_R4	IgG1_G0FN/IgG1_G0N	14	66068133	1	9.16E-15	2.97E-13	+	0.265612	0.2767	+	G	A	0.477994489	0.0339501 +	G	A	0.4936	0.0379
rs743085	LC_IGP52	IgG1_G0n	14	66068133	1	3.03E-19	2.21E-14	-	-0.307292	-0.2875	+	G	A	0.47773544	0.0338349 +	G	A	0.4936	0.0377
rs743085	LC_IGP14	IgG1_G0N	14	66068133	1	6.11E-17	3.77E-11	-	-0.286105	-0.2473	+	G	A	0.477994489	0.033839 +	G	A	0.4936	0.0374
rs743085	LC_IGP55	IgG1_G0Nn	14	66068133	1	2.81E-17	3.88E-11	-	-0.290907	-0.2474	+	G	A	0.47773544	0.0340236 +	G	A	0.4936	0.0374
rs743085	LC_IGP12	IgG1_G1	14	66068133	1	3.89E-16	1.05E-13	-	-0.277919	-0.2809	+	G	A	0.477437996	0.033784 +	G	A	0.4936	0.0378
rs743085	LC_IGP_R2	IgG1_G1F/IgG1_G1	14	66068133	1	2.29E-17	1.25E-13	+	0.289274	0.2794	+	G	A	0.477437996	0.0337401 +	G	A	0.4936	0.0377
rs743085	LC_IGP_R5	IgG1_G1FN/IgG1_G1N	14	66068133	1	1.56E-14	1.87E-17	+	0.264393	0.3212	+	G	A	0.478822878	0.034097 +	G	A	0.4936	0.0378
rs743085	LC_IGP53	IgG1_G1n	14	66068133	1	2.99E-15	1.82E-13	-	-0.271952	-0.278	+	G	A	0.47773544	0.0341242 +	G	A	0.4936	0.0378
rs743085	LC_IGP15	IgG1_G1N	14	66068133	1	1.81E-11	1.06E-10	-	-0.231668	-0.2426	+	G	A	0.478822878	0.0342249 +	G	A	0.4936	0.0376
rs743085	LC_IGP56	IgG1_G1Nn	14	66068133	1	7.57E-12	2.14E-10	-	-0.236869	-0.2382	+	G	A	0.47773544	0.0343403 +	G	A	0.4936	0.0375
rs743085	LC_IGP_R36	IgG1_G1NS1/IgG1_G1N	14	66068133	1	1.92E-08	3.64E-12	+	0.192885	0.2555	+	G	A	0.478770654	0.0341549 +	G	A	0.4936	0.0368
rs743085	LC_IGP_R32	IgG1_G1S1/IgG1_G1	14	66068133	1	4.96E-10	3.76E-10	+	0.21308	0.2271	+	G	A	0.477104587	0.0340471 +	G	A	0.4936	0.0363
rs743085	LC_IGP_R3	IgG1_G2F/IgG1_G2	14	66068133	1	1.78E-15	8.55E-15	+	0.272077	0.291	+	G	A	0.477118582	0.0338635 +	G	A	0.4936	0.0375
rs743085	LC_IGP_R8	IgG1_G2FS1/IgG1_G2S1	14	66068133	1	5.35E-10	1.12E-12	+	0.213068	0.2661	+	G	A	0.476827123	0.0341121 +	G	A	0.4936	0.0374
rs743085	LC_IGP_R26	IgG1_G2N/IgG1_G1N	14	66068133	1	3.36E-12	1.16E-08	+	0.239305	0.209	+	G	A	0.478435802	0.0341121 +	G	A	0.4936	0.0366
rs743085	LC_IGP148	IgG2 FG0n total/G0n	14	66068133	1	6.02E-09	1.26E-13	+	0.200243	0.2772	+	G	A	0.476025862	0.0342438 +	G	A	0.4936	0.0374
rs743085	LC_IGP97	IgG2_G0	14	66068133	1	1.57E-10	9.91E-14	-	-0.219018	-0.2787	+	G	A	0.477118582	0.0340087 +	G	A	0.4936	0.0374
rs743085	LC_IGP138	IgG2_G0n	14	66068133	1	9.30E-11	6.93E-14	-	-0.22254	-0.2809	+	G	A	0.476025862	0.0341261 +	G	A	0.4936	0.0375
rs743085	LC_IGP_R64	IgG2_G2/IgG2_G1	14	66068133	1	7.36E-09	1.90E-12	+	0.199347	0.2582	+	G	A	0.476172583	0.0342924 +	G	A	0.4936	0.0367
rs11158602	LC_IGP78	IgG1 BG0n/G0n	14	66071733	1	1.39E-13	4.26E-10	-	-0.254273	-0.2379	+	G	T	0.488833643	0.0340755 +	G	T	0.5061	0.0381
rs11158602	LC_IGP79	IgG1 BG1n/G1n	14	66071733	1	6.29E-13	2.76E-12	-	-0.247961	-0.2663	+	G	T	0.488833643	0.0341862 +	G	T	0.5061	0.0381
rs11158602	LC_IGP32	IgG1 BG1S1/(BG1+BG1S1)	14	66071733	1	9.59E-09	3.63E-12	+	0.196348	0.2595	+	G	T	0.489814735	0.0340407 +	G	T	0.5061	0.0373
rs11158602	LC_IGP77	IgG1 Bn	14	66071733	1	7.19E-13	8.29E-12	-	-0.247393	-0.2615	+	G	T	0.488833643	0.0341958 +	G	T	0.5061	0.0383
rs11158602	LC_IGP86	IgG1 Bn/Fn total	14	66071733	1	9.09E-14	5.08E-12	-	-0.256751	-0.2643	+	G	T	0.488833643	0.0341453 +	G	T	0.5061	0.0383
rs11158602	LC_IGP84	IgG1 FBn/Bn total	14	66071733	1	5.45E-15	2.94E-16	+	0.269553	0.3156	+	G	T	0.488833643	0.0341559 +	G	T	0.5061	0.0386
rs11158602	LC_IGP62	IgG1 FG0n total/G0n	14	66071733	1	9.94E-18	1.20E-12	+	0.293526	0.2727	+	G	T	0.488833643	0.0338368 +	G	T	0.5061	0.0384
rs11158602	LC_IGP63	IgG1 FG1n total/G1n	14	66071733	1	1.44E-17	5.23E-15	+	0.292958	0.3002	+	G	T	0.488833643	0.0339443 +	G	T	0.5061	0.0384
rs11158602	LC_IGP64	IgG1 FG2n total/G2n	14	66071733	1	4.13E-14	1.02E-15	+	0.260377	0.3064	+	G	T	0.488833643	0.0341454 +	G	T	0.5061	0.0382
rs11158602	LC_IGP68	IgG1 FG2n/G2n	14	66071733	1	2.07E-10	2.87E-09	+	0.219482	0.2254	+	G	T	0.488833643	0.0343117 +	G	T	0.5061	0.038
rs11158602	LC_IGP61	IgG1 Fn total	14	66071733	1	4.10E-17	1.31E-14	+	0.288663	0.2959	+	G	T	0.488833643	0.0339422 +	G	T	0.5061	0.0384
rs11158602	LC_IGP81	IgG1 Fn/Bn	14	66071733	1	3.14E-13	3.03E-11	+	0.251064	0.2531	+	G	T	0.488833643	0.0341598 +	G	T	0.5061	0.0381
rs11158602	LC_IGP21	IgG1 Fucosylation	14	66071733	1	1.59E-15	1.36E-13	+	0.272991	0.2837	+	G	T	0.488580456	0.0339137 +	G	T	0.5061	0.0383
rs11158602	LC_IGP28	IgG1 G1S1/(G1+G1S1)	14	66071733	1	7.27E-11	8.91E-11	+	0.222372	0.2384	+	G	T	0.488672477	0.0339054 +	G	T	0.5061	0.0368
rs11158602	LC_IGP26	IgG1 GS1/(G+GS1)	14	66071733	1	4.53E-08	1.34E-09	+	0.187465	0.229	+	G	T	0.488672477	0.0341169 +	G	T	0.5061	0.0378
rs11158602	LC_IGP27	IgG1 GS1/(G0+G+GS1)	14	66071733	1	2.57E-09	3.10E-08	+	0.203784	0.2075	+	G	T	0.488672477	0.0340189 +	G	T	0.5061	0.0375
rs11158602	LC_IGP11	IgG1_G0	14	66071733	1	1.81E-19	1.59E-14	-	-0.305915	-0.2933	+	G	T	0.488991753	0.0334732 +	G	T	0.5061	0.0382
rs11158602	LC_IGP_R1	IgG1_G0F/IgG1_G0	14	66071733	1	4.56E-17	2.33E-12	+	0.285019	0.2683	+	G	T	0.488991753	0.0335702 +	G	T	0.5061	0.0382
rs11158602	LC_IGP_R4	IgG1_G0FN/IgG1_G0N	14	66071733	1	5.09E-14	2.22E-13	+	0.257449	0.2827	+	G	T	0.489575934	0.0338901 +	G	T	0.5061	0.0386
rs11158602	LC_IGP52	IgG1_G0n	14	66071733	1	2.02E-19	1.30E-14	-	-0.307917	-0.2948	+	G	T	0.488833643	0.0337318 +	G	T	0.5061	0.0383
rs11158602	LC_IGP14	IgG1_G0N	14	66071733	1	1.23E-15	2.64E-11	-	-0.273199	-0.2532	+	G	T	0.489575934	0.0338056 +	G	T	0.5061	0.038
rs11158602	LC_IGP55	IgG1_G0Nn	14	66071733	1	1.95E-16	2.55E-11	-	-0.2824	-0.2536	+	G	T	0.488833643	0.0339686 +	G	T	0.5061	0.038
rs11158602	LC_IGP12	IgG1_G1	14	66071733	1	9.06E-17	4.43E-14	-	-0.282953	-0.2898	+	G	T	0.488991753	0.0336586 +	G	T	0.5061	0.0384

rs11158602	LC_IGP_R2	IgG1_G1F/IgG1_G1	14	66071733	1	1.25E-17	3.31E-14	+	0.290768	0.2905	+	G	T	0.488991753	0.0336323	+	G	T	0.5061	0.0383
rs11158602	LC_IGP_R5	IgG1_G1FN/IgG1_G1N	14	66071733	1	2.79E-14	1.35E-17	+	0.261158	0.328	+	G	T	0.489839791	0.0340175	+	G	T	0.5061	0.0384
rs11158602	LC_IGP53	IgG1_G1n	14	66071733	1	1.08E-15	7.09E-14	-	-0.275433	-0.2872	+	G	T	0.488833643	0.0340075	+	G	T	0.5061	0.0384
rs11158602	LC_IGP15	IgG1_G1N	14	66071733	1	1.99E-11	4.06E-11	-	-0.230569	-0.2519	+	G	T	0.489839791	0.034135	+	G	T	0.5061	0.0382
rs11158602	LC_IGP56	IgG1_G1Nn	14	66071733	1	9.17E-12	7.71E-11	-	-0.235267	-0.2478	+	G	T	0.488833643	0.0342482	+	G	T	0.5061	0.0381
rs11158602	LC_IGP_R36	IgG1_G1NS1/IgG1_G1N	14	66071733	1	9.06E-09	4.27E-12	+	0.196722	0.2589	+	G	T	0.489814735	0.0340475	+	G	T	0.5061	0.0374
rs11158602	LC_IGP_R32	IgG1_G1S1/IgG1_G1	14	66071733	1	1.36E-10	1.43E-10	+	0.219222	0.236	+	G	T	0.488672477	0.0339231	+	G	T	0.5061	0.0368
rs11158602	LC_IGP13	IgG1_G2	14	66071733	1	4.30E-08	2.57E-11	-	-0.18771	-0.252	+	G	T	0.488991753	0.0341025	+	G	T	0.5061	0.0378
rs11158602	LC_IGP_R3	IgG1_G2F/IgG1_G2	14	66071733	1	1.03E-15	2.72E-15	+	0.273545	0.3011	+	G	T	0.488679401	0.0337553	+	G	T	0.5061	0.0381
rs11158602	LC_IGP_R8	IgG1_G2FS1/IgG1_G2S1	14	66071733	1	6.09E-10	2.88E-13	+	0.211766	0.2773	+	G	T	0.48838088	0.0340161	+	G	T	0.5061	0.038
rs11158602	LC_IGP_R26	IgG1_G2N/IgG1_G1N	14	66071733	1	2.34E-12	5.46E-09	+	0.240366	0.2174	+	G	T	0.489493519	0.0340117	+	G	T	0.5061	0.0373
rs11158602	LC_IGP148	IgG2 FG0n total/G0n	14	66071733	1	9.45E-09	1.83E-14	+	0.197068	0.2914	+	G	T	0.487672106	0.0341507	+	G	T	0.506	0.038
rs11158602	LC_IGP97	IgG2_G0	14	66071733	1	8.87E-11	2.23E-14	-	-0.221296	-0.2911	+	G	T	0.488679401	0.0338996	+	G	T	0.5061	0.0381
rs11158602	LC_IGP_R41	IgG2_G0F/IgG2_G0	14	66071733	1	4.69E-08	6.87E-14	+	0.186691	0.2834	+	G	T	0.488679401	0.0340151	+	G	T	0.5061	0.0378
rs11158602	LC_IGP138	IgG2_G0n	14	66071733	1	5.74E-11	1.33E-14	-	-0.224326	-0.294	+	G	T	0.487672106	0.0340143	+	G	T	0.506	0.0382
rs11158602	LC_IGP_R64	IgG2_G2/IgG2_G1	14	66071733	1	1.18E-09	9.50E-13	+	0.208979	0.2659	+	G	T	0.487747552	0.0341537	+	G	T	0.5061	0.0373
rs4902407	LC_IGP78	IgG1 BG0n/G0n	14	66074635	1	1.38E-13	6.96E-10	-	-0.254277	-0.2358	+	G	T	0.488832714	0.0340753	+	G	T	0.5018	0.0382
rs4902407	LC_IGP79	IgG1 BG1n/G1n	14	66074635	1	6.29E-13	4.62E-12	-	-0.247963	-0.2645	+	G	T	0.488832714	0.034186	+	G	T	0.5018	0.0382
rs4902407	LC_IGP32	IgG1 BG1S1/(BG1+BG1S1)	14	66074635	1	9.58E-09	3.90E-12	+	0.19635	0.2601	+	G	T	0.48981381	0.0340406	+	G	T	0.5018	0.0375
rs4902407	LC_IGP77	IgG1 Bn	14	66074635	1	7.18E-13	1.46E-11	-	-0.247396	-0.2593	+	G	T	0.488832714	0.0341957	+	G	T	0.5018	0.0384
rs4902407	LC_IGP86	IgG1 Bn/Fn total	14	66074635	1	9.08E-14	9.44E-12	-	-0.256754	-0.2618	+	G	T	0.488832714	0.0341452	+	G	T	0.5018	0.0384
rs4902407	LC_IGP84	IgG1 FBn/Bn total	14	66074635	1	5.45E-15	6.48E-16	+	0.269555	0.3131	+	G	T	0.488832714	0.0341558	+	G	T	0.5018	0.0388
rs4902407	LC_IGP62	IgG1 FG0n total/G0n	14	66074635	1	9.92E-18	2.52E-12	+	0.293531	0.2697	+	G	T	0.488832714	0.0338367	+	G	T	0.5018	0.0385
rs4902407	LC_IGP63	IgG1 FG1n total/G1n	14	66074635	1	1.44E-17	1.10E-14	+	0.292962	0.2976	+	G	T	0.488832714	0.0339442	+	G	T	0.5018	0.0385
rs4902407	LC_IGP64	IgG1 FG2n total/G2n	14	66074635	1	4.12E-14	2.17E-15	+	0.260379	0.3038	+	G	T	0.488832714	0.0341452	+	G	T	0.5018	0.0383
rs4902407	LC_IGP68	IgG1 FG2n/G2n	14	66074635	1	2.07E-10	3.58E-09	+	0.219484	0.225	+	G	T	0.488832714	0.0343116	+	G	T	0.5018	0.0381
rs4902407	LC_IGP61	IgG1 Fn total	14	66074635	1	4.09E-17	2.91E-14	+	0.288668	0.293	+	G	T	0.488832714	0.0339421	+	G	T	0.5018	0.0385
rs4902407	LC_IGP81	IgG1 Fn/Bn	14	66074635	1	3.14E-13	4.84E-11	+	0.251067	0.2513	+	G	T	0.488832714	0.0341597	+	G	T	0.5018	0.0382
rs4902407	LC_IGP21	IgG1 Fucosylation	14	66074635	1	1.59E-15	2.76E-13	+	0.272995	0.281	+	G	T	0.488579531	0.0339136	+	G	T	0.5018	0.0385
rs4902407	LC_IGP28	IgG1 G1S1/(G1+G1S1)	14	66074635	1	7.26E-11	1.96E-10	+	0.222374	0.2348	+	G	T	0.48867156	0.0339052	+	G	T	0.5018	0.0369
rs4902407	LC_IGP26	IgG1 GS1/(G+GS1)	14	66074635	1	4.53E-08	2.12E-09	+	0.187463	0.2267	+	G	T	0.48867156	0.0341168	+	G	T	0.5018	0.0379
rs4902407	LC_IGP27	IgG1 GS1/(G0+G+GS1)	14	66074635	1	2.57E-09	3.09E-08	+	0.203782	0.2079	+	G	T	0.48867156	0.0340188	+	G	T	0.5018	0.0376
rs4902407	LC_IGP11	IgG1_G0	14	66074635	1	1.81E-19	2.58E-14	-	-0.305917	-0.2918	+	G	T	0.488990837	0.0334731	+	G	T	0.5018	0.0383
rs4902407	LC_IGP_R1	IgG1_G0F/IgG1_G0	14	66074635	1	4.55E-17	4.98E-12	+	0.285025	0.2651	+	G	T	0.488990837	0.03357	+	G	T	0.5018	0.0384
rs4902407	LC_IGP_R4	IgG1_G0FN/IgG1_G0N	14	66074635	1	5.09E-14	4.00E-13	+	0.257453	0.2808	+	G	T	0.489575015	0.03389	+	G	T	0.5018	0.0387
rs4902407	LC_IGP52	IgG1_G0n	14	66074635	1	2.02E-19	2.16E-14	-	-0.307919	-0.2933	+	G	T	0.488832714	0.0337316	+	G	T	0.5018	0.0384
rs4902407	LC_IGP14	IgG1_G0N	14	66074635	1	1.23E-15	3.22E-11	-	-0.2732	-0.253	+	G	T	0.489575015	0.0338055	+	G	T	0.5018	0.0381
rs4902407	LC_IGP55	IgG1_G0Nn	14	66074635	1	1.95E-16	3.20E-11	-	-0.282402	-0.2532	+	G	T	0.488832714	0.0339685	+	G	T	0.5018	0.0382
rs4902407	LC_IGP12	IgG1_G1	14	66074635	1	9.05E-17	1.11E-13	-	-0.282958	-0.2862	+	G	T	0.488990837	0.0336585	+	G	T	0.5018	0.0385
rs4902407	LC_IGP_R2	IgG1_G1F/IgG1_G1	14	66074635	1	1.25E-17	7.13E-14	+	0.290772	0.2876	+	G	T	0.488990837	0.0336321	+	G	T	0.5018	0.0384
rs4902407	LC_IGP_R5	IgG1_G1FN/IgG1_G1N	14	66074635	1	2.79E-14	2.65E-17	+	0.26116	0.3261	+	G	T	0.489838868	0.0340173	+	G	T	0.5018	0.0385
rs4902407	LC_IGP53	IgG1_G1n	14	66074635	1	1.08E-15	1.75E-13	-	-0.275438	-0.2836	+	G	T	0.488832714	0.0340074	+	G	T	0.5018	0.0385
rs4902407	LC_IGP15	IgG1_G1N	14	66074635	1	1.99E-11	7.66E-11	-	-0.230571	-0.2491	+	G	T	0.489838868	0.0341349	+	G	T	0.5018	0.0383
rs4902407	LC_IGP56	IgG1_G1Nn	14	66074635	1	9.17E-12	1.49E-10	-	-0.23527	-0.2449	+	G	T	0.488832714	0.0342481	+	G	T	0.5018	0.0382
rs4902407	LC_IGP_R36	IgG1_G1NS1/IgG1_G1N	14	66074635	1	9.05E-09	4.64E-12	+	0.196723	0.2594	+	G	T	0.48981381	0.0340474	+	G	T	0.5018	0.0375
rs4902407	LC_IGP_R32	IgG1_G1S1/IgG1_G1	14	66074635	1	1.36E-10	3.11E-10	+	0.219223	0.2324	+	G	T	0.48867156	0.033923	+	G	T	0.5018	0.0369
rs4902407	LC_IGP13	IgG1_G2	14	66074635	1	4.29E-08	5.63E-11	-	-0.187715	-0.2485	+	G	T	0.488990837	0.0341024	+	G	T	0.5018	0.0379
rs4902407	LC_IGP_R3	IgG1_G2F/IgG1_G2	14	66074635	1	1.03E-15	5.87E-15	+	0.273547	0.2984	+	G	T	0.488678484	0.0337552	+	G	T	0.5018	0.0382
rs4902407	LC_IGP_R8	IgG1_G2FS1/IgG1_G2S1	14	66074635	1	6.08E-10	7.76E-13	+	0.211771	0.2731	+	G	T	0.488379963	0.034016	+	G	T	0.5018	0.0381
rs4902407	LC_IGP_R26	IgG1_G2N/IgG1_G1N	14	66074635	1	2.34E-12	4.50E-09	+	0.240368	0.2193	+	G	T	0.489492593	0.0340115	+	G	T	0.5018	0.0374
rs4902407	LC_IGP148	IgG2 FG0n total/G0n	14	66074635	1	9.44E-09	2.43E-14	+	0.197075	0.2908	+	G	T	0.487671182	0.0341506	+	G	T	0.5017	0.0381
rs4902407	LC_IGP97	IgG2_G0	14	66074635	1	8.86E-11	2.55E-14	-	-0.221299	-0.2912	+	G	T	0.488678484	0.0338995	+	G	T	0.5018	0.0382
rs4902407	LC_IGP_R41	IgG2_G0F/IgG2_G0	14	66074635	1	4.69E-08	9.14E-14	+	0.186697	0.2828	+	G	T	0.488678484	0.034015	+	G	T	0.5018	0.038
rs4902407	LC_IGP138	IgG2_G0n	14	66074635	1	5.73E-11	1.56E-14	-	-0.22433	-0.294	+	G	T	0.487671182	0.0340141	+	G	T	0.5017	0.0383
rs4902407	LC_IGP_R64	IgG2_G2/IgG2_G1	14	66074635	1	1.18E-09	4.06E-13	+	0.20898	0.2713	+	G	T	0.487746634	0.0341535	+	G	T	0.5018	0.0374
rs7155963	LC_IGP78	IgG1 BG0n/G0n	14	66076029	1	1.48E-13	2.08E-09	-	-0.254224	-0.2334	+	A	T	0.487064746	0.0341092	+	A	T	0.4785	0.039
rs7155963	LC_IGP79	IgG1 BG1n/G1n	14	66076029	1	6.75E-13	2.84E-11	-	-0.24787	-0.259	+	A	T	0.487064746	0.0342201	+	A	T	0.4785	0.0389
rs7155963	LC_IGP32	IgG1 BG1S1/(BG1+BG1S1)	14	66076029	1	1.10E-08	1.58E-11	+	0.195717	0.2563	+	A	T	0.488054562	0.0340753	+	A	T	0.4785	0.038
rs7155963	LC_IGP77	IgG1 Bn	14	66076029	1	7.45E-13	6.92E-11	-	-0.247461	-0.2551	+	A	T	0.487064746	0.0342291	+	A	T	0.4785	0.0391
rs7155963	LC_IGP86	IgG1 Bn/Fn total	14	66076029	1	9.39E-14	4.54E-11	-	-0.256848	-0.2577	+	A	T	0.487064746	0.0341784	+	A	T	0.4785	0.0391
rs7155963	LC_IGP84	IgG1 FBn/Bn total	14	66076029	1	5.61E-15	2.64E-15	+	0.269688	0.3112	+	A	T	0.487064746	0.0341889	+	A	T	0.4785	0.0394
rs7155963	LC_IGP62	IgG1 FG0n total/G0n	14	66076029	1	9.57E-18	9.36E-12	+	0.293949	0.2673	+	A	T	0.487064746	0.0338682	+	A	T	0.4785	0.0392
rs7155963	LC_IGP63	IgG1 FG1n total/G1n	14	66076029	1	1.25E-17	9.71E-14	+	0.293796	0.2914	+	A	T	0.487064746	0.0339735	+	A	T	0.4785	0.0392
rs7155963	LC_IGP64	IgG1 FG2n total/G2n	14	66076029	1	2.29E-14	1.65E-14	+	0.26321	0.2987	+	A	T	0.487064746	0.0341655	+	A	T	0.4785	0.0389
rs7155963	LC_IGP68	IgG1 FG2n/G2n	14	66076029	1	1.10E-10	3.77E-08	+	0.223015	0.2137	+	A	T	0.487064746	0.034331	+	A	T	0.4785	0.0388
rs7155963	LC_IGP61	IgG1 Fn total	14	66076029	1	3.72E-17	1.93E-13	+	0.28932	0.2884	+	A	T	0.487064746	0.0339724	+	A	T	0.4785	0.0392
rs7155963	LC_IGP81	IgG1 Fn/Bn	14	66076029	1	3.24E-13	2.99E-10	+	0.251163	0.2453	+	A	T	0.487064746	0.0341929	+	A	T	0.4785	0.0389
rs7155963	LC_IGP21	IgG1 Fucosylation	14	66076029	1	1.73E-15	3.04E-12	+	0.27291	0.2733	+	A	T	0.486820592	0.0339475	+	A	T	0.4785	0.0392
rs7155963	LC_IGP28	IgG1 G1S1/(G1+G1S1)	14	66076029	1	5.89E-11	6.75E-10	+	0.223643	0.2306	+	A	T	0.4869263	0.0339329	+	A	T	0.4785	0.0374
rs7155963	LC_IGP26	IgG1 GS1/(G+GS1)	14	66076029	1	3.84E-08	9.84E-09	+	0.188641	0.2202	+	A	T	0.4869263	0.0341455	+	A	T	0.4785	0.0384
rs7155963	LC_IGP27	IgG1 GS1/(G0+G+GS1)	14	66076029	1	2.01E-09	1.28E-07	+	0.205321	0.2017	+	A	T	0.4869263	0.0340459	+	A	T	0.4785	0.0382
rs7155963	LC_IGP11	IgG1_G0	14	66076029	1	1.06E-19	1.64E-13	-	-0.308129	-0.288	+	A	T	0.487247709	0.0334936	+	A	T	0.4785	0.039
rs7155963	LC_IGP_R1	IgG1_G0F/IgG1_G0	14	66076029	1	4.33E-17	1.96E-11	+	0.285488	0.2624	+	A	T	0.487247709	0.0336004	+	A	T	0.4785	0.0391
rs7155963	LC_IGP_R4	IgG1_G0FN/IgG1_G0N	14	66076029	1	3.27E-14	1.36E-12	+	0.259574	0.2788	+	A	T	0.487645744	0.033906	+	A	T	0.4785	0.0393
rs7155963	LC_IGP52	IgG1_G0n	14	66076029	1	1.18E-19	1.29E-13	-	-0.310152	-0.2898	+	A	T	0.487064746	0.0337528	+	A	T	0.4785	0.0391
rs7155963	LC_IGP14	IgG1_G0N	14	66076029	1	5.44E-16	1.54E-10	-	-0.276755	-0.2488	+	A	T	0.487645744	0.0338138	+	A	T	0.4785	0.0389
rs7155963	LC_IGP55	IgG1_G0Nn	14	66076029	1	1.50E-16	1.46E-10	-	-0.283724	-0.2494	+	A	T	0.487064746	0.0339954	+	A	T	0.4785	0.0389
rs7155963	LC_IGP12	IgG1_G1	14	66076029	1	7.86E-17	6.33E-13	-	-0.283775	-0.2814	+	A	T	0.487247709	0.0336871	+	A	T	0.4785	0.0391
rs7155963	LC_IGP_R2	IgG1_G1F/IgG1_G1	14	66076029	1	9.48E-18	6.88E-13	+	0.292115	0.2806	+	A	T	0.487247709	0.0336579	+	A	T	0.4785	0.0391
rs7155963	LC_IGP_R5	IgG1_G1FN/IgG1_G1N	14	66076029	1	3.05E-14	1.83E-16	+	0.261014	0.3221	+	A	T	0.488083948	0.0340509	+	A	T	0.4785	0.0391
rs7155963	LC_IGP53	IgG1_G1n	14	66076029	1	9.91E-16	9.32E-13	-	-0.276055	-0.2791	+	A	T	0.487064746	0.034038	+	A	T	0.4785	0.0391
rs7155963	LC_IGP15	IgG1_G1N	14	66076029	1	2.33E-11	3.22E-10	-	-0.230005	-0.2449	+	A	T	0.488083948	0.03417	+	A	T	0.4785	0.0389
rs7155963	LC_IGP56	IgG1_G1Nn	14	66076029	1	1.09E-11	5.69E-10	-	-0.234656	-0.241	+	A	T	0.487064746	0.0342843	+	A	T	0.4785	0.0389
rs7155963	LC_IGP_R36	IgG1_G1NS1/IgG1_G1N	14	66076029	1	1.04E-08	1.82E-11	+	0.196095	0.2558	+	A	T	0.488054562	0.0340821	+	A	T	0.4785	0.0381
rs7155963	LC_IGP_R32	IgG1_G1S1/IgG1_G1	14	66076029	1	1.12E-10	1.01E-09	+	0.220427	0.2285	+	A	T	0.4869263	0.033951	+	A	T	0.4785	0.0374
rs7155963	LC_IGP13	IgG1_G2	14	66076029	1	4.36E-08	1.55E-10	-	-0.1878	-0.2458	+	A	T	0.487247709	0.0341346	+	A	T	0.4785	0.0384
rs7155963	LC_IGP_R3	IgG1_G2F/IgG1_G2	14	66076029	1	5.19E-16	4.76E-14	+	0.276618	0.2929	+	A	T	0.486934291	0.0337729	+	A	T	0.4785	0.0388
rs7155963	LC_IGP_R8	IgG1_G2FS1/IgG1_G2S1	14	66076029	1	5.08E-10	6.25E-12	+	0.212929	0.2662	+	A	T	0.486636836	0.0340444	+	A	T	0.4785	0.0387
rs7155963	LC_IGP_R26	IgG1_G2N/IgG1_G1N	14	66076029	1	2.67E-12	3.50E-08	+	0.239968	0.2097	+	A	T	0.487731173	0.0340462	+	A	T	0.4785	0.038
rs7155963	LC_IGP148	IgG2 FG0n total/G0n	14	66076029	1	7.58E-09	5.94E-13	+	0.198522	0.2786	+	A	T	0.485915025	0.034179	+	A	T	0.4785	0.0387
rs7155963	LC_IGP97	IgG2_G0	14	66076029	1	3.90E-11	4.69E-13	-	-0.225601	-0.2803	+	A	T	0.486752751	0.0339083	+	A	T	0.4785	0.0388
rs7155963	LC_IGP_R41	IgG2_G0F/IgG2_G0	14	66076029	1	2.61E-08	2.49E-12	+	0.190324	0.2695	+	A	T	0.486752751	0.0340287	+	A	T	0.4785	0.0385
rs7155963	LC_IGP138	IgG2_G0n	14	66076029	1	3.78E-11	3.36E-13	-	-0.226634	-0.2824	+	A	T	0.485915025	0.0340384	+	A	T	0.4785	0.0388
rs7155963	LC_IGP_R64	IgG2_G2/IgG2_G1	14	66076029	1	7.95E-10	1.01E-11	+	0.21131	0.2581	+	A	T	0.486001224	0.0341781	+	A	T	0.4785	0.0379
rs8012278	LC_IGP78	IgG1 BG0n/G0n	14	66082573	1	3.55E-14	8.33E-10	-	-0.261234	-0.2304	+	G	A	0.476212206	0.0341675	+	G	A	0.4935	0.0375
rs8012278	LC_IGP79	IgG1 BG1n/G1n	14	66082573	1	3.02E-13	8.45E-12	-	-0.252227	-0.2562	+	G	A	0.476212206	0.0342917	+	G	A	0.4935	0.0375
rs8012278	LC_IGP32	IgG1 BG1S1/(BG1+BG1S1)	14	66082573	1	2.29E-08	3.05E-12	+	0.191938	0.256	+	G	A	0.477255549	0.0341748	+	G	A	0.4935	0.0367
rs8012278	LC_IGP77	IgG1 Bn	14	66082573	1	3.44E-13	2.14E-11	-	-0.251673	-0.2522	+	G	A	0.476212206	0.0343013	+	G	A	0.4935	0.0377
rs8012278	LC_IGP86	IgG1 Bn/Fn total	14	66082573	1	4.90E-14	1.28E-11	-	-0.260412	-0.2552	+	G	A	0.476212206	0.0342531	+	G	A	0.4935	0.0377
rs8012278	LC_IGP84	IgG1 FBn/Bn total	14	66082573	1	2.32E-15	3.41E-16	+	0.274135	0.3096	+	G	A	0.476212206	0.0342587	+	G	A	0.4935	0.038
rs8012278	LC_IGP62	IgG1 FG0n total/G0n	14	66082573	1	1.56E-17	2.59E-12	+	0.292805	0.2645	+	G	A	0.476212206	0.0339659	+	G	A	0.4935	0.0378
rs8012278	LC_IGP63	IgG1 FG1n total/G1n	14	66082573	1	1.17E-17	1.55E-14	+	0.294787	0.2901	+	G	A	0.476212206	0.03406	+	G	A	0.4935	0.0378
rs8012278	LC_IGP64	IgG1 FG2n total/G2n	14	66082573	1	5.51E-14	2.72E-15	+	0.26002	0.2967	+	G	A	0.476212206	0.0342721	+	G	A	0.4935	0.0375
rs8012278	LC_IGP68	IgG1 FG2n/G2n	14	66082573	1	1.79E-10	1.82E-08	+	0.221044	0.2105	+	G	A	0.476212206	0.0344299	+	G	A	0.4935	0.0374
rs8012278	LC_IGP61	IgG1 Fn total	14	66082573	1	7.39E-17	3.65E-14	+	0.287343	0.2862	+	G	A	0.476212206	0.0340745	+	G	A	0.4935	0.0378
rs8012278	LC_IGP81	IgG1 Fn/Bn	14	66082573	1	1.76E-13	1.11E-10	+	0.2546	0.242	+	G	A	0.476212206	0.0342684	+	G	A	0.4935	0.0375
rs8012278	LC_IGP21	IgG1 Fucosylation	14	66082573	1	2.41E-15	4.86E-13	+	0.272249	0.2727	+	G	A	0.475404747	0.0340462	+	G	A	0.4935	0.0377
rs8012278	LC_IGP28	IgG1 G1S1/(G1+G1S1)	14	66082573	1	3.99E-10	2.39E-10	+	0.21436	0.2292	+	G	A	0.475601529	0.0340622	+	G	A	0.4935	0.0362
rs8012278	LC_IGP27	IgG1 GS1/(G0+G+GS1)	14	66082573	1	4.00E-09	2.55E-08	+	0.202054	0.205	+	G	A	0.475601529	0.0341505	+	G	A	0.4935	0.0368
rs8012278	LC_IGP11	IgG1_G0	14	66082573	1	4.74E-19	2.68E-14	-	-0.303487	-0.2858	+	G	A	0.475936775	0.0336121	+	G	A	0.4935	0.0376
rs8012278	LC_IGP_R1	IgG1_G0F/IgG1_G0	14	66082573	1	1.70E-16	5.87E-12	+	0.280843	0.2594	+	G	A	0.475936775	0.0337166	+	G	A	0.4935	0.0377
rs8012278	LC_IGP_R4	IgG1_G0FN/IgG1_G0N	14	66082573	1	6.56E-15	3.11E-13	+	0.267226	0.2763	+	G	A	0.476488977	0.0339675	+	G	A	0.4935	0.0379
rs8012278	LC_IGP52	IgG1_G0n	14	66082573	1	4.19E-19	2.33E-14	-	-0.306316	-0.2871	+	G	A	0.476212206	0.0338662	+	G	A	0.4935	0.0376
rs8012278	LC_IGP14	IgG1_G0N	14	66082573	1	6.47E-17	4.03E-11	-	-0.286084	-0.2468	+	G	A	0.476488977	0.0338644	+	G	A	0.4935	0.0374
rs8012278	LC_IGP55	IgG1_G0Nn	14	66082573	1	2.79E-17	4.14E-11	-	-0.291144	-0.247	+	G	A	0.476212206	0.0340482	+	G	A	0.4935	0.0374
rs8012278	LC_IGP12	IgG1_G1	14	66082573	1	3.97E-16	1.05E-13	-	-0.278042	-0.2807	+	G	A	0.475936775	0.0338088	+	G	A	0.4935	0.0378
rs8012278	LC_IGP_R2	IgG1_G1F/IgG1_G1	14	66082573	1	2.79E-17	1.25E-13	+	0.288714	0.2792	+	G	A	0.475936775	0.0337684	+	G	A	0.4935	0.0377
rs8012278	LC_IGP_R5	IgG1_G1FN/IgG1_G1N	14	66082573	1	1.69E-14	1.86E-17	+	0.264224	0.321	+	G	A	0.477311501	0.0341229	+	G	A	0.4935	0.0378
rs8012278	LC_IGP53	IgG1_G1n	14	66082573	1	2.86E-15	1.81E-13	-	-0.272337	-0.2778	+	G	A	0.476212206	0.034148	+	G	A	0.4935	0.0377

rs8012278	LC_IGP15	IgG1_G1N	14	66082573	1	1.79E-11	1.08E-10	-	-0.23188	-0.2423	+	G	A	0.477311501	0.034249	+	G	A	0.4935	0.0375
rs8012278	LC_IGP56	IgG1_G1Nn	14	66082573	1	7.13E-12	2.17E-10	-	-0.237333	-0.238	+	G	A	0.476212206	0.034364	+	G	A	0.4935	0.0375
rs8012278	LC_IGP_R36	IgG1_G1NS1/IgG1_G1N	14	66082573	1	2.04E-08	3.51E-12	+	0.19267	0.2555	+	G	A	0.477255549	0.0341804	+	G	A	0.4935	0.0367
rs8012278	LC_IGP_R32	IgG1_G1S1/IgG1_G1	14	66082573	1	7.01E-10	3.62E-10	+	0.21139	0.2272	+	G	A	0.475601529	0.0340788	+	G	A	0.4935	0.0362
rs8012278	LC_IGP_R3	IgG1_G2F/IgG1_G2	14	66082573	1	1.88E-15	8.37E-15	+	0.272046	0.2909	+	G	A	0.475616443	0.0338891	+	G	A	0.4935	0.0375
rs8012278	LC_IGP_R8	IgG1_G2FS1/IgG1_G2S1	14	66082573	1	4.58E-10	1.11E-12	+	0.214051	0.266	+	G	A	0.475325901	0.0341337	+	G	A	0.4935	0.0374
rs8012278	LC_IGP_R26	IgG1_G2N/IgG1_G1N	14	66082573	1	4.17E-12	1.18E-08	+	0.238442	0.2087	+	G	A	0.476918827	0.034141	+	G	A	0.4935	0.0366
rs8012278	LC_IGP148	IgG2 FG0n total/G0n	14	66082573	1	6.47E-09	1.34E-13	+	0.199985	0.2767	+	G	A	0.474512623	0.0342707	+	G	A	0.4935	0.0374
rs8012278	LC_IGP97	IgG2_G0	14	66082573	1	2.01E-10	1.07E-13	-	-0.217891	-0.2782	+	G	A	0.475616443	0.0340384	+	G	A	0.4935	0.0374
rs8012278	LC_IGP138	IgG2_G0n	14	66082573	1	1.18E-10	7.42E-14	-	-0.221496	-0.2804	+	G	A	0.474512623	0.0341563	+	G	A	0.4935	0.0375
rs8012278	LC_IGP_R64	IgG2_G2/IgG2_G1	14	66082573	1	8.52E-09	1.97E-12	+	0.19865	0.2578	+	G	A	0.474668605	0.0343206	+	G	A	0.4935	0.0366
rs76601910	LC_IGP61	IgG1 Fn total	14	66087341	1	3.62E-08	1.63E-06	-	-0.23986	-0.2464	+	C	G	0.200538043	0.0433332	+	C	G	0.1663	0.0514
rs2268955	LC_IGP84	IgG1 FBn/Bn total	14	66090009	1	1.05E-08	1.17E-07	+	0.2034	0.207	+	A	T	0.567152416	0.0353607	+	A	T	0.5862	0.0391
rs2268955	LC_IGP63	IgG1 FG1n total/G1n	14	66090009	1	2.12E-10	8.34E-08	+	0.224969	0.2082	+	A	T	0.567152416	0.0351863	+	A	T	0.5862	0.0388
rs2268955	LC_IGP64	IgG1 FG2n total/G2n	14	66090009	1	1.72E-08	2.36E-08	+	0.20013	0.2157	+	A	T	0.567152416	0.0353166	+	A	T	0.5862	0.0386
rs2268955	LC_IGP61	IgG1 Fn total	14	66090009	1	5.38E-10	2.09E-07	+	0.219738	0.2019	+	A	T	0.567152416	0.0351815	+	A	T	0.5862	0.0389
rs2268955	LC_IGP21	IgG1 Fucosylation	14	66090009	1	3.96E-09	1.56E-06	+	0.207625	0.1866	+	A	T	0.566820284	0.0350826	+	A	T	0.5862	0.0388
rs2268955	LC_IGP11	IgG1_G0	14	66090009	1	1.12E-11	4.53E-07	-	-0.237384	-0.1952	+	A	T	0.566821014	0.0347116	+	A	T	0.5862	0.0387
rs2268955	LC_IGP52	IgG1_G0n	14	66090009	1	1.80E-11	4.97E-07	-	-0.236976	-0.1949	+	A	T	0.567152416	0.0350051	+	A	T	0.5862	0.0388
rs2268955	LC_IGP12	IgG1_G1	14	66090009	1	7.54E-10	1.60E-07	-	-0.21573	-0.2035	+	A	T	0.567126451	0.0348448	+	A	T	0.5862	0.0388
rs2268955	LC_IGP_R2	IgG1_G1F/IgG1_G1	14	66090009	1	2.17E-10	1.65E-07	+	0.222542	0.203	+	A	T	0.567126451	0.0348332	+	A	T	0.5862	0.0388
rs2268955	LC_IGP_R5	IgG1_G1FN/IgG1_G1N	14	66090009	1	1.73E-08	1.78E-08	+	0.199325	0.2188	+	A	T	0.567888069	0.0351828	+	A	T	0.5862	0.0388
rs2268955	LC_IGP53	IgG1_G1n	14	66090009	1	1.73E-09	2.27E-07	-	-0.21319	-0.2008	+	A	T	0.567152416	0.0352036	+	A	T	0.5862	0.0388
rs2268955	LC_IGP_R3	IgG1_G2F/IgG1_G2	14	66090009	1	2.14E-09	4.46E-08	+	0.210278	0.211	+	A	T	0.566556235	0.0349261	+	A	T	0.5862	0.0386
rs74245865	LC_IGP61	IgG1 Fn total	14	66094063	1	3.62E-08	1.17E-06	-	-0.239861	-0.2483	+	G	A	0.200537486	0.0433332	+	G	A	0.1656	0.0511
rs12886334	LC_IGP78	IgG1 BG0n/G0n	14	66094671	1	3.18E-14	7.85E-10	-	-0.261474	-0.2304	+	G	T	0.477358426	0.0341333	+	G	T	0.4942	0.0375
rs12886334	LC_IGP79	IgG1 BG1n/G1n	14	66094671	1	2.50E-13	7.91E-12	-	-0.252855	-0.2562	+	G	T	0.477358426	0.0342557	+	G	T	0.4942	0.0374
rs12886334	LC_IGP32	IgG1 BG1S1/(BG1+BG1S1)	14	66094671	1	1.93E-08	2.74E-12	+	0.192757	0.2563	+	G	T	0.478396116	0.0341399	+	G	T	0.4942	0.0367
rs12886334	LC_IGP77	IgG1 Bn	14	66094671	1	2.98E-13	2.02E-11	-	-0.252092	-0.2522	+	G	T	0.477358426	0.0342663	+	G	T	0.4942	0.0376
rs12886334	LC_IGP86	IgG1 Bn/Fn total	14	66094671	1	4.17E-14	1.20E-11	-	-0.260882	-0.2552	+	G	T	0.477358426	0.0342178	+	G	T	0.4942	0.0376
rs12886334	LC_IGP84	IgG1 FBn/Bn total	14	66094671	1	2.07E-15	3.12E-16	+	0.274367	0.3096	+	G	T	0.477358426	0.0342243	+	G	T	0.4942	0.0379
rs12886334	LC_IGP62	IgG1 FG0n total/G0n	14	66094671	1	1.05E-17	2.38E-12	+	0.294087	0.2646	+	G	T	0.477358426	0.0339258	+	G	T	0.4942	0.0377
rs12886334	LC_IGP63	IgG1 FG1n total/G1n	14	66094671	1	7.66E-18	1.42E-14	+	0.296162	0.2901	+	G	T	0.477358426	0.0340194	+	G	T	0.4942	0.0377
rs12886334	LC_IGP64	IgG1 FG2n total/G2n	14	66094671	1	4.86E-14	2.48E-15	+	0.260331	0.2968	+	G	T	0.477358426	0.0342375	+	G	T	0.4942	0.0375
rs12886334	LC_IGP68	IgG1 FG2n/G2n	14	66094671	1	1.59E-10	1.73E-08	+	0.221463	0.2106	+	G	T	0.477358426	0.0343953	+	G	T	0.4942	0.0374
rs12886334	LC_IGP61	IgG1 Fn total	14	66094671	1	5.42E-17	3.34E-14	+	0.288304	0.2863	+	G	T	0.477358426	0.0340362	+	G	T	0.4942	0.0378
rs12886334	LC_IGP81	IgG1 Fn/Bn	14	66094671	1	1.49E-13	1.05E-10	+	0.255105	0.242	+	G	T	0.477358426	0.034233	+	G	T	0.4942	0.0375
rs12886334	LC_IGP21	IgG1 Fucosylation	14	66094671	1	2.10E-15	4.49E-13	+	0.272574	0.2727	+	G	T	0.476545314	0.0340115	+	G	T	0.4942	0.0377
rs12886334	LC_IGP28	IgG1 G1S1/(G1+G1S1)	14	66094671	1	2.73E-10	2.21E-10	+	0.216143	0.2294	+	G	T	0.476733028	0.0340231	+	G	T	0.4942	0.0362
rs12886334	LC_IGP27	IgG1 GS1/(G0+G+GS1)	14	66094671	1	2.97E-09	2.42E-08	+	0.203534	0.2051	+	G	T	0.476733028	0.0341129	+	G	T	0.4942	0.0368
rs12886334	LC_IGP11	IgG1_G0	14	66094671	1	2.81E-19	2.50E-14	-	-0.305127	-0.2858	+	G	T	0.477066891	0.0335706	+	G	T	0.4942	0.0375
rs12886334	LC_IGP_R1	IgG1_G0F/IgG1_G0	14	66094671	1	1.04E-16	5.39E-12	+	0.28252	0.2595	+	G	T	0.477066891	0.0336756	+	G	T	0.4942	0.0376
rs12886334	LC_IGP_R4	IgG1_G0FN/IgG1_G0N	14	66094671	1	6.11E-15	2.88E-13	+	0.26728	0.2764	+	G	T	0.477621862	0.0339348	+	G	T	0.4942	0.0379
rs12886334	LC_IGP52	IgG1_G0n	14	66094671	1	2.46E-19	2.18E-14	-	-0.307988	-0.287	+	G	T	0.477358426	0.0338235	+	G	T	0.4942	0.0376
rs12886334	LC_IGP14	IgG1_G0N	14	66094671	1	5.32E-17	3.85E-11	-	-0.286592	-0.2468	+	G	T	0.477621862	0.0338292	+	G	T	0.4942	0.0373
rs12886334	LC_IGP55	IgG1_G0Nn	14	66094671	1	2.37E-17	3.96E-11	-	-0.291517	-0.2469	+	G	T	0.477358426	0.034013	+	G	T	0.4942	0.0374
rs12886334	LC_IGP12	IgG1_G1	14	66094671	1	2.66E-16	9.56E-14	-	-0.279395	-0.2808	+	G	T	0.477066891	0.0337695	+	G	T	0.4942	0.0377
rs12886334	LC_IGP_R2	IgG1_G1F/IgG1_G1	14	66094671	1	1.72E-17	1.15E-13	+	0.290315	0.2792	+	G	T	0.477066891	0.0337276	+	G	T	0.4942	0.0376
rs12886334	LC_IGP_R5	IgG1_G1FN/IgG1_G1N	14	66094671	1	1.34E-14	1.68E-17	+	0.265001	0.321	+	G	T	0.478449262	0.0340867	+	G	T	0.4942	0.0377
rs12886334	LC_IGP53	IgG1_G1n	14	66094671	1	1.96E-15	1.65E-13	-	-0.273663	-0.2779	+	G	T	0.477358426	0.0341083	+	G	T	0.4942	0.0377
rs12886334	LC_IGP15	IgG1_G1N	14	66094671	1	1.50E-11	1.00E-10	-	-0.232534	-0.2424	+	G	T	0.478449262	0.034214	+	G	T	0.4942	0.0375
rs12886334	LC_IGP56	IgG1_G1Nn	14	66094671	1	6.06E-12	2.02E-10	-	-0.237904	-0.2381	+	G	T	0.477358426	0.0343285	+	G	T	0.4942	0.0374
rs12886334	LC_IGP_R36	IgG1_G1NS1/IgG1_G1N	14	66094671	1	1.73E-08	3.16E-12	+	0.193468	0.2558	+	G	T	0.478396116	0.0341456	+	G	T	0.4942	0.0367
rs12886334	LC_IGP_R32	IgG1_G1S1/IgG1_G1	14	66094671	1	4.97E-10	3.34E-10	+	0.213026	0.2274	+	G	T	0.476733028	0.0340403	+	G	T	0.4942	0.0362
rs12886334	LC_IGP_R3	IgG1_G2F/IgG1_G2	14	66094671	1	1.65E-15	7.63E-15	+	0.272345	0.291	+	G	T	0.476747249	0.0338551	+	G	T	0.4942	0.0374
rs12886334	LC_IGP_R8	IgG1_G2FS1/IgG1_G2S1	14	66094671	1	3.77E-10	9.99E-13	+	0.214883	0.2662	+	G	T	0.476456017	0.0340982	+	G	T	0.4942	0.0373
rs12886334	LC_IGP_R26	IgG1_G2N/IgG1_G1N	14	66094671	1	2.75E-12	1.08E-08	+	0.240203	0.209	+	G	T	0.478060802	0.0341009	+	G	T	0.4942	0.0366
rs12886334	LC_IGP148	IgG2 FG0n total/G0n	14	66094671	1	3.85E-09	1.25E-13	+	0.202729	0.2767	+	G	T	0.475651786	0.0342278	+	G	T	0.4942	0.0373
rs12886334	LC_IGP97	IgG2_G0	14	66094671	1	1.20E-10	1.01E-13	-	-0.220358	-0.2781	+	G	T	0.476747249	0.0339964	+	G	T	0.4942	0.0374
rs12886334	LC_IGP_R41	IgG2_G0F/IgG2_G0	14	66094671	1	3.46E-08	5.67E-13	+	0.189012	0.2677	+	G	T	0.476747249	0.0341	+	G	T	0.4942	0.0371
rs12886334	LC_IGP138	IgG2_G0n	14	66094671	1	7.01E-11	6.98E-14	-	-0.223938	-0.2803	+	G	T	0.475651786	0.0341135	+	G	T	0.4942	0.0374
rs12886334	LC_IGP_R64	IgG2_G2/IgG2_G1	14	66094671	1	6.65E-09	1.83E-12	+	0.199891	0.2578	+	G	T	0.475800796	0.0342835	+	G	T	0.4942	0.0366
rs80297942	LC_IGP78	IgG1 BG0n/G0n	14	66100698	1	1.30E-13	2.03E-09	-	-0.259869	-0.2475	+	A	G	0.458075589	0.0347834	+	A	G	0.4329	0.0413
rs80297942	LC_IGP79	IgG1 BG1n/G1n	14	66100698	1	3.87E-13	1.40E-10	-	-0.255411	-0.2651	+	A	G	0.458075589	0.0348875	+	A	G	0.4329	0.0413
rs80297942	LC_IGP32	IgG1 BG1S1/(BG1+BG1S1)	14	66100698	1	1.17E-08	5.32E-11	+	0.199217	0.2639	+	A	G	0.459121455	0.0347419	+	A	G	0.4331	0.0402
rs80297942	LC_IGP77	IgG1 Bn	14	66100698	1	5.66E-13	1.98E-10	-	-0.253675	-0.264	+	A	G	0.458075589	0.0349026	+	A	G	0.4329	0.0415
rs80297942	LC_IGP86	IgG1 Bn/Fn total	14	66100698	1	8.61E-14	1.28E-10	-	-0.262342	-0.2666	+	A	G	0.458075589	0.0348549	+	A	G	0.4329	0.0415
rs80297942	LC_IGP84	IgG1 FBn/Bn total	14	66100698	1	1.04E-14	4.70E-15	+	0.272343	0.3262	+	A	G	0.458075589	0.0348806	+	A	G	0.4329	0.0416
rs80297942	LC_IGP62	IgG1 FG0n total/G0n	14	66100698	1	6.20E-17	1.90E-11	+	0.292344	0.2786	+	A	G	0.458075589	0.0345799	+	A	G	0.4329	0.0415
rs80297942	LC_IGP63	IgG1 FG1n total/G1n	14	66100698	1	3.78E-17	4.42E-13	+	0.295198	0.3008	+	A	G	0.458075589	0.0346714	+	A	G	0.4329	0.0415
rs80297942	LC_IGP64	IgG1 FG2n total/G2n	14	66100698	1	1.16E-13	8.96E-14	+	0.261128	0.3073	+	A	G	0.458075589	0.034878	+	A	G	0.4329	0.0412
rs80297942	LC_IGP68	IgG1 FG2n/G2n	14	66100698	1	2.44E-10	1.93E-07	+	0.223192	0.2147	+	A	G	0.458075589	0.0350294	+	A	G	0.4329	0.0412
rs80297942	LC_IGP61	IgG1 Fn total	14	66100698	1	2.66E-16	6.88E-13	+	0.287054	0.2987	+	A	G	0.458075589	0.0346884	+	A	G	0.4329	0.0416
rs80297942	LC_IGP81	IgG1 Fn/Bn	14	66100698	1	2.60E-13	1.11E-09	+	0.257181	0.2522	+	A	G	0.458075589	0.0348668	+	A	G	0.4329	0.0414
rs80297942	LC_IGP21	IgG1 Fucosylation	14	66100698	1	9.86E-15	5.96E-12	+	0.270885	0.2854	+	A	G	0.457299938	0.0346649	+	A	G	0.4329	0.0415
rs80297942	LC_IGP28	IgG1 G1S1/(G1+G1S1)	14	66100698	1	1.14E-09	2.73E-09	+	0.212295	0.2358	+	A	G	0.457602141	0.0346661	+	A	G	0.4329	0.0396
rs80297942	LC_IGP27	IgG1 GS1/(G0+G+GS1)	14	66100698	1	6.45E-09	2.34E-08	+	0.202753	0.2257	+	A	G	0.457602141	0.034744	+	A	G	0.4329	0.0404
rs80297942	LC_IGP11	IgG1_G0	14	66100698	1	9.68E-19	8.43E-13	-	-0.30597	-0.2947	+	A	G	0.457959377	0.0341988	+	A	G	0.4329	0.0412
rs80297942	LC_IGP_R1	IgG1_G0F/IgG1_G0	14	66100698	1	4.68E-16	5.92E-11	+	0.28147	0.2709	+	A	G	0.457959377	0.0343113	+	A	G	0.4329	0.0414
rs80297942	LC_IGP_R4	IgG1_G0FN/IgG1_G0N	14	66100698	1	6.78E-14	1.52E-12	+	0.261468	0.2942	+	A	G	0.458468157	0.0345945	+	A	G	0.4329	0.0416
rs80297942	LC_IGP52	IgG1_G0n	14	66100698	1	7.81E-19	7.75E-13	-	-0.309239	-0.2958	+	A	G	0.458075589	0.0344625	+	A	G	0.4329	0.0413
rs80297942	LC_IGP14	IgG1_G0N	14	66100698	1	1.59E-16	2.10E-10	-	-0.287308	-0.261	+	A	G	0.458468157	0.0344597	+	A	G	0.4329	0.0411
rs80297942	LC_IGP55	IgG1_G0Nn	14	66100698	1	6.23E-17	2.19E-10	-	-0.292932	-0.2611	+	A	G	0.458075589	0.0346515	+	A	G	0.4329	0.0411
rs80297942	LC_IGP12	IgG1_G1	14	66100698	1	1.37E-15	1.85E-12	-	-0.277615	-0.2924	+	A	G	0.457959377	0.0344097	+	A	G	0.4329	0.0415
rs80297942	LC_IGP_R2	IgG1_G1F/IgG1_G1	14	66100698	1	8.60E-17	4.03E-12	+	0.28912	0.2877	+	A	G	0.457959377	0.0343664	+	A	G	0.4329	0.0415
rs80297942	LC_IGP_R5	IgG1_G1FN/IgG1_G1N	14	66100698	1	4.42E-14	5.79E-16	+	0.264439	0.3352	+	A	G	0.459222017	0.0347215	+	A	G	0.4329	0.0414
rs80297942	LC_IGP53	IgG1_G1n	14	66100698	1	9.03E-15	3.07E-12	-	-0.272043	-0.2893	+	A	G	0.458075589	0.0347605	+	A	G	0.4329	0.0415
rs80297942	LC_IGP15	IgG1_G1N	14	66100698	1	2.53E-11	1.15E-09	-	-0.234053	-0.2517	+	A	G	0.459222017	0.0348369	+	A	G	0.4329	0.0414
rs80297942	LC_IGP56	IgG1_G1Nn	14	66100698	1	9.34E-12	2.22E-09	-	-0.240077	-0.247	+	A	G	0.458075589	0.0349615	+	A	G	0.4329	0.0413
rs80297942	LC_IGP_R36	IgG1_G1NS1/IgG1_G1N	14	66100698	1	1.11E-08	6.03E-11	+	0.199565	0.2633	+	A	G	0.459121455	0.034749	+	A	G	0.4331	0.0402
rs80297942	LC_IGP_R32	IgG1_G1S1/IgG1_G1	14	66100698	1	2.07E-09	3.49E-09	+	0.209001	0.2344	+	A	G	0.457602141	0.0346837	+	A	G	0.4329	0.0397
rs80297942	LC_IGP_R3	IgG1_G2F/IgG1_G2	14	66100698	1	5.73E-15	3.04E-13	+	0.271945	0.3	+	A	G	0.457628056	0.0344913	+	A	G	0.4329	0.0411
rs80297942	LC_IGP_R8	IgG1_G2FS1/IgG1_G2S1	14	66100698	1	2.13E-09	3.72E-11	+	0.209237	0.2718	+	A	G	0.457348503	0.0347496	+	A	G	0.4329	0.0411
rs80297942	LC_IGP_R26	IgG1_G2N/IgG1_G1N	14	66100698	1	7.51E-12	7.34E-08	+	0.239619	0.2166	+	A	G	0.458847531	0.0347344	+	A	G	0.4329	0.0402
rs80297942	LC_IGP148	IgG2 FG0n total/G0n	14	66100698	1	3.54E-09	2.45E-13	+	0.206909	0.3008	+	A	G	0.456506466	0.0348489	+	A	G	0.4328	0.0411
rs80297942	LC_IGP97	IgG2_G0	14	66100698	1	2.73E-11	2.67E-13	-	-0.231924	-0.3002	+	A	G	0.457627445	0.0345787	+	A	G	0.4329	0.0411
rs80297942	LC_IGP_R41	IgG2_G0F/IgG2_G0	14	66100698	1	2.70E-08	1.34E-12	+	0.193919	0.2898	+	A	G	0.457627445	0.0347097	+	A	G	0.4329	0.0409
rs80297942	LC_IGP138	IgG2_G0n	14	66100698	1	1.64E-11	2.03E-13	-	-0.235412	-0.3022	+	A	G	0.456506466	0.0347039	+	A	G	0.4328	0.0411
rs80297942	LC_IGP_R64	IgG2_G2/IgG2_G1	14	66100698	1	3.78E-09	2.67E-12	+	0.206755	0.282	+	A	G	0.456758568	0.0348901	+	A	G	0.4329	0.0403
rs9796385	LC_IGP78	IgG1 BG0n/G0n	14	66101219	1	1.23E-11	5.80E-09	-	-0.238732	-0.2322	+	G	A	0.448670662	0.0349728	+	G	A	0.4587	0.0399
rs9796385	LC_IGP79	IgG1 BG1n/G1n	14	66101219	1	1.05E-11	4.50E-11	-	-0.240077	-0.2622	+	G	A	0.448670662	0.0350507	+	G	A	0.4587	0.0398
rs9796385	LC_IGP77	IgG1 Bn	14	66101219	1	3.69E-11	1.37E-10	-	-0.23374	-0.2568	+	G	A	0.448670662	0.0350845	+	G	A	0.4587	0.04
rs9796385	LC_IGP86	IgG1 Bn/Fn total	14	66101219	1	5.73E-12	7.91E-11	-	-0.2431	-0.2603	+	G	A	0.448670662	0.0350372	+	G	A	0.4587	0.04
rs9796385	LC_IGP84	IgG1 FBn/Bn total	14	66101219	1	1.13E-12	6.63E-15	+	0.251511	0.3137	+	G	A	0.448670662	0.0350737	+	G	A	0.4587	0.0403
rs9796385	LC_IGP62	IgG1 FG0n total/G0n	14	66101219	1	1.44E-15	3.18E-11	+	0.280079	0.2669	+	G	A	0.448670662	0.0347388	+	G	A	0.4587	0.0402
rs9796385	LC_IGP63	IgG1 FG1n total/G1n	14	66101219	1	3.71E-16	1.71E-13	+	0.286631	0.2956	+	G	A	0.448670662	0.0348123	+	G	A	0.4587	0.0401
rs9796385	LC_IGP64	IgG1 FG2n total/G2n	14	66101219	1	1.46E-12	3.05E-14	+	0.2499	0.3026	+	G	A	0.448670662	0.035025	+	G	A	0.4587	0.0398
rs9796385	LC_IGP68	IgG1 FG2n/G2n	14	66101219	1	1.19E-09	6.30E-08	+	0.215092	0.2153	+	G	A	0.448670662	0.0351563	+	G	A	0.4587	0.0398
rs9796385	LC_IGP61	IgG1 Fn total	14	66101219	1	3.77E-15	5.04E-13	+	0.27659	0.2902	+	G	A	0.448670662	0.0348372	+	G	A	0.4587	0.0402
rs9796385	LC_IGP81	IgG1 Fn/Bn	14	66101219	1	1.36E-11	7.97E-10	+	0.238699	0.2449	+	G	A	0.448670662	0.035044	+	G	A	0.4587	0.0399
rs9796385	LC_IGP21	IgG1 Fucosylation	14	66101219	1	2.45E-13	3.84E-12	+	0.257334	0.2784	+	G	A	0.447561652	0.0348517	+	G	A	0.4587	0.0401
rs9796385	LC_IGP28	IgG1 G1S1/(G1+G1S1)	14	66101219	1	9.94E-11	4.60E-09	+	0.226122	0.224	+	G	A	0.447987462	0.0347318	+	G	A	0.4587	0.0382
rs9796385	LC_IGP26	IgG1 GS1/(G+GS1)	14	66101219	1	2.56E-08	9.22E-09	+	0.19548	0.226	+	G	A	0.447987462	0.03493	+	G	A	0.4587	0.0394
rs9796385	LC_IGP27	IgG1 GS1/(G0+G+GS1)	14	66101219	1	9.79E-10	1.31E-07	+	0.214113	0.2065	+	G	A	0.447987462	0.0348215	+	G	A	0.4587	0.0391
rs9796385	LC_IGP11	IgG1_G0	14	66101219	1	4.91E-18	4.03E-13	-	-0.300925	-0.2899	+	G	A	0.448188149	0.0343643	+	G	A	0.4587	0.04
rs9796385	LC_IGP_R1	IgG1_G0F/IgG1_G0	14	66101219	1	1.49E-15	7.36E-11	+	0.277703	0.2612	+	G	A	0.448188149	0.0344676	+	G	A	0.4587	0.0401
rs9796385	LC_IGP_R4	IgG1_G0FN/IgG1_G0N	14	66101219	1	4.21E-12	5.66E-12	+	0.243096	0.2774	+	G	A	0.448659829	0.0348152	+	G	A	0.4587	0.0403
rs9796385	LC_IGP52	IgG1_G0n	14	66101219	1	9.17E-18	3.95E-13	-	-0.300532	-0.2906	+	G	A	0.448670662	0.0346065	+	G	A	0.4587	0.04
rs9796385	LC_IGP14	IgG1_G0N	14	66101219	1	1.66E-14	3.13E-10	-	-0.268707	-0.2499	+	G	A	0.448659829	0.0346906	+	G	A	0.4587	0.0397

rs9796385	LC_IGP55	IgG1_G0Nn	14	66101219	1	2.02E-14	3.44E-10	-	-0.269195	-0.2496	+	G	A	0.448670662	0.0348668	+	G	A	0.4587	0.0398
rs9796385	LC_IGP12	IgG1_G1	14	66101219	1	4.18E-15	1.04E-12	-	-0.27394	-0.2859	+	G	A	0.448198534	0.0345654	+	G	A	0.4587	0.0401
rs9796385	LC_IGP_R2	IgG1_G1F/IgG1_G1	14	66101219	1	1.69E-16	1.26E-12	+	0.287486	0.2844	+	G	A	0.448198534	0.0345128	+	G	A	0.4587	0.0401
rs9796385	LC_IGP_R5	IgG1_G1FN/IgG1_G1N	14	66101219	1	1.28E-12	4.16E-16	+	0.249688	0.3254	+	G	A	0.449512608	0.0349081	+	G	A	0.4587	0.04
rs9796385	LC_IGP53	IgG1_G1n	14	66101219	1	4.96E-14	1.92E-12	-	-0.265194	-0.2822	+	G	A	0.448670662	0.0348893	+	G	A	0.4587	0.0401
rs9796385	LC_IGP15	IgG1_G1N	14	66101219	1	3.17E-10	4.56E-10	-	-0.221577	-0.2484	+	G	A	0.449512608	0.035006	+	G	A	0.4587	0.0398
rs9796385	LC_IGP56	IgG1_G1Nn	14	66101219	1	2.35E-10	9.93E-10	-	-0.223983	-0.2431	+	G	A	0.448670662	0.0351233	+	G	A	0.4587	0.0398
rs9796385	LC_IGP_R32	IgG1_G1S1/IgG1_G1	14	66101219	1	2.04E-10	6.33E-09	+	0.22238	0.2222	+	G	A	0.447987462	0.0347519	+	G	A	0.4587	0.0383
rs9796385	LC_IGP_R3	IgG1_G2F/IgG1_G2	14	66101219	1	3.88E-14	9.92E-14	+	0.264459	0.2958	+	G	A	0.448001528	0.0346471	+	G	A	0.4587	0.0398
rs9796385	LC_IGP_R8	IgG1_G2FS1/IgG1_G2S1	14	66101219	1	7.41E-09	3.19E-12	+	0.202791	0.2766	+	G	A	0.447727856	0.0348929	+	G	A	0.4587	0.0397
rs9796385	LC_IGP_R26	IgG1_G2N/IgG1_G1N	14	66101219	1	2.80E-11	7.68E-08	+	0.233658	0.2086	+	G	A	0.449445648	0.0348547	+	G	A	0.4587	0.0388
rs9796385	LC_IGP97	IgG2_G0	14	66101219	1	1.87E-09	1.41E-12	-	-0.210216	-0.2813	+	G	A	0.447987469	0.0347847	+	G	A	0.4587	0.0397
rs9796385	LC_IGP138	IgG2_G0n	14	66101219	1	1.60E-09	1.16E-12	-	-0.21194	-0.2827	+	G	A	0.446776478	0.0349234	+	G	A	0.4587	0.0398
rs9796385	LC_IGP_R64	IgG2_G2/IgG2_G1	14	66101219	1	1.05E-08	5.07E-12	+	0.201513	0.2678	+	G	A	0.447042228	0.0350342	+	G	A	0.4587	0.0388
rs145772892	LC_IGP61	IgG1 Fn total	14	66102816	1	3.62E-08	1.21E-06	-	-0.239866	-0.2486	+	T	C	0.200535502	0.043333	+	T	C	0.1656	0.0512
rs12896663	LC_IGP78	IgG1 BG0n/G0n	14	66104969	1	2.96E-14	9.55E-10	-	-0.261721	-0.2296	+	A	G	0.477276952	0.0341229	+	A	G	0.4947	0.0375
rs12896663	LC_IGP79	IgG1 BG1n/G1n	14	66104969	1	2.27E-13	9.29E-12	-	-0.253219	-0.2555	+	A	G	0.477276952	0.0342448	+	A	G	0.4947	0.0375
rs12896663	LC_IGP32	IgG1 BG1S1/(BG1+BG1S1)	14	66104969	1	1.90E-08	3.67E-12	+	0.192821	0.255	+	A	G	0.478315043	0.0341306	+	A	G	0.4947	0.0367
rs12896663	LC_IGP77	IgG1 Bn	14	66104969	1	2.74E-13	2.45E-11	-	-0.252423	-0.2514	+	A	G	0.477276952	0.0342556	+	A	G	0.4947	0.0377
rs12896663	LC_IGP86	IgG1 Bn/Fn total	14	66104969	1	3.78E-14	1.46E-11	-	-0.261248	-0.2544	+	A	G	0.477276952	0.0342068	+	A	G	0.4947	0.0377
rs12896663	LC_IGP84	IgG1 FBn/Bn total	14	66104969	1	1.92E-15	4.33E-16	+	0.274605	0.3086	+	A	G	0.477276952	0.0342139	+	A	G	0.4947	0.038
rs12896663	LC_IGP62	IgG1 FG0n total/G0n	14	66104969	1	9.19E-18	2.96E-12	+	0.294515	0.2637	+	A	G	0.477276952	0.0339143	+	A	G	0.4947	0.0378
rs12896663	LC_IGP63	IgG1 FG1n total/G1n	14	66104969	1	6.55E-18	1.69E-14	+	0.296686	0.2895	+	A	G	0.477276952	0.0340072	+	A	G	0.4947	0.0377
rs12896663	LC_IGP64	IgG1 FG2n total/G2n	14	66104969	1	4.10E-14	2.89E-15	+	0.261016	0.2963	+	A	G	0.477276952	0.034225	+	A	G	0.4947	0.0375
rs12896663	LC_IGP68	IgG1 FG2n/G2n	14	66104969	1	1.36E-10	1.79E-08	+	0.222204	0.2104	+	A	G	0.477276952	0.0343831	+	A	G	0.4947	0.0374
rs12896663	LC_IGP61	IgG1 Fn total	14	66104969	1	4.67E-17	4.07E-14	+	0.288818	0.2856	+	A	G	0.477276952	0.0340243	+	A	G	0.4947	0.0378
rs12896663	LC_IGP81	IgG1 Fn/Bn	14	66104969	1	1.35E-13	1.24E-10	+	0.255491	0.2413	+	A	G	0.477276952	0.034222	+	A	G	0.4947	0.0375
rs12896663	LC_IGP21	IgG1 Fucosylation	14	66104969	1	1.85E-15	5.75E-13	+	0.27304	0.2718	+	A	G	0.476464242	0.034	+	A	G	0.4947	0.0377
rs12896663	LC_IGP28	IgG1 G1S1/(G1+G1S1)	14	66104969	1	2.45E-10	2.48E-10	+	0.216654	0.2289	+	A	G	0.476652599	0.0340121	+	A	G	0.4947	0.0362
rs12896663	LC_IGP27	IgG1 GS1/(G0+G+GS1)	14	66104969	1	2.78E-09	3.21E-08	+	0.203848	0.2036	+	A	G	0.476652599	0.0341028	+	A	G	0.4947	0.0368
rs12896663	LC_IGP11	IgG1_G0	14	66104969	1	2.40E-19	3.48E-14	-	-0.305624	-0.2846	+	A	G	0.476986561	0.0335588	+	A	G	0.4947	0.0376
rs12896663	LC_IGP_R1	IgG1_G0F/IgG1_G0	14	66104969	1	9.07E-17	6.48E-12	+	0.282993	0.2587	+	A	G	0.476986561	0.0336642	+	A	G	0.4947	0.0377
rs12896663	LC_IGP_R4	IgG1_G0FN/IgG1_G0N	14	66104969	1	5.76E-15	3.92E-13	+	0.26746	0.2752	+	A	G	0.477541335	0.0339248	+	A	G	0.4947	0.0379
rs12896663	LC_IGP52	IgG1_G0n	14	66104969	1	2.11E-19	3.01E-14	-	-0.308481	-0.2859	+	A	G	0.477276952	0.0338115	+	A	G	0.4947	0.0376
rs12896663	LC_IGP14	IgG1_G0N	14	66104969	1	4.85E-17	5.15E-11	-	-0.286879	-0.2456	+	A	G	0.477541335	0.0338186	+	A	G	0.4947	0.0374
rs12896663	LC_IGP55	IgG1_G0Nn	14	66104969	1	2.15E-17	5.28E-11	-	-0.291817	-0.2457	+	A	G	0.477276952	0.0340021	+	A	G	0.4947	0.0374
rs12896663	LC_IGP12	IgG1_G1	14	66104969	1	2.28E-16	1.09E-13	-	-0.279945	-0.2804	+	A	G	0.476986561	0.0337577	+	A	G	0.4947	0.0377
rs12896663	LC_IGP_R2	IgG1_G1F/IgG1_G1	14	66104969	1	1.46E-17	1.34E-13	+	0.290881	0.2787	+	A	G	0.476986561	0.0337155	+	A	G	0.4947	0.0377
rs12896663	LC_IGP_R5	IgG1_G1FN/IgG1_G1N	14	66104969	1	1.24E-14	2.26E-17	+	0.265271	0.3201	+	A	G	0.478368389	0.0340763	+	A	G	0.4947	0.0378
rs12896663	LC_IGP53	IgG1_G1n	14	66104969	1	1.69E-15	1.88E-13	-	-0.274208	-0.2775	+	A	G	0.477276952	0.0340963	+	A	G	0.4947	0.0377
rs12896663	LC_IGP15	IgG1_G1N	14	66104969	1	1.38E-11	1.13E-10	-	-0.2329	-0.2419	+	A	G	0.478368389	0.0342033	+	A	G	0.4947	0.0375
rs12896663	LC_IGP56	IgG1_G1Nn	14	66104969	1	5.57E-12	2.28E-10	-	-0.238244	-0.2376	+	A	G	0.477276952	0.0343178	+	A	G	0.4947	0.0375
rs12896663	LC_IGP_R36	IgG1_G1NS1/IgG1_G1N	14	66104969	1	1.69E-08	4.21E-12	+	0.193549	0.2545	+	A	G	0.478315043	0.0341363	+	A	G	0.4947	0.0367
rs12896663	LC_IGP_R32	IgG1_G1S1/IgG1_G1	14	66104969	1	4.38E-10	3.75E-10	+	0.213636	0.2269	+	A	G	0.476652599	0.034029	+	A	G	0.4947	0.0362
rs12896663	LC_IGP_R3	IgG1_G2F/IgG1_G2	14	66104969	1	1.37E-15	8.72E-15	+	0.273053	0.2905	+	A	G	0.47666687	0.0338426	+	A	G	0.4947	0.0375
rs12896663	LC_IGP_R8	IgG1_G2FS1/IgG1_G2S1	14	66104969	1	3.42E-10	1.07E-12	+	0.215348	0.266	+	A	G	0.476375687	0.0340874	+	A	G	0.4947	0.0374
rs12896663	LC_IGP_R26	IgG1_G2N/IgG1_G1N	14	66104969	1	2.67E-12	1.37E-08	+	0.240289	0.2079	+	A	G	0.47797963	0.0340914	+	A	G	0.4947	0.0366
rs12896663	LC_IGP148	IgG2 FG0n total/G0n	14	66104969	1	3.52E-09	1.68E-13	+	0.203188	0.2754	+	A	G	0.475570813	0.0342171	+	A	G	0.4947	0.0374
rs12896663	LC_IGP97	IgG2_G0	14	66104969	1	1.07E-10	1.26E-13	-	-0.220878	-0.2771	+	A	G	0.47666687	0.0339854	+	A	G	0.4947	0.0374
rs12896663	LC_IGP_R41	IgG2_G0F/IgG2_G0	14	66104969	1	3.17E-08	7.45E-13	+	0.189491	0.2665	+	A	G	0.47666687	0.0340894	+	A	G	0.4947	0.0372
rs12896663	LC_IGP138	IgG2_G0n	14	66104969	1	6.24E-11	8.83E-14	-	-0.224469	-0.2793	+	A	G	0.475570813	0.0341023	+	A	G	0.4947	0.0374
rs12896663	LC_IGP_R64	IgG2_G2/IgG2_G1	14	66104969	1	6.54E-09	2.33E-12	+	0.199929	0.2567	+	A	G	0.475720318	0.0342744	+	A	G	0.4947	0.0366
rs11624045	LC_IGP78	IgG1 BG0n/G0n	14	66108018	1	2.96E-14	9.57E-10	-	-0.261722	-0.2295	+	T	C	0.477276022	0.0341228	+	T	C	0.4948	0.0375
rs11624045	LC_IGP79	IgG1 BG1n/G1n	14	66108018	1	2.27E-13	9.34E-12	-	-0.253218	-0.2555	+	T	C	0.477276022	0.0342447	+	T	C	0.4948	0.0375
rs11624045	LC_IGP32	IgG1 BG1S1/(BG1+BG1S1)	14	66108018	1	1.90E-08	3.67E-12	+	0.192821	0.255	+	T	C	0.478314118	0.0341305	+	T	C	0.4948	0.0367
rs11624045	LC_IGP77	IgG1 Bn	14	66108018	1	2.74E-13	2.45E-11	-	-0.252422	-0.2514	+	T	C	0.477276022	0.0342554	+	T	C	0.4948	0.0377
rs11624045	LC_IGP86	IgG1 Bn/Fn total	14	66108018	1	3.78E-14	1.46E-11	-	-0.261248	-0.2544	+	T	C	0.477276022	0.0342067	+	T	C	0.4948	0.0377
rs11624045	LC_IGP84	IgG1 FBn/Bn total	14	66108018	1	1.92E-15	4.24E-16	+	0.274605	0.3087	+	T	C	0.477276022	0.0342138	+	T	C	0.4948	0.038
rs11624045	LC_IGP62	IgG1 FG0n total/G0n	14	66108018	1	9.18E-18	2.93E-12	+	0.294517	0.2637	+	T	C	0.477276022	0.0339141	+	T	C	0.4948	0.0378
rs11624045	LC_IGP63	IgG1 FG1n total/G1n	14	66108018	1	6.55E-18	1.68E-14	+	0.296686	0.2895	+	T	C	0.477276022	0.0340071	+	T	C	0.4948	0.0377
rs11624045	LC_IGP64	IgG1 FG2n total/G2n	14	66108018	1	4.10E-14	2.83E-15	+	0.261015	0.2964	+	T	C	0.477276022	0.0342249	+	T	C	0.4948	0.0375
rs11624045	LC_IGP68	IgG1 FG2n/G2n	14	66108018	1	1.36E-10	1.78E-08	+	0.222203	0.2104	+	T	C	0.477276022	0.034383	+	T	C	0.4948	0.0374
rs11624045	LC_IGP61	IgG1 Fn total	14	66108018	1	4.67E-17	4.02E-14	+	0.288819	0.2856	+	T	C	0.477276022	0.0340241	+	T	C	0.4948	0.0378
rs11624045	LC_IGP81	IgG1 Fn/Bn	14	66108018	1	1.35E-13	1.24E-10	+	0.255491	0.2413	+	T	C	0.477276022	0.0342219	+	T	C	0.4948	0.0375
rs11624045	LC_IGP21	IgG1 Fucosylation	14	66108018	1	1.85E-15	5.68E-13	+	0.273041	0.2718	+	T	C	0.476463317	0.0339999	+	T	C	0.4948	0.0377
rs11624045	LC_IGP28	IgG1 G1S1/(G1+G1S1)	14	66108018	1	2.45E-10	2.49E-10	+	0.216653	0.2289	+	T	C	0.476651682	0.034012	+	T	C	0.4948	0.0362
rs11624045	LC_IGP27	IgG1 GS1/(G0+G+GS1)	14	66108018	1	2.78E-09	3.17E-08	+	0.203843	0.2037	+	T	C	0.476651682	0.0341027	+	T	C	0.4948	0.0368
rs11624045	LC_IGP11	IgG1_G0	14	66108018	1	2.40E-19	3.41E-14	-	-0.305623	-0.2847	+	T	C	0.476985644	0.0335586	+	T	C	0.4948	0.0376
rs11624045	LC_IGP_R1	IgG1_G0F/IgG1_G0	14	66108018	1	9.07E-17	6.43E-12	+	0.282995	0.2587	+	T	C	0.476985644	0.0336641	+	T	C	0.4948	0.0377
rs11624045	LC_IGP_R4	IgG1_G0FN/IgG1_G0N	14	66108018	1	5.76E-15	3.85E-13	+	0.267462	0.2753	+	T	C	0.477540416	0.0339247	+	T	C	0.4948	0.0379
rs11624045	LC_IGP52	IgG1_G0n	14	66108018	1	2.11E-19	2.96E-14	-	-0.308481	-0.286	+	T	C	0.477276022	0.0338114	+	T	C	0.4948	0.0376
rs11624045	LC_IGP14	IgG1_G0N	14	66108018	1	4.85E-17	5.13E-11	-	-0.286878	-0.2456	+	T	C	0.477540416	0.0338185	+	T	C	0.4948	0.0374
rs11624045	LC_IGP55	IgG1_G0Nn	14	66108018	1	2.15E-17	5.26E-11	-	-0.291816	-0.2457	+	T	C	0.477276022	0.034002	+	T	C	0.4948	0.0374
rs11624045	LC_IGP12	IgG1_G1	14	66108018	1	2.28E-16	1.09E-13	-	-0.279946	-0.2804	+	T	C	0.476985644	0.0337576	+	T	C	0.4948	0.0377
rs11624045	LC_IGP_R2	IgG1_G1F/IgG1_G1	14	66108018	1	1.46E-17	1.33E-13	+	0.290881	0.2787	+	T	C	0.476985644	0.0337154	+	T	C	0.4948	0.0377
rs11624045	LC_IGP_R5	IgG1_G1FN/IgG1_G1N	14	66108018	1	1.24E-14	2.22E-17	+	0.265271	0.3202	+	T	C	0.478367466	0.0340762	+	T	C	0.4948	0.0378
rs11624045	LC_IGP53	IgG1_G1n	14	66108018	1	1.69E-15	1.87E-13	-	-0.274209	-0.2775	+	T	C	0.477276022	0.0340962	+	T	C	0.4948	0.0377
rs11624045	LC_IGP15	IgG1_G1N	14	66108018	1	1.38E-11	1.14E-10	-	-0.2329	-0.2419	+	T	C	0.478367466	0.0342032	+	T	C	0.4948	0.0375
rs11624045	LC_IGP56	IgG1_G1Nn	14	66108018	1	5.57E-12	2.30E-10	-	-0.238244	-0.2376	+	T	C	0.477276022	0.0343177	+	T	C	0.4948	0.0375
rs11624045	LC_IGP_R36	IgG1_G1NS1/IgG1_G1N	14	66108018	1	1.69E-08	4.21E-12	+	0.193549	0.2545	+	T	C	0.478314118	0.0341362	+	T	C	0.4948	0.0367
rs11624045	LC_IGP_R32	IgG1_G1S1/IgG1_G1	14	66108018	1	4.38E-10	3.78E-10	+	0.213635	0.2268	+	T	C	0.476651682	0.0340289	+	T	C	0.4948	0.0362
rs11624045	LC_IGP_R3	IgG1_G2F/IgG1_G2	14	66108018	1	1.37E-15	8.55E-15	+	0.273052	0.2906	+	T	C	0.476665954	0.0338425	+	T	C	0.4948	0.0374
rs11624045	LC_IGP_R8	IgG1_G2FS1/IgG1_G2S1	14	66108018	1	3.42E-10	1.06E-12	+	0.21535	0.266	+	T	C	0.476374771	0.0340873	+	T	C	0.4948	0.0374
rs11624045	LC_IGP_R26	IgG1_G2N/IgG1_G1N	14	66108018	1	2.67E-12	1.39E-08	+	0.24029	0.2078	+	T	C	0.477978704	0.0340913	+	T	C	0.4948	0.0366
rs11624045	LC_IGP148	IgG2 FG0n total/G0n	14	66108018	1	3.52E-09	1.74E-13	+	0.203192	0.2752	+	T	C	0.475569889	0.0342169	+	T	C	0.4948	0.0374
rs11624045	LC_IGP97	IgG2_G0	14	66108018	1	1.07E-10	1.29E-13	-	-0.22088	-0.277	+	T	C	0.476665954	0.0339852	+	T	C	0.4948	0.0374
rs11624045	LC_IGP_R41	IgG2_G0F/IgG2_G0	14	66108018	1	3.17E-08	7.70E-13	+	0.189495	0.2663	+	T	C	0.476665954	0.0340893	+	T	C	0.4948	0.0372
rs11624045	LC_IGP138	IgG2_G0n	14	66108018	1	6.24E-11	9.05E-14	-	-0.224471	-0.2792	+	T	C	0.475569889	0.0341022	+	T	C	0.4948	0.0374
rs11624045	LC_IGP_R64	IgG2_G2/IgG2_G1	14	66108018	1	6.54E-09	2.35E-12	+	0.199927	0.2566	+	T	C	0.4757194	0.0342743	+	T	C	0.4948	0.0366
rs61348705	LC_IGP78	IgG1 BG0n/G0n	14	66111019	1	2.98E-09	3.37E-07	+	0.246385	0.2564	+	A	T	0.23393809	0.0412988	+	A	T	0.1997	0.0503
rs61348705	LC_IGP79	IgG1 BG1n/G1n	14	66111019	1	6.22E-09	5.77E-09	+	0.241944	0.2919	+	A	T	0.23393809	0.0414132	+	A	T	0.1997	0.0501
rs61348705	LC_IGP77	IgG1 Bn	14	66111019	1	2.85E-09	2.87E-08	+	0.247326	0.2797	+	A	T	0.23393809	0.041402	+	A	T	0.1997	0.0504
rs61348705	LC_IGP86	IgG1 Bn/Fn total	14	66111019	1	4.81E-10	1.43E-08	+	0.258991	0.286	+	A	T	0.23393809	0.0413485	+	A	T	0.1997	0.0504
rs61348705	LC_IGP84	IgG1 FBn/Bn total	14	66111019	1	4.02E-11	2.86E-12	-	-0.275076	-0.3562	+	A	T	0.23393809	0.0413701	+	A	T	0.1997	0.051
rs61348705	LC_IGP62	IgG1 FG0n total/G0n	14	66111019	1	1.63E-13	1.66E-09	-	-0.305083	-0.3045	+	A	T	0.23393809	0.0410043	+	A	T	0.1997	0.0505
rs61348705	LC_IGP63	IgG1 FG1n total/G1n	14	66111019	1	4.14E-12	2.78E-11	-	-0.287847	-0.3358	+	A	T	0.23393809	0.0412063	+	A	T	0.1997	0.0504
rs61348705	LC_IGP64	IgG1 FG2n total/G2n	14	66111019	1	1.25E-10	1.74E-12	-	-0.267646	-0.3537	+	A	T	0.23393809	0.0413348	+	A	T	0.1997	0.0501
rs61348705	LC_IGP61	IgG1 Fn total	14	66111019	1	4.17E-13	4.59E-11	-	-0.300608	-0.3326	+	A	T	0.23393809	0.0411199	+	A	T	0.1997	0.0505
rs61348705	LC_IGP81	IgG1 Fn/Bn	14	66111019	1	1.67E-09	5.15E-08	-	-0.250781	-0.2729	+	A	T	0.23393809	0.0413658	+	A	T	0.1997	0.0501
rs61348705	LC_IGP21	IgG1 Fucosylation	14	66111019	1	8.69E-12	7.26E-11	-	-0.282748	-0.3288	+	A	T	0.234627667	0.0411142	+	A	T	0.1997	0.0505
rs61348705	LC_IGP11	IgG1_G0	14	66111019	1	2.52E-14	2.53E-10	+	0.312832	0.3174	+	A	T	0.234005545	0.0406804	+	A	T	0.1997	0.0502
rs61348705	LC_IGP_R1	IgG1_G0F/IgG1_G0	14	66111019	1	1.59E-13	4.01E-09	-	-0.30297	-0.2959	+	A	T	0.234005545	0.0407072	+	A	T	0.1997	0.0503
rs61348705	LC_IGP_R4	IgG1_G0FN/IgG1_G0N	14	66111019	1	2.71E-10	2.54E-10	-	-0.26127	-0.3216	+	A	T	0.233967897	0.041116	+	A	T	0.1997	0.0509
rs61348705	LC_IGP52	IgG1_G0n	14	66111019	1	1.78E-14	2.52E-10	+	0.316605	0.3182	+	A	T	0.23393809	0.0409194	+	A	T	0.1997	0.0503
rs61348705	LC_IGP14	IgG1_G0N	14	66111019	1	3.76E-10	2.00E-07	+	0.259116	0.2609	+	A	T	0.233967897	0.0411139	+	A	T	0.1997	0.0502
rs61348705	LC_IGP55	IgG1_G0Nn	14	66111019	1	3.60E-10	2.25E-07	+	0.260492	0.2602	+	A	T	0.23393809	0.0412819	+	A	T	0.1997	0.0503
rs61348705	LC_IGP12	IgG1_G1	14	66111019	1	2.40E-12	2.20E-11	+	0.288639	0.3372	+	A	T	0.234006766	0.0408643	+	A	T	0.1997	0.0504
rs61348705	LC_IGP_R2	IgG1_G1F/IgG1_G1	14	66111019	1	9.68E-12	8.97E-11	-	-0.280713	-0.3258	+	A	T	0.234006766	0.0409153	+	A	T	0.1997	0.0503
rs61348705	LC_IGP_R5	IgG1_G1FN/IgG1_G1N	14	66111019	1	1.59E-10	2.53E-13	-	-0.265197	-0.3706	+	A	T	0.233744205	0.0411937	+	A	T	0.1997	0.0506
rs61348705	LC_IGP53	IgG1_G1n	14	66111019	1	5.23E-12	4.15E-11	+	0.286266	0.3324	+	A	T	0.23393809	0.04118	+	A	T	0.1997	0.0504
rs61348705	LC_IGP15	IgG1_G1N	14	66111019	1	9.99E-09	8.47E-09	+	0.237782	0.2886	+	A	T	0.233744205	0.0412747	+	A	T	0.1997	0.0501
rs61348705	LC_IGP56	IgG1_G1Nn	14	66111019	1	9.84E-09	2.00E-08	+	0.238807	0.281	+	A	T	0.23393809	0.0414322	+	A	T	0.1997	0.0501
rs61348705	LC_IGP_R3	IgG1_G2F/IgG1_G2	14	66111019	1	1.72E-11	1.37E-12	-	-0.277482	-0.3546	+	A	T	0.23414858	0.0409524	+	A	T	0.1997	0.05
rs61348705	LC_IGP148	IgG2 FG0n total/G0n	14	66111019	1	4.43E-08	5.84E-08	-	-0.226328	-0.2712	+	A	T	0.234346722	0.041158	+	A	T	0.1999	0.05
rs61348705	LC_IGP97	IgG2_G0	14	66111019	1	9.61E-10	2.94E-08	+	0.251896	0.2782	+	A	T	0.23414858	0.040946	+	A	T	0.1997	0.0502
rs61348705	LC_IGP_R41	IgG2_G0F/IgG2_G0	14	66111019	1	1.56E-08	1.79E-08	-	-0.233016	-0.2805	+	A	T	0.23414858	0.0410003	+	A	T	0.1997	0.0498
rs61348705	LC_IGP138	IgG2_G0n	14	66111019	1	4.56E-10	3.34E-08	+	0.257199	0.2773	+	A	T	0.234346722	0.0410068	+	A	T	0.1999	0.0502
rs12890902	LC_IGP78	IgG1 BG0n/G0n	14	66116622	1	2.95E-14	7.10E-10	-	-0.26173	-0.2296	+	A	T	0.477274164	0.0341224	+	A	T	0.4978	0.0372
rs12890902	LC_IGP79	IgG1 BG1n/G1n	14	66116622	1	2.27E-13	5.57E-12	-	-0.25322	-0.2563	+	A	T	0.477274164	0.0342443	+	A	T	0.4978	0.0372

rs12890902	LC_IGP32	IgG1 BG1S1/(BG1+BG1S1)	14	66116622	1	1.90E-08	2.63E-12	+	0.192822	0.2551	+	A	T	0.478312269	0.0341302	+	A	T	0.4978	0.0365
rs12890902	LC_IGP77	IgG1 Bn	14	66116622	1	2.73E-13	1.55E-11	-	-0.252425	-0.252	+	A	T	0.477274164	0.0342551	+	A	T	0.4978	0.0374
rs12890902	LC_IGP86	IgG1 Bn/Fn total	14	66116622	1	3.77E-14	9.25E-12	-	-0.261252	-0.2549	+	A	T	0.477274164	0.0342063	+	A	T	0.4978	0.0374
rs12890902	LC_IGP84	IgG1 FBn/Bn total	14	66116622	1	1.92E-15	2.36E-16	+	0.274608	0.309	+	A	T	0.477274164	0.0342134	+	A	T	0.4978	0.0377
rs12890902	LC_IGP62	IgG1 FG0n total/G0n	14	66116622	1	9.15E-18	2.17E-12	+	0.294528	0.2633	+	A	T	0.477274164	0.0339138	+	A	T	0.4978	0.0375
rs12890902	LC_IGP63	IgG1 FG1n total/G1n	14	66116622	1	6.54E-18	1.03E-14	+	0.29669	0.2897	+	A	T	0.477274164	0.0340068	+	A	T	0.4978	0.0374
rs12890902	LC_IGP64	IgG1 FG2n total/G2n	14	66116622	1	4.10E-14	1.47E-15	+	0.261016	0.2973	+	A	T	0.477274164	0.0342246	+	A	T	0.4978	0.0372
rs12890902	LC_IGP68	IgG1 FG2n/G2n	14	66116622	1	1.36E-10	1.15E-08	+	0.222204	0.2117	+	A	T	0.477274164	0.0343827	+	A	T	0.4978	0.0371
rs12890902	LC_IGP61	IgG1 Fn total	14	66116622	1	4.66E-17	2.58E-14	+	0.288827	0.2856	+	A	T	0.477274164	0.0340238	+	A	T	0.4978	0.0375
rs12890902	LC_IGP81	IgG1 Fn/Bn	14	66116622	1	1.35E-13	8.37E-11	+	0.255495	0.2417	+	A	T	0.477274164	0.0342215	+	A	T	0.4978	0.0372
rs12890902	LC_IGP21	IgG1 Fucosylation	14	66116622	1	1.84E-15	3.79E-13	+	0.273048	0.2719	+	A	T	0.476461467	0.0339996	+	A	T	0.4978	0.0374
rs12890902	LC_IGP28	IgG1 G1S1/(G1+G1S1)	14	66116622	1	2.45E-10	2.22E-10	+	0.216654	0.2283	+	A	T	0.476649847	0.0340117	+	A	T	0.4978	0.036
rs12890902	LC_IGP27	IgG1 GS1/(G0+G+GS1)	14	66116622	1	2.78E-09	2.65E-08	+	0.203834	0.2036	+	A	T	0.476649847	0.0341024	+	A	T	0.4978	0.0366
rs12890902	LC_IGP11	IgG1_G0	14	66116622	1	2.40E-19	2.29E-14	-	-0.305625	-0.2845	+	A	T	0.476983812	0.0335583	+	A	T	0.4978	0.0373
rs12890902	LC_IGP_R1	IgG1_G0F/IgG1_G0	14	66116622	1	9.04E-17	4.96E-12	+	0.283004	0.2581	+	A	T	0.476983812	0.0336637	+	A	T	0.4978	0.0374
rs12890902	LC_IGP_R4	IgG1_G0FN/IgG1_G0N	14	66116622	1	5.74E-15	2.59E-13	+	0.267469	0.2752	+	A	T	0.477538579	0.0339244	+	A	T	0.4978	0.0376
rs12890902	LC_IGP52	IgG1_G0n	14	66116622	1	2.10E-19	2.00E-14	-	-0.308484	-0.2857	+	A	T	0.477274164	0.033811	+	A	T	0.4978	0.0374
rs12890902	LC_IGP14	IgG1_G0N	14	66116622	1	4.85E-17	3.38E-11	-	-0.286879	-0.2461	+	A	T	0.477538579	0.0338182	+	A	T	0.4978	0.0371
rs12890902	LC_IGP55	IgG1_G0Nn	14	66116622	1	2.15E-17	3.51E-11	-	-0.291817	-0.2461	+	A	T	0.477274164	0.0340017	+	A	T	0.4978	0.0372
rs12890902	LC_IGP12	IgG1_G1	14	66116622	1	2.28E-16	7.20E-14	-	-0.279954	-0.2804	+	A	T	0.476983812	0.0337572	+	A	T	0.4978	0.0375
rs12890902	LC_IGP_R2	IgG1_G1F/IgG1_G1	14	66116622	1	1.46E-17	8.90E-14	+	0.290886	0.2786	+	A	T	0.476983812	0.0337151	+	A	T	0.4978	0.0374
rs12890902	LC_IGP_R5	IgG1_G1FN/IgG1_G1N	14	66116622	1	1.23E-14	1.11E-17	+	0.265272	0.3209	+	A	T	0.478365621	0.0340758	+	A	T	0.4978	0.0375
rs12890902	LC_IGP53	IgG1_G1n	14	66116622	1	1.69E-15	1.26E-13	-	-0.274218	-0.2774	+	A	T	0.477274164	0.0340959	+	A	T	0.4978	0.0374
rs12890902	LC_IGP15	IgG1_G1N	14	66116622	1	1.37E-11	7.08E-11	-	-0.232904	-0.2427	+	A	T	0.478365621	0.0342029	+	A	T	0.4978	0.0372
rs12890902	LC_IGP56	IgG1_G1Nn	14	66116622	1	5.56E-12	1.48E-10	-	-0.238249	-0.2383	+	A	T	0.477274164	0.0343174	+	A	T	0.4978	0.0372
rs12890902	LC_IGP_R36	IgG1_G1NS1/IgG1_G1N	14	66116622	1	1.69E-08	3.03E-12	+	0.19355	0.2546	+	A	T	0.478312269	0.0341359	+	A	T	0.4978	0.0365
rs12890902	LC_IGP_R32	IgG1_G1S1/IgG1_G1	14	66116622	1	4.38E-10	3.35E-10	+	0.213636	0.2263	+	A	T	0.476649847	0.0340286	+	A	T	0.4978	0.036
rs12890902	LC_IGP_R3	IgG1_G2F/IgG1_G2	14	66116622	1	1.37E-15	4.65E-15	+	0.273053	0.2914	+	A	T	0.47666412	0.0338421	+	A	T	0.4978	0.0372
rs12890902	LC_IGP_R8	IgG1_G2FS1/IgG1_G2S1	14	66116622	1	3.41E-10	6.12E-13	+	0.21536	0.267	+	A	T	0.476372938	0.0340869	+	A	T	0.4978	0.0371
rs12890902	LC_IGP_R26	IgG1_G2N/IgG1_G1N	14	66116622	1	2.67E-12	1.01E-08	+	0.240293	0.2082	+	A	T	0.477976852	0.0340909	+	A	T	0.4978	0.0363
rs12890902	LC_IGP148	IgG2 FG0n total/G0n	14	66116622	1	3.51E-09	1.29E-13	+	0.203204	0.2747	+	A	T	0.475568042	0.0342166	+	A	T	0.4978	0.0371
rs12890902	LC_IGP97	IgG2_G0	14	66116622	1	1.07E-10	8.58E-14	-	-0.220887	-0.2771	+	A	T	0.47666412	0.0339849	+	A	T	0.4978	0.0371
rs12890902	LC_IGP_R41	IgG2_G0F/IgG2_G0	14	66116622	1	3.16E-08	6.02E-13	+	0.189506	0.2656	+	A	T	0.47666412	0.0340889	+	A	T	0.4978	0.0369
rs12890902	LC_IGP138	IgG2_G0n	14	66116622	1	6.22E-11	5.96E-14	-	-0.224479	-0.2792	+	A	T	0.475568042	0.0341018	+	A	T	0.4978	0.0372
rs12890902	LC_IGP_R64	IgG2_G2/IgG2_G1	14	66116622	1	6.54E-09	1.91E-12	+	0.19993	0.2562	+	A	T	0.475717564	0.034274	+	A	T	0.4978	0.0364
rs60708728	LC_IGP61	IgG1 Fn total	14	66116790	1	3.61E-08	1.60E-06	-	-0.239895	-0.2512	+	A	G	0.200537857	0.0433332	+	A	G	0.1699	0.0524
rs2300865	LC_IGP78	IgG1 BG0n/G0n	14	66120015	1	2.81E-14	1.01E-09	-	-0.262179	-0.2279	+	C	G	0.47453005	0.0341532	+	C	G	0.4967	0.0373
rs2300865	LC_IGP79	IgG1 BG1n/G1n	14	66120015	1	3.61E-13	7.82E-12	-	-0.251336	-0.255	+	C	G	0.47453005	0.0342859	+	C	G	0.4967	0.0373
rs2300865	LC_IGP32	IgG1 BG1S1/(BG1+BG1S1)	14	66120015	1	3.99E-08	4.12E-12	+	0.188581	0.2533	+	C	G	0.475580456	0.0341761	+	C	G	0.4967	0.0365
rs2300865	LC_IGP77	IgG1 Bn	14	66120015	1	3.04E-13	2.18E-11	-	-0.25217	-0.2507	+	C	G	0.47453005	0.0342892	+	C	G	0.4967	0.0374
rs2300865	LC_IGP86	IgG1 Bn/Fn total	14	66120015	1	4.19E-14	1.31E-11	-	-0.261036	-0.2536	+	C	G	0.47453005	0.0342403	+	C	G	0.4967	0.0375
rs2300865	LC_IGP84	IgG1 FBn/Bn total	14	66120015	1	2.73E-15	3.65E-16	+	0.273378	0.3076	+	C	G	0.47453005	0.0342526	+	C	G	0.4967	0.0378
rs2300865	LC_IGP62	IgG1 FG0n total/G0n	14	66120015	1	7.81E-18	2.95E-12	+	0.29541	0.2622	+	C	G	0.47453005	0.033942	+	C	G	0.4967	0.0376
rs2300865	LC_IGP63	IgG1 FG1n total/G1n	14	66120015	1	8.01E-18	1.44E-14	+	0.296184	0.2887	+	C	G	0.47453005	0.0340426	+	C	G	0.4967	0.0375
rs2300865	LC_IGP64	IgG1 FG2n total/G2n	14	66120015	1	4.81E-14	2.08E-15	+	0.260549	0.2963	+	C	G	0.47453005	0.0342598	+	C	G	0.4967	0.0373
rs2300865	LC_IGP68	IgG1 FG2n/G2n	14	66120015	1	1.50E-10	1.40E-08	+	0.221907	0.2109	+	C	G	0.47453005	0.0344167	+	C	G	0.4967	0.0372
rs2300865	LC_IGP61	IgG1 Fn total	14	66120015	1	4.90E-17	3.60E-14	+	0.288894	0.2846	+	C	G	0.47453005	0.0340565	+	C	G	0.4967	0.0376
rs2300865	LC_IGP81	IgG1 Fn/Bn	14	66120015	1	1.44E-13	1.14E-10	+	0.25542	0.2404	+	C	G	0.47453005	0.0342548	+	C	G	0.4967	0.0373
rs2300865	LC_IGP21	IgG1 Fucosylation	14	66120015	1	1.70E-15	5.15E-13	+	0.27364	0.2708	+	C	G	0.473730888	0.0340299	+	C	G	0.4967	0.0375
rs2300865	LC_IGP28	IgG1 G1S1/(G1+G1S1)	14	66120015	1	2.69E-10	2.65E-10	+	0.216378	0.2277	+	C	G	0.473939755	0.034045	+	C	G	0.4967	0.036
rs2300865	LC_IGP27	IgG1 GS1/(G0+G+GS1)	14	66120015	1	2.15E-09	2.89E-08	+	0.20546	0.2034	+	C	G	0.473939755	0.0341286	+	C	G	0.4967	0.0367
rs2300865	LC_IGP11	IgG1_G0	14	66120015	1	1.40E-19	2.85E-14	-	-0.307855	-0.2841	+	C	G	0.474277031	0.0335782	+	C	G	0.4967	0.0374
rs2300865	LC_IGP_R1	IgG1_G0F/IgG1_G0	14	66120015	1	7.43E-17	6.48E-12	+	0.284039	0.2572	+	C	G	0.474277031	0.0336906	+	C	G	0.4967	0.0374
rs2300865	LC_IGP_R4	IgG1_G0FN/IgG1_G0N	14	66120015	1	7.49E-15	3.99E-13	+	0.266583	0.2735	+	C	G	0.474826393	0.0339608	+	C	G	0.4967	0.0377
rs2300865	LC_IGP52	IgG1_G0n	14	66120015	1	1.20E-19	2.54E-14	-	-0.310811	-0.2852	+	C	G	0.47453005	0.0338309	+	C	G	0.4967	0.0374
rs2300865	LC_IGP14	IgG1_G0N	14	66120015	1	3.58E-17	4.55E-11	-	-0.288334	-0.2449	+	C	G	0.474826393	0.0338429	+	C	G	0.4967	0.0372
rs2300865	LC_IGP55	IgG1_G0Nn	14	66120015	1	1.50E-17	4.82E-11	-	-0.293486	-0.2448	+	C	G	0.47453005	0.0340258	+	C	G	0.4967	0.0372
rs2300865	LC_IGP12	IgG1_G1	14	66120015	1	3.01E-16	9.93E-14	-	-0.279082	-0.2793	+	C	G	0.474277031	0.0337939	+	C	G	0.4967	0.0375
rs2300865	LC_IGP_R2	IgG1_G1F/IgG1_G1	14	66120015	1	1.56E-17	1.19E-13	+	0.290888	0.2778	+	C	G	0.474277031	0.0337474	+	C	G	0.4967	0.0374
rs2300865	LC_IGP_R5	IgG1_G1FN/IgG1_G1N	14	66120015	1	2.49E-14	1.71E-17	+	0.262475	0.3196	+	C	G	0.475640529	0.0341208	+	C	G	0.4967	0.0376
rs2300865	LC_IGP53	IgG1_G1n	14	66120015	1	2.12E-15	1.75E-13	-	-0.273513	-0.2763	+	C	G	0.47453005	0.0341324	+	C	G	0.4967	0.0375
rs2300865	LC_IGP15	IgG1_G1N	14	66120015	1	2.36E-11	9.82E-11	-	-0.230443	-0.2414	+	C	G	0.475640529	0.0342446	+	C	G	0.4967	0.0373
rs2300865	LC_IGP56	IgG1_G1Nn	14	66120015	1	9.47E-12	2.04E-10	-	-0.235876	-0.2369	+	C	G	0.47453005	0.0343604	+	C	G	0.4967	0.0373
rs2300865	LC_IGP_R36	IgG1_G1NS1/IgG1_G1N	14	66120015	1	3.63E-08	4.73E-12	+	0.189181	0.2528	+	C	G	0.475580456	0.0341822	+	C	G	0.4967	0.0366
rs2300865	LC_IGP_R32	IgG1_G1S1/IgG1_G1	14	66120015	1	4.83E-10	3.98E-10	+	0.213317	0.2257	+	C	G	0.473939755	0.0340621	+	C	G	0.4967	0.0361
rs2300865	LC_IGP_R3	IgG1_G2F/IgG1_G2	14	66120015	1	1.83E-15	6.52E-15	+	0.272102	0.2904	+	C	G	0.473955685	0.0338794	+	C	G	0.4967	0.0373
rs2300865	LC_IGP_R8	IgG1_G2FS1/IgG1_G2S1	14	66120015	1	3.47E-10	8.04E-13	+	0.215468	0.2662	+	C	G	0.473666158	0.034119	+	C	G	0.4967	0.0372
rs2300865	LC_IGP_R26	IgG1_G2N/IgG1_G1N	14	66120015	1	7.82E-12	1.28E-08	+	0.235343	0.2071	+	C	G	0.475241667	0.0341441	+	C	G	0.4967	0.0364
rs2300865	LC_IGP148	IgG2 FG0n total/G0n	14	66120015	1	3.13E-09	1.86E-13	+	0.204026	0.2734	+	C	G	0.472839594	0.034247	+	C	G	0.4967	0.0372
rs2300865	LC_IGP97	IgG2_G0	14	66120015	1	7.34E-11	1.28E-13	-	-0.222996	-0.2755	+	C	G	0.473955685	0.0340087	+	C	G	0.4967	0.0372
rs2300865	LC_IGP_R41	IgG2_G0F/IgG2_G0	14	66120015	1	2.71E-08	8.17E-13	+	0.1906	0.2646	+	C	G	0.473955685	0.0341173	+	C	G	0.4967	0.037
rs2300865	LC_IGP138	IgG2_G0n	14	66120015	1	4.28E-11	8.90E-14	-	-0.22658	-0.2777	+	C	G	0.472839594	0.0341267	+	C	G	0.4967	0.0372
rs2300865	LC_IGP_R64	IgG2_G2/IgG2_G1	14	66120015	1	4.28E-09	2.93E-12	+	0.202532	0.2542	+	C	G	0.473005814	0.0342977	+	C	G	0.4967	0.0364
rs72716418	LC_IGP61	IgG1 Fn total	14	66121045	1	4.74E-08	1.05E-06	-	-0.236486	-0.252	+	A	G	0.203274773	0.0430999	+	A	G	0.1655	0.0516
rs11627184	LC_IGP78	IgG1 BG0n/G0n	14	66121443	1	2.62E-14	9.48E-10	-	-0.262455	-0.2283	+	T	C	0.475153346	0.0341476	+	T	C	0.4967	0.0373
rs11627184	LC_IGP79	IgG1 BG1n/G1n	14	66121443	1	3.39E-13	7.16E-12	-	-0.251588	-0.2554	+	T	C	0.475153346	0.0342805	+	T	C	0.4967	0.0373
rs11627184	LC_IGP77	IgG1 Bn	14	66121443	1	2.85E-13	2.01E-11	-	-0.252441	-0.2511	+	T	C	0.475153346	0.0342837	+	T	C	0.4967	0.0374
rs11627184	LC_IGP86	IgG1 Bn/Fn total	14	66121443	1	3.85E-14	1.20E-11	-	-0.261369	-0.254	+	T	C	0.475153346	0.0342345	+	T	C	0.4967	0.0375
rs11627184	LC_IGP84	IgG1 FBn/Bn total	14	66121443	1	2.72E-15	3.35E-16	+	0.273354	0.308	+	T	C	0.475153346	0.0342484	+	T	C	0.4967	0.0378
rs11627184	LC_IGP62	IgG1 FG0n total/G0n	14	66121443	1	6.23E-18	2.74E-12	+	0.296246	0.2626	+	T	C	0.475153346	0.0339332	+	T	C	0.4967	0.0376
rs11627184	LC_IGP63	IgG1 FG1n total/G1n	14	66121443	1	6.56E-18	1.31E-14	+	0.29692	0.2891	+	T	C	0.475153346	0.0340343	+	T	C	0.4967	0.0375
rs11627184	LC_IGP64	IgG1 FG2n total/G2n	14	66121443	1	3.68E-14	1.89E-15	+	0.261701	0.2967	+	T	C	0.475153346	0.03425	+	T	C	0.4967	0.0373
rs11627184	LC_IGP68	IgG1 FG2n/G2n	14	66121443	1	1.08E-10	1.30E-08	+	0.223574	0.2114	+	T	C	0.475153346	0.0344057	+	T	C	0.4967	0.0372
rs11627184	LC_IGP61	IgG1 Fn total	14	66121443	1	3.85E-17	3.29E-14	+	0.289813	0.285	+	T	C	0.475153346	0.0340473	+	T	C	0.4967	0.0376
rs11627184	LC_IGP81	IgG1 Fn/Bn	14	66121443	1	1.32E-13	1.05E-10	+	0.25581	0.2408	+	T	C	0.475153346	0.0342487	+	T	C	0.4967	0.0373
rs11627184	LC_IGP21	IgG1 Fucosylation	14	66121443	1	1.38E-15	4.71E-13	+	0.274481	0.2712	+	T	C	0.47435111	0.0340214	+	T	C	0.4967	0.0375
rs11627184	LC_IGP28	IgG1 G1S1/(G1+G1S1)	14	66121443	1	3.87E-10	2.47E-10	+	0.214434	0.2281	+	T	C	0.474555046	0.0340483	+	T	C	0.4967	0.036
rs11627184	LC_IGP27	IgG1 GS1/(G0+G+GS1)	14	66121443	1	4.15E-09	2.80E-08	+	0.201764	0.2036	+	T	C	0.474555046	0.0341379	+	T	C	0.4967	0.0367
rs11627184	LC_IGP11	IgG1_G0	14	66121443	1	1.21E-19	2.70E-14	-	-0.308336	-0.2843	+	T	C	0.47489157	0.0335712	+	T	C	0.4967	0.0374
rs11627184	LC_IGP_R1	IgG1_G0F/IgG1_G0	14	66121443	1	5.63E-17	6.01E-12	+	0.285096	0.2576	+	T	C	0.47489157	0.0336809	+	T	C	0.4967	0.0374
rs11627184	LC_IGP_R4	IgG1_G0FN/IgG1_G0N	14	66121443	1	7.43E-15	3.73E-13	+	0.266586	0.2738	+	T	C	0.475442437	0.0339566	+	T	C	0.4967	0.0377
rs11627184	LC_IGP52	IgG1_G0n	14	66121443	1	1.00E-19	2.40E-14	-	-0.311443	-0.2854	+	T	C	0.475153346	0.033823	+	T	C	0.4967	0.0374
rs11627184	LC_IGP14	IgG1_G0N	14	66121443	1	3.75E-17	4.34E-11	-	-0.288122	-0.2451	+	T	C	0.475442437	0.0338398	+	T	C	0.4967	0.0372
rs11627184	LC_IGP55	IgG1_G0Nn	14	66121443	1	1.51E-17	4.59E-11	-	-0.29342	-0.245	+	T	C	0.475153346	0.0340219	+	T	C	0.4967	0.0372
rs11627184	LC_IGP12	IgG1_G1	14	66121443	1	2.48E-16	9.02E-14	-	-0.279825	-0.2798	+	T	C	0.47489157	0.0337859	+	T	C	0.4967	0.0375
rs11627184	LC_IGP_R2	IgG1_G1F/IgG1_G1	14	66121443	1	1.21E-17	1.09E-13	+	0.291826	0.2782	+	T	C	0.47489157	0.0337382	+	T	C	0.4967	0.0374
rs11627184	LC_IGP_R5	IgG1_G1FN/IgG1_G1N	14	66121443	1	2.40E-14	1.55E-17	+	0.262598	0.32	+	T	C	0.476259225	0.0341161	+	T	C	0.4967	0.0375
rs11627184	LC_IGP53	IgG1_G1n	14	66121443	1	1.67E-15	1.59E-13	-	-0.274489	-0.2768	+	T	C	0.475153346	0.0341232	+	T	C	0.4967	0.0375
rs11627184	LC_IGP15	IgG1_G1N	14	66121443	1	2.22E-11	8.97E-11	-	-0.230707	-0.2418	+	T	C	0.476259225	0.0342395	+	T	C	0.4967	0.0373
rs11627184	LC_IGP56	IgG1_G1Nn	14	66121443	1	8.77E-12	1.87E-10	-	-0.236227	-0.2374	+	T	C	0.475153346	0.0343547	+	T	C	0.4967	0.0372
rs11627184	LC_IGP_R32	IgG1_G1S1/IgG1_G1	14	66121443	1	6.64E-10	3.70E-10	+	0.211597	0.2261	+	T	C	0.474555046	0.0340644	+	T	C	0.4967	0.0361
rs11627184	LC_IGP_R3	IgG1_G2F/IgG1_G2	14	66121443	1	1.32E-15	5.93E-15	+	0.273407	0.2908	+	T	C	0.474570599	0.0338686	+	T	C	0.4967	0.0373
rs11627184	LC_IGP_R8	IgG1_G2FS1/IgG1_G2S1	14	66121443	1	1.82E-10	7.36E-13	+	0.218831	0.2666	+	T	C	0.474280696	0.0341016	+	T	C	0.4967	0.0372
rs11627184	LC_IGP_R26	IgG1_G2N/IgG1_G1N	14	66121443	1	7.52E-12	1.21E-08	+	0.235506	0.2074	+	T	C	0.475862654	0.0341393	+	T	C	0.4967	0.0364
rs11627184	LC_IGP148	IgG2 FG0n total/G0n	14	66121443	1	2.42E-09	1.72E-13	+	0.205441	0.2738	+	T	C	0.473459052	0.0342373	+	T	C	0.4967	0.0372
rs11627184	LC_IGP97	IgG2_G0	14	66121443	1	5.00E-11	1.18E-13	-	-0.224906	-0.2759	+	T	C	0.474570599	0.0339967	+	T	C	0.4967	0.0372
rs11627184	LC_IGP_R41	IgG2_G0F/IgG2_G0	14	66121443	1	1.94E-08	7.53E-13	+	0.192544	0.265	+	T	C	0.474570599	0.0341064	+	T	C	0.4967	0.037
rs11627184	LC_IGP138	IgG2_G0n	14	66121443	1	2.89E-11	8.19E-14	-	-0.228527	-0.2781	+	T	C	0.473459052	0.0341142	+	T	C	0.4967	0.0372
rs11627184	LC_IGP_R64	IgG2_G2/IgG2_G1	14	66121443	1	2.52E-09	2.77E-12	+	0.205477	0.2545	+	T	C	0.473621481	0.0342825	+	T	C	0.4967	0.0364
rs11627185	LC_IGP78	IgG1 BG0n/G0n	14	66121492	1	2.62E-14	9.48E-10	-	-0.262455	-0.2283	+	G	A	0.475153346	0.0341476	+	G	A	0.4967	0.0373
rs11627185	LC_IGP79	IgG1 BG1n/G1n	14	66121492	1	3.39E-13	7.16E-12	-	-0.251588	-0.2554	+	G	A	0.475153346	0.0342805	+	G	A	0.4967	0.0373
rs11627185	LC_IGP77	IgG1 Bn	14	66121492	1	2.85E-13	2.01E-11	-	-0.252441	-0.2511	+	G	A	0.475153346	0.0342837	+	G	A	0.4967	0.0374
rs11627185	LC_IGP86	IgG1 Bn/Fn total	14	66121492	1	3.85E-14	1.20E-11	-	-0.261369	-0.254	+	G	A	0.475153346	0.0342345	+	G	A	0.4967	0.0375
rs11627185	LC_IGP84	IgG1 FBn/Bn total	14	66121492	1	2.72E-15	3.35E-16	+	0.273354	0.308	+	G	A	0.475153346	0.0342484	+	G	A	0.4967	0.0378
rs11627185	LC_IGP62	IgG1 FG0n total/G0n	14	66121492	1	6.23E-18	2.74E-12	+	0.296246	0.2626	+	G	A	0.475153346	0.0339332	+	G	A	0.4967	0.0376
rs11627185	LC_IGP63	IgG1 FG1n total/G1n	14	66121492	1	6.56E-18	1.31E-14	+	0.29692	0.2891	+	G	A	0.475153346	0.0340343	+	G	A	0.4967	0.0375
rs11627185	LC_IGP64	IgG1 FG2n total/G2n	14	66121492	1	3.68E-14	1.89E-15	+	0.261701	0.2967	+	G	A	0.475153346	0.03425	+	G	A	0.4967	0.0373
rs11627185	LC_IGP68	IgG1 FG2n/G2n	14	66121492	1	1.08E-10	1.30E-08	+	0.223574	0.2114	+	G	A	0.475153346	0.0344057	+	G	A	0.4967	0.0372
rs11627185	LC_IGP61	IgG1 Fn total	14	66121492	1	3.85E-17	3.29E-14	+	0.289813	0.285	+	G	A	0.475153346	0.0340473	+	G	A	0.4967	0.0376

rs11627185	LC_IGP81	IgG1 Fn/Bn	14	66121492	1	1.32E-13	1.05E-10	+	0.25581	0.2408	+	G	A	0.475153346	0.0342487	+	G	A	0.4967	0.0373
rs11627185	LC_IGP21	IgG1 Fucosylation	14	66121492	1	1.38E-15	4.71E-13	+	0.274481	0.2712	+	G	A	0.47435111	0.0340214	+	G	A	0.4967	0.0375
rs11627185	LC_IGP28	IgG1 G1S1/(G1+G1S1)	14	66121492	1	3.87E-10	2.47E-10	+	0.214434	0.2281	+	G	A	0.474555046	0.0340483	+	G	A	0.4967	0.036
rs11627185	LC_IGP27	IgG1 GS1/(G0+G+GS1)	14	66121492	1	4.15E-09	2.80E-08	+	0.201764	0.2036	+	G	A	0.474555046	0.0341379	+	G	A	0.4967	0.0367
rs11627185	LC_IGP11	IgG1_G0	14	66121492	1	1.21E-19	2.70E-14	-	-0.308336	-0.2843	+	G	A	0.47489157	0.0335712	+	G	A	0.4967	0.0374
rs11627185	LC_IGP_R1	IgG1_G0F/IgG1_G0	14	66121492	1	5.63E-17	6.01E-12	+	0.285096	0.2576	+	G	A	0.47489157	0.0336809	+	G	A	0.4967	0.0374
rs11627185	LC_IGP_R4	IgG1_G0FN/IgG1_G0N	14	66121492	1	7.43E-15	3.73E-13	+	0.266586	0.2738	+	G	A	0.475442437	0.0339566	+	G	A	0.4967	0.0377
rs11627185	LC_IGP52	IgG1_G0n	14	66121492	1	1.00E-19	2.40E-14	-	-0.311443	-0.2854	+	G	A	0.475153346	0.033823	+	G	A	0.4967	0.0374
rs11627185	LC_IGP14	IgG1_G0N	14	66121492	1	3.75E-17	4.34E-11	-	-0.288122	-0.2451	+	G	A	0.475442437	0.0338398	+	G	A	0.4967	0.0372
rs11627185	LC_IGP55	IgG1_G0Nn	14	66121492	1	1.51E-17	4.59E-11	-	-0.29342	-0.245	+	G	A	0.475153346	0.0340219	+	G	A	0.4967	0.0372
rs11627185	LC_IGP12	IgG1_G1	14	66121492	1	2.48E-16	9.02E-14	-	-0.279825	-0.2798	+	G	A	0.47489157	0.0337859	+	G	A	0.4967	0.0375
rs11627185	LC_IGP_R2	IgG1_G1F/IgG1_G1	14	66121492	1	1.21E-17	1.09E-13	+	0.291826	0.2782	+	G	A	0.47489157	0.0337382	+	G	A	0.4967	0.0374
rs11627185	LC_IGP_R5	IgG1_G1FN/IgG1_G1N	14	66121492	1	2.40E-14	1.55E-17	+	0.262598	0.32	+	G	A	0.476259225	0.0341161	+	G	A	0.4967	0.0375
rs11627185	LC_IGP53	IgG1_G1n	14	66121492	1	1.67E-15	1.59E-13	-	-0.274489	-0.2768	+	G	A	0.475153346	0.0341232	+	G	A	0.4967	0.0375
rs11627185	LC_IGP15	IgG1_G1N	14	66121492	1	2.22E-11	8.97E-11	-	-0.230707	-0.2418	+	G	A	0.476259225	0.0342395	+	G	A	0.4967	0.0373
rs11627185	LC_IGP56	IgG1_G1Nn	14	66121492	1	8.77E-12	1.87E-10	-	-0.236227	-0.2374	+	G	A	0.475153346	0.0343547	+	G	A	0.4967	0.0372
rs11627185	LC_IGP_R32	IgG1_G1S1/IgG1_G1	14	66121492	1	6.64E-10	3.70E-10	+	0.211597	0.2261	+	G	A	0.474555046	0.0340644	+	G	A	0.4967	0.0361
rs11627185	LC_IGP_R3	IgG1_G2F/IgG1_G2	14	66121492	1	1.32E-15	5.93E-15	+	0.273407	0.2908	+	G	A	0.474570599	0.0338686	+	G	A	0.4967	0.0373
rs11627185	LC_IGP_R8	IgG1_G2FS1/IgG1_G2S1	14	66121492	1	1.82E-10	7.36E-13	+	0.218831	0.2666	+	G	A	0.474280696	0.0341016	+	G	A	0.4967	0.0372
rs11627185	LC_IGP_R26	IgG1_G2N/IgG1_G1N	14	66121492	1	7.52E-12	1.21E-08	+	0.235506	0.2074	+	G	A	0.475862654	0.0341393	+	G	A	0.4967	0.0364
rs11627185	LC_IGP148	IgG2 FG0n total/G0n	14	66121492	1	2.42E-09	1.72E-13	+	0.205441	0.2738	+	G	A	0.473459052	0.0342373	+	G	A	0.4967	0.0372
rs11627185	LC_IGP97	IgG2_G0	14	66121492	1	5.00E-11	1.18E-13	-	-0.224906	-0.2759	+	G	A	0.474570599	0.0339967	+	G	A	0.4967	0.0372
rs11627185	LC_IGP_R41	IgG2_G0F/IgG2_G0	14	66121492	1	1.94E-08	7.53E-13	+	0.192544	0.265	+	G	A	0.474570599	0.0341064	+	G	A	0.4967	0.037
rs11627185	LC_IGP138	IgG2_G0n	14	66121492	1	2.89E-11	8.19E-14	-	-0.228527	-0.2781	+	G	A	0.473459052	0.0341142	+	G	A	0.4967	0.0372
rs11627185	LC_IGP_R64	IgG2_G2/IgG2_G1	14	66121492	1	2.52E-09	2.77E-12	+	0.205477	0.2545	+	G	A	0.473621481	0.0342825	+	G	A	0.4967	0.0364
rs7493004	LC_IGP78	IgG1 BG0n/G0n	14	66124104	1	2.81E-14	9.89E-10	-	-0.262184	-0.228	+	C	A	0.47452881	0.0341531	+	C	A	0.4967	0.0373
rs7493004	LC_IGP79	IgG1 BG1n/G1n	14	66124104	1	3.61E-13	7.69E-12	-	-0.251338	-0.255	+	C	A	0.47452881	0.0342858	+	C	A	0.4967	0.0373
rs7493004	LC_IGP32	IgG1 BG1S1/(BG1+BG1S1)	14	66124104	1	3.99E-08	4.06E-12	+	0.188583	0.2534	+	C	A	0.475579223	0.0341759	+	C	A	0.4967	0.0365
rs7493004	LC_IGP77	IgG1 Bn	14	66124104	1	3.04E-13	2.14E-11	-	-0.252172	-0.2508	+	C	A	0.47452881	0.0342891	+	C	A	0.4967	0.0374
rs7493004	LC_IGP86	IgG1 Bn/Fn total	14	66124104	1	4.18E-14	1.28E-11	-	-0.261039	-0.2537	+	C	A	0.47452881	0.0342402	+	C	A	0.4967	0.0375
rs7493004	LC_IGP84	IgG1 FBn/Bn total	14	66124104	1	2.72E-15	3.61E-16	+	0.273381	0.3077	+	C	A	0.47452881	0.0342525	+	C	A	0.4967	0.0378
rs7493004	LC_IGP62	IgG1 FG0n total/G0n	14	66124104	1	7.80E-18	2.89E-12	+	0.295416	0.2623	+	C	A	0.47452881	0.0339419	+	C	A	0.4967	0.0376
rs7493004	LC_IGP63	IgG1 FG1n total/G1n	14	66124104	1	8.00E-18	1.42E-14	+	0.296187	0.2888	+	C	A	0.47452881	0.0340425	+	C	A	0.4967	0.0375
rs7493004	LC_IGP64	IgG1 FG2n total/G2n	14	66124104	1	4.81E-14	2.04E-15	+	0.26055	0.2964	+	C	A	0.47452881	0.0342596	+	C	A	0.4967	0.0373
rs7493004	LC_IGP68	IgG1 FG2n/G2n	14	66124104	1	1.50E-10	1.36E-08	+	0.221908	0.2111	+	C	A	0.47452881	0.0344166	+	C	A	0.4967	0.0372
rs7493004	LC_IGP61	IgG1 Fn total	14	66124104	1	4.89E-17	3.53E-14	+	0.288898	0.2847	+	C	A	0.47452881	0.0340563	+	C	A	0.4967	0.0376
rs7493004	LC_IGP81	IgG1 Fn/Bn	14	66124104	1	1.44E-13	1.12E-10	+	0.255423	0.2405	+	C	A	0.47452881	0.0342547	+	C	A	0.4967	0.0373
rs7493004	LC_IGP21	IgG1 Fucosylation	14	66124104	1	1.70E-15	5.03E-13	+	0.273644	0.2709	+	C	A	0.473729655	0.0340298	+	C	A	0.4967	0.0375
rs7493004	LC_IGP28	IgG1 G1S1/(G1+G1S1)	14	66124104	1	2.68E-10	2.58E-10	+	0.216379	0.2278	+	C	A	0.473938532	0.0340449	+	C	A	0.4967	0.036
rs7493004	LC_IGP27	IgG1 GS1/(G0+G+GS1)	14	66124104	1	2.15E-09	2.90E-08	+	0.205456	0.2034	+	C	A	0.473938532	0.0341285	+	C	A	0.4967	0.0367
rs7493004	LC_IGP11	IgG1_G0	14	66124104	1	1.40E-19	2.86E-14	-	-0.307856	-0.284	+	C	A	0.474275809	0.0335781	+	C	A	0.4967	0.0374
rs7493004	LC_IGP_R1	IgG1_G0F/IgG1_G0	14	66124104	1	7.42E-17	6.36E-12	+	0.284044	0.2573	+	C	A	0.474275809	0.0336904	+	C	A	0.4967	0.0374
rs7493004	LC_IGP_R4	IgG1_G0FN/IgG1_G0N	14	66124104	1	7.48E-15	3.93E-13	+	0.266588	0.2736	+	C	A	0.474825168	0.0339607	+	C	A	0.4967	0.0377
rs7493004	LC_IGP52	IgG1_G0n	14	66124104	1	1.20E-19	2.54E-14	-	-0.310812	-0.2851	+	C	A	0.47452881	0.0338308	+	C	A	0.4967	0.0374
rs7493004	LC_IGP14	IgG1_G0N	14	66124104	1	3.58E-17	4.53E-11	-	-0.288334	-0.2449	+	C	A	0.474825168	0.0338427	+	C	A	0.4967	0.0372
rs7493004	LC_IGP55	IgG1_G0Nn	14	66124104	1	1.50E-17	4.79E-11	-	-0.293487	-0.2448	+	C	A	0.47452881	0.0340257	+	C	A	0.4967	0.0372
rs7493004	LC_IGP12	IgG1_G1	14	66124104	1	3.01E-16	9.64E-14	-	-0.279086	-0.2795	+	C	A	0.474275809	0.0337937	+	C	A	0.4967	0.0375
rs7493004	LC_IGP_R2	IgG1_G1F/IgG1_G1	14	66124104	1	1.56E-17	1.17E-13	+	0.290891	0.2778	+	C	A	0.474275809	0.0337472	+	C	A	0.4967	0.0374
rs7493004	LC_IGP_R5	IgG1_G1FN/IgG1_G1N	14	66124104	1	2.48E-14	1.70E-17	+	0.262477	0.3196	+	C	A	0.475639299	0.0341207	+	C	A	0.4967	0.0375
rs7493004	LC_IGP53	IgG1_G1n	14	66124104	1	2.12E-15	1.69E-13	-	-0.273518	-0.2764	+	C	A	0.47452881	0.0341322	+	C	A	0.4967	0.0375
rs7493004	LC_IGP15	IgG1_G1N	14	66124104	1	2.35E-11	9.55E-11	-	-0.230445	-0.2415	+	C	A	0.475639299	0.0342445	+	C	A	0.4967	0.0373
rs7493004	LC_IGP56	IgG1_G1Nn	14	66124104	1	9.46E-12	1.99E-10	-	-0.235879	-0.237	+	C	A	0.47452881	0.0343603	+	C	A	0.4967	0.0373
rs7493004	LC_IGP_R36	IgG1_G1NS1/IgG1_G1N	14	66124104	1	3.63E-08	4.65E-12	+	0.189183	0.2529	+	C	A	0.475579223	0.0341821	+	C	A	0.4967	0.0366
rs7493004	LC_IGP_R32	IgG1_G1S1/IgG1_G1	14	66124104	1	4.83E-10	3.86E-10	+	0.213318	0.2259	+	C	A	0.473938532	0.0340619	+	C	A	0.4967	0.0361
rs7493004	LC_IGP_R3	IgG1_G2F/IgG1_G2	14	66124104	1	1.82E-15	6.40E-15	+	0.272104	0.2905	+	C	A	0.473954462	0.0338792	+	C	A	0.4967	0.0373
rs7493004	LC_IGP_R8	IgG1_G2FS1/IgG1_G2S1	14	66124104	1	3.47E-10	7.86E-13	+	0.215472	0.2663	+	C	A	0.473664936	0.0341189	+	C	A	0.4967	0.0372
rs7493004	LC_IGP_R26	IgG1_G2N/IgG1_G1N	14	66124104	1	7.82E-12	1.28E-08	+	0.235345	0.2071	+	C	A	0.475240432	0.034144	+	C	A	0.4967	0.0364
rs7493004	LC_IGP148	IgG2 FG0n total/G0n	14	66124104	1	3.13E-09	1.76E-13	+	0.204033	0.2736	+	C	A	0.472838362	0.0342468	+	C	A	0.4967	0.0372
rs7493004	LC_IGP97	IgG2_G0	14	66124104	1	7.33E-11	1.20E-13	-	-0.223	-0.2758	+	C	A	0.473954462	0.0340086	+	C	A	0.4967	0.0372
rs7493004	LC_IGP_R41	IgG2_G0F/IgG2_G0	14	66124104	1	2.70E-08	7.75E-13	+	0.190606	0.2648	+	C	A	0.473954462	0.0341172	+	C	A	0.4967	0.037
rs7493004	LC_IGP138	IgG2_G0n	14	66124104	1	4.28E-11	8.39E-14	-	-0.226585	-0.2779	+	C	A	0.472838362	0.0341266	+	C	A	0.4967	0.0372
rs7493004	LC_IGP_R64	IgG2_G2/IgG2_G1	14	66124104	1	4.28E-09	2.88E-12	+	0.202532	0.2543	+	C	A	0.47300459	0.0342976	+	C	A	0.4967	0.0364
rs67957534	LC_IGP78	IgG1 BG0n/G0n	14	66124585	1	2.62E-14	9.62E-10	-	-0.262457	-0.2282	+	G	T	0.475152726	0.0341475	+	G	T	0.4968	0.0373
rs67957534	LC_IGP79	IgG1 BG1n/G1n	14	66124585	1	3.39E-13	7.34E-12	-	-0.251588	-0.2553	+	G	T	0.475152726	0.0342803	+	G	T	0.4968	0.0373
rs67957534	LC_IGP77	IgG1 Bn	14	66124585	1	2.85E-13	2.05E-11	-	-0.252441	-0.251	+	G	T	0.475152726	0.0342836	+	G	T	0.4968	0.0374
rs67957534	LC_IGP86	IgG1 Bn/Fn total	14	66124585	1	3.85E-14	1.22E-11	-	-0.261369	-0.2539	+	G	T	0.475152726	0.0342343	+	G	T	0.4968	0.0375
rs67957534	LC_IGP84	IgG1 FBn/Bn total	14	66124585	1	2.72E-15	3.45E-16	+	0.273353	0.3079	+	G	T	0.475152726	0.0342483	+	G	T	0.4968	0.0378
rs67957534	LC_IGP62	IgG1 FG0n total/G0n	14	66124585	1	6.22E-18	2.80E-12	+	0.296248	0.2625	+	G	T	0.475152726	0.0339331	+	G	T	0.4968	0.0376
rs67957534	LC_IGP63	IgG1 FG1n total/G1n	14	66124585	1	6.56E-18	1.35E-14	+	0.29692	0.289	+	G	T	0.475152726	0.0340342	+	G	T	0.4968	0.0375
rs67957534	LC_IGP64	IgG1 FG2n total/G2n	14	66124585	1	3.68E-14	1.94E-15	+	0.2617	0.2966	+	G	T	0.475152726	0.0342499	+	G	T	0.4968	0.0373
rs67957534	LC_IGP68	IgG1 FG2n/G2n	14	66124585	1	1.08E-10	1.31E-08	+	0.223574	0.2114	+	G	T	0.475152726	0.0344056	+	G	T	0.4968	0.0372
rs67957534	LC_IGP61	IgG1 Fn total	14	66124585	1	3.85E-17	3.36E-14	+	0.289815	0.2849	+	G	T	0.475152726	0.0340472	+	G	T	0.4968	0.0376
rs67957534	LC_IGP81	IgG1 Fn/Bn	14	66124585	1	1.32E-13	1.08E-10	+	0.25581	0.2407	+	G	T	0.475152726	0.0342486	+	G	T	0.4968	0.0373
rs67957534	LC_IGP21	IgG1 Fucosylation	14	66124585	1	1.38E-15	4.81E-13	+	0.274482	0.2712	+	G	T	0.474350493	0.0340212	+	G	T	0.4968	0.0375
rs67957534	LC_IGP28	IgG1 G1S1/(G1+G1S1)	14	66124585	1	3.87E-10	2.52E-10	+	0.214433	0.228	+	G	T	0.474554434	0.0340482	+	G	T	0.4968	0.036
rs67957534	LC_IGP27	IgG1 GS1/(G0+G+GS1)	14	66124585	1	4.15E-09	2.88E-08	+	0.20176	0.2034	+	G	T	0.474554434	0.0341378	+	G	T	0.4968	0.0367
rs67957534	LC_IGP11	IgG1_G0	14	66124585	1	1.21E-19	2.80E-14	-	-0.308336	-0.2841	+	G	T	0.474890959	0.0335711	+	G	T	0.4968	0.0374
rs67957534	LC_IGP_R1	IgG1_G0F/IgG1_G0	14	66124585	1	5.62E-17	6.14E-12	+	0.285098	0.2575	+	G	T	0.474890959	0.0336807	+	G	T	0.4968	0.0374
rs67957534	LC_IGP_R4	IgG1_G0FN/IgG1_G0N	14	66124585	1	7.43E-15	3.82E-13	+	0.266587	0.2737	+	G	T	0.475441825	0.0339565	+	G	T	0.4968	0.0377
rs67957534	LC_IGP52	IgG1_G0n	14	66124585	1	1.00E-19	2.49E-14	-	-0.311444	-0.2852	+	G	T	0.475152726	0.0338228	+	G	T	0.4968	0.0374
rs67957534	LC_IGP14	IgG1_G0N	14	66124585	1	3.74E-17	4.46E-11	-	-0.288122	-0.245	+	G	T	0.475441825	0.0338397	+	G	T	0.4968	0.0372
rs67957534	LC_IGP55	IgG1_G0Nn	14	66124585	1	1.51E-17	4.72E-11	-	-0.293419	-0.2449	+	G	T	0.475152726	0.0340218	+	G	T	0.4968	0.0372
rs67957534	LC_IGP12	IgG1_G1	14	66124585	1	2.48E-16	9.19E-14	-	-0.279826	-0.2797	+	G	T	0.474890959	0.0337858	+	G	T	0.4968	0.0375
rs67957534	LC_IGP_R2	IgG1_G1F/IgG1_G1	14	66124585	1	1.21E-17	1.12E-13	+	0.291826	0.2781	+	G	T	0.474890959	0.0337381	+	G	T	0.4968	0.0374
rs67957534	LC_IGP_R5	IgG1_G1FN/IgG1_G1N	14	66124585	1	2.40E-14	1.61E-17	+	0.262598	0.3198	+	G	T	0.47625861	0.034116	+	G	T	0.4968	0.0375
rs67957534	LC_IGP53	IgG1_G1n	14	66124585	1	1.67E-15	1.61E-13	-	-0.27449	-0.2767	+	G	T	0.475152726	0.0341231	+	G	T	0.4968	0.0375
rs67957534	LC_IGP15	IgG1_G1N	14	66124585	1	2.22E-11	9.12E-11	-	-0.230707	-0.2418	+	G	T	0.47625861	0.0342394	+	G	T	0.4968	0.0373
rs67957534	LC_IGP56	IgG1_G1Nn	14	66124585	1	8.76E-12	1.90E-10	-	-0.236227	-0.2373	+	G	T	0.475152726	0.0343545	+	G	T	0.4968	0.0372
rs67957534	LC_IGP_R32	IgG1_G1S1/IgG1_G1	14	66124585	1	6.64E-10	3.78E-10	+	0.211596	0.226	+	G	T	0.474554434	0.0340643	+	G	T	0.4968	0.0361
rs67957534	LC_IGP_R3	IgG1_G2F/IgG1_G2	14	66124585	1	1.32E-15	6.07E-15	+	0.273407	0.2907	+	G	T	0.474569988	0.0338685	+	G	T	0.4968	0.0373
rs67957534	LC_IGP_R8	IgG1_G2FS1/IgG1_G2S1	14	66124585	1	1.81E-10	7.49E-13	+	0.218834	0.2666	+	G	T	0.474280086	0.0341015	+	G	T	0.4968	0.0372
rs67957534	LC_IGP_R26	IgG1_G2N/IgG1_G1N	14	66124585	1	7.52E-12	1.25E-08	+	0.235507	0.2073	+	G	T	0.475862037	0.0341392	+	G	T	0.4968	0.0364
rs67957534	LC_IGP148	IgG2 FG0n total/G0n	14	66124585	1	2.42E-09	1.69E-13	+	0.205445	0.2738	+	G	T	0.473458436	0.0342372	+	G	T	0.4968	0.0372
rs67957534	LC_IGP97	IgG2_G0	14	66124585	1	5.00E-11	1.15E-13	-	-0.224908	-0.276	+	G	T	0.474569988	0.0339966	+	G	T	0.4968	0.0372
rs67957534	LC_IGP_R41	IgG2_G0F/IgG2_G0	14	66124585	1	1.94E-08	7.39E-13	+	0.192547	0.2651	+	G	T	0.474569988	0.0341063	+	G	T	0.4968	0.037
rs67957534	LC_IGP138	IgG2_G0n	14	66124585	1	2.89E-11	8.03E-14	-	-0.228529	-0.2782	+	G	T	0.473458436	0.0341141	+	G	T	0.4968	0.0372
rs67957534	LC_IGP_R64	IgG2_G2/IgG2_G1	14	66124585	1	2.52E-09	2.80E-12	+	0.205477	0.2544	+	G	T	0.473620869	0.0342824	+	G	T	0.4968	0.0364
rs7142651	LC_IGP78	IgG1 BG0n/G0n	14	66132721	1	2.81E-14	9.83E-10	-	-0.262189	-0.227	+	G	C	0.474526332	0.0341527	+	G	C	0.4965	0.0371
rs7142651	LC_IGP79	IgG1 BG1n/G1n	14	66132721	1	3.61E-13	6.57E-12	-	-0.251337	-0.2548	+	G	C	0.474526332	0.0342855	+	G	C	0.4965	0.0371
rs7142651	LC_IGP32	IgG1 BG1S1/(BG1+BG1S1)	14	66132721	1	3.99E-08	2.86E-12	+	0.188582	0.2541	+	G	C	0.475576757	0.0341756	+	G	C	0.4965	0.0364
rs7142651	LC_IGP77	IgG1 Bn	14	66132721	1	3.04E-13	1.99E-11	-	-0.252172	-0.25	+	G	C	0.474526332	0.0342888	+	G	C	0.4965	0.0373
rs7142651	LC_IGP86	IgG1 Bn/Fn total	14	66132721	1	4.18E-14	1.17E-11	-	-0.26104	-0.253	+	G	C	0.474526332	0.0342399	+	G	C	0.4965	0.0373
rs7142651	LC_IGP84	IgG1 FBn/Bn total	14	66132721	1	2.72E-15	3.67E-16	+	0.27338	0.3062	+	G	C	0.474526332	0.0342522	+	G	C	0.4965	0.0376
rs7142651	LC_IGP62	IgG1 FG0n total/G0n	14	66132721	1	7.78E-18	2.74E-12	+	0.295423	0.2614	+	G	C	0.474526332	0.0339415	+	G	C	0.4965	0.0374
rs7142651	LC_IGP63	IgG1 FG1n total/G1n	14	66132721	1	7.99E-18	1.34E-14	+	0.296189	0.2877	+	G	C	0.474526332	0.0340422	+	G	C	0.4965	0.0374
rs7142651	LC_IGP64	IgG1 FG2n total/G2n	14	66132721	1	4.81E-14	1.96E-15	+	0.260549	0.2952	+	G	C	0.474526332	0.0342593	+	G	C	0.4965	0.0372
rs7142651	LC_IGP68	IgG1 FG2n/G2n	14	66132721	1	1.50E-10	1.32E-08	+	0.221908	0.2103	+	G	C	0.474526332	0.0344162	+	G	C	0.4965	0.037
rs7142651	LC_IGP61	IgG1 Fn total	14	66132721	1	4.89E-17	3.42E-14	+	0.288903	0.2835	+	G	C	0.474526332	0.034056	+	G	C	0.4965	0.0374
rs7142651	LC_IGP81	IgG1 Fn/Bn	14	66132721	1	1.44E-13	1.02E-10	+	0.255424	0.2399	+	G	C	0.474526332	0.0342544	+	G	C	0.4965	0.0371
rs7142651	LC_IGP21	IgG1 Fucosylation	14	66132721	1	1.70E-15	4.84E-13	+	0.273648	0.2699	+	G	C	0.473727189	0.0340295	+	G	C	0.4965	0.0373
rs7142651	LC_IGP28	IgG1 G1S1/(G1+G1S1)	14	66132721	1	2.69E-10	2.83E-10	+	0.216376	0.2263	+	G	C	0.473936086	0.0340446	+	G	C	0.4965	0.0359
rs7142651	LC_IGP27	IgG1 GS1/(G0+G+GS1)	14	66132721	1	2.15E-09	2.84E-08	+	0.205444	0.2027	+	G	C	0.473936086	0.0341283	+	G	C	0.4965	0.0365
rs7142651	LC_IGP11	IgG1_G0	14	66132721	1	1.40E-19	2.36E-14	-	-0.307855	-0.2837	+	G	C	0.474273366	0.0335778	+	G	C	0.4965	0.0372
rs7142651	LC_IGP_R1	IgG1_G0F/IgG1_G0	14	66132721	1	7.40E-17	5.84E-12	+	0.284051	0.2566	+	G	C	0.474273366	0.0336901	+	G	C	0.4965	0.0373
rs7142651	LC_IGP_R4	IgG1_G0FN/IgG1_G0N	14	66132721	1	7.47E-15	4.24E-13	+	0.266591	0.272	+	G	C	0.474822719	0.0339603	+	G	C	0.4965	0.0375
rs7142651	LC_IGP52	IgG1_G0n	14	66132721	1	1.20E-19	2.05E-14	-	-0.310812	-0.285	+	G	C	0.474526332	0.0338304	+	G	C	0.4965	0.0373
rs7142651	LC_IGP14	IgG1_G0N	14	66132721	1	3.58E-17	4.10E-11	-	-0.288332	-0.2444	+	G	C	0.474822719	0.0338424	+	G	C	0.4965	0.037
rs7142651	LC_IGP55	IgG1_G0Nn	14	66132721	1	1.50E-17	4.26E-11	-	-0.293486	-0.2445	+	G	C	0.474526332	0.0340254	+	G	C	0.4965	0.0371
rs7142651	LC_IGP12	IgG1_G1	14	66132721	1	3.00E-16	9.84E-14	-	-0.27909	-0.278	+	G	C	0.474273366	0.0337934	+	G	C	0.4965	0.0374

rs7142651	LC_IGP_R2	IgG1_G1F/IgG1_G1	14	66132721	1	1.56E-17	1.11E-13	+	0.290893	0.2768	+	G	C	0.474273366	0.0337469	+	G	C	0.4965	0.0373
rs7142651	LC_IGP_R5	IgG1_G1FN/IgG1_G1N	14	66132721	1	2.48E-14	1.58E-17	+	0.262475	0.3185	+	G	C	0.475636839	0.0341203	+	G	C	0.4965	0.0374
rs7142651	LC_IGP53	IgG1_G1n	14	66132721	1	2.12E-15	1.69E-13	-	-0.273523	-0.2751	+	G	C	0.474526332	0.0341319	+	G	C	0.4965	0.0373
rs7142651	LC_IGP15	IgG1_G1N	14	66132721	1	2.35E-11	8.82E-11	-	-0.230446	-0.2409	+	G	C	0.475636839	0.0342442	+	G	C	0.4965	0.0371
rs7142651	LC_IGP56	IgG1_G1Nn	14	66132721	1	9.46E-12	1.81E-10	-	-0.235881	-0.2365	+	G	C	0.474526332	0.03436	+	G	C	0.4965	0.0371
rs7142651	LC_IGP_R36	IgG1_G1NS1/IgG1_G1N	14	66132721	1	3.63E-08	3.27E-12	+	0.189182	0.2536	+	G	C	0.475576757	0.0341818	+	G	C	0.4965	0.0364
rs7142651	LC_IGP_R32	IgG1_G1S1/IgG1_G1	14	66132721	1	4.83E-10	4.26E-10	+	0.213315	0.2243	+	G	C	0.473936086	0.0340616	+	G	C	0.4965	0.0359
rs7142651	LC_IGP_R3	IgG1_G2F/IgG1_G2	14	66132721	1	1.82E-15	6.33E-15	+	0.272103	0.2892	+	G	C	0.473952017	0.0338789	+	G	C	0.4965	0.0371
rs7142651	LC_IGP_R8	IgG1_G2FS1/IgG1_G2S1	14	66132721	1	3.46E-10	1.01E-12	+	0.21548	0.2638	+	G	C	0.473662492	0.0341185	+	G	C	0.4965	0.037
rs7142651	LC_IGP_R26	IgG1_G2N/IgG1_G1N	14	66132721	1	7.81E-12	9.92E-09	+	0.235346	0.2078	+	G	C	0.475237963	0.0341436	+	G	C	0.4965	0.0363
rs7142651	LC_IGP148	IgG2 FG0n total/G0n	14	66132721	1	3.12E-09	2.23E-13	+	0.204045	0.2712	+	G	C	0.472835899	0.0342465	+	G	C	0.4965	0.037
rs7142651	LC_IGP97	IgG2_G0	14	66132721	1	7.32E-11	1.28E-13	-	-0.223006	-0.2744	+	G	C	0.473952017	0.0340082	+	G	C	0.4965	0.037
rs7142651	LC_IGP_R41	IgG2_G0F/IgG2_G0	14	66132721	1	2.70E-08	9.02E-13	+	0.190617	0.2628	+	G	C	0.473952017	0.0341168	+	G	C	0.4965	0.0368
rs7142651	LC_IGP138	IgG2_G0n	14	66132721	1	4.27E-11	8.81E-14	-	-0.226591	-0.2766	+	G	C	0.472835899	0.0341263	+	G	C	0.4965	0.0371
rs7142651	LC_IGP_R64	IgG2_G2/IgG2_G1	14	66132721	1	4.28E-09	3.04E-12	+	0.202532	0.2532	+	G	C	0.473002142	0.0342973	+	G	C	0.4965	0.0363
rs72716421	LC_IGP61	IgG1 Fn total	14	66134551	1	4.75E-08	1.45E-06	-	-0.236464	-0.245	+	C	T	0.20325304	0.0430981	+	C	T	0.1669	0.0508
rs35949016	LC_IGP11	IgG1_G0	14	66136163	1	1.44E-08	2.16E-06	+	0.248095	0.2468	+	A	C	0.194563225	0.0435447	+	A	C	0.1597	0.0521
rs1998036	LC_IGP78	IgG1 BG0n/G0n	14	66138199	1	7.33E-14	8.75E-10	-	-0.258218	-0.2286	+	C	G	0.476131351	0.034208	+	C	G	0.499	0.0373
rs1998036	LC_IGP79	IgG1 BG1n/G1n	14	66138199	1	9.23E-13	4.86E-12	-	-0.247235	-0.2574	+	C	G	0.476131351	0.0343405	+	C	G	0.499	0.0372
rs1998036	LC_IGP77	IgG1 Bn	14	66138199	1	7.20E-13	1.59E-11	-	-0.248443	-0.2522	+	C	G	0.476131351	0.0343422	+	C	G	0.499	0.0374
rs1998036	LC_IGP86	IgG1 Bn/Fn total	14	66138199	1	1.02E-13	9.34E-12	-	-0.25733	-0.2552	+	C	G	0.476131351	0.0342938	+	C	G	0.499	0.0374
rs1998036	LC_IGP84	IgG1 FBn/Bn total	14	66138199	1	8.63E-15	2.67E-16	+	0.268736	0.3089	+	C	G	0.476131351	0.0343117	+	C	G	0.499	0.0377
rs1998036	LC_IGP62	IgG1 FG0n total/G0n	14	66138199	1	1.71E-17	2.27E-12	+	0.292669	0.2634	+	C	G	0.476131351	0.0339929	+	C	G	0.499	0.0375
rs1998036	LC_IGP63	IgG1 FG1n total/G1n	14	66138199	1	1.89E-17	9.59E-15	+	0.293147	0.2904	+	C	G	0.476131351	0.0340952	+	C	G	0.499	0.0375
rs1998036	LC_IGP64	IgG1 FG2n total/G2n	14	66138199	1	7.84E-14	1.42E-15	+	0.258637	0.2978	+	C	G	0.476131351	0.0343049	+	C	G	0.499	0.0373
rs1998036	LC_IGP68	IgG1 FG2n/G2n	14	66138199	1	1.58E-10	1.01E-08	+	0.22186	0.2127	+	C	G	0.476131351	0.0344528	+	C	G	0.499	0.0371
rs1998036	LC_IGP61	IgG1 Fn total	14	66138199	1	1.11E-16	2.54E-14	+	0.285909	0.286	+	C	G	0.476131351	0.0341083	+	C	G	0.499	0.0375
rs1998036	LC_IGP81	IgG1 Fn/Bn	14	66138199	1	3.28E-13	8.29E-11	+	0.25195	0.242	+	C	G	0.476131351	0.0343068	+	C	G	0.499	0.0373
rs1998036	LC_IGP21	IgG1 Fucosylation	14	66138199	1	2.84E-15	3.62E-13	+	0.27195	0.2723	+	C	G	0.475017879	0.034097	+	C	G	0.499	0.0375
rs1998036	LC_IGP28	IgG1 G1S1/(G1+G1S1)	14	66138199	1	5.69E-10	2.97E-10	+	0.212626	0.2268	+	C	G	0.475520795	0.0340942	+	C	G	0.499	0.036
rs1998036	LC_IGP27	IgG1 GS1/(G0+G+GS1)	14	66138199	1	3.09E-09	2.24E-08	+	0.20365	0.2049	+	C	G	0.475520795	0.03417	+	C	G	0.499	0.0366
rs1998036	LC_IGP11	IgG1_G0	14	66138199	1	2.05E-19	2.11E-14	-	-0.306782	-0.2854	+	C	G	0.475856139	0.0336194	+	C	G	0.499	0.0373
rs1998036	LC_IGP_R1	IgG1_G0F/IgG1_G0	14	66138199	1	1.32E-16	4.95E-12	+	0.282054	0.2584	+	C	G	0.475856139	0.0337359	+	C	G	0.499	0.0374
rs1998036	LC_IGP_R4	IgG1_G0FN/IgG1_G0N	14	66138199	1	1.76E-14	3.41E-13	+	0.263187	0.2742	+	C	G	0.476409369	0.0340126	+	C	G	0.499	0.0377
rs1998036	LC_IGP52	IgG1_G0n	14	66138199	1	1.84E-19	1.86E-14	-	-0.309584	-0.2866	+	C	G	0.476131351	0.0338741	+	C	G	0.499	0.0374
rs1998036	LC_IGP14	IgG1_G0N	14	66138199	1	7.83E-17	3.85E-11	-	-0.285537	-0.2458	+	C	G	0.476409369	0.0338931	+	C	G	0.499	0.0372
rs1998036	LC_IGP55	IgG1_G0Nn	14	66138199	1	3.58E-17	4.09E-11	-	-0.290375	-0.2457	+	C	G	0.476131351	0.0340786	+	C	G	0.499	0.0372
rs1998036	LC_IGP12	IgG1_G1	14	66138199	1	5.41E-16	6.90E-14	-	-0.276983	-0.2809	+	C	G	0.475856139	0.0338396	+	C	G	0.499	0.0375
rs1998036	LC_IGP_R2	IgG1_G1F/IgG1_G1	14	66138199	1	2.91E-17	8.25E-14	+	0.28875	0.2793	+	C	G	0.475856139	0.0337938	+	C	G	0.499	0.0374
rs1998036	LC_IGP_R5	IgG1_G1FN/IgG1_G1N	14	66138199	1	7.54E-14	1.10E-17	+	0.257849	0.3213	+	C	G	0.477230012	0.0341787	+	C	G	0.499	0.0375
rs1998036	LC_IGP53	IgG1_G1n	14	66138199	1	3.83E-15	1.22E-13	-	-0.271299	-0.2778	+	C	G	0.476131351	0.0341795	+	C	G	0.499	0.0375
rs1998036	LC_IGP15	IgG1_G1N	14	66138199	1	4.81E-11	6.46E-11	-	-0.227094	-0.2436	+	C	G	0.477230012	0.0342944	+	C	G	0.499	0.0373
rs1998036	LC_IGP56	IgG1_G1Nn	14	66138199	1	2.05E-11	1.35E-10	-	-0.232297	-0.2391	+	C	G	0.476131351	0.0344116	+	C	G	0.499	0.0372
rs1998036	LC_IGP_R32	IgG1_G1S1/IgG1_G1	14	66138199	1	9.76E-10	4.37E-10	+	0.209759	0.225	+	C	G	0.475520795	0.0341103	+	C	G	0.499	0.036
rs1998036	LC_IGP_R3	IgG1_G2F/IgG1_G2	14	66138199	1	2.82E-15	4.59E-15	+	0.270562	0.2918	+	C	G	0.475535758	0.033922	+	C	G	0.499	0.0372
rs1998036	LC_IGP_R8	IgG1_G2FS1/IgG1_G2S1	14	66138199	1	4.23E-10	6.96E-13	+	0.214635	0.2665	+	C	G	0.475245266	0.0341568	+	C	G	0.499	0.0371
rs1998036	LC_IGP_R26	IgG1_G2N/IgG1_G1N	14	66138199	1	1.04E-11	8.98E-09	+	0.2342	0.2093	+	C	G	0.476837037	0.0341849	+	C	G	0.499	0.0364
rs1998036	LC_IGP148	IgG2 FG0n total/G0n	14	66138199	1	4.39E-09	1.37E-13	+	0.202327	0.2742	+	C	G	0.474431342	0.0342879	+	C	G	0.4988	0.0371
rs1998036	LC_IGP97	IgG2_G0	14	66138199	1	9.84E-11	6.06E-14	-	-0.221727	-0.2786	+	C	G	0.475535758	0.0340485	+	C	G	0.499	0.0371
rs1998036	LC_IGP_R41	IgG2_G0F/IgG2_G0	14	66138199	1	3.62E-08	4.50E-13	+	0.18906	0.2669	+	C	G	0.475535758	0.0341572	+	C	G	0.499	0.0369
rs1998036	LC_IGP138	IgG2_G0n	14	66138199	1	5.89E-11	5.50E-14	-	-0.225194	-0.2794	+	C	G	0.474431342	0.0341671	+	C	G	0.4988	0.0372
rs1998036	LC_IGP_R64	IgG2_G2/IgG2_G1	14	66138199	1	4.33E-09	1.85E-12	+	0.202665	0.2564	+	C	G	0.474587821	0.0343317	+	C	G	0.499	0.0364
rs12586842	LC_IGP78	IgG1 BG0n/G0n	14	66145022	1	2.65E-14	9.37E-10	-	-0.262407	-0.2272	+	G	T	0.475090458	0.0341463	+	G	T	0.4966	0.0371
rs12586842	LC_IGP79	IgG1 BG1n/G1n	14	66145022	1	3.53E-13	6.10E-12	-	-0.251393	-0.2551	+	G	T	0.475090458	0.0342798	+	G	T	0.4966	0.0371
rs12586842	LC_IGP77	IgG1 Bn	14	66145022	1	2.93E-13	1.86E-11	-	-0.252307	-0.2503	+	G	T	0.475090458	0.0342828	+	G	T	0.4966	0.0373
rs12586842	LC_IGP86	IgG1 Bn/Fn total	14	66145022	1	3.96E-14	1.09E-11	-	-0.261238	-0.2533	+	G	T	0.475090458	0.0342335	+	G	T	0.4966	0.0373
rs12586842	LC_IGP84	IgG1 FBn/Bn total	14	66145022	1	2.79E-15	3.36E-16	+	0.273243	0.3066	+	G	T	0.475090458	0.0342474	+	G	T	0.4966	0.0376
rs12586842	LC_IGP62	IgG1 FG0n total/G0n	14	66145022	1	6.38E-18	2.59E-12	+	0.296138	0.2616	+	G	T	0.475090458	0.0339323	+	G	T	0.4966	0.0374
rs12586842	LC_IGP63	IgG1 FG1n total/G1n	14	66145022	1	6.91E-18	1.24E-14	+	0.296708	0.288	+	G	T	0.475090458	0.0340339	+	G	T	0.4966	0.0374
rs12586842	LC_IGP64	IgG1 FG2n total/G2n	14	66145022	1	3.83E-14	1.81E-15	+	0.261507	0.2955	+	G	T	0.475090458	0.0342494	+	G	T	0.4966	0.0372
rs12586842	LC_IGP68	IgG1 FG2n/G2n	14	66145022	1	1.12E-10	1.26E-08	+	0.223384	0.2105	+	G	T	0.475090458	0.0344049	+	G	T	0.4966	0.037
rs12586842	LC_IGP61	IgG1 Fn total	14	66145022	1	3.96E-17	3.18E-14	+	0.289693	0.2838	+	G	T	0.475090458	0.0340464	+	G	T	0.4966	0.0374
rs12586842	LC_IGP81	IgG1 Fn/Bn	14	66145022	1	1.35E-13	9.54E-11	+	0.255671	0.2402	+	G	T	0.475090458	0.0342478	+	G	T	0.4966	0.0371
rs12586842	LC_IGP21	IgG1 Fucosylation	14	66145022	1	1.43E-15	4.58E-13	+	0.274316	0.2701	+	G	T	0.474288533	0.0340207	+	G	T	0.4966	0.0373
rs12586842	LC_IGP28	IgG1 G1S1/(G1+G1S1)	14	66145022	1	4.09E-10	2.70E-10	+	0.214132	0.2265	+	G	T	0.474492966	0.034048	+	G	T	0.4966	0.0359
rs12586842	LC_IGP27	IgG1 GS1/(G0+G+GS1)	14	66145022	1	4.48E-09	2.80E-08	+	0.201328	0.2027	+	G	T	0.474492966	0.034138	+	G	T	0.4966	0.0365
rs12586842	LC_IGP11	IgG1_G0	14	66145022	1	1.36E-19	2.29E-14	-	-0.30792	-0.2838	+	G	T	0.474829566	0.0335721	+	G	T	0.4966	0.0372
rs12586842	LC_IGP_R1	IgG1_G0F/IgG1_G0	14	66145022	1	5.78E-17	5.52E-12	+	0.284979	0.2568	+	G	T	0.474829566	0.03368	+	G	T	0.4966	0.0373
rs12586842	LC_IGP_R4	IgG1_G0FN/IgG1_G0N	14	66145022	1	7.53E-15	3.98E-13	+	0.26652	0.2722	+	G	T	0.475380282	0.0339554	+	G	T	0.4966	0.0375
rs12586842	LC_IGP52	IgG1_G0n	14	66145022	1	1.11E-19	1.99E-14	-	-0.311048	-0.285	+	G	T	0.475090458	0.0338237	+	G	T	0.4966	0.0372
rs12586842	LC_IGP14	IgG1_G0N	14	66145022	1	4.04E-17	3.99E-11	-	-0.287813	-0.2445	+	G	T	0.475380282	0.0338399	+	G	T	0.4966	0.037
rs12586842	LC_IGP55	IgG1_G0Nn	14	66145022	1	1.62E-17	4.15E-11	-	-0.293136	-0.2445	+	G	T	0.475090458	0.0340219	+	G	T	0.4966	0.0371
rs12586842	LC_IGP12	IgG1_G1	14	66145022	1	2.55E-16	9.14E-14	-	-0.279702	-0.2784	+	G	T	0.474829566	0.033785	+	G	T	0.4966	0.0374
rs12586842	LC_IGP_R2	IgG1_G1F/IgG1_G1	14	66145022	1	1.27E-17	1.03E-13	+	0.291623	0.2771	+	G	T	0.474829566	0.0337377	+	G	T	0.4966	0.0373
rs12586842	LC_IGP_R5	IgG1_G1FN/IgG1_G1N	14	66145022	1	2.51E-14	1.44E-17	+	0.262397	0.3188	+	G	T	0.476196802	0.0341156	+	G	T	0.4966	0.0374
rs12586842	LC_IGP53	IgG1_G1n	14	66145022	1	1.70E-15	1.57E-13	-	-0.274394	-0.2755	+	G	T	0.475090458	0.0341221	+	G	T	0.4966	0.0373
rs12586842	LC_IGP15	IgG1_G1N	14	66145022	1	2.28E-11	8.20E-11	-	-0.230574	-0.2412	+	G	T	0.476196802	0.0342385	+	G	T	0.4966	0.0371
rs12586842	LC_IGP56	IgG1_G1Nn	14	66145022	1	8.95E-12	1.68E-10	-	-0.236114	-0.2368	+	G	T	0.475090458	0.0343536	+	G	T	0.4966	0.0371
rs12586842	LC_IGP_R32	IgG1_G1S1/IgG1_G1	14	66145022	1	7.02E-10	4.07E-10	+	0.211288	0.2245	+	G	T	0.474492966	0.0340641	+	G	T	0.4966	0.0359
rs12586842	LC_IGP_R3	IgG1_G2F/IgG1_G2	14	66145022	1	1.38E-15	5.89E-15	+	0.273231	0.2895	+	G	T	0.474508557	0.033868	+	G	T	0.4966	0.0371
rs12586842	LC_IGP_R8	IgG1_G2FS1/IgG1_G2S1	14	66145022	1	1.85E-10	9.20E-13	+	0.218729	0.2642	+	G	T	0.474218693	0.0341005	+	G	T	0.4966	0.037
rs12586842	LC_IGP_R26	IgG1_G2N/IgG1_G1N	14	66145022	1	7.98E-12	9.84E-09	+	0.235208	0.2078	+	G	T	0.4758	0.034139	+	G	T	0.4966	0.0362
rs12586842	LC_IGP148	IgG2 FG0n total/G0n	14	66145022	1	2.39E-09	2.07E-13	+	0.205511	0.2715	+	G	T	0.473396552	0.0342355	+	G	T	0.4966	0.037
rs12586842	LC_IGP97	IgG2_G0	14	66145022	1	5.19E-11	1.20E-13	-	-0.224713	-0.2746	+	G	T	0.474508557	0.033996	+	G	T	0.4966	0.037
rs12586842	LC_IGP_R41	IgG2_G0F/IgG2_G0	14	66145022	1	1.90E-08	8.36E-13	+	0.192644	0.2632	+	G	T	0.474508557	0.0341046	+	G	T	0.4966	0.0368
rs12586842	LC_IGP138	IgG2_G0n	14	66145022	1	2.97E-11	8.26E-14	-	-0.228377	-0.2768	+	G	T	0.473396552	0.0341134	+	G	T	0.4966	0.0371
rs12586842	LC_IGP_R64	IgG2_G2/IgG2_G1	14	66145022	1	2.73E-09	2.90E-12	+	0.205028	0.2534	+	G	T	0.473559364	0.0342827	+	G	T	0.4966	0.0363
rs2268959	LC_IGP61	IgG1 Fn total	14	66145318	1	4.91E-08	1.45E-06	-	-0.23616	-0.245	+	A	C	0.203310651	0.0430899	+	A	C	0.1669	0.0508
rs12883382	LC_IGP78	IgG1 BG0n/G0n	14	66145396	1	3.30E-14	1.83E-09	-	-0.261615	-0.2236	+	T	C	0.474893742	0.0341735	+	T	C	0.4981	0.0372
rs12883382	LC_IGP79	IgG1 BG1n/G1n	14	66145396	1	4.26E-13	1.28E-11	-	-0.250693	-0.2516	+	T	C	0.474893742	0.0343064	+	T	C	0.4981	0.0372
rs12883382	LC_IGP32	IgG1 BG1S1/(BG1+BG1S1)	14	66145396	1	4.22E-08	5.58E-12	+	0.188329	0.251	+	T	C	0.475942972	0.0341943	+	T	C	0.4981	0.0364
rs12883382	LC_IGP77	IgG1 Bn	14	66145396	1	3.59E-13	3.93E-11	-	-0.251533	-0.2466	+	T	C	0.474893742	0.0343097	+	T	C	0.4981	0.0373
rs12883382	LC_IGP86	IgG1 Bn/Fn total	14	66145396	1	4.97E-14	2.35E-11	-	-0.260409	-0.2496	+	T	C	0.474893742	0.0342609	+	T	C	0.4981	0.0374
rs12883382	LC_IGP84	IgG1 FBn/Bn total	14	66145396	1	3.31E-15	8.56E-16	+	0.272692	0.3027	+	T	C	0.474893742	0.0342737	+	T	C	0.4981	0.0376
rs12883382	LC_IGP62	IgG1 FG0n total/G0n	14	66145396	1	9.28E-18	5.69E-12	+	0.294894	0.2579	+	T	C	0.474893742	0.0339625	+	T	C	0.4981	0.0374
rs12883382	LC_IGP63	IgG1 FG1n total/G1n	14	66145396	1	9.85E-18	2.93E-14	+	0.295532	0.2844	+	T	C	0.474893742	0.0340639	+	T	C	0.4981	0.0374
rs12883382	LC_IGP64	IgG1 FG2n total/G2n	14	66145396	1	5.57E-14	3.77E-15	+	0.260024	0.2927	+	T	C	0.474893742	0.0342799	+	T	C	0.4981	0.0372
rs12883382	LC_IGP68	IgG1 FG2n/G2n	14	66145396	1	1.63E-10	1.99E-08	+	0.221576	0.2081	+	T	C	0.474893742	0.0344356	+	T	C	0.4981	0.0371
rs12883382	LC_IGP61	IgG1 Fn total	14	66145396	1	5.82E-17	7.31E-14	+	0.288353	0.2802	+	T	C	0.474893742	0.034077	+	T	C	0.4981	0.0374
rs12883382	LC_IGP81	IgG1 Fn/Bn	14	66145396	1	1.71E-13	1.97E-10	+	0.254781	0.2365	+	T	C	0.474893742	0.0342754	+	T	C	0.4981	0.0372
rs12883382	LC_IGP21	IgG1 Fucosylation	14	66145396	1	2.10E-15	1.04E-12	+	0.272898	0.2664	+	T	C	0.474093403	0.0340511	+	T	C	0.4981	0.0374
rs12883382	LC_IGP28	IgG1 G1S1/(G1+G1S1)	14	66145396	1	2.98E-10	5.03E-10	+	0.215943	0.2235	+	T	C	0.474299388	0.0340638	+	T	C	0.4981	0.0359
rs12883382	LC_IGP27	IgG1 GS1/(G0+G+GS1)	14	66145396	1	2.49E-09	5.34E-08	+	0.204738	0.1985	+	T	C	0.474299388	0.0341484	+	T	C	0.4981	0.0365
rs12883382	LC_IGP11	IgG1_G0	14	66145396	1	1.74E-19	5.78E-14	-	-0.307211	-0.2795	+	T	C	0.474636225	0.0335991	+	T	C	0.4981	0.0372
rs12883382	LC_IGP_R1	IgG1_G0F/IgG1_G0	14	66145396	1	8.67E-17	1.20E-11	+	0.283566	0.2531	+	T	C	0.474636225	0.0337103	+	T	C	0.4981	0.0373
rs12883382	LC_IGP_R4	IgG1_G0FN/IgG1_G0N	14	66145396	1	8.74E-15	9.01E-13	+	0.266056	0.2685	+	T	C	0.475186467	0.0339808	+	T	C	0.4981	0.0376
rs12883382	LC_IGP52	IgG1_G0n	14	66145396	1	1.50E-19	5.11E-14	-	-0.310154	-0.2807	+	T	C	0.474893742	0.0338523	+	T	C	0.4981	0.0373
rs12883382	LC_IGP14	IgG1_G0N	14	66145396	1	4.45E-17	8.88E-11	-	-0.287619	-0.2402	+	T	C	0.475186467	0.0338641	+	T	C	0.4981	0.037
rs12883382	LC_IGP55	IgG1_G0Nn	14	66145396	1	1.88E-17	9.32E-11	-	-0.292751	-0.2402	+	T	C	0.474893742	0.0340475	+	T	C	0.4981	0.0371
rs12883382	LC_IGP12	IgG1_G1	14	66145396	1	3.53E-16	1.95E-13	-	-0.278577	-0.2752	+	T	C	0.474636225	0.0338137	+	T	C	0.4981	0.0374
rs12883382	LC_IGP_R2	IgG1_G1F/IgG1_G1	14	66145396	1	1.89E-17	2.36E-13	+	0.290294	0.2735	+	T	C	0.474636225	0.0337678	+	T	C	0.4981	0.0373
rs12883382	LC_IGP_R5	IgG1_G1FN/IgG1_G1N	14	66145396	1	2.96E-14	3.82E-17	+	0.261842	0.315	+	T	C	0.476002153	0.0341414	+	T	C	0.4981	0.0374
rs12883382	LC_IGP53	IgG1_G1n	14	66145396	1	2.46E-15	3.31E-13	-	-0.273023	-0.2723	+	T	C	0.474893742	0.0341524	+	T	C	0.4981	0.0374
rs12883382	LC_IGP15	IgG1_G1N	14	66145396	1	2.69E-11	1.55E-10	-	-0.229902	-0.2381	+	T	C	0.476002153	0.0342644	+	T	C	0.4981	0.0372
rs12883382	LC_IGP56	IgG1_G1Nn	14	66145396	1	1.09E-11	3.12E-10	-	-0.235324	-0.2338	+	T	C	0.474893742	0.0343804	+	T	C	0.4981	0.0372
rs12883382	LC_IGP_R36	IgG1_G1NS1/IgG1_G1N	14	66145396	1	3.87E-08	6.41E-12	+	0.188899	0.2505	+	T	C	0.475942972	0.0342006	+	T	C	0.4981	0.0365
rs12883382	LC_IGP_R32	IgG1_G1S1/IgG1_G1	14	66145396	1	5.39E-10	7.53E-10	+	0.21284	0.2215	+	T	C	0.474299388	0.034081	+	T	C	0.4981	0.036
rs12883382	LC_IGP_R3	IgG1_G2F/IgG1_G2	14	66145396	1	2.08E-15	1.19E-14	+	0.271693	0.2868	+	T	C	0.474315098	0.0338986	+	T	C	0.4981	0.0372
rs12883382	LC_IGP_R8	IgG1_G2FS1/IgG1_G2S1	14	66145396	1	3.63E-10	1.59E-12	+	0.215333	0.2621	+	T	C	0.474025351	0.0341366	+	T	C	0.4981	0.0371

rs12883382	LC_IGP_R26	IgG1_G2N/IgG1_G1N	14	66145396	1	8.45E-12	1.82E-08	+	0.235085	0.2042	+	T	C	0.475604012	0.0341627	+	T	C	0.4981	0.0363
rs12883382	LC_IGP148	IgG2 FG0n total/G0n	14	66145396	1	3.34E-09	5.27E-13	+	0.203769	0.267	+	T	C	0.473193042	0.0342652	+	T	C	0.4981	0.037
rs12883382	LC_IGP97	IgG2_G0	14	66145396	1	8.20E-11	3.30E-13	-	-0.222542	-0.2696	+	T	C	0.474315098	0.0340276	+	T	C	0.4981	0.037
rs12883382	LC_IGP_R41	IgG2_G0F/IgG2_G0	14	66145396	1	2.86E-08	2.14E-12	+	0.19037	0.2584	+	T	C	0.474315098	0.0341352	+	T	C	0.4981	0.0368
rs12883382	LC_IGP138	IgG2_G0n	14	66145396	1	4.77E-11	2.29E-13	-	-0.226151	-0.2717	+	T	C	0.473193042	0.0341458	+	T	C	0.4981	0.0371
rs12883382	LC_IGP_R64	IgG2_G2/IgG2_G1	14	66145396	1	4.44E-09	7.26E-12	+	0.202425	0.2486	+	T	C	0.473357099	0.0343153	+	T	C	0.4981	0.0363
rs2268961	LC_IGP78	IgG1 BG0n/G0n	14	66146765	1	2.86E-14	9.65E-10	-	-0.26209	-0.2271	+	C	T	0.474474907	0.0341505	+	C	T	0.4965	0.0371
rs2268961	LC_IGP79	IgG1 BG1n/G1n	14	66146765	1	3.81E-13	6.38E-12	-	-0.251062	-0.2549	+	C	T	0.474474907	0.034284	+	C	T	0.4965	0.0371
rs2268961	LC_IGP32	IgG1 BG1S1/(BG1+BG1S1)	14	66146765	1	4.15E-08	2.74E-12	+	0.188321	0.2543	+	C	T	0.475525586	0.0341738	+	C	T	0.4965	0.0364
rs2268961	LC_IGP77	IgG1 Bn	14	66146765	1	3.16E-13	1.94E-11	-	-0.251967	-0.2501	+	C	T	0.474474907	0.034287	+	C	T	0.4965	0.0373
rs2268961	LC_IGP86	IgG1 Bn/Fn total	14	66146765	1	4.36E-14	1.14E-11	-	-0.260838	-0.2531	+	C	T	0.474474907	0.0342381	+	C	T	0.4965	0.0373
rs2268961	LC_IGP84	IgG1 FBn/Bn total	14	66146765	1	2.82E-15	3.51E-16	+	0.273205	0.3064	+	C	T	0.474474907	0.0342503	+	C	T	0.4965	0.0376
rs2268961	LC_IGP62	IgG1 FG0n total/G0n	14	66146765	1	8.06E-18	2.68E-12	+	0.295262	0.2614	+	C	T	0.474474907	0.0339396	+	C	T	0.4965	0.0374
rs2268961	LC_IGP63	IgG1 FG1n total/G1n	14	66146765	1	8.58E-18	1.30E-14	+	0.295893	0.2878	+	C	T	0.474474907	0.034041	+	C	T	0.4965	0.0374
rs2268961	LC_IGP64	IgG1 FG2n total/G2n	14	66146765	1	5.10E-14	1.91E-15	+	0.260262	0.2952	+	C	T	0.474474907	0.0342579	+	C	T	0.4965	0.0372
rs2268961	LC_IGP68	IgG1 FG2n/G2n	14	66146765	1	1.57E-10	1.31E-08	+	0.221633	0.2102	+	C	T	0.474474907	0.0344146	+	C	T	0.4965	0.037
rs2268961	LC_IGP61	IgG1 Fn total	14	66146765	1	5.09E-17	3.33E-14	+	0.288721	0.2836	+	C	T	0.474474907	0.0340542	+	C	T	0.4965	0.0374
rs2268961	LC_IGP81	IgG1 Fn/Bn	14	66146765	1	1.50E-13	9.93E-11	+	0.255213	0.24	+	C	T	0.474474907	0.0342526	+	C	T	0.4965	0.0371
rs2268961	LC_IGP21	IgG1 Fucosylation	14	66146765	1	1.79E-15	4.79E-13	+	0.27341	0.2698	+	C	T	0.473676017	0.0340279	+	C	T	0.4965	0.0373
rs2268961	LC_IGP28	IgG1 G1S1/(G1+G1S1)	14	66146765	1	2.89E-10	2.76E-10	+	0.215971	0.2264	+	C	T	0.473885321	0.0340435	+	C	T	0.4965	0.0359
rs2268961	LC_IGP27	IgG1 GS1/(G0+G+GS1)	14	66146765	1	2.37E-09	2.82E-08	+	0.204893	0.2026	+	C	T	0.473885321	0.0341277	+	C	T	0.4965	0.0365
rs2268961	LC_IGP11	IgG1_G0	14	66146765	1	1.61E-19	2.34E-14	-	-0.307329	-0.2837	+	C	T	0.474222663	0.033578	+	C	T	0.4965	0.0372
rs2268961	LC_IGP_R1	IgG1_G0F/IgG1_G0	14	66146765	1	7.67E-17	5.72E-12	+	0.283887	0.2566	+	C	T	0.474222663	0.0336882	+	C	T	0.4965	0.0373
rs2268961	LC_IGP_R4	IgG1_G0FN/IgG1_G0N	14	66146765	1	7.63E-15	4.11E-13	+	0.266481	0.2721	+	C	T	0.474771892	0.0339582	+	C	T	0.4965	0.0375
rs2268961	LC_IGP52	IgG1_G0n	14	66146765	1	1.37E-19	2.04E-14	-	-0.31031	-0.2849	+	C	T	0.474474907	0.0338305	+	C	T	0.4965	0.0372
rs2268961	LC_IGP14	IgG1_G0N	14	66146765	1	3.95E-17	4.06E-11	-	-0.287922	-0.2444	+	C	T	0.474771892	0.0338419	+	C	T	0.4965	0.037
rs2268961	LC_IGP55	IgG1_G0Nn	14	66146765	1	1.65E-17	4.23E-11	-	-0.293103	-0.2445	+	C	T	0.474474907	0.0340247	+	C	T	0.4965	0.0371
rs2268961	LC_IGP12	IgG1_G1	14	66146765	1	3.12E-16	9.58E-14	-	-0.278915	-0.2781	+	C	T	0.474222663	0.0337916	+	C	T	0.4965	0.0374
rs2268961	LC_IGP_R2	IgG1_G1F/IgG1_G1	14	66146765	1	1.66E-17	1.09E-13	+	0.290616	0.2769	+	C	T	0.474222663	0.0337457	+	C	T	0.4965	0.0373
rs2268961	LC_IGP_R5	IgG1_G1FN/IgG1_G1N	14	66146765	1	2.64E-14	1.51E-17	+	0.262193	0.3186	+	C	T	0.475585793	0.034119	+	C	T	0.4965	0.0374
rs2268961	LC_IGP53	IgG1_G1n	14	66146765	1	2.18E-15	1.65E-13	-	-0.27338	-0.2752	+	C	T	0.474474907	0.0341298	+	C	T	0.4965	0.0373
rs2268961	LC_IGP15	IgG1_G1N	14	66146765	1	2.44E-11	8.57E-11	-	-0.230253	-0.241	+	C	T	0.475585793	0.0342422	+	C	T	0.4965	0.0371
rs2268961	LC_IGP56	IgG1_G1Nn	14	66146765	1	9.75E-12	1.76E-10	-	-0.235711	-0.2366	+	C	T	0.474474907	0.034358	+	C	T	0.4965	0.0371
rs2268961	LC_IGP_R36	IgG1_G1NS1/IgG1_G1N	14	66146765	1	3.79E-08	3.14E-12	+	0.188908	0.2538	+	C	T	0.475525586	0.03418	+	C	T	0.4965	0.0364
rs2268961	LC_IGP_R32	IgG1_G1S1/IgG1_G1	14	66146765	1	5.21E-10	4.15E-10	+	0.212898	0.2244	+	C	T	0.473885321	0.0340606	+	C	T	0.4965	0.0359
rs2268961	LC_IGP_R3	IgG1_G2F/IgG1_G2	14	66146765	1	1.93E-15	6.19E-15	+	0.271848	0.2892	+	C	T	0.473901284	0.0338775	+	C	T	0.4965	0.0371
rs2268961	LC_IGP_R8	IgG1_G2FS1/IgG1_G2S1	14	66146765	1	3.55E-10	9.62E-13	+	0.215321	0.264	+	C	T	0.47361179	0.0341165	+	C	T	0.4965	0.037
rs2268961	LC_IGP_R26	IgG1_G2N/IgG1_G1N	14	66146765	1	8.36E-12	1.00E-08	+	0.234996	0.2077	+	C	T	0.475186728	0.0341424	+	C	T	0.4965	0.0362
rs2268961	LC_IGP148	IgG2 FG0n total/G0n	14	66146765	1	3.09E-09	2.17E-13	+	0.204095	0.2713	+	C	T	0.472784791	0.0342436	+	C	T	0.4965	0.037
rs2268961	LC_IGP97	IgG2_G0	14	66146765	1	7.66E-11	1.26E-13	-	-0.222762	-0.2744	+	C	T	0.473901284	0.0340066	+	C	T	0.4965	0.037
rs2268961	LC_IGP_R41	IgG2_G0F/IgG2_G0	14	66146765	1	2.65E-08	8.83E-13	+	0.190719	0.2629	+	C	T	0.473901284	0.0341138	+	C	T	0.4965	0.0368
rs2268961	LC_IGP138	IgG2_G0n	14	66146765	1	4.43E-11	8.71E-14	-	-0.226397	-0.2766	+	C	T	0.472784791	0.0341244	+	C	T	0.4965	0.0371
rs2268961	LC_IGP_R64	IgG2_G2/IgG2_G1	14	66146765	1	4.69E-09	3.01E-12	+	0.201991	0.2532	+	C	T	0.472951346	0.0342966	+	C	T	0.4965	0.0363
rs2268962	LC_IGP78	IgG1 BG0n/G0n	14	66147273	1	2.86E-14	1.01E-09	-	-0.262088	-0.2273	+	G	A	0.47447119	0.0341504	+	G	A	0.4956	0.0372
rs2268962	LC_IGP79	IgG1 BG1n/G1n	14	66147273	1	3.82E-13	7.17E-12	-	-0.251052	-0.2547	+	G	A	0.47447119	0.0342839	+	G	A	0.4956	0.0372
rs2268962	LC_IGP32	IgG1 BG1S1/(BG1+BG1S1)	14	66147273	1	4.16E-08	3.02E-12	+	0.188311	0.2542	+	G	A	0.475521887	0.0341738	+	G	A	0.4956	0.0364
rs2268962	LC_IGP77	IgG1 Bn	14	66147273	1	3.17E-13	2.10E-11	-	-0.25196	-0.2501	+	G	A	0.47447119	0.0342869	+	G	A	0.4956	0.0373
rs2268962	LC_IGP86	IgG1 Bn/Fn total	14	66147273	1	4.36E-14	1.24E-11	-	-0.260831	-0.2531	+	G	A	0.47447119	0.034238	+	G	A	0.4956	0.0374
rs2268962	LC_IGP84	IgG1 FBn/Bn total	14	66147273	1	2.83E-15	3.82E-16	+	0.2732	0.3065	+	G	A	0.47447119	0.0342502	+	G	A	0.4956	0.0376
rs2268962	LC_IGP62	IgG1 FG0n total/G0n	14	66147273	1	8.07E-18	2.78E-12	+	0.295257	0.2617	+	G	A	0.47447119	0.0339396	+	G	A	0.4956	0.0374
rs2268962	LC_IGP63	IgG1 FG1n total/G1n	14	66147273	1	8.60E-18	1.39E-14	+	0.295882	0.288	+	G	A	0.47447119	0.034041	+	G	A	0.4956	0.0374
rs2268962	LC_IGP64	IgG1 FG2n total/G2n	14	66147273	1	5.11E-14	2.04E-15	+	0.260253	0.2954	+	G	A	0.47447119	0.0342579	+	G	A	0.4956	0.0372
rs2268962	LC_IGP68	IgG1 FG2n/G2n	14	66147273	1	1.57E-10	1.39E-08	+	0.221623	0.2103	+	G	A	0.47447119	0.0344146	+	G	A	0.4956	0.0371
rs2268962	LC_IGP61	IgG1 Fn total	14	66147273	1	5.09E-17	3.53E-14	+	0.288715	0.2838	+	G	A	0.47447119	0.0340541	+	G	A	0.4956	0.0375
rs2268962	LC_IGP81	IgG1 Fn/Bn	14	66147273	1	1.51E-13	1.07E-10	+	0.255207	0.2401	+	G	A	0.47447119	0.0342525	+	G	A	0.4956	0.0372
rs2268962	LC_IGP21	IgG1 Fucosylation	14	66147273	1	1.79E-15	5.17E-13	+	0.273401	0.27	+	G	A	0.473672318	0.0340279	+	G	A	0.4956	0.0374
rs2268962	LC_IGP28	IgG1 G1S1/(G1+G1S1)	14	66147273	1	2.90E-10	3.03E-10	+	0.215954	0.2263	+	G	A	0.473881651	0.0340435	+	G	A	0.4956	0.0359
rs2268962	LC_IGP27	IgG1 GS1/(G0+G+GS1)	14	66147273	1	2.38E-09	2.78E-08	+	0.204869	0.203	+	G	A	0.473881651	0.0341277	+	G	A	0.4956	0.0366
rs2268962	LC_IGP11	IgG1_G0	14	66147273	1	1.62E-19	2.37E-14	-	-0.307306	-0.2841	+	G	A	0.474218998	0.0335781	+	G	A	0.4956	0.0372
rs2268962	LC_IGP_R1	IgG1_G0F/IgG1_G0	14	66147273	1	7.68E-17	5.91E-12	+	0.283881	0.2569	+	G	A	0.474218998	0.0336882	+	G	A	0.4956	0.0373
rs2268962	LC_IGP_R4	IgG1_G0FN/IgG1_G0N	14	66147273	1	7.64E-15	4.29E-13	+	0.266478	0.2724	+	G	A	0.474768218	0.0339581	+	G	A	0.4956	0.0376
rs2268962	LC_IGP52	IgG1_G0n	14	66147273	1	1.38E-19	2.06E-14	-	-0.310287	-0.2853	+	G	A	0.47447119	0.0338306	+	G	A	0.4956	0.0373
rs2268962	LC_IGP14	IgG1_G0N	14	66147273	1	3.97E-17	4.18E-11	-	-0.287905	-0.2447	+	G	A	0.474768218	0.0338419	+	G	A	0.4956	0.0371
rs2268962	LC_IGP55	IgG1_G0Nn	14	66147273	1	1.65E-17	4.36E-11	-	-0.293087	-0.2447	+	G	A	0.47447119	0.0340247	+	G	A	0.4956	0.0371
rs2268962	LC_IGP12	IgG1_G1	14	66147273	1	3.12E-16	9.92E-14	-	-0.278909	-0.2785	+	G	A	0.474218998	0.0337916	+	G	A	0.4956	0.0374
rs2268962	LC_IGP_R2	IgG1_G1F/IgG1_G1	14	66147273	1	1.67E-17	1.14E-13	+	0.290605	0.2771	+	G	A	0.474218998	0.0337457	+	G	A	0.4956	0.0373
rs2268962	LC_IGP_R5	IgG1_G1FN/IgG1_G1N	14	66147273	1	2.64E-14	1.70E-17	+	0.262183	0.3186	+	G	A	0.475582103	0.0341189	+	G	A	0.4956	0.0374
rs2268962	LC_IGP53	IgG1_G1n	14	66147273	1	2.18E-15	1.71E-13	-	-0.273375	-0.2756	+	G	A	0.47447119	0.0341298	+	G	A	0.4956	0.0374
rs2268962	LC_IGP15	IgG1_G1N	14	66147273	1	2.44E-11	9.52E-11	-	-0.230247	-0.2408	+	G	A	0.475582103	0.0342422	+	G	A	0.4956	0.0372
rs2268962	LC_IGP56	IgG1_G1Nn	14	66147273	1	9.76E-12	1.95E-10	-	-0.235706	-0.2364	+	G	A	0.47447119	0.0343579	+	G	A	0.4956	0.0371
rs2268962	LC_IGP_R36	IgG1_G1NS1/IgG1_G1N	14	66147273	1	3.80E-08	3.47E-12	+	0.188897	0.2538	+	G	A	0.475521887	0.03418	+	G	A	0.4956	0.0365
rs2268962	LC_IGP_R32	IgG1_G1S1/IgG1_G1	14	66147273	1	5.22E-10	4.55E-10	+	0.212881	0.2243	+	G	A	0.473881651	0.0340606	+	G	A	0.4956	0.036
rs2268962	LC_IGP_R3	IgG1_G2F/IgG1_G2	14	66147273	1	1.93E-15	6.63E-15	+	0.271839	0.2894	+	G	A	0.473897616	0.0338775	+	G	A	0.4956	0.0372
rs2268962	LC_IGP_R8	IgG1_G2FS1/IgG1_G2S1	14	66147273	1	3.56E-10	1.04E-12	+	0.215315	0.264	+	G	A	0.473608125	0.0341164	+	G	A	0.4956	0.0371
rs2268962	LC_IGP_R26	IgG1_G2N/IgG1_G1N	14	66147273	1	8.39E-12	1.08E-08	+	0.234978	0.2076	+	G	A	0.475183025	0.0341424	+	G	A	0.4956	0.0363
rs2268962	LC_IGP148	IgG2 FG0n total/G0n	14	66147273	1	3.08E-09	2.18E-13	+	0.2041	0.2718	+	G	A	0.472781096	0.0342435	+	G	A	0.4957	0.037
rs2268962	LC_IGP97	IgG2_G0	14	66147273	1	7.67E-11	1.27E-13	-	-0.222751	-0.2748	+	G	A	0.473897616	0.0340065	+	G	A	0.4956	0.0371
rs2268962	LC_IGP_R41	IgG2_G0F/IgG2_G0	14	66147273	1	2.64E-08	8.94E-13	+	0.190725	0.2633	+	G	A	0.473897616	0.0341137	+	G	A	0.4956	0.0368
rs2268962	LC_IGP138	IgG2_G0n	14	66147273	1	4.43E-11	8.75E-14	-	-0.226388	-0.277	+	G	A	0.472781096	0.0341243	+	G	A	0.4957	0.0371
rs2268962	LC_IGP_R64	IgG2_G2/IgG2_G1	14	66147273	1	4.71E-09	2.90E-12	+	0.201965	0.2538	+	G	A	0.472947674	0.0342966	+	G	A	0.4956	0.0363
rs2064694	LC_IGP78	IgG1 BG0n/G0n	14	66148246	1	2.86E-14	9.66E-10	-	-0.262078	-0.2271	+	T	G	0.474464994	0.0341502	+	T	G	0.4965	0.0371
rs2064694	LC_IGP79	IgG1 BG1n/G1n	14	66148246	1	3.84E-13	6.39E-12	-	-0.251027	-0.2549	+	T	G	0.474464994	0.0342838	+	T	G	0.4965	0.0371
rs2064694	LC_IGP32	IgG1 BG1S1/(BG1+BG1S1)	14	66148246	1	4.18E-08	2.75E-12	+	0.188286	0.2543	+	T	G	0.475515721	0.0341736	+	T	G	0.4965	0.0364
rs2064694	LC_IGP77	IgG1 Bn	14	66148246	1	3.18E-13	1.94E-11	-	-0.251941	-0.2501	+	T	G	0.474464994	0.0342867	+	T	G	0.4965	0.0373
rs2064694	LC_IGP86	IgG1 Bn/Fn total	14	66148246	1	4.38E-14	1.14E-11	-	-0.260812	-0.2531	+	T	G	0.474464994	0.0342378	+	T	G	0.4965	0.0373
rs2064694	LC_IGP84	IgG1 FBn/Bn total	14	66148246	1	2.84E-15	3.52E-16	+	0.273183	0.3064	+	T	G	0.474464994	0.03425	+	T	G	0.4965	0.0376
rs2064694	LC_IGP62	IgG1 FG0n total/G0n	14	66148246	1	8.10E-18	2.68E-12	+	0.295239	0.2614	+	T	G	0.474464994	0.0339394	+	T	G	0.4965	0.0374
rs2064694	LC_IGP63	IgG1 FG1n total/G1n	14	66148246	1	8.66E-18	1.30E-14	+	0.295854	0.2878	+	T	G	0.474464994	0.0340409	+	T	G	0.4965	0.0374
rs2064694	LC_IGP64	IgG1 FG2n total/G2n	14	66148246	1	5.14E-14	1.91E-15	+	0.260227	0.2952	+	T	G	0.474464994	0.0342577	+	T	G	0.4965	0.0372
rs2064694	LC_IGP68	IgG1 FG2n/G2n	14	66148246	1	1.58E-10	1.31E-08	+	0.221599	0.2102	+	T	G	0.474464994	0.0344144	+	T	G	0.4965	0.037
rs2064694	LC_IGP61	IgG1 Fn total	14	66148246	1	5.11E-17	3.34E-14	+	0.288696	0.2836	+	T	G	0.474464994	0.034054	+	T	G	0.4965	0.0374
rs2064694	LC_IGP81	IgG1 Fn/Bn	14	66148246	1	1.51E-13	9.94E-11	+	0.255187	0.24	+	T	G	0.474464994	0.0342523	+	T	G	0.4965	0.0371
rs2064694	LC_IGP21	IgG1 Fucosylation	14	66148246	1	1.80E-15	4.79E-13	+	0.273378	0.2698	+	T	G	0.473666153	0.0340277	+	T	G	0.4965	0.0373
rs2064694	LC_IGP28	IgG1 G1S1/(G1+G1S1)	14	66148246	1	2.92E-10	2.76E-10	+	0.215917	0.2264	+	T	G	0.473875535	0.0340434	+	T	G	0.4965	0.0359
rs2064694	LC_IGP27	IgG1 GS1/(G0+G+GS1)	14	66148246	1	2.40E-09	2.82E-08	+	0.204818	0.2026	+	T	G	0.473875535	0.0341276	+	T	G	0.4965	0.0365
rs2064694	LC_IGP11	IgG1_G0	14	66148246	1	1.64E-19	2.34E-14	-	-0.307255	-0.2837	+	T	G	0.474212889	0.0335781	+	T	G	0.4965	0.0372
rs2064694	LC_IGP_R1	IgG1_G0F/IgG1_G0	14	66148246	1	7.71E-17	5.72E-12	+	0.283863	0.2566	+	T	G	0.474212889	0.033688	+	T	G	0.4965	0.0373
rs2064694	LC_IGP_R4	IgG1_G0FN/IgG1_G0N	14	66148246	1	7.66E-15	4.11E-13	+	0.266465	0.2721	+	T	G	0.474762094	0.0339579	+	T	G	0.4965	0.0375
rs2064694	LC_IGP52	IgG1_G0n	14	66148246	1	1.40E-19	2.04E-14	-	-0.310239	-0.2849	+	T	G	0.474464994	0.0338306	+	T	G	0.4965	0.0372
rs2064694	LC_IGP14	IgG1_G0N	14	66148246	1	4.00E-17	4.06E-11	-	-0.287867	-0.2444	+	T	G	0.474762094	0.0338418	+	T	G	0.4965	0.037
rs2064694	LC_IGP55	IgG1_G0Nn	14	66148246	1	1.67E-17	4.23E-11	-	-0.293052	-0.2445	+	T	G	0.474464994	0.0340246	+	T	G	0.4965	0.0371
rs2064694	LC_IGP12	IgG1_G1	14	66148246	1	3.14E-16	9.58E-14	-	-0.27889	-0.2781	+	T	G	0.474212889	0.0337914	+	T	G	0.4965	0.0374
rs2064694	LC_IGP_R2	IgG1_G1F/IgG1_G1	14	66148246	1	1.68E-17	1.09E-13	+	0.290578	0.2769	+	T	G	0.474212889	0.0337455	+	T	G	0.4965	0.0373
rs2064694	LC_IGP_R5	IgG1_G1FN/IgG1_G1N	14	66148246	1	2.66E-14	1.51E-17	+	0.262156	0.3186	+	T	G	0.475575953	0.0341188	+	T	G	0.4965	0.0374
rs2064694	LC_IGP53	IgG1_G1n	14	66148246	1	2.19E-15	1.65E-13	-	-0.273359	-0.2752	+	T	G	0.474464994	0.0341296	+	T	G	0.4965	0.0373
rs2064694	LC_IGP15	IgG1_G1N	14	66148246	1	2.45E-11	8.57E-11	-	-0.230228	-0.241	+	T	G	0.475575953	0.034242	+	T	G	0.4965	0.0371
rs2064694	LC_IGP56	IgG1_G1Nn	14	66148246	1	9.79E-12	1.76E-10	-	-0.235689	-0.2366	+	T	G	0.474464994	0.0343577	+	T	G	0.4965	0.0371
rs2064694	LC_IGP_R36	IgG1_G1NS1/IgG1_G1N	14	66148246	1	3.82E-08	3.14E-12	+	0.188871	0.2538	+	T	G	0.475515721	0.0341798	+	T	G	0.4965	0.0364
rs2064694	LC_IGP_R32	IgG1_G1S1/IgG1_G1	14	66148246	1	5.26E-10	4.16E-10	+	0.212843	0.2244	+	T	G	0.473875535	0.0340605	+	T	G	0.4965	0.0359
rs2064694	LC_IGP_R3	IgG1_G2F/IgG1_G2	14	66148246	1	1.94E-15	6.20E-15	+	0.271815	0.2892	+	T	G	0.473891504	0.0338773	+	T	G	0.4965	0.0371
rs2064694	LC_IGP_R8	IgG1_G2FS1/IgG1_G2S1	14	66148246	1	3.57E-10	9.62E-13	+	0.2153	0.264	+	T	G	0.473602016	0.0341162	+	T	G	0.4965	0.037
rs2064694	LC_IGP_R26	IgG1_G2N/IgG1_G1N	14	66148246	1	8.45E-12	1.00E-08	+	0.234942	0.2077	+	T	G	0.475176852	0.0341423	+	T	G	0.4965	0.0362
rs2064694	LC_IGP148	IgG2 FG0n total/G0n	14	66148246	1	3.08E-09	2.17E-13	+	0.204102	0.2713	+	T	G	0.472774938	0.0342433	+	T	G	0.4965	0.037
rs2064694	LC_IGP97	IgG2_G0	14	66148246	1	7.71E-11	1.26E-13	-	-0.222725	-0.2744	+	T	G	0.473891504	0.0340064	+	T	G	0.4965	0.037
rs2064694	LC_IGP_R41	IgG2_G0F/IgG2_G0	14	66148246	1	2.64E-08	8.84E-13	+	0.190731	0.2629	+	T	G	0.473891504	0.0341134	+	T	G	0.4965	0.0368
rs2064694	LC_IGP138	IgG2_G0n	14	66148246	1	4.45E-11	8.71E-14	-	-0.226367	-0.2766	+	T	G	0.472774938	0.0341242	+	T	G	0.4965	0.0371
rs2064694	LC_IGP_R64	IgG2_G2/IgG2_G1	14	66148246	1	4.76E-09	3.01E-12	+	0.201913	0.2532	+	T	G	0.472941554	0.0342965	+	T	G	0.4965	0.0363
rs11622271	LC_IGP78	IgG1 BG0n/G0n	14	66149065	1	4.55E-14	1.23E-09	-	-0.260047	-0.2263	+	T	A	0.4735886	0.0341611	+	T	A	0.4951	0.0372
rs11622271	LC_IGP79	IgG1 BG1n/G1n	14	66149065	1	5.47E-13	6.38E-12	-	-0.249399	-0.2557	+	T	A	0.4735886	0.0342924	+	T	A	0.4951	0.0372

rs11622271	LC_IGP32	IgG1 BG1S1/(BG1+BG1S1)	14	66149065	1	4.57E-08	2.76E-12	+	0.187752	0.255	+	T	A	0.47464365	0.0341764	+	T	A	0.4951	0.0365
rs11622271	LC_IGP77	IgG1 Bn	14	66149065	1	4.76E-13	2.18E-11	-	-0.250097	-0.2502	+	T	A	0.4735886	0.0342964	+	T	A	0.4951	0.0374
rs11622271	LC_IGP86	IgG1 Bn/Fn total	14	66149065	1	6.71E-14	1.31E-11	-	-0.258928	-0.2531	+	T	A	0.4735886	0.034248	+	T	A	0.4951	0.0374
rs11622271	LC_IGP84	IgG1 FBn/Bn total	14	66149065	1	4.50E-15	4.16E-16	+	0.271235	0.3066	+	T	A	0.4735886	0.0342609	+	T	A	0.4951	0.0377
rs11622271	LC_IGP62	IgG1 FG0n total/G0n	14	66149065	1	1.38E-17	3.21E-12	+	0.293185	0.2613	+	T	A	0.4735886	0.0339517	+	T	A	0.4951	0.0375
rs11622271	LC_IGP63	IgG1 FG1n total/G1n	14	66149065	1	1.16E-17	1.32E-14	+	0.294719	0.2886	+	T	A	0.4735886	0.0340483	+	T	A	0.4951	0.0375
rs11622271	LC_IGP64	IgG1 FG2n total/G2n	14	66149065	1	5.46E-14	1.88E-15	+	0.259968	0.2962	+	T	A	0.4735886	0.0342602	+	T	A	0.4951	0.0373
rs11622271	LC_IGP68	IgG1 FG2n/G2n	14	66149065	1	1.54E-10	1.26E-08	+	0.221742	0.2112	+	T	A	0.4735886	0.0344151	+	T	A	0.4951	0.0371
rs11622271	LC_IGP61	IgG1 Fn total	14	66149065	1	7.89E-17	3.56E-14	+	0.286983	0.2842	+	T	A	0.4735886	0.0340642	+	T	A	0.4951	0.0375
rs11622271	LC_IGP81	IgG1 Fn/Bn	14	66149065	1	2.25E-13	1.08E-10	+	0.253401	0.2403	+	T	A	0.4735886	0.0342619	+	T	A	0.4951	0.0372
rs11622271	LC_IGP21	IgG1 Fucosylation	14	66149065	1	2.66E-15	5.18E-13	+	0.271756	0.2703	+	T	A	0.47279439	0.0340372	+	T	A	0.4951	0.0374
rs11622271	LC_IGP28	IgG1 G1S1/(G1+G1S1)	14	66149065	1	2.79E-10	2.58E-10	+	0.216172	0.2274	+	T	A	0.473010398	0.0340438	+	T	A	0.4951	0.036
rs11622271	LC_IGP27	IgG1 GS1/(G0+G+GS1)	14	66149065	1	2.05E-09	3.40E-08	+	0.205712	0.2019	+	T	A	0.473010398	0.0341257	+	T	A	0.4951	0.0366
rs11622271	LC_IGP11	IgG1_G0	14	66149065	1	1.99E-19	2.76E-14	-	-0.306557	-0.2837	+	T	A	0.473348809	0.0335833	+	T	A	0.4951	0.0373
rs11622271	LC_IGP_R1	IgG1_G0F/IgG1_G0	14	66149065	1	1.23E-16	6.56E-12	+	0.282017	0.2567	+	T	A	0.473348809	0.0336988	+	T	A	0.4951	0.0374
rs11622271	LC_IGP_R4	IgG1_G0FN/IgG1_G0N	14	66149065	1	8.00E-15	5.46E-13	+	0.266243	0.2714	+	T	A	0.473714023	0.033954	+	T	A	0.4951	0.0376
rs11622271	LC_IGP52	IgG1_G0n	14	66149065	1	1.73E-19	2.37E-14	-	-0.309462	-0.285	+	T	A	0.4735886	0.0338363	+	T	A	0.4951	0.0374
rs11622271	LC_IGP14	IgG1_G0N	14	66149065	1	2.92E-17	5.41E-11	-	-0.289065	-0.2435	+	T	A	0.473714023	0.0338306	+	T	A	0.4951	0.0371
rs11622271	LC_IGP55	IgG1_G0Nn	14	66149065	1	2.25E-17	5.59E-11	-	-0.291892	-0.2436	+	T	A	0.4735886	0.0340321	+	T	A	0.4951	0.0372
rs11622271	LC_IGP12	IgG1_G1	14	66149065	1	4.30E-16	1.00E-13	-	-0.277628	-0.2788	+	T	A	0.473348809	0.0337991	+	T	A	0.4951	0.0375
rs11622271	LC_IGP_R2	IgG1_G1F/IgG1_G1	14	66149065	1	2.10E-17	1.09E-13	+	0.289718	0.2778	+	T	A	0.473348809	0.0337514	+	T	A	0.4951	0.0374
rs11622271	LC_IGP_R5	IgG1_G1FN/IgG1_G1N	14	66149065	1	3.93E-14	1.59E-17	+	0.260453	0.3194	+	T	A	0.474706027	0.0341278	+	T	A	0.4951	0.0375
rs11622271	LC_IGP53	IgG1_G1n	14	66149065	1	3.06E-15	1.71E-13	-	-0.271958	-0.2759	+	T	A	0.4735886	0.0341378	+	T	A	0.4951	0.0374
rs11622271	LC_IGP15	IgG1_G1N	14	66149065	1	3.63E-11	8.82E-11	-	-0.228262	-0.2416	+	T	A	0.474706027	0.0342511	+	T	A	0.4951	0.0372
rs11622271	LC_IGP56	IgG1_G1Nn	14	66149065	1	1.49E-11	1.78E-10	-	-0.233623	-0.2373	+	T	A	0.4735886	0.0343677	+	T	A	0.4951	0.0372
rs11622271	LC_IGP_R36	IgG1_G1NS1/IgG1_G1N	14	66149065	1	4.09E-08	3.18E-12	+	0.188464	0.2545	+	T	A	0.47464365	0.0341822	+	T	A	0.4951	0.0365
rs11622271	LC_IGP_R32	IgG1_G1S1/IgG1_G1	14	66149065	1	4.98E-10	3.97E-10	+	0.213139	0.2253	+	T	A	0.473010398	0.0340607	+	T	A	0.4951	0.036
rs11622271	LC_IGP_R3	IgG1_G2F/IgG1_G2	14	66149065	1	2.03E-15	5.96E-15	+	0.271651	0.2903	+	T	A	0.473026895	0.0338795	+	T	A	0.4951	0.0372
rs11622271	LC_IGP_R8	IgG1_G2FS1/IgG1_G2S1	14	66149065	1	4.31E-10	9.20E-13	+	0.214303	0.265	+	T	A	0.472737935	0.0341215	+	T	A	0.4951	0.0371
rs11622271	LC_IGP_R26	IgG1_G2N/IgG1_G1N	14	66149065	1	1.03E-11	1.05E-08	+	0.234008	0.2081	+	T	A	0.474303704	0.0341474	+	T	A	0.4951	0.0364
rs11622271	LC_IGP148	IgG2 FG0n total/G0n	14	66149065	1	3.92E-09	2.99E-13	+	0.202756	0.2702	+	T	A	0.471903941	0.0342499	+	T	A	0.4951	0.037
rs11622271	LC_IGP97	IgG2_G0	14	66149065	1	5.83E-11	1.63E-13	-	-0.224113	-0.2736	+	T	A	0.472845355	0.0339962	+	T	A	0.4951	0.0371
rs11622271	LC_IGP_R41	IgG2_G0F/IgG2_G0	14	66149065	1	2.24E-08	1.20E-12	+	0.191672	0.2618	+	T	A	0.472845355	0.0341056	+	T	A	0.4951	0.0368
rs11622271	LC_IGP138	IgG2_G0n	14	66149065	1	5.13E-11	1.14E-13	-	-0.225661	-0.2758	+	T	A	0.471903941	0.0341287	+	T	A	0.4951	0.0371
rs11622271	LC_IGP_R64	IgG2_G2/IgG2_G1	14	66149065	1	4.36E-09	3.49E-12	+	0.20242	0.253	+	T	A	0.472075887	0.0342963	+	T	A	0.4951	0.0364
rs12882200	LC_IGP78	IgG1 BG0n/G0n	14	66157861	1	2.66E-14	9.19E-10	-	-0.262305	-0.2286	+	A	C	0.473667906	0.0341352	+	A	C	0.4939	0.0373
rs12882200	LC_IGP79	IgG1 BG1n/G1n	14	66157861	1	4.03E-13	7.01E-12	-	-0.250702	-0.2558	+	A	C	0.473667906	0.0342714	+	A	C	0.4939	0.0373
rs12882200	LC_IGP32	IgG1 BG1S1/(BG1+BG1S1)	14	66157861	1	4.16E-08	3.32E-12	+	0.18824	0.2548	+	A	C	0.474722565	0.03416	+	A	C	0.4939	0.0366
rs12882200	LC_IGP77	IgG1 Bn	14	66157861	1	3.08E-13	2.05E-11	-	-0.251992	-0.2512	+	A	C	0.473667906	0.0342727	+	A	C	0.4939	0.0375
rs12882200	LC_IGP86	IgG1 Bn/Fn total	14	66157861	1	4.28E-14	1.22E-11	-	-0.260813	-0.2541	+	A	C	0.473667906	0.034224	+	A	C	0.4939	0.0375
rs12882200	LC_IGP84	IgG1 FBn/Bn total	14	66157861	1	2.29E-15	4.10E-16	+	0.273987	0.3075	+	A	C	0.473667906	0.0342321	+	A	C	0.4939	0.0378
rs12882200	LC_IGP62	IgG1 FG0n total/G0n	14	66157861	1	9.56E-18	2.56E-12	+	0.294489	0.2631	+	A	C	0.473667906	0.0339296	+	A	C	0.4939	0.0376
rs12882200	LC_IGP63	IgG1 FG1n total/G1n	14	66157861	1	9.75E-18	1.46E-14	+	0.29528	0.2889	+	A	C	0.473667906	0.03403	+	A	C	0.4939	0.0376
rs12882200	LC_IGP64	IgG1 FG2n total/G2n	14	66157861	1	6.16E-14	2.40E-15	+	0.259321	0.2958	+	A	C	0.473667906	0.0342482	+	A	C	0.4939	0.0374
rs12882200	LC_IGP68	IgG1 FG2n/G2n	14	66157861	1	1.85E-10	1.42E-08	+	0.220692	0.2108	+	A	C	0.473667906	0.0344042	+	A	C	0.4939	0.0372
rs12882200	LC_IGP61	IgG1 Fn total	14	66157861	1	5.32E-17	3.60E-14	+	0.288427	0.2848	+	A	C	0.473667906	0.0340415	+	A	C	0.4939	0.0376
rs12882200	LC_IGP81	IgG1 Fn/Bn	14	66157861	1	1.55E-13	1.05E-10	+	0.254962	0.241	+	A	C	0.473667906	0.0342396	+	A	C	0.4939	0.0373
rs12882200	LC_IGP21	IgG1 Fucosylation	14	66157861	1	2.12E-15	5.04E-13	+	0.272587	0.2711	+	A	C	0.472872996	0.0340181	+	A	C	0.4939	0.0375
rs12882200	LC_IGP28	IgG1 G1S1/(G1+G1S1)	14	66157861	1	4.09E-10	2.94E-10	+	0.214065	0.2273	+	A	C	0.473088685	0.0340372	+	A	C	0.4939	0.0361
rs12882200	LC_IGP27	IgG1 GS1/(G0+G+GS1)	14	66157861	1	4.47E-09	3.24E-08	+	0.201268	0.2029	+	A	C	0.473088685	0.0341272	+	A	C	0.4939	0.0367
rs12882200	LC_IGP11	IgG1_G0	14	66157861	1	3.92E-19	2.84E-14	-	-0.303957	-0.2845	+	A	C	0.473427001	0.0335829	+	A	C	0.4939	0.0374
rs12882200	LC_IGP_R1	IgG1_G0F/IgG1_G0	14	66157861	1	9.94E-17	5.42E-12	+	0.282751	0.2583	+	A	C	0.473427001	0.0336803	+	A	C	0.4939	0.0375
rs12882200	LC_IGP_R4	IgG1_G0FN/IgG1_G0N	14	66157861	1	6.16E-15	4.32E-13	+	0.267285	0.2734	+	A	C	0.47397428	0.0339403	+	A	C	0.4939	0.0377
rs12882200	LC_IGP52	IgG1_G0n	14	66157861	1	3.19E-19	2.39E-14	-	-0.30709	-0.2858	+	A	C	0.473667906	0.0338345	+	A	C	0.4939	0.0375
rs12882200	LC_IGP14	IgG1_G0N	14	66157861	1	6.08E-17	4.62E-11	-	-0.286107	-0.245	+	A	C	0.47397428	0.0338373	+	A	C	0.4939	0.0372
rs12882200	LC_IGP55	IgG1_G0Nn	14	66157861	1	2.40E-17	4.71E-11	-	-0.291515	-0.2452	+	A	C	0.473667906	0.0340189	+	A	C	0.4939	0.0373
rs12882200	LC_IGP12	IgG1_G1	14	66157861	1	3.19E-16	1.06E-13	-	-0.278713	-0.2792	+	A	C	0.473427001	0.0337788	+	A	C	0.4939	0.0376
rs12882200	LC_IGP_R2	IgG1_G1F/IgG1_G1	14	66157861	1	2.01E-17	1.19E-13	+	0.289765	0.278	+	A	C	0.473427001	0.0337363	+	A	C	0.4939	0.0375
rs12882200	LC_IGP_R5	IgG1_G1FN/IgG1_G1N	14	66157861	1	2.51E-14	1.93E-17	+	0.262311	0.3194	+	A	C	0.474784748	0.0341041	+	A	C	0.4939	0.0376
rs12882200	LC_IGP53	IgG1_G1n	14	66157861	1	2.07E-15	1.76E-13	-	-0.273491	-0.2765	+	A	C	0.473667906	0.0341151	+	A	C	0.4939	0.0375
rs12882200	LC_IGP15	IgG1_G1N	14	66157861	1	2.37E-11	9.15E-11	-	-0.230309	-0.2419	+	A	C	0.474784748	0.0342278	+	A	C	0.4939	0.0373
rs12882200	LC_IGP56	IgG1_G1Nn	14	66157861	1	9.07E-12	1.82E-10	-	-0.23597	-0.2377	+	A	C	0.473667906	0.0343427	+	A	C	0.4939	0.0373
rs12882200	LC_IGP_R36	IgG1_G1NS1/IgG1_G1N	14	66157861	1	3.80E-08	3.80E-12	+	0.188823	0.2543	+	A	C	0.474722565	0.0341662	+	A	C	0.4939	0.0366
rs12882200	LC_IGP_R32	IgG1_G1S1/IgG1_G1	14	66157861	1	7.38E-10	4.41E-10	+	0.210951	0.2253	+	A	C	0.473088685	0.0340544	+	A	C	0.4939	0.0361
rs12882200	LC_IGP_R3	IgG1_G2F/IgG1_G2	14	66157861	1	2.33E-15	7.71E-15	+	0.270951	0.2897	+	A	C	0.473105134	0.0338681	+	A	C	0.4939	0.0373
rs12882200	LC_IGP_R8	IgG1_G2FS1/IgG1_G2S1	14	66157861	1	3.89E-10	1.19E-12	+	0.214763	0.2642	+	A	C	0.472816127	0.034105	+	A	C	0.4939	0.0372
rs12882200	LC_IGP_R26	IgG1_G2N/IgG1_G1N	14	66157861	1	1.36E-11	1.21E-08	+	0.232519	0.2078	+	A	C	0.474382716	0.0341387	+	A	C	0.4939	0.0365
rs12882200	LC_IGP148	IgG2 FG0n total/G0n	14	66157861	1	3.13E-09	2.91E-13	+	0.203945	0.2714	+	A	C	0.471982759	0.0342306	+	A	C	0.4939	0.0372
rs12882200	LC_IGP97	IgG2_G0	14	66157861	1	1.15E-10	1.78E-13	-	-0.220624	-0.2742	+	A	C	0.473105134	0.0340014	+	A	C	0.4939	0.0372
rs12882200	LC_IGP_R41	IgG2_G0F/IgG2_G0	14	66157861	1	2.52E-08	1.16E-12	+	0.19093	0.263	+	A	C	0.473105134	0.0340995	+	A	C	0.4939	0.037
rs12882200	LC_IGP138	IgG2_G0n	14	66157861	1	6.12E-11	1.23E-13	-	-0.224671	-0.2765	+	A	C	0.471982759	0.0341178	+	A	C	0.4939	0.0373
rs12882200	LC_IGP_R64	IgG2_G2/IgG2_G1	14	66157861	1	8.51E-09	6.24E-12	+	0.198512	0.2506	+	A	C	0.472154223	0.0342955	+	A	C	0.4939	0.0365
rs186501115	LC_IGP11	IgG1_G0	14	66159405	1	4.02E-09	7.85E-07	+	0.252	0.2403	+	A	G	0.203294995	0.0426005	+	A	G	0.2191	0.0487
rs186501115	LC_IGP52	IgG1_G0n	14	66159405	1	3.61E-09	9.97E-07	+	0.25442	0.2384	+	A	G	0.203403908	0.0428756	+	A	G	0.2191	0.0487
rs7152023	LC_IGP84	IgG1 FBn/Bn total	14	66160827	1	1.01E-08	6.80E-08	+	0.202709	0.2071	+	A	C	0.550936493	0.0351937	+	A	C	0.5671	0.0384
rs7152023	LC_IGP63	IgG1 FG1n total/G1n	14	66160827	1	1.95E-10	9.99E-08	+	0.224336	0.2033	+	A	C	0.550936493	0.0350193	+	A	C	0.5671	0.0382
rs7152023	LC_IGP61	IgG1 Fn total	14	66160827	1	9.67E-10	2.50E-07	+	0.215475	0.197	+	A	C	0.550936493	0.0350286	+	A	C	0.5671	0.0382
rs7152023	LC_IGP11	IgG1_G0	14	66160827	1	4.29E-11	3.13E-07	-	-0.229517	-0.1943	+	A	C	0.550220831	0.0345712	+	A	C	0.5671	0.038
rs7152023	LC_IGP52	IgG1_G0n	14	66160827	1	5.37E-11	3.58E-07	-	-0.23029	-0.1937	+	A	C	0.550936493	0.0348638	+	A	C	0.5671	0.038
rs7152023	LC_IGP12	IgG1_G1	14	66160827	1	2.11E-09	1.99E-07	-	-0.208973	-0.1984	+	A	C	0.550526268	0.0346962	+	A	C	0.5671	0.0382
rs7152023	LC_IGP_R2	IgG1_G1F/IgG1_G1	14	66160827	1	3.84E-10	2.63E-07	+	0.218419	0.196	+	A	C	0.550526268	0.0346751	+	A	C	0.5671	0.0381
rs7152023	LC_IGP_R5	IgG1_G1FN/IgG1_G1N	14	66160827	1	2.02E-08	9.89E-09	+	0.19745	0.2188	+	A	C	0.55179059	0.0350184	+	A	C	0.5671	0.0382
rs7152023	LC_IGP53	IgG1_G1n	14	66160827	1	3.00E-09	2.91E-07	-	-0.209066	-0.1956	+	A	C	0.550936493	0.0350499	+	A	C	0.5671	0.0381
rs7152023	LC_IGP_R3	IgG1_G2F/IgG1_G2	14	66160827	1	7.58E-09	7.39E-08	+	0.202031	0.2039	+	A	C	0.549945905	0.0347843	+	A	C	0.5671	0.0379
rs12894902	LC_IGP78	IgG1 BG0n/G0n	14	66161799	1	3.10E-14	2.25E-10	-	-0.262278	-0.2364	+	T	C	0.47238228	0.0342239	+	T	C	0.4923	0.0373
rs12894902	LC_IGP79	IgG1 BG1n/G1n	14	66161799	1	5.76E-13	1.94E-12	-	-0.249679	-0.2621	+	T	C	0.47238228	0.0343646	+	T	C	0.4923	0.0372
rs12894902	LC_IGP32	IgG1 BG1S1/(BG1+BG1S1)	14	66161799	1	2.62E-08	1.15E-12	+	0.191469	0.2597	+	T	C	0.47344328	0.0342351	+	T	C	0.4923	0.0365
rs12894902	LC_IGP77	IgG1 Bn	14	66161799	1	4.15E-13	4.85E-12	-	-0.251248	-0.2584	+	T	C	0.47238228	0.0343646	+	T	C	0.4923	0.0374
rs12894902	LC_IGP86	IgG1 Bn/Fn total	14	66161799	1	5.84E-14	2.89E-12	-	-0.260084	-0.2612	+	T	C	0.47238228	0.0343161	+	T	C	0.4923	0.0374
rs12894902	LC_IGP84	IgG1 FBn/Bn total	14	66161799	1	2.77E-15	7.92E-17	+	0.273866	0.3145	+	T	C	0.47238228	0.0343217	+	T	C	0.4923	0.0378
rs12894902	LC_IGP62	IgG1 FG0n total/G0n	14	66161799	1	1.22E-17	7.83E-13	+	0.294273	0.2689	+	T	C	0.47238228	0.0340195	+	T	C	0.4923	0.0375
rs12894902	LC_IGP63	IgG1 FG1n total/G1n	14	66161799	1	1.42E-17	3.92E-15	+	0.294556	0.2948	+	T	C	0.47238228	0.034123	+	T	C	0.4923	0.0375
rs12894902	LC_IGP64	IgG1 FG2n total/G2n	14	66161799	1	7.14E-14	6.03E-16	+	0.259314	0.3019	+	T	C	0.47238228	0.0343369	+	T	C	0.4923	0.0373
rs12894902	LC_IGP68	IgG1 FG2n/G2n	14	66161799	1	2.30E-10	5.71E-09	+	0.2201	0.2164	+	T	C	0.47238228	0.0344948	+	T	C	0.4923	0.0372
rs12894902	LC_IGP61	IgG1 Fn total	14	66161799	1	7.34E-17	9.76E-15	+	0.287864	0.2908	+	T	C	0.47238228	0.0341334	+	T	C	0.4923	0.0376
rs12894902	LC_IGP81	IgG1 Fn/Bn	14	66161799	1	2.08E-13	2.92E-11	+	0.254286	0.2477	+	T	C	0.47238228	0.0343312	+	T	C	0.4923	0.0372
rs12894902	LC_IGP21	IgG1 Fucosylation	14	66161799	1	3.70E-15	1.25E-13	+	0.27092	0.2778	+	T	C	0.471593711	0.0341145	+	T	C	0.4923	0.0375
rs12894902	LC_IGP28	IgG1 G1S1/(G1+G1S1)	14	66161799	1	5.43E-10	1.95E-10	+	0.213084	0.2292	+	T	C	0.471819572	0.0341271	+	T	C	0.4923	0.036
rs12894902	LC_IGP27	IgG1 GS1/(G0+G+GS1)	14	66161799	1	4.11E-09	2.31E-08	+	0.202237	0.2046	+	T	C	0.471819572	0.0342098	+	T	C	0.4923	0.0366
rs12894902	LC_IGP11	IgG1_G0	14	66161799	1	3.89E-19	9.06E-15	-	-0.304738	-0.2892	+	T	C	0.472159438	0.0336654	+	T	C	0.4923	0.0373
rs12894902	LC_IGP_R1	IgG1_G0F/IgG1_G0	14	66161799	1	1.29E-16	1.92E-12	+	0.282419	0.2634	+	T	C	0.472159438	0.0337685	+	T	C	0.4923	0.0374
rs12894902	LC_IGP_R4	IgG1_G0FN/IgG1_G0N	14	66161799	1	7.78E-15	9.03E-14	+	0.266949	0.2809	+	T	C	0.472703613	0.0340288	+	T	C	0.4923	0.0377
rs12894902	LC_IGP52	IgG1_G0n	14	66161799	1	3.30E-19	7.70E-15	-	-0.307728	-0.2906	+	T	C	0.47238228	0.0339199	+	T	C	0.4923	0.0374
rs12894902	LC_IGP14	IgG1_G0N	14	66161799	1	6.17E-17	1.05E-11	-	-0.286758	-0.2524	+	T	C	0.472703613	0.033921	+	T	C	0.4923	0.0371
rs12894902	LC_IGP55	IgG1_G0Nn	14	66161799	1	2.58E-17	1.07E-11	-	-0.291961	-0.2526	+	T	C	0.47238228	0.0341055	+	T	C	0.4923	0.0372
rs12894902	LC_IGP12	IgG1_G1	14	66161799	1	3.98E-16	2.90E-14	-	-0.278506	-0.2853	+	T	C	0.472159438	0.0338665	+	T	C	0.4923	0.0375
rs12894902	LC_IGP_R2	IgG1_G1F/IgG1_G1	14	66161799	1	2.96E-17	3.52E-14	+	0.28897	0.2837	+	T	C	0.472159438	0.0338273	+	T	C	0.4923	0.0374
rs12894902	LC_IGP_R5	IgG1_G1FN/IgG1_G1N	14	66161799	1	3.17E-14	4.05E-18	+	0.26193	0.3258	+	T	C	0.47350861	0.0341931	+	T	C	0.4923	0.0376
rs12894902	LC_IGP53	IgG1_G1n	14	66161799	1	2.78E-15	5.11E-14	-	-0.272928	-0.2824	+	T	C	0.47238228	0.0342066	+	T	C	0.4923	0.0375
rs12894902	LC_IGP15	IgG1_G1N	14	66161799	1	3.27E-11	2.55E-11	-	-0.229252	-0.2486	+	T	C	0.47350861	0.034319	+	T	C	0.4923	0.0373
rs12894902	LC_IGP56	IgG1_G1Nn	14	66161799	1	1.28E-11	5.38E-11	-	-0.234868	-0.2441	+	T	C	0.47238228	0.0344358	+	T	C	0.4923	0.0372
rs12894902	LC_IGP_R36	IgG1_G1NS1/IgG1_G1N	14	66161799	1	2.48E-08	1.34E-12	+	0.191837	0.2591	+	T	C	0.47344328	0.034242	+	T	C	0.4923	0.0366
rs12894902	LC_IGP_R32	IgG1_G1S1/IgG1_G1	14	66161799	1	9.68E-10	2.97E-10	+	0.210012	0.2272	+	T	C	0.471819572	0.034144	+	T	C	0.4923	0.0361
rs12894902	LC_IGP_R3	IgG1_G2F/IgG1_G2	14	66161799	1	2.61E-15	2.11E-15	+	0.271143	0.2956	+	T	C	0.471836797	0.0339541	+	T	C	0.4923	0.0373
rs12894902	LC_IGP_R8	IgG1_G2FS1/IgG1_G2S1	14	66161799	1	4.29E-10	2.32E-13	+	0.214767	0.2724	+	T	C	0.471548564	0.0341913	+	T	C	0.4923	0.0372
rs12894902	LC_IGP_R26	IgG1_G2N/IgG1_G1N	14	66161799	1	1.12E-11	7.57E-09	+	0.234066	0.2102	+	T	C	0.473101852	0.0342193	+	T	C	0.4923	0.0364
rs12894902	LC_IGP148	IgG2 FG0n total/G0n	14	66161799	1	5.26E-09	8.53E-14	+	0.201515	0.2773	+	T	C	0.470705049	0.034327	+	T	C	0.4923	0.0372
rs12894902	LC_IGP97	IgG2_G0	14	66161799	1	1.31E-10	6.46E-14	-	-0.22049	-0.2788	+	T	C	0.471836797	0.0340881	+	T	C	0.4923	0.0372
rs12894902	LC_IGP_R41	IgG2_G0F/IgG2_G0	14	66161799	1	3.84E-08	3.63E-13	+	0.188888	0.2686	+	T	C	0.471836797	0.0341922	+	T	C	0.4923	0.0369

rs12894902	LC_IGP138	IgG2_G0n	14	66161799	1	7.55E-11	4.27E-14	-	-0.224161	-0.2812	+	T	C	0.470705049	0.0342075 +	T	C	0.4923	0.0372
rs12894902	LC_IGP_R64	IgG2_G2/IgG2_G1	14	66161799	1	9.05E-09	1.90E-12	+	0.198652	0.2566	+	T	C	0.470884333	0.0343819 +	T	C	0.4923	0.0364
rs12588838	LC_IGP78	IgG1 BG0n/G0n	14	66162638	1	2.92E-14	9.80E-10	-	-0.261969	-0.227	+	T	G	0.47438166	0.0341474 +	T	G	0.4966	0.0371
rs12588838	LC_IGP79	IgG1 BG1n/G1n	14	66162638	1	4.06E-13	6.36E-12	-	-0.250745	-0.2549	+	T	G	0.47438166	0.0342818 +	T	G	0.4966	0.0371
rs12588838	LC_IGP32	IgG1 BG1S1/(BG1+BG1S1)	14	66162638	1	4.36E-08	2.80E-12	+	0.188001	0.2542	+	T	G	0.475432799	0.0341714 +	T	G	0.4965	0.0364
rs12588838	LC_IGP77	IgG1 Bn	14	66162638	1	3.31E-13	1.94E-11	-	-0.251737	-0.2501	+	T	G	0.47438166	0.0342844 +	T	G	0.4966	0.0373
rs12588838	LC_IGP86	IgG1 Bn/Fn total	14	66162638	1	4.56E-14	1.14E-11	-	-0.260607	-0.2531	+	T	G	0.47438166	0.0342355 +	T	G	0.4966	0.0373
rs12588838	LC_IGP84	IgG1 FBn/Bn total	14	66162638	1	2.94E-15	3.59E-16	+	0.273002	0.3063	+	T	G	0.47438166	0.0342476 +	T	G	0.4966	0.0376
rs12588838	LC_IGP62	IgG1 FG0n total/G0n	14	66162638	1	8.47E-18	2.75E-12	+	0.295041	0.2613	+	T	G	0.47438166	0.0339372 +	T	G	0.4966	0.0374
rs12588838	LC_IGP63	IgG1 FG1n total/G1n	14	66162638	1	9.35E-18	1.31E-14	+	0.295531	0.2878	+	T	G	0.47438166	0.0340393 +	T	G	0.4966	0.0374
rs12588838	LC_IGP64	IgG1 FG2n total/G2n	14	66162638	1	5.45E-14	1.90E-15	+	0.259942	0.2953	+	T	G	0.47438166	0.0342558 +	T	G	0.4966	0.0372
rs12588838	LC_IGP68	IgG1 FG2n/G2n	14	66162638	1	1.66E-10	1.29E-08	+	0.221327	0.2103	+	T	G	0.47438166	0.0344122 +	T	G	0.4966	0.037
rs12588838	LC_IGP61	IgG1 Fn total	14	66162638	1	5.35E-17	3.37E-14	+	0.288489	0.2835	+	T	G	0.47438166	0.0340518 +	T	G	0.4966	0.0374
rs12588838	LC_IGP81	IgG1 Fn/Bn	14	66162638	1	1.58E-13	9.93E-11	+	0.254974	0.24	+	T	G	0.47438166	0.0342501 +	T	G	0.4966	0.0371
rs12588838	LC_IGP21	IgG1 Fucosylation	14	66162638	1	1.91E-15	4.79E-13	+	0.273114	0.2698	+	T	G	0.473583231	0.0340258 +	T	G	0.4966	0.0373
rs12588838	LC_IGP28	IgG1 G1S1/(G1+G1S1)	14	66162638	1	3.16E-10	2.80E-10	+	0.215482	0.2263	+	T	G	0.473793272	0.0340419 +	T	G	0.4966	0.0359
rs12588838	LC_IGP27	IgG1 GS1/(G0+G+GS1)	14	66162638	1	2.66E-09	2.92E-08	+	0.204234	0.2024	+	T	G	0.473793272	0.0341266 +	T	G	0.4966	0.0365
rs12588838	LC_IGP11	IgG1_G0	14	66162638	1	1.91E-19	2.42E-14	-	-0.306669	-0.2836	+	T	G	0.474130727	0.0335782 +	T	G	0.4966	0.0372
rs12588838	LC_IGP_R1	IgG1_G0F/IgG1_G0	14	66162638	1	8.07E-17	5.84E-12	+	0.283656	0.2565	+	T	G	0.474130727	0.0336859 +	T	G	0.4966	0.0373
rs12588838	LC_IGP_R4	IgG1_G0FN/IgG1_G0N	14	66162638	1	7.86E-15	4.25E-13	+	0.266331	0.2719	+	T	G	0.474679731	0.0339553 +	T	G	0.4966	0.0375
rs12588838	LC_IGP52	IgG1_G0n	14	66162638	1	1.61E-19	2.10E-14	-	-0.309677	-0.2848	+	T	G	0.47438166	0.0338305 +	T	G	0.4966	0.0372
rs12588838	LC_IGP14	IgG1_G0N	14	66162638	1	4.45E-17	4.14E-11	-	-0.287428	-0.2443	+	T	G	0.474679731	0.0338409 +	T	G	0.4966	0.037
rs12588838	LC_IGP55	IgG1_G0Nn	14	66162638	1	1.84E-17	4.31E-11	-	-0.292644	-0.2444	+	T	G	0.47438166	0.0340235 +	T	G	0.4966	0.0371
rs12588838	LC_IGP12	IgG1_G1	14	66162638	1	3.28E-16	9.74E-14	-	-0.278678	-0.2781	+	T	G	0.474130727	0.0337893 +	T	G	0.4966	0.0374
rs12588838	LC_IGP_R2	IgG1_G1F/IgG1_G1	14	66162638	1	1.80E-17	1.09E-13	+	0.290267	0.2768	+	T	G	0.474130727	0.033744 +	T	G	0.4966	0.0373
rs12588838	LC_IGP_R5	IgG1_G1FN/IgG1_G1N	14	66162638	1	2.83E-14	1.54E-17	+	0.26186	0.3186	+	T	G	0.475493235	0.0341169 +	T	G	0.4966	0.0374
rs12588838	LC_IGP53	IgG1_G1n	14	66162638	1	2.27E-15	1.67E-13	-	-0.273181	-0.2752	+	T	G	0.47438166	0.0341272 +	T	G	0.4966	0.0373
rs12588838	LC_IGP15	IgG1_G1N	14	66162638	1	2.54E-11	8.58E-11	-	-0.230026	-0.241	+	T	G	0.475493235	0.0342396 +	T	G	0.4966	0.0371
rs12588838	LC_IGP56	IgG1_G1Nn	14	66162638	1	1.01E-11	1.76E-10	-	-0.235511	-0.2366	+	T	G	0.47438166	0.0343552 +	T	G	0.4966	0.0371
rs12588838	LC_IGP_R36	IgG1_G1NS1/IgG1_G1N	14	66162638	1	4.00E-08	3.21E-12	+	0.188573	0.2537	+	T	G	0.475432799	0.0341776 +	T	G	0.4965	0.0364
rs12588838	LC_IGP_R32	IgG1_G1S1/IgG1_G1	14	66162638	1	5.70E-10	4.21E-10	+	0.212399	0.2243	+	T	G	0.473793272	0.034059 +	T	G	0.4966	0.0359
rs12588838	LC_IGP_R3	IgG1_G2F/IgG1_G2	14	66162638	1	2.06E-15	6.15E-15	+	0.271551	0.2893	+	T	G	0.473809291	0.0338754 +	T	G	0.4966	0.0371
rs12588838	LC_IGP_R8	IgG1_G2FS1/IgG1_G2S1	14	66162638	1	3.68E-10	9.46E-13	+	0.215118	0.264	+	T	G	0.473519853	0.0341138 +	T	G	0.4966	0.037
rs12588838	LC_IGP_R26	IgG1_G2N/IgG1_G1N	14	66162638	1	9.19E-12	1.04E-08	+	0.234514	0.2075	+	T	G	0.475093827	0.0341409 +	T	G	0.4966	0.0362
rs12588838	LC_IGP148	IgG2 FG0n total/G0n	14	66162638	1	3.06E-09	2.23E-13	+	0.204125	0.2712	+	T	G	0.472692118	0.03424 +	T	G	0.4966	0.037
rs12588838	LC_IGP97	IgG2_G0	14	66162638	1	8.15E-11	1.32E-13	-	-0.222422	-0.2742	+	T	G	0.473809291	0.0340044 +	T	G	0.4966	0.037
rs12588838	LC_IGP_R41	IgG2_G0F/IgG2_G0	14	66162638	1	2.60E-08	9.10E-13	+	0.190799	0.2628	+	T	G	0.473809291	0.0341101 +	T	G	0.4966	0.0368
rs12588838	LC_IGP138	IgG2_G0n	14	66162638	1	4.66E-11	9.08E-14	-	-0.226117	-0.2764	+	T	G	0.472692118	0.034122 +	T	G	0.4966	0.0371
rs12588838	LC_IGP_R64	IgG2_G2/IgG2_G1	14	66162638	1	5.28E-09	3.18E-12	+	0.201301	0.2529	+	T	G	0.472859241	0.0342955 +	T	G	0.4966	0.0363
rs11628765	LC_IGP78	IgG1 BG0n/G0n	14	66168449	1	2.98E-14	1.02E-09	-	-0.262052	-0.227	+	C	T	0.474087361	0.0341699 +	C	T	0.4972	0.0372
rs11628765	LC_IGP79	IgG1 BG1n/G1n	14	66168449	1	3.74E-13	6.76E-12	-	-0.251292	-0.2548	+	C	T	0.474087361	0.0343022 +	C	T	0.4972	0.0371
rs11628765	LC_IGP77	IgG1 Bn	14	66168449	1	3.05E-13	2.04E-11	-	-0.25227	-0.2501	+	C	T	0.474087361	0.0343049 +	C	T	0.4972	0.0373
rs11628765	LC_IGP86	IgG1 Bn/Fn total	14	66168449	1	4.24E-14	1.20E-11	-	-0.261097	-0.2531	+	C	T	0.474087361	0.0342562 +	C	T	0.4972	0.0373
rs11628765	LC_IGP84	IgG1 FBn/Bn total	14	66168449	1	2.85E-15	4.11E-16	+	0.273319	0.306	+	C	T	0.474087361	0.0342691 +	C	T	0.4972	0.0376
rs11628765	LC_IGP62	IgG1 FG0n total/G0n	14	66168449	1	9.56E-18	2.71E-12	+	0.294764	0.2617	+	C	T	0.474087361	0.0339617 +	C	T	0.4972	0.0374
rs11628765	LC_IGP63	IgG1 FG1n total/G1n	14	66168449	1	1.08E-17	1.37E-14	+	0.295166	0.2878	+	C	T	0.474087361	0.0340643 +	C	T	0.4972	0.0374
rs11628765	LC_IGP64	IgG1 FG2n total/G2n	14	66168449	1	5.44E-14	2.03E-15	+	0.260119	0.2952	+	C	T	0.474087361	0.0342779 +	C	T	0.4972	0.0372
rs11628765	LC_IGP68	IgG1 FG2n/G2n	14	66168449	1	1.54E-10	1.21E-08	+	0.221849	0.2109	+	C	T	0.474087361	0.034433 +	C	T	0.4972	0.037
rs11628765	LC_IGP61	IgG1 Fn total	14	66168449	1	5.70E-17	3.41E-14	+	0.288429	0.2838	+	C	T	0.474087361	0.0340751 +	C	T	0.4972	0.0374
rs11628765	LC_IGP81	IgG1 Fn/Bn	14	66168449	1	1.46E-13	1.00E-10	+	0.255484	0.2403	+	C	T	0.474087361	0.0342706 +	C	T	0.4972	0.0372
rs11628765	LC_IGP21	IgG1 Fucosylation	14	66168449	1	2.40E-15	4.47E-13	+	0.272229	0.2705	+	C	T	0.473502158	0.0340414 +	C	T	0.4972	0.0374
rs11628765	LC_IGP28	IgG1 G1S1/(G1+G1S1)	14	66168449	1	4.24E-10	2.99E-10	+	0.214004	0.2262	+	C	T	0.473713456	0.0340585 +	C	T	0.4972	0.0359
rs11628765	LC_IGP27	IgG1 GS1/(G0+G+GS1)	14	66168449	1	2.68E-09	3.01E-08	+	0.204267	0.2025	+	C	T	0.473713456	0.0341374 +	C	T	0.4972	0.0366
rs11628765	LC_IGP11	IgG1_G0	14	66168449	1	1.98E-19	2.50E-14	-	-0.306638	-0.2838	+	C	T	0.474051008	0.0335894 +	C	T	0.4972	0.0372
rs11628765	LC_IGP_R1	IgG1_G0F/IgG1_G0	14	66168449	1	8.08E-17	5.56E-12	+	0.283742	0.2571	+	C	T	0.474051008	0.0336965 +	C	T	0.4972	0.0373
rs11628765	LC_IGP_R4	IgG1_G0FN/IgG1_G0N	14	66168449	1	1.54E-14	4.60E-13	+	0.263525	0.2719	+	C	T	0.474599816	0.0339798 +	C	T	0.4972	0.0376
rs11628765	LC_IGP52	IgG1_G0n	14	66168449	1	2.14E-19	2.19E-14	-	-0.308851	-0.2849	+	C	T	0.474087361	0.0338582 +	C	T	0.4972	0.0373
rs11628765	LC_IGP14	IgG1_G0N	14	66168449	1	9.44E-17	4.61E-11	-	-0.284539	-0.2441	+	C	T	0.474599816	0.0338669 +	C	T	0.4972	0.0371
rs11628765	LC_IGP55	IgG1_G0Nn	14	66168449	1	2.09E-17	4.81E-11	-	-0.292325	-0.2441	+	C	T	0.474087361	0.0340482 +	C	T	0.4972	0.0371
rs11628765	LC_IGP12	IgG1_G1	14	66168449	1	3.89E-16	9.78E-14	-	-0.278085	-0.2783	+	C	T	0.474051008	0.0338033 +	C	T	0.4972	0.0374
rs11628765	LC_IGP_R2	IgG1_G1F/IgG1_G1	14	66168449	1	2.10E-17	1.10E-13	+	0.289769	0.2771	+	C	T	0.474051008	0.0337576 +	C	T	0.4972	0.0373
rs11628765	LC_IGP_R5	IgG1_G1FN/IgG1_G1N	14	66168449	1	2.76E-14	1.83E-17	+	0.262141	0.3182	+	C	T	0.475200492	0.0341382 +	C	T	0.4972	0.0374
rs11628765	LC_IGP53	IgG1_G1n	14	66168449	1	2.69E-15	1.70E-13	-	-0.272648	-0.2753	+	C	T	0.474087361	0.0341528 +	C	T	0.4972	0.0374
rs11628765	LC_IGP15	IgG1_G1N	14	66168449	1	2.36E-11	8.89E-11	-	-0.230542	-0.241	+	C	T	0.475200492	0.03426 +	C	T	0.4972	0.0372
rs11628765	LC_IGP56	IgG1_G1Nn	14	66168449	1	9.20E-12	1.83E-10	-	-0.236127	-0.2366	+	C	T	0.474087361	0.0343754 +	C	T	0.4972	0.0371
rs11628765	LC_IGP_R36	IgG1_G1NS1/IgG1_G1N	14	66168449	1	4.69E-08	4.23E-12	+	0.187734	0.2526	+	C	T	0.475139334	0.0342028 +	C	T	0.4972	0.0365
rs11628765	LC_IGP_R32	IgG1_G1S1/IgG1_G1	14	66168449	1	7.64E-10	4.57E-10	+	0.210892	0.2242	+	C	T	0.473713456	0.0340757 +	C	T	0.4972	0.036
rs11628765	LC_IGP_R3	IgG1_G2F/IgG1_G2	14	66168449	1	2.56E-15	6.42E-15	+	0.270727	0.2893	+	C	T	0.473729523	0.0338905 +	C	T	0.4972	0.0371
rs11628765	LC_IGP_R8	IgG1_G2FS1/IgG1_G2S1	14	66168449	1	5.45E-10	8.26E-13	+	0.213093	0.2649	+	C	T	0.473440134	0.0341325 +	C	T	0.4972	0.037
rs11628765	LC_IGP_R26	IgG1_G2N/IgG1_G1N	14	66168449	1	1.20E-11	1.19E-08	+	0.233372	0.207	+	C	T	0.474800617	0.0341682 +	C	T	0.4972	0.0363
rs11628765	LC_IGP148	IgG2 FG0n total/G0n	14	66168449	1	4.25E-09	2.63E-13	+	0.202337	0.2707	+	C	T	0.472611761	0.0342576 +	C	T	0.4973	0.037
rs11628765	LC_IGP97	IgG2_G0	14	66168449	1	9.38E-11	1.55E-13	-	-0.22178	-0.2737	+	C	T	0.473729523	0.0340179 +	C	T	0.4972	0.0371
rs11628765	LC_IGP_R41	IgG2_G0F/IgG2_G0	14	66168449	1	2.87E-08	1.05E-12	+	0.190273	0.2623	+	C	T	0.473729523	0.0341228 +	C	T	0.4972	0.0368
rs11628765	LC_IGP138	IgG2_G0n	14	66168449	1	5.39E-11	1.09E-13	-	-0.225446	-0.2758	+	C	T	0.472611761	0.0341359 +	C	T	0.4973	0.0371
rs11628765	LC_IGP_R64	IgG2_G2/IgG2_G1	14	66168449	1	6.58E-09	3.60E-12	+	0.200111	0.2526	+	C	T	0.472779376	0.0343108 +	C	T	0.4972	0.0363
rs2411351	LC_IGP78	IgG1 BG0n/G0n	14	66171541	1	3.54E-14	9.20E-10	-	-0.261103	-0.2275	+	C	T	0.474048017	0.0341493 +	C	T	0.4984	0.0372
rs2411351	LC_IGP79	IgG1 BG1n/G1n	14	66171541	1	4.50E-13	6.12E-12	-	-0.250254	-0.2552	+	C	T	0.474048017	0.0342817 +	C	T	0.4984	0.0371
rs2411351	LC_IGP77	IgG1 Bn	14	66171541	1	3.58E-13	1.82E-11	-	-0.251359	-0.2506	+	C	T	0.474048017	0.0342838 +	C	T	0.4984	0.0373
rs2411351	LC_IGP86	IgG1 Bn/Fn total	14	66171541	1	4.98E-14	1.06E-11	-	-0.260206	-0.2536	+	C	T	0.474048017	0.0342352 +	C	T	0.4984	0.0373
rs2411351	LC_IGP84	IgG1 FBn/Bn total	14	66171541	1	4.17E-15	3.81E-16	+	0.271505	0.3061	+	C	T	0.474048017	0.0342528 +	C	T	0.4984	0.0376
rs2411351	LC_IGP62	IgG1 FG0n total/G0n	14	66171541	1	1.06E-17	2.36E-12	+	0.294148	0.2622	+	C	T	0.474048017	0.0339397 +	C	T	0.4984	0.0374
rs2411351	LC_IGP63	IgG1 FG1n total/G1n	14	66171541	1	1.43E-17	1.21E-14	+	0.293868	0.2882	+	C	T	0.474048017	0.034046 +	C	T	0.4984	0.0374
rs2411351	LC_IGP64	IgG1 FG2n total/G2n	14	66171541	1	5.93E-14	1.89E-15	+	0.259549	0.2954	+	C	T	0.474048017	0.0342554 +	C	T	0.4984	0.0372
rs2411351	LC_IGP68	IgG1 FG2n/G2n	14	66171541	1	1.37E-10	1.05E-08	+	0.222294	0.2117	+	C	T	0.474048017	0.0344061 +	C	T	0.4984	0.037
rs2411351	LC_IGP61	IgG1 Fn total	14	66171541	1	6.37E-17	2.97E-14	+	0.287778	0.2842	+	C	T	0.474048017	0.0340533 +	C	T	0.4984	0.0374
rs2411351	LC_IGP81	IgG1 Fn/Bn	14	66171541	1	1.65E-13	8.85E-11	+	0.254759	0.2408	+	C	T	0.474048017	0.0342488 +	C	T	0.4984	0.0371
rs2411351	LC_IGP21	IgG1 Fucosylation	14	66171541	1	2.55E-15	3.48E-13	+	0.271872	0.2716	+	C	T	0.473248767	0.03403 +	C	T	0.4984	0.0373
rs2411351	LC_IGP28	IgG1 G1S1/(G1+G1S1)	14	66171541	1	1.09E-09	2.87E-10	+	0.208865	0.2264	+	C	T	0.473462691	0.0340652 +	C	T	0.4984	0.0359
rs2411351	LC_IGP27	IgG1 GS1/(G0+G+GS1)	14	66171541	1	1.05E-08	2.93E-08	+	0.196478	0.2026	+	C	T	0.473462691	0.0341525 +	C	T	0.4984	0.0365
rs2411351	LC_IGP11	IgG1_G0	14	66171541	1	3.66E-19	2.18E-14	-	-0.304279	-0.2842	+	C	T	0.47380055	0.0335894 +	C	T	0.4984	0.0372
rs2411351	LC_IGP_R1	IgG1_G0F/IgG1_G0	14	66171541	1	9.13E-17	4.77E-12	+	0.283153	0.2577	+	C	T	0.47380055	0.0336865 +	C	T	0.4984	0.0373
rs2411351	LC_IGP_R4	IgG1_G0FN/IgG1_G0N	14	66171541	1	1.23E-14	4.21E-13	+	0.264392	0.2721	+	C	T	0.474348745	0.0339625 +	C	T	0.4984	0.0375
rs2411351	LC_IGP52	IgG1_G0n	14	66171541	1	2.91E-19	1.92E-14	-	-0.307497	-0.2853	+	C	T	0.474048017	0.0338406 +	C	T	0.4984	0.0373
rs2411351	LC_IGP14	IgG1_G0N	14	66171541	1	7.88E-17	4.26E-11	-	-0.285153	-0.2443	+	C	T	0.474348745	0.0338506 +	C	T	0.4984	0.037
rs2411351	LC_IGP55	IgG1_G0Nn	14	66171541	1	3.16E-17	4.43E-11	-	-0.2905	-0.2444	+	C	T	0.474048017	0.0340326 +	C	T	0.4984	0.0371
rs2411351	LC_IGP12	IgG1_G1	14	66171541	1	4.97E-16	8.72E-14	-	-0.276981	-0.2786	+	C	T	0.47380055	0.0337958 +	C	T	0.4984	0.0374
rs2411351	LC_IGP_R2	IgG1_G1F/IgG1_G1	14	66171541	1	2.75E-17	9.62E-14	+	0.288611	0.2776	+	C	T	0.47380055	0.0337507 +	C	T	0.4984	0.0373
rs2411351	LC_IGP_R5	IgG1_G1FN/IgG1_G1N	14	66171541	1	4.01E-14	1.70E-17	+	0.260309	0.3182	+	C	T	0.475160824	0.0341221 +	C	T	0.4984	0.0374
rs2411351	LC_IGP53	IgG1_G1n	14	66171541	1	3.14E-15	1.54E-13	-	-0.271789	-0.2756	+	C	T	0.474048017	0.0341318 +	C	T	0.4984	0.0373
rs2411351	LC_IGP15	IgG1_G1N	14	66171541	1	2.71E-11	8.13E-11	-	-0.229694	-0.2414	+	C	T	0.475160824	0.0342388 +	C	T	0.4984	0.0371
rs2411351	LC_IGP56	IgG1_G1Nn	14	66171541	1	1.02E-11	1.68E-10	-	-0.235451	-0.2369	+	C	T	0.474048017	0.0343532 +	C	T	0.4984	0.0371
rs2411351	LC_IGP_R32	IgG1_G1S1/IgG1_G1	14	66171541	1	1.86E-09	4.43E-10	+	0.205969	0.2242	+	C	T	0.473462691	0.0340812 +	C	T	0.4984	0.0359
rs2411351	LC_IGP_R3	IgG1_G2F/IgG1_G2	14	66171541	1	2.17E-15	5.91E-15	+	0.271319	0.2895	+	C	T	0.473478912	0.0338746 +	C	T	0.4984	0.0371
rs2411351	LC_IGP_R8	IgG1_G2FS1/IgG1_G2S1	14	66171541	1	2.91E-10	6.40E-13	+	0.216348	0.2661	+	C	T	0.473189676	0.0341071 +	C	T	0.4984	0.037
rs2411351	LC_IGP_R26	IgG1_G2N/IgG1_G1N	14	66171541	1	1.51E-11	1.21E-08	+	0.232078	0.2067	+	C	T	0.47476142	0.0341489 +	C	T	0.4984	0.0363
rs2411351	LC_IGP148	IgG2 FG0n total/G0n	14	66171541	1	4.34E-09	2.92E-13	+	0.202145	0.27	+	C	T	0.472359298	0.0342454 +	C	T	0.4984	0.037
rs2411351	LC_IGP97	IgG2_G0	14	66171541	1	1.12E-10	1.68E-13	-	-0.220808	-0.2731	+	C	T	0.473478912	0.0340089 +	C	T	0.4984	0.037
rs2411351	LC_IGP_R41	IgG2_G0F/IgG2_G0	14	66171541	1	3.19E-08	1.14E-12	+	0.189581	0.2618	+	C	T	0.473478912	0.0341123 +	C	T	0.4984	0.0368
rs2411351	LC_IGP138	IgG2_G0n	14	66171541	1	6.32E-11	1.20E-13	-	-0.224558	-0.2752	+	C	T	0.472359298	0.0341265 +	C	T	0.4984	0.0371
rs2411351	LC_IGP_R64	IgG2_G2/IgG2_G1	14	66171541	1	4.61E-09	3.89E-12	+	0.202064	0.2521	+	C	T	0.472528458	0.0342909 +	C	T	0.4984	0.0363
rs8018278	LC_IGP78	IgG1 BG0n/G0n	14	66180088	1	1.86E-14	4.27E-10	-	-0.264409	-0.233	+	G	A	0.47417689	0.0342 +	G	A	0.5025	0.0373
rs8018278	LC_IGP79	IgG1 BG1n/G1n	14	66180088	1	3.48E-13	3.75E-12	-	-0.251909	-0.259	+	G	A	0.47417689	0.0343408 +	G	A	0.5025	0.0373
rs8018278	LC_IGP77	IgG1 Bn	14	66180088	1	2.19E-13	8.41E-12	-	-0.254085	-0.2558	+	G	A	0.47417689	0.034338 +	G	A	0.5025	0.0374
rs8018278	LC_IGP86	IgG1 Bn/Fn total	14	66180088	1	3.03E-14	5.24E-12	-	-0.262891	-0.2585	+	G	A	0.47417689	0.0342891 +	G	A	0.5025	0.0375
rs8018278	LC_IGP84	IgG1 FBn/Bn total	14	66180088	1	2.56E-15	2.94E-16	+	0.274095	0.309	+	G	A	0.47417689	0.0343069 +	G	A	0.5025	0.0378
rs8018278	LC_IGP62	IgG1 FG0n total/G0n	14	66180088	1	7.69E-18	1.22E-12	+	0.295952	0.2668	+	G	A	0.47417689	0.0339968 +	G	A	0.5025	0.0376
rs8018278	LC_IGP63	IgG1 FG1n total/G1n	14	66180088	1	1.13E-17	1.14E-14	+	0.295327	0.2899	+	G	A	0.47417689	0.0341051 +	G	A	0.5025	0.0375
rs8018278	LC_IGP64	IgG1 FG2n total/G2n	14	66180088	1	5.42E-14	1.87E-15	+	0.260442	0.297	+	G	A	0.47417689	0.0343179 +	G	A	0.5025	0.0374
rs8018278	LC_IGP68	IgG1 FG2n/G2n	14	66180088	1	1.18E-10	6.12E-09	+	0.223491	0.216	+	G	A	0.47417689	0.0344676 +	G	A	0.5025	0.0372

rs8018278	LC_IGP61	IgG1 Fn total	14	66180088	1	5.08E-17	2.09E-14	+	0.289219	0.2871	+	G	A	0.47417689	0.0341125 +	G	A	0.5025	0.0376
rs8018278	LC_IGP81	IgG1 Fn/Bn	14	66180088	1	1.01E-13	4.14E-11	+	0.257446	0.2461	+	G	A	0.47417689	0.0343029 +	G	A	0.5025	0.0373
rs8018278	LC_IGP21	IgG1 Fucosylation	14	66180088	1	1.72E-15	3.08E-13	+	0.274028	0.2734	+	G	A	0.473377004	0.0340854 +	G	A	0.5025	0.0375
rs8018278	LC_IGP28	IgG1 G1S1/(G1+G1S1)	14	66180088	1	7.80E-10	4.01E-10	+	0.211066	0.2259	+	G	A	0.473589908	0.0341214 +	G	A	0.5025	0.0361
rs8018278	LC_IGP27	IgG1 GS1/(G0+G+GS1)	14	66180088	1	4.35E-09	1.70E-08	+	0.201849	0.2076	+	G	A	0.473589908	0.0341978 +	G	A	0.5025	0.0368
rs8018278	LC_IGP11	IgG1_G0	14	66180088	1	2.18E-19	8.04E-15	-	-0.306739	-0.2907	+	G	A	0.473927611	0.033641 +	G	A	0.5025	0.0374
rs8018278	LC_IGP_R1	IgG1_G0F/IgG1_G0	14	66180088	1	7.77E-17	2.61E-12	+	0.284317	0.2621	+	G	A	0.473927611	0.0337455 +	G	A	0.5025	0.0374
rs8018278	LC_IGP_R4	IgG1_G0FN/IgG1_G0N	14	66180088	1	6.06E-15	1.81E-13	+	0.267917	0.2776	+	G	A	0.474476118	0.034011 +	G	A	0.5025	0.0377
rs8018278	LC_IGP52	IgG1_G0n	14	66180088	1	1.76E-19	6.96E-15	-	-0.30992	-0.2918	+	G	A	0.47417689	0.0338937 +	G	A	0.5025	0.0375
rs8018278	LC_IGP14	IgG1_G0N	14	66180088	1	2.42E-17	1.31E-11	-	-0.290333	-0.2521	+	G	A	0.474476118	0.0338892 +	G	A	0.5025	0.0372
rs8018278	LC_IGP55	IgG1_G0Nn	14	66180088	1	1.03E-17	1.39E-11	-	-0.295445	-0.252	+	G	A	0.47417689	0.0340731 +	G	A	0.5025	0.0373
rs8018278	LC_IGP12	IgG1_G1	14	66180088	1	4.38E-16	8.26E-14	-	-0.278009	-0.2801	+	G	A	0.473927611	0.0338557 +	G	A	0.5025	0.0375
rs8018278	LC_IGP_R2	IgG1_G1F/IgG1_G1	14	66180088	1	2.36E-17	9.08E-14	+	0.28975	0.2792	+	G	A	0.473927611	0.0338099 +	G	A	0.5025	0.0374
rs8018278	LC_IGP_R5	IgG1_G1FN/IgG1_G1N	14	66180088	1	3.03E-14	1.90E-17	+	0.262024	0.3197	+	G	A	0.475288745	0.0341799 +	G	A	0.5025	0.0376
rs8018278	LC_IGP53	IgG1_G1n	14	66180088	1	2.98E-15	1.49E-13	-	-0.272528	-0.2769	+	G	A	0.47417689	0.0341948 +	G	A	0.5025	0.0375
rs8018278	LC_IGP15	IgG1_G1N	14	66180088	1	2.24E-11	4.78E-11	-	-0.231067	-0.2453	+	G	A	0.475288745	0.0342987 +	G	A	0.5025	0.0373
rs8018278	LC_IGP56	IgG1_G1Nn	14	66180088	1	8.66E-12	1.04E-10	-	-0.236696	-0.2406	+	G	A	0.47417689	0.0344143 +	G	A	0.5025	0.0372
rs8018278	LC_IGP_R32	IgG1_G1S1/IgG1_G1	14	66180088	1	1.39E-09	6.26E-10	+	0.207945	0.2236	+	G	A	0.473589908	0.0341383 +	G	A	0.5025	0.0362
rs8018278	LC_IGP_R3	IgG1_G2F/IgG1_G2	14	66180088	1	2.14E-15	5.86E-15	+	0.271866	0.2911	+	G	A	0.473606051	0.033937 +	G	A	0.5025	0.0373
rs8018278	LC_IGP_R8	IgG1_G2FS1/IgG1_G2S1	14	66180088	1	4.72E-10	8.50E-13	+	0.214174	0.2662	+	G	A	0.473316738	0.03418 +	G	A	0.5025	0.0372
rs8018278	LC_IGP_R26	IgG1_G2N/IgG1_G1N	14	66180088	1	1.46E-11	4.96E-09	+	0.232652	0.2132	+	G	A	0.474889815	0.0342122 +	G	A	0.5025	0.0365
rs8018278	LC_IGP148	IgG2 FG0n total/G0n	14	66180088	1	4.70E-09	3.37E-13	+	0.202074	0.2708	+	G	A	0.472487377	0.0343109 +	G	A	0.5025	0.0372
rs8018278	LC_IGP97	IgG2_G0	14	66180088	1	9.93E-11	1.74E-13	-	-0.221816	-0.2747	+	G	A	0.473606051	0.0340694 +	G	A	0.5025	0.0373
rs8018278	LC_IGP_R41	IgG2_G0F/IgG2_G0	14	66180088	1	3.34E-08	1.45E-12	+	0.18965	0.2621	+	G	A	0.473606051	0.0341764 +	G	A	0.5025	0.037
rs8018278	LC_IGP138	IgG2_G0n	14	66180088	1	5.73E-11	1.24E-13	-	-0.225481	-0.2768	+	G	A	0.472487377	0.0341881 +	G	A	0.5025	0.0373
rs8018278	LC_IGP_R64	IgG2_G2/IgG2_G1	14	66180088	1	8.52E-09	3.39E-12	+	0.198919	0.2544	+	G	A	0.472655753	0.034367 +	G	A	0.5025	0.0366
rs11627067	LC_IGP78	IgG1 BG0n/G0n	14	66182953	1	1.92E-13	3.46E-10	-	-0.254097	-0.2338	+	G	T	0.484726146	0.0342561 +	G	T	0.5099	0.0372
rs11627067	LC_IGP79	IgG1 BG1n/G1n	14	66182953	1	2.21E-12	5.67E-12	-	-0.243315	-0.2563	+	G	T	0.484726146	0.0343868 +	G	T	0.5099	0.0372
rs11627067	LC_IGP77	IgG1 Bn	14	66182953	1	1.26E-12	9.36E-12	-	-0.246025	-0.2548	+	G	T	0.484726146	0.034382 +	G	T	0.5099	0.0374
rs11627067	LC_IGP86	IgG1 Bn/Fn total	14	66182953	1	2.22E-13	6.09E-12	-	-0.254012	-0.2572	+	G	T	0.484726146	0.0343382 +	G	T	0.5099	0.0374
rs11627067	LC_IGP84	IgG1 FBn/Bn total	14	66182953	1	5.47E-14	6.93E-16	+	0.260855	0.3043	+	G	T	0.484726146	0.0343785 +	G	T	0.5099	0.0377
rs11627067	LC_IGP62	IgG1 FG0n total/G0n	14	66182953	1	4.02E-16	1.69E-12	+	0.280316	0.2646	+	G	T	0.484726146	0.0340865 +	G	T	0.5099	0.0375
rs11627067	LC_IGP63	IgG1 FG1n total/G1n	14	66182953	1	3.11E-16	3.69E-14	+	0.282187	0.2836	+	G	T	0.484726146	0.0341817 +	G	T	0.5099	0.0375
rs11627067	LC_IGP64	IgG1 FG2n total/G2n	14	66182953	1	3.55E-13	6.87E-15	+	0.251995	0.2902	+	G	T	0.484726146	0.0343646 +	G	T	0.5099	0.0373
rs11627067	LC_IGP68	IgG1 FG2n/G2n	14	66182953	1	2.89E-10	8.55E-09	+	0.218857	0.2136	+	G	T	0.484726146	0.0344937 +	G	T	0.5099	0.0371
rs11627067	LC_IGP61	IgG1 Fn total	14	66182953	1	1.40E-15	5.34E-14	+	0.27578	0.2821	+	G	T	0.484726146	0.0341891 +	G	T	0.5099	0.0375
rs11627067	LC_IGP81	IgG1 Fn/Bn	14	66182953	1	5.79E-13	4.57E-11	+	0.249529	0.2451	+	G	T	0.484726146	0.0343468 +	G	T	0.5099	0.0372
rs11627067	LC_IGP21	IgG1 Fucosylation	14	66182953	1	4.60E-14	1.00E-12	+	0.259972	0.267	+	G	T	0.483874229	0.0341585 +	G	T	0.5099	0.0374
rs11627067	LC_IGP28	IgG1 G1S1/(G1+G1S1)	14	66182953	1	1.76E-09	1.10E-09	+	0.206678	0.2194	+	G	T	0.48400367	0.0341434 +	G	T	0.5099	0.036
rs11627067	LC_IGP27	IgG1 GS1/(G0+G+GS1)	14	66182953	1	1.90E-08	2.16E-08	+	0.193393	0.205	+	G	T	0.48400367	0.0342333 +	G	T	0.5099	0.0366
rs11627067	LC_IGP11	IgG1_G0	14	66182953	1	1.84E-17	9.07E-15	-	-0.290131	-0.2892	+	G	T	0.48432865	0.0337375 +	G	T	0.5099	0.0373
rs11627067	LC_IGP_R1	IgG1_G0F/IgG1_G0	14	66182953	1	2.67E-15	3.65E-12	+	0.270013	0.2598	+	G	T	0.48432865	0.0338237 +	G	T	0.5099	0.0374
rs11627067	LC_IGP_R4	IgG1_G0FN/IgG1_G0N	14	66182953	1	2.03E-13	2.00E-13	+	0.252596	0.2766	+	G	T	0.484902633	0.034091 +	G	T	0.5099	0.0376
rs11627067	LC_IGP52	IgG1_G0n	14	66182953	1	1.64E-17	8.09E-15	-	-0.29287	-0.2903	+	G	T	0.484726146	0.0339953 +	G	T	0.5099	0.0374
rs11627067	LC_IGP14	IgG1_G0N	14	66182953	1	3.65E-16	7.90E-12	-	-0.279588	-0.2541	+	G	T	0.484902633	0.0339528 +	G	T	0.5099	0.0372
rs11627067	LC_IGP55	IgG1_G0Nn	14	66182953	1	2.12E-16	8.46E-12	-	-0.283491	-0.254	+	G	T	0.484726146	0.0341437 +	G	T	0.5099	0.0372
rs11627067	LC_IGP12	IgG1_G1	14	66182953	1	7.70E-15	3.06E-13	-	-0.266137	-0.2732	+	G	T	0.48432865	0.0339205 +	G	T	0.5099	0.0375
rs11627067	LC_IGP_R2	IgG1_G1F/IgG1_G1	14	66182953	1	3.90E-16	2.82E-13	+	0.278649	0.273	+	G	T	0.48432865	0.0338734 +	G	T	0.5099	0.0374
rs11627067	LC_IGP_R5	IgG1_G1FN/IgG1_G1N	14	66182953	1	4.60E-13	6.54E-17	+	0.249883	0.3133	+	G	T	0.485760148	0.0342465 +	G	T	0.5099	0.0375
rs11627067	LC_IGP53	IgG1_G1n	14	66182953	1	5.24E-14	5.47E-13	-	-0.260171	-0.27	+	G	T	0.484726146	0.0342621 +	G	T	0.5099	0.0374
rs11627067	LC_IGP15	IgG1_G1N	14	66182953	1	9.71E-11	7.01E-11	-	-0.223698	-0.2428	+	G	T	0.485760148	0.0343394 +	G	T	0.5099	0.0372
rs11627067	LC_IGP56	IgG1_G1Nn	14	66182953	1	4.22E-11	1.51E-10	-	-0.228847	-0.2382	+	G	T	0.484726146	0.0344549 +	G	T	0.5099	0.0372
rs11627067	LC_IGP_R32	IgG1_G1S1/IgG1_G1	14	66182953	1	3.03E-09	1.70E-09	+	0.203694	0.2171	+	G	T	0.48400367	0.0341596 +	G	T	0.5099	0.036
rs11627067	LC_IGP_R3	IgG1_G2F/IgG1_G2	14	66182953	1	1.54E-14	2.17E-14	+	0.263557	0.2842	+	G	T	0.484013447	0.0339828 +	G	T	0.5099	0.0372
rs11627067	LC_IGP_R8	IgG1_G2FS1/IgG1_G2S1	14	66182953	1	4.10E-09	3.17E-12	+	0.202365	0.2586	+	G	T	0.483717776	0.0342288 +	G	T	0.5099	0.0371
rs11627067	LC_IGP_R26	IgG1_G2N/IgG1_G1N	14	66182953	1	4.02E-10	5.18E-09	+	0.215754	0.2124	+	G	T	0.4854	0.03429 +	G	T	0.5099	0.0364
rs11627067	LC_IGP148	IgG2 FG0n total/G0n	14	66182953	1	1.17E-08	1.42E-12	+	0.196843	0.2627	+	G	T	0.482971675	0.0343322 +	G	T	0.51	0.0371
rs11627067	LC_IGP97	IgG2_G0	14	66182953	1	5.00E-10	7.59E-13	-	-0.213417	-0.2663	+	G	T	0.484013447	0.0341078 +	G	T	0.5099	0.0372
rs11627067	LC_IGP138	IgG2_G0n	14	66182953	1	3.00E-10	5.41E-13	-	-0.216931	-0.2684	+	G	T	0.482971675	0.0342247 +	G	T	0.51	0.0372
rs139525066	LC_IGP78	IgG1 BG0n/G0n	14	66184172	1	7.20E-13	8.38E-07	-	-0.252828	-0.1943	+	C	T	0.582873296	0.0349478 +	C	T	0.6155	0.0394
rs139525066	LC_IGP79	IgG1 BG1n/G1n	14	66184172	1	5.41E-12	2.33E-07	-	-0.243627	-0.2036	+	C	T	0.582873296	0.035072 +	C	T	0.6155	0.0394
rs139525066	LC_IGP77	IgG1 Bn	14	66184172	1	1.90E-12	1.47E-07	-	-0.2488	-0.2081	+	C	T	0.582873296	0.0350568 +	C	T	0.6155	0.0396
rs139525066	LC_IGP86	IgG1 Bn/Fn total	14	66184172	1	3.24E-13	1.29E-07	-	-0.257173	-0.2091	+	C	T	0.582873296	0.0350114 +	C	T	0.6155	0.0396
rs139525066	LC_IGP84	IgG1 FBn/Bn total	14	66184172	1	5.83E-14	5.81E-10	+	0.265621	0.2467	+	C	T	0.582873296	0.0350458 +	C	T	0.6155	0.0398
rs139525066	LC_IGP62	IgG1 FG0n total/G0n	14	66184172	1	3.87E-17	1.07E-08	+	0.295318	0.227	+	C	T	0.582873296	0.0346971 +	C	T	0.6155	0.0397
rs139525066	LC_IGP63	IgG1 FG1n total/G1n	14	66184172	1	2.73E-15	8.32E-09	+	0.278462	0.2282	+	C	T	0.582873296	0.0348901 +	C	T	0.6155	0.0396
rs139525066	LC_IGP64	IgG1 FG2n total/G2n	14	66184172	1	2.31E-13	5.30E-10	+	0.258884	0.2442	+	C	T	0.582873296	0.0350211 +	C	T	0.6155	0.0393
rs139525066	LC_IGP61	IgG1 Fn total	14	66184172	1	1.10E-15	2.94E-09	+	0.282151	0.2353	+	C	T	0.582873296	0.0348462 +	C	T	0.6155	0.0396
rs139525066	LC_IGP81	IgG1 Fn/Bn	14	66184172	1	1.09E-12	2.36E-07	+	0.251343	0.2036	+	C	T	0.582873296	0.0350256 +	C	T	0.6155	0.0394
rs139525066	LC_IGP21	IgG1 Fucosylation	14	66184172	1	7.27E-14	1.33E-08	+	0.26224	0.2249	+	C	T	0.582770345	0.0347376 +	C	T	0.6155	0.0396
rs139525066	LC_IGP28	IgG1 G1S1/(G1+G1S1)	14	66184172	1	8.92E-09	3.91E-07	+	0.201097	0.1926	+	C	T	0.582725076	0.0347904 +	C	T	0.6155	0.038
rs139525066	LC_IGP11	IgG1_G0	14	66184172	1	6.98E-19	6.77E-11	-	-0.307883	-0.2576	+	C	T	0.583234881	0.0342681 +	C	T	0.6155	0.0395
rs139525066	LC_IGP_R1	IgG1_G0F/IgG1_G0	14	66184172	1	9.97E-17	1.68E-08	+	0.288406	0.2232	+	C	T	0.583234881	0.0343555 +	C	T	0.6155	0.0396
rs139525066	LC_IGP_R4	IgG1_G0FN/IgG1_G0N	14	66184172	1	1.72E-12	3.82E-09	+	0.247292	0.2346	+	C	T	0.583744948	0.0347769 +	C	T	0.6155	0.0398
rs139525066	LC_IGP52	IgG1_G0n	14	66184172	1	4.83E-19	7.22E-11	-	-0.312198	-0.2578	+	C	T	0.582873296	0.0345791 +	C	T	0.6155	0.0396
rs139525066	LC_IGP14	IgG1_G0N	14	66184172	1	1.01E-14	4.12E-08	-	-0.270726	-0.2158	+	C	T	0.583744948	0.0346608 +	C	T	0.6155	0.0394
rs139525066	LC_IGP55	IgG1_G0Nn	14	66184172	1	1.97E-15	5.16E-08	-	-0.279607	-0.2146	+	C	T	0.582873296	0.0348525 +	C	T	0.6155	0.0394
rs139525066	LC_IGP12	IgG1_G1	14	66184172	1	5.45E-15	1.47E-08	-	-0.272468	-0.2243	+	C	T	0.582929444	0.0345291 +	C	T	0.6155	0.0396
rs139525066	LC_IGP_R2	IgG1_G1F/IgG1_G1	14	66184172	1	1.15E-15	1.79E-08	+	0.279197	0.2226	+	C	T	0.582929444	0.0345109 +	C	T	0.6155	0.0395
rs139525066	LC_IGP_R5	IgG1_G1FN/IgG1_G1N	14	66184172	1	2.13E-12	3.39E-10	+	0.247196	0.2486	+	C	T	0.584105474	0.0349114 +	C	T	0.6155	0.0396
rs139525066	LC_IGP53	IgG1_G1n	14	66184172	1	1.25E-13	2.57E-08	-	-0.261265	-0.2204	+	C	T	0.582873296	0.0349442 +	C	T	0.6155	0.0396
rs139525066	LC_IGP15	IgG1_G1N	14	66184172	1	2.45E-10	6.79E-07	-	-0.222926	-0.1957	+	C	T	0.584105474	0.0349932 +	C	T	0.6155	0.0394
rs139525066	LC_IGP56	IgG1_G1Nn	14	66184172	1	5.54E-11	1.22E-06	-	-0.231864	-0.191	+	C	T	0.582873296	0.0351281 +	C	T	0.6155	0.0394
rs139525066	LC_IGP_R32	IgG1_G1S1/IgG1_G1	14	66184172	1	1.73E-08	5.79E-07	+	0.197198	0.19	+	C	T	0.582725076	0.0348094 +	C	T	0.6155	0.038
rs139525066	LC_IGP_R3	IgG1_G2F/IgG1_G2	14	66184172	1	1.73E-14	7.64E-10	+	0.267645	0.2415	+	C	T	0.582980134	0.0345809 +	C	T	0.6155	0.0393
rs139525066	LC_IGP_R8	IgG1_G2FS1/IgG1_G2S1	14	66184172	1	2.18E-09	3.74E-09	+	0.209453	0.231	+	C	T	0.582624007	0.0348058 +	C	T	0.6155	0.0392
rs139525066	LC_IGP_R26	IgG1_G2N/IgG1_G1N	14	66184172	1	3.40E-09	2.32E-06	+	0.207918	0.1819	+	C	T	0.583800926	0.0349804 +	C	T	0.6155	0.0385
rs4902412	LC_IGP84	IgG1 FBn/Bn total	14	66187067	1	7.92E-11	1.42E-07	+	0.229117	0.1988	+	C	T	0.539024783	0.0350008 +	C	T	0.5606	0.0378
rs4902412	LC_IGP63	IgG1 FG1n total/G1n	14	66187067	1	1.21E-11	1.65E-06	+	0.238135	0.18	+	C	T	0.539024783	0.0348707 +	C	T	0.5606	0.0376
rs4902412	LC_IGP64	IgG1 FG2n total/G2n	14	66187067	1	6.89E-10	1.06E-07	+	0.21717	0.1986	+	C	T	0.539024783	0.0349927 +	C	T	0.5606	0.0374
rs4902412	LC_IGP61	IgG1 Fn total	14	66187067	1	1.49E-11	1.23E-06	+	0.236895	0.1823	+	C	T	0.539024783	0.0348507 +	C	T	0.5606	0.0376
rs4902412	LC_IGP11	IgG1_G0	14	66187067	1	1.49E-13	1.46E-07	-	-0.255828	-0.1968	+	C	T	0.539086133	0.0343341 +	C	T	0.5606	0.0374
rs4902412	LC_IGP_R4	IgG1_G0FN/IgG1_G0N	14	66187067	1	2.45E-10	1.75E-06	+	0.220941	0.1804	+	C	T	0.539488059	0.0346834 +	C	T	0.5606	0.0377
rs4902412	LC_IGP52	IgG1_G0n	14	66187067	1	4.96E-14	1.80E-07	-	-0.263194	-0.1957	+	C	T	0.539024783	0.034627 +	C	T	0.5606	0.0375
rs4902412	LC_IGP12	IgG1_G1	14	66187067	1	9.55E-11	2.25E-06	-	-0.225126	-0.1776	+	C	T	0.538780696	0.0345463 +	C	T	0.5606	0.0376
rs4902412	LC_IGP_R5	IgG1_G1FN/IgG1_G1N	14	66187067	1	1.19E-09	5.31E-08	+	0.213193	0.2044	+	C	T	0.540273063	0.0348495 +	C	T	0.5606	0.0376
rs4902412	LC_IGP_R3	IgG1_G2F/IgG1_G2	14	66187067	1	9.12E-11	1.75E-07	+	0.225544	0.1948	+	C	T	0.538804401	0.0345727 +	C	T	0.5606	0.0373
rs4143898	LC_IGP84	IgG1 FBn/Bn total	14	66188882	1	1.94E-12	8.52E-08	+	0.246471	0.2025	+	A	T	0.536601301	0.0347425 +	A	T	0.5546	0.0378
rs4143898	LC_IGP63	IgG1 FG1n total/G1n	14	66188882	1	6.91E-14	8.61E-07	+	0.261319	0.1848	+	A	T	0.536601301	0.0345825 +	A	T	0.5546	0.0376
rs4143898	LC_IGP64	IgG1 FG2n total/G2n	14	66188882	1	1.43E-11	4.78E-08	+	0.236292	0.204	+	A	T	0.536601301	0.0347311 +	A	T	0.5546	0.0374
rs4143898	LC_IGP61	IgG1 Fn total	14	66188882	1	1.22E-13	7.43E-07	+	0.258579	0.1859	+	A	T	0.536601301	0.0345701 +	A	T	0.5546	0.0376
rs4143898	LC_IGP11	IgG1_G0	14	66188882	1	5.07E-16	1.29E-07	-	-0.278934	-0.1979	+	A	T	0.536696701	0.0340442 +	A	T	0.5546	0.0375
rs4143898	LC_IGP_R4	IgG1_G0FN/IgG1_G0N	14	66188882	1	6.45E-12	1.61E-06	+	0.23828	0.181	+	A	T	0.537092774	0.0344318 +	A	T	0.5546	0.0377
rs4143898	LC_IGP52	IgG1_G0n	14	66188882	1	1.46E-16	1.59E-07	-	-0.286608	-0.1967	+	A	T	0.536601301	0.0343265 +	A	T	0.5546	0.0375
rs4143898	LC_IGP12	IgG1_G1	14	66188882	1	1.08E-12	1.06E-06	-	-0.246003	-0.1831	+	A	T	0.536391265	0.0342799 +	A	T	0.5546	0.0375
rs4143898	LC_IGP_R2	IgG1_G1F/IgG1_G1	14	66188882	1	1.23E-13	1.99E-06	+	0.256088	0.1781	+	A	T	0.536391265	0.0342492 +	A	T	0.5546	0.0375
rs4143898	LC_IGP_R5	IgG1_G1FN/IgG1_G1N	14	66188882	1	3.09E-11	2.52E-08	+	0.231389	0.2097	+	A	T	0.537867466	0.0345953 +	A	T	0.5546	0.0376
rs4143898	LC_IGP53	IgG1_G1n	14	66188882	1	7.18E-12	1.74E-06	-	-0.239295	-0.1793	+	A	T	0.536601301	0.0346533 +	A	T	0.5546	0.0375
rs4143898	LC_IGP_R3	IgG1_G2F/IgG1_G2	14	66188882	1	1.62E-12	7.95E-08	+	0.244286	0.2002	+	A	T	0.536413509	0.0343153 +	A	T	0.5546	0.0373
rs4143898	LC_IGP_R8	IgG1_G2FS1/IgG1_G2S1	14	66188882	1	1.29E-08	5.87E-07	+	0.19715	0.186	+	A	T	0.536085828	0.0344857 +	A	T	0.5546	0.0372
rs4143898	LC_IGP97	IgG2_G0	14	66188882	1	2.19E-10	2.33E-06	-	-0.219137	-0.1764	+	A	T	0.536107885	0.0343045 +	A	T	0.5546	0.0374
rs11622829	LC_IGP78	IgG1 BG0n/G0n	14	66191782	1	2.31E-14	3.75E-10	-	-0.263615	-0.2339	+	A	T	0.474490397	0.0342236 +	A	T	0.5031	0.0373
rs11622829	LC_IGP79	IgG1 BG1n/G1n	14	66191782	1	3.73E-13	3.49E-12	-	-0.251734	-0.2595	+	A	T	0.474490397	0.0343613 +	A	T	0.5031	0.0373
rs11622829	LC_IGP77	IgG1 Bn	14	66191782	1	2.57E-13	7.72E-12	-	-0.253498	-0.2564	+	A	T	0.474490397	0.0343604 +	A	T	0.5031	0.0375
rs11622829	LC_IGP86	IgG1 Bn/Fn total	14	66191782	1	3.54E-14	4.71E-12	-	-0.262347	-0.2592	+	A	T	0.474490397	0.0343115 +	A	T	0.5031	0.0375
rs11622829	LC_IGP84	IgG1 FBn/Bn total	14	66191782	1	2.80E-15	2.48E-16	+	0.273869	0.3099	+	A	T	0.474490397	0.0343278 +	A	T	0.5031	0.0378
rs11622829	LC_IGP62	IgG1 FG0n total/G0n	14	66191782	1	9.02E-18	1.02E-12	+	0.295501	0.2678	+	A	T	0.474490397	0.034019 +	A	T	0.5031	0.0376
rs11622829	LC_IGP63	IgG1 FG1n total/G1n	14	66191782	1	1.13E-17	1.04E-14	+	0.295499	0.2905	+	A	T	0.474490397	0.034124 +	A	T	0.5031	0.0376
rs11622829	LC_IGP64	IgG1 FG2n total/G2n	14	66191782	1	5.73E-14	1.83E-15	+	0.26034	0.2973	+	A	T	0.474490397	0.0343381 +	A	T	0.5031	0.0374

rs11622829	LC_IGP68	IgG1 FG2n/G2n	14	66191782	1	1.27E-10	6.15E-09	+	0.223248	0.2161	+	A	T	0.474490397	0.0344883	+	A	T	0.5031	0.0372
rs11622829	LC_IGP61	IgG1 Fn total	14	66191782	1	5.38E-17	1.86E-14	+	0.289153	0.2878	+	A	T	0.474490397	0.0341327	+	A	T	0.5031	0.0376
rs11622829	LC_IGP81	IgG1 Fn/Bn	14	66191782	1	1.19E-13	4.02E-11	+	0.256853	0.2464	+	A	T	0.474490397	0.0343255	+	A	T	0.5031	0.0373
rs11622829	LC_IGP21	IgG1 Fucosylation	14	66191782	1	1.73E-15	3.30E-13	+	0.274155	0.2732	+	A	T	0.473688964	0.0341043	+	A	T	0.5031	0.0375
rs11622829	LC_IGP28	IgG1 G1S1/(G1+G1S1)	14	66191782	1	1.15E-09	4.56E-10	+	0.209086	0.2252	+	A	T	0.473899388	0.0341479	+	A	T	0.5031	0.0361
rs11622829	LC_IGP27	IgG1 GS1/(G0+G+GS1)	14	66191782	1	8.24E-09	1.89E-08	+	0.19832	0.207	+	A	T	0.473899388	0.0342295	+	A	T	0.5031	0.0368
rs11622829	LC_IGP11	IgG1_G0	14	66191782	1	2.70E-19	6.98E-15	-	-0.306131	-0.2916	+	A	T	0.474236714	0.0336637	+	A	T	0.5031	0.0374
rs11622829	LC_IGP_R1	IgG1_G0F/IgG1_G0	14	66191782	1	8.84E-17	2.17E-12	+	0.283962	0.2632	+	A	T	0.474236714	0.0337665	+	A	T	0.5031	0.0375
rs11622829	LC_IGP_R4	IgG1_G0FN/IgG1_G0N	14	66191782	1	6.86E-15	1.55E-13	+	0.267538	0.2786	+	A	T	0.474785977	0.0340323	+	A	T	0.5031	0.0377
rs11622829	LC_IGP52	IgG1_G0n	14	66191782	1	2.13E-19	6.00E-15	-	-0.309399	-0.2927	+	A	T	0.474490397	0.0339165	+	A	T	0.5031	0.0375
rs11622829	LC_IGP14	IgG1_G0N	14	66191782	1	3.30E-17	1.19E-11	-	-0.289271	-0.2527	+	A	T	0.474785977	0.0339143	+	A	T	0.5031	0.0373
rs11622829	LC_IGP55	IgG1_G0Nn	14	66191782	1	1.39E-17	1.26E-11	-	-0.294434	-0.2526	+	A	T	0.474490397	0.0340984	+	A	T	0.5031	0.0373
rs11622829	LC_IGP12	IgG1_G1	14	66191782	1	4.43E-16	7.55E-14	-	-0.278113	-0.2807	+	A	T	0.474236714	0.0338744	+	A	T	0.5031	0.0375
rs11622829	LC_IGP_R2	IgG1_G1F/IgG1_G1	14	66191782	1	2.36E-17	8.29E-14	+	0.289898	0.2798	+	A	T	0.474236714	0.0338284	+	A	T	0.5031	0.0375
rs11622829	LC_IGP_R5	IgG1_G1FN/IgG1_G1N	14	66191782	1	3.00E-14	1.70E-17	+	0.262215	0.3204	+	A	T	0.475599938	0.0341986	+	A	T	0.5031	0.0376
rs11622829	LC_IGP53	IgG1_G1n	14	66191782	1	2.89E-15	1.35E-13	-	-0.27281	-0.2775	+	A	T	0.474490397	0.0342132	+	A	T	0.5031	0.0375
rs11622829	LC_IGP15	IgG1_G1N	14	66191782	1	2.42E-11	4.51E-11	-	-0.230798	-0.2458	+	A	T	0.475599938	0.0343194	+	A	T	0.5031	0.0373
rs11622829	LC_IGP56	IgG1_G1Nn	14	66191782	1	9.24E-12	9.68E-11	-	-0.236514	-0.2412	+	A	T	0.474490397	0.0344348	+	A	T	0.5031	0.0373
rs11622829	LC_IGP_R32	IgG1_G1S1/IgG1_G1	14	66191782	1	1.96E-09	6.99E-10	+	0.206183	0.223	+	A	T	0.473899388	0.034164	+	A	T	0.5031	0.0362
rs11622829	LC_IGP_R3	IgG1_G2F/IgG1_G2	14	66191782	1	2.15E-15	5.85E-15	+	0.272002	0.2913	+	A	T	0.473915342	0.0339557	+	A	T	0.5031	0.0373
rs11622829	LC_IGP_R8	IgG1_G2FS1/IgG1_G2S1	14	66191782	1	3.06E-10	8.55E-13	+	0.216587	0.2663	+	A	T	0.47362584	0.0341898	+	A	T	0.5031	0.0372
rs11622829	LC_IGP_R26	IgG1_G2N/IgG1_G1N	14	66191782	1	1.35E-11	5.98E-09	+	0.233182	0.2122	+	A	T	0.47520216	0.0342295	+	A	T	0.5031	0.0365
rs11622829	LC_IGP148	IgG2 FG0n total/G0n	14	66191782	1	4.85E-09	3.65E-13	+	0.202007	0.2705	+	A	T	0.472798953	0.0343304	+	A	T	0.5031	0.0372
rs11622829	LC_IGP97	IgG2_G0	14	66191782	1	9.23E-11	2.02E-13	-	-0.222312	-0.2741	+	A	T	0.473915342	0.0340866	+	A	T	0.5031	0.0373
rs11622829	LC_IGP_R41	IgG2_G0F/IgG2_G0	14	66191782	1	3.17E-08	1.53E-12	+	0.190078	0.2619	+	A	T	0.473915342	0.0341939	+	A	T	0.5031	0.037
rs11622829	LC_IGP138	IgG2_G0n	14	66191782	1	5.34E-11	1.41E-13	-	-0.225961	-0.2762	+	A	T	0.472798953	0.0342054	+	A	T	0.5031	0.0374
rs11622829	LC_IGP_R64	IgG2_G2/IgG2_G1	14	66191782	1	5.62E-09	2.52E-12	+	0.201422	0.2561	+	A	T	0.472965422	0.0343772	+	A	T	0.5031	0.0366
rs72716430	LC_IGP11	IgG1_G0	14	66192915	1	3.56E-08	5.11E-07	+	0.244201	0.259	+	T	G	0.191867934	0.0440946	+	T	G	0.1552	0.0516
rs72716430	LC_IGP52	IgG1_G0n	14	66192915	1	3.33E-08	6.42E-07	+	0.246443	0.2572	+	T	G	0.191814008	0.0444026	+	T	G	0.1552	0.0517
rs7146829	LC_IGP78	IgG1 BG0n/G0n	14	66194531	1	2.34E-13	3.36E-10	-	-0.253286	-0.2343	+	C	T	0.484094176	0.0342721	+	C	T	0.5092	0.0373
rs7146829	LC_IGP79	IgG1 BG1n/G1n	14	66194531	1	2.57E-12	5.35E-12	-	-0.242679	-0.2569	+	C	T	0.484094176	0.0344019	+	C	T	0.5092	0.0373
rs7146829	LC_IGP77	IgG1 Bn	14	66194531	1	1.56E-12	8.84E-12	-	-0.245091	-0.2555	+	C	T	0.484094176	0.0343984	+	C	T	0.5092	0.0374
rs7146829	LC_IGP86	IgG1 Bn/Fn total	14	66194531	1	2.78E-13	5.71E-12	-	-0.25308	-0.2579	+	C	T	0.484094176	0.0343548	+	C	T	0.5092	0.0374
rs7146829	LC_IGP84	IgG1 FBn/Bn total	14	66194531	1	6.86E-14	7.05E-16	+	0.259934	0.3046	+	C	T	0.484094176	0.0343951	+	C	T	0.5092	0.0378
rs7146829	LC_IGP62	IgG1 FG0n total/G0n	14	66194531	1	5.10E-16	1.63E-12	+	0.279438	0.2652	+	C	T	0.484094176	0.0341033	+	C	T	0.5092	0.0376
rs7146829	LC_IGP63	IgG1 FG1n total/G1n	14	66194531	1	4.05E-16	3.49E-14	+	0.281207	0.2843	+	C	T	0.484094176	0.034199	+	C	T	0.5092	0.0375
rs7146829	LC_IGP64	IgG1 FG2n total/G2n	14	66194531	1	5.22E-13	6.30E-15	+	0.250293	0.2909	+	C	T	0.484094176	0.0343846	+	C	T	0.5092	0.0373
rs7146829	LC_IGP68	IgG1 FG2n/G2n	14	66194531	1	3.89E-10	7.44E-09	+	0.217346	0.2147	+	C	T	0.484094176	0.0345118	+	C	T	0.5092	0.0371
rs7146829	LC_IGP61	IgG1 Fn total	14	66194531	1	1.76E-15	5.06E-14	+	0.274921	0.2828	+	C	T	0.484094176	0.0342057	+	C	T	0.5092	0.0376
rs7146829	LC_IGP81	IgG1 Fn/Bn	14	66194531	1	7.14E-13	4.31E-11	+	0.248634	0.2457	+	C	T	0.484094176	0.0343631	+	C	T	0.5092	0.0373
rs7146829	LC_IGP21	IgG1 Fucosylation	14	66194531	1	6.17E-14	9.55E-13	+	0.258758	0.2676	+	C	T	0.483245376	0.0341765	+	C	T	0.5092	0.0375
rs7146829	LC_IGP28	IgG1 G1S1/(G1+G1S1)	14	66194531	1	2.47E-09	1.22E-09	+	0.204866	0.219	+	C	T	0.483379817	0.034162	+	C	T	0.5092	0.036
rs7146829	LC_IGP27	IgG1 GS1/(G0+G+GS1)	14	66194531	1	3.02E-08	2.15E-08	+	0.190698	0.2053	+	C	T	0.483379817	0.0342545	+	C	T	0.5092	0.0367
rs7146829	LC_IGP11	IgG1_G0	14	66194531	1	2.82E-17	8.53E-15	-	-0.288577	-0.2898	+	C	T	0.483705559	0.0337577	+	C	T	0.5092	0.0374
rs7146829	LC_IGP_R1	IgG1_G0F/IgG1_G0	14	66194531	1	3.33E-15	3.50E-12	+	0.269176	0.2604	+	C	T	0.483705559	0.0338398	+	C	T	0.5092	0.0374
rs7146829	LC_IGP_R4	IgG1_G0FN/IgG1_G0N	14	66194531	1	2.49E-13	2.02E-13	+	0.251754	0.277	+	C	T	0.484278016	0.0341068	+	C	T	0.5092	0.0377
rs7146829	LC_IGP52	IgG1_G0n	14	66194531	1	2.47E-17	7.56E-15	-	-0.291372	-0.291	+	C	T	0.484094176	0.0340156	+	C	T	0.5092	0.0374
rs7146829	LC_IGP14	IgG1_G0N	14	66194531	1	5.34E-16	7.63E-12	-	-0.27813	-0.2546	+	C	T	0.484278016	0.0339722	+	C	T	0.5092	0.0372
rs7146829	LC_IGP55	IgG1_G0Nn	14	66194531	1	3.05E-16	8.13E-12	-	-0.282112	-0.2545	+	C	T	0.484094176	0.0341631	+	C	T	0.5092	0.0372
rs7146829	LC_IGP12	IgG1_G1	14	66194531	1	9.57E-15	2.88E-13	-	-0.265303	-0.2739	+	C	T	0.483705559	0.0339365	+	C	T	0.5092	0.0375
rs7146829	LC_IGP_R2	IgG1_G1F/IgG1_G1	14	66194531	1	5.11E-16	2.63E-13	+	0.277643	0.2737	+	C	T	0.483705559	0.0338905	+	C	T	0.5092	0.0374
rs7146829	LC_IGP_R5	IgG1_G1FN/IgG1_G1N	14	66194531	1	5.45E-13	6.83E-17	+	0.249181	0.3135	+	C	T	0.485132841	0.0342615	+	C	T	0.5092	0.0375
rs7146829	LC_IGP53	IgG1_G1n	14	66194531	1	6.31E-14	5.14E-13	-	-0.259438	-0.2707	+	C	T	0.484094176	0.0342778	+	C	T	0.5092	0.0375
rs7146829	LC_IGP15	IgG1_G1N	14	66194531	1	1.10E-10	6.58E-11	-	-0.223127	-0.2435	+	C	T	0.485132841	0.0343536	+	C	T	0.5092	0.0373
rs7146829	LC_IGP56	IgG1_G1Nn	14	66194531	1	4.67E-11	1.41E-10	-	-0.228416	-0.2389	+	C	T	0.484094176	0.0344689	+	C	T	0.5092	0.0372
rs7146829	LC_IGP_R32	IgG1_G1S1/IgG1_G1	14	66194531	1	4.25E-09	1.87E-09	+	0.201865	0.2168	+	C	T	0.483379817	0.0341782	+	C	T	0.5092	0.0361
rs7146829	LC_IGP_R3	IgG1_G2F/IgG1_G2	14	66194531	1	2.18E-14	2.01E-14	+	0.262143	0.2849	+	C	T	0.483389976	0.0340016	+	C	T	0.5092	0.0372
rs7146829	LC_IGP_R8	IgG1_G2FS1/IgG1_G2S1	14	66194531	1	4.54E-09	3.05E-12	+	0.201861	0.2591	+	C	T	0.483094685	0.0342426	+	C	T	0.5092	0.0371
rs7146829	LC_IGP_R26	IgG1_G2N/IgG1_G1N	14	66194531	1	3.40E-10	5.09E-09	+	0.216726	0.2128	+	C	T	0.48477037	0.0342982	+	C	T	0.5092	0.0364
rs7146829	LC_IGP148	IgG2 FG0n total/G0n	14	66194531	1	1.53E-08	1.46E-12	+	0.195347	0.2629	+	C	T	0.482343596	0.0343498	+	C	T	0.5092	0.0371
rs7146829	LC_IGP97	IgG2_G0	14	66194531	1	7.50E-10	7.94E-13	-	-0.211315	-0.2664	+	C	T	0.483389976	0.0341278	+	C	T	0.5092	0.0372
rs7146829	LC_IGP138	IgG2_G0n	14	66194531	1	4.42E-10	5.65E-13	-	-0.21496	-0.2685	+	C	T	0.482343596	0.0342447	+	C	T	0.5092	0.0372
rs11624104	LC_IGP78	IgG1 BG0n/G0n	14	66196137	1	1.62E-13	3.22E-10	-	-0.25516	-0.2348	+	G	A	0.485344796	0.0342918	+	G	A	0.5101	0.0373
rs11624104	LC_IGP79	IgG1 BG1n/G1n	14	66196137	1	1.89E-12	5.23E-12	-	-0.244344	-0.2572	+	G	A	0.485344796	0.034423	+	G	A	0.5101	0.0373
rs11624104	LC_IGP77	IgG1 Bn	14	66196137	1	1.02E-12	8.39E-12	-	-0.247315	-0.2559	+	G	A	0.485344796	0.034417	+	G	A	0.5101	0.0375
rs11624104	LC_IGP86	IgG1 Bn/Fn total	14	66196137	1	1.79E-13	5.57E-12	-	-0.255295	-0.2582	+	G	A	0.485344796	0.0343731	+	G	A	0.5101	0.0375
rs11624104	LC_IGP84	IgG1 FBn/Bn total	14	66196137	1	4.22E-14	6.93E-16	+	0.26231	0.305	+	G	A	0.485344796	0.0344125	+	G	A	0.5101	0.0378
rs11624104	LC_IGP62	IgG1 FG0n total/G0n	14	66196137	1	3.41E-16	1.63E-12	+	0.281302	0.2655	+	G	A	0.485344796	0.0341222	+	G	A	0.5101	0.0376
rs11624104	LC_IGP63	IgG1 FG1n total/G1n	14	66196137	1	2.27E-16	3.61E-14	+	0.283802	0.2844	+	G	A	0.485344796	0.0342143	+	G	A	0.5101	0.0375
rs11624104	LC_IGP64	IgG1 FG2n total/G2n	14	66196137	1	1.98E-13	6.39E-15	+	0.254959	0.2911	+	G	A	0.485344796	0.0343918	+	G	A	0.5101	0.0373
rs11624104	LC_IGP68	IgG1 FG2n/G2n	14	66196137	1	1.67E-10	6.97E-09	+	0.222004	0.2151	+	G	A	0.485344796	0.0345218	+	G	A	0.5101	0.0371
rs11624104	LC_IGP61	IgG1 Fn total	14	66196137	1	1.07E-15	5.20E-14	+	0.277228	0.2829	+	G	A	0.485344796	0.0342227	+	G	A	0.5101	0.0376
rs11624104	LC_IGP81	IgG1 Fn/Bn	14	66196137	1	4.68E-13	3.99E-11	+	0.250796	0.2463	+	G	A	0.485344796	0.0343818	+	G	A	0.5101	0.0373
rs11624104	LC_IGP21	IgG1 Fucosylation	14	66196137	1	3.78E-14	1.00E-12	+	0.261132	0.2676	+	G	A	0.484489827	0.0341932	+	G	A	0.5101	0.0375
rs11624104	LC_IGP28	IgG1 G1S1/(G1+G1S1)	14	66196137	1	1.84E-09	1.18E-09	+	0.206656	0.2193	+	G	A	0.484614373	0.0341828	+	G	A	0.5101	0.036
rs11624104	LC_IGP27	IgG1 GS1/(G0+G+GS1)	14	66196137	1	1.78E-08	1.90E-08	+	0.193998	0.2062	+	G	A	0.484614373	0.0342705	+	G	A	0.5101	0.0367
rs11624104	LC_IGP11	IgG1_G0	14	66196137	1	1.09E-17	7.64E-15	-	-0.292468	-0.2906	+	G	A	0.484938607	0.0337649	+	G	A	0.5101	0.0374
rs11624104	LC_IGP_R1	IgG1_G0F/IgG1_G0	14	66196137	1	2.33E-15	3.56E-12	+	0.270876	0.2606	+	G	A	0.484938607	0.0338591	+	G	A	0.5101	0.0375
rs11624104	LC_IGP_R4	IgG1_G0FN/IgG1_G0N	14	66196137	1	1.56E-13	1.86E-13	+	0.25405	0.2777	+	G	A	0.485514085	0.0341244	+	G	A	0.5101	0.0377
rs11624104	LC_IGP52	IgG1_G0n	14	66196137	1	9.79E-18	6.88E-15	-	-0.295208	-0.2917	+	G	A	0.485344796	0.0340236	+	G	A	0.5101	0.0375
rs11624104	LC_IGP14	IgG1_G0N	14	66196137	1	2.26E-16	6.62E-12	-	-0.281855	-0.2556	+	G	A	0.485514085	0.0339814	+	G	A	0.5101	0.0372
rs11624104	LC_IGP55	IgG1_G0Nn	14	66196137	1	1.31E-16	7.09E-12	-	-0.285763	-0.2555	+	G	A	0.485344796	0.0341728	+	G	A	0.5101	0.0373
rs11624104	LC_IGP12	IgG1_G1	14	66196137	1	6.43E-15	2.96E-13	-	-0.267203	-0.2739	+	G	A	0.484938607	0.033955	+	G	A	0.5101	0.0376
rs11624104	LC_IGP_R2	IgG1_G1F/IgG1_G1	14	66196137	1	2.66E-16	2.72E-13	+	0.280514	0.2737	+	G	A	0.484938607	0.0339038	+	G	A	0.5101	0.0375
rs11624104	LC_IGP_R5	IgG1_G1FN/IgG1_G1N	14	66196137	1	3.73E-13	6.83E-17	+	0.251124	0.3137	+	G	A	0.486374231	0.0342813	+	G	A	0.5101	0.0376
rs11624104	LC_IGP53	IgG1_G1n	14	66196137	1	4.62E-14	5.37E-13	-	-0.261026	-0.2707	+	G	A	0.485344796	0.0342988	+	G	A	0.5101	0.0375
rs11624104	LC_IGP15	IgG1_G1N	14	66196137	1	9.22E-11	6.39E-11	-	-0.224224	-0.2438	+	G	A	0.486374231	0.0343775	+	G	A	0.5101	0.0373
rs11624104	LC_IGP56	IgG1_G1Nn	14	66196137	1	4.06E-11	1.39E-10	-	-0.229307	-0.2391	+	G	A	0.485344796	0.0344936	+	G	A	0.5101	0.0373
rs11624104	LC_IGP_R32	IgG1_G1S1/IgG1_G1	14	66196137	1	3.14E-09	1.81E-09	+	0.203721	0.2171	+	G	A	0.484614373	0.0341988	+	G	A	0.5101	0.0361
rs11624104	LC_IGP_R3	IgG1_G2F/IgG1_G2	14	66196137	1	8.38E-15	2.06E-14	+	0.266459	0.285	+	G	A	0.484623778	0.0340086	+	G	A	0.5101	0.0373
rs11624104	LC_IGP_R8	IgG1_G2FS1/IgG1_G2S1	14	66196137	1	2.94E-09	3.09E-12	+	0.204467	0.2592	+	G	A	0.484327734	0.0342604	+	G	A	0.5101	0.0372
rs11624104	LC_IGP_R26	IgG1_G2N/IgG1_G1N	14	66196137	1	4.68E-10	4.69E-09	+	0.215201	0.2135	+	G	A	0.486016358	0.0343324	+	G	A	0.5101	0.0364
rs11624104	LC_IGP148	IgG2 FG0n total/G0n	14	66196137	1	1.16E-08	1.35E-12	+	0.197124	0.2635	+	G	A	0.483586515	0.0343708	+	G	A	0.5101	0.0372
rs11624104	LC_IGP97	IgG2_G0	14	66196137	1	4.45E-10	7.68E-13	-	-0.214275	-0.2668	+	G	A	0.484623778	0.0341439	+	G	A	0.5101	0.0372
rs11624104	LC_IGP138	IgG2_G0n	14	66196137	1	2.71E-10	5.43E-13	-	-0.217711	-0.269	+	G	A	0.483586515	0.0342613	+	G	A	0.5101	0.0373
rs1535173	LC_IGP78	IgG1 BG0n/G0n	14	66199139	1	1.79E-13	3.05E-10	-	-0.254405	-0.2347	+	G	C	0.48535254	0.0342523	+	G	C	0.5096	0.0373
rs1535173	LC_IGP79	IgG1 BG1n/G1n	14	66199139	1	2.07E-12	4.96E-12	-	-0.243613	-0.2571	+	G	C	0.48535254	0.0343832	+	G	C	0.5096	0.0372
rs1535173	LC_IGP77	IgG1 Bn	14	66199139	1	1.17E-12	8.06E-12	-	-0.24634	-0.2558	+	G	C	0.48535254	0.0343782	+	G	C	0.5096	0.0374
rs1535173	LC_IGP86	IgG1 Bn/Fn total	14	66199139	1	2.04E-13	5.23E-12	-	-0.254389	-0.2582	+	G	C	0.48535254	0.0343341	+	G	C	0.5096	0.0374
rs1535173	LC_IGP84	IgG1 FBn/Bn total	14	66199139	1	5.44E-14	6.15E-16	+	0.260861	0.305	+	G	C	0.48535254	0.0343761	+	G	C	0.5096	0.0377
rs1535173	LC_IGP62	IgG1 FG0n total/G0n	14	66199139	1	3.24E-16	1.49E-12	+	0.281175	0.2655	+	G	C	0.48535254	0.0340798	+	G	C	0.5096	0.0375
rs1535173	LC_IGP63	IgG1 FG1n total/G1n	14	66199139	1	2.56E-16	3.21E-14	+	0.282963	0.2845	+	G	C	0.48535254	0.0341753	+	G	C	0.5096	0.0375
rs1535173	LC_IGP64	IgG1 FG2n total/G2n	14	66199139	1	2.72E-13	5.83E-15	+	0.253197	0.2911	+	G	C	0.48535254	0.0343568	+	G	C	0.5096	0.0373
rs1535173	LC_IGP68	IgG1 FG2n/G2n	14	66199139	1	2.08E-10	7.24E-09	+	0.220581	0.2147	+	G	C	0.48535254	0.0344845	+	G	C	0.5096	0.0371
rs1535173	LC_IGP61	IgG1 Fn total	14	66199139	1	1.11E-15	4.64E-14	+	0.276735	0.283	+	G	C	0.48535254	0.034182	+	G	C	0.5096	0.0375
rs1535173	LC_IGP81	IgG1 Fn/Bn	14	66199139	1	5.25E-13	3.95E-11	+	0.249964	0.246	+	G	C	0.48535254	0.0343425	+	G	C	0.5096	0.0372
rs1535173	LC_IGP21	IgG1 Fucosylation	14	66199139	1	3.75E-14	8.51E-13	+	0.260851	0.268	+	G	C	0.484497534	0.0341518	+	G	C	0.5096	0.0375
rs1535173	LC_IGP28	IgG1 G1S1/(G1+G1S1)	14	66199139	1	2.49E-09	1.17E-09	+	0.204741	0.2191	+	G	C	0.484622018	0.0341481	+	G	C	0.5096	0.036
rs1535173	LC_IGP27	IgG1 GS1/(G0+G+GS1)	14	66199139	1	3.52E-08	1.89E-08	+	0.189701	0.2059	+	G	C	0.484622018	0.0342435	+	G	C	0.5096	0.0366
rs1535173	LC_IGP11	IgG1_G0	14	66199139	1	1.60E-17	7.51E-15	-	-0.290651	-0.2902	+	G	C	0.484946243	0.0337324	+	G	C	0.5096	0.0373
rs1535173	LC_IGP_R1	IgG1_G0F/IgG1_G0	14	66199139	1	2.05E-15	3.24E-12	+	0.271093	0.2606	+	G	C	0.484946243	0.0338161	+	G	C	0.5096	0.0374
rs1535173	LC_IGP_R4	IgG1_G0FN/IgG1_G0N	14	66199139	1	2.01E-13	1.78E-13	+	0.252619	0.2774	+	G	C	0.485521739	0.0340886	+	G	C	0.5096	0.0377
rs1535173	LC_IGP52	IgG1_G0n	14	66199139	1	1.37E-17	6.73E-15	-	-0.293543	-0.2913	+	G	C	0.48535254	0.0339893	+	G	C	0.5096	0.0374
rs1535173	LC_IGP14	IgG1_G0N	14	66199139	1	3.78E-16	6.57E-12	-	-0.279427	-0.2552	+	G	C	0.485521739	0.0339513	+	G	C	0.5096	0.0372
rs1535173	LC_IGP55	IgG1_G0Nn	14	66199139	1	2.12E-16	7.07E-12	-	-0.283478	-0.255	+	G	C	0.48535254	0.0341414	+	G	C	0.5096	0.0372
rs1535173	LC_IGP12	IgG1_G1	14	66199139	1	6.40E-15	2.68E-13	-	-0.266905	-0.274	+	G	C	0.484946243	0.0339144	+	G	C	0.5096	0.0375
rs1535173	LC_IGP_R2	IgG1_G1F/IgG1_G1	14	66199139	1	3.05E-16	2.44E-13	+	0.279625	0.2739	+	G	C	0.484946243	0.0338661	+	G	C	0.5096	0.0374
rs1535173	LC_IGP_R5	IgG1_G1FN/IgG1_G1N	14	66199139	1	4.42E-13	5.97E-17	+	0.250045	0.3138	+	G	C	0.486381919	0.0342436	+	G	C	0.5096	0.0375
rs1535173	LC_IGP53	IgG1_G1n	14	66199139	1	4.17E-14	4.83E-13	-	-0.261172	-0.2708	+	G	C	0.48535254	0.034255	+	G	C	0.5096	0.0375

rs1535173	LC_IGP15	IgG1_G1N	14	66199139	1	9.14E-11	6.15E-11	-	-0.223998	-0.2437	+	G	C	0.486381919	0.034336	+	G	C	0.5096	0.0373
rs1535173	LC_IGP56	IgG1_G1Nn	14	66199139	1	3.90E-11	1.34E-10	-	-0.229235	-0.239	+	G	C	0.48535254	0.0344509	+	G	C	0.5096	0.0372
rs1535173	LC_IGP_R32	IgG1_G1S1/IgG1_G1	14	66199139	1	4.10E-09	1.79E-09	+	0.20198	0.2169	+	G	C	0.484622018	0.0341634	+	G	C	0.5096	0.036
rs1535173	LC_IGP_R3	IgG1_G2F/IgG1_G2	14	66199139	1	1.12E-14	1.87E-14	+	0.264909	0.2851	+	G	C	0.484631418	0.0339739	+	G	C	0.5096	0.0372
rs1535173	LC_IGP_R8	IgG1_G2FS1/IgG1_G2S1	14	66199139	1	2.22E-09	2.75E-12	+	0.205775	0.2595	+	G	C	0.48433537	0.0342138	+	G	C	0.5096	0.0371
rs1535173	LC_IGP_R26	IgG1_G2N/IgG1_G1N	14	66199139	1	3.87E-10	4.55E-09	+	0.215949	0.2133	+	G	C	0.486024074	0.0342871	+	G	C	0.5096	0.0364
rs1535173	LC_IGP148	IgG2 FG0n total/G0n	14	66199139	1	9.11E-09	1.34E-12	+	0.198287	0.2632	+	G	C	0.483594212	0.0343244	+	G	C	0.5096	0.0371
rs1535173	LC_IGP97	IgG2_G0	14	66199139	1	3.44E-10	6.96E-13	-	-0.215374	-0.2669	+	G	C	0.484631418	0.0340979	+	G	C	0.5096	0.0372
rs1535173	LC_IGP138	IgG2_G0n	14	66199139	1	2.05E-10	5.00E-13	-	-0.218925	-0.2689	+	G	C	0.483594212	0.0342143	+	G	C	0.5096	0.0372
rs1535173	LC_IGP_R64	IgG2_G2/IgG2_G1	14	66199139	1	4.79E-08	7.01E-12	+	0.18869	0.2499	+	G	C	0.483694614	0.0344027	+	G	C	0.5096	0.0364
rs3742597	LC_IGP78	IgG1 BG0n/G0n	14	66200177	1	2.10E-10	3.66E-10	+	0.250892	0.2775	+	G	A	0.266144981	0.0392315	+	G	A	0.2255	0.0443
rs3742597	LC_IGP79	IgG1 BG1n/G1n	14	66200177	1	1.61E-09	1.36E-11	+	0.238908	0.299	+	G	A	0.266144981	0.0393712	+	G	A	0.2255	0.0442
rs3742597	LC_IGP77	IgG1 Bn	14	66200177	1	3.27E-10	2.65E-11	+	0.248828	0.2961	+	G	A	0.266144981	0.0393412	+	G	A	0.2255	0.0444
rs3742597	LC_IGP86	IgG1 Bn/Fn total	14	66200177	1	5.82E-11	1.41E-11	+	0.259048	0.3003	+	G	A	0.266144981	0.0392915	+	G	A	0.2255	0.0444
rs3742597	LC_IGP84	IgG1 FBn/Bn total	14	66200177	1	8.54E-12	3.49E-15	-	-0.270557	-0.3525	+	G	A	0.266144981	0.0393253	+	G	A	0.2255	0.0448
rs3742597	LC_IGP62	IgG1 FG0n total/G0n	14	66200177	1	3.74E-14	3.13E-12	-	-0.297752	-0.3106	+	G	A	0.266144981	0.0389794	+	G	A	0.2255	0.0446
rs3742597	LC_IGP63	IgG1 FG1n total/G1n	14	66200177	1	4.22E-12	2.71E-13	-	-0.273772	-0.3253	+	G	A	0.266144981	0.0392071	+	G	A	0.2255	0.0445
rs3742597	LC_IGP64	IgG1 FG2n total/G2n	14	66200177	1	1.94E-11	9.43E-15	-	-0.265499	-0.343	+	G	A	0.266144981	0.0392843	+	G	A	0.2255	0.0443
rs3742597	LC_IGP68	IgG1 FG2n/G2n	14	66200177	1	1.18E-08	3.07E-09	-	-0.225994	-0.2613	+	G	A	0.266144981	0.0394242	+	G	A	0.2255	0.0441
rs3742597	LC_IGP61	IgG1 Fn total	14	66200177	1	3.10E-13	2.14E-13	-	-0.287583	-0.327	+	G	A	0.266144981	0.0391173	+	G	A	0.2255	0.0446
rs3742597	LC_IGP81	IgG1 Fn/Bn	14	66200177	1	2.04E-10	7.42E-11	-	-0.251542	-0.2881	+	G	A	0.266144981	0.0393083	+	G	A	0.2255	0.0442
rs3742597	LC_IGP21	IgG1 Fucosylation	14	66200177	1	1.45E-11	5.29E-13	-	-0.26621	-0.3211	+	G	A	0.266065351	0.0391415	+	G	A	0.2255	0.0445
rs3742597	LC_IGP11	IgG1_G0	14	66200177	1	7.28E-15	3.63E-14	+	0.303911	0.3357	+	G	A	0.265765425	0.0386987	+	G	A	0.2255	0.0443
rs3742597	LC_IGP_R1	IgG1_G0F/IgG1_G0	14	66200177	1	8.39E-14	1.26E-11	-	-0.291668	-0.3008	+	G	A	0.265765425	0.0387383	+	G	A	0.2255	0.0444
rs3742597	LC_IGP_R4	IgG1_G0FN/IgG1_G0N	14	66200177	1	1.22E-10	1.12E-13	-	-0.253442	-0.3321	+	G	A	0.265804042	0.0391146	+	G	A	0.2255	0.0447
rs3742597	LC_IGP52	IgG1_G0n	14	66200177	1	4.26E-15	3.42E-14	+	0.308233	0.3366	+	G	A	0.266144981	0.0388996	+	G	A	0.2255	0.0444
rs3742597	LC_IGP14	IgG1_G0N	14	66200177	1	1.87E-11	1.41E-11	+	0.264181	0.2982	+	G	A	0.265804042	0.039061	+	G	A	0.2255	0.0441
rs3742597	LC_IGP55	IgG1_G0Nn	14	66200177	1	1.05E-11	1.55E-11	+	0.268442	0.298	+	G	A	0.266144981	0.0391945	+	G	A	0.2255	0.0442
rs3742597	LC_IGP12	IgG1_G1	14	66200177	1	3.36E-12	5.64E-13	+	0.27296	0.3209	+	G	A	0.266070861	0.0389124	+	G	A	0.2255	0.0445
rs3742597	LC_IGP_R2	IgG1_G1F/IgG1_G1	14	66200177	1	4.34E-12	1.28E-12	-	-0.271671	-0.3152	+	G	A	0.266070861	0.0389343	+	G	A	0.2255	0.0444
rs3742597	LC_IGP_R5	IgG1_G1FN/IgG1_G1N	14	66200177	1	1.65E-10	5.56E-16	-	-0.252184	-0.3607	+	G	A	0.265718327	0.0392064	+	G	A	0.2255	0.0445
rs3742597	LC_IGP53	IgG1_G1n	14	66200177	1	1.94E-11	1.26E-12	+	0.265012	0.3157	+	G	A	0.266144981	0.0392128	+	G	A	0.2255	0.0445
rs3742597	LC_IGP15	IgG1_G1N	14	66200177	1	5.97E-09	3.62E-11	+	0.229691	0.2929	+	G	A	0.265718327	0.0392706	+	G	A	0.2255	0.0442
rs3742597	LC_IGP56	IgG1_G1Nn	14	66200177	1	4.21E-09	9.99E-11	+	0.232787	0.2858	+	G	A	0.266144981	0.0394013	+	G	A	0.2255	0.0442
rs3742597	LC_IGP_R3	IgG1_G2F/IgG1_G2	14	66200177	1	3.67E-12	9.80E-15	-	-0.272737	-0.3421	+	G	A	0.265927873	0.0389517	+	G	A	0.2255	0.0442
rs3742597	LC_IGP97	IgG2_G0	14	66200177	1	4.67E-10	9.98E-09	+	0.244231	0.253	+	G	A	0.265927873	0.038965	+	G	A	0.2255	0.0441
rs3742597	LC_IGP_R41	IgG2_G0F/IgG2_G0	14	66200177	1	1.91E-08	2.43E-08	-	-0.220502	-0.2446	+	G	A	0.265927873	0.039038	+	G	A	0.2255	0.0438
rs3742597	LC_IGP138	IgG2_G0n	14	66200177	1	2.55E-10	1.05E-08	+	0.24834	0.2528	+	G	A	0.266353448	0.0390214	+	G	A	0.2256	0.0442
rs12888212	LC_IGP78	IgG1 BG0n/G0n	14	66200458	1	1.78E-13	3.12E-10	-	-0.254504	-0.2346	+	C	T	0.485216543	0.0342622	+	C	T	0.5097	0.0373
rs12888212	LC_IGP79	IgG1 BG1n/G1n	14	66200458	1	2.11E-12	5.26E-12	-	-0.243575	-0.2569	+	C	T	0.485216543	0.0343937	+	C	T	0.5097	0.0372
rs12888212	LC_IGP77	IgG1 Bn	14	66200458	1	1.19E-12	8.39E-12	-	-0.246347	-0.2556	+	C	T	0.485216543	0.0343886	+	C	T	0.5097	0.0374
rs12888212	LC_IGP86	IgG1 Bn/Fn total	14	66200458	1	2.07E-13	5.50E-12	-	-0.254396	-0.258	+	C	T	0.485216543	0.0343445	+	C	T	0.5097	0.0374
rs12888212	LC_IGP84	IgG1 FBn/Bn total	14	66200458	1	5.49E-14	6.66E-16	+	0.260904	0.3046	+	C	T	0.485216543	0.0343863	+	C	T	0.5097	0.0377
rs12888212	LC_IGP62	IgG1 FG0n total/G0n	14	66200458	1	3.37E-16	1.64E-12	+	0.2811	0.265	+	C	T	0.485216543	0.0340906	+	C	T	0.5097	0.0375
rs12888212	LC_IGP63	IgG1 FG1n total/G1n	14	66200458	1	2.72E-16	3.62E-14	+	0.282793	0.2839	+	C	T	0.485216543	0.0341867	+	C	T	0.5097	0.0375
rs12888212	LC_IGP64	IgG1 FG2n total/G2n	14	66200458	1	2.84E-13	6.36E-15	+	0.253078	0.2908	+	C	T	0.485216543	0.0343677	+	C	T	0.5097	0.0373
rs12888212	LC_IGP68	IgG1 FG2n/G2n	14	66200458	1	2.15E-10	7.61E-09	+	0.220461	0.2144	+	C	T	0.485216543	0.0344953	+	C	T	0.5097	0.0371
rs12888212	LC_IGP61	IgG1 Fn total	14	66200458	1	1.17E-15	5.22E-14	+	0.276599	0.2825	+	C	T	0.485216543	0.0341931	+	C	T	0.5097	0.0375
rs12888212	LC_IGP81	IgG1 Fn/Bn	14	66200458	1	5.34E-13	4.07E-11	+	0.24996	0.2459	+	C	T	0.485216543	0.0343529	+	C	T	0.5097	0.0372
rs12888212	LC_IGP21	IgG1 Fucosylation	14	66200458	1	3.88E-14	9.34E-13	+	0.260775	0.2675	+	C	T	0.484361591	0.0341626	+	C	T	0.5097	0.0375
rs12888212	LC_IGP28	IgG1 G1S1/(G1+G1S1)	14	66200458	1	2.74E-09	1.28E-09	+	0.204259	0.2185	+	C	T	0.484487462	0.03416	+	C	T	0.5097	0.036
rs12888212	LC_IGP27	IgG1 GS1/(G0+G+GS1)	14	66200458	1	3.83E-08	1.98E-08	+	0.189251	0.2057	+	C	T	0.484487462	0.0342552	+	C	T	0.5097	0.0366
rs12888212	LC_IGP11	IgG1_G0	14	66200458	1	1.70E-17	8.65E-15	-	-0.290505	-0.2896	+	C	T	0.484811851	0.0337434	+	C	T	0.5097	0.0373
rs12888212	LC_IGP_R1	IgG1_G0F/IgG1_G0	14	66200458	1	2.16E-15	3.59E-12	+	0.270957	0.2601	+	C	T	0.484811851	0.0338269	+	C	T	0.5097	0.0374
rs12888212	LC_IGP_R4	IgG1_G0FN/IgG1_G0N	14	66200458	1	1.99E-13	1.88E-13	+	0.252727	0.2771	+	C	T	0.485387018	0.0340984	+	C	T	0.5097	0.0377
rs12888212	LC_IGP52	IgG1_G0n	14	66200458	1	1.45E-17	7.70E-15	-	-0.293404	-0.2907	+	C	T	0.485216543	0.0340005	+	C	T	0.5097	0.0374
rs12888212	LC_IGP14	IgG1_G0N	14	66200458	1	3.79E-16	6.79E-12	-	-0.279494	-0.2551	+	C	T	0.485387018	0.0339612	+	C	T	0.5097	0.0372
rs12888212	LC_IGP55	IgG1_G0Nn	14	66200458	1	2.12E-16	7.29E-12	-	-0.283562	-0.255	+	C	T	0.485216543	0.0341514	+	C	T	0.5097	0.0372
rs12888212	LC_IGP12	IgG1_G1	14	66200458	1	6.78E-15	3.02E-13	-	-0.266737	-0.2734	+	C	T	0.484811851	0.0339254	+	C	T	0.5097	0.0375
rs12888212	LC_IGP_R2	IgG1_G1F/IgG1_G1	14	66200458	1	3.25E-16	2.77E-13	+	0.279448	0.2732	+	C	T	0.484811851	0.0338772	+	C	T	0.5097	0.0374
rs12888212	LC_IGP_R5	IgG1_G1FN/IgG1_G1N	14	66200458	1	4.52E-13	6.56E-17	+	0.250013	0.3134	+	C	T	0.486246617	0.034254	+	C	T	0.5097	0.0375
rs12888212	LC_IGP53	IgG1_G1n	14	66200458	1	4.40E-14	5.42E-13	-	-0.261005	-0.2703	+	C	T	0.485216543	0.0342662	+	C	T	0.5097	0.0375
rs12888212	LC_IGP15	IgG1_G1N	14	66200458	1	9.36E-11	6.43E-11	-	-0.223941	-0.2435	+	C	T	0.486246617	0.0343465	+	C	T	0.5097	0.0373
rs12888212	LC_IGP56	IgG1_G1Nn	14	66200458	1	3.99E-11	1.39E-10	-	-0.229185	-0.2388	+	C	T	0.485216543	0.0344615	+	C	T	0.5097	0.0372
rs12888212	LC_IGP_R32	IgG1_G1S1/IgG1_G1	14	66200458	1	4.53E-09	1.94E-09	+	0.201483	0.2164	+	C	T	0.484487462	0.0341753	+	C	T	0.5097	0.036
rs12888212	LC_IGP_R3	IgG1_G2F/IgG1_G2	14	66200458	1	1.17E-14	2.05E-14	+	0.264803	0.2847	+	C	T	0.484496944	0.0339846	+	C	T	0.5097	0.0372
rs12888212	LC_IGP_R8	IgG1_G2FS1/IgG1_G2S1	14	66200458	1	2.27E-09	3.04E-12	+	0.205715	0.259	+	C	T	0.484200977	0.0342242	+	C	T	0.5097	0.0371
rs12888212	LC_IGP_R26	IgG1_G2N/IgG1_G1N	14	66200458	1	3.92E-10	4.65E-09	+	0.215937	0.2132	+	C	T	0.48588858	0.0342973	+	C	T	0.5097	0.0364
rs12888212	LC_IGP148	IgG2 FG0n total/G0n	14	66200458	1	8.67E-09	1.73E-12	+	0.198627	0.2619	+	C	T	0.483458744	0.0343335	+	C	T	0.5098	0.0371
rs12888212	LC_IGP97	IgG2_G0	14	66200458	1	3.30E-10	9.33E-13	-	-0.215662	-0.2654	+	C	T	0.484496944	0.0341069	+	C	T	0.5097	0.0372
rs12888212	LC_IGP138	IgG2_G0n	14	66200458	1	1.95E-10	6.59E-13	-	-0.219239	-0.2675	+	C	T	0.483458744	0.0342233	+	C	T	0.5098	0.0372
rs927004	LC_IGP78	IgG1 BG0n/G0n	14	66200911	1	1.78E-13	3.18E-10	-	-0.254427	-0.2345	+	C	G	0.485370198	0.0342533	+	C	G	0.5095	0.0373
rs927004	LC_IGP79	IgG1 BG1n/G1n	14	66200911	1	2.01E-12	5.02E-12	-	-0.243745	-0.2571	+	C	G	0.485370198	0.0343837	+	C	G	0.5095	0.0372
rs927004	LC_IGP77	IgG1 Bn	14	66200911	1	1.15E-12	8.25E-12	-	-0.246453	-0.2557	+	C	G	0.485370198	0.0343788	+	C	G	0.5095	0.0374
rs927004	LC_IGP86	IgG1 Bn/Fn total	14	66200911	1	1.99E-13	5.35E-12	-	-0.254499	-0.2581	+	C	G	0.485370198	0.0343347	+	C	G	0.5095	0.0374
rs927004	LC_IGP84	IgG1 FBn/Bn total	14	66200911	1	5.41E-14	6.40E-16	+	0.260895	0.3049	+	C	G	0.485370198	0.034377	+	C	G	0.5095	0.0377
rs927004	LC_IGP62	IgG1 FG0n total/G0n	14	66200911	1	3.29E-16	1.57E-12	+	0.281121	0.2653	+	C	G	0.485370198	0.0340811	+	C	G	0.5095	0.0375
rs927004	LC_IGP63	IgG1 FG1n total/G1n	14	66200911	1	2.53E-16	3.30E-14	+	0.283025	0.2844	+	C	G	0.485370198	0.0341761	+	C	G	0.5095	0.0375
rs927004	LC_IGP64	IgG1 FG2n total/G2n	14	66200911	1	2.62E-13	5.83E-15	+	0.253375	0.2912	+	C	G	0.485370198	0.034357	+	C	G	0.5095	0.0373
rs927004	LC_IGP68	IgG1 FG2n/G2n	14	66200911	1	1.99E-10	7.20E-09	+	0.22082	0.2148	+	C	G	0.485370198	0.0344846	+	C	G	0.5095	0.0371
rs927004	LC_IGP61	IgG1 Fn total	14	66200911	1	1.10E-15	4.83E-14	+	0.276772	0.2829	+	C	G	0.485370198	0.0341829	+	C	G	0.5095	0.0375
rs927004	LC_IGP81	IgG1 Fn/Bn	14	66200911	1	5.12E-13	4.02E-11	+	0.250086	0.246	+	C	G	0.485370198	0.034343	+	C	G	0.5095	0.0372
rs927004	LC_IGP21	IgG1 Fucosylation	14	66200911	1	3.71E-14	8.91E-13	+	0.260909	0.2678	+	C	G	0.484514488	0.0341527	+	C	G	0.5095	0.0375
rs927004	LC_IGP28	IgG1 G1S1/(G1+G1S1)	14	66200911	1	2.50E-09	1.15E-09	+	0.204725	0.2192	+	C	G	0.484639144	0.0341492	+	C	G	0.5095	0.036
rs927004	LC_IGP27	IgG1 GS1/(G0+G+GS1)	14	66200911	1	3.54E-08	1.91E-08	+	0.189683	0.2059	+	C	G	0.484639144	0.0342447	+	C	G	0.5095	0.0366
rs927004	LC_IGP11	IgG1_G0	14	66200911	1	1.59E-17	7.77E-15	-	-0.290682	-0.2902	+	C	G	0.484963348	0.0337333	+	C	G	0.5095	0.0373
rs927004	LC_IGP_R1	IgG1_G0F/IgG1_G0	14	66200911	1	2.08E-15	3.40E-12	+	0.27104	0.2604	+	C	G	0.484963348	0.0338175	+	C	G	0.5095	0.0374
rs927004	LC_IGP_R4	IgG1_G0FN/IgG1_G0N	14	66200911	1	2.03E-13	1.87E-13	+	0.252583	0.2772	+	C	G	0.485538885	0.0340899	+	C	G	0.5095	0.0377
rs927004	LC_IGP52	IgG1_G0n	14	66200911	1	1.36E-17	6.98E-15	-	-0.293572	-0.2912	+	C	G	0.485370198	0.0339903	+	C	G	0.5095	0.0374
rs927004	LC_IGP14	IgG1_G0N	14	66200911	1	3.66E-16	6.79E-12	-	-0.279566	-0.2551	+	C	G	0.485538885	0.0339517	+	C	G	0.5095	0.0372
rs927004	LC_IGP55	IgG1_G0Nn	14	66200911	1	2.06E-16	7.31E-12	-	-0.283614	-0.2549	+	C	G	0.485370198	0.0341418	+	C	G	0.5095	0.0372
rs927004	LC_IGP12	IgG1_G1	14	66200911	1	6.45E-15	2.78E-13	-	-0.266881	-0.2739	+	C	G	0.484963348	0.0339157	+	C	G	0.5095	0.0375
rs927004	LC_IGP_R2	IgG1_G1F/IgG1_G1	14	66200911	1	3.01E-16	2.51E-13	+	0.279683	0.2738	+	C	G	0.484963348	0.0338669	+	C	G	0.5095	0.0374
rs927004	LC_IGP_R5	IgG1_G1FN/IgG1_G1N	14	66200911	1	4.36E-13	6.11E-17	+	0.25012	0.3138	+	C	G	0.486399139	0.0342444	+	C	G	0.5095	0.0375
rs927004	LC_IGP53	IgG1_G1n	14	66200911	1	4.20E-14	5.00E-13	-	-0.261142	-0.2707	+	C	G	0.485370198	0.0342563	+	C	G	0.5095	0.0375
rs927004	LC_IGP15	IgG1_G1N	14	66200911	1	9.01E-11	6.22E-11	-	-0.224078	-0.2437	+	C	G	0.486399139	0.0343368	+	C	G	0.5095	0.0373
rs927004	LC_IGP56	IgG1_G1Nn	14	66200911	1	3.85E-11	1.35E-10	-	-0.229311	-0.239	+	C	G	0.485370198	0.0344517	+	C	G	0.5095	0.0372
rs927004	LC_IGP_R32	IgG1_G1S1/IgG1_G1	14	66200911	1	4.13E-09	1.75E-09	+	0.201952	0.217	+	C	G	0.484639144	0.0341646	+	C	G	0.5095	0.036
rs927004	LC_IGP_R3	IgG1_G2F/IgG1_G2	14	66200911	1	1.08E-14	1.88E-14	+	0.265067	0.2851	+	C	G	0.484648533	0.0339742	+	C	G	0.5095	0.0372
rs927004	LC_IGP_R8	IgG1_G2FS1/IgG1_G2S1	14	66200911	1	2.19E-09	2.77E-12	+	0.205858	0.2595	+	C	G	0.484352474	0.0342147	+	C	G	0.5095	0.0371
rs927004	LC_IGP_R26	IgG1_G2N/IgG1_G1N	14	66200911	1	3.90E-10	4.49E-09	+	0.215908	0.2134	+	C	G	0.486041667	0.0342883	+	C	G	0.5095	0.0364
rs927004	LC_IGP148	IgG2 FG0n total/G0n	14	66200911	1	9.02E-09	1.41E-12	+	0.198348	0.263	+	C	G	0.483611453	0.0343253	+	C	G	0.5096	0.0371
rs927004	LC_IGP97	IgG2_G0	14	66200911	1	3.37E-10	7.51E-13	-	-0.215498	-0.2666	+	C	G	0.484648533	0.0340985	+	C	G	0.5095	0.0372
rs927004	LC_IGP138	IgG2_G0n	14	66200911	1	2.00E-10	5.32E-13	-	-0.219048	-0.2687	+	C	G	0.483611453	0.0342149	+	C	G	0.5096	0.0372
rs927004	LC_IGP_R64	IgG2_G2/IgG2_G1	14	66200911	1	4.65E-08	7.00E-12	+	0.188873	0.2499	+	C	G	0.48371175	0.0344032	+	C	G	0.5095	0.0365
rs1950557	LC_IGP78	IgG1 BG0n/G0n	14	66201757	1	2.91E-10	3.05E-10	+	0.249272	0.2793	+	T	C	0.265799257	0.0392944	+	T	C	0.2249	0.0444
rs1950557	LC_IGP79	IgG1 BG1n/G1n	14	66201757	1	2.65E-09	1.19E-11	+	0.23607	0.3006	+	T	C	0.265799257	0.0394384	+	T	C	0.2249	0.0443
rs1950557	LC_IGP77	IgG1 Bn	14	66201757	1	5.09E-10	2.25E-11	+	0.246475	0.2978	+	T	C	0.265799257	0.039407	+	T	C	0.2249	0.0445
rs1950557	LC_IGP86	IgG1 Bn/Fn total	14	66201757	1	9.53E-11	1.19E-11	+	0.256522	0.3021	+	T	C	0.265799257	0.0393584	+	T	C	0.2249	0.0445
rs1950557	LC_IGP84	IgG1 FBn/Bn total	14	66201757	1	1.40E-11	2.86E-15	-	-0.268132	-0.3544	+	T	C	0.265799257	0.0393924	+	T	C	0.2249	0.0449
rs1950557	LC_IGP62	IgG1 FG0n total/G0n	14	66201757	1	6.18E-14	2.73E-12	-	-0.295631	-0.3122	+	T	C	0.265799257	0.0390461	+	T	C	0.2249	0.0447
rs1950557	LC_IGP63	IgG1 FG1n total/G1n	14	66201757	1	8.39E-12	2.54E-13	-	-0.270337	-0.3265	+	T	C	0.265799257	0.0392785	+	T	C	0.2249	0.0446
rs1950557	LC_IGP64	IgG1 FG2n total/G2n	14	66201757	1	3.64E-11	8.46E-15	-	-0.262271	-0.3444	+	T	C	0.265799257	0.0393544	+	T	C	0.2249	0.0444
rs1950557	LC_IGP68	IgG1 FG2n/G2n	14	66201757	1	1.86E-08	2.78E-09	-	-0.223246	-0.2627	+	T	C	0.265799257	0.0394904	+	T	C	0.2249	0.0442
rs1950557	LC_IGP61	IgG1 Fn total	14	66201757	1	5.73E-13	1.93E-13	-	-0.284742	-0.3285	+	T	C	0.265799257	0.0391869	+	T	C	0.2249	0.0447
rs1950557	LC_IGP81	IgG1 Fn/Bn	14	66201757	1	3.28E-10	6.44E-11	-	-0.249038	-0.2897	+	T	C	0.265799257	0.0393748	+	T	C	0.2249	0.0443
rs1950557	LC_IGP21	IgG1 Fucosylation	14	66201757	1	2.62E-11	4.88E-13	-	-0.263235	-0.3224	+	T	C	0.265721332	0.0392103	+	T	C	0.2249	0.0446
rs1950557	LC_IGP11	IgG1_G0	14	66201757	1	1.21E-14	3.09E-14	+	0.301846	0.3372	+	T	C	0.265424557	0.0387643	+	T	C	0.2249	0.0444
rs1950557	LC_IGP_R1	IgG1_G0F/IgG1_G0	14	66201757	1	1.38E-13	1.13E-11	-	-0.289549	-0.3022	+	T	C	0.265424557	0.0388037	+	T	C	0.2249	0.0445

rs1950557	LC_IGP_R4	IgG1_G0FN/IgG1_G0N	14	66201757	1	1.62E-10	8.91E-14	-	-0.25211	-0.3342	+	T	C	0.265462339	0.0391758	+	T	C	0.2249	0.0448
rs1950557	LC_IGP52	IgG1_G0n	14	66201757	1	7.38E-15	2.94E-14	+	0.305992	0.3382	+	T	C	0.265799257	0.0389673	+	T	C	0.2249	0.0445
rs1950557	LC_IGP14	IgG1_G0N	14	66201757	1	2.69E-11	1.14E-11	+	0.262493	0.3002	+	T	C	0.265462339	0.039124	+	T	C	0.2249	0.0442
rs1950557	LC_IGP55	IgG1_G0Nn	14	66201757	1	1.56E-11	1.26E-11	+	0.266613	0.2998	+	T	C	0.265799257	0.0392588	+	T	C	0.2249	0.0443
rs1950557	LC_IGP12	IgG1_G1	14	66201757	1	5.90E-12	5.42E-13	+	0.270258	0.3219	+	T	C	0.265729994	0.0389795	+	T	C	0.2249	0.0446
rs1950557	LC_IGP_R2	IgG1_G1F/IgG1_G1	14	66201757	1	8.34E-12	1.23E-12	-	-0.268449	-0.3162	+	T	C	0.265729994	0.0390036	+	T	C	0.2249	0.0445
rs1950557	LC_IGP_R5	IgG1_G1FN/IgG1_G1N	14	66201757	1	2.74E-10	4.75E-16	-	-0.249486	-0.3624	+	T	C	0.265375154	0.0392731	+	T	C	0.2249	0.0446
rs1950557	LC_IGP53	IgG1_G1n	14	66201757	1	3.40E-11	1.22E-12	+	0.26218	0.3168	+	T	C	0.265799257	0.0392813	+	T	C	0.2249	0.0446
rs1950557	LC_IGP15	IgG1_G1N	14	66201757	1	8.89E-09	3.16E-11	+	0.227395	0.2945	+	T	C	0.265375154	0.0393347	+	T	C	0.2249	0.0444
rs1950557	LC_IGP56	IgG1_G1Nn	14	66201757	1	6.39E-09	8.84E-11	+	0.230387	0.2873	+	T	C	0.265799257	0.0394666	+	T	C	0.2249	0.0443
rs1950557	LC_IGP_R3	IgG1_G2F/IgG1_G2	14	66201757	1	6.89E-12	8.81E-15	-	-0.26966	-0.3435	+	T	C	0.265586797	0.0390205	+	T	C	0.2249	0.0443
rs1950557	LC_IGP97	IgG2_G0	14	66201757	1	6.59E-10	9.75E-09	+	0.242469	0.2538	+	T	C	0.265586797	0.0390271	+	T	C	0.2249	0.0442
rs1950557	LC_IGP_R41	IgG2_G0F/IgG2_G0	14	66201757	1	2.61E-08	2.40E-08	-	-0.218684	-0.2453	+	T	C	0.265586797	0.0390996	+	T	C	0.2249	0.044
rs1950557	LC_IGP138	IgG2_G0n	14	66201757	1	3.73E-10	1.04E-08	+	0.246391	0.2535	+	T	C	0.266009852	0.0390848	+	T	C	0.225	0.0443
rs8010876	LC_IGP78	IgG1 BG0n/G0n	14	66206976	1	1.88E-13	3.26E-10	-	-0.254224	-0.2344	+	G	A	0.484756506	0.0342603	+	G	A	0.5094	0.0373
rs8010876	LC_IGP79	IgG1 BG1n/G1n	14	66206976	1	2.12E-12	5.19E-12	-	-0.243531	-0.257	+	G	A	0.484756506	0.0343907	+	G	A	0.5094	0.0372
rs8010876	LC_IGP77	IgG1 Bn	14	66206976	1	1.21E-12	8.53E-12	-	-0.246241	-0.2556	+	G	A	0.484756506	0.0343859	+	G	A	0.5094	0.0374
rs8010876	LC_IGP86	IgG1 Bn/Fn total	14	66206976	1	2.14E-13	5.53E-12	-	-0.254225	-0.258	+	G	A	0.484756506	0.0343421	+	G	A	0.5094	0.0374
rs8010876	LC_IGP84	IgG1 FBn/Bn total	14	66206976	1	5.41E-14	6.74E-16	+	0.260945	0.3048	+	G	A	0.484756506	0.0343829	+	G	A	0.5094	0.0377
rs8010876	LC_IGP62	IgG1 FG0n total/G0n	14	66206976	1	4.05E-16	1.63E-12	+	0.280318	0.2652	+	G	A	0.484756506	0.0340914	+	G	A	0.5094	0.0376
rs8010876	LC_IGP63	IgG1 FG1n total/G1n	14	66206976	1	3.04E-16	3.47E-14	+	0.282322	0.2843	+	G	A	0.484756506	0.0341859	+	G	A	0.5094	0.0375
rs8010876	LC_IGP64	IgG1 FG2n total/G2n	14	66206976	1	3.39E-13	6.10E-15	+	0.252239	0.2911	+	G	A	0.484756506	0.0343683	+	G	A	0.5094	0.0373
rs8010876	LC_IGP68	IgG1 FG2n/G2n	14	66206976	1	2.71E-10	7.41E-09	+	0.219217	0.2147	+	G	A	0.484756506	0.0344971	+	G	A	0.5094	0.0371
rs8010876	LC_IGP61	IgG1 Fn total	14	66206976	1	1.38E-15	5.07E-14	+	0.275892	0.2828	+	G	A	0.484756506	0.0341934	+	G	A	0.5094	0.0376
rs8010876	LC_IGP81	IgG1 Fn/Bn	14	66206976	1	5.54E-13	4.17E-11	+	0.249761	0.2459	+	G	A	0.484756506	0.0343506	+	G	A	0.5094	0.0373
rs8010876	LC_IGP21	IgG1 Fucosylation	14	66206976	1	4.45E-14	9.38E-13	+	0.260155	0.2676	+	G	A	0.483897965	0.0341632	+	G	A	0.5094	0.0375
rs8010876	LC_IGP28	IgG1 G1S1/(G1+G1S1)	14	66206976	1	1.77E-09	1.19E-09	+	0.206678	0.219	+	G	A	0.484027829	0.0341488	+	G	A	0.5094	0.036
rs8010876	LC_IGP27	IgG1 GS1/(G0+G+GS1)	14	66206976	1	1.93E-08	1.96E-08	+	0.193334	0.2058	+	G	A	0.484027829	0.0342389	+	G	A	0.5094	0.0366
rs8010876	LC_IGP11	IgG1_G0	14	66206976	1	1.82E-17	8.08E-15	-	-0.290226	-0.2901	+	G	A	0.484352779	0.0337425	+	G	A	0.5094	0.0374
rs8010876	LC_IGP_R1	IgG1_G0F/IgG1_G0	14	66206976	1	2.67E-15	3.55E-12	+	0.270053	0.2603	+	G	A	0.484352779	0.0338291	+	G	A	0.5094	0.0374
rs8010876	LC_IGP_R4	IgG1_G0FN/IgG1_G0N	14	66206976	1	2.01E-13	1.96E-13	+	0.252671	0.2771	+	G	A	0.484926822	0.0340962	+	G	A	0.5094	0.0377
rs8010876	LC_IGP52	IgG1_G0n	14	66206976	1	1.62E-17	7.26E-15	-	-0.292942	-0.2911	+	G	A	0.484756506	0.0339998	+	G	A	0.5094	0.0374
rs8010876	LC_IGP14	IgG1_G0N	14	66206976	1	3.43E-16	6.95E-12	-	-0.279883	-0.255	+	G	A	0.484926822	0.0339569	+	G	A	0.5094	0.0372
rs8010876	LC_IGP55	IgG1_G0Nn	14	66206976	1	2.01E-16	7.48E-12	-	-0.283744	-0.2549	+	G	A	0.484756506	0.0341473	+	G	A	0.5094	0.0372
rs8010876	LC_IGP12	IgG1_G1	14	66206976	1	7.67E-15	2.92E-13	-	-0.266199	-0.2738	+	G	A	0.484352779	0.0339257	+	G	A	0.5094	0.0375
rs8010876	LC_IGP_R2	IgG1_G1F/IgG1_G1	14	66206976	1	3.78E-16	2.64E-13	+	0.278815	0.2736	+	G	A	0.484352779	0.0338781	+	G	A	0.5094	0.0374
rs8010876	LC_IGP_R5	IgG1_G1FN/IgG1_G1N	14	66206976	1	4.46E-13	6.45E-17	+	0.25006	0.3137	+	G	A	0.48578444	0.0342513	+	G	A	0.5094	0.0375
rs8010876	LC_IGP53	IgG1_G1n	14	66206976	1	5.25E-14	5.24E-13	-	-0.2602	-0.2706	+	G	A	0.484756506	0.0342668	+	G	A	0.5094	0.0375
rs8010876	LC_IGP15	IgG1_G1N	14	66206976	1	9.39E-11	6.44E-11	-	-0.223908	-0.2436	+	G	A	0.48578444	0.0343441	+	G	A	0.5094	0.0373
rs8010876	LC_IGP56	IgG1_G1Nn	14	66206976	1	4.12E-11	1.39E-10	-	-0.229002	-0.2389	+	G	A	0.484756506	0.034459	+	G	A	0.5094	0.0372
rs8010876	LC_IGP_R32	IgG1_G1S1/IgG1_G1	14	66206976	1	3.05E-09	1.81E-09	+	0.203679	0.2169	+	G	A	0.484027829	0.0341651	+	G	A	0.5094	0.0361
rs8010876	LC_IGP_R3	IgG1_G2F/IgG1_G2	14	66206976	1	1.46E-14	1.96E-14	+	0.26381	0.285	+	G	A	0.484037592	0.0339871	+	G	A	0.5094	0.0372
rs8010876	LC_IGP_R8	IgG1_G2FS1/IgG1_G2S1	14	66206976	1	3.99E-09	2.90E-12	+	0.202548	0.2594	+	G	A	0.483741906	0.0342336	+	G	A	0.5094	0.0372
rs8010876	LC_IGP_R26	IgG1_G2N/IgG1_G1N	14	66206976	1	4.05E-10	4.57E-09	+	0.215754	0.2134	+	G	A	0.485430247	0.0342948	+	G	A	0.5094	0.0364
rs8010876	LC_IGP148	IgG2 FG0n total/G0n	14	66206976	1	1.14E-08	1.43E-12	+	0.197019	0.263	+	G	A	0.482995998	0.0343371	+	G	A	0.5095	0.0371
rs8010876	LC_IGP97	IgG2_G0	14	66206976	1	4.82E-10	7.73E-13	-	-0.213646	-0.2665	+	G	A	0.484037592	0.0341124	+	G	A	0.5094	0.0372
rs8010876	LC_IGP138	IgG2_G0n	14	66206976	1	2.89E-10	5.47E-13	-	-0.217168	-0.2686	+	G	A	0.482995998	0.0342293	+	G	A	0.5095	0.0372
rs1054218	LC_IGP78	IgG1 BG0n/G0n	14	66209190	1	1.01E-12	1.30E-06	-	-0.251137	-0.187	+	G	C	0.581811338	0.0349466	+	G	C	0.6088	0.0386
rs1054218	LC_IGP79	IgG1 BG1n/G1n	14	66209190	1	6.76E-12	3.12E-07	-	-0.24247	-0.1973	+	G	C	0.581811338	0.0350683	+	G	C	0.6088	0.0386
rs1054218	LC_IGP77	IgG1 Bn	14	66209190	1	2.56E-12	2.04E-07	-	-0.247294	-0.2014	+	G	C	0.581811338	0.0350546	+	G	C	0.6088	0.0388
rs1054218	LC_IGP86	IgG1 Bn/Fn total	14	66209190	1	4.31E-13	1.68E-07	-	-0.255777	-0.2029	+	G	C	0.581811338	0.035009	+	G	C	0.6088	0.0388
rs1054218	LC_IGP84	IgG1 FBn/Bn total	14	66209190	1	8.53E-14	8.65E-10	+	0.263819	0.2396	+	G	C	0.581811338	0.0350454	+	G	C	0.6088	0.0391
rs1054218	LC_IGP62	IgG1 FG0n total/G0n	14	66209190	1	4.94E-17	1.60E-08	+	0.294268	0.2198	+	G	C	0.581811338	0.0346939	+	G	C	0.6088	0.0389
rs1054218	LC_IGP63	IgG1 FG1n total/G1n	14	66209190	1	3.84E-15	1.03E-08	+	0.27692	0.2223	+	G	C	0.581811338	0.0348889	+	G	C	0.6088	0.0388
rs1054218	LC_IGP64	IgG1 FG2n total/G2n	14	66209190	1	3.80E-13	7.54E-10	+	0.256494	0.2376	+	G	C	0.581811338	0.0350232	+	G	C	0.6088	0.0386
rs1054218	LC_IGP61	IgG1 Fn total	14	66209190	1	1.47E-15	3.88E-09	+	0.28084	0.229	+	G	C	0.581811338	0.034844	+	G	C	0.6088	0.0389
rs1054218	LC_IGP81	IgG1 Fn/Bn	14	66209190	1	1.43E-12	3.28E-07	+	0.249967	0.197	+	G	C	0.581811338	0.035023	+	G	C	0.6088	0.0386
rs1054218	LC_IGP21	IgG1 Fucosylation	14	66209190	1	8.87E-14	1.42E-08	+	0.261283	0.2203	+	G	C	0.581704686	0.0347345	+	G	C	0.6088	0.0388
rs1054218	LC_IGP28	IgG1 G1S1/(G1+G1S1)	14	66209190	1	8.65E-09	2.94E-07	+	0.201234	0.1913	+	G	C	0.581668502	0.0347825	+	G	C	0.6088	0.0373
rs1054218	LC_IGP11	IgG1_G0	14	66209190	1	1.22E-18	1.00E-10	-	-0.3057	-0.2502	+	G	C	0.582179597	0.0342724	+	G	C	0.6088	0.0387
rs1054218	LC_IGP_R1	IgG1_G0F/IgG1_G0	14	66209190	1	1.14E-16	2.39E-08	+	0.28779	0.2164	+	G	C	0.582179597	0.034351	+	G	C	0.6088	0.0388
rs1054218	LC_IGP_R4	IgG1_G0FN/IgG1_G0N	14	66209190	1	2.75E-12	6.45E-09	+	0.24498	0.2266	+	G	C	0.582687079	0.0347791	+	G	C	0.6088	0.039
rs1054218	LC_IGP52	IgG1_G0n	14	66209190	1	8.55E-19	1.09E-10	-	-0.309954	-0.2502	+	G	C	0.581811338	0.0345828	+	G	C	0.6088	0.0388
rs1054218	LC_IGP14	IgG1_G0N	14	66209190	1	1.96E-14	6.31E-08	-	-0.267763	-0.2084	+	G	C	0.582687079	0.0346671	+	G	C	0.6088	0.0385
rs1054218	LC_IGP55	IgG1_G0Nn	14	66209190	1	3.89E-15	8.14E-08	-	-0.276622	-0.2069	+	G	C	0.581811338	0.0348586	+	G	C	0.6088	0.0386
rs1054218	LC_IGP12	IgG1_G1	14	66209190	1	6.43E-15	1.71E-08	-	-0.271695	-0.219	+	G	C	0.58187416	0.0345253	+	G	C	0.6088	0.0388
rs1054218	LC_IGP_R2	IgG1_G1F/IgG1_G1	14	66209190	1	1.66E-15	2.15E-08	+	0.277581	0.217	+	G	C	0.58187416	0.0345113	+	G	C	0.6088	0.0388
rs1054218	LC_IGP_R5	IgG1_G1FN/IgG1_G1N	14	66209190	1	2.81E-12	5.04E-10	+	0.245793	0.2416	+	G	C	0.58304305	0.0349095	+	G	C	0.6088	0.0389
rs1054218	LC_IGP53	IgG1_G1n	14	66209190	1	1.40E-13	2.97E-08	-	-0.260679	-0.2152	+	G	C	0.581811338	0.0349382	+	G	C	0.6088	0.0388
rs1054218	LC_IGP15	IgG1_G1N	14	66209190	1	2.66E-10	8.45E-07	-	-0.222426	-0.1901	+	G	C	0.58304305	0.0349871	+	G	C	0.6088	0.0386
rs1054218	LC_IGP56	IgG1_G1Nn	14	66209190	1	6.11E-11	1.54E-06	-	-0.231298	-0.1853	+	G	C	0.581811338	0.0351217	+	G	C	0.6088	0.0386
rs1054218	LC_IGP_R32	IgG1_G1S1/IgG1_G1	14	66209190	1	1.66E-08	4.42E-07	+	0.197431	0.1886	+	G	C	0.581668502	0.0348011	+	G	C	0.6088	0.0374
rs1054218	LC_IGP_R3	IgG1_G2F/IgG1_G2	14	66209190	1	2.86E-14	1.09E-09	+	0.265373	0.2349	+	G	C	0.581924205	0.0345841	+	G	C	0.6088	0.0385
rs1054218	LC_IGP_R8	IgG1_G2FS1/IgG1_G2S1	14	66209190	1	3.20E-09	4.42E-09	+	0.207231	0.2257	+	G	C	0.581568723	0.0348066	+	G	C	0.6088	0.0385
rs8003811	LC_IGP78	IgG1 BG0n/G0n	14	66211250	1	8.85E-13	1.20E-06	-	-0.251911	-0.1873	+	T	C	0.582425341	0.0349609	+	T	C	0.6098	0.0386
rs8003811	LC_IGP79	IgG1 BG1n/G1n	14	66211250	1	6.01E-12	3.00E-07	-	-0.24317	-0.1974	+	T	C	0.582425341	0.0350831	+	T	C	0.6098	0.0385
rs8003811	LC_IGP77	IgG1 Bn	14	66211250	1	2.14E-12	1.91E-07	-	-0.248288	-0.2016	+	T	C	0.582425341	0.0350681	+	T	C	0.6098	0.0387
rs8003811	LC_IGP86	IgG1 Bn/Fn total	14	66211250	1	3.60E-13	1.59E-07	-	-0.256753	-0.203	+	T	C	0.582425341	0.0350224	+	T	C	0.6098	0.0387
rs8003811	LC_IGP84	IgG1 FBn/Bn total	14	66211250	1	6.82E-14	7.77E-10	+	0.264973	0.24	+	T	C	0.582425341	0.0350579	+	T	C	0.6098	0.039
rs8003811	LC_IGP62	IgG1 FG0n total/G0n	14	66211250	1	4.33E-17	1.47E-08	+	0.294945	0.22	+	T	C	0.582425341	0.0347082	+	T	C	0.6098	0.0388
rs8003811	LC_IGP63	IgG1 FG1n total/G1n	14	66211250	1	2.96E-15	9.80E-09	+	0.278181	0.2224	+	T	C	0.582425341	0.0349006	+	T	C	0.6098	0.0388
rs8003811	LC_IGP64	IgG1 FG2n total/G2n	14	66211250	1	2.20E-13	7.05E-10	+	0.25917	0.2377	+	T	C	0.582425341	0.0350289	+	T	C	0.6098	0.0386
rs8003811	LC_IGP61	IgG1 Fn total	14	66211250	1	1.17E-15	3.64E-09	+	0.281958	0.229	+	T	C	0.582425341	0.0348563	+	T	C	0.6098	0.0388
rs8003811	LC_IGP81	IgG1 Fn/Bn	14	66211250	1	1.20E-12	3.03E-07	+	0.25093	0.1974	+	T	C	0.582425341	0.0350366	+	T	C	0.6098	0.0385
rs8003811	LC_IGP21	IgG1 Fucosylation	14	66211250	1	7.56E-14	1.42E-08	+	0.262141	0.2199	+	T	C	0.582312885	0.0347483	+	T	C	0.6098	0.0388
rs8003811	LC_IGP28	IgG1 G1S1/(G1+G1S1)	14	66211250	1	9.33E-09	2.76E-07	+	0.20089	0.1915	+	T	C	0.582271865	0.0348012	+	T	C	0.6098	0.0373
rs8003811	LC_IGP11	IgG1_G0	14	66211250	1	7.36E-19	8.58E-11	-	-0.30777	-0.2507	+	T	C	0.582782224	0.0342788	+	T	C	0.6098	0.0386
rs8003811	LC_IGP_R1	IgG1_G0F/IgG1_G0	14	66211250	1	1.02E-16	2.23E-08	+	0.28838	0.2165	+	T	C	0.582782224	0.0343657	+	T	C	0.6098	0.0387
rs8003811	LC_IGP_R4	IgG1_G0FN/IgG1_G0N	14	66211250	1	2.19E-12	5.57E-09	+	0.246191	0.2272	+	T	C	0.583291182	0.0347918	+	T	C	0.6098	0.039
rs8003811	LC_IGP52	IgG1_G0n	14	66211250	1	5.19E-19	9.43E-11	-	-0.312009	-0.2506	+	T	C	0.582425341	0.0345892	+	T	C	0.6098	0.0387
rs8003811	LC_IGP14	IgG1_G0N	14	66211250	1	1.28E-14	5.51E-08	-	-0.269757	-0.209	+	T	C	0.583291182	0.0346755	+	T	C	0.6098	0.0385
rs8003811	LC_IGP55	IgG1_G0Nn	14	66211250	1	2.54E-15	7.12E-08	-	-0.278597	-0.2075	+	T	C	0.582425341	0.0348667	+	T	C	0.6098	0.0385
rs8003811	LC_IGP12	IgG1_G1	14	66211250	1	5.56E-15	1.60E-08	-	-0.272457	-0.2192	+	T	C	0.582476787	0.0345393	+	T	C	0.6098	0.0388
rs8003811	LC_IGP_R2	IgG1_G1F/IgG1_G1	14	66211250	1	1.19E-15	2.04E-08	+	0.279132	0.217	+	T	C	0.582476787	0.0345213	+	T	C	0.6098	0.0387
rs8003811	LC_IGP_R5	IgG1_G1FN/IgG1_G1N	14	66211250	1	2.37E-12	4.64E-10	+	0.246735	0.2418	+	T	C	0.583649754	0.0349235	+	T	C	0.6098	0.0388
rs8003811	LC_IGP53	IgG1_G1n	14	66211250	1	1.28E-13	2.82E-08	-	-0.261207	-0.2152	+	T	C	0.582425341	0.0349536	+	T	C	0.6098	0.0388
rs8003811	LC_IGP15	IgG1_G1N	14	66211250	1	2.59E-10	8.14E-07	-	-0.222674	-0.1902	+	T	C	0.583649754	0.0350042	+	T	C	0.6098	0.0386
rs8003811	LC_IGP56	IgG1_G1Nn	14	66211250	1	6.08E-11	1.50E-06	-	-0.231443	-0.1853	+	T	C	0.582425341	0.0351388	+	T	C	0.6098	0.0385
rs8003811	LC_IGP_R32	IgG1_G1S1/IgG1_G1	14	66211250	1	1.77E-08	4.14E-07	+	0.197143	0.1888	+	T	C	0.582271865	0.0348196	+	T	C	0.6098	0.0373
rs8003811	LC_IGP_R3	IgG1_G2F/IgG1_G2	14	66211250	1	1.64E-14	1.04E-09	+	0.267976	0.2349	+	T	C	0.5825272	0.0345894	+	T	C	0.6098	0.0385
rs8003811	LC_IGP_R8	IgG1_G2FS1/IgG1_G2S1	14	66211250	1	2.33E-09	4.12E-09	+	0.209124	0.2258	+	T	C	0.58217135	0.0348171	+	T	C	0.6098	0.0384
rs761830	LC_IGP78	IgG1 BG0n/G0n	14	66212986	1	8.75E-13	1.01E-06	-	-0.251983	-0.1889	+	A	G	0.582422243	0.0349636	+	A	G	0.6104	0.0386
rs761830	LC_IGP79	IgG1 BG1n/G1n	14	66212986	1	6.01E-12	2.47E-07	-	-0.243191	-0.199	+	A	G	0.582422243	0.0350859	+	A	G	0.6104	0.0386
rs761830	LC_IGP77	IgG1 Bn	14	66212986	1	2.13E-12	1.60E-07	-	-0.248334	-0.2032	+	A	G	0.582422243	0.0350709	+	A	G	0.6104	0.0388
rs761830	LC_IGP86	IgG1 Bn/Fn total	14	66212986	1	3.58E-13	1.33E-07	-	-0.256799	-0.2046	+	A	G	0.582422243	0.0350252	+	A	G	0.6104	0.0388
rs761830	LC_IGP84	IgG1 FBn/Bn total	14	66212986	1	6.78E-14	6.16E-10	+	0.265019	0.2418	+	A	G	0.582422243	0.0350607	+	A	G	0.6104	0.0391
rs761830	LC_IGP62	IgG1 FG0n total/G0n	14	66212986	1	4.29E-17	1.23E-08	+	0.29501	0.2215	+	A	G	0.582422243	0.0347108	+	A	G	0.6104	0.0389
rs761830	LC_IGP63	IgG1 FG1n total/G1n	14	66212986	1	2.96E-15	8.08E-09	+	0.278211	0.224	+	A	G	0.582422243	0.0349034	+	A	G	0.6104	0.0388
rs761830	LC_IGP64	IgG1 FG2n total/G2n	14	66212986	1	2.21E-13	5.82E-10	+	0.259176	0.2392	+	A	G	0.582422243	0.0350318	+	A	G	0.6104	0.0386
rs761830	LC_IGP61	IgG1 Fn total	14	66212986	1	1.17E-15	3.00E-09	+	0.281999	0.2306	+	A	G	0.582422243	0.0348591	+	A	G	0.6104	0.0389
rs761830	LC_IGP81	IgG1 Fn/Bn	14	66212986	1	1.20E-12	2.56E-07	+	0.250976	0.1989	+	A	G	0.582422243	0.0350393	+	A	G	0.6104	0.0386
rs761830	LC_IGP21	IgG1 Fucosylation	14	66212986	1	7.50E-14	1.22E-08	+	0.262197	0.2213	+	A	G	0.582307337	0.0347512	+	A	G	0.6104	0.0388
rs761830	LC_IGP28	IgG1 G1S1/(G1+G1S1)	14	66212986	1	9.37E-09	2.24E-07	+	0.200884	0.1932	+	A	G	0.582266361	0.0348043	+	A	G	0.6104	0.0373
rs761830	LC_IGP11	IgG1_G0	14	66212986	1	7.27E-19	7.68E-11	-	-0.307844	-0.2518	+	A	G	0.582776726	0.0342815	+	A	G	0.6104	0.0387
rs761830	LC_IGP_R1	IgG1_G0F/IgG1_G0	14	66212986	1	1.01E-16	1.90E-08	+	0.288458	0.218	+	A	G	0.582776726	0.0343685	+	A	G	0.6104	0.0388
rs761830	LC_IGP_R4	IgG1_G0FN/IgG1_G0N	14	66212986	1	2.16E-12	4.49E-09	+	0.246279	0.2289	+	A	G	0.583285671	0.0347946	+	A	G	0.6104	0.039
rs761830	LC_IGP52	IgG1_G0n	14	66212986	1	5.13E-19	8.41E-11	-	-0.312075	-0.2517	+	A	G	0.582422243	0.0345918	+	A	G	0.6104	0.0388
rs761830	LC_IGP14	IgG1_G0N	14	66212986	1	1.26E-14	4.84E-08	-	-0.269848	-0.2102	+	A	G	0.583285671	0.0346783	+	A	G	0.6104	0.0385
rs761830	LC_IGP55	IgG1_G0Nn	14	66212986	1	2.51E-15	6.26E-08	-	-0.278674	-0.2087	+	A	G	0.582422243	0.0348692	+	A	G	0.6104	0.0386

rs761830	LC_IGP12	IgG1_G1	14	66212986	1	5.53E-15	1.32E-08	-	-0.272504	-0.2208	+	A	G	0.582471289	0.0345422	+	A	G	0.6104	0.0388
rs761830	LC_IGP_R2	IgG1_G1F/IgG1_G1	14	66212986	1	1.19E-15	1.72E-08	+	0.279175	0.2185	+	A	G	0.582471289	0.0345243	+	A	G	0.6104	0.0388
rs761830	LC_IGP_R5	IgG1_G1FN/IgG1_G1N	14	66212986	1	2.36E-12	3.64E-10	+	0.24678	0.2436	+	A	G	0.583644219	0.0349265	+	A	G	0.6104	0.0389
rs761830	LC_IGP53	IgG1_G1n	14	66212986	1	1.28E-13	2.33E-08	-	-0.261241	-0.2168	+	A	G	0.582422243	0.0349564	+	A	G	0.6104	0.0388
rs761830	LC_IGP15	IgG1_G1N	14	66212986	1	2.58E-10	6.68E-07	-	-0.222722	-0.1919	+	A	G	0.583644219	0.0350072	+	A	G	0.6104	0.0386
rs761830	LC_IGP56	IgG1_G1Nn	14	66212986	1	6.07E-11	1.23E-06	-	-0.231468	-0.187	+	A	G	0.582422243	0.0351417	+	A	G	0.6104	0.0386
rs761830	LC_IGP_R32	IgG1_G1S1/IgG1_G1	14	66212986	1	1.77E-08	3.37E-07	+	0.197136	0.1906	+	A	G	0.582266361	0.0348228	+	A	G	0.6104	0.0374
rs761830	LC_IGP_R3	IgG1_G2F/IgG1_G2	14	66212986	1	1.64E-14	8.53E-10	+	0.267992	0.2364	+	A	G	0.582521699	0.0345925	+	A	G	0.6104	0.0385
rs761830	LC_IGP_R8	IgG1_G2FS1/IgG1_G2S1	14	66212986	1	2.33E-09	3.50E-09	+	0.209153	0.2271	+	A	G	0.582165852	0.0348201	+	A	G	0.6104	0.0384
rs60273099	LC_IGP78	IgG1 BG0n/G0n	14	66214850	1	2.03E-10	2.99E-10	+	0.251224	0.2797	+	T	C	0.266152726	0.0392508	+	T	C	0.2248	0.0444
rs60273099	LC_IGP79	IgG1 BG1n/G1n	14	66214850	1	1.65E-09	1.21E-11	+	0.238891	0.3007	+	T	C	0.266152726	0.039392	+	T	C	0.2248	0.0444
rs60273099	LC_IGP77	IgG1 Bn	14	66214850	1	3.27E-10	2.27E-11	+	0.248958	0.298	+	T	C	0.266152726	0.0393614	+	T	C	0.2248	0.0446
rs60273099	LC_IGP86	IgG1 Bn/Fn total	14	66214850	1	5.82E-11	1.20E-11	+	0.259185	0.3023	+	T	C	0.266152726	0.0393116	+	T	C	0.2248	0.0446
rs60273099	LC_IGP84	IgG1 FBn/Bn total	14	66214850	1	8.37E-12	2.89E-15	-	-0.270807	-0.3546	+	T	C	0.266152726	0.039345	+	T	C	0.2248	0.0449
rs60273099	LC_IGP62	IgG1 FG0n total/G0n	14	66214850	1	3.61E-14	2.67E-12	-	-0.298082	-0.3126	+	T	C	0.266152726	0.0389986	+	T	C	0.2248	0.0447
rs60273099	LC_IGP63	IgG1 FG1n total/G1n	14	66214850	1	4.28E-12	2.59E-13	-	-0.273832	-0.3266	+	T	C	0.266152726	0.0392275	+	T	C	0.2248	0.0446
rs60273099	LC_IGP64	IgG1 FG2n total/G2n	14	66214850	1	2.03E-11	8.81E-15	-	-0.265393	-0.3444	+	T	C	0.266152726	0.0393055	+	T	C	0.2248	0.0444
rs60273099	LC_IGP68	IgG1 FG2n/G2n	14	66214850	1	1.24E-08	2.85E-09	-	-0.22578	-0.2627	+	T	C	0.266152726	0.0394456	+	T	C	0.2248	0.0442
rs60273099	LC_IGP61	IgG1 Fn total	14	66214850	1	3.11E-13	1.96E-13	-	-0.287711	-0.3286	+	T	C	0.266152726	0.0391375	+	T	C	0.2248	0.0447
rs60273099	LC_IGP81	IgG1 Fn/Bn	14	66214850	1	2.05E-10	6.49E-11	-	-0.251649	-0.2899	+	T	C	0.266152726	0.0393286	+	T	C	0.2248	0.0444
rs60273099	LC_IGP21	IgG1 Fucosylation	14	66214850	1	1.42E-11	4.92E-13	-	-0.266509	-0.3226	+	T	C	0.266089704	0.0391626	+	T	C	0.2248	0.0446
rs60273099	LC_IGP11	IgG1_G0	14	66214850	1	6.86E-15	3.03E-14	+	0.304365	0.3376	+	T	C	0.265789554	0.0387186	+	T	C	0.2248	0.0444
rs60273099	LC_IGP_R1	IgG1_G0F/IgG1_G0	14	66214850	1	7.97E-14	1.11E-11	-	-0.292093	-0.3026	+	T	C	0.265789554	0.0387584	+	T	C	0.2248	0.0446
rs60273099	LC_IGP_R4	IgG1_G0FN/IgG1_G0N	14	66214850	1	1.13E-10	8.64E-14	-	-0.254014	-0.3346	+	T	C	0.26582823	0.0391345	+	T	C	0.2248	0.0448
rs60273099	LC_IGP52	IgG1_G0n	14	66214850	1	4.06E-15	2.89E-14	+	0.308628	0.3385	+	T	C	0.266152726	0.0389184	+	T	C	0.2248	0.0445
rs60273099	LC_IGP14	IgG1_G0N	14	66214850	1	1.76E-11	1.11E-11	+	0.264691	0.3006	+	T	C	0.26582823	0.0390811	+	T	C	0.2248	0.0443
rs60273099	LC_IGP55	IgG1_G0Nn	14	66214850	1	1.01E-11	1.23E-11	+	0.268829	0.3003	+	T	C	0.266152726	0.0392135	+	T	C	0.2248	0.0443
rs60273099	LC_IGP12	IgG1_G1	14	66214850	1	3.35E-12	5.51E-13	+	0.273136	0.3221	+	T	C	0.266094991	0.0389337	+	T	C	0.2248	0.0447
rs60273099	LC_IGP_R2	IgG1_G1F/IgG1_G1	14	66214850	1	4.35E-12	1.26E-12	-	-0.271815	-0.3163	+	T	C	0.266094991	0.0389558	+	T	C	0.2248	0.0446
rs60273099	LC_IGP_R5	IgG1_G1FN/IgG1_G1N	14	66214850	1	1.62E-10	4.88E-16	-	-0.252441	-0.3625	+	T	C	0.26574262	0.0392276	+	T	C	0.2248	0.0447
rs60273099	LC_IGP53	IgG1_G1n	14	66214850	1	1.96E-11	1.24E-12	+	0.265101	0.3168	+	T	C	0.266152726	0.0392332	+	T	C	0.2248	0.0446
rs60273099	LC_IGP15	IgG1_G1N	14	66214850	1	5.91E-09	3.21E-11	+	0.229884	0.2946	+	T	C	0.26574262	0.039292	+	T	C	0.2248	0.0444
rs60273099	LC_IGP56	IgG1_G1Nn	14	66214850	1	4.29E-09	9.00E-11	+	0.232786	0.2874	+	T	C	0.266152726	0.039422	+	T	C	0.2248	0.0443
rs60273099	LC_IGP_R3	IgG1_G2F/IgG1_G2	14	66214850	1	3.80E-12	9.17E-15	-	-0.272704	-0.3435	+	T	C	0.265952017	0.0389739	+	T	C	0.2248	0.0443
rs60273099	LC_IGP97	IgG2_G0	14	66214850	1	4.39E-10	9.74E-09	+	0.244746	0.254	+	T	C	0.265950183	0.0389851	+	T	C	0.2248	0.0443
rs60273099	LC_IGP_R41	IgG2_G0F/IgG2_G0	14	66214850	1	1.85E-08	2.41E-08	-	-0.220829	-0.2455	+	T	C	0.265950183	0.039059	+	T	C	0.2248	0.044
rs60273099	LC_IGP138	IgG2_G0n	14	66214850	1	2.39E-10	1.04E-08	+	0.24885	0.2537	+	T	C	0.266377771	0.0390415	+	T	C	0.225	0.0443
rs61298903	LC_IGP78	IgG1 BG0n/G0n	14	66218595	1	1.97E-10	2.30E-10	+	0.25148	0.2824	+	T	C	0.266181537	0.0392641	+	T	C	0.2244	0.0446
rs61298903	LC_IGP79	IgG1 BG1n/G1n	14	66218595	1	1.67E-09	9.73E-12	+	0.238887	0.303	+	T	C	0.266181537	0.0394063	+	T	C	0.2244	0.0445
rs61298903	LC_IGP77	IgG1 Bn	14	66218595	1	3.26E-10	1.75E-11	+	0.249064	0.3006	+	T	C	0.266181537	0.0393753	+	T	C	0.2244	0.0447
rs61298903	LC_IGP86	IgG1 Bn/Fn total	14	66218595	1	5.80E-11	9.56E-12	+	0.259296	0.3047	+	T	C	0.266181537	0.0393255	+	T	C	0.2244	0.0447
rs61298903	LC_IGP84	IgG1 FBn/Bn total	14	66218595	1	8.29E-12	2.44E-15	-	-0.270957	-0.3568	+	T	C	0.266181537	0.0393588	+	T	C	0.2244	0.0451
rs61298903	LC_IGP62	IgG1 FG0n total/G0n	14	66218595	1	3.51E-14	2.40E-12	-	-0.298322	-0.3143	+	T	C	0.266181537	0.0390118	+	T	C	0.2244	0.0448
rs61298903	LC_IGP63	IgG1 FG1n total/G1n	14	66218595	1	4.31E-12	2.28E-13	-	-0.273886	-0.3284	+	T	C	0.266181537	0.0392416	+	T	C	0.2244	0.0448
rs61298903	LC_IGP64	IgG1 FG2n total/G2n	14	66218595	1	2.09E-11	8.10E-15	-	-0.265319	-0.3459	+	T	C	0.266181537	0.0393201	+	T	C	0.2244	0.0445
rs61298903	LC_IGP68	IgG1 FG2n/G2n	14	66218595	1	1.27E-08	2.49E-09	-	-0.225674	-0.2645	+	T	C	0.266181537	0.0394603	+	T	C	0.2244	0.0444
rs61298903	LC_IGP61	IgG1 Fn total	14	66218595	1	3.11E-13	1.70E-13	-	-0.287809	-0.3306	+	T	C	0.266181537	0.0391514	+	T	C	0.2244	0.0448
rs61298903	LC_IGP81	IgG1 Fn/Bn	14	66218595	1	2.05E-10	4.98E-11	-	-0.251749	-0.2925	+	T	C	0.266181537	0.0393425	+	T	C	0.2244	0.0445
rs61298903	LC_IGP21	IgG1 Fucosylation	14	66218595	1	1.39E-11	3.71E-13	-	-0.26672	-0.3253	+	T	C	0.266129162	0.039177	+	T	C	0.2244	0.0448
rs61298903	LC_IGP11	IgG1_G0	14	66218595	1	6.59E-15	3.12E-14	+	0.304676	0.3386	+	T	C	0.26582865	0.0387322	+	T	C	0.2244	0.0446
rs61298903	LC_IGP_R1	IgG1_G0F/IgG1_G0	14	66218595	1	7.66E-14	1.01E-11	-	-0.2924	-0.3043	+	T	C	0.26582865	0.038772	+	T	C	0.2244	0.0447
rs61298903	LC_IGP_R4	IgG1_G0FN/IgG1_G0N	14	66218595	1	1.08E-10	7.33E-14	-	-0.254389	-0.3367	+	T	C	0.265867422	0.0391481	+	T	C	0.2244	0.045
rs61298903	LC_IGP52	IgG1_G0n	14	66218595	1	3.92E-15	2.94E-14	+	0.308901	0.3397	+	T	C	0.266181537	0.0389314	+	T	C	0.2244	0.0447
rs61298903	LC_IGP14	IgG1_G0N	14	66218595	1	1.68E-11	8.73E-12	+	0.265058	0.3031	+	T	C	0.265867422	0.0390947	+	T	C	0.2244	0.0444
rs61298903	LC_IGP55	IgG1_G0Nn	14	66218595	1	9.74E-12	9.53E-12	+	0.26912	0.3028	+	T	C	0.266181537	0.0392266	+	T	C	0.2244	0.0444
rs61298903	LC_IGP12	IgG1_G1	14	66218595	1	3.32E-12	5.25E-13	+	0.273277	0.3234	+	T	C	0.266134087	0.0389481	+	T	C	0.2244	0.0448
rs61298903	LC_IGP_R2	IgG1_G1F/IgG1_G1	14	66218595	1	4.34E-12	1.14E-12	-	-0.271933	-0.318	+	T	C	0.266134087	0.0389703	+	T	C	0.2244	0.0447
rs61298903	LC_IGP_R5	IgG1_G1FN/IgG1_G1N	14	66218595	1	1.60E-10	4.08E-16	-	-0.252593	-0.3647	+	T	C	0.26578198	0.0392422	+	T	C	0.2244	0.0448
rs61298903	LC_IGP53	IgG1_G1n	14	66218595	1	1.96E-11	1.17E-12	+	0.265187	0.3183	+	T	C	0.266181537	0.0392471	+	T	C	0.2244	0.0448
rs61298903	LC_IGP15	IgG1_G1N	14	66218595	1	5.86E-09	2.64E-11	+	0.230025	0.2968	+	T	C	0.26578198	0.0393066	+	T	C	0.2244	0.0445
rs61298903	LC_IGP56	IgG1_G1Nn	14	66218595	1	4.34E-09	7.38E-11	+	0.232801	0.2896	+	T	C	0.266181537	0.0394362	+	T	C	0.2244	0.0445
rs61298903	LC_IGP_R3	IgG1_G2F/IgG1_G2	14	66218595	1	3.89E-12	8.42E-15	-	-0.272682	-0.345	+	T	C	0.265991137	0.0389891	+	T	C	0.2244	0.0445
rs61298903	LC_IGP97	IgG2_G0	14	66218595	1	4.20E-10	1.06E-08	+	0.245103	0.2542	+	T	C	0.265988081	0.0389988	+	T	C	0.2244	0.0444
rs61298903	LC_IGP_R41	IgG2_G0F/IgG2_G0	14	66218595	1	1.80E-08	2.28E-08	-	-0.221078	-0.2468	+	T	C	0.265988081	0.0390732	+	T	C	0.2244	0.0442
rs61298903	LC_IGP138	IgG2_G0n	14	66218595	1	2.29E-10	1.11E-08	+	0.249201	0.254	+	T	C	0.26641718	0.0390552	+	T	C	0.2245	0.0445
rs10483785	LC_IGP78	IgG1 BG0n/G0n	14	66219517	1	1.10E-13	1.60E-10	-	-0.256826	-0.2404	+	A	G	0.485478934	0.0342711	+	A	G	0.5094	0.0376
rs10483785	LC_IGP79	IgG1 BG1n/G1n	14	66219517	1	1.33E-12	2.72E-12	-	-0.245921	-0.2624	+	A	G	0.485478934	0.034403	+	A	G	0.5094	0.0375
rs10483785	LC_IGP77	IgG1 Bn	14	66219517	1	7.13E-13	4.12E-12	-	-0.24889	-0.2615	+	A	G	0.485478934	0.0343969	+	A	G	0.5094	0.0377
rs10483785	LC_IGP86	IgG1 Bn/Fn total	14	66219517	1	1.22E-13	2.82E-12	-	-0.256934	-0.2636	+	A	G	0.485478934	0.0343525	+	A	G	0.5094	0.0377
rs10483785	LC_IGP84	IgG1 FBn/Bn total	14	66219517	1	3.93E-14	5.13E-16	+	0.262531	0.3084	+	A	G	0.485478934	0.0343984	+	A	G	0.5094	0.038
rs10483785	LC_IGP62	IgG1 FG0n total/G0n	14	66219517	1	2.35E-16	1.22E-12	+	0.282723	0.269	+	A	G	0.485478934	0.0341019	+	A	G	0.5094	0.0379
rs10483785	LC_IGP63	IgG1 FG1n total/G1n	14	66219517	1	1.70E-16	2.56E-14	+	0.284862	0.288	+	A	G	0.485478934	0.0341957	+	A	G	0.5094	0.0378
rs10483785	LC_IGP64	IgG1 FG2n total/G2n	14	66219517	1	2.34E-13	4.60E-15	+	0.254102	0.2943	+	A	G	0.485478934	0.0343827	+	A	G	0.5094	0.0376
rs10483785	LC_IGP68	IgG1 FG2n/G2n	14	66219517	1	1.47E-10	3.79E-09	+	0.222594	0.2202	+	A	G	0.485478934	0.0345064	+	A	G	0.5094	0.0374
rs10483785	LC_IGP61	IgG1 Fn total	14	66219517	1	7.26E-16	3.64E-14	+	0.278728	0.2866	+	A	G	0.485478934	0.0342021	+	A	G	0.5094	0.0378
rs10483785	LC_IGP81	IgG1 Fn/Bn	14	66219517	1	3.08E-13	1.83E-11	+	0.252629	0.2523	+	A	G	0.485478934	0.0343605	+	A	G	0.5094	0.0376
rs10483785	LC_IGP21	IgG1 Fucosylation	14	66219517	1	2.02E-14	5.66E-13	+	0.26389	0.2723	+	A	G	0.484477189	0.0341829	+	A	G	0.5094	0.0378
rs10483785	LC_IGP28	IgG1 G1S1/(G1+G1S1)	14	66219517	1	2.21E-09	8.10E-10	+	0.205658	0.2228	+	A	G	0.484606422	0.0341891	+	A	G	0.5094	0.0363
rs10483785	LC_IGP27	IgG1 GS1/(G0+G+GS1)	14	66219517	1	4.97E-08	1.42E-08	+	0.187861	0.2093	+	A	G	0.484606422	0.034294	+	A	G	0.5094	0.0369
rs10483785	LC_IGP11	IgG1_G0	14	66219517	1	1.52E-17	8.25E-15	-	-0.291223	-0.2923	+	A	G	0.484930666	0.0337741	+	A	G	0.5094	0.0377
rs10483785	LC_IGP_R1	IgG1_G0F/IgG1_G0	14	66219517	1	1.23E-15	2.65E-12	+	0.273555	0.264	+	A	G	0.484930666	0.0338486	+	A	G	0.5094	0.0378
rs10483785	LC_IGP_R4	IgG1_G0FN/IgG1_G0N	14	66219517	1	1.17E-13	1.34E-13	+	0.25539	0.2812	+	A	G	0.485505511	0.034121	+	A	G	0.5094	0.038
rs10483785	LC_IGP52	IgG1_G0n	14	66219517	1	1.42E-17	7.34E-15	-	-0.293665	-0.2935	+	A	G	0.485478934	0.0340188	+	A	G	0.5094	0.0377
rs10483785	LC_IGP14	IgG1_G0N	14	66219517	1	2.32E-16	3.46E-12	-	-0.281768	-0.2607	+	A	G	0.485505511	0.0339846	+	A	G	0.5094	0.0375
rs10483785	LC_IGP55	IgG1_G0Nn	14	66219517	1	1.57E-16	3.71E-12	-	-0.284926	-0.2606	+	A	G	0.485478934	0.0341641	+	A	G	0.5094	0.0375
rs10483785	LC_IGP12	IgG1_G1	14	66219517	1	3.97E-15	2.48E-13	-	-0.26927	-0.2768	+	A	G	0.484930666	0.0339477	+	A	G	0.5094	0.0378
rs10483785	LC_IGP_R2	IgG1_G1F/IgG1_G1	14	66219517	1	1.73E-16	1.93E-13	+	0.282264	0.2774	+	A	G	0.484930666	0.0338975	+	A	G	0.5094	0.0377
rs10483785	LC_IGP_R5	IgG1_G1FN/IgG1_G1N	14	66219517	1	2.68E-13	4.95E-17	+	0.25267	0.3172	+	A	G	0.486365621	0.034277	+	A	G	0.5094	0.0378
rs10483785	LC_IGP53	IgG1_G1n	14	66219517	1	2.82E-14	4.46E-13	-	-0.263109	-0.2736	+	A	G	0.485478934	0.0342759	+	A	G	0.5094	0.0378
rs10483785	LC_IGP15	IgG1_G1N	14	66219517	1	4.72E-11	3.46E-11	-	-0.227667	-0.2489	+	A	G	0.486365621	0.0343663	+	A	G	0.5094	0.0376
rs10483785	LC_IGP56	IgG1_G1Nn	14	66219517	1	2.37E-11	7.62E-11	-	-0.231934	-0.2441	+	A	G	0.485478934	0.0344697	+	A	G	0.5094	0.0375
rs10483785	LC_IGP_R32	IgG1_G1S1/IgG1_G1	14	66219517	1	3.72E-09	1.27E-09	+	0.202792	0.2205	+	A	G	0.484606422	0.0342048	+	A	G	0.5094	0.0363
rs10483785	LC_IGP_R3	IgG1_G2F/IgG1_G2	14	66219517	1	8.22E-15	1.44E-14	+	0.26656	0.2883	+	A	G	0.484615831	0.0340108	+	A	G	0.5094	0.0375
rs10483785	LC_IGP_R8	IgG1_G2FS1/IgG1_G2S1	14	66219517	1	1.60E-09	2.11E-12	+	0.207849	0.2627	+	A	G	0.484319792	0.0342506	+	A	G	0.5094	0.0374
rs10483785	LC_IGP_R26	IgG1_G2N/IgG1_G1N	14	66219517	1	3.36E-10	3.29E-09	+	0.216884	0.2169	+	A	G	0.486149383	0.0343131	+	A	G	0.5094	0.0367
rs10483785	LC_IGP148	IgG2 FG0n total/G0n	14	66219517	1	5.14E-09	1.25E-12	+	0.201818	0.2657	+	A	G	0.483577894	0.0343567	+	A	G	0.5094	0.0374
rs10483785	LC_IGP97	IgG2_G0	14	66219517	1	2.36E-10	7.95E-13	-	-0.217626	-0.2683	+	A	G	0.48461522	0.0341336	+	A	G	0.5094	0.0375
rs10483785	LC_IGP_R41	IgG2_G0F/IgG2_G0	14	66219517	1	3.43E-08	4.43E-12	+	0.18976	0.2576	+	A	G	0.48461522	0.0342235	+	A	G	0.5094	0.0372
rs10483785	LC_IGP138	IgG2_G0n	14	66219517	1	1.32E-10	5.50E-13	-	-0.221489	-0.2706	+	A	G	0.483577894	0.0342492	+	A	G	0.5094	0.0375
rs12887673	LC_IGP78	IgG1 BG0n/G0n	14	66221771	1	1.50E-13	1.61E-10	-	-0.255446	-0.24	+	C	T	0.485153656	0.0342841	+	C	T	0.5099	0.0375
rs12887673	LC_IGP79	IgG1 BG1n/G1n	14	66221771	1	2.08E-12	2.78E-12	-	-0.243828	-0.262	+	C	T	0.485153656	0.0344189	+	C	T	0.5099	0.0375
rs12887673	LC_IGP77	IgG1 Bn	14	66221771	1	1.07E-12	4.22E-12	-	-0.247013	-0.261	+	C	T	0.485153656	0.0344119	+	C	T	0.5099	0.0377
rs12887673	LC_IGP86	IgG1 Bn/Fn total	14	66221771	1	1.90E-13	2.87E-12	-	-0.254979	-0.2632	+	C	T	0.485153656	0.0343681	+	C	T	0.5099	0.0377
rs12887673	LC_IGP84	IgG1 FBn/Bn total	14	66221771	1	5.56E-14	5.16E-16	+	0.261033	0.3079	+	C	T	0.485153656	0.0344121	+	C	T	0.5099	0.038
rs12887673	LC_IGP62	IgG1 FG0n total/G0n	14	66221771	1	3.97E-16	1.23E-12	+	0.280632	0.2685	+	C	T	0.485153656	0.0341193	+	C	T	0.5099	0.0378
rs12887673	LC_IGP63	IgG1 FG1n total/G1n	14	66221771	1	3.02E-16	2.56E-14	+	0.282583	0.2876	+	C	T	0.485153656	0.0342141	+	C	T	0.5099	0.0378
rs12887673	LC_IGP64	IgG1 FG2n total/G2n	14	66221771	1	3.85E-13	4.62E-15	+	0.251862	0.2939	+	C	T	0.485153656	0.0343996	+	C	T	0.5099	0.0375
rs12887673	LC_IGP68	IgG1 FG2n/G2n	14	66221771	1	2.47E-10	3.96E-09	+	0.219902	0.2197	+	C	T	0.485153656	0.0345238	+	C	T	0.5099	0.0373
rs12887673	LC_IGP61	IgG1 Fn total	14	66221771	1	1.33E-15	3.69E-14	+	0.276276	0.2862	+	C	T	0.485153656	0.0342211	+	C	T	0.5099	0.0378
rs12887673	LC_IGP81	IgG1 Fn/Bn	14	66221771	1	4.81E-13	1.91E-11	+	0.25063	0.2517	+	C	T	0.485153656	0.0343762	+	C	T	0.5099	0.0375
rs12887673	LC_IGP21	IgG1 Fucosylation	14	66221771	1	3.36E-14	5.76E-13	+	0.261729	0.2718	+	C	T	0.484145499	0.0342004	+	C	T	0.5099	0.0377
rs12887673	LC_IGP28	IgG1 G1S1/(G1+G1S1)	14	66221771	1	1.95E-09	8.63E-10	+	0.206405	0.2222	+	C	T	0.484277676	0.0341934	+	C	T	0.5099	0.0362
rs12887673	LC_IGP27	IgG1 GS1/(G0+G+GS1)	14	66221771	1	2.68E-08	1.51E-08	+	0.191628	0.2088	+	C	T	0.484277676	0.0342883	+	C	T	0.5099	0.0369
rs12887673	LC_IGP11	IgG1_G0	14	66221771	1	1.97E-17	8.28E-15	-	-0.290272	-0.292	+	C	T	0.484602321	0.0337862	+	C	T	0.5099	0.0376
rs12887673	LC_IGP_R1	IgG1_G0F/IgG1_G0	14	66221771	1	2.14E-15	2.68E-12	+	0.271304	0.2636	+	C	T	0.484602321	0.0338667	+	C	T	0.5099	0.0377
rs12887673	LC_IGP_R4	IgG1_G0FN/IgG1_G0N	14	66221771	1	1.43E-13	1.35E-13	+	0.254512	0.2808	+	C	T	0.485176363	0.0341321	+	C	T	0.5099	0.0379
rs12887673	LC_IGP52	IgG1_G0n	14	66221771	1	1.96E-17	7.33E-15	-	-0.292445	-0.2931	+	C	T	0.485153656	0.034032	+	C	T	0.5099	0.0377
rs12887673	LC_IGP14	IgG1_G0N	14	66221771	1	2.36E-16	3.49E-12	-	-0.281746	-0.2604	+	C	T	0.485176363	0.0339918	+	C	T	0.5099	0.0374
rs12887673	LC_IGP55	IgG1_G0Nn	14	66221771	1	1.73E-16	3.72E-12	-	-0.284605	-0.2603	+	C	T	0.485153656	0.0341724	+	C	T	0.5099	0.0375

rs12887673	LC_IGP12	IgG1_G1	14	66221771	1	6.48E-15	2.47E-13	-	-0.267248	-0.2764	+	C	T	0.484602321	0.0339645	+	C	T	0.5099	0.0378
rs12887673	LC_IGP_R2	IgG1_G1F/IgG1_G1	14	66221771	1	3.10E-16	1.95E-13	+	0.279976	0.277	+	C	T	0.484602321	0.0339162	+	C	T	0.5099	0.0377
rs12887673	LC_IGP_R5	IgG1_G1FN/IgG1_G1N	14	66221771	1	3.80E-13	4.95E-17	+	0.251109	0.3168	+	C	T	0.486035055	0.0342911	+	C	T	0.5099	0.0378
rs12887673	LC_IGP53	IgG1_G1n	14	66221771	1	4.97E-14	4.44E-13	-	-0.260656	-0.2732	+	C	T	0.485153656	0.0342942	+	C	T	0.5099	0.0377
rs12887673	LC_IGP15	IgG1_G1N	14	66221771	1	7.03E-11	3.54E-11	-	-0.225675	-0.2484	+	C	T	0.486035055	0.0343815	+	C	T	0.5099	0.0375
rs12887673	LC_IGP56	IgG1_G1Nn	14	66221771	1	3.92E-11	7.77E-11	-	-0.229443	-0.2437	+	C	T	0.485153656	0.0344866	+	C	T	0.5099	0.0375
rs12887673	LC_IGP_R32	IgG1_G1S1/IgG1_G1	14	66221771	1	3.38E-09	1.34E-09	+	0.203359	0.2199	+	C	T	0.484277676	0.0342098	+	C	T	0.5099	0.0363
rs12887673	LC_IGP_R3	IgG1_G2F/IgG1_G2	14	66221771	1	1.43E-14	1.46E-14	+	0.264236	0.2879	+	C	T	0.484287286	0.034029	+	C	T	0.5099	0.0374
rs12887673	LC_IGP_R8	IgG1_G2FS1/IgG1_G2S1	14	66221771	1	3.47E-09	2.10E-12	+	0.203588	0.2624	+	C	T	0.483991448	0.0342733	+	C	T	0.5099	0.0373
rs12887673	LC_IGP_R26	IgG1_G2N/IgG1_G1N	14	66221771	1	3.99E-10	3.37E-09	+	0.216002	0.2165	+	C	T	0.485825309	0.0343231	+	C	T	0.5099	0.0366
rs12887673	LC_IGP148	IgG2 FG0n total/G0n	14	66221771	1	8.64E-09	1.31E-12	+	0.198889	0.265	+	C	T	0.483246921	0.0343745	+	C	T	0.51	0.0374
rs12887673	LC_IGP97	IgG2_G0	14	66221771	1	4.08E-10	8.46E-13	-	-0.214796	-0.2676	+	C	T	0.484286675	0.0341517	+	C	T	0.5099	0.0374
rs12887673	LC_IGP138	IgG2_G0n	14	66221771	1	2.34E-10	5.83E-13	-	-0.218543	-0.2699	+	C	T	0.483246921	0.0342681	+	C	T	0.51	0.0375
rs35206478	LC_IGP78	IgG1 BG0n/G0n	14	66223213	1	1.62E-10	1.75E-10	+	0.252961	0.2849	+	G	A	0.266459108	0.0393056	+	G	A	0.2247	0.0446
rs35206478	LC_IGP79	IgG1 BG1n/G1n	14	66223213	1	2.16E-09	9.44E-12	+	0.237533	0.3038	+	G	A	0.266459108	0.0394588	+	G	A	0.2247	0.0446
rs35206478	LC_IGP77	IgG1 Bn	14	66223213	1	3.52E-10	1.56E-11	+	0.248904	0.302	+	G	A	0.266459108	0.0394234	+	G	A	0.2247	0.0448
rs35206478	LC_IGP86	IgG1 Bn/Fn total	14	66223213	1	6.30E-11	8.64E-12	+	0.259116	0.3059	+	G	A	0.266459108	0.0393738	+	G	A	0.2247	0.0448
rs35206478	LC_IGP84	IgG1 FBn/Bn total	14	66223213	1	8.57E-12	2.61E-15	-	-0.271093	-0.3568	+	G	A	0.266459108	0.0394059	+	G	A	0.2247	0.0451
rs35206478	LC_IGP62	IgG1 FG0n total/G0n	14	66223213	1	3.11E-14	1.99E-12	-	-0.299293	-0.3159	+	G	A	0.266459108	0.0390548	+	G	A	0.2247	0.0449
rs35206478	LC_IGP63	IgG1 FG1n total/G1n	14	66223213	1	5.36E-12	2.44E-13	-	-0.273002	-0.3283	+	G	A	0.266459108	0.0392931	+	G	A	0.2247	0.0448
rs35206478	LC_IGP64	IgG1 FG2n total/G2n	14	66223213	1	3.22E-11	1.09E-14	-	-0.263144	-0.3441	+	G	A	0.266459108	0.0393768	+	G	A	0.2247	0.0445
rs35206478	LC_IGP68	IgG1 FG2n/G2n	14	66223213	1	1.85E-08	2.72E-09	-	-0.223438	-0.264	+	G	A	0.266459108	0.0395156	+	G	A	0.2247	0.0444
rs35206478	LC_IGP61	IgG1 Fn total	14	66223213	1	3.64E-13	1.74E-13	-	-0.287328	-0.3307	+	G	A	0.266459108	0.0392012	+	G	A	0.2247	0.0449
rs35206478	LC_IGP81	IgG1 Fn/Bn	14	66223213	1	2.24E-10	4.34E-11	-	-0.251503	-0.2942	+	G	A	0.266459108	0.039391	+	G	A	0.2247	0.0446
rs35206478	LC_IGP21	IgG1 Fucosylation	14	66223213	1	1.26E-11	3.43E-13	-	-0.267602	-0.3259	+	G	A	0.266526819	0.0392268	+	G	A	0.2247	0.0448
rs35206478	LC_IGP11	IgG1_G0	14	66223213	1	5.14E-15	2.73E-14	+	0.306277	0.3399	+	G	A	0.266222663	0.0387773	+	G	A	0.2247	0.0447
rs35206478	LC_IGP_R1	IgG1_G0F/IgG1_G0	14	66223213	1	5.90E-14	8.42E-12	-	-0.294101	-0.3059	+	G	A	0.266222663	0.0388169	+	G	A	0.2247	0.0448
rs35206478	LC_IGP_R4	IgG1_G0FN/IgG1_G0N	14	66223213	1	7.20E-11	6.05E-14	-	-0.25709	-0.3382	+	G	A	0.2662624	0.0391904	+	G	A	0.2247	0.045
rs35206478	LC_IGP52	IgG1_G0n	14	66223213	1	3.30E-15	2.55E-14	+	0.310104	0.3409	+	G	A	0.266459108	0.0389732	+	G	A	0.2247	0.0447
rs35206478	LC_IGP14	IgG1_G0N	14	66223213	1	1.13E-11	6.41E-12	+	0.267635	0.3058	+	G	A	0.2662624	0.039137	+	G	A	0.2247	0.0445
rs35206478	LC_IGP55	IgG1_G0Nn	14	66223213	1	7.53E-12	6.98E-12	+	0.27088	0.3055	+	G	A	0.266459108	0.0392667	+	G	A	0.2247	0.0446
rs35206478	LC_IGP12	IgG1_G1	14	66223213	1	3.52E-12	5.90E-13	+	0.27332	0.323	+	G	A	0.2665281	0.0390007	+	G	A	0.2247	0.0448
rs35206478	LC_IGP_R2	IgG1_G1F/IgG1_G1	14	66223213	1	4.78E-12	1.20E-12	-	-0.271752	-0.3179	+	G	A	0.2665281	0.0390239	+	G	A	0.2247	0.0448
rs35206478	LC_IGP_R5	IgG1_G1FN/IgG1_G1N	14	66223213	1	1.63E-10	4.79E-16	-	-0.252825	-0.3642	+	G	A	0.266178659	0.0392944	+	G	A	0.2247	0.0449
rs35206478	LC_IGP53	IgG1_G1n	14	66223213	1	2.25E-11	1.31E-12	+	0.264719	0.3178	+	G	A	0.266459108	0.0392966	+	G	A	0.2247	0.0448
rs35206478	LC_IGP15	IgG1_G1N	14	66223213	1	5.89E-09	2.61E-11	+	0.230296	0.2976	+	G	A	0.266178659	0.0393586	+	G	A	0.2247	0.0446
rs35206478	LC_IGP56	IgG1_G1Nn	14	66223213	1	5.37E-09	7.30E-11	+	0.231686	0.2903	+	G	A	0.266459108	0.0394878	+	G	A	0.2247	0.0446
rs35206478	LC_IGP_R3	IgG1_G2F/IgG1_G2	14	66223213	1	5.64E-12	1.10E-14	-	-0.270994	-0.3434	+	G	A	0.266385391	0.0390491	+	G	A	0.2247	0.0445
rs35206478	LC_IGP148	IgG2 FG0n total/G0n	14	66223213	1	4.60E-08	7.93E-08	-	-0.215665	-0.2384	+	G	A	0.266814347	0.0392682	+	G	A	0.2248	0.0444
rs35206478	LC_IGP97	IgG2_G0	14	66223213	1	2.94E-10	1.25E-08	+	0.247593	0.2533	+	G	A	0.266369499	0.0390429	+	G	A	0.2247	0.0445
rs35206478	LC_IGP_R41	IgG2_G0F/IgG2_G0	14	66223213	1	1.50E-08	2.48E-08	-	-0.222612	-0.2465	+	G	A	0.266369499	0.0391214	+	G	A	0.2247	0.0442
rs35206478	LC_IGP138	IgG2_G0n	14	66223213	1	1.62E-10	1.31E-08	+	0.251616	0.2531	+	G	A	0.266814347	0.0390987	+	G	A	0.2248	0.0445
rs6573624	LC_IGP78	IgG1 BG0n/G0n	14	66226885	1	1.26E-13	1.59E-10	-	-0.257023	-0.2401	+	A	G	0.482312887	0.0343819	+	A	G	0.51	0.0375
rs6573624	LC_IGP79	IgG1 BG1n/G1n	14	66226885	1	2.09E-12	2.79E-12	-	-0.244533	-0.262	+	A	G	0.482312887	0.0345209	+	A	G	0.51	0.0375
rs6573624	LC_IGP77	IgG1 Bn	14	66226885	1	9.64E-13	4.21E-12	-	-0.248257	-0.2611	+	A	G	0.482312887	0.0345116	+	A	G	0.51	0.0377
rs6573624	LC_IGP86	IgG1 Bn/Fn total	14	66226885	1	1.67E-13	2.88E-12	-	-0.256322	-0.2632	+	A	G	0.482312887	0.0344672	+	A	G	0.51	0.0377
rs6573624	LC_IGP84	IgG1 FBn/Bn total	14	66226885	1	3.96E-14	5.37E-16	+	0.263328	0.3078	+	A	G	0.482312887	0.0345068	+	A	G	0.51	0.038
rs6573624	LC_IGP62	IgG1 FG0n total/G0n	14	66226885	1	2.64E-16	1.24E-12	+	0.283139	0.2685	+	A	G	0.482312887	0.0342118	+	A	G	0.51	0.0378
rs6573624	LC_IGP63	IgG1 FG1n total/G1n	14	66226885	1	2.87E-16	2.63E-14	+	0.283628	0.2876	+	A	G	0.482312887	0.0343144	+	A	G	0.51	0.0378
rs6573624	LC_IGP64	IgG1 FG2n total/G2n	14	66226885	1	4.17E-13	4.78E-15	+	0.252232	0.2938	+	A	G	0.482312887	0.0345031	+	A	G	0.51	0.0375
rs6573624	LC_IGP68	IgG1 FG2n/G2n	14	66226885	1	3.03E-10	3.91E-09	+	0.219455	0.2198	+	A	G	0.482312887	0.0346303	+	A	G	0.51	0.0373
rs6573624	LC_IGP61	IgG1 Fn total	14	66226885	1	1.07E-15	3.78E-14	+	0.277991	0.2861	+	A	G	0.482312887	0.0343179	+	A	G	0.51	0.0378
rs6573624	LC_IGP81	IgG1 Fn/Bn	14	66226885	1	4.56E-13	1.89E-11	+	0.251623	0.2518	+	A	G	0.482312887	0.0344769	+	A	G	0.51	0.0375
rs6573624	LC_IGP21	IgG1 Fucosylation	14	66226885	1	2.29E-14	5.88E-13	+	0.264198	0.2718	+	A	G	0.481297472	0.0342955	+	A	G	0.51	0.0377
rs6573624	LC_IGP28	IgG1 G1S1/(G1+G1S1)	14	66226885	1	4.02E-09	8.93E-10	+	0.202966	0.2221	+	A	G	0.481453211	0.0343101	+	A	G	0.51	0.0362
rs6573624	LC_IGP27	IgG1 GS1/(G0+G+GS1)	14	66226885	1	3.68E-08	1.53E-08	+	0.190288	0.2087	+	A	G	0.481453211	0.034397	+	A	G	0.51	0.0369
rs6573624	LC_IGP11	IgG1_G0	14	66226885	1	1.36E-17	8.62E-15	-	-0.292582	-0.2919	+	A	G	0.481781307	0.0338789	+	A	G	0.51	0.0376
rs6573624	LC_IGP_R1	IgG1_G0F/IgG1_G0	14	66226885	1	1.47E-15	2.70E-12	+	0.273669	0.2636	+	A	G	0.481781307	0.0339596	+	A	G	0.51	0.0377
rs6573624	LC_IGP_R4	IgG1_G0FN/IgG1_G0N	14	66226885	1	9.99E-14	1.38E-13	+	0.256892	0.2808	+	A	G	0.482348132	0.034226	+	A	G	0.51	0.038
rs6573624	LC_IGP52	IgG1_G0n	14	66226885	1	1.33E-17	7.64E-15	-	-0.294821	-0.293	+	A	G	0.482312887	0.0341247	+	A	G	0.51	0.0377
rs6573624	LC_IGP14	IgG1_G0N	14	66226885	1	2.09E-16	3.49E-12	-	-0.283084	-0.2604	+	A	G	0.482348132	0.0340903	+	A	G	0.51	0.0374
rs6573624	LC_IGP55	IgG1_G0Nn	14	66226885	1	1.49E-16	3.73E-12	-	-0.286049	-0.2604	+	A	G	0.482312887	0.0342705	+	A	G	0.51	0.0375
rs6573624	LC_IGP12	IgG1_G1	14	66226885	1	5.91E-15	2.53E-13	-	-0.268431	-0.2764	+	A	G	0.481781307	0.0340635	+	A	G	0.51	0.0378
rs6573624	LC_IGP_R2	IgG1_G1F/IgG1_G1	14	66226885	1	3.27E-16	1.98E-13	+	0.28059	0.277	+	A	G	0.481781307	0.034018	+	A	G	0.51	0.0377
rs6573624	LC_IGP_R5	IgG1_G1FN/IgG1_G1N	14	66226885	1	3.21E-13	5.19E-17	+	0.252637	0.3167	+	A	G	0.483194649	0.0343892	+	A	G	0.51	0.0378
rs6573624	LC_IGP53	IgG1_G1n	14	66226885	1	4.55E-14	4.54E-13	-	-0.261821	-0.2732	+	A	G	0.482312887	0.0343939	+	A	G	0.51	0.0377
rs6573624	LC_IGP15	IgG1_G1N	14	66226885	1	6.48E-11	3.54E-11	-	-0.226767	-0.2485	+	A	G	0.483194649	0.0344816	+	A	G	0.51	0.0375
rs6573624	LC_IGP56	IgG1_G1Nn	14	66226885	1	3.59E-11	7.76E-11	-	-0.23057	-0.2437	+	A	G	0.482312887	0.0345869	+	A	G	0.51	0.0375
rs6573624	LC_IGP_R32	IgG1_G1S1/IgG1_G1	14	66226885	1	6.85E-09	1.39E-09	+	0.199964	0.2198	+	A	G	0.481453211	0.0343261	+	A	G	0.51	0.0363
rs6573624	LC_IGP_R3	IgG1_G2F/IgG1_G2	14	66226885	1	1.56E-14	1.50E-14	+	0.264647	0.2879	+	A	G	0.481464548	0.0341322	+	A	G	0.51	0.0374
rs6573624	LC_IGP_R8	IgG1_G2FS1/IgG1_G2S1	14	66226885	1	3.51E-09	2.13E-12	+	0.204135	0.2624	+	A	G	0.481170434	0.0343758	+	A	G	0.51	0.0374
rs6573624	LC_IGP_R26	IgG1_G2N/IgG1_G1N	14	66226885	1	4.79E-10	3.37E-09	+	0.215663	0.2166	+	A	G	0.482994753	0.0344275	+	A	G	0.51	0.0366
rs6573624	LC_IGP148	IgG2 FG0n total/G0n	14	66226885	1	6.36E-09	1.27E-12	+	0.201261	0.2652	+	A	G	0.480403017	0.0344723	+	A	G	0.51	0.0374
rs6573624	LC_IGP97	IgG2_G0	14	66226885	1	3.68E-10	8.30E-13	-	-0.215986	-0.2678	+	A	G	0.481463631	0.0342513	+	A	G	0.51	0.0374
rs6573624	LC_IGP_R41	IgG2_G0F/IgG2_G0	14	66226885	1	4.61E-08	4.50E-12	+	0.188574	0.2572	+	A	G	0.481463631	0.0343385	+	A	G	0.51	0.0372
rs6573624	LC_IGP138	IgG2_G0n	14	66226885	1	2.02E-10	5.71E-13	-	-0.219986	-0.2701	+	A	G	0.480403017	0.0343688	+	A	G	0.51	0.0375
rs7148737	LC_IGP78	IgG1 BG0n/G0n	14	66230032	1	2.60E-12	1.12E-06	-	-0.246539	-0.1911	+	A	C	0.594668216	0.0349575	+	A	C	0.6192	0.0392
rs7148737	LC_IGP79	IgG1 BG1n/G1n	14	66230032	1	2.87E-11	8.51E-07	-	-0.235107	-0.1929	+	A	C	0.594668216	0.03509	+	A	C	0.6192	0.0392
rs7148737	LC_IGP77	IgG1 Bn	14	66230032	1	7.90E-12	3.94E-07	-	-0.241677	-0.1997	+	A	C	0.594668216	0.0350696	+	A	C	0.6192	0.0394
rs7148737	LC_IGP86	IgG1 Bn/Fn total	14	66230032	1	1.74E-12	2.69E-07	-	-0.249065	-0.2026	+	A	C	0.594668216	0.0350296	+	A	C	0.6192	0.0394
rs7148737	LC_IGP84	IgG1 FBn/Bn total	14	66230032	1	3.99E-12	5.04E-10	+	0.245505	0.2466	+	A	C	0.594668216	0.0351185	+	A	C	0.6192	0.0397
rs7148737	LC_IGP62	IgG1 FG0n total/G0n	14	66230032	1	4.46E-15	1.76E-08	+	0.27539	0.2226	+	A	C	0.594668216	0.0347805	+	A	C	0.6192	0.0395
rs7148737	LC_IGP63	IgG1 FG1n total/G1n	14	66230032	1	1.69E-13	1.24E-08	+	0.259923	0.2245	+	A	C	0.594668216	0.0349606	+	A	C	0.6192	0.0394
rs7148737	LC_IGP64	IgG1 FG2n total/G2n	14	66230032	1	9.50E-11	8.57E-10	+	0.228993	0.24	+	A	C	0.594668216	0.0351319	+	A	C	0.6192	0.0391
rs7148737	LC_IGP61	IgG1 Fn total	14	66230032	1	1.15E-13	4.72E-09	+	0.261526	0.2312	+	A	C	0.594668216	0.0349278	+	A	C	0.6192	0.0395
rs7148737	LC_IGP81	IgG1 Fn/Bn	14	66230032	1	3.10E-12	1.01E-06	+	0.246166	0.1918	+	A	C	0.594668216	0.0350303	+	A	C	0.6192	0.0392
rs7148737	LC_IGP21	IgG1 Fucosylation	14	66230032	1	3.72E-12	7.51E-09	+	0.243956	0.2277	+	A	C	0.593625462	0.0348472	+	A	C	0.6192	0.0394
rs7148737	LC_IGP11	IgG1_G0	14	66230032	1	1.48E-15	5.15E-11	-	-0.277523	-0.2581	+	A	C	0.594294136	0.034441	+	A	C	0.6192	0.0393
rs7148737	LC_IGP_R1	IgG1_G0F/IgG1_G0	14	66230032	1	1.23E-14	3.27E-08	+	0.268334	0.2177	+	A	C	0.594294136	0.0344695	+	A	C	0.6192	0.0394
rs7148737	LC_IGP_R4	IgG1_G0FN/IgG1_G0N	14	66230032	1	1.80E-11	3.63E-09	+	0.235649	0.2338	+	A	C	0.594525107	0.0348123	+	A	C	0.6192	0.0396
rs7148737	LC_IGP52	IgG1_G0n	14	66230032	1	8.33E-16	5.33E-11	-	-0.282343	-0.2584	+	A	C	0.594668216	0.0347198	+	A	C	0.6192	0.0394
rs7148737	LC_IGP14	IgG1_G0N	14	66230032	1	1.85E-14	2.26E-08	-	-0.267955	-0.2189	+	A	C	0.594525107	0.0346598	+	A	C	0.6192	0.0392
rs7148737	LC_IGP55	IgG1_G0Nn	14	66230032	1	2.24E-14	2.74E-08	-	-0.268868	-0.2178	+	A	C	0.594668216	0.0348868	+	A	C	0.6192	0.0392
rs7148737	LC_IGP12	IgG1_G1	14	66230032	1	1.11E-12	1.45E-08	-	-0.248537	-0.2235	+	A	C	0.593988699	0.0346531	+	A	C	0.6192	0.0394
rs7148737	LC_IGP_R2	IgG1_G1F/IgG1_G1	14	66230032	1	1.11E-13	2.98E-08	+	0.25935	0.2181	+	A	C	0.593988699	0.0346192	+	A	C	0.6192	0.0394
rs7148737	LC_IGP_R5	IgG1_G1FN/IgG1_G1N	14	66230032	1	4.83E-11	3.88E-10	+	0.231611	0.2469	+	A	C	0.595547048	0.0349811	+	A	C	0.6192	0.0394
rs7148737	LC_IGP53	IgG1_G1n	14	66230032	1	1.18E-11	2.45E-08	-	-0.239278	-0.2197	+	A	C	0.594668216	0.0350237	+	A	C	0.6192	0.0394
rs7148737	LC_IGP15	IgG1_G1N	14	66230032	1	6.50E-10	2.16E-06	-	-0.217644	-0.1858	+	A	C	0.595547048	0.0350176	+	A	C	0.6192	0.0392
rs7148737	LC_IGP_R3	IgG1_G2F/IgG1_G2	14	66230032	1	1.32E-11	1.20E-09	+	0.236699	0.2376	+	A	C	0.594046149	0.0347326	+	A	C	0.6192	0.0391
rs7148737	LC_IGP_R8	IgG1_G2FS1/IgG1_G2S1	14	66230032	1	1.42E-08	1.19E-08	+	0.198694	0.2222	+	A	C	0.593683262	0.034857	+	A	C	0.6192	0.039
rs7149301	LC_IGP78	IgG1 BG0n/G0n	14	66230414	1	4.12E-12	2.37E-10	-	-0.23907	-0.2414	+	G	A	0.5168671	0.0342209	+	G	A	0.5434	0.0381
rs7149301	LC_IGP79	IgG1 BG1n/G1n	14	66230414	1	3.92E-11	9.72E-12	-	-0.228522	-0.2592	+	G	A	0.5168671	0.0343474	+	G	A	0.5434	0.0381
rs7149301	LC_IGP77	IgG1 Bn	14	66230414	1	1.59E-11	1.24E-11	-	-0.233064	-0.2591	+	G	A	0.5168671	0.0343355	+	G	A	0.5434	0.0382
rs7149301	LC_IGP86	IgG1 Bn/Fn total	14	66230414	1	3.17E-12	6.89E-12	-	-0.240875	-0.2624	+	G	A	0.5168671	0.0342944	+	G	A	0.5434	0.0382
rs7149301	LC_IGP84	IgG1 FBn/Bn total	14	66230414	1	6.59E-12	6.99E-16	+	0.237829	0.3111	+	G	A	0.5168671	0.0343793	+	G	A	0.5434	0.0385
rs7149301	LC_IGP62	IgG1 FG0n total/G0n	14	66230414	1	4.84E-14	1.83E-12	+	0.25921	0.2704	+	G	A	0.5168671	0.0340876	+	G	A	0.5434	0.0384
rs7149301	LC_IGP63	IgG1 FG1n total/G1n	14	66230414	1	4.52E-14	2.64E-14	+	0.260278	0.2918	+	G	A	0.5168671	0.0341866	+	G	A	0.5434	0.0383
rs7149301	LC_IGP64	IgG1 FG2n total/G2n	14	66230414	1	8.38E-11	2.93E-15	+	0.224751	0.3004	+	G	A	0.5168671	0.0343793	+	G	A	0.5434	0.0381
rs7149301	LC_IGP68	IgG1 FG2n/G2n	14	66230414	1	6.23E-09	6.26E-09	+	0.2013	0.2202	+	G	A	0.5168671	0.0344579	+	G	A	0.5434	0.0379
rs7149301	LC_IGP61	IgG1 Fn total	14	66230414	1	1.20E-13	4.01E-14	+	0.25575	0.29	+	G	A	0.5168671	0.034183	+	G	A	0.5434	0.0384
rs7149301	LC_IGP81	IgG1 Fn/Bn	14	66230414	1	5.06E-12	7.13E-11	+	0.238551	0.2482	+	G	A	0.5168671	0.0342927	+	G	A	0.5434	0.0381
rs7149301	LC_IGP21	IgG1 Fucosylation	14	66230414	1	1.87E-12	2.49E-13	+	0.242703	0.2802	+	G	A	0.515609741	0.0341883	+	G	A	0.5434	0.0383
rs7149301	LC_IGP28	IgG1 G1S1/(G1+G1S1)	14	66230414	1	1.75E-08	7.06E-10	+	0.193262	0.2268	+	G	A	0.515790826	0.0341248	+	G	A	0.5434	0.0368
rs7149301	LC_IGP11	IgG1_G0	14	66230414	1	1.83E-14	8.11E-15	-	-0.261485	-0.2964	+	G	A	0.51607697	0.033816	+	G	A	0.5434	0.0382
rs7149301	LC_IGP_R1	IgG1_G0F/IgG1_G0	14	66230414	1	1.53E-13	4.56E-12	+	0.252054	0.2646	+	G	A	0.51607697	0.0338441	+	G	A	0.5434	0.0382
rs7149301	LC_IGP_R4	IgG1_G0FN/IgG1_G0N	14	66230414	1	1.17E-11	1.26E-13	+	0.232986	0.2854	+	G	A	0.516422535	0.0340948	+	G	A	0.5434	0.0385
rs7149301	LC_IGP52	IgG1_G0n	14	66230414	1	1.80E-14	6.18E-15	-	-0.263338	-0.2983	+	G	A	0.5168671	0.0340424	+	G	A	0.5434	0.0382
rs7149301	LC_IGP14	IgG1_G0N	14	66230414	1	1.50E-14	3.56E-12	-	-0.263402	-0.2643	+	G	A	0.516422535	0.03395	+	G	A	0.5434	0.038
rs7149301	LC_IGP55	IgG1_G0Nn	14	66230414	1	2.37E-14	3.18E-12	-	-0.262888	-0.2651	+	G	A	0.5168671	0.0341431	+	G	A	0.5434	0.038

rs7149301	LC_IGP12	IgG1_G1	14	66230414	1	5.85E-13	1.45E-13	-	-0.24654	-0.2832	+	G	A	0.51607697	0.0339465	+	G	A	0.5434	0.0383
rs7149301	LC_IGP_R2	IgG1_G1F/IgG1_G1	14	66230414	1	3.16E-14	1.82E-13	+	0.259689	0.2815	+	G	A	0.51607697	0.0339009	+	G	A	0.5434	0.0382
rs7149301	LC_IGP_R5	IgG1_G1FN/IgG1_G1N	14	66230414	1	1.81E-11	7.11E-17	+	0.231818	0.3199	+	G	A	0.517415744	0.0342503	+	G	A	0.5434	0.0383
rs7149301	LC_IGP53	IgG1_G1n	14	66230414	1	3.40E-12	2.23E-13	-	-0.240222	-0.2808	+	G	A	0.5168671	0.03425	+	G	A	0.5434	0.0383
rs7149301	LC_IGP15	IgG1_G1N	14	66230414	1	4.81E-10	1.06E-10	-	-0.214841	-0.246	+	G	A	0.517415744	0.0343	+	G	A	0.5434	0.0381
rs7149301	LC_IGP56	IgG1_G1Nn	14	66230414	1	3.93E-10	2.07E-10	-	-0.216593	-0.2417	+	G	A	0.5168671	0.0344017	+	G	A	0.5434	0.038
rs7149301	LC_IGP_R32	IgG1_G1S1/IgG1_G1	14	66230414	1	2.79E-08	1.03E-09	+	0.190548	0.2249	+	G	A	0.515790826	0.0341393	+	G	A	0.5434	0.0368
rs7149301	LC_IGP_R3	IgG1_G2F/IgG1_G2	14	66230414	1	4.64E-12	6.68E-15	+	0.237156	0.296	+	G	A	0.515781174	0.0340344	+	G	A	0.5434	0.038
rs7149301	LC_IGP_R8	IgG1_G2FS1/IgG1_G2S1	14	66230414	1	2.78E-08	2.22E-12	+	0.190893	0.266	+	G	A	0.515466097	0.0341997	+	G	A	0.5434	0.0379
rs7149301	LC_IGP_R26	IgG1_G2N/IgG1_G1N	14	66230414	1	1.07E-08	7.27E-10	+	0.196985	0.229	+	G	A	0.517421914	0.0342642	+	G	A	0.5434	0.0372
rs7149301	LC_IGP97	IgG2_G0	14	66230414	1	6.92E-09	3.65E-13	-	-0.19856	-0.276	+	G	A	0.515781174	0.034096	+	G	A	0.5434	0.038
rs7149301	LC_IGP138	IgG2_G0n	14	66230414	1	3.47E-09	2.13E-13	-	-0.20318	-0.2792	+	G	A	0.514974138	0.0342011	+	G	A	0.5435	0.038
rs2411405	LC_IGP78	IgG1 BG0n/G0n	14	66232086	1	4.50E-12	2.11E-10	-	-0.238106	-0.242	+	G	A	0.517555762	0.0341454	+	G	A	0.544	0.0381
rs2411405	LC_IGP79	IgG1 BG1n/G1n	14	66232086	1	3.71E-11	9.64E-12	-	-0.228282	-0.2589	+	G	A	0.517555762	0.0342687	+	G	A	0.544	0.038
rs2411405	LC_IGP77	IgG1 Bn	14	66232086	1	1.58E-11	1.18E-11	-	-0.232571	-0.2591	+	G	A	0.517555762	0.0342578	+	G	A	0.544	0.0382
rs2411405	LC_IGP86	IgG1 Bn/Fn total	14	66232086	1	3.19E-12	6.61E-12	-	-0.240311	-0.2624	+	G	A	0.517555762	0.034217	+	G	A	0.544	0.0382
rs2411405	LC_IGP84	IgG1 FBn/Bn total	14	66232086	1	8.31E-12	7.44E-16	+	0.236162	0.3104	+	G	A	0.517555762	0.0343065	+	G	A	0.544	0.0385
rs2411405	LC_IGP62	IgG1 FG0n total/G0n	14	66232086	1	6.08E-14	1.83E-12	+	0.257617	0.27	+	G	A	0.517555762	0.0340153	+	G	A	0.544	0.0383
rs2411405	LC_IGP63	IgG1 FG1n total/G1n	14	66232086	1	5.45E-14	2.89E-14	+	0.25886	0.2909	+	G	A	0.517555762	0.0341134	+	G	A	0.544	0.0383
rs2411405	LC_IGP64	IgG1 FG2n total/G2n	14	66232086	1	9.87E-11	3.37E-15	+	0.2234	0.2994	+	G	A	0.517555762	0.0343051	+	G	A	0.544	0.038
rs2411405	LC_IGP68	IgG1 FG2n/G2n	14	66232086	1	5.66E-09	6.27E-09	+	0.20139	0.2201	+	G	A	0.517555762	0.0343781	+	G	A	0.544	0.0379
rs2411405	LC_IGP61	IgG1 Fn total	14	66232086	1	1.45E-13	4.32E-14	+	0.254311	0.2893	+	G	A	0.517555762	0.0341098	+	G	A	0.544	0.0383
rs2411405	LC_IGP81	IgG1 Fn/Bn	14	66232086	1	4.86E-12	6.62E-11	+	0.238202	0.2484	+	G	A	0.517555762	0.0342145	+	G	A	0.544	0.038
rs2411405	LC_IGP21	IgG1 Fucosylation	14	66232086	1	1.92E-12	2.66E-13	+	0.242032	0.2796	+	G	A	0.516246609	0.0341123	+	G	A	0.544	0.0382
rs2411405	LC_IGP28	IgG1 G1S1/(G1+G1S1)	14	66232086	1	2.95E-08	8.33E-10	+	0.189754	0.2256	+	G	A	0.516423242	0.0340596	+	G	A	0.544	0.0368
rs2411405	LC_IGP11	IgG1_G0	14	66232086	1	2.70E-14	8.19E-15	-	-0.259224	-0.2959	+	G	A	0.516708613	0.033749	+	G	A	0.544	0.0381
rs2411405	LC_IGP_R1	IgG1_G0F/IgG1_G0	14	66232086	1	1.76E-13	4.64E-12	+	0.250869	0.2642	+	G	A	0.516708613	0.0337719	+	G	A	0.544	0.0382
rs2411405	LC_IGP_R4	IgG1_G0FN/IgG1_G0N	14	66232086	1	1.45E-11	1.19E-13	+	0.231413	0.2854	+	G	A	0.517055726	0.0340234	+	G	A	0.544	0.0385
rs2411405	LC_IGP52	IgG1_G0n	14	66232086	1	2.71E-14	6.34E-15	-	-0.260979	-0.2977	+	G	A	0.517555762	0.033974	+	G	A	0.544	0.0382
rs2411405	LC_IGP14	IgG1_G0N	14	66232086	1	1.80E-14	2.95E-12	-	-0.262042	-0.265	+	G	A	0.517055726	0.0338782	+	G	A	0.544	0.038
rs2411405	LC_IGP55	IgG1_G0Nn	14	66232086	1	3.01E-14	2.67E-12	-	-0.261249	-0.2658	+	G	A	0.517555762	0.034071	+	G	A	0.544	0.038
rs2411405	LC_IGP12	IgG1_G1	14	66232086	1	7.03E-13	1.63E-13	-	-0.245148	-0.2823	+	G	A	0.516708613	0.0338751	+	G	A	0.544	0.0383
rs2411405	LC_IGP_R2	IgG1_G1F/IgG1_G1	14	66232086	1	3.74E-14	1.98E-13	+	0.258375	0.2807	+	G	A	0.516708613	0.0338292	+	G	A	0.544	0.0382
rs2411405	LC_IGP_R5	IgG1_G1FN/IgG1_G1N	14	66232086	1	1.91E-11	8.05E-17	+	0.231046	0.3189	+	G	A	0.518051661	0.0341751	+	G	A	0.544	0.0383
rs2411405	LC_IGP53	IgG1_G1n	14	66232086	1	4.13E-12	2.54E-13	-	-0.238754	-0.2798	+	G	A	0.517555762	0.0341767	+	G	A	0.544	0.0382
rs2411405	LC_IGP15	IgG1_G1N	14	66232086	1	4.16E-10	1.05E-10	-	-0.215139	-0.2458	+	G	A	0.518051661	0.0342207	+	G	A	0.544	0.038
rs2411405	LC_IGP56	IgG1_G1Nn	14	66232086	1	3.62E-10	2.09E-10	-	-0.216546	-0.2415	+	G	A	0.517555762	0.0343223	+	G	A	0.544	0.038
rs2411405	LC_IGP_R32	IgG1_G1S1/IgG1_G1	14	66232086	1	4.70E-08	1.23E-09	+	0.187009	0.2236	+	G	A	0.516423242	0.0340741	+	G	A	0.544	0.0368
rs2411405	LC_IGP_R3	IgG1_G2F/IgG1_G2	14	66232086	1	5.43E-12	7.60E-15	+	0.23588	0.295	+	G	A	0.516413203	0.0339621	+	G	A	0.544	0.038
rs2411405	LC_IGP_R8	IgG1_G2FS1/IgG1_G2S1	14	66232086	1	2.65E-08	2.24E-12	+	0.190771	0.2657	+	G	A	0.51609774	0.0341228	+	G	A	0.544	0.0379
rs2411405	LC_IGP_R26	IgG1_G2N/IgG1_G1N	14	66232086	1	1.20E-08	7.22E-10	+	0.195881	0.2288	+	G	A	0.518108025	0.0341896	+	G	A	0.544	0.0371
rs2411405	LC_IGP148	IgG2 FG0n total/G0n	14	66232086	1	4.20E-08	1.77E-12	+	0.188441	0.2672	+	G	A	0.515610837	0.0342074	+	G	A	0.5441	0.0379
rs2411405	LC_IGP97	IgG2_G0	14	66232086	1	4.18E-09	3.64E-13	-	-0.200971	-0.2758	+	G	A	0.516413203	0.0340101	+	G	A	0.544	0.0379
rs2411405	LC_IGP138	IgG2_G0n	14	66232086	1	2.05E-09	2.14E-13	-	-0.205634	-0.2789	+	G	A	0.515610837	0.0341137	+	G	A	0.5441	0.038
rs743084	LC_IGP78	IgG1 BG0n/G0n	14	66232602	1	4.11E-12	2.58E-10	-	-0.238791	-0.2404	+	C	T	0.516930297	0.0341793	+	C	T	0.5441	0.038
rs743084	LC_IGP79	IgG1 BG1n/G1n	14	66232602	1	4.36E-11	1.33E-11	-	-0.2277	-0.2569	+	C	T	0.516930297	0.0343079	+	C	T	0.5441	0.038
rs743084	LC_IGP77	IgG1 Bn	14	66232602	1	1.65E-11	1.59E-11	-	-0.232601	-0.2571	+	C	T	0.516930297	0.0342946	+	C	T	0.5441	0.0382
rs743084	LC_IGP86	IgG1 Bn/Fn total	14	66232602	1	3.33E-12	8.92E-12	-	-0.240348	-0.2604	+	C	T	0.516930297	0.0342538	+	C	T	0.5441	0.0382
rs743084	LC_IGP84	IgG1 FBn/Bn total	14	66232602	1	7.36E-12	1.15E-15	+	0.237002	0.308	+	C	T	0.516930297	0.0343398	+	C	T	0.5441	0.0385
rs743084	LC_IGP62	IgG1 FG0n total/G0n	14	66232602	1	5.46E-14	2.30E-12	+	0.258363	0.2684	+	C	T	0.516930297	0.0340487	+	C	T	0.5441	0.0383
rs743084	LC_IGP63	IgG1 FG1n total/G1n	14	66232602	1	5.52E-14	4.30E-14	+	0.259076	0.2887	+	C	T	0.516930297	0.0341493	+	C	T	0.5441	0.0382
rs743084	LC_IGP64	IgG1 FG2n total/G2n	14	66232602	1	1.00E-10	6.55E-15	+	0.223552	0.2963	+	C	T	0.516930297	0.0343413	+	C	T	0.5441	0.038
rs743084	LC_IGP68	IgG1 FG2n/G2n	14	66232602	1	6.78E-09	8.91E-09	+	0.200568	0.2177	+	C	T	0.516930297	0.0344178	+	C	T	0.5441	0.0379
rs743084	LC_IGP61	IgG1 Fn total	14	66232602	1	1.43E-13	6.47E-14	+	0.254654	0.2869	+	C	T	0.516930297	0.0341451	+	C	T	0.5441	0.0383
rs743084	LC_IGP81	IgG1 Fn/Bn	14	66232602	1	5.25E-12	9.10E-11	+	0.238085	0.2462	+	C	T	0.516930297	0.0342518	+	C	T	0.5441	0.038
rs743084	LC_IGP21	IgG1 Fucosylation	14	66232602	1	1.96E-12	4.14E-13	+	0.242193	0.277	+	C	T	0.51562053	0.0341485	+	C	T	0.5441	0.0382
rs743084	LC_IGP28	IgG1 G1S1/(G1+G1S1)	14	66232602	1	2.13E-08	1.19E-09	+	0.191887	0.2234	+	C	T	0.515802141	0.0340883	+	C	T	0.5441	0.0367
rs743084	LC_IGP11	IgG1_G0	14	66232602	1	2.09E-14	1.16E-14	-	-0.260599	-0.2939	+	C	T	0.516088271	0.0337787	+	C	T	0.5441	0.0381
rs743084	LC_IGP_R1	IgG1_G0F/IgG1_G0	14	66232602	1	1.65E-13	5.86E-12	+	0.251437	0.2625	+	C	T	0.516088271	0.0338055	+	C	T	0.5441	0.0381
rs743084	LC_IGP_R4	IgG1_G0FN/IgG1_G0N	14	66232602	1	1.14E-11	1.54E-13	+	0.232817	0.2836	+	C	T	0.516433864	0.0340539	+	C	T	0.5441	0.0384
rs743084	LC_IGP52	IgG1_G0n	14	66232602	1	2.11E-14	8.96E-15	-	-0.262328	-0.2957	+	C	T	0.516930297	0.0340044	+	C	T	0.5441	0.0381
rs743084	LC_IGP14	IgG1_G0N	14	66232602	1	1.41E-14	4.05E-12	-	-0.263379	-0.2629	+	C	T	0.516433864	0.0339083	+	C	T	0.5441	0.0379
rs743084	LC_IGP55	IgG1_G0Nn	14	66232602	1	2.37E-14	3.64E-12	-	-0.262558	-0.2638	+	C	T	0.516930297	0.0341017	+	C	T	0.5441	0.038
rs743084	LC_IGP12	IgG1_G1	14	66232602	1	6.79E-13	2.27E-13	-	-0.245567	-0.2803	+	C	T	0.516088271	0.0339094	+	C	T	0.5441	0.0382
rs743084	LC_IGP_R2	IgG1_G1F/IgG1_G1	14	66232602	1	3.69E-14	2.87E-13	+	0.258702	0.2784	+	C	T	0.516088271	0.033864	+	C	T	0.5441	0.0381
rs743084	LC_IGP_R5	IgG1_G1FN/IgG1_G1N	14	66232602	1	1.97E-11	1.37E-16	+	0.231137	0.3162	+	C	T	0.517427122	0.0342111	+	C	T	0.5441	0.0382
rs743084	LC_IGP53	IgG1_G1n	14	66232602	1	4.06E-12	3.52E-13	-	-0.239077	-0.2778	+	C	T	0.516930297	0.0342121	+	C	T	0.5441	0.0382
rs743084	LC_IGP15	IgG1_G1N	14	66232602	1	4.86E-10	1.34E-10	-	-0.214522	-0.2441	+	C	T	0.517427122	0.0342594	+	C	T	0.5441	0.038
rs743084	LC_IGP56	IgG1_G1Nn	14	66232602	1	4.30E-10	2.66E-10	-	-0.215843	-0.2398	+	C	T	0.516930297	0.0343618	+	C	T	0.5441	0.038
rs743084	LC_IGP_R32	IgG1_G1S1/IgG1_G1	14	66232602	1	3.38E-08	1.70E-09	+	0.189168	0.2216	+	C	T	0.515802141	0.0341028	+	C	T	0.5441	0.0368
rs743084	LC_IGP_R3	IgG1_G2F/IgG1_G2	14	66232602	1	5.54E-12	1.45E-14	+	0.23603	0.2919	+	C	T	0.515792482	0.0339977	+	C	T	0.5441	0.038
rs743084	LC_IGP_R8	IgG1_G2FS1/IgG1_G2S1	14	66232602	1	3.04E-08	4.08E-12	+	0.190149	0.2626	+	C	T	0.515477398	0.034161	+	C	T	0.5441	0.0379
rs743084	LC_IGP_R26	IgG1_G2N/IgG1_G1N	14	66232602	1	1.23E-08	8.14E-10	+	0.195934	0.2279	+	C	T	0.517484877	0.0342257	+	C	T	0.5441	0.0371
rs743084	LC_IGP97	IgG2_G0	14	66232602	1	6.75E-09	4.22E-13	-	-0.198472	-0.2748	+	C	T	0.515792482	0.0340551	+	C	T	0.5441	0.0379
rs743084	LC_IGP138	IgG2_G0n	14	66232602	1	3.33E-09	2.54E-13	-	-0.203154	-0.2778	+	C	T	0.51498553	0.0341595	+	C	T	0.5442	0.038
rs11625362	LC_IGP78	IgG1 BG0n/G0n	14	66232869	1	4.11E-12	2.16E-10	-	-0.238781	-0.2416	+	A	G	0.516933086	0.0341775	+	A	G	0.5436	0.038
rs11625362	LC_IGP79	IgG1 BG1n/G1n	14	66232869	1	4.37E-11	9.61E-12	-	-0.227675	-0.2588	+	A	G	0.516933086	0.0343062	+	A	G	0.5436	0.038
rs11625362	LC_IGP77	IgG1 Bn	14	66232869	1	1.65E-11	1.18E-11	-	-0.232587	-0.2589	+	A	G	0.516933086	0.0342929	+	A	G	0.5436	0.0382
rs11625362	LC_IGP86	IgG1 Bn/Fn total	14	66232869	1	3.34E-12	6.60E-12	-	-0.240332	-0.2622	+	A	G	0.516933086	0.0342521	+	A	G	0.5436	0.0382
rs11625362	LC_IGP84	IgG1 FBn/Bn total	14	66232869	1	7.39E-12	7.37E-16	+	0.236973	0.3102	+	A	G	0.516933086	0.0343382	+	A	G	0.5436	0.0385
rs11625362	LC_IGP62	IgG1 FG0n total/G0n	14	66232869	1	5.48E-14	1.81E-12	+	0.258333	0.2699	+	A	G	0.516933086	0.0340471	+	A	G	0.5436	0.0383
rs11625362	LC_IGP63	IgG1 FG1n total/G1n	14	66232869	1	5.56E-14	2.83E-14	+	0.259035	0.2909	+	A	G	0.516933086	0.0341477	+	A	G	0.5436	0.0382
rs11625362	LC_IGP64	IgG1 FG2n total/G2n	14	66232869	1	1.01E-10	3.23E-15	+	0.223511	0.2995	+	A	G	0.516933086	0.0343397	+	A	G	0.5436	0.038
rs11625362	LC_IGP68	IgG1 FG2n/G2n	14	66232869	1	6.80E-09	5.95E-09	+	0.200547	0.2202	+	A	G	0.516933086	0.0344161	+	A	G	0.5436	0.0378
rs11625362	LC_IGP61	IgG1 Fn total	14	66232869	1	1.44E-13	4.17E-14	+	0.254616	0.2892	+	A	G	0.516933086	0.0341435	+	A	G	0.5436	0.0383
rs11625362	LC_IGP81	IgG1 Fn/Bn	14	66232869	1	5.25E-12	6.64E-11	+	0.238072	0.2482	+	A	G	0.516933086	0.0342501	+	A	G	0.5436	0.038
rs11625362	LC_IGP21	IgG1 Fucosylation	14	66232869	1	1.96E-12	2.57E-13	+	0.242175	0.2796	+	A	G	0.515621455	0.0341468	+	A	G	0.5436	0.0382
rs11625362	LC_IGP28	IgG1 G1S1/(G1+G1S1)	14	66232869	1	2.14E-08	7.91E-10	+	0.19184	0.2258	+	A	G	0.515803058	0.0340868	+	A	G	0.5436	0.0367
rs11625362	LC_IGP11	IgG1_G0	14	66232869	1	2.10E-14	8.86E-15	-	-0.260567	-0.2954	+	A	G	0.516089188	0.0337771	+	A	G	0.5436	0.0381
rs11625362	LC_IGP_R1	IgG1_G0F/IgG1_G0	14	66232869	1	1.65E-13	4.55E-12	+	0.251415	0.2641	+	A	G	0.516089188	0.0338039	+	A	G	0.5436	0.0382
rs11625362	LC_IGP_R4	IgG1_G0FN/IgG1_G0N	14	66232869	1	1.14E-11	1.21E-13	+	0.23281	0.2851	+	A	G	0.516434783	0.0340522	+	A	G	0.5436	0.0384
rs11625362	LC_IGP52	IgG1_G0n	14	66232869	1	2.12E-14	6.75E-15	-	-0.262293	-0.2972	+	A	G	0.516933086	0.0340028	+	A	G	0.5436	0.0382
rs11625362	LC_IGP14	IgG1_G0N	14	66232869	1	1.40E-14	3.32E-12	-	-0.263377	-0.2642	+	A	G	0.516434783	0.0339066	+	A	G	0.5436	0.0379
rs11625362	LC_IGP55	IgG1_G0Nn	14	66232869	1	2.37E-14	2.96E-12	-	-0.262546	-0.265	+	A	G	0.516933086	0.0341	+	A	G	0.5436	0.038
rs11625362	LC_IGP12	IgG1_G1	14	66232869	1	6.82E-13	1.55E-13	-	-0.245534	-0.2823	+	A	G	0.516089188	0.0339078	+	A	G	0.5436	0.0382
rs11625362	LC_IGP_R2	IgG1_G1F/IgG1_G1	14	66232869	1	3.71E-14	1.92E-13	+	0.258668	0.2806	+	A	G	0.516089188	0.0338624	+	A	G	0.5436	0.0382
rs11625362	LC_IGP_R5	IgG1_G1FN/IgG1_G1N	14	66232869	1	1.97E-11	7.98E-17	+	0.231114	0.3188	+	A	G	0.517428044	0.0342095	+	A	G	0.5436	0.0383
rs11625362	LC_IGP53	IgG1_G1n	14	66232869	1	4.09E-12	2.39E-13	-	-0.239039	-0.2799	+	A	G	0.516933086	0.0342105	+	A	G	0.5436	0.0382
rs11625362	LC_IGP15	IgG1_G1N	14	66232869	1	4.86E-10	1.02E-10	-	-0.214515	-0.2458	+	A	G	0.517428044	0.0342577	+	A	G	0.5436	0.038
rs11625362	LC_IGP56	IgG1_G1Nn	14	66232869	1	4.31E-10	2.01E-10	-	-0.21582	-0.2415	+	A	G	0.516933086	0.0343602	+	A	G	0.5436	0.038
rs11625362	LC_IGP_R32	IgG1_G1S1/IgG1_G1	14	66232869	1	3.41E-08	1.16E-09	+	0.189121	0.2238	+	A	G	0.515803058	0.0341013	+	A	G	0.5436	0.0368
rs11625362	LC_IGP_R3	IgG1_G2F/IgG1_G2	14	66232869	1	5.57E-12	7.27E-15	+	0.235991	0.295	+	A	G	0.515793399	0.0339962	+	A	G	0.5436	0.0379
rs11625362	LC_IGP_R8	IgG1_G2FS1/IgG1_G2S1	14	66232869	1	3.04E-08	2.26E-12	+	0.190125	0.2655	+	A	G	0.515478314	0.0341594	+	A	G	0.5436	0.0378
rs11625362	LC_IGP_R26	IgG1_G2N/IgG1_G1N	14	66232869	1	1.24E-08	7.56E-10	+	0.195899	0.2284	+	A	G	0.517487654	0.0342241	+	A	G	0.5436	0.0371
rs11625362	LC_IGP97	IgG2_G0	14	66232869	1	6.73E-09	3.67E-13	-	-0.198472	-0.2755	+	A	G	0.515793399	0.0340534	+	A	G	0.5436	0.0379
rs11625362	LC_IGP138	IgG2_G0n	14	66232869	1	3.33E-09	2.17E-13	-	-0.203156	-0.2786	+	A	G	0.514986453	0.0341577	+	A	G	0.5437	0.038
rs4080329	LC_IGP78	IgG1 BG0n/G0n	14	66233490	1	3.72E-12	4.42E-07	-	-0.245898	-0.2	+	C	T	0.614622057	0.0351236	+	C	T	0.6454	0.0396
rs4080329	LC_IGP79	IgG1 BG1n/G1n	14	66233490	1	4.16E-11	3.16E-07	-	-0.234254	-0.2022	+	C	T	0.614622057	0.0352569	+	C	T	0.6454	0.0395
rs4080329	LC_IGP77	IgG1 Bn	14	66233490	1	8.17E-12	1.53E-07	-	-0.242602	-0.2086	+	C	T	0.614622057	0.0352292	+	C	T	0.6454	0.0397
rs4080329	LC_IGP86	IgG1 Bn/Fn total	14	66233490	1	1.37E-12	1.08E-07	-	-0.251342	-0.2111	+	C	T	0.614622057	0.0351833	+	C	T	0.6454	0.0397
rs4080329	LC_IGP84	IgG1 FBn/Bn total	14	66233490	1	2.01E-12	3.88E-10	+	0.249986	0.2505	+	C	T	0.614622057	0.0352629	+	C	T	0.6454	0.04
rs4080329	LC_IGP62	IgG1 FG0n total/G0n	14	66233490	1	6.41E-16	6.90E-09	+	0.284932	0.2308	+	C	T	0.614622057	0.0348967	+	C	T	0.6454	0.0398
rs4080329	LC_IGP63	IgG1 FG1n total/G1n	14	66233490	1	7.25E-14	4.69E-09	+	0.265009	0.233	+	C	T	0.614622057	0.0351008	+	C	T	0.6454	0.0398
rs4080329	LC_IGP64	IgG1 FG2n total/G2n	14	66233490	1	1.84E-11	2.67E-10	+	0.238559	0.2495	+	C	T	0.614622057	0.035256	+	C	T	0.6454	0.0395
rs4080329	LC_IGP61	IgG1 Fn total	14	66233490	1	1.61E-14	1.60E-09	+	0.271597	0.2403	+	C	T	0.614622057	0.035044	+	C	T	0.6454	0.0398
rs4080329	LC_IGP81	IgG1 Fn/Bn	14	66233490	1	2.93E-12	2.84E-07	+	0.247562	0.2031	+	C	T	0.614622057	0.0351878	+	C	T	0.6454	0.0396
rs4080329	LC_IGP21	IgG1 Fucosylation	14	66233490	1	5.61E-13	2.36E-09	+	0.25422	0.2373	+	C	T	0.61405672	0.0349732	+	C	T	0.6454	0.0398
rs4080329	LC_IGP11	IgG1_G0	14	66233490	1	8.53E-17	4.11E-11	-	-0.29068	-0.2615	+	C	T	0.61453879	0.0345473	+	C	T	0.6454	0.0396
rs4080329	LC_IGP_R1	IgG1_G0F/IgG1_G0	14	66233490	1	7.61E-16	1.23E-08	+	0.28154	0.2262	+	C	T	0.61453879	0.0345775	+	C	T	0.6454	0.0397

rs4080329	LC_IGP_R4	IgG1_G0FN/IgG1_G0N	14	66233490	1	1.91E-11	1.47E-09	+	0.236492	0.2417	+	C	T	0.614819351	0.0349836	+	C	T	0.6454	0.04
rs4080329	LC_IGP52	IgG1_G0n	14	66233490	1	6.88E-17	3.85E-11	-	-0.293966	-0.2624	+	C	T	0.614622057	0.0348239	+	C	T	0.6454	0.0397
rs4080329	LC_IGP14	IgG1_G0N	14	66233490	1	7.00E-14	1.65E-08	-	-0.263306	-0.2228	+	C	T	0.614819351	0.034857	+	C	T	0.6454	0.0395
rs4080329	LC_IGP55	IgG1_G0Nn	14	66233490	1	3.76E-14	1.80E-08	-	-0.267758	-0.2225	+	C	T	0.614622057	0.0350559	+	C	T	0.6454	0.0395
rs4080329	LC_IGP12	IgG1_G1	14	66233490	1	8.20E-14	5.57E-09	-	-0.261852	-0.2319	+	C	T	0.614233354	0.0347638	+	C	T	0.6454	0.0398
rs4080329	LC_IGP_R2	IgG1_G1F/IgG1_G1	14	66233490	1	1.63E-14	8.84E-09	+	0.269178	0.2283	+	C	T	0.614233354	0.0347443	+	C	T	0.6454	0.0397
rs4080329	LC_IGP_R5	IgG1_G1FN/IgG1_G1N	14	66233490	1	2.75E-11	3.06E-10	+	0.235564	0.2506	+	C	T	0.615313653	0.0351265	+	C	T	0.6454	0.0398
rs4080329	LC_IGP53	IgG1_G1n	14	66233490	1	1.66E-12	8.58E-09	-	-0.250088	-0.2289	+	C	T	0.614622057	0.0351403	+	C	T	0.6454	0.0398
rs4080329	LC_IGP15	IgG1_G1N	14	66233490	1	4.97E-10	8.05E-07	-	-0.220095	-0.1953	+	C	T	0.615313653	0.0351694	+	C	T	0.6454	0.0396
rs4080329	LC_IGP56	IgG1_G1Nn	14	66233490	1	2.31E-10	1.36E-06	-	-0.225217	-0.191	+	C	T	0.614622057	0.0353001	+	C	T	0.6454	0.0395
rs4080329	LC_IGP_R3	IgG1_G2F/IgG1_G2	14	66233490	1	1.49E-12	2.84E-10	+	0.248525	0.2487	+	C	T	0.614303178	0.034853	+	C	T	0.6454	0.0394
rs4080329	LC_IGP_R8	IgG1_G2FS1/IgG1_G2S1	14	66233490	1	5.22E-09	2.01E-09	+	0.205503	0.2359	+	C	T	0.613927917	0.0350004	+	C	T	0.6454	0.0393
rs4080329	LC_IGP_R26	IgG1_G2N/IgG1_G1N	14	66233490	1	4.37E-08	5.62E-07	+	0.193551	0.1934	+	C	T	0.615432099	0.0351822	+	C	T	0.6454	0.0386
rs4080329	LC_IGP97	IgG2_G0	14	66233490	1	7.30E-11	1.58E-06	-	-0.228305	-0.1896	+	C	T	0.613997555	0.0348134	+	C	T	0.6454	0.0395
rs4080329	LC_IGP138	IgG2_G0n	14	66233490	1	1.25E-11	1.33E-06	-	-0.238245	-0.191	+	C	T	0.613300493	0.0349156	+	C	T	0.6453	0.0395
rs11627605	LC_IGP78	IgG1 BG0n/G0n	14	66234313	1	4.55E-12	2.29E-10	-	-0.238037	-0.2414	+	A	G	0.51755886	0.0341425	+	A	G	0.5437	0.0381
rs11627605	LC_IGP79	IgG1 BG1n/G1n	14	66234313	1	3.82E-11	1.01E-11	-	-0.228104	-0.2587	+	A	G	0.51755886	0.0342662	+	A	G	0.5437	0.038
rs11627605	LC_IGP77	IgG1 Bn	14	66234313	1	1.61E-11	1.25E-11	-	-0.232471	-0.2587	+	A	G	0.51755886	0.034255	+	A	G	0.5437	0.0382
rs11627605	LC_IGP86	IgG1 Bn/Fn total	14	66234313	1	3.25E-12	7.03E-12	-	-0.240191	-0.262	+	A	G	0.51755886	0.0342143	+	A	G	0.5437	0.0382
rs11627605	LC_IGP84	IgG1 FBn/Bn total	14	66234313	1	8.64E-12	7.79E-16	+	0.235954	0.3102	+	A	G	0.51755886	0.0343041	+	A	G	0.5437	0.0385
rs11627605	LC_IGP62	IgG1 FG0n total/G0n	14	66234313	1	6.39E-14	1.96E-12	+	0.257375	0.2697	+	A	G	0.51755886	0.0340132	+	A	G	0.5437	0.0383
rs11627605	LC_IGP63	IgG1 FG1n total/G1n	14	66234313	1	5.82E-14	3.04E-14	+	0.258546	0.2908	+	A	G	0.51755886	0.0341116	+	A	G	0.5437	0.0383
rs11627605	LC_IGP64	IgG1 FG2n total/G2n	14	66234313	1	1.05E-10	3.45E-15	+	0.223053	0.2994	+	A	G	0.51755886	0.0343032	+	A	G	0.5437	0.038
rs11627605	LC_IGP68	IgG1 FG2n/G2n	14	66234313	1	5.88E-09	6.49E-09	+	0.201155	0.2199	+	A	G	0.51755886	0.0343757	+	A	G	0.5437	0.0379
rs11627605	LC_IGP61	IgG1 Fn total	14	66234313	1	1.55E-13	4.52E-14	+	0.254004	0.2891	+	A	G	0.51755886	0.034108	+	A	G	0.5437	0.0383
rs11627605	LC_IGP81	IgG1 Fn/Bn	14	66234313	1	4.95E-12	7.07E-11	+	0.2381	0.248	+	A	G	0.51755886	0.0342117	+	A	G	0.5437	0.038
rs11627605	LC_IGP21	IgG1 Fucosylation	14	66234313	1	1.97E-12	2.79E-13	+	0.241894	0.2794	+	A	G	0.516241369	0.0341099	+	A	G	0.5437	0.0382
rs11627605	LC_IGP28	IgG1 G1S1/(G1+G1S1)	14	66234313	1	3.11E-08	7.75E-10	+	0.189433	0.2261	+	A	G	0.516418043	0.0340578	+	A	G	0.5437	0.0368
rs11627605	LC_IGP11	IgG1_G0	14	66234313	1	2.84E-14	9.33E-15	-	-0.258988	-0.2954	+	A	G	0.516703421	0.0337472	+	A	G	0.5437	0.0381
rs11627605	LC_IGP_R1	IgG1_G0F/IgG1_G0	14	66234313	1	1.84E-13	4.92E-12	+	0.25066	0.2639	+	A	G	0.516703421	0.03377	+	A	G	0.5437	0.0382
rs11627605	LC_IGP_R4	IgG1_G0FN/IgG1_G0N	14	66234313	1	1.46E-11	1.29E-13	+	0.231344	0.2849	+	A	G	0.517048377	0.0340205	+	A	G	0.5437	0.0385
rs11627605	LC_IGP52	IgG1_G0n	14	66234313	1	2.86E-14	7.15E-15	-	-0.26072	-0.2972	+	A	G	0.51755886	0.033972	+	A	G	0.5437	0.0382
rs11627605	LC_IGP14	IgG1_G0N	14	66234313	1	1.78E-14	3.47E-12	-	-0.262062	-0.2641	+	A	G	0.517048377	0.0338749	+	A	G	0.5437	0.038
rs11627605	LC_IGP55	IgG1_G0Nn	14	66234313	1	3.02E-14	3.11E-12	-	-0.261208	-0.265	+	A	G	0.51755886	0.034068	+	A	G	0.5437	0.038
rs11627605	LC_IGP12	IgG1_G1	14	66234313	1	7.45E-13	1.68E-13	-	-0.244861	-0.2822	+	A	G	0.516703421	0.0338734	+	A	G	0.5437	0.0383
rs11627605	LC_IGP_R2	IgG1_G1F/IgG1_G1	14	66234313	1	3.96E-14	2.07E-13	+	0.258108	0.2805	+	A	G	0.516703421	0.0338276	+	A	G	0.5437	0.0382
rs11627605	LC_IGP_R5	IgG1_G1FN/IgG1_G1N	14	66234313	1	1.97E-11	8.22E-17	+	0.230873	0.3189	+	A	G	0.51804428	0.0341726	+	A	G	0.5437	0.0383
rs11627605	LC_IGP53	IgG1_G1n	14	66234313	1	4.40E-12	2.59E-13	-	-0.238422	-0.2797	+	A	G	0.51755886	0.0341749	+	A	G	0.5437	0.0382
rs11627605	LC_IGP15	IgG1_G1N	14	66234313	1	4.21E-10	1.09E-10	-	-0.215049	-0.2456	+	A	G	0.51804428	0.0342178	+	A	G	0.5437	0.038
rs11627605	LC_IGP56	IgG1_G1Nn	14	66234313	1	3.72E-10	2.14E-10	-	-0.216369	-0.2413	+	A	G	0.51755886	0.0343198	+	A	G	0.5437	0.038
rs11627605	LC_IGP_R32	IgG1_G1S1/IgG1_G1	14	66234313	1	4.94E-08	1.14E-09	+	0.186691	0.2242	+	A	G	0.516418043	0.0340723	+	A	G	0.5437	0.0368
rs11627605	LC_IGP_R3	IgG1_G2F/IgG1_G2	14	66234313	1	5.79E-12	7.78E-15	+	0.235551	0.295	+	A	G	0.516408007	0.0339606	+	A	G	0.5437	0.038
rs11627605	LC_IGP_R8	IgG1_G2FS1/IgG1_G2S1	14	66234313	1	2.72E-08	2.40E-12	+	0.190583	0.2654	+	A	G	0.516092547	0.0341206	+	A	G	0.5437	0.0379
rs11627605	LC_IGP_R26	IgG1_G2N/IgG1_G1N	14	66234313	1	1.24E-08	7.43E-10	+	0.195668	0.2287	+	A	G	0.518111111	0.0341872	+	A	G	0.5437	0.0372
rs11627605	LC_IGP148	IgG2 FG0n total/G0n	14	66234313	1	4.17E-08	1.87E-12	+	0.188463	0.2669	+	A	G	0.515605603	0.0342044	+	A	G	0.5438	0.0379
rs11627605	LC_IGP97	IgG2_G0	14	66234313	1	4.23E-09	3.82E-13	-	-0.200883	-0.2756	+	A	G	0.516408007	0.0340075	+	A	G	0.5437	0.038
rs11627605	LC_IGP138	IgG2_G0n	14	66234313	1	2.07E-09	2.23E-13	-	-0.205564	-0.2787	+	A	G	0.515605603	0.0341111	+	A	G	0.5438	0.038
rs11627578	LC_IGP78	IgG1 BG0n/G0n	14	66234448	1	4.56E-12	2.18E-10	-	-0.238033	-0.2418	+	T	C	0.517556382	0.0341437	+	T	C	0.544	0.0381
rs11627578	LC_IGP79	IgG1 BG1n/G1n	14	66234448	1	3.83E-11	9.94E-12	-	-0.228108	-0.2589	+	T	C	0.517556382	0.0342673	+	T	C	0.544	0.038
rs11627578	LC_IGP77	IgG1 Bn	14	66234448	1	1.61E-11	1.23E-11	-	-0.232473	-0.259	+	T	C	0.517556382	0.0342562	+	T	C	0.544	0.0382
rs11627578	LC_IGP86	IgG1 Bn/Fn total	14	66234448	1	3.26E-12	6.90E-12	-	-0.240192	-0.2622	+	T	C	0.517556382	0.0342155	+	T	C	0.544	0.0382
rs11627578	LC_IGP84	IgG1 FBn/Bn total	14	66234448	1	8.66E-12	7.65E-16	+	0.235951	0.3104	+	T	C	0.517556382	0.0343053	+	T	C	0.544	0.0385
rs11627578	LC_IGP62	IgG1 FG0n total/G0n	14	66234448	1	6.42E-14	1.92E-12	+	0.257362	0.27	+	T	C	0.517556382	0.0340145	+	T	C	0.544	0.0383
rs11627578	LC_IGP63	IgG1 FG1n total/G1n	14	66234448	1	5.84E-14	3.02E-14	+	0.25854	0.2909	+	T	C	0.517556382	0.0341128	+	T	C	0.544	0.0383
rs11627578	LC_IGP64	IgG1 FG2n total/G2n	14	66234448	1	1.06E-10	3.54E-15	+	0.22304	0.2994	+	T	C	0.517556382	0.0343044	+	T	C	0.544	0.038
rs11627578	LC_IGP68	IgG1 FG2n/G2n	14	66234448	1	5.90E-09	6.72E-09	+	0.201146	0.2198	+	T	C	0.517556382	0.0343769	+	T	C	0.544	0.0379
rs11627578	LC_IGP61	IgG1 Fn total	14	66234448	1	1.55E-13	4.53E-14	+	0.253993	0.2892	+	T	C	0.517556382	0.0341092	+	T	C	0.544	0.0383
rs11627578	LC_IGP81	IgG1 Fn/Bn	14	66234448	1	4.95E-12	6.91E-11	+	0.238102	0.2482	+	T	C	0.517556382	0.0342128	+	T	C	0.544	0.038
rs11627578	LC_IGP21	IgG1 Fucosylation	14	66234448	1	1.98E-12	2.79E-13	+	0.241891	0.2795	+	T	C	0.516239211	0.0341111	+	T	C	0.544	0.0383
rs11627578	LC_IGP28	IgG1 G1S1/(G1+G1S1)	14	66234448	1	3.12E-08	8.08E-10	+	0.189426	0.2259	+	T	C	0.516415902	0.0340591	+	T	C	0.544	0.0368
rs11627578	LC_IGP11	IgG1_G0	14	66234448	1	2.85E-14	8.52E-15	-	-0.258977	-0.296	+	T	C	0.516701283	0.0337485	+	T	C	0.544	0.0381
rs11627578	LC_IGP_R1	IgG1_G0F/IgG1_G0	14	66234448	1	1.85E-13	4.85E-12	+	0.250645	0.2641	+	T	C	0.516701283	0.0337713	+	T	C	0.544	0.0382
rs11627578	LC_IGP_R4	IgG1_G0FN/IgG1_G0N	14	66234448	1	1.47E-11	1.23E-13	+	0.231336	0.2853	+	T	C	0.517045928	0.0340217	+	T	C	0.544	0.0385
rs11627578	LC_IGP52	IgG1_G0n	14	66234448	1	2.87E-14	6.61E-15	-	-0.260709	-0.2978	+	T	C	0.517556382	0.0339732	+	T	C	0.544	0.0382
rs11627578	LC_IGP14	IgG1_G0N	14	66234448	1	1.79E-14	3.06E-12	-	-0.262064	-0.2649	+	T	C	0.517045928	0.0338761	+	T	C	0.544	0.038
rs11627578	LC_IGP55	IgG1_G0Nn	14	66234448	1	3.02E-14	2.78E-12	-	-0.261209	-0.2657	+	T	C	0.517556382	0.0340691	+	T	C	0.544	0.038
rs11627578	LC_IGP12	IgG1_G1	14	66234448	1	7.48E-13	1.71E-13	-	-0.244852	-0.2822	+	T	C	0.516701283	0.0338747	+	T	C	0.544	0.0383
rs11627578	LC_IGP_R2	IgG1_G1F/IgG1_G1	14	66234448	1	3.97E-14	2.08E-13	+	0.258101	0.2806	+	T	C	0.516701283	0.0338288	+	T	C	0.544	0.0382
rs11627578	LC_IGP_R5	IgG1_G1FN/IgG1_G1N	14	66234448	1	1.97E-11	8.08E-17	+	0.230874	0.319	+	T	C	0.51804182	0.0341737	+	T	C	0.544	0.0383
rs11627578	LC_IGP53	IgG1_G1n	14	66234448	1	4.42E-12	2.66E-13	-	-0.238411	-0.2797	+	T	C	0.517556382	0.0341761	+	T	C	0.544	0.0383
rs11627578	LC_IGP15	IgG1_G1N	14	66234448	1	4.21E-10	1.08E-10	-	-0.215051	-0.2457	+	T	C	0.51804182	0.034219	+	T	C	0.544	0.0381
rs11627578	LC_IGP56	IgG1_G1Nn	14	66234448	1	3.73E-10	2.15E-10	-	-0.216371	-0.2414	+	T	C	0.517556382	0.0343209	+	T	C	0.544	0.038
rs11627578	LC_IGP_R32	IgG1_G1S1/IgG1_G1	14	66234448	1	4.95E-08	1.18E-09	+	0.186685	0.224	+	T	C	0.516415902	0.0340735	+	T	C	0.544	0.0368
rs11627578	LC_IGP_R3	IgG1_G2F/IgG1_G2	14	66234448	1	5.81E-12	8.00E-15	+	0.235539	0.295	+	T	C	0.516405868	0.0339618	+	T	C	0.544	0.038
rs11627578	LC_IGP_R8	IgG1_G2FS1/IgG1_G2S1	14	66234448	1	2.72E-08	2.31E-12	+	0.190586	0.2657	+	T	C	0.516090409	0.0341218	+	T	C	0.544	0.0379
rs11627578	LC_IGP_R26	IgG1_G2N/IgG1_G1N	14	66234448	1	1.24E-08	6.95E-10	+	0.195672	0.2292	+	T	C	0.518108642	0.0341884	+	T	C	0.544	0.0372
rs11627578	LC_IGP148	IgG2 FG0n total/G0n	14	66234448	1	4.17E-08	1.84E-12	+	0.188461	0.2672	+	T	C	0.515603448	0.0342056	+	T	C	0.5441	0.0379
rs11627578	LC_IGP97	IgG2_G0	14	66234448	1	4.24E-09	3.82E-13	-	-0.200876	-0.2758	+	T	C	0.516405868	0.0340088	+	T	C	0.544	0.038
rs11627578	LC_IGP138	IgG2_G0n	14	66234448	1	2.08E-09	2.23E-13	-	-0.205557	-0.2789	+	T	C	0.515603448	0.0341123	+	T	C	0.5441	0.038
rs12050231	LC_IGP78	IgG1 BG0n/G0n	14	66235016	1	4.60E-12	2.11E-10	-	-0.238035	-0.2421	+	C	T	0.517548947	0.03415	+	C	T	0.5441	0.0381
rs12050231	LC_IGP79	IgG1 BG1n/G1n	14	66235016	1	3.85E-11	9.90E-12	-	-0.228118	-0.259	+	C	T	0.517548947	0.0342736	+	C	T	0.5441	0.038
rs12050231	LC_IGP77	IgG1 Bn	14	66235016	1	1.62E-11	1.20E-11	-	-0.232486	-0.2592	+	C	T	0.517548947	0.0342625	+	C	T	0.5441	0.0382
rs12050231	LC_IGP86	IgG1 Bn/Fn total	14	66235016	1	3.28E-12	6.74E-12	-	-0.240199	-0.2624	+	C	T	0.517548947	0.0342218	+	C	T	0.5441	0.0382
rs12050231	LC_IGP84	IgG1 FBn/Bn total	14	66235016	1	8.78E-12	7.40E-16	+	0.235925	0.3107	+	C	T	0.517548947	0.0343118	+	C	T	0.5441	0.0385
rs12050231	LC_IGP62	IgG1 FG0n total/G0n	14	66235016	1	6.56E-14	1.89E-12	+	0.257316	0.2701	+	C	T	0.517548947	0.034021	+	C	T	0.5441	0.0384
rs12050231	LC_IGP63	IgG1 FG1n total/G1n	14	66235016	1	5.96E-14	3.06E-14	+	0.258495	0.2909	+	C	T	0.517548947	0.0341194	+	C	T	0.5441	0.0383
rs12050231	LC_IGP64	IgG1 FG2n total/G2n	14	66235016	1	1.08E-10	3.55E-15	+	0.222961	0.2995	+	C	T	0.517548947	0.0343111	+	C	T	0.5441	0.038
rs12050231	LC_IGP68	IgG1 FG2n/G2n	14	66235016	1	5.99E-09	6.67E-09	+	0.201093	0.2199	+	C	T	0.517548947	0.0343834	+	C	T	0.5441	0.0379
rs12050231	LC_IGP61	IgG1 Fn total	14	66235016	1	1.59E-13	4.51E-14	+	0.253935	0.2893	+	C	T	0.517548947	0.0341158	+	C	T	0.5441	0.0383
rs12050231	LC_IGP81	IgG1 Fn/Bn	14	66235016	1	4.98E-12	6.77E-11	+	0.238116	0.2484	+	C	T	0.517548947	0.0342191	+	C	T	0.5441	0.0381
rs12050231	LC_IGP21	IgG1 Fucosylation	14	66235016	1	2.00E-12	2.73E-13	+	0.241883	0.2798	+	C	T	0.516233662	0.0341178	+	C	T	0.5441	0.0383
rs12050231	LC_IGP28	IgG1 G1S1/(G1+G1S1)	14	66235016	1	3.16E-08	8.58E-10	+	0.189386	0.2257	+	C	T	0.516410398	0.0340656	+	C	T	0.5441	0.0368
rs12050231	LC_IGP11	IgG1_G0	14	66235016	1	2.90E-14	8.57E-15	-	-0.258948	-0.296	+	C	T	0.516695785	0.0337551	+	C	T	0.5441	0.0382
rs12050231	LC_IGP_R1	IgG1_G0F/IgG1_G0	14	66235016	1	1.89E-13	4.81E-12	+	0.250593	0.2642	+	C	T	0.516695785	0.033778	+	C	T	0.5441	0.0382
rs12050231	LC_IGP_R4	IgG1_G0FN/IgG1_G0N	14	66235016	1	1.49E-11	1.18E-13	+	0.231308	0.2856	+	C	T	0.517038579	0.0340281	+	C	T	0.5441	0.0385
rs12050231	LC_IGP52	IgG1_G0n	14	66235016	1	2.92E-14	6.64E-15	-	-0.260681	-0.2978	+	C	T	0.517548947	0.0339797	+	C	T	0.5441	0.0382
rs12050231	LC_IGP14	IgG1_G0N	14	66235016	1	1.79E-14	2.98E-12	-	-0.262098	-0.2651	+	C	T	0.517038579	0.0338822	+	C	T	0.5441	0.038
rs12050231	LC_IGP55	IgG1_G0Nn	14	66235016	1	3.03E-14	2.70E-12	-	-0.261242	-0.2659	+	C	T	0.517548947	0.0340754	+	C	T	0.5441	0.038
rs12050231	LC_IGP12	IgG1_G1	14	66235016	1	7.66E-13	1.72E-13	-	-0.24479	-0.2822	+	C	T	0.516695785	0.0338814	+	C	T	0.5441	0.0383
rs12050231	LC_IGP_R2	IgG1_G1F/IgG1_G1	14	66235016	1	4.06E-14	2.11E-13	+	0.25806	0.2806	+	C	T	0.516695785	0.0338355	+	C	T	0.5441	0.0382
rs12050231	LC_IGP_R5	IgG1_G1FN/IgG1_G1N	14	66235016	1	1.99E-11	8.07E-17	+	0.23086	0.3192	+	C	T	0.51803444	0.0341801	+	C	T	0.5441	0.0383
rs12050231	LC_IGP53	IgG1_G1n	14	66235016	1	4.53E-12	2.67E-13	-	-0.238339	-0.2798	+	C	T	0.517548947	0.0341828	+	C	T	0.5441	0.0383
rs12050231	LC_IGP15	IgG1_G1N	14	66235016	1	4.25E-10	1.07E-10	-	-0.21505	-0.2458	+	C	T	0.51803444	0.0342253	+	C	T	0.5441	0.0381
rs12050231	LC_IGP56	IgG1_G1Nn	14	66235016	1	3.76E-10	2.12E-10	-	-0.21637	-0.2415	+	C	T	0.517548947	0.0343273	+	C	T	0.5441	0.038
rs12050231	LC_IGP_R3	IgG1_G2F/IgG1_G2	14	66235016	1	5.96E-12	8.04E-15	+	0.235466	0.295	+	C	T	0.516400367	0.0339686	+	C	T	0.5441	0.038
rs12050231	LC_IGP_R8	IgG1_G2FS1/IgG1_G2S1	14	66235016	1	2.75E-08	2.29E-12	+	0.190563	0.2658	+	C	T	0.516084911	0.0341283	+	C	T	0.5441	0.0379
rs12050231	LC_IGP_R26	IgG1_G2N/IgG1_G1N	14	66235016	1	1.25E-08	7.06E-10	+	0.195683	0.2292	+	C	T	0.518101235	0.0341946	+	C	T	0.5441	0.0372
rs12050231	LC_IGP148	IgG2 FG0n total/G0n	14	66235016	1	4.19E-08	1.88E-12	+	0.18847	0.2672	+	C	T	0.515597906	0.0342121	+	C	T	0.5442	0.0379
rs12050231	LC_IGP97	IgG2_G0	14	66235016	1	4.30E-09	3.90E-13	-	-0.200833	-0.2758	+	C	T	0.516400367	0.0340153	+	C	T	0.5441	0.038
rs12050231	LC_IGP138	IgG2_G0n	14	66235016	1	2.11E-09	2.29E-13	-	-0.205522	-0.2789	+	C	T	0.515597906	0.034119	+	C	T	0.5442	0.038
rs11158611	LC_IGP78	IgG1 BG0n/G0n	14	66235074	1	4.60E-12	2.12E-10	-	-0.238032	-0.2421	+	A	G	0.517546468	0.0341508	+	A	G	0.5441	0.0381
rs11158611	LC_IGP79	IgG1 BG1n/G1n	14	66235074	1	3.85E-11	9.91E-12	-	-0.22812	-0.259	+	A	G	0.517546468	0.0342744	+	A	G	0.5441	0.038
rs11158611	LC_IGP77	IgG1 Bn	14	66235074	1	1.62E-11	1.20E-11	-	-0.232486	-0.2592	+	A	G	0.517546468	0.0342633	+	A	G	0.5441	0.0382
rs11158611	LC_IGP86	IgG1 Bn/Fn total	14	66235074	1	3.29E-12	6.75E-12	-	-0.240199	-0.2624	+	A	G	0.517546468	0.0342226	+	A	G	0.5441	0.0382
rs11158611	LC_IGP84	IgG1 FBn/Bn total	14	66235074	1	8.79E-12	7.40E-16	+	0.235923	0.3107	+	A	G	0.517546468	0.0343126	+	A	G	0.5441	0.0385
rs11158611	LC_IGP62	IgG1 FG0n total/G0n	14	66235074	1	6.57E-14	1.89E-12	+	0.257314	0.2701	+	A	G	0.517546468	0.0340218	+	A	G	0.5441	0.0384
rs11158611	LC_IGP63	IgG1 FG1n total/G1n	14	66235074	1	5.97E-14	3.06E-14	+	0.258495	0.2909	+	A	G	0.517546468	0.0341202	+	A	G	0.5441	0.0383
rs11158611	LC_IGP64	IgG1 FG2n total/G2n	14	66235074	1	1.08E-10	3.56E-15	+	0.22296	0.2995	+	A	G	0.517546468	0.0343119	+	A	G	0.5441	0.0381
rs11158611	LC_IGP68	IgG1 FG2n/G2n	14	66235074	1	6.00E-09	6.68E-09	+	0.201094	0.2199	+	A	G	0.517546468	0.0343842	+	A	G	0.5441	0.0379

rs11158611	LC_IGP61	IgG1 Fn total	14	66235074	1	1.59E-13	4.52E-14	+	0.253933	0.2893	+	A	G	0.517546468	0.0341166	+	A	G	0.5441	0.0383
rs11158611	LC_IGP81	IgG1 Fn/Bn	14	66235074	1	4.99E-12	6.78E-11	+	0.238116	0.2484	+	A	G	0.517546468	0.0342199	+	A	G	0.5441	0.0381
rs11158611	LC_IGP21	IgG1 Fucosylation	14	66235074	1	2.00E-12	2.73E-13	+	0.241889	0.2798	+	A	G	0.516231196	0.0341185	+	A	G	0.5441	0.0383
rs11158611	LC_IGP28	IgG1 G1S1/(G1+G1S1)	14	66235074	1	3.16E-08	8.58E-10	+	0.18939	0.2257	+	A	G	0.516407951	0.0340664	+	A	G	0.5441	0.0368
rs11158611	LC_IGP11	IgG1_G0	14	66235074	1	2.91E-14	8.59E-15	-	-0.258949	-0.296	+	A	G	0.516693341	0.0337558	+	A	G	0.5441	0.0382
rs11158611	LC_IGP_R1	IgG1_G0F/IgG1_G0	14	66235074	1	1.89E-13	4.82E-12	+	0.250591	0.2642	+	A	G	0.516693341	0.0337787	+	A	G	0.5441	0.0382
rs11158611	LC_IGP_R4	IgG1_G0FN/IgG1_G0N	14	66235074	1	1.49E-11	1.18E-13	+	0.231304	0.2856	+	A	G	0.51703613	0.0340289	+	A	G	0.5441	0.0385
rs11158611	LC_IGP52	IgG1_G0n	14	66235074	1	2.92E-14	6.66E-15	-	-0.260683	-0.2978	+	A	G	0.517546468	0.0339805	+	A	G	0.5441	0.0382
rs11158611	LC_IGP14	IgG1_G0N	14	66235074	1	1.79E-14	2.99E-12	-	-0.2621	-0.2651	+	A	G	0.51703613	0.033883	+	A	G	0.5441	0.038
rs11158611	LC_IGP55	IgG1_G0Nn	14	66235074	1	3.04E-14	2.70E-12	-	-0.261244	-0.2659	+	A	G	0.517546468	0.0340761	+	A	G	0.5441	0.038
rs11158611	LC_IGP12	IgG1_G1	14	66235074	1	7.67E-13	1.72E-13	-	-0.244787	-0.2822	+	A	G	0.516693341	0.0338822	+	A	G	0.5441	0.0383
rs11158611	LC_IGP_R2	IgG1_G1F/IgG1_G1	14	66235074	1	4.06E-14	2.12E-13	+	0.25806	0.2806	+	A	G	0.516693341	0.0338363	+	A	G	0.5441	0.0382
rs11158611	LC_IGP_R5	IgG1_G1FN/IgG1_G1N	14	66235074	1	2.00E-11	8.08E-17	+	0.230861	0.3192	+	A	G	0.51803198	0.0341809	+	A	G	0.5441	0.0383
rs11158611	LC_IGP53	IgG1_G1n	14	66235074	1	4.53E-12	2.68E-13	-	-0.238337	-0.2798	+	A	G	0.517546468	0.0341836	+	A	G	0.5441	0.0383
rs11158611	LC_IGP15	IgG1_G1N	14	66235074	1	4.25E-10	1.07E-10	-	-0.215049	-0.2458	+	A	G	0.51803198	0.034226	+	A	G	0.5441	0.0381
rs11158611	LC_IGP56	IgG1_G1Nn	14	66235074	1	3.76E-10	2.12E-10	-	-0.216369	-0.2415	+	A	G	0.517546468	0.0343281	+	A	G	0.5441	0.038
rs11158611	LC_IGP_R3	IgG1_G2F/IgG1_G2	14	66235074	1	5.97E-12	8.06E-15	+	0.235465	0.295	+	A	G	0.516397922	0.0339694	+	A	G	0.5441	0.038
rs11158611	LC_IGP_R8	IgG1_G2FS1/IgG1_G2S1	14	66235074	1	2.75E-08	2.29E-12	+	0.190572	0.2658	+	A	G	0.516082468	0.034129	+	A	G	0.5441	0.0379
rs11158611	LC_IGP_R26	IgG1_G2N/IgG1_G1N	14	66235074	1	1.25E-08	7.05E-10	+	0.195683	0.2292	+	A	G	0.518098765	0.0341953	+	A	G	0.5441	0.0372
rs11158611	LC_IGP148	IgG2 FG0n total/G0n	14	66235074	1	4.19E-08	1.89E-12	+	0.188474	0.2672	+	A	G	0.515595443	0.0342129	+	A	G	0.5442	0.0379
rs11158611	LC_IGP97	IgG2_G0	14	66235074	1	4.30E-09	3.91E-13	-	-0.200835	-0.2758	+	A	G	0.516397922	0.0340161	+	A	G	0.5441	0.038
rs11158611	LC_IGP138	IgG2_G0n	14	66235074	1	2.11E-09	2.29E-13	-	-0.205524	-0.2789	+	A	G	0.515595443	0.0341197	+	A	G	0.5442	0.038
rs11628840	LC_IGP78	IgG1 BG0n/G0n	14	66235642	1	4.66E-12	1.95E-10	-	-0.238005	-0.2424	+	G	A	0.517552664	0.0341559	+	G	A	0.5456	0.0381
rs11628840	LC_IGP79	IgG1 BG1n/G1n	14	66235642	1	3.91E-11	9.22E-12	-	-0.228083	-0.2592	+	G	A	0.517552664	0.0342795	+	G	A	0.5456	0.038
rs11628840	LC_IGP77	IgG1 Bn	14	66235642	1	1.64E-11	1.16E-11	-	-0.232451	-0.2592	+	G	A	0.517552664	0.0342684	+	G	A	0.5456	0.0382
rs11628840	LC_IGP86	IgG1 Bn/Fn total	14	66235642	1	3.34E-12	6.40E-12	-	-0.240162	-0.2626	+	G	A	0.517552664	0.0342278	+	G	A	0.5456	0.0382
rs11628840	LC_IGP84	IgG1 FBn/Bn total	14	66235642	1	8.91E-12	6.55E-16	+	0.235888	0.3111	+	G	A	0.517552664	0.0343177	+	G	A	0.5456	0.0385
rs11628840	LC_IGP62	IgG1 FG0n total/G0n	14	66235642	1	6.72E-14	1.59E-12	+	0.257248	0.2709	+	G	A	0.517552664	0.0340271	+	G	A	0.5456	0.0383
rs11628840	LC_IGP63	IgG1 FG1n total/G1n	14	66235642	1	6.12E-14	2.74E-14	+	0.258427	0.2914	+	G	A	0.517552664	0.0341255	+	G	A	0.5456	0.0383
rs11628840	LC_IGP64	IgG1 FG2n total/G2n	14	66235642	1	1.11E-10	2.99E-15	+	0.222851	0.3002	+	G	A	0.517552664	0.0343173	+	G	A	0.5456	0.038
rs11628840	LC_IGP68	IgG1 FG2n/G2n	14	66235642	1	6.16E-09	5.49E-09	+	0.200972	0.221	+	G	A	0.517552664	0.0343896	+	G	A	0.5456	0.0379
rs11628840	LC_IGP61	IgG1 Fn total	14	66235642	1	1.64E-13	3.88E-14	+	0.253851	0.2899	+	G	A	0.517552664	0.034122	+	G	A	0.5456	0.0383
rs11628840	LC_IGP81	IgG1 Fn/Bn	14	66235642	1	5.06E-12	6.28E-11	+	0.238076	0.2487	+	G	A	0.517552664	0.0342251	+	G	A	0.5456	0.038
rs11628840	LC_IGP21	IgG1 Fucosylation	14	66235642	1	2.03E-12	2.46E-13	+	0.241847	0.2802	+	G	A	0.516239211	0.0341239	+	G	A	0.5456	0.0383
rs11628840	LC_IGP28	IgG1 G1S1/(G1+G1S1)	14	66235642	1	3.19E-08	6.93E-10	+	0.189352	0.2268	+	G	A	0.516415902	0.0340716	+	G	A	0.5456	0.0368
rs11628840	LC_IGP11	IgG1_G0	14	66235642	1	2.97E-14	8.34E-15	-	-0.258895	-0.296	+	G	A	0.516701283	0.0337612	+	G	A	0.5456	0.0381
rs11628840	LC_IGP_R1	IgG1_G0F/IgG1_G0	14	66235642	1	1.94E-13	3.95E-12	+	0.250515	0.2652	+	G	A	0.516701283	0.0337842	+	G	A	0.5456	0.0382
rs11628840	LC_IGP_R4	IgG1_G0FN/IgG1_G0N	14	66235642	1	1.51E-11	1.04E-13	+	0.231278	0.2861	+	G	A	0.517042254	0.0340339	+	G	A	0.5456	0.0385
rs11628840	LC_IGP52	IgG1_G0n	14	66235642	1	2.98E-14	6.40E-15	-	-0.26063	-0.2979	+	G	A	0.517552664	0.0339857	+	G	A	0.5456	0.0382
rs11628840	LC_IGP14	IgG1_G0N	14	66235642	1	1.81E-14	3.14E-12	-	-0.262092	-0.2647	+	G	A	0.517042254	0.0338879	+	G	A	0.5456	0.038
rs11628840	LC_IGP55	IgG1_G0Nn	14	66235642	1	3.07E-14	2.82E-12	-	-0.261234	-0.2656	+	G	A	0.517552664	0.0340812	+	G	A	0.5456	0.038
rs11628840	LC_IGP12	IgG1_G1	14	66235642	1	7.85E-13	1.55E-13	-	-0.244717	-0.2826	+	G	A	0.516701283	0.0338877	+	G	A	0.5456	0.0383
rs11628840	LC_IGP_R2	IgG1_G1F/IgG1_G1	14	66235642	1	4.16E-14	1.82E-13	+	0.257992	0.2812	+	G	A	0.516701283	0.0338417	+	G	A	0.5456	0.0382
rs11628840	LC_IGP_R5	IgG1_G1FN/IgG1_G1N	14	66235642	1	2.02E-11	7.61E-17	+	0.230836	0.3193	+	G	A	0.51803813	0.0341859	+	G	A	0.5456	0.0383
rs11628840	LC_IGP53	IgG1_G1n	14	66235642	1	4.64E-12	2.41E-13	-	-0.238257	-0.2802	+	G	A	0.517552664	0.0341889	+	G	A	0.5456	0.0383
rs11628840	LC_IGP15	IgG1_G1N	14	66235642	1	4.31E-10	1.00E-10	-	-0.215008	-0.2461	+	G	A	0.51803813	0.0342311	+	G	A	0.5456	0.038
rs11628840	LC_IGP56	IgG1_G1Nn	14	66235642	1	3.81E-10	1.98E-10	-	-0.216326	-0.2418	+	G	A	0.517552664	0.0343332	+	G	A	0.5456	0.038
rs11628840	LC_IGP_R3	IgG1_G2F/IgG1_G2	14	66235642	1	6.14E-12	6.50E-15	+	0.235362	0.2959	+	G	A	0.516405868	0.033975	+	G	A	0.5456	0.038
rs11628840	LC_IGP_R8	IgG1_G2FS1/IgG1_G2S1	14	66235642	1	2.80E-08	1.68E-12	+	0.190488	0.2673	+	G	A	0.516090409	0.0341344	+	G	A	0.5456	0.0379
rs11628840	LC_IGP_R26	IgG1_G2N/IgG1_G1N	14	66235642	1	1.25E-08	8.46E-10	+	0.195697	0.2281	+	G	A	0.518104938	0.0342002	+	G	A	0.5456	0.0372
rs11628840	LC_IGP148	IgG2 FG0n total/G0n	14	66235642	1	4.23E-08	2.50E-12	+	0.188443	0.2655	+	G	A	0.515603448	0.0342181	+	G	A	0.5457	0.0379
rs11628840	LC_IGP97	IgG2_G0	14	66235642	1	4.39E-09	5.54E-13	-	-0.20075	-0.2739	+	G	A	0.516405868	0.0340215	+	G	A	0.5456	0.038
rs11628840	LC_IGP138	IgG2_G0n	14	66235642	1	2.15E-09	3.21E-13	-	-0.205445	-0.2771	+	G	A	0.515603448	0.0341252	+	G	A	0.5457	0.038
rs4902415	LC_IGP78	IgG1 BG0n/G0n	14	66235690	1	4.67E-12	1.79E-10	-	-0.238003	-0.243	+	C	T	0.517552974	0.0341563	+	C	T	0.5454	0.0381
rs4902415	LC_IGP79	IgG1 BG1n/G1n	14	66235690	1	3.90E-11	8.67E-12	-	-0.228089	-0.2598	+	C	T	0.517552974	0.0342799	+	C	T	0.5454	0.038
rs4902415	LC_IGP77	IgG1 Bn	14	66235690	1	1.64E-11	1.08E-11	-	-0.232455	-0.2598	+	C	T	0.517552974	0.0342688	+	C	T	0.5454	0.0382
rs4902415	LC_IGP86	IgG1 Bn/Fn total	14	66235690	1	3.34E-12	5.97E-12	-	-0.240165	-0.2632	+	C	T	0.517552974	0.0342281	+	C	T	0.5454	0.0382
rs4902415	LC_IGP84	IgG1 FBn/Bn total	14	66235690	1	8.92E-12	6.20E-16	+	0.23589	0.3116	+	C	T	0.517552974	0.0343181	+	C	T	0.5454	0.0385
rs4902415	LC_IGP62	IgG1 FG0n total/G0n	14	66235690	1	6.74E-14	1.49E-12	+	0.257242	0.2714	+	C	T	0.517552974	0.0340275	+	C	T	0.5454	0.0384
rs4902415	LC_IGP63	IgG1 FG1n total/G1n	14	66235690	1	6.12E-14	2.62E-14	+	0.258428	0.2918	+	C	T	0.517552974	0.0341259	+	C	T	0.5454	0.0383
rs4902415	LC_IGP64	IgG1 FG2n total/G2n	14	66235690	1	1.11E-10	2.97E-15	+	0.222851	0.3004	+	C	T	0.517552974	0.0343177	+	C	T	0.5454	0.0381
rs4902415	LC_IGP68	IgG1 FG2n/G2n	14	66235690	1	6.16E-09	5.33E-09	+	0.200972	0.2214	+	C	T	0.517552974	0.03439	+	C	T	0.5454	0.0379
rs4902415	LC_IGP61	IgG1 Fn total	14	66235690	1	1.64E-13	3.70E-14	+	0.253848	0.2904	+	C	T	0.517552974	0.0341224	+	C	T	0.5454	0.0384
rs4902415	LC_IGP81	IgG1 Fn/Bn	14	66235690	1	5.06E-12	5.86E-11	+	0.238079	0.2493	+	C	T	0.517552974	0.0342254	+	C	T	0.5454	0.0381
rs4902415	LC_IGP21	IgG1 Fucosylation	14	66235690	1	2.03E-12	2.34E-13	+	0.241846	0.2807	+	C	T	0.516239827	0.0341243	+	C	T	0.5454	0.0383
rs4902415	LC_IGP28	IgG1 G1S1/(G1+G1S1)	14	66235690	1	3.19E-08	6.79E-10	+	0.189352	0.2271	+	C	T	0.516416514	0.034072	+	C	T	0.5454	0.0368
rs4902415	LC_IGP11	IgG1_G0	14	66235690	1	2.97E-14	8.14E-15	-	-0.258894	-0.2963	+	C	T	0.516701894	0.0337616	+	C	T	0.5454	0.0382
rs4902415	LC_IGP_R1	IgG1_G0F/IgG1_G0	14	66235690	1	1.95E-13	3.70E-12	+	0.250508	0.2657	+	C	T	0.516701894	0.0337846	+	C	T	0.5454	0.0382
rs4902415	LC_IGP_R4	IgG1_G0FN/IgG1_G0N	14	66235690	1	1.51E-11	9.62E-14	+	0.231276	0.2867	+	C	T	0.51704256	0.0340343	+	C	T	0.5454	0.0385
rs4902415	LC_IGP52	IgG1_G0n	14	66235690	1	2.99E-14	6.23E-15	-	-0.260628	-0.2982	+	C	T	0.517552974	0.0339861	+	C	T	0.5454	0.0382
rs4902415	LC_IGP14	IgG1_G0N	14	66235690	1	1.81E-14	2.97E-12	-	-0.262095	-0.2652	+	C	T	0.51704256	0.0338883	+	C	T	0.5454	0.038
rs4902415	LC_IGP55	IgG1_G0Nn	14	66235690	1	3.07E-14	2.66E-12	-	-0.261238	-0.266	+	C	T	0.517552974	0.0340815	+	C	T	0.5454	0.038
rs4902415	LC_IGP12	IgG1_G1	14	66235690	1	7.86E-13	1.49E-13	-	-0.244716	-0.2831	+	C	T	0.516701894	0.0338881	+	C	T	0.5454	0.0383
rs4902415	LC_IGP_R2	IgG1_G1F/IgG1_G1	14	66235690	1	4.16E-14	1.74E-13	+	0.257993	0.2816	+	C	T	0.516701894	0.0338421	+	C	T	0.5454	0.0382
rs4902415	LC_IGP_R5	IgG1_G1FN/IgG1_G1N	14	66235690	1	2.02E-11	7.31E-17	+	0.23084	0.3197	+	C	T	0.518038438	0.0341863	+	C	T	0.5454	0.0383
rs4902415	LC_IGP53	IgG1_G1n	14	66235690	1	4.65E-12	2.30E-13	-	-0.238254	-0.2806	+	C	T	0.517552974	0.0341893	+	C	T	0.5454	0.0383
rs4902415	LC_IGP15	IgG1_G1N	14	66235690	1	4.31E-10	9.39E-11	-	-0.215011	-0.2466	+	C	T	0.518038438	0.0342315	+	C	T	0.5454	0.0381
rs4902415	LC_IGP56	IgG1_G1Nn	14	66235690	1	3.81E-10	1.85E-10	-	-0.216329	-0.2423	+	C	T	0.517552974	0.0343336	+	C	T	0.5454	0.038
rs4902415	LC_IGP_R3	IgG1_G2F/IgG1_G2	14	66235690	1	6.14E-12	6.44E-15	+	0.235362	0.2962	+	C	T	0.516406479	0.0339754	+	C	T	0.5454	0.038
rs4902415	LC_IGP_R8	IgG1_G2FS1/IgG1_G2S1	14	66235690	1	2.80E-08	1.66E-12	+	0.190493	0.2675	+	C	T	0.51609102	0.0341348	+	C	T	0.5454	0.0379
rs4902415	LC_IGP_R26	IgG1_G2N/IgG1_G1N	14	66235690	1	1.25E-08	8.15E-10	+	0.195694	0.2284	+	C	T	0.518105247	0.0342006	+	C	T	0.5454	0.0372
rs4902415	LC_IGP148	IgG2 FG0n total/G0n	14	66235690	1	4.24E-08	2.25E-12	+	0.188443	0.2662	+	C	T	0.515604064	0.0342185	+	C	T	0.5455	0.0379
rs4902415	LC_IGP97	IgG2_G0	14	66235690	1	4.40E-09	5.09E-13	-	-0.200745	-0.2745	+	C	T	0.516406479	0.0340219	+	C	T	0.5454	0.038
rs4902415	LC_IGP138	IgG2_G0n	14	66235690	1	2.15E-09	2.94E-13	-	-0.205441	-0.2777	+	C	T	0.515604064	0.0341256	+	C	T	0.5455	0.038
rs1003401	LC_IGP78	IgG1 BG0n/G0n	14	66237720	1	1.14E-12	5.36E-07	-	-0.250492	-0.1966	+	A	G	0.59692627	0.0349403	+	A	G	0.6217	0.0392
rs1003401	LC_IGP79	IgG1 BG1n/G1n	14	66237720	1	2.62E-11	6.40E-07	-	-0.235565	-0.1949	+	A	G	0.59692627	0.0350882	+	A	G	0.6217	0.0392
rs1003401	LC_IGP77	IgG1 Bn	14	66237720	1	5.45E-12	2.51E-07	-	-0.243521	-0.2029	+	A	G	0.59692627	0.0350618	+	A	G	0.6217	0.0393
rs1003401	LC_IGP86	IgG1 Bn/Fn total	14	66237720	1	1.06E-12	1.67E-07	-	-0.25144	-0.2059	+	A	G	0.59692627	0.0350192	+	A	G	0.6217	0.0394
rs1003401	LC_IGP84	IgG1 FBn/Bn total	14	66237720	1	1.25E-12	2.12E-10	+	0.25114	0.2518	+	A	G	0.59692627	0.0350939	+	A	G	0.6217	0.0396
rs1003401	LC_IGP62	IgG1 FG0n total/G0n	14	66237720	1	1.57E-15	8.34E-09	+	0.279877	0.2274	+	A	G	0.59692627	0.0347584	+	A	G	0.6217	0.0395
rs1003401	LC_IGP63	IgG1 FG1n total/G1n	14	66237720	1	8.68E-14	8.57E-09	+	0.262993	0.2268	+	A	G	0.59692627	0.0349465	+	A	G	0.6217	0.0394
rs1003401	LC_IGP64	IgG1 FG2n total/G2n	14	66237720	1	7.67E-11	6.16E-10	+	0.230119	0.242	+	A	G	0.59692627	0.0351275	+	A	G	0.6217	0.0391
rs1003401	LC_IGP61	IgG1 Fn total	14	66237720	1	4.45E-14	2.74E-09	+	0.265837	0.2346	+	A	G	0.59692627	0.0349077	+	A	G	0.6217	0.0394
rs1003401	LC_IGP81	IgG1 Fn/Bn	14	66237720	1	2.40E-12	7.29E-07	+	0.247415	0.1941	+	A	G	0.59692627	0.035025	+	A	G	0.6217	0.0392
rs1003401	LC_IGP21	IgG1 Fucosylation	14	66237720	1	1.26E-12	4.70E-09	+	0.249159	0.2307	+	A	G	0.595844328	0.034822	+	A	G	0.6217	0.0394
rs1003401	LC_IGP11	IgG1_G0	14	66237720	1	8.55E-16	3.73E-11	-	-0.279815	-0.2598	+	A	G	0.59649328	0.0344276	+	A	G	0.6217	0.0393
rs1003401	LC_IGP_R1	IgG1_G0F/IgG1_G0	14	66237720	1	4.75E-15	1.76E-08	+	0.272439	0.2217	+	A	G	0.59649328	0.0344477	+	A	G	0.6217	0.0394
rs1003401	LC_IGP_R4	IgG1_G0FN/IgG1_G0N	14	66237720	1	3.98E-12	1.26E-09	+	0.243106	0.2404	+	A	G	0.596717085	0.0347768	+	A	G	0.6217	0.0396
rs1003401	LC_IGP52	IgG1_G0n	14	66237720	1	5.07E-16	3.71E-11	-	-0.284427	-0.2603	+	A	G	0.59692627	0.0347094	+	A	G	0.6217	0.0394
rs1003401	LC_IGP14	IgG1_G0N	14	66237720	1	1.19E-14	1.43E-08	-	-0.269871	-0.2217	+	A	G	0.596717085	0.0346466	+	A	G	0.6217	0.0391
rs1003401	LC_IGP55	IgG1_G0Nn	14	66237720	1	1.55E-14	1.68E-08	-	-0.270504	-0.2209	+	A	G	0.59692627	0.0348791	+	A	G	0.6217	0.0392
rs1003401	LC_IGP12	IgG1_G1	14	66237720	1	3.71E-13	8.80E-09	-	-0.253685	-0.2267	+	A	G	0.596187844	0.0346279	+	A	G	0.6217	0.0394
rs1003401	LC_IGP_R2	IgG1_G1F/IgG1_G1	14	66237720	1	5.18E-14	2.27E-08	+	0.262769	0.2198	+	A	G	0.596187844	0.0346012	+	A	G	0.6217	0.0393
rs1003401	LC_IGP_R5	IgG1_G1FN/IgG1_G1N	14	66237720	1	2.38E-11	2.13E-10	+	0.235227	0.2504	+	A	G	0.597748462	0.0349628	+	A	G	0.6217	0.0394
rs1003401	LC_IGP53	IgG1_G1n	14	66237720	1	4.02E-12	1.43E-08	-	-0.244646	-0.2233	+	A	G	0.59692627	0.0350008	+	A	G	0.6217	0.0394
rs1003401	LC_IGP15	IgG1_G1N	14	66237720	1	4.74E-10	1.48E-06	-	-0.219352	-0.1886	+	A	G	0.597748462	0.0350076	+	A	G	0.6217	0.0392
rs1003401	LC_IGP_R3	IgG1_G2F/IgG1_G2	14	66237720	1	8.58E-12	8.41E-10	+	0.238834	0.2397	+	A	G	0.596246638	0.0347214	+	A	G	0.6217	0.0391
rs1003401	LC_IGP_R8	IgG1_G2FS1/IgG1_G2S1	14	66237720	1	6.66E-09	8.33E-09	+	0.203113	0.2244	+	A	G	0.595882407	0.034839	+	A	G	0.6217	0.039
rs4902416	LC_IGP78	IgG1 BG0n/G0n	14	66238090	1	4.41E-12	2.61E-10	-	-0.238718	-0.241	+	C	G	0.51688165	0.0342185	+	C	G	0.5447	0.0381
rs4902416	LC_IGP79	IgG1 BG1n/G1n	14	66238090	1	4.67E-11	1.23E-11	-	-0.2276	-0.2582	+	C	G	0.51688165	0.0343472	+	C	G	0.5447	0.0381
rs4902416	LC_IGP77	IgG1 Bn	14	66238090	1	1.75E-11	1.56E-11	-	-0.232566	-0.258	+	C	G	0.51688165	0.0343337	+	C	G	0.5447	0.0383
rs4902416	LC_IGP86	IgG1 Bn/Fn total	14	66238090	1	3.59E-12	8.79E-12	-	-0.240263	-0.2613	+	C	G	0.51688165	0.0342931	+	C	G	0.5447	0.0383
rs4902416	LC_IGP84	IgG1 FBn/Bn total	14	66238090	1	8.27E-12	8.99E-16	+	0.236693	0.31	+	C	G	0.51688165	0.0343802	+	C	G	0.5447	0.0386
rs4902416	LC_IGP62	IgG1 FG0n total/G0n	14	66238090	1	6.54E-14	2.34E-12	+	0.257851	0.2692	+	C	G	0.51688165	0.03409	+	C	G	0.5447	0.0384
rs4902416	LC_IGP63	IgG1 FG1n total/G1n	14	66238090	1	6.64E-14	4.09E-14	+	0.258542	0.2899	+	C	G	0.51688165	0.0341909	+	C	G	0.5447	0.0384
rs4902416	LC_IGP64	IgG1 FG2n total/G2n	14	66238090	1	1.23E-10	4.22E-15	+	0.22276	0.2993	+	C	G	0.51688165	0.0343834	+	C	G	0.5447	0.0381
rs4902416	LC_IGP68	IgG1 FG2n/G2n	14	66238090	1	7.83E-09	7.08E-09	+	0.199962	0.2201	+	C	G	0.51688165	0.0344588	+	C	G	0.5447	0.038
rs4902416	LC_IGP61	IgG1 Fn total	14	66238090	1	1.75E-13	5.76E-14	+	0.254024	0.2885	+	C	G	0.51688165	0.0341871	+	C	G	0.5447	0.0384
rs4902416	LC_IGP81	IgG1 Fn/Bn	14	66238090	1	5.59E-12	8.28E-11	+	0.238042	0.2476	+	C	G	0.51688165	0.034291	+	C	G	0.5447	0.0381

rs4902416	LC_IGP21	IgG1 Fucosylation	14	66238090	1	2.17E-12	3.28E-13	+	0.241994	0.2794	+	C	G	0.515579214	0.03419	+	C	G	0.5447	0.0384
rs4902416	LC_IGP28	IgG1 G1S1/(G1+G1S1)	14	66238090	1	2.38E-08	7.37E-10	+	0.191455	0.2272	+	C	G	0.515761152	0.0341296	+	C	G	0.5447	0.0369
rs4902416	LC_IGP11	IgG1_G0	14	66238090	1	2.46E-14	1.24E-14	-	-0.260195	-0.2948	+	C	G	0.516047333	0.0338206	+	C	G	0.5447	0.0382
rs4902416	LC_IGP_R1	IgG1_G0F/IgG1_G0	14	66238090	1	1.98E-13	5.87E-12	+	0.250885	0.2635	+	C	G	0.516047333	0.0338479	+	C	G	0.5447	0.0383
rs4902416	LC_IGP_R4	IgG1_G0FN/IgG1_G0N	14	66238090	1	1.26E-11	1.43E-13	+	0.2326	0.2848	+	C	G	0.516379047	0.034093	+	C	G	0.5447	0.0385
rs4902416	LC_IGP52	IgG1_G0n	14	66238090	1	2.48E-14	9.58E-15	-	-0.261915	-0.2967	+	C	G	0.51688165	0.0340453	+	C	G	0.5447	0.0383
rs4902416	LC_IGP14	IgG1_G0N	14	66238090	1	1.43E-14	4.13E-12	-	-0.263585	-0.2637	+	C	G	0.516379047	0.0339457	+	C	G	0.5447	0.038
rs4902416	LC_IGP55	IgG1_G0Nn	14	66238090	1	2.44E-14	3.72E-12	-	-0.262717	-0.2646	+	C	G	0.51688165	0.03414	+	C	G	0.5447	0.0381
rs4902416	LC_IGP12	IgG1_G1	14	66238090	1	8.25E-13	2.36E-13	-	-0.244945	-0.281	+	C	G	0.516047333	0.0339521	+	C	G	0.5447	0.0384
rs4902416	LC_IGP_R2	IgG1_G1F/IgG1_G1	14	66238090	1	4.41E-14	2.79E-13	+	0.258216	0.2796	+	C	G	0.516047333	0.0339063	+	C	G	0.5447	0.0383
rs4902416	LC_IGP_R5	IgG1_G1FN/IgG1_G1N	14	66238090	1	2.16E-11	9.98E-17	+	0.230926	0.3186	+	C	G	0.517372068	0.0342506	+	C	G	0.5447	0.0384
rs4902416	LC_IGP53	IgG1_G1n	14	66238090	1	4.98E-12	3.61E-13	-	-0.238359	-0.2786	+	C	G	0.51688165	0.0342541	+	C	G	0.5447	0.0383
rs4902416	LC_IGP15	IgG1_G1N	14	66238090	1	5.20E-10	1.33E-10	-	-0.214403	-0.2449	+	C	G	0.517372068	0.0342983	+	C	G	0.5447	0.0381
rs4902416	LC_IGP56	IgG1_G1Nn	14	66238090	1	4.66E-10	2.60E-10	-	-0.215661	-0.2406	+	C	G	0.51688165	0.0344014	+	C	G	0.5447	0.0381
rs4902416	LC_IGP_R32	IgG1_G1S1/IgG1_G1	14	66238090	1	3.75E-08	1.08E-09	+	0.188763	0.2252	+	C	G	0.515761152	0.034144	+	C	G	0.5447	0.0369
rs4902416	LC_IGP_R3	IgG1_G2F/IgG1_G2	14	66238090	1	6.83E-12	9.31E-15	+	0.235291	0.295	+	C	G	0.515751518	0.0340409	+	C	G	0.5447	0.0381
rs4902416	LC_IGP_R8	IgG1_G2FS1/IgG1_G2S1	14	66238090	1	3.31E-08	2.36E-12	+	0.18986	0.2661	+	C	G	0.51543646	0.0342018	+	C	G	0.5447	0.038
rs4902416	LC_IGP_R26	IgG1_G2N/IgG1_G1N	14	66238090	1	1.29E-08	9.03E-10	+	0.195883	0.2282	+	C	G	0.517436409	0.0342643	+	C	G	0.5447	0.0373
rs4902416	LC_IGP97	IgG2_G0	14	66238090	1	7.55E-09	7.52E-13	-	-0.19806	-0.2732	+	C	G	0.515751518	0.0340963	+	C	G	0.5447	0.0381
rs4902416	LC_IGP138	IgG2_G0n	14	66238090	1	3.71E-09	4.33E-13	-	-0.202795	-0.2765	+	C	G	0.514944264	0.0342011	+	C	G	0.5448	0.0382
rs7156078	LC_IGP78	IgG1 BG0n/G0n	14	66238701	1	1.15E-12	6.76E-07	-	-0.250545	-0.195	+	A	G	0.596919145	0.0349487	+	A	G	0.6211	0.0392
rs7156078	LC_IGP79	IgG1 BG1n/G1n	14	66238701	1	2.62E-11	8.30E-07	-	-0.23564	-0.1932	+	A	G	0.596919145	0.0350966	+	A	G	0.6211	0.0392
rs7156078	LC_IGP77	IgG1 Bn	14	66238701	1	5.45E-12	3.23E-07	-	-0.24358	-0.2012	+	A	G	0.596919145	0.0350702	+	A	G	0.6211	0.0394
rs7156078	LC_IGP86	IgG1 Bn/Fn total	14	66238701	1	1.06E-12	2.19E-07	-	-0.251496	-0.2041	+	A	G	0.596919145	0.0350277	+	A	G	0.6211	0.0394
rs7156078	LC_IGP84	IgG1 FBn/Bn total	14	66238701	1	1.26E-12	2.94E-10	+	0.251173	0.2499	+	A	G	0.596919145	0.0351024	+	A	G	0.6211	0.0396
rs7156078	LC_IGP62	IgG1 FG0n total/G0n	14	66238701	1	1.60E-15	1.18E-08	+	0.279853	0.2252	+	A	G	0.596919145	0.0347672	+	A	G	0.6211	0.0395
rs7156078	LC_IGP63	IgG1 FG1n total/G1n	14	66238701	1	8.74E-14	1.25E-08	+	0.263023	0.2245	+	A	G	0.596919145	0.034955	+	A	G	0.6211	0.0394
rs7156078	LC_IGP64	IgG1 FG2n total/G2n	14	66238701	1	7.75E-11	8.69E-10	+	0.230121	0.2403	+	A	G	0.596919145	0.0351361	+	A	G	0.6211	0.0392
rs7156078	LC_IGP61	IgG1 Fn total	14	66238701	1	4.52E-14	3.98E-09	+	0.265827	0.2324	+	A	G	0.596919145	0.0349164	+	A	G	0.6211	0.0395
rs7156078	LC_IGP81	IgG1 Fn/Bn	14	66238701	1	2.40E-12	9.20E-07	+	0.247479	0.1926	+	A	G	0.596919145	0.0350334	+	A	G	0.6211	0.0392
rs7156078	LC_IGP21	IgG1 Fucosylation	14	66238701	1	1.26E-12	6.26E-09	+	0.249215	0.2291	+	A	G	0.595840629	0.0348308	+	A	G	0.6211	0.0394
rs7156078	LC_IGP11	IgG1_G0	14	66238701	1	8.75E-16	5.14E-11	-	-0.27979	-0.2582	+	A	G	0.596489615	0.0344367	+	A	G	0.6211	0.0393
rs7156078	LC_IGP_R1	IgG1_G0F/IgG1_G0	14	66238701	1	4.86E-15	2.52E-08	+	0.272403	0.2194	+	A	G	0.596489615	0.0344569	+	A	G	0.6211	0.0394
rs7156078	LC_IGP_R4	IgG1_G0FN/IgG1_G0N	14	66238701	1	4.02E-12	1.68E-09	+	0.243118	0.2387	+	A	G	0.596710043	0.0347852	+	A	G	0.6211	0.0396
rs7156078	LC_IGP52	IgG1_G0n	14	66238701	1	5.18E-16	5.17E-11	-	-0.284406	-0.2587	+	A	G	0.596919145	0.0347181	+	A	G	0.6211	0.0394
rs7156078	LC_IGP14	IgG1_G0N	14	66238701	1	1.19E-14	1.67E-08	-	-0.269941	-0.2209	+	A	G	0.596710043	0.0346548	+	A	G	0.6211	0.0392
rs7156078	LC_IGP55	IgG1_G0Nn	14	66238701	1	1.54E-14	1.99E-08	-	-0.270585	-0.22	+	A	G	0.596919145	0.0348874	+	A	G	0.6211	0.0392
rs7156078	LC_IGP12	IgG1_G1	14	66238701	1	3.77E-13	1.31E-08	-	-0.253666	-0.2242	+	A	G	0.596184178	0.034637	+	A	G	0.6211	0.0394
rs7156078	LC_IGP_R2	IgG1_G1F/IgG1_G1	14	66238701	1	5.21E-14	3.36E-08	+	0.262809	0.2174	+	A	G	0.596184178	0.03461	+	A	G	0.6211	0.0394
rs7156078	LC_IGP_R5	IgG1_G1FN/IgG1_G1N	14	66238701	1	2.38E-11	2.93E-10	+	0.235276	0.2487	+	A	G	0.59774139	0.0349711	+	A	G	0.6211	0.0395
rs7156078	LC_IGP53	IgG1_G1n	14	66238701	1	4.09E-12	2.12E-08	-	-0.244622	-0.2208	+	A	G	0.596919145	0.0350096	+	A	G	0.6211	0.0394
rs7156078	LC_IGP15	IgG1_G1N	14	66238701	1	4.75E-10	1.93E-06	-	-0.219394	-0.1868	+	A	G	0.59774139	0.035016	+	A	G	0.6211	0.0392
rs7156078	LC_IGP_R3	IgG1_G2F/IgG1_G2	14	66238701	1	8.67E-12	1.20E-09	+	0.23884	0.2379	+	A	G	0.596242971	0.0347303	+	A	G	0.6211	0.0391
rs7156078	LC_IGP_R8	IgG1_G2FS1/IgG1_G2S1	14	66238701	1	6.62E-09	1.15E-08	+	0.203203	0.2226	+	A	G	0.595878742	0.0348476	+	A	G	0.6211	0.039
rs12894034	LC_IGP78	IgG1 BG0n/G0n	14	66238763	1	5.34E-12	2.54E-10	-	-0.237987	-0.2412	+	C	T	0.517173792	0.0342508	+	C	T	0.5448	0.0381
rs12894034	LC_IGP79	IgG1 BG1n/G1n	14	66238763	1	4.99E-11	1.23E-11	-	-0.22746	-0.2583	+	C	T	0.517173792	0.034377	+	C	T	0.5448	0.0381
rs12894034	LC_IGP77	IgG1 Bn	14	66238763	1	2.02E-11	1.53E-11	-	-0.232043	-0.2582	+	C	T	0.517173792	0.0343651	+	C	T	0.5448	0.0383
rs12894034	LC_IGP86	IgG1 Bn/Fn total	14	66238763	1	4.14E-12	8.65E-12	-	-0.239776	-0.2615	+	C	T	0.517173792	0.0343245	+	C	T	0.5448	0.0383
rs12894034	LC_IGP84	IgG1 FBn/Bn total	14	66238763	1	9.02E-12	8.74E-16	+	0.236464	0.3103	+	C	T	0.517173792	0.0344105	+	C	T	0.5448	0.0386
rs12894034	LC_IGP62	IgG1 FG0n total/G0n	14	66238763	1	7.60E-14	2.32E-12	+	0.257396	0.2694	+	C	T	0.517173792	0.0341214	+	C	T	0.5448	0.0384
rs12894034	LC_IGP63	IgG1 FG1n total/G1n	14	66238763	1	6.76E-14	4.17E-14	+	0.258676	0.2899	+	C	T	0.517173792	0.0342195	+	C	T	0.5448	0.0384
rs12894034	LC_IGP64	IgG1 FG2n total/G2n	14	66238763	1	1.31E-10	4.27E-15	+	0.222581	0.2994	+	C	T	0.517173792	0.0344133	+	C	T	0.5448	0.0382
rs12894034	LC_IGP68	IgG1 FG2n/G2n	14	66238763	1	8.46E-09	7.06E-09	+	0.199678	0.2202	+	C	T	0.517173792	0.0344889	+	C	T	0.5448	0.038
rs12894034	LC_IGP61	IgG1 Fn total	14	66238763	1	1.87E-13	5.77E-14	+	0.253919	0.2886	+	C	T	0.517173792	0.0342168	+	C	T	0.5448	0.0384
rs12894034	LC_IGP81	IgG1 Fn/Bn	14	66238763	1	6.49E-12	8.18E-11	+	0.237517	0.2477	+	C	T	0.517173792	0.0343225	+	C	T	0.5448	0.0381
rs12894034	LC_IGP21	IgG1 Fucosylation	14	66238763	1	2.20E-12	3.23E-13	+	0.242126	0.2796	+	C	T	0.515872072	0.0342188	+	C	T	0.5448	0.0384
rs12894034	LC_IGP28	IgG1 G1S1/(G1+G1S1)	14	66238763	1	3.36E-08	7.85E-10	+	0.189563	0.2269	+	C	T	0.516051682	0.0341646	+	C	T	0.5448	0.0369
rs12894034	LC_IGP11	IgG1_G0	14	66238763	1	2.97E-14	1.26E-14	-	-0.259595	-0.2948	+	C	T	0.516337508	0.0338522	+	C	T	0.5448	0.0382
rs12894034	LC_IGP_R1	IgG1_G0F/IgG1_G0	14	66238763	1	2.25E-13	5.85E-12	+	0.250517	0.2636	+	C	T	0.516337508	0.0338784	+	C	T	0.5448	0.0383
rs12894034	LC_IGP_R4	IgG1_G0FN/IgG1_G0N	14	66238763	1	1.41E-11	1.38E-13	+	0.232225	0.2851	+	C	T	0.516667789	0.0341233	+	C	T	0.5448	0.0385
rs12894034	LC_IGP52	IgG1_G0n	14	66238763	1	2.94E-14	9.68E-15	-	-0.261399	-0.2967	+	C	T	0.517173792	0.034077	+	C	T	0.5448	0.0383
rs12894034	LC_IGP14	IgG1_G0N	14	66238763	1	1.88E-14	4.04E-12	-	-0.26263	-0.2639	+	C	T	0.516667789	0.0339791	+	C	T	0.5448	0.038
rs12894034	LC_IGP55	IgG1_G0Nn	14	66238763	1	3.16E-14	3.64E-12	-	-0.261807	-0.2648	+	C	T	0.517173792	0.0341736	+	C	T	0.5448	0.0381
rs12894034	LC_IGP12	IgG1_G1	14	66238763	1	8.51E-13	2.39E-13	-	-0.245004	-0.281	+	C	T	0.516337508	0.0339807	+	C	T	0.5448	0.0384
rs12894034	LC_IGP_R2	IgG1_G1F/IgG1_G1	14	66238763	1	4.51E-14	2.85E-13	+	0.258339	0.2795	+	C	T	0.516337508	0.0339346	+	C	T	0.5448	0.0383
rs12894034	LC_IGP_R5	IgG1_G1FN/IgG1_G1N	14	66238763	1	2.16E-11	1.00E-16	+	0.231108	0.3187	+	C	T	0.517662054	0.0342788	+	C	T	0.5448	0.0384
rs12894034	LC_IGP53	IgG1_G1n	14	66238763	1	4.96E-12	3.66E-13	-	-0.238575	-0.2786	+	C	T	0.517173792	0.0342824	+	C	T	0.5448	0.0383
rs12894034	LC_IGP15	IgG1_G1N	14	66238763	1	5.61E-10	1.32E-10	-	-0.214169	-0.245	+	C	T	0.517662054	0.0343281	+	C	T	0.5448	0.0381
rs12894034	LC_IGP56	IgG1_G1Nn	14	66238763	1	4.96E-10	2.58E-10	-	-0.215505	-0.2407	+	C	T	0.517173792	0.0344312	+	C	T	0.5448	0.0381
rs12894034	LC_IGP_R3	IgG1_G2F/IgG1_G2	14	66238763	1	7.02E-12	9.42E-15	+	0.235348	0.295	+	C	T	0.51604187	0.0340695	+	C	T	0.5448	0.0381
rs12894034	LC_IGP_R8	IgG1_G2FS1/IgG1_G2S1	14	66238763	1	2.31E-08	2.34E-12	+	0.192144	0.2662	+	C	T	0.515726634	0.0342226	+	C	T	0.5448	0.038
rs12894034	LC_IGP_R26	IgG1_G2N/IgG1_G1N	14	66238763	1	1.22E-08	9.19E-10	+	0.196393	0.2282	+	C	T	0.517727469	0.0342913	+	C	T	0.5448	0.0373
rs12894034	LC_IGP97	IgG2_G0	14	66238763	1	7.20E-09	7.70E-13	-	-0.198489	-0.2732	+	C	T	0.51604187	0.0341234	+	C	T	0.5448	0.0381
rs12894034	LC_IGP138	IgG2_G0n	14	66238763	1	3.54E-09	4.47E-13	-	-0.203219	-0.2764	+	C	T	0.515236761	0.0342284	+	C	T	0.5449	0.0382
rs1984855	LC_IGP78	IgG1 BG0n/G0n	14	66239257	1	1.08E-12	6.19E-07	-	-0.25086	-0.1963	+	C	G	0.596324659	0.0349496	+	C	G	0.6195	0.0394
rs1984855	LC_IGP79	IgG1 BG1n/G1n	14	66239257	1	2.58E-11	7.77E-07	-	-0.235725	-0.1943	+	C	G	0.596324659	0.0350986	+	C	G	0.6195	0.0393
rs1984855	LC_IGP77	IgG1 Bn	14	66239257	1	4.96E-12	2.98E-07	-	-0.244066	-0.2025	+	C	G	0.596324659	0.0350705	+	C	G	0.6195	0.0395
rs1984855	LC_IGP86	IgG1 Bn/Fn total	14	66239257	1	9.55E-13	2.06E-07	-	-0.252013	-0.2053	+	C	G	0.596324659	0.0350277	+	C	G	0.6195	0.0395
rs1984855	LC_IGP84	IgG1 FBn/Bn total	14	66239257	1	1.13E-12	2.95E-10	+	0.251717	0.2508	+	C	G	0.596324659	0.0351024	+	C	G	0.6195	0.0398
rs1984855	LC_IGP62	IgG1 FG0n total/G0n	14	66239257	1	1.43E-15	1.14E-08	+	0.280339	0.2262	+	C	G	0.596324659	0.0347671	+	C	G	0.6195	0.0396
rs1984855	LC_IGP63	IgG1 FG1n total/G1n	14	66239257	1	7.98E-14	1.27E-08	+	0.263457	0.2252	+	C	G	0.596324659	0.0349553	+	C	G	0.6195	0.0396
rs1984855	LC_IGP64	IgG1 FG2n total/G2n	14	66239257	1	5.94E-11	9.30E-10	+	0.231523	0.2407	+	C	G	0.596324659	0.0351328	+	C	G	0.6195	0.0393
rs1984855	LC_IGP61	IgG1 Fn total	14	66239257	1	3.86E-14	4.05E-09	+	0.266565	0.2331	+	C	G	0.596324659	0.0349153	+	C	G	0.6195	0.0396
rs1984855	LC_IGP81	IgG1 Fn/Bn	14	66239257	1	2.18E-12	8.27E-07	+	0.247961	0.194	+	C	G	0.596324659	0.0350337	+	C	G	0.6195	0.0394
rs1984855	LC_IGP21	IgG1 Fucosylation	14	66239257	1	1.08E-12	6.39E-09	+	0.250001	0.2298	+	C	G	0.595250617	0.0348301	+	C	G	0.6195	0.0396
rs1984855	LC_IGP11	IgG1_G0	14	66239257	1	8.85E-16	5.42E-11	-	-0.279762	-0.2589	+	C	G	0.595905009	0.0344395	+	C	G	0.6195	0.0395
rs1984855	LC_IGP_R1	IgG1_G0F/IgG1_G0	14	66239257	1	4.39E-15	2.44E-08	+	0.272859	0.2204	+	C	G	0.595905009	0.0344573	+	C	G	0.6195	0.0395
rs1984855	LC_IGP_R4	IgG1_G0FN/IgG1_G0N	14	66239257	1	3.65E-12	1.62E-09	+	0.243599	0.2399	+	C	G	0.596122474	0.0347855	+	C	G	0.6195	0.0398
rs1984855	LC_IGP52	IgG1_G0n	14	66239257	1	5.16E-16	5.45E-11	-	-0.284437	-0.2593	+	C	G	0.596324659	0.0347204	+	C	G	0.6195	0.0396
rs1984855	LC_IGP14	IgG1_G0N	14	66239257	1	1.26E-14	1.55E-08	-	-0.26969	-0.2222	+	C	G	0.596122474	0.0346583	+	C	G	0.6195	0.0393
rs1984855	LC_IGP55	IgG1_G0Nn	14	66239257	1	1.62E-14	1.85E-08	-	-0.270397	-0.2212	+	C	G	0.596324659	0.0348906	+	C	G	0.6195	0.0393
rs1984855	LC_IGP12	IgG1_G1	14	66239257	1	3.27E-13	1.32E-08	-	-0.254344	-0.225	+	C	G	0.595599572	0.0346366	+	C	G	0.6195	0.0396
rs1984855	LC_IGP_R2	IgG1_G1F/IgG1_G1	14	66239257	1	4.68E-14	3.43E-08	+	0.263306	0.218	+	C	G	0.595599572	0.0346103	+	C	G	0.6195	0.0395
rs1984855	LC_IGP_R5	IgG1_G1FN/IgG1_G1N	14	66239257	1	2.42E-11	2.90E-10	+	0.235216	0.2497	+	C	G	0.597151292	0.0349736	+	C	G	0.6195	0.0396
rs1984855	LC_IGP53	IgG1_G1n	14	66239257	1	3.47E-12	2.13E-08	-	-0.245439	-0.2215	+	C	G	0.596324659	0.0350084	+	C	G	0.6195	0.0396
rs1984855	LC_IGP15	IgG1_G1N	14	66239257	1	4.52E-10	1.73E-06	-	-0.219679	-0.1882	+	C	G	0.597151292	0.035017	+	C	G	0.6195	0.0394
rs1984855	LC_IGP_R3	IgG1_G2F/IgG1_G2	14	66239257	1	6.63E-12	1.25E-09	+	0.240184	0.2384	+	C	G	0.595658007	0.0347273	+	C	G	0.6195	0.0392
rs1984855	LC_IGP_R8	IgG1_G2FS1/IgG1_G2S1	14	66239257	1	5.14E-09	1.25E-08	+	0.20468	0.2228	+	C	G	0.595294136	0.034845	+	C	G	0.6195	0.0391
rs730807	LC_IGP78	IgG1 BG0n/G0n	14	66239290	1	4.18E-12	2.33E-10	-	-0.239111	-0.2424	+	T	C	0.516275103	0.0342363	+	T	C	0.5433	0.0382
rs730807	LC_IGP79	IgG1 BG1n/G1n	14	66239290	1	4.62E-11	1.15E-11	-	-0.227786	-0.2594	+	T	C	0.516275103	0.034366	+	T	C	0.5433	0.0382
rs730807	LC_IGP77	IgG1 Bn	14	66239290	1	1.60E-11	1.41E-11	-	-0.233149	-0.2594	+	T	C	0.516275103	0.0343508	+	T	C	0.5433	0.0384
rs730807	LC_IGP86	IgG1 Bn/Fn total	14	66239290	1	3.25E-12	8.13E-12	-	-0.240869	-0.2626	+	T	C	0.516275103	0.0343101	+	T	C	0.5433	0.0384
rs730807	LC_IGP84	IgG1 FBn/Bn total	14	66239290	1	7.55E-12	8.98E-16	+	0.237272	0.3112	+	T	C	0.516275103	0.0343973	+	T	C	0.5433	0.0387
rs730807	LC_IGP62	IgG1 FG0n total/G0n	14	66239290	1	6.01E-14	2.28E-12	+	0.258366	0.2703	+	T	C	0.516275103	0.0341072	+	T	C	0.5433	0.0385
rs730807	LC_IGP63	IgG1 FG1n total/G1n	14	66239290	1	6.20E-14	4.36E-14	+	0.258991	0.2906	+	T	C	0.516275103	0.0342084	+	T	C	0.5433	0.0385
rs730807	LC_IGP64	IgG1 FG2n total/G2n	14	66239290	1	9.76E-11	4.71E-15	+	0.224064	0.2998	+	T	C	0.516275103	0.0343977	+	T	C	0.5433	0.0383
rs730807	LC_IGP68	IgG1 FG2n/G2n	14	66239290	1	6.41E-09	6.70E-09	+	0.201229	0.2211	+	T	C	0.516275103	0.0344737	+	T	C	0.5433	0.0381
rs730807	LC_IGP61	IgG1 Fn total	14	66239290	1	1.53E-13	6.01E-14	+	0.254752	0.2893	+	T	C	0.516275103	0.0342033	+	T	C	0.5433	0.0385
rs730807	LC_IGP81	IgG1 Fn/Bn	14	66239290	1	5.10E-12	7.24E-11	+	0.238623	0.2491	+	T	C	0.516275103	0.0343081	+	T	C	0.5433	0.0382
rs730807	LC_IGP21	IgG1 Fucosylation	14	66239290	1	1.87E-12	3.37E-13	+	0.242829	0.2803	+	T	C	0.514979664	0.0342066	+	T	C	0.5433	0.0385
rs730807	LC_IGP28	IgG1 G1S1/(G1+G1S1)	14	66239290	1	2.49E-08	6.22E-10	+	0.191293	0.2288	+	T	C	0.51516637	0.0341499	+	T	C	0.5433	0.037
rs730807	LC_IGP11	IgG1_G0	14	66239290	1	2.51E-14	1.36E-14	-	-0.260255	-0.2954	+	T	C	0.515453278	0.0338402	+	T	C	0.5433	0.0384
rs730807	LC_IGP_R1	IgG1_G0F/IgG1_G0	14	66239290	1	1.84E-13	5.73E-12	+	0.251362	0.2645	+	T	C	0.515453278	0.0338656	+	T	C	0.5433	0.0384
rs730807	LC_IGP_R4	IgG1_G0FN/IgG1_G0N	14	66239290	1	1.16E-11	1.36E-13	+	0.233114	0.2862	+	T	C	0.515779557	0.0341102	+	T	C	0.5433	0.0387
rs730807	LC_IGP52	IgG1_G0n	14	66239290	1	2.50E-14	1.05E-14	-	-0.262035	-0.2973	+	T	C	0.516275103	0.0340643	+	T	C	0.5433	0.0384
rs730807	LC_IGP14	IgG1_G0N	14	66239290	1	1.51E-14	3.74E-12	-	-0.263507	-0.2651	+	T	C	0.515779557	0.0339655	+	T	C	0.5433	0.0382
rs730807	LC_IGP55	IgG1_G0Nn	14	66239290	1	2.54E-14	3.39E-12	-	-0.262693	-0.2659	+	T	C	0.516275103	0.0341598	+	T	C	0.5433	0.0382
rs730807	LC_IGP12	IgG1_G1	14	66239290	1	7.36E-13	2.46E-13	-	-0.245612	-0.2817	+	T	C	0.515453278	0.033969	+	T	C	0.5433	0.0385
rs730807	LC_IGP_R2	IgG1_G1F/IgG1_G1	14	66239290	1	4.06E-14	2.99E-13	+	0.258727	0.2802	+	T	C	0.515453278	0.0339238	+	T	C	0.5433	0.0384

rs730807	LC_IGP_R5	IgG1_G1FN/IgG1_G1N	14	66239290	1	2.21E-11	1.01E-16	+	0.230943	0.3197	+	T	C	0.516769998	0.0342698	+	T	C	0.5433	0.0385
rs730807	LC_IGP53	IgG1_G1n	14	66239290	1	4.36E-12	3.74E-13	-	-0.239136	-0.2794	+	T	C	0.516275103	0.0342704	+	T	C	0.5433	0.0384
rs730807	LC_IGP15	IgG1_G1N	14	66239290	1	4.96E-10	1.17E-10	-	-0.214767	-0.2463	+	T	C	0.516769998	0.0343162	+	T	C	0.5433	0.0382
rs730807	LC_IGP56	IgG1_G1Nn	14	66239290	1	4.39E-10	2.29E-10	-	-0.216095	-0.2421	+	T	C	0.516275103	0.0344193	+	T	C	0.5433	0.0382
rs730807	LC_IGP_R32	IgG1_G1S1/IgG1_G1	14	66239290	1	3.91E-08	9.27E-10	+	0.188628	0.2268	+	T	C	0.51516637	0.0341641	+	T	C	0.5433	0.037
rs730807	LC_IGP_R3	IgG1_G2F/IgG1_G2	14	66239290	1	5.40E-12	1.01E-14	+	0.236559	0.2955	+	T	C	0.5151571	0.0340554	+	T	C	0.5433	0.0382
rs730807	LC_IGP_R8	IgG1_G2FS1/IgG1_G2S1	14	66239290	1	2.64E-08	2.62E-12	+	0.191297	0.2664	+	T	C	0.514842404	0.0342167	+	T	C	0.5433	0.0381
rs730807	LC_IGP_R26	IgG1_G2N/IgG1_G1N	14	66239290	1	1.71E-08	7.03E-10	+	0.194328	0.2305	+	T	C	0.516832109	0.0342889	+	T	C	0.5433	0.0374
rs730807	LC_IGP97	IgG2_G0	14	66239290	1	9.91E-09	7.89E-13	-	-0.196612	-0.274	+	T	C	0.5151571	0.0341212	+	T	C	0.5433	0.0382
rs730807	LC_IGP138	IgG2_G0n	14	66239290	1	4.71E-09	4.55E-13	-	-0.201562	-0.2773	+	T	C	0.514345452	0.0342257	+	T	C	0.5434	0.0383
rs761831	LC_IGP78	IgG1 BG0n/G0n	14	66239370	1	4.18E-12	2.58E-10	-	-0.239117	-0.2417	+	C	T	0.516268587	0.0342372	+	C	T	0.5429	0.0382
rs761831	LC_IGP79	IgG1 BG1n/G1n	14	66239370	1	4.62E-11	1.21E-11	-	-0.227792	-0.259	+	C	T	0.516268587	0.0343669	+	C	T	0.5429	0.0382
rs761831	LC_IGP77	IgG1 Bn	14	66239370	1	1.60E-11	1.51E-11	-	-0.233155	-0.2589	+	C	T	0.516268587	0.0343517	+	C	T	0.5429	0.0384
rs761831	LC_IGP86	IgG1 Bn/Fn total	14	66239370	1	3.25E-12	8.68E-12	-	-0.240875	-0.2621	+	C	T	0.516268587	0.0343109	+	C	T	0.5429	0.0384
rs761831	LC_IGP84	IgG1 FBn/Bn total	14	66239370	1	7.55E-12	9.67E-16	+	0.237279	0.3107	+	C	T	0.516268587	0.0343982	+	C	T	0.5429	0.0387
rs761831	LC_IGP62	IgG1 FG0n total/G0n	14	66239370	1	6.03E-14	2.45E-12	+	0.258361	0.2698	+	C	T	0.516268587	0.0341081	+	C	T	0.5429	0.0385
rs761831	LC_IGP63	IgG1 FG1n total/G1n	14	66239370	1	6.21E-14	4.53E-14	+	0.258988	0.2903	+	C	T	0.516268587	0.0342093	+	C	T	0.5429	0.0385
rs761831	LC_IGP64	IgG1 FG2n total/G2n	14	66239370	1	9.79E-11	4.73E-15	+	0.224054	0.2997	+	C	T	0.516268587	0.0343986	+	C	T	0.5429	0.0382
rs761831	LC_IGP68	IgG1 FG2n/G2n	14	66239370	1	6.42E-09	6.70E-09	+	0.201219	0.221	+	C	T	0.516268587	0.0344746	+	C	T	0.5429	0.0381
rs761831	LC_IGP61	IgG1 Fn total	14	66239370	1	1.54E-13	6.25E-14	+	0.254746	0.289	+	C	T	0.516268587	0.0342042	+	C	T	0.5429	0.0385
rs761831	LC_IGP81	IgG1 Fn/Bn	14	66239370	1	5.10E-12	7.77E-11	+	0.238628	0.2486	+	C	T	0.516268587	0.0343089	+	C	T	0.5429	0.0382
rs761831	LC_IGP21	IgG1 Fucosylation	14	66239370	1	1.88E-12	3.55E-13	+	0.242827	0.2799	+	C	T	0.514973181	0.0342075	+	C	T	0.5429	0.0385
rs761831	LC_IGP28	IgG1 G1S1/(G1+G1S1)	14	66239370	1	2.49E-08	5.80E-10	+	0.191289	0.2292	+	C	T	0.515159939	0.0341507	+	C	T	0.5429	0.037
rs761831	LC_IGP11	IgG1_G0	14	66239370	1	2.52E-14	1.53E-14	-	-0.260246	-0.2947	+	C	T	0.515446854	0.0338411	+	C	T	0.5429	0.0384
rs761831	LC_IGP_R1	IgG1_G0F/IgG1_G0	14	66239370	1	1.85E-13	6.08E-12	+	0.251352	0.264	+	C	T	0.515446854	0.0338665	+	C	T	0.5429	0.0384
rs761831	LC_IGP_R4	IgG1_G0FN/IgG1_G0N	14	66239370	1	1.16E-11	1.52E-13	+	0.233121	0.2854	+	C	T	0.515773117	0.034111	+	C	T	0.5429	0.0386
rs761831	LC_IGP52	IgG1_G0n	14	66239370	1	2.51E-14	1.17E-14	-	-0.262026	-0.2966	+	C	T	0.516268587	0.0340652	+	C	T	0.5429	0.0384
rs761831	LC_IGP14	IgG1_G0N	14	66239370	1	1.51E-14	4.43E-12	-	-0.263516	-0.2641	+	C	T	0.515773117	0.0339663	+	C	T	0.5429	0.0382
rs761831	LC_IGP55	IgG1_G0Nn	14	66239370	1	2.54E-14	3.98E-12	-	-0.262702	-0.265	+	C	T	0.516268587	0.0341606	+	C	T	0.5429	0.0382
rs761831	LC_IGP12	IgG1_G1	14	66239370	1	7.38E-13	2.50E-13	-	-0.245605	-0.2815	+	C	T	0.515446854	0.0339699	+	C	T	0.5429	0.0385
rs761831	LC_IGP_R2	IgG1_G1F/IgG1_G1	14	66239370	1	4.07E-14	3.07E-13	+	0.258722	0.2799	+	C	T	0.515446854	0.0339247	+	C	T	0.5429	0.0384
rs761831	LC_IGP_R5	IgG1_G1FN/IgG1_G1N	14	66239370	1	2.21E-11	1.06E-16	+	0.230947	0.3195	+	C	T	0.51676353	0.0342707	+	C	T	0.5429	0.0385
rs761831	LC_IGP53	IgG1_G1n	14	66239370	1	4.37E-12	3.78E-13	-	-0.239128	-0.2792	+	C	T	0.516268587	0.0342713	+	C	T	0.5429	0.0384
rs761831	LC_IGP15	IgG1_G1N	14	66239370	1	4.97E-10	1.21E-10	-	-0.21477	-0.246	+	C	T	0.51676353	0.034317	+	C	T	0.5429	0.0382
rs761831	LC_IGP56	IgG1_G1Nn	14	66239370	1	4.39E-10	2.36E-10	-	-0.216097	-0.2418	+	C	T	0.516268587	0.0344202	+	C	T	0.5429	0.0382
rs761831	LC_IGP_R32	IgG1_G1S1/IgG1_G1	14	66239370	1	3.92E-08	8.62E-10	+	0.188624	0.2272	+	C	T	0.515159939	0.034165	+	C	T	0.5429	0.037
rs761831	LC_IGP_R3	IgG1_G2F/IgG1_G2	14	66239370	1	5.41E-12	1.01E-14	+	0.236548	0.2954	+	C	T	0.515150672	0.0340563	+	C	T	0.5429	0.0382
rs761831	LC_IGP_R8	IgG1_G2FS1/IgG1_G2S1	14	66239370	1	2.65E-08	2.82E-12	+	0.191295	0.2659	+	C	T	0.51483598	0.0342175	+	C	T	0.5429	0.0381
rs761831	LC_IGP_R26	IgG1_G2N/IgG1_G1N	14	66239370	1	1.72E-08	7.44E-10	+	0.194328	0.23	+	C	T	0.516825617	0.0342898	+	C	T	0.5429	0.0374
rs761831	LC_IGP97	IgG2_G0	14	66239370	1	9.93E-09	8.11E-13	-	-0.196605	-0.2737	+	C	T	0.515150672	0.0341221	+	C	T	0.5429	0.0382
rs761831	LC_IGP138	IgG2_G0n	14	66239370	1	4.72E-09	4.65E-13	-	-0.201555	-0.277	+	C	T	0.514338978	0.0342266	+	C	T	0.543	0.0383
rs2411404	LC_IGP78	IgG1 BG0n/G0n	14	66239401	1	4.18E-12	2.47E-10	-	-0.239112	-0.242	+	G	C	0.516269517	0.0342376	+	G	C	0.543	0.0382
rs2411404	LC_IGP79	IgG1 BG1n/G1n	14	66239401	1	4.63E-11	1.19E-11	-	-0.227786	-0.2591	+	G	C	0.516269517	0.0343674	+	G	C	0.543	0.0382
rs2411404	LC_IGP77	IgG1 Bn	14	66239401	1	1.60E-11	1.46E-11	-	-0.233151	-0.2591	+	G	C	0.516269517	0.0343522	+	G	C	0.543	0.0384
rs2411404	LC_IGP86	IgG1 Bn/Fn total	14	66239401	1	3.25E-12	8.40E-12	-	-0.240871	-0.2623	+	G	C	0.516269517	0.0343114	+	G	C	0.543	0.0384
rs2411404	LC_IGP84	IgG1 FBn/Bn total	14	66239401	1	7.57E-12	9.29E-16	+	0.237271	0.3109	+	G	C	0.516269517	0.0343987	+	G	C	0.543	0.0387
rs2411404	LC_IGP62	IgG1 FG0n total/G0n	14	66239401	1	6.03E-14	2.37E-12	+	0.258361	0.2699	+	G	C	0.516269517	0.0341086	+	G	C	0.543	0.0385
rs2411404	LC_IGP63	IgG1 FG1n total/G1n	14	66239401	1	6.22E-14	4.49E-14	+	0.258984	0.2904	+	G	C	0.516269517	0.0342098	+	G	C	0.543	0.0385
rs2411404	LC_IGP64	IgG1 FG2n total/G2n	14	66239401	1	9.80E-11	4.66E-15	+	0.22405	0.2997	+	G	C	0.516269517	0.0343991	+	G	C	0.543	0.0382
rs2411404	LC_IGP68	IgG1 FG2n/G2n	14	66239401	1	6.43E-09	6.53E-09	+	0.201217	0.2212	+	G	C	0.516269517	0.0344751	+	G	C	0.543	0.0381
rs2411404	LC_IGP61	IgG1 Fn total	14	66239401	1	1.54E-13	6.10E-14	+	0.254744	0.2891	+	G	C	0.516269517	0.0342047	+	G	C	0.543	0.0385
rs2411404	LC_IGP81	IgG1 Fn/Bn	14	66239401	1	5.10E-12	7.52E-11	+	0.238625	0.2488	+	G	C	0.516269517	0.0343094	+	G	C	0.543	0.0382
rs2411404	LC_IGP21	IgG1 Fucosylation	14	66239401	1	1.88E-12	3.42E-13	+	0.24283	0.2801	+	G	C	0.514974414	0.034208	+	G	C	0.543	0.0385
rs2411404	LC_IGP28	IgG1 G1S1/(G1+G1S1)	14	66239401	1	2.50E-08	6.06E-10	+	0.191279	0.229	+	G	C	0.515161162	0.0341512	+	G	C	0.543	0.037
rs2411404	LC_IGP11	IgG1_G0	14	66239401	1	2.52E-14	1.51E-14	-	-0.260253	-0.2948	+	G	C	0.515448076	0.0338415	+	G	C	0.543	0.0384
rs2411404	LC_IGP_R1	IgG1_G0F/IgG1_G0	14	66239401	1	1.85E-13	5.91E-12	+	0.251355	0.2642	+	G	C	0.515448076	0.0338669	+	G	C	0.543	0.0384
rs2411404	LC_IGP_R4	IgG1_G0FN/IgG1_G0N	14	66239401	1	1.16E-11	1.44E-13	+	0.233111	0.2857	+	G	C	0.515774036	0.0341115	+	G	C	0.543	0.0386
rs2411404	LC_IGP52	IgG1_G0n	14	66239401	1	2.51E-14	1.15E-14	-	-0.262033	-0.2967	+	G	C	0.516269517	0.0340657	+	G	C	0.543	0.0384
rs2411404	LC_IGP14	IgG1_G0N	14	66239401	1	1.51E-14	4.27E-12	-	-0.263513	-0.2643	+	G	C	0.515774036	0.0339668	+	G	C	0.543	0.0382
rs2411404	LC_IGP55	IgG1_G0Nn	14	66239401	1	2.54E-14	3.83E-12	-	-0.262699	-0.2652	+	G	C	0.516269517	0.0341611	+	G	C	0.543	0.0382
rs2411404	LC_IGP12	IgG1_G1	14	66239401	1	7.39E-13	2.47E-13	-	-0.245603	-0.2816	+	G	C	0.515448076	0.0339704	+	G	C	0.543	0.0385
rs2411404	LC_IGP_R2	IgG1_G1F/IgG1_G1	14	66239401	1	4.08E-14	3.05E-13	+	0.25872	0.28	+	G	C	0.515448076	0.0339252	+	G	C	0.543	0.0384
rs2411404	LC_IGP_R5	IgG1_G1FN/IgG1_G1N	14	66239401	1	2.21E-11	1.05E-16	+	0.230941	0.3195	+	G	C	0.516764453	0.0342711	+	G	C	0.543	0.0385
rs2411404	LC_IGP53	IgG1_G1n	14	66239401	1	4.38E-12	3.72E-13	-	-0.239125	-0.2793	+	G	C	0.516269517	0.0342717	+	G	C	0.543	0.0384
rs2411404	LC_IGP15	IgG1_G1N	14	66239401	1	4.97E-10	1.19E-10	-	-0.214765	-0.2462	+	G	C	0.516764453	0.0343175	+	G	C	0.543	0.0382
rs2411404	LC_IGP56	IgG1_G1Nn	14	66239401	1	4.40E-10	2.31E-10	-	-0.216092	-0.2419	+	G	C	0.516269517	0.0344206	+	G	C	0.543	0.0382
rs2411404	LC_IGP_R32	IgG1_G1S1/IgG1_G1	14	66239401	1	3.92E-08	9.04E-10	+	0.188615	0.227	+	G	C	0.515161162	0.0341655	+	G	C	0.543	0.037
rs2411404	LC_IGP_R3	IgG1_G2F/IgG1_G2	14	66239401	1	5.42E-12	9.99E-15	+	0.236545	0.2954	+	G	C	0.515151895	0.0340568	+	G	C	0.543	0.0382
rs2411404	LC_IGP_R8	IgG1_G2FS1/IgG1_G2S1	14	66239401	1	2.65E-08	2.75E-12	+	0.191294	0.2661	+	G	C	0.514837202	0.034218	+	G	C	0.543	0.0381
rs2411404	LC_IGP_R26	IgG1_G2N/IgG1_G1N	14	66239401	1	1.72E-08	7.58E-10	+	0.194323	0.23	+	G	C	0.516826543	0.0342903	+	G	C	0.543	0.0374
rs2411404	LC_IGP97	IgG2_G0	14	66239401	1	9.94E-09	8.02E-13	-	-0.196601	-0.2738	+	G	C	0.515151895	0.0341226	+	G	C	0.543	0.0382
rs2411404	LC_IGP138	IgG2_G0n	14	66239401	1	4.72E-09	4.64E-13	-	-0.201552	-0.277	+	G	C	0.514340209	0.0342271	+	G	C	0.5431	0.0383
rs1075566	LC_IGP78	IgG1 BG0n/G0n	14	66239457	1	4.19E-12	2.44E-10	-	-0.239103	-0.2422	+	T	C	0.516270136	0.0342379	+	T	C	0.5436	0.0383
rs1075566	LC_IGP79	IgG1 BG1n/G1n	14	66239457	1	4.63E-11	1.27E-11	-	-0.227785	-0.2589	+	T	C	0.516270136	0.0343676	+	T	C	0.5436	0.0382
rs1075566	LC_IGP77	IgG1 Bn	14	66239457	1	1.60E-11	1.52E-11	-	-0.233146	-0.259	+	T	C	0.516270136	0.0343524	+	T	C	0.5436	0.0384
rs1075566	LC_IGP86	IgG1 Bn/Fn total	14	66239457	1	3.26E-12	8.85E-12	-	-0.240864	-0.2622	+	T	C	0.516270136	0.0343117	+	T	C	0.5436	0.0384
rs1075566	LC_IGP84	IgG1 FBn/Bn total	14	66239457	1	7.59E-12	9.54E-16	+	0.237257	0.311	+	T	C	0.516270136	0.034399	+	T	C	0.5436	0.0387
rs1075566	LC_IGP62	IgG1 FG0n total/G0n	14	66239457	1	6.06E-14	2.43E-12	+	0.258345	0.27	+	T	C	0.516270136	0.0341089	+	T	C	0.5436	0.0385
rs1075566	LC_IGP63	IgG1 FG1n total/G1n	14	66239457	1	6.24E-14	4.86E-14	+	0.258975	0.2901	+	T	C	0.516270136	0.0342101	+	T	C	0.5436	0.0385
rs1075566	LC_IGP64	IgG1 FG2n total/G2n	14	66239457	1	9.83E-11	5.34E-15	+	0.224037	0.2992	+	T	C	0.516270136	0.0343994	+	T	C	0.5436	0.0383
rs1075566	LC_IGP68	IgG1 FG2n/G2n	14	66239457	1	6.44E-09	7.51E-09	+	0.201213	0.2204	+	T	C	0.516270136	0.0344754	+	T	C	0.5436	0.0381
rs1075566	LC_IGP61	IgG1 Fn total	14	66239457	1	1.55E-13	6.60E-14	+	0.254729	0.2889	+	T	C	0.516270136	0.034205	+	T	C	0.5436	0.0385
rs1075566	LC_IGP81	IgG1 Fn/Bn	14	66239457	1	5.11E-12	7.79E-11	+	0.23862	0.2488	+	T	C	0.516270136	0.0343097	+	T	C	0.5436	0.0382
rs1075566	LC_IGP21	IgG1 Fucosylation	14	66239457	1	1.88E-12	3.63E-13	+	0.242815	0.28	+	T	C	0.514975031	0.0342083	+	T	C	0.5436	0.0385
rs1075566	LC_IGP28	IgG1 G1S1/(G1+G1S1)	14	66239457	1	2.50E-08	5.95E-10	+	0.191284	0.2291	+	T	C	0.515161774	0.0341515	+	T	C	0.5436	0.037
rs1075566	LC_IGP11	IgG1_G0	14	66239457	1	2.53E-14	1.49E-14	-	-0.260242	-0.2951	+	T	C	0.515448687	0.0338418	+	T	C	0.5436	0.0384
rs1075566	LC_IGP_R1	IgG1_G0F/IgG1_G0	14	66239457	1	1.86E-13	6.10E-12	+	0.25134	0.2642	+	T	C	0.515448687	0.0338672	+	T	C	0.5436	0.0384
rs1075566	LC_IGP_R4	IgG1_G0FN/IgG1_G0N	14	66239457	1	1.17E-11	1.41E-13	+	0.233096	0.2861	+	T	C	0.515774648	0.0341118	+	T	C	0.5436	0.0387
rs1075566	LC_IGP52	IgG1_G0n	14	66239457	1	2.51E-14	1.15E-14	-	-0.262021	-0.2969	+	T	C	0.516270136	0.034066	+	T	C	0.5436	0.0384
rs1075566	LC_IGP14	IgG1_G0N	14	66239457	1	1.51E-14	4.00E-12	-	-0.263511	-0.2648	+	T	C	0.515774648	0.033967	+	T	C	0.5436	0.0382
rs1075566	LC_IGP55	IgG1_G0Nn	14	66239457	1	2.55E-14	3.62E-12	-	-0.262696	-0.2656	+	T	C	0.516270136	0.0341613	+	T	C	0.5436	0.0382
rs1075566	LC_IGP12	IgG1_G1	14	66239457	1	7.41E-13	2.71E-13	-	-0.24559	-0.2813	+	T	C	0.515448687	0.0339707	+	T	C	0.5436	0.0385
rs1075566	LC_IGP_R2	IgG1_G1F/IgG1_G1	14	66239457	1	4.09E-14	3.34E-13	+	0.258711	0.2796	+	T	C	0.515448687	0.0339254	+	T	C	0.5436	0.0384
rs1075566	LC_IGP_R5	IgG1_G1FN/IgG1_G1N	14	66239457	1	2.22E-11	1.09E-16	+	0.230934	0.3195	+	T	C	0.516765068	0.0342714	+	T	C	0.5436	0.0385
rs1075566	LC_IGP53	IgG1_G1n	14	66239457	1	4.39E-12	4.13E-13	-	-0.239111	-0.2789	+	T	C	0.516270136	0.034272	+	T	C	0.5436	0.0385
rs1075566	LC_IGP15	IgG1_G1N	14	66239457	1	4.98E-10	1.27E-10	-	-0.214761	-0.2459	+	T	C	0.516765068	0.0343178	+	T	C	0.5436	0.0382
rs1075566	LC_IGP56	IgG1_G1Nn	14	66239457	1	4.40E-10	2.49E-10	-	-0.216088	-0.2416	+	T	C	0.516270136	0.0344209	+	T	C	0.5436	0.0382
rs1075566	LC_IGP_R32	IgG1_G1S1/IgG1_G1	14	66239457	1	3.92E-08	8.93E-10	+	0.18862	0.227	+	T	C	0.515161774	0.0341658	+	T	C	0.5436	0.037
rs1075566	LC_IGP_R3	IgG1_G2F/IgG1_G2	14	66239457	1	5.44E-12	1.15E-14	+	0.236533	0.2949	+	T	C	0.515152506	0.0340571	+	T	C	0.5436	0.0382
rs1075566	LC_IGP_R8	IgG1_G2FS1/IgG1_G2S1	14	66239457	1	2.65E-08	2.87E-12	+	0.19129	0.266	+	T	C	0.514837813	0.0342183	+	T	C	0.5436	0.0381
rs1075566	LC_IGP_R26	IgG1_G2N/IgG1_G1N	14	66239457	1	1.72E-08	7.60E-10	+	0.194331	0.2301	+	T	C	0.51682716	0.0342905	+	T	C	0.5436	0.0374
rs1075566	LC_IGP97	IgG2_G0	14	66239457	1	9.94E-09	7.75E-13	-	-0.196602	-0.2742	+	T	C	0.515152506	0.0341228	+	T	C	0.5436	0.0383
rs1075566	LC_IGP138	IgG2_G0n	14	66239457	1	4.72E-09	4.46E-13	-	-0.201553	-0.2774	+	T	C	0.514340825	0.0342273	+	T	C	0.5437	0.0383
rs7157449	LC_IGP78	IgG1 BG0n/G0n	14	66240137	1	4.25E-12	1.93E-10	-	-0.239088	-0.2435	+	G	T	0.516261772	0.0342452	+	G	T	0.5439	0.0382
rs7157449	LC_IGP79	IgG1 BG1n/G1n	14	66240137	1	4.68E-11	9.37E-12	-	-0.22778	-0.2604	+	G	T	0.516261772	0.0343748	+	G	T	0.5439	0.0382
rs7157449	LC_IGP77	IgG1 Bn	14	66240137	1	1.62E-11	1.19E-11	-	-0.233144	-0.2603	+	G	T	0.516261772	0.0343596	+	G	T	0.5439	0.0384
rs7157449	LC_IGP86	IgG1 Bn/Fn total	14	66240137	1	3.30E-12	6.75E-12	-	-0.240855	-0.2635	+	G	T	0.516261772	0.0343189	+	G	T	0.5439	0.0384
rs7157449	LC_IGP84	IgG1 FBn/Bn total	14	66240137	1	7.74E-12	7.76E-16	+	0.237213	0.3119	+	G	T	0.516261772	0.0344064	+	G	T	0.5439	0.0387
rs7157449	LC_IGP62	IgG1 FG0n total/G0n	14	66240137	1	6.23E-14	1.94E-12	+	0.258271	0.2711	+	G	T	0.516261772	0.0341164	+	G	T	0.5439	0.0385
rs7157449	LC_IGP63	IgG1 FG1n total/G1n	14	66240137	1	6.42E-14	3.67E-14	+	0.258903	0.2914	+	G	T	0.516261772	0.0342176	+	G	T	0.5439	0.0385
rs7157449	LC_IGP64	IgG1 FG2n total/G2n	14	66240137	1	1.01E-10	4.44E-15	+	0.223922	0.3	+	G	T	0.516261772	0.034407	+	G	T	0.5439	0.0383
rs7157449	LC_IGP68	IgG1 FG2n/G2n	14	66240137	1	6.58E-09	5.81E-09	+	0.201127	0.222	+	G	T	0.516261772	0.0344828	+	G	T	0.5439	0.0381
rs7157449	LC_IGP61	IgG1 Fn total	14	66240137	1	1.60E-13	5.10E-14	+	0.25464	0.2901	+	G	T	0.516261772	0.0342126	+	G	T	0.5439	0.0385
rs7157449	LC_IGP81	IgG1 Fn/Bn	14	66240137	1	5.16E-12	6.08E-11	+	0.238619	0.2501	+	G	T	0.516261772	0.0343169	+	G	T	0.5439	0.0382
rs7157449	LC_IGP21	IgG1 Fucosylation	14	66240137	1	1.91E-12	3.00E-13	+	0.242791	0.2809	+	G	T	0.514969174	0.0342159	+	G	T	0.5439	0.0385
rs7157449	LC_IGP28	IgG1 G1S1/(G1+G1S1)	14	66240137	1	2.53E-08	4.32E-10	+	0.191235	0.231	+	G	T	0.515155963	0.0341589	+	G	T	0.5439	0.037
rs7157449	LC_IGP11	IgG1_G0	14	66240137	1	2.59E-14	1.37E-14	-	-0.260192	-0.2954	+	G	T	0.515442883	0.0338494	+	G	T	0.5439	0.0384
rs7157449	LC_IGP_R1	IgG1_G0F/IgG1_G0	14	66240137	1	1.91E-13	4.88E-12	+	0.251256	0.2653	+	G	T	0.515442883	0.033875	+	G	T	0.5439	0.0384
rs7157449	LC_IGP_R4	IgG1_G0FN/IgG1_G0N	14	66240137	1	1.19E-11	1.17E-13	+	0.233054	0.2869	+	G	T	0.515766381	0.0341191	+	G	T	0.5439	0.0387

rs7157449	LC_IGP52	IgG1_G0n	14	66240137	1	2.57E-14	1.05E-14	-	-0.261972	-0.2973	+	G	T	0.516261772	0.0340734	+	G	T	0.5439	0.0384
rs7157449	LC_IGP14	IgG1_G0N	14	66240137	1	1.52E-14	3.52E-12	-	-0.263543	-0.2654	+	G	T	0.515766381	0.033974	+	G	T	0.5439	0.0382
rs7157449	LC_IGP55	IgG1_G0Nn	14	66240137	1	2.56E-14	3.16E-12	-	-0.262726	-0.2663	+	G	T	0.516261772	0.0341684	+	G	T	0.5439	0.0382
rs7157449	LC_IGP12	IgG1_G1	14	66240137	1	7.65E-13	2.03E-13	-	-0.245498	-0.2827	+	G	T	0.515442883	0.0339784	+	G	T	0.5439	0.0385
rs7157449	LC_IGP_R2	IgG1_G1F/IgG1_G1	14	66240137	1	4.20E-14	2.51E-13	+	0.258645	0.281	+	G	T	0.515442883	0.0339331	+	G	T	0.5439	0.0384
rs7157449	LC_IGP_R5	IgG1_G1FN/IgG1_G1N	14	66240137	1	2.25E-11	8.52E-17	+	0.230903	0.3205	+	G	T	0.516756765	0.0342786	+	G	T	0.5439	0.0385
rs7157449	LC_IGP53	IgG1_G1n	14	66240137	1	4.53E-12	3.10E-13	-	-0.239005	-0.2803	+	G	T	0.516261772	0.0342797	+	G	T	0.5439	0.0384
rs7157449	LC_IGP15	IgG1_G1N	14	66240137	1	5.04E-10	9.12E-11	-	-0.214742	-0.2477	+	G	T	0.516756765	0.0343249	+	G	T	0.5439	0.0382
rs7157449	LC_IGP56	IgG1_G1Nn	14	66240137	1	4.46E-10	1.80E-10	-	-0.216067	-0.2434	+	G	T	0.516261772	0.0344281	+	G	T	0.5439	0.0382
rs7157449	LC_IGP_R32	IgG1_G1S1/IgG1_G1	14	66240137	1	3.98E-08	6.48E-10	+	0.188576	0.229	+	G	T	0.515155963	0.0341732	+	G	T	0.5439	0.0371
rs7157449	LC_IGP_R3	IgG1_G2F/IgG1_G2	14	66240137	1	5.62E-12	9.24E-15	+	0.236426	0.2959	+	G	T	0.515146699	0.0340649	+	G	T	0.5439	0.0382
rs7157449	LC_IGP_R8	IgG1_G2FS1/IgG1_G2S1	14	66240137	1	2.68E-08	2.33E-12	+	0.191264	0.267	+	G	T	0.51483201	0.0342257	+	G	T	0.5439	0.0381
rs7157449	LC_IGP_R26	IgG1_G2N/IgG1_G1N	14	66240137	1	1.73E-08	4.74E-10	+	0.194336	0.2327	+	G	T	0.516818827	0.0342975	+	G	T	0.5439	0.0374
rs7157449	LC_IGP97	IgG2_G0	14	66240137	1	1.01E-08	8.60E-13	-	-0.19653	-0.2736	+	G	T	0.515146699	0.0341304	+	G	T	0.5439	0.0383
rs7157449	LC_IGP138	IgG2_G0n	14	66240137	1	4.81E-09	5.03E-13	-	-0.201488	-0.2768	+	G	T	0.514334975	0.0342349	+	G	T	0.544	0.0383
rs6573625	LC_IGP78	IgG1 BG0n/G0n	14	66240634	1	6.91E-12	4.03E-07	-	-0.243466	-0.2012	+	C	T	0.613209418	0.0352286	+	C	T	0.6447	0.0397
rs6573625	LC_IGP79	IgG1 BG1n/G1n	14	66240634	1	8.11E-11	2.88E-07	-	-0.231357	-0.2035	+	C	T	0.613209418	0.0353634	+	C	T	0.6447	0.0397
rs6573625	LC_IGP77	IgG1 Bn	14	66240634	1	1.65E-11	1.44E-07	-	-0.23967	-0.2095	+	C	T	0.613209418	0.0353363	+	C	T	0.6447	0.0398
rs6573625	LC_IGP86	IgG1 Bn/Fn total	14	66240634	1	2.83E-12	1.01E-07	-	-0.24846	-0.2123	+	C	T	0.613209418	0.0352907	+	C	T	0.6447	0.0399
rs6573625	LC_IGP84	IgG1 FBn/Bn total	14	66240634	1	3.63E-12	3.51E-10	+	0.247725	0.2518	+	C	T	0.613209418	0.0353678	+	C	T	0.6447	0.0401
rs6573625	LC_IGP62	IgG1 FG0n total/G0n	14	66240634	1	1.23E-15	6.94E-09	+	0.282914	0.2314	+	C	T	0.613209418	0.0350018	+	C	T	0.6447	0.04
rs6573625	LC_IGP63	IgG1 FG1n total/G1n	14	66240634	1	1.62E-13	4.74E-09	+	0.261985	0.2337	+	C	T	0.613209418	0.0352098	+	C	T	0.6447	0.0399
rs6573625	LC_IGP64	IgG1 FG2n total/G2n	14	66240634	1	4.93E-11	2.86E-10	+	0.234089	0.25	+	C	T	0.613209418	0.0353692	+	C	T	0.6447	0.0396
rs6573625	LC_IGP61	IgG1 Fn total	14	66240634	1	3.56E-14	1.59E-09	+	0.268757	0.2411	+	C	T	0.613209418	0.0351525	+	C	T	0.6447	0.04
rs6573625	LC_IGP81	IgG1 Fn/Bn	14	66240634	1	6.11E-12	2.73E-07	+	0.244557	0.204	+	C	T	0.613209418	0.0352956	+	C	T	0.6447	0.0397
rs6573625	LC_IGP21	IgG1 Fucosylation	14	66240634	1	1.06E-12	2.14E-09	+	0.251883	0.239	+	C	T	0.612673551	0.0350814	+	C	T	0.6447	0.0399
rs6573625	LC_IGP28	IgG1 G1S1/(G1+G1S1)	14	66240634	1	4.62E-08	7.37E-08	+	0.192728	0.2069	+	C	T	0.612695107	0.0350964	+	C	T	0.6447	0.0384
rs6573625	LC_IGP11	IgG1_G0	14	66240634	1	2.02E-16	6.22E-11	-	-0.287924	-0.26	+	C	T	0.613168296	0.0346579	+	C	T	0.6447	0.0398
rs6573625	LC_IGP_R1	IgG1_G0F/IgG1_G0	14	66240634	1	1.40E-15	1.27E-08	+	0.279726	0.2267	+	C	T	0.613168296	0.0346828	+	C	T	0.6447	0.0398
rs6573625	LC_IGP_R4	IgG1_G0FN/IgG1_G0N	14	66240634	1	3.43E-11	1.35E-09	+	0.23412	0.243	+	C	T	0.613423148	0.0350861	+	C	T	0.6447	0.0401
rs6573625	LC_IGP52	IgG1_G0n	14	66240634	1	1.64E-16	5.68E-11	-	-0.291164	-0.2611	+	C	T	0.613209418	0.0349335	+	C	T	0.6447	0.0398
rs6573625	LC_IGP14	IgG1_G0N	14	66240634	1	1.70E-13	1.97E-08	-	-0.259914	-0.2223	+	C	T	0.613423148	0.0349656	+	C	T	0.6447	0.0396
rs6573625	LC_IGP55	IgG1_G0Nn	14	66240634	1	9.11E-14	2.09E-08	-	-0.26442	-0.2221	+	C	T	0.613209418	0.0351665	+	C	T	0.6447	0.0396
rs6573625	LC_IGP12	IgG1_G1	14	66240634	1	1.56E-13	5.63E-09	-	-0.259617	-0.2326	+	C	T	0.612862859	0.0348705	+	C	T	0.6447	0.0399
rs6573625	LC_IGP_R2	IgG1_G1F/IgG1_G1	14	66240634	1	3.73E-14	9.26E-09	+	0.266228	0.2288	+	C	T	0.612862859	0.0348547	+	C	T	0.6447	0.0398
rs6573625	LC_IGP_R5	IgG1_G1FN/IgG1_G1N	14	66240634	1	5.05E-11	2.75E-10	+	0.233027	0.2521	+	C	T	0.613911439	0.03523	+	C	T	0.6447	0.0399
rs6573625	LC_IGP53	IgG1_G1n	14	66240634	1	2.92E-12	8.30E-09	-	-0.247964	-0.2298	+	C	T	0.613209418	0.0352443	+	C	T	0.6447	0.0399
rs6573625	LC_IGP15	IgG1_G1N	14	66240634	1	8.58E-10	7.00E-07	-	-0.217641	-0.1969	+	C	T	0.613911439	0.0352717	+	C	T	0.6447	0.0397
rs6573625	LC_IGP56	IgG1_G1Nn	14	66240634	1	4.01E-10	1.15E-06	-	-0.222794	-0.1928	+	C	T	0.613209418	0.0354041	+	C	T	0.6447	0.0396
rs6573625	LC_IGP_R3	IgG1_G2F/IgG1_G2	14	66240634	1	4.17E-12	2.99E-10	+	0.244203	0.2494	+	C	T	0.612931846	0.034968	+	C	T	0.6447	0.0396
rs6573625	LC_IGP_R8	IgG1_G2FS1/IgG1_G2S1	14	66240634	1	1.14E-08	2.09E-09	+	0.201447	0.2365	+	C	T	0.612557422	0.0351106	+	C	T	0.6447	0.0395
rs6573625	LC_IGP97	IgG2_G0	14	66240634	1	1.06E-10	1.97E-06	-	-0.226955	-0.1888	+	C	T	0.612626222	0.0349145	+	C	T	0.6447	0.0397
rs6573625	LC_IGP138	IgG2_G0n	14	66240634	1	1.78E-11	1.67E-06	-	-0.237125	-0.1902	+	C	T	0.611919027	0.0350177	+	C	T	0.6446	0.0397
rs6573626	LC_IGP78	IgG1 BG0n/G0n	14	66240695	1	4.28E-12	1.53E-10	-	-0.239088	-0.247	+	C	G	0.516249071	0.0342507	+	C	G	0.542	0.0386
rs6573626	LC_IGP79	IgG1 BG1n/G1n	14	66240695	1	4.70E-11	6.39E-12	-	-0.227787	-0.2649	+	C	G	0.516249071	0.0343803	+	C	G	0.542	0.0386
rs6573626	LC_IGP77	IgG1 Bn	14	66240695	1	1.63E-11	9.67E-12	-	-0.233153	-0.2638	+	C	G	0.516249071	0.0343651	+	C	G	0.542	0.0387
rs6573626	LC_IGP86	IgG1 Bn/Fn total	14	66240695	1	3.32E-12	5.41E-12	-	-0.240857	-0.2671	+	C	G	0.516249071	0.0343244	+	C	G	0.542	0.0388
rs6573626	LC_IGP84	IgG1 FBn/Bn total	14	66240695	1	7.84E-12	6.30E-16	+	0.237185	0.3154	+	C	G	0.516249071	0.034412	+	C	G	0.542	0.039
rs6573626	LC_IGP62	IgG1 FG0n total/G0n	14	66240695	1	6.38E-14	1.28E-12	+	0.25821	0.2756	+	C	G	0.516249071	0.0341222	+	C	G	0.542	0.0388
rs6573626	LC_IGP63	IgG1 FG1n total/G1n	14	66240695	1	6.56E-14	2.09E-14	+	0.258846	0.2968	+	C	G	0.516249071	0.0342234	+	C	G	0.542	0.0388
rs6573626	LC_IGP64	IgG1 FG2n total/G2n	14	66240695	1	1.04E-10	2.41E-15	+	0.223827	0.3056	+	C	G	0.516249071	0.0344129	+	C	G	0.542	0.0386
rs6573626	LC_IGP68	IgG1 FG2n/G2n	14	66240695	1	6.70E-09	3.84E-09	+	0.201054	0.2267	+	C	G	0.516249071	0.0344886	+	C	G	0.542	0.0385
rs6573626	LC_IGP61	IgG1 Fn total	14	66240695	1	1.64E-13	3.39E-14	+	0.254568	0.2947	+	C	G	0.516249071	0.0342184	+	C	G	0.542	0.0389
rs6573626	LC_IGP81	IgG1 Fn/Bn	14	66240695	1	5.20E-12	4.63E-11	+	0.238626	0.254	+	C	G	0.516249071	0.0343223	+	C	G	0.542	0.0386
rs6573626	LC_IGP21	IgG1 Fucosylation	14	66240695	1	1.94E-12	2.50E-13	+	0.242765	0.2843	+	C	G	0.514958385	0.0342218	+	C	G	0.542	0.0388
rs6573626	LC_IGP28	IgG1 G1S1/(G1+G1S1)	14	66240695	1	2.56E-08	3.48E-10	+	0.191196	0.2343	+	C	G	0.51514526	0.0341647	+	C	G	0.542	0.0373
rs6573626	LC_IGP11	IgG1_G0	14	66240695	1	2.64E-14	5.09E-15	-	-0.260138	-0.3026	+	C	G	0.515432193	0.0338553	+	C	G	0.542	0.0387
rs6573626	LC_IGP_R1	IgG1_G0F/IgG1_G0	14	66240695	1	1.96E-13	3.08E-12	+	0.251183	0.2701	+	C	G	0.515432193	0.0338809	+	C	G	0.542	0.0387
rs6573626	LC_IGP_R4	IgG1_G0FN/IgG1_G0N	14	66240695	1	1.20E-11	8.13E-14	+	0.23303	0.2909	+	C	G	0.515753827	0.0341246	+	C	G	0.542	0.039
rs6573626	LC_IGP52	IgG1_G0n	14	66240695	1	2.63E-14	4.15E-15	-	-0.261919	-0.3042	+	C	G	0.516249071	0.0340792	+	C	G	0.542	0.0388
rs6573626	LC_IGP14	IgG1_G0N	14	66240695	1	1.52E-14	2.03E-12	-	-0.263572	-0.2706	+	C	G	0.515753827	0.0339793	+	C	G	0.542	0.0385
rs6573626	LC_IGP55	IgG1_G0Nn	14	66240695	1	2.57E-14	1.93E-12	-	-0.262754	-0.2712	+	C	G	0.516249071	0.0341738	+	C	G	0.542	0.0385
rs6573626	LC_IGP12	IgG1_G1	14	66240695	1	7.84E-13	1.34E-13	-	-0.245424	-0.2872	+	C	G	0.515432193	0.0339844	+	C	G	0.542	0.0388
rs6573626	LC_IGP_R2	IgG1_G1F/IgG1_G1	14	66240695	1	4.29E-14	1.35E-13	+	0.25859	0.2867	+	C	G	0.515432193	0.033939	+	C	G	0.542	0.0387
rs6573626	LC_IGP_R5	IgG1_G1FN/IgG1_G1N	14	66240695	1	2.28E-11	5.34E-17	+	0.230885	0.3253	+	C	G	0.516744157	0.0342842	+	C	G	0.542	0.0388
rs6573626	LC_IGP53	IgG1_G1n	14	66240695	1	4.65E-12	2.26E-13	-	-0.238922	-0.2843	+	C	G	0.516249071	0.0342856	+	C	G	0.542	0.0388
rs6573626	LC_IGP15	IgG1_G1N	14	66240695	1	5.07E-10	7.45E-11	-	-0.214739	-0.2511	+	C	G	0.516744157	0.0343304	+	C	G	0.542	0.0386
rs6573626	LC_IGP56	IgG1_G1Nn	14	66240695	1	4.49E-10	1.58E-10	-	-0.216064	-0.2464	+	C	G	0.516249071	0.0344337	+	C	G	0.542	0.0385
rs6573626	LC_IGP_R32	IgG1_G1S1/IgG1_G1	14	66240695	1	4.02E-08	5.13E-10	+	0.188541	0.2324	+	C	G	0.51514526	0.0341789	+	C	G	0.542	0.0374
rs6573626	LC_IGP_R3	IgG1_G2F/IgG1_G2	14	66240695	1	5.76E-12	5.00E-15	+	0.236339	0.3015	+	C	G	0.515136002	0.0340709	+	C	G	0.542	0.0385
rs6573626	LC_IGP_R8	IgG1_G2FS1/IgG1_G2S1	14	66240695	1	2.71E-08	9.08E-13	+	0.191238	0.2744	+	C	G	0.514821319	0.0342314	+	C	G	0.542	0.0384
rs6573626	LC_IGP_R26	IgG1_G2N/IgG1_G1N	14	66240695	1	1.73E-08	3.84E-10	+	0.194352	0.2358	+	C	G	0.516806173	0.0343029	+	C	G	0.542	0.0377
rs6573626	LC_IGP97	IgG2_G0	14	66240695	1	1.03E-08	1.99E-13	-	-0.196468	-0.2835	+	C	G	0.515136002	0.0341362	+	C	G	0.542	0.0386
rs6573626	LC_IGP138	IgG2_G0n	14	66240695	1	4.89E-09	1.11E-13	-	-0.201432	-0.287	+	C	G	0.5143242	0.0342408	+	C	G	0.5422	0.0386
rs7158556	LC_IGP78	IgG1 BG0n/G0n	14	66240729	1	6.92E-12	4.08E-07	-	-0.24346	-0.2011	+	A	T	0.613210967	0.0352298	+	A	T	0.6446	0.0397
rs7158556	LC_IGP79	IgG1 BG1n/G1n	14	66240729	1	8.14E-11	2.94E-07	-	-0.23135	-0.2033	+	A	T	0.613210967	0.0353645	+	A	T	0.6446	0.0397
rs7158556	LC_IGP77	IgG1 Bn	14	66240729	1	1.66E-11	1.47E-07	-	-0.239663	-0.2094	+	A	T	0.613210967	0.0353375	+	A	T	0.6446	0.0398
rs7158556	LC_IGP86	IgG1 Bn/Fn total	14	66240729	1	2.84E-12	1.02E-07	-	-0.248453	-0.2121	+	A	T	0.613210967	0.0352919	+	A	T	0.6446	0.0399
rs7158556	LC_IGP84	IgG1 FBn/Bn total	14	66240729	1	3.65E-12	3.56E-10	+	0.247713	0.2517	+	A	T	0.613210967	0.0353689	+	A	T	0.6446	0.0401
rs7158556	LC_IGP62	IgG1 FG0n total/G0n	14	66240729	1	1.23E-15	7.04E-09	+	0.282905	0.2313	+	A	T	0.613210967	0.035003	+	A	T	0.6446	0.04
rs7158556	LC_IGP63	IgG1 FG1n total/G1n	14	66240729	1	1.63E-13	4.83E-09	+	0.261974	0.2336	+	A	T	0.613210967	0.0352109	+	A	T	0.6446	0.0399
rs7158556	LC_IGP64	IgG1 FG2n total/G2n	14	66240729	1	4.95E-11	2.92E-10	+	0.234072	0.2499	+	A	T	0.613210967	0.0353704	+	A	T	0.6446	0.0396
rs7158556	LC_IGP61	IgG1 Fn total	14	66240729	1	3.57E-14	1.62E-09	+	0.268745	0.241	+	A	T	0.613210967	0.0351537	+	A	T	0.6446	0.04
rs7158556	LC_IGP81	IgG1 Fn/Bn	14	66240729	1	6.13E-12	2.79E-07	+	0.244552	0.2039	+	A	T	0.613210967	0.0352967	+	A	T	0.6446	0.0397
rs7158556	LC_IGP21	IgG1 Fucosylation	14	66240729	1	1.06E-12	2.19E-09	+	0.251876	0.2389	+	A	T	0.612675401	0.0350826	+	A	T	0.6446	0.0399
rs7158556	LC_IGP28	IgG1 G1S1/(G1+G1S1)	14	66240729	1	4.63E-08	7.45E-08	+	0.192723	0.2068	+	A	T	0.612696942	0.0350976	+	A	T	0.6446	0.0384
rs7158556	LC_IGP11	IgG1_G0	14	66240729	1	2.03E-16	6.35E-11	-	-0.287918	-0.2599	+	A	T	0.613170128	0.0346591	+	A	T	0.6446	0.0398
rs7158556	LC_IGP_R1	IgG1_G0F/IgG1_G0	14	66240729	1	1.40E-15	1.29E-08	+	0.279718	0.2266	+	A	T	0.613170128	0.0346839	+	A	T	0.6446	0.0398
rs7158556	LC_IGP_R4	IgG1_G0FN/IgG1_G0N	14	66240729	1	3.44E-11	1.36E-09	+	0.234111	0.2429	+	A	T	0.613424679	0.0350872	+	A	T	0.6446	0.0401
rs7158556	LC_IGP52	IgG1_G0n	14	66240729	1	1.65E-16	5.79E-11	-	-0.291158	-0.261	+	A	T	0.613210967	0.0349346	+	A	T	0.6446	0.0398
rs7158556	LC_IGP14	IgG1_G0N	14	66240729	1	1.70E-13	2.01E-08	-	-0.259913	-0.2222	+	A	T	0.613424679	0.0349667	+	A	T	0.6446	0.0396
rs7158556	LC_IGP55	IgG1_G0Nn	14	66240729	1	9.13E-14	2.13E-08	-	-0.264419	-0.222	+	A	T	0.613210967	0.0351676	+	A	T	0.6446	0.0396
rs7158556	LC_IGP12	IgG1_G1	14	66240729	1	1.56E-13	5.72E-09	-	-0.259606	-0.2325	+	A	T	0.612864692	0.0348716	+	A	T	0.6446	0.0399
rs7158556	LC_IGP_R2	IgG1_G1F/IgG1_G1	14	66240729	1	3.74E-14	9.45E-09	+	0.266219	0.2286	+	A	T	0.612864692	0.0348559	+	A	T	0.6446	0.0398
rs7158556	LC_IGP_R5	IgG1_G1FN/IgG1_G1N	14	66240729	1	5.07E-11	2.80E-10	+	0.233015	0.252	+	A	T	0.613912977	0.0352312	+	A	T	0.6446	0.0399
rs7158556	LC_IGP53	IgG1_G1n	14	66240729	1	2.94E-12	8.42E-09	-	-0.247951	-0.2297	+	A	T	0.613210967	0.0352455	+	A	T	0.6446	0.0399
rs7158556	LC_IGP15	IgG1_G1N	14	66240729	1	8.61E-10	7.14E-07	-	-0.217633	-0.1968	+	A	T	0.613912977	0.0352729	+	A	T	0.6446	0.0397
rs7158556	LC_IGP56	IgG1_G1Nn	14	66240729	1	4.02E-10	1.17E-06	-	-0.222786	-0.1926	+	A	T	0.613210967	0.0354053	+	A	T	0.6446	0.0396
rs7158556	LC_IGP_R3	IgG1_G2F/IgG1_G2	14	66240729	1	4.19E-12	3.05E-10	+	0.244188	0.2492	+	A	T	0.61293368	0.0349692	+	A	T	0.6446	0.0396
rs7158556	LC_IGP_R8	IgG1_G2FS1/IgG1_G2S1	14	66240729	1	1.14E-08	2.13E-09	+	0.201442	0.2364	+	A	T	0.612559255	0.0351117	+	A	T	0.6446	0.0395
rs7158556	LC_IGP97	IgG2_G0	14	66240729	1	1.06E-10	1.99E-06	-	-0.226963	-0.1888	+	A	T	0.612628056	0.0349156	+	A	T	0.6446	0.0397
rs7158556	LC_IGP138	IgG2_G0n	14	66240729	1	1.77E-11	1.69E-06	-	-0.237134	-0.1902	+	A	T	0.611920874	0.0350188	+	A	T	0.6446	0.0397
rs12894466	LC_IGP78	IgG1 BG0n/G0n	14	66240767	1	4.28E-12	2.63E-10	-	-0.23909	-0.2417	+	A	G	0.516245983	0.0342514	+	A	G	0.5429	0.0382
rs12894466	LC_IGP79	IgG1 BG1n/G1n	14	66240767	1	4.71E-11	1.26E-11	-	-0.227784	-0.259	+	A	G	0.516245983	0.0343811	+	A	G	0.5429	0.0382
rs12894466	LC_IGP77	IgG1 Bn	14	66240767	1	1.63E-11	1.56E-11	-	-0.233155	-0.2588	+	A	G	0.516245983	0.0343659	+	A	G	0.5429	0.0384
rs12894466	LC_IGP86	IgG1 Bn/Fn total	14	66240767	1	3.33E-12	8.96E-12	-	-0.240857	-0.262	+	A	G	0.516245983	0.0343252	+	A	G	0.5429	0.0384
rs12894466	LC_IGP84	IgG1 FBn/Bn total	14	66240767	1	7.85E-12	9.77E-16	+	0.237182	0.3109	+	A	G	0.516245983	0.0344128	+	A	G	0.5429	0.0387
rs12894466	LC_IGP62	IgG1 FG0n total/G0n	14	66240767	1	6.39E-14	2.53E-12	+	0.258203	0.2698	+	A	G	0.516245983	0.034123	+	A	G	0.5429	0.0385
rs12894466	LC_IGP63	IgG1 FG1n total/G1n	14	66240767	1	6.58E-14	4.78E-14	+	0.258837	0.2903	+	A	G	0.516245983	0.0342242	+	A	G	0.5429	0.0385
rs12894466	LC_IGP64	IgG1 FG2n total/G2n	14	66240767	1	1.04E-10	4.95E-15	+	0.223816	0.2996	+	A	G	0.516245983	0.0344138	+	A	G	0.5429	0.0383
rs12894466	LC_IGP68	IgG1 FG2n/G2n	14	66240767	1	6.72E-09	7.07E-09	+	0.201044	0.2208	+	A	G	0.516245983	0.0344894	+	A	G	0.5429	0.0381
rs12894466	LC_IGP61	IgG1 Fn total	14	66240767	1	1.64E-13	6.54E-14	+	0.254559	0.2889	+	A	G	0.516245983	0.0342193	+	A	G	0.5429	0.0385
rs12894466	LC_IGP81	IgG1 Fn/Bn	14	66240767	1	5.20E-12	8.04E-11	+	0.238627	0.2485	+	A	G	0.516245983	0.0343231	+	A	G	0.5429	0.0382
rs12894466	LC_IGP21	IgG1 Fucosylation	14	66240767	1	1.94E-12	3.64E-13	+	0.242764	0.28	+	A	G	0.51495562	0.0342226	+	A	G	0.5429	0.0385
rs12894466	LC_IGP28	IgG1 G1S1/(G1+G1S1)	14	66240767	1	2.57E-08	5.96E-10	+	0.191193	0.2292	+	A	G	0.515142517	0.0341655	+	A	G	0.5429	0.037
rs12894466	LC_IGP11	IgG1_G0	14	66240767	1	2.65E-14	1.59E-14	-	-0.260133	-0.2948	+	A	G	0.515429454	0.0338561	+	A	G	0.5429	0.0384
rs12894466	LC_IGP_R1	IgG1_G0F/IgG1_G0	14	66240767	1	1.97E-13	6.32E-12	+	0.251173	0.264	+	A	G	0.515429454	0.0338818	+	A	G	0.5429	0.0384
rs12894466	LC_IGP_R4	IgG1_G0FN/IgG1_G0N	14	66240767	1	1.21E-11	1.53E-13	+	0.233029	0.2855	+	A	G	0.515750775	0.0341254	+	A	G	0.5429	0.0387
rs12894466	LC_IGP52	IgG1_G0n	14	66240767	1	2.64E-14	1.22E-14	-	-0.261914	-0.2967	+	A	G	0.516245983	0.0340799	+	A	G	0.5429	0.0385

rs12894466	LC_IGP14	IgG1_G0N	14	66240767	1	1.52E-14	4.55E-12	-	-0.263581	-0.2641	+	A	G	0.515750775	0.03398	+	A	G	0.5429	0.0382
rs12894466	LC_IGP55	IgG1_G0Nn	14	66240767	1	2.57E-14	4.08E-12	-	-0.262763	-0.265	+	A	G	0.516245983	0.0341745	+	A	G	0.5429	0.0382
rs12894466	LC_IGP12	IgG1_G1	14	66240767	1	7.87E-13	2.64E-13	-	-0.245413	-0.2815	+	A	G	0.515429454	0.0339853	+	A	G	0.5429	0.0385
rs12894466	LC_IGP_R2	IgG1_G1F/IgG1_G1	14	66240767	1	4.31E-14	3.27E-13	+	0.258582	0.2798	+	A	G	0.515429454	0.0339399	+	A	G	0.5429	0.0384
rs12894466	LC_IGP_R5	IgG1_G1FN/IgG1_G1N	14	66240767	1	2.28E-11	1.08E-16	+	0.230879	0.3196	+	A	G	0.516741093	0.034285	+	A	G	0.5429	0.0385
rs12894466	LC_IGP53	IgG1_G1n	14	66240767	1	4.67E-12	3.97E-13	-	-0.23891	-0.2791	+	A	G	0.516245983	0.0342864	+	A	G	0.5429	0.0385
rs12894466	LC_IGP15	IgG1_G1N	14	66240767	1	5.08E-10	1.25E-10	-	-0.214734	-0.246	+	A	G	0.516741093	0.0343312	+	A	G	0.5429	0.0382
rs12894466	LC_IGP56	IgG1_G1Nn	14	66240767	1	4.50E-10	2.43E-10	-	-0.216059	-0.2417	+	A	G	0.516245983	0.0344345	+	A	G	0.5429	0.0382
rs12894466	LC_IGP_R32	IgG1_G1S1/IgG1_G1	14	66240767	1	4.02E-08	8.85E-10	+	0.188538	0.2272	+	A	G	0.515142517	0.0341797	+	A	G	0.5429	0.0371
rs12894466	LC_IGP_R3	IgG1_G2F/IgG1_G2	14	66240767	1	5.78E-12	1.06E-14	+	0.236328	0.2953	+	A	G	0.515133261	0.0340718	+	A	G	0.5429	0.0382
rs12894466	LC_IGP_R8	IgG1_G2FS1/IgG1_G2S1	14	66240767	1	2.71E-08	2.89E-12	+	0.191234	0.266	+	A	G	0.51481858	0.0342322	+	A	G	0.5429	0.0381
rs12894466	LC_IGP_R26	IgG1_G2N/IgG1_G1N	14	66240767	1	1.73E-08	7.37E-10	+	0.194347	0.2303	+	A	G	0.516803097	0.0343037	+	A	G	0.5429	0.0374
rs12894466	LC_IGP97	IgG2_G0	14	66240767	1	1.03E-08	8.64E-13	-	-0.196463	-0.2736	+	A	G	0.515133261	0.034137	+	A	G	0.5429	0.0383
rs12894466	LC_IGP138	IgG2_G0n	14	66240767	1	4.90E-09	4.94E-13	-	-0.201428	-0.2769	+	A	G	0.514321437	0.0342417	+	A	G	0.543	0.0383
rs112850324	LC_IGP78	IgG1 BG0n/G0n	14	66241579	1	4.63E-10	1.92E-09	+	0.250268	0.3668	+	A	G	0.256531908	0.0399153	+	A	G	0.1484	0.0611
rs112850324	LC_IGP79	IgG1 BG1n/G1n	14	66241579	1	1.01E-08	3.43E-10	+	0.230817	0.3852	+	A	G	0.256531908	0.0400828	+	A	G	0.1484	0.0614
rs112850324	LC_IGP77	IgG1 Bn	14	66241579	1	1.29E-09	3.42E-10	+	0.244431	0.3864	+	A	G	0.256531908	0.0400409	+	A	G	0.1484	0.0616
rs112850324	LC_IGP86	IgG1 Bn/Fn total	14	66241579	1	2.18E-10	2.93E-10	+	0.2555	0.3877	+	A	G	0.256531908	0.0399891	+	A	G	0.1484	0.0615
rs112850324	LC_IGP84	IgG1 FBn/Bn total	14	66241579	1	2.29E-11	6.36E-13	-	-0.269466	-0.448	+	A	G	0.256531908	0.0400155	+	A	G	0.1484	0.0623
rs112850324	LC_IGP62	IgG1 FG0n total/G0n	14	66241579	1	6.72E-14	1.73E-10	-	-0.299789	-0.3897	+	A	G	0.256531908	0.039654	+	A	G	0.1484	0.061
rs112850324	LC_IGP63	IgG1 FG1n total/G1n	14	66241579	1	9.81E-12	6.47E-11	-	-0.273643	-0.4007	+	A	G	0.256531908	0.0398918	+	A	G	0.1484	0.0613
rs112850324	LC_IGP64	IgG1 FG2n total/G2n	14	66241579	1	1.21E-11	5.19E-12	-	-0.272715	-0.4222	+	A	G	0.256531908	0.0399384	+	A	G	0.1484	0.0612
rs112850324	LC_IGP68	IgG1 FG2n/G2n	14	66241579	1	8.89E-09	3.35E-08	-	-0.231741	-0.3335	+	A	G	0.256531908	0.0400853	+	A	G	0.1484	0.0604
rs112850324	LC_IGP61	IgG1 Fn total	14	66241579	1	5.92E-13	4.51E-11	-	-0.288986	-0.404	+	A	G	0.256531908	0.0397957	+	A	G	0.1484	0.0614
rs112850324	LC_IGP81	IgG1 Fn/Bn	14	66241579	1	7.95E-10	7.16E-10	-	-0.247366	-0.3768	+	A	G	0.256531908	0.0400072	+	A	G	0.1484	0.0612
rs112850324	LC_IGP21	IgG1 Fucosylation	14	66241579	1	6.63E-12	9.03E-11	-	-0.275185	-0.3977	+	A	G	0.257122072	0.0397851	+	A	G	0.1484	0.0613
rs112850324	LC_IGP11	IgG1_G0	14	66241579	1	3.04E-15	1.80E-12	+	0.313302	0.4286	+	A	G	0.256586133	0.0393282	+	A	G	0.1484	0.0608
rs112850324	LC_IGP_R1	IgG1_G0F/IgG1_G0	14	66241579	1	6.26E-14	6.14E-10	-	-0.298075	-0.3751	+	A	G	0.256586133	0.0393822	+	A	G	0.1484	0.0606
rs112850324	LC_IGP_R4	IgG1_G0FN/IgG1_G0N	14	66241579	1	9.60E-11	3.02E-12	-	-0.259132	-0.4309	+	A	G	0.256602266	0.0397692	+	A	G	0.1484	0.0618
rs112850324	LC_IGP52	IgG1_G0n	14	66241579	1	2.21E-15	1.70E-12	+	0.316656	0.4296	+	A	G	0.256531908	0.0395429	+	A	G	0.1484	0.0609
rs112850324	LC_IGP14	IgG1_G0N	14	66241579	1	1.61E-11	2.73E-11	+	0.269499	0.4082	+	A	G	0.256602266	0.0397168	+	A	G	0.1484	0.0613
rs112850324	LC_IGP55	IgG1_G0Nn	14	66241579	1	1.58E-11	3.21E-11	+	0.27066	0.407	+	A	G	0.256531908	0.0398684	+	A	G	0.1484	0.0613
rs112850324	LC_IGP12	IgG1_G1	14	66241579	1	4.48E-12	2.48E-10	+	0.275945	0.3869	+	A	G	0.25689157	0.0395719	+	A	G	0.1484	0.0611
rs112850324	LC_IGP_R2	IgG1_G1F/IgG1_G1	14	66241579	1	5.26E-12	2.72E-10	-	-0.275165	-0.3848	+	A	G	0.25689157	0.0395919	+	A	G	0.1484	0.0609
rs112850324	LC_IGP_R5	IgG1_G1FN/IgG1_G1N	14	66241579	1	3.50E-10	1.16E-13	-	-0.251837	-0.4597	+	A	G	0.256483087	0.0398858	+	A	G	0.1484	0.0619
rs112850324	LC_IGP53	IgG1_G1n	14	66241579	1	3.15E-11	5.16E-10	+	0.266709	0.3798	+	A	G	0.256531908	0.0398888	+	A	G	0.1484	0.0611
rs112850324	LC_IGP15	IgG1_G1N	14	66241579	1	2.18E-08	9.89E-10	+	0.224807	0.3741	+	A	G	0.256483087	0.0399639	+	A	G	0.1484	0.0612
rs112850324	LC_IGP56	IgG1_G1Nn	14	66241579	1	2.83E-08	2.46E-09	+	0.223812	0.3648	+	A	G	0.256531908	0.0401149	+	A	G	0.1484	0.0612
rs112850324	LC_IGP_R3	IgG1_G2F/IgG1_G2	14	66241579	1	1.81E-12	4.23E-12	-	-0.281211	-0.4226	+	A	G	0.256742971	0.0395891	+	A	G	0.1484	0.061
rs112850324	LC_IGP_R8	IgG1_G2FS1/IgG1_G2S1	14	66241579	1	2.78E-08	2.70E-10	-	-0.222313	-0.3809	+	A	G	0.256847282	0.0398251	+	A	G	0.1484	0.0603
rs112850324	LC_IGP97	IgG2_G0	14	66241579	1	2.19E-10	7.71E-07	+	0.252997	0.3057	+	A	G	0.256698655	0.0396052	+	A	G	0.1484	0.0618
rs112850324	LC_IGP138	IgG2_G0n	14	66241579	1	1.24E-10	8.04E-07	+	0.256866	0.3052	+	A	G	0.257101293	0.0396612	+	A	G	0.1485	0.0619
rs7142018	LC_IGP78	IgG1 BG0n/G0n	14	66241673	1	9.36E-12	1.71E-09	-	-0.23519	-0.2366	+	T	G	0.511149938	0.0342517	+	T	G	0.519	0.0393
rs7142018	LC_IGP79	IgG1 BG1n/G1n	14	66241673	1	7.42E-11	3.54E-11	-	-0.225358	-0.2602	+	T	G	0.511149938	0.0343745	+	T	G	0.519	0.0393
rs7142018	LC_IGP77	IgG1 Bn	14	66241673	1	3.35E-11	5.51E-11	-	-0.229443	-0.2586	+	T	G	0.511149938	0.0343648	+	T	G	0.519	0.0394
rs7142018	LC_IGP86	IgG1 Bn/Fn total	14	66241673	1	6.59E-12	2.77E-11	-	-0.237449	-0.2628	+	T	G	0.511149938	0.0343234	+	T	G	0.519	0.0395
rs7142018	LC_IGP84	IgG1 FBn/Bn total	14	66241673	1	1.59E-11	7.35E-15	+	0.233588	0.31	+	T	G	0.511149938	0.0344115	+	T	G	0.519	0.0398
rs7142018	LC_IGP62	IgG1 FG0n total/G0n	14	66241673	1	9.99E-14	1.26E-11	+	0.256099	0.2683	+	T	G	0.511149938	0.0341163	+	T	G	0.519	0.0396
rs7142018	LC_IGP63	IgG1 FG1n total/G1n	14	66241673	1	7.80E-14	1.96E-13	+	0.257961	0.2912	+	T	G	0.511149938	0.0342117	+	T	G	0.519	0.0396
rs7142018	LC_IGP64	IgG1 FG2n total/G2n	14	66241673	1	1.06E-10	2.34E-14	+	0.223622	0.3007	+	T	G	0.511149938	0.034398	+	T	G	0.519	0.0394
rs7142018	LC_IGP68	IgG1 FG2n/G2n	14	66241673	1	8.54E-09	2.08E-08	+	0.19956	0.2199	+	T	G	0.511149938	0.0344783	+	T	G	0.519	0.0392
rs7142018	LC_IGP61	IgG1 Fn total	14	66241673	1	1.91E-13	3.00E-13	+	0.25375	0.2891	+	T	G	0.511149938	0.0342064	+	T	G	0.519	0.0396
rs7142018	LC_IGP81	IgG1 Fn/Bn	14	66241673	1	1.03E-11	3.68E-10	+	0.2352	0.2463	+	T	G	0.511149938	0.0343213	+	T	G	0.519	0.0393
rs7142018	LC_IGP21	IgG1 Fucosylation	14	66241673	1	1.94E-12	1.15E-12	+	0.242673	0.2817	+	T	G	0.509886868	0.0342086	+	T	G	0.519	0.0396
rs7142018	LC_IGP28	IgG1 G1S1/(G1+G1S1)	14	66241673	1	1.77E-08	3.71E-09	+	0.193289	0.2242	+	T	G	0.510114067	0.0341439	+	T	G	0.519	0.038
rs7142018	LC_IGP11	IgG1_G0	14	66241673	1	4.28E-14	8.56E-14	-	-0.257935	-0.2949	+	T	G	0.510407147	0.0338516	+	T	G	0.519	0.0395
rs7142018	LC_IGP_R1	IgG1_G0F/IgG1_G0	14	66241673	1	2.63E-13	3.02E-11	+	0.249757	0.2626	+	T	G	0.510407147	0.0338734	+	T	G	0.519	0.0395
rs7142018	LC_IGP_R4	IgG1_G0FN/IgG1_G0N	14	66241673	1	3.52E-11	1.88E-12	+	0.227621	0.28	+	T	G	0.510714023	0.0341321	+	T	G	0.519	0.0398
rs7142018	LC_IGP52	IgG1_G0n	14	66241673	1	4.12E-14	7.04E-14	-	-0.259832	-0.2964	+	T	G	0.511149938	0.0340733	+	T	G	0.519	0.0396
rs7142018	LC_IGP14	IgG1_G0N	14	66241673	1	4.76E-14	3.70E-11	-	-0.258501	-0.2596	+	T	G	0.510714023	0.0339883	+	T	G	0.519	0.0392
rs7142018	LC_IGP55	IgG1_G0Nn	14	66241673	1	7.61E-14	3.44E-11	-	-0.257841	-0.2603	+	T	G	0.511149938	0.0341812	+	T	G	0.519	0.0393
rs7142018	LC_IGP12	IgG1_G1	14	66241673	1	8.15E-13	9.32E-13	-	-0.245146	-0.2826	+	T	G	0.510407147	0.0339716	+	T	G	0.519	0.0396
rs7142018	LC_IGP_R2	IgG1_G1F/IgG1_G1	14	66241673	1	5.05E-14	1.24E-12	+	0.257775	0.2806	+	T	G	0.510407147	0.0339288	+	T	G	0.519	0.0395
rs7142018	LC_IGP_R5	IgG1_G1FN/IgG1_G1N	14	66241673	1	3.63E-11	8.92E-16	+	0.228436	0.319	+	T	G	0.511682657	0.034278	+	T	G	0.519	0.0397
rs7142018	LC_IGP53	IgG1_G1n	14	66241673	1	4.42E-12	1.45E-12	-	-0.239059	-0.2799	+	T	G	0.511149938	0.0342692	+	T	G	0.519	0.0395
rs7142018	LC_IGP15	IgG1_G1N	14	66241673	1	8.11E-10	3.08E-10	-	-0.212108	-0.2472	+	T	G	0.511682657	0.0343242	+	T	G	0.519	0.0393
rs7142018	LC_IGP56	IgG1_G1Nn	14	66241673	1	6.80E-10	6.16E-10	-	-0.213729	-0.2426	+	T	G	0.511149938	0.034427	+	T	G	0.519	0.0392
rs7142018	LC_IGP_R32	IgG1_G1S1/IgG1_G1	14	66241673	1	2.71E-08	5.30E-09	+	0.190826	0.2222	+	T	G	0.510114067	0.0341576	+	T	G	0.519	0.0381
rs7142018	LC_IGP_R3	IgG1_G2F/IgG1_G2	14	66241673	1	5.45E-12	5.48E-14	+	0.236521	0.2957	+	T	G	0.510107885	0.0340565	+	T	G	0.519	0.0393
rs7142018	LC_IGP_R8	IgG1_G2FS1/IgG1_G2S1	14	66241673	1	2.54E-08	4.14E-12	+	0.191545	0.2718	+	T	G	0.509796274	0.0342172	+	T	G	0.519	0.0392
rs7142018	LC_IGP_R26	IgG1_G2N/IgG1_G1N	14	66241673	1	2.19E-08	9.80E-09	+	0.192855	0.22	+	T	G	0.511725926	0.0342918	+	T	G	0.519	0.0384
rs7142018	LC_IGP97	IgG2_G0	14	66241673	1	2.04E-08	1.80E-12	-	-0.192411	-0.2769	+	T	G	0.510107885	0.0341368	+	T	G	0.519	0.0393
rs7142018	LC_IGP138	IgG2_G0n	14	66241673	1	9.58E-09	1.20E-12	-	-0.197513	-0.2794	+	T	G	0.509258929	0.0342423	+	T	G	0.519	0.0393
rs7142219	LC_IGP78	IgG1 BG0n/G0n	14	66241781	1	4.32E-12	2.75E-10	-	-0.239103	-0.2426	+	A	G	0.516223668	0.034259	+	A	G	0.545	0.0384
rs7142219	LC_IGP79	IgG1 BG1n/G1n	14	66241781	1	4.72E-11	9.58E-12	-	-0.227819	-0.2619	+	A	G	0.516223668	0.0343886	+	A	G	0.545	0.0384
rs7142219	LC_IGP77	IgG1 Bn	14	66241781	1	1.64E-11	1.26E-11	-	-0.233191	-0.2614	+	A	G	0.516223668	0.0343734	+	A	G	0.545	0.0386
rs7142219	LC_IGP86	IgG1 Bn/Fn total	14	66241781	1	3.35E-12	7.25E-12	-	-0.240883	-0.2646	+	A	G	0.516223668	0.0343328	+	A	G	0.545	0.0386
rs7142219	LC_IGP84	IgG1 FBn/Bn total	14	66241781	1	8.01E-12	1.14E-15	+	0.237138	0.3122	+	A	G	0.516223668	0.0344207	+	A	G	0.545	0.039
rs7142219	LC_IGP62	IgG1 FG0n total/G0n	14	66241781	1	6.57E-14	2.91E-12	+	0.258137	0.2704	+	A	G	0.516223668	0.034131	+	A	G	0.545	0.0387
rs7142219	LC_IGP63	IgG1 FG1n total/G1n	14	66241781	1	6.75E-14	4.37E-14	+	0.258778	0.2925	+	A	G	0.516223668	0.0342322	+	A	G	0.545	0.0387
rs7142219	LC_IGP64	IgG1 FG2n total/G2n	14	66241781	1	1.07E-10	4.78E-15	+	0.223714	0.3017	+	A	G	0.516223668	0.0344218	+	A	G	0.545	0.0385
rs7142219	LC_IGP68	IgG1 FG2n/G2n	14	66241781	1	6.82E-09	5.17E-09	+	0.201002	0.224	+	A	G	0.516223668	0.0344972	+	A	G	0.545	0.0383
rs7142219	LC_IGP61	IgG1 Fn total	14	66241781	1	1.69E-13	6.45E-14	+	0.254481	0.2906	+	A	G	0.516223668	0.0342273	+	A	G	0.545	0.0388
rs7142219	LC_IGP81	IgG1 Fn/Bn	14	66241781	1	5.21E-12	6.37E-11	+	0.238668	0.2511	+	A	G	0.516223668	0.0343306	+	A	G	0.545	0.0384
rs7142219	LC_IGP21	IgG1 Fucosylation	14	66241781	1	1.97E-12	3.67E-13	+	0.242752	0.2816	+	A	G	0.514935882	0.0342306	+	A	G	0.545	0.0388
rs7142219	LC_IGP28	IgG1 G1S1/(G1+G1S1)	14	66241781	1	2.61E-08	7.44E-10	+	0.191135	0.229	+	A	G	0.515122936	0.0341735	+	A	G	0.545	0.0372
rs7142219	LC_IGP11	IgG1_G0	14	66241781	1	2.72E-14	1.71E-14	-	-0.260084	-0.2963	+	A	G	0.515409896	0.0338642	+	A	G	0.545	0.0386
rs7142219	LC_IGP_R1	IgG1_G0F/IgG1_G0	14	66241781	1	2.03E-13	7.15E-12	+	0.251099	0.2646	+	A	G	0.515409896	0.0338899	+	A	G	0.545	0.0386
rs7142219	LC_IGP_R4	IgG1_G0FN/IgG1_G0N	14	66241781	1	1.23E-11	1.97E-13	+	0.23298	0.286	+	A	G	0.51572872	0.0341331	+	A	G	0.545	0.0389
rs7142219	LC_IGP52	IgG1_G0n	14	66241781	1	2.70E-14	1.30E-14	-	-0.261866	-0.2982	+	A	G	0.516223668	0.0340879	+	A	G	0.545	0.0387
rs7142219	LC_IGP14	IgG1_G0N	14	66241781	1	1.52E-14	4.40E-12	-	-0.263643	-0.2658	+	A	G	0.51572872	0.0339873	+	A	G	0.545	0.0384
rs7142219	LC_IGP55	IgG1_G0Nn	14	66241781	1	2.56E-14	3.86E-12	-	-0.262825	-0.2668	+	A	G	0.516223668	0.0341819	+	A	G	0.545	0.0384
rs7142219	LC_IGP12	IgG1_G1	14	66241781	1	8.13E-13	2.65E-13	-	-0.245316	-0.2832	+	A	G	0.515409896	0.0339935	+	A	G	0.545	0.0387
rs7142219	LC_IGP_R2	IgG1_G1F/IgG1_G1	14	66241781	1	4.42E-14	3.00E-13	+	0.25853	0.2819	+	A	G	0.515409896	0.033948	+	A	G	0.545	0.0386
rs7142219	LC_IGP_R5	IgG1_G1FN/IgG1_G1N	14	66241781	1	2.32E-11	1.05E-16	+	0.230854	0.3219	+	A	G	0.516718942	0.0342927	+	A	G	0.545	0.0388
rs7142219	LC_IGP53	IgG1_G1n	14	66241781	1	4.82E-12	3.91E-13	-	-0.238801	-0.2809	+	A	G	0.516223668	0.0342945	+	A	G	0.545	0.0387
rs7142219	LC_IGP15	IgG1_G1N	14	66241781	1	5.11E-10	9.91E-11	-	-0.214751	-0.2487	+	A	G	0.516718942	0.0343387	+	A	G	0.545	0.0384
rs7142219	LC_IGP56	IgG1_G1Nn	14	66241781	1	4.52E-10	1.90E-10	-	-0.216075	-0.2445	+	A	G	0.516223668	0.0344421	+	A	G	0.545	0.0384
rs7142219	LC_IGP_R32	IgG1_G1S1/IgG1_G1	14	66241781	1	4.09E-08	1.11E-09	+	0.188485	0.227	+	A	G	0.515122936	0.0341877	+	A	G	0.545	0.0373
rs7142219	LC_IGP_R3	IgG1_G2F/IgG1_G2	14	66241781	1	5.96E-12	1.03E-14	+	0.236236	0.2974	+	A	G	0.515113692	0.03408	+	A	G	0.545	0.0384
rs7142219	LC_IGP_R8	IgG1_G2FS1/IgG1_G2S1	14	66241781	1	2.72E-08	2.98E-12	+	0.19125	0.2674	+	A	G	0.514799023	0.0342399	+	A	G	0.545	0.0383
rs7142219	LC_IGP_R26	IgG1_G2N/IgG1_G1N	14	66241781	1	1.74E-08	4.83E-10	+	0.194366	0.2344	+	A	G	0.516780864	0.0343112	+	A	G	0.545	0.0377
rs7142219	LC_IGP97	IgG2_G0	14	66241781	1	1.05E-08	8.76E-13	-	-0.19641	-0.2757	+	A	G	0.515113692	0.034145	+	A	G	0.545	0.0386
rs7142219	LC_IGP138	IgG2_G0n	14	66241781	1	4.98E-09	4.94E-13	-	-0.201382	-0.279	+	A	G	0.514301724	0.0342497	+	A	G	0.5451	0.0386
rs12050182	LC_IGP78	IgG1 BG0n/G0n	14	66243080	1	3.51E-12	3.25E-10	-	-0.240014	-0.2414	+	T	C	0.515745353	0.0342418	+	T	C	0.5404	0.0384
rs12050182	LC_IGP79	IgG1 BG1n/G1n	14	66243080	1	3.96E-11	1.52E-11	-	-0.228631	-0.259	+	T	C	0.515745353	0.034372	+	T	C	0.5404	0.0384
rs12050182	LC_IGP77	IgG1 Bn	14	66243080	1	1.36E-11	1.92E-11	-	-0.234035	-0.2587	+	T	C	0.515745353	0.0343565	+	T	C	0.5404	0.0386
rs12050182	LC_IGP86	IgG1 Bn/Fn total	14	66243080	1	2.72E-12	1.10E-11	-	-0.241786	-0.262	+	T	C	0.515745353	0.0343155	+	T	C	0.5404	0.0386
rs12050182	LC_IGP84	IgG1 FBn/Bn total	14	66243080	1	6.24E-12	1.38E-15	+	0.23827	0.3105	+	T	C	0.515745353	0.0344025	+	T	C	0.5404	0.0389
rs12050182	LC_IGP62	IgG1 FG0n total/G0n	14	66243080	1	4.92E-14	3.46E-12	+	0.259321	0.2692	+	T	C	0.515745353	0.0341121	+	T	C	0.5404	0.0387
rs12050182	LC_IGP63	IgG1 FG1n total/G1n	14	66243080	1	5.16E-14	6.12E-14	+	0.259876	0.2903	+	T	C	0.515745353	0.0342137	+	T	C	0.5404	0.0387
rs12050182	LC_IGP64	IgG1 FG2n total/G2n	14	66243080	1	8.28E-11	7.77E-15	+	0.224968	0.2989	+	T	C	0.515745353	0.0344034	+	T	C	0.5404	0.0385
rs12050182	LC_IGP68	IgG1 FG2n/G2n	14	66243080	1	5.62E-09	9.46E-09	+	0.202036	0.22	+	T	C	0.515745353	0.0344802	+	T	C	0.5404	0.0383
rs12050182	LC_IGP61	IgG1 Fn total	14	66243080	1	1.29E-13	8.95E-14	+	0.255603	0.2886	+	T	C	0.515745353	0.0342088	+	T	C	0.5404	0.0387
rs12050182	LC_IGP81	IgG1 Fn/Bn	14	66243080	1	4.33E-12	1.02E-10	+	0.239475	0.2482	+	T	C	0.515745353	0.0343138	+	T	C	0.5404	0.0384
rs12050182	LC_IGP21	IgG1 Fucosylation	14	66243080	1	1.53E-12	5.01E-13	+	0.243861	0.2796	+	T	C	0.514462392	0.0342129	+	T	C	0.5404	0.0387
rs12050182	LC_IGP28	IgG1 G1S1/(G1+G1S1)	14	66243080	1	1.93E-08	6.61E-10	+	0.192841	0.2296	+	T	C	0.514653211	0.0341551	+	T	C	0.5404	0.0372
rs12050182	LC_IGP11	IgG1_G0	14	66243080	1	1.80E-14	2.25E-14	-	-0.261781	-0.2946	+	T	C	0.514940745	0.0338435	+	T	C	0.5404	0.0386
rs12050182	LC_IGP_R1	IgG1_G0F/IgG1_G0	14	66243080	1	1.55E-13	8.78E-12	+	0.252217	0.2633	+	T	C	0.514940745	0.0338722	+	T	C	0.5404	0.0386

rs12050182	LC_IGP_R4	IgG1_G0FN/IgG1_G0N	14	66243080	1	9.28E-12	2.14E-13	+	0.234272	0.2849	+	T	C	0.515255971	0.0341146	+	T	C	0.5404	0.0388
rs12050182	LC_IGP52	IgG1_G0n	14	66243080	1	1.78E-14	1.76E-14	-	-0.263575	-0.2964	+	T	C	0.515745353	0.0340663	+	T	C	0.5404	0.0386
rs12050182	LC_IGP14	IgG1_G0N	14	66243080	1	1.05E-14	5.76E-12	-	-0.265119	-0.264	+	T	C	0.515255971	0.0339672	+	T	C	0.5404	0.0383
rs12050182	LC_IGP55	IgG1_G0Nn	14	66243080	1	1.76E-14	5.24E-12	-	-0.264358	-0.2648	+	T	C	0.515745353	0.0341612	+	T	C	0.5404	0.0384
rs12050182	LC_IGP12	IgG1_G1	14	66243080	1	6.47E-13	3.13E-13	-	-0.246283	-0.2818	+	T	C	0.514940745	0.0339766	+	T	C	0.5404	0.0387
rs12050182	LC_IGP_R2	IgG1_G1F/IgG1_G1	14	66243080	1	3.38E-14	4.28E-13	+	0.259614	0.2797	+	T	C	0.514940745	0.0339302	+	T	C	0.5404	0.0386
rs12050182	LC_IGP_R5	IgG1_G1FN/IgG1_G1N	14	66243080	1	1.87E-11	1.36E-16	+	0.231824	0.3199	+	T	C	0.516244157	0.0342754	+	T	C	0.5404	0.0387
rs12050182	LC_IGP53	IgG1_G1n	14	66243080	1	3.92E-12	4.81E-13	-	-0.239707	-0.2794	+	T	C	0.515745353	0.0342773	+	T	C	0.5404	0.0386
rs12050182	LC_IGP15	IgG1_G1N	14	66243080	1	4.48E-10	1.38E-10	-	-0.215367	-0.2464	+	T	C	0.516244157	0.0343231	+	T	C	0.5404	0.0384
rs12050182	LC_IGP56	IgG1_G1Nn	14	66243080	1	3.98E-10	2.75E-10	-	-0.216674	-0.242	+	T	C	0.515745353	0.0344264	+	T	C	0.5404	0.0383
rs12050182	LC_IGP_R32	IgG1_G1S1/IgG1_G1	14	66243080	1	3.03E-08	9.75E-10	+	0.190212	0.2276	+	T	C	0.514653211	0.0341693	+	T	C	0.5404	0.0372
rs12050182	LC_IGP_R3	IgG1_G2F/IgG1_G2	14	66243080	1	4.46E-12	1.66E-14	+	0.237542	0.2945	+	T	C	0.514644254	0.0340619	+	T	C	0.5404	0.0384
rs12050182	LC_IGP_R8	IgG1_G2FS1/IgG1_G2S1	14	66243080	1	2.25E-08	3.92E-12	+	0.192314	0.2657	+	T	C	0.514329872	0.0342238	+	T	C	0.5404	0.0383
rs12050182	LC_IGP_R26	IgG1_G2N/IgG1_G1N	14	66243080	1	1.33E-08	5.70E-10	+	0.19588	0.2328	+	T	C	0.516304321	0.0342926	+	T	C	0.5404	0.0376
rs12050182	LC_IGP97	IgG2_G0	14	66243080	1	8.77E-09	8.11E-13	-	-0.197374	-0.2754	+	T	C	0.514644254	0.0341291	+	T	C	0.5404	0.0385
rs12050182	LC_IGP138	IgG2_G0n	14	66243080	1	4.15E-09	4.80E-13	-	-0.202342	-0.2785	+	T	C	0.513828818	0.0342337	+	T	C	0.5405	0.0385
rs8022764	LC_IGP78	IgG1 BG0n/G0n	14	66244655	1	4.49E-12	5.01E-10	-	-0.239076	-0.2434	+	G	T	0.515317534	0.0342831	+	G	T	0.5214	0.0391
rs8022764	LC_IGP79	IgG1 BG1n/G1n	14	66244655	1	4.64E-11	3.71E-11	-	-0.228063	-0.2585	+	G	T	0.515317534	0.0344116	+	G	T	0.5214	0.0391
rs8022764	LC_IGP77	IgG1 Bn	14	66244655	1	1.71E-11	4.21E-11	-	-0.233127	-0.2589	+	G	T	0.515317534	0.0343976	+	G	T	0.5214	0.0392
rs8022764	LC_IGP86	IgG1 Bn/Fn total	14	66244655	1	3.45E-12	2.83E-11	-	-0.240901	-0.2613	+	G	T	0.515317534	0.0343567	+	G	T	0.5214	0.0393
rs8022764	LC_IGP84	IgG1 FBn/Bn total	14	66244655	1	8.03E-12	3.89E-15	+	0.237282	0.311	+	G	T	0.515317534	0.0344441	+	G	T	0.5214	0.0396
rs8022764	LC_IGP62	IgG1 FG0n total/G0n	14	66244655	1	6.50E-14	7.84E-12	+	0.258365	0.2699	+	G	T	0.515317534	0.0341539	+	G	T	0.5214	0.0394
rs8022764	LC_IGP63	IgG1 FG1n total/G1n	14	66244655	1	6.24E-14	2.59E-13	+	0.259305	0.2879	+	G	T	0.515317534	0.0342537	+	G	T	0.5214	0.0394
rs8022764	LC_IGP64	IgG1 FG2n total/G2n	14	66244655	1	9.72E-11	3.27E-14	+	0.22438	0.2963	+	G	T	0.515317534	0.0344431	+	G	T	0.5214	0.039
rs8022764	LC_IGP68	IgG1 FG2n/G2n	14	66244655	1	6.14E-09	1.71E-08	+	0.201741	0.22	+	G	T	0.515317534	0.0345184	+	G	T	0.5214	0.039
rs8022764	LC_IGP61	IgG1 Fn total	14	66244655	1	1.64E-13	3.51E-13	+	0.254796	0.2866	+	G	T	0.515317534	0.0342498	+	G	T	0.5214	0.0394
rs8022764	LC_IGP81	IgG1 Fn/Bn	14	66244655	1	5.44E-12	2.04E-10	+	0.238618	0.2487	+	G	T	0.515317534	0.0343548	+	G	T	0.5214	0.0391
rs8022764	LC_IGP21	IgG1 Fucosylation	14	66244655	1	1.84E-12	1.28E-12	+	0.243255	0.2796	+	G	T	0.514024661	0.0342531	+	G	T	0.5214	0.0394
rs8022764	LC_IGP28	IgG1 G1S1/(G1+G1S1)	14	66244655	1	2.10E-08	3.29E-09	+	0.192548	0.2239	+	G	T	0.51421896	0.0341927	+	G	T	0.5214	0.0378
rs8022764	LC_IGP11	IgG1_G0	14	66244655	1	2.30E-14	3.02E-14	-	-0.260991	-0.2986	+	G	T	0.514507025	0.033884	+	G	T	0.5214	0.0393
rs8022764	LC_IGP_R1	IgG1_G0F/IgG1_G0	14	66244655	1	2.04E-13	2.03E-11	+	0.251243	0.2637	+	G	T	0.514507025	0.0339134	+	G	T	0.5214	0.0393
rs8022764	LC_IGP_R4	IgG1_G0FN/IgG1_G0N	14	66244655	1	1.18E-11	3.55E-13	+	0.233359	0.2878	+	G	T	0.514819045	0.0341551	+	G	T	0.5214	0.0396
rs8022764	LC_IGP52	IgG1_G0n	14	66244655	1	2.27E-14	2.29E-14	-	-0.262796	-0.3006	+	G	T	0.515317534	0.0341073	+	G	T	0.5214	0.0394
rs8022764	LC_IGP14	IgG1_G0N	14	66244655	1	1.33E-14	5.16E-12	-	-0.264394	-0.2694	+	G	T	0.514819045	0.0340073	+	G	T	0.5214	0.039
rs8022764	LC_IGP55	IgG1_G0Nn	14	66244655	1	2.30E-14	4.60E-12	-	-0.263483	-0.2703	+	G	T	0.515317534	0.0342028	+	G	T	0.5214	0.0391
rs8022764	LC_IGP12	IgG1_G1	14	66244655	1	7.89E-13	1.62E-12	-	-0.24562	-0.2779	+	G	T	0.514507025	0.0340163	+	G	T	0.5214	0.0393
rs8022764	LC_IGP_R2	IgG1_G1F/IgG1_G1	14	66244655	1	4.08E-14	2.01E-12	+	0.259059	0.2762	+	G	T	0.514507025	0.0339697	+	G	T	0.5214	0.0393
rs8022764	LC_IGP_R5	IgG1_G1FN/IgG1_G1N	14	66244655	1	2.23E-11	3.67E-16	+	0.231198	0.3208	+	G	T	0.515805351	0.0343147	+	G	T	0.5214	0.0394
rs8022764	LC_IGP53	IgG1_G1n	14	66244655	1	4.71E-12	2.30E-12	-	-0.23908	-0.2758	+	G	T	0.515317534	0.0343173	+	G	T	0.5214	0.0393
rs8022764	LC_IGP15	IgG1_G1N	14	66244655	1	5.19E-10	3.78E-10	-	-0.214807	-0.2447	+	G	T	0.515805351	0.0343619	+	G	T	0.5214	0.0391
rs8022764	LC_IGP56	IgG1_G1Nn	14	66244655	1	4.64E-10	7.20E-10	-	-0.216079	-0.2404	+	G	T	0.515317534	0.0344659	+	G	T	0.5214	0.039
rs8022764	LC_IGP_R32	IgG1_G1S1/IgG1_G1	14	66244655	1	3.29E-08	4.73E-09	+	0.189919	0.222	+	G	T	0.51421896	0.0342069	+	G	T	0.5214	0.0379
rs8022764	LC_IGP_R3	IgG1_G2F/IgG1_G2	14	66244655	1	5.19E-12	7.18E-14	+	0.237065	0.2917	+	G	T	0.514210269	0.0341007	+	G	T	0.5214	0.039
rs8022764	LC_IGP_R8	IgG1_G2FS1/IgG1_G2S1	14	66244655	1	2.70E-08	1.88E-11	+	0.191443	0.2612	+	G	T	0.513896151	0.0342634	+	G	T	0.5214	0.0389
rs8022764	LC_IGP_R26	IgG1_G2N/IgG1_G1N	14	66244655	1	1.45E-08	1.93E-10	+	0.195555	0.244	+	G	T	0.515878086	0.0343305	+	G	T	0.5214	0.0383
rs8022764	LC_IGP97	IgG2_G0	14	66244655	1	1.02E-08	4.78E-12	-	-0.196708	-0.2704	+	G	T	0.514210269	0.034168	+	G	T	0.5214	0.0391
rs8022764	LC_IGP138	IgG2_G0n	14	66244655	1	4.85E-09	2.99E-12	-	-0.201669	-0.2734	+	G	T	0.513405788	0.0342733	+	G	T	0.5214	0.0392
rs11625882	LC_IGP78	IgG1 BG0n/G0n	14	66245199	1	4.48E-12	2.64E-10	-	-0.239018	-0.2419	+	A	G	0.516209727	0.0342726	+	A	G	0.543	0.0383
rs11625882	LC_IGP79	IgG1 BG1n/G1n	14	66245199	1	4.87E-11	1.31E-11	-	-0.22775	-0.259	+	A	G	0.516209727	0.0344021	+	A	G	0.543	0.0383
rs11625882	LC_IGP77	IgG1 Bn	14	66245199	1	1.69E-11	1.59E-11	-	-0.233127	-0.259	+	A	G	0.516209727	0.0343869	+	A	G	0.543	0.0384
rs11625882	LC_IGP86	IgG1 Bn/Fn total	14	66245199	1	3.47E-12	9.19E-12	-	-0.240804	-0.2621	+	A	G	0.516209727	0.0343464	+	A	G	0.543	0.0384
rs11625882	LC_IGP84	IgG1 FBn/Bn total	14	66245199	1	8.37E-12	9.77E-16	+	0.237008	0.3112	+	A	G	0.516209727	0.0344345	+	A	G	0.543	0.0388
rs11625882	LC_IGP62	IgG1 FG0n total/G0n	14	66245199	1	7.04E-14	2.61E-12	+	0.257926	0.2699	+	A	G	0.516209727	0.0341452	+	A	G	0.543	0.0386
rs11625882	LC_IGP63	IgG1 FG1n total/G1n	14	66245199	1	7.22E-14	5.22E-14	+	0.258575	0.2902	+	A	G	0.516209727	0.0342464	+	A	G	0.543	0.0386
rs11625882	LC_IGP64	IgG1 FG2n total/G2n	14	66245199	1	1.16E-10	5.35E-15	+	0.223417	0.2996	+	A	G	0.516209727	0.0344363	+	A	G	0.543	0.0383
rs11625882	LC_IGP68	IgG1 FG2n/G2n	14	66245199	1	7.23E-09	7.79E-09	+	0.200741	0.2204	+	A	G	0.516209727	0.0345113	+	A	G	0.543	0.0382
rs11625882	LC_IGP61	IgG1 Fn total	14	66245199	1	1.82E-13	6.97E-14	+	0.254236	0.2889	+	A	G	0.516209727	0.0342417	+	A	G	0.543	0.0386
rs11625882	LC_IGP81	IgG1 Fn/Bn	14	66245199	1	5.39E-12	8.30E-11	+	0.238598	0.2486	+	A	G	0.516209727	0.0343442	+	A	G	0.543	0.0383
rs11625882	LC_IGP21	IgG1 Fucosylation	14	66245199	1	2.06E-12	3.67E-13	+	0.24264	0.2803	+	A	G	0.51492725	0.034245	+	A	G	0.543	0.0386
rs11625882	LC_IGP28	IgG1 G1S1/(G1+G1S1)	14	66245199	1	2.70E-08	6.46E-10	+	0.191018	0.2292	+	A	G	0.515114373	0.0341874	+	A	G	0.543	0.0371
rs11625882	LC_IGP11	IgG1_G0	14	66245199	1	2.89E-14	1.63E-14	-	-0.259925	-0.2951	+	A	G	0.515401344	0.0338785	+	A	G	0.543	0.0384
rs11625882	LC_IGP_R1	IgG1_G0F/IgG1_G0	14	66245199	1	2.18E-13	6.55E-12	+	0.250864	0.2641	+	A	G	0.515401344	0.0339046	+	A	G	0.543	0.0384
rs11625882	LC_IGP_R4	IgG1_G0FN/IgG1_G0N	14	66245199	1	1.28E-11	1.54E-13	+	0.232856	0.2858	+	A	G	0.515714942	0.0341466	+	A	G	0.543	0.0387
rs11625882	LC_IGP52	IgG1_G0n	14	66245199	1	2.86E-14	1.24E-14	-	-0.261708	-0.297	+	A	G	0.516209727	0.0341018	+	A	G	0.543	0.0385
rs11625882	LC_IGP14	IgG1_G0N	14	66245199	1	1.55E-14	4.49E-12	-	-0.263649	-0.2644	+	A	G	0.515714942	0.0340003	+	A	G	0.543	0.0382
rs11625882	LC_IGP55	IgG1_G0Nn	14	66245199	1	2.62E-14	4.01E-12	-	-0.262825	-0.2653	+	A	G	0.516209727	0.0341952	+	A	G	0.543	0.0382
rs11625882	LC_IGP12	IgG1_G1	14	66245199	1	8.73E-13	2.88E-13	-	-0.245082	-0.2813	+	A	G	0.515401344	0.0340082	+	A	G	0.543	0.0385
rs11625882	LC_IGP_R2	IgG1_G1F/IgG1_G1	14	66245199	1	4.72E-14	3.63E-13	+	0.258336	0.2796	+	A	G	0.515401344	0.0339625	+	A	G	0.543	0.0385
rs11625882	LC_IGP_R5	IgG1_G1FN/IgG1_G1N	14	66245199	1	2.40E-11	1.08E-16	+	0.230756	0.32	+	A	G	0.516705105	0.0343062	+	A	G	0.543	0.0386
rs11625882	LC_IGP53	IgG1_G1n	14	66245199	1	5.19E-12	4.32E-13	-	-0.238538	-0.279	+	A	G	0.516209727	0.0343089	+	A	G	0.543	0.0385
rs11625882	LC_IGP15	IgG1_G1N	14	66245199	1	5.28E-10	1.28E-10	-	-0.214656	-0.246	+	A	G	0.516705105	0.0343522	+	A	G	0.543	0.0383
rs11625882	LC_IGP56	IgG1_G1Nn	14	66245199	1	4.68E-10	2.49E-10	-	-0.215974	-0.2418	+	A	G	0.516209727	0.0344557	+	A	G	0.543	0.0382
rs11625882	LC_IGP_R32	IgG1_G1S1/IgG1_G1	14	66245199	1	4.21E-08	9.58E-10	+	0.188379	0.2272	+	A	G	0.515114373	0.0342016	+	A	G	0.543	0.0372
rs11625882	LC_IGP_R3	IgG1_G2F/IgG1_G2	14	66245199	1	6.44E-12	1.16E-14	+	0.235953	0.2952	+	A	G	0.515105134	0.0340949	+	A	G	0.543	0.0382
rs11625882	LC_IGP_R8	IgG1_G2FS1/IgG1_G2S1	14	66245199	1	2.82E-08	3.03E-12	+	0.191116	0.266	+	A	G	0.51479047	0.0342539	+	A	G	0.543	0.0381
rs11625882	LC_IGP_R26	IgG1_G2N/IgG1_G1N	14	66245199	1	1.76E-08	6.69E-10	+	0.194355	0.2312	+	A	G	0.516766975	0.0343243	+	A	G	0.543	0.0374
rs11625882	LC_IGP97	IgG2_G0	14	66245199	1	1.10E-08	8.99E-13	-	-0.196219	-0.2739	+	A	G	0.515105134	0.0341592	+	A	G	0.543	0.0383
rs11625882	LC_IGP138	IgG2_G0n	14	66245199	1	5.21E-09	5.10E-13	-	-0.201206	-0.2772	+	A	G	0.514293103	0.0342641	+	A	G	0.543	0.0384
rs12887957	LC_IGP78	IgG1 BG0n/G0n	14	66246592	1	4.50E-12	2.93E-10	-	-0.239028	-0.2415	+	A	G	0.516192069	0.0342773	+	A	G	0.5415	0.0383
rs12887957	LC_IGP79	IgG1 BG1n/G1n	14	66246592	1	4.88E-11	1.45E-11	-	-0.227775	-0.2586	+	A	G	0.516192069	0.0344067	+	A	G	0.5415	0.0383
rs12887957	LC_IGP77	IgG1 Bn	14	66246592	1	1.69E-11	1.69E-11	-	-0.233156	-0.2588	+	A	G	0.516192069	0.0343915	+	A	G	0.5415	0.0384
rs12887957	LC_IGP86	IgG1 Bn/Fn total	14	66246592	1	3.47E-12	9.97E-12	-	-0.240825	-0.2618	+	A	G	0.516192069	0.034351	+	A	G	0.5415	0.0385
rs12887957	LC_IGP84	IgG1 FBn/Bn total	14	66246592	1	8.48E-12	1.12E-15	+	0.236977	0.3108	+	A	G	0.516192069	0.0344394	+	A	G	0.5415	0.0388
rs12887957	LC_IGP62	IgG1 FG0n total/G0n	14	66246592	1	7.17E-14	3.20E-12	+	0.257881	0.269	+	A	G	0.516192069	0.0341501	+	A	G	0.5415	0.0386
rs12887957	LC_IGP63	IgG1 FG1n total/G1n	14	66246592	1	7.34E-14	6.11E-14	+	0.258536	0.2896	+	A	G	0.516192069	0.0342513	+	A	G	0.5415	0.0386
rs12887957	LC_IGP64	IgG1 FG2n total/G2n	14	66246592	1	1.17E-10	6.62E-15	+	0.22336	0.2988	+	A	G	0.516192069	0.0344412	+	A	G	0.5415	0.0384
rs12887957	LC_IGP68	IgG1 FG2n/G2n	14	66246592	1	7.27E-09	9.89E-09	+	0.200729	0.2191	+	A	G	0.516192069	0.034516	+	A	G	0.5415	0.0382
rs12887957	LC_IGP61	IgG1 Fn total	14	66246592	1	1.86E-13	8.43E-14	+	0.254187	0.2882	+	A	G	0.516192069	0.0342466	+	A	G	0.5415	0.0386
rs12887957	LC_IGP81	IgG1 Fn/Bn	14	66246592	1	5.38E-12	9.24E-11	+	0.238632	0.2482	+	A	G	0.516192069	0.0343487	+	A	G	0.5415	0.0383
rs12887957	LC_IGP21	IgG1 Fucosylation	14	66246592	1	2.08E-12	4.19E-13	+	0.242634	0.2798	+	A	G	0.514911221	0.0342499	+	A	G	0.5415	0.0386
rs12887957	LC_IGP28	IgG1 G1S1/(G1+G1S1)	14	66246592	1	2.73E-08	8.21E-10	+	0.190973	0.2281	+	A	G	0.515098471	0.0341923	+	A	G	0.5415	0.0371
rs12887957	LC_IGP11	IgG1_G0	14	66246592	1	2.93E-14	1.74E-14	-	-0.259891	-0.295	+	A	G	0.515385461	0.0338835	+	A	G	0.5415	0.0385
rs12887957	LC_IGP_R1	IgG1_G0F/IgG1_G0	14	66246592	1	2.22E-13	8.28E-12	+	0.250814	0.263	+	A	G	0.515385461	0.0339096	+	A	G	0.5415	0.0385
rs12887957	LC_IGP_R4	IgG1_G0FN/IgG1_G0N	14	66246592	1	1.30E-11	1.77E-13	+	0.23282	0.2853	+	A	G	0.515697489	0.0341514	+	A	G	0.5415	0.0387
rs12887957	LC_IGP52	IgG1_G0n	14	66246592	1	2.91E-14	1.34E-14	-	-0.261675	-0.2968	+	A	G	0.516192069	0.0341067	+	A	G	0.5415	0.0385
rs12887957	LC_IGP14	IgG1_G0N	14	66246592	1	1.55E-14	4.39E-12	-	-0.263693	-0.2648	+	A	G	0.515697489	0.0340047	+	A	G	0.5415	0.0382
rs12887957	LC_IGP55	IgG1_G0Nn	14	66246592	1	2.62E-14	3.94E-12	-	-0.262869	-0.2656	+	A	G	0.516192069	0.0341997	+	A	G	0.5415	0.0383
rs12887957	LC_IGP12	IgG1_G1	14	66246592	1	8.91E-13	3.33E-13	-	-0.245017	-0.2808	+	A	G	0.515385461	0.0340132	+	A	G	0.5415	0.0386
rs12887957	LC_IGP_R2	IgG1_G1F/IgG1_G1	14	66246592	1	4.80E-14	4.45E-13	+	0.258302	0.2787	+	A	G	0.515385461	0.0339675	+	A	G	0.5415	0.0385
rs12887957	LC_IGP_R5	IgG1_G1FN/IgG1_G1N	14	66246592	1	2.43E-11	1.16E-16	+	0.230733	0.3199	+	A	G	0.516687577	0.0343109	+	A	G	0.5415	0.0386
rs12887957	LC_IGP53	IgG1_G1n	14	66246592	1	5.30E-12	5.01E-13	-	-0.238466	-0.2784	+	A	G	0.516192069	0.0343139	+	A	G	0.5415	0.0385
rs12887957	LC_IGP15	IgG1_G1N	14	66246592	1	5.29E-10	1.40E-10	-	-0.214668	-0.2457	+	A	G	0.516687577	0.0343567	+	A	G	0.5415	0.0383
rs12887957	LC_IGP56	IgG1_G1Nn	14	66246592	1	4.69E-10	2.74E-10	-	-0.215987	-0.2414	+	A	G	0.516192069	0.0344603	+	A	G	0.5415	0.0382
rs12887957	LC_IGP_R32	IgG1_G1S1/IgG1_G1	14	66246592	1	4.26E-08	1.22E-09	+	0.188338	0.226	+	A	G	0.515098471	0.0342065	+	A	G	0.5415	0.0372
rs12887957	LC_IGP_R3	IgG1_G2F/IgG1_G2	14	66246592	1	6.56E-12	1.50E-14	+	0.2359	0.2942	+	A	G	0.515089242	0.0340999	+	A	G	0.5415	0.0383
rs12887957	LC_IGP_R8	IgG1_G2FS1/IgG1_G2S1	14	66246592	1	2.82E-08	4.22E-12	+	0.191135	0.2644	+	A	G	0.514774588	0.0342586	+	A	G	0.5415	0.0382
rs12887957	LC_IGP_R26	IgG1_G2N/IgG1_G1N	14	66246592	1	1.77E-08	5.56E-10	+	0.194344	0.2325	+	A	G	0.516749383	0.0343289	+	A	G	0.5415	0.0375
rs12887957	LC_IGP97	IgG2_G0	14	66246592	1	1.11E-08	6.69E-13	-	-0.196191	-0.2757	+	A	G	0.515089242	0.0341641	+	A	G	0.5415	0.0384
rs12887957	LC_IGP138	IgG2_G0n	14	66246592	1	5.25E-09	3.84E-13	-	-0.201182	-0.279	+	A	G	0.514277094	0.034269	+	A	G	0.5416	0.0384
rs2236067	LC_IGP78	IgG1 BG0n/G0n	14	66248012	1	1.32E-12	5.06E-07	-	-0.250099	-0.198	+	G	A	0.596580545	0.0349827	+	G	A	0.62	0.0394
rs2236067	LC_IGP79	IgG1 BG1n/G1n	14	66248012	1	3.22E-11	6.72E-07	-	-0.234783	-0.1956	+	G	A	0.596580545	0.0351322	+	G	A	0.62	0.0394
rs2236067	LC_IGP77	IgG1 Bn	14	66248012	1	6.08E-12	2.60E-07	-	-0.243255	-0.2037	+	G	A	0.596580545	0.0351037	+	G	A	0.62	0.0396
rs2236067	LC_IGP86	IgG1 Bn/Fn total	14	66248012	1	1.19E-12	1.77E-07	-	-0.251161	-0.2066	+	G	A	0.596580545	0.0350613	+	G	A	0.62	0.0396
rs2236067	LC_IGP84	IgG1 FBn/Bn total	14	66248012	1	1.45E-12	2.48E-10	+	0.250735	0.2522	+	G	A	0.596580545	0.0351366	+	G	A	0.62	0.0399
rs2236067	LC_IGP62	IgG1 FG0n total/G0n	14	66248012	1	2.16E-15	1.01E-08	+	0.278829	0.2274	+	G	A	0.596580545	0.0348045	+	G	A	0.62	0.0397
rs2236067	LC_IGP63	IgG1 FG1n total/G1n	14	66248012	1	1.03E-13	1.18E-08	+	0.262504	0.226	+	G	A	0.596580545	0.0349896	+	G	A	0.62	0.0396
rs2236067	LC_IGP64	IgG1 FG2n total/G2n	14	66248012	1	7.11E-11	9.66E-10	+	0.230779	0.2408	+	G	A	0.596580545	0.0351655	+	G	A	0.62	0.0394
rs2236067	LC_IGP61	IgG1 Fn total	14	66248012	1	5.35E-14	3.71E-09	+	0.265309	0.234	+	G	A	0.596580545	0.034951	+	G	A	0.62	0.0397
rs2236067	LC_IGP81	IgG1 Fn/Bn	14	66248012	1	2.65E-12	7.32E-07	+	0.247203	0.1952	+	G	A	0.596580545	0.0350667	+	G	A	0.62	0.0394

rs2236067	LC_IGP21	IgG1 Fucosylation	14	66248012	1	1.34E-12	5.74E-09	+	0.249159	0.231	+	G	A	0.595522503	0.0348659	+	G	A	0.62	0.0396
rs2236067	LC_IGP11	IgG1_G0	14	66248012	1	1.43E-15	6.08E-11	-	-0.278002	-0.2587	+	G	A	0.596174404	0.0344799	+	G	A	0.62	0.0395
rs2236067	LC_IGP_R1	IgG1_G0F/IgG1_G0	14	66248012	1	6.73E-15	2.21E-08	+	0.271265	0.2214	+	G	A	0.596174404	0.0344966	+	G	A	0.62	0.0396
rs2236067	LC_IGP_R4	IgG1_G0FN/IgG1_G0N	14	66248012	1	4.56E-12	1.31E-09	+	0.242708	0.2415	+	G	A	0.596375383	0.0348184	+	G	A	0.62	0.0398
rs2236067	LC_IGP52	IgG1_G0n	14	66248012	1	8.32E-16	6.08E-11	-	-0.28267	-0.2592	+	G	A	0.596580545	0.0347591	+	G	A	0.62	0.0396
rs2236067	LC_IGP14	IgG1_G0N	14	66248012	1	1.64E-14	1.49E-08	-	-0.268767	-0.2227	+	G	A	0.596375383	0.0346919	+	G	A	0.62	0.0393
rs2236067	LC_IGP55	IgG1_G0Nn	14	66248012	1	2.07E-14	1.75E-08	-	-0.269513	-0.2219	+	G	A	0.596580545	0.0349247	+	G	A	0.62	0.0394
rs2236067	LC_IGP12	IgG1_G1	14	66248012	1	4.25E-13	1.21E-08	-	-0.25336	-0.2258	+	G	A	0.595868968	0.0346727	+	G	A	0.62	0.0396
rs2236067	LC_IGP_R2	IgG1_G1F/IgG1_G1	14	66248012	1	5.85E-14	3.26E-08	+	0.262539	0.2186	+	G	A	0.595868968	0.0346456	+	G	A	0.62	0.0396
rs2236067	LC_IGP_R5	IgG1_G1FN/IgG1_G1N	14	66248012	1	3.07E-11	2.47E-10	+	0.234176	0.2511	+	G	A	0.597405289	0.0350073	+	G	A	0.62	0.0397
rs2236067	LC_IGP53	IgG1_G1n	14	66248012	1	4.42E-12	1.93E-08	-	-0.244448	-0.2225	+	G	A	0.596580545	0.0350424	+	G	A	0.62	0.0396
rs2236067	LC_IGP15	IgG1_G1N	14	66248012	1	5.58E-10	1.43E-06	-	-0.218702	-0.1898	+	G	A	0.597405289	0.0350501	+	G	A	0.62	0.0394
rs2236067	LC_IGP_R3	IgG1_G2F/IgG1_G2	14	66248012	1	8.17E-12	1.28E-09	+	0.239365	0.2386	+	G	A	0.595927567	0.0347624	+	G	A	0.62	0.0393
rs2236067	LC_IGP_R8	IgG1_G2FS1/IgG1_G2S1	14	66248012	1	5.85E-09	1.18E-08	+	0.204115	0.2234	+	G	A	0.595563531	0.0348784	+	G	A	0.62	0.0392
rs968540	LC_IGP78	IgG1 BG0n/G0n	14	66249064	1	9.30E-12	3.62E-07	+	0.242319	0.2022	+	T	G	0.386329616	0.0352848	+	T	G	0.3552	0.0398
rs968540	LC_IGP79	IgG1 BG1n/G1n	14	66249064	1	1.07E-10	2.84E-07	+	0.230233	0.2039	+	T	G	0.386329616	0.0354192	+	T	G	0.3552	0.0397
rs968540	LC_IGP77	IgG1 Bn	14	66249064	1	2.18E-11	1.39E-07	+	0.238594	0.21	+	T	G	0.386329616	0.0353922	+	T	G	0.3552	0.0399
rs968540	LC_IGP86	IgG1 Bn/Fn total	14	66249064	1	3.79E-12	9.61E-08	+	0.247358	0.2129	+	T	G	0.386329616	0.0353469	+	T	G	0.3552	0.0399
rs968540	LC_IGP84	IgG1 FBn/Bn total	14	66249064	1	5.07E-12	3.06E-10	-	-0.246417	-0.2531	+	T	G	0.386329616	0.0354249	+	T	G	0.3552	0.0402
rs968540	LC_IGP62	IgG1 FG0n total/G0n	14	66249064	1	1.99E-15	6.50E-09	-	-0.28125	-0.2322	+	T	G	0.386329616	0.0350616	+	T	G	0.3552	0.04
rs968540	LC_IGP63	IgG1 FG1n total/G1n	14	66249064	1	2.23E-13	4.79E-09	-	-0.260869	-0.234	+	T	G	0.386329616	0.0352664	+	T	G	0.3552	0.04
rs968540	LC_IGP64	IgG1 FG2n total/G2n	14	66249064	1	6.13E-11	2.98E-10	-	-0.233276	-0.2502	+	T	G	0.386329616	0.0354239	+	T	G	0.3552	0.0397
rs968540	LC_IGP61	IgG1 Fn total	14	66249064	1	5.29E-14	1.57E-09	-	-0.267331	-0.2416	+	T	G	0.386329616	0.0352107	+	T	G	0.3552	0.04
rs968540	LC_IGP81	IgG1 Fn/Bn	14	66249064	1	7.95E-12	2.71E-07	-	-0.243585	-0.2043	+	T	G	0.386329616	0.0353511	+	T	G	0.3552	0.0397
rs968540	LC_IGP21	IgG1 Fucosylation	14	66249064	1	1.41E-12	2.02E-09	-	-0.250891	-0.2399	+	T	G	0.386847719	0.0351398	+	T	G	0.3552	0.04
rs968540	LC_IGP11	IgG1_G0	14	66249064	1	3.42E-16	6.66E-11	+	0.286185	0.2601	+	T	G	0.386357361	0.0347201	+	T	G	0.3552	0.0398
rs968540	LC_IGP_R1	IgG1_G0F/IgG1_G0	14	66249064	1	2.26E-15	1.23E-08	-	-0.278094	-0.2273	+	T	G	0.386357361	0.0347442	+	T	G	0.3552	0.0399
rs968540	LC_IGP_R4	IgG1_G0FN/IgG1_G0N	14	66249064	1	4.66E-11	1.13E-09	-	-0.232863	-0.2445	+	T	G	0.386121249	0.0351416	+	T	G	0.3552	0.0401
rs968540	LC_IGP52	IgG1_G0n	14	66249064	1	2.76E-16	6.03E-11	+	0.289408	0.2612	+	T	G	0.386329616	0.034994	+	T	G	0.3552	0.0399
rs968540	LC_IGP14	IgG1_G0N	14	66249064	1	2.38E-13	1.93E-08	+	0.258711	0.2228	+	T	G	0.386121249	0.0350215	+	T	G	0.3552	0.0396
rs968540	LC_IGP55	IgG1_G0Nn	14	66249064	1	1.28E-13	2.02E-08	+	0.263234	0.2226	+	T	G	0.386329616	0.0352234	+	T	G	0.3552	0.0397
rs968540	LC_IGP12	IgG1_G1	14	66249064	1	2.17E-13	5.61E-09	+	0.258476	0.233	+	T	G	0.386662798	0.034929	+	T	G	0.3552	0.04
rs968540	LC_IGP_R2	IgG1_G1F/IgG1_G1	14	66249064	1	4.89E-14	9.63E-09	-	-0.265397	-0.2289	+	T	G	0.386662798	0.034912	+	T	G	0.3552	0.0399
rs968540	LC_IGP_R5	IgG1_G1FN/IgG1_G1N	14	66249064	1	6.93E-11	2.52E-10	-	-0.231694	-0.2531	+	T	G	0.385630996	0.0352862	+	T	G	0.3552	0.04
rs968540	LC_IGP53	IgG1_G1n	14	66249064	1	3.98E-12	8.15E-09	+	0.246786	0.2304	+	T	G	0.386329616	0.0353008	+	T	G	0.3552	0.04
rs968540	LC_IGP15	IgG1_G1N	14	66249064	1	1.12E-09	6.77E-07	+	0.216474	0.1974	+	T	G	0.385630996	0.0353268	+	T	G	0.3552	0.0397
rs968540	LC_IGP56	IgG1_G1Nn	14	66249064	1	5.22E-10	1.10E-06	+	0.221648	0.1934	+	T	G	0.386329616	0.0354598	+	T	G	0.3552	0.0397
rs968540	LC_IGP_R3	IgG1_G2F/IgG1_G2	14	66249064	1	5.39E-12	3.13E-10	-	-0.243302	-0.2495	+	T	G	0.386593521	0.0350251	+	T	G	0.3552	0.0396
rs968540	LC_IGP_R8	IgG1_G2FS1/IgG1_G2S1	14	66249064	1	1.35E-08	2.11E-09	-	-0.20075	-0.2368	+	T	G	0.386968235	0.0351659	+	T	G	0.3552	0.0395
rs968540	LC_IGP97	IgG2_G0	14	66249064	1	9.36E-11	2.04E-06	+	0.227954	0.189	+	T	G	0.386899144	0.0349634	+	T	G	0.3552	0.0398
rs968540	LC_IGP138	IgG2_G0n	14	66249064	1	1.55E-11	1.72E-06	+	0.23816	0.1904	+	T	G	0.387602833	0.035067	+	T	G	0.3552	0.0398
rs58102398	LC_IGP78	IgG1 BG0n/G0n	14	66250157	1	3.51E-10	1.28E-10	+	0.248116	0.2891	+	G	A	0.267814126	0.0392966	+	G	A	0.2292	0.045
rs58102398	LC_IGP79	IgG1 BG1n/G1n	14	66250157	1	3.75E-09	5.76E-12	+	0.233817	0.3095	+	G	A	0.267814126	0.0394444	+	G	A	0.2292	0.0449
rs58102398	LC_IGP77	IgG1 Bn	14	66250157	1	6.51E-10	1.09E-11	+	0.244944	0.3067	+	G	A	0.267814126	0.0394106	+	G	A	0.2292	0.0451
rs58102398	LC_IGP86	IgG1 Bn/Fn total	14	66250157	1	1.21E-10	6.06E-12	+	0.255101	0.3106	+	G	A	0.267814126	0.0393618	+	G	A	0.2292	0.0452
rs58102398	LC_IGP84	IgG1 FBn/Bn total	14	66250157	1	1.50E-11	2.01E-15	-	-0.267753	-0.3612	+	G	A	0.267814126	0.0393917	+	G	A	0.2292	0.0455
rs58102398	LC_IGP62	IgG1 FG0n total/G0n	14	66250157	1	1.54E-13	1.48E-12	-	-0.290954	-0.3203	+	G	A	0.267814126	0.0390656	+	G	A	0.2292	0.0453
rs58102398	LC_IGP63	IgG1 FG1n total/G1n	14	66250157	1	6.60E-12	1.71E-13	-	-0.271657	-0.3331	+	G	A	0.267814126	0.0392705	+	G	A	0.2292	0.0452
rs58102398	LC_IGP64	IgG1 FG2n total/G2n	14	66250157	1	4.51E-11	9.93E-15	-	-0.26101	-0.3474	+	G	A	0.267814126	0.0393573	+	G	A	0.2292	0.0449
rs58102398	LC_IGP68	IgG1 FG2n/G2n	14	66250157	1	2.84E-08	2.22E-09	-	-0.220353	-0.2676	+	G	A	0.267814126	0.0394982	+	G	A	0.2292	0.0448
rs58102398	LC_IGP61	IgG1 Fn total	14	66250157	1	7.22E-13	1.23E-13	-	-0.283503	-0.3355	+	G	A	0.267814126	0.0391901	+	G	A	0.2292	0.0453
rs58102398	LC_IGP81	IgG1 Fn/Bn	14	66250157	1	4.29E-10	2.93E-11	-	-0.247373	-0.299	+	G	A	0.267814126	0.039379	+	G	A	0.2292	0.045
rs58102398	LC_IGP21	IgG1 Fucosylation	14	66250157	1	1.50E-11	2.46E-13	-	-0.266412	-0.331	+	G	A	0.268034525	0.0391969	+	G	A	0.2292	0.0452
rs58102398	LC_IGP11	IgG1_G0	14	66250157	1	2.04E-14	2.28E-14	+	0.299286	0.3438	+	G	A	0.267716555	0.0387761	+	G	A	0.2292	0.045
rs58102398	LC_IGP_R1	IgG1_G0F/IgG1_G0	14	66250157	1	2.79E-13	6.05E-12	-	-0.285923	-0.3105	+	G	A	0.267716555	0.0388197	+	G	A	0.2292	0.0451
rs58102398	LC_IGP_R4	IgG1_G0FN/IgG1_G0N	14	66250157	1	1.28E-10	4.35E-14	-	-0.253487	-0.3426	+	G	A	0.267759951	0.03917	+	G	A	0.2292	0.0454
rs58102398	LC_IGP52	IgG1_G0n	14	66250157	1	1.43E-14	2.10E-14	+	0.302716	0.345	+	G	A	0.267814126	0.0389809	+	G	A	0.2292	0.0451
rs58102398	LC_IGP14	IgG1_G0N	14	66250157	1	1.81E-11	5.01E-12	+	0.264747	0.3098	+	G	A	0.267759951	0.0391143	+	G	A	0.2292	0.0449
rs58102398	LC_IGP55	IgG1_G0Nn	14	66250157	1	1.46E-11	5.35E-12	+	0.26697	0.3097	+	G	A	0.267814126	0.039255	+	G	A	0.2292	0.0449
rs58102398	LC_IGP12	IgG1_G1	14	66250157	1	4.66E-12	4.18E-13	+	0.271553	0.3276	+	G	A	0.268021991	0.0389739	+	G	A	0.2292	0.0452
rs58102398	LC_IGP_R2	IgG1_G1F/IgG1_G1	14	66250157	1	4.63E-12	8.07E-13	-	-0.271698	-0.3228	+	G	A	0.268021991	0.0389897	+	G	A	0.2292	0.0451
rs58102398	LC_IGP_R5	IgG1_G1FN/IgG1_G1N	14	66250157	1	1.95E-10	3.44E-16	-	-0.25155	-0.3693	+	G	A	0.267682657	0.0392646	+	G	A	0.2292	0.0453
rs58102398	LC_IGP53	IgG1_G1n	14	66250157	1	3.08E-11	9.13E-13	+	0.262735	0.3226	+	G	A	0.267814126	0.0392766	+	G	A	0.2292	0.0452
rs58102398	LC_IGP15	IgG1_G1N	14	66250157	1	9.70E-09	1.63E-11	+	0.226815	0.303	+	G	A	0.267682657	0.0393363	+	G	A	0.2292	0.045
rs58102398	LC_IGP56	IgG1_G1Nn	14	66250157	1	1.08E-08	4.45E-11	+	0.226905	0.2959	+	G	A	0.267814126	0.0394768	+	G	A	0.2292	0.0449
rs58102398	LC_IGP_R3	IgG1_G2F/IgG1_G2	14	66250157	1	8.19E-12	9.01E-15	-	-0.268699	-0.3474	+	G	A	0.267880196	0.0390243	+	G	A	0.2292	0.0448
rs58102398	LC_IGP148	IgG2 FG0n total/G0n	14	66250157	1	2.93E-08	2.41E-08	-	-0.218586	-0.2497	+	G	A	0.268320197	0.039224	+	G	A	0.2294	0.0448
rs58102398	LC_IGP97	IgG2_G0	14	66250157	1	1.73E-10	3.87E-09	+	0.250553	0.2642	+	G	A	0.267819682	0.0390001	+	G	A	0.2292	0.0449
rs58102398	LC_IGP_R41	IgG2_G0F/IgG2_G0	14	66250157	1	9.56E-09	8.17E-09	-	-0.225426	-0.2569	+	G	A	0.267819682	0.0390804	+	G	A	0.2292	0.0446
rs58102398	LC_IGP138	IgG2_G0n	14	66250157	1	1.01E-10	4.20E-09	+	0.254171	0.2638	+	G	A	0.268320197	0.0390542	+	G	A	0.2294	0.0449
rs7142165	LC_IGP78	IgG1 BG0n/G0n	14	66250232	1	5.81E-12	4.18E-10	+	0.238288	0.2401	+	A	G	0.483991945	0.034354	+	A	G	0.4576	0.0384
rs7142165	LC_IGP79	IgG1 BG1n/G1n	14	66250232	1	5.93E-11	2.45E-11	+	0.22725	0.2564	+	A	G	0.483991945	0.0344825	+	A	G	0.4576	0.0384
rs7142165	LC_IGP77	IgG1 Bn	14	66250232	1	2.05E-11	2.73E-11	+	0.23266	0.2568	+	A	G	0.483991945	0.0344672	+	A	G	0.4576	0.0386
rs7142165	LC_IGP86	IgG1 Bn/Fn total	14	66250232	1	4.41E-12	1.62E-11	+	0.24018	0.2599	+	A	G	0.483991945	0.0344275	+	A	G	0.4576	0.0386
rs7142165	LC_IGP84	IgG1 FBn/Bn total	14	66250232	1	1.21E-11	1.64E-15	-	-0.235725	-0.31	+	A	G	0.483991945	0.0345185	+	A	G	0.4576	0.0389
rs7142165	LC_IGP62	IgG1 FG0n total/G0n	14	66250232	1	1.22E-13	5.31E-12	-	-0.256032	-0.2672	+	A	G	0.483991945	0.0342323	+	A	G	0.4576	0.0387
rs7142165	LC_IGP63	IgG1 FG1n total/G1n	14	66250232	1	1.22E-13	1.26E-13	-	-0.256777	-0.2869	+	A	G	0.483991945	0.0343333	+	A	G	0.4576	0.0387
rs7142165	LC_IGP64	IgG1 FG2n total/G2n	14	66250232	1	2.03E-10	1.30E-14	-	-0.220969	-0.2964	+	A	G	0.483991945	0.0345243	+	A	G	0.4576	0.0385
rs7142165	LC_IGP68	IgG1 FG2n/G2n	14	66250232	1	1.08E-08	1.79E-08	-	-0.198866	-0.2159	+	A	G	0.483991945	0.0345961	+	A	G	0.4576	0.0383
rs7142165	LC_IGP61	IgG1 Fn total	14	66250232	1	3.27E-13	1.60E-13	-	-0.252125	-0.2859	+	A	G	0.483991945	0.0343298	+	A	G	0.4576	0.0388
rs7142165	LC_IGP81	IgG1 Fn/Bn	14	66250232	1	6.63E-12	1.50E-10	-	-0.238116	-0.2461	+	A	G	0.483991945	0.0344246	+	A	G	0.4576	0.0384
rs7142165	LC_IGP21	IgG1 Fucosylation	14	66250232	1	2.88E-12	6.59E-13	-	-0.24161	-0.2785	+	A	G	0.485230888	0.0343326	+	A	G	0.4576	0.0388
rs7142165	LC_IGP28	IgG1 G1S1/(G1+G1S1)	14	66250232	1	3.61E-08	1.24E-09	-	-0.189711	-0.2265	+	A	G	0.485042508	0.034273	+	A	G	0.4576	0.0373
rs7142165	LC_IGP11	IgG1_G0	14	66250232	1	4.75E-14	3.00E-14	+	0.258349	0.2934	+	A	G	0.484755345	0.0339676	+	A	G	0.4576	0.0386
rs7142165	LC_IGP_R1	IgG1_G0F/IgG1_G0	14	66250232	1	3.90E-13	1.42E-11	-	-0.248806	-0.2609	+	A	G	0.484755345	0.0339954	+	A	G	0.4576	0.0386
rs7142165	LC_IGP_R4	IgG1_G0FN/IgG1_G0N	14	66250232	1	1.85E-11	2.47E-13	-	-0.231573	-0.2845	+	A	G	0.484484385	0.0342289	+	A	G	0.4576	0.0389
rs7142165	LC_IGP52	IgG1_G0n	14	66250232	1	4.66E-14	2.35E-14	+	0.260144	0.2952	+	A	G	0.483991945	0.0341875	+	A	G	0.4576	0.0387
rs7142165	LC_IGP14	IgG1_G0N	14	66250232	1	1.80E-14	6.44E-12	+	0.26359	0.2635	+	A	G	0.484484385	0.0340778	+	A	G	0.4576	0.0384
rs7142165	LC_IGP55	IgG1_G0Nn	14	66250232	1	3.05E-14	5.84E-12	+	0.262738	0.2643	+	A	G	0.483991945	0.0342741	+	A	G	0.4576	0.0384
rs7142165	LC_IGP12	IgG1_G1	14	66250232	1	1.56E-12	6.59E-13	+	0.242937	0.2782	+	A	G	0.484755345	0.0340992	+	A	G	0.4576	0.0387
rs7142165	LC_IGP_R2	IgG1_G1F/IgG1_G1	14	66250232	1	7.98E-14	9.61E-13	-	-0.256617	-0.2756	+	A	G	0.484755345	0.0340523	+	A	G	0.4576	0.0386
rs7142165	LC_IGP_R5	IgG1_G1FN/IgG1_G1N	14	66250232	1	3.25E-11	1.77E-16	-	-0.229742	-0.3191	+	A	G	0.48349508	0.0343879	+	A	G	0.4576	0.0387
rs7142165	LC_IGP53	IgG1_G1n	14	66250232	1	9.35E-12	9.86E-13	+	0.236194	0.2758	+	A	G	0.483991945	0.0343972	+	A	G	0.4576	0.0387
rs7142165	LC_IGP15	IgG1_G1N	14	66250232	1	6.57E-10	2.20E-10	+	0.213948	0.2437	+	A	G	0.48349508	0.0344322	+	A	G	0.4576	0.0384
rs7142165	LC_IGP56	IgG1_G1Nn	14	66250232	1	5.86E-10	4.26E-10	+	0.215238	0.2394	+	A	G	0.483991945	0.0345367	+	A	G	0.4576	0.0384
rs7142165	LC_IGP_R3	IgG1_G2F/IgG1_G2	14	66250232	1	1.17E-11	3.02E-14	-	-0.233604	-0.2917	+	A	G	0.48505165	0.0341865	+	A	G	0.4576	0.0384
rs7142165	LC_IGP_R8	IgG1_G2FS1/IgG1_G2S1	14	66250232	1	3.55E-08	6.65E-12	-	-0.190176	-0.2628	+	A	G	0.485366219	0.0343384	+	A	G	0.4576	0.0383
rs7142165	LC_IGP_R26	IgG1_G2N/IgG1_G1N	14	66250232	1	2.01E-08	5.78E-10	-	-0.194011	-0.233	+	A	G	0.483433951	0.0344026	+	A	G	0.4576	0.0376
rs7142165	LC_IGP97	IgG2_G0	14	66250232	1	1.58E-08	1.19E-12	+	0.194569	0.2738	+	A	G	0.48505165	0.0342462	+	A	G	0.4576	0.0385
rs7142165	LC_IGP138	IgG2_G0n	14	66250232	1	7.40E-09	6.79E-13	+	0.199666	0.2771	+	A	G	0.48586484	0.0343518	+	A	G	0.4575	0.0386
rs60734251	LC_IGP78	IgG1 BG0n/G0n	14	66250274	1	3.54E-10	1.29E-10	+	0.248096	0.2891	+	G	T	0.267796778	0.0393015	+	G	T	0.2292	0.045
rs60734251	LC_IGP79	IgG1 BG1n/G1n	14	66250274	1	3.79E-09	5.79E-12	+	0.233781	0.3095	+	G	T	0.267796778	0.0394494	+	G	T	0.2292	0.0449
rs60734251	LC_IGP77	IgG1 Bn	14	66250274	1	6.60E-10	1.10E-11	+	0.244895	0.3066	+	G	T	0.267796778	0.0394156	+	G	T	0.2292	0.0451
rs60734251	LC_IGP86	IgG1 Bn/Fn total	14	66250274	1	1.22E-10	6.10E-12	+	0.255055	0.3106	+	G	T	0.267796778	0.0393669	+	G	T	0.2292	0.0452
rs60734251	LC_IGP84	IgG1 FBn/Bn total	14	66250274	1	1.51E-11	2.03E-15	-	-0.267738	-0.3612	+	G	T	0.267796778	0.0393966	+	G	T	0.2292	0.0455
rs60734251	LC_IGP62	IgG1 FG0n total/G0n	14	66250274	1	1.55E-13	1.49E-12	-	-0.290942	-0.3203	+	G	T	0.267796778	0.0390705	+	G	T	0.2292	0.0453
rs60734251	LC_IGP63	IgG1 FG1n total/G1n	14	66250274	1	6.64E-12	1.73E-13	-	-0.27166	-0.3331	+	G	T	0.267796778	0.0392754	+	G	T	0.2292	0.0452
rs60734251	LC_IGP64	IgG1 FG2n total/G2n	14	66250274	1	4.55E-11	1.01E-14	-	-0.260996	-0.3474	+	G	T	0.267796778	0.0393622	+	G	T	0.2292	0.0449
rs60734251	LC_IGP68	IgG1 FG2n/G2n	14	66250274	1	2.87E-08	2.24E-09	-	-0.220288	-0.2676	+	G	T	0.267796778	0.0395032	+	G	T	0.2292	0.0448
rs60734251	LC_IGP61	IgG1 Fn total	14	66250274	1	7.29E-13	1.25E-13	-	-0.283488	-0.3355	+	G	T	0.267796778	0.0391951	+	G	T	0.2292	0.0453
rs60734251	LC_IGP81	IgG1 Fn/Bn	14	66250274	1	4.35E-10	2.96E-11	-	-0.247322	-0.299	+	G	T	0.267796778	0.039384	+	G	T	0.2292	0.045
rs60734251	LC_IGP21	IgG1 Fucosylation	14	66250274	1	1.50E-11	2.49E-13	-	-0.266455	-0.331	+	G	T	0.268017263	0.0392016	+	G	T	0.2292	0.0452
rs60734251	LC_IGP11	IgG1_G0	14	66250274	1	2.05E-14	2.32E-14	+	0.299297	0.3438	+	G	T	0.26769945	0.0387808	+	G	T	0.2292	0.045
rs60734251	LC_IGP_R1	IgG1_G0F/IgG1_G0	14	66250274	1	2.81E-13	6.12E-12	-	-0.285912	-0.3104	+	G	T	0.26769945	0.0388245	+	G	T	0.2292	0.0451
rs60734251	LC_IGP_R4	IgG1_G0FN/IgG1_G0N	14	66250274	1	1.29E-10	4.38E-14	-	-0.25349	-0.3426	+	G	T	0.267742805	0.0391748	+	G	T	0.2292	0.0454
rs60734251	LC_IGP52	IgG1_G0n	14	66250274	1	1.44E-14	2.14E-14	+	0.302728	0.3449	+	G	T	0.267796778	0.0389857	+	G	T	0.2292	0.0451
rs60734251	LC_IGP14	IgG1_G0N	14	66250274	1	1.82E-11	5.05E-12	+	0.264756	0.3098	+	G	T	0.267742805	0.0391191	+	G	T	0.2292	0.0449
rs60734251	LC_IGP55	IgG1_G0Nn	14	66250274	1	1.47E-11	5.40E-12	+	0.266981	0.3096	+	G	T	0.267796778	0.0392598	+	G	T	0.2292	0.0449
rs60734251	LC_IGP12	IgG1_G1	14	66250274	1	4.69E-12	4.24E-13	+	0.271543	0.3275	+	G	T	0.268004887	0.0389787	+	G	T	0.2292	0.0452

rs60734251	LC_IGP_R2	IgG1_G1F/IgG1_G1	14	66250274	1	4.65E-12	8.19E-13	-	-0.271711	-0.3228	+	G	T	0.268004887	0.0389944	+	G	T	0.2292	0.0451
rs60734251	LC_IGP_R5	IgG1_G1FN/IgG1_G1N	14	66250274	1	1.96E-10	3.47E-16	-	-0.251545	-0.3692	+	G	T	0.267665437	0.0392694	+	G	T	0.2292	0.0453
rs60734251	LC_IGP53	IgG1_G1n	14	66250274	1	3.10E-11	9.25E-13	+	0.262727	0.3225	+	G	T	0.267796778	0.0392815	+	G	T	0.2292	0.0452
rs60734251	LC_IGP15	IgG1_G1N	14	66250274	1	9.81E-09	1.64E-11	+	0.226763	0.3029	+	G	T	0.267665437	0.0393413	+	G	T	0.2292	0.045
rs60734251	LC_IGP56	IgG1_G1Nn	14	66250274	1	1.09E-08	4.46E-11	+	0.226853	0.2959	+	G	T	0.267796778	0.0394818	+	G	T	0.2292	0.0449
rs60734251	LC_IGP_R3	IgG1_G2F/IgG1_G2	14	66250274	1	8.24E-12	9.16E-15	-	-0.268695	-0.3474	+	G	T	0.267863081	0.0390291	+	G	T	0.2292	0.0448
rs60734251	LC_IGP148	IgG2 FG0n total/G0n	14	66250274	1	2.94E-08	2.43E-08	-	-0.218595	-0.2497	+	G	T	0.268302956	0.0392288	+	G	T	0.2294	0.0448
rs60734251	LC_IGP97	IgG2_G0	14	66250274	1	1.74E-10	3.90E-09	+	0.250539	0.2642	+	G	T	0.26780165	0.0390049	+	G	T	0.2292	0.0449
rs60734251	LC_IGP_R41	IgG2_G0F/IgG2_G0	14	66250274	1	9.61E-09	8.23E-09	-	-0.225417	-0.2569	+	G	T	0.26780165	0.0390852	+	G	T	0.2292	0.0446
rs60734251	LC_IGP138	IgG2_G0n	14	66250274	1	1.02E-10	4.23E-09	+	0.254147	0.2637	+	G	T	0.268302956	0.0390591	+	G	T	0.2294	0.0449
rs59853675	LC_IGP78	IgG1 BG0n/G0n	14	66250679	1	2.25E-10	1.27E-10	+	0.25132	0.2901	+	G	A	0.267055143	0.0393658	+	G	A	0.229	0.0451
rs59853675	LC_IGP79	IgG1 BG1n/G1n	14	66250679	1	3.04E-09	4.45E-12	+	0.235653	0.3121	+	G	A	0.267055143	0.0395196	+	G	A	0.229	0.0451
rs59853675	LC_IGP77	IgG1 Bn	14	66250679	1	4.98E-10	9.45E-12	+	0.247097	0.3086	+	G	A	0.267055143	0.0394843	+	G	A	0.229	0.0453
rs59853675	LC_IGP86	IgG1 Bn/Fn total	14	66250679	1	8.99E-11	5.16E-12	+	0.257374	0.3126	+	G	A	0.267055143	0.0394348	+	G	A	0.229	0.0453
rs59853675	LC_IGP84	IgG1 FBn/Bn total	14	66250679	1	1.02E-11	1.69E-15	-	-0.270493	-0.3633	+	G	A	0.267055143	0.0394625	+	G	A	0.229	0.0456
rs59853675	LC_IGP62	IgG1 FG0n total/G0n	14	66250679	1	7.20E-14	1.32E-12	-	-0.295444	-0.322	+	G	A	0.267055143	0.0391269	+	G	A	0.229	0.0454
rs59853675	LC_IGP63	IgG1 FG1n total/G1n	14	66250679	1	3.99E-12	1.16E-13	-	-0.274997	-0.3365	+	G	A	0.267055143	0.0393384	+	G	A	0.229	0.0453
rs59853675	LC_IGP64	IgG1 FG2n total/G2n	14	66250679	1	3.40E-11	6.47E-15	-	-0.263185	-0.3509	+	G	A	0.267055143	0.0394305	+	G	A	0.229	0.045
rs59853675	LC_IGP68	IgG1 FG2n/G2n	14	66250679	1	2.45E-08	1.70E-09	-	-0.221809	-0.2704	+	G	A	0.267055143	0.039575	+	G	A	0.229	0.0449
rs59853675	LC_IGP61	IgG1 Fn total	14	66250679	1	4.15E-13	9.00E-14	-	-0.287011	-0.3384	+	G	A	0.267055143	0.0392566	+	G	A	0.229	0.0454
rs59853675	LC_IGP81	IgG1 Fn/Bn	14	66250679	1	3.25E-10	2.49E-11	-	-0.249575	-0.301	+	G	A	0.267055143	0.0394524	+	G	A	0.229	0.0451
rs59853675	LC_IGP21	IgG1 Fucosylation	14	66250679	1	7.28E-12	1.63E-13	-	-0.271007	-0.3345	+	G	A	0.267279285	0.0392592	+	G	A	0.229	0.0454
rs59853675	LC_IGP11	IgG1_G0	14	66250679	1	9.24E-15	1.90E-14	+	0.303779	0.3458	+	G	A	0.266968235	0.0388355	+	G	A	0.229	0.0452
rs59853675	LC_IGP_R1	IgG1_G0F/IgG1_G0	14	66250679	1	1.30E-13	5.27E-12	-	-0.290426	-0.3123	+	G	A	0.266968235	0.0388799	+	G	A	0.229	0.0453
rs59853675	LC_IGP_R4	IgG1_G0FN/IgG1_G0N	14	66250679	1	7.70E-11	4.23E-14	-	-0.256993	-0.3439	+	G	A	0.267009798	0.0392371	+	G	A	0.229	0.0455
rs59853675	LC_IGP52	IgG1_G0n	14	66250679	1	6.32E-15	1.74E-14	+	0.307357	0.347	+	G	A	0.267055143	0.0390406	+	G	A	0.229	0.0452
rs59853675	LC_IGP14	IgG1_G0N	14	66250679	1	1.05E-11	4.78E-12	+	0.268337	0.3109	+	G	A	0.267009798	0.0391805	+	G	A	0.229	0.045
rs59853675	LC_IGP55	IgG1_G0Nn	14	66250679	1	8.55E-12	5.09E-12	+	0.270526	0.3108	+	G	A	0.267055143	0.039322	+	G	A	0.229	0.045
rs59853675	LC_IGP12	IgG1_G1	14	66250679	1	2.77E-12	2.91E-13	+	0.274936	0.3308	+	G	A	0.267273671	0.03904	+	G	A	0.229	0.0453
rs59853675	LC_IGP_R2	IgG1_G1F/IgG1_G1	14	66250679	1	2.73E-12	5.44E-13	-	-0.275139	-0.3262	+	G	A	0.267273671	0.0390556	+	G	A	0.229	0.0452
rs59853675	LC_IGP_R5	IgG1_G1FN/IgG1_G1N	14	66250679	1	1.39E-10	2.53E-16	-	-0.25405	-0.3721	+	G	A	0.266929274	0.0393359	+	G	A	0.229	0.0454
rs59853675	LC_IGP53	IgG1_G1n	14	66250679	1	1.88E-11	6.39E-13	+	0.266088	0.3258	+	G	A	0.267055143	0.0393447	+	G	A	0.229	0.0453
rs59853675	LC_IGP15	IgG1_G1N	14	66250679	1	8.14E-09	1.33E-11	+	0.228433	0.3053	+	G	A	0.266929274	0.0394116	+	G	A	0.229	0.0451
rs59853675	LC_IGP56	IgG1_G1Nn	14	66250679	1	9.10E-09	3.62E-11	+	0.228505	0.2983	+	G	A	0.267055143	0.039553	+	G	A	0.229	0.0451
rs59853675	LC_IGP_R3	IgG1_G2F/IgG1_G2	14	66250679	1	5.94E-12	5.77E-15	-	-0.27102	-0.351	+	G	A	0.267131418	0.0390952	+	G	A	0.229	0.0449
rs59853675	LC_IGP148	IgG2 FG0n total/G0n	14	66250679	1	2.00E-08	2.71E-08	-	-0.22163	-0.2497	+	G	A	0.267565887	0.0392941	+	G	A	0.2292	0.0449
rs59853675	LC_IGP97	IgG2_G0	14	66250679	1	1.11E-10	4.34E-09	+	0.253664	0.2642	+	G	A	0.267068154	0.0390683	+	G	A	0.229	0.045
rs59853675	LC_IGP_R41	IgG2_G0F/IgG2_G0	14	66250679	1	6.42E-09	9.13E-09	-	-0.22849	-0.257	+	G	A	0.267068154	0.0391498	+	G	A	0.229	0.0447
rs59853675	LC_IGP138	IgG2_G0n	14	66250679	1	6.36E-11	4.71E-09	+	0.257396	0.2638	+	G	A	0.267565887	0.039122	+	G	A	0.2292	0.045
rs7143026	LC_IGP78	IgG1 BG0n/G0n	14	66250956	1	3.26E-11	1.33E-07	+	0.237427	0.2106	+	G	A	0.394974907	0.0355379	+	G	A	0.3638	0.0399
rs7143026	LC_IGP79	IgG1 BG1n/G1n	14	66250956	1	3.64E-10	2.38E-07	+	0.225028	0.206	+	G	A	0.394974907	0.0356727	+	G	A	0.3638	0.0399
rs7143026	LC_IGP77	IgG1 Bn	14	66250956	1	8.54E-11	8.43E-08	+	0.232941	0.2146	+	G	A	0.394974907	0.0356485	+	G	A	0.3638	0.0401
rs7143026	LC_IGP86	IgG1 Bn/Fn total	14	66250956	1	1.58E-11	6.67E-08	+	0.24172	0.2165	+	G	A	0.394974907	0.0356043	+	G	A	0.3638	0.0401
rs7143026	LC_IGP84	IgG1 FBn/Bn total	14	66250956	1	2.43E-11	3.05E-10	-	-0.239989	-0.2548	+	G	A	0.394974907	0.0356858	+	G	A	0.3638	0.0405
rs7143026	LC_IGP62	IgG1 FG0n total/G0n	14	66250956	1	8.77E-15	4.39E-09	-	-0.276547	-0.2364	+	G	A	0.394974907	0.0353182	+	G	A	0.3638	0.0403
rs7143026	LC_IGP63	IgG1 FG1n total/G1n	14	66250956	1	1.01E-12	6.06E-09	-	-0.255332	-0.2337	+	G	A	0.394974907	0.0355249	+	G	A	0.3638	0.0402
rs7143026	LC_IGP64	IgG1 FG2n total/G2n	14	66250956	1	2.72E-10	3.95E-10	-	-0.226737	-0.2498	+	G	A	0.394974907	0.035683	+	G	A	0.3638	0.0399
rs7143026	LC_IGP68	IgG1 FG2n/G2n	14	66250956	1	1.63E-08	2.09E-06	-	-0.202984	-0.1889	+	G	A	0.394974907	0.0357598	+	G	A	0.3638	0.0398
rs7143026	LC_IGP61	IgG1 Fn total	14	66250956	1	2.05E-13	1.66E-09	-	-0.262749	-0.2426	+	G	A	0.394974907	0.0354656	+	G	A	0.3638	0.0402
rs7143026	LC_IGP81	IgG1 Fn/Bn	14	66250956	1	3.11E-11	1.68E-07	-	-0.238135	-0.2088	+	G	A	0.394974907	0.0356071	+	G	A	0.3638	0.0399
rs7143026	LC_IGP21	IgG1 Fucosylation	14	66250956	1	2.75E-12	1.52E-09	-	-0.249253	-0.2433	+	G	A	0.395466091	0.0353856	+	G	A	0.3638	0.0403
rs7143026	LC_IGP11	IgG1_G0	14	66250956	1	2.35E-15	8.32E-11	+	0.279863	0.2601	+	G	A	0.394903482	0.0349866	+	G	A	0.3638	0.0401
rs7143026	LC_IGP_R1	IgG1_G0F/IgG1_G0	14	66250956	1	8.80E-15	9.75E-09	-	-0.273989	-0.2303	+	G	A	0.394903482	0.0349988	+	G	A	0.3638	0.0402
rs7143026	LC_IGP_R4	IgG1_G0FN/IgG1_G0N	14	66250956	1	1.65E-10	4.82E-10	-	-0.227652	-0.2516	+	G	A	0.394721066	0.0353915	+	G	A	0.3638	0.0404
rs7143026	LC_IGP52	IgG1_G0n	14	66250956	1	1.86E-15	7.03E-11	+	0.283143	0.2618	+	G	A	0.394974907	0.0352592	+	G	A	0.3638	0.0401
rs7143026	LC_IGP14	IgG1_G0N	14	66250956	1	1.42E-12	9.29E-09	+	0.251855	0.2285	+	G	A	0.394721066	0.0352817	+	G	A	0.3638	0.0398
rs7143026	LC_IGP55	IgG1_G0Nn	14	66250956	1	7.74E-13	9.03E-09	+	0.256372	0.229	+	G	A	0.394974907	0.0354881	+	G	A	0.3638	0.0398
rs7143026	LC_IGP12	IgG1_G1	14	66250956	1	8.35E-13	7.20E-09	+	0.253767	0.2326	+	G	A	0.395205864	0.0351827	+	G	A	0.3638	0.0402
rs7143026	LC_IGP_R2	IgG1_G1F/IgG1_G1	14	66250956	1	1.97E-13	1.30E-08	-	-0.2607	-0.228	+	G	A	0.395205864	0.0351666	+	G	A	0.3638	0.0401
rs7143026	LC_IGP_R5	IgG1_G1FN/IgG1_G1N	14	66250956	1	2.68E-10	3.86E-10	-	-0.225887	-0.252	+	G	A	0.394273985	0.0355393	+	G	A	0.3638	0.0403
rs7143026	LC_IGP53	IgG1_G1n	14	66250956	1	1.29E-11	9.75E-09	+	0.24244	0.2305	+	G	A	0.394974907	0.0355523	+	G	A	0.3638	0.0402
rs7143026	LC_IGP15	IgG1_G1N	14	66250956	1	3.26E-09	5.10E-07	+	0.211696	0.2004	+	G	A	0.394273985	0.0355741	+	G	A	0.3638	0.0399
rs7143026	LC_IGP56	IgG1_G1Nn	14	66250956	1	1.48E-09	7.82E-07	+	0.217164	0.1969	+	G	A	0.394974907	0.0357096	+	G	A	0.3638	0.0399
rs7143026	LC_IGP_R3	IgG1_G2F/IgG1_G2	14	66250956	1	1.56E-11	4.29E-10	-	-0.239552	-0.2489	+	G	A	0.395144866	0.0352762	+	G	A	0.3638	0.0399
rs7143026	LC_IGP_R8	IgG1_G2FS1/IgG1_G2S1	14	66250956	1	1.21E-08	2.41E-09	-	-0.202735	-0.2372	+	G	A	0.395514356	0.0353936	+	G	A	0.3638	0.0398
rs7143026	LC_IGP138	IgG2_G0n	14	66250956	1	2.81E-11	2.08E-06	+	0.23668	0.1897	+	G	A	0.396191195	0.03531	+	G	A	0.3639	0.04
rs67662065	LC_IGP78	IgG1 BG0n/G0n	14	66250999	1	2.76E-10	2.55E-11	+	0.249927	0.3001	+	T	G	0.268957869	0.0393462	+	T	G	0.2339	0.045
rs67662065	LC_IGP79	IgG1 BG1n/G1n	14	66250999	1	4.46E-09	1.52E-12	+	0.233018	0.3184	+	T	G	0.268957869	0.0395041	+	T	G	0.2339	0.045
rs67662065	LC_IGP77	IgG1 Bn	14	66250999	1	7.04E-10	2.44E-12	+	0.244808	0.3166	+	T	G	0.268957869	0.039468	+	T	G	0.2339	0.0452
rs67662065	LC_IGP86	IgG1 Bn/Fn total	14	66250999	1	1.32E-10	1.36E-12	+	0.254938	0.3204	+	T	G	0.268957869	0.0394194	+	T	G	0.2339	0.0452
rs67662065	LC_IGP84	IgG1 FBn/Bn total	14	66250999	1	1.18E-11	4.80E-16	-	-0.26946	-0.3701	+	T	G	0.268957869	0.0394416	+	T	G	0.2339	0.0456
rs67662065	LC_IGP62	IgG1 FG0n total/G0n	14	66250999	1	1.25E-13	4.52E-13	-	-0.292421	-0.3282	+	T	G	0.268957869	0.0391157	+	T	G	0.2339	0.0453
rs67662065	LC_IGP63	IgG1 FG1n total/G1n	14	66250999	1	7.37E-12	5.01E-14	-	-0.271425	-0.3414	+	T	G	0.268957869	0.0393285	+	T	G	0.2339	0.0453
rs67662065	LC_IGP64	IgG1 FG2n total/G2n	14	66250999	1	5.94E-11	3.53E-15	-	-0.259773	-0.3544	+	T	G	0.268957869	0.0394193	+	T	G	0.2339	0.045
rs67662065	LC_IGP68	IgG1 FG2n/G2n	14	66250999	1	3.90E-08	1.10E-09	-	-0.218462	-0.2734	+	T	G	0.268957869	0.0395614	+	T	G	0.2339	0.0449
rs67662065	LC_IGP61	IgG1 Fn total	14	66250999	1	7.19E-13	3.67E-14	-	-0.28392	-0.3435	+	T	G	0.268957869	0.0392453	+	T	G	0.2339	0.0454
rs67662065	LC_IGP81	IgG1 Fn/Bn	14	66250999	1	4.83E-10	7.91E-12	-	-0.247001	-0.3079	+	T	G	0.268957869	0.0394373	+	T	G	0.2339	0.045
rs67662065	LC_IGP21	IgG1 Fucosylation	14	66250999	1	1.76E-11	5.69E-14	-	-0.265875	-0.3405	+	T	G	0.269164612	0.039255	+	T	G	0.2339	0.0453
rs67662065	LC_IGP11	IgG1_G0	14	66250999	1	2.23E-14	8.81E-15	+	0.299248	0.3499	+	T	G	0.268844533	0.0388322	+	T	G	0.2339	0.0451
rs67662065	LC_IGP_R1	IgG1_G0F/IgG1_G0	14	66250999	1	2.49E-13	2.17E-12	-	-0.286903	-0.3175	+	T	G	0.268844533	0.0388711	+	T	G	0.2339	0.0452
rs67662065	LC_IGP_R4	IgG1_G0FN/IgG1_G0N	14	66250999	1	8.97E-11	9.72E-15	-	-0.25594	-0.3521	+	T	G	0.268890692	0.0392164	+	T	G	0.2339	0.0455
rs67662065	LC_IGP52	IgG1_G0n	14	66250999	1	1.55E-14	7.87E-15	+	0.302763	0.3513	+	T	G	0.268957869	0.0390376	+	T	G	0.2339	0.0452
rs67662065	LC_IGP14	IgG1_G0N	14	66250999	1	1.64E-11	1.03E-12	+	0.265675	0.3198	+	T	G	0.268890692	0.0391667	+	T	G	0.2339	0.0449
rs67662065	LC_IGP55	IgG1_G0Nn	14	66250999	1	1.35E-11	1.06E-12	+	0.267805	0.3199	+	T	G	0.268957869	0.0393084	+	T	G	0.2339	0.0449
rs67662065	LC_IGP12	IgG1_G1	14	66250999	1	4.35E-12	1.35E-13	+	0.272312	0.3354	+	T	G	0.269149969	0.0390266	+	T	G	0.2339	0.0453
rs67662065	LC_IGP_R2	IgG1_G1F/IgG1_G1	14	66250999	1	5.60E-12	2.61E-13	-	-0.271032	-0.3307	+	T	G	0.269149969	0.0390486	+	T	G	0.2339	0.0452
rs67662065	LC_IGP_R5	IgG1_G1FN/IgG1_G1N	14	66250999	1	1.74E-10	8.99E-17	-	-0.252565	-0.3776	+	T	G	0.268817958	0.0393168	+	T	G	0.2339	0.0454
rs67662065	LC_IGP53	IgG1_G1n	14	66250999	1	2.93E-11	2.98E-13	+	0.2634	0.3304	+	T	G	0.268957869	0.0393307	+	T	G	0.2339	0.0453
rs67662065	LC_IGP15	IgG1_G1N	14	66250999	1	1.08E-08	4.40E-12	+	0.226392	0.3118	+	T	G	0.268817958	0.0393938	+	T	G	0.2339	0.045
rs67662065	LC_IGP56	IgG1_G1Nn	14	66250999	1	1.19E-08	1.22E-11	+	0.226568	0.3049	+	T	G	0.268957869	0.0395347	+	T	G	0.2339	0.045
rs67662065	LC_IGP_R3	IgG1_G2F/IgG1_G2	14	66250999	1	9.78E-12	3.39E-15	-	-0.268083	-0.3541	+	T	G	0.269008863	0.0390829	+	T	G	0.2339	0.045
rs67662065	LC_IGP148	IgG2 FG0n total/G0n	14	66250999	1	4.14E-08	2.44E-08	-	-0.216514	-0.2506	+	T	G	0.269457204	0.0392873	+	T	G	0.2341	0.0449
rs67662065	LC_IGP97	IgG2_G0	14	66250999	1	2.38E-10	4.77E-09	+	0.249002	0.2635	+	T	G	0.268944377	0.0390621	+	T	G	0.2339	0.045
rs67662065	LC_IGP_R41	IgG2_G0F/IgG2_G0	14	66250999	1	1.24E-08	8.37E-09	-	-0.224035	-0.2576	+	T	G	0.268944377	0.039141	+	T	G	0.2339	0.0447
rs67662065	LC_IGP138	IgG2_G0n	14	66250999	1	1.39E-10	5.01E-09	+	0.252644	0.2633	+	T	G	0.269457204	0.0391166	+	T	G	0.2341	0.045
rs28470717	LC_IGP62	IgG1 FG0n total/G0n	14	66251050	1	1.94E-10	1.27E-12	-	-0.227073	-0.2926	+	C	T	0.449127323	0.0354401	+	C	T	0.3984	0.0412
rs28470717	LC_IGP63	IgG1 FG1n total/G1n	14	66251050	1	9.16E-10	8.68E-14	-	-0.219166	-0.3072	+	C	T	0.449127323	0.035578	+	C	T	0.3984	0.0412
rs28470717	LC_IGP64	IgG1 FG2n total/G2n	14	66251050	1	4.38E-08	2.11E-14	-	-0.196377	-0.3128	+	C	T	0.449127323	0.0356983	+	C	T	0.3984	0.0409
rs28470717	LC_IGP61	IgG1 Fn total	14	66251050	1	4.92E-10	1.58E-13	-	-0.222487	-0.3043	+	C	T	0.449127323	0.0355397	+	C	T	0.3984	0.0412
rs28470717	LC_IGP21	IgG1 Fucosylation	14	66251050	1	1.38E-09	4.61E-13	-	-0.216572	-0.2986	+	C	T	0.449955302	0.0355456	+	C	T	0.3984	0.0413
rs28470717	LC_IGP11	IgG1_G0	14	66251050	1	6.11E-12	8.23E-15	+	0.243571	0.3189	+	C	T	0.449503665	0.0351567	+	C	T	0.3984	0.0411
rs28470717	LC_IGP_R1	IgG1_G0F/IgG1_G0	14	66251050	1	1.15E-10	3.51E-12	-	-0.228394	-0.2859	+	C	T	0.449503665	0.0352028	+	C	T	0.3984	0.0411
rs28470717	LC_IGP52	IgG1_G0n	14	66251050	1	7.78E-12	6.90E-15	+	0.243659	0.3205	+	C	T	0.449127323	0.0353454	+	C	T	0.3984	0.0412
rs28470717	LC_IGP14	IgG1_G0N	14	66251050	1	3.79E-09	8.86E-13	+	0.209764	0.2918	+	C	T	0.449096448	0.0353993	+	C	T	0.3984	0.0408
rs28470717	LC_IGP55	IgG1_G0Nn	14	66251050	1	2.81E-09	7.72E-13	+	0.212576	0.2929	+	C	T	0.449127323	0.0355709	+	C	T	0.3984	0.0409
rs28470717	LC_IGP12	IgG1_G1	14	66251050	1	9.58E-10	4.36E-13	+	0.217334	0.2979	+	C	T	0.449503665	0.0353257	+	C	T	0.3984	0.0411
rs28470717	LC_IGP_R2	IgG1_G1F/IgG1_G1	14	66251050	1	2.33E-10	5.31E-13	-	-0.225215	-0.2964	+	C	T	0.449503665	0.0353103	+	C	T	0.3984	0.0411
rs28470717	LC_IGP53	IgG1_G1n	14	66251050	1	4.49E-09	7.84E-13	+	0.209859	0.2945	+	C	T	0.449127323	0.0355844	+	C	T	0.3984	0.0411
rs28470717	LC_IGP_R3	IgG1_G2F/IgG1_G2	14	66251050	1	2.88E-09	4.28E-14	-	-0.211351	-0.3084	+	C	T	0.449778423	0.035395	+	C	T	0.3984	0.0408
rs28470717	LC_IGP97	IgG2_G0	14	66251050	1	5.43E-09	1.12E-12	+	0.207294	0.2913	+	C	T	0.449471271	0.0353448	+	C	T	0.3984	0.041
rs28470717	LC_IGP138	IgG2_G0n	14	66251050	1	3.96E-09	5.09E-13	+	0.209524	0.2963	+	C	T	0.449882081	0.0354036	+	C	T	0.3983	0.041
rs6573627	LC_IGP78	IgG1 BG0n/G0n	14	66252326	1	1.93E-11	2.05E-10	+	0.233282	0.2428	+	T	C	0.493451673	0.0345108	+	T	C	0.4644	0.0382
rs6573627	LC_IGP79	IgG1 BG1n/G1n	14	66252326	1	2.38E-10	1.49E-11	+	0.220852	0.2578	+	T	C	0.493451673	0.0346439	+	T	C	0.4644	0.0382
rs6573627	LC_IGP77	IgG1 Bn	14	66252326	1	7.63E-11	1.53E-11	+	0.226863	0.2586	+	T	C	0.493451673	0.0346269	+	T	C	0.4644	0.0383
rs6573627	LC_IGP86	IgG1 Bn/Fn total	14	66252326	1	1.77E-11	9.38E-12	+	0.234235	0.2613	+	T	C	0.493451673	0.0345887	+	T	C	0.4644	0.0383
rs6573627	LC_IGP84	IgG1 FBn/Bn total	14	66252326	1	6.94E-11	1.12E-15	-	-0.227769	-0.3102	+	T	C	0.493451673	0.0346876	+	T	C	0.4644	0.0387
rs6573627	LC_IGP62	IgG1 FG0n total/G0n	14	66252326	1	5.00E-13	4.07E-12	-	-0.250566	-0.2672	+	T	C	0.493451673	0.0343931	+	T	C	0.4644	0.0385
rs6573627	LC_IGP63	IgG1 FG1n total/G1n	14	66252326	1	6.12E-13	9.49E-14	-	-0.250366	-0.2868	+	T	C	0.493451673	0.0344988	+	T	C	0.4644	0.0385
rs6573627	LC_IGP64	IgG1 FG2n total/G2n	14	66252326	1	1.08E-09	8.03E-15	-	-0.212758	-0.2976	+	T	C	0.493451673	0.0346919	+	T	C	0.4644	0.0383

rs6573627	LC_IGP68	IgG1 FG2n/G2n	14	66252326	1	2.80E-08	1.07E-08	-	-0.19394	-0.2184	+	T	C	0.493451673	0.0347487	+	T	C	0.4644	0.0382
rs6573627	LC_IGP61	IgG1 Fn total	14	66252326	1	1.39E-12	1.11E-13	-	-0.246339	-0.2862	+	T	C	0.493451673	0.0344918	+	T	C	0.4644	0.0385
rs6573627	LC_IGP81	IgG1 Fn/Bn	14	66252326	1	2.39E-11	9.42E-11	-	-0.232652	-0.2474	+	T	C	0.493451673	0.0345836	+	T	C	0.4644	0.0382
rs6573627	LC_IGP21	IgG1 Fucosylation	14	66252326	1	6.68E-12	2.63E-13	-	-0.238505	-0.2818	+	T	C	0.494614673	0.0344875	+	T	C	0.4644	0.0385
rs6573627	LC_IGP28	IgG1 G1S1/(G1+G1S1)	14	66252326	1	4.91E-08	1.61E-09	-	-0.18861	-0.2238	+	T	C	0.494358104	0.0344152	+	T	C	0.4644	0.0371
rs6573627	LC_IGP11	IgG1_G0	14	66252326	1	2.67E-13	3.03E-14	+	0.251629	0.2912	+	T	C	0.49405956	0.0341368	+	T	C	0.4644	0.0383
rs6573627	LC_IGP_R1	IgG1_G0F/IgG1_G0	14	66252326	1	1.44E-12	1.28E-11	-	-0.243725	-0.2601	+	T	C	0.49405956	0.0341561	+	T	C	0.4644	0.0384
rs6573627	LC_IGP_R4	IgG1_G0FN/IgG1_G0N	14	66252326	1	9.48E-11	1.36E-13	-	-0.224174	-0.286	+	T	C	0.493834354	0.0343936	+	T	C	0.4644	0.0386
rs6573627	LC_IGP52	IgG1_G0n	14	66252326	1	2.37E-13	2.30E-14	+	0.25382	0.2932	+	T	C	0.493451673	0.0343527	+	T	C	0.4644	0.0384
rs6573627	LC_IGP14	IgG1_G0N	14	66252326	1	9.35E-14	3.71E-12	+	0.257321	0.2645	+	T	C	0.493834354	0.0342422	+	T	C	0.4644	0.0381
rs6573627	LC_IGP55	IgG1_G0Nn	14	66252326	1	1.46E-13	3.12E-12	+	0.256728	0.2657	+	T	C	0.493451673	0.0344384	+	T	C	0.4644	0.0381
rs6573627	LC_IGP12	IgG1_G1	14	66252326	1	6.34E-12	5.36E-13	+	0.237192	0.2778	+	T	C	0.49405956	0.0342624	+	T	C	0.4644	0.0385
rs6573627	LC_IGP_R2	IgG1_G1F/IgG1_G1	14	66252326	1	3.50E-13	7.78E-13	-	-0.250948	-0.2752	+	T	C	0.49405956	0.0342168	+	T	C	0.4644	0.0384
rs6573627	LC_IGP_R5	IgG1_G1FN/IgG1_G1N	14	66252326	1	1.94E-10	1.45E-16	-	-0.221393	-0.3184	+	T	C	0.492891451	0.0345554	+	T	C	0.4644	0.0385
rs6573627	LC_IGP53	IgG1_G1n	14	66252326	1	3.09E-11	7.73E-13	+	0.231135	0.2757	+	T	C	0.493451673	0.0345542	+	T	C	0.4644	0.0385
rs6573627	LC_IGP15	IgG1_G1N	14	66252326	1	2.42E-09	1.35E-10	+	0.207566	0.2452	+	T	C	0.492891451	0.0345898	+	T	C	0.4644	0.0382
rs6573627	LC_IGP56	IgG1_G1Nn	14	66252326	1	1.86E-09	2.52E-10	+	0.209713	0.2413	+	T	C	0.493451673	0.0346933	+	T	C	0.4644	0.0381
rs6573627	LC_IGP_R3	IgG1_G2F/IgG1_G2	14	66252326	1	5.20E-11	1.88E-14	-	-0.227062	-0.2929	+	T	C	0.494361553	0.0343528	+	T	C	0.4644	0.0382
rs6573627	LC_IGP_R26	IgG1_G2N/IgG1_G1N	14	66252326	1	2.39E-08	7.67E-10	-	-0.193704	-0.23	+	T	C	0.492865123	0.0345369	+	T	C	0.4644	0.0374
rs6573627	LC_IGP97	IgG2_G0	14	66252326	1	2.60E-08	2.36E-12	+	0.19238	0.2688	+	T	C	0.494361247	0.0343922	+	T	C	0.4644	0.0383
rs6573627	LC_IGP138	IgG2_G0n	14	66252326	1	1.14E-08	1.20E-12	+	0.197948	0.2728	+	T	C	0.495236453	0.0344995	+	T	C	0.4643	0.0384
rs61378474	LC_IGP78	IgG1 BG0n/G0n	14	66253437	1	3.39E-11	8.61E-10	+	0.231819	0.2333	+	A	G	0.499678129	0.0347283	+	A	G	0.4666	0.038
rs61378474	LC_IGP79	IgG1 BG1n/G1n	14	66253437	1	6.56E-10	5.10E-11	+	0.216691	0.2495	+	A	G	0.499678129	0.0348716	+	A	G	0.4666	0.038
rs61378474	LC_IGP77	IgG1 Bn	14	66253437	1	1.86E-10	5.50E-11	+	0.223539	0.2502	+	A	G	0.499678129	0.034852	+	A	G	0.4666	0.0382
rs61378474	LC_IGP86	IgG1 Bn/Fn total	14	66253437	1	4.59E-11	3.71E-11	+	0.2308	0.2526	+	A	G	0.499678129	0.0348148	+	A	G	0.4666	0.0382
rs61378474	LC_IGP84	IgG1 FBn/Bn total	14	66253437	1	1.45E-10	7.73E-15	-	-0.22527	-0.2993	+	A	G	0.499678129	0.0349098	+	A	G	0.4666	0.0385
rs61378474	LC_IGP62	IgG1 FG0n total/G0n	14	66253437	1	1.14E-12	2.49E-11	-	-0.248199	-0.2558	+	A	G	0.499678129	0.0346153	+	A	G	0.4666	0.0383
rs61378474	LC_IGP63	IgG1 FG1n total/G1n	14	66253437	1	1.75E-12	5.00E-13	-	-0.246857	-0.2766	+	A	G	0.499678129	0.0347265	+	A	G	0.4666	0.0383
rs61378474	LC_IGP64	IgG1 FG2n total/G2n	14	66253437	1	2.30E-09	3.35E-14	-	-0.209809	-0.2888	+	A	G	0.499678129	0.0349144	+	A	G	0.4666	0.0381
rs61378474	LC_IGP68	IgG1 FG2n/G2n	14	66253437	1	3.62E-08	2.25E-08	-	-0.193529	-0.2121	+	A	G	0.499678129	0.0349611	+	A	G	0.4666	0.0379
rs61378474	LC_IGP61	IgG1 Fn total	14	66253437	1	3.97E-12	6.60E-13	-	-0.242737	-0.2755	+	A	G	0.499678129	0.0347194	+	A	G	0.4666	0.0383
rs61378474	LC_IGP81	IgG1 Fn/Bn	14	66253437	1	6.67E-11	3.04E-10	-	-0.228786	-0.2393	+	A	G	0.499678129	0.0348112	+	A	G	0.4666	0.038
rs61378474	LC_IGP21	IgG1 Fucosylation	14	66253437	1	1.37E-11	5.24E-13	-	-0.236398	-0.2766	+	A	G	0.500801171	0.0347104	+	A	G	0.4666	0.0383
rs61378474	LC_IGP11	IgG1_G0	14	66253437	1	6.55E-13	2.12E-13	+	0.248982	0.28	+	A	G	0.500189371	0.0343576	+	A	G	0.4666	0.0381
rs61378474	LC_IGP_R1	IgG1_G0F/IgG1_G0	14	66253437	1	3.65E-12	7.57E-11	-	-0.240737	-0.2488	+	A	G	0.500189371	0.0343777	+	A	G	0.4666	0.0382
rs61378474	LC_IGP_R4	IgG1_G0FN/IgG1_G0N	14	66253437	1	1.49E-10	1.06E-12	-	-0.223126	-0.2739	+	A	G	0.499988977	0.0346062	+	A	G	0.4666	0.0384
rs61378474	LC_IGP52	IgG1_G0n	14	66253437	1	6.01E-13	1.57E-13	+	0.251014	0.2821	+	A	G	0.499678129	0.0345769	+	A	G	0.4666	0.0382
rs61378474	LC_IGP14	IgG1_G0N	14	66253437	1	2.36E-13	1.42E-11	+	0.25463	0.2562	+	A	G	0.499988977	0.0344638	+	A	G	0.4666	0.0379
rs61378474	LC_IGP55	IgG1_G0Nn	14	66253437	1	3.56E-13	1.19E-11	+	0.254158	0.2574	+	A	G	0.499678129	0.0346624	+	A	G	0.4666	0.038
rs61378474	LC_IGP12	IgG1_G1	14	66253437	1	1.60E-11	2.89E-12	+	0.234035	0.2674	+	A	G	0.500189371	0.0344845	+	A	G	0.4666	0.0383
rs61378474	LC_IGP_R2	IgG1_G1F/IgG1_G1	14	66253437	1	9.33E-13	4.17E-12	-	-0.24787	-0.2648	+	A	G	0.500189371	0.0344399	+	A	G	0.4666	0.0382
rs61378474	LC_IGP_R5	IgG1_G1FN/IgG1_G1N	14	66253437	1	4.60E-10	8.05E-16	-	-0.21807	-0.3086	+	A	G	0.499072571	0.0347768	+	A	G	0.4666	0.0383
rs61378474	LC_IGP53	IgG1_G1n	14	66253437	1	7.78E-11	3.80E-12	+	0.227759	0.2657	+	A	G	0.499678129	0.0347795	+	A	G	0.4666	0.0383
rs61378474	LC_IGP15	IgG1_G1N	14	66253437	1	6.36E-09	4.37E-10	+	0.20325	0.2372	+	A	G	0.499072571	0.0348135	+	A	G	0.4666	0.038
rs61378474	LC_IGP56	IgG1_G1Nn	14	66253437	1	4.73E-09	7.80E-10	+	0.205627	0.2334	+	A	G	0.499678129	0.0349197	+	A	G	0.4666	0.038
rs61378474	LC_IGP_R3	IgG1_G2F/IgG1_G2	14	66253437	1	9.94E-11	8.86E-14	-	-0.225069	-0.2834	+	A	G	0.50049511	0.0345706	+	A	G	0.4666	0.038
rs61378474	LC_IGP97	IgG2_G0	14	66253437	1	1.18E-08	1.20E-11	+	0.198216	0.2583	+	A	G	0.500494804	0.0345805	+	A	G	0.4666	0.0381
rs61378474	LC_IGP138	IgG2_G0n	14	66253437	1	4.74E-09	5.77E-12	+	0.204249	0.2626	+	A	G	0.501415333	0.0346897	+	A	G	0.4665	0.0381
rs4400971	LC_IGP78	IgG1 BG0n/G0n	14	66254578	1	3.95E-11	1.25E-09	+	0.23061	0.23	+	C	A	0.500311958	0.0346688	+	C	A	0.4682	0.0379
rs4400971	LC_IGP79	IgG1 BG1n/G1n	14	66254578	1	8.65E-10	8.02E-11	+	0.214783	0.246	+	C	A	0.500311958	0.0348145	+	C	A	0.4682	0.0378
rs4400971	LC_IGP77	IgG1 Bn	14	66254578	1	2.49E-10	8.54E-11	+	0.221576	0.2466	+	C	A	0.500311958	0.0347952	+	C	A	0.4682	0.038
rs4400971	LC_IGP86	IgG1 Bn/Fn total	14	66254578	1	6.23E-11	5.93E-11	+	0.228803	0.2489	+	C	A	0.500311958	0.0347585	+	C	A	0.4682	0.038
rs4400971	LC_IGP84	IgG1 FBn/Bn total	14	66254578	1	2.18E-10	1.25E-14	-	-0.222681	-0.2957	+	C	A	0.500311958	0.0348555	+	C	A	0.4682	0.0383
rs4400971	LC_IGP62	IgG1 FG0n total/G0n	14	66254578	1	1.30E-12	3.66E-11	-	-0.247111	-0.2524	+	C	A	0.500311958	0.0345556	+	C	A	0.4682	0.0382
rs4400971	LC_IGP63	IgG1 FG1n total/G1n	14	66254578	1	2.90E-12	8.40E-13	-	-0.243996	-0.2728	+	C	A	0.500311958	0.0346744	+	C	A	0.4682	0.0381
rs4400971	LC_IGP64	IgG1 FG2n total/G2n	14	66254578	1	4.96E-09	6.09E-14	-	-0.205039	-0.2846	+	C	A	0.500311958	0.0348675	+	C	A	0.4682	0.0379
rs4400971	LC_IGP61	IgG1 Fn total	14	66254578	1	6.06E-12	1.06E-12	-	-0.240233	-0.2718	+	C	A	0.500311958	0.0346657	+	C	A	0.4682	0.0382
rs4400971	LC_IGP81	IgG1 Fn/Bn	14	66254578	1	8.85E-11	4.54E-10	-	-0.226911	-0.2359	+	C	A	0.500311958	0.0347543	+	C	A	0.4682	0.0378
rs4400971	LC_IGP21	IgG1 Fucosylation	14	66254578	1	2.18E-11	8.33E-13	-	-0.233638	-0.273	+	C	A	0.501422318	0.0346587	+	C	A	0.4682	0.0382
rs4400971	LC_IGP11	IgG1_G0	14	66254578	1	9.55E-13	5.10E-13	+	0.24679	0.2744	+	C	A	0.500804826	0.0343051	+	C	A	0.4682	0.038
rs4400971	LC_IGP_R1	IgG1_G0F/IgG1_G0	14	66254578	1	4.01E-12	1.09E-10	-	-0.239872	-0.2455	+	C	A	0.500804826	0.0343193	+	C	A	0.4682	0.038
rs4400971	LC_IGP_R4	IgG1_G0FN/IgG1_G0N	14	66254578	1	1.95E-10	1.60E-12	-	-0.221328	-0.2704	+	C	A	0.500615432	0.03455	+	C	A	0.4682	0.0383
rs4400971	LC_IGP52	IgG1_G0n	14	66254578	1	8.68E-13	3.56E-13	+	0.248841	0.2768	+	C	A	0.500311958	0.0345222	+	C	A	0.4682	0.0381
rs4400971	LC_IGP14	IgG1_G0N	14	66254578	1	3.45E-13	3.11E-11	+	0.25244	0.2509	+	C	A	0.500615432	0.0344101	+	C	A	0.4682	0.0378
rs4400971	LC_IGP55	IgG1_G0Nn	14	66254578	1	5.21E-13	2.55E-11	+	0.251935	0.2522	+	C	A	0.500311958	0.0346078	+	C	A	0.4682	0.0378
rs4400971	LC_IGP12	IgG1_G1	14	66254578	1	2.32E-11	4.65E-12	+	0.231761	0.2637	+	C	A	0.500804826	0.0344317	+	C	A	0.4682	0.0381
rs4400971	LC_IGP_R2	IgG1_G1F/IgG1_G1	14	66254578	1	1.59E-12	6.95E-12	-	-0.244928	-0.2609	+	C	A	0.500804826	0.0343904	+	C	A	0.4682	0.038
rs4400971	LC_IGP_R5	IgG1_G1FN/IgG1_G1N	14	66254578	1	6.83E-10	1.42E-15	-	-0.215536	-0.3047	+	C	A	0.499701722	0.0347226	+	C	A	0.4682	0.0382
rs4400971	LC_IGP53	IgG1_G1n	14	66254578	1	1.08E-10	5.65E-12	+	0.225621	0.2624	+	C	A	0.500311958	0.0347237	+	C	A	0.4682	0.0381
rs4400971	LC_IGP15	IgG1_G1N	14	66254578	1	7.59E-09	6.38E-10	+	0.201854	0.2339	+	C	A	0.499701722	0.0347547	+	C	A	0.4682	0.0378
rs4400971	LC_IGP56	IgG1_G1Nn	14	66254578	1	5.63E-09	1.07E-09	+	0.204249	0.2305	+	C	A	0.500311958	0.0348603	+	C	A	0.4682	0.0378
rs4400971	LC_IGP_R3	IgG1_G2F/IgG1_G2	14	66254578	1	2.31E-10	1.65E-13	-	-0.220263	-0.2791	+	C	A	0.501110941	0.0345273	+	C	A	0.4682	0.0378
rs4400971	LC_IGP97	IgG2_G0	14	66254578	1	1.60E-08	1.75E-11	+	0.196079	0.2549	+	C	A	0.501110636	0.0345261	+	C	A	0.4682	0.0379
rs4400971	LC_IGP138	IgG2_G0n	14	66254578	1	6.39E-09	8.59E-12	+	0.202177	0.2592	+	C	A	0.502035714	0.0346346	+	C	A	0.4681	0.038
rs56379231	LC_IGP78	IgG1 BG0n/G0n	14	66255397	1	1.02E-10	6.20E-07	+	0.231363	0.1956	+	C	A	0.402513011	0.0355534	+	C	A	0.3649	0.0392
rs56379231	LC_IGP79	IgG1 BG1n/G1n	14	66255397	1	1.73E-09	6.31E-07	+	0.216202	0.1953	+	C	A	0.402513011	0.0356972	+	C	A	0.3649	0.0392
rs56379231	LC_IGP77	IgG1 Bn	14	66255397	1	3.85E-10	2.98E-07	+	0.224706	0.2018	+	C	A	0.402513011	0.035672	+	C	A	0.3649	0.0394
rs56379231	LC_IGP86	IgG1 Bn/Fn total	14	66255397	1	8.17E-11	2.37E-07	+	0.233069	0.2037	+	C	A	0.402513011	0.0356307	+	C	A	0.3649	0.0394
rs56379231	LC_IGP84	IgG1 FBn/Bn total	14	66255397	1	1.43E-10	1.63E-09	-	-0.230521	-0.2396	+	C	A	0.402513011	0.0357152	+	C	A	0.3649	0.0397
rs56379231	LC_IGP62	IgG1 FG0n total/G0n	14	66255397	1	6.79E-14	3.20E-08	-	-0.267224	-0.2186	+	C	A	0.402513011	0.0353533	+	C	A	0.3649	0.0395
rs56379231	LC_IGP63	IgG1 FG1n total/G1n	14	66255397	1	9.34E-12	2.32E-08	-	-0.24421	-0.2206	+	C	A	0.402513011	0.0355641	+	C	A	0.3649	0.0395
rs56379231	LC_IGP64	IgG1 FG2n total/G2n	14	66255397	1	2.13E-09	1.60E-09	-	-0.215083	-0.237	+	C	A	0.402513011	0.0357185	+	C	A	0.3649	0.0393
rs56379231	LC_IGP61	IgG1 Fn total	14	66255397	1	1.85E-12	8.52E-09	-	-0.252115	-0.2277	+	C	A	0.402513011	0.0355041	+	C	A	0.3649	0.0395
rs56379231	LC_IGP81	IgG1 Fn/Bn	14	66255397	1	1.52E-10	5.62E-07	-	-0.229661	-0.1962	+	C	A	0.402513011	0.0356323	+	C	A	0.3649	0.0392
rs56379231	LC_IGP21	IgG1 Fucosylation	14	66255397	1	1.52E-11	2.80E-09	-	-0.24067	-0.2351	+	C	A	0.402866523	0.035419	+	C	A	0.3649	0.0396
rs56379231	LC_IGP11	IgG1_G0	14	66255397	1	3.33E-14	1.03E-09	+	0.268152	0.24	+	C	A	0.402235797	0.0350382	+	C	A	0.3649	0.0393
rs56379231	LC_IGP_R1	IgG1_G0F/IgG1_G0	14	66255397	1	8.09E-14	6.86E-08	-	-0.264009	-0.2126	+	C	A	0.402235797	0.0350412	+	C	A	0.3649	0.0394
rs56379231	LC_IGP_R4	IgG1_G0FN/IgG1_G0N	14	66255397	1	7.06E-10	4.37E-09	-	-0.219611	-0.2328	+	C	A	0.402117269	0.0354112	+	C	A	0.3649	0.0397
rs56379231	LC_IGP52	IgG1_G0n	14	66255397	1	2.55E-14	8.22E-10	+	0.271496	0.2419	+	C	A	0.402513011	0.0353067	+	C	A	0.3649	0.0394
rs56379231	LC_IGP14	IgG1_G0N	14	66255397	1	9.57E-12	5.48E-08	+	0.242326	0.2126	+	C	A	0.402117269	0.0353112	+	C	A	0.3649	0.0391
rs56379231	LC_IGP55	IgG1_G0Nn	14	66255397	1	4.69E-12	5.13E-08	+	0.247464	0.2133	+	C	A	0.402513011	0.035518	+	C	A	0.3649	0.0392
rs56379231	LC_IGP12	IgG1_G1	14	66255397	1	7.68E-12	3.18E-08	+	0.242894	0.2185	+	C	A	0.402541234	0.0352288	+	C	A	0.3649	0.0395
rs56379231	LC_IGP_R2	IgG1_G1F/IgG1_G1	14	66255397	1	1.88E-12	5.14E-08	-	-0.249946	-0.2146	+	C	A	0.402541234	0.0352134	+	C	A	0.3649	0.0394
rs56379231	LC_IGP_R5	IgG1_G1FN/IgG1_G1N	14	66255397	1	1.88E-09	1.37E-09	-	-0.214919	-0.2396	+	C	A	0.40170203	0.0355688	+	C	A	0.3649	0.0395
rs56379231	LC_IGP53	IgG1_G1n	14	66255397	1	9.41E-11	3.76E-08	+	0.232004	0.2172	+	C	A	0.402513011	0.0355862	+	C	A	0.3649	0.0395
rs56379231	LC_IGP15	IgG1_G1N	14	66255397	1	1.40E-08	1.39E-06	+	0.202977	0.1893	+	C	A	0.40170203	0.035593	+	C	A	0.3649	0.0392
rs56379231	LC_IGP56	IgG1_G1Nn	14	66255397	1	5.38E-09	1.96E-06	+	0.209611	0.1864	+	C	A	0.402513011	0.0357283	+	C	A	0.3649	0.0392
rs56379231	LC_IGP_R3	IgG1_G2F/IgG1_G2	14	66255397	1	1.34E-10	1.76E-09	-	-0.228302	-0.2359	+	C	A	0.402481663	0.0353178	+	C	A	0.3649	0.0392
rs56379231	LC_IGP_R8	IgG1_G2FS1/IgG1_G2S1	14	66255397	1	3.45E-08	1.04E-08	-	-0.196315	-0.2236	+	C	A	0.402845449	0.0354128	+	C	A	0.3649	0.0391
rs7151846	LC_IGP78	IgG1 BG0n/G0n	14	66255781	1	4.13E-11	1.28E-09	+	0.230486	0.2295	+	G	C	0.500360905	0.034685	+	G	C	0.4682	0.0378
rs7151846	LC_IGP79	IgG1 BG1n/G1n	14	66255781	1	8.68E-10	8.41E-11	+	0.214862	0.2454	+	G	C	0.500360905	0.0348298	+	G	C	0.4682	0.0378
rs7151846	LC_IGP77	IgG1 Bn	14	66255781	1	2.53E-10	8.99E-11	+	0.221585	0.246	+	G	C	0.500360905	0.0348108	+	G	C	0.4682	0.038
rs7151846	LC_IGP86	IgG1 Bn/Fn total	14	66255781	1	6.36E-11	6.27E-11	+	0.228801	0.2482	+	G	C	0.500360905	0.0347741	+	G	C	0.4682	0.038
rs7151846	LC_IGP84	IgG1 FBn/Bn total	14	66255781	1	2.22E-10	1.29E-14	-	-0.222684	-0.295	+	G	C	0.500360905	0.0348711	+	G	C	0.4682	0.0383
rs7151846	LC_IGP62	IgG1 FG0n total/G0n	14	66255781	1	1.41E-12	3.88E-11	-	-0.246821	-0.2517	+	G	C	0.500360905	0.0345725	+	G	C	0.4682	0.0381
rs7151846	LC_IGP63	IgG1 FG1n total/G1n	14	66255781	1	2.99E-12	9.28E-13	-	-0.243954	-0.2719	+	G	C	0.500360905	0.0346903	+	G	C	0.4682	0.0381
rs7151846	LC_IGP64	IgG1 FG2n total/G2n	14	66255781	1	5.14E-09	6.76E-14	-	-0.204931	-0.2837	+	G	C	0.500360905	0.0348835	+	G	C	0.4682	0.0379
rs7151846	LC_IGP61	IgG1 Fn total	14	66255781	1	6.40E-12	1.15E-12	-	-0.240072	-0.271	+	G	C	0.500360905	0.034682	+	G	C	0.4682	0.0381
rs7151846	LC_IGP81	IgG1 Fn/Bn	14	66255781	1	9.04E-11	4.77E-10	-	-0.226898	-0.2353	+	G	C	0.500360905	0.03477	+	G	C	0.4682	0.0378
rs7151846	LC_IGP21	IgG1 Fucosylation	14	66255781	1	2.26E-11	8.67E-13	-	-0.233553	-0.2723	+	G	C	0.501461776	0.0346754	+	G	C	0.4682	0.0381
rs7151846	LC_IGP11	IgG1_G0	14	66255781	1	1.03E-12	5.61E-13	+	0.24653	0.2734	+	G	C	0.500843922	0.0343223	+	G	C	0.4682	0.0379
rs7151846	LC_IGP_R1	IgG1_G0F/IgG1_G0	14	66255781	1	4.40E-12	1.18E-10	-	-0.239531	-0.2447	+	G	C	0.500843922	0.0343368	+	G	C	0.4682	0.038
rs7151846	LC_IGP_R4	IgG1_G0FN/IgG1_G0N	14	66255781	1	2.03E-10	1.62E-12	-	-0.221208	-0.2699	+	G	C	0.500663809	0.0345658	+	G	C	0.4682	0.0382
rs7151846	LC_IGP52	IgG1_G0n	14	66255781	1	9.40E-13	3.91E-13	+	0.248577	0.2758	+	G	C	0.500360905	0.034539	+	G	C	0.4682	0.038
rs7151846	LC_IGP14	IgG1_G0N	14	66255781	1	3.57E-13	3.29E-11	+	0.252389	0.2502	+	G	C	0.500663809	0.0344258	+	G	C	0.4682	0.0377
rs7151846	LC_IGP55	IgG1_G0Nn	14	66255781	1	5.38E-13	2.68E-11	+	0.251892	0.2516	+	G	C	0.500360905	0.0346236	+	G	C	0.4682	0.0378
rs7151846	LC_IGP12	IgG1_G1	14	66255781	1	2.46E-11	5.15E-12	+	0.231586	0.2627	+	G	C	0.500843922	0.0344485	+	G	C	0.4682	0.0381
rs7151846	LC_IGP_R2	IgG1_G1F/IgG1_G1	14	66255781	1	1.65E-12	7.75E-12	-	-0.244858	-0.26	+	G	C	0.500843922	0.0344069	+	G	C	0.4682	0.038
rs7151846	LC_IGP_R5	IgG1_G1FN/IgG1_G1N	14	66255781	1	6.85E-10	1.48E-15	-	-0.21561	-0.3041	+	G	C	0.499750308	0.0347378	+	G	C	0.4682	0.0381

rs7151846	LC_IGP53	IgG1_G1n	14	66255781	1	1.14E-10	6.21E-12	+	0.225444	0.2616	+	G	C	0.500360905	0.03474	+	G	C	0.4682	0.038
rs7151846	LC_IGP15	IgG1_G1N	14	66255781	1	7.69E-09	6.65E-10	+	0.201869	0.2334	+	G	C	0.499750308	0.0347701	+	G	C	0.4682	0.0378
rs7151846	LC_IGP56	IgG1_G1Nn	14	66255781	1	5.70E-09	1.11E-09	+	0.204274	0.23	+	G	C	0.500360905	0.0348758	+	G	C	0.4682	0.0378
rs7151846	LC_IGP_R3	IgG1_G2F/IgG1_G2	14	66255781	1	2.41E-10	1.84E-13	-	-0.220136	-0.2781	+	G	C	0.501150061	0.0345439	+	G	C	0.4682	0.0378
rs7151846	LC_IGP97	IgG2_G0	14	66255781	1	1.63E-08	1.92E-11	+	0.196064	0.2539	+	G	C	0.501149756	0.0345421	+	G	C	0.4682	0.0378
rs7151846	LC_IGP138	IgG2_G0n	14	66255781	1	6.49E-09	9.42E-12	+	0.202182	0.2582	+	G	C	0.502075123	0.0346508	+	G	C	0.4681	0.0379
rs56720460	LC_IGP78	IgG1 BG0n/G0n	14	66256336	1	5.51E-11	1.04E-09	+	0.229254	0.2307	+	T	C	0.500471809	0.034728	+	T	C	0.4682	0.0378
rs56720460	LC_IGP79	IgG1 BG1n/G1n	14	66256336	1	1.33E-09	7.45E-11	+	0.212718	0.2459	+	T	C	0.500471809	0.034876	+	T	C	0.4682	0.0378
rs56720460	LC_IGP77	IgG1 Bn	14	66256336	1	3.72E-10	7.50E-11	+	0.219752	0.2469	+	T	C	0.500471809	0.0348561	+	T	C	0.4682	0.0379
rs56720460	LC_IGP86	IgG1 Bn/Fn total	14	66256336	1	9.45E-11	5.35E-11	+	0.226981	0.249	+	T	C	0.500471809	0.0348196	+	T	C	0.4682	0.038
rs56720460	LC_IGP84	IgG1 FBn/Bn total	14	66256336	1	3.28E-10	9.03E-15	-	-0.220835	-0.2966	+	T	C	0.500471809	0.0349166	+	T	C	0.4682	0.0383
rs56720460	LC_IGP62	IgG1 FG0n total/G0n	14	66256336	1	2.22E-12	3.17E-11	-	-0.244936	-0.2527	+	T	C	0.500471809	0.0346189	+	T	C	0.4682	0.0381
rs56720460	LC_IGP63	IgG1 FG1n total/G1n	14	66256336	1	4.21E-12	8.11E-13	-	-0.24255	-0.2724	+	T	C	0.500471809	0.0347345	+	T	C	0.4682	0.038
rs56720460	LC_IGP64	IgG1 FG2n total/G2n	14	66256336	1	6.99E-09	5.67E-14	-	-0.20336	-0.2843	+	T	C	0.500471809	0.0349273	+	T	C	0.4682	0.0378
rs56720460	LC_IGP61	IgG1 Fn total	14	66256336	1	9.31E-12	9.69E-13	-	-0.238482	-0.2717	+	T	C	0.500471809	0.0347269	+	T	C	0.4682	0.0381
rs56720460	LC_IGP81	IgG1 Fn/Bn	14	66256336	1	1.32E-10	4.10E-10	-	-0.225138	-0.2361	+	T	C	0.500471809	0.0348151	+	T	C	0.4682	0.0378
rs56720460	LC_IGP21	IgG1 Fucosylation	14	66256336	1	3.46E-11	7.96E-13	-	-0.231648	-0.2726	+	T	C	0.501566583	0.0347217	+	T	C	0.4682	0.0381
rs56720460	LC_IGP11	IgG1_G0	14	66256336	1	1.76E-12	5.02E-13	+	0.244372	0.274	+	T	C	0.501088271	0.0343848	+	T	C	0.4682	0.0379
rs56720460	LC_IGP_R1	IgG1_G0F/IgG1_G0	14	66256336	1	5.95E-12	1.01E-10	-	-0.23842	-0.2455	+	T	C	0.501088271	0.0343944	+	T	C	0.4682	0.038
rs56720460	LC_IGP_R4	IgG1_G0FN/IgG1_G0N	14	66256336	1	2.72E-10	1.15E-12	-	-0.219978	-0.2716	+	T	C	0.500914268	0.0346234	+	T	C	0.4682	0.0382
rs56720460	LC_IGP52	IgG1_G0n	14	66256336	1	1.87E-12	3.47E-13	+	0.245584	0.2764	+	T	C	0.500471809	0.0345903	+	T	C	0.4682	0.038
rs56720460	LC_IGP14	IgG1_G0N	14	66256336	1	5.09E-13	2.82E-11	+	0.25112	0.251	+	T	C	0.500914268	0.0344844	+	T	C	0.4682	0.0377
rs56720460	LC_IGP55	IgG1_G0Nn	14	66256336	1	8.85E-13	2.28E-11	+	0.24982	0.2525	+	T	C	0.500471809	0.0346711	+	T	C	0.4682	0.0378
rs56720460	LC_IGP12	IgG1_G1	14	66256336	1	2.95E-11	4.42E-12	+	0.231005	0.2633	+	T	C	0.501088271	0.0345039	+	T	C	0.4682	0.038
rs56720460	LC_IGP_R2	IgG1_G1F/IgG1_G1	14	66256336	1	2.57E-12	6.99E-12	-	-0.243101	-0.2603	+	T	C	0.501088271	0.0344676	+	T	C	0.4682	0.038
rs56720460	LC_IGP_R5	IgG1_G1FN/IgG1_G1N	14	66256336	1	9.06E-10	1.11E-15	-	-0.2144	-0.3053	+	T	C	0.500001845	0.0347955	+	T	C	0.4682	0.0381
rs56720460	LC_IGP53	IgG1_G1n	14	66256336	1	1.31E-10	5.20E-12	+	0.224952	0.2623	+	T	C	0.500471809	0.03478	+	T	C	0.4682	0.038
rs56720460	LC_IGP15	IgG1_G1N	14	66256336	1	9.83E-09	5.80E-10	+	0.200731	0.234	+	T	C	0.500001845	0.0348271	+	T	C	0.4682	0.0378
rs56720460	LC_IGP56	IgG1_G1Nn	14	66256336	1	7.55E-09	9.52E-10	+	0.202846	0.2307	+	T	C	0.500471809	0.034919	+	T	C	0.4682	0.0377
rs56720460	LC_IGP_R3	IgG1_G2F/IgG1_G2	14	66256336	1	3.11E-10	1.61E-13	-	-0.219106	-0.2786	+	T	C	0.50139456	0.034601	+	T	C	0.4682	0.0378
rs56720460	LC_IGP97	IgG2_G0	14	66256336	1	2.53E-08	1.81E-11	+	0.193642	0.2542	+	T	C	0.501253667	0.034588	+	T	C	0.4682	0.0378
rs56720460	LC_IGP138	IgG2_G0n	14	66256336	1	1.02E-08	8.89E-12	+	0.199795	0.2585	+	T	C	0.502179802	0.0346975	+	T	C	0.4682	0.0379
rs56922569	LC_IGP78	IgG1 BG0n/G0n	14	66256472	1	5.44E-11	1.02E-09	+	0.228688	0.2309	+	T	A	0.498317535	0.0346317	+	T	A	0.4674	0.0378
rs56922569	LC_IGP79	IgG1 BG1n/G1n	14	66256472	1	7.13E-10	8.02E-11	+	0.215576	0.2456	+	T	A	0.498317535	0.0347664	+	T	A	0.4674	0.0378
rs56922569	LC_IGP77	IgG1 Bn	14	66256472	1	2.47E-10	7.63E-11	+	0.22135	0.247	+	T	A	0.498317535	0.034751	+	T	A	0.4674	0.038
rs56922569	LC_IGP86	IgG1 Bn/Fn total	14	66256472	1	6.06E-11	5.58E-11	+	0.228658	0.249	+	T	A	0.498317535	0.0347139	+	T	A	0.4674	0.038
rs56922569	LC_IGP84	IgG1 FBn/Bn total	14	66256472	1	2.15E-10	9.04E-15	-	-0.222487	-0.2968	+	T	A	0.498317535	0.0348111	+	T	A	0.4674	0.0383
rs56922569	LC_IGP62	IgG1 FG0n total/G0n	14	66256472	1	1.20E-12	3.24E-11	-	-0.247163	-0.2527	+	T	A	0.498317535	0.0345103	+	T	A	0.4674	0.0381
rs56922569	LC_IGP63	IgG1 FG1n total/G1n	14	66256472	1	2.12E-12	9.05E-13	-	-0.245193	-0.272	+	T	A	0.498317535	0.034624	+	T	A	0.4674	0.0381
rs56922569	LC_IGP64	IgG1 FG2n total/G2n	14	66256472	1	3.70E-09	6.19E-14	-	-0.206463	-0.2841	+	T	A	0.498317535	0.0348173	+	T	A	0.4674	0.0379
rs56922569	LC_IGP61	IgG1 Fn total	14	66256472	1	5.28E-12	1.04E-12	-	-0.240606	-0.2715	+	T	A	0.498317535	0.0346189	+	T	A	0.4674	0.0381
rs56922569	LC_IGP81	IgG1 Fn/Bn	14	66256472	1	8.69E-11	4.07E-10	-	-0.226717	-0.2363	+	T	A	0.498317535	0.03471	+	T	A	0.4674	0.0378
rs56922569	LC_IGP21	IgG1 Fucosylation	14	66256472	1	1.70E-11	8.13E-13	-	-0.234596	-0.2726	+	T	A	0.499421394	0.0346104	+	T	A	0.4674	0.0381
rs56922569	LC_IGP11	IgG1_G0	14	66256472	1	8.05E-13	5.22E-13	+	0.247287	0.2739	+	T	A	0.498822237	0.0342598	+	T	A	0.4674	0.0379
rs56922569	LC_IGP_R1	IgG1_G0F/IgG1_G0	14	66256472	1	3.44E-12	1.04E-10	-	-0.240309	-0.2454	+	T	A	0.498822237	0.0342744	+	T	A	0.4674	0.038
rs56922569	LC_IGP_R4	IgG1_G0FN/IgG1_G0N	14	66256472	1	2.40E-10	1.06E-12	-	-0.219963	-0.2722	+	T	A	0.498644214	0.0345109	+	T	A	0.4674	0.0382
rs56922569	LC_IGP52	IgG1_G0n	14	66256472	1	7.30E-13	3.58E-13	+	0.249341	0.2764	+	T	A	0.498317535	0.0344749	+	T	A	0.4674	0.038
rs56922569	LC_IGP14	IgG1_G0N	14	66256472	1	4.43E-13	2.75E-11	+	0.250965	0.2513	+	T	A	0.498644214	0.0343721	+	T	A	0.4674	0.0377
rs56922569	LC_IGP55	IgG1_G0Nn	14	66256472	1	7.05E-13	2.19E-11	+	0.2502	0.2529	+	T	A	0.498317535	0.0345704	+	T	A	0.4674	0.0378
rs56922569	LC_IGP12	IgG1_G1	14	66256472	1	1.76E-11	5.00E-12	+	0.232853	0.2628	+	T	A	0.498822237	0.0343841	+	T	A	0.4674	0.0381
rs56922569	LC_IGP_R2	IgG1_G1F/IgG1_G1	14	66256472	1	1.17E-12	7.70E-12	-	-0.246064	-0.2599	+	T	A	0.498822237	0.0343423	+	T	A	0.4674	0.038
rs56922569	LC_IGP_R5	IgG1_G1FN/IgG1_G1N	14	66256472	1	5.67E-10	1.19E-15	-	-0.216277	-0.3051	+	T	A	0.497722019	0.0346755	+	T	A	0.4674	0.0381
rs56922569	LC_IGP53	IgG1_G1n	14	66256472	1	8.42E-11	5.81E-12	+	0.226653	0.2618	+	T	A	0.498317535	0.0346745	+	T	A	0.4674	0.038
rs56922569	LC_IGP15	IgG1_G1N	14	66256472	1	6.35E-09	6.27E-10	+	0.202636	0.2337	+	T	A	0.497722019	0.0347077	+	T	A	0.4674	0.0378
rs56922569	LC_IGP56	IgG1_G1Nn	14	66256472	1	4.82E-09	1.02E-09	+	0.204884	0.2305	+	T	A	0.498317535	0.034813	+	T	A	0.4674	0.0378
rs56922569	LC_IGP_R3	IgG1_G2F/IgG1_G2	14	66256472	1	1.74E-10	1.76E-13	-	-0.221494	-0.2784	+	T	A	0.49912714	0.0344792	+	T	A	0.4674	0.0378
rs56922569	LC_IGP148	IgG2 FG0n total/G0n	14	66256472	1	2.76E-08	2.91E-11	-	-0.193487	-0.2512	+	T	A	0.500037254	0.0346541	+	T	A	0.4674	0.0378
rs56922569	LC_IGP97	IgG2_G0	14	66256472	1	7.88E-09	1.75E-11	+	0.199973	0.2547	+	T	A	0.499126834	0.0344693	+	T	A	0.4674	0.0379
rs56922569	LC_IGP138	IgG2_G0n	14	66256472	1	3.12E-09	8.62E-12	+	0.206026	0.2589	+	T	A	0.500037254	0.0345765	+	T	A	0.4674	0.0379
rs59636756	LC_IGP78	IgG1 BG0n/G0n	14	66256489	1	5.41E-11	1.02E-09	+	0.228712	0.2309	+	C	T	0.498309789	0.0346314	+	C	T	0.4674	0.0378
rs59636756	LC_IGP79	IgG1 BG1n/G1n	14	66256489	1	7.10E-10	8.05E-11	+	0.215599	0.2456	+	C	T	0.498309789	0.0347661	+	C	T	0.4674	0.0378
rs59636756	LC_IGP77	IgG1 Bn	14	66256489	1	2.45E-10	7.66E-11	+	0.22138	0.247	+	C	T	0.498309789	0.0347507	+	C	T	0.4674	0.038
rs59636756	LC_IGP86	IgG1 Bn/Fn total	14	66256489	1	6.03E-11	5.61E-11	+	0.228687	0.2489	+	C	T	0.498309789	0.0347136	+	C	T	0.4674	0.038
rs59636756	LC_IGP84	IgG1 FBn/Bn total	14	66256489	1	2.14E-10	9.01E-15	-	-0.222504	-0.2968	+	C	T	0.498309789	0.0348108	+	C	T	0.4674	0.0383
rs59636756	LC_IGP62	IgG1 FG0n total/G0n	14	66256489	1	1.20E-12	3.23E-11	-	-0.247166	-0.2528	+	C	T	0.498309789	0.0345101	+	C	T	0.4674	0.0381
rs59636756	LC_IGP63	IgG1 FG1n total/G1n	14	66256489	1	2.12E-12	9.06E-13	-	-0.245198	-0.2719	+	C	T	0.498309789	0.0346238	+	C	T	0.4674	0.0381
rs59636756	LC_IGP64	IgG1 FG2n total/G2n	14	66256489	1	3.71E-09	6.20E-14	-	-0.206454	-0.2841	+	C	T	0.498309789	0.0348172	+	C	T	0.4674	0.0379
rs59636756	LC_IGP61	IgG1 Fn total	14	66256489	1	5.28E-12	1.04E-12	-	-0.240603	-0.2715	+	C	T	0.498309789	0.0346188	+	C	T	0.4674	0.0381
rs59636756	LC_IGP81	IgG1 Fn/Bn	14	66256489	1	8.64E-11	4.09E-10	-	-0.226746	-0.2362	+	C	T	0.498309789	0.0347097	+	C	T	0.4674	0.0378
rs59636756	LC_IGP21	IgG1 Fucosylation	14	66256489	1	1.71E-11	8.11E-13	-	-0.234573	-0.2726	+	C	T	0.499413687	0.0346103	+	C	T	0.4674	0.0381
rs59636756	LC_IGP11	IgG1_G0	14	66256489	1	8.02E-13	5.24E-13	+	0.247301	0.2739	+	C	T	0.4988146	0.0342596	+	C	T	0.4674	0.0379
rs59636756	LC_IGP_R1	IgG1_G0F/IgG1_G0	14	66256489	1	3.44E-12	1.04E-10	-	-0.240308	-0.2454	+	C	T	0.4988146	0.0342742	+	C	T	0.4674	0.038
rs59636756	LC_IGP_R4	IgG1_G0FN/IgG1_G0N	14	66256489	1	2.39E-10	1.06E-12	-	-0.219978	-0.2722	+	C	T	0.498636558	0.0345107	+	C	T	0.4674	0.0382
rs59636756	LC_IGP52	IgG1_G0n	14	66256489	1	7.28E-13	3.59E-13	+	0.249351	0.2764	+	C	T	0.498309789	0.0344747	+	C	T	0.4674	0.038
rs59636756	LC_IGP14	IgG1_G0N	14	66256489	1	4.40E-13	2.77E-11	+	0.250999	0.2513	+	C	T	0.498636558	0.0343718	+	C	T	0.4674	0.0377
rs59636756	LC_IGP55	IgG1_G0Nn	14	66256489	1	7.00E-13	2.21E-11	+	0.250232	0.2528	+	C	T	0.498309789	0.0345701	+	C	T	0.4674	0.0378
rs59636756	LC_IGP12	IgG1_G1	14	66256489	1	1.76E-11	5.00E-12	+	0.232855	0.2628	+	C	T	0.4988146	0.034384	+	C	T	0.4674	0.0381
rs59636756	LC_IGP_R2	IgG1_G1F/IgG1_G1	14	66256489	1	1.17E-12	7.71E-12	-	-0.246065	-0.2599	+	C	T	0.4988146	0.0343421	+	C	T	0.4674	0.038
rs59636756	LC_IGP_R5	IgG1_G1FN/IgG1_G1N	14	66256489	1	5.65E-10	1.19E-15	-	-0.216292	-0.3051	+	C	T	0.49771433	0.0346753	+	C	T	0.4674	0.0381
rs59636756	LC_IGP53	IgG1_G1n	14	66256489	1	8.42E-11	5.81E-12	+	0.226651	0.2618	+	C	T	0.498309789	0.0346743	+	C	T	0.4674	0.038
rs59636756	LC_IGP15	IgG1_G1N	14	66256489	1	6.32E-09	6.29E-10	+	0.202665	0.2337	+	C	T	0.49771433	0.0347075	+	C	T	0.4674	0.0378
rs59636756	LC_IGP56	IgG1_G1Nn	14	66256489	1	4.80E-09	1.02E-09	+	0.20491	0.2305	+	C	T	0.498309789	0.0348127	+	C	T	0.4674	0.0378
rs59636756	LC_IGP_R3	IgG1_G2F/IgG1_G2	14	66256489	1	1.74E-10	1.76E-13	-	-0.221478	-0.2784	+	C	T	0.499119499	0.0344791	+	C	T	0.4674	0.0378
rs59636756	LC_IGP148	IgG2 FG0n total/G0n	14	66256489	1	2.76E-08	2.92E-11	-	-0.193485	-0.2512	+	C	T	0.500029557	0.0346539	+	C	T	0.4673	0.0378
rs59636756	LC_IGP97	IgG2_G0	14	66256489	1	7.85E-09	1.75E-11	+	0.19999	0.2547	+	C	T	0.499119193	0.0344691	+	C	T	0.4674	0.0379
rs59636756	LC_IGP138	IgG2_G0n	14	66256489	1	3.11E-09	8.63E-12	+	0.206037	0.2589	+	C	T	0.500029557	0.0345763	+	C	T	0.4673	0.0379
rs55845504	LC_IGP78	IgG1 BG0n/G0n	14	66256968	1	1.20E-10	4.97E-07	+	0.230339	0.1971	+	T	C	0.400923172	0.0355371	+	T	C	0.3639	0.0392
rs55845504	LC_IGP79	IgG1 BG1n/G1n	14	66256968	1	1.60E-09	5.76E-07	+	0.216535	0.1958	+	T	C	0.400923172	0.0356755	+	T	C	0.3639	0.0392
rs55845504	LC_IGP77	IgG1 Bn	14	66256968	1	3.80E-10	2.57E-07	+	0.224648	0.2027	+	T	C	0.400923172	0.0356517	+	T	C	0.3639	0.0393
rs55845504	LC_IGP86	IgG1 Bn/Fn total	14	66256968	1	7.92E-11	2.10E-07	+	0.233102	0.2044	+	T	C	0.400923172	0.0356101	+	T	C	0.3639	0.0394
rs55845504	LC_IGP84	IgG1 FBn/Bn total	14	66256968	1	1.20E-10	1.15E-09	-	-0.231349	-0.2417	+	T	C	0.400923172	0.0356914	+	T	C	0.3639	0.0397
rs55845504	LC_IGP62	IgG1 FG0n total/G0n	14	66256968	1	5.13E-14	2.38E-08	-	-0.268363	-0.2204	+	T	C	0.400923172	0.0353275	+	T	C	0.3639	0.0395
rs55845504	LC_IGP63	IgG1 FG1n total/G1n	14	66256968	1	6.54E-12	1.91E-08	-	-0.245879	-0.2218	+	T	C	0.400923172	0.0355364	+	T	C	0.3639	0.0395
rs55845504	LC_IGP64	IgG1 FG2n total/G2n	14	66256968	1	1.57E-09	1.27E-09	-	-0.216738	-0.2382	+	T	C	0.400923172	0.0356918	+	T	C	0.3639	0.0392
rs55845504	LC_IGP61	IgG1 Fn total	14	66256968	1	1.47E-12	6.69E-09	-	-0.253095	-0.2291	+	T	C	0.400923172	0.0354793	+	T	C	0.3639	0.0395
rs55845504	LC_IGP81	IgG1 Fn/Bn	14	66256968	1	1.53E-10	4.73E-07	-	-0.229506	-0.1973	+	T	C	0.400923172	0.0356125	+	T	C	0.3639	0.0392
rs55845504	LC_IGP21	IgG1 Fucosylation	14	66256968	1	1.14E-11	2.21E-09	-	-0.242017	-0.2363	+	T	C	0.40126942	0.0353947	+	T	C	0.3639	0.0395
rs55845504	LC_IGP11	IgG1_G0	14	66256968	1	1.97E-14	8.94E-10	+	0.27038	0.2404	+	T	C	0.400653635	0.0350096	+	T	C	0.3639	0.0392
rs55845504	LC_IGP_R1	IgG1_G0F/IgG1_G0	14	66256968	1	5.86E-14	5.17E-08	-	-0.265345	-0.2144	+	T	C	0.400653635	0.0350169	+	T	C	0.3639	0.0394
rs55845504	LC_IGP_R4	IgG1_G0FN/IgG1_G0N	14	66256968	1	6.53E-10	2.95E-09	-	-0.219925	-0.2352	+	T	C	0.400546234	0.0353902	+	T	C	0.3639	0.0396
rs55845504	LC_IGP52	IgG1_G0n	14	66256968	1	1.51E-14	6.81E-10	+	0.273711	0.2426	+	T	C	0.400923172	0.0352756	+	T	C	0.3639	0.0393
rs55845504	LC_IGP14	IgG1_G0N	14	66256968	1	9.06E-12	5.04E-08	+	0.242472	0.2128	+	T	C	0.400546234	0.0352907	+	T	C	0.3639	0.039
rs55845504	LC_IGP55	IgG1_G0Nn	14	66256968	1	4.70E-12	4.57E-08	+	0.247317	0.2138	+	T	C	0.400923172	0.0354981	+	T	C	0.3639	0.0391
rs55845504	LC_IGP12	IgG1_G1	14	66256968	1	5.20E-12	2.56E-08	+	0.244716	0.2198	+	T	C	0.400959071	0.0352028	+	T	C	0.3639	0.0395
rs55845504	LC_IGP_R2	IgG1_G1F/IgG1_G1	14	66256968	1	1.28E-12	4.10E-08	-	-0.25168	-0.216	+	T	C	0.400959071	0.0351876	+	T	C	0.3639	0.0394
rs55845504	LC_IGP_R5	IgG1_G1FN/IgG1_G1N	14	66256968	1	1.55E-09	1.04E-09	-	-0.215889	-0.2411	+	T	C	0.400124231	0.0355454	+	T	C	0.3639	0.0395
rs55845504	LC_IGP53	IgG1_G1n	14	66256968	1	6.73E-11	2.91E-08	+	0.233655	0.2188	+	T	C	0.400923172	0.035559	+	T	C	0.3639	0.0394
rs55845504	LC_IGP15	IgG1_G1N	14	66256968	1	1.31E-08	1.28E-06	+	0.203242	0.1898	+	T	C	0.400124231	0.0355724	+	T	C	0.3639	0.0392
rs55845504	LC_IGP56	IgG1_G1Nn	14	66256968	1	5.25E-09	1.74E-06	+	0.209635	0.1872	+	T	C	0.400923172	0.0357078	+	T	C	0.3639	0.0392
rs55845504	LC_IGP_R3	IgG1_G2F/IgG1_G2	14	66256968	1	9.97E-11	1.40E-09	-	-0.229764	-0.2371	+	T	C	0.400898533	0.0352938	+	T	C	0.3639	0.0392
rs55845504	LC_IGP_R8	IgG1_G2FS1/IgG1_G2S1	14	66256968	1	2.73E-08	7.91E-09	-	-0.197662	-0.2253	+	T	C	0.401261454	0.0353903	+	T	C	0.3639	0.039
rs11848463	LC_IGP78	IgG1 BG0n/G0n	14	66257294	1	5.70E-11	1.00E-09	+	0.228522	0.2307	+	A	G	0.498343556	0.0346441	+	A	G	0.4673	0.0378
rs11848463	LC_IGP79	IgG1 BG1n/G1n	14	66257294	1	7.25E-10	8.04E-11	+	0.215556	0.2452	+	A	G	0.498343556	0.0347782	+	A	G	0.4673	0.0377
rs11848463	LC_IGP77	IgG1 Bn	14	66257294	1	2.54E-10	7.64E-11	+	0.221261	0.2466	+	A	G	0.498343556	0.0347631	+	A	G	0.4673	0.0379
rs11848463	LC_IGP86	IgG1 Bn/Fn total	14	66257294	1	6.26E-11	5.64E-11	+	0.228563	0.2485	+	A	G	0.498343556	0.034726	+	A	G	0.4673	0.0379
rs11848463	LC_IGP84	IgG1 FBn/Bn total	14	66257294	1	2.21E-10	8.42E-15	-	-0.222414	-0.2967	+	A	G	0.498343556	0.0348231	+	A	G	0.4673	0.0382
rs11848463	LC_IGP62	IgG1 FG0n total/G0n	14	66257294	1	1.30E-12	3.19E-11	-	-0.246885	-0.2524	+	A	G	0.498343556	0.0345232	+	A	G	0.4673	0.038
rs11848463	LC_IGP63	IgG1 FG1n total/G1n	14	66257294	1	2.19E-12	9.16E-13	-	-0.245117	-0.2714	+	A	G	0.498343556	0.0346361	+	A	G	0.4673	0.038
rs11848463	LC_IGP64	IgG1 FG2n total/G2n	14	66257294	1	3.82E-09	6.30E-14	-	-0.206346	-0.2835	+	A	G	0.498343556	0.0348294	+	A	G	0.4673	0.0378

rs11848463	LC_IGP61	IgG1 Fn total	14	66257294	1	5.54E-12	1.04E-12	-	-0.240446	-0.271	+	A	G	0.498343556	0.0346313	+	A	G	0.4673	0.038
rs11848463	LC_IGP81	IgG1 Fn/Bn	14	66257294	1	9.00E-11	4.07E-10	-	-0.226611	-0.2359	+	A	G	0.498343556	0.0347221	+	A	G	0.4673	0.0377
rs11848463	LC_IGP21	IgG1 Fucosylation	14	66257294	1	1.75E-11	8.23E-13	-	-0.234536	-0.272	+	A	G	0.499438964	0.034623	+	A	G	0.4673	0.038
rs11848463	LC_IGP11	IgG1_G0	14	66257294	1	8.53E-13	5.55E-13	+	0.247099	0.273	+	A	G	0.498839951	0.0342728	+	A	G	0.4673	0.0379
rs11848463	LC_IGP_R1	IgG1_G0F/IgG1_G0	14	66257294	1	3.73E-12	1.05E-10	-	-0.239999	-0.245	+	A	G	0.498839951	0.0342878	+	A	G	0.4673	0.0379
rs11848463	LC_IGP_R4	IgG1_G0FN/IgG1_G0N	14	66257294	1	2.49E-10	9.95E-13	-	-0.219833	-0.2721	+	A	G	0.498670239	0.0345229	+	A	G	0.4673	0.0382
rs11848463	LC_IGP52	IgG1_G0n	14	66257294	1	7.74E-13	3.78E-13	+	0.249146	0.2755	+	A	G	0.498343556	0.0344875	+	A	G	0.4673	0.0379
rs11848463	LC_IGP14	IgG1_G0N	14	66257294	1	4.57E-13	2.85E-11	+	0.250905	0.2507	+	A	G	0.498670239	0.034384	+	A	G	0.4673	0.0377
rs11848463	LC_IGP55	IgG1_G0Nn	14	66257294	1	7.26E-13	2.25E-11	+	0.250141	0.2522	+	A	G	0.498343556	0.0345824	+	A	G	0.4673	0.0377
rs11848463	LC_IGP12	IgG1_G1	14	66257294	1	1.85E-11	5.05E-12	+	0.232685	0.2623	+	A	G	0.498839951	0.0343969	+	A	G	0.4673	0.038
rs11848463	LC_IGP_R2	IgG1_G1F/IgG1_G1	14	66257294	1	1.21E-12	7.88E-12	-	-0.24598	-0.2594	+	A	G	0.498839951	0.0343548	+	A	G	0.4673	0.0379
rs11848463	LC_IGP_R5	IgG1_G1FN/IgG1_G1N	14	66257294	1	5.75E-10	1.13E-15	-	-0.216275	-0.3048	+	A	G	0.497748155	0.0346871	+	A	G	0.4673	0.038
rs11848463	LC_IGP53	IgG1_G1n	14	66257294	1	8.84E-11	5.79E-12	+	0.226476	0.2614	+	A	G	0.498343556	0.0346868	+	A	G	0.4673	0.038
rs11848463	LC_IGP15	IgG1_G1N	14	66257294	1	6.51E-09	6.29E-10	+	0.202561	0.2333	+	A	G	0.497748155	0.0347196	+	A	G	0.4673	0.0377
rs11848463	LC_IGP56	IgG1_G1Nn	14	66257294	1	4.94E-09	1.01E-09	+	0.204809	0.2302	+	A	G	0.498343556	0.0348249	+	A	G	0.4673	0.0377
rs11848463	LC_IGP_R3	IgG1_G2F/IgG1_G2	14	66257294	1	1.80E-10	1.82E-13	-	-0.221382	-0.2777	+	A	G	0.499144866	0.0344917	+	A	G	0.4673	0.0377
rs11848463	LC_IGP148	IgG2 FG0n total/G0n	14	66257294	1	2.74E-08	2.99E-11	-	-0.193605	-0.2505	+	A	G	0.500054803	0.0346658	+	A	G	0.4672	0.0377
rs11848463	LC_IGP97	IgG2_G0	14	66257294	1	7.97E-09	1.85E-11	+	0.199974	0.2538	+	A	G	0.499144254	0.0344813	+	A	G	0.4673	0.0378
rs11848463	LC_IGP138	IgG2_G0n	14	66257294	1	3.15E-09	9.06E-12	+	0.206036	0.2581	+	A	G	0.500054803	0.0345886	+	A	G	0.4672	0.0378
rs11848443	LC_IGP78	IgG1 BG0n/G0n	14	66257419	1	5.73E-11	1.01E-09	+	0.228503	0.2306	+	T	C	0.498350682	0.034646	+	T	C	0.4673	0.0378
rs11848443	LC_IGP79	IgG1 BG1n/G1n	14	66257419	1	7.26E-10	8.10E-11	+	0.21556	0.2451	+	T	C	0.498350682	0.03478	+	T	C	0.4673	0.0377
rs11848443	LC_IGP77	IgG1 Bn	14	66257419	1	2.55E-10	7.70E-11	+	0.221254	0.2465	+	T	C	0.498350682	0.034765	+	T	C	0.4673	0.0379
rs11848443	LC_IGP86	IgG1 Bn/Fn total	14	66257419	1	6.29E-11	5.69E-11	+	0.228556	0.2484	+	T	C	0.498350682	0.0347279	+	T	C	0.4673	0.0379
rs11848443	LC_IGP84	IgG1 FBn/Bn total	14	66257419	1	2.22E-10	8.49E-15	-	-0.222408	-0.2966	+	T	C	0.498350682	0.034825	+	T	C	0.4673	0.0382
rs11848443	LC_IGP62	IgG1 FG0n total/G0n	14	66257419	1	1.31E-12	3.23E-11	-	-0.246847	-0.2523	+	T	C	0.498350682	0.0345253	+	T	C	0.4673	0.038
rs11848443	LC_IGP63	IgG1 FG1n total/G1n	14	66257419	1	2.20E-12	9.31E-13	-	-0.245112	-0.2713	+	T	C	0.498350682	0.034638	+	T	C	0.4673	0.038
rs11848443	LC_IGP64	IgG1 FG2n total/G2n	14	66257419	1	3.84E-09	6.40E-14	-	-0.206334	-0.2834	+	T	C	0.498350682	0.0348313	+	T	C	0.4673	0.0378
rs11848443	LC_IGP61	IgG1 Fn total	14	66257419	1	5.58E-12	1.05E-12	-	-0.240428	-0.2708	+	T	C	0.498350682	0.0346333	+	T	C	0.4673	0.038
rs11848443	LC_IGP81	IgG1 Fn/Bn	14	66257419	1	9.03E-11	4.10E-10	-	-0.226602	-0.2358	+	T	C	0.498350682	0.034724	+	T	C	0.4673	0.0377
rs11848443	LC_IGP21	IgG1 Fucosylation	14	66257419	1	1.75E-11	8.29E-13	-	-0.234532	-0.2719	+	T	C	0.499444821	0.034625	+	T	C	0.4673	0.038
rs11848443	LC_IGP11	IgG1_G0	14	66257419	1	8.61E-13	5.63E-13	+	0.247072	0.2729	+	T	C	0.498845754	0.0342748	+	T	C	0.4673	0.0378
rs11848443	LC_IGP_R1	IgG1_G0F/IgG1_G0	14	66257419	1	3.78E-12	1.06E-10	-	-0.239955	-0.2448	+	T	C	0.498845754	0.0342899	+	T	C	0.4673	0.0379
rs11848443	LC_IGP_R4	IgG1_G0FN/IgG1_G0N	14	66257419	1	2.50E-10	1.00E-12	-	-0.219816	-0.272	+	T	C	0.498677281	0.0345249	+	T	C	0.4673	0.0382
rs11848443	LC_IGP52	IgG1_G0n	14	66257419	1	7.81E-13	3.84E-13	+	0.249118	0.2754	+	T	C	0.498350682	0.0344895	+	T	C	0.4673	0.0379
rs11848443	LC_IGP14	IgG1_G0N	14	66257419	1	4.59E-13	2.87E-11	+	0.250901	0.2506	+	T	C	0.498677281	0.0343858	+	T	C	0.4673	0.0377
rs11848443	LC_IGP55	IgG1_G0Nn	14	66257419	1	7.29E-13	2.27E-11	+	0.250138	0.2521	+	T	C	0.498350682	0.0345843	+	T	C	0.4673	0.0377
rs11848443	LC_IGP12	IgG1_G1	14	66257419	1	1.87E-11	5.13E-12	+	0.232665	0.2622	+	T	C	0.498845754	0.034399	+	T	C	0.4673	0.038
rs11848443	LC_IGP_R2	IgG1_G1F/IgG1_G1	14	66257419	1	1.22E-12	8.01E-12	-	-0.245975	-0.2592	+	T	C	0.498845754	0.0343568	+	T	C	0.4673	0.0379
rs11848443	LC_IGP_R5	IgG1_G1FN/IgG1_G1N	14	66257419	1	5.75E-10	1.14E-15	-	-0.21628	-0.3047	+	T	C	0.497755228	0.034689	+	T	C	0.4673	0.038
rs11848443	LC_IGP53	IgG1_G1n	14	66257419	1	8.89E-11	5.87E-12	+	0.226455	0.2612	+	T	C	0.498350682	0.0346888	+	T	C	0.4673	0.038
rs11848443	LC_IGP15	IgG1_G1N	14	66257419	1	6.53E-09	6.33E-10	+	0.202556	0.2332	+	T	C	0.497755228	0.0347215	+	T	C	0.4673	0.0377
rs11848443	LC_IGP56	IgG1_G1Nn	14	66257419	1	4.96E-09	1.01E-09	+	0.204805	0.2301	+	T	C	0.498350682	0.0348268	+	T	C	0.4673	0.0377
rs11848443	LC_IGP_R3	IgG1_G2F/IgG1_G2	14	66257419	1	1.81E-10	1.85E-13	-	-0.221371	-0.2776	+	T	C	0.499150672	0.0344937	+	T	C	0.4673	0.0377
rs11848443	LC_IGP148	IgG2 FG0n total/G0n	14	66257419	1	2.73E-08	3.03E-11	-	-0.193625	-0.2504	+	T	C	0.500060653	0.0346677	+	T	C	0.4672	0.0377
rs11848443	LC_IGP97	IgG2_G0	14	66257419	1	7.98E-09	1.87E-11	+	0.199976	0.2537	+	T	C	0.499150061	0.0344832	+	T	C	0.4673	0.0378
rs11848443	LC_IGP138	IgG2_G0n	14	66257419	1	3.15E-09	9.17E-12	+	0.206039	0.2579	+	T	C	0.500060653	0.0345906	+	T	C	0.4672	0.0378
rs11848444	LC_IGP78	IgG1 BG0n/G0n	14	66257467	1	5.75E-11	1.08E-09	+	0.228491	0.23	+	G	A	0.49835254	0.0346466	+	G	A	0.4675	0.0377
rs11848444	LC_IGP79	IgG1 BG1n/G1n	14	66257467	1	7.26E-10	8.90E-11	+	0.215559	0.2444	+	G	A	0.49835254	0.0347806	+	G	A	0.4675	0.0377
rs11848444	LC_IGP77	IgG1 Bn	14	66257467	1	2.56E-10	8.37E-11	+	0.221248	0.2459	+	G	A	0.49835254	0.0347656	+	G	A	0.4675	0.0379
rs11848444	LC_IGP86	IgG1 Bn/Fn total	14	66257467	1	6.30E-11	6.17E-11	+	0.228549	0.2478	+	G	A	0.49835254	0.0347285	+	G	A	0.4675	0.0379
rs11848444	LC_IGP84	IgG1 FBn/Bn total	14	66257467	1	2.22E-10	9.66E-15	-	-0.222404	-0.2958	+	G	A	0.49835254	0.0348255	+	G	A	0.4675	0.0382
rs11848444	LC_IGP62	IgG1 FG0n total/G0n	14	66257467	1	1.32E-12	3.57E-11	-	-0.246832	-0.2516	+	G	A	0.49835254	0.0345259	+	G	A	0.4675	0.038
rs11848444	LC_IGP63	IgG1 FG1n total/G1n	14	66257467	1	2.20E-12	1.07E-12	-	-0.245111	-0.2704	+	G	A	0.49835254	0.0346385	+	G	A	0.4675	0.038
rs11848444	LC_IGP64	IgG1 FG2n total/G2n	14	66257467	1	3.84E-09	7.01E-14	-	-0.206331	-0.2828	+	G	A	0.49835254	0.0348319	+	G	A	0.4675	0.0378
rs11848444	LC_IGP61	IgG1 Fn total	14	66257467	1	5.59E-12	1.18E-12	-	-0.24042	-0.2701	+	G	A	0.49835254	0.0346339	+	G	A	0.4675	0.038
rs11848444	LC_IGP81	IgG1 Fn/Bn	14	66257467	1	9.05E-11	4.44E-10	-	-0.226594	-0.2352	+	G	A	0.49835254	0.0347246	+	G	A	0.4675	0.0377
rs11848444	LC_IGP21	IgG1 Fucosylation	14	66257467	1	1.75E-11	9.10E-13	-	-0.234534	-0.2712	+	G	A	0.499446054	0.0346255	+	G	A	0.4675	0.038
rs11848444	LC_IGP11	IgG1_G0	14	66257467	1	8.62E-13	5.97E-13	+	0.247066	0.2724	+	G	A	0.498846976	0.0342754	+	G	A	0.4675	0.0378
rs11848444	LC_IGP_R1	IgG1_G0F/IgG1_G0	14	66257467	1	3.80E-12	1.18E-10	-	-0.239938	-0.2441	+	G	A	0.498846976	0.0342906	+	G	A	0.4675	0.0379
rs11848444	LC_IGP_R4	IgG1_G0FN/IgG1_G0N	14	66257467	1	2.51E-10	1.11E-12	-	-0.219807	-0.2713	+	G	A	0.498679118	0.0345254	+	G	A	0.4675	0.0381
rs11848444	LC_IGP52	IgG1_G0n	14	66257467	1	7.83E-13	4.10E-13	+	0.249112	0.2749	+	G	A	0.49835254	0.03449	+	G	A	0.4675	0.0379
rs11848444	LC_IGP14	IgG1_G0N	14	66257467	1	4.59E-13	2.98E-11	+	0.250899	0.2502	+	G	A	0.498679118	0.0343864	+	G	A	0.4675	0.0376
rs11848444	LC_IGP55	IgG1_G0Nn	14	66257467	1	7.30E-13	2.37E-11	+	0.250135	0.2518	+	G	A	0.49835254	0.0345848	+	G	A	0.4675	0.0377
rs11848444	LC_IGP12	IgG1_G1	14	66257467	1	1.87E-11	5.95E-12	+	0.232658	0.2612	+	G	A	0.498846976	0.0343996	+	G	A	0.4675	0.038
rs11848444	LC_IGP_R2	IgG1_G1F/IgG1_G1	14	66257467	1	1.22E-12	9.14E-12	-	-0.245974	-0.2584	+	G	A	0.498846976	0.0343574	+	G	A	0.4675	0.0379
rs11848444	LC_IGP_R5	IgG1_G1FN/IgG1_G1N	14	66257467	1	5.76E-10	1.31E-15	-	-0.216281	-0.3039	+	G	A	0.497757073	0.0346895	+	G	A	0.4675	0.038
rs11848444	LC_IGP53	IgG1_G1n	14	66257467	1	8.91E-11	6.86E-12	+	0.226447	0.2603	+	G	A	0.49835254	0.0346894	+	G	A	0.4675	0.0379
rs11848444	LC_IGP15	IgG1_G1N	14	66257467	1	6.54E-09	7.00E-10	+	0.20255	0.2325	+	G	A	0.497757073	0.034722	+	G	A	0.4675	0.0377
rs11848444	LC_IGP56	IgG1_G1Nn	14	66257467	1	4.97E-09	1.13E-09	+	0.204798	0.2293	+	G	A	0.49835254	0.0348274	+	G	A	0.4675	0.0377
rs11848444	LC_IGP_R3	IgG1_G2F/IgG1_G2	14	66257467	1	1.81E-10	2.04E-13	-	-0.221369	-0.2769	+	G	A	0.499151895	0.0344943	+	G	A	0.4675	0.0377
rs11848444	LC_IGP148	IgG2 FG0n total/G0n	14	66257467	1	2.73E-08	3.49E-11	-	-0.193635	-0.2495	+	G	A	0.500061884	0.0346683	+	G	A	0.4674	0.0377
rs11848444	LC_IGP97	IgG2_G0	14	66257467	1	7.98E-09	2.07E-11	+	0.199984	0.2529	+	G	A	0.499151284	0.0344838	+	G	A	0.4675	0.0378
rs11848444	LC_IGP138	IgG2_G0n	14	66257467	1	3.15E-09	1.03E-11	+	0.206047	0.2572	+	G	A	0.500061884	0.0345911	+	G	A	0.4674	0.0378
rs61988977	LC_IGP78	IgG1 BG0n/G0n	14	66258032	1	3.82E-11	1.31E-09	+	0.231351	0.2292	+	A	C	0.501946097	0.0347538	+	A	C	0.4695	0.0378
rs61988977	LC_IGP79	IgG1 BG1n/G1n	14	66258032	1	1.07E-09	7.28E-11	+	0.214123	0.2459	+	A	C	0.501946097	0.0349051	+	A	C	0.4695	0.0377
rs61988977	LC_IGP77	IgG1 Bn	14	66258032	1	2.79E-10	8.28E-11	+	0.221527	0.2462	+	A	C	0.501946097	0.0348836	+	A	C	0.4695	0.0379
rs61988977	LC_IGP86	IgG1 Bn/Fn total	14	66258032	1	6.90E-11	6.14E-11	+	0.228844	0.2481	+	A	C	0.501946097	0.0348465	+	A	C	0.4695	0.0379
rs61988977	LC_IGP84	IgG1 FBn/Bn total	14	66258032	1	1.90E-10	1.19E-14	-	-0.223977	-0.295	+	A	C	0.501946097	0.0349387	+	A	C	0.4695	0.0382
rs61988977	LC_IGP62	IgG1 FG0n total/G0n	14	66258032	1	1.23E-12	4.68E-11	-	-0.248001	-0.2503	+	A	C	0.501946097	0.0346398	+	A	C	0.4695	0.038
rs61988977	LC_IGP63	IgG1 FG1n total/G1n	14	66258032	1	2.77E-12	1.04E-12	-	-0.244817	-0.2708	+	A	C	0.501946097	0.0347592	+	A	C	0.4695	0.038
rs61988977	LC_IGP64	IgG1 FG2n total/G2n	14	66258032	1	4.20E-09	6.56E-14	-	-0.206509	-0.2834	+	A	C	0.501946097	0.0349502	+	A	C	0.4695	0.0378
rs61988977	LC_IGP61	IgG1 Fn total	14	66258032	1	5.50E-12	1.28E-12	-	-0.241306	-0.27	+	A	C	0.501946097	0.0347493	+	A	C	0.4695	0.038
rs61988977	LC_IGP81	IgG1 Fn/Bn	14	66258032	1	1.02E-10	4.34E-10	-	-0.226721	-0.2356	+	A	C	0.501946097	0.0348432	+	A	C	0.4695	0.0378
rs61988977	LC_IGP21	IgG1 Fucosylation	14	66258032	1	1.71E-11	1.01E-12	-	-0.235467	-0.271	+	A	C	0.50301603	0.034742	+	A	C	0.4695	0.038
rs61988977	LC_IGP11	IgG1_G0	14	66258032	1	1.04E-12	7.73E-13	+	0.246986	0.2714	+	A	C	0.502384239	0.0343933	+	A	C	0.4695	0.0379
rs61988977	LC_IGP_R1	IgG1_G0F/IgG1_G0	14	66258032	1	4.08E-12	1.50E-10	-	-0.240381	-0.243	+	A	C	0.502384239	0.034406	+	A	C	0.4695	0.0379
rs61988977	LC_IGP_R4	IgG1_G0FN/IgG1_G0N	14	66258032	1	1.65E-10	1.58E-12	-	-0.222743	-0.2697	+	A	C	0.502230863	0.0346312	+	A	C	0.4695	0.0382
rs61988977	LC_IGP52	IgG1_G0n	14	66258032	1	9.37E-13	5.25E-13	+	0.249095	0.2739	+	A	C	0.501946097	0.0346091	+	A	C	0.4695	0.038
rs61988977	LC_IGP14	IgG1_G0N	14	66258032	1	3.74E-13	3.55E-11	+	0.252679	0.2496	+	A	C	0.502230863	0.0344961	+	A	C	0.4695	0.0377
rs61988977	LC_IGP55	IgG1_G0Nn	14	66258032	1	5.49E-13	2.79E-11	+	0.252308	0.2512	+	A	C	0.501946097	0.0346945	+	A	C	0.4695	0.0377
rs61988977	LC_IGP12	IgG1_G1	14	66258032	1	1.96E-11	6.53E-12	+	0.233196	0.2611	+	A	C	0.502384239	0.0345148	+	A	C	0.4695	0.038
rs61988977	LC_IGP_R2	IgG1_G1F/IgG1_G1	14	66258032	1	1.46E-12	9.07E-12	-	-0.245944	-0.2587	+	A	C	0.502384239	0.0344752	+	A	C	0.4695	0.0379
rs61988977	LC_IGP_R5	IgG1_G1FN/IgG1_G1N	14	66258032	1	6.97E-10	1.32E-15	-	-0.215952	-0.3041	+	A	C	0.501324108	0.034808	+	A	C	0.4695	0.038
rs61988977	LC_IGP53	IgG1_G1n	14	66258032	1	9.03E-11	7.39E-12	+	0.227137	0.2602	+	A	C	0.501946097	0.0348056	+	A	C	0.4695	0.038
rs61988977	LC_IGP15	IgG1_G1N	14	66258032	1	8.97E-09	6.14E-10	+	0.201371	0.2336	+	A	C	0.501324108	0.0348433	+	A	C	0.4695	0.0378
rs61988977	LC_IGP56	IgG1_G1Nn	14	66258032	1	6.58E-09	9.79E-10	+	0.20385	0.2305	+	A	C	0.501946097	0.0349498	+	A	C	0.4695	0.0377
rs61988977	LC_IGP_R3	IgG1_G2F/IgG1_G2	14	66258032	1	1.86E-10	1.88E-13	-	-0.221974	-0.2776	+	A	C	0.50269132	0.0346099	+	A	C	0.4695	0.0377
rs61988977	LC_IGP97	IgG2_G0	14	66258032	1	1.72E-08	3.84E-11	+	0.196148	0.2496	+	A	C	0.502690709	0.0346146	+	A	C	0.4695	0.0378
rs61988977	LC_IGP138	IgG2_G0n	14	66258032	1	6.84E-09	1.91E-11	+	0.2023	0.2538	+	A	C	0.503627463	0.0347246	+	A	C	0.4694	0.0378
rs61988978	LC_IGP78	IgG1 BG0n/G0n	14	66258111	1	2.71E-09	3.07E-10	+	0.209207	0.2409	+	C	T	0.491254647	0.0349711	+	C	T	0.4516	0.0383
rs61988978	LC_IGP79	IgG1 BG1n/G1n	14	66258111	1	6.45E-09	1.87E-11	+	0.204675	0.2564	+	C	T	0.491254647	0.0350708	+	C	T	0.4516	0.0382
rs61988978	LC_IGP77	IgG1 Bn	14	66258111	1	3.14E-09	2.39E-11	+	0.208887	0.2564	+	C	T	0.491254647	0.0350625	+	C	T	0.4516	0.0384
rs61988978	LC_IGP86	IgG1 Bn/Fn total	14	66258111	1	7.58E-10	1.70E-11	+	0.216834	0.2585	+	C	T	0.491254647	0.0350248	+	C	T	0.4516	0.0384
rs61988978	LC_IGP84	IgG1 FBn/Bn total	14	66258111	1	2.35E-09	1.25E-14	-	-0.21093	-0.2985	+	C	T	0.491254647	0.0351199	+	C	T	0.4516	0.0387
rs61988978	LC_IGP62	IgG1 FG0n total/G0n	14	66258111	1	1.28E-11	1.21E-11	-	-0.237462	-0.2611	+	C	T	0.491254647	0.0348161	+	C	T	0.4516	0.0385
rs61988978	LC_IGP63	IgG1 FG1n total/G1n	14	66258111	1	2.52E-11	2.40E-13	-	-0.234731	-0.2817	+	C	T	0.491254647	0.0349332	+	C	T	0.4516	0.0385
rs61988978	LC_IGP64	IgG1 FG2n total/G2n	14	66258111	1	6.21E-09	2.56E-14	-	-0.204989	-0.291	+	C	T	0.491254647	0.0350863	+	C	T	0.4516	0.0382
rs61988978	LC_IGP61	IgG1 Fn total	14	66258111	1	3.33E-11	3.41E-13	-	-0.233154	-0.2802	+	C	T	0.491254647	0.0349146	+	C	T	0.4516	0.0385
rs61988978	LC_IGP81	IgG1 Fn/Bn	14	66258111	1	1.06E-09	9.09E-11	-	-0.214891	-0.2476	+	C	T	0.491254647	0.0350203	+	C	T	0.4516	0.0382
rs61988978	LC_IGP21	IgG1 Fucosylation	14	66258111	1	1.29E-10	2.01E-13	-	-0.225893	-0.2831	+	C	T	0.491760173	0.0349096	+	C	T	0.4516	0.0385
rs61988978	LC_IGP28	IgG1 G1S1/(G1+G1S1)	14	66258111	1	1.38E-08	7.69E-10	-	-0.198224	-0.2271	+	C	T	0.491527829	0.0347444	+	C	T	0.4516	0.0369
rs61988978	LC_IGP11	IgG1_G0	14	66258111	1	2.06E-12	7.09E-13	+	0.244662	0.2749	+	C	T	0.491232743	0.0345315	+	C	T	0.4516	0.0383
rs61988978	LC_IGP_R1	IgG1_G0F/IgG1_G0	14	66258111	1	1.44E-11	3.64E-11	-	-0.235072	-0.2541	+	C	T	0.491232743	0.0345563	+	C	T	0.4516	0.0384
rs61988978	LC_IGP_R4	IgG1_G0FN/IgG1_G0N	14	66258111	1	1.57E-08	6.86E-13	-	-0.198025	-0.2777	+	C	T	0.491052664	0.0348519	+	C	T	0.4516	0.0387
rs61988978	LC_IGP52	IgG1_G0n	14	66258111	1	2.66E-12	4.33E-13	+	0.245012	0.2781	+	C	T	0.491254647	0.0347578	+	C	T	0.4516	0.0384
rs61988978	LC_IGP14	IgG1_G0N	14	66258111	1	6.10E-11	2.24E-11	+	0.228694	0.255	+	C	T	0.491052664	0.0347277	+	C	T	0.4516	0.0381
rs61988978	LC_IGP55	IgG1_G0Nn	14	66258111	1	3.98E-11	1.58E-11	+	0.23219	0.2573	+	C	T	0.491254647	0.0349122	+	C	T	0.4516	0.0382
rs61988978	LC_IGP12	IgG1_G1	14	66258111	1	6.56E-11	1.64E-12	+	0.227895	0.2716	+	C	T	0.491232743	0.0346645	+	C	T	0.4516	0.0384
rs61988978	LC_IGP_R2	IgG1_G1F/IgG1_G1	14	66258111	1	8.36E-12	1.49E-12	-	-0.238376	-0.2714	+	C	T	0.491232743	0.034636	+	C	T	0.4516	0.0384

rs61988978	LC_IGP_R5	IgG1_G1FN/IgG1_G1N	14	66258111	1	3.13E-09	1.46E-15	-	-0.208327	-0.3074	+	C	T	0.490712792	0.0349678	+	C	T	0.4516	0.0385
rs61988978	LC_IGP53	IgG1_G1n	14	66258111	1	4.61E-10	1.62E-12	+	0.219262	0.2714	+	C	T	0.491254647	0.0349675	+	C	T	0.4516	0.0384
rs61988978	LC_IGP15	IgG1_G1N	14	66258111	1	3.35E-08	1.67E-10	+	0.194201	0.2441	+	C	T	0.490712792	0.0349993	+	C	T	0.4516	0.0382
rs61988978	LC_IGP56	IgG1_G1Nn	14	66258111	1	3.06E-08	2.27E-10	+	0.195396	0.242	+	C	T	0.491254647	0.0351101	+	C	T	0.4516	0.0382
rs61988978	LC_IGP_R32	IgG1_G1S1/IgG1_G1	14	66258111	1	1.82E-08	9.31E-10	-	-0.196612	-0.2263	+	C	T	0.491527829	0.0347553	+	C	T	0.4516	0.037
rs61988978	LC_IGP_R3	IgG1_G2F/IgG1_G2	14	66258111	1	3.74E-10	5.67E-14	-	-0.219036	-0.2866	+	C	T	0.491533007	0.0347486	+	C	T	0.4516	0.0381
rs61988978	LC_IGP97	IgG2_G0	14	66258111	1	6.02E-09	3.80E-12	+	0.203006	0.2656	+	C	T	0.491532396	0.0347171	+	C	T	0.4516	0.0382
rs61988978	LC_IGP138	IgG2_G0n	14	66258111	1	2.95E-09	1.45E-12	+	0.207844	0.2712	+	C	T	0.4923867	0.0348299	+	C	T	0.4515	0.0383
rs28459012	LC_IGP78	IgG1 BG0n/G0n	14	66258528	1	4.81E-11	1.39E-09	+	0.229942	0.2279	+	C	T	0.500496283	0.0347238	+	C	T	0.4685	0.0376
rs28459012	LC_IGP79	IgG1 BG1n/G1n	14	66258528	1	9.06E-10	1.07E-10	+	0.214845	0.2426	+	C	T	0.500496283	0.0348665	+	C	T	0.4685	0.0376
rs28459012	LC_IGP77	IgG1 Bn	14	66258528	1	2.75E-10	1.08E-10	+	0.221379	0.2437	+	C	T	0.500496283	0.0348483	+	C	T	0.4685	0.0378
rs28459012	LC_IGP86	IgG1 Bn/Fn total	14	66258528	1	6.97E-11	7.69E-11	+	0.228558	0.2459	+	C	T	0.500496283	0.0348118	+	C	T	0.4685	0.0378
rs28459012	LC_IGP84	IgG1 FBn/Bn total	14	66258528	1	2.41E-10	1.53E-14	-	-0.222471	-0.2928	+	C	T	0.500496283	0.0349087	+	C	T	0.4685	0.0381
rs28459012	LC_IGP62	IgG1 FG0n total/G0n	14	66258528	1	1.84E-12	4.88E-11	-	-0.245829	-0.2491	+	C	T	0.500496283	0.0346136	+	C	T	0.4685	0.0379
rs28459012	LC_IGP63	IgG1 FG1n total/G1n	14	66258528	1	3.40E-12	1.46E-12	-	-0.243584	-0.268	+	C	T	0.500496283	0.0347286	+	C	T	0.4685	0.0379
rs28459012	LC_IGP64	IgG1 FG2n total/G2n	14	66258528	1	5.80E-09	1.01E-13	-	-0.204434	-0.2801	+	C	T	0.500496283	0.034922	+	C	T	0.4685	0.0377
rs28459012	LC_IGP61	IgG1 Fn total	14	66258528	1	7.64E-12	1.67E-12	-	-0.239446	-0.2675	+	C	T	0.500496283	0.0347214	+	C	T	0.4685	0.0379
rs28459012	LC_IGP81	IgG1 Fn/Bn	14	66258528	1	9.95E-11	5.81E-10	-	-0.226628	-0.2329	+	C	T	0.500496283	0.0348078	+	C	T	0.4685	0.0376
rs28459012	LC_IGP21	IgG1 Fucosylation	14	66258528	1	2.52E-11	1.12E-12	-	-0.233266	-0.2693	+	C	T	0.501568434	0.0347153	+	C	T	0.4685	0.0378
rs28459012	LC_IGP11	IgG1_G0	14	66258528	1	1.34E-12	7.50E-13	+	0.245575	0.2704	+	C	T	0.500949908	0.0343645	+	C	T	0.4685	0.0377
rs28459012	LC_IGP_R1	IgG1_G0F/IgG1_G0	14	66258528	1	5.84E-12	1.59E-10	-	-0.238417	-0.2416	+	C	T	0.500949908	0.0343795	+	C	T	0.4685	0.0378
rs28459012	LC_IGP_R4	IgG1_G0FN/IgG1_G0N	14	66258528	1	2.34E-10	1.66E-12	-	-0.22069	-0.2684	+	C	T	0.500797918	0.034604	+	C	T	0.4685	0.038
rs28459012	LC_IGP52	IgG1_G0n	14	66258528	1	1.21E-12	5.25E-13	+	0.247629	0.2727	+	C	T	0.500496283	0.0345798	+	C	T	0.4685	0.0378
rs28459012	LC_IGP14	IgG1_G0N	14	66258528	1	4.12E-13	3.62E-11	+	0.251986	0.2484	+	C	T	0.500797918	0.0344638	+	C	T	0.4685	0.0375
rs28459012	LC_IGP55	IgG1_G0Nn	14	66258528	1	6.15E-13	2.94E-11	+	0.251525	0.2498	+	C	T	0.500496283	0.034662	+	C	T	0.4685	0.0376
rs28459012	LC_IGP12	IgG1_G1	14	66258528	1	2.99E-11	8.17E-12	+	0.230853	0.2588	+	C	T	0.500949908	0.0344895	+	C	T	0.4685	0.0379
rs28459012	LC_IGP_R2	IgG1_G1F/IgG1_G1	14	66258528	1	1.91E-12	1.27E-11	-	-0.244432	-0.2558	+	C	T	0.500949908	0.0344468	+	C	T	0.4685	0.0378
rs28459012	LC_IGP_R5	IgG1_G1FN/IgG1_G1N	14	66258528	1	7.19E-10	1.90E-15	-	-0.215569	-0.3013	+	C	T	0.499884994	0.0347743	+	C	T	0.4685	0.0379
rs28459012	LC_IGP53	IgG1_G1n	14	66258528	1	1.37E-10	9.61E-12	+	0.224733	0.2577	+	C	T	0.500496283	0.0347795	+	C	T	0.4685	0.0378
rs28459012	LC_IGP15	IgG1_G1N	14	66258528	1	8.25E-09	8.21E-10	+	0.201666	0.2309	+	C	T	0.499884994	0.0348071	+	C	T	0.4685	0.0376
rs28459012	LC_IGP56	IgG1_G1Nn	14	66258528	1	6.07E-09	1.35E-09	+	0.204111	0.2276	+	C	T	0.500496283	0.034913	+	C	T	0.4685	0.0376
rs28459012	LC_IGP_R3	IgG1_G2F/IgG1_G2	14	66258528	1	2.78E-10	2.84E-13	-	-0.219615	-0.2744	+	C	T	0.501256112	0.034584	+	C	T	0.4685	0.0376
rs28459012	LC_IGP97	IgG2_G0	14	66258528	1	1.74E-08	2.66E-11	+	0.195878	0.2505	+	C	T	0.501255501	0.0345806	+	C	T	0.4685	0.0376
rs28459012	LC_IGP138	IgG2_G0n	14	66258528	1	6.85E-09	1.33E-11	+	0.202087	0.2546	+	C	T	0.50218165	0.0346896	+	C	T	0.4684	0.0376
rs28616692	LC_IGP78	IgG1 BG0n/G0n	14	66258683	1	4.84E-11	1.28E-09	+	0.229924	0.2283	+	T	C	0.500501859	0.0347252	+	T	C	0.4684	0.0376
rs28616692	LC_IGP79	IgG1 BG1n/G1n	14	66258683	1	9.06E-10	9.97E-11	+	0.214856	0.243	+	T	C	0.500501859	0.0348678	+	T	C	0.4684	0.0376
rs28616692	LC_IGP77	IgG1 Bn	14	66258683	1	2.75E-10	1.00E-10	+	0.221383	0.2441	+	T	C	0.500501859	0.0348497	+	T	C	0.4684	0.0378
rs28616692	LC_IGP86	IgG1 Bn/Fn total	14	66258683	1	6.98E-11	7.17E-11	+	0.22856	0.2462	+	T	C	0.500501859	0.0348131	+	T	C	0.4684	0.0378
rs28616692	LC_IGP84	IgG1 FBn/Bn total	14	66258683	1	2.42E-10	1.28E-14	-	-0.222477	-0.2934	+	T	C	0.500501859	0.03491	+	T	C	0.4684	0.0381
rs28616692	LC_IGP62	IgG1 FG0n total/G0n	14	66258683	1	1.86E-12	4.39E-11	-	-0.245783	-0.2496	+	T	C	0.500501859	0.0346151	+	T	C	0.4684	0.0379
rs28616692	LC_IGP63	IgG1 FG1n total/G1n	14	66258683	1	3.40E-12	1.30E-12	-	-0.243583	-0.2684	+	T	C	0.500501859	0.03473	+	T	C	0.4684	0.0378
rs28616692	LC_IGP64	IgG1 FG2n total/G2n	14	66258683	1	5.82E-09	8.95E-14	-	-0.204428	-0.2806	+	T	C	0.500501859	0.0349233	+	T	C	0.4684	0.0376
rs28616692	LC_IGP61	IgG1 Fn total	14	66258683	1	7.68E-12	1.48E-12	-	-0.239428	-0.268	+	T	C	0.500501859	0.0347228	+	T	C	0.4684	0.0379
rs28616692	LC_IGP81	IgG1 Fn/Bn	14	66258683	1	9.97E-11	5.38E-10	-	-0.226627	-0.2333	+	T	C	0.500501859	0.0348091	+	T	C	0.4684	0.0376
rs28616692	LC_IGP21	IgG1 Fucosylation	14	66258683	1	2.52E-11	1.05E-12	-	-0.23327	-0.2695	+	T	C	0.501572441	0.0347167	+	T	C	0.4684	0.0378
rs28616692	LC_IGP11	IgG1_G0	14	66258683	1	1.36E-12	7.19E-13	+	0.245522	0.2705	+	T	C	0.500953879	0.0343662	+	T	C	0.4684	0.0377
rs28616692	LC_IGP_R1	IgG1_G0F/IgG1_G0	14	66258683	1	5.91E-12	1.45E-10	-	-0.238361	-0.2421	+	T	C	0.500953879	0.0343811	+	T	C	0.4684	0.0378
rs28616692	LC_IGP_R4	IgG1_G0FN/IgG1_G0N	14	66258683	1	2.35E-10	1.45E-12	-	-0.220675	-0.269	+	T	C	0.500803429	0.0346053	+	T	C	0.4684	0.038
rs28616692	LC_IGP52	IgG1_G0n	14	66258683	1	1.23E-12	5.00E-13	+	0.247578	0.2729	+	T	C	0.500501859	0.0345814	+	T	C	0.4684	0.0378
rs28616692	LC_IGP14	IgG1_G0N	14	66258683	1	4.14E-13	3.52E-11	+	0.251971	0.2485	+	T	C	0.500803429	0.0344652	+	T	C	0.4684	0.0375
rs28616692	LC_IGP55	IgG1_G0Nn	14	66258683	1	6.18E-13	2.84E-11	+	0.251513	0.25	+	T	C	0.500501859	0.0346634	+	T	C	0.4684	0.0376
rs28616692	LC_IGP12	IgG1_G1	14	66258683	1	3.00E-11	7.24E-12	+	0.230834	0.2593	+	T	C	0.500953879	0.034491	+	T	C	0.4684	0.0378
rs28616692	LC_IGP_R2	IgG1_G1F/IgG1_G1	14	66258683	1	1.91E-12	1.14E-11	-	-0.244428	-0.2563	+	T	C	0.500953879	0.0344482	+	T	C	0.4684	0.0378
rs28616692	LC_IGP_R5	IgG1_G1FN/IgG1_G1N	14	66258683	1	7.18E-10	1.61E-15	-	-0.215581	-0.3019	+	T	C	0.499890529	0.0347755	+	T	C	0.4684	0.0379
rs28616692	LC_IGP53	IgG1_G1n	14	66258683	1	1.37E-10	8.42E-12	+	0.224717	0.2583	+	T	C	0.500501859	0.0347809	+	T	C	0.4684	0.0378
rs28616692	LC_IGP15	IgG1_G1N	14	66258683	1	8.26E-09	7.69E-10	+	0.201667	0.2312	+	T	C	0.499890529	0.0348084	+	T	C	0.4684	0.0376
rs28616692	LC_IGP56	IgG1_G1Nn	14	66258683	1	6.08E-09	1.25E-09	+	0.204116	0.228	+	T	C	0.500501859	0.0349143	+	T	C	0.4684	0.0375
rs28616692	LC_IGP_R3	IgG1_G2F/IgG1_G2	14	66258683	1	2.79E-10	2.57E-13	-	-0.219604	-0.2748	+	T	C	0.501260086	0.0345855	+	T	C	0.4684	0.0376
rs28616692	LC_IGP97	IgG2_G0	14	66258683	1	1.75E-08	2.62E-11	+	0.195858	0.2505	+	T	C	0.501259474	0.0345821	+	T	C	0.4684	0.0376
rs28616692	LC_IGP138	IgG2_G0n	14	66258683	1	6.88E-09	1.30E-11	+	0.202071	0.2547	+	T	C	0.502185653	0.034691	+	T	C	0.4683	0.0376
rs4131097	LC_IGP78	IgG1 BG0n/G0n	14	66259165	1	5.12E-10	2.12E-10	+	0.247725	0.2812	+	C	G	0.271093866	0.0396123	+	C	G	0.2309	0.0443
rs4131097	LC_IGP79	IgG1 BG1n/G1n	14	66259165	1	1.94E-08	1.34E-11	+	0.224638	0.2988	+	C	G	0.271093866	0.0397917	+	C	G	0.2309	0.0442
rs4131097	LC_IGP77	IgG1 Bn	14	66259165	1	2.72E-09	2.06E-11	+	0.237783	0.2975	+	C	G	0.271093866	0.0397524	+	C	G	0.2309	0.0444
rs4131097	LC_IGP86	IgG1 Bn/Fn total	14	66259165	1	5.27E-10	1.39E-11	+	0.248121	0.3003	+	C	G	0.271093866	0.0397044	+	C	G	0.2309	0.0444
rs4131097	LC_IGP84	IgG1 FBn/Bn total	14	66259165	1	3.31E-11	1.17E-14	-	-0.265265	-0.3455	+	C	G	0.271093866	0.0397182	+	C	G	0.2309	0.0448
rs4131097	LC_IGP62	IgG1 FG0n total/G0n	14	66259165	1	1.22E-13	8.80E-12	-	-0.294421	-0.3043	+	C	G	0.271093866	0.0393648	+	C	G	0.2309	0.0446
rs4131097	LC_IGP63	IgG1 FG1n total/G1n	14	66259165	1	3.17E-11	7.37E-13	-	-0.264831	-0.319	+	C	G	0.271093866	0.0396147	+	C	G	0.2309	0.0445
rs4131097	LC_IGP64	IgG1 FG2n total/G2n	14	66259165	1	1.33E-10	2.70E-14	-	-0.256634	-0.3364	+	C	G	0.271093866	0.0396904	+	C	G	0.2309	0.0442
rs4131097	LC_IGP61	IgG1 Fn total	14	66259165	1	1.92E-12	6.69E-13	-	-0.280407	-0.3201	+	C	G	0.271093866	0.0395195	+	C	G	0.2309	0.0446
rs4131097	LC_IGP81	IgG1 Fn/Bn	14	66259165	1	2.03E-09	5.42E-11	-	-0.239535	-0.2899	+	C	G	0.271093866	0.0397236	+	C	G	0.2309	0.0442
rs4131097	LC_IGP21	IgG1 Fucosylation	14	66259165	1	1.59E-11	3.09E-13	-	-0.26815	-0.3246	+	C	G	0.271298705	0.0395035	+	C	G	0.2309	0.0445
rs4131097	LC_IGP11	IgG1_G0	14	66259165	1	4.29E-14	2.86E-13	+	0.297863	0.3237	+	C	G	0.270950825	0.0390922	+	C	G	0.2309	0.0443
rs4131097	LC_IGP_R1	IgG1_G0F/IgG1_G0	14	66259165	1	1.99E-13	4.20E-11	-	-0.289873	-0.2933	+	C	G	0.270950825	0.0391108	+	C	G	0.2309	0.0445
rs4131097	LC_IGP_R4	IgG1_G0FN/IgG1_G0N	14	66259165	1	1.12E-10	1.82E-13	-	-0.25626	-0.3291	+	C	G	0.271002143	0.03947	+	C	G	0.2309	0.0447
rs4131097	LC_IGP52	IgG1_G0n	14	66259165	1	2.85E-14	2.42E-13	+	0.301633	0.3254	+	C	G	0.271093866	0.0393015	+	C	G	0.2309	0.0444
rs4131097	LC_IGP14	IgG1_G0N	14	66259165	1	6.36E-11	9.22E-12	+	0.259526	0.301	+	C	G	0.271002143	0.0394468	+	C	G	0.2309	0.0441
rs4131097	LC_IGP55	IgG1_G0Nn	14	66259165	1	5.20E-11	9.23E-12	+	0.261709	0.3013	+	C	G	0.271093866	0.0395918	+	C	G	0.2309	0.0442
rs4131097	LC_IGP12	IgG1_G1	14	66259165	1	1.36E-11	2.55E-12	+	0.267644	0.3116	+	C	G	0.271256261	0.0392998	+	C	G	0.2309	0.0445
rs4131097	LC_IGP_R2	IgG1_G1F/IgG1_G1	14	66259165	1	1.99E-11	3.48E-12	-	-0.265606	-0.3087	+	C	G	0.271256261	0.0393248	+	C	G	0.2309	0.0444
rs4131097	LC_IGP_R5	IgG1_G1FN/IgG1_G1N	14	66259165	1	7.03E-10	2.03E-15	-	-0.245616	-0.3535	+	C	G	0.270938807	0.0395988	+	C	G	0.2309	0.0445
rs4131097	LC_IGP53	IgG1_G1n	14	66259165	1	7.90E-11	4.89E-12	+	0.259275	0.3073	+	C	G	0.271093866	0.0396057	+	C	G	0.2309	0.0445
rs4131097	LC_IGP15	IgG1_G1N	14	66259165	1	3.74E-08	4.48E-11	+	0.219358	0.2911	+	C	G	0.270938807	0.0396726	+	C	G	0.2309	0.0442
rs4131097	LC_IGP56	IgG1_G1Nn	14	66259165	1	3.89E-08	1.12E-10	+	0.219879	0.2847	+	C	G	0.271093866	0.0398155	+	C	G	0.2309	0.0441
rs4131097	LC_IGP_R3	IgG1_G2F/IgG1_G2	14	66259165	1	1.88E-11	2.68E-14	-	-0.266072	-0.3358	+	C	G	0.271116443	0.0393449	+	C	G	0.2309	0.0441
rs4131097	LC_IGP148	IgG2 FG0n total/G0n	14	66259165	1	2.68E-08	9.36E-08	-	-0.220895	-0.236	+	C	G	0.271580357	0.0395265	+	C	G	0.231	0.0442
rs4131097	LC_IGP97	IgG2_G0	14	66259165	1	3.49E-10	4.14E-08	+	0.248266	0.2425	+	C	G	0.271012225	0.039319	+	C	G	0.2309	0.0442
rs4131097	LC_IGP_R41	IgG2_G0F/IgG2_G0	14	66259165	1	1.03E-08	3.45E-08	-	-0.226696	-0.2426	+	C	G	0.271012225	0.0393851	+	C	G	0.2309	0.044
rs4131097	LC_IGP138	IgG2_G0n	14	66259165	1	1.89E-10	3.69E-08	+	0.252414	0.2436	+	C	G	0.271580357	0.0393723	+	C	G	0.231	0.0442
rs4073416	LC_IGP84	IgG1 FBn/Bn total	14	66259394	1	1.26E-08	1.88E-07	-	-0.203754	-0.1982	+	C	T	0.439811648	0.0356142	+	C	T	0.415	0.038
rs4073416	LC_IGP21	IgG1 Fucosylation	14	66259394	1	6.77E-09	1.27E-06	-	-0.206115	-0.183	+	C	T	0.440828607	0.0353678	+	C	T	0.415	0.0378
rs4073416	LC_IGP11	IgG1_G0	14	66259394	1	1.30E-10	5.96E-07	+	0.226598	0.1879	+	C	T	0.439850336	0.0350254	+	C	T	0.415	0.0376
rs4073416	LC_IGP_R4	IgG1_G0FN/IgG1_G0N	14	66259394	1	1.10E-08	1.00E-06	-	-0.202643	-0.1858	+	C	T	0.439863748	0.0352757	+	C	T	0.415	0.038
rs4073416	LC_IGP52	IgG1_G0n	14	66259394	1	5.59E-11	5.54E-07	+	0.232795	0.1888	+	C	T	0.439811648	0.0352765	+	C	T	0.415	0.0377
rs4073416	LC_IGP_R3	IgG1_G2F/IgG1_G2	14	66259394	1	3.48E-08	2.01E-07	-	-0.195356	-0.1951	+	C	T	0.440119193	0.0352496	+	C	T	0.415	0.0375
rs4439682	LC_IGP78	IgG1 BG0n/G0n	14	66259428	1	7.33E-10	5.49E-10	+	0.216057	0.2354	+	G	A	0.47975062	0.0348695	+	G	A	0.4416	0.038
rs4439682	LC_IGP79	IgG1 BG1n/G1n	14	66259428	1	3.65E-09	2.77E-11	+	0.207529	0.2524	+	G	A	0.47975062	0.0349845	+	G	A	0.4416	0.0379
rs4439682	LC_IGP77	IgG1 Bn	14	66259428	1	1.45E-09	3.34E-11	+	0.212823	0.2525	+	G	A	0.47975062	0.034972	+	G	A	0.4416	0.0381
rs4439682	LC_IGP86	IgG1 Bn/Fn total	14	66259428	1	3.32E-10	2.44E-11	+	0.220868	0.2544	+	G	A	0.47975062	0.0349332	+	G	A	0.4416	0.0381
rs4439682	LC_IGP84	IgG1 FBn/Bn total	14	66259428	1	1.25E-09	1.04E-14	-	-0.214021	-0.2971	+	G	A	0.47975062	0.0350322	+	G	A	0.4416	0.0384
rs4439682	LC_IGP62	IgG1 FG0n total/G0n	14	66259428	1	2.75E-12	1.62E-11	-	-0.244512	-0.2574	+	G	A	0.47975062	0.03471	+	G	A	0.4416	0.0382
rs4439682	LC_IGP63	IgG1 FG1n total/G1n	14	66259428	1	7.66E-12	2.72E-13	-	-0.240211	-0.279	+	G	A	0.47975062	0.034834	+	G	A	0.4416	0.0382
rs4439682	LC_IGP64	IgG1 FG2n total/G2n	14	66259428	1	4.43E-09	1.52E-14	-	-0.206523	-0.2918	+	G	A	0.47975062	0.0350049	+	G	A	0.4416	0.038
rs4439682	LC_IGP61	IgG1 Fn total	14	66259428	1	1.01E-11	3.42E-13	-	-0.238647	-0.278	+	G	A	0.47975062	0.0348155	+	G	A	0.4416	0.0382
rs4439682	LC_IGP81	IgG1 Fn/Bn	14	66259428	1	4.43E-10	1.13E-10	-	-0.219234	-0.2444	+	G	A	0.47975062	0.0349277	+	G	A	0.4416	0.0379
rs4439682	LC_IGP21	IgG1 Fucosylation	14	66259428	1	3.43E-11	2.80E-13	-	-0.232298	-0.2789	+	G	A	0.48026233	0.0348124	+	G	A	0.4416	0.0382
rs4439682	LC_IGP28	IgG1 G1S1/(G1+G1S1)	14	66259428	1	1.08E-08	1.01E-09	-	-0.199293	-0.224	+	G	A	0.480120183	0.0346714	+	G	A	0.4416	0.0367
rs4439682	LC_IGP11	IgG1_G0	14	66259428	1	5.45E-13	5.83E-13	+	0.250445	0.2738	+	G	A	0.479839035	0.0344375	+	G	A	0.4416	0.038
rs4439682	LC_IGP_R1	IgG1_G0F/IgG1_G0	14	66259428	1	2.98E-12	4.58E-11	-	-0.242298	-0.2508	+	G	A	0.479839035	0.0344572	+	G	A	0.4416	0.0381
rs4439682	LC_IGP_R4	IgG1_G0FN/IgG1_G0N	14	66259428	1	3.94E-09	7.46E-13	-	-0.205713	-0.2749	+	G	A	0.479644825	0.0347555	+	G	A	0.4416	0.0383
rs4439682	LC_IGP52	IgG1_G0n	14	66259428	1	6.83E-13	3.66E-13	+	0.250971	0.2768	+	G	A	0.47975062	0.0346555	+	G	A	0.4416	0.0381
rs4439682	LC_IGP14	IgG1_G0N	14	66259428	1	1.29E-11	3.13E-11	+	0.236133	0.2512	+	G	A	0.479644825	0.0346278	+	G	A	0.4416	0.0378
rs4439682	LC_IGP55	IgG1_G0Nn	14	66259428	1	9.34E-12	2.21E-11	+	0.239016	0.2534	+	G	A	0.47975062	0.0348076	+	G	A	0.4416	0.0379
rs4439682	LC_IGP12	IgG1_G1	14	66259428	1	1.72E-11	1.59E-12	+	0.23423	0.2697	+	G	A	0.479839035	0.0345701	+	G	A	0.4416	0.0382
rs4439682	LC_IGP_R2	IgG1_G1F/IgG1_G1	14	66259428	1	1.90E-12	1.54E-12	-	-0.245102	-0.2692	+	G	A	0.479839035	0.0345385	+	G	A	0.4416	0.0381
rs4439682	LC_IGP_R5	IgG1_G1FN/IgG1_G1N	14	66259428	1	2.18E-09	1.23E-15	-	-0.209981	-0.3059	+	G	A	0.479255843	0.0348932	+	G	A	0.4416	0.0382
rs4439682	LC_IGP53	IgG1_G1n	14	66259428	1	1.25E-10	1.62E-12	+	0.225779	0.2694	+	G	A	0.47975062	0.0348659	+	G	A	0.4416	0.0381
rs4439682	LC_IGP15	IgG1_G1N	14	66259428	1	1.93E-08	2.35E-10	+	0.197179	0.2403	+	G	A	0.479255843	0.0349206	+	G	A	0.4416	0.0379
rs4439682	LC_IGP56	IgG1_G1Nn	14	66259428	1	1.82E-08	3.25E-10	+	0.198127	0.2381	+	G	A	0.47975062	0.0350248	+	G	A	0.4416	0.0379
rs4439682	LC_IGP_R32	IgG1_G1S1/IgG1_G1	14	66259428	1	1.42E-08	1.34E-09	-	-0.197701	-0.2226	+	G	A	0.480120183	0.0346823	+	G	A	0.4416	0.0367

rs4439682	LC_IGP_R3	IgG1_G2F/IgG1_G2	14	66259428	1	1.76E-10	3.31E-14	-	-0.222604	-0.2874	+	G	A	0.480132335	0.0346651	+	G	A	0.4416	0.0379
rs4439682	LC_IGP148	IgG2 FG0n total/G0n	14	66259428	1	3.54E-08	3.83E-12	-	-0.193005	-0.2628	+	G	A	0.48090548	0.0348428	+	G	A	0.4415	0.0378
rs4439682	LC_IGP97	IgG2_G0	14	66259428	1	2.04E-09	5.32E-12	+	0.208755	0.2614	+	G	A	0.480131724	0.0346269	+	G	A	0.4416	0.0379
rs4439682	LC_IGP_R41	IgG2_G0F/IgG2_G0	14	66259428	1	3.76E-08	8.92E-12	-	-0.191686	-0.2568	+	G	A	0.480131724	0.0346753	+	G	A	0.4416	0.0376
rs4439682	LC_IGP138	IgG2_G0n	14	66259428	1	9.02E-10	2.12E-12	+	0.214046	0.2667	+	G	A	0.48090548	0.0347337	+	G	A	0.4415	0.038
rs4131095	LC_IGP63	IgG1 FG1n total/G1n	14	66259437	1	3.39E-09	8.68E-07	-	-0.21259	-0.1889	+	A	G	0.420284077	0.0357613	+	A	G	0.3888	0.0384
rs4131095	LC_IGP61	IgG1 Fn total	14	66259437	1	1.57E-09	5.63E-07	-	-0.216913	-0.1923	+	A	G	0.420284077	0.0357195	+	A	G	0.3888	0.0384
rs4131095	LC_IGP21	IgG1 Fucosylation	14	66259437	1	9.58E-09	3.43E-07	-	-0.205658	-0.1958	+	A	G	0.420743527	0.0356541	+	A	G	0.3888	0.0384
rs4131095	LC_IGP11	IgG1_G0	14	66259437	1	7.76E-11	3.54E-07	+	0.23112	0.1946	+	A	G	0.419949297	0.0352932	+	A	G	0.3888	0.0382
rs4131095	LC_IGP52	IgG1_G0n	14	66259437	1	4.50E-11	3.06E-07	+	0.235689	0.196	+	A	G	0.420284077	0.0355373	+	A	G	0.3888	0.0383
rs4131095	LC_IGP12	IgG1_G1	14	66259437	1	3.50E-09	1.10E-06	+	0.210534	0.1871	+	A	G	0.420254734	0.0354516	+	A	G	0.3888	0.0384
rs4131095	LC_IGP_R2	IgG1_G1F/IgG1_G1	14	66259437	1	9.64E-10	1.61E-06	-	-0.217997	-0.1837	+	A	G	0.420254734	0.0354388	+	A	G	0.3888	0.0383
rs4131095	LC_IGP53	IgG1_G1n	14	66259437	1	2.36E-08	1.17E-06	+	0.200734	0.1865	+	A	G	0.420284077	0.0357753	+	A	G	0.3888	0.0384
rs4131095	LC_IGP_R3	IgG1_G2F/IgG1_G2	14	66259437	1	2.62E-08	2.86E-08	-	-0.198647	-0.2116	+	A	G	0.42020599	0.0355227	+	A	G	0.3888	0.0381
rs4073415	LC_IGP78	IgG1 BG0n/G0n	14	66259530	1	5.32E-11	1.55E-09	+	0.229557	0.2269	+	A	G	0.500829615	0.0347449	+	A	G	0.4686	0.0376
rs4073415	LC_IGP79	IgG1 BG1n/G1n	14	66259530	1	9.88E-10	1.24E-10	+	0.214482	0.2415	+	A	G	0.500829615	0.0348874	+	A	G	0.4686	0.0375
rs4073415	LC_IGP77	IgG1 Bn	14	66259530	1	3.02E-10	1.25E-10	+	0.220992	0.2425	+	A	G	0.500829615	0.0348694	+	A	G	0.4686	0.0377
rs4073415	LC_IGP86	IgG1 Bn/Fn total	14	66259530	1	7.71E-11	8.93E-11	+	0.228162	0.2446	+	A	G	0.500829615	0.034833	+	A	G	0.4686	0.0377
rs4073415	LC_IGP84	IgG1 FBn/Bn total	14	66259530	1	2.65E-10	1.66E-14	-	-0.222093	-0.2918	+	A	G	0.500829615	0.0349298	+	A	G	0.4686	0.038
rs4073415	LC_IGP62	IgG1 FG0n total/G0n	14	66259530	1	2.04E-12	5.55E-11	-	-0.24545	-0.2479	+	A	G	0.500829615	0.0346348	+	A	G	0.4686	0.0378
rs4073415	LC_IGP63	IgG1 FG1n total/G1n	14	66259530	1	3.81E-12	1.75E-12	-	-0.243165	-0.2665	+	A	G	0.500829615	0.03475	+	A	G	0.4686	0.0378
rs4073415	LC_IGP64	IgG1 FG2n total/G2n	14	66259530	1	6.44E-09	1.18E-13	-	-0.203942	-0.2788	+	A	G	0.500829615	0.0349433	+	A	G	0.4686	0.0376
rs4073415	LC_IGP61	IgG1 Fn total	14	66259530	1	8.56E-12	1.96E-12	-	-0.239016	-0.2661	+	A	G	0.500829615	0.0347428	+	A	G	0.4686	0.0378
rs4073415	LC_IGP81	IgG1 Fn/Bn	14	66259530	1	1.10E-10	6.63E-10	-	-0.226226	-0.2318	+	A	G	0.500829615	0.0348289	+	A	G	0.4686	0.0375
rs4073415	LC_IGP21	IgG1 Fucosylation	14	66259530	1	2.77E-11	1.33E-12	-	-0.232913	-0.2679	+	A	G	0.501889025	0.0347371	+	A	G	0.4686	0.0378
rs4073415	LC_IGP11	IgG1_G0	14	66259530	1	1.51E-12	9.27E-13	+	0.245136	0.2688	+	A	G	0.501267562	0.0343863	+	A	G	0.4686	0.0376
rs4073415	LC_IGP_R1	IgG1_G0F/IgG1_G0	14	66259530	1	6.53E-12	1.85E-10	-	-0.238003	-0.2403	+	A	G	0.501267562	0.0344011	+	A	G	0.4686	0.0377
rs4073415	LC_IGP_R4	IgG1_G0FN/IgG1_G0N	14	66259530	1	2.54E-10	1.81E-12	-	-0.220364	-0.2675	+	A	G	0.501127372	0.0346244	+	A	G	0.4686	0.038
rs4073415	LC_IGP52	IgG1_G0n	14	66259530	1	1.36E-12	6.48E-13	+	0.247201	0.2711	+	A	G	0.500829615	0.0346013	+	A	G	0.4686	0.0377
rs4073415	LC_IGP14	IgG1_G0N	14	66259530	1	4.61E-13	4.31E-11	+	0.251597	0.247	+	A	G	0.501127372	0.0344848	+	A	G	0.4686	0.0375
rs4073415	LC_IGP55	IgG1_G0Nn	14	66259530	1	6.85E-13	3.48E-11	+	0.251157	0.2485	+	A	G	0.500829615	0.0346833	+	A	G	0.4686	0.0375
rs4073415	LC_IGP12	IgG1_G1	14	66259530	1	3.34E-11	9.66E-12	+	0.230422	0.2574	+	A	G	0.501267562	0.0345112	+	A	G	0.4686	0.0378
rs4073415	LC_IGP_R2	IgG1_G1F/IgG1_G1	14	66259530	1	2.14E-12	1.52E-11	-	-0.244029	-0.2543	+	A	G	0.501267562	0.0344685	+	A	G	0.4686	0.0377
rs4073415	LC_IGP_R5	IgG1_G1FN/IgG1_G1N	14	66259530	1	7.92E-10	2.10E-15	-	-0.215154	-0.3002	+	A	G	0.500215867	0.0347951	+	A	G	0.4686	0.0378
rs4073415	LC_IGP53	IgG1_G1n	14	66259530	1	1.51E-10	1.11E-11	+	0.224324	0.2564	+	A	G	0.500829615	0.0348007	+	A	G	0.4686	0.0378
rs4073415	LC_IGP15	IgG1_G1N	14	66259530	1	8.99E-09	9.44E-10	+	0.20127	0.2298	+	A	G	0.500215867	0.0348277	+	A	G	0.4686	0.0376
rs4073415	LC_IGP56	IgG1_G1Nn	14	66259530	1	6.59E-09	1.52E-09	+	0.203745	0.2266	+	A	G	0.500829615	0.0349338	+	A	G	0.4686	0.0375
rs4073415	LC_IGP_R3	IgG1_G2F/IgG1_G2	14	66259530	1	3.12E-10	3.38E-13	-	-0.219129	-0.273	+	A	G	0.50157396	0.0346058	+	A	G	0.4686	0.0375
rs4073415	LC_IGP97	IgG2_G0	14	66259530	1	1.90E-08	3.17E-11	+	0.19546	0.2489	+	A	G	0.501573349	0.0346019	+	A	G	0.4686	0.0375
rs4073415	LC_IGP138	IgG2_G0n	14	66259530	1	7.46E-09	1.57E-11	+	0.201709	0.2531	+	A	G	0.502501846	0.0347111	+	A	G	0.4686	0.0376
rs3892139	LC_IGP61	IgG1 Fn total	14	66259865	1	4.28E-09	1.78E-06	-	-0.21204	-0.1888	+	A	G	0.417796829	0.0359056	+	A	G	0.3768	0.0395
rs3892139	LC_IGP21	IgG1 Fucosylation	14	66259865	1	2.32E-08	8.22E-07	-	-0.201187	-0.1946	+	A	G	0.418260531	0.0358365	+	A	G	0.3768	0.0395
rs3892139	LC_IGP11	IgG1_G0	14	66259865	1	2.27E-10	6.87E-07	+	0.226405	0.1946	+	A	G	0.417489055	0.0354766	+	A	G	0.3768	0.0392
rs3892139	LC_IGP52	IgG1_G0n	14	66259865	1	1.33E-10	5.70E-07	+	0.230995	0.1964	+	A	G	0.417796829	0.0357242	+	A	G	0.3768	0.0393
rs11850120	LC_IGP63	IgG1 FG1n total/G1n	14	66260379	1	5.25E-09	1.08E-06	-	-0.209834	-0.1878	+	C	T	0.423612811	0.0357401	+	C	T	0.3896	0.0385
rs11850120	LC_IGP61	IgG1 Fn total	14	66260379	1	1.98E-09	7.28E-07	-	-0.215388	-0.1909	+	C	T	0.423612811	0.035694	+	C	T	0.3896	0.0385
rs11850120	LC_IGP21	IgG1 Fucosylation	14	66260379	1	1.21E-08	4.56E-07	-	-0.204067	-0.1943	+	C	T	0.424075263	0.0356246	+	C	T	0.3896	0.0385
rs11850120	LC_IGP11	IgG1_G0	14	66260379	1	1.36E-10	4.53E-07	+	0.22791	0.1935	+	C	T	0.4232502	0.0352705	+	C	T	0.3896	0.0384
rs11850120	LC_IGP52	IgG1_G0n	14	66260379	1	7.51E-11	3.92E-07	+	0.23279	0.1949	+	C	T	0.423612811	0.0355179	+	C	T	0.3896	0.0384
rs11850120	LC_IGP12	IgG1_G1	14	66260379	1	5.32E-09	1.41E-06	+	0.207893	0.1857	+	C	T	0.423555636	0.035426	+	C	T	0.3896	0.0385
rs11850120	LC_IGP_R2	IgG1_G1F/IgG1_G1	14	66260379	1	1.45E-09	2.03E-06	-	-0.215495	-0.1825	+	C	T	0.423555636	0.035413	+	C	T	0.3896	0.0384
rs11850120	LC_IGP53	IgG1_G1n	14	66260379	1	3.42E-08	1.49E-06	+	0.198269	0.1851	+	C	T	0.423612811	0.0357526	+	C	T	0.3896	0.0385
rs11850120	LC_IGP_R3	IgG1_G2F/IgG1_G2	14	66260379	1	4.17E-08	3.64E-08	-	-0.195589	-0.2105	+	C	T	0.42350891	0.0354979	+	C	T	0.3896	0.0382
rs11845834	LC_IGP63	IgG1 FG1n total/G1n	14	66260602	1	5.35E-09	1.18E-06	-	-0.209723	-0.1872	+	A	C	0.423631351	0.035741	+	A	C	0.39	0.0385
rs11845834	LC_IGP61	IgG1 Fn total	14	66260602	1	2.03E-09	7.92E-07	-	-0.215243	-0.1904	+	A	C	0.423631351	0.035695	+	A	C	0.39	0.0386
rs11845834	LC_IGP21	IgG1 Fucosylation	14	66260602	1	1.22E-08	4.91E-07	-	-0.20401	-0.1938	+	A	C	0.424089396	0.0356257	+	A	C	0.39	0.0385
rs11845834	LC_IGP11	IgG1_G0	14	66260602	1	1.41E-10	4.68E-07	+	0.227713	0.1934	+	A	C	0.423264203	0.0352721	+	A	C	0.39	0.0384
rs11845834	LC_IGP52	IgG1_G0n	14	66260602	1	7.80E-11	4.08E-07	+	0.232594	0.1948	+	A	C	0.423631351	0.0355192	+	A	C	0.39	0.0384
rs11845834	LC_IGP12	IgG1_G1	14	66260602	1	5.46E-09	1.57E-06	+	0.207744	0.1849	+	A	C	0.42356964	0.0354273	+	A	C	0.39	0.0385
rs11845834	LC_IGP_R2	IgG1_G1F/IgG1_G1	14	66260602	1	1.47E-09	2.21E-06	-	-0.215389	-0.1819	+	A	C	0.42356964	0.0354142	+	A	C	0.39	0.0384
rs11845834	LC_IGP53	IgG1_G1n	14	66260602	1	3.50E-08	1.66E-06	+	0.198124	0.1843	+	A	C	0.423631351	0.0357536	+	A	C	0.39	0.0385
rs11845834	LC_IGP_R3	IgG1_G2F/IgG1_G2	14	66260602	1	4.24E-08	3.89E-08	-	-0.195488	-0.2101	+	A	C	0.423522922	0.0354991	+	A	C	0.39	0.0382
rs8018379	LC_IGP78	IgG1 BG0n/G0n	14	66261937	1	6.53E-10	5.80E-09	+	0.219114	0.2228	+	G	C	0.444732032	0.0352566	+	G	C	0.4055	0.0383
rs8018379	LC_IGP79	IgG1 BG1n/G1n	14	66261937	1	2.14E-08	3.26E-11	+	0.199336	0.2537	+	G	C	0.444732032	0.035413	+	G	C	0.4055	0.0382
rs8018379	LC_IGP77	IgG1 Bn	14	66261937	1	6.29E-09	1.10E-10	+	0.206711	0.2478	+	G	C	0.444732032	0.035394	+	G	C	0.4055	0.0384
rs8018379	LC_IGP86	IgG1 Bn/Fn total	14	66261937	1	1.46E-09	8.24E-11	+	0.215104	0.2498	+	G	C	0.444732032	0.0353552	+	G	C	0.4055	0.0384
rs8018379	LC_IGP84	IgG1 FBn/Bn total	14	66261937	1	5.58E-10	1.72E-14	-	-0.220931	-0.297	+	G	C	0.444732032	0.0354063	+	G	C	0.4055	0.0387
rs8018379	LC_IGP62	IgG1 FG0n total/G0n	14	66261937	1	1.69E-11	3.57E-10	-	-0.2382	-0.2418	+	G	C	0.444732032	0.0351365	+	G	C	0.4055	0.0386
rs8018379	LC_IGP63	IgG1 FG1n total/G1n	14	66261937	1	1.40E-11	1.43E-12	-	-0.239859	-0.2727	+	G	C	0.444732032	0.0352361	+	G	C	0.4055	0.0385
rs8018379	LC_IGP64	IgG1 FG2n total/G2n	14	66261937	1	1.91E-09	8.41E-14	-	-0.213681	-0.2859	+	G	C	0.444732032	0.035378	+	G	C	0.4055	0.0383
rs8018379	LC_IGP61	IgG1 Fn total	14	66261937	1	2.01E-11	3.49E-12	-	-0.237841	-0.2681	+	G	C	0.444732032	0.0352191	+	G	C	0.4055	0.0385
rs8018379	LC_IGP81	IgG1 Fn/Bn	14	66261937	1	3.30E-09	4.96E-10	-	-0.21037	-0.2378	+	G	C	0.444732032	0.0353608	+	G	C	0.4055	0.0382
rs8018379	LC_IGP21	IgG1 Fucosylation	14	66261937	1	7.86E-11	3.13E-12	-	-0.230813	-0.2685	+	G	C	0.44540783	0.0352546	+	G	C	0.4055	0.0385
rs8018379	LC_IGP11	IgG1_G0	14	66261937	1	2.33E-11	4.49E-12	+	0.235178	0.266	+	G	C	0.444686316	0.0349424	+	G	C	0.4055	0.0384
rs8018379	LC_IGP_R1	IgG1_G0F/IgG1_G0	14	66261937	1	7.69E-11	1.26E-09	-	-0.228932	-0.2334	+	G	C	0.444686316	0.0349517	+	G	C	0.4055	0.0384
rs8018379	LC_IGP_R4	IgG1_G0FN/IgG1_G0N	14	66261937	1	1.02E-09	9.98E-12	-	-0.216065	-0.263	+	G	C	0.444763013	0.0351757	+	G	C	0.4055	0.0386
rs8018379	LC_IGP52	IgG1_G0n	14	66261937	1	1.47E-11	3.47E-12	+	0.238723	0.2679	+	G	C	0.444732032	0.0351082	+	G	C	0.4055	0.0385
rs8018379	LC_IGP14	IgG1_G0N	14	66261937	1	3.32E-11	1.10E-10	+	0.234355	0.2466	+	G	C	0.444763013	0.0350948	+	G	C	0.4055	0.0382
rs8018379	LC_IGP55	IgG1_G0Nn	14	66261937	1	4.06E-11	9.84E-11	+	0.234189	0.2476	+	G	C	0.444732032	0.0352279	+	G	C	0.4055	0.0383
rs8018379	LC_IGP12	IgG1_G1	14	66261937	1	8.53E-11	1.72E-11	+	0.228897	0.2591	+	G	C	0.444686316	0.0350315	+	G	C	0.4055	0.0385
rs8018379	LC_IGP_R2	IgG1_G1F/IgG1_G1	14	66261937	1	1.68E-11	1.40E-11	-	-0.237355	-0.2597	+	G	C	0.444686316	0.0350118	+	G	C	0.4055	0.0384
rs8018379	LC_IGP_R5	IgG1_G1FN/IgG1_G1N	14	66261937	1	2.18E-09	4.58E-16	-	-0.212447	-0.3131	+	G	C	0.444217712	0.0353048	+	G	C	0.4055	0.0385
rs8018379	LC_IGP53	IgG1_G1n	14	66261937	1	1.40E-10	2.24E-11	+	0.22769	0.2574	+	G	C	0.444732032	0.0352578	+	G	C	0.4055	0.0385
rs8018379	LC_IGP_R3	IgG1_G2F/IgG1_G2	14	66261937	1	1.93E-10	2.02E-13	-	-0.224854	-0.2808	+	G	C	0.44495813	0.0350937	+	G	C	0.4055	0.0382
rs8007846	LC_IGP62	IgG1 FG0n total/G0n	14	66262963	1	3.33E-09	1.15E-06	-	-0.206678	-0.1826	+	G	A	0.478314746	0.0347503	+	G	A	0.4444	0.0375
rs8007846	LC_IGP63	IgG1 FG1n total/G1n	14	66262963	1	3.91E-10	1.50E-08	-	-0.219181	-0.2123	+	G	A	0.478314746	0.0348075	+	G	A	0.4444	0.0375
rs8007846	LC_IGP64	IgG1 FG2n total/G2n	14	66262963	1	2.27E-08	1.11E-09	-	-0.196231	-0.2273	+	G	A	0.478314746	0.0349294	+	G	A	0.4444	0.0373
rs8007846	LC_IGP61	IgG1 Fn total	14	66262963	1	1.14E-09	3.88E-08	-	-0.213187	-0.2063	+	G	A	0.478314746	0.0348056	+	G	A	0.4444	0.0375
rs8007846	LC_IGP21	IgG1 Fucosylation	14	66262963	1	2.44E-09	1.80E-08	-	-0.209111	-0.211	+	G	A	0.479038224	0.0348551	+	G	A	0.4444	0.0375
rs8007846	LC_IGP11	IgG1_G0	14	66262963	1	6.50E-10	9.71E-09	+	0.214674	0.2142	+	G	A	0.478008552	0.034541	+	G	A	0.4444	0.0373
rs8007846	LC_IGP52	IgG1_G0n	14	66262963	1	4.10E-10	1.03E-08	+	0.218118	0.2142	+	G	A	0.478314746	0.0346812	+	G	A	0.4444	0.0374
rs8007846	LC_IGP14	IgG1_G0N	14	66262963	1	2.63E-08	5.84E-08	+	0.194261	0.2017	+	G	A	0.477954685	0.0347385	+	G	A	0.4444	0.0372
rs8007846	LC_IGP55	IgG1_G0Nn	14	66262963	1	2.43E-08	6.67E-08	+	0.195449	0.201	+	G	A	0.478314746	0.0348639	+	G	A	0.4444	0.0372
rs8007846	LC_IGP12	IgG1_G1	14	66262963	1	1.22E-09	1.22E-07	+	0.211592	0.1984	+	G	A	0.478008552	0.0346152	+	G	A	0.4444	0.0375
rs8007846	LC_IGP_R2	IgG1_G1F/IgG1_G1	14	66262963	1	1.56E-10	8.03E-08	-	-0.222754	-0.2008	+	G	A	0.478008552	0.0345869	+	G	A	0.4444	0.0374
rs8007846	LC_IGP53	IgG1_G1n	14	66262963	1	1.73E-09	1.87E-07	+	0.210828	0.1953	+	G	A	0.478314746	0.0348117	+	G	A	0.4444	0.0375
rs8007846	LC_IGP_R3	IgG1_G2F/IgG1_G2	14	66262963	1	2.20E-09	2.39E-09	-	-0.208611	-0.2222	+	G	A	0.478300733	0.034674	+	G	A	0.4444	0.0372
rs4899183	LC_IGP14	IgG1_G0N	14	66275755	2	1.17E-08	1.96E-06	+	0.210149	0.191	+	G	A	0.656857414	0.0366516	+	G	A	0.6341	0.0402
rs73396531	LC_IGP22	IgG1 Bisecting_GlcNAc	22	24100654	1	4.90E-10	1.66E-07	-	-0.320888	-0.2768	+	G	A	0.172028555	0.0512506	+	G	A	0.1618	0.0529
rs73396531	LC_IGP73	IgG1 Bn total	22	24100654	1	6.43E-09	2.02E-07	-	-0.297476	-0.2757	+	G	A	0.171917028	0.0509676	+	G	A	0.1616	0.053
rs73396531	LC_IGP70	IgG1 FBG0n/G0n	22	24100654	1	1.02E-08	1.52E-06	-	-0.294102	-0.2581	+	G	A	0.171917028	0.0510854	+	G	A	0.1616	0.0537
rs73396531	LC_IGP71	IgG1 FBG1n/G1n	22	24100654	1	2.39E-08	1.67E-06	-	-0.286301	-0.2534	+	G	A	0.171917028	0.0510416	+	G	A	0.1616	0.0529
rs73396531	LC_IGP69	IgG1 FBn	22	24100654	1	1.40E-09	5.08E-07	-	-0.310485	-0.2679	+	G	A	0.171917028	0.0509763	+	G	A	0.1616	0.0533
rs73396531	LC_IGP82	IgG1 FBn/Fn	22	24100654	1	1.27E-08	2.51E-07	-	-0.291863	-0.2729	+	G	A	0.171917028	0.0510291	+	G	A	0.1616	0.0529
rs73396531	LC_IGP83	IgG1 FBn/Fn total	22	24100654	1	1.22E-08	3.17E-07	-	-0.292144	-0.2706	+	G	A	0.171917028	0.0510204	+	G	A	0.1616	0.0529
rs73396531	LC_IGP5	IgG1_G1FN	22	24100654	1	1.50E-09	1.11E-06	-	-0.309127	-0.258	+	G	A	0.172081467	0.0508479	+	G	A	0.1616	0.053
rs73396531	LC_IGP162	IgG2 BG2n total/G2n	22	24100654	1	3.94E-08	1.18E-09	-	-0.283588	-0.327	+	G	A	0.171718656	0.0513763	+	G	A	0.1619	0.0538
rs73396531	LC_IGP108	IgG2 Bisecting_GlcNAc	22	24100654	1	5.48E-09	1.31E-11	-	-0.300578	-0.3644	+	G	A	0.171894926	0.0512596	+	G	A	0.1608	0.0538
rs73396531	LC_IGP159	IgG2 Bn total	22	24100654	1	6.67E-09	2.15E-11	-	-0.297853	-0.3603	+	G	A	0.171718656	0.0510896	+	G	A	0.1619	0.0538
rs73396531	LC_IGP156	IgG2 FBG0n/G0n	22	24100654	1	1.15E-08	9.23E-11	-	-0.293157	-0.3488	+	G	A	0.171718656	0.0510968	+	G	A	0.1619	0.0538
rs73396531	LC_IGP155	IgG2 FBn	22	24100654	1	9.87E-10	1.95E-11	-	-0.313759	-0.3618	+	G	A	0.171718656	0.0510363	+	G	A	0.1619	0.0539
rs73396531	LC_IGP168	IgG2 FBn/Fn	22	24100654	1	8.85E-09	2.27E-11	-	-0.295363	-0.3599	+	G	A	0.171718656	0.0510851	+	G	A	0.1619	0.0538
rs73396531	LC_IGP169	IgG2 FBn/Fn total	22	24100654	1	8.19E-09	2.21E-11	-	-0.296049	-0.3601	+	G	A	0.171718656	0.051087	+	G	A	0.1619	0.0538
rs73396531	LC_IGP135	IgG2_G0FNn	22	24100654	1	3.72E-09	1.19E-09	-	-0.302579	-0.3159	+	G	A	0.171718656	0.0510337	+	G	A	0.1619	0.0519
rs7292927	LC_IGP74	IgG1 BG0n total/G0n	22	24101892	1	2.85E-08	4.33E-07	-	-0.282659	-0.2687	+	T	C	0.171575897	0.050672	+	T	C	0.164	0.0532
rs7292927	LC_IGP75	IgG1 BG1n total/G1n	22	24101892	1	3.46E-08	6.03E-07	-	-0.280769	-0.2624	+	T	C	0.171575897	0.0506471	+	T	C	0.164	0.0526
rs7292927	LC_IGP22	IgG1 Bisecting_GlcNAc	22	24101892	1	2.04E-10	1.04E-07	-	-0.325341	-0.2808	+	T	C	0.171725506	0.0508338	+	T	C	0.1642	0.0528
rs7292927	LC_IGP73	IgG1 Bn total	22	24101892	1	2.53E-09	1.25E-07	-	-0.303041	-0.2798	+	T	C	0.171575897	0.0505601	+	T	C	0.164	0.0529
rs7292927	LC_IGP70	IgG1 FBG0n/G0n	22	24101892	1	4.43E-09	8.60E-07	-	-0.299004	-0.2641	+	T	C	0.171575897	0.05068	+	T	C	0.164	0.0537

rs7292927	LC_IGP71	IgG1 FBG1n/G1n	22	24101892	1	9.79E-09	1.08E-06	-	-0.291886	-0.2574	+	T	C	0.171575897	0.0506349	+	T	C	0.164	0.0528
rs7292927	LC_IGP69	IgG1 FBn	22	24101892	1	5.38E-10	3.13E-07	-	-0.315834	-0.2723	+	T	C	0.171575897	0.050568	+	T	C	0.164	0.0532
rs7292927	LC_IGP82	IgG1 FBn/Fn	22	24101892	1	5.04E-09	1.59E-07	-	-0.297551	-0.2769	+	T	C	0.171575897	0.0506214	+	T	C	0.164	0.0528
rs7292927	LC_IGP83	IgG1 FBn/Fn total	22	24101892	1	4.91E-09	2.00E-07	-	-0.297717	-0.2746	+	T	C	0.171575897	0.0506131	+	T	C	0.164	0.0528
rs7292927	LC_IGP_R11	IgG1_G0FN/IgG1_G0F	22	24101892	1	2.87E-08	7.86E-07	-	-0.281024	-0.262	+	T	C	0.171933609	0.0503976	+	T	C	0.164	0.053
rs7292927	LC_IGP5	IgG1_G1FN	22	24101892	1	6.29E-10	5.66E-07	-	-0.313805	-0.2644	+	T	C	0.171746335	0.050448	+	T	C	0.164	0.0529
rs7292927	LC_IGP_R12	IgG1_G1FN/IgG1_G1F	22	24101892	1	4.17E-08	1.06E-06	-	-0.277498	-0.2559	+	T	C	0.171746335	0.0503664	+	T	C	0.164	0.0524
rs7292927	LC_IGP50	IgG1_G1FNn	22	24101892	1	3.67E-09	1.29E-06	-	-0.301152	-0.2559	+	T	C	0.171575897	0.050775	+	T	C	0.164	0.0528
rs7292927	LC_IGP162	IgG2 BG2n total/G2n	22	24101892	1	1.92E-08	8.32E-10	-	-0.287854	-0.33	+	T	C	0.171377364	0.0509745	+	T	C	0.1643	0.0538
rs7292927	LC_IGP108	IgG2 Bisecting_GlcNAc	22	24101892	1	2.74E-09	6.67E-12	-	-0.304079	-0.3697	+	T	C	0.171587094	0.0508495	+	T	C	0.1632	0.0538
rs7292927	LC_IGP159	IgG2 Bn total	22	24101892	1	3.02E-09	1.13E-11	-	-0.302299	-0.3655	+	T	C	0.171377364	0.0506878	+	T	C	0.1643	0.0538
rs7292927	LC_IGP156	IgG2 FBG0n/G0n	22	24101892	1	5.49E-09	4.34E-11	-	-0.297238	-0.3552	+	T	C	0.171377364	0.0506968	+	T	C	0.1643	0.0539
rs7292927	LC_IGP157	IgG2 FBG1n/G1n	22	24101892	1	2.90E-08	1.32E-09	-	-0.283439	-0.33	+	T	C	0.171377364	0.0508437	+	T	C	0.1643	0.0544
rs7292927	LC_IGP155	IgG2 FBn	22	24101892	1	3.67E-10	1.02E-11	-	-0.319288	-0.367	+	T	C	0.171377364	0.0506289	+	T	C	0.1643	0.0539
rs7292927	LC_IGP168	IgG2 FBn/Fn	22	24101892	1	3.67E-09	1.23E-11	-	-0.300589	-0.3648	+	T	C	0.171377364	0.0506806	+	T	C	0.1643	0.0538
rs7292927	LC_IGP169	IgG2 FBn/Fn total	22	24101892	1	3.40E-09	1.20E-11	-	-0.301256	-0.365	+	T	C	0.171377364	0.0506826	+	T	C	0.1643	0.0538
rs7292927	LC_IGP171	IgG2 Fn/Bn total	22	24101892	1	3.68E-08	2.20E-11	+	0.280883	0.3594	+	T	C	0.171377364	0.0507713	+	T	C	0.1643	0.0537
rs7292927	LC_IGP135	IgG2_G0FNn	22	24101892	1	1.75E-09	4.55E-10	-	-0.306528	-0.3233	+	T	C	0.171377364	0.0506335	+	T	C	0.1643	0.0519
rs7292927	LC_IGP_R52	IgG2_G1FN/IgG2_G1F	22	24101892	1	3.62E-08	1.60E-09	-	-0.280062	-0.3274	+	T	C	0.17185125	0.0505975	+	T	C	0.164	0.0543
rs7292927	LC_IGP_R53	IgG2_G2FN/IgG2_G2F	22	24101892	1	2.86E-08	1.24E-09	-	-0.282707	-0.3282	+	T	C	0.171933609	0.0506918	+	T	C	0.164	0.054
rs113554134	LC_IGP74	IgG1 BG0n total/G0n	22	24103584	1	3.89E-08	3.94E-07	-	-0.277438	-0.2648	+	A	G	0.183962105	0.0502362	+	A	G	0.1738	0.0522
rs113554134	LC_IGP22	IgG1 Bisecting_GlcNAc	22	24103584	1	4.38E-10	2.42E-07	-	-0.316515	-0.2682	+	A	G	0.184222053	0.0504068	+	A	G	0.174	0.0519
rs113554134	LC_IGP73	IgG1 Bn total	22	24103584	1	4.30E-09	2.27E-07	-	-0.296011	-0.2695	+	A	G	0.183962105	0.050132	+	A	G	0.1738	0.0521
rs113554134	LC_IGP70	IgG1 FBG0n/G0n	22	24103584	1	4.70E-09	5.61E-07	-	-0.29587	-0.2632	+	A	G	0.183962105	0.0502366	+	A	G	0.1738	0.0526
rs113554134	LC_IGP71	IgG1 FBG1n/G1n	22	24103584	1	1.50E-08	1.32E-06	-	-0.285692	-0.2507	+	A	G	0.183962105	0.050203	+	A	G	0.1738	0.0518
rs113554134	LC_IGP69	IgG1 FBn	22	24103584	1	6.52E-10	3.81E-07	-	-0.311557	-0.2654	+	A	G	0.183962105	0.0501296	+	A	G	0.1738	0.0523
rs113554134	LC_IGP82	IgG1 FBn/Fn	22	24103584	1	8.11E-09	2.96E-07	-	-0.290956	-0.2663	+	A	G	0.183962105	0.0501911	+	A	G	0.1738	0.052
rs113554134	LC_IGP83	IgG1 FBn/Fn total	22	24103584	1	7.86E-09	3.71E-07	-	-0.29117	-0.2641	+	A	G	0.183962105	0.0501828	+	A	G	0.1738	0.052
rs113554134	LC_IGP_R11	IgG1_G0FN/IgG1_G0F	22	24103584	1	4.07E-08	6.85E-07	-	-0.275592	-0.2587	+	A	G	0.184282297	0.0499808	+	A	G	0.1738	0.0521
rs113554134	LC_IGP5	IgG1_G1FN	22	24103584	1	1.43E-09	8.02E-07	-	-0.304597	-0.2559	+	A	G	0.184097761	0.050044	+	A	G	0.1738	0.0519
rs113554134	LC_IGP50	IgG1_G1FNn	22	24103584	1	6.95E-09	1.48E-06	-	-0.293196	-0.2496	+	A	G	0.183962105	0.0503483	+	A	G	0.1738	0.0518
rs113554134	LC_IGP160	IgG2 BG0n total/G0n	22	24103584	1	2.31E-08	3.68E-11	-	-0.282515	-0.3497	+	A	G	0.183782064	0.0503167	+	A	G	0.1741	0.0529
rs113554134	LC_IGP161	IgG2 BG1n total/G1n	22	24103584	1	4.67E-08	1.05E-09	-	-0.276532	-0.3252	+	A	G	0.183782064	0.0503727	+	A	G	0.1741	0.0533
rs113554134	LC_IGP162	IgG2 BG2n total/G2n	22	24103584	1	1.06E-08	1.18E-09	-	-0.290467	-0.3214	+	A	G	0.183782064	0.0505121	+	A	G	0.1741	0.0528
rs113554134	LC_IGP108	IgG2 Bisecting_GlcNAc	22	24103584	1	1.02E-09	8.27E-12	-	-0.30941	-0.3613	+	A	G	0.184049349	0.0503722	+	A	G	0.1729	0.0529
rs113554134	LC_IGP159	IgG2 Bn total	22	24103584	1	1.16E-09	1.05E-11	-	-0.307388	-0.3595	+	A	G	0.183782064	0.050217	+	A	G	0.1741	0.0529
rs113554134	LC_IGP156	IgG2 FBG0n/G0n	22	24103584	1	2.02E-09	2.60E-11	-	-0.302867	-0.3527	+	A	G	0.183782064	0.0502246	+	A	G	0.1741	0.0529
rs113554134	LC_IGP157	IgG2 FBG1n/G1n	22	24103584	1	1.25E-08	1.34E-09	-	-0.288256	-0.3241	+	A	G	0.183782064	0.0503749	+	A	G	0.1741	0.0534
rs113554134	LC_IGP155	IgG2 FBn	22	24103584	1	1.27E-10	8.83E-12	-	-0.324658	-0.3616	+	A	G	0.183782064	0.0501553	+	A	G	0.1741	0.053
rs113554134	LC_IGP168	IgG2 FBn/Fn	22	24103584	1	1.34E-09	1.14E-11	-	-0.30619	-0.3589	+	A	G	0.183782064	0.0502082	+	A	G	0.1741	0.0529
rs113554134	LC_IGP169	IgG2 FBn/Fn total	22	24103584	1	1.24E-09	1.11E-11	-	-0.306842	-0.3591	+	A	G	0.183782064	0.0502101	+	A	G	0.1741	0.0529
rs113554134	LC_IGP171	IgG2 Fn/Bn total	22	24103584	1	1.45E-08	2.12E-11	+	0.286551	0.3533	+	A	G	0.183782064	0.0503005	+	A	G	0.1741	0.0528
rs113554134	LC_IGP_R51	IgG2_G0FN/IgG2_G0F	22	24103584	1	2.73E-08	3.51E-11	-	-0.279542	-0.3499	+	A	G	0.184584888	0.0500501	+	A	G	0.1738	0.0528
rs113554134	LC_IGP135	IgG2_G0FNn	22	24103584	1	3.98E-10	4.04E-10	-	-0.315619	-0.3182	+	A	G	0.183782064	0.0501472	+	A	G	0.1741	0.0509
rs113554134	LC_IGP_R52	IgG2_G1FN/IgG2_G1F	22	24103584	1	1.51E-08	1.63E-09	-	-0.285284	-0.3216	+	A	G	0.184210221	0.0501425	+	A	G	0.1738	0.0533
rs113554134	LC_IGP_R53	IgG2_G2FN/IgG2_G2F	22	24103584	1	1.91E-08	2.07E-09	-	-0.283794	-0.3182	+	A	G	0.184282297	0.0502502	+	A	G	0.1738	0.0531
rs61117377	LC_IGP74	IgG1 BG0n total/G0n	22	24107801	1	2.07E-08	3.00E-07	-	-0.280674	-0.267	+	C	T	0.184461246	0.0498118	+	C	T	0.1752	0.0521
rs61117377	LC_IGP75	IgG1 BG1n total/G1n	22	24107801	1	4.18E-08	1.01E-06	-	-0.274396	-0.2522	+	C	T	0.184461246	0.0498026	+	C	T	0.1752	0.0516
rs61117377	LC_IGP22	IgG1 Bisecting_GlcNAc	22	24107801	1	2.53E-10	1.84E-07	-	-0.318141	-0.2701	+	C	T	0.184779032	0.0499747	+	C	T	0.1754	0.0518
rs61117377	LC_IGP73	IgG1 Bn total	22	24107801	1	2.20E-09	1.84E-07	-	-0.299082	-0.2709	+	C	T	0.184461246	0.0497072	+	C	T	0.1752	0.052
rs61117377	LC_IGP70	IgG1 FBG0n/G0n	22	24107801	1	2.33E-09	4.88E-07	-	-0.299215	-0.264	+	C	T	0.184461246	0.0498099	+	C	T	0.1752	0.0525
rs61117377	LC_IGP71	IgG1 FBG1n/G1n	22	24107801	1	8.25E-09	1.38E-06	-	-0.288425	-0.2496	+	C	T	0.184461246	0.0497798	+	C	T	0.1752	0.0517
rs61117377	LC_IGP69	IgG1 FBn	22	24107801	1	3.11E-10	3.47E-07	-	-0.314761	-0.2657	+	C	T	0.184461246	0.0497026	+	C	T	0.1752	0.0521
rs61117377	LC_IGP82	IgG1 FBn/Fn	22	24107801	1	4.34E-09	2.51E-07	-	-0.293778	-0.2673	+	C	T	0.184461246	0.0497672	+	C	T	0.1752	0.0518
rs61117377	LC_IGP83	IgG1 FBn/Fn total	22	24107801	1	4.25E-09	3.11E-07	-	-0.293909	-0.2651	+	C	T	0.184461246	0.0497592	+	C	T	0.1752	0.0518
rs61117377	LC_IGP_R11	IgG1_G0FN/IgG1_G0F	22	24107801	1	2.38E-08	5.16E-07	-	-0.278016	-0.261	+	C	T	0.184753478	0.0495641	+	C	T	0.1752	0.052
rs61117377	LC_IGP5	IgG1_G1FN	22	24107801	1	1.03E-09	6.83E-07	-	-0.304743	-0.2571	+	C	T	0.184569421	0.0496323	+	C	T	0.1752	0.0518
rs61117377	LC_IGP50	IgG1_G1FNn	22	24107801	1	4.77E-09	1.36E-06	-	-0.293949	-0.2501	+	C	T	0.184461246	0.0499305	+	C	T	0.1752	0.0518
rs61117377	LC_IGP160	IgG2 BG0n total/G0n	22	24107801	1	1.23E-08	1.80E-11	-	-0.28567	-0.3551	+	C	T	0.18423874	0.0498942	+	C	T	0.1754	0.0528
rs61117377	LC_IGP161	IgG2 BG1n total/G1n	22	24107801	1	2.38E-08	6.75E-10	-	-0.280202	-0.3285	+	C	T	0.18423874	0.0499486	+	C	T	0.1754	0.0532
rs61117377	LC_IGP162	IgG2 BG2n total/G2n	22	24107801	1	4.38E-09	7.33E-10	-	-0.29554	-0.3252	+	C	T	0.18423874	0.0500803	+	C	T	0.1754	0.0528
rs61117377	LC_IGP108	IgG2 Bisecting_GlcNAc	22	24107801	1	5.09E-10	4.38E-12	-	-0.312353	-0.3661	+	C	T	0.184552781	0.0499392	+	C	T	0.1742	0.0529
rs61117377	LC_IGP159	IgG2 Bn total	22	24107801	1	5.19E-10	5.48E-12	-	-0.311254	-0.3642	+	C	T	0.18423874	0.0497901	+	C	T	0.1754	0.0528
rs61117377	LC_IGP156	IgG2 FBG0n/G0n	22	24107801	1	9.18E-10	1.26E-11	-	-0.306738	-0.358	+	C	T	0.18423874	0.0497981	+	C	T	0.1754	0.0528
rs61117377	LC_IGP157	IgG2 FBG1n/G1n	22	24107801	1	5.61E-09	8.58E-10	-	-0.292673	-0.3274	+	C	T	0.18423874	0.0499469	+	C	T	0.1754	0.0534
rs61117377	LC_IGP155	IgG2 FBn	22	24107801	1	4.45E-11	4.58E-12	-	-0.329838	-0.3663	+	C	T	0.18423874	0.0497216	+	C	T	0.1754	0.053
rs61117377	LC_IGP168	IgG2 FBn/Fn	22	24107801	1	5.37E-10	6.14E-12	-	-0.310914	-0.3634	+	C	T	0.18423874	0.0497782	+	C	T	0.1754	0.0528
rs61117377	LC_IGP169	IgG2 FBn/Fn total	22	24107801	1	4.95E-10	5.92E-12	-	-0.311568	-0.3637	+	C	T	0.18423874	0.04978	+	C	T	0.1754	0.0528
rs61117377	LC_IGP171	IgG2 Fn/Bn total	22	24107801	1	7.22E-09	1.08E-11	+	0.290112	0.3583	+	C	T	0.18423874	0.0498762	+	C	T	0.1754	0.0527
rs61117377	LC_IGP_R51	IgG2_G0FN/IgG2_G0F	22	24107801	1	1.32E-08	1.70E-11	-	-0.283465	-0.3553	+	C	T	0.185056261	0.0496277	+	C	T	0.1752	0.0528
rs61117377	LC_IGP135	IgG2_G0FNn	22	24107801	1	1.43E-10	2.24E-10	-	-0.320896	-0.3231	+	C	T	0.18423874	0.0497143	+	C	T	0.1754	0.0509
rs61117377	LC_IGP_R52	IgG2_G1FN/IgG2_G1F	22	24107801	1	7.24E-09	1.04E-09	-	-0.289154	-0.3249	+	C	T	0.18468217	0.049718	+	C	T	0.1752	0.0532
rs61117377	LC_IGP_R53	IgG2_G2FN/IgG2_G2F	22	24107801	1	6.00E-09	1.24E-09	-	-0.291302	-0.3222	+	C	T	0.184753478	0.0498128	+	C	T	0.1752	0.053
rs73396548	LC_IGP74	IgG1 BG0n total/G0n	22	24109285	1	5.19E-09	1.27E-07	-	-0.290843	-0.2796	+	A	G	0.170443885	0.0495219	+	A	G	0.1659	0.0529
rs73396548	LC_IGP75	IgG1 BG1n total/G1n	22	24109285	1	5.96E-09	3.86E-07	-	-0.289522	-0.2653	+	A	G	0.170443885	0.0494959	+	A	G	0.1659	0.0523
rs73396548	LC_IGP22	IgG1 Bisecting_GlcNAc	22	24109285	1	5.10E-11	3.14E-08	-	-0.328519	-0.2902	+	A	G	0.170805773	0.0496742	+	A	G	0.1661	0.0524
rs73396548	LC_IGP73	IgG1 Bn total	22	24109285	1	3.56E-10	3.75E-08	-	-0.311824	-0.2894	+	A	G	0.170443885	0.0494047	+	A	G	0.1659	0.0526
rs73396548	LC_IGP70	IgG1 FBG0n/G0n	22	24109285	1	8.95E-10	1.62E-07	-	-0.305315	-0.2797	+	A	G	0.170443885	0.0495327	+	A	G	0.1659	0.0534
rs73396548	LC_IGP71	IgG1 FBG1n/G1n	22	24109285	1	2.44E-09	4.51E-07	-	-0.29694	-0.2644	+	A	G	0.170443885	0.049495	+	A	G	0.1659	0.0524
rs73396548	LC_IGP69	IgG1 FBn	22	24109285	1	1.08E-10	6.28E-08	-	-0.321145	-0.2857	+	A	G	0.170443885	0.049423	+	A	G	0.1659	0.0528
rs73396548	LC_IGP82	IgG1 FBn/Fn	22	24109285	1	8.78E-10	5.50E-08	-	-0.305084	-0.2851	+	A	G	0.170443885	0.0494711	+	A	G	0.1659	0.0525
rs73396548	LC_IGP83	IgG1 FBn/Fn total	22	24109285	1	8.88E-10	6.66E-08	-	-0.304954	-0.2833	+	A	G	0.170443885	0.049464	+	A	G	0.1659	0.0525
rs73396548	LC_IGP85	IgG1 Fn/Bn total	22	24109285	1	1.71E-08	2.06E-07	+	0.280848	0.272	+	A	G	0.170443885	0.0495506	+	A	G	0.1659	0.0524
rs73396548	LC_IGP_R11	IgG1_G0FN/IgG1_G0F	22	24109285	1	7.08E-09	2.40E-07	-	-0.286746	-0.2728	+	A	G	0.170782646	0.0492714	+	A	G	0.1659	0.0528
rs73396548	LC_IGP5	IgG1_G1FN	22	24109285	1	1.72E-10	2.68E-07	-	-0.316918	-0.2708	+	A	G	0.170594874	0.0493226	+	A	G	0.1659	0.0526
rs73396548	LC_IGP_R12	IgG1_G1FN/IgG1_G1F	22	24109285	1	1.04E-08	7.57E-07	-	-0.283283	-0.2578	+	A	G	0.170594874	0.0492405	+	A	G	0.1659	0.0521
rs73396548	LC_IGP50	IgG1_G1FNn	22	24109285	1	9.21E-10	6.63E-07	-	-0.305699	-0.2618	+	A	G	0.170443885	0.049632	+	A	G	0.1659	0.0527
rs73396548	LC_IGP160	IgG2 BG0n total/G0n	22	24109285	1	1.26E-08	1.61E-11	-	-0.283973	-0.3615	+	A	G	0.170199857	0.0496425	+	A	G	0.1662	0.0537
rs73396548	LC_IGP161	IgG2 BG1n total/G1n	22	24109285	1	1.29E-08	7.52E-10	-	-0.283981	-0.3312	+	A	G	0.170199857	0.0496775	+	A	G	0.1662	0.0538
rs73396548	LC_IGP162	IgG2 BG2n total/G2n	22	24109285	1	4.00E-09	6.95E-10	-	-0.294795	-0.3291	+	A	G	0.170199857	0.049824	+	A	G	0.1662	0.0534
rs73396548	LC_IGP108	IgG2 Bisecting_GlcNAc	22	24109285	1	6.35E-10	2.00E-12	-	-0.309029	-0.3767	+	A	G	0.170615115	0.0496892	+	A	G	0.1651	0.0536
rs73396548	LC_IGP159	IgG2 Bn total	22	24109285	1	3.67E-10	3.72E-12	-	-0.312353	-0.3718	+	A	G	0.170199857	0.0495276	+	A	G	0.1662	0.0535
rs73396548	LC_IGP156	IgG2 FBG0n/G0n	22	24109285	1	6.72E-10	1.15E-11	-	-0.307621	-0.3643	+	A	G	0.170199857	0.0495366	+	A	G	0.1662	0.0537
rs73396548	LC_IGP157	IgG2 FBG1n/G1n	22	24109285	1	3.84E-09	1.00E-09	-	-0.294296	-0.3295	+	A	G	0.170199857	0.0496827	+	A	G	0.1662	0.0539
rs73396548	LC_IGP155	IgG2 FBn	22	24109285	1	3.01E-11	3.22E-12	-	-0.331008	-0.3735	+	A	G	0.170199857	0.0494582	+	A	G	0.1662	0.0536
rs73396548	LC_IGP168	IgG2 FBn/Fn	22	24109285	1	3.85E-10	4.22E-12	-	-0.311895	-0.3708	+	A	G	0.170199857	0.0495163	+	A	G	0.1662	0.0535
rs73396548	LC_IGP169	IgG2 FBn/Fn total	22	24109285	1	3.56E-10	4.20E-12	-	-0.312529	-0.3709	+	A	G	0.170199857	0.0495181	+	A	G	0.1662	0.0535
rs73396548	LC_IGP171	IgG2 Fn/Bn total	22	24109285	1	5.58E-09	7.54E-12	+	0.290775	0.3657	+	A	G	0.170199857	0.049616	+	A	G	0.1662	0.0534
rs73396548	LC_IGP_R51	IgG2_G0FN/IgG2_G0F	22	24109285	1	1.19E-08	1.51E-11	-	-0.282859	-0.3618	+	A	G	0.171085663	0.0493637	+	A	G	0.1659	0.0536
rs73396548	LC_IGP135	IgG2_G0FNn	22	24109285	1	2.20E-10	7.37E-11	-	-0.316026	-0.3363	+	A	G	0.170199857	0.0494755	+	A	G	0.1662	0.0516
rs73396548	LC_IGP_R52	IgG2_G1FN/IgG2_G1F	22	24109285	1	5.11E-09	1.23E-09	-	-0.290526	-0.327	+	A	G	0.170699085	0.049449	+	A	G	0.1659	0.0538
rs73396548	LC_IGP_R53	IgG2_G2FN/IgG2_G2F	22	24109285	1	3.72E-09	1.01E-09	-	-0.293703	-0.3269	+	A	G	0.170782646	0.0495402	+	A	G	0.1659	0.0535
rs73396549	LC_IGP74	IgG1 BG0n total/G0n	22	24109409	1	4.47E-09	8.87E-08	-	-0.29188	-0.2828	+	T	C	0.169261615	0.0494864	+	T	C	0.1635	0.0529
rs73396549	LC_IGP75	IgG1 BG1n total/G1n	22	24109409	1	5.25E-09	2.71E-07	-	-0.290386	-0.2686	+	T	C	0.169261615	0.0494611	+	T	C	0.1635	0.0522
rs73396549	LC_IGP22	IgG1 Bisecting_GlcNAc	22	24109409	1	4.90E-11	2.66E-08	-	-0.328615	-0.2915	+	T	C	0.169620678	0.0496423	+	T	C	0.1637	0.0524
rs73396549	LC_IGP73	IgG1 Bn total	22	24109409	1	3.17E-10	2.87E-08	-	-0.312504	-0.2915	+	T	C	0.169261615	0.0493703	+	T	C	0.1635	0.0525
rs73396549	LC_IGP70	IgG1 FBG0n/G0n	22	24109409	1	8.84E-10	9.95E-08	-	-0.305224	-0.2841	+	T	C	0.169261615	0.0495013	+	T	C	0.1635	0.0533
rs73396549	LC_IGP71	IgG1 FBG1n/G1n	22	24109409	1	2.34E-09	2.93E-07	-	-0.297079	-0.2683	+	T	C	0.169261615	0.0494628	+	T	C	0.1635	0.0523
rs73396549	LC_IGP69	IgG1 FBn	22	24109409	1	1.09E-10	4.32E-08	-	-0.320888	-0.2888	+	T	C	0.169261615	0.0493922	+	T	C	0.1635	0.0527
rs73396549	LC_IGP82	IgG1 FBn/Fn	22	24109409	1	7.79E-10	4.04E-08	-	-0.305836	-0.2877	+	T	C	0.169261615	0.0494365	+	T	C	0.1635	0.0524
rs73396549	LC_IGP83	IgG1 FBn/Fn total	22	24109409	1	7.83E-10	4.91E-08	-	-0.30575	-0.2858	+	T	C	0.169261615	0.0494293	+	T	C	0.1635	0.0524
rs73396549	LC_IGP85	IgG1 Fn/Bn total	22	24109409	1	1.36E-08	1.69E-07	+	0.282591	0.2736	+	T	C	0.169261615	0.0495128	+	T	C	0.1635	0.0523
rs73396549	LC_IGP_R11	IgG1_G0FN/IgG1_G0F	22	24109409	1	6.10E-09	1.69E-07	-	-0.287784	-0.2759	+	T	C	0.169613488	0.0492357	+	T	C	0.1635	0.0528
rs73396549	LC_IGP5	IgG1_G1FN	22	24109409	1	1.70E-10	1.92E-07	-	-0.316785	-0.2736	+	T	C	0.169425208	0.0492911	+	T	C	0.1635	0.0525
rs73396549	LC_IGP_R12	IgG1_G1FN/IgG1_G1F	22	24109409	1	9.01E-09	5.23E-07	-	-0.284324	-0.2613	+	T	C	0.169425208	0.049205	+	T	C	0.1635	0.0521
rs73396549	LC_IGP50	IgG1_G1FNn	22	24109409	1	8.64E-10	4.34E-07	-	-0.30601	-0.2656	+	T	C	0.169261615	0.049599	+	T	C	0.1635	0.0526
rs73396549	LC_IGP160	IgG2 BG0n total/G0n	22	24109409	1	1.43E-08	1.42E-11	-	-0.28272	-0.362	+	T	C	0.169031456	0.0496126	+	T	C	0.1638	0.0536
rs73396549	LC_IGP161	IgG2 BG1n total/G1n	22	24109409	1	1.48E-08	6.77E-10	-	-0.282659	-0.3317	+	T	C	0.169031456	0.0496479	+	T	C	0.1638	0.0538
rs73396549	LC_IGP162	IgG2 BG2n total/G2n	22	24109409	1	4.52E-09	6.95E-10	-	-0.293588	-0.3284	+	T	C	0.169031456	0.049794	+	T	C	0.1638	0.0533

rs73396549	LC_IGP108	IgG2 Bisecting_GlcNAc	22	24109409	1	8.40E-10	2.16E-12	-	-0.306635	-0.3757	+	T	C	0.16944869	0.0496638	+	T	C	0.1627	0.0535
rs73396549	LC_IGP159	IgG2 Bn total	22	24109409	1	4.33E-10	3.60E-12	-	-0.310868	-0.3716	+	T	C	0.169031456	0.0494991	+	T	C	0.1638	0.0534
rs73396549	LC_IGP156	IgG2 FBG0n/G0n	22	24109409	1	7.88E-10	9.89E-12	-	-0.306165	-0.365	+	T	C	0.169031456	0.0495079	+	T	C	0.1638	0.0536
rs73396549	LC_IGP157	IgG2 FBG1n/G1n	22	24109409	1	4.62E-09	8.94E-10	-	-0.292584	-0.3302	+	T	C	0.169031456	0.0496546	+	T	C	0.1638	0.0539
rs73396549	LC_IGP155	IgG2 FBn	22	24109409	1	3.94E-11	3.05E-12	-	-0.328834	-0.3735	+	T	C	0.169031456	0.0494327	+	T	C	0.1638	0.0535
rs73396549	LC_IGP168	IgG2 FBn/Fn	22	24109409	1	4.65E-10	4.08E-12	-	-0.310243	-0.3705	+	T	C	0.169031456	0.0494884	+	T	C	0.1638	0.0534
rs73396549	LC_IGP169	IgG2 FBn/Fn total	22	24109409	1	4.30E-10	4.09E-12	-	-0.310868	-0.3706	+	T	C	0.169031456	0.0494902	+	T	C	0.1638	0.0534
rs73396549	LC_IGP171	IgG2 Fn/Bn total	22	24109409	1	6.25E-09	7.67E-12	+	0.289643	0.3651	+	T	C	0.169031456	0.0495858	+	T	C	0.1638	0.0534
rs73396549	LC_IGP_R51	IgG2_G0FN/IgG2_G0F	22	24109409	1	1.37E-08	1.34E-11	-	-0.281534	-0.3622	+	T	C	0.16991671	0.049336	+	T	C	0.1635	0.0536
rs73396549	LC_IGP135	IgG2_G0FNn	22	24109409	1	3.04E-10	8.23E-11	-	-0.313337	-0.3349	+	T	C	0.169031456	0.0494517	+	T	C	0.1638	0.0516
rs73396549	LC_IGP_R52	IgG2_G1FN/IgG2_G1F	22	24109409	1	5.95E-09	1.13E-09	-	-0.289085	-0.3274	+	T	C	0.169528705	0.0494221	+	T	C	0.1635	0.0538
rs73396549	LC_IGP_R53	IgG2_G2FN/IgG2_G2F	22	24109409	1	4.70E-09	1.04E-09	-	-0.291613	-0.3261	+	T	C	0.169613488	0.0495156	+	T	C	0.1635	0.0534
rs5760020	LC_IGP22	IgG1 Bisecting_GlcNAc	22	24133092	1	1.08E-08	1.13E-06	+	0.229388	0.2036	+	A	G	0.722171875	0.0399087	+	A	G	0.7335	0.0418
rs5760020	LC_IGP73	IgG1 Bn total	22	24133092	1	2.45E-08	8.68E-07	+	0.222214	0.2064	+	A	G	0.722394672	0.039648	+	A	G	0.7335	0.042
rs5760020	LC_IGP69	IgG1 FBn	22	24133092	1	1.77E-08	6.76E-07	+	0.224696	0.2092	+	A	G	0.722394672	0.0396835	+	A	G	0.7335	0.0421
rs5760020	LC_IGP82	IgG1 FBn/Fn	22	24133092	1	4.41E-08	9.07E-07	+	0.21832	0.2056	+	A	G	0.722394672	0.0396936	+	A	G	0.7335	0.0419
rs5760020	LC_IGP83	IgG1 FBn/Fn total	22	24133092	1	4.42E-08	9.97E-07	+	0.218258	0.2048	+	A	G	0.722394672	0.0396878	+	A	G	0.7335	0.0419
rs2073389	LC_IGP22	IgG1 Bisecting_GlcNAc	22	24133493	1	1.08E-08	1.88E-06	+	0.229378	0.1997	+	T	C	0.722173576	0.0399083	+	T	C	0.7341	0.0419
rs2073389	LC_IGP73	IgG1 Bn total	22	24133493	1	2.45E-08	1.42E-06	+	0.222204	0.2027	+	T	C	0.722396668	0.0396476	+	T	C	0.7341	0.042
rs2073389	LC_IGP69	IgG1 FBn	22	24133493	1	1.77E-08	1.11E-06	+	0.224687	0.2054	+	T	C	0.722396668	0.039683	+	T	C	0.7341	0.0422
rs2073389	LC_IGP82	IgG1 FBn/Fn	22	24133493	1	4.41E-08	1.47E-06	+	0.218312	0.2019	+	T	C	0.722396668	0.0396931	+	T	C	0.7341	0.0419
rs2073389	LC_IGP83	IgG1 FBn/Fn total	22	24133493	1	4.43E-08	1.62E-06	+	0.21825	0.2011	+	T	C	0.722396668	0.0396873	+	T	C	0.7341	0.0419
rs9624326	LC_IGP74	IgG1 BG0n total/G0n	22	24135043	1	1.86E-09	7.24E-09	-	-0.280761	-0.2868	+	T	C	0.173937503	0.0464477	+	T	C	0.1728	0.0496
rs9624326	LC_IGP75	IgG1 BG1n total/G1n	22	24135043	1	2.38E-09	5.90E-08	-	-0.278713	-0.267	+	T	C	0.173937503	0.0464264	+	T	C	0.1728	0.0492
rs9624326	LC_IGP22	IgG1 Bisecting_GlcNAc	22	24135043	1	5.11E-11	3.77E-09	-	-0.308235	-0.2909	+	T	C	0.174190095	0.0466086	+	T	C	0.173	0.0494
rs9624326	LC_IGP73	IgG1 Bn total	22	24135043	1	1.16E-10	4.33E-09	-	-0.30056	-0.2907	+	T	C	0.173937503	0.0463351	+	T	C	0.1728	0.0495
rs9624326	LC_IGP70	IgG1 FBG0n/G0n	22	24135043	1	4.64E-10	3.24E-09	-	-0.291329	-0.2958	+	T	C	0.173937503	0.0464682	+	T	C	0.1728	0.05
rs9624326	LC_IGP71	IgG1 FBG1n/G1n	22	24135043	1	1.44E-09	4.02E-08	-	-0.282634	-0.271	+	T	C	0.173937503	0.0464364	+	T	C	0.1728	0.0494
rs9624326	LC_IGP72	IgG1 FBG2n/G2n	22	24135043	1	2.33E-08	1.68E-06	-	-0.262104	-0.2378	+	T	C	0.173937503	0.0466933	+	T	C	0.1728	0.0497
rs9624326	LC_IGP69	IgG1 FBn	22	24135043	1	5.42E-11	3.14E-09	-	-0.306185	-0.2943	+	T	C	0.173937503	0.0463642	+	T	C	0.1728	0.0497
rs9624326	LC_IGP82	IgG1 FBn/Fn	22	24135043	1	2.17E-10	5.12E-09	-	-0.296407	-0.2886	+	T	C	0.173937503	0.0463895	+	T	C	0.1728	0.0494
rs9624326	LC_IGP83	IgG1 FBn/Fn total	22	24135043	1	2.20E-10	6.02E-09	-	-0.296278	-0.2873	+	T	C	0.173937503	0.046383	+	T	C	0.1728	0.0494
rs9624326	LC_IGP85	IgG1 Fn/Bn total	22	24135043	1	3.94E-09	4.06E-08	+	0.27503	0.2703	+	T	C	0.173937503	0.0464623	+	T	C	0.1728	0.0492
rs9624326	LC_IGP_R11	IgG1_G0FN/IgG1_G0F	22	24135043	1	5.26E-09	1.10E-08	-	-0.270908	-0.2827	+	T	C	0.174327823	0.0461495	+	T	C	0.1728	0.0495
rs9624326	LC_IGP5	IgG1_G1FN	22	24135043	1	8.96E-10	2.51E-08	-	-0.285054	-0.2756	+	T	C	0.1741292	0.0462509	+	T	C	0.1728	0.0495
rs9624326	LC_IGP_R12	IgG1_G1FN/IgG1_G1F	22	24135043	1	8.93E-09	1.08E-07	-	-0.266594	-0.2608	+	T	C	0.1741292	0.0461241	+	T	C	0.1728	0.0491
rs9624326	LC_IGP50	IgG1_G1FNn	22	24135043	1	2.09E-09	7.11E-08	-	-0.280798	-0.2666	+	T	C	0.173937503	0.0466029	+	T	C	0.1728	0.0495
rs9624326	LC_IGP160	IgG2 BG0n total/G0n	22	24135043	1	1.25E-08	1.77E-12	-	-0.265963	-0.3531	+	T	C	0.173782234	0.0464768	+	T	C	0.1731	0.0501
rs9624326	LC_IGP161	IgG2 BG1n total/G1n	22	24135043	1	8.30E-10	3.14E-10	-	-0.286765	-0.3171	+	T	C	0.173782234	0.0464334	+	T	C	0.1731	0.0504
rs9624326	LC_IGP162	IgG2 BG2n total/G2n	22	24135043	1	1.58E-09	2.49E-09	-	-0.283048	-0.2992	+	T	C	0.173782234	0.0466211	+	T	C	0.1731	0.0502
rs9624326	LC_IGP108	IgG2 Bisecting_GlcNAc	22	24135043	1	6.67E-11	6.15E-13	-	-0.305427	-0.3616	+	T	C	0.174022009	0.0464711	+	T	C	0.1719	0.0503
rs9624326	LC_IGP159	IgG2 Bn total	22	24135043	1	8.05E-11	1.29E-12	-	-0.303128	-0.3557	+	T	C	0.173782234	0.0463273	+	T	C	0.1731	0.0501
rs9624326	LC_IGP156	IgG2 FBG0n/G0n	22	24135043	1	4.88E-10	9.10E-13	-	-0.290321	-0.3581	+	T	C	0.173782234	0.0463691	+	T	C	0.1731	0.0501
rs9624326	LC_IGP157	IgG2 FBG1n/G1n	22	24135043	1	2.91E-10	3.25E-10	-	-0.294603	-0.3177	+	T	C	0.173782234	0.0464427	+	T	C	0.1731	0.0505
rs9624326	LC_IGP158	IgG2 FBG2n/G2n	22	24135043	1	8.39E-09	1.46E-09	-	-0.269825	-0.305	+	T	C	0.173782234	0.0465945	+	T	C	0.1731	0.0504
rs9624326	LC_IGP155	IgG2 FBn	22	24135043	1	5.12E-12	7.59E-13	-	-0.321672	-0.3601	+	T	C	0.173782234	0.0462551	+	T	C	0.1731	0.0502
rs9624326	LC_IGP168	IgG2 FBn/Fn	22	24135043	1	4.53E-11	1.32E-12	-	-0.307004	-0.3556	+	T	C	0.173782234	0.0462992	+	T	C	0.1731	0.0501
rs9624326	LC_IGP169	IgG2 FBn/Fn total	22	24135043	1	4.23E-11	1.33E-12	-	-0.307494	-0.3555	+	T	C	0.173782234	0.0463012	+	T	C	0.1731	0.0501
rs9624326	LC_IGP171	IgG2 Fn/Bn total	22	24135043	1	9.85E-10	2.82E-12	+	0.285295	0.3495	+	T	C	0.173782234	0.046404	+	T	C	0.1731	0.05
rs9624326	LC_IGP_R51	IgG2_G0FN/IgG2_G0F	22	24135043	1	7.95E-09	1.64E-12	-	-0.268159	-0.3535	+	T	C	0.174632887	0.0462356	+	T	C	0.1728	0.0501
rs9624326	LC_IGP135	IgG2_G0FNn	22	24135043	1	8.90E-11	3.35E-11	-	-0.302212	-0.3222	+	T	C	0.173782234	0.0462956	+	T	C	0.1731	0.0486
rs34556611	LC_IGP91	IgG2_G1FN	22	24149549	1	4.23E-08	2.64E-08	-	-0.255863	-0.2816	+	T	TC	0.175082972	0.046461	+	T	TC	0.1727	0.0506
rs9624326	LC_IGP_R52	IgG2_G1FN/IgG2_G1F	22	24135043	1	1.97E-10	4.57E-10	-	-0.296098	-0.3142	+	T	C	0.17423557	0.0462333	+	T	C	0.1728	0.0504
rs9624326	LC_IGP_R53	IgG2_G2FN/IgG2_G2F	22	24135043	1	9.82E-11	2.21E-09	-	-0.301576	-0.3008	+	T	C	0.174327823	0.046309	+	T	C	0.1728	0.0503
rs17630758	LC_IGP74	IgG1 BG0n total/G0n	22	24136542	1	1.86E-09	7.25E-09	-	-0.28075	-0.2867	+	A	G	0.173938093	0.046447	+	A	G	0.1728	0.0496
rs17630758	LC_IGP75	IgG1 BG1n total/G1n	22	24136542	1	2.38E-09	5.91E-08	-	-0.27871	-0.267	+	A	G	0.173938093	0.0464258	+	A	G	0.1728	0.0492
rs17630758	LC_IGP22	IgG1 Bisecting_GlcNAc	22	24136542	1	5.12E-11	3.78E-09	-	-0.308226	-0.2909	+	A	G	0.174190691	0.046608	+	A	G	0.173	0.0494
rs17630758	LC_IGP73	IgG1 Bn total	22	24136542	1	1.16E-10	4.34E-09	-	-0.300552	-0.2906	+	A	G	0.173938093	0.0463345	+	A	G	0.1728	0.0495
rs17630758	LC_IGP70	IgG1 FBG0n/G0n	22	24136542	1	4.65E-10	3.25E-09	-	-0.291317	-0.2958	+	A	G	0.173938093	0.0464676	+	A	G	0.1728	0.05
rs17630758	LC_IGP71	IgG1 FBG1n/G1n	22	24136542	1	1.44E-09	4.03E-08	-	-0.282634	-0.271	+	A	G	0.173938093	0.0464357	+	A	G	0.1728	0.0494
rs17630758	LC_IGP72	IgG1 FBG2n/G2n	22	24136542	1	2.33E-08	1.68E-06	-	-0.262099	-0.2378	+	A	G	0.173938093	0.0466926	+	A	G	0.1728	0.0497
rs17630758	LC_IGP69	IgG1 FBn	22	24136542	1	5.42E-11	3.15E-09	-	-0.306178	-0.2943	+	A	G	0.173938093	0.0463635	+	A	G	0.1728	0.0497
rs17630758	LC_IGP82	IgG1 FBn/Fn	22	24136542	1	2.17E-10	5.13E-09	-	-0.296398	-0.2886	+	A	G	0.173938093	0.0463889	+	A	G	0.1728	0.0494
rs17630758	LC_IGP83	IgG1 FBn/Fn total	22	24136542	1	2.20E-10	6.03E-09	-	-0.29627	-0.2873	+	A	G	0.173938093	0.0463823	+	A	G	0.1728	0.0494
rs17630758	LC_IGP85	IgG1 Fn/Bn total	22	24136542	1	3.94E-09	4.07E-08	+	0.275019	0.2703	+	A	G	0.173938093	0.0464616	+	A	G	0.1728	0.0492
rs17630758	LC_IGP_R11	IgG1_G0FN/IgG1_G0F	22	24136542	1	5.26E-09	1.10E-08	-	-0.270895	-0.2827	+	A	G	0.174328406	0.0461489	+	A	G	0.1728	0.0495
rs17630758	LC_IGP5	IgG1_G1FN	22	24136542	1	8.95E-10	2.52E-08	-	-0.285055	-0.2756	+	A	G	0.174129782	0.0462502	+	A	G	0.1728	0.0495
rs17630758	LC_IGP_R12	IgG1_G1FN/IgG1_G1F	22	24136542	1	8.93E-09	1.09E-07	-	-0.26659	-0.2607	+	A	G	0.174129782	0.0461235	+	A	G	0.1728	0.0491
rs17630758	LC_IGP50	IgG1_G1FNn	22	24136542	1	2.09E-09	7.12E-08	-	-0.280799	-0.2666	+	A	G	0.173938093	0.0466022	+	A	G	0.1728	0.0495
rs17630758	LC_IGP160	IgG2 BG0n total/G0n	22	24136542	1	1.25E-08	1.78E-12	-	-0.265957	-0.3531	+	A	G	0.17378175	0.0464761	+	A	G	0.1731	0.0501
rs17630758	LC_IGP161	IgG2 BG1n total/G1n	22	24136542	1	8.31E-10	3.15E-10	-	-0.286757	-0.3171	+	A	G	0.17378175	0.0464327	+	A	G	0.1731	0.0504
rs17630758	LC_IGP162	IgG2 BG2n total/G2n	22	24136542	1	1.58E-09	2.50E-09	-	-0.283035	-0.2992	+	A	G	0.17378175	0.0466204	+	A	G	0.1731	0.0502
rs17630758	LC_IGP108	IgG2 Bisecting_GlcNAc	22	24136542	1	6.67E-11	6.17E-13	-	-0.305418	-0.3616	+	A	G	0.17402152	0.0464705	+	A	G	0.1719	0.0503
rs17630758	LC_IGP159	IgG2 Bn total	22	24136542	1	8.06E-11	1.29E-12	-	-0.303119	-0.3557	+	A	G	0.17378175	0.0463266	+	A	G	0.1731	0.0501
rs17630758	LC_IGP156	IgG2 FBG0n/G0n	22	24136542	1	4.89E-10	9.12E-13	-	-0.290312	-0.3581	+	A	G	0.17378175	0.0463685	+	A	G	0.1731	0.0501
rs17630758	LC_IGP157	IgG2 FBG1n/G1n	22	24136542	1	2.91E-10	3.26E-10	-	-0.294598	-0.3176	+	A	G	0.17378175	0.046442	+	A	G	0.1731	0.0505
rs17630758	LC_IGP158	IgG2 FBG2n/G2n	22	24136542	1	8.39E-09	1.46E-09	-	-0.269819	-0.305	+	A	G	0.17378175	0.0465939	+	A	G	0.1731	0.0504
rs17630758	LC_IGP155	IgG2 FBn	22	24136542	1	5.12E-12	7.61E-13	-	-0.321664	-0.3601	+	A	G	0.17378175	0.0462544	+	A	G	0.1731	0.0502
rs17630758	LC_IGP168	IgG2 FBn/Fn	22	24136542	1	4.53E-11	1.32E-12	-	-0.306996	-0.3556	+	A	G	0.17378175	0.0462986	+	A	G	0.1731	0.0501
rs17630758	LC_IGP169	IgG2 FBn/Fn total	22	24136542	1	4.24E-11	1.34E-12	-	-0.307486	-0.3555	+	A	G	0.17378175	0.0463006	+	A	G	0.1731	0.0501
rs17630758	LC_IGP171	IgG2 Fn/Bn total	22	24136542	1	9.86E-10	2.83E-12	+	0.285285	0.3495	+	A	G	0.17378175	0.0464034	+	A	G	0.1731	0.05
rs17630758	LC_IGP_R51	IgG2_G0FN/IgG2_G0F	22	24136542	1	7.96E-09	1.65E-12	-	-0.268151	-0.3535	+	A	G	0.17463347	0.0462349	+	A	G	0.1728	0.0501
rs17630758	LC_IGP135	IgG2_G0FNn	22	24136542	1	8.91E-11	3.35E-11	-	-0.302199	-0.3222	+	A	G	0.17378175	0.0462949	+	A	G	0.1731	0.0486
rs9620326	LC_IGP91	IgG2_G1FN	22	24146629	1	4.04E-08	2.78E-08	-	-0.255847	-0.281	+	T	C	0.174873093	0.0463894	+	T	C	0.1727	0.0506
rs17630758	LC_IGP_R52	IgG2_G1FN/IgG2_G1F	22	24136542	1	1.97E-10	4.58E-10	-	-0.296092	-0.3142	+	A	G	0.174236153	0.0462326	+	A	G	0.1728	0.0504
rs17630758	LC_IGP_R53	IgG2_G2FN/IgG2_G2F	22	24136542	1	9.83E-11	2.22E-09	-	-0.301566	-0.3007	+	A	G	0.174328406	0.0463084	+	A	G	0.1728	0.0503
rs5760023	LC_IGP22	IgG1 Bisecting_GlcNAc	22	24138301	1	1.13E-08	1.90E-06	+	0.229127	0.1996	+	T	C	0.722053924	0.0399137	+	T	C	0.7342	0.0419
rs5760023	LC_IGP73	IgG1 Bn total	22	24138301	1	2.69E-08	1.43E-06	+	0.221588	0.2026	+	T	C	0.722278053	0.0396541	+	T	C	0.7342	0.042
rs5760023	LC_IGP69	IgG1 FBn	22	24138301	1	1.89E-08	1.13E-06	+	0.224243	0.2053	+	T	C	0.722278053	0.039689	+	T	C	0.7342	0.0422
rs5760023	LC_IGP82	IgG1 FBn/Fn	22	24138301	1	4.95E-08	1.49E-06	+	0.217527	0.2018	+	T	C	0.722278053	0.0397002	+	T	C	0.7342	0.0419
rs5760023	LC_IGP83	IgG1 FBn/Fn total	22	24138301	1	4.97E-08	1.63E-06	+	0.217461	0.201	+	T	C	0.722278053	0.0396944	+	T	C	0.7342	0.0419
rs9624327	LC_IGP74	IgG1 BG0n total/G0n	22	24139274	1	2.22E-09	3.56E-08	-	-0.278984	-0.2679	+	T	C	0.17415772	0.0463785	+	T	C	0.1922	0.0486
rs9624327	LC_IGP75	IgG1 BG1n total/G1n	22	24139274	1	2.77E-09	3.55E-07	-	-0.27714	-0.2458	+	T	C	0.17415772	0.0463565	+	T	C	0.1922	0.0483
rs9624327	LC_IGP22	IgG1 Bisecting_GlcNAc	22	24139274	1	6.17E-11	2.52E-08	-	-0.306444	-0.2699	+	T	C	0.17441285	0.0465389	+	T	C	0.1924	0.0484
rs9624327	LC_IGP73	IgG1 Bn total	22	24139274	1	1.41E-10	3.07E-08	-	-0.298732	-0.269	+	T	C	0.17415772	0.0462665	+	T	C	0.1922	0.0486
rs9624327	LC_IGP70	IgG1 FBG0n/G0n	22	24139274	1	5.69E-10	1.80E-08	-	-0.289384	-0.276	+	T	C	0.17415772	0.0463998	+	T	C	0.1922	0.049
rs9624327	LC_IGP71	IgG1 FBG1n/G1n	22	24139274	1	1.70E-09	2.60E-07	-	-0.280947	-0.2494	+	T	C	0.17415772	0.0463669	+	T	C	0.1922	0.0484
rs9624327	LC_IGP69	IgG1 FBn	22	24139274	1	6.72E-11	2.32E-08	-	-0.304217	-0.2725	+	T	C	0.17415772	0.0462962	+	T	C	0.1922	0.0488
rs9624327	LC_IGP82	IgG1 FBn/Fn	22	24139274	1	2.62E-10	3.27E-08	-	-0.294616	-0.2678	+	T	C	0.17415772	0.0463207	+	T	C	0.1922	0.0485
rs9624327	LC_IGP83	IgG1 FBn/Fn total	22	24139274	1	2.64E-10	3.86E-08	-	-0.294509	-0.2664	+	T	C	0.17415772	0.046314	+	T	C	0.1922	0.0485
rs9624327	LC_IGP85	IgG1 Fn/Bn total	22	24139274	1	4.52E-09	2.16E-07	+	0.273542	0.2505	+	T	C	0.17415772	0.0463919	+	T	C	0.1922	0.0483
rs9624327	LC_IGP_R11	IgG1_G0FN/IgG1_G0F	22	24139274	1	6.18E-09	4.90E-08	-	-0.269242	-0.2646	+	T	C	0.174547732	0.0460817	+	T	C	0.1922	0.0485
rs9624327	LC_IGP5	IgG1_G1FN	22	24139274	1	1.11E-09	3.85E-07	-	-0.283039	-0.2467	+	T	C	0.174349243	0.0461843	+	T	C	0.1922	0.0486
rs9624327	LC_IGP_R12	IgG1_G1FN/IgG1_G1F	22	24139274	1	1.02E-08	6.18E-07	-	-0.265137	-0.2398	+	T	C	0.174349243	0.0460555	+	T	C	0.1922	0.0481
rs9624327	LC_IGP50	IgG1_G1FNn	22	24139274	1	2.56E-09	8.45E-07	-	-0.278808	-0.2392	+	T	C	0.17415772	0.0465342	+	T	C	0.1922	0.0486
rs9624327	LC_IGP160	IgG2 BG0n total/G0n	22	24139274	1	1.47E-08	9.48E-13	-	-0.264225	-0.3491	+	T	C	0.174004482	0.0464079	+	T	C	0.1925	0.0489
rs9624327	LC_IGP161	IgG2 BG1n total/G1n	22	24139274	1	1.03E-09	2.45E-10	-	-0.284746	-0.3116	+	T	C	0.174004482	0.0463659	+	T	C	0.1925	0.0492
rs9624327	LC_IGP162	IgG2 BG2n total/G2n	22	24139274	1	1.93E-09	2.55E-09	-	-0.281079	-0.2922	+	T	C	0.174004482	0.046553	+	T	C	0.1925	0.049
rs9624327	LC_IGP108	IgG2 Bisecting_GlcNAc	22	24139274	1	8.50E-11	3.98E-13	-	-0.303261	-0.3559	+	T	C	0.174243791	0.0464039	+	T	C	0.1914	0.0491
rs9624327	LC_IGP159	IgG2 Bn total	22	24139274	1	1.01E-10	8.46E-13	-	-0.301051	-0.35	+	T	C	0.174004482	0.0462604	+	T	C	0.1925	0.0489
rs9624327	LC_IGP156	IgG2 FBG0n/G0n	22	24139274	1	5.94E-10	5.16E-13	-	-0.288448	-0.3534	+	T	C	0.174004482	0.0463013	+	T	C	0.1925	0.0489
rs9624327	LC_IGP157	IgG2 FBG1n/G1n	22	24139274	1	3.60E-10	2.39E-10	-	-0.292601	-0.3125	+	T	C	0.174004482	0.0463752	+	T	C	0.1925	0.0493
rs9624327	LC_IGP158	IgG2 FBG2n/G2n	22	24139274	1	9.08E-09	1.02E-09	-	-0.268776	-0.3005	+	T	C	0.174004482	0.0465231	+	T	C	0.1925	0.0492
rs9624327	LC_IGP155	IgG2 FBn	22	24139274	1	6.51E-12	4.81E-13	-	-0.319597	-0.3545	+	T	C	0.174004482	0.0461886	+	T	C	0.1925	0.049
rs9624327	LC_IGP168	IgG2 FBn/Fn	22	24139274	1	5.74E-11	8.13E-13	-	-0.304905	-0.3504	+	T	C	0.174004482	0.0462326	+	T	C	0.1925	0.049
rs9624327	LC_IGP169	IgG2 FBn/Fn total	22	24139274	1	5.34E-11	8.44E-13	-	-0.305419	-0.35	+	T	C	0.174004482	0.0462345	+	T	C	0.1925	0.0489
rs9624327	LC_IGP171	IgG2 Fn/Bn total	22	24139274	1	1.21E-09	1.84E-12	+	0.283331	0.3442	+	T	C	0.174004482	0.0463364	+	T	C	0.1925	0.0488
rs9624327	LC_IGP_R51	IgG2_G0FN/IgG2_G0F	22	24139274	1	9.49E-09	8.80E-13	-	-0.266363	-0.3495	+	T	C	0.174852796	0.0461682	+	T	C	0.1922	0.0489
rs9624327	LC_IGP135	IgG2_G0FNn	22	24139274	1	1.12E-10	1.55E-11	-	-0.300146	-0.3197	+	T	C	0.174004482	0.0462287	+	T	C	0.1925	0.0474

rs17548631	LC_IGP91	IgG2_G1FN	22	24144125	1	4.28E-08	2.96E-08	-	-0.254033	-0.2803	+	C	A	0.174908425	0.0461446	+	C	A	0.1727	0.0506
rs9624327	LC_IGP_R52	IgG2_G1FN/IgG2_G1F	22	24139274	1	2.45E-10	3.26E-10	-	-0.294075	-0.3092	+	T	C	0.174455748	0.046167	+	T	C	0.1922	0.0492
rs9624327	LC_IGP_R53	IgG2_G2FN/IgG2_G2F	22	24139274	1	1.15E-10	1.66E-09	-	-0.299977	-0.296	+	T	C	0.174547732	0.0462411	+	T	C	0.1922	0.0491
rs5760025	LC_IGP22	IgG1 Bisecting_GlcNAc	22	24139866	1	1.12E-08	1.86E-06	+	0.228939	0.1998	+	A	G	0.72236875	0.0398723	+	A	G	0.734	0.0419
rs5760025	LC_IGP73	IgG1 Bn total	22	24139866	1	2.53E-08	1.40E-06	+	0.221782	0.2029	+	A	G	0.722591698	0.0396119	+	A	G	0.734	0.042
rs5760025	LC_IGP69	IgG1 FBn	22	24139866	1	1.74E-08	1.10E-06	+	0.224576	0.2056	+	A	G	0.722591698	0.0396462	+	A	G	0.734	0.0422
rs5760025	LC_IGP82	IgG1 FBn/Fn	22	24139866	1	4.49E-08	1.45E-06	+	0.217982	0.2021	+	A	G	0.722591698	0.0396571	+	A	G	0.734	0.0419
rs5760025	LC_IGP83	IgG1 FBn/Fn total	22	24139866	1	4.51E-08	1.60E-06	+	0.217919	0.2013	+	A	G	0.722591698	0.0396513	+	A	G	0.734	0.0419
rs12167679	LC_IGP74	IgG1 BG0n total/G0n	22	24141690	1	1.07E-09	1.01E-09	-	-0.275779	-0.2855	+	G	C	0.198717947	0.0449475	+	G	C	0.1974	0.0467
rs12167679	LC_IGP75	IgG1 BG1n total/G1n	22	24141690	1	8.85E-10	3.07E-08	-	-0.276935	-0.2576	+	G	C	0.198717947	0.0449151	+	G	C	0.1974	0.0465
rs12167679	LC_IGP22	IgG1 Bisecting_GlcNAc	22	24141690	1	2.03E-11	1.96E-09	-	-0.304409	-0.2795	+	G	C	0.199196224	0.0450839	+	G	C	0.1976	0.0466
rs12167679	LC_IGP73	IgG1 Bn total	22	24141690	1	4.50E-11	1.66E-09	-	-0.297309	-0.2817	+	G	C	0.198717947	0.0448278	+	G	C	0.1974	0.0467
rs12167679	LC_IGP70	IgG1 FBG0n/G0n	22	24141690	1	4.80E-10	7.10E-10	-	-0.281782	-0.2905	+	G	C	0.198717947	0.0449834	+	G	C	0.1974	0.0471
rs12167679	LC_IGP71	IgG1 FBG1n/G1n	22	24141690	1	7.94E-10	2.80E-08	-	-0.277848	-0.259	+	G	C	0.198717947	0.0449355	+	G	C	0.1974	0.0466
rs12167679	LC_IGP69	IgG1 FBn	22	24141690	1	3.04E-11	1.82E-09	-	-0.300227	-0.2819	+	G	C	0.198717947	0.044866	+	G	C	0.1974	0.0469
rs12167679	LC_IGP82	IgG1 FBn/Fn	22	24141690	1	8.69E-11	1.92E-09	-	-0.293151	-0.28	+	G	C	0.198717947	0.0448814	+	G	C	0.1974	0.0466
rs12167679	LC_IGP83	IgG1 FBn/Fn total	22	24141690	1	8.59E-11	2.15E-09	-	-0.293192	-0.2791	+	G	C	0.198717947	0.0448744	+	G	C	0.1974	0.0466
rs12167679	LC_IGP85	IgG1 Fn/Bn total	22	24141690	1	1.18E-09	1.04E-08	+	0.275032	0.2661	+	G	C	0.198717947	0.0449439	+	G	C	0.1974	0.0465
rs12167679	LC_IGP_R11	IgG1_G0FN/IgG1_G0F	22	24141690	1	8.28E-09	1.36E-09	-	-0.258933	-0.2827	+	G	C	0.199398327	0.0446982	+	G	C	0.1974	0.0466
rs12167679	LC_IGP5	IgG1_G1FN	22	24141690	1	6.87E-10	6.83E-08	-	-0.277922	-0.2523	+	G	C	0.198909558	0.0447825	+	G	C	0.1974	0.0468
rs12167679	LC_IGP_R12	IgG1_G1FN/IgG1_G1F	22	24141690	1	2.83E-09	4.90E-08	-	-0.266657	-0.253	+	G	C	0.198909558	0.0446363	+	G	C	0.1974	0.0464
rs12167679	LC_IGP50	IgG1_G1FNn	22	24141690	1	1.64E-09	1.53E-07	-	-0.273562	-0.2456	+	G	C	0.198717947	0.0451063	+	G	C	0.1974	0.0468
rs12167679	LC_IGP160	IgG2 BG0n total/G0n	22	24141690	1	3.40E-08	4.58E-12	-	-0.249677	-0.3271	+	G	C	0.198720666	0.0450156	+	G	C	0.1977	0.0473
rs12167679	LC_IGP161	IgG2 BG1n total/G1n	22	24141690	1	1.81E-09	7.74E-10	-	-0.271987	-0.2928	+	G	C	0.198720666	0.0449677	+	G	C	0.1977	0.0476
rs12167679	LC_IGP162	IgG2 BG2n total/G2n	22	24141690	1	2.64E-09	7.96E-09	-	-0.270241	-0.2734	+	G	C	0.198720666	0.0451422	+	G	C	0.1977	0.0474
rs12167679	LC_IGP108	IgG2 Bisecting_GlcNAc	22	24141690	1	2.69E-10	3.29E-12	-	-0.286077	-0.33	+	G	C	0.199443497	0.0450101	+	G	C	0.1964	0.0474
rs12167679	LC_IGP159	IgG2 Bn total	22	24141690	1	1.95E-10	4.84E-12	-	-0.287448	-0.327	+	G	C	0.198720666	0.0448677	+	G	C	0.1977	0.0473
rs12167679	LC_IGP156	IgG2 FBG0n/G0n	22	24141690	1	1.63E-09	2.47E-12	-	-0.272473	-0.3316	+	G	C	0.198720666	0.0449169	+	G	C	0.1977	0.0473
rs12167679	LC_IGP157	IgG2 FBG1n/G1n	22	24141690	1	3.16E-10	8.89E-10	-	-0.284589	-0.2926	+	G	C	0.198720666	0.0449579	+	G	C	0.1977	0.0478
rs12167679	LC_IGP158	IgG2 FBG2n/G2n	22	24141690	1	1.75E-08	5.96E-09	-	-0.255541	-0.2772	+	G	C	0.198720666	0.0451225	+	G	C	0.1977	0.0476
rs12167679	LC_IGP155	IgG2 FBn	22	24141690	1	6.46E-12	3.17E-12	-	-0.309895	-0.3305	+	G	C	0.198720666	0.0447803	+	G	C	0.1977	0.0474
rs12167679	LC_IGP168	IgG2 FBn/Fn	22	24141690	1	6.44E-11	4.92E-12	-	-0.29484	-0.327	+	G	C	0.198720666	0.0448263	+	G	C	0.1977	0.0473
rs12167679	LC_IGP169	IgG2 FBn/Fn total	22	24141690	1	6.19E-11	4.90E-12	-	-0.295129	-0.327	+	G	C	0.198720666	0.044829	+	G	C	0.1977	0.0473
rs12167679	LC_IGP171	IgG2 Fn/Bn total	22	24141690	1	2.58E-09	7.72E-12	+	0.269227	0.3231	+	G	C	0.198720666	0.0449441	+	G	C	0.1977	0.0472
rs12167679	LC_IGP_R51	IgG2_G0FN/IgG2_G0F	22	24141690	1	1.57E-08	4.07E-12	-	-0.254515	-0.3278	+	G	C	0.199703392	0.0447932	+	G	C	0.1974	0.0473
rs12167679	LC_IGP135	IgG2_G0FNn	22	24141690	1	2.19E-11	8.86E-11	-	-0.301811	-0.297	+	G	C	0.198720666	0.0447753	+	G	C	0.1977	0.0458
rs12167679	LC_IGP_R52	IgG2_G1FN/IgG2_G1F	22	24141690	1	3.15E-10	1.06E-09	-	-0.283452	-0.2906	+	G	C	0.199336504	0.0447772	+	G	C	0.1974	0.0476
rs12167679	LC_IGP_R53	IgG2_G2FN/IgG2_G2F	22	24141690	1	6.29E-10	7.97E-09	-	-0.279228	-0.2741	+	G	C	0.199398327	0.0448904	+	G	C	0.1974	0.0475
rs9608185	LC_IGP22	IgG1 Bisecting_GlcNAc	22	24142977	1	6.52E-09	1.72E-06	+	0.233731	0.2022	+	C	G	0.709955938	0.0400605	+	C	G	0.7075	0.0423
rs9608185	LC_IGP73	IgG1 Bn total	22	24142977	1	2.35E-08	1.35E-06	+	0.223324	0.2048	+	C	G	0.710521685	0.039796	+	C	G	0.7075	0.0424
rs9608185	LC_IGP69	IgG1 FBn	22	24142977	1	3.88E-08	1.27E-06	+	0.220105	0.2062	+	C	G	0.710521685	0.0398514	+	C	G	0.7075	0.0426
rs9608185	LC_IGP82	IgG1 FBn/Fn	22	24142977	1	4.89E-08	1.48E-06	+	0.218403	0.2036	+	C	G	0.710521685	0.0398452	+	C	G	0.7075	0.0423
rs9608185	LC_IGP83	IgG1 FBn/Fn total	22	24142977	1	4.88E-08	1.59E-06	+	0.218386	0.2029	+	C	G	0.710521685	0.0398392	+	C	G	0.7075	0.0423
rs9608186	LC_IGP22	IgG1 Bisecting_GlcNAc	22	24142978	1	6.52E-09	1.72E-06	+	0.233734	0.2022	+	T	C	0.709955625	0.0400604	+	T	C	0.7075	0.0423
rs9608186	LC_IGP73	IgG1 Bn total	22	24142978	1	2.35E-08	1.35E-06	+	0.223327	0.2048	+	T	C	0.710521375	0.0397959	+	T	C	0.7075	0.0424
rs9608186	LC_IGP69	IgG1 FBn	22	24142978	1	3.87E-08	1.27E-06	+	0.220109	0.2062	+	T	C	0.710521375	0.0398514	+	T	C	0.7075	0.0426
rs9608186	LC_IGP82	IgG1 FBn/Fn	22	24142978	1	4.89E-08	1.48E-06	+	0.218406	0.2036	+	T	C	0.710521375	0.0398451	+	T	C	0.7075	0.0423
rs9608186	LC_IGP83	IgG1 FBn/Fn total	22	24142978	1	4.88E-08	1.59E-06	+	0.21839	0.2029	+	T	C	0.710521375	0.0398392	+	T	C	0.7075	0.0423
rs17548631	LC_IGP74	IgG1 BG0n total/G0n	22	24144125	1	2.00E-09	7.31E-09	-	-0.27774	-0.2873	+	C	A	0.1744114	0.0460447	+	C	A	0.1727	0.0497
rs17548631	LC_IGP75	IgG1 BG1n total/G1n	22	24144125	1	2.31E-09	6.54E-08	-	-0.276521	-0.2665	+	C	A	0.1744114	0.0460206	+	C	A	0.1727	0.0493
rs17548631	LC_IGP22	IgG1 Bisecting_GlcNAc	22	24144125	1	5.54E-11	4.07E-09	-	-0.304985	-0.2908	+	C	A	0.1746875	0.0462023	+	C	A	0.1729	0.0494
rs17548631	LC_IGP73	IgG1 Bn total	22	24144125	1	1.21E-10	4.62E-09	-	-0.297644	-0.2906	+	C	A	0.1744114	0.0459321	+	C	A	0.1727	0.0496
rs17548631	LC_IGP70	IgG1 FBG0n/G0n	22	24144125	1	4.28E-10	3.27E-09	-	-0.289375	-0.2964	+	C	A	0.1744114	0.0460606	+	C	A	0.1727	0.0501
rs17548631	LC_IGP71	IgG1 FBG1n/G1n	22	24144125	1	1.35E-09	4.34E-08	-	-0.280628	-0.2707	+	C	A	0.1744114	0.0460296	+	C	A	0.1727	0.0494
rs17548631	LC_IGP72	IgG1 FBG2n/G2n	22	24144125	1	2.49E-08	1.83E-06	-	-0.259301	-0.2374	+	C	A	0.1744114	0.0462878	+	C	A	0.1727	0.0497
rs17548631	LC_IGP69	IgG1 FBn	22	24144125	1	5.17E-11	3.29E-09	-	-0.303843	-0.2943	+	C	A	0.1744114	0.0459584	+	C	A	0.1727	0.0498
rs17548631	LC_IGP82	IgG1 FBn/Fn	22	24144125	1	2.15E-10	5.55E-09	-	-0.293906	-0.2884	+	C	A	0.1744114	0.0459845	+	C	A	0.1727	0.0495
rs17548631	LC_IGP83	IgG1 FBn/Fn total	22	24144125	1	2.18E-10	6.48E-09	-	-0.293747	-0.2871	+	C	A	0.1744114	0.0459782	+	C	A	0.1727	0.0495
rs17548631	LC_IGP85	IgG1 Fn/Bn total	22	24144125	1	4.14E-09	4.36E-08	+	0.272254	0.2701	+	C	A	0.1744114	0.0460584	+	C	A	0.1727	0.0493
rs17548631	LC_IGP_R11	IgG1_G0FN/IgG1_G0F	22	24144125	1	5.18E-09	1.12E-08	-	-0.268677	-0.2832	+	C	A	0.174801708	0.0457505	+	C	A	0.1727	0.0496
rs17548631	LC_IGP5	IgG1_G1FN	22	24144125	1	9.84E-10	2.59E-08	-	-0.28191	-0.2758	+	C	A	0.174603175	0.0458535	+	C	A	0.1727	0.0495
rs17548631	LC_IGP_R12	IgG1_G1FN/IgG1_G1F	22	24144125	1	8.65E-09	1.20E-07	-	-0.264531	-0.2602	+	C	A	0.174603175	0.0457245	+	C	A	0.1727	0.0492
rs17548631	LC_IGP50	IgG1_G1FNn	22	24144125	1	2.17E-09	7.30E-08	-	-0.278072	-0.2667	+	C	A	0.1744114	0.0461974	+	C	A	0.1727	0.0495
rs17548631	LC_IGP160	IgG2 BG0n total/G0n	22	24144125	1	1.11E-08	1.62E-12	-	-0.26456	-0.355	+	C	A	0.174261084	0.0460682	+	C	A	0.173	0.0503
rs17548631	LC_IGP161	IgG2 BG1n total/G1n	22	24144125	1	7.41E-10	2.81E-10	-	-0.285094	-0.3189	+	C	A	0.174261084	0.0460252	+	C	A	0.173	0.0505
rs17548631	LC_IGP162	IgG2 BG2n total/G2n	22	24144125	1	1.30E-09	2.34E-09	-	-0.282012	-0.3006	+	C	A	0.174261084	0.0462091	+	C	A	0.173	0.0503
rs17548631	LC_IGP108	IgG2 Bisecting_GlcNAc	22	24144125	1	6.07E-11	5.57E-13	-	-0.303406	-0.3635	+	C	A	0.174519529	0.0460629	+	C	A	0.1719	0.0504
rs17548631	LC_IGP159	IgG2 Bn total	22	24144125	1	7.08E-11	1.19E-12	-	-0.301364	-0.3574	+	C	A	0.174261084	0.0459197	+	C	A	0.173	0.0503
rs17548631	LC_IGP156	IgG2 FBG0n/G0n	22	24144125	1	4.29E-10	8.29E-13	-	-0.288712	-0.36	+	C	A	0.174261084	0.0459612	+	C	A	0.173	0.0503
rs17548631	LC_IGP157	IgG2 FBG1n/G1n	22	24144125	1	2.83E-10	2.88E-10	-	-0.292219	-0.3195	+	C	A	0.174261084	0.0460369	+	C	A	0.173	0.0507
rs17548631	LC_IGP158	IgG2 FBG2n/G2n	22	24144125	1	7.19E-09	1.23E-09	-	-0.268671	-0.3071	+	C	A	0.174261084	0.0461839	+	C	A	0.173	0.0505
rs17548631	LC_IGP155	IgG2 FBn	22	24144125	1	4.73E-12	6.88E-13	-	-0.319375	-0.3619	+	C	A	0.174261084	0.0458495	+	C	A	0.173	0.0504
rs17548631	LC_IGP168	IgG2 FBn/Fn	22	24144125	1	4.04E-11	1.20E-12	-	-0.305097	-0.3574	+	C	A	0.174261084	0.0458923	+	C	A	0.173	0.0503
rs17548631	LC_IGP169	IgG2 FBn/Fn total	22	24144125	1	3.78E-11	1.21E-12	-	-0.305569	-0.3573	+	C	A	0.174261084	0.0458943	+	C	A	0.173	0.0503
rs17548631	LC_IGP171	IgG2 Fn/Bn total	22	24144125	1	8.68E-10	2.55E-12	+	0.283735	0.3513	+	C	A	0.174261084	0.0459958	+	C	A	0.173	0.0502
rs17548631	LC_IGP_R51	IgG2_G0FN/IgG2_G0F	22	24144125	1	7.02E-09	1.48E-12	-	-0.266802	-0.3554	+	C	A	0.175106772	0.0458331	+	C	A	0.1727	0.0502
rs17548631	LC_IGP135	IgG2_G0FNn	22	24144125	1	8.97E-11	3.28E-11	-	-0.299517	-0.3233	+	C	A	0.174261084	0.045892	+	C	A	0.173	0.0487
rs9624326	LC_IGP91	IgG2_G1FN	22	24135043	1	4.27E-08	3.49E-08	-	-0.256253	-0.2781	+	T	C	0.17443425	0.0465467	+	T	C	0.1728	0.0504
rs17548631	LC_IGP_R52	IgG2_G1FN/IgG2_G1F	22	24144125	1	1.82E-10	4.05E-10	-	-0.294075	-0.3161	+	C	A	0.174709835	0.0458314	+	C	A	0.1727	0.0506
rs17548631	LC_IGP_R53	IgG2_G2FN/IgG2_G2F	22	24144125	1	7.99E-11	1.92E-09	-	-0.30039	-0.3027	+	C	A	0.174801708	0.0459033	+	C	A	0.1727	0.0504
rs5760029	LC_IGP22	IgG1 Bisecting_GlcNAc	22	24144280	1	8.15E-09	2.23E-06	+	0.229826	0.1993	+	T	C	0.726573438	0.0396501	+	T	C	0.7364	0.0421
rs5760029	LC_IGP73	IgG1 Bn total	22	24144280	1	1.96E-08	1.68E-06	+	0.222343	0.2023	+	T	C	0.726775403	0.0393938	+	T	C	0.7364	0.0422
rs5760029	LC_IGP69	IgG1 FBn	22	24144280	1	1.57E-08	1.43E-06	+	0.22408	0.2044	+	T	C	0.726775403	0.0394316	+	T	C	0.7364	0.0424
rs5760029	LC_IGP82	IgG1 FBn/Fn	22	24144280	1	3.97E-08	1.72E-06	+	0.217678	0.2016	+	T	C	0.726775403	0.039442	+	T	C	0.7364	0.0422
rs5760029	LC_IGP83	IgG1 FBn/Fn total	22	24144280	1	3.99E-08	1.89E-06	+	0.217606	0.2008	+	T	C	0.726775403	0.0394362	+	T	C	0.7364	0.0422
rs9620326	LC_IGP74	IgG1 BG0n total/G0n	22	24146629	1	1.82E-09	7.39E-09	-	-0.280007	-0.2874	+	T	C	0.174346978	0.046299	+	T	C	0.1727	0.0497
rs9620326	LC_IGP75	IgG1 BG1n total/G1n	22	24146629	1	2.21E-09	6.88E-08	-	-0.278398	-0.2662	+	T	C	0.174346978	0.0462762	+	T	C	0.1727	0.0493
rs9620326	LC_IGP22	IgG1 Bisecting_GlcNAc	22	24146629	1	5.23E-11	4.24E-09	-	-0.307081	-0.2906	+	T	C	0.174592188	0.0464579	+	T	C	0.1729	0.0495
rs9620326	LC_IGP73	IgG1 Bn total	22	24146629	1	1.11E-10	4.80E-09	-	-0.29991	-0.2904	+	T	C	0.174346978	0.0461861	+	T	C	0.1727	0.0496
rs9620326	LC_IGP70	IgG1 FBG0n/G0n	22	24146629	1	4.10E-10	3.30E-09	-	-0.291295	-0.2965	+	T	C	0.174346978	0.0463165	+	T	C	0.1727	0.0501
rs9620326	LC_IGP71	IgG1 FBG1n/G1n	22	24146629	1	1.26E-09	4.54E-08	-	-0.282722	-0.2704	+	T	C	0.174346978	0.0462845	+	T	C	0.1727	0.0494
rs9620326	LC_IGP72	IgG1 FBG2n/G2n	22	24146629	1	1.87E-08	1.91E-06	-	-0.263049	-0.237	+	T	C	0.174346978	0.0465381	+	T	C	0.1727	0.0498
rs9620326	LC_IGP69	IgG1 FBn	22	24146629	1	4.70E-11	3.40E-09	-	-0.306181	-0.2942	+	T	C	0.174346978	0.0462123	+	T	C	0.1727	0.0498
rs9620326	LC_IGP82	IgG1 FBn/Fn	22	24146629	1	1.96E-10	5.80E-09	-	-0.296192	-0.2882	+	T	C	0.174346978	0.0462386	+	T	C	0.1727	0.0495
rs9620326	LC_IGP83	IgG1 FBn/Fn total	22	24146629	1	1.99E-10	6.76E-09	-	-0.296035	-0.2869	+	T	C	0.174346978	0.0462322	+	T	C	0.1727	0.0495
rs9620326	LC_IGP85	IgG1 Fn/Bn total	22	24146629	1	3.86E-09	4.53E-08	+	0.274305	0.2699	+	T	C	0.174346978	0.0463135	+	T	C	0.1727	0.0494
rs9620326	LC_IGP_R11	IgG1_G0FN/IgG1_G0F	22	24146629	1	5.16E-09	1.13E-08	-	-0.270139	-0.2833	+	T	C	0.174766398	0.0459946	+	T	C	0.1727	0.0496
rs9620326	LC_IGP5	IgG1_G1FN	22	24146629	1	8.13E-10	2.66E-08	-	-0.284809	-0.2757	+	T	C	0.174567843	0.0460933	+	T	C	0.1727	0.0496
rs9620326	LC_IGP_R12	IgG1_G1FN/IgG1_G1F	22	24146629	1	8.47E-09	1.27E-07	-	-0.266109	-0.2599	+	T	C	0.174567843	0.0459682	+	T	C	0.1727	0.0492
rs9620326	LC_IGP50	IgG1_G1FNn	22	24146629	1	1.82E-09	7.45E-08	-	-0.280922	-0.2667	+	T	C	0.174346978	0.0464504	+	T	C	0.1727	0.0496
rs9620326	LC_IGP160	IgG2 BG0n total/G0n	22	24146629	1	1.36E-08	1.56E-12	-	-0.264401	-0.3556	+	T	C	0.174228126	0.0463213	+	T	C	0.173	0.0503
rs9620326	LC_IGP161	IgG2 BG1n total/G1n	22	24146629	1	7.90E-10	2.69E-10	-	-0.286152	-0.3195	+	T	C	0.174228126	0.0462744	+	T	C	0.173	0.0506
rs9620326	LC_IGP162	IgG2 BG2n total/G2n	22	24146629	1	1.73E-09	2.29E-09	-	-0.281417	-0.301	+	T	C	0.174228126	0.0464653	+	T	C	0.173	0.0504
rs9620326	LC_IGP108	IgG2 Bisecting_GlcNAc	22	24146629	1	7.29E-11	5.37E-13	-	-0.303777	-0.3641	+	T	C	0.174486548	0.0463168	+	T	C	0.1719	0.0505
rs9620326	LC_IGP159	IgG2 Bn total	22	24146629	1	8.28E-11	1.15E-12	-	-0.30191	-0.358	+	T	C	0.174228126	0.0461708	+	T	C	0.173	0.0504
rs9620326	LC_IGP156	IgG2 FBG0n/G0n	22	24146629	1	4.97E-10	7.99E-13	-	-0.289215	-0.3606	+	T	C	0.174228126	0.0462122	+	T	C	0.173	0.0504
rs9620326	LC_IGP157	IgG2 FBG1n/G1n	22	24146629	1	2.59E-10	2.75E-10	-	-0.294429	-0.3202	+	T	C	0.174228126	0.0462818	+	T	C	0.173	0.0507
rs9620326	LC_IGP158	IgG2 FBG2n/G2n	22	24146629	1	9.31E-09	1.15E-09	-	-0.268099	-0.3079	+	T	C	0.174228126	0.0464392	+	T	C	0.173	0.0506
rs9620326	LC_IGP155	IgG2 FBn	22	24146629	1	4.95E-12	6.63E-13	-	-0.320795	-0.3625	+	T	C	0.174228126	0.0460971	+	T	C	0.173	0.0504
rs9620326	LC_IGP168	IgG2 FBn/Fn	22	24146629	1	4.43E-11	1.16E-12	-	-0.306114	-0.358	+	T	C	0.174228126	0.0461414	+	T	C	0.173	0.0504
rs9620326	LC_IGP169	IgG2 FBn/Fn total	22	24146629	1	4.13E-11	1.17E-12	-	-0.306615	-0.3579	+	T	C	0.174228126	0.0461434	+	T	C	0.173	0.0504
rs9620326	LC_IGP171	IgG2 Fn/Bn total	22	24146629	1	1.00E-09	2.45E-12	+	0.284187	0.3519	+	T	C	0.174228126	0.046247	+	T	C	0.173	0.0502
rs9620326	LC_IGP_R51	IgG2_G0FN/IgG2_G0F	22	24146629	1	8.13E-09	1.43E-12	-	-0.267097	-0.356	+	T	C	0.175069677	0.0460823	+	T	C	0.1727	0.0503
rs9620326	LC_IGP135	IgG2_G0FNn	22	24146629	1	8.70E-11	3.27E-11	-	-0.301344	-0.3236	+	T	C	0.174228126	0.0461378	+	T	C	0.173	0.0488
rs17630758	LC_IGP91	IgG2_G1FN	22	24136542	1	4.27E-08	3.50E-08	-	-0.256251	-0.2781	+	A	G	0.174434833	0.046546	+	A	G	0.1728	0.0504
rs9620326	LC_IGP_R52	IgG2_G1FN/IgG2_G1F	22	24146629	1	1.77E-10	3.87E-10	-	-0.295839	-0.3167	+	T	C	0.174674482	0.0460756	+	T	C	0.1727	0.0506
rs9620326	LC_IGP_R53	IgG2_G2FN/IgG2_G2F	22	24146629	1	1.12E-10	1.82E-09	-	-0.299655	-0.3034	+	T	C	0.174766398	0.0461577	+	T	C	0.1727	0.0505
rs34556611	LC_IGP74	IgG1 BG0n total/G0n	22	24149549	1	2.04E-09	7.46E-09	-	-0.27962	-0.2875	+	T	TC	0.174535151	0.0463794	+	T	TC	0.1727	0.0497
rs34556611	LC_IGP75	IgG1 BG1n total/G1n	22	24149549	1	2.38E-09	7.17E-08	-	-0.278288	-0.2659	+	T	TC	0.174535151	0.0463555	+	T	TC	0.1727	0.0494

rs34556611	LC_IGP22	IgG1 Bisecting_GlcNAc	22	24149549	1	5.91E-11	4.38E-09	-	-0.30674	-0.2904	+	T	TC	0.17475902	0.0465378	+	T	TC	0.1729	0.0495
rs34556611	LC_IGP73	IgG1 Bn total	22	24149549	1	1.24E-10	4.95E-09	-	-0.299658	-0.2902	+	T	TC	0.174535151	0.0462661	+	T	TC	0.1727	0.0496
rs34556611	LC_IGP70	IgG1 FBG0n/G0n	22	24149549	1	4.37E-10	3.33E-09	-	-0.291314	-0.2966	+	T	TC	0.174535151	0.0463956	+	T	TC	0.1727	0.0502
rs34556611	LC_IGP71	IgG1 FBG1n/G1n	22	24149549	1	1.31E-09	4.69E-08	-	-0.282893	-0.2702	+	T	TC	0.174535151	0.0463629	+	T	TC	0.1727	0.0495
rs34556611	LC_IGP72	IgG1 FBG2n/G2n	22	24149549	1	1.75E-08	1.97E-06	-	-0.264017	-0.2368	+	T	TC	0.174535151	0.0466139	+	T	TC	0.1727	0.0498
rs34556611	LC_IGP69	IgG1 FBn	22	24149549	1	4.94E-11	3.48E-09	-	-0.30636	-0.2941	+	T	TC	0.174535151	0.0462908	+	T	TC	0.1727	0.0498
rs34556611	LC_IGP82	IgG1 FBn/Fn	22	24149549	1	2.10E-10	6.00E-09	-	-0.296208	-0.288	+	T	TC	0.174535151	0.0463177	+	T	TC	0.1727	0.0495
rs34556611	LC_IGP83	IgG1 FBn/Fn total	22	24149549	1	2.13E-10	6.98E-09	-	-0.296053	-0.2868	+	T	TC	0.174535151	0.0463112	+	T	TC	0.1727	0.0495
rs34556611	LC_IGP85	IgG1 Fn/Bn total	22	24149549	1	4.26E-09	4.67E-08	+	0.274008	0.2698	+	T	TC	0.174535151	0.0463935	+	T	TC	0.1727	0.0494
rs34556611	LC_IGP_R11	IgG1_G0FN/IgG1_G0F	22	24149549	1	5.78E-09	1.14E-08	-	-0.269687	-0.2833	+	T	TC	0.174976149	0.0460674	+	T	TC	0.1727	0.0496
rs34556611	LC_IGP5	IgG1_G1FN	22	24149549	1	8.46E-10	2.70E-08	-	-0.284952	-0.2757	+	T	TC	0.174777721	0.0461643	+	T	TC	0.1727	0.0496
rs34556611	LC_IGP_R12	IgG1_G1FN/IgG1_G1F	22	24149549	1	9.17E-09	1.32E-07	-	-0.265899	-0.2596	+	T	TC	0.174777721	0.0460401	+	T	TC	0.1727	0.0492
rs34556611	LC_IGP50	IgG1_G1FNn	22	24149549	1	1.85E-09	7.58E-08	-	-0.281268	-0.2666	+	T	TC	0.174535151	0.0465283	+	T	TC	0.1727	0.0496
rs34556611	LC_IGP160	IgG2 BG0n total/G0n	22	24149549	1	1.57E-08	1.52E-12	-	-0.263637	-0.356	+	T	TC	0.174442291	0.0463957	+	T	TC	0.173	0.0503
rs34556611	LC_IGP161	IgG2 BG1n total/G1n	22	24149549	1	8.60E-10	2.60E-10	-	-0.28597	-0.3199	+	T	TC	0.174442291	0.0463469	+	T	TC	0.173	0.0506
rs34556611	LC_IGP162	IgG2 BG2n total/G2n	22	24149549	1	1.99E-09	2.25E-09	-	-0.280763	-0.3014	+	T	TC	0.174442291	0.0465398	+	T	TC	0.173	0.0504
rs34556611	LC_IGP108	IgG2 Bisecting_GlcNAc	22	24149549	1	8.49E-11	5.21E-13	-	-0.303181	-0.3645	+	T	TC	0.174701972	0.0463915	+	T	TC	0.1718	0.0505
rs34556611	LC_IGP159	IgG2 Bn total	22	24149549	1	9.46E-11	1.12E-12	-	-0.301441	-0.3584	+	T	TC	0.174442291	0.0462445	+	T	TC	0.173	0.0504
rs34556611	LC_IGP156	IgG2 FBG0n/G0n	22	24149549	1	5.65E-10	7.78E-13	-	-0.28872	-0.361	+	T	TC	0.174442291	0.0462859	+	T	TC	0.173	0.0504
rs34556611	LC_IGP157	IgG2 FBG1n/G1n	22	24149549	1	2.70E-10	2.65E-10	-	-0.294568	-0.3206	+	T	TC	0.174442291	0.0463532	+	T	TC	0.173	0.0508
rs34556611	LC_IGP158	IgG2 FBG2n/G2n	22	24149549	1	1.07E-08	1.09E-09	-	-0.267386	-0.3085	+	T	TC	0.174442291	0.0465136	+	T	TC	0.173	0.0506
rs34556611	LC_IGP155	IgG2 FBn	22	24149549	1	5.55E-12	6.44E-13	-	-0.320538	-0.363	+	T	TC	0.174442291	0.0461702	+	T	TC	0.173	0.0505
rs34556611	LC_IGP168	IgG2 FBn/Fn	22	24149549	1	4.96E-11	1.13E-12	-	-0.305807	-0.3584	+	T	TC	0.174442291	0.0462146	+	T	TC	0.173	0.0504
rs34556611	LC_IGP169	IgG2 FBn/Fn total	22	24149549	1	4.62E-11	1.14E-12	-	-0.30632	-0.3583	+	T	TC	0.174442291	0.0462165	+	T	TC	0.173	0.0504
rs34556611	LC_IGP171	IgG2 Fn/Bn total	22	24149549	1	1.13E-09	2.38E-12	+	0.283753	0.3523	+	T	TC	0.174442291	0.0463204	+	T	TC	0.173	0.0503
rs34556611	LC_IGP_R51	IgG2_G0FN/IgG2_G0F	22	24149549	1	9.12E-09	1.38E-12	-	-0.266606	-0.3565	+	T	TC	0.175275904	0.0461557	+	T	TC	0.1727	0.0503
rs34556611	LC_IGP135	IgG2_G0FNn	22	24149549	1	9.51E-11	3.27E-11	-	-0.301179	-0.3238	+	T	TC	0.174442291	0.0462102	+	T	TC	0.173	0.0488
rs9624327	LC_IGP91	IgG2_G1FN	22	24139274	1	4.87E-08	4.20E-08	-	-0.25478	-0.27	+	T	C	0.174654294	0.0464775	+	T	C	0.1922	0.0492
rs34556611	LC_IGP_R52	IgG2_G1FN/IgG2_G1F	22	24149549	1	1.87E-10	3.73E-10	-	-0.295931	-0.3172	+	T	TC	0.174884488	0.0461469	+	T	TC	0.1727	0.0506
rs34556611	LC_IGP_R53	IgG2_G2FN/IgG2_G2F	22	24149549	1	1.34E-10	1.74E-09	-	-0.298839	-0.304	+	T	TC	0.174976149	0.0462327	+	T	TC	0.1727	0.0505
rs112775985	LC_IGP74	IgG1 BG0n total/G0n	22	24152519	1	3.78E-09	3.49E-08	-	-0.278065	-0.2852	+	G	C	0.170907473	0.0469202	+	G	C	0.1631	0.0517
rs112775985	LC_IGP75	IgG1 BG1n total/G1n	22	24152519	1	4.77E-09	3.24E-07	-	-0.276093	-0.2621	+	G	C	0.170907473	0.0468983	+	G	C	0.1631	0.0513
rs112775985	LC_IGP22	IgG1 Bisecting_GlcNAc	22	24152519	1	1.17E-10	2.15E-08	-	-0.3054	-0.2882	+	G	C	0.171087814	0.047087	+	G	C	0.1633	0.0515
rs112775985	LC_IGP73	IgG1 Bn total	22	24152519	1	2.65E-10	2.03E-08	-	-0.297641	-0.2894	+	G	C	0.170907473	0.0468097	+	G	C	0.1631	0.0516
rs112775985	LC_IGP70	IgG1 FBG0n/G0n	22	24152519	1	6.13E-10	2.45E-08	-	-0.292128	-0.2898	+	G	C	0.170907473	0.0469287	+	G	C	0.1631	0.052
rs112775985	LC_IGP71	IgG1 FBG1n/G1n	22	24152519	1	2.24E-09	2.56E-07	-	-0.28206	-0.2641	+	G	C	0.170907473	0.0469011	+	G	C	0.1631	0.0512
rs112775985	LC_IGP69	IgG1 FBn	22	24152519	1	8.48E-11	1.94E-08	-	-0.306052	-0.2898	+	G	C	0.170907473	0.0468284	+	G	C	0.1631	0.0516
rs112775985	LC_IGP82	IgG1 FBn/Fn	22	24152519	1	4.80E-10	2.77E-08	-	-0.293552	-0.286	+	G	C	0.170907473	0.0468638	+	G	C	0.1631	0.0515
rs112775985	LC_IGP83	IgG1 FBn/Fn total	22	24152519	1	4.80E-10	3.31E-08	-	-0.293522	-0.2844	+	G	C	0.170907473	0.0468568	+	G	C	0.1631	0.0515
rs112775985	LC_IGP85	IgG1 Fn/Bn total	22	24152519	1	1.06E-08	1.55E-07	+	0.269977	0.27	+	G	C	0.170907473	0.0469428	+	G	C	0.1631	0.0514
rs112775985	LC_IGP_R11	IgG1_G0FN/IgG1_G0F	22	24152519	1	1.01E-08	5.92E-08	-	-0.268406	-0.2798	+	G	C	0.171347052	0.0466063	+	G	C	0.1631	0.0516
rs112775985	LC_IGP5	IgG1_G1FN	22	24152519	1	1.07E-09	1.29E-07	-	-0.286485	-0.2717	+	G	C	0.171148056	0.0466961	+	G	C	0.1631	0.0514
rs112775985	LC_IGP_R12	IgG1_G1FN/IgG1_G1F	22	24152519	1	1.79E-08	6.29E-07	-	-0.263617	-0.2548	+	G	C	0.171148056	0.0465826	+	G	C	0.1631	0.0512
rs112775985	LC_IGP50	IgG1_G1FNn	22	24152519	1	2.20E-09	2.90E-07	-	-0.283134	-0.2639	+	G	C	0.170907473	0.0470582	+	G	C	0.1631	0.0514
rs112775985	LC_IGP160	IgG2 BG0n total/G0n	22	24152519	1	2.00E-08	3.14E-12	-	-0.264721	-0.364	+	G	C	0.170783756	0.0469346	+	G	C	0.1634	0.0522
rs112775985	LC_IGP161	IgG2 BG1n total/G1n	22	24152519	1	1.44E-09	8.96E-10	-	-0.285403	-0.3213	+	G	C	0.170783756	0.0468931	+	G	C	0.1634	0.0524
rs112775985	LC_IGP162	IgG2 BG2n total/G2n	22	24152519	1	8.92E-10	4.05E-09	-	-0.290034	-0.3077	+	G	C	0.170783756	0.0470509	+	G	C	0.1634	0.0523
rs112775985	LC_IGP108	IgG2 Bisecting_GlcNAc	22	24152519	1	1.07E-10	8.85E-13	-	-0.304955	-0.3747	+	G	C	0.171104058	0.0469168	+	G	C	0.1622	0.0524
rs112775985	LC_IGP159	IgG2 Bn total	22	24152519	1	1.31E-10	2.00E-12	-	-0.302607	-0.3675	+	G	C	0.170783756	0.0467841	+	G	C	0.1634	0.0522
rs112775985	LC_IGP156	IgG2 FBG0n/G0n	22	24152519	1	7.96E-10	1.81E-12	-	-0.28951	-0.3681	+	G	C	0.170783756	0.0468265	+	G	C	0.1634	0.0522
rs112775985	LC_IGP157	IgG2 FBG1n/G1n	22	24152519	1	7.23E-10	1.01E-09	-	-0.290774	-0.321	+	G	C	0.170783756	0.0469126	+	G	C	0.1634	0.0526
rs112775985	LC_IGP158	IgG2 FBG2n/G2n	22	24152519	1	1.55E-08	3.10E-09	-	-0.26751	-0.3114	+	G	C	0.170783756	0.0470576	+	G	C	0.1634	0.0526
rs112775985	LC_IGP155	IgG2 FBn	22	24152519	1	1.24E-11	1.30E-12	-	-0.318869	-0.3712	+	G	C	0.170783756	0.0467225	+	G	C	0.1634	0.0523
rs112775985	LC_IGP168	IgG2 FBn/Fn	22	24152519	1	1.01E-10	2.07E-12	-	-0.304389	-0.3674	+	G	C	0.170783756	0.0467646	+	G	C	0.1634	0.0523
rs112775985	LC_IGP169	IgG2 FBn/Fn total	22	24152519	1	9.17E-11	2.12E-12	-	-0.305064	-0.367	+	G	C	0.170783756	0.0467659	+	G	C	0.1634	0.0522
rs112775985	LC_IGP171	IgG2 Fn/Bn total	22	24152519	1	1.45E-09	4.60E-12	+	0.28514	0.3604	+	G	C	0.170783756	0.0468588	+	G	C	0.1634	0.0521
rs112775985	LC_IGP_R51	IgG2_G0FN/IgG2_G0F	22	24152519	1	1.34E-08	2.95E-12	-	-0.266573	-0.3641	+	G	C	0.171646014	0.0466901	+	G	C	0.1631	0.0522
rs112775985	LC_IGP135	IgG2_G0FNn	22	24152519	1	1.64E-10	1.32E-11	-	-0.300809	-0.3439	+	G	C	0.170783756	0.0467556	+	G	C	0.1634	0.0508
rs112775985	LC_IGP_R52	IgG2_G1FN/IgG2_G1F	22	24152519	1	4.84E-10	1.41E-09	-	-0.292434	-0.3174	+	G	C	0.171252606	0.0466986	+	G	C	0.1631	0.0524
rs112775985	LC_IGP_R53	IgG2_G2FN/IgG2_G2F	22	24152519	1	1.44E-10	4.37E-09	-	-0.301766	-0.3076	+	G	C	0.171347052	0.0467599	+	G	C	0.1631	0.0524
rs7286004	LC_IGP74	IgG1 BG0n total/G0n	22	24155074	1	2.44E-09	1.21E-08	-	-0.278718	-0.2845	+	G	C	0.174626782	0.0464548	+	G	C	0.1752	0.0499
rs7286004	LC_IGP75	IgG1 BG1n total/G1n	22	24155074	1	2.69E-09	1.47E-07	-	-0.2778	-0.2604	+	G	C	0.174626782	0.0464293	+	G	C	0.1752	0.0495
rs7286004	LC_IGP22	IgG1 Bisecting_GlcNAc	22	24155074	1	6.78E-11	8.53E-09	-	-0.306254	-0.2861	+	G	C	0.174827579	0.0466118	+	G	C	0.1754	0.0497
rs7286004	LC_IGP73	IgG1 Bn total	22	24155074	1	1.45E-10	9.26E-09	-	-0.299007	-0.2862	+	G	C	0.174626782	0.0463408	+	G	C	0.1752	0.0498
rs7286004	LC_IGP70	IgG1 FBG0n/G0n	22	24155074	1	5.19E-10	6.33E-09	-	-0.290516	-0.2925	+	G	C	0.174626782	0.0464708	+	G	C	0.1752	0.0504
rs7286004	LC_IGP71	IgG1 FBG1n/G1n	22	24155074	1	1.43E-09	9.45E-08	-	-0.282688	-0.265	+	G	C	0.174626782	0.0464357	+	G	C	0.1752	0.0497
rs7286004	LC_IGP69	IgG1 FBn	22	24155074	1	5.60E-11	6.58E-09	-	-0.305961	-0.2902	+	G	C	0.174626782	0.0463646	+	G	C	0.1752	0.05
rs7286004	LC_IGP82	IgG1 FBn/Fn	22	24155074	1	2.40E-10	1.14E-08	-	-0.295707	-0.2839	+	G	C	0.174626782	0.0463917	+	G	C	0.1752	0.0497
rs7286004	LC_IGP83	IgG1 FBn/Fn total	22	24155074	1	2.43E-10	1.33E-08	-	-0.295554	-0.2826	+	G	C	0.174626782	0.0463853	+	G	C	0.1752	0.0497
rs7286004	LC_IGP85	IgG1 Fn/Bn total	22	24155074	1	4.91E-09	8.37E-08	+	0.273343	0.2658	+	G	C	0.174626782	0.0464679	+	G	C	0.1752	0.0496
rs7286004	LC_IGP_R11	IgG1_G0FN/IgG1_G0F	22	24155074	1	7.08E-09	1.87E-08	-	-0.26849	-0.2803	+	G	C	0.175091727	0.0461355	+	G	C	0.1752	0.0498
rs7286004	LC_IGP5	IgG1_G1FN	22	24155074	1	9.17E-10	5.66E-08	-	-0.284742	-0.2703	+	G	C	0.17489337	0.0462292	+	G	C	0.1752	0.0498
rs7286004	LC_IGP_R12	IgG1_G1FN/IgG1_G1F	22	24155074	1	1.04E-08	2.69E-07	-	-0.265299	-0.2541	+	G	C	0.17489337	0.046106	+	G	C	0.1752	0.0494
rs7286004	LC_IGP50	IgG1_G1FNn	22	24155074	1	1.95E-09	1.45E-07	-	-0.281305	-0.2618	+	G	C	0.174626782	0.0466004	+	G	C	0.1752	0.0498
rs7286004	LC_IGP160	IgG2 BG0n total/G0n	22	24155074	1	1.94E-08	1.27E-12	-	-0.262318	-0.3583	+	G	C	0.174560339	0.0464645	+	G	C	0.1755	0.0505
rs7286004	LC_IGP161	IgG2 BG1n total/G1n	22	24155074	1	1.00E-09	3.85E-10	-	-0.285228	-0.3176	+	G	C	0.174560339	0.0464141	+	G	C	0.1755	0.0507
rs7286004	LC_IGP162	IgG2 BG2n total/G2n	22	24155074	1	2.41E-09	3.57E-09	-	-0.279704	-0.2983	+	G	C	0.174560339	0.0466083	+	G	C	0.1755	0.0505
rs7286004	LC_IGP108	IgG2 Bisecting_GlcNAc	22	24155074	1	1.06E-10	5.89E-13	-	-0.302023	-0.3647	+	G	C	0.17481833	0.0464608	+	G	C	0.1743	0.0506
rs7286004	LC_IGP159	IgG2 Bn total	22	24155074	1	1.17E-10	1.25E-12	-	-0.300383	-0.3586	+	G	C	0.174560339	0.0463132	+	G	C	0.1755	0.0505
rs7286004	LC_IGP156	IgG2 FBG0n/G0n	22	24155074	1	6.90E-10	6.51E-13	-	-0.287657	-0.3633	+	G	C	0.174560339	0.0463543	+	G	C	0.1755	0.0505
rs7286004	LC_IGP157	IgG2 FBG1n/G1n	22	24155074	1	2.97E-10	4.01E-10	-	-0.294294	-0.3181	+	G	C	0.174560339	0.0464188	+	G	C	0.1755	0.0509
rs7286004	LC_IGP158	IgG2 FBG2n/G2n	22	24155074	1	1.26E-08	2.02E-09	-	-0.266474	-0.3042	+	G	C	0.174560339	0.0465813	+	G	C	0.1755	0.0507
rs7286004	LC_IGP155	IgG2 FBn	22	24155074	1	6.68E-12	7.07E-13	-	-0.319763	-0.3632	+	G	C	0.174560339	0.0462381	+	G	C	0.1755	0.0506
rs7286004	LC_IGP168	IgG2 FBn/Fn	22	24155074	1	6.00E-11	1.22E-12	-	-0.304917	-0.3589	+	G	C	0.174560339	0.0462826	+	G	C	0.1755	0.0505
rs7286004	LC_IGP169	IgG2 FBn/Fn total	22	24155074	1	5.59E-11	1.24E-12	-	-0.305436	-0.3586	+	G	C	0.174560339	0.0462845	+	G	C	0.1755	0.0505
rs7286004	LC_IGP171	IgG2 Fn/Bn total	22	24155074	1	1.37E-09	2.73E-12	+	0.282684	0.3524	+	G	C	0.174560339	0.0463888	+	G	C	0.1755	0.0504
rs7286004	LC_IGP_R51	IgG2_G0FN/IgG2_G0F	22	24155074	1	1.10E-08	1.16E-12	-	-0.265532	-0.3588	+	G	C	0.175390095	0.0462238	+	G	C	0.1752	0.0505
rs7286004	LC_IGP135	IgG2_G0FNn	22	24155074	1	1.11E-10	2.47E-11	-	-0.300489	-0.3269	+	G	C	0.174560339	0.0462774	+	G	C	0.1755	0.049
rs7286004	LC_IGP91	IgG2_G1FN	22	24155074	1	4.52E-08	4.27E-08	-	-0.255669	-0.2782	+	G	C	0.17519862	0.0465259	+	G	C	0.1752	0.0508
rs7286004	LC_IGP_R52	IgG2_G1FN/IgG2_G1F	22	24155074	1	2.10E-10	5.62E-10	-	-0.295511	-0.3146	+	G	C	0.175000208	0.0462128	+	G	C	0.1752	0.0507
rs7286004	LC_IGP_R53	IgG2_G2FN/IgG2_G2F	22	24155074	1	1.67E-10	3.19E-09	-	-0.297719	-0.2998	+	G	C	0.175091727	0.0463014	+	G	C	0.1752	0.0506
rs58211555	LC_IGP108	IgG2 Bisecting_GlcNAc	22	24160325	1	2.03E-08	2.88E-08	-	-0.391214	-0.4784	+	G	GT	0.092526155	0.0693943	+	G	GT	0.0826	0.0862
rs58211555	LC_IGP159	IgG2 Bn total	22	24160325	1	2.06E-08	5.12E-08	-	-0.390352	-0.4624	+	G	GT	0.092044644	0.0692709	+	G	GT	0.0834	0.0849
rs58211555	LC_IGP156	IgG2 FBG0n/G0n	22	24160325	1	3.01E-08	1.63E-08	-	-0.385735	-0.4773	+	G	GT	0.092044644	0.0692739	+	G	GT	0.0834	0.0845
rs58211555	LC_IGP157	IgG2 FBG1n/G1n	22	24160325	1	2.10E-08	1.67E-06	-	-0.390807	-0.4128	+	G	GT	0.092044644	0.0693905	+	G	GT	0.0834	0.0862
rs58211555	LC_IGP155	IgG2 FBn	22	24160325	1	3.68E-09	3.27E-08	-	-0.410442	-0.4702	+	G	GT	0.092044644	0.0692066	+	G	GT	0.0834	0.0851
rs58211555	LC_IGP168	IgG2 FBn/Fn	22	24160325	1	8.44E-09	4.34E-08	-	-0.400747	-0.4655	+	G	GT	0.092044644	0.069216	+	G	GT	0.0834	0.085
rs58211555	LC_IGP169	IgG2 FBn/Fn total	22	24160325	1	8.40E-09	4.27E-08	-	-0.400837	-0.4653	+	G	GT	0.092044644	0.0692217	+	G	GT	0.0834	0.0849
rs9624334	LC_IGP74	IgG1 BG0n total/G0n	22	24166256	1	2.37E-08	2.54E-08	-	-0.265573	-0.2836	+	C	G	0.170352343	0.0473362	+	C	G	0.1598	0.0509
rs9624334	LC_IGP75	IgG1 BG1n total/G1n	22	24166256	1	1.61E-08	4.04E-07	-	-0.268562	-0.2562	+	C	G	0.170352343	0.0472964	+	C	G	0.1598	0.0506
rs9624334	LC_IGP22	IgG1 Bisecting_GlcNAc	22	24166256	1	7.50E-10	2.78E-08	-	-0.294314	-0.2816	+	C	G	0.170304894	0.0475256	+	C	G	0.16	0.0507
rs9624334	LC_IGP73	IgG1 Bn total	22	24166256	1	1.63E-09	3.15E-08	-	-0.286445	-0.2813	+	C	G	0.170352343	0.0472244	+	C	G	0.1598	0.0508
rs9624334	LC_IGP70	IgG1 FBG0n/G0n	22	24166256	1	3.41E-09	1.74E-08	-	-0.281371	-0.2893	+	C	G	0.170352343	0.0473415	+	C	G	0.1598	0.0513
rs9624334	LC_IGP71	IgG1 FBG1n/G1n	22	24166256	1	5.36E-09	4.56E-07	-	-0.277471	-0.2557	+	C	G	0.170352343	0.0472896	+	C	G	0.1598	0.0507
rs9624334	LC_IGP69	IgG1 FBn	22	24166256	1	3.60E-10	3.64E-08	-	-0.298029	-0.281	+	C	G	0.170352343	0.0472327	+	C	G	0.1598	0.051
rs9624334	LC_IGP82	IgG1 FBn/Fn	22	24166256	1	1.86E-09	3.53E-08	-	-0.285702	-0.2796	+	C	G	0.170352343	0.0472657	+	C	G	0.1598	0.0507
rs9624334	LC_IGP83	IgG1 FBn/Fn total	22	24166256	1	1.84E-09	3.97E-08	-	-0.28572	-0.2786	+	C	G	0.170352343	0.0472584	+	C	G	0.1598	0.0507
rs9624334	LC_IGP85	IgG1 Fn/Bn total	22	24166256	1	4.36E-08	1.45E-07	+	0.260502	0.2659	+	C	G	0.170352343	0.0473469	+	C	G	0.1598	0.0506
rs9624334	LC_IGP5	IgG1_G1FN	22	24166256	1	1.76E-09	4.56E-07	-	-0.284382	-0.2565	+	C	G	0.170863461	0.0469812	+	C	G	0.1598	0.0508
rs9624334	LC_IGP50	IgG1_G1FNn	22	24166256	1	2.64E-09	1.10E-06	-	-0.283916	-0.2478	+	C	G	0.170352343	0.0474282	+	C	G	0.1598	0.0509
rs9624334	LC_IGP161	IgG2 BG1n total/G1n	22	24166256	1	9.50E-09	3.43E-10	-	-0.27242	-0.3251	+	C	G	0.170520527	0.047216	+	C	G	0.1601	0.0518
rs9624334	LC_IGP162	IgG2 BG2n total/G2n	22	24166256	1	2.67E-08	2.27E-09	-	-0.265043	-0.3082	+	C	G	0.170520527	0.0474179	+	C	G	0.1601	0.0516
rs9624334	LC_IGP108	IgG2 Bisecting_GlcNAc	22	24166256	1	1.72E-09	3.69E-13	-	-0.286292	-0.3754	+	C	G	0.170816276	0.0472634	+	C	G	0.1592	0.0517
rs9624334	LC_IGP159	IgG2 Bn total	22	24166256	1	1.61E-09	9.45E-13	-	-0.285937	-0.3678	+	C	G	0.170520527	0.0471241	+	C	G	0.1601	0.0515
rs9624334	LC_IGP156	IgG2 FBG0n/G0n	22	24166256	1	6.74E-09	1.11E-12	-	-0.27484	-0.3665	+	C	G	0.170520527	0.047156	+	C	G	0.1601	0.0515
rs9624334	LC_IGP157	IgG2 FBG1n/G1n	22	24166256	1	2.10E-09	3.84E-10	-	-0.284403	-0.3251	+	C	G	0.170520527	0.0472125	+	C	G	0.1601	0.0519
rs9624334	LC_IGP155	IgG2 FBn	22	24166256	1	1.09E-10	5.63E-13	-	-0.305696	-0.3721	+	C	G	0.170520527	0.0470526	+	C	G	0.1601	0.0516
rs9624334	LC_IGP168	IgG2 FBn/Fn	22	24166256	1	9.68E-10	9.54E-13	-	-0.289698	-0.3678	+	C	G	0.170520527	0.0470975	+	C	G	0.1601	0.0515
rs9624334	LC_IGP169	IgG2 FBn/Fn total	22	24166256	1	8.97E-10	9.61E-13	-	-0.290284	-0.3677	+	C	G	0.170520527	0.0470994	+	C	G	0.1601	0.0515

rs9624334	LC_IGP171	IgG2 Fn/Bn total	22	24166256	1	1.59E-08	2.01E-12	+	0.268086	0.3616	+	C	G	0.170520527	0.0471959	+	C	G	0.1601	0.0514
rs9624334	LC_IGP135	IgG2_G0FNn	22	24166256	1	1.97E-09	8.33E-12	-	-0.284217	-0.3414	+	C	G	0.170520527	0.0470947	+	C	G	0.1601	0.05
rs9624334	LC_IGP_R52	IgG2_G1FN/IgG2_G1F	22	24166256	1	1.94E-09	4.56E-10	-	-0.283807	-0.3229	+	C	G	0.170968239	0.0470087	+	C	G	0.1598	0.0518
rs9624334	LC_IGP_R53	IgG2_G2FN/IgG2_G2F	22	24166256	1	5.00E-09	1.75E-09	-	-0.277062	-0.3111	+	C	G	0.171064678	0.0471306	+	C	G	0.1598	0.0517
rs2186369	LC_IGP_R52	IgG2_G1FN/IgG2_G1F	22	24170996	1	1.30E-09	2.67E-10	-	-0.286322	-0.327	+	G	T	0.172480867	0.046917	+	G	T	0.1606	0.0518
rs2186369	LC_IGP_R53	IgG2_G2FN/IgG2_G2F	22	24170996	1	3.94E-09	1.08E-09	-	-0.278452	-0.3149	+	G	T	0.172575261	0.0470435	+	G	T	0.1606	0.0516
rs2186369	LC_IGP108	IgG2 Bisecting_GlcNAc	22	24170996	1	1.55E-09	2.50E-13	-	-0.286669	-0.3778	+	G	T	0.172267625	0.0471916	+	G	T	0.1599	0.0516
rs2186369	LC_IGP135	IgG2_G0FNn	22	24170996	1	1.29E-09	8.65E-12	-	-0.286928	-0.341	+	G	T	0.172046388	0.0470004	+	G	T	0.1608	0.05
rs2186369	LC_IGP155	IgG2 FBn	22	24170996	1	7.25E-11	3.45E-13	-	-0.308018	-0.3754	+	G	T	0.172046388	0.046959	+	G	T	0.1608	0.0516
rs2186369	LC_IGP156	IgG2 FBG0n/G0n	22	24170996	1	6.75E-09	6.38E-13	-	-0.274347	-0.3704	+	G	T	0.172046388	0.0470737	+	G	T	0.1608	0.0515
rs2186369	LC_IGP157	IgG2 FBG1n/G1n	22	24170996	1	1.23E-09	2.25E-10	-	-0.287985	-0.3292	+	G	T	0.172046388	0.0471148	+	G	T	0.1608	0.0519
rs2186369	LC_IGP159	IgG2 Bn total	22	24170996	1	1.58E-09	5.77E-13	-	-0.285574	-0.371	+	G	T	0.172046388	0.0470413	+	G	T	0.1608	0.0515
rs2186369	LC_IGP161	IgG2 BG1n total/G1n	22	24170996	1	8.35E-09	2.00E-10	-	-0.272965	-0.3293	+	G	T	0.172046388	0.0471299	+	G	T	0.1608	0.0518
rs2186369	LC_IGP162	IgG2 BG2n total/G2n	22	24170996	1	3.33E-08	1.46E-09	-	-0.262739	-0.3117	+	G	T	0.172046388	0.0473414	+	G	T	0.1608	0.0515
rs2186369	LC_IGP168	IgG2 FBn/Fn	22	24170996	1	7.63E-10	5.90E-13	-	-0.290958	-0.3709	+	G	T	0.172046388	0.0470086	+	G	T	0.1608	0.0515
rs2186369	LC_IGP169	IgG2 FBn/Fn total	22	24170996	1	7.13E-10	5.88E-13	-	-0.291486	-0.3709	+	G	T	0.172046388	0.0470106	+	G	T	0.1608	0.0515
rs2186369	LC_IGP171	IgG2 Fn/Bn total	22	24170996	1	1.73E-08	1.17E-12	+	0.266943	0.3653	+	G	T	0.172046388	0.0471158	+	G	T	0.1608	0.0514
rs2186369	LC_IGP22	IgG1 Bisecting_GlcNAc	22	24170996	1	6.45E-10	1.37E-08	-	-0.295058	-0.288	+	G	T	0.171803324	0.0474585	+	G	T	0.1607	0.0507
rs2186369	LC_IGP5	IgG1_G1FN	22	24170996	1	2.37E-09	2.68E-07	-	-0.281643	-0.2616	+	G	T	0.172373715	0.046909	+	G	T	0.1606	0.0508
rs2186369	LC_IGP50	IgG1_G1FNn	22	24170996	1	3.57E-09	6.79E-07	-	-0.281231	-0.2527	+	G	T	0.171836745	0.0473772	+	G	T	0.1606	0.0509
rs2186369	LC_IGP69	IgG1 FBn	22	24170996	1	3.23E-10	2.03E-08	-	-0.298439	-0.2864	+	G	T	0.171836745	0.0471704	+	G	T	0.1606	0.0511
rs2186369	LC_IGP70	IgG1 FBG0n/G0n	22	24170996	1	3.77E-09	9.45E-09	-	-0.280256	-0.2948	+	G	T	0.171836745	0.0472848	+	G	T	0.1606	0.0514
rs2186369	LC_IGP71	IgG1 FBG1n/G1n	22	24170996	1	5.06E-09	2.61E-07	-	-0.27757	-0.2612	+	G	T	0.171836745	0.0472285	+	G	T	0.1606	0.0507
rs2186369	LC_IGP73	IgG1 Bn total	22	24170996	1	1.47E-09	1.57E-08	-	-0.286913	-0.2876	+	G	T	0.171836745	0.047162	+	G	T	0.1606	0.0509
rs2186369	LC_IGP74	IgG1 BG0n total/G0n	22	24170996	1	2.37E-08	1.27E-08	-	-0.265256	-0.2899	+	G	T	0.171836745	0.0472767	+	G	T	0.1606	0.0509
rs2186369	LC_IGP75	IgG1 BG1n total/G1n	22	24170996	1	1.58E-08	2.12E-07	-	-0.268375	-0.2625	+	G	T	0.171836745	0.0472365	+	G	T	0.1606	0.0506
rs2186369	LC_IGP82	IgG1 FBn/Fn	22	24170996	1	1.62E-09	1.80E-08	-	-0.286382	-0.2857	+	G	T	0.171836745	0.0472024	+	G	T	0.1606	0.0508
rs2186369	LC_IGP83	IgG1 FBn/Fn total	22	24170996	1	1.62E-09	1.98E-08	-	-0.286322	-0.2849	+	G	T	0.171836745	0.0471955	+	G	T	0.1606	0.0508
rs2186369	LC_IGP85	IgG1 Fn/Bn total	22	24170996	1	3.92E-08	6.77E-08	+	0.261065	0.2731	+	G	T	0.171836745	0.0472844	+	G	T	0.1606	0.0506
rs34864242	LC_IGP74	IgG1 BG0n total/G0n	22	24175440	1	2.42E-08	1.36E-08	-	-0.265418	-0.2892	+	T	G	0.171836479	0.0473371	+	T	G	0.1605	0.0509
rs34864242	LC_IGP75	IgG1 BG1n total/G1n	22	24175440	1	1.61E-08	2.31E-07	-	-0.268576	-0.2616	+	T	G	0.171836479	0.0472966	+	T	G	0.1605	0.0506
rs34864242	LC_IGP22	IgG1 Bisecting_GlcNAc	22	24175440	1	6.55E-10	1.49E-08	-	-0.295315	-0.2873	+	T	G	0.171803163	0.0475189	+	T	G	0.1606	0.0507
rs34864242	LC_IGP73	IgG1 Bn total	22	24175440	1	1.49E-09	1.70E-08	-	-0.287143	-0.2869	+	T	G	0.171836479	0.0472221	+	T	G	0.1605	0.0509
rs34864242	LC_IGP70	IgG1 FBG0n/G0n	22	24175440	1	3.87E-09	1.02E-08	-	-0.280399	-0.2938	+	T	G	0.171836479	0.0473454	+	T	G	0.1605	0.0513
rs34864242	LC_IGP71	IgG1 FBG1n/G1n	22	24175440	1	5.16E-09	2.85E-07	-	-0.277772	-0.2602	+	T	G	0.171836479	0.0472888	+	T	G	0.1605	0.0507
rs34864242	LC_IGP69	IgG1 FBn	22	24175440	1	3.31E-10	2.21E-08	-	-0.298659	-0.2855	+	T	G	0.171836479	0.0472306	+	T	G	0.1605	0.051
rs34864242	LC_IGP82	IgG1 FBn/Fn	22	24175440	1	1.66E-09	1.96E-08	-	-0.286576	-0.285	+	T	G	0.171836479	0.0472627	+	T	G	0.1605	0.0508
rs34864242	LC_IGP83	IgG1 FBn/Fn total	22	24175440	1	1.66E-09	2.15E-08	-	-0.286512	-0.2841	+	T	G	0.171836479	0.0472558	+	T	G	0.1605	0.0508
rs34864242	LC_IGP85	IgG1 Fn/Bn total	22	24175440	1	3.99E-08	7.26E-08	+	0.261246	0.2726	+	T	G	0.171836479	0.0473447	+	T	G	0.1605	0.0506
rs34864242	LC_IGP5	IgG1_G1FN	22	24175440	1	2.42E-09	2.95E-07	-	-0.281812	-0.2607	+	T	G	0.172375925	0.0469677	+	T	G	0.1605	0.0508
rs34864242	LC_IGP50	IgG1_G1FNn	22	24175440	1	3.65E-09	7.41E-07	-	-0.281409	-0.2518	+	T	G	0.171836479	0.0474378	+	T	G	0.1605	0.0509
rs34864242	LC_IGP161	IgG2 BG1n total/G1n	22	24175440	1	8.41E-09	2.17E-10	-	-0.273241	-0.3286	+	T	G	0.17205002	0.0471884	+	T	G	0.1607	0.0518
rs34864242	LC_IGP162	IgG2 BG2n total/G2n	22	24175440	1	3.37E-08	1.55E-09	-	-0.262968	-0.3113	+	T	G	0.17205002	0.0474003	+	T	G	0.1607	0.0516
rs34864242	LC_IGP108	IgG2 Bisecting_GlcNAc	22	24175440	1	1.56E-09	2.72E-13	-	-0.286972	-0.3773	+	T	G	0.172270875	0.04725	+	T	G	0.1598	0.0516
rs34864242	LC_IGP159	IgG2 Bn total	22	24175440	1	1.60E-09	6.22E-13	-	-0.285842	-0.3705	+	T	G	0.17205002	0.0470998	+	T	G	0.1607	0.0515
rs34864242	LC_IGP156	IgG2 FBG0n/G0n	22	24175440	1	6.83E-09	6.94E-13	-	-0.274595	-0.3697	+	T	G	0.17205002	0.0471323	+	T	G	0.1607	0.0515
rs34864242	LC_IGP157	IgG2 FBG1n/G1n	22	24175440	1	1.24E-09	2.44E-10	-	-0.288271	-0.3285	+	T	G	0.17205002	0.0471734	+	T	G	0.1607	0.0519
rs34864242	LC_IGP155	IgG2 FBn	22	24175440	1	7.34E-11	3.72E-13	-	-0.308315	-0.3748	+	T	G	0.17205002	0.0470174	+	T	G	0.1607	0.0516
rs34864242	LC_IGP168	IgG2 FBn/Fn	22	24175440	1	7.72E-10	6.37E-13	-	-0.291235	-0.3704	+	T	G	0.17205002	0.047067	+	T	G	0.1607	0.0515
rs34864242	LC_IGP169	IgG2 FBn/Fn total	22	24175440	1	7.21E-10	6.35E-13	-	-0.291763	-0.3703	+	T	G	0.17205002	0.047069	+	T	G	0.1607	0.0515
rs34864242	LC_IGP171	IgG2 Fn/Bn total	22	24175440	1	1.75E-08	1.27E-12	+	0.26719	0.3647	+	T	G	0.17205002	0.0471744	+	T	G	0.1607	0.0514
rs34864242	LC_IGP135	IgG2_G0FNn	22	24175440	1	1.29E-09	9.08E-12	-	-0.287272	-0.3409	+	T	G	0.17205002	0.0470586	+	T	G	0.1607	0.05
rs34864242	LC_IGP_R52	IgG2_G1FN/IgG2_G1F	22	24175440	1	1.31E-09	2.91E-10	-	-0.286608	-0.3264	+	T	G	0.172482426	0.0469755	+	T	G	0.1605	0.0518
rs34864242	LC_IGP_R53	IgG2_G2FN/IgG2_G2F	22	24175440	1	4.01E-09	1.15E-09	-	-0.278651	-0.3145	+	T	G	0.172576617	0.0471023	+	T	G	0.1605	0.0517
rs5030614	LC_IGP74	IgG1 BG0n total/G0n	22	24176677	1	1.64E-08	8.79E-08	-	-0.360907	-0.3151	+	G	GCAACAGGTCATGTTCAATTTCTT	0.106236655	0.0635898	+	G	GCAACAGGTCATGTTCAATTTCTT	0.1298	0.0589
rs5030614	LC_IGP75	IgG1 BG1n total/G1n	22	24176677	1	5.61E-09	7.91E-07	-	-0.372147	-0.2876	+	G	GCAACAGGTCATGTTCAATTTCTT	0.106236655	0.0635101	+	G	GCAACAGGTCATGTTCAATTTCTT	0.1298	0.0583
rs5030614	LC_IGP22	IgG1 Bisecting_GlcNAc	22	24176677	1	4.45E-10	1.44E-07	-	-0.399604	-0.3088	+	G	GCAACAGGTCATGTTCAATTTCTT	0.106509039	0.0636674	+	G	GCAACAGGTCATGTTCAATTTCTT	0.1299	0.0587
rs5030614	LC_IGP73	IgG1 Bn total	22	24176677	1	9.36E-10	1.23E-07	-	-0.390534	-0.3106	+	G	GCAACAGGTCATGTTCAATTTCTT	0.106236655	0.0634325	+	G	GCAACAGGTCATGTTCAATTTCTT	0.1298	0.0587
rs5030614	LC_IGP70	IgG1 FBG0n/G0n	22	24176677	1	3.62E-09	1.16E-07	-	-0.377464	-0.3159	+	G	GCAACAGGTCATGTTCAATTTCTT	0.106236655	0.0636133	+	G	GCAACAGGTCATGTTCAATTTCTT	0.1298	0.0596
rs5030614	LC_IGP71	IgG1 FBG1n/G1n	22	24176677	1	2.24E-09	1.40E-06	-	-0.38192	-0.2831	+	G	GCAACAGGTCATGTTCAATTTCTT	0.106236655	0.0635079	+	G	GCAACAGGTCATGTTCAATTTCTT	0.1298	0.0587
rs5030614	LC_IGP69	IgG1 FBn	22	24176677	1	2.83E-10	2.61E-07	-	-0.402815	-0.305	+	G	GCAACAGGTCATGTTCAATTTCTT	0.106236655	0.0634557	+	G	GCAACAGGTCATGTTCAATTTCTT	0.1298	0.0592
rs5030614	LC_IGP82	IgG1 FBn/Fn	22	24176677	1	4.85E-10	1.38E-07	-	-0.397408	-0.3081	+	G	GCAACAGGTCATGTTCAATTTCTT	0.106236655	0.0634576	+	G	GCAACAGGTCATGTTCAATTTCTT	0.1298	0.0585
rs5030614	LC_IGP83	IgG1 FBn/Fn total	22	24176677	1	5.07E-10	1.53E-07	-	-0.396908	-0.3071	+	G	GCAACAGGTCATGTTCAATTTCTT	0.106236655	0.06345	+	G	GCAACAGGTCATGTTCAATTTCTT	0.1298	0.0585
rs5030614	LC_IGP85	IgG1 Fn/Bn total	22	24176677	1	1.35E-08	3.54E-07	+	0.362929	0.2966	+	G	GCAACAGGTCATGTTCAATTTCTT	0.106236655	0.0635736	+	G	GCAACAGGTCATGTTCAATTTCTT	0.1298	0.0582
rs5030614	LC_IGP_R11	IgG1_G0FN/IgG1_G0F	22	24176677	1	4.15E-08	1.05E-07	-	-0.348477	-0.3122	+	G	GCAACAGGTCATGTTCAATTTCTT	0.106041487	0.0632381	+	G	GCAACAGGTCATGTTCAATTTCTT	0.1298	0.0587
rs5030614	LC_IGP5	IgG1_G1FN	22	24176677	1	4.22E-09	1.88E-06	-	-0.374412	-0.2806	+	G	GCAACAGGTCATGTTCAATTTCTT	0.105851018	0.0633788	+	G	GCAACAGGTCATGTTCAATTTCTT	0.1298	0.0589
rs5030614	LC_IGP_R12	IgG1_G1FN/IgG1_G1F	22	24176677	1	2.56E-08	1.23E-06	-	-0.353632	-0.2814	+	G	GCAACAGGTCATGTTCAATTTCTT	0.105851018	0.0631863	+	G	GCAACAGGTCATGTTCAATTTCTT	0.1298	0.058
rs5030614	LC_IGP161	IgG2 BG1n total/G1n	22	24176677	1	1.32E-08	2.31E-09	-	-0.363079	-0.3565	+	G	GCAACAGGTCATGTTCAATTTCTT	0.106313711	0.0635511	+	G	GCAACAGGTCATGTTCAATTTCTT	0.13	0.0597
rs5030614	LC_IGP108	IgG2 Bisecting_GlcNAc	22	24176677	1	1.30E-08	1.41E-11	-	-0.364535	-0.4008	+	G	GCAACAGGTCATGTTCAATTTCTT	0.106178369	0.0637736	+	G	GCAACAGGTCATGTTCAATTTCTT	0.1294	0.0593
rs5030614	LC_IGP159	IgG2 Bn total	22	24176677	1	1.15E-08	2.04E-11	-	-0.364253	-0.3968	+	G	GCAACAGGTCATGTTCAATTTCTT	0.106313711	0.0634897	+	G	GCAACAGGTCATGTTCAATTTCTT	0.13	0.0592
rs5030614	LC_IGP156	IgG2 FBG0n/G0n	22	24176677	1	3.09E-08	3.02E-11	-	-0.353376	-0.3936	+	G	GCAACAGGTCATGTTCAATTTCTT	0.106313711	0.0635158	+	G	GCAACAGGTCATGTTCAATTTCTT	0.13	0.0592
rs5030614	LC_IGP157	IgG2 FBG1n/G1n	22	24176677	1	1.84E-09	2.80E-09	-	-0.384081	-0.3558	+	G	GCAACAGGTCATGTTCAATTTCTT	0.106313711	0.0635289	+	G	GCAACAGGTCATGTTCAATTTCTT	0.13	0.0599
rs5030614	LC_IGP155	IgG2 FBn	22	24176677	1	3.75E-10	1.36E-11	-	-0.399404	-0.4013	+	G	GCAACAGGTCATGTTCAATTTCTT	0.106313711	0.063366	+	G	GCAACAGGTCATGTTCAATTTCTT	0.13	0.0594
rs5030614	LC_IGP168	IgG2 FBn/Fn	22	24176677	1	3.08E-09	2.21E-11	-	-0.378024	-0.3962	+	G	GCAACAGGTCATGTTCAATTTCTT	0.106313711	0.0634233	+	G	GCAACAGGTCATGTTCAATTTCTT	0.13	0.0592
rs5030614	LC_IGP169	IgG2 FBn/Fn total	22	24176677	1	3.05E-09	2.16E-11	-	-0.378152	-0.3962	+	G	GCAACAGGTCATGTTCAATTTCTT	0.106313711	0.0634283	+	G	GCAACAGGTCATGTTCAATTTCTT	0.13	0.0592
rs5030614	LC_IGP135	IgG2_G0FNn	22	24176677	1	1.73E-08	4.57E-10	-	-0.359523	-0.3587	+	G	GCAACAGGTCATGTTCAATTTCTT	0.106313711	0.0634581	+	G	GCAACAGGTCATGTTCAATTTCTT	0.13	0.0576
rs5030614	LC_IGP_R52	IgG2_G1FN/IgG2_G1F	22	24176677	1	2.18E-09	2.91E-09	-	-0.38142	-0.3544	+	G	GCAACAGGTCATGTTCAATTTCTT	0.105915679	0.0633846	+	G	GCAACAGGTCATGTTCAATTTCTT	0.1298	0.0597
rs3177243	LC_IGP74	IgG1 BG0n total/G0n	22	24179922	1	2.69E-08	3.25E-08	-	-0.26611	-0.2825	+	C	G	0.171838921	0.0476216	+	C	G	0.1598	0.0511
rs3177243	LC_IGP75	IgG1 BG1n total/G1n	22	24179922	1	1.77E-08	5.01E-07	-	-0.269393	-0.255	+	C	G	0.171838921	0.0475806	+	C	G	0.1598	0.0508
rs3177243	LC_IGP22	IgG1 Bisecting_GlcNAc	22	24179922	1	7.22E-10	3.37E-08	-	-0.296334	-0.281	+	C	G	0.171807814	0.0478033	+	C	G	0.16	0.0509
rs3177243	LC_IGP73	IgG1 Bn total	22	24179922	1	1.65E-09	3.85E-08	-	-0.288086	-0.2806	+	C	G	0.171838921	0.0475058	+	C	G	0.1598	0.051
rs3177243	LC_IGP70	IgG1 FBG0n/G0n	22	24179922	1	4.48E-09	3.23E-08	-	-0.280919	-0.2849	+	C	G	0.171838921	0.0476311	+	C	G	0.1598	0.0515
rs3177243	LC_IGP71	IgG1 FBG1n/G1n	22	24179922	1	5.82E-09	7.54E-07	-	-0.278475	-0.2518	+	C	G	0.171838921	0.0475734	+	C	G	0.1598	0.0509
rs3177243	LC_IGP69	IgG1 FBn	22	24179922	1	3.78E-10	6.24E-08	-	-0.299438	-0.2773	+	C	G	0.171838921	0.0475153	+	C	G	0.1598	0.0512
rs3177243	LC_IGP82	IgG1 FBn/Fn	22	24179922	1	1.88E-09	4.55E-08	-	-0.287329	-0.2784	+	C	G	0.171838921	0.0475473	+	C	G	0.1598	0.0509
rs3177243	LC_IGP83	IgG1 FBn/Fn total	22	24179922	1	1.88E-09	4.96E-08	-	-0.287249	-0.2776	+	C	G	0.171838921	0.0475404	+	C	G	0.1598	0.0509
rs3177243	LC_IGP85	IgG1 Fn/Bn total	22	24179922	1	4.37E-08	1.32E-07	+	0.262035	0.2679	+	C	G	0.171838921	0.0476288	+	C	G	0.1598	0.0508
rs3177243	LC_IGP5	IgG1_G1FN	22	24179922	1	2.78E-09	7.08E-07	-	-0.282386	-0.253	+	C	G	0.172381354	0.0472453	+	C	G	0.1598	0.051
rs3177243	LC_IGP50	IgG1_G1FNn	22	24179922	1	4.17E-09	1.66E-06	-	-0.282034	-0.2445	+	C	G	0.171838921	0.0477237	+	C	G	0.1598	0.051
rs3177243	LC_IGP161	IgG2 BG1n total/G1n	22	24179922	1	8.75E-09	5.80E-10	-	-0.274525	-0.3217	+	C	G	0.172065485	0.0474647	+	C	G	0.16	0.0519
rs3177243	LC_IGP162	IgG2 BG2n total/G2n	22	24179922	1	3.59E-08	3.06E-09	-	-0.263973	-0.3066	+	C	G	0.172065485	0.0476785	+	C	G	0.16	0.0517
rs3177243	LC_IGP108	IgG2 Bisecting_GlcNAc	22	24179922	1	1.60E-09	7.04E-13	-	-0.288419	-0.3718	+	C	G	0.172284865	0.0475256	+	C	G	0.1591	0.0518
rs3177243	LC_IGP159	IgG2 Bn total	22	24179922	1	1.69E-09	1.66E-12	-	-0.287113	-0.3647	+	C	G	0.172065485	0.047376	+	C	G	0.16	0.0516
rs3177243	LC_IGP156	IgG2 FBG0n/G0n	22	24179922	1	7.22E-09	2.20E-12	-	-0.275759	-0.3628	+	C	G	0.172065485	0.0474087	+	C	G	0.16	0.0517
rs3177243	LC_IGP157	IgG2 FBG1n/G1n	22	24179922	1	1.30E-09	6.58E-10	-	-0.289598	-0.3215	+	C	G	0.172065485	0.0474498	+	C	G	0.16	0.052
rs3177243	LC_IGP155	IgG2 FBn	22	24179922	1	7.80E-11	1.02E-12	-	-0.309677	-0.3688	+	C	G	0.172065485	0.0472933	+	C	G	0.16	0.0518
rs3177243	LC_IGP168	IgG2 FBn/Fn	22	24179922	1	8.14E-10	1.72E-12	-	-0.292534	-0.3644	+	C	G	0.172065485	0.047343	+	C	G	0.16	0.0516
rs3177243	LC_IGP169	IgG2 FBn/Fn total	22	24179922	1	7.61E-10	1.71E-12	-	-0.29306	-0.3644	+	C	G	0.172065485	0.0473451	+	C	G	0.16	0.0516
rs3177243	LC_IGP171	IgG2 Fn/Bn total	22	24179922	1	1.83E-08	3.37E-12	+	0.268381	0.3588	+	C	G	0.172065485	0.0474507	+	C	G	0.16	0.0515
rs3177243	LC_IGP135	IgG2_G0FNn	22	24179922	1	1.32E-09	1.40E-11	-	-0.288784	-0.3387	+	C	G	0.172065485	0.0473336	+	C	G	0.16	0.0501
rs3177243	LC_IGP_R52	IgG2_G1FN/IgG2_G1F	22	24179922	1	1.36E-09	7.61E-10	-	-0.287985	-0.3196	+	C	G	0.172489712	0.0472505	+	C	G	0.1598	0.0519
rs3177243	LC_IGP_R53	IgG2_G2FN/IgG2_G2F	22	24179922	1	4.40E-09	2.26E-09	-	-0.279562	-0.3097	+	C	G	0.172583695	0.0473793	+	C	G	0.1598	0.0518
rs6519476	LC_IGP155	IgG2 FBn	22	24182500	2	1.19E-09	9.57E-09	-	-0.259124	-0.2448	+	A	G	0.259420418	0.0423553	+	A	G	0.2553	0.0427
rs6519476	LC_IGP_R52	IgG2_G1FN/IgG2_G1F	22	24182500	2	5.37E-09	5.09E-08	-	-0.247479	-0.2332	+	A	G	0.260017874	0.0421839	+	A	G	0.2557	0.0428
rs6519476	LC_IGP157	IgG2 FBG1n/G1n	22	24182500	2	6.10E-09	7.31E-08	-	-0.248224	-0.2314	+	A	G	0.259420418	0.0424654	+	A	G	0.2553	0.043
rs6519476	LC_IGP169	IgG2 FBn/Fn total	22	24182500	2	6.24E-09	8.46E-09	-	-0.247593	-0.2452	+	A	G	0.259420418	0.0423858	+	A	G	0.2553	0.0426
rs6519476	LC_IGP168	IgG2 FBn/Fn	22	24182500	2	6.76E-09	8.69E-09	-	-0.247014	-0.245	+	A	G	0.259420418	0.0423843	+	A	G	0.2553	0.0426
rs6519476	LC_IGP159	IgG2 Bn total	22	24182500	2	7.93E-09	8.73E-09	-	-0.245937	-0.245	+	A	G	0.259420418	0.0423993	+	A	G	0.2553	0.0426
rs6519476	LC_IGP108	IgG2 Bisecting_GlcNAc	22	24182500	2	1.07E-08	2.08E-08	-	-0.244508	-0.2386	+	A	G	0.259597805	0.0425335	+	A	G	0.2545	0.0426
rs6519476	LC_IGP161	IgG2 BG1n total/G1n	22	24182500	2	1.58E-08	5.32E-08	-	-0.241216	-0.2331	+	A	G	0.259420418	0.0424542	+	A	G	0.2553	0.0428
rs6519476	LC_IGP156	IgG2 FBG0n/G0n	22	24182500	2	3.37E-08	2.01E-08	-	-0.235379	-0.2389	+	A	G	0.259420418	0.0424283	+	A	G	0.2553	0.0426
rs6519476	LC_IGP171	IgG2 Fn/Bn total	22	24182500	2	4.83E-08	8.44E-09	+	0.232773	0.2446	+	A	G	0.259420418	0.0424515	+	A	G	0.2553	0.0425
rs6519476	LC_IGP135	IgG2_G0FNn	22	24182500	2	4.93E-08	1.30E-06	-	-0.232416	-0.1992	+	A	G	0.259420418	0.0424141	+	A	G	0.2553	0.0412
rs8138673	LC_IGP_R52	IgG2_G1FN/IgG2_G1F	22	24189032	2	4.95E-08	9.85E-08	-	-0.245019	-0.2501	+	G	C	0.282184789	0.044722	+	G	C	0.2554	0.0469
rs137680	LC_IGP_R81	IgG4_G0FN/IgG4_G0F	22	39737929	3	7.82E-11	3.35E-07	-	-0.271863	-0.2543	+	T	C	0.594363026	0.0415231	+	T	C	0.4789	0.0498
rs137682	LC_IGP_R11	IgG1_G0FN/IgG1_G0F	22	39738425	4	8.23E-10	2.93E-07	-	-0.246657	-0.2185	+	C	A	0.738216436	0.0399317	+	C	A	0.7726	0.0426
rs137683	LC_IGP_R11	IgG1_G0FN/IgG1_G0F	22	39738501	4	4.01E-10	1.17E-08	-	-0.24507	-0.2421	+	C	G	0.722126602	0.0389498	+	C	G	0.7566	0.0424
rs137686	LC_IGP_R11	IgG1_G0FN/IgG1_G0F	22	39739638	4	3.37E-11	1.40E-09	-	-0.246355	-0.2497	+	G	C	0.705613179	0.0369067	+	G	C	0.7336	0.0412
rs137690	LC_IGP_R11	IgG1_G0FN/IgG1_G0F	22	39742825	4	2.29E-11	1.36E-09	-	-0.248823	-0.25	+	G	C	0.705164124	0.0369547	+	G	C	0.7336	0.0413

rs9611156	LC_IGP_R11	IgG1_G0FN/IgG1_G0F	22	39743170	4	7.83E-11	1.29E-09	-	-0.242825	-0.2517	+	T	G	0.701215985	0.0370887	+	T	G	0.7267	0.0415
rs137700	LC_IGP_R11	IgG1_G0FN/IgG1_G0F	22	39749072	4	1.64E-11	1.36E-09	-	-0.251573	-0.2591	+	G	A	0.70502227	0.03709	+	G	A	0.7027	0.0428
rs137701	LC_IGP_R11	IgG1_G0FN/IgG1_G0F	22	39750146	4	5.83E-12	1.21E-09	-	-0.258379	-0.2552	+	C	T	0.7115241	0.037257	+	C	T	0.7305	0.042
rs137702	LC_IGP_R11	IgG1_G0FN/IgG1_G0F	22	39750158	4	5.82E-12	4.41E-10	-	-0.258382	-0.2622	+	C	T	0.711524405	0.037257	+	C	T	0.7373	0.042
rs137705	LC_IGP_R11	IgG1_G0FN/IgG1_G0F	22	39751394	4	2.18E-08	2.89E-09	-	-0.200703	-0.2357	+	C	T	0.653327334	0.0356827	+	C	T	0.6726	0.0397
rs137706	LC_IGP_R11	IgG1_G0FN/IgG1_G0F	22	39751400	4	3.47E-12	1.16E-09	-	-0.261167	-0.2582	+	G	T	0.708907871	0.0372549	+	G	T	0.7192	0.0424
rs137707	LC_IGP_R11	IgG1_G0FN/IgG1_G0F	22	39751401	4	3.46E-12	1.13E-09	-	-0.261169	-0.2584	+	C	T	0.708908176	0.0372548	+	C	T	0.7192	0.0424
rs137708	LC_IGP_R11	IgG1_G0FN/IgG1_G0F	22	39751774	4	4.07E-12	5.36E-10	-	-0.260276	-0.2615	+	G	A	0.709212325	0.0372518	+	G	A	0.7316	0.0421
rs137709	LC_IGP_R11	IgG1_G0FN/IgG1_G0F	22	39752203	4	5.34E-12	7.82E-11	-	-0.25959	-0.2764	+	ATGGGATTACAGG	A	0.707157413	0.0373631	+	ATGGGATTACAGG	A	0.7033	0.0425
rs2413586	LC_IGP_R11	IgG1_G0FN/IgG1_G0F	22	39753722	4	2.57E-08	1.41E-08	-	-0.200105	-0.2345	+	T	C	0.646302013	0.0357587	+	T	C	0.6137	0.0413
rs2413587	LC_IGP_R11	IgG1_G0FN/IgG1_G0F	22	39753779	4	2.47E-08	1.86E-09	-	-0.199619	-0.2375	+	C	T	0.653479866	0.0356296	+	C	T	0.673	0.0395
rs5757630	LC_IGP_R11	IgG1_G0FN/IgG1_G0F	22	39754433	4	2.60E-08	1.86E-09	-	-0.199177	-0.2376	+	T	G	0.653509457	0.0356057	+	T	G	0.6735	0.0395
rs2413588	LC_IGP_R11	IgG1_G0FN/IgG1_G0F	22	39755175	4	2.97E-08	1.77E-09	-	-0.197853	-0.2382	+	G	A	0.654362416	0.0355208	+	G	A	0.6747	0.0396
rs760742	LC_IGP_R11	IgG1_G0FN/IgG1_G0F	22	39755190	4	2.96E-08	1.60E-09	-	-0.197914	-0.2391	+	T	G	0.654468578	0.0355279	+	T	G	0.6752	0.0396
rs2413589	LC_IGP_R11	IgG1_G0FN/IgG1_G0F	22	39755689	4	3.91E-12	1.61E-10	-	-0.260181	-0.2676	+	C	T	0.710450275	0.0372071	+	C	T	0.7363	0.0419
rs5757633	LC_IGP_R11	IgG1_G0FN/IgG1_G0F	22	39756270	4	2.95E-08	1.63E-09	-	-0.198314	-0.2392	+	G	A	0.654368822	0.0355965	+	G	A	0.6752	0.0397
rs4821887	LC_IGP_R11	IgG1_G0FN/IgG1_G0F	22	39756985	4	2.64E-12	1.46E-10	-	-0.263565	-0.2692	+	T	C	0.710935937	0.0373891	+	T	C	0.7368	0.042
rs5757642	LC_IGP_R11	IgG1_G0FN/IgG1_G0F	22	39764824	4	1.25E-19	2.44E-17	-	-0.325151	-0.3238	+	C	T	0.608274863	0.0354142	+	C	T	0.643	0.0382
rs7286714	LC_IGP_R11	IgG1_G0FN/IgG1_G0F	22	39765604	4	2.38E-19	1.20E-17	-	-0.322009	-0.3306	+	G	C	0.601641245	0.0353558	+	G	C	0.6329	0.0386
rs5757644	LC_IGP_R11	IgG1_G0FN/IgG1_G0F	22	39766440	4	1.67E-19	1.07E-17	-	-0.323596	-0.3316	+	G	C	0.60171568	0.0353722	+	G	C	0.6329	0.0387
rs5750806	LC_IGP_R11	IgG1_G0FN/IgG1_G0F	22	39767011	4	1.51E-19	1.04E-17	-	-0.324028	-0.3318	+	G	A	0.601745514	0.0353757	+	G	A	0.633	0.0387
rs9611162	LC_IGP_R11	IgG1_G0FN/IgG1_G0F	22	39767247	4	1.47E-19	1.05E-17	-	-0.324148	-0.332	+	A	C	0.601740696	0.0353772	+	A	C	0.633	0.0387
rs9611164	LC_IGP_R11	IgG1_G0FN/IgG1_G0F	22	39767291	4	1.42E-19	7.22E-18	-	-0.324424	-0.336	+	A	G	0.60149817	0.0353929	+	A	G	0.6248	0.039
rs5757645	LC_IGP_R11	IgG1_G0FN/IgG1_G0F	22	39768479	4	1.20E-19	9.26E-18	-	-0.32504	-0.3328	+	A	G	0.601792556	0.0353864	+	A	G	0.6328	0.0388
rs1569499	LC_IGP_R11	IgG1_G0FN/IgG1_G0F	22	39769818	4	4.06E-20	7.67E-18	-	-0.330279	-0.332	+	C	T	0.606561318	0.0354852	+	C	T	0.64	0.0386
rs4821888	LC_IGP_R11	IgG1_G0FN/IgG1_G0F	22	39770597	4	3.03E-20	7.31E-18	-	-0.331477	-0.3323	+	G	A	0.607441493	0.0354892	+	G	A	0.6413	0.0386
rs4820377	LC_IGP_R11	IgG1_G0FN/IgG1_G0F	22	39770780	4	4.57E-20	6.22E-18	-	-0.329945	-0.3322	+	A	G	0.607897869	0.0354993	+	A	G	0.6422	0.0385
rs6001566	LC_IGP_R11	IgG1_G0FN/IgG1_G0F	22	39774448	4	1.00E-25	6.50E-19	-	-0.378965	-0.357	+	G	A	0.626967968	0.0355291	+	G	A	0.6567	0.0402
rs6519190	LC_IGP_R11	IgG1_G0FN/IgG1_G0F	22	39774525	4	7.05E-26	2.03E-19	-	-0.379821	-0.3621	+	G	A	0.627102579	0.0354937	+	G	A	0.6569	0.0402
rs5757647	LC_IGP_R11	IgG1_G0FN/IgG1_G0F	22	39775047	4	1.25E-25	5.10E-19	-	-0.378353	-0.3555	+	A	C	0.629250458	0.0355448	+	A	C	0.6616	0.0399
rs5757648	LC_IGP_R11	IgG1_G0FN/IgG1_G0F	22	39775156	4	1.22E-25	6.13E-19	-	-0.378404	-0.3548	+	A	G	0.629255034	0.0355434	+	A	G	0.6609	0.0399
rs9611165	LC_IGP_R11	IgG1_G0FN/IgG1_G0F	22	39775250	4	1.20E-25	5.05E-19	-	-0.378451	-0.3555	+	G	A	0.629261135	0.0355421	+	G	A	0.6616	0.0399
rs9611166	LC_IGP_R11	IgG1_G0FN/IgG1_G0F	22	39775268	4	1.20E-25	5.04E-19	-	-0.378454	-0.3556	+	C	T	0.629260525	0.0355418	+	C	T	0.6616	0.0399
rs6001567	LC_IGP_R11	IgG1_G0FN/IgG1_G0F	22	39775400	4	1.18E-25	5.02E-19	-	-0.378501	-0.3556	+	T	A	0.629265711	0.0355398	+	T	A	0.6616	0.0399
rs9611167	LC_IGP_R11	IgG1_G0FN/IgG1_G0F	22	39775583	4	1.08E-25	9.01E-19	-	-0.378486	-0.3551	+	A	G	0.62500122	0.03551	+	A	G	0.6543	0.0401
rs6001568	LC_IGP_R11	IgG1_G0FN/IgG1_G0F	22	39775786	4	7.82E-26	5.89E-19	-	-0.379252	-0.3539	+	A	G	0.630751373	0.0354743	+	A	G	0.6632	0.0398
rs4821889	LC_IGP_R11	IgG1_G0FN/IgG1_G0F	22	39777254	4	9.53E-26	6.34E-19	-	-0.378189	-0.357	+	C	T	0.625502746	0.0354403	+	C	T	0.654	0.0402
rs4821890	LC_IGP_R11	IgG1_G0FN/IgG1_G0F	22	39777523	4	9.10E-26	6.07E-19	-	-0.378306	-0.3572	+	A	G	0.625520134	0.0354359	+	A	G	0.6541	0.0402
rs1010169	LC_IGP_R11	IgG1_G0FN/IgG1_G0F	22	39778167	4	8.98E-26	5.35E-19	-	-0.378338	-0.3542	+	G	A	0.630963392	0.0354345	+	G	A	0.6636	0.0398
rs1010170	LC_IGP_R11	IgG1_G0FN/IgG1_G0F	22	39778327	4	8.71E-26	4.05E-19	-	-0.378416	-0.3559	+	C	T	0.630961257	0.0354319	+	C	T	0.6629	0.0398
rs5757650	LC_IGP_R11	IgG1_G0FN/IgG1_G0F	22	39778419	4	1.26E-25	6.19E-19	-	-0.376911	-0.356	+	C	T	0.628325808	0.0354112	+	C	T	0.6583	0.0401
rs756640	LC_IGP_R11	IgG1_G0FN/IgG1_G0F	22	39779300	4	6.88E-26	5.98E-19	-	-0.378924	-0.3572	+	A	G	0.625589994	0.0354017	+	A	G	0.654	0.0402
rs7423	LC_IGP_R11	IgG1_G0FN/IgG1_G0F	22	39781429	4	3.92E-26	5.21E-19	-	-0.380716	-0.3579	+	T	C	0.626133236	0.0353855	+	T	C	0.6542	0.0402
rs1007337	LC_IGP_R11	IgG1_G0FN/IgG1_G0F	22	39781585	4	7.15E-26	5.35E-19	-	-0.37831	-0.3568	+	T	C	0.627876144	0.0353571	+	T	C	0.6587	0.0401
rs5757652	LC_IGP_R11	IgG1_G0FN/IgG1_G0F	22	39781855	4	1.48E-26	1.09E-18	-	-0.382478	-0.3615	+	C	T	0.624656193	0.0352351	+	C	T	0.6322	0.041
rs9611169	LC_IGP_R11	IgG1_G0FN/IgG1_G0F	22	39783027	4	4.14E-26	4.31E-19	-	-0.379282	-0.355	+	T	C	0.632014338	0.0352696	+	T	C	0.664	0.0398
rs9611170	LC_IGP_R11	IgG1_G0FN/IgG1_G0F	22	39784845	4	9.36E-27	4.63E-21	-	-0.384058	-0.3832	+	C	G	0.628837401	0.0352355	+	C	G	0.6725	0.0407
rs2142848	LC_IGP_R11	IgG1_G0FN/IgG1_G0F	22	39785242	4	1.45E-26	5.90E-21	-	-0.382589	-0.3818	+	T	C	0.630293167	0.0352384	+	T	C	0.6717	0.0406
rs4821891	LC_IGP_R11	IgG1_G0FN/IgG1_G0F	22	39785381	4	1.45E-26	8.06E-21	-	-0.382588	-0.3811	+	T	C	0.630293777	0.0352382	+	T	C	0.671	0.0407
rs2413590	LC_IGP_R11	IgG1_G0FN/IgG1_G0F	22	39790191	4	1.18E-26	8.63E-21	-	-0.384726	-0.3788	+	C	G	0.636996949	0.0353702	+	C	G	0.6799	0.0405
rs5750808	LC_IGP_R11	IgG1_G0FN/IgG1_G0F	22	39790987	4	1.17E-26	8.63E-21	-	-0.384728	-0.3788	+	A	G	0.63699878	0.0353689	+	A	G	0.6799	0.0405
rs5750809	LC_IGP_R11	IgG1_G0FN/IgG1_G0F	22	39791491	4	5.95E-26	7.29E-21	-	-0.379367	-0.3814	+	G	T	0.634429225	0.0353952	+	G	T	0.6727	0.0407
rs5750810	LC_IGP_R11	IgG1_G0FN/IgG1_G0F	22	39792943	4	1.16E-26	8.54E-21	-	-0.384725	-0.3788	+	G	A	0.637005186	0.0353656	+	G	A	0.6799	0.0405
rs5750811	LC_IGP_R11	IgG1_G0FN/IgG1_G0F	22	39793066	4	1.16E-26	8.55E-21	-	-0.384723	-0.3788	+	G	T	0.637005186	0.0353654	+	G	T	0.6799	0.0405
rs5750812	LC_IGP_R11	IgG1_G0FN/IgG1_G0F	22	39793079	4	9.24E-27	8.97E-21	-	-0.385662	-0.3806	+	A	G	0.632947834	0.0353785	+	A	G	0.6727	0.0407
rs4321460	LC_IGP_R11	IgG1_G0FN/IgG1_G0F	22	39793655	4	1.49E-26	2.75E-17	-	-0.384331	-0.3644	+	G	A	0.635403905	0.0354078	+	G	A	0.6362	0.0431
rs4386422	LC_IGP_R11	IgG1_G0FN/IgG1_G0F	22	39793734	4	7.90E-26	6.15E-20	-	-0.381916	-0.3817	+	T	C	0.628008542	0.0357268	+	T	C	0.6293	0.0418
rs4384886	LC_IGP_R11	IgG1_G0FN/IgG1_G0F	22	39793766	4	9.17E-27	8.83E-21	-	-0.385672	-0.3808	+	T	C	0.63295058	0.0353773	+	T	C	0.6729	0.0407
rs5757654	LC_IGP_R11	IgG1_G0FN/IgG1_G0F	22	39794124	4	9.17E-27	3.69E-20	-	-0.385669	-0.3821	+	A	G	0.632936242	0.0353769	+	A	G	0.6493	0.0415
rs7292066	LC_IGP_R11	IgG1_G0FN/IgG1_G0F	22	39794241	4	1.05E-26	1.05E-20	-	-0.384804	-0.3788	+	A	G	0.63528737	0.0353418	+	A	G	0.6764	0.0406
rs5750813	LC_IGP_R11	IgG1_G0FN/IgG1_G0F	22	39795228	4	7.80E-27	8.80E-21	-	-0.386309	-0.3787	+	C	G	0.636499085	0.0353843	+	C	G	0.6796	0.0405
rs4821892	LC_IGP_R11	IgG1_G0FN/IgG1_G0F	22	39795683	4	1.15E-26	8.86E-21	-	-0.384721	-0.3784	+	C	G	0.637007627	0.0353609	+	C	G	0.6799	0.0405
rs5757655	LC_IGP_R11	IgG1_G0FN/IgG1_G0F	22	39797178	4	1.78E-26	8.09E-21	-	-0.381449	-0.38	+	C	G	0.630937157	0.0351986	+	C	G	0.6737	0.0406
rs4821893	LC_IGP_R11	IgG1_G0FN/IgG1_G0F	22	39797779	4	3.36E-26	7.41E-21	-	-0.379262	-0.3782	+	A	G	0.634708969	0.0352008	+	A	G	0.6796	0.0404
rs5750814	LC_IGP_R11	IgG1_G0FN/IgG1_G0F	22	39797987	4	5.34E-26	7.46E-21	-	-0.377728	-0.3805	+	C	T	0.631031117	0.0352076	+	C	T	0.6729	0.0406
rs5757657	LC_IGP_R11	IgG1_G0FN/IgG1_G0F	22	39798429	4	5.31E-26	7.43E-21	-	-0.377734	-0.3805	+	T	G	0.631033557	0.0352064	+	T	G	0.6729	0.0406
rs5750815	LC_IGP_R11	IgG1_G0FN/IgG1_G0F	22	39798449	4	3.24E-26	8.62E-21	-	-0.37906	-0.3774	+	C	T	0.635448444	0.0351699	+	C	T	0.6794	0.0403
rs4429561	LC_IGP_R11	IgG1_G0FN/IgG1_G0F	22	39799789	4	4.14E-26	2.14E-20	-	-0.377969	-0.3842	+	G	A	0.633452715	0.0351476	+	G	A	0.6474	0.0415
rs35509952	LC_IGP_R11	IgG1_G0FN/IgG1_G0F	22	39800563	4	5.09E-26	1.57E-20	-	-0.377754	-0.3781	+	AC	A	0.631052776	0.0351944	+	AC	A	0.6705	0.0407
rs4337572	LC_IGP_R11	IgG1_G0FN/IgG1_G0F	22	39800704	4	5.06E-26	8.43E-21	-	-0.377754	-0.38	+	G	C	0.6310482	0.0351927	+	G	C	0.6732	0.0406
rs6001582	LC_IGP_R11	IgG1_G0FN/IgG1_G0F	22	39806153	4	1.88E-25	1.42E-20	+	0.374073	0.3762	+	C	T	0.364817572	0.0352778	+	C	T	0.3245	0.0405
rs4821894	LC_IGP_R11	IgG1_G0FN/IgG1_G0F	22	39809820	4	4.29E-26	1.20E-20	-	-0.379087	-0.3758	+	C	G	0.637207749	0.0352627	+	C	G	0.6803	0.0403
rs5750816	LC_IGP_R11	IgG1_G0FN/IgG1_G0F	22	39810379	4	5.37E-26	1.48E-20	-	-0.376795	-0.3756	+	T	C	0.633503356	0.0351221	+	T	C	0.678	0.0404
rs5757659	LC_IGP_R11	IgG1_G0FN/IgG1_G0F	22	39812409	4	4.61E-26	1.20E-20	-	-0.377599	-0.3758	+	G	T	0.635753508	0.0351479	+	G	T	0.6803	0.0403
rs6001585	LC_IGP_R11	IgG1_G0FN/IgG1_G0F	22	39812986	4	3.58E-21	1.55E-16	-	-0.372688	-0.369	+	A	C	0.744356315	0.0389191	+	A	C	0.7832	0.0447
rs6001587	LC_IGP_R11	IgG1_G0FN/IgG1_G0F	22	39819008	4	5.20E-26	1.46E-20	-	-0.376937	-0.3849	+	C	T	0.63526083	0.0351251	+	C	T	0.6569	0.0414
rs6001588	LC_IGP_R11	IgG1_G0FN/IgG1_G0F	22	39819049	4	5.53E-26	1.13E-20	-	-0.377364	-0.3766	+	C	G	0.635204393	0.0351846	+	C	G	0.6796	0.0404
rs7288760	LC_IGP_R11	IgG1_G0FN/IgG1_G0F	22	39819969	4	8.35E-27	8.76E-20	-	-0.390872	-0.3779	+	G	A	0.641304454	0.035824	+	G	A	0.6705	0.0415
rs5750818	LC_IGP_R11	IgG1_G0FN/IgG1_G0F	22	39820885	4	4.08E-26	1.55E-20	-	-0.377339	-0.3755	+	G	A	0.634838316	0.0350843	+	G	A	0.6782	0.0404
rs5757663	LC_IGP_R11	IgG1_G0FN/IgG1_G0F	22	39821319	4	4.61E-26	1.24E-20	-	-0.377616	-0.3755	+	C	T	0.635743746	0.0351494	+	C	T	0.6793	0.0403
rs5757664	LC_IGP_R11	IgG1_G0FN/IgG1_G0F	22	39821536	4	4.07E-26	1.54E-20	-	-0.377362	-0.3755	+	G	A	0.634835265	0.0350854	+	G	A	0.6782	0.0404
rs5757665	LC_IGP_R11	IgG1_G0FN/IgG1_G0F	22	39821641	4	4.58E-26	1.18E-20	-	-0.377633	-0.376	+	G	A	0.635749542	0.0351492	+	G	A	0.6803	0.0403
rs5757667	LC_IGP_R11	IgG1_G0FN/IgG1_G0F	22	39822116	4	1.21E-26	1.68E-20	-	-0.382485	-0.3851	+	G	A	0.624489018	0.0351731	+	G	A	0.6533	0.0415
rs4821895	LC_IGP_R11	IgG1_G0FN/IgG1_G0F	22	39823015	4	3.31E-26	1.21E-20	-	-0.379119	-0.3776	+	G	A	0.632880496	0.0351824	+	G	A	0.6777	0.0405
rs739141	LC_IGP_R11	IgG1_G0FN/IgG1_G0F	22	39824450	4	2.46E-20	8.86E-17	-	-0.332152	-0.3337	+	T	C	0.602684626	0.0354724	+	T	C	0.6369	0.0401
rs743838	LC_IGP_R11	IgG1_G0FN/IgG1_G0F	22	39824707	4	1.56E-25	8.74E-21	-	-0.375495	-0.3791	+	G	T	0.637717511	0.0353498	+	G	T	0.6805	0.0406
rs5750820	LC_IGP_R11	IgG1_G0FN/IgG1_G0F	22	39825322	4	2.56E-26	1.12E-20	-	-0.381049	-0.379	+	G	T	0.633296827	0.0352785	+	G	T	0.6781	0.0406
rs5750821	LC_IGP_R11	IgG1_G0FN/IgG1_G0F	22	39825492	4	2.42E-26	1.05E-20	-	-0.380923	-0.3794	+	T	A	0.633002441	0.0352491	+	T	A	0.678	0.0407
rs5750822	LC_IGP_R11	IgG1_G0FN/IgG1_G0F	22	39826788	4	3.12E-26	9.95E-21	-	-0.379974	-0.3797	+	A	G	0.633397193	0.035243	+	A	G	0.678	0.0407
rs7949	LC_IGP_R11	IgG1_G0FN/IgG1_G0F	22	39827553	4	2.12E-26	9.65E-21	-	-0.381611	-0.3801	+	A	G	0.632858755	0.0352694	+	A	G	0.6778	0.0407
rs5757670	LC_IGP_R11	IgG1_G0FN/IgG1_G0F	22	39829736	4	2.29E-26	1.76E-20	-	-0.38195	-0.3894	+	A	G	0.630845333	0.0353267	+	A	G	0.6513	0.042
rs5750823	LC_IGP_R11	IgG1_G0FN/IgG1_G0F	22	39829973	4	5.23E-30	1.17E-24	-	-0.417525	-0.4325	+	T	C	0.672593045	0.0359591	+	T	C	0.7181	0.0422
rs5750825	LC_IGP_R11	IgG1_G0FN/IgG1_G0F	22	39831278	4	1.26E-29	1.08E-24	-	-0.416093	-0.4336	+	G	A	0.673424039	0.0360889	+	G	A	0.7182	0.0423
rs1972280	LC_IGP_R11	IgG1_G0FN/IgG1_G0F	22	39831986	4	4.57E-30	1.84E-24	-	-0.418268	-0.4404	+	A	T	0.67073734	0.035984	+	A	T	0.6934	0.0432
rs5750826	LC_IGP_R11	IgG1_G0FN/IgG1_G0F	22	39832113	4	1.29E-18	1.26E-16	-	-0.316359	-0.3307	+	G	A	0.534676022	0.0354936	+	G	A	0.5447	0.04
rs4821896	LC_IGP_R11	IgG1_G0FN/IgG1_G0F	22	39833437	4	4.55E-30	1.60E-23	-	-0.418605	-0.4318	+	C	T	0.67195078	0.0360121	+	C	T	0.6945	0.0432
rs73167342	LC_IGP_R11	IgG1_G0FN/IgG1_G0F	22	39834102	4	5.18E-31	5.07E-25	-	-0.426864	-0.4467	+	G	C	0.662517999	0.0361028	+	G	C	0.6853	0.0432
rs5750828	LC_IGP_R11	IgG1_G0FN/IgG1_G0F	22	39835083	4	3.66E-31	6.66E-26	-	-0.4282	-0.4527	+	C	T	0.659293167	0.0361191	+	C	T	0.697	0.043
rs4821897	LC_IGP_R11	IgG1_G0FN/IgG1_G0F	22	39835587	4	5.93E-30	1.50E-24	-	-0.417504	-0.4353	+	G	A	0.674044539	0.035993	+	G	A	0.7207	0.0426
rs10644269	LC_IGP_R11	IgG1_G0FN/IgG1_G0F	22	39836972	4	6.73E-30	2.78E-25	-	-0.417938	-0.4397	+	CTT	C	0.670996949	0.0360672	+	CTT	C	0.7136	0.0423
rs6001594	LC_IGP_R11	IgG1_G0FN/IgG1_G0F	22	39837472	4	5.24E-30	1.64E-24	-	-0.41837	-0.4358	+	T	C	0.673210799	0.0360321	+	T	C	0.7194	0.0426
rs2899318	LC_IGP_R11	IgG1_G0FN/IgG1_G0F	22	39837625	4	5.44E-30	1.66E-24	-	-0.418247	-0.4359	+	G	A	0.67316443	0.0360321	+	G	A	0.7194	0.0427
rs5757673	LC_IGP_R11	IgG1_G0FN/IgG1_G0F	22	39837920	4	2.75E-30	1.12E-24	-	-0.420896	-0.4469	+	C	T	0.67116443	0.0360648	+	C	T	0.6948	0.0436
rs9611176	LC_IGP_R11	IgG1_G0FN/IgG1_G0F	22	39838003	4	2.31E-30	2.57E-25	-	-0.422278	-0.4445	+	G	A	0.672186089	0.0361339	+	G	A	0.7147	0.0428
rs5750829	LC_IGP_R11	IgG1_G0FN/IgG1_G0F	22	39838018	4	2.29E-30	1.70E-23	-	-0.421428	-0.4395	+	G	A	0.669838316	0.0360578	+	G	A	0.6816	0.044
rs4821898	LC_IGP_R11	IgG1_G0FN/IgG1_G0F	22	39838352	4	2.40E-30	4.64E-24	-	-0.421097	-0.4358	+	T	C	0.672802929	0.0360427	+	T	C	0.7209	0.0431
rs5757675	LC_IGP_R11	IgG1_G0FN/IgG1_G0F	22	39838892	4	2.49E-30	1.20E-24	-	-0.421186	-0.4385	+	T	G	0.670128432	0.0360608	+	T	G	0.7151	0.0428
rs6001595	LC_IGP_R11	IgG1_G0FN/IgG1_G0F	22	39839293	4	1.28E-30	7.06E-25	-	-0.42546	-0.4514	+	T	C	0.668755644	0.0362385	+	T	C	0.6911	0.0438
rs8138462	LC_IGP_R11	IgG1_G0FN/IgG1_G0F	22	39839670	4	2.50E-30	1.17E-24	-	-0.421236	-0.439	+	C	G	0.67009457	0.0360664	+	C	G	0.7151	0.0428
rs9611177	LC_IGP_R11	IgG1_G0FN/IgG1_G0F	22	39840130	4	3.27E-13	4.09E-10	-	-0.280969	-0.2511	+	T	C	0.376544539	0.0382619	+	T	C	0.4109	0.0402
rs5750830	LC_IGP_R11	IgG1_G0FN/IgG1_G0F	22	39840828	4	5.98E-31	2.37E-24	-	-0.426692	-0.4427	+	A	C	0.678398414	0.0361283	+	A	C	0.73	0.0435
rs28685576	LC_IGP_R11	IgG1_G0FN/IgG1_G0F	22	39841533	4	6.57E-20	4.22E-16	-	-0.327402	-0.3255	+	A	C	0.535545149	0.035381	+	A	C	0.5638	0.04
rs5757676	LC_IGP_R11	IgG1_G0FN/IgG1_G0F	22	39841700	4	6.33E-22	9.67E-17	-	-0.385964	-0.3905	+	C	T	0.749258999	0.0395323	+	C	T	0.7901	0.047
rs7364148	LC_IGP_R11	IgG1_G0FN/IgG1_G0F	22	39842165	4	1.41E-30	2.80E-24	-	-0.425532	-0.441	+	C	T	0.679300793	0.0362716	+	C	T	0.7301	0.0434
rs5750833	LC_IGP_R11	IgG1_G0FN/IgG1_G0F	22	39843091	4	7.69E-31	2.41E-24	-	-0.426967	-0.4419	+	A	C	0.675612874	0.0362221	+	A	C	0.7264	0.0434
rs5757678	LC_IGP_R11	IgG1_G0FN/IgG1_G0F	22	39843409	4	2.99E-31	1.96E-24	-	-0.428631	-0.4425	+	T	C	0.674742831	0.0360998	+	T	C	0.7263	0.0434
rs9306335	LC_IGP_R11	IgG1_G0FN/IgG1_G0F	22	39843537	4	1.41E-30	6.52E-24	-	-0.425566	-0.4407	+	C	T	0.679273948	0.0362737	+	C	T	0.7218	0.0437
rs8137426	LC_IGP_R11	IgG1_G0FN/IgG1_G0F	22	39844350	4	1.41E-30	3.03E-24	-	-0.425574	-0.4412	+	G	T	0.679257779	0.0362747	+	G	T	0.7302	0.0434
rs8136980	LC_IGP_R11	IgG1_G0FN/IgG1_G0F	22	39844574	4	1.06E-30	3.15E-24	-	-0.425967	-0.4419	+	A	G	0.674505385	0.0362269	+	A	G	0.7232	0.0435

rs5757680	LC_IGP_R11	IgG1_G0FN/IgG1_G0F	22	39844793	4	1.32E-30	2.89E-24	-	-0.426159	-0.4416	+	C	T	0.678321538	0.0363051	+	C	T	0.7299	0.0434
rs5757681	LC_IGP_R11	IgG1_G0FN/IgG1_G0F	22	39845547	4	5.25E-31	2.04E-24	-	-0.429338	-0.4423	+	G	A	0.678251482	0.0363159	+	G	A	0.729	0.0434
rs1005522	LC_IGP_R11	IgG1_G0FN/IgG1_G0F	22	39845898	4	3.59E-31	2.46E-24	-	-0.444858	-0.4478	+	A	C	0.700887431	0.0375185	+	A	C	0.7434	0.044
rs5757682	LC_IGP_R11	IgG1_G0FN/IgG1_G0F	22	39848259	4	3.22E-30	2.25E-24	-	-0.42391	-0.4444	+	G	T	0.681310555	0.0363687	+	G	T	0.7305	0.0436
rs5757683	LC_IGP_R11	IgG1_G0FN/IgG1_G0F	22	39850174	4	4.08E-30	1.83E-24	-	-0.421904	-0.4461	+	A	G	0.676438574	0.0362647	+	A	G	0.7252	0.0437
rs5757684	LC_IGP_R11	IgG1_G0FN/IgG1_G0F	22	39851584	4	4.91E-30	1.46E-24	-	-0.421118	-0.4516	+	A	G	0.675863529	0.0362501	+	A	G	0.7252	0.0441
rs1557541	LC_IGP_R11	IgG1_G0FN/IgG1_G0F	22	39851970	4	5.41E-30	1.40E-24	-	-0.420657	-0.4529	+	A	C	0.67571202	0.0362383	+	A	C	0.7252	0.0442
rs1557542	LC_IGP_R11	IgG1_G0FN/IgG1_G0F	22	39852350	4	5.98E-30	1.38E-24	-	-0.42009	-0.4543	+	C	G	0.675515253	0.0362186	+	C	G	0.7252	0.0444
rs1557543	LC_IGP_R11	IgG1_G0FN/IgG1_G0F	22	39852648	4	2.59E-30	8.81E-25	-	-0.424327	-0.4552	+	T	C	0.679792556	0.0363413	+	T	C	0.7284	0.0443
rs6001599	LC_IGP_R11	IgG1_G0FN/IgG1_G0F	22	39852720	4	4.61E-30	1.22E-24	-	-0.422328	-0.4549	+	G	A	0.68035784	0.0363362	+	G	A	0.7294	0.0444
rs6001600	LC_IGP_R11	IgG1_G0FN/IgG1_G0F	22	39852921	4	6.30E-30	1.07E-24	-	-0.419821	-0.456	+	T	G	0.675449252	0.0362103	+	T	G	0.7246	0.0444
rs113200473	LC_IGP_R11	IgG1_G0FN/IgG1_G0F	22	39853740	4	2.48E-30	2.14E-22	-	-0.424478	-0.4371	+	A	G	0.678557352	0.0363417	+	A	G	0.7005	0.0449
rs5995735	LC_IGP_R11	IgG1_G0FN/IgG1_G0F	22	39854421	4	2.10E-30	8.52E-25	-	-0.424729	-0.4577	+	G	C	0.678995119	0.0363166	+	G	C	0.7285	0.0445
rs5757685	LC_IGP_R11	IgG1_G0FN/IgG1_G0F	22	39855540	4	3.71E-30	8.52E-25	-	-0.422423	-0.459	+	C	G	0.679400854	0.0362812	+	C	G	0.7286	0.0446
rs738286	LC_IGP_R11	IgG1_G0FN/IgG1_G0F	22	39855575	4	4.44E-30	1.24E-24	-	-0.420943	-0.4583	+	A	C	0.676006711	0.0362061	+	A	C	0.7256	0.0447
rs738287	LC_IGP_R11	IgG1_G0FN/IgG1_G0F	22	39855728	4	3.80E-30	8.32E-25	-	-0.422266	-0.4592	+	C	G	0.679360891	0.0362747	+	C	G	0.7287	0.0447
rs738289	LC_IGP_R11	IgG1_G0FN/IgG1_G0F	22	39855883	4	3.90E-30	8.37E-25	-	-0.42212	-0.4594	+	T	C	0.679339536	0.0362701	+	T	C	0.7287	0.0447
rs738290	LC_IGP_R11	IgG1_G0FN/IgG1_G0F	22	39856032	4	1.66E-22	4.68E-17	-	-0.344078	-0.3457	+	C	A	0.577181208	0.0347342	+	C	A	0.6048	0.0412
rs738285	LC_IGP_R11	IgG1_G0FN/IgG1_G0F	22	39856356	4	3.97E-30	8.26E-25	-	-0.422362	-0.46	+	C	G	0.679362721	0.0362961	+	C	G	0.7287	0.0447
rs2413592	LC_IGP_R11	IgG1_G0FN/IgG1_G0F	22	39858196	4	1.16E-29	2.86E-24	-	-0.421539	-0.4578	+	G	A	0.677890373	0.0365383	+	G	A	0.7239	0.045
rs909674	LC_IGP_R11	IgG1_G0FN/IgG1_G0F	22	39859169	4	1.96E-28	7.00E-24	-	-0.41404	-0.4562	+	A	C	0.670655583	0.0367324	+	A	C	0.7201	0.0453
rs2008174	LC_IGP_R11	IgG1_G0FN/IgG1_G0F	22	39860130	4	1.95E-28	1.63E-23	-	-0.415944	-0.4539	+	T	C	0.670826619	0.0369004	+	T	C	0.7191	0.0454
rs3959642	LC_IGP_R11	IgG1_G0FN/IgG1_G0F	22	39860589	4	1.96E-28	1.89E-23	-	-0.416812	-0.4532	+	G	A	0.670909701	0.0369779	+	G	A	0.7188	0.0454
rs7286917	LC_IGP_R11	IgG1_G0FN/IgG1_G0F	22	39860868	4	2.62E-28	8.03E-23	-	-0.416812	-0.4498	+	G	A	0.671125181	0.0370695	+	G	A	0.7161	0.0457
rs5757642	LC_IGP_R12	IgG1_G1FN/IgG1_G1F	22	39764824	4	2.76E-09	1.55E-08	-	-0.214486	-0.2151	+	C	T	0.608340965	0.035878	+	C	T	0.643	0.038
rs7286714	LC_IGP_R12	IgG1_G1FN/IgG1_G1F	22	39765604	4	4.08E-09	1.02E-08	-	-0.211763	-0.2204	+	G	C	0.601703297	0.035813	+	G	C	0.6329	0.0385
rs5757644	LC_IGP_R12	IgG1_G1FN/IgG1_G1F	22	39766440	4	3.30E-09	9.70E-09	-	-0.213156	-0.221	+	G	C	0.601777778	0.0358328	+	G	C	0.6329	0.0385
rs5750806	LC_IGP_R12	IgG1_G1FN/IgG1_G1F	22	39767011	4	3.11E-09	9.65E-09	-	-0.213533	-0.221	+	G	A	0.60180763	0.0358372	+	G	A	0.633	0.0385
rs9611162	LC_IGP_R12	IgG1_G1FN/IgG1_G1F	22	39767247	4	3.06E-09	9.74E-09	-	-0.213644	-0.2211	+	A	C	0.601802808	0.035839	+	A	C	0.633	0.0385
rs9611164	LC_IGP_R12	IgG1_G1FN/IgG1_G1F	22	39767291	4	3.04E-09	7.45E-09	-	-0.213791	-0.2245	+	A	G	0.601560134	0.0358554	+	A	G	0.6248	0.0388
rs5757645	LC_IGP_R12	IgG1_G1FN/IgG1_G1F	22	39768479	4	2.70E-09	9.12E-09	-	-0.214453	-0.2217	+	A	G	0.601854701	0.03585	+	A	G	0.6328	0.0386
rs1569499	LC_IGP_R12	IgG1_G1FN/IgG1_G1F	22	39769818	4	1.43E-09	7.86E-09	-	-0.218877	-0.2216	+	C	T	0.606626374	0.0359602	+	C	T	0.64	0.0384
rs4821888	LC_IGP_R12	IgG1_G1FN/IgG1_G1F	22	39770597	4	1.15E-09	9.57E-09	-	-0.220208	-0.2203	+	G	A	0.607507086	0.0359659	+	G	A	0.6413	0.0384
rs4820377	LC_IGP_R12	IgG1_G1FN/IgG1_G1F	22	39770780	4	1.64E-09	8.21E-09	-	-0.218177	-0.2207	+	A	G	0.607963741	0.0359748	+	A	G	0.6422	0.0383
rs6001566	LC_IGP_R12	IgG1_G1FN/IgG1_G1F	22	39774448	4	1.87E-12	3.99E-10	-	-0.256569	-0.25	+	G	A	0.627045482	0.0361437	+	G	A	0.6567	0.04
rs6519190	LC_IGP_R12	IgG1_G1FN/IgG1_G1F	22	39774525	4	1.99E-12	2.45E-10	-	-0.256049	-0.253	+	G	A	0.627180175	0.0361167	+	G	A	0.6569	0.04
rs5757647	LC_IGP_R12	IgG1_G1FN/IgG1_G1F	22	39775047	4	2.79E-12	3.54E-10	-	-0.254638	-0.249	+	A	C	0.629329365	0.0361636	+	A	C	0.6616	0.0397
rs5757648	LC_IGP_R12	IgG1_G1FN/IgG1_G1F	22	39775156	4	2.75E-12	4.28E-10	-	-0.254702	-0.2479	+	A	G	0.629333944	0.0361623	+	A	G	0.6609	0.0397
rs9611165	LC_IGP_R12	IgG1_G1FN/IgG1_G1F	22	39775250	4	2.72E-12	3.52E-10	-	-0.25476	-0.2491	+	G	A	0.629340049	0.036161	+	G	A	0.6616	0.0397
rs9611166	LC_IGP_R12	IgG1_G1FN/IgG1_G1F	22	39775268	4	2.72E-12	3.52E-10	-	-0.254763	-0.2491	+	C	T	0.629339438	0.0361607	+	C	T	0.6616	0.0397
rs6001567	LC_IGP_R12	IgG1_G1FN/IgG1_G1F	22	39775400	4	2.67E-12	3.51E-10	-	-0.25483	-0.2491	+	T	A	0.629344628	0.0361588	+	T	A	0.6616	0.0397
rs9611167	LC_IGP_R12	IgG1_G1FN/IgG1_G1F	22	39775583	4	2.51E-12	4.92E-10	-	-0.254943	-0.2484	+	A	G	0.625077534	0.0361289	+	A	G	0.6543	0.0399
rs6001568	LC_IGP_R12	IgG1_G1FN/IgG1_G1F	22	39775786	4	1.71E-12	4.52E-10	-	-0.25666	-0.2467	+	A	G	0.630831197	0.0360915	+	A	G	0.6632	0.0396
rs4821889	LC_IGP_R12	IgG1_G1FN/IgG1_G1F	22	39777254	4	2.11E-12	3.86E-10	-	-0.255325	-0.2502	+	C	T	0.625579365	0.036057	+	C	T	0.654	0.04
rs4821890	LC_IGP_R12	IgG1_G1FN/IgG1_G1F	22	39777523	4	2.04E-12	3.74E-10	-	-0.255476	-0.2503	+	A	G	0.625596764	0.0360529	+	A	G	0.6541	0.04
rs1010169	LC_IGP_R12	IgG1_G1FN/IgG1_G1F	22	39778167	4	1.74E-12	4.42E-10	-	-0.256271	-0.2468	+	G	A	0.631043346	0.0360484	+	G	A	0.6636	0.0396
rs1010170	LC_IGP_R12	IgG1_G1FN/IgG1_G1F	22	39778327	4	1.71E-12	3.57E-10	-	-0.256351	-0.2484	+	C	T	0.631041209	0.0360459	+	C	T	0.6629	0.0396
rs5757650	LC_IGP_R12	IgG1_G1FN/IgG1_G1F	22	39778419	4	3.03E-12	3.73E-10	-	-0.253276	-0.2497	+	C	T	0.628404151	0.0360293	+	C	T	0.6583	0.0398
rs756640	LC_IGP_R12	IgG1_G1FN/IgG1_G1F	22	39779300	4	1.63E-12	3.86E-10	-	-0.256399	-0.2501	+	A	G	0.625666667	0.0360193	+	A	G	0.654	0.04
rs7423	LC_IGP_R12	IgG1_G1FN/IgG1_G1F	22	39781429	4	1.15E-12	3.78E-10	-	-0.258072	-0.2503	+	T	C	0.62621024	0.0360076	+	T	C	0.6542	0.04
rs1007337	LC_IGP_R12	IgG1_G1FN/IgG1_G1F	22	39781585	4	2.08E-12	3.51E-10	-	-0.254856	-0.2501	+	T	C	0.627954212	0.0359784	+	T	C	0.6587	0.0399
rs5757652	LC_IGP_R12	IgG1_G1FN/IgG1_G1F	22	39781855	4	1.26E-12	3.68E-10	-	-0.256685	-0.2553	+	C	T	0.624732295	0.0358776	+	C	T	0.6322	0.0407
rs9611169	LC_IGP_R12	IgG1_G1FN/IgG1_G1F	22	39783027	4	7.27E-13	4.50E-10	-	-0.259482	-0.2466	+	T	C	0.632094933	0.0358787	+	T	C	0.664	0.0395
rs9611170	LC_IGP_R12	IgG1_G1FN/IgG1_G1F	22	39784845	4	2.79E-13	1.57E-11	-	-0.264092	-0.2728	+	C	G	0.628916056	0.0358555	+	C	G	0.6725	0.0405
rs2142848	LC_IGP_R12	IgG1_G1FN/IgG1_G1F	22	39785242	4	4.92E-13	1.58E-11	-	-0.26131	-0.2726	+	T	C	0.630372711	0.0358613	+	T	C	0.6717	0.0404
rs4821891	LC_IGP_R12	IgG1_G1FN/IgG1_G1F	22	39785381	4	4.92E-13	1.90E-11	-	-0.261311	-0.2719	+	T	C	0.630373321	0.0358611	+	T	C	0.671	0.0405
rs2413590	LC_IGP_R12	IgG1_G1FN/IgG1_G1F	22	39790191	4	3.65E-13	2.36E-11	-	-0.263772	-0.2692	+	C	G	0.637078144	0.0359935	+	C	G	0.6799	0.0403
rs5750808	LC_IGP_R12	IgG1_G1FN/IgG1_G1F	22	39790987	4	3.63E-13	2.36E-11	-	-0.263784	-0.2692	+	A	G	0.637079976	0.0359921	+	A	G	0.6799	0.0403
rs5750809	LC_IGP_R12	IgG1_G1FN/IgG1_G1F	22	39791491	4	1.16E-12	1.21E-11	-	-0.258038	-0.2744	+	G	T	0.634508852	0.0360087	+	G	T	0.6727	0.0405
rs5750810	LC_IGP_R12	IgG1_G1FN/IgG1_G1F	22	39792943	4	3.60E-13	2.36E-11	-	-0.263806	-0.2693	+	G	A	0.637086386	0.0359888	+	G	A	0.6799	0.0403
rs5750811	LC_IGP_R12	IgG1_G1FN/IgG1_G1F	22	39793066	4	3.60E-13	2.36E-11	-	-0.263804	-0.2692	+	G	T	0.637086386	0.0359886	+	G	T	0.6799	0.0403
rs5750812	LC_IGP_R12	IgG1_G1FN/IgG1_G1F	22	39793079	4	3.92E-13	2.10E-11	-	-0.263517	-0.2716	+	A	G	0.633026557	0.0360088	+	A	G	0.6727	0.0405
rs4321460	LC_IGP_R12	IgG1_G1FN/IgG1_G1F	22	39793655	4	6.02E-13	2.25E-09	-	-0.261581	-0.2569	+	G	A	0.635484127	0.0360375	+	G	A	0.6362	0.043
rs4386422	LC_IGP_R12	IgG1_G1FN/IgG1_G1F	22	39793734	4	7.96E-13	2.62E-11	-	-0.262295	-0.2769	+	T	C	0.628084249	0.0363314	+	T	C	0.6293	0.0415
rs4384886	LC_IGP_R12	IgG1_G1FN/IgG1_G1F	22	39793766	4	3.90E-13	2.00E-11	-	-0.26354	-0.2719	+	T	C	0.633029304	0.0360076	+	T	C	0.6729	0.0406
rs5757654	LC_IGP_R12	IgG1_G1FN/IgG1_G1F	22	39794124	4	3.90E-13	2.97E-11	-	-0.263541	-0.2754	+	A	G	0.633014957	0.0360071	+	A	G	0.6493	0.0414
rs7292066	LC_IGP_R12	IgG1_G1FN/IgG1_G1F	22	39794241	4	5.15E-13	2.21E-11	-	-0.261907	-0.2705	+	A	G	0.635367521	0.0359744	+	A	G	0.6764	0.0404
rs5750813	LC_IGP_R12	IgG1_G1FN/IgG1_G1F	22	39795228	4	2.90E-13	2.38E-11	-	-0.26504	-0.2692	+	C	G	0.636579976	0.0360119	+	C	G	0.6796	0.0403
rs4821892	LC_IGP_R12	IgG1_G1FN/IgG1_G1F	22	39795683	4	3.55E-13	2.44E-11	-	-0.263838	-0.2689	+	C	G	0.637088828	0.0359841	+	C	G	0.6799	0.0403
rs5757655	LC_IGP_R12	IgG1_G1FN/IgG1_G1F	22	39797178	4	4.58E-13	2.39E-11	-	-0.261327	-0.2699	+	C	G	0.631017094	0.0358148	+	C	G	0.6737	0.0404
rs4821893	LC_IGP_R12	IgG1_G1FN/IgG1_G1F	22	39797779	4	5.94E-13	2.19E-11	-	-0.259984	-0.2688	+	A	G	0.634791209	0.0358089	+	A	G	0.6796	0.0402
rs5750814	LC_IGP_R12	IgG1_G1FN/IgG1_G1F	22	39797987	4	1.11E-12	1.71E-11	-	-0.256934	-0.272	+	C	T	0.631111111	0.0358191	+	C	T	0.6729	0.0404
rs5757657	LC_IGP_R12	IgG1_G1FN/IgG1_G1F	22	39798429	4	1.10E-12	1.70E-11	-	-0.256948	-0.272	+	T	G	0.631113553	0.0358178	+	T	G	0.6729	0.0404
rs5750815	LC_IGP_R12	IgG1_G1FN/IgG1_G1F	22	39798449	4	4.94E-13	2.32E-11	-	-0.26066	-0.2683	+	C	T	0.635531136	0.0357743	+	C	T	0.6794	0.0401
rs4429561	LC_IGP_R12	IgG1_G1FN/IgG1_G1F	22	39799789	4	9.72E-13	9.77E-12	-	-0.257168	-0.2819	+	G	A	0.633534188	0.0357608	+	G	A	0.6474	0.0414
rs35509952	LC_IGP_R12	IgG1_G1FN/IgG1_G1F	22	39800563	4	1.06E-12	2.48E-11	-	-0.257064	-0.2704	+	AC	A	0.631132784	0.0358057	+	AC	A	0.6705	0.0405
rs4337572	LC_IGP_R12	IgG1_G1FN/IgG1_G1F	22	39800704	4	1.05E-12	1.93E-11	-	-0.257072	-0.2713	+	G	C	0.631128205	0.035804	+	G	C	0.6732	0.0404
rs6001582	LC_IGP_R12	IgG1_G1FN/IgG1_G1F	22	39806153	4	1.25E-12	2.76E-11	+	0.256638	0.2682	+	C	T	0.364737179	0.0358658	+	C	T	0.3245	0.0403
rs4821894	LC_IGP_R12	IgG1_G1FN/IgG1_G1F	22	39809820	4	4.96E-13	3.12E-11	-	-0.26127	-0.2664	+	C	G	0.63726221	0.0358618	+	C	G	0.6803	0.0401
rs5750816	LC_IGP_R12	IgG1_G1FN/IgG1_G1F	22	39810379	4	9.43E-13	3.67E-11	-	-0.257092	-0.266	+	T	C	0.63358486	0.0357286	+	T	C	0.678	0.0402
rs5757659	LC_IGP_R12	IgG1_G1FN/IgG1_G1F	22	39812409	4	5.49E-13	3.13E-11	-	-0.259929	-0.2664	+	G	T	0.635836386	0.0357466	+	G	T	0.6803	0.0401
rs6001585	LC_IGP_R12	IgG1_G1FN/IgG1_G1F	22	39812986	4	9.29E-10	5.45E-09	-	-0.243185	-0.2595	+	A	C	0.744200244	0.0394949	+	A	C	0.7832	0.0445
rs6001587	LC_IGP_R12	IgG1_G1FN/IgG1_G1F	22	39819008	4	5.59E-13	2.81E-11	-	-0.25965	-0.2742	+	C	T	0.635343407	0.0357212	+	C	T	0.6569	0.0412
rs6001588	LC_IGP_R12	IgG1_G1FN/IgG1_G1F	22	39819049	4	6.16E-13	2.50E-11	-	-0.259614	-0.2681	+	C	G	0.635289072	0.0357825	+	C	G	0.6796	0.0402
rs7288760	LC_IGP_R12	IgG1_G1FN/IgG1_G1F	22	39819969	4	7.53E-13	1.77E-10	-	-0.263646	-0.2631	+	G	A	0.64139072	0.036479	+	G	A	0.6705	0.0412
rs5750818	LC_IGP_R12	IgG1_G1FN/IgG1_G1F	22	39820885	4	6.76E-13	3.77E-11	-	-0.258473	-0.2659	+	G	A	0.634920635	0.035689	+	G	A	0.6782	0.0402
rs5757663	LC_IGP_R12	IgG1_G1FN/IgG1_G1F	22	39821319	4	5.50E-13	2.76E-11	-	-0.259928	-0.267	+	C	T	0.635826618	0.0357482	+	C	T	0.6793	0.0401
rs5757664	LC_IGP_R12	IgG1_G1FN/IgG1_G1F	22	39821536	4	6.76E-13	3.76E-11	-	-0.258481	-0.266	+	G	A	0.634917582	0.0356901	+	G	A	0.6782	0.0402
rs5757665	LC_IGP_R12	IgG1_G1FN/IgG1_G1F	22	39821641	4	5.49E-13	3.06E-11	-	-0.259943	-0.2666	+	G	A	0.635832418	0.035748	+	G	A	0.6803	0.0401
rs5757667	LC_IGP_R12	IgG1_G1FN/IgG1_G1F	22	39822116	4	4.38E-13	3.08E-11	-	-0.261414	-0.2743	+	G	A	0.624565018	0.035796	+	G	A	0.6533	0.0413
rs4821895	LC_IGP_R12	IgG1_G1FN/IgG1_G1F	22	39823015	4	7.65E-13	3.06E-11	-	-0.258625	-0.2679	+	G	A	0.63296162	0.0357959	+	G	A	0.6777	0.0403
rs739141	LC_IGP_R12	IgG1_G1FN/IgG1_G1F	22	39824450	4	1.44E-09	6.44E-09	-	-0.218819	-0.2313	+	T	C	0.602739074	0.035958	+	T	C	0.6369	0.0398
rs743838	LC_IGP_R12	IgG1_G1FN/IgG1_G1F	22	39824707	4	1.43E-12	2.29E-11	-	-0.256528	-0.2696	+	G	T	0.637801587	0.0359462	+	G	T	0.6805	0.0403
rs5750820	LC_IGP_R12	IgG1_G1FN/IgG1_G1F	22	39825322	4	5.81E-13	2.73E-11	-	-0.260709	-0.2694	+	G	T	0.633378205	0.0358934	+	G	T	0.6781	0.0404
rs5750821	LC_IGP_R12	IgG1_G1FN/IgG1_G1F	22	39825492	4	6.38E-13	2.60E-11	-	-0.260053	-0.2697	+	T	A	0.633083639	0.0358667	+	T	A	0.678	0.0404
rs5750822	LC_IGP_R12	IgG1_G1FN/IgG1_G1F	22	39826788	4	7.45E-13	2.44E-11	-	-0.259213	-0.2701	+	A	G	0.633478632	0.0358583	+	A	G	0.678	0.0404
rs7949	LC_IGP_R12	IgG1_G1FN/IgG1_G1F	22	39827553	4	6.13E-13	2.39E-11	-	-0.260414	-0.2704	+	A	G	0.632939866	0.0358894	+	A	G	0.6778	0.0405
rs5757670	LC_IGP_R12	IgG1_G1FN/IgG1_G1F	22	39829736	4	8.72E-13	3.92E-11	-	-0.259107	-0.2752	+	A	G	0.630925214	0.0359535	+	A	G	0.6513	0.0416
rs5750823	LC_IGP_R12	IgG1_G1FN/IgG1_G1F	22	39829973	4	1.13E-14	7.38E-14	-	-0.286072	-0.314	+	T	C	0.672393162	0.0366971	+	T	C	0.7181	0.042
rs5750825	LC_IGP_R12	IgG1_G1FN/IgG1_G1F	22	39831278	4	1.94E-14	6.69E-14	-	-0.284447	-0.3152	+	G	A	0.673224664	0.0368218	+	G	A	0.7182	0.0421
rs1972280	LC_IGP_R12	IgG1_G1FN/IgG1_G1F	22	39831986	4	1.01E-14	4.84E-14	-	-0.286846	-0.3236	+	A	T	0.670536325	0.0367231	+	A	T	0.6934	0.0429
rs5750826	LC_IGP_R12	IgG1_G1FN/IgG1_G1F	22	39832113	4	5.11E-10	3.16E-08	-	-0.224322	-0.22	+	G	A	0.534749084	0.0358719	+	G	A	0.5447	0.0398
rs4821896	LC_IGP_R12	IgG1_G1FN/IgG1_G1F	22	39833437	4	1.02E-14	1.88E-13	-	-0.286986	-0.3163	+	C	T	0.671750506	0.0367521	+	C	T	0.6945	0.043
rs73167342	LC_IGP_R12	IgG1_G1FN/IgG1_G1F	22	39834102	4	2.58E-15	1.19E-14	-	-0.294426	-0.3325	+	G	C	0.662311966	0.0368635	+	G	C	0.6853	0.0431
rs5750828	LC_IGP_R12	IgG1_G1FN/IgG1_G1F	22	39835083	4	1.37E-15	5.83E-15	-	-0.297488	-0.3351	+	C	T	0.659085165	0.0368741	+	C	T	0.697	0.0429
rs4821897	LC_IGP_R12	IgG1_G1FN/IgG1_G1F	22	39835587	4	1.29E-14	9.65E-14	-	-0.285711	-0.3154	+	G	A	0.673845543	0.0367317	+	G	A	0.7207	0.0424
rs10644269	LC_IGP_R12	IgG1_G1FN/IgG1_G1F	22	39836972	4	1.89E-14	1.27E-14	-	-0.284489	-0.3245	+	CTT	C	0.670796093	0.0368133	+	CTT	C	0.7136	0.0421
rs6001594	LC_IGP_R12	IgG1_G1FN/IgG1_G1F	22	39837472	4	1.27E-14	9.28E-14	-	-0.286134	-0.3163	+	T	C	0.673011294	0.0367741	+	T	C	0.7194	0.0424
rs2899318	LC_IGP_R12	IgG1_G1FN/IgG1_G1F	22	39837625	4	1.31E-14	9.43E-14	-	-0.285985	-0.3162	+	G	A	0.672964896	0.0367739	+	G	A	0.7194	0.0425
rs5757673	LC_IGP_R12	IgG1_G1FN/IgG1_G1F	22	39837920	4	4.55E-15	2.84E-14	-	-0.291233	-0.3295	+	C	T	0.670963675	0.0367993	+	C	T	0.6948	0.0433
rs9611176	LC_IGP_R12	IgG1_G1FN/IgG1_G1F	22	39838003	4	5.17E-15	2.47E-14	-	-0.291238	-0.3241	+	G	A	0.671985958	0.0368765	+	G	A	0.7147	0.0425
rs5750829	LC_IGP_R12	IgG1_G1FN/IgG1_G1F	22	39838018	4	7.02E-15	1.72E-13	-	-0.289219	-0.3228	+	G	A	0.669636752	0.0368059	+	G	A	0.6816	0.0438
rs4821898	LC_IGP_R12	IgG1_G1FN/IgG1_G1F	22	39838352	4	7.05E-15	2.79E-13	-	-0.289069	-0.3134	+	T	C	0.672603175	0.0367894	+	T	C	0.7209	0.0429
rs5757675	LC_IGP_R12	IgG1_G1FN/IgG1_G1F	22	39838892	4	7.07E-15	8.06E-14	-	-0.2892	-0.318	+	T	G	0.669927045	0.0368073	+	T	G	0.7151	0.0426
rs6001595	LC_IGP_R12	IgG1_G1FN/IgG1_G1F	22	39839293	4	3.71E-15	4.16E-14	-	-0.29371	-0.3291	+	T	C	0.668555556	0.0369895	+	T	C	0.6911	0.0436
rs8138462	LC_IGP_R12	IgG1_G1FN/IgG1_G1F	22	39839670	4	7.19E-15	7.86E-14	-	-0.289164	-0.3184	+	C	G	0.669893162	0.0368133	+	C	G	0.7151	0.0426
rs5750830	LC_IGP_R12	IgG1_G1FN/IgG1_G1F	22	39840828	4	3.07E-15	9.42E-14	-	-0.293814	-0.3227	+	A	C	0.678202076	0.0368891	+	A	C	0.73	0.0433
rs28685576	LC_IGP_R12	IgG1_G1FN/IgG1_G1F	22	39841533	4	2.22E-10	4.54E-08	-	-0.228622	-0.2186	+	A	C	0.535619048	0.0358047	+	A	C	0.5638	0.04
rs5757676	LC_IGP_R12	IgG1_G1FN/IgG1_G1F	22	39841700	4	4.60E-10	2.02E-09	-	-0.2517	-0.2809	+	C	T	0.749105922	0.0401423	+	C	T	0.7901	0.0468

rs7364148	LC_IGP_R12	IgG1_G1FN/IgG1_G1F	22	39842165	4	5.42E-15	1.16E-13	-	-0.292214	-0.3207	+	C	T	0.679105006	0.0370287	+	C	T	0.7301	0.0432
rs5750833	LC_IGP_R12	IgG1_G1FN/IgG1_G1F	22	39843091	4	6.91E-15	1.11E-13	-	-0.290802	-0.3216	+	A	C	0.675414835	0.0369975	+	A	C	0.7264	0.0433
rs5757678	LC_IGP_R12	IgG1_G1FN/IgG1_G1F	22	39843409	4	4.06E-15	9.85E-14	-	-0.292429	-0.3222	+	T	C	0.674544261	0.0368818	+	T	C	0.7263	0.0433
rs9306335	LC_IGP_R12	IgG1_G1FN/IgG1_G1F	22	39843537	4	5.46E-15	2.29E-13	-	-0.2922	-0.3194	+	C	T	0.679078144	0.0370311	+	C	T	0.7218	0.0436
rs8137426	LC_IGP_R12	IgG1_G1FN/IgG1_G1F	22	39844350	4	5.48E-15	1.21E-13	-	-0.292184	-0.3209	+	G	T	0.679061966	0.0370322	+	G	T	0.7302	0.0433
rs8136980	LC_IGP_R12	IgG1_G1FN/IgG1_G1F	22	39844574	4	6.71E-15	1.03E-13	-	-0.290917	-0.3228	+	A	G	0.67430667	0.0369946	+	A	G	0.7232	0.0434
rs5757680	LC_IGP_R12	IgG1_G1FN/IgG1_G1F	22	39844793	4	5.02E-15	1.18E-13	-	-0.292847	-0.3212	+	C	T	0.678125153	0.0370629	+	C	T	0.7299	0.0433
rs5757681	LC_IGP_R12	IgG1_G1FN/IgG1_G1F	22	39845547	4	3.85E-15	8.92E-14	-	-0.294326	-0.3223	+	G	A	0.678055054	0.0370887	+	G	A	0.729	0.0432
rs1005522	LC_IGP_R12	IgG1_G1FN/IgG1_G1F	22	39845898	4	2.44E-15	2.73E-14	-	-0.3063	-0.3338	+	A	C	0.700704823	0.0383143	+	A	C	0.7434	0.0439
rs5757682	LC_IGP_R12	IgG1_G1FN/IgG1_G1F	22	39848259	4	1.19E-14	8.86E-14	-	-0.28919	-0.3239	+	G	T	0.681115995	0.0371268	+	G	T	0.7305	0.0434
rs5757683	LC_IGP_R12	IgG1_G1FN/IgG1_G1F	22	39850174	4	2.15E-14	5.85E-14	-	-0.285528	-0.3275	+	A	G	0.67624104	0.0370287	+	A	G	0.7252	0.0436
rs5757684	LC_IGP_R12	IgG1_G1FN/IgG1_G1F	22	39851584	4	2.71E-14	4.54E-14	-	-0.284291	-0.3323	+	A	G	0.675665644	0.0370148	+	A	G	0.7252	0.044
rs1557541	LC_IGP_R12	IgG1_G1FN/IgG1_G1F	22	39851970	4	2.95E-14	4.28E-14	-	-0.283782	-0.3334	+	A	C	0.675514042	0.0370026	+	A	C	0.7252	0.0442
rs1557542	LC_IGP_R12	IgG1_G1FN/IgG1_G1F	22	39852350	4	3.20E-14	4.08E-14	-	-0.283227	-0.3347	+	C	G	0.675317155	0.0369819	+	C	G	0.7252	0.0443
rs1557543	LC_IGP_R12	IgG1_G1FN/IgG1_G1F	22	39852648	4	1.45E-14	2.90E-14	-	-0.288076	-0.3355	+	T	C	0.67959707	0.0371082	+	T	C	0.7284	0.0441
rs6001599	LC_IGP_R12	IgG1_G1FN/IgG1_G1F	22	39852720	4	1.84E-14	3.84E-14	-	-0.286808	-0.3346	+	G	A	0.680162698	0.0370953	+	G	A	0.7294	0.0442
rs6001600	LC_IGP_R12	IgG1_G1FN/IgG1_G1F	22	39852921	4	3.35E-14	3.40E-14	-	-0.282934	-0.3358	+	T	G	0.675251114	0.0369734	+	T	G	0.7246	0.0443
rs113200473	LC_IGP_R12	IgG1_G1FN/IgG1_G1F	22	39853740	4	1.50E-14	1.58E-13	-	-0.287923	-0.33	+	A	G	0.678361111	0.0371105	+	A	G	0.7005	0.0447
rs5995735	LC_IGP_R12	IgG1_G1FN/IgG1_G1F	22	39854421	4	1.38E-14	2.80E-14	-	-0.288158	-0.3374	+	G	C	0.678799145	0.0370865	+	G	C	0.7285	0.0444
rs5757685	LC_IGP_R12	IgG1_G1FN/IgG1_G1F	22	39855540	4	1.91E-14	2.75E-14	-	-0.286243	-0.3385	+	C	G	0.679205128	0.0370449	+	C	G	0.7286	0.0445
rs738286	LC_IGP_R12	IgG1_G1FN/IgG1_G1F	22	39855575	4	2.98E-14	3.66E-14	-	-0.283512	-0.3379	+	A	C	0.675808913	0.0369742	+	A	C	0.7256	0.0446
rs738287	LC_IGP_R12	IgG1_G1FN/IgG1_G1F	22	39855728	4	1.95E-14	2.62E-14	-	-0.286096	-0.3388	+	C	G	0.67916514	0.0370382	+	C	G	0.7287	0.0445
rs738289	LC_IGP_R12	IgG1_G1FN/IgG1_G1F	22	39855883	4	1.98E-14	2.61E-14	-	-0.285965	-0.339	+	T	C	0.679143773	0.0370332	+	T	C	0.7287	0.0445
rs738290	LC_IGP_R12	IgG1_G1FN/IgG1_G1F	22	39856032	4	1.35E-11	2.19E-09	-	-0.239882	-0.2467	+	C	A	0.577228327	0.0352176	+	C	A	0.6048	0.0412
rs738285	LC_IGP_R12	IgG1_G1FN/IgG1_G1F	22	39856356	4	2.03E-14	2.53E-14	-	-0.286047	-0.3395	+	C	G	0.679166972	0.03706	+	C	G	0.7287	0.0446
rs2413592	LC_IGP_R12	IgG1_G1FN/IgG1_G1F	22	39858196	4	3.89E-14	4.69E-14	-	-0.284676	-0.3382	+	G	A	0.677693725	0.0372977	+	G	A	0.7239	0.0448
rs909674	LC_IGP_R12	IgG1_G1FN/IgG1_G1F	22	39859169	4	2.01E-13	2.22E-13	-	-0.27766	-0.3304	+	A	C	0.670456349	0.0374695	+	A	C	0.7201	0.045
rs2008174	LC_IGP_R12	IgG1_G1FN/IgG1_G1F	22	39860130	4	2.12E-13	3.35E-13	-	-0.278666	-0.3291	+	T	C	0.670627796	0.0376422	+	T	C	0.7191	0.0452
rs3959642	LC_IGP_R12	IgG1_G1FN/IgG1_G1F	22	39860589	4	2.18E-13	3.48E-13	-	-0.279103	-0.3288	+	G	A	0.670710928	0.037722	+	G	A	0.7188	0.0452
rs7286917	LC_IGP_R12	IgG1_G1FN/IgG1_G1F	22	39860868	4	2.39E-13	1.17E-12	-	-0.279281	-0.3237	+	G	A	0.670926539	0.0378108	+	G	A	0.7161	0.0455
rs6001595	LC_IGP_R13	IgG1_G2FN/IgG1_G2F	22	39839293	4	2.43E-08	1.81E-11	-	-0.210269	-0.2949	+	T	C	0.668503056	0.037509	+	T	C	0.6911	0.0439
rs1005522	LC_IGP_R13	IgG1_G2FN/IgG1_G2F	22	39845898	4	8.43E-09	4.97E-12	-	-0.224897	-0.3059	+	A	C	0.70064456	0.0388429	+	A	C	0.7434	0.0443
rs1569499	LC_IGP_R51	IgG2_G0FN/IgG2_G0F	22	39769818	4	4.51E-08	1.37E-06	-	-0.198677	-0.1887	+	C	T	0.606561318	0.036151	+	C	T	0.64	0.0391
rs4821888	LC_IGP_R51	IgG2_G0FN/IgG2_G0F	22	39770597	4	4.73E-08	1.59E-06	-	-0.198427	-0.1877	+	G	A	0.607441493	0.0361625	+	G	A	0.6413	0.0391
rs6519190	LC_IGP_R51	IgG2_G0FN/IgG2_G0F	22	39774525	4	1.63E-09	1.70E-06	-	-0.220648	-0.1948	+	G	A	0.627102579	0.0363792	+	G	A	0.6569	0.0407
rs5757647	LC_IGP_R51	IgG2_G0FN/IgG2_G0F	22	39775047	4	1.97E-09	2.26E-06	-	-0.219798	-0.1909	+	A	C	0.629250458	0.036423	+	A	C	0.6616	0.0404
rs9611165	LC_IGP_R51	IgG2_G0FN/IgG2_G0F	22	39775250	4	1.93E-09	2.26E-06	-	-0.219913	-0.1909	+	G	A	0.629261135	0.0364206	+	G	A	0.6616	0.0404
rs9611166	LC_IGP_R51	IgG2_G0FN/IgG2_G0F	22	39775268	4	1.92E-09	2.27E-06	-	-0.219919	-0.1909	+	C	T	0.629260525	0.0364202	+	C	T	0.6616	0.0404
rs6001567	LC_IGP_R51	IgG2_G0FN/IgG2_G0F	22	39775400	4	1.90E-09	2.26E-06	-	-0.219988	-0.1909	+	T	A	0.629265711	0.0364183	+	T	A	0.6616	0.0404
rs4337572	LC_IGP_R51	IgG2_G0FN/IgG2_G0F	22	39800704	4	1.73E-09	2.33E-06	-	-0.21847	-0.1947	+	G	C	0.6310482	0.0360792	+	G	C	0.6732	0.0412
rs5750823	LC_IGP_R51	IgG2_G0FN/IgG2_G0F	22	39829973	4	5.73E-10	8.60E-08	-	-0.230941	-0.2292	+	T	C	0.672898109	0.0370381	+	T	C	0.7181	0.0428
rs5750825	LC_IGP_R51	IgG2_G0FN/IgG2_G0F	22	39831278	4	6.62E-10	8.24E-08	-	-0.230808	-0.23	+	G	A	0.673729103	0.0371552	+	G	A	0.7182	0.0429
rs1972280	LC_IGP_R51	IgG2_G0FN/IgG2_G0F	22	39831986	4	4.28E-10	1.22E-07	-	-0.232805	-0.2317	+	A	T	0.671042404	0.0370602	+	A	T	0.6934	0.0438
rs4821896	LC_IGP_R51	IgG2_G0FN/IgG2_G0F	22	39833437	4	5.85E-10	3.34E-07	-	-0.231174	-0.2237	+	C	T	0.672255844	0.0370962	+	C	T	0.6945	0.0438
rs73167342	LC_IGP_R51	IgG2_G0FN/IgG2_G0F	22	39834102	4	3.68E-10	1.13E-07	-	-0.234743	-0.2328	+	G	C	0.662823063	0.0372274	+	G	C	0.6853	0.0439
rs5750828	LC_IGP_R51	IgG2_G0FN/IgG2_G0F	22	39835083	4	3.87E-10	2.87E-08	-	-0.234616	-0.2429	+	C	T	0.659598231	0.0372527	+	C	T	0.697	0.0438
rs4821897	LC_IGP_R51	IgG2_G0FN/IgG2_G0F	22	39835587	4	1.09E-09	1.08E-07	-	-0.22738	-0.2294	+	G	A	0.674349603	0.0370845	+	G	A	0.7207	0.0432
rs10644269	LC_IGP_R51	IgG2_G0FN/IgG2_G0F	22	39836972	4	1.40E-09	3.57E-08	-	-0.226362	-0.2369	+	CTT	C	0.671359671	0.0371676	+	CTT	C	0.7136	0.043
rs6001594	LC_IGP_R51	IgG2_G0FN/IgG2_G0F	22	39837472	4	9.17E-10	1.06E-07	-	-0.228657	-0.23	+	T	C	0.673515863	0.0371237	+	T	C	0.7194	0.0433
rs2899318	LC_IGP_R51	IgG2_G0FN/IgG2_G0F	22	39837625	4	9.29E-10	1.09E-07	-	-0.22858	-0.2297	+	G	A	0.673469494	0.0371232	+	G	A	0.7194	0.0433
rs5757673	LC_IGP_R51	IgG2_G0FN/IgG2_G0F	22	39837920	4	9.08E-10	1.37E-07	-	-0.229014	-0.2329	+	C	T	0.671469494	0.0371715	+	C	T	0.6948	0.0442
rs9611176	LC_IGP_R51	IgG2_G0FN/IgG2_G0F	22	39838003	4	1.04E-09	5.14E-08	-	-0.228676	-0.2361	+	G	A	0.672491153	0.0372497	+	G	A	0.7147	0.0433
rs5750829	LC_IGP_R51	IgG2_G0FN/IgG2_G0F	22	39838018	4	6.34E-10	4.27E-07	-	-0.231097	-0.225	+	G	A	0.67014338	0.0371603	+	G	A	0.6816	0.0445
rs4821898	LC_IGP_R51	IgG2_G0FN/IgG2_G0F	22	39838352	4	6.85E-10	1.45E-07	-	-0.230541	-0.2299	+	T	C	0.673107993	0.0371457	+	T	C	0.7209	0.0437
rs5757675	LC_IGP_R51	IgG2_G0FN/IgG2_G0F	22	39838892	4	7.28E-10	1.10E-07	-	-0.230297	-0.2305	+	T	G	0.670433496	0.0371646	+	T	G	0.7151	0.0434
rs6001595	LC_IGP_R51	IgG2_G0FN/IgG2_G0F	22	39839293	4	8.75E-10	8.89E-08	-	-0.230443	-0.2372	+	T	C	0.669060708	0.0373666	+	T	C	0.6911	0.0444
rs8138462	LC_IGP_R51	IgG2_G0FN/IgG2_G0F	22	39839670	4	7.40E-10	1.08E-07	-	-0.230236	-0.2308	+	C	G	0.670399634	0.0371706	+	C	G	0.7151	0.0435
rs5750830	LC_IGP_R51	IgG2_G0FN/IgG2_G0F	22	39840828	4	1.31E-09	1.44E-07	-	-0.227475	-0.2321	+	A	C	0.678703478	0.0372802	+	A	C	0.73	0.0441
rs7364148	LC_IGP_R51	IgG2_G0FN/IgG2_G0F	22	39842165	4	1.24E-09	2.00E-07	-	-0.228575	-0.2292	+	C	T	0.679605857	0.0374074	+	C	T	0.7301	0.0441
rs5750833	LC_IGP_R51	IgG2_G0FN/IgG2_G0F	22	39843091	4	5.12E-10	1.83E-07	-	-0.233555	-0.2303	+	A	C	0.675917938	0.0373501	+	A	C	0.7264	0.0442
rs5757678	LC_IGP_R51	IgG2_G0FN/IgG2_G0F	22	39843409	4	4.60E-10	1.74E-07	-	-0.233521	-0.2308	+	T	C	0.675047895	0.0372428	+	T	C	0.7263	0.0442
rs9306335	LC_IGP_R51	IgG2_G0FN/IgG2_G0F	22	39843537	4	1.25E-09	2.75E-07	-	-0.228537	-0.2284	+	C	T	0.679579012	0.0374098	+	C	T	0.7218	0.0444
rs8137426	LC_IGP_R51	IgG2_G0FN/IgG2_G0F	22	39844350	4	1.25E-09	1.99E-07	-	-0.22852	-0.2294	+	G	T	0.679562843	0.0374109	+	G	T	0.7302	0.0441
rs8136980	LC_IGP_R51	IgG2_G0FN/IgG2_G0F	22	39844574	4	4.84E-10	1.77E-07	-	-0.23387	-0.2314	+	A	G	0.674810449	0.0373464	+	A	G	0.7232	0.0443
rs5757680	LC_IGP_R51	IgG2_G0FN/IgG2_G0F	22	39844793	4	9.39E-10	1.98E-07	-	-0.230444	-0.2296	+	C	T	0.678626602	0.0374373	+	C	T	0.7299	0.0441
rs5757681	LC_IGP_R51	IgG2_G0FN/IgG2_G0F	22	39845547	4	7.77E-10	1.69E-07	-	-0.231765	-0.2306	+	G	A	0.678556546	0.037465	+	G	A	0.729	0.0441
rs1005522	LC_IGP_R51	IgG2_G0FN/IgG2_G0F	22	39845898	4	6.91E-10	2.66E-07	-	-0.240222	-0.2303	+	A	C	0.701192495	0.038714	+	A	C	0.7434	0.0448
rs5757682	LC_IGP_R51	IgG2_G0FN/IgG2_G0F	22	39848259	4	3.00E-09	1.89E-07	-	-0.223731	-0.2308	+	G	T	0.681615619	0.0375087	+	G	T	0.7305	0.0443
rs5757683	LC_IGP_R51	IgG2_G0FN/IgG2_G0F	22	39850174	4	1.39E-09	1.34E-07	-	-0.22769	-0.2344	+	A	G	0.676743638	0.0373784	+	A	G	0.7252	0.0445
rs5757684	LC_IGP_R51	IgG2_G0FN/IgG2_G0F	22	39851584	4	1.76E-09	1.13E-07	-	-0.226171	-0.2382	+	A	G	0.676168593	0.0373644	+	A	G	0.7252	0.0449
rs1557541	LC_IGP_R51	IgG2_G0FN/IgG2_G0F	22	39851970	4	1.90E-09	1.08E-07	-	-0.225602	-0.2392	+	A	C	0.676017084	0.0373519	+	A	C	0.7252	0.045
rs1557542	LC_IGP_R51	IgG2_G0FN/IgG2_G0F	22	39852350	4	2.09E-09	1.05E-07	-	-0.224907	-0.2401	+	C	G	0.675820317	0.0373313	+	C	G	0.7252	0.0452
rs1557543	LC_IGP_R51	IgG2_G0FN/IgG2_G0F	22	39852648	4	3.48E-09	9.00E-08	-	-0.222663	-0.2405	+	T	C	0.680097621	0.0374886	+	T	C	0.7284	0.045
rs6001599	LC_IGP_R51	IgG2_G0FN/IgG2_G0F	22	39852720	4	4.57E-09	1.07E-07	-	-0.220884	-0.2395	+	G	A	0.680662904	0.0374763	+	G	A	0.7294	0.0451
rs6001600	LC_IGP_R51	IgG2_G0FN/IgG2_G0F	22	39852921	4	2.17E-09	8.98E-08	-	-0.224612	-0.2415	+	T	G	0.675754316	0.0373225	+	T	G	0.7246	0.0452
rs113200473	LC_IGP_R51	IgG2_G0FN/IgG2_G0F	22	39853740	4	2.70E-09	3.57E-07	-	-0.224232	-0.2309	+	A	G	0.678862416	0.0374843	+	A	G	0.7005	0.0454
rs5995735	LC_IGP_R51	IgG2_G0FN/IgG2_G0F	22	39854421	4	3.23E-09	8.47E-08	-	-0.223006	-0.2422	+	G	C	0.679300183	0.037466	+	G	C	0.7285	0.0452
rs5757685	LC_IGP_R51	IgG2_G0FN/IgG2_G0F	22	39855540	4	4.40E-09	8.11E-08	-	-0.220809	-0.2433	+	C	G	0.679705918	0.0374236	+	C	G	0.7286	0.0453
rs738286	LC_IGP_R51	IgG2_G0FN/IgG2_G0F	22	39855575	4	2.37E-09	9.33E-08	-	-0.224116	-0.243	+	A	C	0.676311775	0.037328	+	A	C	0.7256	0.0455
rs738287	LC_IGP_R51	IgG2_G0FN/IgG2_G0F	22	39855728	4	4.47E-09	7.83E-08	-	-0.220676	-0.2436	+	C	G	0.679665955	0.0374168	+	C	G	0.7287	0.0454
rs738289	LC_IGP_R51	IgG2_G0FN/IgG2_G0F	22	39855883	4	4.54E-09	7.76E-08	-	-0.220547	-0.2438	+	T	C	0.6796446	0.0374117	+	T	C	0.7287	0.0454
rs738285	LC_IGP_R51	IgG2_G0FN/IgG2_G0F	22	39856356	4	4.57E-09	7.59E-08	-	-0.220664	-0.2442	+	C	G	0.679667785	0.0374383	+	C	G	0.7287	0.0454
rs2413592	LC_IGP_R51	IgG2_G0FN/IgG2_G0F	22	39858196	4	4.42E-09	1.62E-07	-	-0.222195	-0.24	+	G	A	0.678195437	0.0376625	+	G	A	0.7239	0.0458
rs909674	LC_IGP_R51	IgG2_G0FN/IgG2_G0F	22	39859169	4	1.50E-08	2.61E-07	-	-0.215222	-0.2369	+	A	C	0.670960037	0.0378246	+	A	C	0.7201	0.046
rs2008174	LC_IGP_R51	IgG2_G0FN/IgG2_G0F	22	39860130	4	1.44E-08	4.30E-07	-	-0.216461	-0.2335	+	T	C	0.671131073	0.0379967	+	T	C	0.7191	0.0462
rs3959642	LC_IGP_R51	IgG2_G0FN/IgG2_G0F	22	39860589	4	1.42E-08	4.57E-07	-	-0.217024	-0.2328	+	G	A	0.671214155	0.0380761	+	G	A	0.7188	0.0461
rs7286917	LC_IGP_R51	IgG2_G0FN/IgG2_G0F	22	39860868	4	1.32E-08	9.56E-07	-	-0.217978	-0.228	+	G	A	0.671429635	0.038162	+	G	A	0.7161	0.0465
rs137682	LC_IGP_R81	IgG4_G0FN/IgG4_G0F	22	39738425	4	1.74E-10	6.82E-12	-	-0.257906	-0.2927	+	C	A	0.738521195	0.0401523	+	C	A	0.7726	0.0426
rs137683	LC_IGP_R81	IgG4_G0FN/IgG4_G0F	22	39738501	4	3.77E-12	1.60E-14	-	-0.273563	-0.3265	+	C	G	0.722431361	0.0390901	+	C	G	0.7566	0.0425
rs137686	LC_IGP_R81	IgG4_G0FN/IgG4_G0F	22	39739638	4	1.41E-14	1.17E-15	-	-0.287138	-0.3317	+	G	C	0.705918243	0.0369689	+	G	C	0.7336	0.0414
rs137690	LC_IGP_R81	IgG4_G0FN/IgG4_G0F	22	39742825	4	1.11E-14	1.11E-15	-	-0.288669	-0.332	+	G	C	0.705469189	0.0370202	+	G	C	0.7336	0.0414
rs9611156	LC_IGP_R81	IgG4_G0FN/IgG4_G0F	22	39743170	4	2.05E-14	2.50E-15	-	-0.286644	-0.3292	+	T	G	0.701516779	0.0371408	+	T	G	0.7267	0.0416
rs137699	LC_IGP_R81	IgG4_G0FN/IgG4_G0F	22	39748854	4	8.05E-11	4.14E-12	-	-0.233432	-0.2745	+	G	A	0.648849603	0.0356779	+	G	A	0.6761	0.0396
rs137700	LC_IGP_R81	IgG4_G0FN/IgG4_G0F	22	39749072	4	7.31E-15	3.20E-15	-	-0.291753	-0.3384	+	G	A	0.705327334	0.0371537	+	G	A	0.7027	0.0429
rs137701	LC_IGP_R81	IgG4_G0FN/IgG4_G0F	22	39750146	4	1.35E-15	1.86E-15	-	-0.301043	-0.3353	+	C	T	0.711829164	0.0373069	+	C	T	0.7305	0.0422
rs137702	LC_IGP_R81	IgG4_G0FN/IgG4_G0F	22	39750158	4	1.35E-15	4.92E-16	-	-0.30105	-0.3423	+	C	T	0.711829469	0.0373069	+	C	T	0.7373	0.0422
rs137703	LC_IGP_R81	IgG4_G0FN/IgG4_G0F	22	39750241	4	9.91E-11	1.18E-12	-	-0.23244	-0.2815	+	G	A	0.648769067	0.0356998	+	G	A	0.6711	0.0396
rs137705	LC_IGP_R81	IgG4_G0FN/IgG4_G0F	22	39751394	4	1.15E-11	6.15E-13	-	-0.244377	-0.2871	+	C	T	0.653327334	0.0357532	+	C	T	0.6726	0.0399
rs137706	LC_IGP_R81	IgG4_G0FN/IgG4_G0F	22	39751400	4	3.08E-16	4.42E-15	-	-0.307781	-0.3336	+	G	T	0.709212935	0.0372829	+	G	T	0.7192	0.0425
rs137707	LC_IGP_R81	IgG4_G0FN/IgG4_G0F	22	39751401	4	3.08E-16	4.46E-15	-	-0.307782	-0.3336	+	C	T	0.70921324	0.0372828	+	C	T	0.7192	0.0425
rs137708	LC_IGP_R81	IgG4_G0FN/IgG4_G0F	22	39751774	4	3.44E-16	1.17E-15	-	-0.307236	-0.3393	+	G	A	0.709517389	0.0372787	+	G	A	0.7316	0.0424
rs137709	LC_IGP_R81	IgG4_G0FN/IgG4_G0F	22	39752203	4	4.24E-16	8.15E-16	-	-0.307171	-0.3449	+	ATGGGATTACAGG	A	0.707462477	0.0373885	+	ATGGGATTACAGG	A	0.7033	0.0428
rs2413586	LC_IGP_R81	IgG4_G0FN/IgG4_G0F	22	39753722	4	7.15E-12	3.40E-12	-	-0.247311	-0.2864	+	T	C	0.646302013	0.0358157	+	T	C	0.6137	0.0412
rs2413587	LC_IGP_R81	IgG4_G0FN/IgG4_G0F	22	39753779	4	1.31E-11	3.41E-13	-	-0.243334	-0.289	+	C	T	0.653479866	0.0357001	+	C	T	0.673	0.0397
rs5757630	LC_IGP_R81	IgG4_G0FN/IgG4_G0F	22	39754433	4	1.44E-11	3.97E-13	-	-0.242692	-0.2884	+	T	G	0.653509457	0.0356771	+	T	G	0.6735	0.0397
rs2413588	LC_IGP_R81	IgG4_G0FN/IgG4_G0F	22	39755175	4	2.24E-11	3.51E-13	-	-0.239811	-0.2895	+	G	A	0.654362416	0.0355987	+	G	A	0.6747	0.0398
rs760742	LC_IGP_R81	IgG4_G0FN/IgG4_G0F	22	39755190	4	2.16E-11	2.53E-13	-	-0.240049	-0.2914	+	T	G	0.654468578	0.035605	+	T	G	0.6752	0.0398
rs2413589	LC_IGP_R81	IgG4_G0FN/IgG4_G0F	22	39755689	4	5.57E-16	3.98E-16	-	-0.304728	-0.3425	+	C	T	0.710755339	0.0372458	+	C	T	0.7363	0.0421
rs5757631	LC_IGP_R81	IgG4_G0FN/IgG4_G0F	22	39755953	4	7.07E-11	4.54E-13	-	-0.233949	-0.2865	+	C	T	0.648268456	0.0356484	+	C	T	0.6708	0.0396
rs5757632	LC_IGP_R81	IgG4_G0FN/IgG4_G0F	22	39756097	4	6.95E-11	4.51E-13	-	-0.234092	-0.2866	+	C	A	0.648274863	0.035656	+	C	A	0.6708	0.0396
rs5757633	LC_IGP_R81	IgG4_G0FN/IgG4_G0F	22	39756270	4	2.02E-11	2.41E-13	-	-0.240847	-0.2919	+	G	A	0.654368822	0.0356724	+	G	A	0.6752	0.0399
rs2049986	LC_IGP_R81	IgG4_G0FN/IgG4_G0F	22	39756854	4	7.66E-11	3.86E-13	-	-0.233822	-0.288	+	C	G	0.646723917	0.0356951	+	C	G	0.669	0.0397
rs4821887	LC_IGP_R81	IgG4_G0FN/IgG4_G0F	22	39756985	4	3.32E-16	3.22E-16	-	-0.308612	-0.3447	+	T	C	0.711241001	0.0374251	+	T	C	0.7368	0.0422
rs5757636	LC_IGP_R81	IgG4_G0FN/IgG4_G0F	22	39758509	4	5.50E-10	8.79E-09	-	-0.319414	-0.2922	+	G	A	0.854682527	0.051173	+	G	A	0.8497	0.0508
rs5757637	LC_IGP_R81	IgG4_G0FN/IgG4_G0F	22	39758541	4	5.37E-10	6.23E-09	-	-0.319555	-0.2931	+	G	A	0.854721148	0.0511662	+	G	A	0.8497	0.0504
rs10745050	LC_IGP_R81	IgG4_G0FN/IgG4_G0F	22	39758881	4	4.47E-08	5.11E-11	-	-0.261556	-0.3288	+	G	A	0.814737559	0.0475813	+	G	A	0.8144	0.0501
rs5757642	LC_IGP_R81	IgG4_G0FN/IgG4_G0F	22	39764824	4	5.03E-22	1.41E-26	-	-0.34772	-0.4082	+	C	T	0.608274863	0.0355256	+	C	T	0.643	0.0383
rs7286714	LC_IGP_R81	IgG4_G0FN/IgG4_G0F	22	39765604	4	1.35E-21	5.67E-27	-	-0.343446	-0.4163	+	G	C	0.601641245	0.0354744	+	G	C	0.6329	0.0387
rs5757644	LC_IGP_R81	IgG4_G0FN/IgG4_G0F	22	39766440	4	9.22E-22	4.23E-27	-	-0.345066	-0.4179	+	G	C	0.60171568	0.0354902	+	G	C	0.6329	0.0388

rs5750806	LC_IGP_R81	IgG4_G0FN/IgG4_G0F	22	39767011	4	8.26E-22	4.15E-27	-	-0.34552	-0.418	+	G	A	0.601745514	0.0354935	+	G	A	0.633	0.0388
rs9611162	LC_IGP_R81	IgG4_G0FN/IgG4_G0F	22	39767247	4	8.04E-22	4.02E-27	-	-0.345635	-0.4182	+	A	C	0.601740696	0.035495	+	A	C	0.633	0.0388
rs9611164	LC_IGP_R81	IgG4_G0FN/IgG4_G0F	22	39767291	4	7.99E-22	1.43E-27	-	-0.34582	-0.4251	+	A	G	0.60149817	0.0355114	+	A	G	0.6248	0.0391
rs5757645	LC_IGP_R81	IgG4_G0FN/IgG4_G0F	22	39768479	4	6.47E-22	3.14E-27	-	-0.346554	-0.4194	+	A	G	0.601792556	0.0355039	+	A	G	0.6328	0.0388
rs1569499	LC_IGP_R81	IgG4_G0FN/IgG4_G0F	22	39769818	4	1.23E-22	3.73E-27	-	-0.353692	-0.4164	+	C	T	0.606561318	0.0355907	+	C	T	0.64	0.0386
rs4821888	LC_IGP_R81	IgG4_G0FN/IgG4_G0F	22	39770597	4	1.10E-22	2.62E-27	-	-0.354193	-0.4178	+	G	A	0.607441493	0.0355985	+	G	A	0.6413	0.0386
rs4820377	LC_IGP_R81	IgG4_G0FN/IgG4_G0F	22	39770780	4	1.48E-22	2.75E-27	-	-0.35315	-0.4164	+	A	G	0.607897869	0.0356062	+	A	G	0.6422	0.0385
rs6001566	LC_IGP_R81	IgG4_G0FN/IgG4_G0F	22	39774448	4	6.51E-28	7.05E-31	-	-0.39777	-0.463	+	G	A	0.626967968	0.0356509	+	G	A	0.6567	0.0401
rs6519190	LC_IGP_R81	IgG4_G0FN/IgG4_G0F	22	39774525	4	2.62E-28	5.66E-31	-	-0.400323	-0.4637	+	G	A	0.627102579	0.0356033	+	G	A	0.6569	0.0401
rs5757647	LC_IGP_R81	IgG4_G0FN/IgG4_G0F	22	39775047	4	1.38E-28	6.70E-30	-	-0.402785	-0.4518	+	A	C	0.629250458	0.0356283	+	A	C	0.6616	0.0398
rs5757648	LC_IGP_R81	IgG4_G0FN/IgG4_G0F	22	39775156	4	1.36E-28	1.08E-29	-	-0.402819	-0.45	+	A	G	0.629255034	0.035627	+	A	G	0.6609	0.0398
rs9611165	LC_IGP_R81	IgG4_G0FN/IgG4_G0F	22	39775250	4	1.34E-28	6.63E-30	-	-0.402852	-0.4518	+	G	A	0.629261135	0.0356258	+	G	A	0.6616	0.0398
rs9611166	LC_IGP_R81	IgG4_G0FN/IgG4_G0F	22	39775268	4	1.33E-28	6.65E-30	-	-0.40286	-0.4518	+	C	T	0.629260525	0.0356254	+	C	T	0.6616	0.0398
rs6001567	LC_IGP_R81	IgG4_G0FN/IgG4_G0F	22	39775400	4	1.31E-28	6.61E-30	-	-0.402906	-0.4519	+	T	A	0.629265711	0.0356234	+	T	A	0.6616	0.0398
rs9611167	LC_IGP_R81	IgG4_G0FN/IgG4_G0F	22	39775583	4	3.87E-28	5.94E-30	-	-0.399179	-0.4554	+	A	G	0.62500122	0.0356188	+	A	G	0.6543	0.04
rs6001568	LC_IGP_R81	IgG4_G0FN/IgG4_G0F	22	39775786	4	1.52E-28	5.93E-30	-	-0.401797	-0.451	+	A	G	0.630751373	0.0355698	+	A	G	0.6632	0.0397
rs4821889	LC_IGP_R81	IgG4_G0FN/IgG4_G0F	22	39777254	4	1.46E-28	4.42E-30	-	-0.401491	-0.4569	+	C	T	0.625502746	0.0355305	+	C	T	0.654	0.0401
rs4821890	LC_IGP_R81	IgG4_G0FN/IgG4_G0F	22	39777523	4	1.39E-28	4.41E-30	-	-0.401599	-0.457	+	A	G	0.625520134	0.0355262	+	A	G	0.6541	0.0401
rs1010169	LC_IGP_R81	IgG4_G0FN/IgG4_G0F	22	39778167	4	1.16E-28	5.38E-30	-	-0.402136	-0.4515	+	G	A	0.630963392	0.0355213	+	G	A	0.6636	0.0397
rs1010170	LC_IGP_R81	IgG4_G0FN/IgG4_G0F	22	39778327	4	1.13E-28	4.06E-30	-	-0.402197	-0.453	+	C	T	0.630961257	0.0355187	+	C	T	0.6629	0.0397
rs5757650	LC_IGP_R81	IgG4_G0FN/IgG4_G0F	22	39778419	4	1.33E-28	7.46E-30	-	-0.401372	-0.4536	+	C	T	0.628325808	0.0354936	+	C	T	0.6583	0.04
rs756640	LC_IGP_R81	IgG4_G0FN/IgG4_G0F	22	39779300	4	1.04E-28	3.96E-30	-	-0.40217	-0.4574	+	A	G	0.625589994	0.0354918	+	A	G	0.654	0.0401
rs7423	LC_IGP_R81	IgG4_G0FN/IgG4_G0F	22	39781429	4	5.09E-29	2.81E-30	-	-0.404345	-0.4591	+	T	C	0.626133236	0.0354721	+	T	C	0.6542	0.0402
rs1007337	LC_IGP_R81	IgG4_G0FN/IgG4_G0F	22	39781585	4	9.25E-29	6.29E-30	-	-0.402029	-0.4549	+	T	C	0.627876144	0.0354436	+	T	C	0.6587	0.04
rs5757652	LC_IGP_R81	IgG4_G0FN/IgG4_G0F	22	39781855	4	1.08E-28	2.51E-29	-	-0.400549	-0.4595	+	C	T	0.624656193	0.0353583	+	C	T	0.6322	0.0409
rs9611169	LC_IGP_R81	IgG4_G0FN/IgG4_G0F	22	39783027	4	7.20E-29	5.20E-30	-	-0.401941	-0.4523	+	T	C	0.632014338	0.0353623	+	T	C	0.664	0.0397
rs9611170	LC_IGP_R81	IgG4_G0FN/IgG4_G0F	22	39784845	4	6.79E-30	1.79E-32	-	-0.409126	-0.4832	+	C	G	0.628837401	0.0353093	+	C	G	0.6725	0.0407
rs2142848	LC_IGP_R81	IgG4_G0FN/IgG4_G0F	22	39785242	4	1.57E-29	3.50E-32	-	-0.406514	-0.48	+	T	C	0.630293167	0.0353208	+	T	C	0.6717	0.0406
rs4821891	LC_IGP_R81	IgG4_G0FN/IgG4_G0F	22	39785381	4	1.57E-29	7.33E-32	-	-0.406511	-0.478	+	T	C	0.630293777	0.0353206	+	T	C	0.671	0.0407
rs2413590	LC_IGP_R81	IgG4_G0FN/IgG4_G0F	22	39790191	4	3.07E-29	7.83E-32	-	-0.40602	-0.4755	+	C	G	0.636996949	0.0354718	+	C	G	0.6799	0.0405
rs5750808	LC_IGP_R81	IgG4_G0FN/IgG4_G0F	22	39790987	4	3.06E-29	7.80E-32	-	-0.406019	-0.4755	+	A	G	0.63699878	0.0354704	+	A	G	0.6799	0.0405
rs5750809	LC_IGP_R81	IgG4_G0FN/IgG4_G0F	22	39791491	4	1.50E-27	2.96E-32	-	-0.393752	-0.4811	+	G	T	0.634429225	0.0355457	+	G	T	0.6727	0.0407
rs5750810	LC_IGP_R81	IgG4_G0FN/IgG4_G0F	22	39792943	4	3.04E-29	7.78E-32	-	-0.406007	-0.4755	+	G	A	0.637005186	0.0354672	+	G	A	0.6799	0.0405
rs5750811	LC_IGP_R81	IgG4_G0FN/IgG4_G0F	22	39793066	4	3.04E-29	7.79E-32	-	-0.406007	-0.4755	+	G	T	0.637005186	0.035467	+	G	T	0.6799	0.0405
rs5750812	LC_IGP_R81	IgG4_G0FN/IgG4_G0F	22	39793079	4	2.10E-29	6.99E-32	-	-0.407346	-0.4786	+	A	G	0.632947834	0.0354772	+	A	G	0.6727	0.0407
rs4321460	LC_IGP_R81	IgG4_G0FN/IgG4_G0F	22	39793655	4	1.50E-29	2.79E-26	-	-0.408607	-0.4607	+	G	A	0.635403905	0.0354889	+	G	A	0.6362	0.0434
rs4386422	LC_IGP_R81	IgG4_G0FN/IgG4_G0F	22	39793734	4	1.58E-28	4.13E-30	-	-0.404528	-0.4792	+	T	C	0.628008542	0.0358237	+	T	C	0.6293	0.042
rs4384886	LC_IGP_R81	IgG4_G0FN/IgG4_G0F	22	39793766	4	2.08E-29	8.61E-32	-	-0.407357	-0.478	+	T	C	0.63295058	0.0354759	+	T	C	0.6729	0.0407
rs5757653	LC_IGP_R81	IgG4_G0FN/IgG4_G0F	22	39794071	4	3.52E-09	7.75E-08	-	-0.280161	-0.2705	+	T	C	0.200112239	0.0471819	+	T	C	0.1834	0.0504
rs5757654	LC_IGP_R81	IgG4_G0FN/IgG4_G0F	22	39794124	4	2.08E-29	3.41E-31	-	-0.407363	-0.4837	+	A	G	0.632936242	0.0354755	+	A	G	0.6493	0.0416
rs7292066	LC_IGP_R81	IgG4_G0FN/IgG4_G0F	22	39794241	4	3.69E-29	1.62E-31	-	-0.405159	-0.474	+	A	G	0.63528737	0.0354496	+	A	G	0.6764	0.0406
rs5750813	LC_IGP_R81	IgG4_G0FN/IgG4_G0F	22	39795228	4	2.51E-29	8.31E-32	-	-0.406913	-0.4753	+	C	G	0.636499085	0.0354904	+	C	G	0.6796	0.0405
rs4821892	LC_IGP_R81	IgG4_G0FN/IgG4_G0F	22	39795683	4	3.01E-29	8.59E-32	-	-0.405989	-0.4749	+	C	G	0.637007627	0.0354626	+	C	G	0.6799	0.0405
rs3830119	LC_IGP_R81	IgG4_G0FN/IgG4_G0F	22	39796097	4	3.02E-09	4.13E-08	-	-0.283274	-0.2837	+	C	T	0.201598915	0.0475026	+	C	T	0.1812	0.0517
rs5757655	LC_IGP_R81	IgG4_G0FN/IgG4_G0F	22	39797178	4	1.41E-29	4.24E-32	-	-0.406326	-0.4792	+	C	G	0.630937157	0.0352742	+	C	G	0.6737	0.0406
rs4821893	LC_IGP_R81	IgG4_G0FN/IgG4_G0F	22	39797779	4	2.82E-29	6.22E-32	-	-0.404084	-0.4749	+	A	G	0.634708969	0.0352776	+	A	G	0.6796	0.0404
rs5750814	LC_IGP_R81	IgG4_G0FN/IgG4_G0F	22	39797987	4	3.61E-29	6.10E-32	-	-0.403294	-0.4779	+	C	T	0.631031117	0.0352799	+	C	T	0.6729	0.0406
rs5757657	LC_IGP_R81	IgG4_G0FN/IgG4_G0F	22	39798429	4	3.58E-29	6.08E-32	-	-0.403303	-0.478	+	T	G	0.631033557	0.0352786	+	T	G	0.6729	0.0406
rs5750815	LC_IGP_R81	IgG4_G0FN/IgG4_G0F	22	39798449	4	4.53E-29	9.00E-32	-	-0.40229	-0.4734	+	C	T	0.635448444	0.0352575	+	C	T	0.6794	0.0404
rs4429561	LC_IGP_R81	IgG4_G0FN/IgG4_G0F	22	39799789	4	3.77E-29	1.21E-30	-	-0.402532	-0.4774	+	G	A	0.633452715	0.0352261	+	G	A	0.6474	0.0415
rs35509952	LC_IGP_R81	IgG4_G0FN/IgG4_G0F	22	39800563	4	3.45E-29	1.57E-31	-	-0.403289	-0.4752	+	AC	A	0.631052776	0.0352667	+	AC	A	0.6705	0.0407
rs4337572	LC_IGP_R81	IgG4_G0FN/IgG4_G0F	22	39800704	4	3.43E-29	5.98E-32	-	-0.403291	-0.4782	+	G	C	0.6310482	0.0352649	+	G	C	0.6732	0.0406
rs6001582	LC_IGP_R81	IgG4_G0FN/IgG4_G0F	22	39806153	4	1.73E-28	1.11E-31	+	0.398954	0.474	+	C	T	0.364817572	0.0353568	+	C	T	0.3245	0.0405
rs4821894	LC_IGP_R81	IgG4_G0FN/IgG4_G0F	22	39809820	4	3.73E-29	7.81E-32	-	-0.40388	-0.4738	+	C	G	0.637207749	0.0353405	+	C	G	0.6803	0.0403
rs5750816	LC_IGP_R81	IgG4_G0FN/IgG4_G0F	22	39810379	4	2.58E-29	1.00E-31	-	-0.403336	-0.4738	+	T	C	0.633503356	0.0351869	+	T	C	0.678	0.0404
rs5757659	LC_IGP_R81	IgG4_G0FN/IgG4_G0F	22	39812409	4	4.61E-29	7.82E-32	-	-0.401898	-0.4738	+	G	T	0.635753508	0.0352284	+	G	T	0.6803	0.0403
rs6001585	LC_IGP_R81	IgG4_G0FN/IgG4_G0F	22	39812986	4	9.75E-27	4.44E-25	-	-0.423509	-0.463	+	A	C	0.744356315	0.0388693	+	A	C	0.7832	0.0448
rs3859849	LC_IGP_R81	IgG4_G0FN/IgG4_G0F	22	39817017	4	6.49E-11	4.43E-11	-	-0.301067	-0.3159	+	A	C	0.222187994	0.0457839	+	A	C	0.2448	0.0479
rs6001587	LC_IGP_R81	IgG4_G0FN/IgG4_G0F	22	39819008	4	5.94E-29	1.49E-31	-	-0.400828	-0.4837	+	C	T	0.63526083	0.0352084	+	C	T	0.6569	0.0414
rs6001588	LC_IGP_R81	IgG4_G0FN/IgG4_G0F	22	39819049	4	6.45E-29	1.07E-31	-	-0.401241	-0.4734	+	C	G	0.635204393	0.0352685	+	C	G	0.6796	0.0404
rs7288760	LC_IGP_R81	IgG4_G0FN/IgG4_G0F	22	39819969	4	5.49E-31	1.01E-31	-	-0.423644	-0.4871	+	G	A	0.641305064	0.0358468	+	G	A	0.6705	0.0416
rs5750818	LC_IGP_R81	IgG4_G0FN/IgG4_G0F	22	39820885	4	4.87E-29	1.04E-31	-	-0.401031	-0.4737	+	G	A	0.634838316	0.0351684	+	G	A	0.6782	0.0404
rs5757663	LC_IGP_R81	IgG4_G0FN/IgG4_G0F	22	39821319	4	4.62E-29	4.14E-32	-	-0.401908	-0.4757	+	C	T	0.635743746	0.0352299	+	C	T	0.6793	0.0403
rs5757664	LC_IGP_R81	IgG4_G0FN/IgG4_G0F	22	39821536	4	4.85E-29	1.03E-31	-	-0.401055	-0.4738	+	G	A	0.634835265	0.0351695	+	G	A	0.6782	0.0404
rs5757665	LC_IGP_R81	IgG4_G0FN/IgG4_G0F	22	39821641	4	4.58E-29	7.68E-32	-	-0.40193	-0.474	+	G	A	0.635749542	0.0352297	+	G	A	0.6803	0.0404
rs5757666	LC_IGP_R81	IgG4_G0FN/IgG4_G0F	22	39821713	4	6.01E-10	4.97E-09	-	-0.308402	-0.3392	+	A	G	0.180460096	0.049522	+	A	G	0.1722	0.058
rs5757667	LC_IGP_R81	IgG4_G0FN/IgG4_G0F	22	39822116	4	3.51E-30	1.02E-31	-	-0.41033	-0.4856	+	G	A	0.624489018	0.0352271	+	G	A	0.6533	0.0414
rs4821895	LC_IGP_R81	IgG4_G0FN/IgG4_G0F	22	39823015	4	1.94E-29	7.37E-32	-	-0.405022	-0.4761	+	G	A	0.632880496	0.0352515	+	G	A	0.6777	0.0405
rs739141	LC_IGP_R81	IgG4_G0FN/IgG4_G0F	22	39824450	4	1.64E-22	3.05E-27	-	-0.352643	-0.4342	+	T	C	0.602684626	0.0355949	+	T	C	0.6369	0.0402
rs743838	LC_IGP_R81	IgG4_G0FN/IgG4_G0F	22	39824707	4	2.61E-29	5.03E-32	-	-0.405653	-0.4774	+	G	T	0.637717511	0.0353925	+	G	T	0.6805	0.0405
rs5750820	LC_IGP_R81	IgG4_G0FN/IgG4_G0F	22	39825322	4	2.66E-29	6.02E-32	-	-0.405221	-0.4779	+	G	T	0.633296827	0.0353601	+	G	T	0.6781	0.0406
rs5750821	LC_IGP_R81	IgG4_G0FN/IgG4_G0F	22	39825492	4	1.57E-29	5.58E-32	-	-0.406506	-0.4782	+	T	A	0.633002441	0.0353205	+	T	A	0.678	0.0406
rs5750822	LC_IGP_R81	IgG4_G0FN/IgG4_G0F	22	39826788	4	1.66E-29	6.04E-32	-	-0.406208	-0.478	+	A	G	0.633397193	0.0353101	+	A	G	0.678	0.0406
rs7949	LC_IGP_R81	IgG4_G0FN/IgG4_G0F	22	39827553	4	1.36E-29	5.54E-32	-	-0.407214	-0.4786	+	A	G	0.632858755	0.0353406	+	A	G	0.6778	0.0407
rs5757670	LC_IGP_R81	IgG4_G0FN/IgG4_G0F	22	39829736	4	1.91E-29	3.82E-30	-	-0.406813	-0.4766	+	A	G	0.630845333	0.0354037	+	A	G	0.6513	0.0418
rs5750823	LC_IGP_R81	IgG4_G0FN/IgG4_G0F	22	39829973	4	2.54E-34	5.13E-37	-	-0.449768	-0.5363	+	T	C	0.672593045	0.0359752	+	T	C	0.7181	0.0422
rs5750824	LC_IGP_R81	IgG4_G0FN/IgG4_G0F	22	39830123	4	2.84E-09	5.71E-08	-	-0.282382	-0.2575	+	G	A	0.19716481	0.0472706	+	G	A	0.1844	0.0474
rs5750825	LC_IGP_R81	IgG4_G0FN/IgG4_G0F	22	39831278	4	2.43E-34	4.96E-37	-	-0.451283	-0.5373	+	G	A	0.673424039	0.0360848	+	G	A	0.7182	0.0423
rs1972280	LC_IGP_R81	IgG4_G0FN/IgG4_G0F	22	39831986	4	2.77E-34	1.60E-36	-	-0.449865	-0.5431	+	A	T	0.67073734	0.036005	+	A	T	0.6934	0.043
rs5750826	LC_IGP_R81	IgG4_G0FN/IgG4_G0F	22	39832113	4	1.60E-21	4.71E-26	-	-0.343828	-0.4218	+	G	A	0.534676022	0.03558	+	G	A	0.5447	0.04
rs4821896	LC_IGP_R81	IgG4_G0FN/IgG4_G0F	22	39833437	4	2.38E-34	1.42E-35	-	-0.450662	-0.5362	+	C	T	0.67195078	0.0360298	+	C	T	0.6945	0.0431
rs73167342	LC_IGP_R81	IgG4_G0FN/IgG4_G0F	22	39834102	4	7.71E-35	2.53E-38	-	-0.455612	-0.5559	+	G	C	0.662517999	0.0361435	+	G	C	0.6853	0.0429
rs5750828	LC_IGP_R81	IgG4_G0FN/IgG4_G0F	22	39835083	4	3.71E-35	3.17E-38	-	-0.457998	-0.5568	+	C	T	0.659293167	0.0361513	+	C	T	0.697	0.0431
rs4821897	LC_IGP_R81	IgG4_G0FN/IgG4_G0F	22	39835587	4	4.16E-34	7.24E-37	-	-0.448743	-0.539	+	G	A	0.674044539	0.0360171	+	G	A	0.7207	0.0425
rs10644269	LC_IGP_R81	IgG4_G0FN/IgG4_G0F	22	39836972	4	3.09E-34	4.11E-37	-	-0.450486	-0.5378	+	CTT	C	0.670996949	0.0360821	+	CTT	C	0.7136	0.0422
rs6001594	LC_IGP_R81	IgG4_G0FN/IgG4_G0F	22	39837472	4	3.87E-34	6.86E-37	-	-0.449475	-0.54	+	T	C	0.673210799	0.0360574	+	T	C	0.7194	0.0426
rs2899318	LC_IGP_R81	IgG4_G0FN/IgG4_G0F	22	39837625	4	3.84E-34	6.82E-37	-	-0.449482	-0.5402	+	G	A	0.67316443	0.0360565	+	G	A	0.7194	0.0426
rs5757673	LC_IGP_R81	IgG4_G0FN/IgG4_G0F	22	39837920	4	4.74E-35	2.27E-36	-	-0.456054	-0.5459	+	C	T	0.67116443	0.0360582	+	C	T	0.6948	0.0434
rs9611176	LC_IGP_R81	IgG4_G0FN/IgG4_G0F	22	39838003	4	1.73E-34	2.78E-38	-	-0.453287	-0.5513	+	G	A	0.672186089	0.0361595	+	G	A	0.7147	0.0426
rs5750829	LC_IGP_R81	IgG4_G0FN/IgG4_G0F	22	39838018	4	1.95E-34	1.42E-34	-	-0.451988	-0.5375	+	G	A	0.669838316	0.0360862	+	G	A	0.6816	0.0438
rs4821898	LC_IGP_R81	IgG4_G0FN/IgG4_G0F	22	39838352	4	1.94E-34	1.85E-36	-	-0.451802	-0.5419	+	T	C	0.672802929	0.03607	+	T	C	0.7209	0.043
rs5757675	LC_IGP_R81	IgG4_G0FN/IgG4_G0F	22	39838892	4	2.02E-34	3.11E-37	-	-0.451903	-0.5443	+	T	G	0.670128432	0.0360881	+	T	G	0.7151	0.0427
rs6001595	LC_IGP_R81	IgG4_G0FN/IgG4_G0F	22	39839293	4	2.95E-34	8.63E-36	-	-0.453222	-0.5453	+	T	C	0.668755644	0.0362889	+	T	C	0.6911	0.0437
rs8138462	LC_IGP_R81	IgG4_G0FN/IgG4_G0F	22	39839670	4	2.04E-34	3.11E-37	-	-0.451937	-0.5447	+	C	G	0.67009457	0.0360939	+	C	G	0.7151	0.0427
rs9611177	LC_IGP_R81	IgG4_G0FN/IgG4_G0F	22	39840130	4	4.14E-12	1.13E-13	-	-0.269386	-0.2987	+	T	C	0.376544539	0.0385691	+	T	C	0.4109	0.0402
rs5750830	LC_IGP_R81	IgG4_G0FN/IgG4_G0F	22	39840828	4	2.49E-34	1.09E-37	-	-0.452539	-0.5562	+	A	C	0.678398414	0.0361916	+	A	C	0.73	0.0433
rs28685576	LC_IGP_R81	IgG4_G0FN/IgG4_G0F	22	39841533	4	1.71E-22	3.79E-25	-	-0.351388	-0.4138	+	A	C	0.535545149	0.0354829	+	A	C	0.5638	0.04
rs5757676	LC_IGP_R81	IgG4_G0FN/IgG4_G0F	22	39841700	4	2.67E-27	1.86E-26	-	-0.435264	-0.4981	+	C	T	0.749258999	0.039492	+	C	T	0.7901	0.0468
rs7364148	LC_IGP_R81	IgG4_G0FN/IgG4_G0F	22	39842165	4	2.30E-34	1.66E-37	-	-0.45432	-0.5535	+	C	T	0.679300793	0.0363144	+	C	T	0.7301	0.0432
rs5750833	LC_IGP_R81	IgG4_G0FN/IgG4_G0F	22	39843091	4	1.10E-34	1.58E-37	-	-0.456003	-0.5541	+	A	C	0.675612874	0.0362619	+	A	C	0.7264	0.0433
rs5757678	LC_IGP_R81	IgG4_G0FN/IgG4_G0F	22	39843409	4	1.09E-34	1.54E-37	-	-0.454736	-0.5542	+	T	C	0.674742831	0.0361601	+	T	C	0.7263	0.0433
rs9306335	LC_IGP_R81	IgG4_G0FN/IgG4_G0F	22	39843537	4	2.33E-34	5.74E-38	-	-0.45431	-0.5603	+	C	T	0.679273948	0.0363169	+	C	T	0.7218	0.0435
rs8137426	LC_IGP_R81	IgG4_G0FN/IgG4_G0F	22	39844350	4	2.34E-34	1.74E-37	-	-0.454311	-0.5538	+	G	T	0.679257779	0.036318	+	G	T	0.7302	0.0433
[truncated: 764,024 more chars]
