# Supplementary material for: Genome-Wide Association Study on Immunoglobulin G Glycosylation Patterns
Source: Front Immunol. 2018 Feb 26;9:277. doi: 10.3389/fimmu.2018.00277 (PMC5834439; doi:10.3389/fimmu.2018.00277)
Supplement: Table S6 — List of replicated phenotypic traits for each gene region. [file Table_6.PDF]

Lists of Associated IgG Traits per Gene Locus

Suppl. Table 7a: IgG glycan traits associated to variant on chromosome 3 within the *ST6GAL1* region

***ST6GAL1* (chromosome 3)**		
**IgG1**	**IgG2**	**IgG4**
IgG1 FG1S1/(FG1+FG1S1)	IgG2 FG1S1/(FG1+FG1S1)	IgG4 FG1S1/(FG1+FG1S1)
IgG1 FG2S1/(FG2+FG2S1)	IgG2 FG2S1/(FG2+FG2S1)	IgG4 FG2S1/(FG2+FG2S1)
IgG1 SA per Gal	IgG2 SA per Gal	IgG4 SA per Gal
IgG1_G1FS1/IgG1_G1F	IgG2_G1FS1/IgG2_G1F	IgG4_G1FS1/IgG4_G1F
IgG1_G2FS1/IgG1_G2F	IgG2_G2FS1/IgG2_G2F	IgG4_G2FS1/IgG4_G2F
IgG1 FGS1/(FG+FGS1)	IgG2 FGS1/(FG+FGS1)	IgG4 FGS1/(FG+FGS1)
IgG1_G1FS1	IgG2_G1FS1	IgG4_G1FS1
IgG1 FGS1/(F+FG+FGS1)	IgG2 FGS1/(F+FG+FGS1)	
IgG1 G2S1/(G2+G2S1)		
IgG1_G2S1/IgG1_G2		
	IgG2 Sialylation	

Suppl. Table 7b: IgG glycan traits associated to variant on chromosome 9 within the *B4GALT1* region

***B4GAL1* (chromosome 9)**		
**IgG1**	**IgG2**	**IgG4**
IgG1_G2FS1/IgG1_G1FS1	IgG2_G2FS1/IgG2_G1FS1	IgG4_G2FS1/IgG4_G1FS1
		IgG4 FGS1/(FG+FGS1)
IgG1_G2F/IgG1_G1F	IgG2_G2F/IgG2_G1F	IgG4_G2F/IgG4_G1F
		IgG4_G2FS1
IgG1_G2F	IgG2_G2F	IgG4_G2F
IgG1_G2Fn	IgG2_G2Fn	IgG4_G2Fn
IgG1_G2n		IgG4_G2n
IgG1 Galactosylation	IgG2 Galactosylation	
IgG1_G0FN		
IgG1_G0FNn		
IgG1_G0n	IgG2_G0n	
IgG1_G1F/IgG1_G0F	IgG2_G1F/IgG2_G0F	
IgG1_G1FN/IgG1_G0FN	IgG2_G1FN/IgG2_G0FN	
IgG1_G1n	IgG2_G1n	
IgG1_G1/IgG1_G0		
	IgG2_G1Fn	
	IgG2_G1F	

Suppl. Table 7c: IgG glycan traits associated to variant on chromosome 14 within the *FUT8* region

***FUT8* (chromosome 14)**		
**IgG1**	**IgG2**	**Subclass Comparison**
IgG1 BG0n/G0n		
IgG1 BG1n/G1n		
IgG1 BG1S1/(BG1+BG1S1)		
IgG1 BGS1/(BG0+BG+BGS1)		
IgG1 Bn		
IgG1 Bn/Fn total		

IgG1 FBn/Bn total		
IgG1 FG0n total/G0n	IgG2 FG0n total/G0n	
IgG1 FG1n total/G1n		
IgG1 FG2n total/G2n		
IgG1 FG2n/G2n		
IgG1 Fn		
IgG1 Fn total		
IgG1 Fn/Bn		
IgG1 Fucosylation		
IgG1 G1S1/(G1+G1S1)		
IgG1 GS1/(G+GS1)		
IgG1 GS1/(G0+G+GS1)		
IgG1_G0	IgG2_G0	
IgG1_G0F/IgG1_G0	IgG2_G0F/IgG2_G0	
IgG1_G0FN/IgG1_G0N		
IgG1_G0n	IgG2_G0n	
IgG1_G0N		
IgG1_G0Nn		
IgG1_G1		
IgG1_G1F/IgG1_G1		
IgG1_G1Fn		
IgG1_G1FN/IgG1_G1N		
IgG1_G1n		
IgG1_G1N		IgG1_G1N(20)/IgG2_G1N(20)
IgG1_G1Nn		
IgG1_G1NS1/IgG1_G1N		
IgG1_G1S1/IgG1_G1		
IgG1_G2		IgG1_G2(20)/IgG2_G2(20)
IgG1_G2F/IgG1_G2		
IgG1_G2FS1/IgG1_G2S1		
IgG1_G2n		
IgG1_G2N/IgG1_G1N		
	IgG2_G2/IgG2_G1	

Suppl. Table 7d: IgG glycan traits associated to variant on chromosome 22 within the *MGAT3* region

***MGAT3* (chromosome 22)**			
**IgG1**	**IgG2**	**IgG4**	**Subclass Comparison**
IgG1 BG0n total/G0n	IgG2 BG0n total/G0n		
IgG1 BG1n total/G1n			
IgG1 BG2n total/G2n			
IgG1 Bisecting_GlcNAc			
IgG1 Bn total			
IgG1 FBG0n/G0n	IgG2 FBG0n/G0n		
IgG1 FBG1n/G1n			
IgG1 FBG2n/G2n			
IgG1 FBn			
IgG1 FBn/Fn			
IgG1 FBn/Fn total			
IgG1 FG0n/G0n			
IgG1 Fn/Bn total			

IgG1_G0FN		IgG4_G0FN	
IgG1_G0FN/IgG1_G0F	IgG2_G0FN/IgG2_G0F	IgG4_G0FN/IgG4_G0F	
IgG1_G0FNn	IgG2_G0FNn	IgG4_G0FNn	
IgG1_G1FN		IgG4_G1FN	IgG1_G1FN(10)/IgG4_G1FN<br>IgG2_G1FN(10)/IgG4_G1FN
IgG1_G1FN/IgG1_G1F		IgG4_G1FN/IgG4_G1F	
IgG1_G1FNn		IgG4_G1FNn	
IgG1_G2FN/IgG1_G2F		IgG4_G2FN/IgG4_G2F	
		IgG4_G0F	IgG2_G0F(10)/IgG4_G0F
		IgG4_G2FN	IgG2_G2FN(10)/IgG4_G2FN
	IgG2_G0FN(10)/(IgG1_G0FN(10) + IgG2_G0FN(10) +IgG4_G0FN(10))		
	IgG2_G1FN(10)/(IgG1_G1FN(10) + IgG2_G1FN(10) +IgG4_G1FN(10))	IgG4_G1FN(10)/(IgG1_G1FN(10) + IgG2_G1FN(10) +IgG4_G1FN(10))	
	IgG2_G2FN(10)/(IgG1_G2FN(10) + IgG2_G2FN(10) +IgG4_G2FN(10))	IgG4_G2FN(10)/(IgG1_G2FN(10) + IgG2_G2FN(10) +IgG4_G2FN(10))	
		IgG4 Bisecting_GlcNAc	
		IgG4_G0Fn	
		IgG4_G2FNn	

Suppl. Table 7e: IgG glycan traits associated to variant on chromosome 22 within the *SMARCB1 – DERL3* region

***SMARCB1 – DERL3* (chromosome 22)**	
**IgG1**	**IgG2**
IgG1 BG0n total/G0n	IgG2 BG0n total/G0n
IgG1 BG1n total/G1n	IgG2 BG1n total/G1n
	IgG2 BG2n total/G2n
IgG1 Bisecting_GlcNAc	IgG2 Bisecting_GlcNAc
IgG1 Bn total	IgG2 Bn total
IgG1 FBG0n/G0n	IgG2 FBG0n/G0n
IgG1 FBG1n/G1n	IgG2 FBG1n/G1n
IgG1 FBG2n/G2n	IgG2 FBG2n/G2n
IgG1 FBn	IgG2 FBn
IgG1 FBn/Fn	IgG2 FBn/Fn
IgG1 FBn/Fn total	IgG2 FBn/Fn total
IgG1 Fn/Bn total	IgG2 Fn/Bn total
IgG1_G0FN/IgG1_G0F	IgG2_G0FN/IgG2_G0F
IgG1_G1FN	IgG2_G1FN
IgG1_G1FN/IgG1_G1F	IgG2_G1FN/IgG2_G1F
IgG1_G1FNn	
	IgG2_G2FN/IgG2_G2F
	IgG2_G0FNn

Suppl. Table 7e: IgG glycan traits associated to variant on chromosome 7 within the *RUNX3* region

***IKZF1* (chromosome 7)**
**IgG1**
IgG1 BG0n/G0n
IgG1 Bn
IgG1 Bn/Fn total

IgG1 Fn/Bn
IgG1 BG1n/G1n
IgG1_G0N
IgG1_G0Nn
IgG1_G1N
IgG1_G1N/IgG1_G1
IgG1_G1Nn
IgG1 FBn/Bn total

Suppl. Table 7f: IgG glycan traits associated to variant on chromosome 1 within the *RUNX3* region

***RUNX3* (chromosome 1)**	
**IgG2**	**IgG4**
IgG2_G0FN	
IgG2_G0FNn	
IgG2_G1Fn	
IgG2_G0n	
IgG2_G1F	
IgG2_G1F/IgG2_G0F	
IgG2_G1n	
IgG2_G2F	IgG4_G2F
	IgG4_G2Fn
