# Supplementary material for: Genome-Wide Association Study on Immunoglobulin G Glycosylation Patterns
Source: Front Immunol. 2018 Feb 26;9:277. doi: 10.3389/fimmu.2018.00277 (PMC5834439; doi:10.3389/fimmu.2018.00277)
Supplement: Table S7 — Complete list of results for subclass comparisons of immunoglobulin G (IgG) glycopeptide traits. [file Table_7.PDF]

Subclass Comparisons of SNP-Glycan Associations on Meta-Analyzed Data

IgG Glycan Trait	SNP	Chromosome	Position	*p* (IgG1 vs IgG2/3)	*p* (IgG1 vs. IgG4)	*p* (IgG2/3 vs. IgG4)
BG1n/G1n	rs11847263	14	65775695	2.28E-08	1	1
BG1n/G1n	rs1760978	14	65771047	1.49E-07	1	1
BG1S1/(BG1+BG1S1)	rs10138570	14	65860038	1.29E-07	1	1
BG1S1/(BG1+BG1S1)	rs11158592	14	65859968	1.94E-07	1	1
BG1S1/(BG1+BG1S1)	rs11158593	14	65859984	1.88E-07	1	1
BG1S1/(BG1+BG1S1)	rs7152441	14	65827903	3.54E-07	1	1
Bisecting_GlcNAc	rs1005522	22	39845898	5.76E-05	0.064857372	4.77E-09
Bisecting_GlcNAc	rs1007337	22	39781585	0.001703348	0.020413896	5.15E-08
Bisecting_GlcNAc	rs1010169	22	39778167	0.001469671	0.020373744	4.02E-08
Bisecting_GlcNAc	rs1010170	22	39778327	0.001392015	0.021194345	4.13E-08
Bisecting_GlcNAc	rs10644269	22	39836972	8.21E-05	0.042941547	2.57E-09
Bisecting_GlcNAc	rs113200473	22	39853740	9.03E-05	0.081805369	1.61E-08
Bisecting_GlcNAc	rs1557541	22	39851970	9.49E-05	0.040733913	2.85E-09
Bisecting_GlcNAc	rs1557542	22	39852350	9.40E-05	0.042477826	3.12E-09
Bisecting_GlcNAc	rs1557543	22	39852648	6.56E-05	0.049807295	2.77E-09
Bisecting_GlcNAc	rs1972280	22	39831986	9.41E-05	0.04235485	3.04E-09
Bisecting_GlcNAc	rs2008174	22	39860130	8.86E-05	0.043938774	3.00E-09
Bisecting_GlcNAc	rs2142848	22	39785242	0.000751277	0.028878486	3.01E-08
Bisecting_GlcNAc	rs2413590	22	39790191	0.000639498	0.032098332	2.97E-08
Bisecting_GlcNAc	rs2413592	22	39858196	8.46E-05	0.049328299	3.90E-09
Bisecting_GlcNAc	rs2899318	22	39837625	9.75E-05	0.037493072	2.36E-09
Bisecting_GlcNAc	rs35509952	22	39800563	0.000802374	0.029551202	3.39E-08
Bisecting_GlcNAc	rs3959642	22	39860589	8.69E-05	0.044712358	3.04E-09
Bisecting_GlcNAc	rs4321460	22	39793655	0.000722724	0.041058919	6.40E-08
Bisecting_GlcNAc	rs4337572	22	39800704	0.000840827	0.027828272	3.20E-08
Bisecting_GlcNAc	rs4384886	22	39793766	0.00070185	0.032953378	3.64E-08
Bisecting_GlcNAc	rs4386422	22	39793734	0.001106651	0.035700363	8.57E-08
Bisecting_GlcNAc	rs4429561	22	39799789	0.000762144	0.03676581	5.29E-08
Bisecting_GlcNAc	rs4821889	22	39777254	0.001768016	0.021474965	5.94E-08
Bisecting_GlcNAc	rs4821890	22	39777523	0.001752436	0.021624409	5.94E-08
Bisecting_GlcNAc	rs4821891	22	39785381	0.000737242	0.030051768	3.19E-08
Bisecting_GlcNAc	rs4821892	22	39795683	0.00063343	0.032521678	3.01E-08
Bisecting_GlcNAc	rs4821893	22	39797779	0.000704594	0.028668681	2.68E-08
Bisecting_GlcNAc	rs4821894	22	39809820	0.000652896	0.032516527	3.15E-08
Bisecting_GlcNAc	rs4821895	22	39823015	0.000811924	0.026446116	2.80E-08
Bisecting_GlcNAc	rs4821896	22	39833437	9.31E-05	0.041844344	2.90E-09
Bisecting_GlcNAc	rs4821897	22	39835587	9.02E-05	0.036812733	2.01E-09
Bisecting_GlcNAc	rs4821898	22	39838352	9.80E-05	0.03589036	2.14E-09
Bisecting_GlcNAc	rs5750808	22	39790987	0.000639498	0.032098332	2.97E-08

Bisecting_GlcNAc	rs5750809	22	39791491	0.000846514	0.039333668	7.02E-08
Bisecting_GlcNAc	rs5750810	22	39792943	0.00063343	0.03230941	2.97E-08
Bisecting_GlcNAc	rs5750811	22	39793066	0.00063343	0.03230941	2.97E-08
Bisecting_GlcNAc	rs5750812	22	39793079	0.00070185	0.032315278	3.48E-08
Bisecting_GlcNAc	rs5750813	22	39795228	0.000621456	0.033165663	3.05E-08
Bisecting_GlcNAc	rs5750814	22	39797987	0.000825245	0.028203471	3.20E-08
Bisecting_GlcNAc	rs5750815	22	39798449	0.000671855	0.031563915	2.98E-08
Bisecting_GlcNAc	rs5750816	22	39810379	0.000875189	0.025465122	2.80E-08
Bisecting_GlcNAc	rs5750818	22	39820885	0.000774567	0.029535822	3.21E-08
Bisecting_GlcNAc	rs5750820	22	39825322	0.000758386	0.028774442	2.93E-08
Bisecting_GlcNAc	rs5750821	22	39825492	0.000794882	0.02737562	2.92E-08
Bisecting_GlcNAc	rs5750822	22	39826788	0.000787455	0.027010622	2.79E-08
Bisecting_GlcNAc	rs5750823	22	39829973	0.000107018	0.034581135	2.22E-09
Bisecting_GlcNAc	rs5750825	22	39831278	0.000120425	0.031046662	2.04E-09
Bisecting_GlcNAc	rs5750828	22	39835083	7.39E-05	0.051984922	3.83E-09
Bisecting_GlcNAc	rs5750829	22	39838018	8.32E-05	0.052631943	4.41E-09
Bisecting_GlcNAc	rs5750830	22	39840828	6.04E-05	0.037574288	1.25E-09
Bisecting_GlcNAc	rs5750833	22	39843091	8.76E-05	0.03207785	1.37E-09
Bisecting_GlcNAc	rs5757647	22	39775047	0.001688265	0.019709306	4.71E-08
Bisecting_GlcNAc	rs5757648	22	39775156	0.00161468	0.020129465	4.58E-08
Bisecting_GlcNAc	rs5757650	22	39778419	0.001861136	0.019709306	5.54E-08
Bisecting_GlcNAc	rs5757652	22	39781855	0.001233337	0.027479237	5.73E-08
Bisecting_GlcNAc	rs5757654	22	39794124	0.000717623	0.035260803	4.24E-08
Bisecting_GlcNAc	rs5757655	22	39797178	0.000780092	0.027642293	2.80E-08
Bisecting_GlcNAc	rs5757657	22	39798429	0.000833002	0.02801533	3.20E-08
Bisecting_GlcNAc	rs5757659	22	39812409	0.000711317	0.029733456	2.85E-08
Bisecting_GlcNAc	rs5757663	22	39821319	0.000738811	0.027995045	2.64E-08
Bisecting_GlcNAc	rs5757664	22	39821536	0.000774567	0.029535822	3.21E-08
Bisecting_GlcNAc	rs5757665	22	39821641	0.000704594	0.029733456	2.80E-08
Bisecting_GlcNAc	rs5757667	22	39822116	0.000934672	0.031383412	4.94E-08
Bisecting_GlcNAc	rs5757670	22	39829736	0.000710966	0.036634164	4.56E-08
Bisecting_GlcNAc	rs5757673	22	39837920	6.18E-05	0.049712467	2.47E-09
Bisecting_GlcNAc	rs5757675	22	39838892	8.98E-05	0.037031166	2.03E-09
Bisecting_GlcNAc	rs5757676	22	39841700	0.002711768	0.018452429	8.38E-08
Bisecting_GlcNAc	rs5757678	22	39843409	7.76E-05	0.035989751	1.62E-09
Bisecting_GlcNAc	rs5757680	22	39844793	7.26E-05	0.035655465	1.38E-09
Bisecting_GlcNAc	rs5757681	22	39845547	6.82E-05	0.036097936	1.29E-09
Bisecting_GlcNAc	rs5757682	22	39848259	6.00E-05	0.039786964	1.37E-09
Bisecting_GlcNAc	rs5757683	22	39850174	8.90E-05	0.036535544	1.93E-09
Bisecting_GlcNAc	rs5757684	22	39851584	9.49E-05	0.039764921	2.69E-09
Bisecting_GlcNAc	rs5757685	22	39855540	7.17E-05	0.055485487	4.16E-09
Bisecting_GlcNAc	rs5995735	22	39854421	6.88E-05	0.054856008	3.81E-09

Bisecting_GlcNAc	rs6001566	22	39774448	0.001592713	0.021032016	4.90E-08
Bisecting_GlcNAc	rs6001567	22	39775400	0.001673306	0.019709306	4.64E-08
Bisecting_GlcNAc	rs6001568	22	39775786	0.001482995	0.021342919	4.65E-08
Bisecting_GlcNAc	rs6001585	22	39812986	0.003225364	0.017983081	1.13E-07
Bisecting_GlcNAc	rs6001587	22	39819008	0.000582968	0.033820937	2.96E-08
Bisecting_GlcNAc	rs6001588	22	39819049	0.000684777	0.032091671	3.30E-08
Bisecting_GlcNAc	rs6001594	22	39837472	9.95E-05	0.037725858	2.47E-09
Bisecting_GlcNAc	rs6001595	22	39839293	5.36E-05	0.065119355	4.36E-09
Bisecting_GlcNAc	rs6001599	22	39852720	6.81E-05	0.050031536	2.96E-09
Bisecting_GlcNAc	rs6001600	22	39852921	9.40E-05	0.044022488	3.41E-09
Bisecting_GlcNAc	rs6519190	22	39774525	0.001635792	0.021926016	5.59E-08
Bisecting_GlcNAc	rs7286917	22	39860868	8.90E-05	0.061047521	7.41E-09
Bisecting_GlcNAc	rs7288760	22	39819969	0.000444834	0.024294549	9.52E-09
Bisecting_GlcNAc	rs7292066	22	39794241	0.00070599	0.034041266	4.04E-08
Bisecting_GlcNAc	rs73167342	22	39834102	6.44E-05	0.043505496	1.79E-09
Bisecting_GlcNAc	rs7364148	22	39842165	6.69E-05	0.035217629	1.16E-09
Bisecting_GlcNAc	rs738285	22	39856356	7.17E-05	0.056440987	4.35E-09
Bisecting_GlcNAc	rs738286	22	39855575	0.000102402	0.048882628	5.04E-09
Bisecting_GlcNAc	rs738287	22	39855728	7.17E-05	0.055802478	4.23E-09
Bisecting_GlcNAc	rs738289	22	39855883	7.17E-05	0.056120976	4.29E-09
Bisecting_GlcNAc	rs739141	22	39824450	0.002272882	0.027169833	1.52E-07
Bisecting_GlcNAc	rs7423	22	39781429	0.001673306	0.020648624	5.28E-08
Bisecting_GlcNAc	rs743838	22	39824707	0.000737242	0.026649835	2.44E-08
Bisecting_GlcNAc	rs756640	22	39779300	0.001718554	0.021380967	5.94E-08
Bisecting_GlcNAc	rs7949	22	39827553	0.000772794	0.02737562	2.79E-08
Bisecting_GlcNAc	rs8136980	22	39844574	0.000102934	0.031877344	1.76E-09
Bisecting_GlcNAc	rs8137426	22	39844350	6.82E-05	0.035217629	1.22E-09
Bisecting_GlcNAc	rs8138462	22	39839670	9.14E-05	0.03760152	2.31E-09
Bisecting_GlcNAc	rs909674	22	39859169	8.25E-05	0.044904598	2.97E-09
Bisecting_GlcNAc	rs9306335	22	39843537	6.95E-05	0.031095707	8.97E-10
Bisecting_GlcNAc	rs9611165	22	39775250	0.001688265	0.019709306	4.71E-08
Bisecting_GlcNAc	rs9611166	22	39775268	0.001688265	0.019709306	4.71E-08
Bisecting_GlcNAc	rs9611167	22	39775583	0.001867726	0.022540227	7.37E-08
Bisecting_GlcNAc	rs9611169	22	39783027	0.001311619	0.023324969	4.60E-08
Bisecting_GlcNAc	rs9611170	22	39784845	0.000809931	0.026829705	2.88E-08
Bisecting_GlcNAc	rs9611176	22	39838003	7.42E-05	0.0373698	1.67E-09
Bisecting_GlcNAc	rs6001582	22	39806153	0.001066936	0.026987319	4.63E-08
FBG0n/G0n	rs1005522	22	39845898	2.21E-07	1	1
FBG0n/G0n	rs1557543	22	39852648	2.87E-07	1	1
FBG0n/G0n	rs2413592	22	39858196	4.06E-07	1	1
FBG0n/G0n	rs5750830	22	39840828	3.47E-07	1	1
FBG0n/G0n	rs5757681	22	39845547	4.02E-07	1	1

FBG0n/G0n	rs5757682	22	39848259	3.16E-07	1	1
FBG0n/G0n	rs5757685	22	39855540	3.00E-07	1	1
FBG0n/G0n	rs5995735	22	39854421	2.83E-07	1	1
FBG0n/G0n	rs6001595	22	39839293	3.57E-07	1	1
FBG0n/G0n	rs6001599	22	39852720	3.02E-07	1	1
FBG0n/G0n	rs738285	22	39856356	3.00E-07	1	1
FBG0n/G0n	rs738287	22	39855728	3.04E-07	1	1
FBG0n/G0n	rs738289	22	39855883	3.04E-07	1	1
FBn/Bn total	rs10138052	14	65833440	2.61E-07	1	1
FBn/Bn total	rs10148907	14	65833372	2.61E-07	1	1
FBn/Bn total	rs11847263	14	65775695	2.62E-07	1	1
FBn/Bn total	rs60488013	14	65825854	2.81E-07	1	1
FBn/Bn total	rs66492349	14	65839489	2.50E-07	1	1
FBn/Bn total	rs10138570	14	65860038	2.28E-07	1	1
FBn/Bn total	rs11158592	14	65859968	2.49E-07	1	1
FBn/Bn total	rs11158593	14	65859984	2.15E-07	1	1
FG2n total/G2n	rs10132229	14	65777560	2.99E-07	1	1
FG2n total/G2n	rs10134589	14	65781234	9.18E-08	1	1
FG2n total/G2n	rs10137464	14	65785829	1.58E-07	1	1
FG2n total/G2n	rs10138052	14	65833440	7.39E-09	1	1
FG2n total/G2n	rs10138662	14	65779482	1.56E-07	1	1
FG2n total/G2n	rs10142617	14	65773671	2.82E-07	1	1
FG2n total/G2n	rs10144975	14	65773982	2.59E-07	1	1
FG2n total/G2n	rs10148907	14	65833372	7.39E-09	1	1
FG2n total/G2n	rs11158587	14	65782712	1.56E-07	1	1
FG2n total/G2n	rs112850324	14	66241579	2.60E-07	1	1
FG2n total/G2n	rs11847263	14	65775695	9.37E-09	1	1
FG2n total/G2n	rs17102587	14	65774477	2.30E-07	1	1
FG2n total/G2n	rs17102598	14	65784860	1.56E-07	1	1
FG2n total/G2n	rs1760978	14	65771047	9.83E-09	1	1
FG2n total/G2n	rs1950557	14	66201757	8.52E-08	1	1
FG2n total/G2n	rs28740895	14	65783523	1.53E-07	1	1
FG2n total/G2n	rs35206478	14	66223213	7.85E-08	1	1
FG2n total/G2n	rs3742597	14	66200177	7.02E-08	1	1
FG2n total/G2n	rs57071950	14	65788437	1.99E-07	1	1
FG2n total/G2n	rs58102398	14	66250157	1.08E-07	1	1
FG2n total/G2n	rs59853675	14	66250679	8.97E-08	1	1
FG2n total/G2n	rs60273099	14	66214850	6.92E-08	1	1
FG2n total/G2n	rs60488013	14	65825854	8.19E-09	1	1
FG2n total/G2n	rs60734251	14	66250274	1.08E-07	1	1
FG2n total/G2n	rs61298903	14	66218595	6.19E-08	1	1
FG2n total/G2n	rs61348705	14	66111019	1.44E-07	1	1

FG2n total/G2n	rs6573598	14	65783019	1.56E-07	1	1
FG2n total/G2n	rs6573599	14	65783127	1.58E-07	1	1
FG2n total/G2n	rs6573600	14	65783270	1.70E-07	1	1
FG2n total/G2n	rs6573602	14	65784610	1.56E-07	1	1
FG2n total/G2n	rs6573604	14	65787941	1.86E-07	1	1
FG2n total/G2n	rs66492349	14	65839489	1.08E-08	1	1
FG2n total/G2n	rs67662065	14	66250999	7.87E-08	1	1
FG2n total/G2n	rs7151212	14	65781622	1.61E-07	1	1
FG2n total/G2n	rs7151301	14	65781496	1.58E-07	1	1
FG2n total/G2n	rs8004239	14	65783460	1.67E-07	1	1
FG2n total/G2n	rs8006316	14	65787115	1.69E-07	1	1
FG2n total/G2n	rs8010759	14	65784559	3.64E-07	1	1
FG2n total/G2n	rs8012054	14	65774762	2.06E-07	1	1
FG2n total/G2n	rs8013568	14	65774875	2.01E-07	1	1
FG2n total/G2n	rs8017974	14	65775187	1.83E-07	1	1
FG2n total/G2n	rs8019473	14	65779128	1.55E-07	1	1
FG2n total/G2n	rs8019767	14	65782785	1.56E-07	1	1
FG2n total/G2n	rs10138570	14	65860038	8.90E-10	1	1
FG2n total/G2n	rs111568962	14	65897898	2.76E-07	1	1
FG2n total/G2n	rs11158591	14	65855762	1.39E-09	1	1
FG2n total/G2n	rs11158592	14	65859968	8.90E-10	1	1
FG2n total/G2n	rs11158593	14	65859984	8.45E-10	1	1
FG2n total/G2n	rs11158596	14	65911975	2.34E-07	1	1
FG2n total/G2n	rs11158602	14	66071733	2.69E-07	1	1
FG2n total/G2n	rs11620749	14	65899804	2.80E-07	1	1
FG2n total/G2n	rs11621121	14	65822493	4.61E-09	1	1
FG2n total/G2n	rs11621604	14	65840774	9.76E-09	1	1
FG2n total/G2n	rs11621680	14	66014681	2.51E-07	1	1
FG2n total/G2n	rs11622014	14	66041645	3.16E-07	1	1
FG2n total/G2n	rs11622271	14	66149065	4.07E-07	1	1
FG2n total/G2n	rs11622829	14	66191782	4.20E-07	1	1
FG2n total/G2n	rs11623920	14	65819314	5.34E-09	1	1
FG2n total/G2n	rs11627084	14	65978836	3.85E-07	1	1
FG2n total/G2n	rs11627184	14	66121443	4.19E-07	1	1
FG2n total/G2n	rs11627185	14	66121492	4.19E-07	1	1
FG2n total/G2n	rs11627402	14	65841322	6.02E-08	1	1
FG2n total/G2n	rs11627598	14	65797766	2.66E-08	1	1
FG2n total/G2n	rs11628196	14	65891241	2.59E-07	1	1
FG2n total/G2n	rs12586842	14	66145022	3.98E-07	1	1
FG2n total/G2n	rs12589698	14	65920435	1.79E-07	1	1
FG2n total/G2n	rs12878872	14	65804665	3.99E-09	1	1
FG2n total/G2n	rs12879202	14	65977182	2.41E-07	1	1

FG2n total/G2n	rs12879971	14	65901604	2.50E-07	1	1
FG2n total/G2n	rs12880725	14	65799805	2.50E-08	1	1
FG2n total/G2n	rs12881911	14	65831221	9.29E-09	1	1
FG2n total/G2n	rs12882269	14	65847144	7.20E-09	1	1
FG2n total/G2n	rs12883815	14	65923367	1.71E-07	1	1
FG2n total/G2n	rs12886005	14	65809247	5.69E-09	1	1
FG2n total/G2n	rs12886168	14	65809286	1.09E-08	1	1
FG2n total/G2n	rs12887134	14	66045543	3.45E-07	1	1
FG2n total/G2n	rs12890123	14	65998069	2.50E-07	1	1
FG2n total/G2n	rs12890978	14	65982890	2.46E-07	1	1
FG2n total/G2n	rs12892058	14	65903441	1.62E-07	1	1
FG2n total/G2n	rs12893094	14	65789077	2.62E-08	1	1
FG2n total/G2n	rs12895074	14	65976793	3.31E-07	1	1
FG2n total/G2n	rs1815654	14	65855232	2.12E-09	1	1
FG2n total/G2n	rs1953416	14	65878807	1.43E-07	1	1
FG2n total/G2n	rs1954052	14	65805709	5.27E-09	1	1
FG2n total/G2n	rs2002692	14	65899454	2.80E-07	1	1
FG2n total/G2n	rs2149841	14	66010319	2.17E-07	1	1
FG2n total/G2n	rs2184603	14	65930670	2.83E-07	1	1
FG2n total/G2n	rs2411816	14	65994880	4.00E-07	1	1
FG2n total/G2n	rs2411822	14	65878395	2.23E-07	1	1
FG2n total/G2n	rs3825640	14	65961204	2.13E-07	1	1
FG2n total/G2n	rs4456409	14	65897967	2.11E-07	1	1
FG2n total/G2n	rs4899174	14	65811400	5.69E-09	1	1
FG2n total/G2n	rs4899179	14	65926748	2.88E-07	1	1
FG2n total/G2n	rs4902385	14	65778179	1.59E-07	1	1
FG2n total/G2n	rs4902386	14	65778290	1.57E-07	1	1
FG2n total/G2n	rs4902391	14	65815979	8.41E-09	1	1
FG2n total/G2n	rs4902393	14	65839514	8.02E-09	1	1
FG2n total/G2n	rs4902400	14	65957115	3.56E-07	1	1
FG2n total/G2n	rs4902407	14	66074635	3.60E-07	1	1
FG2n total/G2n	rs7152441	14	65827903	1.29E-07	1	1
FG2n total/G2n	rs7153297	14	66003466	2.25E-07	1	1
FG2n total/G2n	rs7155541	14	66046242	3.26E-07	1	1
FG2n total/G2n	rs7155963	14	66076029	1.88E-07	1	1
FG2n total/G2n	rs7157006	14	65828207	6.48E-09	1	1
FG2n total/G2n	rs7158347	14	65828955	8.15E-09	1	1
FG2n total/G2n	rs747541	14	65805410	1.07E-08	1	1
FG2n total/G2n	rs8022094	14	65846841	7.31E-09	1	1
FG2n total/G2n	rs867972	14	65895761	1.43E-07	1	1
FG2n total/G2n	rs883081	14	65880621	1.29E-07	1	1
FG2n total/G2n	rs883082	14	65880940	1.55E-07	1	1

FG2n total/G2n	rs899959	14	65791928	2.96E-08	1	1
FG2n total/G2n	rs9796385	14	66101219	3.90E-07	1	1
FG2n/G2n	rs10138052	14	65833440	4.09E-08	1	1
FG2n/G2n	rs10148907	14	65833372	4.04E-08	1	1
FG2n/G2n	rs11847263	14	65775695	1.32E-09	1	1
FG2n/G2n	rs28740895	14	65783523	2.74E-08	1	1
FG2n/G2n	rs60488013	14	65825854	4.44E-08	1	1
FG2n/G2n	rs66492349	14	65839489	5.84E-08	1	1
FG2n/G2n	rs10138570	14	65860038	1.98E-08	1	1
FG2n/G2n	rs11158591	14	65855762	2.45E-09	1	1
FG2n/G2n	rs11158592	14	65859968	1.94E-08	1	1
FG2n/G2n	rs11158593	14	65859984	2.04E-08	1	1
FG2n/G2n	rs11621121	14	65822493	4.30E-09	1	1
FG2n/G2n	rs11621604	14	65840774	1.24E-08	1	1
FG2n/G2n	rs11623920	14	65819314	4.90E-09	1	1
FG2n/G2n	rs11627402	14	65841322	4.65E-08	1	1
FG2n/G2n	rs11627598	14	65797766	2.43E-09	1	1
FG2n/G2n	rs12589698	14	65920435	3.45E-07	1	1
FG2n/G2n	rs12878872	14	65804665	3.62E-09	1	1
FG2n/G2n	rs12880725	14	65799805	2.13E-09	1	1
FG2n/G2n	rs12881911	14	65831221	1.16E-08	1	1
FG2n/G2n	rs12882269	14	65847144	9.46E-09	1	1
FG2n/G2n	rs12883815	14	65923367	3.26E-07	1	1
FG2n/G2n	rs12886005	14	65809247	5.50E-09	1	1
FG2n/G2n	rs12886168	14	65809286	6.27E-09	1	1
FG2n/G2n	rs12890978	14	65982890	4.21E-07	1	1
FG2n/G2n	rs12892058	14	65903441	3.35E-07	1	1
FG2n/G2n	rs12893094	14	65789077	2.18E-09	1	1
FG2n/G2n	rs1815654	14	65855232	3.80E-09	1	1
FG2n/G2n	rs1953416	14	65878807	3.07E-07	1	1
FG2n/G2n	rs1954052	14	65805709	5.25E-09	1	1
FG2n/G2n	rs4456409	14	65897967	4.03E-07	1	1
FG2n/G2n	rs4899174	14	65811400	5.41E-09	1	1
FG2n/G2n	rs4902391	14	65815979	6.27E-09	1	1
FG2n/G2n	rs4902393	14	65839514	7.40E-09	1	1
FG2n/G2n	rs7152441	14	65827903	3.60E-07	1	1
FG2n/G2n	rs7153297	14	66003466	3.93E-07	1	1
FG2n/G2n	rs7157006	14	65828207	8.55E-09	1	1
FG2n/G2n	rs7158347	14	65828955	1.11E-08	1	1
FG2n/G2n	rs747541	14	65805410	5.77E-09	1	1
FG2n/G2n	rs8022094	14	65846841	8.19E-09	1	1
FG2n/G2n	rs867972	14	65895761	2.98E-07	1	1

FG2n/G2n	rs883081	14	65880621	3.21E-07	1	1
FG2n/G2n	rs883082	14	65880940	4.00E-07	1	1
FG2n/G2n	rs899959	14	65791928	2.42E-09	1	1
_G0FN/G0F	rs1005522	22	39845898	2.82E-07	0.15807287	7.35E-11
_G0FN/G0F	rs1007337	22	39781585	2.24E-05	0.132385795	1.03E-08
_G0FN/G0F	rs1010169	22	39778167	1.83E-05	0.132385795	7.88E-09
_G0FN/G0F	rs1010170	22	39778327	1.77E-05	0.133073246	7.63E-09
_G0FN/G0F	rs10644269	22	39836972	5.90E-07	0.119717709	6.79E-11
_G0FN/G0F	rs113200473	22	39853740	5.37E-07	0.169456804	1.95E-10
_G0FN/G0F	rs1557541	22	39851970	4.52E-07	0.121653678	5.07E-11
_G0FN/G0F	rs1557542	22	39852350	4.83E-07	0.12601977	5.86E-11
_G0FN/G0F	rs1557543	22	39852648	2.87E-07	0.136312323	3.93E-11
_G0FN/G0F	rs1972280	22	39831986	8.29E-07	0.118143182	1.01E-10
_G0FN/G0F	rs2008174	22	39860130	4.02E-07	0.131516301	5.35E-11
_G0FN/G0F	rs2142848	22	39785242	5.95E-06	0.13732777	2.06E-09
_G0FN/G0F	rs2413590	22	39790191	5.00E-06	0.151964495	2.39E-09
_G0FN/G0F	rs2413592	22	39858196	3.90E-07	0.130076547	4.96E-11
_G0FN/G0F	rs2899318	22	39837625	6.23E-07	0.112673581	5.96E-11
_G0FN/G0F	rs35509952	22	39800563	6.82E-06	0.135965693	2.51E-09
_G0FN/G0F	rs3959642	22	39860589	4.30E-07	0.133468654	5.78E-11
_G0FN/G0F	rs4321460	22	39793655	7.87E-06	0.170600214	6.16E-09
_G0FN/G0F	rs4337572	22	39800704	7.24E-06	0.131111283	2.26E-09
_G0FN/G0F	rs4384886	22	39793766	5.56E-06	0.150465283	2.52E-09
_G0FN/G0F	rs4386422	22	39793734	8.57E-06	0.159642216	5.48E-09
_G0FN/G0F	rs4429561	22	39799789	8.17E-06	0.160459748	5.32E-09
_G0FN/G0F	rs4820377	22	39770780	0.000183705	0.164624847	3.11E-07
_G0FN/G0F	rs4821888	22	39770597	0.000200048	0.16379178	3.56E-07
_G0FN/G0F	rs4821889	22	39777254	2.24E-05	0.129759944	9.66E-09
_G0FN/G0F	rs4821890	22	39777523	2.21E-05	0.130434255	9.66E-09
_G0FN/G0F	rs4821891	22	39785381	6.10E-06	0.143068802	2.55E-09
_G0FN/G0F	rs4821892	22	39795683	5.06E-06	0.152724407	2.47E-09
_G0FN/G0F	rs4821893	22	39797779	6.07E-06	0.13658979	2.19E-09
_G0FN/G0F	rs4821894	22	39809820	6.38E-06	0.131746829	2.09E-09
_G0FN/G0F	rs4821895	22	39823015	7.99E-06	0.128419446	2.41E-09
_G0FN/G0F	rs4821896	22	39833437	7.72E-07	0.114051961	7.57E-11
_G0FN/G0F	rs4821897	22	39835587	5.79E-07	0.113257609	5.41E-11
_G0FN/G0F	rs4821898	22	39838352	6.56E-07	0.112815137	6.40E-11
_G0FN/G0F	rs5750808	22	39790987	5.00E-06	0.151964495	2.39E-09
_G0FN/G0F	rs5750809	22	39791491	6.13E-06	0.17512521	4.90E-09
_G0FN/G0F	rs5750810	22	39792943	5.00E-06	0.151964495	2.39E-09
_G0FN/G0F	rs5750811	22	39793066	5.00E-06	0.151964495	2.39E-09
_G0FN/G0F	rs5750812	22	39793079	5.49E-06	0.148224077	2.36E-09

_G0FN/G0F	rs5750813	22	39795228	4.94E-06	0.155021495	2.51E-09
_G0FN/G0F	rs5750814	22	39797987	6.80E-06	0.133158724	2.19E-09
_G0FN/G0F	rs5750815	22	39798449	6.07E-06	0.145210196	2.65E-09
_G0FN/G0F	rs5750816	22	39810379	8.37E-06	0.125661755	2.44E-09
_G0FN/G0F	rs5750818	22	39820885	7.03E-06	0.13585072	2.43E-09
_G0FN/G0F	rs5750820	22	39825322	7.44E-06	0.134578321	2.72E-09
_G0FN/G0F	rs5750821	22	39825492	7.81E-06	0.129088343	2.42E-09
_G0FN/G0F	rs5750822	22	39826788	7.62E-06	0.128419446	2.31E-09
_G0FN/G0F	rs5750823	22	39829973	7.92E-07	0.107367893	6.83E-11
_G0FN/G0F	rs5750825	22	39831278	9.37E-07	0.09988511	6.74E-11
_G0FN/G0F	rs5750828	22	39835083	4.62E-07	0.122536288	5.34E-11
_G0FN/G0F	rs5750829	22	39838018	5.37E-07	0.14230256	1.05E-10
_G0FN/G0F	rs5750830	22	39840828	3.16E-07	0.114840868	2.64E-11
_G0FN/G0F	rs5750833	22	39843091	4.62E-07	0.107428671	3.47E-11
_G0FN/G0F	rs5757642	22	39764824	0.000219532	0.163828773	3.89E-07
_G0FN/G0F	rs5757647	22	39775047	2.09E-05	0.132385795	9.51E-09
_G0FN/G0F	rs5757648	22	39775156	1.99E-05	0.136552037	9.81E-09
_G0FN/G0F	rs5757650	22	39778419	2.43E-05	0.130339943	1.11E-08
_G0FN/G0F	rs5757652	22	39781855	1.43E-05	0.166883063	1.18E-08
_G0FN/G0F	rs5757654	22	39794124	5.75E-06	0.146840618	2.56E-09
_G0FN/G0F	rs5757655	22	39797178	6.48E-06	0.130434255	1.92E-09
_G0FN/G0F	rs5757657	22	39798429	6.80E-06	0.133158724	2.19E-09
_G0FN/G0F	rs5757659	22	39812409	6.37E-06	0.133763458	2.04E-09
_G0FN/G0F	rs5757663	22	39821319	6.77E-06	0.126984826	1.88E-09
_G0FN/G0F	rs5757664	22	39821536	6.94E-06	0.136552037	2.43E-09
_G0FN/G0F	rs5757665	22	39821641	6.29E-06	0.13449767	2.14E-09
_G0FN/G0F	rs5757667	22	39822116	9.91E-06	0.124040088	2.92E-09
_G0FN/G0F	rs5757670	22	39829736	6.15E-06	0.181708698	5.28E-09
_G0FN/G0F	rs5757673	22	39837920	4.41E-07	0.118934357	4.22E-11
_G0FN/G0F	rs5757675	22	39838892	5.35E-07	0.112235015	4.75E-11
_G0FN/G0F	rs5757676	22	39841700	6.72E-05	0.085398937	1.26E-08
_G0FN/G0F	rs5757678	22	39843409	4.06E-07	0.118995555	4.10E-11
_G0FN/G0F	rs5757680	22	39844793	3.80E-07	0.105773179	2.55E-11
_G0FN/G0F	rs5757681	22	39845547	3.66E-07	0.111443707	2.72E-11
_G0FN/G0F	rs5757682	22	39848259	2.74E-07	0.112085689	2.03E-11
_G0FN/G0F	rs5757683	22	39850174	4.82E-07	0.110946056	4.06E-11
_G0FN/G0F	rs5757684	22	39851584	4.64E-07	0.119240785	4.91E-11
_G0FN/G0F	rs5757685	22	39855540	2.86E-07	0.147259005	5.51E-11
_G0FN/G0F	rs5995735	22	39854421	2.72E-07	0.145163693	4.85E-11
_G0FN/G0F	rs6001566	22	39774448	1.81E-05	0.127753247	6.97E-09
_G0FN/G0F	rs6001567	22	39775400	2.06E-05	0.133073246	9.51E-09
_G0FN/G0F	rs6001568	22	39775786	1.85E-05	0.137256144	8.92E-09

_G0FN/G0F	rs6001585	22	39812986	9.28E-05	0.095131343	2.70E-08
_G0FN/G0F	rs6001587	22	39819008	5.50E-06	0.144591764	2.15E-09
_G0FN/G0F	rs6001588	22	39819049	6.63E-06	0.140132361	2.65E-09
_G0FN/G0F	rs6001594	22	39837472	6.31E-07	0.112673581	6.06E-11
_G0FN/G0F	rs6001595	22	39839293	3.96E-07	0.16491702	1.21E-10
_G0FN/G0F	rs6001599	22	39852720	2.79E-07	0.133028437	3.50E-11
_G0FN/G0F	rs6001600	22	39852921	4.77E-07	0.129169018	6.27E-11
_G0FN/G0F	rs6519190	22	39774525	1.81E-05	0.135230832	8.28E-09
_G0FN/G0F	rs7286917	22	39860868	4.70E-07	0.158793458	1.25E-10
_G0FN/G0F	rs7288760	22	39819969	3.92E-06	0.088555891	3.25E-10
_G0FN/G0F	rs7292066	22	39794241	5.61E-06	0.162856917	3.24E-09
_G0FN/G0F	rs73167342	22	39834102	4.10E-07	0.111729797	3.18E-11
_G0FN/G0F	rs7364148	22	39842165	3.46E-07	0.106322746	2.30E-11
_G0FN/G0F	rs738285	22	39856356	2.99E-07	0.15010193	5.79E-11
_G0FN/G0F	rs738286	22	39855575	4.28E-07	0.139687867	7.67E-11
_G0FN/G0F	rs738287	22	39855728	3.03E-07	0.147979472	5.61E-11
_G0FN/G0F	rs738289	22	39855883	3.07E-07	0.149391879	5.89E-11
_G0FN/G0F	rs738290	22	39856032	0.000121724	0.210703139	3.64E-07
_G0FN/G0F	rs739141	22	39824450	1.97E-05	0.138739604	9.64E-09
_G0FN/G0F	rs7423	22	39781429	2.19E-05	0.126428916	8.67E-09
_G0FN/G0F	rs743838	22	39824707	7.45E-06	0.113910057	1.52E-09
_G0FN/G0F	rs756640	22	39779300	2.29E-05	0.129759944	9.97E-09
_G0FN/G0F	rs7949	22	39827553	7.73E-06	0.13188034	2.51E-09
_G0FN/G0F	rs8136980	22	39844574	5.67E-07	0.101989061	3.88E-11
_G0FN/G0F	rs8137426	22	39844350	3.46E-07	0.106874575	2.34E-11
_G0FN/G0F	rs8138462	22	39839670	5.54E-07	0.112235015	5.06E-11
_G0FN/G0F	rs909674	22	39859169	4.12E-07	0.133992716	5.87E-11
_G0FN/G0F	rs9306335	22	39843537	3.76E-07	0.093928186	1.71E-11
_G0FN/G0F	rs9611165	22	39775250	2.09E-05	0.133073246	9.66E-09
_G0FN/G0F	rs9611166	22	39775268	2.09E-05	0.133073246	9.66E-09
_G0FN/G0F	rs9611167	22	39775583	2.40E-05	0.138032296	1.28E-08
_G0FN/G0F	rs9611169	22	39783027	1.81E-05	0.136477522	8.50E-09
_G0FN/G0F	rs9611170	22	39784845	7.62E-06	0.129138067	2.47E-09
_G0FN/G0F	rs9611176	22	39838003	4.28E-07	0.107678352	3.10E-11
_G0FN/G0F	rs6001582	22	39806153	9.83E-06	0.133846678	3.60E-09
_G0n	rs1003401	14	66237720	0.075789151	4.34E-12	2.51E-07
_G0n	rs10138570	14	65860038	0.01968314	6.54E-17	1.89E-09
_G0n	rs10138671	14	65860092	0.177399764	4.48E-08	3.74E-05
_G0n	rs10483780	14	65980170	0.171822825	6.76E-15	1.28E-10
_G0n	rs10483785	14	66219517	0.164011293	5.13E-14	8.19E-10
_G0n	rs1054218	14	66209190	0.066358464	7.77E-12	5.34E-07
_G0n	rs1075566	14	66239457	0.241963926	4.04E-13	1.15E-09

_G0n	rs111568962	14	65897898	0.133745607	1.08E-13	3.09E-09
_G0n	rs11158591	14	65855762	0.259317338	1.20E-14	4.65E-11
_G0n	rs11158592	14	65859968	0.018404766	6.86E-17	2.28E-09
_G0n	rs11158593	14	65859984	0.018267313	6.86E-17	2.32E-09
_G0n	rs11158596	14	65911975	0.155707661	3.00E-14	6.68E-10
_G0n	rs11158601	14	66025363	0.164889771	1.49E-14	2.85E-10
_G0n	rs11158602	14	66071733	0.188959348	2.29E-14	2.77E-10
_G0n	rs11158611	14	66235074	0.280114277	3.64E-13	5.88E-10
_G0n	rs113131712	14	66064120	0.146196455	1.55E-14	4.89E-10
_G0n	rs11620749	14	65899804	0.158166243	3.30E-14	6.33E-10
_G0n	rs11621121	14	65822493	0.186596638	1.09E-14	1.56E-10
_G0n	rs11621604	14	65840774	0.307040556	3.40E-14	5.62E-11
_G0n	rs11621680	14	66014681	0.188022176	5.12E-13	3.69E-09
_G0n	rs11622014	14	66041645	0.131235954	6.47E-15	3.43E-10
_G0n	rs11622271	14	66149065	0.159839764	1.25E-14	2.75E-10
_G0n	rs11622829	14	66191782	0.136276469	1.66E-14	5.93E-10
_G0n	rs11623662	14	66021192	0.304553006	1.97E-08	4.45E-06
_G0n	rs11623920	14	65819314	0.186718062	9.39E-15	1.37E-10
_G0n	rs11624045	14	66108018	0.167485994	1.21E-14	2.46E-10
_G0n	rs11624104	14	66196137	0.141517098	8.61E-14	2.04E-09
_G0n	rs11625362	14	66232869	0.256069194	3.63E-13	8.44E-10
_G0n	rs11625882	14	66245199	0.241772763	4.48E-13	1.35E-09
_G0n	rs11627067	14	66182953	0.150859522	7.79E-14	1.50E-09
_G0n	rs11627084	14	65978836	0.155703615	7.39E-15	1.95E-10
_G0n	rs11627184	14	66121443	0.171882439	7.89E-15	1.45E-10
_G0n	rs11627185	14	66121492	0.171882439	7.89E-15	1.45E-10
_G0n	rs11627402	14	65841322	0.296633894	9.11E-14	1.53E-10
_G0n	rs11627578	14	66234448	0.280114277	3.71E-13	5.98E-10
_G0n	rs11627598	14	65797766	0.066522597	3.69E-13	5.45E-08
_G0n	rs11627605	14	66234313	0.282600528	3.41E-13	5.44E-10
_G0n	rs11628196	14	65891241	0.150078422	3.80E-15	1.41E-10
_G0n	rs11628611	14	65828525	0.356851754	4.52E-08	5.49E-06
_G0n	rs11628765	14	66168449	0.163213494	1.59E-14	3.12E-10
_G0n	rs11628840	14	66235642	0.269110423	3.94E-13	7.47E-10
_G0n	rs11851576	14	65900283	0.177001143	2.17E-10	5.63E-07
_G0n	rs12050182	14	66243080	0.246252783	3.44E-13	1.01E-09
_G0n	rs12050231	14	66235016	0.280114277	3.64E-13	5.88E-10
_G0n	rs12586842	14	66145022	0.169271584	7.80E-15	1.65E-10
_G0n	rs12588838	14	66162638	0.164936602	1.02E-14	2.25E-10
_G0n	rs12589698	14	65920435	0.120306741	2.72E-14	1.45E-09
_G0n	rs12878872	14	65804665	0.126229944	4.55E-15	2.71E-10
_G0n	rs12879202	14	65977182	0.127750902	4.55E-14	1.65E-09

_G0n	rs12879805	14	66047795	0.166602961	9.00E-15	1.81E-10
_G0n	rs12879971	14	65901604	0.146932167	2.56E-14	7.25E-10
_G0n	rs12880725	14	65799805	0.06332708	3.20E-13	5.54E-08
_G0n	rs12881755	14	65801811	0.285324747	1.65E-09	6.97E-07
_G0n	rs12881911	14	65831221	0.245848699	1.43E-14	6.71E-11
_G0n	rs12882200	14	66157861	0.166622605	8.62E-15	1.93E-10
_G0n	rs12882269	14	65847144	0.301178037	1.06E-14	2.21E-11
_G0n	rs12883382	14	66145396	0.159839764	1.63E-14	3.41E-10
_G0n	rs12883815	14	65923367	0.119642495	2.50E-14	1.37E-09
_G0n	rs12886005	14	65809247	0.180361371	8.61E-15	1.45E-10
_G0n	rs12886168	14	65809286	0.180361371	1.54E-14	2.35E-10
_G0n	rs12886334	14	66094671	0.167485994	9.84E-15	2.11E-10
_G0n	rs12887134	14	66045543	0.127697675	9.62E-15	5.18E-10
_G0n	rs12887673	14	66221771	0.155710074	7.52E-14	1.34E-09
_G0n	rs12887957	14	66246592	0.251934219	3.66E-13	9.76E-10
_G0n	rs12888212	14	66200458	0.154075968	7.01E-14	1.28E-09
_G0n	rs12889002	14	66063582	0.164889771	1.39E-14	2.70E-10
_G0n	rs12890123	14	65998069	0.128399329	4.51E-14	1.74E-09
_G0n	rs12890902	14	66116622	0.170121636	1.23E-14	2.17E-10
_G0n	rs12890978	14	65982890	0.126303319	5.20E-14	2.09E-09
_G0n	rs12892058	14	65903441	0.119574986	2.78E-14	1.37E-09
_G0n	rs12893094	14	65789077	0.063868641	2.59E-13	4.62E-08
_G0n	rs12894034	14	66238763	0.253301334	5.95E-13	1.34E-09
_G0n	rs12894466	14	66240767	0.241963926	4.12E-13	1.17E-09
_G0n	rs12894902	14	66161799	0.162355799	4.80E-15	1.28E-10
_G0n	rs12895074	14	65976793	0.125268715	1.20E-13	4.20E-09
_G0n	rs12895337	14	65807442	0.272818402	1.44E-10	1.05E-07
_G0n	rs12896663	14	66104969	0.16835274	1.01E-14	2.11E-10
_G0n	rs139525066	14	66184172	0.048061062	1.04E-11	1.33E-06
_G0n	rs1535173	14	66199139	0.154888167	6.58E-14	1.20E-09
_G0n	rs1815654	14	65855232	0.199566351	1.25E-14	1.34E-10
_G0n	rs1953416	14	65878807	0.126303319	5.24E-14	2.06E-09
_G0n	rs1954052	14	65805709	0.178488666	5.24E-15	9.94E-11
_G0n	rs1958561	14	66036761	0.164889771	1.43E-14	2.74E-10
_G0n	rs1984855	14	66239257	0.075571239	4.76E-12	2.71E-07
_G0n	rs1998036	14	66138199	0.166622605	1.01E-14	2.19E-10
_G0n	rs2002692	14	65899454	0.158166243	3.30E-14	6.33E-10
_G0n	rs2064694	14	66148246	0.16322621	9.95E-15	2.29E-10
_G0n	rs2149841	14	66010319	0.120911322	5.25E-15	4.10E-10
_G0n	rs2184603	14	65930670	0.146932167	4.54E-15	1.76E-10
_G0n	rs2236067	14	66248012	0.080604132	4.09E-12	2.06E-07
_G0n	rs2268961	14	66146765	0.16322621	9.95E-15	2.29E-10

_G0n	rs2268962	14	66147273	0.164065259	1.17E-14	2.38E-10
_G0n	rs2300865	14	66120015	0.164065259	1.03E-14	2.13E-10
_G0n	rs2411351	14	66171541	0.161519977	1.59E-14	3.23E-10
_G0n	rs2411355	14	66060707	0.179834741	1.89E-14	2.82E-10
_G0n	rs2411356	14	66053161	0.33663437	1.85E-08	3.21E-06
_G0n	rs2411357	14	66051100	0.179834741	1.81E-14	2.72E-10
_G0n	rs2411404	14	66239401	0.241963926	4.29E-13	1.21E-09
_G0n	rs2411405	14	66232086	0.278876742	3.41E-13	5.74E-10
_G0n	rs2411816	14	65994880	0.164875393	5.20E-14	8.79E-10
_G0n	rs2411822	14	65878395	0.158168689	4.67E-14	9.26E-10
_G0n	rs2411823	14	65865066	0.18348718	5.27E-08	3.92E-05
_G0n	rs3783709	14	66058664	0.179834741	1.89E-14	2.82E-10
_G0n	rs3825640	14	65961204	0.118845285	4.85E-15	3.76E-10
_G0n	rs4080329	14	66233490	0.090653277	1.66E-11	4.48E-07
_G0n	rs4143898	14	66188882	0.238262472	9.65E-10	7.83E-07
_G0n	rs4359350	14	65863748	0.14130768	2.02E-09	5.99E-06
_G0n	rs4456409	14	65897967	0.125268715	5.46E-14	2.19E-09
_G0n	rs4581615	14	66055943	0.179834741	1.89E-14	2.82E-10
_G0n	rs4587890	14	65863784	0.162151869	9.08E-09	1.36E-05
_G0n	rs4899174	14	65811400	0.180361371	1.07E-14	1.73E-10
_G0n	rs4899178	14	65862987	0.166242216	9.52E-09	1.33E-05
_G0n	rs4899179	14	65926748	0.15327581	3.34E-14	7.69E-10
_G0n	rs4902385	14	65778179	0.017458085	2.82E-12	4.02E-06
_G0n	rs4902386	14	65778290	0.017390022	1.24E-12	2.24E-06
_G0n	rs4902391	14	65815979	0.175920304	2.12E-14	3.35E-10
_G0n	rs4902393	14	65839514	0.197039243	1.62E-14	1.75E-10
_G0n	rs4902400	14	65957115	0.161495748	4.38E-14	8.19E-10
_G0n	rs4902407	14	66074635	0.194654812	4.00E-14	3.95E-10
_G0n	rs4902412	14	66187067	0.265509326	4.99E-09	2.19E-06
_G0n	rs4902415	14	66235690	0.271529702	4.28E-13	7.65E-10
_G0n	rs4902416	14	66238090	0.246660513	5.15E-13	1.41E-09
_G0n	rs6573609	14	65861402	0.162963802	9.08E-09	1.34E-05
_G0n	rs6573611	14	65865255	0.186296603	5.05E-08	3.61E-05
_G0n	rs6573615	14	66046534	0.31097683	1.13E-08	2.63E-06
_G0n	rs6573624	14	66226885	0.153282967	5.39E-14	1.05E-09
_G0n	rs6573625	14	66240634	0.098274458	1.18E-11	2.87E-07
_G0n	rs6573626	14	66240695	0.255159965	4.50E-13	1.03E-09
_G0n	rs67957534	14	66124585	0.17276794	8.07E-15	1.45E-10
_G0n	rs7142018	14	66241673	0.244137076	1.52E-13	4.83E-10
_G0n	rs7142219	14	66241781	0.244926808	4.41E-13	1.18E-09
_G0n	rs7142651	14	66132721	0.161519977	1.03E-14	2.25E-10
_G0n	rs7143666	14	65809977	0.280443036	1.05E-10	7.19E-08

_G0n	rs7146742	14	66032934	0.283849247	3.75E-09	1.41E-06
_G0n	rs7146829	14	66194531	0.146911252	7.79E-14	1.64E-09
_G0n	rs7148737	14	66230032	0.078058176	1.48E-11	5.91E-07
_G0n	rs7149301	14	66230414	0.246864845	3.64E-13	9.59E-10
_G0n	rs7152441	14	65827903	0.114967203	1.07E-13	4.86E-09
_G0n	rs7153297	14	66003466	0.120437575	4.78E-14	2.31E-09
_G0n	rs7154536	14	65991737	0.224575518	5.75E-10	6.14E-07
_G0n	rs7155541	14	66046242	0.128399329	8.62E-15	4.65E-10
_G0n	rs7155963	14	66076029	0.161446517	4.68E-14	8.65E-10
_G0n	rs7156078	14	66238701	0.078171627	5.16E-12	2.85E-07
_G0n	rs7157006	14	65828207	0.239183909	1.00E-14	5.62E-11
_G0n	rs7157449	14	66240137	0.237530486	3.43E-13	1.07E-09
_G0n	rs7158347	14	65828955	0.246972039	1.44E-14	6.76E-11
_G0n	rs7158556	14	66240729	0.098822427	1.18E-11	2.83E-07
_G0n	rs7160780	14	66052713	0.305830671	1.58E-08	3.66E-06
_G0n	rs7161123	14	66052901	0.179834741	1.93E-14	2.87E-10
_G0n	rs730807	14	66239290	0.239739979	4.12E-13	1.21E-09
_G0n	rs743084	14	66232602	0.25840707	3.78E-13	8.44E-10
_G0n	rs743085	14	66068133	0.164889771	1.39E-14	2.70E-10
_G0n	rs747541	14	65805410	0.179466558	1.17E-14	1.90E-10
_G0n	rs7493004	14	66124104	0.16577883	1.03E-14	2.05E-10
_G0n	rs761830	14	66212986	0.062023152	9.40E-12	7.19E-07
_G0n	rs761831	14	66239370	0.241963926	4.29E-13	1.21E-09
_G0n	rs78230848	14	66016384	0.165694843	8.77E-14	1.33E-09
_G0n	rs8003811	14	66211250	0.062793475	1.09E-11	7.82E-07
_G0n	rs8005710	14	66065666	0.156524753	1.49E-14	3.76E-10
_G0n	rs8010876	14	66206976	0.149270615	8.66E-14	1.69E-09
_G0n	rs8012278	14	66082573	0.165728568	1.34E-14	2.82E-10
_G0n	rs8014475	14	65811537	0.281069483	2.38E-10	1.45E-07
_G0n	rs8016322	14	66051536	0.308396649	1.06E-08	2.57E-06
_G0n	rs8018278	14	66180088	0.136978305	1.29E-14	5.14E-10
_G0n	rs8022094	14	65846841	0.30245856	2.13E-14	3.99E-11
_G0n	rs8022764	14	66244655	0.204499806	4.05E-12	1.41E-08
_G0n	rs80297942	14	66100698	0.279880494	9.05E-14	1.81E-10
_G0n	rs867972	14	65895761	0.111893838	1.73E-14	1.26E-09
_G0n	rs883081	14	65880621	0.114925331	4.70E-15	3.99E-10
_G0n	rs883082	14	65880940	0.116220518	5.61E-15	4.44E-10
_G0n	rs899959	14	65791928	0.063037505	5.05E-13	7.90E-08
_G0n	rs927004	14	66200911	0.154888167	7.01E-14	1.26E-09
_G0n	rs9796385	14	66101219	0.148828756	1.15E-12	1.53E-08
_G0n	rs9972106	14	66023131	0.267869502	1.19E-08	4.34E-06
_G0n	chr14:65855901:I	14	65855901	0.044631204	1.66E-08	0.000280413

_G0n	rs10131385	14	65773555	0.01424116	6.41E-13	2.10E-06
_G0n	rs10132229	14	65777560	0.018916808	2.99E-13	6.84E-07
_G0n	rs10134589	14	65781234	0.01184411	1.04E-12	3.83E-06
_G0n	rs10135194	14	65770978	0.016976279	5.88E-13	1.46E-06
_G0n	rs10137464	14	65785829	0.015553432	1.02E-12	2.48E-06
_G0n	rs10138052	14	65833440	0.032408833	3.53E-15	9.72E-09
_G0n	rs10138151	14	65789729	0.014415442	6.53E-13	2.02E-06
_G0n	rs10138662	14	65779482	0.015644132	1.07E-12	2.53E-06
_G0n	rs10138765	14	65860239	0.048521208	2.43E-08	0.000311165
_G0n	rs10142617	14	65773671	0.016104649	8.23E-13	2.01E-06
_G0n	rs10144503	14	65784109	0.014995069	9.09E-13	2.33E-06
_G0n	rs10144975	14	65773982	0.014354091	1.05E-12	2.77E-06
_G0n	rs10147958	14	65778833	0.015063649	8.66E-13	2.23E-06
_G0n	rs10148907	14	65833372	0.032210413	3.46E-15	9.72E-09
_G0n	rs10150168	14	65791249	0.015085211	8.91E-13	2.30E-06
_G0n	rs11158587	14	65782712	0.015553432	1.04E-12	2.50E-06
_G0n	rs111950176	14	65843359	0.036808647	3.10E-09	0.000135046
_G0n	rs112850324	14	66241579	0.092249404	3.33E-12	1.36E-07
_G0n	rs11845834	14	66260602	0.563167729	1.06E-08	2.56E-07
_G0n	rs11847263	14	65775695	0.022539238	9.46E-17	1.56E-09
_G0n	rs11848443	14	66257419	0.38545793	3.17E-12	1.02E-09
_G0n	rs11848444	14	66257467	0.383941751	3.57E-12	1.15E-09
_G0n	rs11848463	14	66257294	0.38545793	3.11E-12	1.00E-09
_G0n	rs11850120	14	66260379	0.568677621	9.80E-09	2.28E-07
_G0n	rs12147994	14	65751627	0.083946313	4.03E-09	3.23E-05
_G0n	rs12436299	14	65785194	0.014926764	9.09E-13	2.35E-06
_G0n	rs1256519	14	65736324	0.132547739	1.92E-09	6.74E-06
_G0n	rs1256527	14	65740296	0.124437728	1.40E-09	6.25E-06
_G0n	rs17102587	14	65774477	0.015258934	9.91E-13	2.43E-06
_G0n	rs17102598	14	65784860	0.015553432	1.04E-12	2.50E-06
_G0n	rs1760978	14	65771047	0.043831928	1.54E-13	7.91E-08
_G0n	rs186501115	14	66159405	0.390574663	2.90E-07	1.95E-05
_G0n	rs1950557	14	66201757	0.088912857	6.76E-14	6.66E-09
_G0n	rs28459012	14	66258528	0.361639289	2.88E-12	1.24E-09
_G0n	rs28470717	14	66251050	0.434838901	7.23E-12	1.25E-09
_G0n	rs28557275	14	65790415	0.015570217	8.39E-13	2.09E-06
_G0n	rs28595734	14	65792269	0.015017022	8.91E-13	2.32E-06
_G0n	rs28616692	14	66258683	0.363100357	3.05E-12	1.28E-09
_G0n	rs28666010	14	65758148	0.098849176	2.69E-10	3.09E-06
_G0n	rs28668545	14	65858308	0.033575756	2.19E-08	0.000517246
_G0n	rs28740895	14	65783523	0.054893164	7.28E-14	2.57E-08
_G0n	rs35206478	14	66223213	0.087302658	3.60E-14	4.28E-09

_G0n	rs3742597	14	66200177	0.089822362	6.52E-14	6.29E-09
_G0n	rs3892139	14	66259865	0.631640947	5.78E-08	7.56E-07
_G0n	rs4073415	14	66259530	0.364565115	3.50E-12	1.42E-09
_G0n	rs4073416	14	66259394	0.430657281	8.70E-09	6.46E-07
_G0n	rs4131095	14	66259437	0.585360124	1.39E-08	2.76E-07
_G0n	rs4131097	14	66259165	0.126146811	1.75E-14	7.94E-10
_G0n	rs4400971	14	66254578	0.357713982	2.32E-12	1.08E-09
_G0n	rs4439682	14	66259428	0.495105305	9.34E-12	8.40E-10
_G0n	rs55845504	14	66256968	0.192356198	5.49E-11	1.38E-07
_G0n	rs56379231	14	66255397	0.195976256	4.67E-11	1.22E-07
_G0n	rs56720460	14	66256336	0.363517666	2.66E-12	1.14E-09
_G0n	rs56922569	14	66256472	0.380920366	2.82E-12	9.70E-10
_G0n	rs57071950	14	65788437	0.015800152	9.04E-13	2.14E-06
_G0n	rs57507878	14	65843372	0.046591701	3.96E-09	0.000106986
_G0n	rs58102398	14	66250157	0.134150998	5.17E-14	1.57E-09
_G0n	rs59136469	14	65811352	0.019675639	4.18E-08	0.001644699
_G0n	rs59636756	14	66256489	0.380920366	2.77E-12	9.54E-10
_G0n	rs59853675	14	66250679	0.124471616	2.46E-14	1.07E-09
_G0n	rs60273099	14	66214850	0.088460896	4.95E-14	5.29E-09
_G0n	rs60488013	14	65825854	0.033827675	2.25E-15	6.57E-09
_G0n	rs60734251	14	66250274	0.134150998	4.99E-14	1.52E-09
_G0n	rs61161789	14	65843373	0.049628867	7.36E-09	0.000141753
_G0n	rs61298903	14	66218595	0.088195813	4.00E-14	4.59E-09
_G0n	rs61348705	14	66111019	0.246865431	6.53E-13	1.69E-09
_G0n	rs61378474	14	66253437	0.344404091	2.02E-12	1.21E-09
_G0n	rs61988977	14	66258032	0.342056718	2.14E-12	1.22E-09
_G0n	rs61988978	14	66258111	0.521329967	1.06E-11	7.32E-10
_G0n	rs6573598	14	65783019	0.015553432	1.04E-12	2.50E-06
_G0n	rs6573599	14	65783127	0.015644132	1.05E-12	2.50E-06
_G0n	rs6573600	14	65783270	0.016490093	9.63E-13	2.03E-06
_G0n	rs6573602	14	65784610	0.015553432	1.02E-12	2.48E-06
_G0n	rs6573604	14	65787941	0.015708793	9.32E-13	2.21E-06
_G0n	rs6573627	14	66252326	0.271395819	4.89E-13	9.37E-10
_G0n	rs66492349	14	65839489	0.032313029	4.72E-15	1.31E-08
_G0n	rs67662065	14	66250999	0.112062702	1.31E-14	8.96E-10
_G0n	rs7142165	14	66250232	0.251501335	3.98E-13	1.07E-09
_G0n	rs7143026	14	66250956	0.123697608	1.99E-11	2.34E-07
_G0n	rs7151212	14	65781622	0.017490213	2.19E-12	3.25E-06
_G0n	rs7151301	14	65781496	0.015644132	1.05E-12	2.50E-06
_G0n	rs7151846	14	66255781	0.359159406	2.41E-12	1.10E-09
_G0n	rs7156013	14	65789201	0.01961916	2.13E-12	2.68E-06
_G0n	rs7159888	14	65758642	0.052322234	1.23E-12	2.59E-07

_G0n	rs72712459	14	65734600	0.058256357	5.73E-12	6.02E-07
_G0n	rs73278053	14	65788490	0.015610994	7.80E-13	1.98E-06
_G0n	rs73278083	14	65807694	0.025261966	2.90E-08	0.000930212
_G0n	rs73282213	14	65852139	0.032175128	2.87E-08	0.000651156
_G0n	rs73282250	14	65857146	0.032989921	2.39E-08	0.000562884
_G0n	rs73282260	14	65858964	0.042462728	1.20E-08	0.00024526
_G0n	rs76824828	14	65758236	0.068567487	1.71E-10	4.95E-06
_G0n	rs76922762	14	65737580	0.110655709	1.27E-07	0.000225758
_G0n	rs79230737	14	65742463	0.101096854	6.14E-08	0.00015942
_G0n	rs80001898	14	65832786	0.02105359	1.34E-08	0.000724081
_G0n	rs8004239	14	65783460	0.015258934	1.05E-12	2.48E-06
_G0n	rs8006316	14	65787115	0.01537342	9.45E-13	2.40E-06
_G0n	rs8007846	14	66262963	0.516589299	1.27E-07	3.57E-06
_G0n	rs8010759	14	65784559	0.018778654	5.52E-13	1.11E-06
_G0n	rs8012054	14	65774762	0.017862665	1.64E-12	2.66E-06
_G0n	rs8013568	14	65774875	0.015347987	1.04E-12	2.48E-06
_G0n	rs8017974	14	65775187	0.015437498	1.10E-12	2.55E-06
_G0n	rs8018379	14	66261937	0.296694185	3.92E-11	2.78E-08
_G0n	rs8019473	14	65779128	0.016424002	1.77E-12	3.20E-06
_G0n	rs8019767	14	65782785	0.015553432	1.04E-12	2.50E-06
_G0n	rs9635250	14	65799348	0.014825459	1.61E-12	3.64E-06
_G0n	rs968540	14	66249064	0.107925982	1.29E-11	2.37E-07
_G1FN	rs1005522	22	39845898	1.37E-06	0.001867573	2.38E-15
_G1FN	rs1007337	22	39781585	8.16E-05	0.000887562	4.84E-13
_G1FN	rs1010169	22	39778167	7.32E-05	0.000921768	4.32E-13
_G1FN	rs1010170	22	39778327	6.78E-05	0.000930508	3.85E-13
_G1FN	rs10644269	22	39836972	1.83E-06	0.001011202	9.58E-16
_G1FN	rs113200473	22	39853740	1.63E-06	0.002529444	5.93E-15
_G1FN	rs1557541	22	39851970	2.17E-06	0.000981125	1.16E-15
_G1FN	rs1557542	22	39852350	2.23E-06	0.001077705	1.43E-15
_G1FN	rs1557543	22	39852648	1.69E-06	0.00116599	1.08E-15
_G1FN	rs1569499	22	39769818	0.000374246	0.002288241	4.59E-11
_G1FN	rs1972280	22	39831986	1.61E-06	0.001331555	1.36E-15
_G1FN	rs2008174	22	39860130	3.21E-06	0.001339035	4.70E-15
_G1FN	rs2142848	22	39785242	1.71E-05	0.002263127	2.31E-13
_G1FN	rs2413590	22	39790191	2.29E-05	0.001629966	1.83E-13
_G1FN	rs2413592	22	39858196	2.11E-06	0.001453878	2.56E-15
_G1FN	rs2899318	22	39837625	2.04E-06	0.000909994	8.44E-16
_G1FN	rs35509952	22	39800563	2.07E-05	0.002405722	3.60E-13
_G1FN	rs3959642	22	39860589	3.32E-06	0.001316536	4.79E-15
_G1FN	rs4321460	22	39793655	4.95E-05	0.002829936	2.51E-12
_G1FN	rs4337572	22	39800704	1.97E-05	0.002201102	2.90E-13

_G1FN	rs4384886	22	39793766	2.14E-05	0.002025217	2.61E-13
_G1FN	rs4386422	22	39793734	5.09E-05	0.00207205	1.24E-12
_G1FN	rs4429561	22	39799789	2.14E-05	0.003231652	7.32E-13
_G1FN	rs4820377	22	39770780	0.000336406	0.002520189	5.02E-11
_G1FN	rs4821888	22	39770597	0.000352193	0.002227714	3.91E-11
_G1FN	rs4821889	22	39777254	6.68E-05	0.00117118	6.00E-13
_G1FN	rs4821890	22	39777523	6.61E-05	0.001182047	6.00E-13
_G1FN	rs4821891	22	39785381	1.80E-05	0.002343632	2.70E-13
_G1FN	rs4821892	22	39795683	2.29E-05	0.001659512	1.90E-13
_G1FN	rs4821893	22	39797779	2.06E-05	0.001857225	2.04E-13
_G1FN	rs4821894	22	39809820	2.06E-05	0.002528467	4.01E-13
_G1FN	rs4821895	22	39823015	2.46E-05	0.001927866	3.13E-13
_G1FN	rs4821896	22	39833437	1.59E-06	0.001382187	1.55E-15
_G1FN	rs4821897	22	39835587	1.94E-06	0.000874598	7.94E-16
_G1FN	rs4821898	22	39838352	2.19E-06	0.000816428	7.95E-16
_G1FN	rs5750806	22	39767011	0.000359405	0.003485263	1.00E-10
_G1FN	rs5750808	22	39790987	2.29E-05	0.001677722	2.06E-13
_G1FN	rs5750809	22	39791491	2.66E-05	0.002207498	5.00E-13
_G1FN	rs5750810	22	39792943	2.32E-05	0.001677722	2.10E-13
_G1FN	rs5750811	22	39793066	2.32E-05	0.001677722	2.10E-13
_G1FN	rs5750812	22	39793079	2.11E-05	0.002061149	2.66E-13
_G1FN	rs5750813	22	39795228	2.14E-05	0.001723337	1.95E-13
_G1FN	rs5750814	22	39797987	1.93E-05	0.002201102	2.91E-13
_G1FN	rs5750815	22	39798449	1.90E-05	0.002008683	2.34E-13
_G1FN	rs5750816	22	39810379	2.63E-05	0.001837262	3.31E-13
_G1FN	rs5750818	22	39820885	2.16E-05	0.002099791	3.05E-13
_G1FN	rs5750820	22	39825322	2.32E-05	0.001965813	2.80E-13
_G1FN	rs5750821	22	39825492	2.43E-05	0.001897313	2.80E-13
_G1FN	rs5750822	22	39826788	2.57E-05	0.001831013	2.86E-13
_G1FN	rs5750823	22	39829973	2.24E-06	0.000829996	8.58E-16
_G1FN	rs5750825	22	39831278	2.56E-06	0.000692489	6.68E-16
_G1FN	rs5750826	22	39832113	0.001408155	0.002525335	6.23E-10
_G1FN	rs5750828	22	39835083	9.69E-07	0.001299897	6.33E-16
_G1FN	rs5750829	22	39838018	1.06E-06	0.001601456	1.18E-15
_G1FN	rs5750830	22	39840828	1.43E-06	0.000984044	6.26E-16
_G1FN	rs5750833	22	39843091	2.06E-06	0.0007262	5.41E-16
_G1FN	rs5757642	22	39764824	0.000343099	0.002337497	4.28E-11
_G1FN	rs5757644	22	39766440	0.000353963	0.003453291	1.04E-10
_G1FN	rs5757645	22	39768479	0.000355782	0.003485263	9.83E-11
_G1FN	rs5757647	22	39775047	8.49E-05	0.000899019	5.05E-13
_G1FN	rs5757648	22	39775156	8.31E-05	0.000924816	5.15E-13
_G1FN	rs5757650	22	39778419	8.40E-05	0.000882192	4.77E-13

_G1FN	rs5757652	22	39781855	6.92E-05	0.001579	1.12E-12
_G1FN	rs5757654	22	39794124	2.07E-05	0.002576919	4.61E-13
_G1FN	rs5757655	22	39797178	1.93E-05	0.002051247	2.50E-13
_G1FN	rs5757657	22	39798429	1.93E-05	0.002201102	2.91E-13
_G1FN	rs5757659	22	39812409	2.04E-05	0.002062902	2.67E-13
_G1FN	rs5757663	22	39821319	2.21E-05	0.002081272	3.11E-13
_G1FN	rs5757664	22	39821536	2.14E-05	0.002118461	3.05E-13
_G1FN	rs5757665	22	39821641	2.04E-05	0.002062902	2.67E-13
_G1FN	rs5757667	22	39822116	2.69E-05	0.002014	3.78E-13
_G1FN	rs5757670	22	39829736	3.22E-05	0.00220732	6.84E-13
_G1FN	rs5757673	22	39837920	1.20E-06	0.001239197	7.98E-16
_G1FN	rs5757675	22	39838892	1.69E-06	0.000990055	8.34E-16
_G1FN	rs5757676	22	39841700	8.61E-05	0.004268472	1.37E-11
_G1FN	rs5757678	22	39843409	1.83E-06	0.000918847	7.21E-16
_G1FN	rs5757680	22	39844793	1.51E-06	0.000790782	4.21E-16
_G1FN	rs5757681	22	39845547	1.53E-06	0.000783652	4.21E-16
_G1FN	rs5757682	22	39848259	1.56E-06	0.000894732	5.56E-16
_G1FN	rs5757683	22	39850174	2.09E-06	0.000852869	7.57E-16
_G1FN	rs5757684	22	39851584	2.11E-06	0.000963868	1.01E-15
_G1FN	rs5757685	22	39855540	1.76E-06	0.001308338	1.68E-15
_G1FN	rs5995735	22	39854421	1.60E-06	0.00129709	1.41E-15
_G1FN	rs6001566	22	39774448	5.36E-05	0.001149722	3.94E-13
_G1FN	rs6001567	22	39775400	8.49E-05	0.000899019	5.05E-13
_G1FN	rs6001568	22	39775786	7.32E-05	0.000948219	4.57E-13
_G1FN	rs6001585	22	39812986	6.95E-05	0.003440711	5.85E-12
_G1FN	rs6001587	22	39819008	1.66E-05	0.002867547	3.91E-13
_G1FN	rs6001588	22	39819049	2.21E-05	0.002276689	3.58E-13
_G1FN	rs6001594	22	39837472	2.07E-06	0.000926653	8.97E-16
_G1FN	rs6001595	22	39839293	1.38E-06	0.001501215	1.49E-15
_G1FN	rs6001599	22	39852720	1.69E-06	0.001186495	1.13E-15
_G1FN	rs6001600	22	39852921	2.23E-06	0.001096772	1.49E-15
_G1FN	rs6519190	22	39774525	6.25E-05	0.001260766	6.24E-13
_G1FN	rs7286714	22	39765604	0.000353963	0.003513363	1.07E-10
_G1FN	rs7286917	22	39860868	2.72E-06	0.002230471	1.04E-14
_G1FN	rs7288760	22	39819969	8.36E-06	0.001710845	4.02E-14
_G1FN	rs7292066	22	39794241	2.21E-05	0.001883752	2.50E-13
_G1FN	rs73167342	22	39834102	8.81E-07	0.001925369	1.30E-15
_G1FN	rs7364148	22	39842165	1.69E-06	0.00079536	4.79E-16
_G1FN	rs738285	22	39856356	1.81E-06	0.001380967	1.79E-15
_G1FN	rs738286	22	39855575	2.25E-06	0.001213417	1.89E-15
_G1FN	rs738287	22	39855728	1.76E-06	0.001331104	1.75E-15
_G1FN	rs738289	22	39855883	1.76E-06	0.001354234	1.82E-15

_G1FN	rs738290	22	39856032	0.000505638	0.002854261	1.21E-10
_G1FN	rs739141	22	39824450	2.95E-05	0.005261259	3.43E-12
_G1FN	rs7423	22	39781429	6.83E-05	0.001115114	5.35E-13
_G1FN	rs743838	22	39824707	2.95E-05	0.00170131	3.40E-13
_G1FN	rs756640	22	39779300	6.83E-05	0.001189858	6.11E-13
_G1FN	rs7949	22	39827553	2.37E-05	0.001880534	2.64E-13
_G1FN	rs8136980	22	39844574	2.03E-06	0.000790782	6.85E-16
_G1FN	rs8137426	22	39844350	1.71E-06	0.000805225	5.37E-16
_G1FN	rs8138462	22	39839670	1.64E-06	0.000999007	8.17E-16
_G1FN	rs909674	22	39859169	2.72E-06	0.001285383	2.95E-15
_G1FN	rs9306335	22	39843537	1.72E-06	0.000614722	2.63E-16
_G1FN	rs9611162	22	39767247	0.000359405	0.003515375	1.02E-10
_G1FN	rs9611164	22	39767291	0.000374372	0.003580028	1.13E-10
_G1FN	rs9611165	22	39775250	8.49E-05	0.000899019	5.05E-13
_G1FN	rs9611166	22	39775268	8.49E-05	0.000899019	5.05E-13
_G1FN	rs9611167	22	39775583	7.29E-05	0.001272399	8.29E-13
_G1FN	rs9611169	22	39783027	7.43E-05	0.001074914	6.07E-13
_G1FN	rs9611170	22	39784845	2.24E-05	0.002131435	3.34E-13
_G1FN	rs9611176	22	39838003	1.40E-06	0.000999007	6.27E-16
_G1FN	rs6001582	22	39806153	2.51E-05	0.002320008	4.87E-13
_G1FN/G1F	rs1005522	22	39845898	1.23E-05	0.012594922	8.27E-12
_G1FN/G1F	rs1007337	22	39781585	0.000285912	0.005897559	2.24E-10
_G1FN/G1F	rs1010169	22	39778167	0.000213362	0.0061932	1.41E-10
_G1FN/G1F	rs1010170	22	39778327	0.000208996	0.006243296	1.39E-10
_G1FN/G1F	rs10644269	22	39836972	8.64E-06	0.010631204	3.23E-12
_G1FN/G1F	rs113200473	22	39853740	1.08E-05	0.019542801	1.87E-11
_G1FN/G1F	rs1557541	22	39851970	1.38E-05	0.008711978	3.86E-12
_G1FN/G1F	rs1557542	22	39852350	1.36E-05	0.008970264	4.07E-12
_G1FN/G1F	rs1557543	22	39852648	1.00E-05	0.010132567	3.59E-12
_G1FN/G1F	rs1569499	22	39769818	0.001171217	0.011447133	9.76E-09
_G1FN/G1F	rs1972280	22	39831986	9.77E-06	0.011203425	4.42E-12
_G1FN/G1F	rs2008174	22	39860130	1.69E-05	0.010509721	8.98E-12
_G1FN/G1F	rs2142848	22	39785242	0.000116187	0.008949553	1.26E-10
_G1FN/G1F	rs2413590	22	39790191	0.000107827	0.008218097	9.25E-11
_G1FN/G1F	rs2413592	22	39858196	1.24E-05	0.0116607	6.94E-12
_G1FN/G1F	rs28685576	22	39841533	0.002673257	0.011560775	3.68E-08
_G1FN/G1F	rs2899318	22	39837625	9.41E-06	0.008948063	2.42E-12
_G1FN/G1F	rs35509952	22	39800563	0.000123835	0.009517494	1.60E-10
_G1FN/G1F	rs3959642	22	39860589	1.83E-05	0.010854879	1.09E-11
_G1FN/G1F	rs4321460	22	39793655	0.000278152	0.014036882	1.37E-09
_G1FN/G1F	rs4337572	22	39800704	0.000131957	0.008812385	1.49E-10
_G1FN/G1F	rs4384886	22	39793766	0.000120516	0.008997675	1.35E-10

_G1FN/G1F	rs4386422	22	39793734	0.000200971	0.012900384	7.07E-10
_G1FN/G1F	rs4429561	22	39799789	0.000147235	0.012567828	3.63E-10
_G1FN/G1F	rs4820377	22	39770780	0.00096996	0.01283311	9.03E-09
_G1FN/G1F	rs4821888	22	39770597	0.001089416	0.011783232	8.61E-09
_G1FN/G1F	rs4821889	22	39777254	0.000265101	0.006809576	2.48E-10
_G1FN/G1F	rs4821890	22	39777523	0.000265101	0.00686393	2.53E-10
_G1FN/G1F	rs4821891	22	39785381	0.000118685	0.009088626	1.35E-10
_G1FN/G1F	rs4821892	22	39795683	0.000107827	0.00834697	9.57E-11
_G1FN/G1F	rs4821893	22	39797779	0.000114396	0.008107092	9.88E-11
_G1FN/G1F	rs4821894	22	39809820	0.000103872	0.010223174	1.42E-10
_G1FN/G1F	rs4821895	22	39823015	0.000132332	0.007903672	1.14E-10
_G1FN/G1F	rs4821896	22	39833437	1.04E-05	0.010804722	4.57E-12
_G1FN/G1F	rs4821897	22	39835587	9.74E-06	0.008694773	2.26E-12
_G1FN/G1F	rs4821898	22	39838352	1.05E-05	0.007923405	2.14E-12
_G1FN/G1F	rs5750806	22	39767011	0.00134677	0.014690774	2.05E-08
_G1FN/G1F	rs5750808	22	39790987	0.000107827	0.008218097	9.25E-11
_G1FN/G1F	rs5750809	22	39791491	0.000129764	0.01112984	2.44E-10
_G1FN/G1F	rs5750810	22	39792943	0.000107827	0.008218097	9.25E-11
_G1FN/G1F	rs5750811	22	39793066	0.000107827	0.008218097	9.25E-11
_G1FN/G1F	rs5750812	22	39793079	0.000117991	0.009067045	1.33E-10
_G1FN/G1F	rs5750813	22	39795228	0.000106354	0.008403317	8.78E-11
_G1FN/G1F	rs5750814	22	39797987	0.000127835	0.008880732	1.44E-10
_G1FN/G1F	rs5750815	22	39798449	0.0001096	0.009040228	1.17E-10
_G1FN/G1F	rs5750816	22	39810379	0.000141493	0.007856816	1.30E-10
_G1FN/G1F	rs5750818	22	39820885	0.000121962	0.009040228	1.39E-10
_G1FN/G1F	rs5750820	22	39825322	0.000119954	0.008282309	1.12E-10
_G1FN/G1F	rs5750821	22	39825492	0.000127835	0.007842096	1.10E-10
_G1FN/G1F	rs5750822	22	39826788	0.000130569	0.007842096	1.14E-10
_G1FN/G1F	rs5750823	22	39829973	1.10E-05	0.007907255	2.39E-12
_G1FN/G1F	rs5750825	22	39831278	1.16E-05	0.007041319	1.89E-12
_G1FN/G1F	rs5750826	22	39832113	0.002407062	0.011934137	3.46E-08
_G1FN/G1F	rs5750828	22	39835083	8.14E-06	0.012909932	4.49E-12
_G1FN/G1F	rs5750829	22	39838018	8.69E-06	0.01337563	5.41E-12
_G1FN/G1F	rs5750830	22	39840828	6.25E-06	0.009086882	1.34E-12
_G1FN/G1F	rs5750833	22	39843091	9.74E-06	0.006844542	1.39E-12
_G1FN/G1F	rs5757642	22	39764824	0.000979025	0.012046192	7.55E-09
_G1FN/G1F	rs5757644	22	39766440	0.001318416	0.014800601	1.96E-08
_G1FN/G1F	rs5757645	22	39768479	0.001362341	0.014770073	1.99E-08
_G1FN/G1F	rs5757647	22	39775047	0.000230819	0.005850059	1.54E-10
_G1FN/G1F	rs5757648	22	39775156	0.000228462	0.005850059	1.52E-10
_G1FN/G1F	rs5757650	22	39778419	0.000285912	0.005756092	2.13E-10
_G1FN/G1F	rs5757652	22	39781855	0.000253992	0.009067045	4.59E-10

_G1FN/G1F	rs5757654	22	39794124	0.000130095	0.010866031	2.32E-10
_G1FN/G1F	rs5757655	22	39797178	0.000123835	0.008477648	1.24E-10
_G1FN/G1F	rs5757657	22	39798429	0.000127835	0.008880732	1.44E-10
_G1FN/G1F	rs5757659	22	39812409	0.0001096	0.009040228	1.17E-10
_G1FN/G1F	rs5757663	22	39821319	0.000115625	0.008764288	1.19E-10
_G1FN/G1F	rs5757664	22	39821536	0.000120669	0.009110414	1.39E-10
_G1FN/G1F	rs5757665	22	39821641	0.000108431	0.009040228	1.15E-10
_G1FN/G1F	rs5757667	22	39822116	0.000176241	0.008908204	2.44E-10
_G1FN/G1F	rs5757670	22	39829736	0.000131457	0.011193895	2.52E-10
_G1FN/G1F	rs5757673	22	39837920	7.44E-06	0.011662741	3.32E-12
_G1FN/G1F	rs5757675	22	39838892	9.11E-06	0.009139269	2.30E-12
_G1FN/G1F	rs5757676	22	39841700	0.000341245	0.013746737	1.79E-09
_G1FN/G1F	rs5757678	22	39843409	8.76E-06	0.007961123	1.55E-12
_G1FN/G1F	rs5757680	22	39844793	7.39E-06	0.00754229	1.05E-12
_G1FN/G1F	rs5757681	22	39845547	7.21E-06	0.007215861	9.91E-13
_G1FN/G1F	rs5757682	22	39848259	8.71E-06	0.008739711	2.09E-12
_G1FN/G1F	rs5757683	22	39850174	1.24E-05	0.007939241	2.78E-12
_G1FN/G1F	rs5757684	22	39851584	1.31E-05	0.008531271	3.73E-12
_G1FN/G1F	rs5757685	22	39855540	1.11E-05	0.011024684	4.90E-12
_G1FN/G1F	rs5995735	22	39854421	1.04E-05	0.010791889	4.20E-12
_G1FN/G1F	rs6001566	22	39774448	0.000209528	0.007079826	1.91E-10
_G1FN/G1F	rs6001567	22	39775400	0.000230819	0.005850059	1.54E-10
_G1FN/G1F	rs6001568	22	39775786	0.000206845	0.006344585	1.41E-10
_G1FN/G1F	rs6001585	22	39812986	0.00031824	0.011864233	1.17E-09
_G1FN/G1F	rs6001587	22	39819008	0.000103294	0.011690662	1.90E-10
_G1FN/G1F	rs6001588	22	39819049	0.000106128	0.009764313	1.32E-10
_G1FN/G1F	rs6001594	22	39837472	9.52E-06	0.009082079	2.56E-12
_G1FN/G1F	rs6001595	22	39839293	8.59E-06	0.014518555	6.87E-12
_G1FN/G1F	rs6001599	22	39852720	9.93E-06	0.010577837	4.05E-12
_G1FN/G1F	rs6001600	22	39852921	1.38E-05	0.009168445	4.36E-12
_G1FN/G1F	rs6519190	22	39774525	0.000211697	0.007364758	2.11E-10
_G1FN/G1F	rs7286714	22	39765604	0.001330723	0.015134438	2.09E-08
_G1FN/G1F	rs7286917	22	39860868	1.95E-05	0.013765985	2.13E-11
_G1FN/G1F	rs7288760	22	39819969	6.22E-05	0.008136425	3.73E-11
_G1FN/G1F	rs7292066	22	39794241	0.000123093	0.008997675	1.40E-10
_G1FN/G1F	rs73167342	22	39834102	6.68E-06	0.014425897	4.64E-12
_G1FN/G1F	rs7364148	22	39842165	7.46E-06	0.007777698	1.25E-12
_G1FN/G1F	rs738285	22	39856356	1.15E-05	0.011405468	6.10E-12
_G1FN/G1F	rs738286	22	39855575	1.52E-05	0.009975261	6.24E-12
_G1FN/G1F	rs738287	22	39855728	1.10E-05	0.011182349	4.99E-12
_G1FN/G1F	rs738289	22	39855883	1.10E-05	0.01126193	5.07E-12
_G1FN/G1F	rs738290	22	39856032	0.001274348	0.011786236	1.08E-08

_G1FN/G1F	rs739141	22	39824450	0.000276809	0.013302633	1.15E-09
_G1FN/G1F	rs7423	22	39781429	0.000270537	0.006491502	2.32E-10
_G1FN/G1F	rs743838	22	39824707	0.000134774	0.007422965	1.06E-10
_G1FN/G1F	rs756640	22	39779300	0.000273293	0.00686393	2.66E-10
_G1FN/G1F	rs7949	22	39827553	0.000127407	0.007896303	1.03E-10
_G1FN/G1F	rs8136980	22	39844574	1.13E-05	0.007210552	1.82E-12
_G1FN/G1F	rs8137426	22	39844350	7.46E-06	0.00766229	1.21E-12
_G1FN/G1F	rs8138462	22	39839670	9.01E-06	0.009207205	2.30E-12
_G1FN/G1F	rs909674	22	39859169	1.58E-05	0.010390234	7.89E-12
_G1FN/G1F	rs9306335	22	39843537	8.31E-06	0.006434147	9.41E-13
_G1FN/G1F	rs9611162	22	39767247	0.00133435	0.014690774	2.02E-08
_G1FN/G1F	rs9611164	22	39767291	0.001456768	0.014308259	2.21E-08
_G1FN/G1F	rs9611165	22	39775250	0.000230819	0.005850059	1.54E-10
_G1FN/G1F	rs9611166	22	39775268	0.000230819	0.005850059	1.54E-10
_G1FN/G1F	rs9611167	22	39775583	0.000270537	0.007192589	3.01E-10
_G1FN/G1F	rs9611169	22	39783027	0.000212834	0.007044967	1.94E-10
_G1FN/G1F	rs9611170	22	39784845	0.000131957	0.00815433	1.26E-10
_G1FN/G1F	rs9611176	22	39838003	7.83E-06	0.009004739	1.76E-12
_G1FN/G1F	rs6001582	22	39806153	0.000154992	0.008812385	1.88E-10
_G1FN/G1N	rs10138052	14	65833440	8.06E-09	1	1
_G1FN/G1N	rs10148907	14	65833372	7.95E-09	1	1
_G1FN/G1N	rs112850324	14	66241579	7.54E-08	1	1
_G1FN/G1N	rs11847263	14	65775695	2.02E-09	1	1
_G1FN/G1N	rs1760978	14	65771047	3.48E-08	1	1
_G1FN/G1N	rs1950557	14	66201757	1.11E-08	1	1
_G1FN/G1N	rs28740895	14	65783523	5.06E-08	1	1
_G1FN/G1N	rs35206478	14	66223213	7.85E-09	1	1
_G1FN/G1N	rs3742597	14	66200177	9.53E-09	1	1
_G1FN/G1N	rs4131097	14	66259165	4.98E-08	1	1
_G1FN/G1N	rs58102398	14	66250157	1.03E-08	1	1
_G1FN/G1N	rs59853675	14	66250679	7.76E-09	1	1
_G1FN/G1N	rs60273099	14	66214850	1.02E-08	1	1
_G1FN/G1N	rs60488013	14	65825854	7.96E-09	1	1
_G1FN/G1N	rs60734251	14	66250274	1.04E-08	1	1
_G1FN/G1N	rs61298903	14	66218595	7.61E-09	1	1
_G1FN/G1N	rs61348705	14	66111019	2.38E-07	1	1
_G1FN/G1N	rs66492349	14	65839489	8.43E-09	1	1
_G1FN/G1N	rs67662065	14	66250999	2.58E-09	1	1
_G1FN/G1N	rs72712459	14	65734600	7.89E-08	1	1
_G1FN/G1N	rs10138570	14	65860038	5.20E-09	1	1
_G1FN/G1N	rs11158591	14	65855762	4.26E-08	1	1
_G1FN/G1N	rs11158592	14	65859968	6.23E-09	1	1

_G1FN/G1N	rs11158593	14	65859984	5.64E-09	1	1
_G1FN/G1N	rs11621121	14	65822493	1.27E-07	1	1
_G1FN/G1N	rs11623920	14	65819314	1.45E-07	1	1
_G1FN/G1N	rs11627598	14	65797766	1.10E-07	1	1
_G1FN/G1N	rs12878872	14	65804665	7.00E-08	1	1
_G1FN/G1N	rs12880725	14	65799805	1.06E-07	1	1
_G1FN/G1N	rs12881911	14	65831221	2.77E-07	1	1
_G1FN/G1N	rs12882269	14	65847144	1.94E-07	1	1
_G1FN/G1N	rs12886005	14	65809247	1.35E-07	1	1
_G1FN/G1N	rs12886168	14	65809286	1.93E-07	1	1
_G1FN/G1N	rs12893094	14	65789077	9.49E-08	1	1
_G1FN/G1N	rs1815654	14	65855232	3.90E-08	1	1
_G1FN/G1N	rs1954052	14	65805709	8.81E-08	1	1
_G1FN/G1N	rs4899174	14	65811400	1.48E-07	1	1
_G1FN/G1N	rs4902391	14	65815979	1.39E-07	1	1
_G1FN/G1N	rs4902393	14	65839514	2.19E-07	1	1
_G1FN/G1N	rs7157006	14	65828207	2.24E-07	1	1
_G1FN/G1N	rs7158347	14	65828955	2.48E-07	1	1
_G1FN/G1N	rs747541	14	65805410	1.79E-07	1	1
_G1FN/G1N	rs8022094	14	65846841	2.12E-07	1	1
_G1FN/G1N	rs899959	14	65791928	1.55E-07	1	1
_G1FNn	rs1005522	22	39845898	1.21E-06	0.011086395	1.37E-13
_G1FNn	rs1007337	22	39781585	9.43E-05	0.003625552	1.01E-11
_G1FNn	rs1010169	22	39778167	7.71E-05	0.003874472	8.31E-12
_G1FNn	rs1010170	22	39778327	7.22E-05	0.004074944	8.74E-12
_G1FNn	rs10644269	22	39836972	2.09E-06	0.005490818	5.64E-14
_G1FNn	rs113200473	22	39853740	1.64E-06	0.012418347	2.80E-13
_G1FNn	rs1557541	22	39851970	2.21E-06	0.005390814	5.24E-14
_G1FNn	rs1557542	22	39852350	2.16E-06	0.005727463	6.23E-14
_G1FNn	rs1557543	22	39852648	1.66E-06	0.006706059	5.57E-14
_G1FNn	rs1569499	22	39769818	0.00065738	0.006733598	1.04E-09
_G1FNn	rs1972280	22	39831986	1.85E-06	0.006803222	7.51E-14
_G1FNn	rs2008174	22	39860130	2.81E-06	0.007217669	1.65E-13
_G1FNn	rs2142848	22	39785242	1.72E-05	0.008005829	3.75E-12
_G1FNn	rs2413590	22	39790191	2.21E-05	0.006126047	3.01E-12
_G1FNn	rs2413592	22	39858196	1.85E-06	0.008386537	1.27E-13
_G1FNn	rs2899318	22	39837625	2.21E-06	0.004919728	4.43E-14
_G1FNn	rs35509952	22	39800563	2.04E-05	0.008257946	5.31E-12
_G1FNn	rs3959642	22	39860589	2.91E-06	0.007113438	1.68E-13
_G1FNn	rs4321460	22	39793655	4.06E-05	0.011075561	3.29E-11
_G1FNn	rs4337572	22	39800704	2.04E-05	0.007700389	4.35E-12
_G1FNn	rs4384886	22	39793766	2.11E-05	0.006999332	3.81E-12

_G1FNn	rs4386422	22	39793734	5.14E-05	0.007982923	2.24E-11
_G1FNn	rs4429561	22	39799789	2.24E-05	0.010722192	1.14E-11
_G1FNn	rs4820377	22	39770780	0.000599722	0.007594241	1.11E-09
_G1FNn	rs4821888	22	39770597	0.000620178	0.006679468	9.29E-10
_G1FNn	rs4821889	22	39777254	7.12E-05	0.004653125	1.14E-11
_G1FNn	rs4821890	22	39777523	7.12E-05	0.004691464	1.16E-11
_G1FNn	rs4821891	22	39785381	1.80E-05	0.00819426	4.27E-12
_G1FNn	rs4821892	22	39795683	2.21E-05	0.006175099	3.07E-12
_G1FNn	rs4821893	22	39797779	2.06E-05	0.006964562	3.63E-12
_G1FNn	rs4821894	22	39809820	2.09E-05	0.009421674	7.94E-12
_G1FNn	rs4821895	22	39823015	2.51E-05	0.006954551	4.77E-12
_G1FNn	rs4821896	22	39833437	1.88E-06	0.006692178	7.12E-14
_G1FNn	rs4821897	22	39835587	2.07E-06	0.004842814	3.69E-14
_G1FNn	rs4821898	22	39838352	2.56E-06	0.004511253	4.34E-14
_G1FNn	rs5750806	22	39767011	0.000635853	0.009468453	1.94E-09
_G1FNn	rs5750808	22	39790987	2.21E-05	0.006126047	3.01E-12
_G1FNn	rs5750809	22	39791491	2.66E-05	0.00786765	7.20E-12
_G1FNn	rs5750810	22	39792943	2.24E-05	0.006126047	3.07E-12
_G1FNn	rs5750811	22	39793066	2.24E-05	0.006126047	3.07E-12
_G1FNn	rs5750812	22	39793079	2.11E-05	0.007109903	3.95E-12
_G1FNn	rs5750813	22	39795228	2.11E-05	0.006274266	2.96E-12
_G1FNn	rs5750814	22	39797987	1.90E-05	0.007821289	4.04E-12
_G1FNn	rs5750815	22	39798449	1.97E-05	0.007594412	4.13E-12
_G1FNn	rs5750816	22	39810379	2.57E-05	0.006641159	4.45E-12
_G1FNn	rs5750818	22	39820885	2.29E-05	0.007594412	5.24E-12
_G1FNn	rs5750820	22	39825322	2.34E-05	0.006954551	4.43E-12
_G1FNn	rs5750821	22	39825492	2.45E-05	0.006738491	4.43E-12
_G1FNn	rs5750822	22	39826788	2.54E-05	0.006632741	4.51E-12
_G1FNn	rs5750823	22	39829973	2.49E-06	0.004472728	4.51E-14
_G1FNn	rs5750825	22	39831278	2.80E-06	0.003840623	3.53E-14
_G1FNn	rs5750828	22	39835083	1.17E-06	0.006249142	2.99E-14
_G1FNn	rs5750829	22	39838018	1.18E-06	0.008104262	5.86E-14
_G1FNn	rs5750830	22	39840828	1.48E-06	0.006242874	4.27E-14
_G1FNn	rs5750833	22	39843091	2.14E-06	0.004338987	2.81E-14
_G1FNn	rs5757642	22	39764824	0.000577442	0.00743413	9.55E-10
_G1FNn	rs5757644	22	39766440	0.000635853	0.009468453	1.94E-09
_G1FNn	rs5757645	22	39768479	0.000629693	0.009394987	1.88E-09
_G1FNn	rs5757647	22	39775047	8.48E-05	0.003844947	9.23E-12
_G1FNn	rs5757648	22	39775156	8.74E-05	0.00390981	1.05E-11
_G1FNn	rs5757650	22	39778419	8.93E-05	0.003564994	8.88E-12
_G1FNn	rs5757652	22	39781855	7.53E-05	0.006168908	2.22E-11
_G1FNn	rs5757654	22	39794124	2.09E-05	0.008191795	5.23E-12

_G1FNn	rs5757655	22	39797178	1.90E-05	0.007405436	3.55E-12
_G1FNn	rs5757657	22	39798429	1.90E-05	0.007821289	4.04E-12
_G1FNn	rs5757659	22	39812409	2.06E-05	0.007714268	4.45E-12
_G1FNn	rs5757663	22	39821319	2.24E-05	0.007835818	5.25E-12
_G1FNn	rs5757664	22	39821536	2.27E-05	0.007594412	5.14E-12
_G1FNn	rs5757665	22	39821641	2.14E-05	0.00777483	4.96E-12
_G1FNn	rs5757667	22	39822116	2.45E-05	0.007438687	5.56E-12
_G1FNn	rs5757670	22	39829736	3.25E-05	0.008067603	1.01E-11
_G1FNn	rs5757673	22	39837920	1.39E-06	0.006785624	4.79E-14
_G1FNn	rs5757675	22	39838892	1.82E-06	0.005360303	3.89E-14
_G1FNn	rs5757676	22	39841700	0.000111267	0.017411345	3.96E-10
_G1FNn	rs5757678	22	39843409	1.91E-06	0.005232431	4.11E-14
_G1FNn	rs5757680	22	39844793	1.59E-06	0.005071219	2.85E-14
_G1FNn	rs5757681	22	39845547	1.65E-06	0.004954709	2.85E-14
_G1FNn	rs5757682	22	39848259	1.64E-06	0.005352171	3.22E-14
_G1FNn	rs5757683	22	39850174	2.16E-06	0.004729439	3.51E-14
_G1FNn	rs5757684	22	39851584	2.24E-06	0.005268323	5.04E-14
_G1FNn	rs5757685	22	39855540	1.69E-06	0.007433881	8.17E-14
_G1FNn	rs5995735	22	39854421	1.57E-06	0.007324991	7.04E-14
_G1FNn	rs6001566	22	39774448	5.90E-05	0.004769216	8.08E-12
_G1FNn	rs6001567	22	39775400	8.58E-05	0.003844947	9.40E-12
_G1FNn	rs6001568	22	39775786	7.63E-05	0.004040266	8.94E-12
_G1FNn	rs6001585	22	39812986	9.20E-05	0.013309793	1.63E-10
_G1FNn	rs6001587	22	39819008	1.72E-05	0.010251177	6.55E-12
_G1FNn	rs6001588	22	39819049	2.14E-05	0.008274868	5.74E-12
_G1FNn	rs6001594	22	39837472	2.26E-06	0.004919728	4.60E-14
_G1FNn	rs6001595	22	39839293	1.24E-06	0.008916495	8.06E-14
_G1FNn	rs6001599	22	39852720	1.69E-06	0.006908963	6.39E-14
_G1FNn	rs6001600	22	39852921	2.16E-06	0.005815058	6.46E-14
_G1FNn	rs6519190	22	39774525	6.87E-05	0.005258362	1.29E-11
_G1FNn	rs7286714	22	39765604	0.000635853	0.009691935	2.04E-09
_G1FNn	rs7286917	22	39860868	2.60E-06	0.010415727	3.48E-13
_G1FNn	rs7288760	22	39819969	1.04E-05	0.007067802	1.26E-12
_G1FNn	rs7292066	22	39794241	2.29E-05	0.006624393	3.68E-12
_G1FNn	rs73167342	22	39834102	1.07E-06	0.008760256	6.16E-14
_G1FNn	rs7364148	22	39842165	1.79E-06	0.005190201	3.65E-14
_G1FNn	rs738285	22	39856356	1.69E-06	0.007656029	8.80E-14
_G1FNn	rs738286	22	39855575	2.13E-06	0.00645568	8.17E-14
_G1FNn	rs738287	22	39855728	1.71E-06	0.007488869	8.48E-14
_G1FNn	rs738289	22	39855883	1.71E-06	0.007599941	8.80E-14
_G1FNn	rs738290	22	39856032	0.000474465	0.009506602	1.19E-09
_G1FNn	rs739141	22	39824450	3.02E-05	0.016109197	4.54E-11

_G1FNn	rs7423	22	39781429	7.43E-05	0.004465588	1.06E-11
_G1FNn	rs743838	22	39824707	2.85E-05	0.006738491	5.61E-12
_G1FNn	rs756640	22	39779300	7.43E-05	0.004730089	1.27E-11
_G1FNn	rs7949	22	39827553	2.37E-05	0.006791933	4.27E-12
_G1FNn	rs8136980	22	39844574	2.20E-06	0.004692212	3.94E-14
_G1FNn	rs8137426	22	39844350	1.79E-06	0.005110603	3.51E-14
_G1FNn	rs8138462	22	39839670	1.88E-06	0.005402154	4.32E-14
_G1FNn	rs909674	22	39859169	2.41E-06	0.006822471	1.03E-13
_G1FNn	rs9306335	22	39843537	1.84E-06	0.004206276	2.20E-14
_G1FNn	rs9611162	22	39767247	0.000635853	0.009394987	1.91E-09
_G1FNn	rs9611164	22	39767291	0.000653539	0.009444274	2.02E-09
_G1FNn	rs9611165	22	39775250	8.58E-05	0.003844947	9.40E-12
_G1FNn	rs9611166	22	39775268	8.48E-05	0.003844947	9.23E-12
_G1FNn	rs9611167	22	39775583	8.26E-05	0.00496779	1.54E-11
_G1FNn	rs9611169	22	39783027	8.25E-05	0.004615005	1.31E-11
_G1FNn	rs9611170	22	39784845	2.19E-05	0.007700389	5.03E-12
_G1FNn	rs9611176	22	39838003	1.54E-06	0.005973723	4.08E-14
_G1FNn	rs6001582	22	39806153	2.63E-05	0.007640569	6.38E-12
_G1n	rs1003401	14	66237720	0.010780016	2.49E-12	8.66E-06
_G1n	rs10138570	14	65860038	0.000346348	4.13E-24	6.91E-11
_G1n	rs10138671	14	65860092	0.009974309	2.32E-15	9.39E-08
_G1n	rs10483780	14	65980170	0.027980452	2.73E-18	7.62E-11
_G1n	rs10483785	14	66219517	0.032892704	1.47E-17	1.78E-10
_G1n	rs1054218	14	66209190	0.024362197	2.47E-14	7.91E-08
_G1n	rs1075566	14	66239457	0.02106213	2.61E-15	2.36E-08
_G1n	rs111568962	14	65897898	0.012151411	3.18E-19	1.17E-10
_G1n	rs11158591	14	65855762	0.00129444	9.10E-18	9.39E-08
_G1n	rs11158592	14	65859968	0.000380735	1.98E-24	3.61E-11
_G1n	rs11158593	14	65859984	0.000372829	2.04E-24	3.82E-11
_G1n	rs11158596	14	65911975	0.016773758	4.07E-19	6.72E-11
_G1n	rs11158601	14	66025363	0.028179919	1.43E-18	4.60E-11
_G1n	rs11158602	14	66071733	0.02378546	1.72E-19	1.45E-11
_G1n	rs11158611	14	66235074	0.024130498	2.32E-15	1.60E-08
_G1n	rs113131712	14	66064120	0.025087489	1.61E-18	7.41E-11
_G1n	rs11620749	14	65899804	0.017158211	5.14E-19	7.32E-11
_G1n	rs11621121	14	65822493	0.001894847	6.02E-18	3.52E-08
_G1n	rs11621604	14	65840774	0.003559529	3.29E-17	3.98E-08
_G1n	rs11621680	14	66014681	0.016570497	9.59E-20	2.37E-11
_G1n	rs11622014	14	66041645	0.020072474	1.19E-18	9.55E-11
_G1n	rs11622271	14	66149065	0.024206227	1.11E-18	5.48E-11
_G1n	rs11622829	14	66191782	0.023036439	6.03E-19	3.93E-11
_G1n	rs11623662	14	66021192	0.031197208	1.05E-14	2.68E-08

_G1n	rs11623920	14	65819314	0.002014285	5.18E-18	2.88E-08
_G1n	rs11624045	14	66108018	0.026051833	1.06E-18	4.52E-11
_G1n	rs11624104	14	66196137	0.038255624	1.02E-17	8.77E-11
_G1n	rs11625362	14	66232869	0.020115224	1.62E-15	1.86E-08
_G1n	rs11625882	14	66245199	0.02106213	3.04E-15	2.63E-08
_G1n	rs11627067	14	66182953	0.035123546	1.96E-17	1.67E-10
_G1n	rs11627084	14	65978836	0.024415448	9.63E-19	4.96E-11
_G1n	rs11627184	14	66121443	0.025466717	1.20E-18	5.08E-11
_G1n	rs11627185	14	66121492	0.025466717	1.20E-18	5.08E-11
_G1n	rs11627402	14	65841322	0.005588732	8.36E-16	1.40E-07
_G1n	rs11627578	14	66234448	0.023854197	2.27E-15	1.48E-08
_G1n	rs11627598	14	65797766	0.000136267	4.44E-15	6.27E-05
_G1n	rs11627605	14	66234313	0.023511935	2.32E-15	1.55E-08
_G1n	rs11628196	14	65891241	0.022209354	6.18E-19	4.40E-11
_G1n	rs11628611	14	65828525	0.155016022	4.68E-12	4.00E-08
_G1n	rs11628765	14	66168449	0.027365768	9.80E-19	3.62E-11
_G1n	rs11628840	14	66235642	0.023614565	1.48E-15	1.22E-08
_G1n	rs11851576	14	65900283	0.124646086	8.74E-12	1.24E-07
_G1n	rs12050182	14	66243080	0.021216254	4.01E-15	3.08E-08
_G1n	rs12050231	14	66235016	0.024130498	2.32E-15	1.60E-08
_G1n	rs12586842	14	66145022	0.025283239	1.39E-18	5.80E-11
_G1n	rs12588838	14	66162638	0.026591962	1.50E-18	5.38E-11
_G1n	rs12589698	14	65920435	0.011664836	3.44E-19	1.37E-10
_G1n	rs12878872	14	65804665	0.00115318	3.39E-18	6.03E-08
_G1n	rs12879202	14	65977182	0.013018794	5.54E-19	1.50E-10
_G1n	rs12879805	14	66047795	0.027782204	1.66E-18	5.35E-11
_G1n	rs12879971	14	65901604	0.015665415	4.28E-19	8.24E-11
_G1n	rs12880725	14	65799805	0.000128067	3.35E-15	5.75E-05
_G1n	rs12881755	14	65801811	0.013474389	1.85E-09	0.000430509
_G1n	rs12881911	14	65831221	0.003170538	2.06E-17	3.59E-08
_G1n	rs12882200	14	66157861	0.026212199	2.02E-18	6.99E-11
_G1n	rs12882269	14	65847144	0.002832136	3.45E-17	5.57E-08
_G1n	rs12883382	14	66145396	0.025837133	2.34E-18	8.11E-11
_G1n	rs12883815	14	65923367	0.01175723	3.27E-19	1.29E-10
_G1n	rs12886005	14	65809247	0.002089933	5.44E-18	2.79E-08
_G1n	rs12886168	14	65809286	0.002032958	2.26E-17	7.42E-08
_G1n	rs12886334	14	66094671	0.027001278	1.12E-18	4.27E-11
_G1n	rs12887134	14	66045543	0.019924474	9.29E-19	8.09E-11
_G1n	rs12887673	14	66221771	0.033930359	1.74E-17	1.67E-10
_G1n	rs12887957	14	66246592	0.021842618	5.05E-15	3.50E-08
_G1n	rs12888212	14	66200458	0.034166184	2.10E-17	1.84E-10
_G1n	rs12889002	14	66063582	0.027585168	1.43E-18	4.87E-11

_G1n	rs12890123	14	65998069	0.013430355	5.10E-19	1.35E-10
_G1n	rs12890902	14	66116622	0.026212199	1.06E-18	4.15E-11
_G1n	rs12890978	14	65982890	0.014178319	8.62E-19	1.75E-10
_G1n	rs12892058	14	65903441	0.011664836	2.48E-19	1.08E-10
_G1n	rs12893094	14	65789077	0.00013267	4.58E-15	6.35E-05
_G1n	rs12894034	14	66238763	0.020813006	1.61E-15	1.82E-08
_G1n	rs12894466	14	66240767	0.020908982	2.72E-15	2.47E-08
_G1n	rs12894902	14	66161799	0.025313196	1.43E-18	6.09E-11
_G1n	rs12895074	14	65976793	0.016619062	7.69E-19	1.06E-10
_G1n	rs12895337	14	65807442	0.019348217	1.63E-10	5.18E-05
_G1n	rs12895401	14	66064827	0.193799427	4.39E-12	1.88E-08
_G1n	rs12896663	14	66104969	0.026051833	1.09E-18	4.60E-11
_G1n	rs139525066	14	66184172	0.022703382	1.06E-14	5.33E-08
_G1n	rs1535173	14	66199139	0.033695926	1.69E-17	1.67E-10
_G1n	rs1815654	14	65855232	0.001193455	5.38E-18	7.65E-08
_G1n	rs1953416	14	65878807	0.010948581	4.73E-19	2.06E-10
_G1n	rs1954052	14	65805709	0.002002628	4.11E-18	2.50E-08
_G1n	rs1958559	14	66052055	0.195519	3.22E-12	1.44E-08
_G1n	rs1958560	14	66036795	0.178359574	1.98E-12	1.28E-08
_G1n	rs1958561	14	66036761	0.027782204	1.33E-18	4.52E-11
_G1n	rs1984855	14	66239257	0.010847209	3.52E-12	1.14E-05
_G1n	rs1998036	14	66138199	0.027671941	1.71E-18	4.90E-11
_G1n	rs2002692	14	65899454	0.017158211	5.27E-19	7.45E-11
_G1n	rs2064694	14	66148246	0.026212199	1.39E-18	5.28E-11
_G1n	rs2064695	14	66067088	0.190081067	4.92E-12	2.20E-08
_G1n	rs2149841	14	66010319	0.017550376	4.28E-19	6.24E-11
_G1n	rs2184603	14	65930670	0.022047072	7.18E-19	5.02E-11
_G1n	rs2236067	14	66248012	0.012168967	5.15E-12	1.22E-05
_G1n	rs2268955	14	66090009	0.166277006	1.47E-12	1.29E-08
_G1n	rs2268961	14	66146765	0.026212199	1.39E-18	5.28E-11
_G1n	rs2268962	14	66147273	0.026024081	1.17E-18	4.72E-11
_G1n	rs2300865	14	66120015	0.02640149	1.14E-18	4.46E-11
_G1n	rs2411351	14	66171541	0.027170516	1.14E-18	4.13E-11
_G1n	rs2411355	14	66060707	0.031985535	1.24E-18	2.76E-11
_G1n	rs2411356	14	66053161	0.030570028	5.57E-15	1.76E-08
_G1n	rs2411357	14	66051100	0.031656407	1.28E-18	2.75E-11
_G1n	rs2411404	14	66239401	0.020605597	2.44E-15	2.36E-08
_G1n	rs2411405	14	66232086	0.023444727	1.98E-15	1.53E-08
_G1n	rs2411816	14	65994880	0.018914907	9.29E-19	9.21E-11
_G1n	rs2411822	14	65878395	0.016027484	2.28E-19	4.90E-11
_G1n	rs2411823	14	65865066	0.009368842	1.15E-15	6.33E-08
_G1n	rs3783709	14	66058664	0.031985535	1.24E-18	2.76E-11

_G1n	rs3825640	14	65961204	0.016941469	5.73E-19	9.21E-11
_G1n	rs4080329	14	66233490	0.009480288	9.54E-14	1.30E-06
_G1n	rs4143898	14	66188882	0.172517539	5.41E-12	3.24E-08
_G1n	rs4359350	14	65863748	0.015974091	1.33E-14	1.22E-07
_G1n	rs4456409	14	65897967	0.011457494	5.13E-19	1.90E-10
_G1n	rs4581615	14	66055943	0.031985535	1.24E-18	2.76E-11
_G1n	rs4587890	14	65863784	0.012240531	1.03E-15	3.48E-08
_G1n	rs4899174	14	65811400	0.00207078	5.71E-18	2.93E-08
_G1n	rs4899178	14	65862987	0.01271335	1.08E-15	3.33E-08
_G1n	rs4899179	14	65926748	0.016647299	5.36E-19	8.39E-11
_G1n	rs4902385	14	65778179	0.000557272	2.86E-13	0.000123508
_G1n	rs4902386	14	65778290	0.000553427	2.55E-13	0.000116807
_G1n	rs4902391	14	65815979	0.001785453	2.37E-17	9.46E-08
_G1n	rs4902393	14	65839514	0.002315006	1.48E-17	4.97E-08
_G1n	rs4902400	14	65957115	0.018221457	7.80E-19	8.87E-11
_G1n	rs4902407	14	66074635	0.028420809	1.68E-19	8.78E-12
_G1n	rs4902415	14	66235690	0.023785469	1.52E-15	1.22E-08
_G1n	rs4902416	14	66238090	0.021331505	1.65E-15	1.60E-08
_G1n	rs6573609	14	65861402	0.012240531	1.08E-15	3.59E-08
_G1n	rs6573611	14	65865255	0.00945501	8.53E-16	5.54E-08
_G1n	rs6573615	14	66046534	0.032050596	9.09E-15	2.26E-08
_G1n	rs6573624	14	66226885	0.040356546	1.96E-17	1.25E-10
_G1n	rs6573625	14	66240634	0.008277781	3.14E-13	3.68E-06
_G1n	rs6573626	14	66240695	0.024023844	1.26E-15	1.04E-08
_G1n	rs67957534	14	66124585	0.025466717	1.20E-18	5.08E-11
_G1n	rs7142018	14	66241673	0.017013603	3.62E-14	2.35E-07
_G1n	rs7142219	14	66241781	0.020646899	3.72E-15	3.17E-08
_G1n	rs7142651	14	66132721	0.025837133	1.36E-18	5.38E-11
_G1n	rs7143666	14	65809977	0.019674138	3.63E-11	1.94E-05
_G1n	rs7146829	14	66194531	0.032888668	2.80E-17	2.82E-10
_G1n	rs7148737	14	66230032	0.014942047	3.13E-12	5.84E-06
_G1n	rs7149301	14	66230414	0.019101086	1.23E-15	1.66E-08
_G1n	rs7152023	14	66160827	0.192077779	1.40E-11	5.07E-08
_G1n	rs7152441	14	65827903	0.020796159	1.82E-19	2.26E-11
_G1n	rs7153297	14	66003466	0.011625255	2.51E-19	1.13E-10
_G1n	rs7154536	14	65991737	0.152555146	7.17E-12	5.92E-08
_G1n	rs7155541	14	66046242	0.019486181	8.41E-19	7.94E-11
_G1n	rs7155963	14	66076029	0.018495574	6.52E-19	7.47E-11
_G1n	rs7156078	14	66238701	0.01146572	2.96E-12	8.77E-06
_G1n	rs7157006	14	65828207	0.002770597	1.06E-17	2.67E-08
_G1n	rs7157449	14	66240137	0.020908982	2.78E-15	2.51E-08
_G1n	rs7158347	14	65828955	0.003084522	2.01E-17	3.37E-08

_G1n	rs7158556	14	66240729	0.008277781	3.20E-13	3.73E-06
_G1n	rs7160780	14	66052713	0.029348116	3.12E-15	1.27E-08
_G1n	rs7161123	14	66052901	0.031985535	1.24E-18	2.76E-11
_G1n	rs730807	14	66239290	0.02045535	2.33E-15	2.32E-08
_G1n	rs743084	14	66232602	0.02092401	2.17E-15	1.92E-08
_G1n	rs743085	14	66068133	0.027585168	1.43E-18	4.87E-11
_G1n	rs747541	14	65805410	0.002032958	2.06E-17	6.98E-08
_G1n	rs7493004	14	66124104	0.02640149	1.20E-18	4.63E-11
_G1n	rs761830	14	66212986	0.025772404	1.18E-14	4.10E-08
_G1n	rs761831	14	66239370	0.02045535	2.44E-15	2.39E-08
_G1n	rs78230848	14	66016384	0.019486178	1.35E-18	1.10E-10
_G1n	rs8003811	14	66211250	0.026319149	1.31E-14	4.23E-08
_G1n	rs8005710	14	66065666	0.025708889	1.45E-18	5.90E-11
_G1n	rs8010876	14	66206976	0.035366434	1.54E-17	1.36E-10
_G1n	rs8012278	14	66082573	0.027980454	1.76E-18	5.31E-11
_G1n	rs8014475	14	65811537	0.019211076	1.27E-10	4.46E-05
_G1n	rs8016322	14	66051536	0.027404946	2.68E-15	1.33E-08
_G1n	rs8018278	14	66180088	0.024592755	8.55E-19	4.32E-11
_G1n	rs8022094	14	65846841	0.003349779	2.61E-17	3.46E-08
_G1n	rs8022764	14	66244655	0.022176798	1.41E-15	1.44E-08
_G1n	rs80297942	14	66100698	0.034042436	1.62E-17	1.73E-10
_G1n	rs867972	14	65895761	0.010189751	2.24E-19	1.37E-10
_G1n	rs883081	14	65880621	0.014622035	1.52E-19	4.54E-11
_G1n	rs883082	14	65880940	0.015428055	1.88E-19	4.51E-11
_G1n	rs899959	14	65791928	0.000151744	1.88E-15	3.41E-05
_G1n	rs927004	14	66200911	0.033930357	1.58E-17	1.55E-10
_G1n	rs9796385	14	66101219	0.01212717	3.60E-18	7.00E-10
_G1n	rs9972106	14	66023131	0.022070182	6.19E-15	3.94E-08
_G1n	rs10047879	14	65760543	0.001244616	4.97E-11	0.00090029
_G1n	rs10131385	14	65773555	0.003854096	3.30E-08	0.008643573
_G1n	rs10132229	14	65777560	0.002500552	1.92E-08	0.009527516
_G1n	rs10134589	14	65781234	0.000520477	4.71E-14	4.76E-05
_G1n	rs10135194	14	65770978	0.004415964	3.50E-08	0.008136003
_G1n	rs10137464	14	65785829	0.000656012	1.28E-13	6.56E-05
_G1n	rs10138052	14	65833440	0.001350452	1.41E-14	8.03E-06
_G1n	rs10138151	14	65789729	0.003279909	1.92E-08	0.007595328
_G1n	rs10138662	14	65779482	0.000656012	1.26E-13	6.50E-05
_G1n	rs10142617	14	65773671	0.000719507	1.38E-13	6.28E-05
_G1n	rs10144503	14	65784109	0.003341748	2.98E-08	0.00935339
_G1n	rs10144975	14	65773982	0.000741911	1.10E-13	5.34E-05
_G1n	rs10147958	14	65778833	0.003341748	3.01E-08	0.009398625
_G1n	rs10148907	14	65833372	0.001350452	1.41E-14	8.03E-06

_G1n	rs10150168	14	65791249	0.003530282	2.41E-08	0.007975656
_G1n	rs11158587	14	65782712	0.000656012	1.28E-13	6.56E-05
_G1n	rs111950176	14	65843359	0.002654307	1.35E-10	0.000703188
_G1n	rs112850324	14	66241579	0.004976772	1.82E-11	0.00010845
_G1n	rs11845834	14	66260602	0.204671457	1.71E-08	1.27E-05
_G1n	rs11847263	14	65775695	0.000384014	1.38E-16	2.57E-06
_G1n	rs11848443	14	66257419	0.061654798	2.97E-13	6.32E-08
_G1n	rs11848444	14	66257467	0.061272242	2.97E-13	6.42E-08
_G1n	rs11848463	14	66257294	0.061272242	2.91E-13	6.32E-08
_G1n	rs11850120	14	66260379	0.203709935	1.68E-08	1.27E-05
_G1n	rs12147994	14	65751627	0.002041272	2.42E-08	0.013012768
_G1n	rs12436299	14	65785194	0.003341748	2.98E-08	0.00935339
_G1n	rs1256519	14	65736324	0.010044381	1.30E-08	0.001999779
_G1n	rs1256536	14	65762476	0.00078146	3.17E-11	0.001084054
_G1n	rs1256540	14	65764069	0.000770017	1.12E-10	0.002159056
_G1n	rs1269068	14	65767333	0.000542246	7.04E-11	0.002401296
_G1n	rs17102587	14	65774477	0.000721684	1.22E-13	5.58E-05
_G1n	rs17102598	14	65784860	0.000656012	1.28E-13	6.56E-05
_G1n	rs1760978	14	65771047	8.86E-05	2.16E-14	0.000219006
_G1n	rs1950557	14	66201757	0.005206388	1.12E-12	1.58E-05
_G1n	rs28459012	14	66258528	0.060767453	7.00E-13	1.15E-07
_G1n	rs28470717	14	66251050	0.07057045	7.44E-13	8.46E-08
_G1n	rs28557275	14	65790415	0.003420923	2.70E-08	0.008703445
_G1n	rs28595734	14	65792269	0.003530282	2.39E-08	0.00793687
_G1n	rs28616692	14	66258683	0.060011967	6.32E-13	1.10E-07
_G1n	rs28740895	14	65783523	0.002137333	1.52E-13	1.70E-05
_G1n	rs35206478	14	66223213	0.004621679	1.12E-12	1.95E-05
_G1n	rs3742597	14	66200177	0.004980205	7.72E-13	1.39E-05
_G1n	rs4073415	14	66259530	0.062690706	8.39E-13	1.22E-07
_G1n	rs4131095	14	66259437	0.187022434	7.47E-09	7.99E-06
_G1n	rs4131097	14	66259165	0.015447385	2.62E-11	2.24E-05
_G1n	rs4400971	14	66254578	0.055312648	4.15E-13	1.06E-07
_G1n	rs4439682	14	66259428	0.048688592	5.28E-14	3.02E-08
_G1n	rs55845504	14	66256968	0.027637923	1.85E-11	6.89E-06
_G1n	rs56379231	14	66255397	0.030389106	3.44E-11	8.85E-06
_G1n	rs56720460	14	66256336	0.058772539	6.22E-13	1.14E-07
_G1n	rs56922569	14	66256472	0.059761631	2.74E-13	6.42E-08
_G1n	rs57071950	14	65788437	0.000708542	2.09E-13	7.91E-05
_G1n	rs57507878	14	65843372	0.003687029	5.31E-10	0.001056352
_G1n	rs58102398	14	66250157	0.004797064	1.40E-12	2.12E-05
_G1n	rs59636756	14	66256489	0.059761631	2.74E-13	6.42E-08
_G1n	rs59853675	14	66250679	0.004322369	9.91E-13	2.03E-05

_G1n	rs60273099	14	66214850	0.004906065	8.29E-13	1.44E-05
_G1n	rs60488013	14	65825854	0.00133907	9.46E-15	6.39E-06
_G1n	rs60734251	14	66250274	0.004832807	1.45E-12	2.15E-05
_G1n	rs61161789	14	65843373	0.004162308	9.38E-10	0.001233302
_G1n	rs61298903	14	66218595	0.00476186	9.21E-13	1.60E-05
_G1n	rs61348705	14	66111019	0.002405332	9.92E-12	0.000180104
_G1n	rs61378474	14	66253437	0.053589941	1.46E-13	5.22E-08
_G1n	rs61988977	14	66258032	0.050154308	6.61E-13	1.74E-07
_G1n	rs61988978	14	66258111	0.069483614	1.86E-13	3.01E-08
_G1n	rs6573598	14	65783019	0.000656012	1.28E-13	6.56E-05
_G1n	rs6573599	14	65783127	0.000656012	1.28E-13	6.56E-05
_G1n	rs6573600	14	65783270	0.000685009	1.56E-13	7.14E-05
_G1n	rs6573602	14	65784610	0.000656012	1.28E-13	6.56E-05
_G1n	rs6573604	14	65787941	0.000692386	1.81E-13	7.50E-05
_G1n	rs6573627	14	66252326	0.024999747	5.95E-14	1.65E-07
_G1n	rs66492349	14	65839489	0.001408716	1.99E-14	9.31E-06
_G1n	rs67662065	14	66250999	0.003746639	1.69E-12	3.44E-05
_G1n	rs7142165	14	66250232	0.025430995	1.66E-14	5.91E-08
_G1n	rs7143026	14	66250956	0.011825238	1.63E-12	5.98E-06
_G1n	rs7151212	14	65781622	0.000629092	3.61E-13	0.000119889
_G1n	rs7151301	14	65781496	0.000656012	1.26E-13	6.50E-05
_G1n	rs7151846	14	66255781	0.055414126	4.31E-13	1.06E-07
_G1n	rs7159888	14	65758642	0.005592446	2.85E-12	2.75E-05
_G1n	rs72712459	14	65734600	0.002845887	1.15E-09	0.002051099
_G1n	rs73278053	14	65788490	0.003400431	2.60E-08	0.008589896
_G1n	rs8004239	14	65783460	0.00064595	9.59E-14	5.68E-05
_G1n	rs8006316	14	65787115	0.000661097	1.45E-13	6.98E-05
_G1n	rs8007846	14	66262963	0.037333631	2.34E-11	4.31E-06
_G1n	rs8010759	14	65784559	0.002721385	2.68E-08	0.010738192
_G1n	rs8012054	14	65774762	0.000764962	1.99E-13	7.05E-05
_G1n	rs8013568	14	65774875	0.000710705	1.17E-13	5.53E-05
_G1n	rs8017974	14	65775187	0.000676574	1.04E-13	5.63E-05
_G1n	rs8018379	14	66261937	0.026240818	8.16E-13	8.57E-07
_G1n	rs8019473	14	65779128	0.000709564	1.59E-13	6.60E-05
_G1n	rs8019767	14	65782785	0.000656012	1.28E-13	6.56E-05
_G1n	rs9635250	14	65799348	0.003647107	2.72E-08	0.00823097
_G1n	rs968540	14	66249064	0.009517335	5.27E-13	3.84E-06
_G1N	rs11847263	14	65775695	1.14E-07	1	1
_G1N/G1	rs6421315	7	50355207	5.10E-10	1	1
_G1N/G1	rs7782210	7	50348745	2.93E-10	1	1
_G1N/G1	rs7789913	7	50352695	4.65E-10	1	1
_G1N/G1	rs7797255	7	50351604	4.06E-10	1	1

_G1N/G1	rs7804185	7	50353144	3.75E-10	1	1
_G1Nn	rs11847263	14	65775695	1.10E-07	1	1
_G1NS1/G1N	rs10138570	14	65860038	1.02E-07	1	1
_G1NS1/G1N	rs11158592	14	65859968	1.54E-07	1	1
_G1NS1/G1N	rs11158593	14	65859984	1.50E-07	1	1
_G1NS1/G1N	rs7152441	14	65827903	2.89E-07	1	1
_G2F/G2	rs10138052	14	65833440	5.12E-08	1	1
_G2F/G2	rs10148907	14	65833372	5.12E-08	1	1
_G2F/G2	rs112850324	14	66241579	3.18E-07	1	1
_G2F/G2	rs11847263	14	65775695	6.09E-08	1	1
_G2F/G2	rs1760978	14	65771047	5.86E-08	1	1
_G2F/G2	rs1950557	14	66201757	2.28E-07	1	1
_G2F/G2	rs35206478	14	66223213	2.10E-07	1	1
_G2F/G2	rs3742597	14	66200177	1.97E-07	1	1
_G2F/G2	rs58102398	14	66250157	2.43E-07	1	1
_G2F/G2	rs59853675	14	66250679	1.97E-07	1	1
_G2F/G2	rs60273099	14	66214850	1.96E-07	1	1
_G2F/G2	rs60488013	14	65825854	5.78E-08	1	1
_G2F/G2	rs60734251	14	66250274	2.40E-07	1	1
_G2F/G2	rs61298903	14	66218595	1.76E-07	1	1
_G2F/G2	rs61348705	14	66111019	2.35E-07	1	1
_G2F/G2	rs66492349	14	65839489	8.02E-08	1	1
_G2F/G2	rs67662065	14	66250999	1.71E-07	1	1
_G2F/G2	rs10138570	14	65860038	5.62E-09	1	1
_G2F/G2	rs11158591	14	65855762	5.20E-09	1	1
_G2F/G2	rs11158592	14	65859968	5.72E-09	1	1
_G2F/G2	rs11158593	14	65859984	5.53E-09	1	1
_G2F/G2	rs11621121	14	65822493	2.14E-08	1	1
_G2F/G2	rs11621604	14	65840774	4.95E-08	1	1
_G2F/G2	rs11623920	14	65819314	2.50E-08	1	1
_G2F/G2	rs11627402	14	65841322	2.00E-07	1	1
_G2F/G2	rs11627598	14	65797766	1.32E-07	1	1
_G2F/G2	rs12878872	14	65804665	1.68E-08	1	1
_G2F/G2	rs12880725	14	65799805	1.20E-07	1	1
_G2F/G2	rs12881911	14	65831221	2.97E-08	1	1
_G2F/G2	rs12882269	14	65847144	2.49E-08	1	1
_G2F/G2	rs12886005	14	65809247	2.54E-08	1	1
_G2F/G2	rs12886168	14	65809286	3.65E-08	1	1
_G2F/G2	rs12893094	14	65789077	1.33E-07	1	1
_G2F/G2	rs1815654	14	65855232	8.04E-09	1	1
_G2F/G2	rs1954052	14	65805709	2.24E-08	1	1
_G2F/G2	rs4899174	14	65811400	2.54E-08	1	1

_G2F/G2	rs4902391	14	65815979	3.17E-08	1	1
_G2F/G2	rs4902393	14	65839514	3.48E-08	1	1
_G2F/G2	rs7157006	14	65828207	2.17E-08	1	1
_G2F/G2	rs7158347	14	65828955	2.62E-08	1	1
_G2F/G2	rs747541	14	65805410	3.59E-08	1	1
_G2F/G2	rs8022094	14	65846841	2.87E-08	1	1
_G2F/G2	rs899959	14	65791928	1.70E-07	1	1
_G2FN/G2F	rs1005522	22	39845898	2.10E-05	0.336355559	1.71E-07
_G2FN/G2F	rs6001595	22	39839293	3.72E-05	0.201639816	6.29E-08
_G2FS1/G2S1	rs10138052	14	65833440	3.64E-07	1	1
_G2FS1/G2S1	rs10148907	14	65833372	3.64E-07	1	1
_G2FS1/G2S1	rs11847263	14	65775695	3.12E-07	1	1
_G2FS1/G2S1	rs60488013	14	65825854	3.67E-07	1	1
_G2n	rs10138570	14	65860038	2.59E-05	3.00E-12	0.005339771
_G2n	rs11158592	14	65859968	2.97E-05	2.02E-12	0.004078686
_G2n	rs11158593	14	65859984	2.79E-05	1.98E-12	0.004224591
_G2n	rs11627598	14	65797766	2.72E-05	6.35E-09	0.105403885
_G2n	rs12880725	14	65799805	2.53E-05	5.87E-09	0.106172369
_G2n	rs12893094	14	65789077	2.66E-05	5.28E-09	0.09834479
_G2n	rs899959	14	65791928	2.82E-05	3.78E-09	0.086552332
_G2n	rs11847263	14	65775695	0.000107217	1.11E-10	0.009738676
_G2n	rs1760978	14	65771047	2.49E-05	9.35E-09	0.125105361
_G0Fn	rs1005522	22	39845898	0.140352724	0.00031025	3.83E-07
_G0Fn	rs10644269	22	39836972	0.13568659	0.000228617	2.23E-07
_G0Fn	rs113200473	22	39853740	0.119724268	0.000376745	3.15E-07
_G0Fn	rs1557541	22	39851970	0.110415322	0.000221379	1.19E-07
_G0Fn	rs1557542	22	39852350	0.109320409	0.000232397	1.24E-07
_G0Fn	rs1557543	22	39852648	0.116656422	0.000252507	1.75E-07
_G0Fn	rs1972280	22	39831986	0.143140214	0.000211677	2.33E-07
_G0Fn	rs2008174	22	39860130	0.082228759	0.000594082	2.35E-07
_G0Fn	rs2413592	22	39858196	0.107069393	0.000461728	3.12E-07
_G0Fn	rs2899318	22	39837625	0.151529936	0.00019891	2.60E-07
_G0Fn	rs3959642	22	39860589	0.083514489	0.000573157	2.32E-07
_G0Fn	rs4821896	22	39833437	0.139747849	0.000195503	1.95E-07
_G0Fn	rs4821897	22	39835587	0.147962746	0.000200906	2.38E-07
_G0Fn	rs4821898	22	39838352	0.162541582	0.000203955	3.18E-07
_G0Fn	rs5750823	22	39829973	0.152944239	0.000202398	2.64E-07
_G0Fn	rs5750825	22	39831278	0.155136943	0.00020366	2.91E-07
_G0Fn	rs5750828	22	39835083	0.124425854	0.000123415	7.54E-08
_G0Fn	rs5750829	22	39838018	0.112357137	0.000243024	1.42E-07
_G0Fn	rs5750830	22	39840828	0.134676038	0.000343801	3.76E-07
_G0Fn	rs5750833	22	39843091	0.129580769	0.000267811	2.40E-07

_G0Fn	rs5757673	22	39837920	0.137214107	0.000212687	2.08E-07
_G0Fn	rs5757675	22	39838892	0.135759098	0.000231371	2.27E-07
_G0Fn	rs5757678	22	39843409	0.126423893	0.000290515	2.63E-07
_G0Fn	rs5757680	22	39844793	0.134107369	0.000318786	3.35E-07
_G0Fn	rs5757681	22	39845547	0.139954603	0.000285326	3.40E-07
_G0Fn	rs5757682	22	39848259	0.112494549	0.000247242	1.48E-07
_G0Fn	rs5757683	22	39850174	0.112494542	0.000190671	1.02E-07
_G0Fn	rs5757684	22	39851584	0.110966019	0.00021711	1.18E-07
_G0Fn	rs5757685	22	39855540	0.116718553	0.000278343	1.93E-07
_G0Fn	rs5995735	22	39854421	0.119025004	0.000267907	1.93E-07
_G0Fn	rs6001594	22	39837472	0.151519091	0.000200906	2.55E-07
_G0Fn	rs6001595	22	39839293	0.153734864	0.000202976	2.69E-07
_G0Fn	rs6001599	22	39852720	0.112211085	0.000282199	1.75E-07
_G0Fn	rs6001600	22	39852921	0.109866787	0.000236007	1.31E-07
_G0Fn	rs7286917	22	39860868	0.087302566	0.000595666	2.62E-07
_G0Fn	rs73167342	22	39834102	0.131942908	0.000177458	1.46E-07
_G0Fn	rs7364148	22	39842165	0.131473667	0.00032321	3.28E-07
_G0Fn	rs738285	22	39856356	0.116781588	0.000289159	2.11E-07
_G0Fn	rs738286	22	39855575	0.104654556	0.000253969	1.25E-07
_G0Fn	rs738287	22	39855728	0.116718551	0.000286419	2.01E-07
_G0Fn	rs738289	22	39855883	0.116718551	0.000289159	2.04E-07
_G0Fn	rs8136980	22	39844574	0.1204908	0.000320136	2.64E-07
_G0Fn	rs8137426	22	39844350	0.132109373	0.00032321	3.32E-07
_G0Fn	rs8138462	22	39839670	0.135759098	0.000231371	2.27E-07
_G0Fn	rs909674	22	39859169	0.083753712	0.000440891	1.61E-07
_G0Fn	rs9306335	22	39843537	0.13998305	0.000272293	3.08E-07
_G0Fn	rs9611176	22	39838003	0.14940525	0.000242065	3.19E-07
_G1FN	rs28685576	22	39841533	0.001891185	0.002194675	8.24E-10
_G1FNn	rs28685576	22	39841533	0.002546871	0.006697994	1.10E-08
_G1FNn	rs5750826	22	39832113	0.002010959	0.007142458	7.67E-09
_G2FN/G2F	rs1007337	22	39781585	0.000744184	0.046527822	8.18E-08
_G2FN/G2F	rs1010169	22	39778167	0.000610538	0.056027599	9.37E-08
_G2FN/G2F	rs1010170	22	39778327	0.000587518	0.057054343	9.24E-08
_G2FN/G2F	rs10644269	22	39836972	4.32E-05	0.1368431	2.35E-08
_G2FN/G2F	rs113200473	22	39853740	3.12E-05	0.209668666	5.39E-08
_G2FN/G2F	rs1557541	22	39851970	2.52E-05	0.134074339	1.08E-08
_G2FN/G2F	rs1557542	22	39852350	2.63E-05	0.134760008	1.09E-08
_G2FN/G2F	rs1557543	22	39852648	2.39E-05	0.16698252	1.96E-08
_G2FN/G2F	rs1972280	22	39831986	4.49E-05	0.146545097	3.03E-08
_G2FN/G2F	rs2008174	22	39860130	2.46E-05	0.135792301	1.08E-08
_G2FN/G2F	rs2142848	22	39785242	0.00026478	0.076166024	5.83E-08
_G2FN/G2F	rs2413590	22	39790191	0.000251768	0.080157391	6.18E-08

_G2FN/G2F	rs2413592	22	39858196	3.76E-05	0.163902688	3.31E-08
_G2FN/G2F	rs2899318	22	39837625	4.22E-05	0.130361262	1.97E-08
_G2FN/G2F	rs35509952	22	39800563	0.000301624	0.08815972	1.02E-07
_G2FN/G2F	rs3959642	22	39860589	2.46E-05	0.139650331	1.20E-08
_G2FN/G2F	rs4321460	22	39793655	0.000533461	0.070471273	1.33E-07
_G2FN/G2F	rs4337572	22	39800704	0.000286919	0.076166024	6.55E-08
_G2FN/G2F	rs4384886	22	39793766	0.000278818	0.072980182	5.64E-08
_G2FN/G2F	rs4386422	22	39793734	0.000540044	0.106889901	3.85E-07
_G2FN/G2F	rs4429561	22	39799789	0.000273704	0.109842807	1.58E-07
_G2FN/G2F	rs4821889	22	39777254	0.000768143	0.049928423	9.58E-08
_G2FN/G2F	rs4821890	22	39777523	0.000760961	0.049928423	9.45E-08
_G2FN/G2F	rs4821891	22	39785381	0.000262128	0.078808462	6.27E-08
_G2FN/G2F	rs4821892	22	39795683	0.000249239	0.080611182	6.18E-08
_G2FN/G2F	rs4821893	22	39797779	0.00025906	0.082700351	6.98E-08
_G2FN/G2F	rs4821894	22	39809820	0.000207065	0.088448809	6.03E-08
_G2FN/G2F	rs4821895	22	39823015	0.000261693	0.067644731	4.24E-08
_G2FN/G2F	rs4821896	22	39833437	4.68E-05	0.150812838	3.29E-08
_G2FN/G2F	rs4821897	22	39835587	4.45E-05	0.129719747	2.09E-08
_G2FN/G2F	rs4821898	22	39838352	4.25E-05	0.103556273	9.64E-09
_G2FN/G2F	rs5750808	22	39790987	0.000251768	0.080157391	6.18E-08
_G2FN/G2F	rs5750809	22	39791491	0.000317331	0.079471839	8.44E-08
_G2FN/G2F	rs5750810	22	39792943	0.000249239	0.080611182	6.18E-08
_G2FN/G2F	rs5750811	22	39793066	0.000249239	0.080611182	6.18E-08
_G2FN/G2F	rs5750812	22	39793079	0.000276045	0.073819814	5.72E-08
_G2FN/G2F	rs5750813	22	39795228	0.000239358	0.080611182	5.83E-08
_G2FN/G2F	rs5750814	22	39797987	0.000298627	0.076601342	7.04E-08
_G2FN/G2F	rs5750815	22	39798449	0.000236461	0.091948164	8.06E-08
_G2FN/G2F	rs5750816	22	39810379	0.000286539	0.068439624	4.99E-08
_G2FN/G2F	rs5750818	22	39820885	0.000237384	0.079837288	5.31E-08
_G2FN/G2F	rs5750820	22	39825322	0.00023219	0.075732726	4.76E-08
_G2FN/G2F	rs5750821	22	39825492	0.000246733	0.071097972	4.42E-08
_G2FN/G2F	rs5750822	22	39826788	0.000246733	0.071097972	4.42E-08
_G2FN/G2F	rs5750823	22	39829973	4.88E-05	0.118397315	1.89E-08
_G2FN/G2F	rs5750825	22	39831278	5.65E-05	0.10862423	1.73E-08
_G2FN/G2F	rs5750828	22	39835083	5.96E-05	0.158829752	5.60E-08
_G2FN/G2F	rs5750829	22	39838018	3.80E-05	0.185412579	4.97E-08
_G2FN/G2F	rs5750830	22	39840828	2.47E-05	0.12499218	8.48E-09
_G2FN/G2F	rs5750833	22	39843091	2.61E-05	0.11040657	6.06E-09
_G2FN/G2F	rs5757647	22	39775047	0.000809884	0.048293174	1.01E-07
_G2FN/G2F	rs5757648	22	39775156	0.000802327	0.047995099	9.86E-08
_G2FN/G2F	rs5757650	22	39778419	0.000794835	0.044817116	8.30E-08
_G2FN/G2F	rs5757652	22	39781855	0.00064347	0.059395104	1.13E-07

_G2FN/G2F	rs5757654	22	39794124	0.000297067	0.080010012	7.53E-08
_G2FN/G2F	rs5757655	22	39797178	0.000262128	0.077038686	5.92E-08
_G2FN/G2F	rs5757657	22	39798429	0.000301624	0.076601342	7.14E-08
_G2FN/G2F	rs5757659	22	39812409	0.000252317	0.081772393	6.41E-08
_G2FN/G2F	rs5757663	22	39821319	0.000265429	0.079489426	6.41E-08
_G2FN/G2F	rs5757664	22	39821536	0.00024378	0.080852902	6.03E-08
_G2FN/G2F	rs5757665	22	39821641	0.000247244	0.08223531	6.31E-08
_G2FN/G2F	rs5757667	22	39822116	0.000430107	0.068222812	8.67E-08
_G2FN/G2F	rs5757670	22	39829736	0.000324512	0.073540052	6.92E-08
_G2FN/G2F	rs5757673	22	39837920	6.07E-05	0.164067923	6.32E-08
_G2FN/G2F	rs5757675	22	39838892	4.49E-05	0.129173903	2.08E-08
_G2FN/G2F	rs5757678	22	39843409	2.17E-05	0.120143766	6.31E-09
_G2FN/G2F	rs5757680	22	39844793	2.44E-05	0.136527802	1.09E-08
_G2FN/G2F	rs5757681	22	39845547	2.39E-05	0.130715159	9.27E-09
_G2FN/G2F	rs5757682	22	39848259	2.14E-05	0.146002475	1.12E-08
_G2FN/G2F	rs5757683	22	39850174	2.34E-05	0.13271223	9.43E-09
_G2FN/G2F	rs5757684	22	39851584	2.52E-05	0.133432451	1.06E-08
_G2FN/G2F	rs5757685	22	39855540	2.49E-05	0.170033887	2.19E-08
_G2FN/G2F	rs5995735	22	39854421	2.31E-05	0.170033887	1.99E-08
_G2FN/G2F	rs6001566	22	39774448	0.000583479	0.058571061	9.55E-08
_G2FN/G2F	rs6001567	22	39775400	0.000784523	0.048293174	9.47E-08
_G2FN/G2F	rs6001568	22	39775786	0.000618828	0.057522153	9.65E-08
_G2FN/G2F	rs6001587	22	39819008	0.000235525	0.109771284	1.24E-07
_G2FN/G2F	rs6001588	22	39819049	0.000238878	0.087468959	7.18E-08
_G2FN/G2F	rs6001594	22	39837472	4.22E-05	0.130361262	1.97E-08
_G2FN/G2F	rs6001599	22	39852720	2.49E-05	0.15734971	1.73E-08
_G2FN/G2F	rs6001600	22	39852921	2.69E-05	0.136704213	1.17E-08
_G2FN/G2F	rs6519190	22	39774525	0.000566944	0.069875123	1.40E-07
_G2FN/G2F	rs7286917	22	39860868	2.01E-05	0.186849848	2.36E-08
_G2FN/G2F	rs7288760	22	39819969	0.000264269	0.083490212	7.36E-08
_G2FN/G2F	rs7292066	22	39794241	0.000253322	0.077919482	5.91E-08
_G2FN/G2F	rs73167342	22	39834102	3.81E-05	0.178192344	4.23E-08
_G2FN/G2F	rs7364148	22	39842165	2.61E-05	0.12509638	9.14E-09
_G2FN/G2F	rs738285	22	39856356	2.69E-05	0.173069971	2.56E-08
_G2FN/G2F	rs738286	22	39855575	2.99E-05	0.135437618	1.39E-08
_G2FN/G2F	rs738287	22	39855728	2.52E-05	0.171575159	2.29E-08
_G2FN/G2F	rs738289	22	39855883	2.52E-05	0.171575159	2.29E-08
_G2FN/G2F	rs738290	22	39856032	0.001954453	0.045218673	3.26E-07
_G2FN/G2F	rs7423	22	39781429	0.000737198	0.047698572	8.54E-08
_G2FN/G2F	rs743838	22	39824707	0.000292718	0.073175012	6.09E-08
_G2FN/G2F	rs756640	22	39779300	0.000751232	0.049196746	9.45E-08
_G2FN/G2F	rs7949	22	39827553	0.000236946	0.070688421	4.11E-08

_G2FN/G2F	rs8136980	22	39844574	3.17E-05	0.109365602	8.01E-09
_G2FN/G2F	rs8137426	22	39844350	2.67E-05	0.123873715	9.14E-09
_G2FN/G2F	rs8138462	22	39839670	4.44E-05	0.1285392	2.03E-08
_G2FN/G2F	rs909674	22	39859169	2.22E-05	0.129409892	8.63E-09
_G2FN/G2F	rs9306335	22	39843537	2.81E-05	0.122169385	9.43E-09
_G2FN/G2F	rs9611165	22	39775250	0.000802327	0.048293174	1.00E-07
_G2FN/G2F	rs9611166	22	39775268	0.000802327	0.048293174	1.00E-07
_G2FN/G2F	rs9611167	22	39775583	0.000775387	0.050545087	1.00E-07
_G2FN/G2F	rs9611169	22	39783027	0.000533974	0.062936255	1.01E-07
_G2FN/G2F	rs9611170	22	39784845	0.000234556	0.080157391	5.58E-08
_G2FN/G2F	rs9611176	22	39838003	4.30E-05	0.146545097	2.86E-08
_G2FN/G2F	rs6001582	22	39806153	0.000331344	0.069360275	6.03E-08
_G2n	chr9:33152337:D	9	33152337	0.419453356	8.48E-06	1.30E-07
_G2n	rs10758195	9	33174587	0.48102216	6.44E-06	1.67E-07
_G2n	rs10813951	9	33128021	0.461349381	5.88E-06	1.25E-07
_G2n	rs10813957	9	33153527	0.451023996	7.99E-06	1.63E-07
_G2n	rs10971417	9	33121480	0.452591804	5.42E-06	1.06E-07
_G2n	rs10971434	9	33168891	0.482561834	4.58E-06	1.14E-07
_G2n	rs12342831	9	33124872	0.443440829	5.09E-06	9.02E-08
_G2n	rs1969977	9	33120203	0.376481567	4.63E-06	4.16E-08
_G2n	rs3780480	9	33163486	0.512025187	1.04E-05	3.75E-07
_G2n	rs3780486	9	33139453	0.453890719	6.85E-06	1.40E-07
