# Supplementary material for: Genome-Wide Association Study on Immunoglobulin G Glycosylation Patterns
Source: Front Immunol. 2018 Feb 26;9:277. doi: 10.3389/fimmu.2018.00277 (PMC5834439; doi:10.3389/fimmu.2018.00277)
Supplement: Table S8 — Overview of results from subclass comparisons of immunoglobulin G (IgG) glycopeptide traits. [file Table_8.PDF]

Significant Subclass Comparisons of SNP-Glycan Associations on Meta-Analyzed Data

IgG Glycan Trait	Gene Locus	minimal ***p*** (IgG1 vs. IgG2/3)	minimal ***p*** (IgG1 vs. IgG4)	minimal ***p*** (IgG2/3 vs. IgG4)
_G1N/G1	*IKZF1*	2.93E-10	NA	NA
_G2n	*B4GALT1*	3.76E-01	4.58E-06	4.16E-08
BG1n/G1n	*FUT8*	2.28E-08	NA	NA
BG1S1/(BG1+BG1S1)	*FUT8*	1.29E-07	NA	NA
FBn/Bn total	*FUT8*	2.15E-07	NA	NA
FG2n total/G2n	*FUT8*	8.45E-10	NA	NA
FG2n/G2n	*FUT8*	1.32E-09	NA	NA
_G0n	*FUT8*	1.18E-02	6.54E-17	2.21E-11
_G1FN/G1N	*FUT8*	2.02E-09	NA	NA
_G1n	*FUT8*	8.86E-05	1.98E-24	8.78E-12
_G1N	*FUT8*	1.14E-07	NA	NA
_G1Nn	*FUT8*	1.10E-07	NA	NA
_G1NS1/G1N	*FUT8*	1.02E-07	NA	NA
_G2F/G2	*FUT8*	5.20E-09	NA	NA
_G2FS1/G2S1	*FUT8*	3.12E-07	NA	NA
_G2n	*FUT8*	2.49E-05	1.98E-12	4.08E-03
Bisecting_GlcNAc	*MGAT3*	5.36E-05	1.80E-02	8.97E-10
FBG0n/G0n	*MGAT3*	2.21E-07	NA	NA
_G0Fn	*MGAT3*	8.22E-02	1.23E-04	7.54E-08
_G0FN/G0F	*MGAT3*	2.72E-07	8.54E-02	1.71E-11
_G1FN	*MGAT3*	8.81E-07	6.15E-04	2.63E-16
_G1FN/G1F	*MGAT3*	6.25E-06	5.76E-03	9.41E-13
_G1FNn	*MGAT3*	1.07E-06	3.56E-03	2.20E-14
_G2FN/G2F	*MGAT3*	2.01E-05	4.48E-02	6.06E-09

significance threshold for subclass comparisons:

4.22E-07
