# Supplementary material for: Genome-Wide Association Study on Immunoglobulin G Glycosylation Patterns
Source: Front Immunol. 2018 Feb 26;9:277. doi: 10.3389/fimmu.2018.00277 (PMC5834439; doi:10.3389/fimmu.2018.00277)
Supplement: Table S9 — Results from joint linear models for replicated SNPs on chromosome 1. [file Table_9.PDF]

Results of Joint Models for Replicated SNPs on Chromosome 1 (*RUNX3*)

GWAS Code	SNP1	SNP2	SNP3	n	Adjusted R²	Standard Error of beta(SNP1)	beta(SNP1)	*p* (SNP1)	Standard Error of beta(SNP2)	beta(SNP2)	*p* (SNP2)	Standard Error of beta(SNP3)	beta(SNP2)2	*p* (SNP3)
LC_IGP133	rs10903120	NA	NA	1622	0.018225484	0.117564417	-0.655932295	2.82E-08	NA	NA	NA	NA	NA	NA
LC_IGP133	rs10903120	rs16830188	NA	1621	0.029232513	0.223104694	0.180844531	0.417725047	0.250526932	-1.103201178	1.13E-05	NA	NA	NA
LC_IGP133	rs10903120	rs112702191	NA	1621	0.017790915	0.664746674	-0.308270834	0.642894993	0.657678693	-0.34947626	0.595229382	NA	NA	NA
LC_IGP133	rs16830188	NA	NA	1622	0.029437773	0.131258495	-0.930238765	2.04E-12	NA	NA	NA	NA	NA	NA
LC_IGP133	rs16830188	rs10903120	NA	1621	0.029232513	0.250526932	-1.103201178	1.13E-05	0.223104694	0.180844531	0.417725047	NA	NA	NA
LC_IGP133	rs16830188	rs112702191	NA	1621	0.029089393	0.243270123	-1.062651139	1.33E-05	0.214338734	0.138575733	0.51802978	NA	NA	NA
LC_IGP133	rs112702191	NA	NA	1622	0.018266241	0.116311988	-0.649659545	2.73E-08	NA	NA	NA	NA	NA	NA
LC_IGP133	rs112702191	rs10903120	NA	1621	0.017790915	0.657678693	-0.34947626	0.595229382	0.664746674	-0.308270834	0.642894993	NA	NA	NA
LC_IGP133	rs112702191	rs16830188	NA	1621	0.029089393	0.214338734	0.138575733	0.51802978	0.243270123	-1.062651139	1.33E-05	NA	NA	NA
LC_IGP133	rs10903120	rs16830188	rs112702191	1620	0.028712756	0.681321006	0.415228098	0.542314574	0.250782214	-1.099664154	1.23E-05	0.65450312	-0.238303886	0.71583165
LC_IGP135	rs10903120	NA	NA	1622	0.024319777	0.116631854	0.75093934	1.58E-10	NA	NA	NA	NA	NA	NA
LC_IGP135	rs10903120	rs16830188	NA	1621	0.031859333	0.221724528	0.05369064	0.80869441	0.248977128	0.919248195	0.000229779	NA	NA	NA
LC_IGP135	rs10903120	rs112702191	NA	1621	0.023774337	0.65951203	0.552188896	0.402564871	0.652499706	0.199787925	0.759500426	NA	NA	NA
LC_IGP135	rs16830188	NA	NA	1622	0.032421215	0.130422437	0.970598725	1.60E-13	NA	NA	NA	NA	NA	NA
LC_IGP135	rs16830188	rs10903120	NA	1621	0.031859333	0.248977128	0.919248195	0.000229779	0.221724528	0.05369064	0.80869441	NA	NA	NA
LC_IGP135	rs16830188	rs112702191	NA	1621	0.031872043	0.241745804	0.913063415	0.000164491	0.212995697	0.060213389	0.777444688	NA	NA	NA
LC_IGP135	rs112702191	NA	NA	1622	0.023954283	0.115413366	0.737490032	2.16E-10	NA	NA	NA	NA	NA	NA
LC_IGP135	rs112702191	rs10903120	NA	1621	0.023774337	0.652499706	0.199787925	0.759500426	0.65951203	0.552188896	0.402564871	NA	NA	NA
LC_IGP135	rs112702191	rs16830188	NA	1621	0.031872043	0.212995697	0.060213389	0.777444688	0.241745804	0.913063415	0.000164491	NA	NA	NA
LC_IGP135	rs10903120	rs16830188	rs112702191	1620	0.031277901	0.67712828	-0.051564437	0.939307851	0.249238945	0.917659817	0.000239144	0.650475426	0.107015582	0.869343097
LC_IGP90	rs10903120	NA	NA	1638	0.025523354	0.116572526	0.772625746	4.61E-11	NA	NA	NA	NA	NA	NA
LC_IGP90	rs10903120	rs16830188	NA	1637	0.034125617	0.221501542	0.02774495	0.900334288	0.248756193	0.982141079	8.21E-05	NA	NA	NA
LC_IGP90	rs10903120	rs112702191	NA	1637	0.025075162	0.659327698	0.45012446	0.494891646	0.652305407	0.324175901	0.619277284	NA	NA	NA
LC_IGP90	rs16830188	NA	NA	1638	0.034706032	0.130297905	1.008680482	1.71E-14	NA	NA	NA	NA	NA	NA
LC_IGP90	rs16830188	rs10903120	NA	1637	0.034125617	0.248756193	0.982141079	8.21E-05	0.221501542	0.02774495	0.900334288	NA	NA	NA
LC_IGP90	rs16830188	rs112702191	NA	1637	0.034148075	0.241534204	0.961534572	7.16E-05	0.212780169	0.049332144	0.816686924	NA	NA	NA
LC_IGP90	rs112702191	NA	NA	1638	0.025392946	0.115338665	0.762487186	5.16E-11	NA	NA	NA	NA	NA	NA
LC_IGP90	rs112702191	rs10903120	NA	1637	0.025075162	0.652305407	0.324175901	0.619277284	0.659327698	0.45012446	0.494891646	NA	NA	NA
LC_IGP90	rs112702191	rs16830188	NA	1637	0.034148075	0.212780169	0.049332144	0.816686924	0.241534204	0.961534572	7.16E-05	NA	NA	NA
LC_IGP90	rs10903120	rs16830188	rs112702191	1636	0.033606137	0.676564725	-0.19373679	0.774643514	0.2490101	0.97879798	8.82E-05	0.64993331	0.22518046	0.729036316
LC_IGP_R62	rs10903120	NA	NA	1637	0.008479351	0.118152573	-0.457724285	0.000111278	NA	NA	NA	NA	NA	NA
LC_IGP_R62	rs10903120	rs16830188	NA	1636	0.022788116	0.223866093	0.495078817	0.027139352	0.251408963	-1.25628211	6.45E-07	NA	NA	NA
LC_IGP_R62	rs10903120	rs112702191	NA	1636	0.008721179	0.668020186	0.320050026	0.631929863	0.660902625	-0.781810135	0.237003615	NA	NA	NA
LC_IGP_R62	rs16830188	NA	NA	1637	0.020465545	0.131884755	-0.782721898	3.58E-09	NA	NA	NA	NA	NA	NA
LC_IGP_R62	rs16830188	rs10903120	NA	1636	0.022788116	0.251408963	-1.25628211	6.45E-07	0.223866093	0.495078817	0.027139352	NA	NA	NA
LC_IGP_R62	rs16830188	rs112702191	NA	1636	0.021722397	0.244245543	-1.144958846	2.99E-06	0.215170188	0.379038791	0.078327603	NA	NA	NA
LC_IGP_R62	rs112702191	NA	NA	1637	0.009187728	0.116851928	-0.470160983	5.99E-05	NA	NA	NA	NA	NA	NA
LC_IGP_R62	rs112702191	rs10903120	NA	1636	0.008721179	0.660902625	-0.781810135	0.237003615	0.668020186	0.320050026	0.631929863	NA	NA	NA
LC_IGP_R62	rs112702191	rs16830188	NA	1636	0.021722397	0.215170188	0.379038791	0.078327603	0.244245543	-1.144958846	2.99E-06	NA	NA	NA
LC_IGP_R62	rs10903120	rs16830188	rs112702191	1635	0.022786408	0.683602175	1.140061462	0.095561527	0.251598099	-1.246547927	8.00E-07	0.656690194	-0.655750434	0.31815131
LC_IGP144	rs10903120	NA	NA	1622	0.008741226	0.118154656	0.462347404	9.49E-05	NA	NA	NA	NA	NA	NA
LC_IGP144	rs10903120	rs16830188	NA	1621	0.022303161	0.223944553	-0.462283234	0.039150396	0.25147002	1.219027078	1.37E-06	NA	NA	NA
LC_IGP144	rs10903120	rs112702191	NA	1621	0.009321483	0.66774074	-0.455404814	0.495331055	0.660640924	0.922542901	0.162774358	NA	NA	NA
LC_IGP144	rs16830188	NA	NA	1622	0.02033738	0.131898938	0.776892483	4.68E-09	NA	NA	NA	NA	NA	NA
LC_IGP144	rs16830188	rs10903120	NA	1621	0.022303161	0.25147002	1.219027078	1.37E-06	0.223944553	-0.462283234	0.039150396	NA	NA	NA
LC_IGP144	rs16830188	rs112702191	NA	1621	0.021184288	0.244307568	1.09575584	7.80E-06	0.215252798	-0.333705396	0.121265864	NA	NA	NA
LC_IGP144	rs112702191	NA	NA	1622	0.009648166	0.116844876	0.479085725	4.33E-05	NA	NA	NA	NA	NA	NA
LC_IGP144	rs112702191	rs10903120	NA	1621	0.009321483	0.660640924	0.922542901	0.162774358	0.66774074	-0.455404814	0.495331055	NA	NA	NA
LC_IGP144	rs112702191	rs16830188	NA	1621	0.021184288	0.215252798	-0.333705396	0.121265864	0.244307568	1.09575584	7.80E-06	NA	NA	NA
LC_IGP144	rs10903120	rs16830188	rs112702191	1620	0.022596169	0.68360032	-1.249618708	0.067733967	0.251621189	1.207145596	1.75E-06	0.656692716	0.800504512	0.223023918
LC_IGP145	rs10903120	NA	NA	1622	0.006729215	0.118431929	-0.410183416	0.000547153	NA	NA	NA	NA	NA	NA
LC_IGP145	rs10903120	rs16830188	NA	1621	0.020709514	0.224425304	0.529282993	0.018472415	0.252009861	-1.238586462	9.78E-07	NA	NA	NA
LC_IGP145	rs10903120	rs112702191	NA	1621	0.007282845	0.6693171	0.498950085	0.456100893	0.662200523	-0.913879194	0.167757198	NA	NA	NA
LC_IGP145	rs16830188	NA	NA	1622	0.017955174	0.132234977	-0.732372297	3.55E-08	NA	NA	NA	NA	NA	NA
LC_IGP145	rs16830188	rs10903120	NA	1621	0.020709514	0.252009861	-1.238586462	9.78E-07	0.224425304	0.529282993	0.018472415	NA	NA	NA
LC_IGP145	rs16830188	rs112702191	NA	1621	0.019384097	0.244857593	-1.110435446	6.18E-06	0.215737411	0.395660742	0.06683837	NA	NA	NA
LC_IGP145	rs112702191	NA	NA	1622	0.007554764	0.117123987	-0.428019167	0.000265981	NA	NA	NA	NA	NA	NA
LC_IGP145	rs112702191	rs10903120	NA	1621	0.007282845	0.662200523	-0.913879194	0.167757198	0.6693171	0.498950085	0.456100893	NA	NA	NA
LC_IGP145	rs112702191	rs16830188	NA	1621	0.019384097	0.215737411	0.395660742	0.06683837	0.244857593	-1.110435446	6.18E-06	NA	NA	NA
LC_IGP145	rs10903120	rs16830188	rs112702191	1620	0.020975508	0.68507745	1.306136699	0.056755225	0.252164894	-1.226863158	1.25E-06	0.658111704	-0.789847426	0.230247914
LC_IGP175	rs10903120	NA	NA	1639	0.012849152	0.116945246	-0.5528295	2.47E-06	NA	NA	NA	NA	NA	NA
LC_IGP175	rs10903120	rs16830188	NA	1638	0.022694782	0.222083426	0.23874571	0.282520903	0.249412377	-1.043716058	3.01E-05	NA	NA	NA
LC_IGP175	rs10903120	rs112702191	NA	1638	0.012420978	0.661441457	-0.903047612	0.172354993	0.654395344	0.352035699	0.590680722	NA	NA	NA
LC_IGP175	rs16830188	NA	NA	1639	0.022601951	0.130685799	-0.815340774	5.60E-10	NA	NA	NA	NA	NA	NA
LC_IGP175	rs16830188	rs10903120	NA	1638	0.022694782	0.249412377	-1.043716058	3.01E-05	0.222083426	0.23874571	0.282520903	NA	NA	NA
LC_IGP175	rs16830188	rs112702191	NA	1638	0.022931715	0.242144665	-1.069410127	1.07E-05	0.213315219	0.265846539	0.212846623	NA	NA	NA
LC_IGP175	rs112702191	NA	NA	1639	0.011900395	0.115755055	-0.527310968	5.62E-06	NA	NA	NA	NA	NA	NA
LC_IGP175	rs112702191	rs10903120	NA	1638	0.012420978	0.654395344	0.352035699	0.590680722	0.661441457	-0.903047612	0.172354993	NA	NA	NA
LC_IGP175	rs112702191	rs16830188	NA	1638	0.022931715	0.213315219	0.265846539	0.212846623	0.242144665	-1.069410127	1.07E-05	NA	NA	NA
LC_IGP175	rs10903120	rs16830188	rs112702191	1637	0.022393192	0.678272599	-0.212002433	0.754653047	0.249638311	-1.050519026	2.71E-05	0.651572304	0.458273753	0.481946881
LC_IGP199	rs10903120	NA	NA	1635	0.013107983	0.117033892	-0.557964408	2.03E-06	NA	NA	NA	NA	NA	NA
LC_IGP199	rs10903120	rs16830188	NA	1634	0.022804771	0.222393584	0.228350285	0.304673922	0.249724389	-1.036408501	3.49E-05	NA	NA	NA
LC_IGP199	rs10903120	rs112702191	NA	1634	0.012626626	0.661841617	-0.851399299	0.198482443	0.654788207	0.294958127	0.652436094	NA	NA	NA
LC_IGP199	rs16830188	NA	NA	1635	0.022772325	0.130771593	-0.817962353	5.07E-10	NA	NA	NA	NA	NA	NA
LC_IGP199	rs16830188	rs10903120	NA	1634	0.022804771	0.249724389	-1.036408501	3.49E-05	0.222393584	0.228350285	0.304673922	NA	NA	NA
LC_IGP199	rs16830188	rs112702191	NA	1634	0.022994719	0.242436175	-1.057105332	1.38E-05	0.213602088	0.250212955	0.241609813	NA	NA	NA
LC_IGP199	rs112702191	NA	NA	1635	0.012231164	0.115838058	-0.534087104	4.33E-06	NA	NA	NA	NA	NA	NA
LC_IGP199	rs112702191	rs10903120	NA	1634	0.012626626	0.654788207	0.294958127	0.652436094	0.661841617	-0.851399299	0.198482443	NA	NA	NA
LC_IGP199	rs112702191	rs16830188	NA	1634	0.022994719	0.213602088	0.250212955	0.241609813	0.242436175	-1.057105332	1.38E-05	NA	NA	NA

LC_IGP199	rs10903120	rs16830188	rs112702191	1633	0.022431939	0.678787795	-0.165316909	0.807612068	0.249959487	-1.042349924	3.20E-05	0.65201784	0.400241851	0.539399243
LC_IGP88	rs10903120	NA	NA	1638	0.017053076	0.117567405	-0.637849421	6.65E-08	NA	NA	NA	NA	NA	NA
LC_IGP88	rs10903120	rs16830188	NA	1637	0.025827741	0.223380737	0.117330919	0.599479959	0.250866613	-0.995721246	7.53E-05	NA	NA	NA
LC_IGP88	rs10903120	rs112702191	NA	1637	0.016477918	0.665001688	-0.503539323	0.449038981	0.657914973	-0.13500666	0.837438404	NA	NA	NA
LC_IGP88	rs16830188	NA	NA	1638	0.026258393	0.131413781	-0.883488479	2.45E-11	NA	NA	NA	NA	NA	NA
LC_IGP88	rs16830188	rs10903120	NA	1637	0.025827741	0.250866613	-0.995721246	7.53E-05	0.223380737	0.117330919	0.599479959	NA	NA	NA
LC_IGP88	rs16830188	rs112702191	NA	1637	0.025800162	0.243589175	-0.981743018	5.83E-05	0.2145892	0.102810221	0.631929708	NA	NA	NA
LC_IGP88	rs112702191	NA	NA	1638	0.016734095	0.116333397	-0.625328129	8.75E-08	NA	NA	NA	NA	NA	NA
LC_IGP88	rs112702191	rs10903120	NA	1637	0.016477918	0.657914973	-0.13500666	0.837438404	0.665001688	-0.503539323	0.449038981	NA	NA	NA
LC_IGP88	rs112702191	rs16830188	NA	1637	0.025800162	0.2145892	0.102810221	0.631929708	0.243589175	-0.981743018	5.83E-05	NA	NA	NA
LC_IGP88	rs10903120	rs16830188	rs112702191	1636	0.025233919	0.682335666	0.151141166	0.824726427	0.251131587	-0.995211031	7.72E-05	0.655471884	-0.034374561	0.958182541
LC_IGP89	rs10903120	NA	NA	1638	0.012834826	0.117387802	-0.554460585	2.52E-06	NA	NA	NA	NA	NA	NA
LC_IGP89	rs10903120	rs16830188	NA	1637	0.02368011	0.222807635	0.276995313	0.21397074	0.250222994	-1.096292183	1.25E-05	NA	NA	NA
LC_IGP89	rs10903120	rs112702191	NA	1637	0.0122412	0.663985779	-0.472861479	0.476469641	0.656913877	-0.082022817	0.900649053	NA	NA	NA
LC_IGP89	rs16830188	NA	NA	1638	0.023354935	0.131127441	-0.831332608	2.97E-10	NA	NA	NA	NA	NA	NA
LC_IGP89	rs16830188	rs10903120	NA	1637	0.02368011	0.250222994	-1.096292183	1.25E-05	0.222807635	0.276995313	0.21397074	NA	NA	NA
LC_IGP89	rs16830188	rs112702191	NA	1637	0.023601669	0.242971005	-1.074571713	1.04E-05	0.214045923	0.254518505	0.234579207	NA	NA	NA
LC_IGP89	rs112702191	NA	NA	1638	0.012538392	0.116154978	-0.542474404	3.25E-06	NA	NA	NA	NA	NA	NA
LC_IGP89	rs112702191	rs10903120	NA	1637	0.0122412	0.656913877	-0.082022817	0.900649053	0.663985779	-0.472861479	0.476469641	NA	NA	NA
LC_IGP89	rs112702191	rs16830188	NA	1637	0.023601669	0.214045923	0.254518505	0.234579207	0.242971005	-1.074571713	1.04E-05	NA	NA	NA
LC_IGP89	rs10903120	rs16830188	rs112702191	1636	0.023084504	0.680578678	0.248570643	0.714983766	0.250487438	-1.096721231	1.27E-05	0.653789264	0.028899359	0.964748092
