# Supplementary material for: Genome-Wide Association Study on Immunoglobulin G Glycosylation Patterns
Source: Front Immunol. 2018 Feb 26;9:277. doi: 10.3389/fimmu.2018.00277 (PMC5834439; doi:10.3389/fimmu.2018.00277)
Supplement: Table S10 — Comparison of ultra-performance liquid chromatography (UPLC)-measured and LC/MS-measured immunoglobulin G (IgG) glycan traits [adapted from Huffman et al. (24)]. [file Table_10.PDF]

Comparison of UPLC-measured and LC/MS-measured IgG Glycan Traits (adapted from Huffmann et al. [23])

Glycan Class	UPLC Glycan Trait	Glycan scheme[a]	UPLC Code		LC-ESI-MS					
			Total IgG		IgG1		IgG2 & IgG3		IgG4	
			Trait	UPLC Code	Trait	GWAS Code	Trait	GWAS Code	Trait	GWAS Code
Initial Glycans (total)	FA2	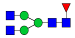	GP4	IGP3	IgG1_G0F	LC_IGP1	IgG2_G0F	LC_IGP87	IgG4_G0F	LC_IGP173
	FA2B	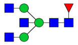	GP6	IGP5	IgG1_G0FN	LC_IGP4	IgG2_G0FN	LC_IGP90	IgG4_G0FN	LC_IGP176
	FA2G1	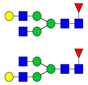	GP8<br>GP9<br>GP8<br>GP9<br>GP8<br>GP9	IGP7<br>IGP8<br>IGP7<br>IGP8<br>IGP7<br>IGP8	IgG1_G1F	LC_IGP2	IgG2_G1F	LC_IGP88	IgG4_G1F	LC_IGP174
	FA2BG1	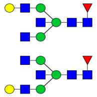	GP10<br>GP11<br>GP10<br>GP11<br>GP10<br>GP11	IGP9<br>IGP10<br>IGP9<br>IGP10<br>IGP9<br>IGP10	IgG1_G1FN	LC_IGP5	IgG2_G1FN	LC_IGP91	IgG4_G1FN	LC_IGP177
	FA2G2	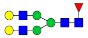	GP14	IGP13	IgG1_G2F	LC_IGP3	IgG2_G2F	LC_IGP89	IgG4_G2F	LC_IGP175
	FA2BG2	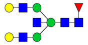	GP15	IGP14	IgG1_G2FN	LC_IGP6	IgG2_G2FN	LC_IGP92	IgG4_G2FN	LC_IGP178
	FA2G1S1	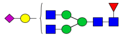	GP16	IGP15	IgG1_G1FS1	LC_IGP7	IgG2_G1FS1	LC_IGP93	IgG4_G1FS1	LC_IGP179
	FA2G2S1	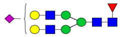	GP18	IGP17	IgG1_G2FS1	LC_IGP8	IgG2_G2FS1	LC_IGP94	IgG4_G2FS1	LC_IGP180
Summarizing Glycans (total)	FGS/(FG+FGS)		FGS/(FG+FGS)	IGP24	IgG1 FGS1/(FG+FGS1)	LC_IGP34	IgG2 FGS1/(FG+FGS1)	LC_IGP120	IgG4 FGS1/(FG+FGS1)	LC_IGP187
	FGS/(F+FG+FGS)		FGS/(F+FG+FGS)	IGP26	IgG1 FGS1/(F+FG+FGS1)	LC_IGP35	IgG2 FGS1/(F+FG+FGS1)	LC_IGP121	IgG4 FGS1/(F+FG+FGS1)	LC_IGP188
	FG1S1/(FG1+FG1S1)		FG1S1/(FG1+FG1S1)	IGP28	IgG1 FG1S1/(FG1+FG1S1)	LC_IGP36	IgG2 FG1S1/(FG1+FG1S1)	LC_IGP122	IgG4 FG1S1/(FG1+FG1S1)	LC_IGP189
	FG2S1/(FG2+FG2S1+FG2S2)		FG2S1/(FG2+FG2S1+FG2S2)	IGP30	IgG1 FG2S1/(FG2+FG2S1)	LC_IGP37	IgG2 FG2S1/(FG2+FG2S1)	LC_IGP123	IgG4 FG2S1/(FG2+FG2S1)	LC_IGP190
Summarizing Glycans (neutral)	G0n		G0^{n}	IGP55	IgG1 G0n	LC_IGP52	IgG2 G0n	LC_IGP138		
	G1n		G1^{n}	IGP56	IgG1 G1n	LC_IGP53	IgG2 G1n	LC_IGP139		
	G2n		G2^{n}	IGP57	IgG1 G2n	LC_IGP54	IgG2 G2n	LC_IGP140		

a Glycan schemes are given in terms of blue square (N-acetylglucosamine), red triangle (fucose), green circle (mannose), yellow circle (galactose), and purple diamond (N-acetylneuraminic acid).
