# Supplementary material for: Genome-Wide Association Study on Immunoglobulin G Glycosylation Patterns
Source: Front Immunol. 2018 Feb 26;9:277. doi: 10.3389/fimmu.2018.00277 (PMC5834439; doi:10.3389/fimmu.2018.00277)
Supplement: Table S11 — Replicated association and comparison to the study by Lauc et al. (21). [file Table_11.PDF]

Replicated Association and Comparison to the Study by Lauc et al [21]

Chromosome	LD-Block per Chromosome	Minimal Position	Maximal Position	Gene Locus	SNP with lowest *p*-value[1]	Lowest *p*-value (from either discovery or replication cohort)	Effect Size	Trait with lowest *p*-value	Replicated Trait with Similar Trait for UPLC data	UPLC trait	*p*-value in Lauc et al [21][2]	Associated SNP in Lauc et al [21] (position, LD to lead-SNP in current study)[3]
1	1	25296560	25298841	*RUNX3*	rs16830188	1.71E-14	1.00868	LC_IGP90				
					rs16830188	5.60E-10	-0.815341		LC_IGP175	IGP13	0.06158214	rs10751776 (chr1:25296743; LD = NA)
					rs16830188	2.45E-11	-0.883488		LC_IGP88	IGP8	0.4136774	rs10751776 (chr1:25296743; LD = NA)
					rs16830188	2.45E-11	-0.883488		LC_IGP88	IGP7	0.004747253	rs10751776 (chr1:25296743; LD = NA)
					rs16830188					IGP69	7.25E-06	rs10751776 (chr1:25296743; LD = NA)
3	1	186705790	186708013	*ST6GAL1***	rs13082825	1.60E-23	-0.4665	LC_IGP122		IGP28	4.37E-11	rs11923417 (chr3:186705790; LD = 0.58)
					rs13082825	5.71E-14	-0.3455		LC_IGP189	IGP28	4.37E-11	rs11923417 (chr3:186705790; LD = 0.58)
					rs11923417	3.92E-17	-0.4199		LC_IGP36	IGP28	4.37E-11	rs11923417 (chr3:186705790; LD = 0.58)
					rs13082825	3.62E-14	-0.3456		LC_IGP120	IGP24	1.72E-08	rs11923417 (chr3:186705790; LD = 0.58)
					rs13082825	6.65E-14	-0.3417		LC_IGP190	IGP30	0.000177746	rs11923417 (chr3:186705790; LD = 0.58)
					rs13082825	5.69E-19	-0.4154		LC_IGP123	IGP30	0.000177746	rs11923417 (chr3:186705790; LD = 0.58)
					rs13082825	1.32E-16	-0.307515		LC_IGP37	IGP30	0.000177746	rs11923417 (chr3:186705790; LD = 0.58)
					rs35832957	4.38E-10	-0.3736		LC_IGP7	IGP15	1.04E-11	rs11923417 (chr3:186705790; LD = 0.58)
										IGP29	5.88E-23	rs11923417 (chr3:186705790; LD = 0.58)
3	2	186708571	186711453	*ST6GAL1***	rs4012171	2.79E-19	-0.4471	LC_IGP122		IGP28*	6.22E-14	rs4012171
					rs4012171	1.76E-15	-0.397		LC_IGP36	IGP28*	6.22E-14	rs4012171
					rs4012171	1.28E-15	-0.3988		LC_IGP123	IGP30	0.005296511	rs4012171
					rs4012171	4.39E-10	-0.3029		LC_IGP190	IGP30	0.005296511	rs4012171
					rs4012171	9.62E-15	-0.322089		LC_IGP37	IGP30	0.005296511	rs4012171
					rs4012171	2.98E-12	-0.3399		LC_IGP120	IGP24*	4.59E-11	rs4012171
					rs4012171	1.81E-13	-0.3552		LC_IGP93	IGP15*	2.04E-13	rs4012171
										IGP29*	3.23E-29	rs4012171
3	3	186712711	186744563	*ST6GAL1***	rs11710456*	6.30E-73	-0.7558	LC_IGP_R74				
					rs11710456*	2.07E-42	-0.5699		LC_IGP7	IGP15*	2.05E-45	rs11710456
					rs11710456*	7.03E-47	-0.5876		LC_IGP93	IGP15*	2.05E-45	rs11710456
					rs11710456*	1.65E-32	-0.4848		LC_IGP179	IGP15*	2.05E-45	rs11710456
					rs11710456*	7.18E-21	-0.368408		LC_IGP34	IGP24*	4.30E-31	rs11710456
					rs11710456*	1.24E-46	-0.5886		LC_IGP120	IGP24*	4.30E-31	rs11710456
					rs11710456*	2.24E-24	-0.4054		LC_IGP187	IGP24*	4.30E-31	rs11710456
					rs11710456*	8.72E-17	-0.3321		LC_IGP121	IGP26*	2.47E-13	rs11710456
					rs11710456*	1.38E-08	-0.2236		LC_IGP35	IGP26*	2.47E-13	rs11710456
					rs11710456*	6.51E-73	-0.7558		LC_IGP122	IGP28*	8.53E-37	rs11710456
					rs11710456*	1.63E-50	-0.6132		LC_IGP189	IGP28*	8.53E-37	rs11710456
					rs11710456*	9.68E-62	-0.6987		LC_IGP36	IGP28*	8.53E-37	rs11710456
					rs11710456*	1.21E-44	-0.542453		LC_IGP37	IGP30	2.84E-06	rs11710456
					rs11710456*	4.76E-45	-0.576		LC_IGP190	IGP30	2.84E-06	rs11710456
					rs11710456*	4.70E-59	-0.678		LC_IGP123	IGP30	2.84E-06	rs11710456
										IGP29*	6.12E-75	rs11710456
3	4	186754722	186782999	*ST6GAL1***	rs57679165	4.60E-23	-0.4698	LC_IGP_R74				
					rs57679165	5.41E-15	-0.3637		LC_IGP93	IGP15*	2.42E-15	rs16861533 (chr3:186754722; LD = 0.59)
					rs57679165	9.23E-16	-0.3739		LC_IGP120	IGP24*	6.69E-12	rs16861533 (chr3:186754722; LD = 0.59)
					rs57679165	2.91E-19	-0.4282		LC_IGP36	IGP28*	2.99E-11	rs16861533 (chr3:186754722; LD = 0.59)
					rs57679165	4.72E-23	-0.4696		LC_IGP122	IGP28*	2.99E-11	rs16861533 (chr3:186754722; LD = 0.59)
					rs57679165	8.07E-16	-0.3742		LC_IGP189	IGP28*	2.99E-11	rs16861533 (chr3:186754722; LD = 0.59)
					rs57679165	1.00E-19	-0.4321		LC_IGP123	IGP30	2.96E-05	rs16861533 (chr3:186754722; LD = 0.59)
					rs57679165	1.46E-14	-0.344232		LC_IGP37	IGP30	2.96E-05	rs16861533 (chr3:186754722; LD = 0.59)
					rs57679165	4.09E-15	-0.3656		LC_IGP190	IGP30	2.96E-05	rs16861533 (chr3:186754722; LD = 0.59)
										IGP29*	1.10E-25	rs16861533 (chr3:186754722; LD = 0.59)
7	1	50336551	50355207	*IKZF1***	rs7782210*	2.38E-13	-0.2853	LC_IGP56			1.87E-13	rs16861533 (chr3:186754722; LD = 0.59)
										IGP63	3.02E-13	rs6421315 (chr7:50355207; LD = 0.97)
9	1	33113322	33113322	*B4GALT1***	rs7019909	2.90E-08	-0.30427	LC_IGP_R89				
										IGP39	8.29E-06	rs7019909
9	2	33119241	33180813	*B4GALT1***	rs12342831*	2.96E-20	-0.3813	LC_IGP_R89				
					rs12342831*	3.24E-08	-0.2336		LC_IGP175	IGP13*	5.19E-08	rs12342831
					rs12337458	9.18E-11	1.03476		LC_IGP3	IGP13*	5.19E-08	rs12342831
					rs12337458	5.95E-10	0.9829		LC_IGP89	IGP13*	5.19E-08	rs12342831
					rs3780481	2.44E-11	-0.901079		LC_IGP4	IGP5	0.1102551	rs12342831
					rs12342831*	4.01E-16	-0.3346		LC_IGP180	IGP17*	2.70E-11	rs12342831
					rs10971420	2.44E-12	-0.2689		LC_IGP187	IGP24*	2.70E-10	rs12342831

					rs113197944	1.25E-10	0.8764		LC_IGP88	IGP7	0.01011209	rs12342831
					rs113197944	1.25E-10	0.8764		LC_IGP88	IGP7	0.01011209	rs12342831
14	1	65734600	66262963	*FUT8***	rs11158592*	1.32E-24	-0.348762	LC_IGP11				
					rs11158592*	6.04E-15	-0.2972		LC_IGP138	IGP55	0.01908447	rs11158592
					rs11158592*	6.63E-24	-0.346241		LC_IGP52	IGP55	0.01908447	rs11158592
					rs11158592*	7.53E-18	-0.297683		LC_IGP53	IGP56	0.005632521	rs11158592
					rs11158592*	6.54E-13	-0.2699		LC_IGP54	IGP57	0.2005604	rs11158592
					rs11158592*					IGP42	3.93E-16	rs11158592
14	2	66275755	66275755	*FUT8***	rs4899183	1.17E-08	0.210149	LC_IGP14				
										IGP42	1.30E-12	rs10149325 (chr14:66277367; LD = 0.74)
22	1	24100654	24179922	*SMARCB1-DERL3***	rs2186369*	2.50E-13	-0.3778	LC_IGP108				
					rs2186369*	2.37E-09	-0.281643		LC_IGP5	IGP9*	3.00E-13	rs2186369
					rs34556611	2.64E-08	-0.2816		LC_IGP91	IGP9*	3.00E-13	rs2186369
					rs2186369*	2.37E-09	-0.281643		LC_IGP5	IGP10*	9.32E-10	rs2186369
					rs34556611	2.64E-08	-0.2816		LC_IGP91	IGP10*	9.32E-10	rs2186369
					rs2186369*					IGP72*	8.63E-17	rs2186369
22	2	24182500	24189032	*SMARCB1-DERL3***	rs6519476	1.19E-09	-0.259124	LC_IGP155				
										IGP49	3.56E-09	rs6519476
22	3	39737929	39737929	*MGAT3***	rs137680	7.82E-11	-0.271863	LC_IGP_R81				
22	4	39738425	39860868	*MGAT3***	rs73167342*	2.53E-38	-0.5559	LC_IGP_R81				
					rs73167342*	2.73E-14	-0.284738		LC_IGP4	IGP5*	1.10E-10	rs909674 (chr22: 39859169; LD = 0.83)
					rs73167342*	2.00E-18	-0.3733		LC_IGP176	IGP5*	1.10E-10	rs909674 (chr22: 39859169; LD = 0.83)
					rs73167342*	1.49E-16	-0.308365		LC_IGP5	IGP9*	2.80E-09	rs909674 (chr22: 39859169; LD = 0.83)
					rs73167342*	1.35E-29	-0.417789		LC_IGP177	IGP9*	2.80E-09	rs909674 (chr22: 39859169; LD = 0.83)
					rs73167342*	1.49E-16	-0.308365		LC_IGP5	IGP10	1.21E-05	rs909674 (chr22: 39859169; LD = 0.83)
					rs73167342*	1.35E-29	-0.417789		LC_IGP177	IGP10	1.21E-05	rs909674 (chr22: 39859169; LD = 0.83)
					rs73167342*	9.33E-11	-0.274		LC_IGP178	IGP14	0.009998935	rs909674 (chr22: 39859169; LD = 0.83)
					rs73167342*	2.44E-11	0.250743		LC_IGP173	IGP30	1.90E-06	rs909674 (chr22: 39859169; LD = 0.83)
										IGP40	9.66E-25	rs909674 (chr22: 39859169; LD = 0.83)
22	5	39873937	39873937	*MGAT3***	rs12484278	3.66E-12	0.320619	LC_IGP_R81			1.99E-07	
					rs12484278	2.01E-09	0.275964		LC_IGP177	IGP9	0.03625394	rs12484278
					rs12484278	2.01E-09	0.275964		LC_IGP177	IGP10	0.1637232	rs12484278
										IGP40	1.99E-07	rs12484278
22	6	39889080	39893932	*MGAT3***	rs34692520	1.39E-12	0.308965	LC_IGP_R81				
										IGP40	0.01109271	rs6001609 (chr22:39892859; LD = NA)

** SNPs in this gene locus were associated to UPLC-measured IgG glycan traits in the discovery meta-analysis in Lauc et al [21]

* strongly suggestive association of the SNP with lowest *p*-value or a SNP in the same LD-block with a comparable IgG glycan trait has been shown in Lauc et al [21]

[1] Lead-SNP for the LD-block if not otherwise indicated; or, if no association to the lead-SNP was replicated for the corresponding trait, SNP with lowest *p*-value from the LD-block

[2] *p*-values as published in Lauc et al [21] if available; if not available: lookup in the appropriate cohorts; last row per LD-block gives the SNP-glycan combination with lowest *p*-value in Lauc et al [21]

[3] Lead-SNP as in current study, if available; else, SNP with lowest *p*-value of the appropriate LD-block. If no SNP in LD (>0.5) to the current lead-SNP was available in the data from Lauc et al [21], the SNP with lowest *p*-value in the range of the LD-block is taken. The last option is indicated by an LD to the lead-SNP with N

A.
