# Supplementary material for: Genome-Wide Association Study on Immunoglobulin G Glycosylation Patterns
Source: Front Immunol. 2018 Feb 26;9:277. doi: 10.3389/fimmu.2018.00277 (PMC5834439; doi:10.3389/fimmu.2018.00277)
Supplement: Table S12 — Summary of replicated association and comparison to study by Lauc et al. (21). [file Table_12.PDF]

Confirmation of Loci Reported in Lauc et al [21]

Chromosome	SNP with Lowest *p* -value in Lauc et al [21]	Lowest *p* -value in Lauc et al [21]	Interval Size (from Lauc et al [21])	Genes in Interval (from Lauc et al [21])	Associated Traits in Lauc et al [21] [1]	Comparable Trait in Current Study (LS/MS)	SNP with Lowest *p* -value in Current Study[2]	Lowest *p* -value in Current Study
3	rs11710456	6.12E-75	14.2	*ST6GAL1*	IGP29			
		2.04841E-45			IGP15	LC_IGP7	rs11710456	2.07E-42
		2.04841E-45			IGP15	LC_IGP93	rs11710456	7.03E-47
		2.04841E-45			IGP15	LC_IGP179	rs11710456	1.65E-32
		4.30429E-31			IGP24	LC_IGP34	rs11710456	7.18E-21
		4.30429E-31			IGP24	LC_IGP120	rs11710456	1.24E-46
		4.30429E-31			IGP24	LC_IGP187	rs11710456	2.24E-24
		2.47142E-13			IGP26	LC_IGP121	rs11710456	8.72E-17
		2.47142E-13			IGP26	LC_IGP35	rs11710456	1.38E-08
		8.5338E-37			IGP28	LC_IGP122	rs11710456	6.51E-73
		8.5338E-37			IGP28	LC_IGP189	rs11710456	1.63E-50
		8.5338E-37			IGP28	LC_IGP36	rs11710456	9.68E-62
		2.84306E-06			IGP30	LC_IGP37	rs11710456	1.21E-44
		2.84306E-06			IGP30	LC_IGP190	rs11710456	4.76E-45
		2.84306E-06			IGP30	LC_IGP123	rs11710456	4.70E-59
						LC_IGP_R74	rs11710456	6.30E-73
7	rs6421315	3.02256E-13	21.4	*IKZF1*	IGP63			
						LC_IGP56	rs7782210 (LD = 0.97)	2.38E-13
9	rs12342831	5.18939E-08	60.1	*B4GALT1*	IGP13	LC_IGP175	rs12342831	3.24E-08
		5.18939E-08			IGP13	LC_IGP3	rs12337458[§]	9.18E-11
		5.18939E-08			IGP13	LC_IGP89	rs12337458[§]	5.95E-10
		0.1102551			IGP5	LC_IGP4	rs3780481[§]	2.44E-11
		2.70013E-11			IGP17	LC_IGP180	rs12342831	4.01E-16
		2.70249E-10			IGP24	LC_IGP187	rs10971420 (LD = 0.75)	2.44E-12
		0.01011209			IGP7	LC_IGP88	rs113197944[§]	1.25E-10
		0.01011209			IGP7	LC_IGP88	rs113197944[§]	1.25E-10
						LC_IGP_R89	rs12342831	2.96E-20
14	rs11847263	1.08E-22	17.1	*FUT8*	IGP59			
		0.5179092			IGP55	LC_IGP138	rs11158592 (LD = 0.23)	6.04E-15
		0.5179092			IGP55	LC_IGP52	rs11158592 (LD = 0.23)	6.63E-24
		0.04862762			IGP56	LC_IGP53	rs11158592 (LD = 0.23)	7.53E-18
		0.5960842			IGP57	LC_IGP54	rs11158592 (LD = 0.23)	6.54E-13
						LC_IGP11	rs11158592 (LD = 0.23)	1.32E-24

22	rs2186369	8.63E-17	49.4	*SMARCB1-DERL3*	IGP72			
		2.99837E-13			IGP9	LC_IGP5	rs2186369	2.37E-09
		2.99837E-13			IGP9	LC_IGP91	rs9620326 (LD = 0.97)	2.78E-08
		9.31701E-10			IGP10	LC_IGP5	rs2186369	2.37E-09
		9.31701E-10			IGP10	LC_IGP91	rs9620326 (LD = 0.97)	2.78E-08
						LC_IGP108	rs2186369	2.50E-13
22	rs909674	9.66E-25	27.9	*MGAT3*	IGP40			
		1.10324E-10			IGP5	LC_IGP4	rs73167342 (LD = 0.83)	2.73E-14
		1.10324E-10			IGP5	LC_IGP176	rs73167342 (LD = 0.83)	2.00E-18
		2.8042E-09			IGP9	LC_IGP5	rs73167342 (LD = 0.83)	1.49E-16
		2.8042E-09			IGP9	LC_IGP177	rs73167342 (LD = 0.83)	1.35E-29
		1.20528E-05			IGP10	LC_IGP5	rs73167342 (LD = 0.83)	1.49E-16
		1.20528E-05			IGP10	LC_IGP177	rs73167342 (LD = 0.83)	1.35E-29
		0.009998935			IGP14	LC_IGP178	rs73167342 (LD = 0.83)	9.33E-11
		1.90178E-06			IGP30	LC_IGP173	rs73167342 (LD = 0.83)	2.44E-11
						LC_IGP_R81	rs73167342 (LD = 0.83)	2.53E-38

1 Traits with lowest *p* -values and with comparable traits to durrent study based on LC/MS measurement

2 SNP in the same locus with lowest *p* -value to the given trait; same SNP or SNP in LD to the lead-SNP from Lauc et al [21]

§ no LD info was found for the two SNPs, however the SNPs belong to the same locus in the current study
