# Supplementary material for: Genome-Wide Association Study on Immunoglobulin G Glycosylation Patterns
Source: Front Immunol. 2018 Feb 26;9:277. doi: 10.3389/fimmu.2018.00277 (PMC5834439; doi:10.3389/fimmu.2018.00277)
Supplement: Supplementary file 20 [file Data_Sheet_1.doc]

# Additional Description of Replicated Loci

Four out of the seven replicated loci are located on genes known to encode glycosyltransferases: beta-galactoside alpha-2,6-sialyltransferase encoded by *ST6GAL1* on chromosome 3q27.3, beta-1,4 galactosyltransferase encoded by *B4GALT1* on chromosome 9p21.1, fucosyltransferase 8 encoded by *FUT8* on chromosome 14q23.3 and mannosyl (beta-1,4-)-glycoprotein beta-1,4-*N*-acetylglucosaminyltransferase (*N*-acetylglucosaminyltransferase III) encoded by *MGAT3* on chromosome 22q13.1.

Phenotypic traits associated with the ***ST6GAL1*** locus can all be linked to differing sialylation of the IgG glycans. Interestingly, we likewise detect the within-subclass ratios G1FS1/G1F, G2FS1/GSF and G2S1/G2 (only for IgG1) being linked with the same effect directions to SNPs within the *ST6GAL1* gene for each of the subclasses. The gene *ST6GAL1* on chromosome 3 encodes for a sialyltransferase. The enzyme adds sialic acid residues predominantly to *N*-glycans in the Golgi apparatus [1]. Higher levels of IgG sialylation seem to have an anti-inflammatory effect, as shown by studies in mice likely due to changes in Fc receptor binding [2]. It is thus plausible that traits associated to this locus are mainly reflecting sialylation and within-subclass ratios describe the addition of sialic acid.

Within the area of the gene *ST6GAL1*, we retrieve four separate LD-blocks (S4b, S5b Figs). Even though also the corresponding regional plots (S6b.i – iv Figs) show rather isolated signals, all SNPs covering the locus associate largely to the same IgG glycan traits. Indeed, the most significant SNPs from three out of the four LD-blocks (rs13082825, rs4012171, rs11710456) have been shown to affect the gene expression level of St6Gal1 (*p* = 1 x 10^-4^, *p* = 9 x 10^-6^, *p* = 6.3 x 10^-19^, resp.) [3]. The lead SNP of the fourth LD block, rs57679165, is in low LD with those three SNPs mentioned. Lauc et al [4] proposed at least two independent signals within the region. Our approach of defining LD-blocks can support the hypothesis of at least two independent signals, even though all of them might affect similar IgG glycan structures.

On chromosome 7, twelve SNPs, spanning over 18,656 bp, were located in close proximity to or within the gene ***IKZF1*** (see S6c Fig) and associate to several similar glycan traits each.

All replicated variants are in high LD, so that we observe only one LD-block which is supported by the regional association plot (S5c, S6c Figs).

Its role in IgG glycosylation has been extensively discussed in [4]. In this publication, the authors assign the role of down-regulation of fucosylation and up-regulation of bisecting GlcNAc attachment of IgG to this gene. While the binding of *IKZF1* to the promoter region of *MGAT3* explains the association of IgG glycan structures with bisecting GlcNAc [4], the effect on IgG fucosylation could not be explained by the mere binding within the regulatory region of *FUT8* [4]. Still, as for UPLC-measured glycan traits, we recognize similar associations between *IKZF1* and IgG fucosylation, even if these are weaker (minimal *p* = 2.38 x 10^-13^) than associations to *FUT8* (minimal *p* = 1.32 x 10^-24^). As effect directions of SNPs within the *IKZF1* locus of fucosylated and afucosylated glycan structures are opposite, its influence on the attachment of a core-fucose is quite likely. However, we would suggest further functional experiments to determine the appropriate subclass-specific pathway steps affected.

Most of the traits associated to SNPs in the ***B4GALT1*** region are related to glycans which are neutral, i.e. not sialylated, and to glycans which are present on all IgG subclasses. Associated within-subclass ratios are also related to asialylated glycans (G1F/G0F, G1FN/G0FN in IgG1 and IgG2 and G1/G0 in IgG1), except for G2FS1/G1FS1, in all three subclasses and reflect the addition of a galactose to the final glycan structure. The majority of glycan traits associated to the *B4GALT1* locus are asialylated.

High IgG galactosylation and sialylation levels block the pro-inflammatory effector function of IgG in cooperation with Fc gamma receptors [5]. However, only one glycan trait in IgG4 is significantly associated to variants within both the *B4GALT1* and *ST6GAL1* (LC_IGP187). We thus hypothesize that IgG glycosylation mediated by the two enzymes St6Gal1 and B4Galt1 both contribute to the anti-inflammatory function of IgG by catalyzing different steps in the IgG glycosylation pathway. However, it should be kept in mind that sialylation is not possible without galactosylation.

While the replicated SNPs on chromosome 9 within *B4GALT1* are assigned to several LD-blocks initially, almost all the blocks widely overlap, resulting in only two separate LD-blocks (S5d Fig). However, the regional plots (S6d.i, S6d.ii Figs) as well as the similar impact on gene expression found for the lead SNPs of the LD-blocks [6, 7], let us suggest that we observe only one contributing signal within this locus.

On chromosome 14, we replicated SNPs within a region from 142,710 bp downstream of the gene ***FUT8*** to 64,916 bp upstream of the gene. The variant rs11158592 had the strongest association within the locus (*p*(KORA) = 1.32 x 10^-24^ and *p*(LLS) = 2.39 x 10^-19^ for LC_IGP11). In general, variants at this locus were associated with 45 different glycan traits, mainly representing fucosylation. About half of the associated glycans are afucosylated and associate to the SNPs with opposite effect directions compared to the fucosylated traits. Additionally, within-subclass ratios for IgG1 and IgG2 are mimicking the attachment of core-fucose. The association to within-subclass ratios not representing the addition of fucose might be a mere technical coincidence. Alternatively, these associations may be a hint for the preferred fucosylation of specific glycan structures. Subclass comparing analyses moreover show that IgG1 and IgG2 are potentially influenced by *FUT8* to different extents.

Within the region of the gene *FUT8*, we would assume more than one independent signal simply due to its large range. However, the LD-blocks largely overlap (S5e and S6e.i – iii Figs) except for a single SNP (rs4899183) at the outermost part of the locus (chr14: 66275755) being associated to one glycan trait only (LC_IGP14, *p*(KORA) = 1.17 x 10^-8^ *p*(LLS) =1.96 x 10^-6^). Exploring the large region of 541 kbp indicates that there possibly is more than one independent signal within the locus. A detailed analysis of the functional relevance of the single markers and especially their influence on different glycan structures remains to be examined in further studies.

The strongest signal in the ***SMARCB1-DERL3*** locus arises from rs2186369 (LC_IGP108: *p*(LLS) = 2.5 x 10 ^-13^ , *p*(KORA) = 1.55 x 10 ^-9^).The highly correlated (r^2^ ≥ 0.5) variants stretch over a region 88 kbp of the *SMARCB1-DERL3* locus thereby covering more than two genes. The marker rs2186369 lies within the 7^th^ intron of *SMARCB1* (chr22:24,129,150-chr22:24,176,703) and correlated variants cover the gene on its total length (SNPs in chr22:24,100,654 – chr22:24,179,922), from 30 kbp downstream up to 3 kbp upstream of the gene. Associated glycans for the two computationally identified LD-blocks widely overlap (Fig 2). The traits only belong to subclasses IgG1 and IgG2 and are largely fucosylated asialylated structures with bisecting GlcNAcs. Summarizing derived traits (e.g. FBG0n/G0n), as well as the significant within-subclass ratios, hint at the addition of bisecting GlcNAc as primary regulatory influence caused by the associated variants. For the G-allele of the strongest SNP, rs2186369, we detect a decrease in bisecting glycan structures.

Association of expression of nearby quantitative trait loci (cis eQTLs) to *CHCHD10* (*p* = 2.7 x 10^-10^ for rs2186369) is stronger than to *SMARCB1* (*p* = 0.003 for rs2186369) [3] but varies for different tissues [8]. In contrast, rs8138673 seems to be much more strongly associated to gene expression of *SMARCB1* (*p* = 1 x 10^-35^) [3], however, it is likewise predicted to have functional relevance for the gene *DERL3* (chr22:24,176,690-chr22:24,181,315), which overlaps with *SMARCB1* [9]. Both *SMARCB1* and *DERL3* have tumor suppressor features. While *SMARCB1* has been most prominently shown to associate to sarcomas of different kinds [10-14], *DERL3* is known to reside in the endoplasmic reticulum and contributes to the degradation of misfolded proteins [15-17]. As IgG glycosylation happens mainly in the Golgi, *DERL3* is the more favorable candidate gene within the locus as it was proposed by [4]. However, given the LD-structure of all significant variants in the region, covering both genes, and, related eQTLS, further functional studies should be implemented to reveal the true relationships between these variants and IgG glycan traits.

The strongest association for the ***MGAT3*** locus can be found for rs73167342 with LC_IGP_R81 (*p*(LLS) = 2.53 x 10^-38^, *p*(KORA) = 7.71 x 10^-35^). While our approach yields four disitinct LD-blocks, the largest LD-block includes variants in high LD (> 0.5) to this SNP and extends over three genes, *SYNGR1*, *TAB1* and *MGAT3*. The remaining two LD blocks lie similarly in the *MGAT3* region (S6g.ii and S6g.iv Figs). Traits relating to asialylated glycans from all measured IgG subclasses, of which almost all are fucosylated and decorated with a bisecting GlcNAc, are altered by variants in the locus. The *SMARCB1-DERL3* locus is associated to similar glycan traits, especially ratios, as the *MGAT3* locus. Both loci are similarly associated to traits expressing the addition of bisecting GlcNAc. Within-subclass ratios significantly differently associated to SNPs in the *MGAT3* locus hint at a subclass-specific manner of the addition of bisecting GlcNAc by the enzyme encoded by *MGAT3*.

Several underlying genes have been found for the replicated SNPs in the *MGAT3* locus on chromosome 22q13.1 (*MGAT3*, *TAB1*, *SYNGR1*). Since the essential role of *N*-acetylglucosaminyltransferase, the addition of a bisecting GlcNAc, for the *N*-glycan biosynthesis has been validated before [18] and variants within the whole locus have been shown to be associated to UPLC-measured IgG glycans, *MGAT3* is very likely the underlying functional gene for the signals on this locus. Inspecting the regional association plot (S6g.i Fig) for the strongest SNP rs73167342, implies one signal within the locus and *MGAT3* being the most plausible candidate to explain the observed associations. Additionally, it has been shown that there are multiple promoters influencing the expression of *MGAT3* [19], which may explain the large spread of closely linked variants being significantly associated to IgG glycan structures. With *MGAT3* being the most plausible underlying gene, we suppose only one causal signal within the area.

With our study, we can confirm 6 loci suggestively associated to IgG glycosylation by Lauc et al [21] (S13 Table). However, we cannot confirm any of the novel loci suggested by Shen et al [20], most likely due to power issues.We compared these previous results to our new insights as far as possible. Therefore, we used the relation between UPLC-measured glycan traits and LC/MS-measured glycan traits as suggested by Huffmann et al [22] (see S10 Table). Additionally, we obtained LD information from snipa [50]. We looked up our lead-SNPs for each LD-block in the discovery meta-analysis from Lauc et al [21] for comparable UPLC-traits. As the study in [21] was conducted on HapMap 2.5 imputed genotype data, we additionally checked for SNPs in high LD or close to our lead-SNP if the lead-SNP itself was not available. The lookup of our new results in the data from the study by Lauc et al [21] can be found in S11 Table and are summarized in S12 Table.

# References

1. Hennet, T., et al., *Immune regulation by the ST6Gal sialyltransferase.* Proc Natl Acad Sci U S A, 1998. **95**(8): p. 4504-9.

2. Kaneko, Y., F. Nimmerjahn, and J.V. Ravetch, *Anti-inflammatory activity of immunoglobulin G resulting from Fc sialylation.* Science, 2006. **313**(5787): p. 670-3.

3. Westra, H.J., et al., *Systematic identification of trans eQTLs as putative drivers of known disease associations.* Nat Genet, 2013. **45**(10): p. 1238-43.

4. Lauc, G., et al., *Loci associated with N-glycosylation of human immunoglobulin G show pleiotropy with autoimmune diseases and haematological cancers.* PLoS Genet, 2013. **9**(1): p. e1003225.

5. Karsten, C.M., et al., *Anti-inflammatory activity of IgG1 mediated by Fc galactosylation and association of FcgammaRIIB and dectin-1.* Nat Med, 2012. **18**(9): p. 1401-6.

6. Lappalainen, T., et al., *Transcriptome and genome sequencing uncovers functional variation in humans.* Nature, 2013. **501**(7468): p. 506-11.

7. Consortium, G.T., *Human genomics. The Genotype-Tissue Expression (GTEx) pilot analysis: multitissue gene regulation in humans.* Science, 2015. **348**(6235): p. 648-60.

8. Arnold, M., et al., *SNiPA: an interactive, genetic variant-centered annotation browser.* Bioinformatics, 2015. **31**(8): p. 1334-6.

9. Kent, W.J., et al., *The human genome browser at UCSC.* Genome Res, 2002. **12**(6): p. 996-1006.

10. Modena, P., et al., *SMARCB1/INI1 tumor suppressor gene is frequently inactivated in epithelioid sarcomas.* Cancer Res, 2005. **65**(10): p. 4012-9.

11. Pottier, N., et al., *Expression of SMARCB1 modulates steroid sensitivity in human lymphoblastoid cells: identification of a promoter SNP that alters PARP1 binding and SMARCB1 expression.* Hum Mol Genet, 2007. **16**(19): p. 2261-71.

12. Ito, J., et al., *The diagnostic utility of reduced immunohistochemical expression of SMARCB1 in synovial sarcomas: a validation study.* Hum Pathol, 2016. **47**(1): p. 32-7.

13. Kosho, T., N. Okamoto, and C. Coffin-Siris Syndrome International, *Genotype-phenotype correlation of Coffin-Siris syndrome caused by mutations in SMARCB1, SMARCA4, SMARCE1, and ARID1A.* Am J Med Genet C Semin Med Genet, 2014. **166C**(3): p. 262-75.

14. Arnold, M.A., et al., *A unique pattern of INI1 immunohistochemistry distinguishes synovial sarcoma from its histologic mimics.* Hum Pathol, 2013. **44**(5): p. 881-7.

15. Nagai, A., et al., *USP14 inhibits ER-associated degradation via interaction with IRE1alpha.* Biochem Biophys Res Commun, 2009. **379**(4): p. 995-1000.

16. Sato, T., et al., *STT3B-dependent posttranslational N-glycosylation as a surveillance system for secretory protein.* Mol Cell, 2012. **47**(1): p. 99-110.

17. Oda, Y., et al., *Derlin-2 and Derlin-3 are regulated by the mammalian unfolded protein response and are required for ER-associated degradation.* J Cell Biol, 2006. **172**(3): p. 383-93.

18. Taniguchi, N., *Handbook of Glycosyltransferases and Related Genes*. 2014.

19. Koyama, N., et al., *Human N-acetylglucosaminyltransferase III gene is transcribed from multiple promoters.* Eur J Biochem, 1996. **238**(3): p. 853-61.

20. Shen, X., et al., *Multivariate discovery and replication of five novel loci associated with Immunoglobulin G N-glycosylation.* Nat Commun, 2017. **8**(1): p. 447.
